# Supplementary material for: Heat Stress Reduces the Susceptibility of Caenorhabditis elegans to Orsay Virus Infection
Source: Genes (Basel). 2021 Jul 28;12(8):1161. doi: 10.3390/genes12081161 (PMC8392475; doi:10.3390/genes12081161)
Supplement: Supplementary file 1 [file Supplementary Table S1.pdf › Supplementary Table S1.pdf]

strain	treatment1	chromosome	gene_bp_start	gene_bp_end	gene_WBID	gene_public_name	gene_sequence_name	FC	spots
CB4856	CT	I	3747	3909	WBGene00023193	Y74C9A.6	Y74C9A.6	0.0572822361547409	AGIWUR10283
CB4856	CT	I	4116	10230	WBGene00022277	homt-1	Y74C9A.3	0.038138245083827	AGIWUR36142
CB4856	CT	I	11495	16837	WBGene00022276	nlp-40	Y74C9A.2	-0.290645897988796	AGIWUR15573;AGIWUR25172
CB4856	CT	I	17487	26781	WBGene00022278	rcor-1	Y74C9A.4	-0.153594825448508	AGIWUR26476
CB4856	CT	I	27591	32544	WBGene00022279	sesn-1	Y74C9A.5	-0.378278118250027	AGIWUR22971
CB4856	CT	I	43733	44677	WBGene00022275	Y74C9A.1	Y74C9A.1	-0.031110273790933	AGIWUR5034
CB4856	CT	I	47467	49857	WBGene00044345	Y48G1C.12	Y48G1C.12	-0.225212687792613	AGIWUR8212;AGIWUR32887;AGIWUR43575
CB4856	CT	I	49919	54426	WBGene00021677	pgs-1	Y48G1C.4	0.0350669441511222	AGIWUR6338;AGIWUR29294;AGIWUR41966
CB4856	CT	I	55293	64066	WBGene00021678	Y48G1C.5	Y48G1C.5	0.174870530280208	AGIWUR12519
CB4856	CT	I	71425	81071	WBGene00000812	csk-1	Y48G1C.2	-0.115343558351268	AGIWUR18740;AGIWUR27071;AGIWUR29609;AGIWUR40038
CB4856	CT	I	81193	91020	WBGene00021683	Y48G1C.10	Y48G1C.10	-0.159181607424891	AGIWUR1487;AGIWUR6768;AGIWUR17886
CB4856	CT	I	91380	92877	WBGene00021682	Y48G1C.9	Y48G1C.9	-0.0643977165767584	AGIWUR6641;AGIWUR16497;AGIWUR29495
CB4856	CT	I	93024	94884	WBGene00021676	Y48G1C.1	Y48G1C.1	-0.0886182158283813	AGIWUR20733
CB4856	CT	I	96544	108223	WBGene00021681	Y48G1C.8	Y48G1C.8	0.0847028940421772	AGIWUR2118;AGIWUR4958;AGIWUR17289;AGIWUR20729;AGIWUR23172;AGIWUR25122;AGIWUR30532;AGIWUR33151;AGIWUR37921
CB4856	CT	I	108670	110107	WBGene00004274	rab-11.1	F53G12.1	-0.0813247821160523	AGIWUR7280;AGIWUR18237
CB4856	CT	I	111038	112417	WBGene00004418	rpl-7	F53G12.10	0.0604872196828339	AGIWUR2263;AGIWUR3392;AGIWUR5751;AGIWUR6722;AGIWUR12865;AGIWUR17613;AGIWUR21588;AGIWUR25879;AGIWUR34428;AGIWUR39201;AGIWUR39669
CB4856	CT	I	112285	113721	WBGene00018774	F53G12.9	F53G12.9	0.162815290391424	AGIWUR6433;AGIWUR10455;AGIWUR10794;AGIWUR22121;AGIWUR25979;AGIWUR27830;AGIWUR36366;AGIWUR36737
CB4856	CT	I	113807	114704	WBGene00018773	F53G12.8	F53G12.8	0.0867855403734694	AGIWUR11234;AGIWUR20914

CB4856	CT	I	115739	117438	WBGene00000622	col-45	F53G12.7	0.0393579449488968	AGIWUR14279
CB4856	CT	I	118109	120870	WBGene00004962	spe-8	F53G12.6	-0.099367608613401	AGIWUR621;AGIWUR6081;AGIWUR40104;AGIWUR43527
CB4856	CT	I	127297	134065	WBGene00003229	mex-3	F53G12.5	0.0468958207680874	AGIWUR13975;AGIWUR29437;AGIWUR44108
CB4856	CT	I	134337	137282	WBGene00018772	F53G12.4	F53G12.4	-0.0158516177266748	AGIWUR42484
CB4856	CT	I	146486	154850	WBGene00000253	bli-3	F56C11.1	-0.0246854270564416	AGIWUR3807
CB4856	CT	I	160172	166819	WBGene00004225	ptr-11	F56C11.2	0.0161465713386075	AGIWUR42837
CB4856	CT	I	171340	175989	WBGene00018958	F56C11.6	F56C11.6	0.389780367443706	AGIWUR6883
CB4856	CT	I	178538	182161	WBGene00018957	F56C11.5	F56C11.5	-0.0502291879875365	AGIWUR2702
CB4856	CT	I	182079	182772	WBGene00018955	F56C11.3	F56C11.3	-0.185917067845597	AGIWUR23793
CB4856	CT	I	183364	208465	WBGene00000227	atm-1	Y48G1BL.2	-0.151293849699115	AGIWUR463;AGIWUR33245;AGIWUR38423;AGIWUR39307
CB4856	CT	I	216028	216996	WBGene00021662	snpc-3.3	Y48G1BL.1	-0.0185274495888024	AGIWUR11670
CB4856	CT	I	219735	221575	WBGene00021664	Y48G1BL.5	Y48G1BL.5	-0.237189515236789	AGIWUR13754
CB4856	CT	I	238616	239021	WBGene00021672	Y48G1BM.7	Y48G1BM.7	0.0882180883144956	AGIWUR38722
CB4856	CT	I	240491	245414	WBGene00021668	Y48G1BM.2	Y48G1BM.2	0.181885964170025	AGIWUR29927
CB4856	CT	I	248624	251580	WBGene00021673	Y48G1BM.8	Y48G1BM.8	-0.0548076430134667	AGIWUR38995
CB4856	CT	I	252118	254277	WBGene00021667	snpc-3.2	Y48G1BM.1	-0.373210301497416	AGIWUR11205
CB4856	CT	I	254277	259157	WBGene00021675	Y48G1BR.1	Y48G1BR.1	-0.0178752911133779	AGIWUR4179;AGIWUR5959;AGIWUR23967;AGIWUR27432;AGIWUR32377;AGIWUR37148;AGIWUR43002;AGIWUR43138
CB4856	CT	I	280120	280445	WBGene00016904	C53D5.3	C53D5.3	0.422220948526773	AGIWUR33409
CB4856	CT	I	283500	286115	WBGene00016903	marc-4	C53D5.2	0.819719933843396	AGIWUR3252
CB4856	CT	I	286088	289185	WBGene00016905	ztf-3	C53D5.4	-0.21420088809607	AGIWUR12744
CB4856	CT	I	291215	305468	WBGene00016902	C53D5.1	C53D5.1	-0.349173554746477	AGIWUR2825
CB4856	CT	I	305811	311130	WBGene00016906	C53D5.5	C53D5.5	-0.195706948587421	AGIWUR41850

CB4856	CT	I	310981	314989	WBGene00002077	imb-3	C53D5.6	-0.0708606006805663	AGIWUR3906;AGIWUR8209;AGIWUR34608
CB4856	CT	I	318390	323352	WBGene00002079	xpo-2	Y48G1A.5	0.161450280193128	AGIWUR26191
CB4856	CT	I	323855	330978	WBGene00021660	Y48G1A.4	Y48G1A.4	-0.150921125701396	AGIWUR8979
CB4856	CT	I	332611	337839	WBGene00000917	daf-25	Y48G1A.3	-0.197287697146631	AGIWUR35344
CB4856	CT	I	343069	348424	WBGene00021661	mbtr-1	Y48G1A.6	-0.0997927925252426	AGIWUR1177
CB4856	CT	I	348460	357193	WBGene00021657	Y48G1A.1	Y48G1A.1	0.0943400378224055	AGIWUR10987
CB4856	CT	I	357240	363901	WBGene00020087	R119.1	R119.1	-0.0891612073275496	AGIWUR25426
CB4856	CT	I	364510	368319	WBGene00020091	rnp-8	R119.7	0.0331028299221323	AGIWUR25376
CB4856	CT	I	368370	373445	WBGene00020088	R119.2	R119.2	0.311581015441076	AGIWUR10202
CB4856	CT	I	373571	379073	WBGene00020089	R119.3	R119.3	0.101971769202334	AGIWUR3041;AGIWUR17217;AGIWUR27220;AGIWUR29433;AGIWUR30374;AGIWUR42548
CB4856	CT	I	379071	381902	WBGene00004143	pqn-59	R119.4	-0.00755355376780287	AGIWUR5926;AGIWUR7401;AGIWUR13641;AGIWUR16067;AGIWUR19397;AGIWUR19860;AGIWUR21791;AGIWUR26455;AGIWUR28874;AGIWUR32613;AGIWUR36219;AGIWUR36591;AGIWUR38419
CB4856	CT	I	382648	388564	WBGene00006385	taf-4	R119.6	0.111638982131804	AGIWUR11265
CB4856	CT	I	388470	394415	WBGene00020090	R119.5	R119.5	0.00705356294970549	AGIWUR1299;AGIWUR1860
CB4856	CT	I	398891	412915	WBGene00021733	otub-2	Y50C1A.1	-0.0144190768681725	AGIWUR9308;AGIWUR14492;AGIWUR36666
CB4856	CT	I	412911	418660	WBGene00021734	Y50C1A.2	Y50C1A.2	0.1948161650412	AGIWUR12911
CB4856	CT	I	420922	423713	WBGene00015570	C07F11.2	C07F11.2	0.0410718121082576	AGIWUR14464
CB4856	CT	I	443999	461605	WBGene00006593	tol-1	C07F11.1	0.0842135211940898	AGIWUR2677;AGIWUR10919;AGIWUR11990;AGIWUR12403;AGIWUR13788;AGIWUR18746;AGIWUR19485;AGIWUR26817;AGIWUR32956;AGIWUR39071;AGIWUR41796
CB4856	CT	I	461995	462651	WBGene00021026	W04C9.4	W04C9.4	-0.0456568870172571	AGIWUR40613
CB4856	CT	I	462657	467131	WBGene00021027	W04C9.5	W04C9.5	0.0515223369171238	AGIWUR32438
CB4856	CT	I	477582	481954	WBGene00021025	cutl-13	W04C9.3	0.750713757470874	AGIWUR7157;AGIWUR17034;AGIWUR44870
CB4856	CT	I	482040	483880	WBGene00235388	W04C9.9	W04C9.9	-0.517608023612533	AGIWUR43955
CB4856	CT	I	488607	489905	WBGene00021024	W04C9.2	W04C9.2	-0.160627648178302	AGIWUR6890
CB4856	CT	I	489969	496150	WBGene00001814	haf-4	W04C9.1	0.0579868486135879	AGIWUR32786

CB4856	CT	I	496144	502042	WBGene00022035	Y65B4BL.3	Y65B4BL.3	-0.0827939667261894	AGIWUR355
CB4856	CT	I	502932	506250	WBGene00022036	Y65B4BL.4	Y65B4BL.4	0.0610760405113346	AGIWUR42967
CB4856	CT	I	506469	509056	WBGene00022034	deps-1	Y65B4BL.2	0.0821849768817398	AGIWUR44389
CB4856	CT	I	511052	519800	WBGene00022037	acs-13	Y65B4BL.5	0.0050543734294628	AGIWUR5213;AGIWUR6411;AGIWUR12098;AGIWUR26016;AGIWUR28762;AGIWUR30485;AGIWUR41922
CB4856	CT	I	520109	521554	WBGene00022038	Y65B4BL.6	Y65B4BL.6	-0.217045221787076	AGIWUR10694;AGIWUR14351;AGIWUR18806;AGIWUR24805;AGIWUR30981;AGIWUR33410;AGIWUR36376;AGIWUR36747;AGIWUR36896;AGIWUR42899;AGIWUR44259
CB4856	CT	I	530969	533084	WBGene00022033	Y65B4BL.1	Y65B4BL.1	0.470045849203705	AGIWUR40224
CB4856	CT	I	534455	535344	WBGene00022043	psf-3	Y65B4BR.8	-0.199356635367785	AGIWUR31655
CB4856	CT	I	535791	536670	WBGene00022042	icd-2	Y65B4BR.5	0.0602303362877965	AGIWUR5033;AGIWUR8895;AGIWUR9943;AGIWUR17402;AGIWUR44103;AGIWUR45197
CB4856	CT	I	537122	542200	WBGene00007009	wwp-1	Y65B4BR.4	0.0180817802141391	AGIWUR1688;AGIWUR20352;AGIWUR38522
CB4856	CT	I	550696	556638	WBGene00004235	ptr-21	Y65B4BR.3	-0.0854133372573074	AGIWUR8387
CB4856	CT	I	557375	561671	WBGene00002393	lpr-1	Y65B4BR.2	0.381466082590725	AGIWUR5420
CB4856	CT	I	562736	565181	WBGene00022040	Y65B4BR.1	Y65B4BR.1	0.785507910116486	AGIWUR36108
CB4856	CT	I	565436	566414	WBGene00000434	ceh-9	Y65B4BR.9	-0.0012082377008249	AGIWUR20254;AGIWUR37142
CB4856	CT	I	572177	575120	WBGene00001725	grl-16	Y65B4BR.6	-0.496892188452303	AGIWUR7933;AGIWUR10290;AGIWUR17115
CB4856	CT	I	582288	583748	WBGene00044427	F56A6.5	F56A6.5	0.298488160576461	AGIWUR5027;AGIWUR22482;AGIWUR31223
CB4856	CT	I	585831	607601	WBGene00002040	hum-7	F56A6.2	-0.147096106529993	AGIWUR35233
CB4856	CT	I	614651	629444	WBGene00018923	eme-1	F56A6.4	-0.0666797568102773	AGIWUR9658;AGIWUR17702;AGIWUR40576
CB4856	CT	I	629492	636350	WBGene00022029	Y65B4A.6	Y65B4A.6	-0.315067790522942	AGIWUR16088
CB4856	CT	I	636804	637852	WBGene00022027	vps-20	Y65B4A.3	-0.200687121144678	AGIWUR2007;AGIWUR15178;AGIWUR23419;AGIWUR28868;AGIWUR29377;AGIWUR42530
CB4856	CT	I	638129	653986	WBGene00022026	Y65B4A.2	Y65B4A.2	-0.0228837148173255	

									AGIWUR28421
CB4856	CT	I	654090	658810	WBGene00022025	Y65B4A.1	Y65B4A.1	0.033133463123022	AGIWUR30815
CB4856	CT	I	659557	663686	WBGene00022031	Y65B4A.8	Y65B4A.8	-0.22236907166428	AGIWUR33639
CB4856	CT	I	667615	674413	WBGene00044461	Y18H1A.14	Y18H1A.14	0.194714823821787	AGIWUR26159;AGIWUR30290
CB4856	CT	I	682876	686599	WBGene00021214	hasp-2	Y18H1A.10	0.104009099859017	AGIWUR12962
CB4856	CT	I	688286	691267	WBGene00021207	Y18H1A.1	Y18H1A.1	-0.295719539018139	AGIWUR42845
CB4856	CT	I	692026	694948	WBGene00021210	Y18H1A.4	Y18H1A.4	0.00998442583920678	AGIWUR32934;AGIWUR40014
CB4856	CT	I	694982	698322	WBGene00021208	Y18H1A.2	Y18H1A.2	0.044337008904229	AGIWUR21289
CB4856	CT	I	700274	715874	WBGene00021209	hgap-1	Y18H1A.3	-0.109564898607659	AGIWUR7556;AGIWUR13243;AGIWUR29984;AGIWUR33686;AGIWUR39061;AGIWUR39297
CB4856	CT	I	717893	719816	WBGene00004332	rec-1	Y18H1A.7	0.0080689938977212	AGIWUR12224
CB4856	CT	I	719929	731078	WBGene00004028	pif-1	Y18H1A.6	-0.00209956163948632	AGIWUR23096
CB4856	CT	I	731068	736552	WBGene00044434	Y18H1A.15	Y18H1A.15	-0.0969114144793606	AGIWUR8664
CB4856	CT	I	741913	745570	WBGene00000623	col-46	Y18H1A.13	1.25672959205179	AGIWUR13435
CB4856	CT	I	748228	757010	WBGene00021213	Y18H1A.9	Y18H1A.9	0.121974987400336	AGIWUR1232
CB4856	CT	I	758756	760429	WBGene00021212	Y18H1A.8	Y18H1A.8	-0.363303772415374	AGIWUR19119
CB4856	CT	I	760848	761849	WBGene00020282	mps-3	T06A4.2	0.0649145700321814	AGIWUR43985
CB4856	CT	I	763597	770720	WBGene00020283	T06A4.3	T06A4.3	-0.4560010342373	AGIWUR4393
CB4856	CT	I	770892	776369	WBGene00020281	T06A4.1	T06A4.1	0.0430341809898209	AGIWUR22830
CB4856	CT	I	803368	835277	WBGene00022518	zfh-2	ZC123.3	0.18634714764485	AGIWUR8335;AGIWUR26889;AGIWUR32482;AGIWUR36028;AGIWUR37303;AGIWUR42904
CB4856	CT	I	837996	841746	WBGene00022517	ZC123.1	ZC123.1	0.152289174675247	AGIWUR1437;AGIWUR5409;AGIWUR11894;AGIWUR21175;AGIWUR35381;AGIWUR36327
CB4856	CT	I	841742	846763	WBGene00022519	ZC123.4	ZC123.4	0.131686846384255	AGIWUR20395;AGIWUR21786;AGIWUR27160;AGIWUR33235;AGIWUR38509
CB4856	CT	I	853451	872103	WBGene00022388	Y95B8A.8	Y95B8A.8	-0.282843410744972	

AGIWUR12290;AGIWUR40942
CB4856 CT I 873164 882713 WBGene00022389 pde-6 Y95B8A.10 -0.195001998200536 AGIWUR2879;AGIWUR8164;AGIWUR17662;AGIWUR23206;AGIWUR27165;AGIWUR27349
CB4856 CT I 882918 890194 WBGene00022386 Y95B8A.6 Y95B8A.6 0.956823940741959 AGIWUR26061;AGIWUR39290;AGIWUR39599
CB4856 CT I 897286 906077 WBGene00001678 gpa-16 Y95B8A.5 -0.133916880962758 AGIWUR36673
CB4856 CT I 907448 911189 WBGene00022385 Y95B8A.4 Y95B8A.4 -0.143835048536303 AGIWUR27919
CB4856 CT I 912415 926877 WBGene00022391 Y95B8A.12 Y95B8A.12 0.0437704107750161 AGIWUR20888;AGIWUR31755
CB4856 CT I 938464 941773 WBGene00022383 Y95B8A.2 Y95B8A.2 -0.391300169589703 AGIWUR3724;AGIWUR34637
CB4856 CT I 942257 947710 WBGene00003548 nas-30 Y95B8A.1 -0.341988244277148 AGIWUR13336
CB4856 CT I 948569 957404 WBGene00003238 mig-1 Y34D9B.1 0.32964244231385 AGIWUR19889;AGIWUR24156;AGIWUR39680;AGIWUR41925
CB4856 CT I 963700 969426 WBGene00018787 cutl-20 F54A5.2 0.340881374377121 AGIWUR36883
CB4856 CT I 969089 976550 WBGene00018788 shc-1 F54A5.3 -0.000317291776342987 AGIWUR1520;AGIWUR6328;AGIWUR7799;AGIWUR16360;AGIWUR21266;AGIWUR22224;AGIWUR23180;AGIWUR24163;AGIWUR25177;AGIWUR28900;AGIWUR30401;AGIWUR31527;AGIWUR33487;AGIWUR43199
CB4856 CT I 989671 992181 WBGene00018786 hmbx-1 F54A5.1 0.0138910401529992 AGIWUR17420;AGIWUR21529;AGIWUR26430;AGIWUR28932;AGIWUR31846;AGIWUR38211
CB4856 CT I 996045 1005066 WBGene00016932 C54G6.3 C54G6.3 -0.329660320585722 AGIWUR22;AGIWUR16210;AGIWUR29308;AGIWUR31907;AGIWUR35462;AGIWUR41023
CB4856 CT I 1006102 1010765 WBGene00016931 C54G6.2 C54G6.2 -0.097224519687753 AGIWUR6501
CB4856 CT I 1011371 1014455 WBGene00016930 madf-6 C54G6.1 0.110709753318485 AGIWUR5134;AGIWUR11965;AGIWUR20597;AGIWUR20929;AGIWUR32037
CB4856 CT I 1016403 1017180 WBGene00005002 spp-17 C54G6.5 0.0278636445245617 AGIWUR29378
CB4856 CT I 1018835 1019229 WBGene00021335 spp-23 Y34D9A.11 -0.486721864107726 AGIWUR9839;AGIWUR10201;AGIWUR16077;AGIWUR24534;AGIWUR34141;AGIWUR39770
CB4856 CT I 1019827 1029910 WBGene00021327 mrpl-38 Y34D9A.1 0.189791431745228 AGIWUR42178
CB4856 CT I 1030629 1033436 WBGene00004952 spd-1 Y34D9A.4 -0.0378672141904666 AGIWUR8339;AGIWUR10414;AGIWUR10601;AGIWUR13233;AGIWUR34053
CB4856 CT I 1034443 1040868 WBGene00021329 Y34D9A.3 Y34D9A.3 -0.107873846326134 AGIWUR17558;AGIWUR19082;AGIWUR25332;AGIWUR28801;AGIWUR35935;AGIWUR41040;AGIWUR41363
CB4856 CT I 1041474 1048329 WBGene00021332 Y34D9A.7 Y34D9A.7 -0.422698518293391 AGIWUR3469

CB4856	CT	I	1049596	1050727	WBGene00021331	glrx-10	Y34D9A.6	-0.0142175702509899	AGIWUR11461
CB4856	CT	I	1050921	1053826	WBGene00021333	Y34D9A.8	Y34D9A.8	0.0191908912235816	AGIWUR13006;AGIWUR14778;AGIWUR23259;AGIWUR23416;AGIWUR24085;AGIWUR32213
CB4856	CT	I	1053894	1066899	WBGene00021334	vps-4	Y34D9A.10	0.0481231804463143	AGIWUR2517;AGIWUR33071;AGIWUR33422
CB4856	CT	I	1068484	1075010	WBGene00001745	gsa-1	R06A10.2	0.0219668801763202	AGIWUR7028
CB4856	CT	I	1084630	1088473	WBGene00019909	R06A10.1	R06A10.1	-0.0569372119365372	AGIWUR12833
CB4856	CT	I	1088514	1089742	WBGene00044895	R06A10.5	R06A10.5	0.123956525761516	AGIWUR8078;AGIWUR13536;AGIWUR18834;AGIWUR20617;AGIWUR32661
CB4856	CT	I	1089666	1095796	WBGene00019911	R06A10.4	R06A10.4	-0.138913005799147	AGIWUR18722
CB4856	CT	I	1112418	1115501	WBGene00236809	ZK993.5	ZK993.5	-0.0345491160477929	AGIWUR41688
CB4856	CT	I	1128324	1131737	WBGene00022837	ceh-45	ZK993.1	0.713128771832602	AGIWUR30087
CB4856	CT	I	1136371	1154386	WBGene00021689	Y48G8AL.10	Y48G8AL.10	-0.118209361406406	AGIWUR4185;AGIWUR5373;AGIWUR25625
CB4856	CT	I	1154511	1156020	WBGene00000165	aps-3	Y48G8AL.14	-0.220657185224148	AGIWUR10923
CB4856	CT	I	1156579	1157474	WBGene00004429	rpl-17	Y48G8AL.8	-0.00533880974089129	AGIWUR4065;AGIWUR7333;AGIWUR10981;AGIWUR14030;AGIWUR16076;AGIWUR16834;AGIWUR21488;AGIWUR22895;AGIWUR33449;AGIWUR37938;AGIWUR38748;AGIWUR41085;AGIWUR41517
CB4856	CT	I	1157571	1162939	WBGene00021687	Y48G8AL.7	Y48G8AL.7	-0.0640140497313126	AGIWUR25543
CB4856	CT	I	1162880	1175868	WBGene00001816	haf-6	Y48G8AL.11	-0.157114358443225	AGIWUR20067
CB4856	CT	I	1176471	1190829	WBGene00004880	smg-2	Y48G8AL.6	-0.0932598126143986	AGIWUR59
CB4856	CT	I	1193158	1211434	WBGene00021686	Y48G8AL.5	Y48G8AL.5	-0.338679541455603	AGIWUR18159;AGIWUR45123
CB4856	CT	I	1211485	1254669	WBGene00021685	herc-1	Y48G8AL.1	-0.136943525767833	AGIWUR13334
CB4856	CT	I	1254770	1258258	WBGene00021692	Y48G8AL.15	Y48G8AL.15	0.0377888733602099	AGIWUR13059;AGIWUR26397;AGIWUR27751
CB4856	CT	I	1259374	1268155	WBGene00001707	grh-1	Y48G8AR.1	-0.0891175296782478	AGIWUR17365
CB4856	CT	I	1282101	1285435	WBGene00021693	Y48G8AR.2	Y48G8AR.2	0.253351791572936	AGIWUR39120

CB4856	CT	I	1287304	1287959	WBGene00021694	Y48G8AR.3	Y48G8AR.3	0.0993456609134089	AGIWUR41090
CB4856	CT	I	1305562	1328348	WBGene00003400	dapk-1	K12C11.4	-0.371603475245288	AGIWUR11986
CB4856	CT	I	1332698	1334074	WBGene00044896	K12C11.5	K12C11.5	-0.0589654310800602	AGIWUR7756;AGIWUR20555;AGIWUR32336
CB4856	CT	I	1335321	1336867	WBGene00019674	K12C11.3	K12C11.3	-0.231505441650063	AGIWUR13813;AGIWUR27282;AGIWUR39984;AGIWUR42143
CB4856	CT	I	1337012	1338216	WBGene00045052	K12C11.6	K12C11.6	-0.00370241809629412	AGIWUR12512;AGIWUR16762;AGIWUR45006
CB4856	CT	I	1338749	1339773	WBGene00045053	K12C11.7	K12C11.7	0.152043123375619	AGIWUR14477;AGIWUR15520;AGIWUR29050;AGIWUR30108;AGIWUR34743;AGIWUR36565;AGIWUR42371
CB4856	CT	I	1340678	1341258	WBGene00004888	smo-1	K12C11.2	-0.36628676558778	AGIWUR36454;AGIWUR38737
CB4856	CT	I	1341218	1344692	WBGene00019673	K12C11.1	K12C11.1	-0.0744633061335636	AGIWUR3302;AGIWUR43117
CB4856	CT	I	1348503	1359595	WBGene00022363	Y92H12BL.1	Y92H12BL.1	-0.142484376105614	AGIWUR15759;AGIWUR28550;AGIWUR44414
CB4856	CT	I	1365309	1376409	WBGene00022366	Y92H12BL.5	Y92H12BL.5	0.0993744323290819	AGIWUR35560
CB4856	CT	I	1376942	1385057	WBGene00022365	Y92H12BL.4	Y92H12BL.4	-0.0524157035015858	AGIWUR6416;AGIWUR23727
CB4856	CT	I	1385818	1392759	WBGene00022367	Y92H12BM.1	Y92H12BM.1	-0.332361493109663	AGIWUR24013;AGIWUR35109
CB4856	CT	I	1394569	1402975	WBGene00022372	Y92H12BR.7	Y92H12BR.7	-0.315613575080846	AGIWUR22492
CB4856	CT	I	1405935	1413100	WBGene00022371	set-29	Y92H12BR.6	0.00963345703506135	AGIWUR42567
CB4856	CT	I	1413915	1415807	WBGene00022368	Y92H12BR.2	Y92H12BR.2	-0.0098417602126446	AGIWUR36024
CB4856	CT	I	1416586	1425201	WBGene00022369	Y92H12BR.3	Y92H12BR.3	-0.316740278309293	AGIWUR1737;AGIWUR4253;AGIWUR5022;AGIWUR5477;AGIWUR7001;AGIWUR8655;AGIWUR21433;AGIWUR25311;AGIWUR27839;AGIWUR31391;AGIWUR41245
CB4856	CT	I	1427227	1433695	WBGene00022370	Y92H12BR.4	Y92H12BR.4	0.155496299641678	AGIWUR17553;AGIWUR20837;AGIWUR27978;AGIWUR31709;AGIWUR35420
CB4856	CT	I	1436279	1441331	WBGene00022373	mrpl-15	Y92H12BR.8	0.00168775129001197	AGIWUR31574
CB4856	CT	I	1442048	1443942	WBGene00018159	F37F2.2	F37F2.2	-0.327556709845631	AGIWUR8713
CB4856	CT	I	1446497	1465358	WBGene00004969	spe-15	F47G6.4	-0.102413203682023	

									AGIWUR2637;AGIWUR3005;AGIWUR4351;AGIWUR25255;AGIWUR32395;AGIWUR35658
CB4856	CT	I	1465597	1469587	WBGene00018580	F47G6.3	F47G6.3	0.00697013485501685	AGIWUR38276
CB4856	CT	I	1471227	1479920	WBGene00018579	acs-16	F47G6.2	0.440155472248684	AGIWUR42596
CB4856	CT	I	1483083	1490494	WBGene00001115	dyb-1	F47G6.1	0.110625584304652	AGIWUR6684;AGIWUR22860;AGIWUR35939
CB4856	CT	I	1490887	1514411	WBGene00022361	Y92H12A.5	Y92H12A.5	-0.318499524934	AGIWUR13887;AGIWUR31743
CB4856	CT	I	1514755	1540911	WBGene00022360	Y92H12A.4	Y92H12A.4	-0.295483491749567	AGIWUR7916;AGIWUR18838
CB4856	CT	I	1543525	1556196	WBGene00022358	Y92H12A.2	Y92H12A.2	0.117842093836737	AGIWUR12716
CB4856	CT	I	1566929	1580196	WBGene00005077	src-1	Y92H12A.1	0.174071638648029	AGIWUR29323;AGIWUR36808
CB4856	CT	I	1581394	1593668	WBGene00022270	Y73E7A.3	Y73E7A.3	-0.334295575994757	AGIWUR9496
CB4856	CT	I	1594659	1596184	WBGene00022271	cpx-1	Y73E7A.4	-0.209228927363542	AGIWUR20188
CB4856	CT	I	1600624	1601457	WBGene00022272	Y73E7A.5	Y73E7A.5	-0.127213792145401	AGIWUR28873
CB4856	CT	I	1601838	1606926	WBGene00022269	Y73E7A.2	Y73E7A.2	-0.251131293296261	AGIWUR16524
CB4856	CT	I	1607658	1610041	WBGene00022273	Y73E7A.6	Y73E7A.6	-0.215286803674752	AGIWUR32464;AGIWUR39665
CB4856	CT	I	1610390	1620080	WBGene00044440	adpr-1	Y73E7A.9	-0.099147231154042	AGIWUR8254;AGIWUR35568
CB4856	CT	I	1621346	1628476	WBGene00000269	bre-4	Y73E7A.7	-0.167882617414467	AGIWUR34680
CB4856	CT	I	1629003	1633498	WBGene00022268	Y73E7A.1	Y73E7A.1	0.0260175833198417	AGIWUR1633;AGIWUR3458;AGIWUR3977;AGIWUR7843;AGIWUR11159;AGIWUR21506;AGIWUR22736;AGIWUR23690;AGIWUR42544
CB4856	CT	I	1634613	1644107	WBGene00022274	Y73E7A.8	Y73E7A.8	0.665535684996062	AGIWUR20107
CB4856	CT	I	1644588	1651394	WBGene00003111	mab-20	Y71G12B.20	-0.0115088655884437	AGIWUR27582;AGIWUR40456
CB4856	CT	I	1652901	1655399	WBGene00022156	Y71G12B.18	Y71G12B.18	-0.295360300185104	AGIWUR116
CB4856	CT	I	1655616	1656726	WBGene00004168	pqn-88	Y71G12B.21	-0.113406691381311	AGIWUR26050
CB4856	CT	I	1657513	1663331	WBGene00044358	Y71G12B.33	Y71G12B.33	-0.15353673386176	AGIWUR2249;AGIWUR8734;AGIWUR20752;AGIWUR30652

CB4856	CT	I	1667501	1669792	WBGene00022155	Y71G12B.17	Y71G12B.17	-0.0421000758689516
AGIWUR42831
CB4856	CT	I	1670036	1672742	WBGene00022158	Y71G12B.23	Y71G12B.23	0.154037533447747
AGIWUR5132;AGIWUR11929;AGIWUR32676
CB4856	CT	I	1680450	1685063	WBGene00022154	drag-1	Y71G12B.16	-0.199637682338393
AGIWUR35035
CB4856	CT	I	1686216	1693457	WBGene00044348	Y71G12B.31	Y71G12B.31	0.080565079659082
AGIWUR1166;AGIWUR4811;AGIWUR35486
CB4856	CT	I	1698094	1701128	WBGene00006702	ubc-3	Y71G12B.15	0.09946902575299
AGIWUR4490;AGIWUR30533
CB4856	CT	I	1701361	1701808	WBGene00003080	lsm-6	Y71G12B.14	0.0287120607881612
AGIWUR4447;AGIWUR11056
CB4856	CT	I	1702105	1708008	WBGene00022153	Y71G12B.13	Y71G12B.13	-0.217305842883317
AGIWUR12172
CB4856	CT	I	1709587	1715568	WBGene00022152	atg-5	Y71G12B.12	-0.0971791937252373
AGIWUR7357;AGIWUR29374;AGIWUR37289
CB4856	CT	I	1721592	1740670	WBGene00006771	tln-1	Y71G12B.11	-0.146765909392495
AGIWUR12058;AGIWUR13591;AGIWUR32962;AGIWUR33419;AGIWUR38217;AGIWUR43568
CB4856	CT	I	1740647	1751055	WBGene00022159	mppa-1	Y71G12B.24	-0.26776499784318
AGIWUR24046
CB4856	CT	I	1751481	1754410	WBGene00022150	Y71G12B.10	Y71G12B.10	0.143085799864926
AGIWUR41998
CB4856	CT	I	1755329	1766400	WBGene00022149	lin-65	Y71G12B.9	-0.133212198019838
AGIWUR776;AGIWUR21654
CB4856	CT	I	1767887	1790157	WBGene00022148	ddx-27	Y71G12B.8	-0.0704284902204737
AGIWUR18548
CB4856	CT	I	1793254	1796424	WBGene00022146	Y71G12B.6	Y71G12B.6	-0.118290735577856
AGIWUR4786
CB4856	CT	I	1797435	1798722	WBGene00022162	Y71G12B.27	Y71G12B.27	0.216384100977551
AGIWUR15897
CB4856	CT	I	1799717	1806986	WBGene00022145	Y71G12B.5	Y71G12B.5	0.170293169088531
AGIWUR2625;AGIWUR25042
CB4856	CT	I	1808091	1811835	WBGene00022144	pghm-1	Y71G12B.4	0.573352814295478
AGIWUR4145;AGIWUR9299;AGIWUR10064;AGIWUR17445;AGIWUR18149;AGIWUR45023
CB4856	CT	I	1812892	1813581	WBGene00022143	Y71G12B.3	Y71G12B.3	0.0442587358403301
AGIWUR14630
CB4856	CT	I	1813909	1816061	WBGene00044349	Y71G12B.32	Y71G12B.32	0.0372018710424937
AGIWUR3178;AGIWUR36722;AGIWUR39264
CB4856	CT	I	1816150	1818496	WBGene00022142	Y71G12B.2	Y71G12B.2	-0.0077973652631993

AGIWUR13069
CB4856 CT I 1818581 1822948 WBGene00044347 Y71G12B.30 Y71G12B.30 -0.159627659531608
AGIWUR19858;AGIWUR26230
CB4856 CT I 1823447 1825967 WBGene00022141 chaf-2 Y71G12B.1 0.098141609492302
AGIWUR6848
CB4856 CT I 1832115 1832641 WBGene00044746 C53H9.3 C53H9.3 -0.131752540027914
AGIWUR17914;AGIWUR20995;AGIWUR22306;AGIWUR23217;AGIWUR33050;AGIWUR42007
CB4856 CT I 1832897 1834764 WBGene00016907 C53H9.2 C53H9.2 -0.0124603405802803
AGIWUR5438;AGIWUR6589;AGIWUR6611;AGIWUR16807;AGIWUR17795;AGIWUR20304;AGIWUR26029;AGIWUR27807;AGIWUR31482;AG
IWUR33037;AGIWUR34381;AGIWUR34900
CB4856 CT I 1834881 1835439 WBGene00004441 rpl-27 C53H9.1 0.0317598927932181 AGIWUR15399
CB4856 CT I 1835631 1841936 WBGene00001196 egl-30 M01D7.7 -0.14306788861719
AGIWUR554;AGIWUR27713
CB4856 CT I 1842445 1843227 WBGene00001309 emr-1 M01D7.6 0.00686540270273284 AGIWUR40352
CB4856 CT I 1843384 1843899 WBGene00003750 nlp-12 M01D7.5 0.446247393417075
AGIWUR7286;AGIWUR33113
CB4856 CT I 1845221 1850523 WBGene00006461 tag-96 M01D7.4 -0.115818793103013 AGIWUR22089
CB4856 CT I 1851681 1852280 WBGene00044897 M01D7.9 M01D7.9 -0.0374113315930877
AGIWUR1984;AGIWUR13390;AGIWUR16714
CB4856 CT I 1856459 1860578 WBGene00019706 M01D7.1 M01D7.1 0.287305945143225 AGIWUR43544
CB4856 CT I 1862607 1872306 WBGene00004743 scm-1 M01D7.2 -0.239000723157464 AGIWUR25971
CB4856 CT I 1874033 1883639 WBGene00022139 tub-2 Y71G12A.3 0.00321894238572185
AGIWUR28588
CB4856 CT I 1885205 1894905 WBGene00022140 Y71G12A.4 Y71G12A.4 -0.0560351321734878
AGIWUR36911
CB4856 CT I 1895161 1923088 WBGene00022138 trpp-10 Y71G12A.2 -0.16665127028644
AGIWUR19988;AGIWUR29483
CB4856 CT I 1924300 1932260 WBGene00021766 hex-4 Y51F10.5 -0.405785475712593
AGIWUR620
CB4856 CT I 1932352 1945212 WBGene00021765 Y51F10.4 Y51F10.4 0.184805845222762
AGIWUR33035
CB4856 CT I 1950594 1958527 WBGene00021771 spe-48 Y51F10.10 0.138579355406517
AGIWUR38998
CB4856 CT I 1958707 1965267 WBGene00044438 Y51F10.11 Y51F10.11 -0.308773227248803
AGIWUR3528;AGIWUR6982;AGIWUR40531
CB4856 CT I 1967772 1973882 WBGene00021764 Y51F10.3 Y51F10.3 -0.0805133779309681
AGIWUR6418
CB4856 CT I 1977993 1983436 WBGene00021763 Y51F10.2 Y51F10.2 -0.285995808423666
AGIWUR19000

CB4856	CT	I	1988246 2010525 WBGene00021238	gap-3	Y20F4.3 -0.1718627186208
AGIWUR11087;AGIWUR20405;AGIWUR25539;AGIWUR31637;AGIWUR32847;AGIWUR44046
CB4856	CT	I	2026277 2033966 WBGene00021237	crtc-1	Y20F4.2 0.249345436901475	AGIWUR16307
CB4856	CT	I	2034383 2043805 WBGene00021239	Y20F4.4 Y20F4.4 0.365961641233268	AGIWUR3718
CB4856	CT	I	2049140 2050177 WBGene00044435	Y20F4.8 Y20F4.8 0.0378564318217212
AGIWUR4442;AGIWUR5343;AGIWUR18496
CB4856	CT	I	2061667 2064077 WBGene00021346	Y37E3.1 Y37E3.1 -0.0225593854601644	AGIWUR16488
CB4856	CT	I	2064909 2065481 WBGene00021348	moag-4	Y37E3.4 -0.14970695989726	AGIWUR5629
CB4856	CT	I	2065727 2069049 WBGene00021349	arl-13	Y37E3.5 0.49115518545645	AGIWUR20970
CB4856	CT	I	2069086 2069587 WBGene00004409	rla-1	Y37E3.7 0.20506520625301
AGIWUR3039;AGIWUR4288;AGIWUR14249;AGIWUR27765;AGIWUR37184;AGIWUR40288
CB4856	CT	I	2069681 2070607 WBGene00021350	Y37E3.8 Y37E3.8 -0.00404132510047894
AGIWUR13326;AGIWUR16500;AGIWUR18905;AGIWUR33748;AGIWUR34085;AGIWUR35002;AGIWUR38325;AGIWUR39997
CB4856	CT	I	2071352 2072547 WBGene00004014	phb-1	Y37E3.9 -0.178443770411011	AGIWUR8000
CB4856	CT	I	2072680 2076906 WBGene00021351	eif-2alpha	Y37E3.10	-0.23412895273008
AGIWUR6037;AGIWUR23060;AGIWUR26110;AGIWUR41959
CB4856	CT	I	2078959 2085662 WBGene00021352	Y37E3.11	Y37E3.11
-0.00574465042138428	AGIWUR2995
CB4856	CT	I	2085673 2093822 WBGene00021353	iglr-3	Y37E3.13	0.165027507907212
AGIWUR15489;AGIWUR30008
CB4856	CT	I	2097346 2123443 WBGene00003799	npp-13	Y37E3.15	-0.183001576536742
AGIWUR11120;AGIWUR13240
CB4856	CT	I	2128590 2145971 WBGene00021355	Y37E3.17	Y37E3.17	-0.0481403374042699
AGIWUR25279
CB4856	CT	I	2160042 2209629 WBGene00021009	afd-1	W03F11.6	-0.00147028269201321
AGIWUR4041;AGIWUR5559;AGIWUR9161;AGIWUR9303;AGIWUR14028;AGIWUR14832;AGIWUR15541;AGIWUR17457;AGIWUR21460;AGI
WUR36718;AGIWUR37855;AGIWUR39179;AGIWUR39762
CB4856	CT	I	2216100 2219479 WBGene00021008	W03F11.5	W03F11.5	0.0907856717973328
AGIWUR42538
CB4856	CT	I	2219471 2222313 WBGene00021005	ule-1	W03F11.1	-0.325321699286706
AGIWUR3046
CB4856	CT	I	2223307 2233099 WBGene00001542	gcy-17	W03F11.2	0.0163483334872954
AGIWUR33861
CB4856	CT	I	2233744 2234957 WBGene00021006	dct-9	W03F11.3	-0.0427674668186775
AGIWUR22187
CB4856	CT	I	2235617 2241210 WBGene00021007	W03F11.4	W03F11.4	0.566268782760927
AGIWUR9560
CB4856	CT	I	2242230 2247223 WBGene00017399	lgc-51	F12B6.3 0.0957357733485189	AGIWUR28630
CB4856	CT	I	2250131 2254901 WBGene00017398	slc-17.6	F12B6.2 0.273330269598357

AGIWUR20287;AGIWUR25238;AGIWUR39652;AGIWUR42417
CB4856 CT I 2255923 2272866 WBGene00000020 abt-2 F12B6.1 -0.077767648787173
AGIWUR23301;AGIWUR28644
CB4856 CT I 2279835 2281516 WBGene00021458 Y39G10AL.1 Y39G10AL.1 0.0313645100019642
AGIWUR36209
CB4856 CT I 2281504 2291183 WBGene00000408 cdk-7 Y39G10AL.3 0.186629827151613
AGIWUR39048
CB4856 CT I 2293132 2297312 WBGene00021469 Y39G10AR.11 Y39G10AR.11 0.448950925437682
AGIWUR22260
CB4856 CT I 2297280 2304393 WBGene00021470 tpxl-1 Y39G10AR.12 0.136680200462402
AGIWUR12614;AGIWUR36527
CB4856 CT I 2304512 2306816 WBGene00002047 icp-1 Y39G10AR.13 -0.0839428447758657
AGIWUR14808;AGIWUR28043;AGIWUR29130;AGIWUR29237;AGIWUR38297
CB4856 CT I 2306985 2308975 WBGene00021467 Y39G10AR.9 Y39G10AR.9 -0.257740010333102
AGIWUR6667
CB4856 CT I 2308976 2312129 WBGene00021476 nsun-4 Y39G10AR.21 -0.145667146214935
AGIWUR13426
CB4856 CT I 2312496 2313008 WBGene00021475 tbca-1 Y39G10AR.20 0.0586410755619582
AGIWUR41500
CB4856 CT I 2313271 2316003 WBGene00021466 eif-2gamma Y39G10AR.8 -0.0761671053220296
AGIWUR1012;AGIWUR30510
CB4856 CT I 2316307 2318305 WBGene00021465 ekl-7 Y39G10AR.7 -0.133329412760503
AGIWUR18593
CB4856 CT I 2318291 2321847 WBGene00003156 mcm-4 Y39G10AR.14 -0.218110714229272
AGIWUR23145
CB4856 CT I 2322025 2322596 WBGene00219275 Y39G10AR.32 Y39G10AR.32 -0.286345434539683
AGIWUR27494
CB4856 CT I 2327989 2330162 WBGene00021477 srbc-64 Y39G10AR.22 0.0814450129596072
AGIWUR16180
CB4856 CT I 2330895 2332652 WBGene00021472 Y39G10AR.16 Y39G10AR.16 -0.367504325839318
AGIWUR4806;AGIWUR12047;AGIWUR19458;AGIWUR24864;AGIWUR39806
CB4856 CT I 2333312 2338693 WBGene00021464 ugt-31 Y39G10AR.6 -0.351114341476981
AGIWUR16968
CB4856 CT I 2342216 2350468 WBGene00021463 zeel-1 Y39G10AR.5 0.210367472326789
AGIWUR22312
CB4856 CT I 2357883 2372964 WBGene00021461 nekl-1 Y39G10AR.3 -0.0294394267800047
AGIWUR3700;AGIWUR12255
CB4856 CT I 2377089 2381851 WBGene00021473 sydn-1 Y39G10AR.17 -0.0340003820354449
AGIWUR29573;AGIWUR29726

CB4856	CT	I	2382953	2401492	WBGene00021474	dot-1.1	Y39G10AR.18	-0.0503584816415543	AGIWUR2332;AGIWUR3782;AGIWUR25161;AGIWUR29638
CB4856	CT	I	2402232	2409987	WBGene00021460	zwl-1	Y39G10AR.2	0.080046336783564	AGIWUR39214;AGIWUR43207;AGIWUR45076
CB4856	CT	I	2415133	2418521	WBGene00017741	F23C8.13	F23C8.13	0.0775610562218887	AGIWUR805
CB4856	CT	I	2418450	2420801	WBGene00017734	F23C8.5	F23C8.5	-0.114505169906937	AGIWUR9817;AGIWUR12095;AGIWUR12773;AGIWUR21578;AGIWUR22886;AGIWUR43078
CB4856	CT	I	2420803	2421816	WBGene00017735	did-2	F23C8.6	-0.0213701286267796	AGIWUR36365
CB4856	CT	I	2429675	2432601	WBGene00017737	F23C8.8	F23C8.8	-0.160961448889015	AGIWUR29692
CB4856	CT	I	2432570	2433755	WBGene00017738	F23C8.9	F23C8.9	-0.0866295737369485	AGIWUR11516;AGIWUR12714;AGIWUR24496;AGIWUR39273;AGIWUR44822;AGIWUR45164
CB4856	CT	I	2434004	2435708	WBGene00017733	ubxn-1	F23C8.4	-0.277314442252713	AGIWUR26378
CB4856	CT	I	2438767	2442948	WBGene00017732	F23C8.3	F23C8.3	0.0855572111447929	AGIWUR4209;AGIWUR24398;AGIWUR32650;AGIWUR34132
CB4856	CT	I	2445420	2445983	WBGene00023273	F23C8.2	F23C8.2	-0.0175780790194668	AGIWUR10294
CB4856	CT	I	2446740	2448369	WBGene00017731	F23C8.1	F23C8.1	-0.113035926334772	AGIWUR34435
CB4856	CT	I	2449414	2451366	WBGene00017739	F23C8.11	F23C8.11	0.0723684319281777	AGIWUR5077
CB4856	CT	I	2451908	2464637	WBGene00002141	inx-19	T16H5.1	0.127010426883251	AGIWUR2403
CB4856	CT	I	2468787	2471972	WBGene00022280	Y74C10AL.2	Y74C10AL.2	0.0488185192297212	AGIWUR21766
CB4856	CT	I	2472066	2485391	WBGene00022281	abtm-1	Y74C10AR.3	-0.212872059626829	AGIWUR5309;AGIWUR8973;AGIWUR10270;AGIWUR25468;AGIWUR30612;AGIWUR33610
CB4856	CT	I	2485867	2489780	WBGene00001232	eif-3.I	Y74C10AR.1	-0.0473626616542049	AGIWUR29413
CB4856	CT	I	2489976	2497371	WBGene00021277	Y23H5B.6	Y23H5B.6	0.0247866068912028	AGIWUR38087
CB4856	CT	I	2498134	2506259	WBGene00021276	Y23H5B.5	Y23H5B.5	-0.453978342933237	AGIWUR5878;AGIWUR6191;AGIWUR6274;AGIWUR28260
CB4856	CT	I	2506891	2513710	WBGene00021278	Y23H5B.7	Y23H5B.7	0.213020166387357	AGIWUR29322;AGIWUR30588
CB4856	CT	I	2533268	2542114	WBGene00021275	dmsr-2	Y23H5B.4	0.0747317394240037	AGIWUR14185
CB4856	CT	I	2544749	2546163	WBGene00021274	Y23H5B.3	Y23H5B.3	0.0677021605501906	AGIWUR14576
CB4856	CT	I	2546897	2548358	WBGene00021279	Y23H5B.8	Y23H5B.8	0.701657681844123	AGIWUR24104
CB4856	CT	I	2549296	2550543	WBGene00021273	Y23H5B.2	Y23H5B.2	-0.0280328140258135	

AGIWUR20479
CB4856 CT I 2551375 2551590 WBGene00021280 nspd-8 Y23H5B.9 0.0978310969961864
AGIWUR21495
CB4856 CT I 2556252 2557425 WBGene00194701 Y23H5B.12 Y23H5B.12 -0.378944345406208
AGIWUR19243
CB4856 CT I 2558300 2560110 WBGene00016950 C55C2.4 C55C2.4 0.0602734355122037 AGIWUR14938
CB4856 CT I 2560396 2561820 WBGene00016949 C55C2.3 C55C2.3 -0.0112876751365221
AGIWUR5924;AGIWUR10632
CB4856 CT I 2562616 2563049 WBGene00006047 ssp-19 C55C2.2 0.302723947522722 AGIWUR13085
CB4856 CT I 2568872 2570746 WBGene00016948 scrt-1 C55C2.1 0.246585507549242 AGIWUR37839
CB4856 CT I 2571782 2576020 WBGene00000006 aat-5 C55C2.5 0.143034479669394
AGIWUR2506;AGIWUR10049;AGIWUR18292;AGIWUR40198
CB4856 CT I 2580935 2586126 WBGene00001404 fbp-1 K07A3.1 0.075870257756808
AGIWUR9040;AGIWUR12995;AGIWUR25728;AGIWUR35028;AGIWUR35818
CB4856 CT I 2587527 2595294 WBGene00004226 ptr-12 K07A3.2 0.00692330437249566
AGIWUR617;AGIWUR33884;AGIWUR35603
CB4856 CT I 2599979 2602627 WBGene00019461 K07A3.3 K07A3.3 0.0247283034490522
AGIWUR13808;AGIWUR24193;AGIWUR42169
CB4856 CT I 2606754 2617360 WBGene00000832 ctn-1 Y23H5A.5 0.0116868852382397
AGIWUR10113;AGIWUR17733;AGIWUR41210;AGIWUR41252
CB4856 CT I 2617560 2620425 WBGene00021271 spe-47 Y23H5A.4 -0.0941352418949357
AGIWUR24731
CB4856 CT I 2620654 2621859 WBGene00021270 Y23H5A.3 Y23H5A.3 -0.201244015525765
AGIWUR40693
CB4856 CT I 2621957 2623186 WBGene00021269 Y23H5A.2 Y23H5A.2 0.0746558718032482
AGIWUR22648
CB4856 CT I 2623102 2628577 WBGene00000800 cars-1 Y23H5A.7 0.0962758718745107
AGIWUR23131
CB4856 CT I 2629685 2632320 WBGene00000801 cars-2 Y23H5A.1 -0.241747789163913
AGIWUR1077;AGIWUR6380;AGIWUR10780;AGIWUR19734;AGIWUR20638
CB4856 CT I 2632987 2634561 WBGene00023172 Y23H5A.8 Y23H5A.8 -0.0418084241587717
AGIWUR14216
CB4856 CT I 2638649 2639419 WBGene00018231 F40E3.3 F40E3.3 0.0487581560320161
AGIWUR4066;AGIWUR10758;AGIWUR23936;AGIWUR24120;AGIWUR24171;AGIWUR31356;AGIWUR35745;AGIWUR37922;AGIWUR39709;
AGIWUR43197
CB4856 CT I 2642724 2643778 WBGene00018230 mage-1 F40E3.2 -0.129553583142181 AGIWUR23200
CB4856 CT I 2645273 2657241 WBGene00018232 F40E3.5 F40E3.5 0.374928232799714
AGIWUR26138;AGIWUR32507
CB4856 CT I 2668160 2672434 WBGene00017979 F32B5.6 F32B5.6 0.154304993532813

AGIWUR7646;AGIWUR13992;AGIWUR30184
CB4856	CT	I	2672940	2682797	WBGene00017980	F32B5.7	F32B5.7	-0.0917314899954076
AGIWUR6695;AGIWUR6824;AGIWUR13525;AGIWUR18240;AGIWUR27541;AGIWUR31729
CB4856	CT	I	2686639	2689493	WBGene00000788	cpz-1	F32B5.8	0.0487105968458899	AGIWUR41569
CB4856	CT	I	2692074	2692467	WBGene00017978	F32B5.4	F32B5.4	0.196995206193881
AGIWUR7463;AGIWUR9486;AGIWUR10833;AGIWUR14697;AGIWUR21940;AGIWUR33121;AGIWUR33897;AGIWUR44678;AGIWUR45085
CB4856	CT	I	2692757	2694084	WBGene00017977	F32B5.3	F32B5.3	0.0602011650218692	AGIWUR18941
CB4856	CT	I	2697399	2699955	WBGene00017976	F32B5.2	F32B5.2	0.103323089536907	AGIWUR23268
CB4856	CT	I	2706883	2717549	WBGene00003006	lin-17	Y71F9B.5	0.281067068351805
AGIWUR27001;AGIWUR35050
CB4856	CT	I	2718027	2721475	WBGene00001048	dnj-30	Y71F9B.16	0.259134579317825
AGIWUR40146
CB4856	CT	I	2721879	2726724	WBGene00022128	Y71F9B.6	Y71F9B.6	-0.511068746431267
AGIWUR19507
CB4856	CT	I	2727014	2727545	WBGene00004920	snr-7	Y71F9B.4	-0.508421060064124
AGIWUR7048
CB4856	CT	I	2730004	2731453	WBGene00022127	yop-1	Y71F9B.3	-0.071856054160377
AGIWUR5660;AGIWUR6385;AGIWUR8144;AGIWUR16646;AGIWUR35160;AGIWUR37496
CB4856	CT	I	2731562	2735371	WBGene00022126	Y71F9B.2	Y71F9B.2	-0.171749000101475
AGIWUR8231
CB4856	CT	I	2735345	2742508	WBGene00022129	lron-11	Y71F9B.8	0.473273217608281
AGIWUR4975;AGIWUR8612
CB4856	CT	I	2755540	2758204	WBGene00004043	plk-2	Y71F9B.7	0.0711141577957815
AGIWUR32009
CB4856	CT	I	2759023	2761044	WBGene00022130	Y71F9B.9	Y71F9B.9	0.940328235803225
AGIWUR15089
CB4856	CT	I	2761158	2776097	WBGene00004946	sop-3	Y71F9B.10	0.071536232032436
AGIWUR13710;AGIWUR15065;AGIWUR23349
CB4856	CT	I	2776370	2778726	WBGene00022131	Y71F9B.13	Y71F9B.13	0.0528564874029476
AGIWUR18999;AGIWUR21201
CB4856	CT	I	2780850	2784276	WBGene00022133	Y71F9B.15	Y71F9B.15	-0.0176407115841504
AGIWUR16378;AGIWUR33305
CB4856	CT	I	2784620	2786077	WBGene00020990	W03D8.9	W03D8.9	-0.00974714552690514	AGIWUR3317
CB4856	CT	I	2786332	2787514	WBGene00020991	W03D8.10	W03D8.10	-0.0171928225575861
AGIWUR21745
CB4856	CT	I	2791687	2794976	WBGene00020989	W03D8.8	W03D8.8	-0.439508341532813
AGIWUR22441;AGIWUR29065;AGIWUR38128
CB4856	CT	I	2796130	2797238	WBGene00020988	W03D8.7	W03D8.7	-0.160592480030989	AGIWUR11786
CB4856	CT	I	2798175	2800878	WBGene00020985	W03D8.2	W03D8.2	0.257305534137636	AGIWUR7975

CB4856	CT	I	2802822 2815786 WBGene00002174	itx-1	W03D8.6 0.292780406083846	AGIWUR14038
CB4856	CT	I	2816415 2817646 WBGene00020987	W03D8.5 W03D8.5 -0.0407385985085824
AGIWUR3546;AGIWUR4156;AGIWUR9484;AGIWUR19425;AGIWUR24118
CB4856	CT	I	2818744 2820701 WBGene00044796	W03D8.11	W03D8.11	-0.324575819820188
AGIWUR42963
CB4856	CT	I	2820869 2822300 WBGene00020986	W03D8.3 W03D8.3 -0.0016488437270219	AGIWUR40700
CB4856	CT	I	2823254 2830311 WBGene00004077	pop-1	W10C8.2 0.2171446315506 AGIWUR37103
CB4856	CT	I	2832872 2833253 WBGene00021126	W10C8.3 W10C8.3 0.307512487442198	AGIWUR28937
CB4856	CT	I	2845859 2849792 WBGene00021125	ccb-2	W10C8.1 -0.0585468081748513
AGIWUR17713;AGIWUR29981;AGIWUR44951
CB4856	CT	I	2851963 2853903 WBGene00021127	W10C8.4 W10C8.4 -0.0213313452442572
AGIWUR2101;AGIWUR26297;AGIWUR28145
CB4856	CT	I	2858524 2859913 WBGene00044433	W10C8.6 W10C8.6 0.445771405999764
AGIWUR1574;AGIWUR2177;AGIWUR33520;AGIWUR42210;AGIWUR45158
CB4856	CT	I	2862403 2864446 WBGene00022116	Y71F9AL.11	Y71F9AL.11	0.71007076668803
AGIWUR36633
CB4856	CT	I	2866508 2870167 WBGene00022115	Y71F9AL.10	Y71F9AL.10	0.200427832321594
AGIWUR30152
CB4856	CT	I	2870608 2875289 WBGene00022117	Y71F9AL.12	Y71F9AL.12	-0.340586149714724
AGIWUR29739
CB4856	CT	I	2875395 2876324 WBGene00004412	rpl-1	Y71F9AL.13	-0.154465945890202
AGIWUR13419;AGIWUR14964
CB4856	CT	I	2876932 2878523 WBGene00022114	Y71F9AL.9	Y71F9AL.9	0.0255641651946457
AGIWUR15225;AGIWUR43288
CB4856	CT	I	2878578 2880929 WBGene00022113	Y71F9AL.8	Y71F9AL.8	0.294871031875994
AGIWUR29805
CB4856	CT	I	2880919 2883454 WBGene00022118	dnc-5	Y71F9AL.14	-0.100247225660718
AGIWUR1789;AGIWUR5524;AGIWUR13460;AGIWUR25120;AGIWUR26338
CB4856	CT	I	2883546 2893718 WBGene00000199	arx-1	Y71F9AL.16	0.273584083883794
AGIWUR11177
CB4856	CT	I	2895329 2900860 WBGene00022119	copa-1	Y71F9AL.17	-0.0671370584246276
AGIWUR19351
CB4856	CT	I	2901325 2903006 WBGene00001791	gst-43	Y71F9AL.5	-0.161340289198616
AGIWUR21982
CB4856	CT	I	2903596 2907751 WBGene00022110	Y71F9AL.4	Y71F9AL.4	0.235432660638672
AGIWUR25316
CB4856	CT	I	2925212 2928134 WBGene00022107	Y71F9AL.1	Y71F9AL.1	-0.311092696224438
AGIWUR2864
CB4856	CT	I	2928395 2929687 WBGene00022122	trap-1	Y71F9AM.6	-0.206254438595735

AGIWUR4502;AGIWUR4985;AGIWUR5165;AGIWUR5707;AGIWUR13179;AGIWUR27433;AGIWUR45195

CB4856	CT	I	2929736	2931361	WBGene00003836	nxt-1	Y71F9AM.5	-0.00875922169740662	AGIWUR15059
CB4856	CT	I	2931461	2947152	WBGene00022121	cogc-3	Y71F9AM.4	-0.150514880089421	AGIWUR4108;AGIWUR6047;AGIWUR36855;AGIWUR41452
CB4856	CT	I	2949600	2957910	WBGene00000237	bam-2	Y71F9AR.1	0.385827366396693	AGIWUR41421
CB4856	CT	I	2981531	2983076	WBGene00044894	Y71F9AR.4	Y71F9AR.4	-0.277377553705117	AGIWUR18847
CB4856	CT	I	2983213	2985167	WBGene00022123	Y71F9AR.2	Y71F9AR.2	-0.15857508576501	AGIWUR19262
CB4856	CT	I	2986104	2995914	WBGene00022124	supr-1	Y71F9AR.3	0.337390258483726	AGIWUR10209
CB4856	CT	I	2996401	3001382	WBGene00001046	dnj-28	Y54E10BL.4	-0.546018058732541	AGIWUR1394
CB4856	CT	I	3001360	3002847	WBGene00021839	nduf-5	Y54E10BL.5	-0.0315234252898353	AGIWUR6233
CB4856	CT	I	3015742	3021531	WBGene00021843	Y54E10BR.4	Y54E10BR.4	0.0102445132354418	AGIWUR20452
CB4856	CT	I	3021651	3028396	WBGene00021842	Y54E10BR.3	Y54E10BR.3	-0.0857466104591147	AGIWUR41419
CB4856	CT	I	3028307	3031092	WBGene00021844	Y54E10BR.5	Y54E10BR.5	-0.240459802454834	AGIWUR27706;AGIWUR30378
CB4856	CT	I	3031212	3032938	WBGene00021845	rpb-7	Y54E10BR.6	-0.221506741319324	AGIWUR32718
CB4856	CT	I	3032935	3034761	WBGene00021841	Y54E10BR.2	Y54E10BR.2	-0.21887394829736	AGIWUR2238;AGIWUR18683;AGIWUR24455;AGIWUR43905
CB4856	CT	I	3035193	3047794	WBGene00003387	mod-5	Y54E10BR.7	0.117821999669639	AGIWUR24272
CB4856	CT	I	3052018	3059185	WBGene00021846	ztf-23	Y54E10BR.8	-0.00173035083145834	AGIWUR33528
CB4856	CT	I	3059151	3069393	WBGene00021840	Y54E10BR.1	Y54E10BR.1	0.0531193822105323	AGIWUR14801
CB4856	CT	I	3069325	3070150	WBGene00000205	arx-7	M01B12.3	0.0176913068489952	AGIWUR5428
CB4856	CT	I	3070973	3084874	WBGene00019697	M01B12.4	M01B12.4	-0.120953470851951	AGIWUR16943;AGIWUR36776;AGIWUR37540
CB4856	CT	I	3085011	3096285	WBGene00019698	riok-1	M01B12.5	0.101919626268369	AGIWUR5372;AGIWUR5467;AGIWUR12452;AGIWUR27824;AGIWUR28472;AGIWUR30070;AGIWUR34208;AGIWUR36598;AGIWUR41932

CB4856	CT	I	3094513	3109596	WBGene00003654	nhr-64	C45E1.1	0.304948159869857	AGIWUR14818
CB4856	CT	I	3115036	3117628	WBGene00016663	C45E1.4	C45E1.4	0.210598691932478	AGIWUR41381
CB4856	CT	I	3137185	3138447	WBGene00000626	col-49	K09H9.3	-0.430267851157609	AGIWUR23696
CB4856	CT	I	3138541	3140601	WBGene00045387	K09H9.8	K09H9.8	0.445114154300384	AGIWUR6683;AGIWUR9499;AGIWUR29974
CB4856	CT	I	3141704	3143197	WBGene00019597	K09H9.5	K09H9.5	-0.251370365748761	AGIWUR19387
CB4856	CT	I	3144410	3147793	WBGene00003062	lpd-6	K09H9.6	-0.228540193405767	AGIWUR13406
CB4856	CT	I	3147996	3150349	WBGene00019595	K09H9.2	K09H9.2	-0.21735289292411	AGIWUR25014
CB4856	CT	I	3150346	3162387	WBGene00006888	vbh-1	Y54E10A.9	-0.261980340036692	AGIWUR36846
CB4856	CT	I	3162803	3163649	WBGene00021829	mrpl-17	Y54E10A.7	-0.296363205545637	AGIWUR1305
CB4856	CT	I	3163688	3167925	WBGene00021828	Y54E10A.6	Y54E10A.6	-0.263692982087719	AGIWUR24704
CB4856	CT	I	3167908	3171230	WBGene00021827	dnc-6	Y54E10A.5	1.77635683940025e-15	AGIWUR5057;AGIWUR9073;AGIWUR40125
CB4856	CT	I	3173243	3176319	WBGene00021830	Y54E10A.10	Y54E10A.10	0.263066907648106	AGIWUR13178
CB4856	CT	I	3176419	3192857	WBGene00021831	Y54E10A.11	Y54E10A.11	-0.168643430572669	AGIWUR2164;AGIWUR16574
CB4856	CT	I	3193528	3198838	WBGene00021832	Y54E10A.12	Y54E10A.12	-0.0497904681610688	AGIWUR12213
CB4856	CT	I	3199540	3203229	WBGene00021833	Y54E10A.13	Y54E10A.13	-0.150296768977172	AGIWUR9596
CB4856	CT	I	3206730	3209294	WBGene00021835	Y54E10A.17	Y54E10A.17	-0.38294690327783	AGIWUR37690
CB4856	CT	I	3211046	3217366	WBGene00001481	fog-1	Y54E10A.4	-0.306387603085199	AGIWUR13603;AGIWUR15435;AGIWUR35965
CB4856	CT	I	3217296	3222950	WBGene00004404	rom-5	Y54E10A.14	0.0715468567598112	AGIWUR37258
CB4856	CT	I	3223160	3228104	WBGene00000413	cdt-1	Y54E10A.15	-0.194094906880602	AGIWUR7164;AGIWUR8441;AGIWUR9365;AGIWUR10177;AGIWUR14248;AGIWUR14397;AGIWUR28748;AGIWUR40356
CB4856	CT	I	3229431	3233294	WBGene00021826	txl-1	Y54E10A.3	-0.350639975527347	AGIWUR39852
CB4856	CT	I	3233391	3255678	WBGene00003258	cogc-1	Y54E10A.2	-0.375714432025655	AGIWUR30427;AGIWUR32820;AGIWUR39620
CB4856	CT	I	3259871	3264254	WBGene00021553	tyr-5	Y44E3B.2	-0.09149442114804	AGIWUR23134
CB4856	CT	I	3264237	3266762	WBGene00021552	zip-4	Y44E3B.1	0.0313821443974085	

AGIWUR2699;AGIWUR20566;AGIWUR20700;AGIWUR26560;AGIWUR28062;AGIWUR30568;AGIWUR32351
CB4856	CT	I	3267094 3269116 WBGene00020915	nol-5	W01B11.3	0.0636446831431989
AGIWUR37743
CB4856	CT	I	3269406 3280883 WBGene00004154	pqn-72	W01B11.5	-0.244899056609348
AGIWUR12149;AGIWUR23263;AGIWUR25848;AGIWUR35656;AGIWUR42564;AGIWUR43889
CB4856	CT	I	3287336 3293127 WBGene00020914	sulp-6	W01B11.2	-0.000169415945704188
AGIWUR2355;AGIWUR10745;AGIWUR30073;AGIWUR36750;AGIWUR37204;AGIWUR39158
CB4856	CT	I	3293142 3294356 WBGene00020917	W01B11.6	W01B11.6	-0.181199361253189
AGIWUR11957;AGIWUR23982;AGIWUR24179
CB4856	CT	I	3299606 3300490 WBGene00020913	W01B11.1	W01B11.1	-0.243851176394431
AGIWUR1462
CB4856	CT	I	3302001 3307587 WBGene00000036	ace-2	Y44E3A.2	-0.28247718433361
AGIWUR33576
CB4856	CT	I	3317978 3318584 WBGene00021548	trx-4	Y44E3A.3	-0.0894554153599962
AGIWUR13327
CB4856	CT	I	3319540 3330673 WBGene00021549	Y44E3A.4	Y44E3A.4	-0.218224726814245
AGIWUR4866;AGIWUR12826;AGIWUR21456;AGIWUR37487
CB4856	CT	I	3338367 3340219 WBGene00021547	Y44E3A.1	Y44E3A.1	0.294327504837728
AGIWUR6438;AGIWUR21696;AGIWUR28706
CB4856	CT	I	3340231 3349907 WBGene00021551	Y44E3A.6	Y44E3A.6	-0.0276071770248576
AGIWUR19599;AGIWUR28799
CB4856	CT	I	3367640 3368550 WBGene00021035	W05F2.3 W05F2.3 0.0488118551417305
AGIWUR11491;AGIWUR17920;AGIWUR23022;AGIWUR38692;AGIWUR40671;AGIWUR42685
CB4856	CT	I	3368785 3371444 WBGene00021038	W05F2.6 W05F2.6 0.128692000806758	AGIWUR10259
CB4856	CT	I	3371769 3373834 WBGene00021037	fbxa-203	W05F2.5 -0.156483281186647
AGIWUR18896
CB4856	CT	I	3374776 3407189 WBGene00021036	W05F2.4 W05F2.4 0.324595162761646
AGIWUR1629;AGIWUR8478;AGIWUR12583;AGIWUR15753;AGIWUR22393;AGIWUR23436;AGIWUR32189;AGIWUR34613
CB4856	CT	I	3408033 3415238 WBGene00019362	cdk-2	K03E5.3 -0.336666685847508	AGIWUR23928
CB4856	CT	I	3419600 3424211 WBGene00019361	K03E5.2 K03E5.2 -0.0893942106166836
AGIWUR1571;AGIWUR13604;AGIWUR16735;AGIWUR17165;AGIWUR21604;AGIWUR26478;AGIWUR26766;AGIWUR27970;AGIWUR30728;
AGIWUR31092;AGIWUR37714;AGIWUR39861;AGIWUR43156;AGIWUR43908
CB4856	CT	I	3427177 3433207 WBGene00019360	neto-1	K03E5.1 -0.234794881185541	AGIWUR34916
CB4856	CT	I	3433157 3438543 WBGene00004775	sep-1	Y47G6A.12	-0.183307053533095
AGIWUR2398
CB4856	CT	I	3439108 3449175 WBGene00006440	tag-63	Y47G6A.28	-0.178315574287014
AGIWUR25852
CB4856	CT	I	3449326 3462177 WBGene00003422	msh-6	Y47G6A.11	-0.126804002591289
AGIWUR22181

CB4856	CT	I	3462270 3468187 WBGene00021640	Y47G6A.14	Y47G6A.14	-0.158601049894456
AGIWUR1119
CB4856	CT	I	3467132 3478515 WBGene00004978	spg-7	Y47G6A.10	-0.26622551739832
AGIWUR21359
CB4856	CT	I	3479329 3481439 WBGene00021638	Y47G6A.9	Y47G6A.9	-0.164511717251131
AGIWUR35431
CB4856	CT	I	3481987 3484499 WBGene00000794	crn-1	Y47G6A.8	0.00362286692886116
AGIWUR13801;AGIWUR22565
CB4856	CT	I	3487812 3499706 WBGene00021643	ccep-290	Y47G6A.17	0.0474083386460897
AGIWUR3330;AGIWUR15688
CB4856	CT	I	3502738 3508421 WBGene00021637	Y47G6A.7	Y47G6A.7	-0.0538358895503546
AGIWUR6082;AGIWUR7088;AGIWUR24590
CB4856	CT	I	3508397 3512735 WBGene00021644	Y47G6A.18	Y47G6A.18	-0.0764321664293064
AGIWUR4776;AGIWUR5770;AGIWUR6663;AGIWUR14345;AGIWUR18070;AGIWUR30279;AGIWUR31587;AGIWUR36047;AGIWUR38200;AG
IWUR38330;AGIWUR41216
CB4856	CT	I	3516450 3523387 WBGene00021636	pcaf-1	Y47G6A.6	0.00249735716046473
AGIWUR32442
CB4856	CT	I	3523313 3529956 WBGene00021645	Y47G6A.19	Y47G6A.19	-0.602909320963258
AGIWUR31354
CB4856	CT	I	3533885 3539196 WBGene00021635	Y47G6A.5	Y47G6A.5	0.237542144345808
AGIWUR37951
CB4856	CT	I	3539554 3546904 WBGene00004389	rnp-6	Y47G6A.20	-0.200905080719707
AGIWUR16653;AGIWUR31705;AGIWUR35799;AGIWUR39341
CB4856	CT	I	3547071 3552005 WBGene00021634	rde-10	Y47G6A.4	-0.0131967975064562
AGIWUR39093
CB4856	CT	I	3551949 3559807 WBGene00021646	Y47G6A.21	Y47G6A.21	0.0580467994204774
AGIWUR20916;AGIWUR42118
CB4856	CT	I	3559923 3562851 WBGene00021647	Y47G6A.22	Y47G6A.22	-0.163430684913724
AGIWUR28433
CB4856	CT	I	3564040 3565068 WBGene00045399	Y47G6A.33	Y47G6A.33	-0.457554109499452
AGIWUR25152;AGIWUR41843
CB4856	CT	I	3565012 3600729 WBGene00003060	lpd-3	Y47G6A.23	-0.076801244827422
AGIWUR25264;AGIWUR43063
CB4856	CT	I	3601291 3602908 WBGene00021633	Y47G6A.3	Y47G6A.3	-0.0330681430173208
AGIWUR21723
CB4856	CT	I	3603343 3604537 WBGene00044436	Y47G6A.31	Y47G6A.31	-0.110107001353326
AGIWUR2009;AGIWUR4486;AGIWUR11467;AGIWUR16255
CB4856	CT	I	3604520 3605763 WBGene00021648	mis-12	Y47G6A.24	0.155751416388927
AGIWUR31284

CB4856	CT	I	3606411	3608345	WBGene00021649	Y47G6A.25	Y47G6A.25	-0.325865145313148	AGIWUR16764;AGIWUR17972;AGIWUR30368;AGIWUR34454;AGIWUR38799
CB4856	CT	I	3608395	3612301	WBGene00002144	inx-22	Y47G6A.2	-0.244035664414998	AGIWUR32737
CB4856	CT	I	3611909	3618317	WBGene00002143	inx-21	Y47G6A.1	-0.0545084801580078	AGIWUR2419
CB4856	CT	I	3618889	3619697	WBGene00021650	Y47G6A.26	Y47G6A.26	0.336734599910779	AGIWUR10804
CB4856	CT	I	3620582	3625087	WBGene00044437	Y47G6A.32	Y47G6A.32	0.0511790298948647	AGIWUR28152;AGIWUR35782;AGIWUR39596
CB4856	CT	I	3629471	3631112	WBGene00000284	cah-6	T28F2.3	-0.259967096367112	AGIWUR29909
CB4856	CT	I	3631086	3636485	WBGene00020902	jmjc-1	T28F2.4	0.13803396140855	AGIWUR10716;AGIWUR27941
CB4856	CT	I	3637353	3638819	WBGene00020901	T28F2.2	T28F2.2	-0.0739631937052327	AGIWUR3655;AGIWUR17862;AGIWUR19553;AGIWUR28347;AGIWUR30674;AGIWUR35823
CB4856	CT	I	3639648	3641347	WBGene00020900	T28F2.1	T28F2.1	-0.0967512276263758	AGIWUR41008
CB4856	CT	I	3641668	3647009	WBGene00000368	ccb-1	T28F2.5	-0.062414012200545	AGIWUR17902;AGIWUR36522
CB4856	CT	I	3651230	3652599	WBGene00000627	col-50	T28F2.6	0.132902543262881	AGIWUR28502;AGIWUR33906
CB4856	CT	I	3653997	3655265	WBGene00000628	col-51	T28F2.8	-0.598297499871173	AGIWUR29327
CB4856	CT	I	3655413	3662183	WBGene00020903	acd-5	T28F2.7	-0.0551659351833216	AGIWUR13129
CB4856	CT	I	3666923	3671700	WBGene00004890	smp-2	D1037.2	-0.272144385008527	AGIWUR14570
CB4856	CT	I	3680178	3680944	WBGene00001501	ftn-2	D1037.3	-0.0972989803561379	AGIWUR8517;AGIWUR21576;AGIWUR30113;AGIWUR39905;AGIWUR40881;AGIWUR45149
CB4856	CT	I	3682772	3684118	WBGene00004272	rab-8	D1037.4	-0.0821745092887909	AGIWUR8091;AGIWUR21531;AGIWUR23567;AGIWUR31383;AGIWUR34408;AGIWUR40705;AGIWUR44617
CB4856	CT	I	3684783	3690192	WBGene00017025	D1037.1	D1037.1	-0.0260639505913076	AGIWUR23371
CB4856	CT	I	3690181	3694021	WBGene00017026	ipla-4	D1037.5	0.0827636582554776	AGIWUR39546
CB4856	CT	I	3694296	3697549	WBGene00003418	msh-2	H26D21.2	-0.100805953673046	AGIWUR4168
CB4856	CT	I	3698102	3699300	WBGene00002042	hus-1	H26D21.1	-0.328690330858016	AGIWUR20692
CB4856	CT	I	3706030	3709435	WBGene00017651	F21A9.2	F21A9.2	0.202243382054709	AGIWUR4947
CB4856	CT	I	3712133	3713134	WBGene00017650	F21A9.1	F21A9.1	0.329029187148882	AGIWUR22805
CB4856	CT	I	3713142	3721157	WBGene00003040	lin-59	T12F5.4	0.380256021239026	AGIWUR22950;AGIWUR24884;AGIWUR25052
CB4856	CT	I	3726011	3731265	WBGene00001601	glh-4	T12F5.3	-0.167044581346108	AGIWUR15176
CB4856	CT	I	3732982	3740479	WBGene00004147	larp-5	T12F5.5	-0.0241757527844664	

AGIWUR11520;AGIWUR29583;AGIWUR33557
CB4856 CT I 3741456 3742646 WBGene00020466 sld-2 T12F5.1 -0.401282770355294 AGIWUR14577
CB4856 CT I 3748523 3753958 WBGene00002135 inx-13 Y8G1A.2 -0.104658482512481
AGIWUR5735;AGIWUR7745;AGIWUR14145;AGIWUR26568;AGIWUR30702;AGIWUR38532
CB4856 CT I 3755457 3759077 WBGene00002134 inx-12 ZK770.3 -0.0896238003671623
AGIWUR5815;AGIWUR12211;AGIWUR19906
CB4856 CT I 3769681 3773876 WBGene00022815 asic-1 ZK770.1 0.0358950274112262 AGIWUR39041
CB4856 CT I 3774706 3777468 WBGene00016324 C32E8.6 C32E8.6 0.232177388990424
AGIWUR13968;AGIWUR42073
CB4856 CT I 3778089 3781690 WBGene00004368 ric-19 C32E8.7 0.523494626395891 AGIWUR5505
CB4856 CT I 3782118 3783294 WBGene00016323 C32E8.5 C32E8.5 0.237065735816377
AGIWUR10554;AGIWUR18167
CB4856 CT I 3783835 3784435 WBGene00016322 C32E8.4 C32E8.4 0.199730623709966
AGIWUR25087;AGIWUR28898;AGIWUR38913
CB4856 CT I 3786588 3787494 WBGene00016321 tppp-1 C32E8.3 -0.03913780044752 AGIWUR830
CB4856 CT I 3788036 3788909 WBGene00004425 rpl-13 C32E8.2 0.11902363811059
AGIWUR4831;AGIWUR12028;AGIWUR21601;AGIWUR24937;AGIWUR36210;AGIWUR40534;AGIWUR40558;AGIWUR40887
CB4856 CT I 3789309 3792797 WBGene00004217 ptr-2 C32E8.8 -0.151008640317166
AGIWUR13785;AGIWUR26858;AGIWUR38652
CB4856 CT I 3794556 3798069 WBGene00016325 C32E8.9 C32E8.9 -0.346784288220736 AGIWUR631
CB4856 CT I 3798566 3805737 WBGene00006751 unc-11 C32E8.10 -0.182226552831484
AGIWUR7869;AGIWUR17953;AGIWUR22953;AGIWUR24588;AGIWUR25836;AGIWUR39231;AGIWUR39504
CB4856 CT I 3807458 3816809 WBGene00016326 ubr-1 C32E8.11 -0.0609687278227717
AGIWUR21445
CB4856 CT I 3817996 3821505 WBGene00003797 npp-11 F53F10.5 -0.0194238084298242
AGIWUR20808
CB4856 CT I 3821656 3821990 WBGene00023066 F53F10.7 F53F10.7 0.120629118301774
AGIWUR7872;AGIWUR22404;AGIWUR22849;AGIWUR28979;AGIWUR36927
CB4856 CT I 3824947 3826514 WBGene00006833 unc-108 F53F10.4 0.18656375822339
AGIWUR4694;AGIWUR13234;AGIWUR19555;AGIWUR21037;AGIWUR23542;AGIWUR33385;AGIWUR35111;AGIWUR36324;AGIWUR37033;
AGIWUR41608
CB4856 CT I 3827219 3828143 WBGene00018765 mpc-2 F53F10.3 -0.303995639385914
AGIWUR21074
CB4856 CT I 3828239 3829308 WBGene00044423 F53F10.8 F53F10.8
-0.00825178941686033 AGIWUR6429;AGIWUR15090;AGIWUR36207;AGIWUR43512
CB4856 CT I 3830562 3834124 WBGene00018764 F53F10.2 F53F10.2 -0.199630036615522
AGIWUR2264;AGIWUR6729;AGIWUR9892;AGIWUR13356;AGIWUR14555;AGIWUR19732;AGIWUR23173;AGIWUR25744;AGIWUR26007;AG
IWUR27943;AGIWUR29611;AGIWUR36716;AGIWUR42029
CB4856 CT I 3834115 3837807 WBGene00018763 F53F10.1 F53F10.1 0.352818878753599

AGIWUR22095
CB4856	CT	I	3838237 3839899 WBGene00020187	gsp-4	T03F1.5 0.512086457425774	AGIWUR8768
CB4856	CT	I	3839809 3841558 WBGene00020188	T03F1.6 T03F1.6 0.0283833139655538
AGIWUR6276;AGIWUR15360;AGIWUR19567
CB4856	CT	I	3853810 3854233 WBGene00020192	T03F1.11	T03F1.11	0.342710584981837
AGIWUR41326
CB4856	CT	I	3854169 3859292 WBGene00020189	tfbm-1	T03F1.7 0.0249225224429672	AGIWUR20942
CB4856	CT	I	3859376 3862729 WBGene00020190	guk-1	T03F1.8 -0.0808191603658415
AGIWUR2473;AGIWUR5012;AGIWUR19709;AGIWUR27356
CB4856	CT	I	3862775 3865938 WBGene00001832	hcp-4	T03F1.9 -0.315985675588366
AGIWUR6379;AGIWUR7648;AGIWUR29704;AGIWUR37109
CB4856	CT	I	3867541 3869109 WBGene00020185	pgk-1	T03F1.3 0.095067593161918	AGIWUR13438
CB4856	CT	I	3869214 3870114 WBGene00000764	coq-4	T03F1.2 -0.168930058704499
AGIWUR4077;AGIWUR4428;AGIWUR8265
CB4856	CT	I	3870642 3871267 WBGene00219725	linc-63 T03F1.13	-0.0464600238862065
AGIWUR21463
CB4856	CT	I	3871474 3873428 WBGene00020191	clec-53 T03F1.10	-0.518824490653232
AGIWUR12742
CB4856	CT	I	3874037 3875995 WBGene00020184	uba-5	T03F1.1 -0.109088503265852
AGIWUR13281;AGIWUR14762;AGIWUR40606
CB4856	CT	I	3875974 3876711 WBGene00020193	T03F1.12	T03F1.12	-0.159032215114983
AGIWUR6414;AGIWUR30180;AGIWUR33159;AGIWUR36650;AGIWUR37016
CB4856	CT	I	3877063 3880747 WBGene00016837	C50F2.3 C50F2.3 -0.183841211867257	AGIWUR4648
CB4856	CT	I	3881816 3888313 WBGene00016836	C50F2.2 C50F2.2 0.134212232196866	AGIWUR20836
CB4856	CT	I	3888331 3891598 WBGene00016838	C50F2.4 C50F2.4 -0.239924131917515	AGIWUR21618
CB4856	CT	I	3892332 3895271 WBGene00016835	C50F2.1 C50F2.1 0.118468165773948	AGIWUR30897
CB4856	CT	I	3898046 3899840 WBGene00001430	fkb-5	C50F2.6 0.062976161747434
AGIWUR566;AGIWUR5699;AGIWUR6375;AGIWUR8997;AGIWUR14812;AGIWUR17905;AGIWUR26306
CB4856	CT	I	3900407 3901437 WBGene00016840	C50F2.7 C50F2.7 0.23045901342107	AGIWUR34430
CB4856	CT	I	3901432 3905208 WBGene00016841	magu-3	C50F2.8 0.760054123998707	AGIWUR10248
CB4856	CT	I	3908747 3909701 WBGene00003950	pbs-4	T20F5.2 -0.239906139977653
AGIWUR3637;AGIWUR19846;AGIWUR21083;AGIWUR23708;AGIWUR40592;AGIWUR43430
CB4856	CT	I	3909629 3910544 WBGene00020625	mrrf-1	T20F5.3 0.0399314711423155	AGIWUR14724
CB4856	CT	I	3912635 3915458 WBGene00020626	T20F5.4 T20F5.4 1.32316353240973	AGIWUR17512
CB4856	CT	I	3916064 3916391 WBGene00023142	T20F5.1 T20F5.1 0.00252562096915643	AGIWUR5544
CB4856	CT	I	3917911 3920172 WBGene00020627	T20F5.5 T20F5.5 -0.48373391403711	AGIWUR7752
CB4856	CT	I	3920177 3925162 WBGene00020628	T20F5.6 T20F5.6 -0.0692391648668824
AGIWUR29764;AGIWUR39526;AGIWUR41846
CB4856	CT	I	3925434 3927887 WBGene00020629	T20F5.7 T20F5.7 -0.0607879094028014

AGIWUR1843;AGIWUR5114;AGIWUR7038;AGIWUR9628;AGIWUR12435;AGIWUR24370;AGIWUR34443
CB4856	CT	I	3930830	3931552	WBGene00020653	T21E3.2	T21E3.2	-0.105235862621001	AGIWUR6826
CB4856	CT	I	3932210	3934813	WBGene00020652	egg-4	T21E3.1	-0.392604037838408	AGIWUR3682
CB4856	CT	I	3935272	3953991	WBGene00003072	lrp-2	T21E3.3	0.104401946523295	AGIWUR29102;AGIWUR33825
CB4856	CT	I	3968118	3970474	WBGene00018531	F47B3.7	F47B3.7	0.0122594027470662	AGIWUR2971
CB4856	CT	I	3974063	3977351	WBGene00017909	F28H1.4	F28H1.4	-0.275849487578459	AGIWUR4979
CB4856	CT	I	3978493	3979538	WBGene00017910	F28H1.5	F28H1.5	0.18523349419505	AGIWUR27721
CB4856	CT	I	3983171	3986915	WBGene00000197	aars-2	F28H1.3	-0.256424525430124	AGIWUR5656;AGIWUR5960;AGIWUR22497;AGIWUR31140;AGIWUR35129;AGIWUR42993
CB4856	CT	I	3987842	3988699	WBGene00000779	cpn-3	F28H1.2	-0.231929747521347	AGIWUR1643
CB4856	CT	I	3995709	3996950	WBGene00017908	F28H1.1	F28H1.1	0.503342748342346	AGIWUR6699;AGIWUR23035;AGIWUR27226;AGIWUR33403;AGIWUR36968;AGIWUR44156;AGIWUR44775
CB4856	CT	I	3999348	4031739	WBGene00006805	unc-73	F55C7.7	0.101916017555498	AGIWUR1149;AGIWUR3451;AGIWUR8764;AGIWUR9185;AGIWUR9442;AGIWUR24092;AGIWUR29201;AGIWUR29929;AGIWUR30982;AGIWUR34557;AGIWUR35974;AGIWUR36594;AGIWUR42637
CB4856	CT	I	4035702	4090884	WBGene00006820	unc-89	C09D1.1	-0.211940058809477	AGIWUR6300;AGIWUR7462;AGIWUR9086;AGIWUR10960;AGIWUR10976;AGIWUR12117;AGIWUR13718;AGIWUR19966;AGIWUR21298;AGIWUR28254;AGIWUR29472;AGIWUR32385;AGIWUR33294;AGIWUR33479;AGIWUR43580
CB4856	CT	I	4094179	4098330	WBGene00016066	acd-2	C24G7.4	-0.371547776070718	AGIWUR36711
CB4856	CT	I	4103713	4108045	WBGene00016064	acd-1	C24G7.2	-0.10336749451591	AGIWUR8785;AGIWUR10183
CB4856	CT	I	4109407	4112385	WBGene00016063	delm-2	C24G7.1	-0.0672771517399626	AGIWUR10048;AGIWUR20136
CB4856	CT	I	4114734	4116370	WBGene00000867	cyb-2.2	H31G24.4	-0.269115123875993	AGIWUR32371
CB4856	CT	I	4123481	4124723	WBGene00019253	H31G24.1	H31G24.1	0.236118687902213	AGIWUR24730
CB4856	CT	I	4126056	4129307	WBGene00003029	lin-44	E01A2.3	0.287510334977423	AGIWUR3426;AGIWUR26281;AGIWUR28498;AGIWUR29789;AGIWUR34306;AGIWUR44057
CB4856	CT	I	4129469	4132292	WBGene00002696	let-504	E01A2.4	0.0201789437291424	AGIWUR28032
CB4856	CT	I	4132500	4135239	WBGene00017085	E01A2.2	E01A2.2	-0.105484730748188	AGIWUR606;AGIWUR16300;AGIWUR28446;AGIWUR41107
CB4856	CT	I	4135239	4137074	WBGene00017087	E01A2.5	E01A2.5	0.169123537613531	AGIWUR9789
CB4856	CT	I	4137134	4140634	WBGene00017088	akir-1	E01A2.6	0.079563909965815	AGIWUR15450
CB4856	CT	I	4139514	4142367	WBGene00017084	E01A2.1	E01A2.1	-0.0199170713895853	AGIWUR44336
CB4856	CT	I	4148832	4149429	WBGene00020040	R12E2.15	R12E2.15	-0.33063897613443	AGIWUR12434;AGIWUR26472
CB4856	CT	I	4151099	4151761	WBGene00020032	R12E2.6	R12E2.6	0.0122594353085113	AGIWUR21859

CB4856	CT	I	4153957	4154550	WBGene00020033	R12E2.7	R12E2.7	-0.866397996663792	AGIWUR14061
CB4856	CT	I	4155542	4158733	WBGene00020034	R12E2.8	R12E2.8	-0.176530073153192	AGIWUR2190
CB4856	CT	I	4159014	4161149	WBGene00002137	inx-15	R12E2.9	0.0172627099505469	AGIWUR34283
CB4856	CT	I	4162035	4165015	WBGene00002138	inx-16	R12E2.5	-0.621489151697601	AGIWUR26075
CB4856	CT	I	4165135	4167229	WBGene00002139	inx-17	R12E2.4	-0.322620957941601	AGIWUR9327;AGIWUR17226;AGIWUR20101;AGIWUR22560
CB4856	CT	I	4172433	4173739	WBGene00020036	R12E2.11	R12E2.11	-0.221859794475421	AGIWUR10106
CB4856	CT	I	4173743	4174774	WBGene00020037	mrps-6	R12E2.12	-0.216534224697444	AGIWUR38727
CB4856	CT	I	4175319	4177271	WBGene00004464	rpn-8	R12E2.3	-0.123798078303516	AGIWUR18623;AGIWUR22338
CB4856	CT	I	4177305	4178447	WBGene00020038	R12E2.13	R12E2.13	-0.169593321069536	AGIWUR3754;AGIWUR18414;AGIWUR22505;AGIWUR41014;AGIWUR42132
CB4856	CT	I	4178812	4182158	WBGene00020031	suco-1	R12E2.2	0.0657407334831372	AGIWUR2927
CB4856	CT	I	4182221	4184992	WBGene00020030	R12E2.1	R12E2.1	0.0226493809850865	AGIWUR29439
CB4856	CT	I	4190142	4193129	WBGene00015975	cas-2	C18E3.6	-0.177132603190742	AGIWUR19315
CB4856	CT	I	4193241	4203303	WBGene00015974	C18E3.5	C18E3.5	0.0877011101144163	AGIWUR18123
CB4856	CT	I	4203458	4208537	WBGene00015972	C18E3.3	C18E3.3	0.232569743859703	AGIWUR9106
CB4856	CT	I	4208515	4210341	WBGene00001985	hop-1	C18E3.8	0.012127022846947	AGIWUR15216
CB4856	CT	I	4210979	4212667	WBGene00015971	swsn-2.2	C18E3.2	0.0146698451108964	AGIWUR22115
CB4856	CT	I	4212708	4214317	WBGene00015970	C18E3.1	C18E3.1	0.318019748904275	AGIWUR5693;AGIWUR8501;AGIWUR13498
CB4856	CT	I	4214294	4215768	WBGene00015976	C18E3.9	C18E3.9	-0.410628073355682	AGIWUR20276
CB4856	CT	I	4229577	4236039	WBGene00003516	nab-1	C43E11.6	0.154418182564486	AGIWUR2476;AGIWUR3623;AGIWUR14586;AGIWUR14740;AGIWUR15070;AGIWUR28263;AGIWUR34361;AGIWUR39076
CB4856	CT	I	4239088	4242727	WBGene00003584	ndx-7	C43E11.7	-0.014619945201499	AGIWUR3786
CB4856	CT	I	4242821	4247582	WBGene00016606	exoc-7	C43E11.8	-0.212297851196048	AGIWUR18146
CB4856	CT	I	4247754	4249415	WBGene00007001	tufm-2	C43E11.4	-0.17360056623585З	AGIWUR11352;AGIWUR18532;AGIWUR37882;AGIWUR40266
CB4856	CT	I	4249482	4253170	WBGene00016608	cogc-5	C43E11.11	-0.119164075947737	AGIWUR16563
CB4856	CT	I	4253242	4254139	WBGene00016609	C43E11.12	C43E11.12	-0.0824077733798152	AGIWUR19632
CB4856	CT	I	4254380	4264699	WBGene00016603	met-1	C43E11.3	-0.0981064540768859	AGIWUR2423;AGIWUR5245;AGIWUR8984;AGIWUR14864;AGIWUR15911;AGIWUR26980;AGIWUR38107

CB4856 CT I 4265109 4267238 WBGene00016602 mus-81 C43E11.2 -0.0528263356063903
AGIWUR1293
CB4856 CT I 4267199 4267967 WBGene00016607 C43E11.9 C43E11.9 -0.0476253337558266
AGIWUR3376;AGIWUR11694
CB4856 CT I 4268070 4270287 WBGene00000382 cdc-6 C43E11.10 -0.128698384342623
AGIWUR23938
CB4856 CT I 4270697 4273332 WBGene00016601 acin-1 C43E11.1 -0.0659104067574052
AGIWUR26821
CB4856 CT I 4273896 4274742 WBGene00021631 Y47D9A.4 Y47D9A.4 -0.66608621710906
AGIWUR20658
CB4856 CT I 4277684 4279622 WBGene00021632 Y47D9A.5 Y47D9A.5 -0.122551722733927
AGIWUR13379
CB4856 CT I 4298184 4302243 WBGene00021629 scpl-3 Y47D9A.2 0.253087634051961
AGIWUR34572;AGIWUR41718
CB4856 CT I 4303930 4308244 WBGene00021628 Y47D9A.1 Y47D9A.1 -0.0625774843612383
AGIWUR1935;AGIWUR8700;AGIWUR37027
CB4856 CT I 4321095 4359080 WBGene00000140 anc-1 ZK973.6 0.0531927372678282
AGIWUR2453;AGIWUR11299;AGIWUR11875;AGIWUR21024;AGIWUR28673;AGIWUR37202;AGIWUR38158
CB4856 CT I 4359207 4362548 WBGene00000045 acr-6 ZK973.5 -0.13355214823989 AGIWUR12825
CB4856 CT I 4363415 4364048 WBGene00022833 ZK973.4 ZK973.4 -0.313762002873418 AGIWUR43472
CB4856 CT I 4364906 4367049 WBGene00022834 ZK973.8 ZK973.8 0.0061863486944187 AGIWUR38768
CB4856 CT I 4368089 4370504 WBGene00022832 pdp-1 ZK973.3 0.190861994112943 AGIWUR38319
CB4856 CT I 4370419 4372138 WBGene00022835 ZK973.9 ZK973.9 -0.0525223740092731 AGIWUR26896
CB4856 CT I 4372255 4373018 WBGene00003061 lpd-5 ZK973.10 -0.271179746125171
AGIWUR9768
CB4856 CT I 4373656 4379329 WBGene00022831 cec-10 ZK973.2 0.158534303879131 AGIWUR11355
CB4856 CT I 4379325 4381948 WBGene00022836 ZK973.11 ZK973.11 0.0833215339901123
AGIWUR40438
CB4856 CT I 4382565 4385218 WBGene00022830 ZK973.1 ZK973.1 -0.137639594732654 AGIWUR15329
CB4856 CT I 4386785 4401980 WBGene00000962 dhc-1 T21E12.4 0.0103071568651441
AGIWUR25546;AGIWUR28865
CB4856 CT I 4401956 4404585 WBGene00020655 T21E12.3 T21E12.3 -0.156160850439187
AGIWUR37344
CB4856 CT I 4409689 4426137 WBGene00015704 tiam-1 C11D9.1 0.111957008303431
AGIWUR16;AGIWUR16270
CB4856 CT I 4428467 4444342 WBGene00016562 C41D11.3 C41D11.3 -0.0895572307788859
AGIWUR18411;AGIWUR25427;AGIWUR32861;AGIWUR34009
CB4856 CT I 4444719 4446598 WBGene00001231 eif-3.H C41D11.2 -0.213089361405377
AGIWUR21203

CB4856	CT	I	4446577	4448461	WBGene00000787	cps-6	C41D11.8	0.336431945340545	AGIWUR1461;AGIWUR12582;AGIWUR32366
CB4856	CT	I	4448576	4449774	WBGene00016567	C41D11.9	C41D11.9	0.375274082547767	AGIWUR9473
CB4856	CT	I	4449879	4450927	WBGene00016564	cmt-1	C41D11.5	0.041054532784365	AGIWUR43287
CB4856	CT	I	4451200	4456805	WBGene00016566	eri-7	C41D11.7	-0.120288494990621	AGIWUR27075
CB4856	CT	I	4457383	4469461	WBGene00016561	eri-6	C41D11.1	0.0975816157706078	AGIWUR9474;AGIWUR15095;AGIWUR33241
CB4856	CT	I	4481203	4508930	WBGene00020131	gcy-28	T01A4.1	-0.0198571224630317	AGIWUR8023;AGIWUR9811;AGIWUR10432;AGIWUR11693;AGIWUR25389;AGIWUR29324
CB4856	CT	I	4508917	4511126	WBGene00022470	mrpl-19	Y119C1B.4	-0.419799385617026	AGIWUR28354
CB4856	CT	I	4511440	4516512	WBGene00022471	Y119C1B.5	Y119C1B.5	0.0275004584742859	AGIWUR39560
CB4856	CT	I	4517260	4518491	WBGene00022472	Y119C1B.6	Y119C1B.6	0.160621870957328	AGIWUR7587;AGIWUR14994;AGIWUR16399;AGIWUR27205;AGIWUR37427;AGIWUR38985
CB4856	CT	I	4520070	4522768	WBGene00044754	Y119C1B.12	Y119C1B.12	-0.308188335514243	AGIWUR5460;AGIWUR27225;AGIWUR42488
CB4856	CT	I	4526232	4534798	WBGene00022473	bet-1	Y119C1B.8	0.018455064593633	AGIWUR1031;AGIWUR3966;AGIWUR43474
CB4856	CT	I	4537449	4539725	WBGene00022474	zig-9	Y119C1B.9	0.0432380028726165	AGIWUR17606
CB4856	CT	I	4549695	4552758	WBGene00022469	Y119C1B.3	Y119C1B.3	0.327759676530587	AGIWUR19268
CB4856	CT	I	4552851	4553121	WBGene00044441	Y119C1B.11	Y119C1B.11	-0.00467143726293706	AGIWUR4602;AGIWUR7834;AGIWUR9928;AGIWUR28645;AGIWUR31236
CB4856	CT	I	4554126	4555203	WBGene00022467	Y119C1B.1	Y119C1B.1	0.0535291047506128	AGIWUR24218
CB4856	CT	I	4555343	4556814	WBGene00022475	Y119C1B.10	Y119C1B.10	-0.233870211503442	AGIWUR39830
CB4856	CT	I	4557664	4563694	WBGene00017010	D1007.15	D1007.15	0.117004959188917	AGIWUR9638
CB4856	CT	I	4564035	4565976	WBGene00004113	pqn-24	D1007.14	-0.00521783929903208	AGIWUR5942
CB4856	CT	I	4566495	4568385	WBGene00017009	D1007.13	D1007.13	0.0388561249330106	AGIWUR36815
CB4856	CT	I	4568962	4570027	WBGene00000440	ceh-17	D1007.1	0.282638234002125	AGIWUR7465

CB4856	CT	I	4571214	4572787	WBGene00000629	col-52	D1007.2	-0.0211373660297234	AGIWUR32251
CB4856	CT	I	4575782	4577204	WBGene00017001	D1007.3	D1007.3	-0.110436016131935	AGIWUR11775
CB4856	CT	I	4578695	4579740	WBGene00044421	D1007.18	D1007.18	-0.00262440781602802	AGIWUR1057;AGIWUR2948;AGIWUR11364;AGIWUR23273;AGIWUR28118
CB4856	CT	I	4579964	4580601	WBGene00017002	D1007.4	D1007.4	-0.190537047282378	AGIWUR33335
CB4856	CT	I	4580692	4583810	WBGene00017003	D1007.5	D1007.5	-0.0135891563972645	AGIWUR6591;AGIWUR13479
CB4856	CT	I	4583873	4584505	WBGene00004479	rps-10	D1007.6	-0.433444783817096	AGIWUR23072;AGIWUR34651
CB4856	CT	I	4585115	4586177	WBGene00004436	rpl-24.1	D1007.12	-0.0777814359116178	AGIWUR2234;AGIWUR7117;AGIWUR7732;AGIWUR16379;AGIWUR17943;AGIWUR18712;AGIWUR21241;AGIWUR28846;AGIWUR31144;AGIWUR31405;AGIWUR32422;AGIWUR32535
CB4856	CT	I	4586273	4589463	WBGene00017007	D1007.10	D1007.10	-0.0899970090407516	AGIWUR22139;AGIWUR22225
CB4856	CT	I	4589380	4589899	WBGene00045048	D1007.19	D1007.19	0.237470314839701	AGIWUR9696;AGIWUR15492;AGIWUR15856;AGIWUR24796;AGIWUR32400
CB4856	CT	I	4590430	4591587	WBGene00017006	D1007.9	D1007.9	-0.0335534231065795	AGIWUR6994
CB4856	CT	I	4597914	4602666	WBGene00017004	nrd-1	D1007.7	-0.306483574155251	AGIWUR12783;AGIWUR16196;AGIWUR18660;AGIWUR21356;AGIWUR22525;AGIWUR37310
CB4856	CT	I	4603217	4604196	WBGene00017011	eaf-1	D1007.16	-0.0415780560455516	AGIWUR42215
CB4856	CT	I	4604175	4605385	WBGene00017005	D1007.8	D1007.8	-0.174985788123838	AGIWUR31149
CB4856	CT	I	4605655	4610202	WBGene00016654	C44E4.5	C44E4.5	-0.24820934391383	AGIWUR38528;AGIWUR40268
CB4856	CT	I	4610147	4633377	WBGene00016650	ubr-4	C44E4.1	-0.0776303888677674	AGIWUR26053;AGIWUR26807;AGIWUR44636
CB4856	CT	I	4634103	4635620	WBGene00016653	C44E4.4	C44E4.4	-0.0859871899102993	AGIWUR10614;AGIWUR20679
CB4856	CT	I	4635722	4637328	WBGene00016655	acbp-1	C44E4.6	-0.114222196899501	AGIWUR39810
CB4856	CT	I	4637432	4640882	WBGene00016652	got-2.1	C44E4.3	0.124475025748346	AGIWUR9876
CB4856	CT	I	4641003	4644069	WBGene00022466	Y119C1A.1	Y119C1A.1	-0.0658707175583242	AGIWUR36777
CB4856	CT	I	4644677	4645657	WBGene00015008	lmd-2	B0041.3	-0.13025827205189	AGIWUR22463
CB4856	CT	I	4645641	4646963	WBGene00004415	rpl-4	B0041.4	0.0684559733359347	AGIWUR1725;AGIWUR9422;AGIWUR14581;AGIWUR19810;AGIWUR33413;AGIWUR42912
CB4856	CT	I	4647280	4648275	WBGene00015011	B0041.8	B0041.8	-0.374182733307293	AGIWUR864;AGIWUR12611;AGIWUR13671;AGIWUR30350
CB4856	CT	I	4648272	4650770	WBGene00015009	B0041.5	B0041.5	-0.154475321691763	AGIWUR43682
CB4856	CT	I	4652145	4652906	WBGene00015010	ptps-1	B0041.6	-0.416767574594813	

AGIWUR1589;AGIWUR18373;AGIWUR27336;AGIWUR28856;AGIWUR39809;AGIWUR42507
CB4856 CT I 4653471 4660151 WBGene00015007 ain-2 B0041.2 -0.0419044904957966
AGIWUR108;AGIWUR2251;AGIWUR8966;AGIWUR12167;AGIWUR12517;AGIWUR13698;AGIWUR29300;AGIWUR30748;AGIWUR30865;AGI
WUR32722
CB4856 CT I 4665744 4671738 WBGene00006961 xnp-1 B0041.7 0.0472936992653539
AGIWUR6803;AGIWUR7645;AGIWUR8360;AGIWUR9104;AGIWUR18059;AGIWUR25526;AGIWUR26185;AGIWUR26197;AGIWUR32396;AGI
WUR34797;AGIWUR35523;AGIWUR41946
CB4856 CT I 4675135 4679409 WBGene00006791 unc-57 T04D1.3 -0.0467189442808014
AGIWUR19596;AGIWUR20386;AGIWUR30589
CB4856 CT I 4679671 4682755 WBGene00020212 T04D1.2 T04D1.2 0.313578309838137 AGIWUR20292
CB4856 CT I 4685865 4698575 WBGene00007053 chd-7 T04D1.4 0.430103215283054 AGIWUR42196
CB4856 CT I 4704598 4705881 WBGene00021112 W09C3.4 W09C3.4 -0.279664999199428 AGIWUR1005
CB4856 CT I 4706682 4708762 WBGene00021111 W09C3.3 W09C3.3 -0.22092121398129 AGIWUR16434
CB4856 CT I 4709262 4710712 WBGene00021113 gsp-3 W09C3.6 -0.0743516425222417
AGIWUR17793;AGIWUR31790
CB4856 CT I 4711414 4713572 WBGene00021110 W09C3.2 W09C3.2 0.145507871942139 AGIWUR13150
CB4856 CT I 4714320 4715485 WBGene00021114 W09C3.7 W09C3.7 0.511736755693786 AGIWUR37490
CB4856 CT I 4716059 4717441 WBGene00021115 W09C3.8 W09C3.8 0.103700955531735 AGIWUR9668
CB4856 CT I 4719789 4721098 WBGene00015696 C10H11.7 C10H11.7 -0.187230627651694
AGIWUR22151
CB4856 CT I 4721131 4723960 WBGene00015695 ugt-26 C10H11.6 -0.463420145411625
AGIWUR8772
CB4856 CT I 4724065 4726779 WBGene00015694 ugt-27 C10H11.5 -0.0850847629394869
AGIWUR3550;AGIWUR34887;AGIWUR35735
CB4856 CT I 4727280 4730366 WBGene00015693 ugt-28 C10H11.4 -0.18057200496044
AGIWUR10641;AGIWUR42541
CB4856 CT I 4731603 4735276 WBGene00015692 ugt-25 C10H11.3 -0.311316624263569
AGIWUR33026
CB4856 CT I 4735471 4738100 WBGene00015697 mlst-8 C10H11.8 -0.0794181263466029
AGIWUR33168
CB4856 CT I 4738171 4744321 WBGene00002694 let-502 C10H11.9 0.0846048631384484
AGIWUR13661;AGIWUR36987
CB4856 CT I 4747490 4759601 WBGene00015691 viln-1 C10H11.1 -0.117618692973389
AGIWUR26405
CB4856 CT I 4759820 4761321 WBGene00015698 kca-1 C10H11.10 -0.0776273428720531
AGIWUR10472;AGIWUR41596
CB4856 CT I 4769694 4771358 WBGene00019767 rpa-2 M04F3.1 -0.0269699387442692 AGIWUR40091
CB4856 CT I 4771348 4773673 WBGene00019770 M04F3.4 M04F3.4 0.0164854721296539
AGIWUR6019;AGIWUR14997;AGIWUR27654;AGIWUR28372;AGIWUR32364

CB4856	CT	I	4773818 4788358 WBGene00019771	M04F3.5 M04F3.5 0.00726790654891829
AGIWUR32895;AGIWUR36661
CB4856	CT	I	4789190 4789592 WBGene00023067	rpl-41.1	F54D7.6 0.0811010597171293
AGIWUR16924
CB4856	CT	I	4790292 4790649 WBGene00023068	rpl-41.2	F54D7.7 -0.0246300828454622
AGIWUR5284;AGIWUR28223
CB4856	CT	I	4790644 4792560 WBGene00006984	zig-7	F54D7.4 0.485367882834247	AGIWUR1938
CB4856	CT	I	4800043 4804268 WBGene00018798	gnrr-1	F54D7.3 0.154199563320649	AGIWUR35863
CB4856	CT	I	4805607 4809674 WBGene00018797	F54D7.2 F54D7.2 -0.1899661470787
AGIWUR13252;AGIWUR32663;AGIWUR38228;AGIWUR41575
CB4856	CT	I	4821933 4824811 WBGene00019012	F57C9.6 F57C9.6 0.209016946925906	AGIWUR8277
CB4856	CT	I	4824921 4826633 WBGene00019013	tofu-4	F57C9.7 -0.314227124219803
AGIWUR17499;AGIWUR26308
CB4856	CT	I	4827072 4829794 WBGene00002034	htp-3	F57C9.5 0.0449151586873704	AGIWUR12950
CB4856	CT	I	4830135 4835619 WBGene00019011	F57C9.4 F57C9.4 -0.196038817940268
AGIWUR3295;AGIWUR5793;AGIWUR12169;AGIWUR24527;AGIWUR27565;AGIWUR37268
CB4856	CT	I	4836187 4844966 WBGene00019010	glo-2	F57C9.3 -0.139113319012106	AGIWUR34322
CB4856	CT	I	4845013 4847336 WBGene00019009	clec-90 F57C9.2 -0.0799294681176916
AGIWUR3253;AGIWUR3898;AGIWUR4431;AGIWUR28280;AGIWUR28715;AGIWUR32897
CB4856	CT	I	4848425 4850717 WBGene00019008	pdxk-1	F57C9.1 -0.0466619797273156
AGIWUR4151;AGIWUR8744;AGIWUR38672;AGIWUR42153;AGIWUR43420;AGIWUR43542
CB4856	CT	I	4850682 4867825 WBGene00015303	rga-6	C01F4.2 0.0872419997484677
AGIWUR6175;AGIWUR8844
CB4856	CT	I	4884903 4895495 WBGene00017795	F25F8.1 F25F8.1 0.300349529475378	AGIWUR32285
CB4856	CT	I	4896003 4897541 WBGene00017674	F21F3.4 F21F3.4 -0.23659794662521	AGIWUR17618
CB4856	CT	I	4897620 4900977 WBGene00006774	unc-38	F21F3.5 -0.0164939733081475	AGIWUR19032
CB4856	CT	I	4901918 4903264 WBGene00017673	icmt-1	F21F3.3 1.1422043882992 AGIWUR10653
CB4856	CT	I	4903437 4905138 WBGene00017672	F21F3.2 F21F3.2 -0.142617274803379	AGIWUR36704
CB4856	CT	I	4906786 4911829 WBGene00017671	pgal-1	F21F3.1 0.170087825840415
AGIWUR10360;AGIWUR11221;AGIWUR39787
CB4856	CT	I	4911805 4914047 WBGene00017675	F21F3.6 F21F3.6 0.112899154920743
AGIWUR6443;AGIWUR19391;AGIWUR23423;AGIWUR28728
CB4856	CT	I	4918372 4920770 WBGene00017676	F21F3.7 F21F3.7 0.0907713083755004
AGIWUR4819;AGIWUR10338;AGIWUR11236;AGIWUR12715;AGIWUR18328;AGIWUR22480;AGIWUR36384;AGIWUR42305
CB4856	CT	I	4920707 4926233 WBGene00002075	imb-1	F28B3.8 0.0272897029755317
AGIWUR17237;AGIWUR19494;AGIWUR21477;AGIWUR23298;AGIWUR26174;AGIWUR40448
CB4856	CT	I	4926592 4928840 WBGene00017886	F28B3.5 F28B3.5 -0.353266033800613
AGIWUR3834;AGIWUR29211
CB4856	CT	I	4928654 4932147 WBGene00017888	acl-11	F28B3.9 -0.296207117697278	AGIWUR26348

CB4856 CT I 4934118 4949332 WBGene00017885 dip-2 F28B3.4 -0.0347983165237649
AGIWUR3431;AGIWUR18607;AGIWUR29662;AGIWUR33088;AGIWUR34513;AGIWUR42148
CB4856 CT I 4949327 4950410 WBGene00017889 F28B3.10 F28B3.10 -0.0882019400314533
AGIWUR37298
CB4856 CT I 4950583 4951550 WBGene00017887 F28B3.6 F28B3.6 -0.0738548294411387
AGIWUR14419;AGIWUR15223;AGIWUR19224;AGIWUR29297;AGIWUR31798;AGIWUR41653
CB4856 CT I 4951570 4957667 WBGene00001860 him-1 F28B3.7 -0.177510872740829 AGIWUR5462
CB4856 CT I 4957776 4958309 WBGene00044431 W03G9.8 W03G9.8 -0.243410610389883
AGIWUR521;AGIWUR2774;AGIWUR10077;AGIWUR19733;AGIWUR42359
CB4856 CT I 4962451 4964745 WBGene00021014 W03G9.2 W03G9.2 -0.0115969048627953 AGIWUR17245
CB4856 CT I 4964667 4967089 WBGene00000155 app-1 W03G9.4 0.114682397020083 AGIWUR31837
CB4856 CT I 4968007 4970245 WBGene00021016 W03G9.5 W03G9.5 0.0232091018120979 AGIWUR898
CB4856 CT I 4969923 4972243 WBGene00044432 W03G9.9 W03G9.9 0.0483884152395655
AGIWUR4097;AGIWUR15675
CB4856 CT I 4980307 4983958 WBGene00004900 snf-1 W03G9.1 0.116621295099279 AGIWUR37640
CB4856 CT I 4984760 4986739 WBGene00003906 paf-1 W03G9.6 0.0268633134008471 AGIWUR30136
CB4856 CT I 4986954 4989454 WBGene00021017 cil-7 W03G9.7 0.180321885175964 AGIWUR41717
CB4856 CT I 4989568 4994675 WBGene00001117 dyf-1 F54C1.5 0.257387335924801 AGIWUR4202
CB4856 CT I 4996839 5003161 WBGene00003221 mes-3 F54C1.3 -0.0467035117246191
AGIWUR28141;AGIWUR30866;AGIWUR40884
CB4856 CT I 5003351 5005163 WBGene00001050 dom-3 F54C1.2 -0.217752645454485 AGIWUR4782
CB4856 CT I 5004924 5006057 WBGene00018792 F54C1.8 F54C1.8 0.176602491310315
AGIWUR8345;AGIWUR9536;AGIWUR21501;AGIWUR36845
CB4856 CT I 5006706 5007991 WBGene00006058 sst-20 F54C1.9 0.397969032640084 AGIWUR11233
CB4856 CT I 5008770 5013581 WBGene00018789 F54C1.1 F54C1.1 0.112815828674114 AGIWUR29730
CB4856 CT I 5014351 5016260 WBGene00018791 F54C1.6 F54C1.6 0.204915200780745 AGIWUR27151
CB4856 CT I 5018770 5020166 WBGene00003934 pat-10 F54C1.7 0.0463013701796324
AGIWUR6495;AGIWUR16624;AGIWUR26202;AGIWUR38199
CB4856 CT I 5020748 5023210 WBGene00016730 C46H11.6 C46H11.6 -0.24103905487352
AGIWUR39021
CB4856 CT I 5023889 5041149 WBGene00002979 lfe-2 C46H11.4 0.210749156430199
AGIWUR527;AGIWUR15498;AGIWUR15600;AGIWUR22329;AGIWUR39734
CB4856 CT I 5041492 5042055 WBGene00016729 C46H11.3 C46H11.3 -0.1861644665039
AGIWUR2153;AGIWUR17010;AGIWUR19831;AGIWUR31265;AGIWUR41786
CB4856 CT I 5042102 5043453 WBGene00016731 C46H11.7 C46H11.7 0.0573536815279763
AGIWUR10507
CB4856 CT I 5044201 5045720 WBGene00016732 phat-1 C46H11.8 0.0409866651787407
AGIWUR10717
CB4856 CT I 5050040 5052523 WBGene00016728 C46H11.2 C46H11.2 0.769209671506283

AGIWUR43572
CB4856	CT	I	5052529 5054960 WBGene00016734	C46H11.10	C46H11.10	0.184400387793455
AGIWUR25478
CB4856	CT	I	5055686 5056039 WBGene00016727	C46H11.1	C46H11.1	-0.0284946966970545
AGIWUR1249;AGIWUR10642;AGIWUR11922;AGIWUR15339;AGIWUR28513;AGIWUR41170
CB4856	CT	I	5058251 5068829 WBGene00016735	fhod-1	C46H11.11	0.365473919689567
AGIWUR2364
CB4856	CT	I	5079911 5082279 WBGene00016258	vha-16	C30F8.2 -0.018930925265078
AGIWUR9041;AGIWUR12777;AGIWUR38333
CB4856	CT	I	5086317 5093793 WBGene00002213	kin-32	C30F8.4 0.251699918039636	AGIWUR30640
CB4856	CT	I	5096601 5100579 WBGene00016259	C30F8.3 C30F8.3 0.0336142533661103	AGIWUR38945
CB4856	CT	I	5100751 5101674 WBGene00022465	ift-20	Y110A7A.20	0.647781906773554
AGIWUR43171
CB4856	CT	I	5101682 5103467 WBGene00004959	spe-5	Y110A7A.12	0.284839997583971
AGIWUR45082
CB4856	CT	I	5103581 5104956 WBGene00000502	chp-1	Y110A7A.13	-0.344626866259972
AGIWUR23337
CB4856	CT	I	5105110 5106204 WBGene00003924	pas-3	Y110A7A.14	-0.229331562230159
AGIWUR17561;AGIWUR38486;AGIWUR39496;AGIWUR41863
CB4856	CT	I	5106204 5107625 WBGene00022460	use-1	Y110A7A.11	-0.236594000025288
AGIWUR8138;AGIWUR10961;AGIWUR39888
CB4856	CT	I	5107833 5110183 WBGene00000001	aap-1	Y110A7A.10	-0.0431859878744856
AGIWUR5137
CB4856	CT	I	5110075 5111154 WBGene00045212	Y110A7A.21	Y110A7A.21	0.0802448245583172
AGIWUR7396
CB4856	CT	I	5114119 5118852 WBGene00022463	elpc-1	Y110A7A.16	0.264331692225483
AGIWUR39107
CB4856	CT	I	5119223 5123985 WBGene00022459	Y110A7A.9	Y110A7A.9	0.0848651088109467
AGIWUR5638;AGIWUR13186;AGIWUR37146
CB4856	CT	I	5123916 5127199 WBGene00003132	mat-1	Y110A7A.17	0.0685089891368271
AGIWUR10232;AGIWUR10763;AGIWUR11746;AGIWUR18147;AGIWUR21523
CB4856	CT	I	5127828 5129611 WBGene00022458	prp-31	Y110A7A.8	-0.267925876727897
AGIWUR13052
CB4856	CT	I	5129640 5131065 WBGene00022457	Y110A7A.7	Y110A7A.7	0.286167944214295
AGIWUR36758
CB4856	CT	I	5131288 5134101 WBGene00022456	pfkb-1.1	Y110A7A.6	-0.106734863046361
AGIWUR12324;AGIWUR19557;AGIWUR24616;AGIWUR42679
CB4856	CT	I	5134709 5137971 WBGene00003475	mtm-1	Y110A7A.5	-0.0871759708398514
AGIWUR8180

CB4856	CT	I	5138256	5139382	WBGene00022455	tyms-1	Y110A7A.4	-0.0991267595810488	AGIWUR26036;AGIWUR30844;AGIWUR32359
CB4856	CT	I	5141675	5149226	WBGene00006797	unc-63	Y110A7A.3	-0.2669233419145	AGIWUR19692
CB4856	CT	I	5154529	5158467	WBGene00022454	Y110A7A.2	Y110A7A.2	0.407604697257714	AGIWUR12450
CB4856	CT	I	5158511	5162231	WBGene00004094	ppw-2	Y110A7A.18	0.100190017052046	AGIWUR38604
CB4856	CT	I	5165152	5171313	WBGene00001833	hcp-6	Y110A7A.1	-0.320314839403	AGIWUR23546;AGIWUR27668;AGIWUR29091
CB4856	CT	I	5171285	5176664	WBGene00004955	spd-5	F56A3.4	0.0719069667356695	AGIWUR43280
CB4856	CT	I	5176466	5182801	WBGene00003792	npp-6	F56A3.3	-0.124131513145992	AGIWUR31488;AGIWUR37695
CB4856	CT	I	5182736	5186234	WBGene00018909	slx-1	F56A3.2	-0.0424174711677612	AGIWUR23385
CB4856	CT	I	5186926	5188995	WBGene00000272	bro-1	F56A3.5	0.254288965398798	AGIWUR18690
CB4856	CT	I	5190142	5195243	WBGene00018908	fncm-1	F56A3.1	-0.109338005066215	AGIWUR16044
CB4856	CT	I	5196078	5200102	WBGene00016328	pde-5	C32E12.2	-0.0134277710278363	AGIWUR4483;AGIWUR25559;AGIWUR44681
CB4856	CT	I	5200258	5203504	WBGene00016329	osr-1	C32E12.3	0.186321854046977	AGIWUR37480
CB4856	CT	I	5204540	5215218	WBGene00004771	sem-2	C32E12.5	-0.220801331978289	AGIWUR14738;AGIWUR27347
CB4856	CT	I	5230549	5242607	WBGene00016330	C32E12.4	C32E12.4	0.453014837994868	AGIWUR34566
CB4856	CT	I	5244283	5245402	WBGene00015092	mrpl-47	B0261.4	-0.146073263290331	AGIWUR25197
CB4856	CT	I	5245743	5263413	WBGene00002583	let-363	B0261.2	0.249614194714738	AGIWUR44044
CB4856	CT	I	5263694	5268380	WBGene00015091	B0261.1	B0261.1	0.0820317181452008	AGIWUR18289
CB4856	CT	I	5269545	5270941	WBGene00015095	B0261.7	B0261.7	-0.0734029484156125	AGIWUR8322
CB4856	CT	I	5271265	5272037	WBGene00015096	B0261.8	B0261.8	-0.0414573387603632	AGIWUR2556
CB4856	CT	I	5272846	5274339	WBGene00015312	C01G8.1	C01G8.1	-0.183117748657143	AGIWUR694;AGIWUR16178;AGIWUR34561
CB4856	CT	I	5274693	5278708	WBGene00000540	cln-3.2	C01G8.2	0.128969900247302	AGIWUR30422
CB4856	CT	I	5278803	5281526	WBGene00000965	dhs-1	C01G8.3	-0.180902432920584	AGIWUR15951
CB4856	CT	I	5282085	5283944	WBGene00001022	dnj-4	C01G8.4	-0.0847329937331196	AGIWUR42
CB4856	CT	I	5284062	5291604	WBGene00001333	erm-1	C01G8.5	0.160983205576571	AGIWUR16002
CB4856	CT	I	5298755	5300331	WBGene00015313	hpo-32	C01G8.6	-0.0428538861162586	AGIWUR26468
CB4856	CT	I	5300901	5317392	WBGene00002717	let-526	C01G8.9	0.358589045618327	AGIWUR7817;AGIWUR8550;AGIWUR8678;AGIWUR20851;AGIWUR25435;AGIWUR30166;AGIWUR37870
CB4856	CT	I	5317491	5318124	WBGene00018598	F48C1.5	F48C1.5	-0.195323644951785	AGIWUR33414

CB4856	CT	I	5318225	5319301	WBGene00018599	F48C1.6	F48C1.6	-0.251279943069536	AGIWUR37907
CB4856	CT	I	5319720	5321117	WBGene00018595	fbxa-145	F48C1.2	-0.531502495949027	AGIWUR1383
CB4856	CT	I	5324511	5330222	WBGene00018594	aman-3	F48C1.1	0.0353125456695169	AGIWUR25737;AGIWUR28725;AGIWUR31540
CB4856	CT	I	5330578	5332194	WBGene00004965	spe-11	F48C1.7	0.153034433135264	AGIWUR39988
CB4856	CT	I	5332249	5332780	WBGene00018601	F48C1.9	F48C1.9	1.44855835642631	AGIWUR10340
CB4856	CT	I	5338922	5342012	WBGene00018865	F55A12.6	F55A12.6	-0.111144992799664	AGIWUR15674
CB4856	CT	I	5342180	5344724	WBGene00000150	apm-1	F55A12.7	0.194030747544854	AGIWUR9311
CB4856	CT	I	5345927	5349657	WBGene00018864	F55A12.5	F55A12.5	-0.0439480565297252	AGIWUR43597
CB4856	CT	I	5349646	5353767	WBGene00018866	nath-10	F55A12.8	-0.0426463514463933	AGIWUR6506
CB4856	CT	I	5354160	5357026	WBGene00000966	dhs-2	F55A12.4	0.0392240640433563	AGIWUR6834;AGIWUR39323
CB4856	CT	I	5358522	5365170	WBGene00004087	ppk-1	F55A12.3	0.220622938221817	AGIWUR9665
CB4856	CT	I	5365265	5366875	WBGene00018863	F55A12.2	F55A12.2	-0.223790224520139	AGIWUR5975;AGIWUR16690;AGIWUR23746
CB4856	CT	I	5366944	5367829	WBGene00018867	F55A12.10	F55A12.10	0.118691951026339	AGIWUR25431
CB4856	CT	I	5368478	5372643	WBGene00018862	wago-2	F55A12.1	-0.175199483258412	AGIWUR28246
CB4856	CT	I	5372854	5384508	WBGene00004131	pqn-44	F55A12.9	-0.112184481491827	AGIWUR896;AGIWUR27262
CB4856	CT	I	5384639	5385737	WBGene00016042	C24A11.5	C24A11.5	-0.203765415459014	AGIWUR7145
CB4856	CT	I	5396269	5396503	WBGene00016043	C24A11.6	C24A11.6	0.144189670103115	AGIWUR14300
CB4856	CT	I	5401757	5402896	WBGene00016040	C24A11.1	C24A11.1	0.0704996506257487	AGIWUR5355
CB4856	CT	I	5402889	5408344	WBGene00001491	frm-4	C24A11.8	0.010401657932964	AGIWUR981;AGIWUR4212;AGIWUR6365;AGIWUR15596;AGIWUR16887;AGIWUR31981
CB4856	CT	I	5412841	5415629	WBGene00000761	coq-1	C24A11.9	-0.1534424240836	AGIWUR43800
CB4856	CT	I	5416649	5420731	WBGene00016278	C30H7.2	C30H7.2	0.00739910633863694	AGIWUR21269
CB4856	CT	I	5421313	5428457	WBGene00017855	F27C1.6	F27C1.6	-0.231954492774721	AGIWUR3947

CB4856	CT	I	5428469	5429908	WBGene00000230	atp-3	F27C1.7	0.0132764974680244	AGIWUR4068;AGIWUR34961
CB4856	CT	I	5430164	5431449	WBGene00017852	F27C1.2	F27C1.2	0.0264740358539575	AGIWUR2440;AGIWUR34813
CB4856	CT	I	5432158	5433054	WBGene00001067	dpy-5	F27C1.8	-0.341613860967373	AGIWUR27120;AGIWUR44335
CB4856	CT	I	5433907	5443026	WBGene00017858	F27C1.11	F27C1.11	-0.136714231647839	AGIWUR5701;AGIWUR9333;AGIWUR22774;AGIWUR27305;AGIWUR27976
CB4856	CT	I	5443878	5446855	WBGene00017860	F27C1.13	F27C1.13	0.0913776078037967	AGIWUR6830;AGIWUR24304;AGIWUR35087;AGIWUR39993;AGIWUR41117;AGIWUR42850
CB4856	CT	I	5455730	5459550	WBGene00020262	ferl-1	T05E8.1	0.170872428459797	AGIWUR22748
CB4856	CT	I	5463480	5469627	WBGene00001859	hil-8	T05E8.2	-0.0674555515838922	AGIWUR8595;AGIWUR8928;AGIWUR15131;AGIWUR28796;AGIWUR36374
CB4856	CT	I	5472321	5479438	WBGene00020263	let-355	T05E8.3	-0.126954927869033	AGIWUR30390
CB4856	CT	I	5479550	5484643	WBGene00015635	C09D4.4	C09D4.4	0.27762841781589	AGIWUR10054
CB4856	CT	I	5484779	5486354	WBGene00004431	rpl-19	C09D4.5	0.0567920357888099	AGIWUR34311
CB4856	CT	I	5486487	5487150	WBGene00015636	C09D4.6	C09D4.6	0.00824576323056991	AGIWUR45087
CB4856	CT	I	5488309	5490626	WBGene00015634	C09D4.3	C09D4.3	-0.0454273616923011	AGIWUR5653
CB4856	CT	I	5491424	5493263	WBGene00015633	C09D4.2	C09D4.2	0.486264183386119	AGIWUR16931
CB4856	CT	I	5497679	5505447	WBGene00015632	C09D4.1	C09D4.1	0.039295527882425	AGIWUR27450
CB4856	CT	I	5505377	5507023	WBGene00019076	mrpl-24	F59A3.3	0.0562198288030231	AGIWUR34650
CB4856	CT	I	5507123	5509288	WBGene00019077	zipt-11	F59A3.4	0.266839126106852	AGIWUR5874;AGIWUR13042;AGIWUR14294;AGIWUR18200
CB4856	CT	I	5509473	5511705	WBGene00019079	rpa-4	F59A3.6	-0.220860233183487	AGIWUR22494;AGIWUR27515;AGIWUR32418;AGIWUR38402
CB4856	CT	I	5512048	5514557	WBGene00019075	F59A3.2	F59A3.2	-0.231605843389703	AGIWUR6715
CB4856	CT	I	5514651	5516025	WBGene00185086	F59A3.12	F59A3.12	-0.118535491326982	AGIWUR27384
CB4856	CT	I	5520389	5529763	WBGene00019074	cpd-1	F59A3.1	-0.107633068006328	AGIWUR832;AGIWUR9804;AGIWUR16154;AGIWUR23805;AGIWUR36548;AGIWUR40265;AGIWUR40556
CB4856	CT	I	5530618	5533710	WBGene00019082	pup-3	F59A3.9	-0.428914690212974	AGIWUR5052
CB4856	CT	I	5542630	5543334	WBGene00019083	F59A3.10	F59A3.10	-0.0742769906259522	AGIWUR23620
CB4856	CT	I	5546120	5561642	WBGene00006594	tom-1	M01A10.2	0.0233067711090351	AGIWUR11147;AGIWUR11431;AGIWUR21101;AGIWUR27403;AGIWUR28716;AGIWUR31664
CB4856	CT	I	5563827	5566265	WBGene00019692	M01A10.1	M01A10.1	-0.139506025733864	AGIWUR14718
CB4856	CT	I	5566343	5570081	WBGene00019713	pqn-52	M01E11.4	0.0374205205394178	AGIWUR6355;AGIWUR8778;AGIWUR14340;AGIWUR18170;AGIWUR19043;AGIWUR39174

CB4856	CT	I	5571787	5573019	WBGene00000474	cey-3	M01E11.5	-0.146457876066987	AGIWUR15351;AGIWUR34621
CB4856	CT	I	5573214	5576749	WBGene00019712	rmh-1	M01E11.3	-0.23662260466492	AGIWUR30964
CB4856	CT	I	5579666	5581979	WBGene00019711	M01E11.2	M01E11.2	-0.156336655656878	AGIWUR34357
CB4856	CT	I	5582005	5583471	WBGene00019710	M01E11.1	M01E11.1	-0.10348244254925	AGIWUR18685
CB4856	CT	I	5583407	5607529	WBGene00006508	tns-1	M01E11.7	-0.0645006990775689	AGIWUR6532;AGIWUR6811;AGIWUR11533;AGIWUR11928;AGIWUR15727;AGIWUR18697;AGIWUR19135;AGIWUR21857;AGIWUR30199;AGIWUR31547;AGIWUR36030;AGIWUR38255
CB4856	CT	I	5607820	5608841	WBGene00006726	ubl-5	F46F11.4	-0.301976676596485	AGIWUR38965
CB4856	CT	I	5609310	5610270	WBGene00006919	vha-10	F46F11.5	-0.244832350341864	AGIWUR5301;AGIWUR16359
CB4856	CT	I	5610375	5616402	WBGene00018512	trpp-8	F46F11.9	-0.175249203688386	AGIWUR28006
CB4856	CT	I	5616593	5618682	WBGene00018509	F46F11.6	F46F11.6	-0.13429366880843	AGIWUR31483
CB4856	CT	I	5619569	5620668	WBGene00000473	cey-2	F46F11.2	-0.0809807460865937	AGIWUR19929;AGIWUR20244;AGIWUR20969;AGIWUR39577;AGIWUR39981;AGIWUR43534
CB4856	CT	I	5620751	5623035	WBGene00018510	F46F11.7	F46F11.7	0.0844560064286686	AGIWUR6851
CB4856	CT	I	5623019	5624475	WBGene00018513	F46F11.10	F46F11.10	-0.335367843680059	AGIWUR14352;AGIWUR29230
CB4856	CT	I	5624558	5626288	WBGene00018511	F46F11.8	F46F11.8	0.244294678028403	AGIWUR13272
CB4856	CT	I	5627819	5637622	WBGene00018508	F46F11.1	F46F11.1	0.0415426988066816	AGIWUR40503
CB4856	CT	I	5643310	5644057	WBGene00044616	M01A12.4	M01A12.4	-0.106637902135096	AGIWUR32316
CB4856	CT	I	5644729	5646370	WBGene00000630	col-53	M01A12.1	0.0610064500819636	AGIWUR31336;AGIWUR38774
CB4856	CT	I	5646369	5649656	WBGene00018890	F55F8.2	F55F8.2	-0.175815271243206	AGIWUR37570
CB4856	CT	I	5649756	5653648	WBGene00018891	F55F8.3	F55F8.3	-0.0216003354745027	AGIWUR43787
CB4856	CT	I	5653804	5655857	WBGene00018892	cir-1	F55F8.4	0.0751492075901981	AGIWUR18388;AGIWUR44318
CB4856	CT	I	5655950	5658118	WBGene00018893	wdr-12	F55F8.5	-0.10729655874432	AGIWUR44268
CB4856	CT	I	5657980	5658723	WBGene00018894	mff-2	F55F8.6	-0.156754397691046	AGIWUR22067

CB4856	CT	I	5662143	5667160	WBGene00004224	ptr-10	F55F8.1	0.815009340715018	AGIWUR34950
CB4856	CT	I	5667069	5668837	WBGene00018896	F55F8.8	F55F8.8	-0.343119894816471	AGIWUR44721
CB4856	CT	I	5669950	5673352	WBGene00018897	zipt-2.4	F55F8.9	-0.195558855139758	AGIWUR33516
CB4856	CT	I	5674304	5676609	WBGene00020558	T19B4.5	T19B4.5	-0.123663992107156	AGIWUR11276;AGIWUR12993;AGIWUR32131
CB4856	CT	I	5676827	5677646	WBGene00001039	dnj-21	T19B4.4	-0.282118524201985	AGIWUR16865
CB4856	CT	I	5678638	5679862	WBGene00020557	T19B4.3	T19B4.3	-0.165149720156937	AGIWUR27841;AGIWUR39756;AGIWUR40511;AGIWUR44979
CB4856	CT	I	5679844	5691503	WBGene00006776	unc-40	T19B4.7	0.278604179327033	AGIWUR10286
CB4856	CT	I	5691838	5697506	WBGene00003793	npp-7	T19B4.2	-0.0191409250740842	AGIWUR623;AGIWUR6784;AGIWUR10030;AGIWUR11279;AGIWUR12845;AGIWUR28597;AGIWUR30194;AGIWUR36089;AGIWUR37196
CB4856	CT	I	5702156	5706838	WBGene00020556	pamn-1	T19B4.1	-0.0406632917537042	AGIWUR1427;AGIWUR1919;AGIWUR4740;AGIWUR7671;AGIWUR18929;AGIWUR19185;AGIWUR19546;AGIWUR19808;AGIWUR21670;AGIWUR22000;AGIWUR33507;AGIWUR34825;AGIWUR36338
CB4856	CT	I	5716574	5725772	WBGene00002989	lim-7	C04F1.3	-0.178401478285707	AGIWUR315
CB4856	CT	I	5726308	5729310	WBGene00015442	C04F1.1	C04F1.1	0.0416830463489379	AGIWUR21075;AGIWUR37345
CB4856	CT	I	5729793	5735842	WBGene00018994	F56H1.3	F56H1.3	0.030983375758753	AGIWUR3566
CB4856	CT	I	5741186	5741622	WBGene00269393	F56H1.11	F56H1.11	0.035189060994341	AGIWUR11632
CB4856	CT	I	5741868	5743571	WBGene00004505	rpt-5	F56H1.4	0.0394598340735808	AGIWUR4545;AGIWUR10484;AGIWUR34653;AGIWUR44921
CB4856	CT	I	5744148	5745617	WBGene00044305	rad-8	F56H1.6	-0.291133172173573	AGIWUR15763;AGIWUR22891
CB4856	CT	I	5746633	5754278	WBGene00000493	che-14	F56H1.1	0.0524905550061225	AGIWUR27291
CB4856	CT	I	5754312	5760803	WBGene00018995	ccpp-1	F56H1.5	-0.108476396675179	AGIWUR12374;AGIWUR14910;AGIWUR28454
CB4856	CT	I	5760897	5762435	WBGene00045483	oxy-5	F56H1.7	0.0301846475635568	AGIWUR6195;AGIWUR37147
CB4856	CT	I	5764030	5768190	WBGene00020694	dhhc-11	T22E7.2	0.626236870361312	AGIWUR20471
CB4856	CT	I	5768919	5773627	WBGene00020693	lron-8	T22E7.1	-0.097086796412349	AGIWUR7310;AGIWUR37476
CB4856	CT	I	5774585	5775610	WBGene00001856	hil-5	B0414.3	0.203997385704712	AGIWUR31929;AGIWUR38263
CB4856	CT	I	5776154	5788610	WBGene00004393	rnt-1	B0414.2	0.429802852486791	AGIWUR35389
CB4856	CT	I	5788634	5791752	WBGene00000772	cpb-3	B0414.5	0.385997396422651	AGIWUR11416
CB4856	CT	I	5792324	5795114	WBGene00001600	glh-3	B0414.6	-0.0915767082661585	AGIWUR26903
CB4856	CT	I	5795404	5801299	WBGene00003472	mtk-1	B0414.7	0.0311047111415474	

AGIWUR5791;AGIWUR9780;AGIWUR32266
CB4856 CT I 5801872 5803266 WBGene00015175 srz-4 B0414.1 0.0201407268275613 AGIWUR4046
CB4856 CT I 5803959 5807291 WBGene00015176 vps-51 B0414.8 -0.353416938287252 AGIWUR1676
CB4856 CT I 5807709 5815637 WBGene00003020 lin-35 C32F10.2 -0.0551505689470382
AGIWUR39684
CB4856 CT I 5815609 5818132 WBGene00001973 hmg-3 C32F10.5 -0.160783112552895
AGIWUR20478
CB4856 CT I 5818477 5821134 WBGene00003601 nhr-2 C32F10.6 -0.0676979852497563
AGIWUR39715
CB4856 CT I 5822841 5826148 WBGene00016333 C32F10.8 C32F10.8 -0.133556671040498
AGIWUR11408;AGIWUR13429;AGIWUR22672;AGIWUR24518;AGIWUR28667
CB4856 CT I 5829072 5834640 WBGene00016331 obr-4 C32F10.1 -0.0289949618357692
AGIWUR16969;AGIWUR31133
CB4856 CT I 5834644 5837392 WBGene00018002 twk-47 F33D11.5 0.31747082486127
AGIWUR6580
CB4856 CT I 5840289 5841888 WBGene00000436 ceh-12 F33D11.4 -0.0296629575491973
AGIWUR24005
CB4856 CT I 5842709 5844050 WBGene00000631 col-54 F33D11.3 -0.41833126104343
AGIWUR26608
CB4856 CT I 5844836 5846466 WBGene00018003 F33D11.6 F33D11.6 0.032262009455545
AGIWUR20606
CB4856 CT I 5847477 5849391 WBGene00018001 F33D11.2 F33D11.2 -0.334005912398295
AGIWUR26643
CB4856 CT I 5852045 5852962 WBGene00018000 F33D11.1 F33D11.1 -0.21546504209157
AGIWUR2935;AGIWUR8296;AGIWUR17179;AGIWUR33917;AGIWUR37035;AGIWUR38448
CB4856 CT I 5854261 5855047 WBGene00018005 F33D11.8 F33D11.8 -0.0387829414095129
AGIWUR1231;AGIWUR12588
CB4856 CT I 5857548 5862557 WBGene00018006 hpo-3 F33D11.9 -0.175670238206177
AGIWUR20778;AGIWUR26526;AGIWUR29283
CB4856 CT I 5862644 5866431 WBGene00018009 dhhc-3 F33D11.12 -0.0830386843607709
AGIWUR35887
CB4856 CT I 5866514 5868139 WBGene00018007 F33D11.10 F33D11.10 0.0726148771547965
AGIWUR11065;AGIWUR33616
CB4856 CT I 5868266 5869954 WBGene00018008 vpr-1 F33D11.11 -0.0716569774379821
AGIWUR24026
CB4856 CT I 5872174 5874343 WBGene00016421 cdc-7 C34G6.5 -0.207103589083061 AGIWUR4240
CB4856 CT I 5874422 5878954 WBGene00016422 noah-1 C34G6.6 1.14546648364535 AGIWUR31175
CB4856 CT I 5886613 5895970 WBGene00003996 pgp-2 C34G6.4 -0.34190053916776 AGIWUR25666
CB4856 CT I 5898303 5902112 WBGene00016419 tyr-4 C34G6.2 -0.0230913497350187 AGIWUR39659

CB4856	CT	I	5902106	5904695	WBGene00004109	stam-1	C34G6.7	0.000120332473565909	AGIWUR1601;AGIWUR1672;AGIWUR38163;AGIWUR39752
CB4856	CT	I	5906662	5918229	WBGene00016418	hpo-27	C34G6.1	-0.0717165502927894	AGIWUR1717;AGIWUR13154;AGIWUR14544
CB4856	CT	I	5919055	5935416	WBGene00015028	B0207.5	B0207.5	-0.359438428447493	AGIWUR5023
CB4856	CT	I	5935370	5936957	WBGene00015029	B0207.6	B0207.6	-0.320599040240367	AGIWUR21951
CB4856	CT	I	5937477	5938885	WBGene00000099	air-2	B0207.4	-0.0775841339488696	AGIWUR8154;AGIWUR34964
CB4856	CT	I	5942419	5945537	WBGene00001676	gpa-14	B0207.3	0.175361367326486	AGIWUR7895
CB4856	CT	I	5945581	5947620	WBGene00015030	B0207.7	B0207.7	-0.363548350113081	AGIWUR22759
CB4856	CT	I	5951136	5951742	WBGene00015031	B0207.8	B0207.8	0.028229891365898	AGIWUR38503
CB4856	CT	I	5955031	5956883	WBGene00015027	B0207.2	B0207.2	-0.447640535220447	AGIWUR25443;AGIWUR37060;AGIWUR41309
CB4856	CT	I	5957461	5958457	WBGene00015032	B0207.9	B0207.9	-0.229842772950176	AGIWUR15640
CB4856	CT	I	5959008	5959526	WBGene00015033	B0207.10	B0207.10	0.254168154987003	AGIWUR5455
CB4856	CT	I	5962953	5964183	WBGene00015034	B0207.11	B0207.11	0.112610017921472	AGIWUR25597
CB4856	CT	I	5965062	5967211	WBGene00015026	B0207.1	B0207.1	-0.0348771262490661	AGIWUR35700
CB4856	CT	I	5967250	5973320	WBGene00000232	avr-14	B0207.12	-0.0904601584141682	AGIWUR636;AGIWUR9364;AGIWUR18879;AGIWUR37041;AGIWUR37132
CB4856	CT	I	5981778	5985869	WBGene00015504	C06A5.6	C06A5.6	-0.135577407152002	AGIWUR39294
CB4856	CT	I	5986443	5990581	WBGene00015501	C06A5.3	C06A5.3	0.0469027485746594	AGIWUR35558
CB4856	CT	I	5990741	5992658	WBGene00004803	sir-2.4	C06A5.11	0.165448674964029	AGIWUR43540
CB4856	CT	I	5992661	6003363	WBGene00006823	unc-94	C06A5.7	0.0259116147593481	AGIWUR4768;AGIWUR18849;AGIWUR18987;AGIWUR20008;AGIWUR20709;AGIWUR27311;AGIWUR32220;AGIWUR33730;AGIWUR35148;AGIWUR36712;AGIWUR40863
CB4856	CT	I	6004599	6007595	WBGene00044414	C06A5.12	C06A5.12	-0.15042957668218	AGIWUR28163;AGIWUR31238;AGIWUR34035;AGIWUR42647;AGIWUR42784
CB4856	CT	I	6008105	6009606	WBGene00015505	C06A5.8	C06A5.8	0.106030614312036	AGIWUR34087;AGIWUR37759
CB4856	CT	I	6009782	6011586	WBGene00004380	rnf-1	C06A5.9	-0.31104604688729	AGIWUR4178;AGIWUR27456
CB4856	CT	I	6011977	6022516	WBGene00015499	inst-1	C06A5.1	-0.36582437448453	AGIWUR33571
CB4856	CT	I	6022660	6026874	WBGene00000814	csn-2	B0025.2	-0.111082360008723	AGIWUR5887
CB4856	CT	I	6027194	6028370	WBGene00015001	B0025.4	B0025.4	0.661740433634407	AGIWUR11731
CB4856	CT	I	6028486	6033457	WBGene00006932	vps-34	B0025.1	-0.0988405408022652	AGIWUR18092;AGIWUR25361;AGIWUR41664;AGIWUR42560

CB4856	CT	I	6035452	6037745	WBGene00016155	C27A12.4	C27A12.4	-0.0162513578987857	AGIWUR43310
CB4856	CT	I	6038406	6041265	WBGene00006492	let-391	C27A12.3	-0.290430984935995	AGIWUR4733
CB4856	CT	I	6041672	6046018	WBGene00016154	C27A12.2	C27A12.2	-0.274154567878838	AGIWUR17644
CB4856	CT	I	6046832	6050462	WBGene00000429	ceh-2	C27A12.5	0.527762610553415	AGIWUR28248
CB4856	CT	I	6053785	6055821	WBGene00016156	C27A12.6	C27A12.6	-0.0364546036819116	AGIWUR2214;AGIWUR7904;AGIWUR27980;AGIWUR37666
CB4856	CT	I	6055947	6058424	WBGene00016157	C27A12.7	C27A12.7	0.0780725840645089	AGIWUR7052;AGIWUR18453;AGIWUR30076;AGIWUR32444
CB4856	CT	I	6058546	6061033	WBGene00016158	ari-1	C27A12.8	0.0753392163509812	AGIWUR43402
CB4856	CT	I	6061752	6068040	WBGene00016159	C27A12.9	C27A12.9	-0.0899256316359778	AGIWUR2352;AGIWUR10114;AGIWUR11058;AGIWUR38416;AGIWUR44563;AGIWUR45035
CB4856	CT	I	6068526	6070122	WBGene00022749	ZK484.3	ZK484.3	0.122950830780979	AGIWUR23472;AGIWUR25484;AGIWUR42142
CB4856	CT	I	6070236	6078895	WBGene00022750	ZK484.4	ZK484.4	-0.16437547778658	AGIWUR32976;AGIWUR33451
CB4856	CT	I	6081782	6085962	WBGene00001819	haf-9	ZK484.2	-0.442757887157796	AGIWUR2028;AGIWUR12808;AGIWUR25179;AGIWUR39792;AGIWUR43940
CB4856	CT	I	6087366	6087764	WBGene00022751	ZK484.5	ZK484.5	0.398585593109217	AGIWUR40045
CB4856	CT	I	6090015	6090919	WBGene00022752	ZK484.6	ZK484.6	-0.107234523676631	AGIWUR15679
CB4856	CT	I	6093305	6094426	WBGene00022748	oaz-1	ZK484.1	0.522504135351656	AGIWUR5633;AGIWUR6260;AGIWUR9669;AGIWUR10174;AGIWUR10329;AGIWUR11262;AGIWUR14480;AGIWUR16682;AGIWUR17416;AGIWUR18340;AGIWUR18545;AGIWUR22968;AGIWUR23831;AGIWUR23930;AGIWUR25183;AGIWUR25365;AGIWUR26539;AGIWUR26756;AGIWUR30941;AGIWUR34252;AGIWUR34836;AGIWUR40956;AGIWUR41471;AGIWUR45198
CB4856	CT	I	6095060	6096589	WBGene00022753	ZK484.7	ZK484.7	0.0184534949323059	AGIWUR24816
CB4856	CT	I	6097494	6097837	WBGene00022754	nspd-1	ZK484.8	0.199115539533061	AGIWUR17934
CB4856	CT	I	6098927	6099731	WBGene00020840	spch-3	T27A3.4	0.388145025426436	AGIWUR32101
CB4856	CT	I	6100261	6101789	WBGene00020841	T27A3.5	T27A3.5	0.0692658559231738	AGIWUR19455
CB4856	CT	I	6102032	6102463	WBGene00006044	ssp-16	T27A3.3	-0.165083219286294	AGIWUR14575;AGIWUR23833;AGIWUR26401;AGIWUR29267;AGIWUR33358;AGIWUR42127
CB4856	CT	I	6104805	6105589	WBGene00044611	T27A3.8	T27A3.8	1.13330836433455	AGIWUR22882
CB4856	CT	I	6105863	6107715	WBGene00020842	T27A3.6	T27A3.6	-0.289074723816148	AGIWUR18567;AGIWUR24268;AGIWUR41070
CB4856	CT	I	6107809	6109679	WBGene00020843	T27A3.7	T27A3.7	0.0573728381600844	AGIWUR37549
CB4856	CT	I	6110134	6114537	WBGene00020839	usp-5	T27A3.2	0.0849169376603491	AGIWUR22004

CB4856 CT I 6122590 6126851 WBGene00020838 trak-1 T27A3.1 -0.113087007490307
AGIWUR9827;AGIWUR13114;AGIWUR18998;AGIWUR19263;AGIWUR20155;AGIWUR21043;AGIWUR21050;AGIWUR21240;AGIWUR25994;AGIWUR26331;AGIWUR34093
CB4856 CT I 6133245 6137978 WBGene00003878 pept-3 F56F4.5 0.0684534747474004 AGIWUR25162
CB4856 CT I 6137994 6138955 WBGene00018981 F56F4.4 F56F4.4 -0.143354321876576 AGIWUR7191
CB4856 CT I 6139216 6140444 WBGene00018982 abt-6 F56F4.6 0.0595262806936461
AGIWUR6130;AGIWUR7439;AGIWUR21554
CB4856 CT I 6141469 6144386 WBGene00018980 F56F4.3 F56F4.3 -0.0766899572730906
AGIWUR1365;AGIWUR31670
CB4856 CT I 6145455 6146477 WBGene00018979 ttr-55 F56F4.2 -0.0135353604676345
AGIWUR9831;AGIWUR12974;AGIWUR17577;AGIWUR23966;AGIWUR27059
CB4856 CT I 6146461 6147068 WBGene00050896 ttr-56 F56F4.9 0.266151758245447 AGIWUR33891
CB4856 CT I 6149197 6150480 WBGene00045498 F56F4.8 F56F4.8 -0.178854138758062
AGIWUR18973;AGIWUR22403;AGIWUR42734
CB4856 CT I 6154075 6157438 WBGene00020379 T09B4.5 T09B4.5 0.855505900984634
AGIWUR8668;AGIWUR31230
CB4856 CT I 6157473 6159264 WBGene00020380 T09B4.6 T09B4.6 0.173285523666517 AGIWUR26255
CB4856 CT I 6164719 6166539 WBGene00020382 T09B4.8 T09B4.8 -0.515125859557649 AGIWUR22457
CB4856 CT I 6167940 6168642 WBGene00020378 T09B4.4 T09B4.4 -0.0367057243381552 AGIWUR8464
CB4856 CT I 6176252 6178057 WBGene00020377 T09B4.3 T09B4.3 -0.35968302780123 AGIWUR20677
CB4856 CT I 6177983 6180840 WBGene00020383 tin-44 T09B4.9 -0.313348157850101 AGIWUR33554
CB4856 CT I 6181314 6183913 WBGene00000500 chn-1 T09B4.10 -0.198563639128466
AGIWUR3531;AGIWUR14093;AGIWUR30546
CB4856 CT I 6184072 6185368 WBGene00020376 T09B4.2 T09B4.2 0.0574143897248245 AGIWUR5828
CB4856 CT I 6185462 6188121 WBGene00020375 pigv-1 T09B4.1 -0.217575868647041 AGIWUR2739
CB4856 CT I 6188157 6192632 WBGene00020259 T05E7.3 T05E7.3 -0.0565940638290567
AGIWUR8483;AGIWUR13425;AGIWUR16427;AGIWUR36048
CB4856 CT I 6192552 6194897 WBGene00020260 T05E7.4 T05E7.4 0.968794507767658 AGIWUR3464
CB4856 CT I 6199141 6200193 WBGene00000632 col-55 T05E7.2 -0.0146638717076319 AGIWUR45093
CB4856 CT I 6200798 6202076 WBGene00020261 vet-1 T05E7.5 -0.0118932939135306 AGIWUR29881
CB4856 CT I 6203897 6206927 WBGene00020258 T05E7.1 T05E7.1 0.710880380712204 AGIWUR11566
CB4856 CT I 6206955 6211155 WBGene00020347 ech-1.2 T08B2.7 -0.186714633941594
AGIWUR1878;AGIWUR13286;AGIWUR24847;AGIWUR27700
CB4856 CT I 6211270 6211885 WBGene00020348 mrpl-23 T08B2.8 -0.240015229266533 AGIWUR12288
CB4856 CT I 6211986 6214645 WBGene00001497 fars-1 T08B2.9 -0.265431954329182
AGIWUR15691;AGIWUR18498;AGIWUR21754;AGIWUR24091;AGIWUR31467;AGIWUR34640;AGIWUR39566
CB4856 CT I 6215669 6219911 WBGene00020346 rbm-5 T08B2.5 -0.0852239086376327
AGIWUR389;AGIWUR3390;AGIWUR17098;AGIWUR17725;AGIWUR21019;AGIWUR24671;AGIWUR42870;AGIWUR45169
CB4856 CT I 6220090 6220766 WBGene00004486 rps-17 T08B2.10 -0.000370386724622307

AGIWUR6912;AGIWUR15081;AGIWUR16480;AGIWUR16946;AGIWUR21104;AGIWUR24229;AGIWUR24253;AGIWUR26223;AGIWUR28324;AGIWUR28853;AGIWUR32494;AGIWUR38938;AGIWUR40673;AGIWUR43660
CB4856 CT I 6221245 6223116 WBGene00020349 T08B2.11 T08B2.11 0.429091618909119 AGIWUR41230
CB4856 CT I 6232777 6234280 WBGene00000633 col-56 T08B2.2 0.25479579684899 AGIWUR18477;AGIWUR21810;AGIWUR23961
CB4856 CT I 6249556 6250197 WBGene00005000 spp-15 C48E7.10 0.0392992859217092 AGIWUR26334
CB4856 CT I 6250475 6252834 WBGene00000583 cof-2 C48E7.5 0.113816866739093 AGIWUR25222;AGIWUR43373
CB4856 CT I 6252986 6253584 WBGene00016754 cebp-2 C48E7.11 -0.12402159732779 AGIWUR4905
CB4856 CT I 6253701 6254947 WBGene00003059 lpd-2 C48E7.3 -0.182902094982786 AGIWUR36039
CB4856 CT I 6255035 6257791 WBGene00016750 let-611 C48E7.2 -0.14839017085319 AGIWUR8583
CB4856 CT I 6260249 6264540 WBGene00016749 C48E7.1 C48E7.1 0.18183573647854 AGIWUR25489
CB4856 CT I 6265691 6269073 WBGene00016753 oac-9 C48E7.8 -0.15271342310338 AGIWUR7858
CB4856 CT I 6269561 6271541 WBGene00006689 twk-37 C48E7.9 -0.127434726302562 AGIWUR32998
CB4856 CT I 6271866 6273780 WBGene00015688 C10G11.8 C10G11.8 0.0400829486206575 AGIWUR25031;AGIWUR33880;AGIWUR36459
CB4856 CT I 6284973 6289340 WBGene00015687 chdp-1 C10G11.7 -0.0548622403111931 AGIWUR4250
CB4856 CT I 6289433 6293855 WBGene00015686 C10G11.6 C10G11.6 -0.169659662589446 AGIWUR8034
CB4856 CT I 6295067 6297625 WBGene00004068 pnk-1 C10G11.5 0.529396501940225 AGIWUR13687;AGIWUR29299;AGIWUR36480;AGIWUR43272
CB4856 CT I 6297767 6299729 WBGene00005272 srh-49 C10G11.4 0.101107298431278 AGIWUR16346
CB4856 CT I 6300493 6302298 WBGene00005274 srh-51 C10G11.3 -0.171414005755886 AGIWUR12171;AGIWUR16140
CB4856 CT I 6302474 6304263 WBGene00005273 srh-50 C10G11.2 0.53622773735138 AGIWUR41687
CB4856 CT I 6304916 6306363 WBGene00015690 C10G11.10 C10G11.10 0.115930723172914 AGIWUR33367
CB4856 CT I 6309114 6310781 WBGene00017817 F26B1.5 F26B1.5 -0.108134810632919 AGIWUR7184;AGIWUR31624
CB4856 CT I 6311229 6312413 WBGene00000634 col-58 F26B1.4 -0.189059506961978 AGIWUR2202
CB4856 CT I 6312750 6314624 WBGene00005171 srg-14 F26B1.6 -0.539119624752743 AGIWUR3070
CB4856 CT I 6315233 6317267 WBGene00002073 ima-2 F26B1.3 0.0499627510023828 AGIWUR10902;AGIWUR14196;AGIWUR25439;AGIWUR31579;AGIWUR37813

CB4856 CT I 6317701 6320365 WBGene00017816 hrpk-1 F26B1.2 -0.0748204268208403 AGIWUR1418;AGIWUR1827;AGIWUR3187;AGIWUR5329;AGIWUR9787;AGIWUR16221;AGIWUR17680;AGIWUR18701;AGIWUR18921;AGIWUR26852;AGIWUR27039;AGIWUR36351;AGIWUR39126;AGIWUR43994
CB4856 CT I 6320289 6324018 WBGene00002601 let-381 F26B1.7 0.452531441100753 AGIWUR36974
CB4856 CT I 6342231 6356678 WBGene00000254 bli-4 K04F10.4 0.000248456192787572 AGIWUR3978;AGIWUR12697;AGIWUR17473;AGIWUR25337;AGIWUR32461;AGIWUR34393;AGIWUR42192;AGIWUR44317
CB4856 CT I 6356614 6358410 WBGene00003499 mut-2 K04F10.6 -0.071783453249413 AGIWUR2874;AGIWUR9366;AGIWUR9500;AGIWUR26345;AGIWUR33780;AGIWUR36604;AGIWUR41011;AGIWUR42263
CB4856 CT I 6358585 6360457 WBGene00019396 K04F10.3 K04F10.3 0.139075926054275 AGIWUR13704
CB4856 CT I 6360844 6364172 WBGene00019395 K04F10.2 K04F10.2 0.136564562799655 AGIWUR4876
CB4856 CT I 6364886 6366224 WBGene00019394 K04F10.1 K04F10.1 0.111394386723381 AGIWUR3231
CB4856 CT I 6366331 6368078 WBGene00019399 K04F10.7 K04F10.7 -0.276936373286699 AGIWUR6668
CB4856 CT I 6368270 6369598 WBGene00022586 ZC308.4 ZC308.4 -0.127256548515678 AGIWUR37754
CB4856 CT I 6369934 6386183 WBGene00001596 gld-2 ZC308.1 -0.0623657034419995 AGIWUR1922;AGIWUR5338;AGIWUR9092;AGIWUR24999
CB4856 CT I 6386149 6392524 WBGene00022592 klu-2 ZC328.2 0.00165295865110693 AGIWUR9565
CB4856 CT I 6404587 6405705 WBGene00022591 cuti-1 ZC328.1 -0.0264682204460591 AGIWUR28749;AGIWUR40109;AGIWUR42212
CB4856 CT I 6405882 6406936 WBGene00022594 ZC328.5 ZC328.5 0.0732178302584092 AGIWUR29601
CB4856 CT I 6407458 6409212 WBGene00004721 san-1 ZC328.4 -0.122956691288113 AGIWUR10499;AGIWUR16704;AGIWUR24958;AGIWUR38192;AGIWUR40688
CB4856 CT I 6409050 6417010 WBGene00022593 ZC328.3 ZC328.3 0.0354883576365657 AGIWUR2496;AGIWUR16461;AGIWUR19232
CB4856 CT I 6418565 6418809 WBGene00019992 R10A10.1 R10A10.1 0.134867038617328 AGIWUR38650
CB4856 CT I 6420660 6421985 WBGene00019993 rbx-2 R10A10.2 -0.169318599077901 AGIWUR20356
CB4856 CT I 6421978 6425794 WBGene00018156 ncbp-1 F37E3.1 -0.2555658100495 AGIWUR26304
CB4856 CT I 6425793 6428942 WBGene00018157 lron-6 F37E3.2 -0.0778275384052138 AGIWUR1813
CB4856 CT I 6429525 6431586 WBGene00018158 comp-1 F37E3.3 -0.139212771197639 AGIWUR1030;AGIWUR34352
CB4856 CT I 6436058 6441192 WBGene00004924 snt-4 T23H2.2 0.364569042967641 AGIWUR23814
CB4856 CT I 6441637 6446465 WBGene00020742 T23H2.3 T23H2.3 -0.0150549494857088 AGIWUR2836;AGIWUR2981;AGIWUR5671;AGIWUR11513;AGIWUR12657;AGIWUR14273;AGIWUR15264;AGIWUR15865;AGIWUR20917;AGIWUR26561;AGIWUR34382;AGIWUR43457

CB4856 CT I 6447304 6453933 WBGene00003798 npp-12 T23H2.1 0.0121624490321821
AGIWUR565;AGIWUR8913;AGIWUR37341;AGIWUR41730
CB4856 CT I 6453875 6456984 WBGene00020743 mam-8 T23H2.4 0.113927213878544 AGIWUR24944
CB4856 CT I 6457061 6458479 WBGene00004273 rab-10 T23H2.5 -0.116440715238518
AGIWUR1929;AGIWUR2575;AGIWUR4282;AGIWUR8097;AGIWUR10011;AGIWUR21666;AGIWUR34705
CB4856 CT I 6460433 6464286 WBGene00019432 knl-2 K06A5.4 0.0864340537086008 AGIWUR32152
CB4856 CT I 6464197 6466941 WBGene00019433 acdh-3 K06A5.6 -0.227081948142075 AGIWUR7557
CB4856 CT I 6466752 6467515 WBGene00019431 K06A5.3 K06A5.3 0.0529194127833499 AGIWUR8782
CB4856 CT I 6468113 6469392 WBGene00019430 K06A5.2 K06A5.2 0.216753591956984 AGIWUR24586
CB4856 CT I 6469386 6471995 WBGene00000386 cdc-25.1 K06A5.7 0.216919581061049
AGIWUR40783
CB4856 CT I 6474183 6489030 WBGene00019434 nmtn-1 K06A5.8 0.300841025707173
AGIWUR3470;AGIWUR27663;AGIWUR38621
CB4856 CT I 6490716 6492727 WBGene00000937 dbr-1 C55B7.8 -0.0923298309147498 AGIWUR15559
CB4856 CT I 6492722 6494754 WBGene00007018 mdt-18 C55B7.9 -0.178922405058168 AGIWUR24134
CB4856 CT I 6494216 6496109 WBGene00016944 uri-1 C55B7.5 -0.0753551231674763 AGIWUR23828
CB4856 CT I 6497436 6499560 WBGene00016943 acdh-1 C55B7.4 -0.451217118144658
AGIWUR9071;AGIWUR12896;AGIWUR13562;AGIWUR24409;AGIWUR29961
CB4856 CT I 6507894 6511630 WBGene00001627 gly-2 C55B7.2 0.169602986313839 AGIWUR38944
CB4856 CT I 6511612 6513843 WBGene00016947 C55B7.11 C55B7.11 -0.0566369325608012
AGIWUR317;AGIWUR10946;AGIWUR17661;AGIWUR19575;AGIWUR40605
CB4856 CT I 6513928 6517507 WBGene00001599 glh-2 C55B7.1 -0.287604169209679 AGIWUR14020
CB4856 CT I 6517575 6520879 WBGene00000483 che-1 C55B7.12 0.673557970074288
AGIWUR40311
CB4856 CT I 6525045 6528689 WBGene00020414 T10E9.4 T10E9.4 0.95790437082568 AGIWUR11816
CB4856 CT I 6529158 6530099 WBGene00020416 T10E9.6 T10E9.6 0.0037766352651909 AGIWUR1670
CB4856 CT I 6530131 6533031 WBGene00020417 nuo-2 T10E9.7 -0.140632450609338
AGIWUR3800;AGIWUR20696;AGIWUR23397
CB4856 CT I 6534072 6539193 WBGene00020413 T10E9.3 T10E9.3 -0.420963820177146 AGIWUR7632
CB4856 CT I 6539129 6541084 WBGene00020418 T10E9.8 T10E9.8 -0.110448731450962
AGIWUR8524;AGIWUR10486;AGIWUR10560;AGIWUR16490;AGIWUR32642;AGIWUR35271
CB4856 CT I 6542090 6544966 WBGene00020412 T10E9.2 T10E9.2 -0.214769609347611 AGIWUR39070
CB4856 CT I 6545110 6546593 WBGene00020411 T10E9.1 T10E9.1 -0.227539901187315 AGIWUR29543
CB4856 CT I 6548644 6550447 WBGene00019005 F57B10.8 F57B10.8 -0.103161454383358
AGIWUR34149
CB4856 CT I 6550536 6552665 WBGene00019006 spg-20 F57B10.9 0.0771338520881031
AGIWUR2257
CB4856 CT I 6552834 6553379 WBGene00019007 ucr-11 F57B10.14 -0.168818349396165
AGIWUR26529

CB4856	CT	I	6553481	6554290	WBGene00000896	dad-1	F57B10.10	-0.165510001547688	AGIWUR15439
CB4856	CT	I	6554729	6558623	WBGene00006607	tre-1	F57B10.7	0.061564409670428	AGIWUR30347
CB4856	CT	I	6558696	6562346	WBGene00019004	xpg-1	F57B10.6	0.27330909155781	AGIWUR13434
CB4856	CT	I	6562699	6563660	WBGene00019003	F57B10.5	F57B10.5	-0.0596399550262703	AGIWUR37962
CB4856	CT	I	6563629	6564451	WBGene00000236	bag-1	F57B10.11	-0.222429746285339	AGIWUR18247
CB4856	CT	I	6564749	6565868	WBGene00003184	mei-2	F57B10.12	-0.22118408211753	AGIWUR37281
CB4856	CT	I	6566173	6568905	WBGene00019002	F57B10.4	F57B10.4	0.00238710455312585	AGIWUR37891
CB4856	CT	I	6569494	6572598	WBGene00019001	ipgm-1	F57B10.3	-0.110337859323995	AGIWUR7348;AGIWUR17880;AGIWUR19488;AGIWUR23845
CB4856	CT	I	6574004	6585510	WBGene00002783	let-607	F57B10.1	-0.0497416115332392	AGIWUR10303;AGIWUR13630;AGIWUR16293;AGIWUR22955;AGIWUR28124;AGIWUR31341
CB4856	CT	I	6585039	6587882	WBGene00006624	try-6	F48A9.3	-0.104716673861248	AGIWUR17630
CB4856	CT	I	6592332	6593831	WBGene00018582	F48A9.2	F48A9.2	-0.172317726682158	AGIWUR22685
CB4856	CT	I	6594320	6596231	WBGene00018581	F48A9.1	F48A9.1	-0.297755055105679	AGIWUR30733
CB4856	CT	I	6596919	6598928	WBGene00017067	D2092.6	D2092.6	-0.000264822079050653	AGIWUR13165
CB4856	CT	I	6598981	6607520	WBGene00017066	maco-1	D2092.5	-0.0128461044777186	AGIWUR6251;AGIWUR33086
CB4856	CT	I	6612188	6615990	WBGene00017065	D2092.4	D2092.4	-0.301958792781365	AGIWUR33325
CB4856	CT	I	6616304	6618560	WBGene00006645	tsp-19	D2092.7	-0.262936673415597	AGIWUR38000
CB4856	CT	I	6620665	6621292	WBGene00017068	D2092.8	D2092.8	-0.581827871505506	AGIWUR24576
CB4856	CT	I	6621687	6624894	WBGene00000050	acr-11	D2092.3	-0.257299391457856	AGIWUR4258
CB4856	CT	I	6625066	6626788	WBGene00017064	ppfr-2	D2092.2	-0.0986078156035024	AGIWUR39480
CB4856	CT	I	6627045	6627914	WBGene00044610	D2092.10	D2092.10	0.0396849596823357	AGIWUR21189;AGIWUR22339;AGIWUR38102;AGIWUR40477
CB4856	CT	I	6629717	6637429	WBGene00017063	mctp-1	D2092.1	0.045583273806642	AGIWUR30994
CB4856	CT	I	6640662	6642643	WBGene00006625	try-7	ZC581.6	-0.220100334539859	AGIWUR4027
CB4856	CT	I	6651220	6655430	WBGene00022633	ZC581.3	ZC581.3	0.398125591839355	AGIWUR20637
CB4856	CT	I	6655608	6658371	WBGene00022632	ZC581.2	ZC581.2	-0.104258149848034	AGIWUR13462;AGIWUR32856;AGIWUR33302
CB4856	CT	I	6660436	6662315	WBGene00022634	ZC581.7	ZC581.7	0.114854354470927	AGIWUR41254
CB4856	CT	I	6663050	6665160	WBGene00022631	nekl-2	ZC581.1	-0.274663305465793	AGIWUR4970
CB4856	CT	I	6665077	6673840	WBGene00022635	ZC581.9	ZC581.9	-0.0636464215822983	AGIWUR14388

CB4856	CT	I	6687349	6691662	WBGene00020712	ckr-1	T23B3.4	-0.143801813177248	AGIWUR27855
CB4856	CT	I	6706766	6708919	WBGene00020714	T23B3.6	T23B3.6	0.607374671689819	AGIWUR31861
CB4856	CT	I	6711512	6712327	WBGene00020711	rmd-5	T23B3.3	-0.187828006385777	AGIWUR43249
CB4856	CT	I	6715949	6717090	WBGene00020710	T23B3.2	T23B3.2	0.181022826095004	AGIWUR4376
CB4856	CT	I	6717819	6719430	WBGene00020709	T23B3.1	T23B3.1	-0.0386600930339824	AGIWUR41299
CB4856	CT	I	6719368	6723059	WBGene00020933	W02D3.4	W02D3.4	-0.184911119587625	AGIWUR22746
CB4856	CT	I	6724917	6728330	WBGene00003170	mec-6	W02D3.3	-0.730695929361662	AGIWUR969
CB4856	CT	I	6728290	6729376	WBGene00002258	lbp-6	W02D3.5	-0.424793096860791	AGIWUR30217
CB4856	CT	I	6729996	6730959	WBGene00007057	ant-1.2	W02D3.6	0.223834974002916	AGIWUR30633
CB4856	CT	I	6731267	6732280	WBGene00002257	lbp-5	W02D3.7	0.133257535304323	AGIWUR35562;AGIWUR35903
CB4856	CT	I	6734029	6736161	WBGene00004883	smg-5	W02D3.8	-0.121157521972911	AGIWUR40678
CB4856	CT	I	6736680	6741175	WBGene00020932	dhod-1	W02D3.2	-0.256246813613842	AGIWUR16146
CB4856	CT	I	6741274	6741877	WBGene00020931	cytb-5.2	W02D3.1	0.164107758306178	AGIWUR6119;AGIWUR17412
CB4856	CT	I	6741984	6742358	WBGene00020937	W02D3.12	W02D3.12	-0.244691657387477	AGIWUR27444
CB4856	CT	I	6742293	6745341	WBGene00006773	unc-37	W02D3.9	-0.0684623600339496	AGIWUR5383;AGIWUR17092
CB4856	CT	I	6745991	6753031	WBGene00020935	fnci-1	W02D3.10	-0.142697950216396	AGIWUR9400;AGIWUR35508
CB4856	CT	I	6753219	6756348	WBGene00020936	hrpf-1	W02D3.11	-0.0359931846926762	AGIWUR3301;AGIWUR4929;AGIWUR6674;AGIWUR15926;AGIWUR19943;AGIWUR35377
CB4856	CT	I	6757763	6758178	WBGene00001682	gpc-2	F08B6.2	-0.118873194519424	AGIWUR8016;AGIWUR25894
CB4856	CT	I	6758181	6760179	WBGene00017240	calu-2	F08B6.3	-0.249678376730726	AGIWUR31
CB4856	CT	I	6761228	6767797	WBGene00006819	unc-87	F08B6.4	0.017877070279158	AGIWUR5112;AGIWUR7202;AGIWUR19433;AGIWUR32709
CB4856	CT	I	6773976	6780151	WBGene00004111	pqn-21	C37A2.5	-7.44177573448468e-05	AGIWUR8188;AGIWUR13214;AGIWUR28740;AGIWUR31351;AGIWUR36292;AGIWUR38150;AGIWUR42687
CB4856	CT	I	6780806	6784396	WBGene00000871	cye-1	C37A2.4	-0.225951260321166	AGIWUR21118;AGIWUR35011
CB4856	CT	I	6784854	6787139	WBGene00016491	acdh-5	C37A2.3	0.0405723326142464	AGIWUR16440;AGIWUR35452;AGIWUR44135
CB4856	CT	I	6788032	6789002	WBGene00016492	C37A2.6	C37A2.6	0.15049868634428	AGIWUR6422;AGIWUR37330
CB4856	CT	I	6789039	6789738	WBGene00016493	C37A2.7	C37A2.7	0.0933398601186255	AGIWUR6374;AGIWUR44421
CB4856	CT	I	6791025	6797470	WBGene00004110	pqn-20	C37A2.2	-0.373841455844786	AGIWUR19579

CB4856	CT	I	6798232 6799674 WBGene00016494	C37A2.8 C37A2.8 -0.0245299389767339
AGIWUR28525;AGIWUR44472
CB4856	CT	I	6800386 6804860 WBGene00017098	E02D9.1 E02D9.1 0.103961698054514
AGIWUR17002;AGIWUR18835;AGIWUR27530;AGIWUR29337;AGIWUR32622;AGIWUR35400;AGIWUR42665
CB4856	CT	I	6804788 6810451 WBGene00019324	K02F2.5 K02F2.5 -0.101227213539247
AGIWUR7002;AGIWUR14204;AGIWUR16418;AGIWUR24188;AGIWUR38556;AGIWUR42759
CB4856	CT	I	6812664 6814248 WBGene00006740	ulp-5	K02F2.4 -0.250373439624944	AGIWUR25907
CB4856	CT	I	6814511 6834687 WBGene00019323	teg-4	K02F2.3 0.0529118509259163	AGIWUR33779
CB4856	CT	I	6836375 6838140 WBGene00019322	ahcy-1	K02F2.2 -0.342964765143126
AGIWUR287;AGIWUR1378;AGIWUR4956;AGIWUR8493;AGIWUR9338;AGIWUR9562;AGIWUR12159;AGIWUR14900;AGIWUR17808;AGIWUR
17829;AGIWUR19142;AGIWUR23216;AGIWUR23428;AGIWUR26001;AGIWUR28582;AGIWUR29154;AGIWUR32426
CB4856	CT	I	6838354 6843343 WBGene00001056	dpf-3	K02F2.1 -0.138772906732433	AGIWUR15334
CB4856	CT	I	6843589 6845134 WBGene00001075	dpy-14	H27M09.4	1.74156953270617
AGIWUR26750
CB4856	CT	I	6845175 6846952 WBGene00019248	H27M09.5	H27M09.5	0.191790847255897
AGIWUR12565
CB4856	CT	I	6847511 6849923 WBGene00019247	syp-4	H27M09.3	-0.116422769272756
AGIWUR16231
CB4856	CT	I	6850409 6851356 WBGene00019246	rpb-5	H27M09.2	-0.0637151346592795
AGIWUR41547
CB4856	CT	I	6851372 6854140 WBGene00019245	sacy-1	H27M09.1	-0.157717693010493
AGIWUR16956;AGIWUR24226;AGIWUR38168;AGIWUR39686;AGIWUR41837
CB4856	CT	I	6862397 6870424 WBGene00044898	let-383 T21G5.6 -0.150688032484352
AGIWUR3358;AGIWUR6039;AGIWUR10814;AGIWUR19853;AGIWUR35561
CB4856	CT	I	6870415 6871911 WBGene00020660	T21G5.2 T21G5.2 0.00179684885914533
AGIWUR21753;AGIWUR29068;AGIWUR35710
CB4856	CT	I	6872597 6873755 WBGene00020661	smz-2	T21G5.4 0.110391964684272	AGIWUR42609
CB4856	CT	I	6874304 6876928 WBGene00020659	T21G5.1 T21G5.1 -0.0706029887024844	AGIWUR26740
CB4856	CT	I	6877080 6888217 WBGene00006423	asd-2	T21G5.5 0.305013257278683
AGIWUR22524;AGIWUR24899;AGIWUR25729;AGIWUR34706;AGIWUR38832;AGIWUR43537;AGIWUR44294
CB4856	CT	I	6889254 6891501 WBGene00016742	C48B6.4 C48B6.4 0.251997962627899	AGIWUR13786
CB4856	CT	I	6897755 6900619 WBGene00016743	srb-15	C48B6.5 -0.222058676614871	AGIWUR12549
CB4856	CT	I	6900947 6913579 WBGene00004879	smg-1	C48B6.6 0.0474189206824516
AGIWUR26267;AGIWUR29121;AGIWUR35234
CB4856	CT	I	6915049 6916609 WBGene00016741	C48B6.3 C48B6.3 -0.150262050881175
AGIWUR3569;AGIWUR8775;AGIWUR16126;AGIWUR22913;AGIWUR32725;AGIWUR33646
CB4856	CT	I	6916766 6917560 WBGene00016740	C48B6.2 C48B6.2 -0.362424257533561	AGIWUR3988
CB4856	CT	I	6923076 6923688 WBGene00016746	C48B6.10	C48B6.10	-0.31343092548623
AGIWUR2589;AGIWUR8621;AGIWUR26363;AGIWUR28730;AGIWUR30871;AGIWUR44258

CB4856	CT	I	6924288	6925463	WBGene00016745	C48B6.9	C48B6.9	-0.0505775447440335	AGIWUR21629
CB4856	CT	I	6926262	6929664	WBGene00020399	ztf-4	T10B11.3	0.0211688889406538	AGIWUR20363
CB4856	CT	I	6931138	6936928	WBGene00020398	cerk-1	T10B11.2	0.255919722996456	AGIWUR8530
CB4856	CT	I	6937504	6939618	WBGene00020400	T10B11.4	T10B11.4	-0.149800674719864	AGIWUR31514
CB4856	CT	I	6940249	6941950	WBGene00020401	T10B11.5	T10B11.5	0.00983646955750039	AGIWUR43470
CB4856	CT	I	6943176	6945358	WBGene00020402	T10B11.6	T10B11.6	-0.156388668423	AGIWUR14326
CB4856	CT	I	6945596	6950499	WBGene00020403	T10B11.7	T10B11.7	-0.667569605707873	AGIWUR41683
CB4856	CT	I	6951135	6951982	WBGene00020397	pcbd-1	T10B11.1	-0.102272917413904	AGIWUR16908
CB4856	CT	I	6951900	6954252	WBGene00020404	T10B11.8	T10B11.8	-0.270960172194698	AGIWUR11281
CB4856	CT	I	6954414	6961139	WBGene00016260	rege-1	C30F12.1	0.448444334438689	AGIWUR409;AGIWUR6954;AGIWUR24743;AGIWUR28854;AGIWUR31266;AGIWUR32912
CB4856	CT	I	6967670	6970190	WBGene00016261	C30F12.2	C30F12.2	-0.254333215361028	AGIWUR4610;AGIWUR34084
CB4856	CT	I	6970367	6971879	WBGene00016262	C30F12.3	C30F12.3	0.357590631563767	AGIWUR15866
CB4856	CT	I	6972157	6973789	WBGene00016263	C30F12.4	C30F12.4	-0.177413981345701	AGIWUR17475
CB4856	CT	I	6974558	6977100	WBGene00016266	idhg-2	C30F12.7	0.0675867822148835	AGIWUR38700
CB4856	CT	I	6989067	6992106	WBGene00016265	trhr-1	C30F12.6	-0.155712608525908	AGIWUR4257
CB4856	CT	I	6991951	6999706	WBGene00016264	C30F12.5	C30F12.5	-0.0491903380585681	AGIWUR3647;AGIWUR41737
CB4856	CT	I	7002870	7003481	WBGene00010371	H06O01.4	H06O01.4	0.0137301136707091	AGIWUR24652
CB4856	CT	I	7005443	7009190	WBGene00010370	ctg-1	H06O01.3	0.113125702234791	AGIWUR39363
CB4856	CT	I	7009139	7015212	WBGene00010369	chd-1	H06O01.2	0.0816633361336656	AGIWUR350
CB4856	CT	I	7015912	7021743	WBGene00003964	pdi-3	H06O01.1	-0.159084225228519	AGIWUR20997;AGIWUR22066

CB4856 CT I 7021946 7029076 WBGene00008852 ubql-1 F15C11.2 -0.0820162638820863 AGIWUR2160;AGIWUR4824;AGIWUR10320;AGIWUR10692;AGIWUR12397;AGIWUR13433;AGIWUR13487;AGIWUR23339;AGIWUR37152;AGIWUR44055
CB4856 CT I 7038900 7048832 WBGene00004773 sem-4 F15C11.1 0.2672079400147 AGIWUR17306;AGIWUR32821;AGIWUR36613;AGIWUR38195;AGIWUR39733
CB4856 CT I 7050042 7051712 WBGene00044764 W01A8.7 W01A8.7 0.0648399420292911 AGIWUR24025
CB4856 CT I 7057170 7059351 WBGene00007024 plin-1 W01A8.1 -0.0578641706640788 AGIWUR6882;AGIWUR21896;AGIWUR25710;AGIWUR39335;AGIWUR41766
CB4856 CT I 7065263 7067899 WBGene00012165 cutl-6 W01A8.3 0.13193128527933 AGIWUR6403
CB4856 CT I 7068106 7069186 WBGene00012166 nuo-6 W01A8.4 -0.348681939243507 AGIWUR5024;AGIWUR14516;AGIWUR20848
CB4856 CT I 7069440 7070728 WBGene00012167 tofu-5 W01A8.5 -0.0935593003698365 AGIWUR10884;AGIWUR16983;AGIWUR23898;AGIWUR29769;AGIWUR44504
CB4856 CT I 7070649 7074733 WBGene00012168 W01A8.6 W01A8.6 -0.461273979448233 AGIWUR2324
CB4856 CT I 7075918 7079566 WBGene00002198 kin-14 F22D6.1 0.0590256299527372 AGIWUR11797
CB4856 CT I 7079868 7081520 WBGene00009050 F22D6.2 F22D6.2 0.295206754071178 AGIWUR7047;AGIWUR7697;AGIWUR28913;AGIWUR33699
CB4856 CT I 7088030 7090062 WBGene00003815 nars-1 F22D6.3 -0.225190863806272 AGIWUR8353;AGIWUR28891;AGIWUR31912
CB4856 CT I 7090623 7091371 WBGene00009051 nduf-6 F22D6.4 -0.156650928026524 AGIWUR24434
CB4856 CT I 7091309 7094537 WBGene00004186 prpf-4 F22D6.5 0.65996016345921 AGIWUR11477
CB4856 CT I 7095486 7098406 WBGene00009052 ekl-1 F22D6.6 -0.221094218737164 AGIWUR22881
CB4856 CT I 7098981 7099497 WBGene00043279 F22D6.8 F22D6.8 -0.00247600955852612 AGIWUR5007;AGIWUR5895;AGIWUR19127;AGIWUR27377;AGIWUR36296;AGIWUR44085
CB4856 CT I 7099810 7101775 WBGene00009054 F22D6.9 F22D6.9 0.204447405197659 AGIWUR19730
CB4856 CT I 7102216 7103392 WBGene00009056 F22D6.15 F22D6.15 0.0213317222145761 AGIWUR11066;AGIWUR24483;AGIWUR44167
CB4856 CT I 7105302 7106926 WBGene00000636 col-60 F22D6.10 -0.433979226447438 AGIWUR14941
CB4856 CT I 7110240 7112287 WBGene00001643 gly-18 F22D6.11 0.0995759715923974 AGIWUR10272
CB4856 CT I 7112903 7115001 WBGene00001644 gly-19 F22D6.12 0.139323717345831 AGIWUR30017;AGIWUR32849
CB4856 CT I 7116092 7116889 WBGene00010740 K10D3.6 K10D3.6 2.28108583786673 AGIWUR34630;AGIWUR43978
CB4856 CT I 7117808 7121545 WBGene00001614 glr-3 K10D3.1 -0.0544093747325247 AGIWUR1117;AGIWUR20863
CB4856 CT I 7121657 7122194 WBGene00219281 K10D3.8 K10D3.8 0.414557780966465 AGIWUR40179
CB4856 CT I 7122614 7128423 WBGene00006753 unc-14 K10D3.2 -0.0428167718122507

									AGIWUR5593;AGIWUR16242;AGIWUR16992;AGIWUR17744;AGIWUR22269;AGIWUR37434
CB4856	CT	I	7132747	7134312	WBGene00006394	taf-11.2	K10D3.3	-0.267359050849556	AGIWUR23016;AGIWUR35624;AGIWUR37192
CB4856	CT	I	7134738	7139973	WBGene00010738	K10D3.4	K10D3.4	1.00245498361598	AGIWUR12834
CB4856	CT	I	7140278	7147230	WBGene00010427	hpo-11	H37N21.1	-0.0466738854576443	AGIWUR9880;AGIWUR43725
CB4856	CT	I	7149937	7151887	WBGene00010645	fecl-1	K07G5.6	-0.0567179494555408	AGIWUR6918
CB4856	CT	I	7152463	7156868	WBGene00010641	crml-1	K07G5.1	0.000434510176910763	AGIWUR923;AGIWUR2297;AGIWUR24001;AGIWUR40638
CB4856	CT	I	7159714	7160917	WBGene00006963	xpa-1	K07G5.2	0.273101996104565	AGIWUR9973
CB4856	CT	I	7161016	7166518	WBGene00010642	mks-6	K07G5.3	0.202606105196749	AGIWUR17006;AGIWUR35676
CB4856	CT	I	7166932	7168783	WBGene00010643	K07G5.4	K07G5.4	0.258360753195005	AGIWUR19839
CB4856	CT	I	7169997	7172189	WBGene00010644	K07G5.5	K07G5.5	-0.0291832908085965	AGIWUR36732
CB4856	CT	I	7172463	7174156	WBGene00010870	let-522	M05B5.2	0.619649390227796	AGIWUR13948
CB4856	CT	I	7177953	7179430	WBGene00010869	M05B5.1	M05B5.1	0.242490355704046	AGIWUR15124
CB4856	CT	I	7183053	7185709	WBGene00010873	trpa-2	M05B5.6	0.437321686346468	AGIWUR13437
CB4856	CT	I	7185695	7188298	WBGene00010871	M05B5.3	M05B5.3	0.0308767687465146	AGIWUR6763
CB4856	CT	I	7188465	7190581	WBGene00010872	M05B5.4	M05B5.4	-0.0894862008897306	AGIWUR6384;AGIWUR17510
CB4856	CT	I	7190834	7193924	WBGene00001949	hlh-2	M05B5.5	-0.0750260887389977	AGIWUR8155;AGIWUR10611;AGIWUR18355
CB4856	CT	I	7202242	7203533	WBGene00000637	col-61	C01H6.1	-0.0573077344776056	AGIWUR17413
CB4856	CT	I	7204541	7207717	WBGene00007252	mlt-2	C01H6.2	-0.18254362439766	AGIWUR13393
CB4856	CT	I	7208028	7209866	WBGene00007253	C01H6.3	C01H6.3	0.0683154233710876	AGIWUR12247
CB4856	CT	I	7210515	7212579	WBGene00007254	C01H6.4	C01H6.4	-0.412015142339713	AGIWUR31742;AGIWUR32511
CB4856	CT	I	7214745	7221781	WBGene00003622	nhr-23	C01H6.5	-0.166609414528182	AGIWUR5349;AGIWUR8686;AGIWUR11774;AGIWUR22468;AGIWUR27636;AGIWUR31994;AGIWUR33632;AGIWUR34495;AGIWUR36432
CB4856	CT	I	7222296	7224381	WBGene00007255	cnnm-4	C01H6.6	0.202564887467237	AGIWUR485;AGIWUR20649;AGIWUR41720
CB4856	CT	I	7224656	7227767	WBGene00007256	swsn-9	C01H6.7	0.0897670819628988	AGIWUR16237
CB4856	CT	I	7227859	7229434	WBGene00007257	C01H6.8	C01H6.8	-0.52953927607063	AGIWUR18870;AGIWUR23323;AGIWUR27055;AGIWUR27094
CB4856	CT	I	7229433	7233121	WBGene00007258	hasp-1	C01H6.9	-0.121270889703902	AGIWUR21484;AGIWUR22965
CB4856	CT	I	7233645	7234501	WBGene00011062	R06C7.2	R06C7.2	-0.14204187885966	AGIWUR43578
CB4856	CT	I	7235371	7238790	WBGene00011061	wago-1	R06C7.1	-0.157434539172828	AGIWUR2605;AGIWUR31860

CB4856	CT	I	7238994	7243967	WBGene00000963	dhp-1	R06C7.3	0.101139591656294	AGIWUR11332;AGIWUR32947
CB4856	CT	I	7246849	7248741	WBGene00011064	adsl-1	R06C7.5	-0.235855603791614	AGIWUR2006;AGIWUR8376;AGIWUR29052;AGIWUR41518
CB4856	CT	I	7248828	7251422	WBGene00003041	lin-61	R06C7.7	-0.224589241233766	AGIWUR29775;AGIWUR42249
CB4856	CT	I	7251941	7255223	WBGene00000275	bub-1	R06C7.8	-0.145253684876202	AGIWUR20021
CB4856	CT	I	7255380	7258700	WBGene00011066	ztf-15	R06C7.9	-0.118174147847693	AGIWUR32881
CB4856	CT	I	7258915	7267117	WBGene00002348	myo-1	R06C7.10	0.375413958057909	AGIWUR24541
CB4856	CT	I	7269179	7273005	WBGene00006656	twk-1	F21C3.1	-0.0231452456112269	AGIWUR16353
CB4856	CT	I	7274282	7278573	WBGene00009001	tyr-3	F21C3.2	-0.221359097946483	AGIWUR19725
CB4856	CT	I	7278802	7279609	WBGene00077548	F21C3.7	F21C3.7	-0.0218432982600163	AGIWUR21181;AGIWUR29371;AGIWUR42066
CB4856	CT	I	7279658	7280501	WBGene00009002	hint-1	F21C3.3	-0.121716875268065	AGIWUR34525
CB4856	CT	I	7280552	7282871	WBGene00004324	rde-2	F21C3.4	-0.285789319606192	AGIWUR37884
CB4856	CT	I	7282964	7283818	WBGene00009004	pfd-6	F21C3.5	-0.337873266181504	AGIWUR14191
CB4856	CT	I	7283907	7285762	WBGene00009005	F21C3.6	F21C3.6	-0.224614589034596	AGIWUR6232
CB4856	CT	I	7291272	7293331	WBGene00008762	ztf-2	F13G3.1	-0.173215691121419	AGIWUR38805
CB4856	CT	I	7294038	7295687	WBGene00005131	srd-53	F13G3.2	-0.164115991128048	AGIWUR18194
CB4856	CT	I	7295753	7297004	WBGene00008770	F13G3.12	F13G3.12	-0.194155295727602	AGIWUR44355
CB4856	CT	I	7296913	7299983	WBGene00008763	F13G3.3	F13G3.3	0.00801820038816992	AGIWUR37892
CB4856	CT	I	7300062	7300913	WBGene00008764	dylt-1	F13G3.4	0.00375659437850651	AGIWUR23442;AGIWUR40549
CB4856	CT	I	7301007	7303227	WBGene00008765	ttx-7	F13G3.5	-0.0992434015266284	AGIWUR26166;AGIWUR32307
CB4856	CT	I	7303300	7304690	WBGene00008766	F13G3.6	F13G3.6	0.0902501925040422	AGIWUR33952
CB4856	CT	I	7304918	7307010	WBGene00008767	F13G3.7	F13G3.7	-0.169531541752391	AGIWUR7091;AGIWUR24366;AGIWUR27990
CB4856	CT	I	7307117	7308626	WBGene00008769	mrpl-13	F13G3.11	-0.132274964814571	AGIWUR7546;AGIWUR8371;AGIWUR24043;AGIWUR27410;AGIWUR37354;AGIWUR37886
CB4856	CT	I	7308603	7311939	WBGene00001136	eat-5	F13G3.8	-0.0699117976610326	AGIWUR15708
CB4856	CT	I	7312797	7313525	WBGene00003236	mif-3	F13G3.9	-0.125848639772778	AGIWUR6050
CB4856	CT	I	7313585	7314277	WBGene00008768	F13G3.10	F13G3.10	0.234661609060883	AGIWUR10810
CB4856	CT	I	7317646	7318337	WBGene00009915	F52A8.1	F52A8.1	-0.169182215364309	AGIWUR17070;AGIWUR32537
CB4856	CT	I	7318290	7321085	WBGene00001680	gpb-2	F52A8.2	0.298913104578366	

AGIWUR5017;AGIWUR30919
CB4856	CT	I	7322270 7324415 WBGene00009916	F52A8.3 F52A8.3 -0.155462623398251
AGIWUR15576;AGIWUR38874;AGIWUR39839
CB4856	CT	I	7339204 7345949 WBGene00009917	glb-18	F52A8.4 -0.072583917760257	AGIWUR25073
CB4856	CT	I	7345932 7347427 WBGene00009918	gcsh-2	F52A8.5 0.234147983939689	AGIWUR45177
CB4856	CT	I	7347222 7350386 WBGene00009919	kbrl-1	F52A8.6 -0.211644904561617
AGIWUR12791;AGIWUR16926;AGIWUR22612;AGIWUR29694;AGIWUR39394;AGIWUR40419
CB4856	CT	I	7350312 7352235 WBGene00002136	inx-14	F07A5.1 -0.0147066292726397
AGIWUR519;AGIWUR43444
CB4856	CT	I	7352415 7353701 WBGene00008541	F07A5.2 F07A5.2 0.388120675705949	AGIWUR31313
CB4856	CT	I	7354491 7357180 WBGene00008542	scav-6	F07A5.3 -0.464576981762992	AGIWUR34532
CB4856	CT	I	7357180 7359100 WBGene00008543	F07A5.4 F07A5.4 0.176243227200091
AGIWUR5394;AGIWUR32250;AGIWUR43624
CB4856	CT	I	7360199 7371534 WBGene00006306	sue-1	F07A5.5 0.0758621369732912
AGIWUR7614;AGIWUR42629
CB4856	CT	I	7376604 7383197 WBGene00006754	unc-15	F07A5.7 -0.093299822285946
AGIWUR979;AGIWUR5073;AGIWUR7505;AGIWUR11096;AGIWUR28192;AGIWUR34853;AGIWUR44241
CB4856	CT	I	7384398 7388792 WBGene00007597	C15A11.2	C15A11.2	0.0535921102014694
AGIWUR25007
CB4856	CT	I	7389049 7395918 WBGene00004944	sol-1	C15A11.3	0.44778318692355
AGIWUR36489
CB4856	CT	I	7398824 7401023 WBGene00007598	C15A11.4	C15A11.4	-0.880892316076086
AGIWUR3755
CB4856	CT	I	7404950 7408511 WBGene00007599	C15A11.7	C15A11.7	0.327137282630035
AGIWUR5504;AGIWUR10256;AGIWUR20323
CB4856	CT	I	7422785 7454088 WBGene00006752	unc-13	ZK524.2 0.352644785644572	AGIWUR25843
CB4856	CT	I	7454660 7456953 WBGene00004958	spe-4	ZK524.1 0.366434681859365	AGIWUR9983
CB4856	CT	I	7457153 7460756 WBGene00003074	lars-2	ZK524.3 0.00558529301445532	AGIWUR37098
CB4856	CT	I	7460858 7466424 WBGene00013994	ZK524.4 ZK524.4 0.0658978629567901
AGIWUR7548;AGIWUR15682;AGIWUR18485;AGIWUR23215;AGIWUR24454;AGIWUR32072;AGIWUR39429;AGIWUR40886
CB4856	CT	I	7472611 7476946 WBGene00012136	T28F4.1 T28F4.1 0.303854720208385	AGIWUR23973
CB4856	CT	I	7480108 7483392 WBGene00012137	asic-2	T28F4.2 0.0885201125038018	AGIWUR28627
CB4856	CT	I	7484159 7486302 WBGene00012138	T28F4.3 T28F4.3 -0.184574728986656
AGIWUR3863;AGIWUR23076;AGIWUR41466
CB4856	CT	I	7486577 7490174 WBGene00012139	T28F4.4 T28F4.4 -0.122347439858036	AGIWUR26867
CB4856	CT	I	7491816 7492934 WBGene00012140	T28F4.5 T28F4.5 0.191731919572529
AGIWUR3589;AGIWUR3804;AGIWUR5333;AGIWUR18046;AGIWUR25627;AGIWUR36240
CB4856	CT	I	7492788 7494700 WBGene00012141	T28F4.6 T28F4.6 -0.0998763792409303	AGIWUR22039
CB4856	CT	I	7495437 7503577 WBGene00007042	pbrm-1	C26C6.1 -0.0196883822443912

AGIWUR4581;AGIWUR8361;AGIWUR8965;AGIWUR20774
CB4856 CT I 7503576 7505580 WBGene00007742 C26C6.8 C26C6.8 0.0131628649572115
AGIWUR24774;AGIWUR26471
CB4856 CT I 7505951 7506693 WBGene00007740 C26C6.6 C26C6.6 -0.255497369683026 AGIWUR1050
CB4856 CT I 7508721 7512593 WBGene00007741 glb-8 C26C6.7 -0.00673280359052697 AGIWUR32542
CB4856 CT I 7512918 7513726 WBGene00077553 C26C6.10 C26C6.10 -0.335683051618334
AGIWUR8783;AGIWUR12734;AGIWUR16803;AGIWUR31486;AGIWUR45154
CB4856 CT I 7514117 7515126 WBGene00007743 C26C6.9 C26C6.9 -0.704491962546019 AGIWUR8485
CB4856 CT I 7522116 7526703 WBGene00001648 goa-1 C26C6.2 0.136737331407225
AGIWUR13528;AGIWUR17695;AGIWUR38775;AGIWUR41116
CB4856 CT I 7527224 7531715 WBGene00003552 nas-36 C26C6.3 -0.304085061239477 AGIWUR16556
CB4856 CT I 7532315 7535997 WBGene00007739 C26C6.4 C26C6.4 0.302591900549819 AGIWUR19863
CB4856 CT I 7536204 7542157 WBGene00000938 dcp-66 C26C6.5 -0.00616084859528954
AGIWUR1334;AGIWUR14944;AGIWUR20106;AGIWUR32961;AGIWUR44832
CB4856 CT I 7552975 7554553 WBGene00012029 T25G3.1 T25G3.1 -0.124448140629301 AGIWUR18597
CB4856 CT I 7554855 7559916 WBGene00000496 chs-1 T25G3.2 -0.147378396381441 AGIWUR19128
CB4856 CT I 7559839 7562349 WBGene00012030 T25G3.3 T25G3.3 -0.0179460405465406
AGIWUR3427;AGIWUR4620;AGIWUR5004;AGIWUR7005;AGIWUR12640;AGIWUR22358;AGIWUR23711;AGIWUR27358;AGIWUR31286;AGIWUR31532;AGIWUR40279
CB4856 CT I 7564584 7567719 WBGene00012031 T25G3.4 T25G3.4 0.0772595545874926
AGIWUR186;AGIWUR12674;AGIWUR13829;AGIWUR42301
CB4856 CT I 7567775 7571250 WBGene00008411 mans-1 D2030.1 -0.0805599596926072
AGIWUR1155;AGIWUR3168;AGIWUR4637;AGIWUR12685;AGIWUR20584;AGIWUR26209;AGIWUR26452;AGIWUR30258;AGIWUR38235;AGIWUR41375;AGIWUR42688
CB4856 CT I 7574195 7578564 WBGene00008412 D2030.2 D2030.2 0.689744604105314
AGIWUR13197;AGIWUR43842;AGIWUR43976
CB4856 CT I 7578512 7580833 WBGene00008413 D2030.3 D2030.3 0.221469993257334
AGIWUR13349;AGIWUR37279
CB4856 CT I 7580944 7581628 WBGene00008414 D2030.4 D2030.4 -0.193012288152932 AGIWUR31361
CB4856 CT I 7582106 7583349 WBGene00008415 mce-1 D2030.5 -0.0012748703639085
AGIWUR7421;AGIWUR9392;AGIWUR21647;AGIWUR32440;AGIWUR39252;AGIWUR44166
CB4856 CT I 7583810 7585340 WBGene00008421 D2030.12 D2030.12 0.115554868299643
AGIWUR34170
CB4856 CT I 7585324 7588367 WBGene00004178 prg-1 D2030.6 0.400930423387765 AGIWUR10305
CB4856 CT I 7588580 7590565 WBGene00008417 D2030.7 D2030.7 -0.274751864217162 AGIWUR29435
CB4856 CT I 7590543 7591204 WBGene00008420 D2030.11 D2030.11 -0.0709382060580594
AGIWUR30935
CB4856 CT I 7591288 7595198 WBGene00008418 D2030.8 D2030.8 0.0602004779500867 AGIWUR31838
CB4856 CT I 7596507 7602203 WBGene00008419 wdr-23 D2030.9 0.0316680306901906

AGIWUR1501;AGIWUR4494;AGIWUR8516;AGIWUR23572;AGIWUR25857;AGIWUR41529
CB4856 CT I 7602307 7608245 WBGene00000084 aex-1 D2030.10 0.076013363586215
AGIWUR10350;AGIWUR16639;AGIWUR17325;AGIWUR19915;AGIWUR29156;AGIWUR30595;AGIWUR37321
CB4856 CT I 7614176 7634135 WBGene00003071 lrp-1 F29D11.1 0.346217191181371
AGIWUR29566
CB4856 CT I 7634494 7639780 WBGene00009254 capg-1 F29D11.2 -0.0652499098739874
AGIWUR1032;AGIWUR21417
CB4856 CT I 7640338 7641767 WBGene00009140 F26A3.1 F26A3.1 0.0496104216495752
AGIWUR2059;AGIWUR17278;AGIWUR34924
CB4856 CT I 7642270 7643328 WBGene00009141 ncbp-2 F26A3.2 -0.130266077509713 AGIWUR28835
CB4856 CT I 7643431 7649777 WBGene00004508 rrf-1 F26A3.8 -0.133657380876906
AGIWUR5335;AGIWUR25229
CB4856 CT I 7650418 7656336 WBGene00001214 ego-1 F26A3.3 -0.144315016811264 AGIWUR44352
CB4856 CT I 7656978 7658291 WBGene00009142 F26A3.4 F26A3.4 0.817742076832591
AGIWUR14432;AGIWUR20058;AGIWUR35343
CB4856 CT I 7667729 7669900 WBGene00009143 F26A3.5 F26A3.5 -0.41013149544593 AGIWUR25193
CB4856 CT I 7670314 7672714 WBGene00009144 del-3 F26A3.6 0.528785720131898 AGIWUR30425
CB4856 CT I 7674734 7676127 WBGene00009145 F26A3.7 F26A3.7 -0.158330065119845 AGIWUR23284
CB4856 CT I 7676668 7679061 WBGene00011975 T24B1.1 T24B1.1 -0.273230695694348 AGIWUR18387
CB4856 CT I 7679454 7683041 WBGene00011972 T23G11.7 T23G11.7 -0.0823041108551497
AGIWUR25258;AGIWUR36273
CB4856 CT I 7683341 7694210 WBGene00011970 rlbp-1 T23G11.5 -0.0402616305881587
AGIWUR25068
CB4856 CT I 7694325 7695233 WBGene00011969 T23G11.4 T23G11.4 -0.17620802147076
AGIWUR38478
CB4856 CT I 7695661 7698227 WBGene00001595 gld-1 T23G11.3 -0.208760705910668
AGIWUR3931;AGIWUR7691;AGIWUR8949;AGIWUR9950;AGIWUR13665;AGIWUR16668;AGIWUR17200;AGIWUR25690;AGIWUR30259;AGIWUR35080;AGIWUR37229;AGIWUR42811
CB4856 CT I 7698301 7699037 WBGene00011973 T23G11.10 T23G11.10 0.0614069104078219
AGIWUR28833
CB4856 CT I 7699543 7700958 WBGene00001647 gna-2 T23G11.2 -0.0767910240100704
AGIWUR38355
CB4856 CT I 7701365 7702053 WBGene00011968 T23G11.1 T23G11.1 0.306769717594372
AGIWUR4190
CB4856 CT I 7702429 7704584 WBGene00009187 F27D4.1 F27D4.1 -0.287901707953839 AGIWUR30511
CB4856 CT I 7704565 7707049 WBGene00009188 lsy-22 F27D4.2 -0.0985193026921873
AGIWUR3195;AGIWUR10700;AGIWUR10886;AGIWUR11509;AGIWUR13652;AGIWUR16965;AGIWUR18957;AGIWUR21690;AGIWUR23469;AGIWUR26882;AGIWUR38016;AGIWUR41279
CB4856 CT I 7723754 7724656 WBGene00009191 F27D4.7 F27D4.7 -0.0915873520581307 AGIWUR32828

CB4856 CT   I   7725019 7727112 WBGene00009189 F27D4.4 F27D4.4 0.0505864127345959   AGIWUR45003
CB4856 CT   I   7727267 7729092 WBGene00006518 bckd-1B F27D4.5 -0.27447396070361
AGIWUR6557;AGIWUR28991;AGIWUR29478
CB4856 CT   I   7729184 7729878 WBGene00077543 F27D4.8 F27D4.8 0.119567055816107
AGIWUR8217;AGIWUR12100;AGIWUR27032;AGIWUR32572;AGIWUR43980
CB4856 CT   I   7731121 7737995 WBGene00009190 F27D4.6 F27D4.6 0.371926208241888
AGIWUR9006;AGIWUR33873;AGIWUR45113
CB4856 CT   I   7755778 7756353 WBGene00010291 F58H10.1   F58H10.1   0.116156366940282
AGIWUR742;AGIWUR9492;AGIWUR18367;AGIWUR22334;AGIWUR37012;AGIWUR40797;AGIWUR44521
CB4856 CT   I   7766532 7772231 WBGene00003818 nsf-1   H15N14.2   -0.0562962551675348
AGIWUR5898;AGIWUR23021;AGIWUR40675
CB4856 CT   I   7773180 7777652 WBGene00000079 adr-1   H15N14.1   -0.0431201108676147
AGIWUR36832;AGIWUR41920
CB4856 CT   I   7786205 7793539 WBGene00008401 D2005.6 D2005.6 0.302628604880126   AGIWUR22650
CB4856 CT   I   7800458 7801822 WBGene00003746 nlp-8   D2005.2 0.660241716920275   AGIWUR14278
CB4856 CT   I   7813761 7815042 WBGene00008398 D2005.3 D2005.3 0.161516261065358   AGIWUR39084
CB4856 CT   I   7819579 7820489 WBGene00086551 D2005.7 D2005.7 -0.0730266496256142   AGIWUR22887
CB4856 CT   I   7820831 7826433 WBGene00008400 drh-3   D2005.5 -0.189633530531351   AGIWUR6818
CB4856 CT   I   7826442 7831629 WBGene00009271 glna-3 F30F8.2 0.133955353241952   AGIWUR18761
CB4856 CT   I   7832692 7835453 WBGene00009270 F30F8.1 F30F8.1 -0.149974286391807   AGIWUR20487
CB4856 CT   I   7835814 7836967 WBGene00009272 gras-1 F30F8.3 -0.10667685127966   AGIWUR42039
CB4856 CT   I   7837063 7840022 WBGene00009276 F30F8.9 F30F8.9 -0.055452837579673
AGIWUR12971;AGIWUR13463
CB4856 CT   I   7840442 7846923 WBGene00009277 F30F8.10   F30F8.10   -0.255646945595532
AGIWUR8230;AGIWUR31083;AGIWUR35509;AGIWUR43910
CB4856 CT   I   7847246 7848920 WBGene00009274 F30F8.5 F30F8.5 -0.0699214885255905   AGIWUR42023
CB4856 CT   I   7848975 7853631 WBGene00006386 taf-5   F30F8.8 0.26510367830166   AGIWUR13028
CB4856 CT   I   7853750 7857850 WBGene00000163 apb-3   R11A5.1 -0.132870237998521   AGIWUR978
CB4856 CT   I   7857823 7859417 WBGene00011230 nud-2   R11A5.2 0.00747842472508076   AGIWUR24764
CB4856 CT   I   7862736 7863553 WBGene00011231 R11A5.3 R11A5.3 0.239603809761654   AGIWUR16938
CB4856 CT   I   7866136 7872080 WBGene00011232 pck-2   R11A5.4 0.158306421826765   AGIWUR14830
CB4856 CT   I   7872057 7875554 WBGene00011235 suro-1 R11A5.7 -0.341434157396592
AGIWUR1591;AGIWUR9643;AGIWUR18550;AGIWUR25192;AGIWUR29119
CB4856 CT   I   7884319 7885769 WBGene00010110 F55D12.1   F55D12.1   -0.228348280061537
AGIWUR28202
CB4856 CT   I   7889310 7894864 WBGene00010111 F55D12.2   F55D12.2   -0.372237296164135
AGIWUR30725
CB4856 CT   I   7896125 7897725 WBGene00010112 nhr-191 F55D12.3   0.107145927063351
AGIWUR40247

CB4856	CT	I	7899037 7903334 WBGene00006790	unc-55	F55D12.4	0.0884452507523719
AGIWUR29846
CB4856	CT	I	7906826 7914269 WBGene00010113	F55D12.5	F55D12.5	0.038967052128605
AGIWUR6230
CB4856	CT	I	7913937 7918341 WBGene00008991	F20G4.2 F20G4.2 -0.153252167926677
AGIWUR833;AGIWUR9193;AGIWUR38969
CB4856	CT	I	7918443 7926338 WBGene00008990	smgl-1	F20G4.1 -0.0529073269552391	AGIWUR24802
CB4856	CT	I	7926623 7934577 WBGene00003777	nmy-2	F20G4.3 -0.109443339644514
AGIWUR1539;AGIWUR3678;AGIWUR4118;AGIWUR15521;AGIWUR21394;AGIWUR21683;AGIWUR26098;AGIWUR31723;AGIWUR36619;AG
IWUR38685;AGIWUR42013;AGIWUR45084
CB4856	CT	I	7936223 7938328 WBGene00011912	T22C1.1 T22C1.1 -0.288471173109482	AGIWUR12142
CB4856	CT	I	7938560 7940388 WBGene00011914	T22C1.3 T22C1.3 -0.0424285818305172	AGIWUR24799
CB4856	CT	I	7940893 7941994 WBGene00011916	T22C1.5 T22C1.5 0.0223711701254494
AGIWUR618;AGIWUR3363;AGIWUR8190;AGIWUR8908;AGIWUR9720;AGIWUR28946
CB4856	CT	I	7942719 7945588 WBGene00011917	T22C1.6 T22C1.6 0.0392626450402656	AGIWUR19335
CB4856	CT	I	7946186 7951593 WBGene00002179	jph-1	T22C1.7 -0.107290874216447
AGIWUR3743;AGIWUR13804;AGIWUR18557;AGIWUR18715;AGIWUR22762;AGIWUR37124
CB4856	CT	I	7955977 7958505 WBGene00011918	T22C1.8 T22C1.8 -0.0935790604977438	AGIWUR19143
CB4856	CT	I	7958647 7960158 WBGene00011919	T22C1.9 T22C1.9 -0.0104746018004418
AGIWUR14647;AGIWUR17388;AGIWUR33828;AGIWUR40991
CB4856	CT	I	7960388 7961460 WBGene00011921	T22C1.12	T22C1.12	-0.153023784496485
AGIWUR39086
CB4856	CT	I	7962052 7972443 WBGene00004318	rbg-2	T22C1.10	0.217273126773593
AGIWUR34380
CB4856	CT	I	7972473 7977078 WBGene00010366	H05L14.1	H05L14.1	-0.058226267164379
AGIWUR3563
CB4856	CT	I	7980237 7993159 WBGene00010367	H05L14.2	H05L14.2	-0.322408259194546
AGIWUR24280
CB4856	CT	I	7995738 7996669 WBGene00008315	C54G4.5 C54G4.5 -0.121363441551567	AGIWUR19383
CB4856	CT	I	7996728 8003179 WBGene00008314	C54G4.4 C54G4.4 -0.0695689194646754	AGIWUR2268
CB4856	CT	I	8005127 8006448 WBGene00008313	C54G4.3 C54G4.3 -0.0808485002088339
AGIWUR11601;AGIWUR24007;AGIWUR43355
CB4856	CT	I	8006650 8009118 WBGene00008312	C54G4.2 C54G4.2 -0.0863445599574488	AGIWUR25325
CB4856	CT	I	8014412 8019572 WBGene00008311	rskn-2	C54G4.1 0.181409909180193
AGIWUR7952;AGIWUR17948;AGIWUR28022
CB4856	CT	I	8019501 8022730 WBGene00008317	C54G4.7 C54G4.7 0.144724684141627
AGIWUR32071;AGIWUR34039;AGIWUR43802
CB4856	CT	I	8022674 8024422 WBGene00000869	cyc-1	C54G4.8 -0.0148603705055139
AGIWUR8905;AGIWUR9205;AGIWUR12984;AGIWUR23480;AGIWUR28992;AGIWUR36574

CB4856	CT	I	8024880 8026525 WBGene00008318	C54G4.9 C54G4.9 -0.122636237442208	AGIWUR42787
CB4856	CT	I	8026606 8028752 WBGene00010560	eif-2beta	K04G2.1 -0.296647814373998
AGIWUR9491;AGIWUR22485;AGIWUR24936;AGIWUR35928;AGIWUR40176
CB4856	CT	I	8030959 8032886 WBGene00045192	aho-3	K04G2.2 -0.0152667936727031	AGIWUR36029
CB4856	CT	I	8032863 8035696 WBGene00010562	cdc-48.3	K04G2.3 0.0560518477067653
AGIWUR8959
CB4856	CT	I	8036944 8039752 WBGene00010564	ath-1	K04G2.5 -0.0993155924507736
AGIWUR7565;AGIWUR22477;AGIWUR35481
CB4856	CT	I	8039879 8040526 WBGene00010569	scbp-2	K04G2.11	-0.0313156202019638
AGIWUR10517
CB4856	CT	I	8040799 8043277 WBGene00010565	vacl-14 K04G2.6 -0.0837208225868231	AGIWUR459
CB4856	CT	I	8047345 8049351 WBGene00010566	K04G2.7 K04G2.7 -0.0246883779381948	AGIWUR13374
CB4856	CT	I	8050043 8055489 WBGene00000156	apr-1	K04G2.8 0.047937313859356
AGIWUR2386;AGIWUR8642;AGIWUR15749;AGIWUR23225;AGIWUR24248;AGIWUR24940;AGIWUR33692;AGIWUR41192
CB4856	CT	I	8055459 8057029 WBGene00010567	K04G2.9 K04G2.9 -0.135045579012774	AGIWUR13708
CB4856	CT	I	8057762 8060557 WBGene00010568	K04G2.10	K04G2.10	0.0197383346511533
AGIWUR31263
CB4856	CT	I	8060791 8061793 WBGene00008935	F18C12.4	F18C12.4	-0.172839037491405
AGIWUR11272
CB4856	CT	I	8062059 8081671 WBGene00000485	che-3	F18C12.1	0.302222036913637
AGIWUR19754;AGIWUR31660;AGIWUR42151
CB4856	CT	I	8083583 8095574 WBGene00004378	rme-8	F18C12.2	-0.0615682481788937
AGIWUR1718;AGIWUR4249
CB4856	CT	I	8095740 8098444 WBGene00008934	F18C12.3	F18C12.3	-0.215526745102625
AGIWUR23155
CB4856	CT	I	8103601 8109227 WBGene00005009	spr-4	C09H6.1 0.0275530414824203	AGIWUR22535
CB4856	CT	I	8109746 8119314 WBGene00002999	lin-10	C09H6.2 0.195066250027043	AGIWUR34214
CB4856	CT	I	8125801 8130091 WBGene00003136	mau-2	C09H6.3 -0.114946829181044	AGIWUR29187
CB4856	CT	I	8133891 8135051 WBGene00012113	T28B8.1 T28B8.1 1.15247273670636
AGIWUR234;AGIWUR7128;AGIWUR13741;AGIWUR18450;AGIWUR31586;AGIWUR35869
CB4856	CT	I	8139468 8142537 WBGene00012116	del-4	T28B8.5 0.110856474206926	AGIWUR41269
CB4856	CT	I	8147400 8149723 WBGene00002101	ins-18	T28B8.2 0.397248327984745	AGIWUR37506
CB4856	CT	I	8150331 8151368 WBGene00044122	T28B8.6 T28B8.6 -0.114232312658498
AGIWUR3015;AGIWUR12297;AGIWUR24914
CB4856	CT	I	8151985 8159732 WBGene00012114	T28B8.3 T28B8.3 0.176143550668042	AGIWUR24289
CB4856	CT	I	8160512 8168643 WBGene00012115	T28B8.4 T28B8.4 0.0788451495639695
AGIWUR7458;AGIWUR11534;AGIWUR12150;AGIWUR29517;AGIWUR36686
CB4856	CT	I	8172721 8173489 WBGene00023413	H31B20.2	H31B20.2	-0.0110671001823883
AGIWUR3366;AGIWUR40756

CB4856 CT I 8174888 8208740 WBGene00008570 kcnl-2 F08A10.1 -0.0163102484730562
AGIWUR11841;AGIWUR13112;AGIWUR15015;AGIWUR27508;AGIWUR31988;AGIWUR43818
CB4856 CT I 8208712 8210703 WBGene00011379 T02E1.7 T02E1.7 -0.0692018024164716 AGIWUR28236
CB4856 CT I 8210931 8212411 WBGene00011378 T02E1.6 T02E1.6 0.379657953738235 AGIWUR11385
CB4856 CT I 8212362 8218361 WBGene00000967 dhs-3 T02E1.5 -0.428820111304521
AGIWUR11837;AGIWUR21469
CB4856 CT I 8219998 8223009 WBGene00011375 T02E1.2 T02E1.2 -0.116578447486155 AGIWUR13580
CB4856 CT I 8224539 8226297 WBGene00004966 spe-12 T02E1.1 0.0967084292336606 AGIWUR35507
CB4856 CT I 8226509 8240637 WBGene00013955 kri-1 ZK265.1 -0.032774248874329
AGIWUR16436;AGIWUR37773;AGIWUR41475
CB4856 CT I 8242104 8243572 WBGene00013956 ZK265.3 ZK265.3 -0.0455329486955947
AGIWUR13680;AGIWUR16508;AGIWUR18985
CB4856 CT I 8243695 8244963 WBGene00000639 col-63 ZK265.2 0.068067670956971 AGIWUR22009
CB4856 CT I 8245722 8250896 WBGene00000433 ceh-8 ZK265.4 0.654936763760716 AGIWUR32521
CB4856 CT I 8253452 8254961 WBGene00044094 fitm-2 ZK265.9 -0.0833976907338965 AGIWUR37013
CB4856 CT I 8255348 8256312 WBGene00013958 ZK265.6 ZK265.6 -0.122076719116151
AGIWUR7197;AGIWUR34833;AGIWUR42498
CB4856 CT I 8256360 8258939 WBGene00013957 sre-23 ZK265.5 0.209873488151147
AGIWUR18546;AGIWUR38634
CB4856 CT I 8262039 8268990 WBGene00013959 ZK265.7 ZK265.7 -0.18481282329417 AGIWUR1840
CB4856 CT I 8270304 8275701 WBGene00013960 cutl-8 ZK265.8 0.258000184100443
AGIWUR23965;AGIWUR30635
CB4856 CT I 8275611 8288124 WBGene00011344 T01G9.2 T01G9.2 0.171689161529486
AGIWUR22898;AGIWUR27052
CB4856 CT I 8288959 8291077 WBGene00002196 kin-10 T01G9.6 -0.084790318683666
AGIWUR14009;AGIWUR14529
CB4856 CT I 8291289 8293640 WBGene00003788 npp-2 T01G9.4 -0.162855767006612 AGIWUR31441
CB4856 CT I 8294881 8296940 WBGene00003183 mei-1 T01G9.5 -0.219302824981825
AGIWUR22653;AGIWUR25821;AGIWUR25892;AGIWUR43030
CB4856 CT I 8296917 8307566 WBGene00009920 abts-1 F52B5.1 -0.0590882191736117 AGIWUR30789
CB4856 CT I 8313228 8315432 WBGene00009923 F52B5.7 F52B5.7 0.10520579202333 AGIWUR36330
CB4856 CT I 8315898 8318250 WBGene00009921 F52B5.2 F52B5.2 -0.121639670588984
AGIWUR12506;AGIWUR37654;AGIWUR39112
CB4856 CT I 8318347 8323987 WBGene00009922 F52B5.3 F52B5.3 -0.142251254918609 AGIWUR15537
CB4856 CT I 8324120 8328853 WBGene00000467 cep-1 F52B5.5 -0.200368440938483 AGIWUR34163
CB4856 CT I 8329062 8329684 WBGene00004439 rpl-25.2 F52B5.6 0.00999946881504776
AGIWUR14531;AGIWUR21549;AGIWUR23326;AGIWUR37715;AGIWUR38978;AGIWUR41115
CB4856 CT I 8330552 8332783 WBGene00007911 C34B7.1 C34B7.1 -0.388408982966562
AGIWUR2961;AGIWUR20830;AGIWUR24915

CB4856	CT	I	8333082 8336144 WBGene00007914	mys-4	C34B7.4 -0.352008618564771	AGIWUR30760
CB4856	CT	I	8336966 8341826 WBGene00007912	C34B7.2 C34B7.2 -0.0804100199183582
AGIWUR3075;AGIWUR7142;AGIWUR24662;AGIWUR29512
CB4856	CT	I	8341709 8346023 WBGene00007913	cyp-36A1	C34B7.3 -0.460044218220787
AGIWUR26344
CB4856	CT	I	8353410 8357828 WBGene00006531	tba-5	F16D3.1 0.0468787674215245
AGIWUR20180;AGIWUR43630
CB4856	CT	I	8358254 8363858 WBGene00004684	rsd-6	F16D3.2 0.0479083863761431	AGIWUR25032
CB4856	CT	I	8363954 8369917 WBGene00008887	tbcd-1	F16D3.4 -0.284138700536463	AGIWUR15687
CB4856	CT	I	8377601 8392768 WBGene00011827	T19A6.1 T19A6.1 -0.0311387898303064
AGIWUR416;AGIWUR7327;AGIWUR31170;AGIWUR37062
CB4856	CT	I	8394728 8398626 WBGene00003596	ngp-1	T19A6.2 0.0708368833194282
AGIWUR14013;AGIWUR17089;AGIWUR36590
CB4856	CT	I	8398918 8400472 WBGene00011828	nepr-1	T19A6.3 -0.110900309284828	AGIWUR20226
CB4856	CT	I	8401389 8403939 WBGene00011829	T19A6.4 T19A6.4 0.344992006994586
AGIWUR3638;AGIWUR42337;AGIWUR43968
CB4856	CT	I	8404547 8405955 WBGene00008529	F02E9.3 F02E9.3 0.0416302186967203
AGIWUR27790;AGIWUR41404
CB4856	CT	I	8406828 8407645 WBGene00008528	F02E9.1 F02E9.1 -0.216841988162432	AGIWUR14333
CB4856	CT	I	8407802 8410804 WBGene00003014	lin-28	F02E9.2 0.0657871357398636
AGIWUR28453;AGIWUR32342
CB4856	CT	I	8413253 8416195 WBGene00008531	F02E9.7 F02E9.7 0.0382432960205215	AGIWUR27934
CB4856	CT	I	8435958 8438638 WBGene00008648	F10D11.2	F10D11.2	0.0461793471233918
AGIWUR38994
CB4856	CT	I	8439756 8440470 WBGene00008649	F10D11.3	F10D11.3	-0.0702005471731351
AGIWUR20074
CB4856	CT	I	8441543 8442452 WBGene00008650	F10D11.4	F10D11.4	0.00102689299550995
AGIWUR38060
CB4856	CT	I	8442695 8444934 WBGene00008651	F10D11.5	F10D11.5	-0.429435002450166
AGIWUR6906
CB4856	CT	I	8445351 8451023 WBGene00008652	F10D11.6	F10D11.6	-0.22421577833417
AGIWUR25958
CB4856	CT	I	8460225 8463015 WBGene00006844	unc-120 D1081.2 0.526153557634364	AGIWUR32798
CB4856	CT	I	8463356 8469309 WBGene00008381	D1081.3 D1081.3 -0.176748298100263	AGIWUR4534
CB4856	CT	I	8469922 8471852 WBGene00045508	D1081.10	D1081.10	-0.0496489118311239
AGIWUR32516;AGIWUR38018
CB4856	CT	I	8472556 8473481 WBGene00050879	D1081.11	D1081.11	-0.345336272433533
AGIWUR24143
CB4856	CT	I	8474061 8474767 WBGene00086554	D1081.12	D1081.12	0.0363070199199469

AGIWUR6030;AGIWUR32272
CB4856 CT I 8481152 8482538 WBGene00008383 D1081.5 D1081.5 -0.124380204852589 AGIWUR28444
CB4856 CT I 8482759 8485694 WBGene00008384 D1081.6 D1081.6 -0.234575948427491
AGIWUR9889;AGIWUR12027;AGIWUR23754;AGIWUR31661;AGIWUR41169
CB4856 CT I 8485876 8487043 WBGene00008387 D1081.9 D1081.9 -0.148490244208702 AGIWUR17852
CB4856 CT I 8487618 8490938 WBGene00008385 D1081.7 D1081.7 0.194040263695449 AGIWUR13110
CB4856 CT I 8491291 8495855 WBGene00008386 cdc-5L D1081.8 -0.0697650996582748 AGIWUR1233
CB4856 CT I 8498114 8502412 WBGene00000431 ceh-6 K02B12.1 0.341778044289494
AGIWUR39847
CB4856 CT I 8509041 8511412 WBGene00010496 sec-12 K02B12.3 0.179945278681178
AGIWUR33956
CB4856 CT I 8511600 8512505 WBGene00010495 K02B12.2 K02B12.2 0.616683150978989
AGIWUR10637
CB4856 CT I 8513919 8517535 WBGene00010497 axl-1 K02B12.4 0.069014457278759
AGIWUR10856;AGIWUR17622
CB4856 CT I 8517481 8526621 WBGene00010498 K02B12.5 K02B12.5 0.132304126917038
AGIWUR18930
CB4856 CT I 8529380 8531338 WBGene00006976 zhp-3 K02B12.8 -0.173549158277055
AGIWUR5845
CB4856 CT I 8531760 8534527 WBGene00001677 gpa-15 M04C7.1 -0.375845099965295 AGIWUR18844
CB4856 CT I 8540025 8544415 WBGene00010855 M04C7.4 M04C7.4 -0.0104218136653689 AGIWUR8927
CB4856 CT I 8546063 8546725 WBGene00010854 M04C7.3 M04C7.3 0.00556742514536079 AGIWUR33468
CB4856 CT I 8549513 8570998 WBGene00001651 gon-2 T01H8.5 0.0155683749987832
AGIWUR3928;AGIWUR20657;AGIWUR27009;AGIWUR28841;AGIWUR29968;AGIWUR31752;AGIWUR33924
CB4856 CT I 8571557 8573749 WBGene00011353 T01H8.2 T01H8.2 -0.291600751647874 AGIWUR21519
CB4856 CT I 8573855 8580157 WBGene00011352 rskn-1 T01H8.1 -0.206364889119454
AGIWUR2751;AGIWUR11425;AGIWUR16452;AGIWUR19665;AGIWUR25198
CB4856 CT I 8584081 8587505 WBGene00000904 daf-8 R05D11.1 0.0494298632281289
AGIWUR2662;AGIWUR6435;AGIWUR14094
CB4856 CT I 8591215 8591978 WBGene00004305 ran-4 R05D11.3 -0.15686075073878
AGIWUR243;AGIWUR4908
CB4856 CT I 8592092 8592926 WBGene00011033 R05D11.5 R05D11.5 -0.261561830140867
AGIWUR32579
CB4856 CT I 8593027 8596225 WBGene00011037 R05D11.9 R05D11.9 -0.103581096834169
AGIWUR22806
CB4856 CT I 8596333 8598552 WBGene00011034 paxt-1 R05D11.6 -0.139575241563962
AGIWUR40684
CB4856 CT I 8598658 8600923 WBGene00011032 R05D11.4 R05D11.4 -0.146127158183478
AGIWUR137;AGIWUR4605;AGIWUR11633;AGIWUR20068;AGIWUR30339;AGIWUR31404

CB4856	CT	I	8601339	8602034	WBGene00011035	snrp-27	R05D11.7	-0.217926694096985	AGIWUR38331
CB4856	CT	I	8602125	8605165	WBGene00011036	edc-3	R05D11.8	-0.218394798455847	AGIWUR28087
CB4856	CT	I	8606404	8608631	WBGene00009664	idha-1	F43G9.1	-0.00486314282683686	AGIWUR1214;AGIWUR8312;AGIWUR18719;AGIWUR29935;AGIWUR31785;AGIWUR34086;AGIWUR35501;AGIWUR37048;AGIWUR43639
CB4856	CT	I	8609925	8611066	WBGene00009665	lmd-1	F43G9.2	-0.179989315386001	AGIWUR9510;AGIWUR16545;AGIWUR21608;AGIWUR26157
CB4856	CT	I	8612728	8615175	WBGene00009666	slc-25A42	F43G9.3	0.122700316055308	AGIWUR38648
CB4856	CT	I	8615388	8616420	WBGene00009668	cfim-1	F43G9.5	-0.140174569881911	AGIWUR2016
CB4856	CT	I	8617400	8618889	WBGene00009670	F43G9.8	F43G9.8	0.272343369365155	AGIWUR17255
CB4856	CT	I	8623017	8631184	WBGene00001414	fer-1	F43G9.6	-0.606380210742801	AGIWUR32229
CB4856	CT	I	8633925	8636044	WBGene00000468	ces-1	F43G9.11	0.789381090848151	AGIWUR40030
CB4856	CT	I	8636123	8637971	WBGene00000777	cpn-1	F43G9.9	0.0437305203150444	AGIWUR7568
CB4856	CT	I	8638514	8640236	WBGene00009671	mfap-1	F43G9.10	0.133453982056238	AGIWUR36870
CB4856	CT	I	8640508	8644399	WBGene00009672	F43G9.12	F43G9.12	0.00843376277592123	AGIWUR3156;AGIWUR6132;AGIWUR7150;AGIWUR13796
CB4856	CT	I	8644460	8646304	WBGene00012150	syx-17	VF39H2L.1	-0.0293645620251937	AGIWUR33011
CB4856	CT	I	8646451	8651777	WBGene00000890	sig-7	F39H2.2	-0.264462192170783	AGIWUR6319;AGIWUR9396;AGIWUR10898;AGIWUR19443;AGIWUR40323;AGIWUR41019
CB4856	CT	I	8658874	8659967	WBGene00009562	flp-22	F39H2.1	0.268502128500343	AGIWUR19806
CB4856	CT	I	8660820	8663127	WBGene00045237	mrt-1	F39H2.5	-0.0999322527322803	AGIWUR15553
CB4856	CT	I	8663048	8664143	WBGene00006377	syp-3	F39H2.4	-0.148176009522455	AGIWUR18940
CB4856	CT	I	8664925	8666499	WBGene00009563	F39H2.3	F39H2.3	-0.31196098561043	AGIWUR1893
CB4856	CT	I	8666595	8669792	WBGene00010620	K07A12.1	K07A12.1	-0.310744809983547	AGIWUR4177
CB4856	CT	I	8676666	8681325	WBGene00010621	egg-6	K07A12.2	-0.0715479242734967	AGIWUR2537;AGIWUR6447;AGIWUR13392;AGIWUR18818;AGIWUR31594;AGIWUR44231
CB4856	CT	I	8683220	8687217	WBGene00001990	hot-5	K07A12.6	0.059392353632667	AGIWUR18893
CB4856	CT	I	8687634	8690937	WBGene00010622	K07A12.4	K07A12.4	-0.193690852879145	AGIWUR12861;AGIWUR13225;AGIWUR40426
CB4856	CT	I	8691039	8692622	WBGene00010624	mrps-15	K07A12.7	-0.493420790970085	AGIWUR271
CB4856	CT	I	8692946	8693468	WBGene00000209	asg-1	K07A12.3	-0.0213631370420195	

AGIWUR21398
CB4856	CT	I	8693451 8695159 WBGene00010623	K07A12.5	K07A12.5	0.0700966428484353
AGIWUR40
CB4856	CT	I	8695205 8696376 WBGene00003953	pbs-7	F39H11.5	-0.251001094714908
AGIWUR15172;AGIWUR32863;AGIWUR39094;AGIWUR40619
CB4856	CT	I	8696603 8697596 WBGene00009565	F39H11.1	F39H11.1	-0.330740540718583
AGIWUR20511
CB4856	CT	I	8697923 8701971 WBGene00006577	tlf-1	F39H11.2	0.033034551896943
AGIWUR18615;AGIWUR19525;AGIWUR27512;AGIWUR44922
CB4856	CT	I	8702478 8705825 WBGene00000409	cdk-8	F39H11.3	-0.00827984061129428
AGIWUR21102
CB4856	CT	I	8705670 8724279 WBGene00000527	cle-1	C36B1.1 0.0658351155399064
AGIWUR3067;AGIWUR23941;AGIWUR27821;AGIWUR32409;AGIWUR40629;AGIWUR41723
CB4856	CT	I	8727214 8728497 WBGene00003925	pas-4	C36B1.4 -0.25459211049996
AGIWUR1803;AGIWUR20887;AGIWUR43761
CB4856	CT	I	8728687 8730054 WBGene00007971	rpb-3	C36B1.3 -0.183703595569318	AGIWUR39563
CB4856	CT	I	8730156 8730922 WBGene00044614	C36B1.14	C36B1.14	-0.207008185445659
AGIWUR5597
CB4856	CT	I	8730905 8732125 WBGene00044120	C36B1.13	C36B1.13	0.781254642238961
AGIWUR3219;AGIWUR15030;AGIWUR36543
CB4856	CT	I	8732219 8733436 WBGene00007973	C36B1.6 C36B1.6 0.557418705625579
AGIWUR10616;AGIWUR14532;AGIWUR16176;AGIWUR16962;AGIWUR17067;AGIWUR30963
CB4856	CT	I	8733413 8735717 WBGene00007972	prp-4	C36B1.5 -0.0581617634600775	AGIWUR6599
CB4856	CT	I	8736213 8737038 WBGene00007974	dhfr-1	C36B1.7 -0.306904486612286
AGIWUR4679;AGIWUR12507;AGIWUR26096
CB4856	CT	I	8737072 8742548 WBGene00007975	gls-1	C36B1.8 -0.0639408743444037
AGIWUR14858;AGIWUR18949
CB4856	CT	I	8743849 8746925 WBGene00007976	C36B1.9 C36B1.9 -0.302246122830224	AGIWUR42991
CB4856	CT	I	8749616 8751397 WBGene00007977	gska-3	C36B1.10	0.151870468455648
AGIWUR27792
CB4856	CT	I	8752236 8755678 WBGene00007979	imp-1	C36B1.12	-0.203088454623206
AGIWUR17031
CB4856	CT	I	8755846 8757603 WBGene00007978	C36B1.11	C36B1.11	-0.355548047406881
AGIWUR2671
CB4856	CT	I	8758156 8759032 WBGene00008440	DY3.8	DY3.8	-0.316485305172186
AGIWUR28207;AGIWUR35048
CB4856	CT	I	8759204 8759788 WBGene00006574	tin-13	DY3.1	-0.256874310532966
AGIWUR18692;AGIWUR32809
CB4856	CT	I	8762986 8765633 WBGene00003052	lmn-1	DY3.2	0.0803291509144035

AGIWUR31393;AGIWUR32136;AGIWUR45124
CB4856	CT	I	8768325 8771248 WBGene00006618	trt-1	DY3.4	0.0812207524328059	AGIWUR33053
CB4856	CT	I	8771275 8777514 WBGene00004115	pqn-26	DY3.5	0.0109432977531485	AGIWUR42740
CB4856	CT	I	8779731 8785415 WBGene00008439	mfb-1	DY3.6	0.425085470520428
AGIWUR12938;AGIWUR30025
CB4856	CT	I	8792965 8797293 WBGene00006324	sup-17	DY3.7	0.053411746989962	AGIWUR24707
CB4856	CT	I	8798785 8801694 WBGene00009453	F36A2.3 F36A2.3 -0.181421790547578	AGIWUR5208
CB4856	CT	I	8801612 8804697 WBGene00009452	F36A2.2 F36A2.2 0.216806508229691	AGIWUR32436
CB4856	CT	I	8804799 8808081 WBGene00009451	cids-2	F36A2.1 0.0699203392923717	AGIWUR13864
CB4856	CT	I	8808775 8813298 WBGene00009455	phip-1	F36A2.8 -0.107447223026805
AGIWUR9233;AGIWUR16121;AGIWUR31991;AGIWUR34048;AGIWUR40925
CB4856	CT	I	8813761 8814979 WBGene00009454	F36A2.7 F36A2.7 0.0468323480849193
AGIWUR18419;AGIWUR27255
CB4856	CT	I	8815554 8816193 WBGene00004484	rps-15	F36A2.6 0.0177381565633681
AGIWUR10589;AGIWUR16337;AGIWUR20215;AGIWUR23222;AGIWUR31135;AGIWUR34061
CB4856	CT	I	8816978 8821359 WBGene00009456	F36A2.9 F36A2.9 -0.0529552484989502	AGIWUR32707
CB4856	CT	I	8822188 8823637 WBGene00009458	F36A2.11	F36A2.11	0.0196139534419491
AGIWUR14729
CB4856	CT	I	8824401 8825628 WBGene00023490	F36A2.14	F36A2.14	0.126026319892762
AGIWUR38352
CB4856	CT	I	8826167 8826670 WBGene00009459	F36A2.12	F36A2.12	-0.0914143533906424
AGIWUR3383;AGIWUR3491;AGIWUR20812;AGIWUR24892
CB4856	CT	I	8828048 8838719 WBGene00009460	ubr-5	F36A2.13	-0.0987740119070217
AGIWUR2272;AGIWUR9912;AGIWUR27719;AGIWUR32971;AGIWUR34063;AGIWUR44432
CB4856	CT	I	8840634 8841163 WBGene00009250	F29D10.1	F29D10.1	0.399477338566692
AGIWUR6675
CB4856	CT	I	8841770 8842323 WBGene00009251	F29D10.2	F29D10.2	-0.953553235751436
AGIWUR35680
CB4856	CT	I	8842552 8845595 WBGene00009252	F29D10.3	F29D10.3	-0.348224594247962
AGIWUR40719
CB4856	CT	I	8845809 8851116 WBGene00002035	hum-1	F29D10.4	-0.0977109664992106
AGIWUR26300
CB4856	CT	I	8853686 8861274 WBGene00010137	ztf-26	F55H12.6	0.104294067556843
AGIWUR10402;AGIWUR26678;AGIWUR32959;AGIWUR43440
CB4856	CT	I	8863996 8865475 WBGene00219283	F55H12.7	F55H12.7	1.85848472382125
AGIWUR10168;AGIWUR13074;AGIWUR22738;AGIWUR23038;AGIWUR37370
CB4856	CT	I	8866907 8868293 WBGene00010133	F55H12.2	F55H12.2	0.787948645571248
AGIWUR12233
CB4856	CT	I	8869709 8871698 WBGene00010136	F55H12.5	F55H12.5	-0.0072410169760424

AGIWUR5794
CB4856 CT I 8871791 8875253 WBGene00004901 snf-2 F55H12.1 -0.00510249860486045
AGIWUR9487;AGIWUR19311
CB4856 CT I 8876319 8893051 WBGene00010134 F55H12.3 F55H12.3 0.00766889907764678
AGIWUR9070
CB4856 CT I 8893308 8894137 WBGene00010135 F55H12.4 F55H12.4 -0.43411200920562
AGIWUR3304;AGIWUR5417;AGIWUR36249
CB4856 CT I 8897613 8901435 WBGene00006765 unc-29 T08G11.5 -0.236376861423974
AGIWUR16459
CB4856 CT I 8902747 8905534 WBGene00011631 T08G11.4 T08G11.4 -0.28371734588454
AGIWUR14591
CB4856 CT I 8905644 8916805 WBGene00011629 T08G11.1 T08G11.1 0.054609983693533
AGIWUR1995;AGIWUR15550;AGIWUR25916
CB4856 CT I 8919329 8920516 WBGene00044684 T08G11.2 T08G11.2 0.267359789368075
AGIWUR9790;AGIWUR15705
CB4856 CT I 8921190 8925327 WBGene00011630 T08G11.3 T08G11.3 0.0736688569289567
AGIWUR19825
CB4856 CT I 8928748 8931089 WBGene00006641 tsp-15 F53B6.1 -0.0565215839455373 AGIWUR2572
CB4856 CT I 8931035 8942287 WBGene00009958 madd-4 F53B6.2 -0.01302444728932
AGIWUR22167;AGIWUR26682;AGIWUR29905;AGIWUR37895;AGIWUR41106
CB4856 CT I 8945533 8946205 WBGene00009964 fip-6 F53B6.9 -0.133762858844385
AGIWUR7478;AGIWUR11131;AGIWUR11435;AGIWUR20023;AGIWUR25202;AGIWUR43705
CB4856 CT I 8946686 8947201 WBGene00009963 fipr-26 F53B6.8 0.774474665872372 AGIWUR33720
CB4856 CT I 8947304 8948711 WBGene00009959 F53B6.4 F53B6.4 0.124742909797006
AGIWUR31357;AGIWUR34697
CB4856 CT I 8948778 8954394 WBGene00009962 F53B6.7 F53B6.7 0.0170964648699687 AGIWUR3335
CB4856 CT I 8954559 8957482 WBGene00009961 cutl-7 F53B6.6 0.33451019881037 AGIWUR18610
CB4856 CT I 8958061 8960889 WBGene00043049 F32H2.11 F32H2.11 0.202939260672963
AGIWUR30582
CB4856 CT I 8962322 8966129 WBGene00001568 snpc-4 F32H2.1 -0.0630053589919477 AGIWUR16674
CB4856 CT I 8966130 8967004 WBGene00007026 mdt-31 F32H2.2 -0.0221998724597618 AGIWUR34254
CB4856 CT I 8966985 8970158 WBGene00004953 spd-2 F32H2.3 -0.0487311911110009 AGIWUR12491
CB4856 CT I 8970293 8971031 WBGene00009346 F32H2.10 F32H2.10 -0.250062902126182
AGIWUR31683
CB4856 CT I 8971139 8973063 WBGene00009341 thoc-3 F32H2.4 0.0472251237481309 AGIWUR33313
CB4856 CT I 8973066 8974575 WBGene00050880 F32H2.12 F32H2.12 0.176915270686502
AGIWUR1994;AGIWUR33278
CB4856 CT I 8975384 8986534 WBGene00009342 fasn-1 F32H2.5 0.160626991955588
AGIWUR11210;AGIWUR43314

CB4856	CT	I	8989938	8993461	WBGene00009345	F32H2.8	F32H2.8	0.151511232719047	AGIWUR40399
CB4856	CT	I	8994002	8998593	WBGene00006532	tba-6	F32H2.9	0.360873578660775
AGIWUR6102;AGIWUR8788;AGIWUR19549
CB4856	CT	I	8998532	8999564	WBGene00009343	F32H2.6	F32H2.6	0.422823416468949	AGIWUR6481
CB4856	CT	I	9000097	9001721	WBGene00009480	F36F2.7	F36F2.7	0.183711834941146	AGIWUR19884
CB4856	CT	I	9001913	9003963	WBGene00009478	syx-7	F36F2.4	-0.221646764695713	AGIWUR4349
CB4856	CT	I	9004513	9008708	WBGene00009479	fcp-1	F36F2.6	-0.0401680138632416
AGIWUR1634;AGIWUR1989;AGIWUR12067;AGIWUR12943;AGIWUR19990;AGIWUR23535;AGIWUR23931;AGIWUR33444;AGIWUR33850;A
GIWUR35249;AGIWUR37967;AGIWUR38798
CB4856	CT	I	9009260	9013885	WBGene00009477	rbpl-1	F36F2.3	0.129623345270158
AGIWUR8448;AGIWUR9874;AGIWUR11081;AGIWUR20523;AGIWUR42216;AGIWUR44180
CB4856	CT	I	9015357	9015711	WBGene00009481	F36F2.8	F36F2.8	0.249151875613851	AGIWUR18272
CB4856	CT	I	9016755	9017994	WBGene00009476	F36F2.2	F36F2.2	0.988550614786643	AGIWUR2277
CB4856	CT	I	9018623	9020304	WBGene00009475	F36F2.1	F36F2.1	0.048302241147633	AGIWUR26314
CB4856	CT	I	9020945	9025553	WBGene00006525	tax-2	F36F2.5	0.147030015232213	AGIWUR29674
CB4856	CT	I	9025779	9031232	WBGene00007854	C31H5.1	C31H5.1	0.251029679961888	AGIWUR2925
CB4856	CT	I	9031274	9036695	WBGene00000058	acr-19	C31H5.3	0.2429001462858	AGIWUR7763
CB4856	CT	I	9041374	9041988	WBGene00007858	C31H5.7	C31H5.7	0.39673332661838	AGIWUR33167
CB4856	CT	I	9042270	9042599	WBGene00007855	C31H5.4	C31H5.4	0.403326713474508	AGIWUR26962
CB4856	CT	I	9043315	9046889	WBGene00007857	C31H5.6	C31H5.6	-0.202693402993503	AGIWUR44224
CB4856	CT	I	9048188	9049427	WBGene00007856	C31H5.5	C31H5.5	-0.0972626512206638	AGIWUR15377
CB4856	CT	I	9049460	9055457	WBGene00001843	hgo-1	W06D4.1	-0.0836491691094654	AGIWUR36152
CB4856	CT	I	9056293	9057822	WBGene00012296	spe-46	W06D4.2	-0.0734807166894207
AGIWUR906;AGIWUR18763;AGIWUR24886
CB4856	CT	I	9058852	9060473	WBGene00012297	W06D4.3	W06D4.3	-0.0959652004350744
AGIWUR9186;AGIWUR28315;AGIWUR40836
CB4856	CT	I	9060493	9063160	WBGene00012298	prmt-7	W06D4.4	-0.235954811168613	AGIWUR3456
CB4856	CT	I	9063588	9064403	WBGene00006503	snx-3	W06D4.5	-0.294334345390681	AGIWUR28774
CB4856	CT	I	9065087	9070264	WBGene00004298	rad-54	W06D4.6	-0.151704273563823
AGIWUR159;AGIWUR21282;AGIWUR26162
CB4856	CT	I	9070247	9075077	WBGene00011531	rsbp-1	T06D10.1	0.0271814336491243
AGIWUR241;AGIWUR1536;AGIWUR4469;AGIWUR5365;AGIWUR12649;AGIWUR14014;AGIWUR18637;AGIWUR23781;AGIWUR24397;AGIW
UR26444;AGIWUR26777;AGIWUR40003;AGIWUR43991
CB4856	CT	I	9077565	9080024	WBGene00011532	chaf-1	T06D10.2	-0.00549182792986613
AGIWUR26057
CB4856	CT	I	9095957	9100862	WBGene00003182	mef-2	W10D5.1	0.0217422463119745
AGIWUR1934;AGIWUR2035;AGIWUR36060;AGIWUR40628
CB4856	CT	I	9100846	9107939	WBGene00001574	gei-17	W10D5.3	0.0822847662716271
AGIWUR1675;AGIWUR2117;AGIWUR7167;AGIWUR7510;AGIWUR9339;AGIWUR11898;AGIWUR12040;AGIWUR15245;AGIWUR21852;AGIW

UR23780;AGIWUR23859;AGIWUR28852;AGIWUR32519;AGIWUR36685;AGIWUR37731;AGIWUR37792;AGIWUR37857;AGIWUR39942;AGIWUR42717
CB4856 CT I 9108173 9110918 WBGene00012376 nduf-7 W10D5.2 0.143286127290358 AGIWUR9870;AGIWUR19362;AGIWUR25841;AGIWUR39958;AGIWUR42432
CB4856 CT I 9118115 9122398 WBGene00014115 gld-4 ZK858.1 -0.211222811288394 AGIWUR8183;AGIWUR20297;AGIWUR27306
CB4856 CT I 9123182 9124509 WBGene00014116 ZK858.2 ZK858.2 0.333060099514507 AGIWUR39603
CB4856 CT I 9124664 9126155 WBGene00014121 ZK858.8 ZK858.8 0.0737233659699488 AGIWUR34245
CB4856 CT I 9126140 9127101 WBGene00014117 clec-91 ZK858.3 -0.15077893786502 AGIWUR32994
CB4856 CT I 9132538 9135753 WBGene00003209 mel-26 ZK858.4 -0.0262429732905467 AGIWUR17332
CB4856 CT I 9135764 9138361 WBGene00014118 ZK858.5 ZK858.5 -0.0463260633097153 AGIWUR21277
CB4856 CT I 9138267 9145632 WBGene00014119 ZK858.6 ZK858.6 -0.0505086479976953 AGIWUR1164
CB4856 CT I 9149474 9153252 WBGene00010421 slc-36.4 H32K16.1 0.196551129325109 AGIWUR12001;AGIWUR14839;AGIWUR39085
CB4856 CT I 9154228 9154789 WBGene00010422 H32K16.2 H32K16.2 1.37388376000834 AGIWUR19106
CB4856 CT I 9155513 9157411 WBGene00009129 F25H5.7 F25H5.7 -0.00248958055054338 AGIWUR22773
CB4856 CT I 9159015 9159757 WBGene00009128 mrpl-54 F25H5.6 -0.210662430143419 AGIWUR41190
CB4856 CT I 9159938 9163261 WBGene00009127 clsp-1 F25H5.5 0.00952020773376461 AGIWUR34600
CB4856 CT I 9163531 9166952 WBGene00001167 eef-2 F25H5.4 -0.00288647630152283 AGIWUR2082;AGIWUR2145;AGIWUR3539;AGIWUR3615;AGIWUR6960;AGIWUR18490;AGIWUR19460;AGIWUR20868;AGIWUR29742;AGIWUR34740;AGIWUR37900;AGIWUR42189;AGIWUR44830
CB4856 CT I 9168432 9177488 WBGene00009126 pyk-1 F25H5.3 -0.177916809341336 AGIWUR13861;AGIWUR19221;AGIWUR20544;AGIWUR20814
CB4856 CT I 9177664 9178703 WBGene00009130 F25H5.8 F25H5.8 -0.108677625676817 AGIWUR17782
CB4856 CT I 9179941 9181119 WBGene00009125 F25H5.2 F25H5.2 -0.233415146938457 AGIWUR3832
CB4856 CT I 9181819 9220766 WBGene00006407 lim-9 F25H5.1 -0.0416838486892601 AGIWUR4251;AGIWUR5434;AGIWUR8051;AGIWUR12308;AGIWUR12581;AGIWUR14843;AGIWUR27883;AGIWUR28401;AGIWUR34213;AGIWUR37608;AGIWUR40066
CB4856 CT I 9221834 9226390 WBGene00008107 aspm-1 C45G3.1 0.110170768646249 AGIWUR10033;AGIWUR19229;AGIWUR22943;AGIWUR26742;AGIWUR35966;AGIWUR38022
CB4856 CT I 9226905 9228184 WBGene00001590 C45G3.3 C45G3.3 -0.306668521256657 AGIWUR3937;AGIWUR4120;AGIWUR8367;AGIWUR41921
CB4856 CT I 9228163 9232097 WBGene00044008 gip-2 C45G3.5 -0.0263898458782563 AGIWUR39028
CB4856 CT I 9232071 9233560 WBGene00000147 aph-1 VF36H2L.1 0.010597393566961 AGIWUR20506
CB4856 CT I 9234174 9245924 WBGene00009498 tat-5 F36H2.1 0.0799279759536766 AGIWUR33349
CB4856 CT I 9246028 9249383 WBGene00009499 ent-6 F36H2.2 0.0759095493726551 AGIWUR33843
CB4856 CT I 9249542 9250488 WBGene00044247 F36H2.4 F36H2.4 -0.121263563396835

AGIWUR1039;AGIWUR13806;AGIWUR14703;AGIWUR14903
CB4856 CT I 9251838 9261657 WBGene00009500 F36H2.3 F36H2.3 0.0457246255748811
AGIWUR5375;AGIWUR18730
CB4856 CT I 9265951 9270919 WBGene00008779 F14B4.1 F14B4.1 0.91941161747154 AGIWUR35273
CB4856 CT I 9271443 9278689 WBGene00008780 hxk-1 F14B4.2 -0.0544631865722955
AGIWUR9996;AGIWUR14587;AGIWUR24932
CB4856 CT I 9280963 9286577 WBGene00008781 rpoa-2 F14B4.3 -0.212329131872478 AGIWUR24437
CB4856 CT I 9287437 9289770 WBGene00009173 prom-1 F26H9.1 -0.231079333605553 AGIWUR21546
CB4856 CT I 9289753 9294035 WBGene00009174 F26H9.2 F26H9.2 -0.103758565902561
AGIWUR18009;AGIWUR21415;AGIWUR26246
CB4856 CT I 9305751 9306844 WBGene00009176 nmat-2 F26H9.4 -0.36873114190853 AGIWUR38957
CB4856 CT I 9308442 9310221 WBGene00004268 rab-5 F26H9.6 0.00526278216070608
AGIWUR6586;AGIWUR20214;AGIWUR26895;AGIWUR43354
CB4856 CT I 9310329 9312641 WBGene00006732 uev-3 F26H9.7 -0.0476142288982189 AGIWUR29491
CB4856 CT I 9312635 9314488 WBGene00009177 F26H9.5 F26H9.5 -0.0174598312154366 AGIWUR38121
CB4856 CT I 9317736 9327279 WBGene00009178 uggt-2 F26H9.8 0.0162542221094188 AGIWUR43492
CB4856 CT I 9327857 9330928 WBGene00007533 cbl-1 C12C8.2 -0.356701640136316
AGIWUR4013;AGIWUR4482;AGIWUR30682;AGIWUR40616
CB4856 CT I 9334812 9342496 WBGene00003026 lin-41 C12C8.3 -0.120249369466353
AGIWUR30548;AGIWUR38608
CB4856 CT I 9344724 9349558 WBGene00010857 M04C9.2 M04C9.2 0.34649431871671
AGIWUR9274;AGIWUR27747;AGIWUR37255
CB4856 CT I 9350274 9352700 WBGene00010856 M04C9.1 M04C9.1 -0.249982635734984
AGIWUR13465;AGIWUR15038;AGIWUR30895;AGIWUR32551;AGIWUR38207
CB4856 CT I 9352882 9354541 WBGene00010858 M04C9.3 M04C9.3 -0.105125522155986
AGIWUR17011;AGIWUR34564;AGIWUR40124
CB4856 CT I 9355072 9357079 WBGene00010859 M04C9.4 M04C9.4 -0.330874073037931 AGIWUR12795
CB4856 CT I 9359075 9364180 WBGene00001121 dyf-5 M04C9.5 0.379216642056446 AGIWUR3172
CB4856 CT I 9364099 9389982 WBGene00008878 ppfr-1 F16A11.3 -0.136598613737531
AGIWUR6600;AGIWUR24113;AGIWUR33603
CB4856 CT I 9390829 9393095 WBGene00008877 rtcb-1 F16A11.2 -0.153100199467001
AGIWUR39556
CB4856 CT I 9393948 9404024 WBGene00008876 F16A11.1 F16A11.1 0.0682587781053652
AGIWUR4470
CB4856 CT I 9408356 9410094 WBGene00007641 C17E4.1 C17E4.1 0.194462021567533 AGIWUR21934
CB4856 CT I 9414456 9416703 WBGene00219313 C17E4.20 C17E4.20 0.197766112111999
AGIWUR33741
CB4856 CT I 9416704 9418884 WBGene00007643 marc-3 C17E4.3 -0.0376825096851654
AGIWUR7149;AGIWUR44386

CB4856	CT	I	9419368	9419998	WBGene00007644	C17E4.4	C17E4.4	0.0692264776287814	AGIWUR38083
CB4856	CT	I	9419973	9420813	WBGene00044236	C17E4.11	C17E4.11	0.0627125394963333	AGIWUR27536;AGIWUR36882
CB4856	CT	I	9420907	9421992	WBGene00003904	pabp-2	C17E4.5	-0.0222635702097511	AGIWUR3676;AGIWUR21561;AGIWUR38882;AGIWUR41249
CB4856	CT	I	9422556	9431120	WBGene00007645	C17E4.6	C17E4.6	-0.0540569860254809	AGIWUR13805;AGIWUR17385;AGIWUR29208
CB4856	CT	I	9431098	9433828	WBGene00007646	nkb-1	C17E4.9	-0.215737535840005	AGIWUR8987;AGIWUR12137;AGIWUR17620;AGIWUR22816;AGIWUR23728;AGIWUR43795
CB4856	CT	I	9435377	9441029	WBGene00007647	C17E4.10	C17E4.10	0.0166709013270481	AGIWUR23779;AGIWUR25582;AGIWUR42996
CB4856	CT	I	9441010	9450731	WBGene00003172	mec-8	F46A9.6	0.222460527182031	AGIWUR10739;AGIWUR11464;AGIWUR28789;AGIWUR38678;AGIWUR43431;AGIWUR44090
CB4856	CT	I	9457456	9458842	WBGene00009754	F46A9.1	F46A9.1	0.766239011377678	AGIWUR34224
CB4856	CT	I	9459287	9460550	WBGene00009755	F46A9.2	F46A9.2	0.153374313099773	AGIWUR16726
CB4856	CT	I	9463760	9468917	WBGene00006681	twk-29	F46A9.3	-0.286328147422081	AGIWUR11445
CB4856	CT	I	9469704	9470633	WBGene00004808	skr-2	F46A9.4	-0.258627858486431	AGIWUR3845
CB4856	CT	I	9471341	9472642	WBGene00004807	skr-1	F46A9.5	0.309476534249802	AGIWUR10859;AGIWUR17688;AGIWUR21590;AGIWUR24111;AGIWUR37526
CB4856	CT	I	9472642	9474794	WBGene00009260	calm-1	F30A10.1	-0.214342078161993	AGIWUR3316
CB4856	CT	I	9476597	9477181	WBGene00045473	F30A10.14	F30A10.14	0.151425223332881	AGIWUR13773
CB4856	CT	I	9477478	9478098	WBGene00009268	F30A10.11	F30A10.11	0.349637533218017	AGIWUR10060
CB4856	CT	I	9478470	9479091	WBGene00044680	F30A10.13	F30A10.13	-0.307481135582355	AGIWUR16460;AGIWUR40269
CB4856	CT	I	9479101	9480774	WBGene00009261	F30A10.2	F30A10.2	0.025248354906152	AGIWUR14176;AGIWUR14360;AGIWUR22666
CB4856	CT	I	9481629	9482275	WBGene00009269	F30A10.12	F30A10.12	0.0614242145017347	AGIWUR5625;AGIWUR19501
CB4856	CT	I	9483810	9486540	WBGene00009263	F30A10.4	F30A10.4	-0.303335917552674	AGIWUR16094
CB4856	CT	I	9486642	9488229	WBGene00006061	stl-1	F30A10.5	0.166198856971683	AGIWUR12020
CB4856	CT	I	9488488	9492620	WBGene00009264	sac-1	F30A10.6	-0.168482017277038	AGIWUR8404;AGIWUR15613;AGIWUR21401
CB4856	CT	I	9492615	9494589	WBGene00009262	F30A10.3	F30A10.3	0.0330086054787362	AGIWUR7533;AGIWUR30325;AGIWUR31913

CB4856	CT	I	9501268	9505173	WBGene00006062	stn-1	F30A10.8	0.0795217657343104	AGIWUR11443;AGIWUR37826
CB4856	CT	I	9506553	9511184	WBGene00009267	usp-48	F30A10.10	0.0345992818428158	AGIWUR41266
CB4856	CT	I	9511181	9512236	WBGene00004887	smn-1	C41G7.1	-0.124994410844778	AGIWUR40754
CB4856	CT	I	9512428	9514755	WBGene00002226	klp-16	C41G7.2	-0.117365014272212	AGIWUR16204
CB4856	CT	I	9515002	9516776	WBGene00077771	C41G7.9	C41G7.9	0.0501676756149898	AGIWUR9965;AGIWUR12718;AGIWUR34772
CB4856	CT	I	9519244	9521275	WBGene00008062	set-32	C41G7.4	-0.222587994504581	AGIWUR11730;AGIWUR16788;AGIWUR18476
CB4856	CT	I	9530816	9532831	WBGene00077525	C41G7.8	C41G7.8	-0.301117360677342	AGIWUR15227;AGIWUR15913;AGIWUR20146;AGIWUR21311
CB4856	CT	I	9533580	9536271	WBGene00008064	C41G7.7	C41G7.7	-0.0656277902839042	AGIWUR40452
CB4856	CT	I	9536465	9540966	WBGene00007984	irx-1	C36F7.1	0.437650287361016	AGIWUR3595;AGIWUR4726;AGIWUR12384;AGIWUR15187;AGIWUR40383
CB4856	CT	I	9544733	9558096	WBGene00007985	swah-1	C36F7.2	-0.3543236138057	AGIWUR4941;AGIWUR23912;AGIWUR33178
CB4856	CT	I	9565394	9574377	WBGene00004372	rig-5	C36F7.4	0.341537084197839	AGIWUR610;AGIWUR8403;AGIWUR17086;AGIWUR19523;AGIWUR44163
CB4856	CT	I	9574762	9576141	WBGene00007986	C36F7.5	C36F7.5	-0.274363973418525	AGIWUR26301
CB4856	CT	I	9578526	9581267	WBGene00002276	lem-3	F42H11.2	-0.152461995430599	AGIWUR4832
CB4856	CT	I	9581786	9582583	WBGene00044987	K07A1.17	K07A1.17	0.157237317190482	AGIWUR11068
CB4856	CT	I	9582682	9583533	WBGene00010608	K07A1.1	K07A1.1	-0.122788047510056	AGIWUR3309;AGIWUR7293;AGIWUR13669;AGIWUR14108;AGIWUR25109;AGIWUR29244;AGIWUR34922;AGIWUR40044
CB4856	CT	I	9583869	9584817	WBGene00010617	K07A1.13	K07A1.13	0.0435210460827964	AGIWUR31686
CB4856	CT	I	9585317	9587206	WBGene00010609	dut-1	K07A1.2	-0.0944253372272694	AGIWUR17209;AGIWUR24219;AGIWUR34406
CB4856	CT	I	9587169	9588132	WBGene00010610	K07A1.3	K07A1.3	0.341252561921245	AGIWUR10401;AGIWUR13401;AGIWUR21452
CB4856	CT	I	9588710	9589931	WBGene00010611	K07A1.4	K07A1.4	0.0412345705105714	AGIWUR21507
CB4856	CT	I	9591939	9593103	WBGene00010613	K07A1.6	K07A1.6	0.138599000786328	AGIWUR12219;AGIWUR17738;AGIWUR20345;AGIWUR27107;AGIWUR29821
CB4856	CT	I	9595276	9598363	WBGene00010614	cri-1	K07A1.7	0.145141968379987	AGIWUR6642;AGIWUR9009;AGIWUR42113
CB4856	CT	I	9605896	9609025	WBGene00000177	aqp-9	K07A1.16	-0.274300587820919	AGIWUR14146;AGIWUR43748

CB4856 CT I 9608926 9609705 WBGene00010619 K07A1.15 K07A1.15 -0.201924921335342
AGIWUR34358
CB4856 CT I 9609501 9611620 WBGene00002070 ile-1 K07A1.8 -0.0815512577043656
AGIWUR11387;AGIWUR26158;AGIWUR44299;AGIWUR45019
CB4856 CT I 9611815 9615082 WBGene00010615 ate-1 K07A1.9 -0.08614803091419
AGIWUR5578;AGIWUR19246
CB4856 CT I 9615195 9616474 WBGene00010616 K07A1.10 K07A1.10 -0.290449295669784
AGIWUR8008;AGIWUR36377;AGIWUR40995;AGIWUR43062
CB4856 CT I 9616508 9618107 WBGene00004312 rba-1 K07A1.11 -0.174090167992404
AGIWUR21971
CB4856 CT I 9618207 9619760 WBGene00003036 lin-53 K07A1.12 -0.0168681165027573
AGIWUR784;AGIWUR14734;AGIWUR15058;AGIWUR17077;AGIWUR22021;AGIWUR28468
CB4856 CT I 9621160 9625796 WBGene00004488 rps-19 T05F1.3 -0.0277690261426127
AGIWUR1014;AGIWUR3605;AGIWUR6371;AGIWUR9406;AGIWUR15578;AGIWUR17883;AGIWUR19787;AGIWUR21395;AGIWUR23441;AGI
WUR32203;AGIWUR35058;AGIWUR41713
CB4856 CT I 9626241 9630848 WBGene00002027 hsr-9 T05F1.6 0.146857705881207 AGIWUR37000
CB4856 CT I 9631388 9632402 WBGene00011490 T05F1.4 T05F1.4 0.0298692880138525 AGIWUR14722
CB4856 CT I 9633183 9635945 WBGene00011491 T05F1.5 T05F1.5 -0.356102185242026 AGIWUR9793
CB4856 CT I 9638886 9641736 WBGene00011492 T05F1.7 T05F1.7 -0.378593992432155 AGIWUR266
CB4856 CT I 9642344 9643804 WBGene00011493 T05F1.8 T05F1.8 -0.0362154274944935 AGIWUR38596
CB4856 CT I 9645699 9647676 WBGene00011494 T05F1.9 T05F1.9 0.740142101887521 AGIWUR9312
CB4856 CT I 9647603 9649238 WBGene00000968 dhs-4 T05F1.10 0.101798056405489
AGIWUR27482
CB4856 CT I 9650499 9652946 WBGene00011497 T05F1.13 T05F1.13 -0.199377385580773
AGIWUR14401
CB4856 CT I 9653021 9657040 WBGene00011495 T05F1.11 T05F1.11 1.15571436187755
AGIWUR25086
CB4856 CT I 9657012 9657871 WBGene00007278 lab-1 C03D6.6 0.255389765530609 AGIWUR9702
CB4856 CT I 9658274 9660080 WBGene00007277 asfl-1 C03D6.5 -0.0809808097846911 AGIWUR14465
CB4856 CT I 9660174 9664915 WBGene00003800 npp-14 C03D6.4 -0.112027115558062 AGIWUR18409
CB4856 CT I 9665194 9668664 WBGene00000466 cel-1 C03D6.3 -0.139043507579758
AGIWUR2773;AGIWUR8832;AGIWUR16240;AGIWUR35365
CB4856 CT I 9668628 9670726 WBGene00007275 C03D6.1 C03D6.1 -0.229337164483789 AGIWUR13138
CB4856 CT I 9673668 9678553 WBGene00007297 C04F12.1 C04F12.1 0.114905288790343
AGIWUR33114
CB4856 CT I 9685398 9688653 WBGene00002069 ikb-1 C04F12.3 0.759456601008498
AGIWUR2266
CB4856 CT I 9689185 9690084 WBGene00004426 rpl-14 C04F12.4 0.0397731336516358
AGIWUR38081

CB4856	CT	I	9690079	9690557	WBGene00007299	C04F12.5	C04F12.5	-0.0262737687221741	AGIWUR2681;AGIWUR6569;AGIWUR15195;AGIWUR17394;AGIWUR19832;AGIWUR21206
CB4856	CT	I	9692701	9693781	WBGene00007300	C04F12.6	C04F12.6	0.0715916119819209	AGIWUR7919
CB4856	CT	I	9695707	9696762	WBGene00007301	C04F12.7	C04F12.7	0.262720523792527	AGIWUR8242
CB4856	CT	I	9697237	9699561	WBGene00007302	C04F12.8	C04F12.8	-0.0318894188809367	AGIWUR19847
CB4856	CT	I	9710093	9710767	WBGene00007303	rnh-1.3	C04F12.9	-0.408500042362144	AGIWUR2458
CB4856	CT	I	9711064	9712719	WBGene00001405	fce-1	C04F12.10	-0.0913639852611521	AGIWUR36986
CB4856	CT	I	9712825	9715230	WBGene00007621	C16C2.4	C16C2.4	0.0701345669478108	AGIWUR25185
CB4856	CT	I	9715321	9728108	WBGene00007620	ocrl-1	C16C2.3	-0.182862218025507	AGIWUR34995
CB4856	CT	I	9737823	9744190	WBGene00001582	gfi-2	K02A11.1	0.0711070840146979	AGIWUR27008;AGIWUR33816
CB4856	CT	I	9744242	9745990	WBGene00010487	K02A11.2	K02A11.2	-0.182129334086512	AGIWUR32384
CB4856	CT	I	9745967	9747923	WBGene00010488	endu-1	K02A11.3	0.371309257732676	AGIWUR26684
CB4856	CT	I	9748517	9749953	WBGene00044607	K02A11.4	K02A11.4	0.334253405539538	AGIWUR1620;AGIWUR25104;AGIWUR38391;AGIWUR44265;AGIWUR45015
CB4856	CT	I	9754122	9757898	WBGene00006352	sur-6	F26E4.1	0.14343879840849	AGIWUR2096;AGIWUR2215;AGIWUR9115;AGIWUR12953;AGIWUR13411;AGIWUR24929
CB4856	CT	I	9757854	9760787	WBGene00009157	F26E4.2	F26E4.2	-0.223883894137108	AGIWUR8064
CB4856	CT	I	9762403	9767873	WBGene00009158	F26E4.3	F26E4.3	-0.46404787941683	AGIWUR4512;AGIWUR27007
CB4856	CT	I	9768836	9771068	WBGene00009160	F26E4.5	F26E4.5	-0.0882562678202099	AGIWUR23988
CB4856	CT	I	9771061	9772565	WBGene00009159	F26E4.4	F26E4.4	0.0442985308463264	AGIWUR27196
CB4856	CT	I	9773172	9774012	WBGene00009161	F26E4.6	F26E4.6	-0.359888969511672	AGIWUR5123
CB4856	CT	I	9774165	9779541	WBGene00009162	F26E4.7	F26E4.7	0.245609236384327	AGIWUR18395
CB4856	CT	I	9780287	9781418	WBGene00009165	gpx-1	F26E4.12	-0.158341420825202	AGIWUR41088
CB4856	CT	I	9781338	9785722	WBGene00009163	drsh-1	F26E4.10	-0.0560239658304411	AGIWUR9346;AGIWUR17525
CB4856	CT	I	9785778	9787624	WBGene00006528	tba-1	F26E4.8	-0.0839986439858826	AGIWUR26699;AGIWUR27386;AGIWUR38577
CB4856	CT	I	9788749	9790050	WBGene00000371	cco-1	F26E4.9	-0.0918391797368159	AGIWUR2388;AGIWUR7324;AGIWUR10896;AGIWUR25457;AGIWUR26663;AGIWUR29113;AGIWUR38370

CB4856 CT I 9793756 9796675 WBGene00009164 hrdl-1 F26E4.11 0.130569589713009
AGIWUR477;AGIWUR5508;AGIWUR9022
CB4856 CT I 9808904 9813075 WBGene00003569 ncx-4 F35C12.2 0.0911184381053345
AGIWUR18843;AGIWUR26111;AGIWUR27942;AGIWUR30673
CB4856 CT I 9813034 9823448 WBGene00009408 F35C12.3 F35C12.3 -0.557695426306639
AGIWUR41111
CB4856 CT I 9829827 9830599 WBGene00013449 Y67A6A.1 Y67A6A.1 -0.173648237783755
AGIWUR44360
CB4856 CT I 9839598 9844809 WBGene00003652 nhr-62 Y67A6A.2 -0.147915807837979
AGIWUR2485
CB4856 CT I 9847406 9850082 WBGene00006978 zig-1 K10C3.3 -0.124443511433654
AGIWUR21169;AGIWUR39109;AGIWUR39381
CB4856 CT I 9850020 9851791 WBGene00010730 ensa-1 K10C3.2 0.0229780010116496
AGIWUR18495;AGIWUR30294;AGIWUR30891;AGIWUR35413;AGIWUR35421;AGIWUR41141
CB4856 CT I 9853658 9863492 WBGene00010731 K10C3.4 K10C3.4 -0.117647797883855
AGIWUR1891;AGIWUR30585
CB4856 CT I 9863588 9869055 WBGene00010732 K10C3.5 K10C3.5 0.014057689417637 AGIWUR42407
CB4856 CT I 9869632 9874676 WBGene00003639 nhr-49 K10C3.6 0.156670369552392
AGIWUR7928;AGIWUR12085;AGIWUR13755;AGIWUR22758
CB4856 CT I 9874698 9879119 WBGene00002142 inx-20 T23H4.1 0.336721773719441 AGIWUR21242
CB4856 CT I 9879183 9881504 WBGene00003659 nhr-69 T23H4.2 0.3721284706576
AGIWUR5029;AGIWUR19276;AGIWUR24449
CB4856 CT I 9886200 9888154 WBGene00003524 nas-5 T23H4.3 -0.0283767815126978 AGIWUR21924
CB4856 CT I 9888356 9892223 WBGene00003842 oct-1 F52F12.1 -0.0876701600423804
AGIWUR6287;AGIWUR17666;AGIWUR21319
CB4856 CT I 9893698 9895596 WBGene00000640 col-64 F52F12.2 0.156428306681414
AGIWUR24554
CB4856 CT I 9895693 9900817 WBGene00003396 mom-4 F52F12.3 0.0799387858096781
AGIWUR385;AGIWUR4265;AGIWUR14861;AGIWUR22928;AGIWUR26295;AGIWUR27202;AGIWUR30831;AGIWUR34120
CB4856 CT I 9901255 9902929 WBGene00009937 lsl-1 F52F12.4 -0.132516018709151
AGIWUR13054
CB4856 CT I 9902943 9910545 WBGene00009939 ztf-11 F52F12.6 0.298044669356674
AGIWUR22662;AGIWUR22912
CB4856 CT I 9921211 9921961 WBGene00009938 F52F12.5 F52F12.5 -0.0828497322027673
AGIWUR14086
CB4856 CT I 9923610 9924280 WBGene00009941 F52F12.8 F52F12.8 0.184511533549353
AGIWUR14551
CB4856 CT I 9925978 9929032 WBGene00009942 F52F12.9 F52F12.9 -0.0230834136279974
AGIWUR9358;AGIWUR35586

CB4856 CT I 9928946 9930885 WBGene00009940 strl-1 F52F12.7 -0.0773397124837238
AGIWUR24560
CB4856 CT I 9930972 9931882 WBGene00013696 Y106G6A.4 Y106G6A.4 0.114967689866532
AGIWUR2031
CB4856 CT I 9931870 9934290 WBGene00013695 epg-8 Y106G6A.2 0.0278599173875094
AGIWUR2744;AGIWUR18245;AGIWUR18984;AGIWUR28290;AGIWUR31270;AGIWUR32991;AGIWUR41204;AGIWUR43220;AGIWUR43432
CB4856 CT I 9934639 9936022 WBGene00013697 dsbn-1 Y106G6A.5 -0.147894144064413
AGIWUR37460
CB4856 CT I 9936125 9938378 WBGene00013694 Y106G6A.1 Y106G6A.1 0.0161281023768467
AGIWUR3372;AGIWUR9642;AGIWUR28741
CB4856 CT I 9947805 9953387 WBGene00005023 sqv-5 T24D1.1 0.222875730223879
AGIWUR9388;AGIWUR42746
CB4856 CT I 9953409 9955873 WBGene00011985 T24D1.2 T24D1.2 -0.0308898860122611
AGIWUR6789;AGIWUR19798;AGIWUR22341
CB4856 CT I 9956150 9958357 WBGene00011988 har-2 T24D1.5 0.0251082607831314 AGIWUR42509
CB4856 CT I 9958782 9960155 WBGene00011986 T24D1.3 T24D1.3 -0.234861696746226 AGIWUR3679
CB4856 CT I 9960370 9961973 WBGene00007043 tag-179 T24D1.4 -0.158981010240954
AGIWUR14556;AGIWUR29087
CB4856 CT I 9965580 9970037 WBGene00003397 mom-5 T23D8.1 -0.257640873997223
AGIWUR7889;AGIWUR22460
CB4856 CT I 9970037 9973177 WBGene00006633 tsp-7 T23D8.2 0.0326901647675406 AGIWUR25168
CB4856 CT I 9973377 9976315 WBGene00011944 T23D8.3 T23D8.3 0.0526524686535517 AGIWUR31007
CB4856 CT I 9976393 9979662 WBGene00001226 eif-3.C T23D8.4 -0.186828929203759
AGIWUR3943;AGIWUR14459
CB4856 CT I 9980064 9984848 WBGene00006379 sys-1 T23D8.9 0.035250803993101
AGIWUR584;AGIWUR11881;AGIWUR12778;AGIWUR27780;AGIWUR30185;AGIWUR34013;AGIWUR43769
CB4856 CT I 9988341 9988826 WBGene00001941 his-67 T23D8.5 0.12639989375786 AGIWUR20059
CB4856 CT I 9989061 9989617 WBGene00001942 his-68 T23D8.6 0.0225186939718363
AGIWUR9020;AGIWUR10398;AGIWUR19130;AGIWUR38394;AGIWUR41721
CB4856 CT I 9989802 9993975 WBGene00011945 hpo-24 T23D8.7 -0.188436565575193
AGIWUR8843;AGIWUR15233;AGIWUR36871
CB4856 CT I 9994731 9999928 WBGene00000476 cfi-1 T23D8.8 0.180033308850422
AGIWUR4844;AGIWUR34287;AGIWUR44917
CB4856 CT I 10014547 10015495 WBGene00007274 C03C11.1 C03C11.1
-0.187282794119868 AGIWUR25138
CB4856 CT I 10016778 10018114 WBGene00001483 fog-3 C03C11.2
-0.481334871961201 AGIWUR2227
CB4856 CT I 10023810 10025831 WBGene00008663 eak-6 F10G8.4 -0.0217899337012515
AGIWUR19346

CB4856	CT	I	10026188	10030058	WBGene00003803	rae-1	F10G8.3	-0.246087832735663	AGIWUR14990
CB4856	CT	I	10030174	10046760	WBGene00008665	ercc-1	F10G8.7	0.0189458984837154	AGIWUR7402;AGIWUR8445;AGIWUR8743;AGIWUR10512
CB4856	CT	I	10046442	10048028	WBGene00008664	nubp-1	F10G8.6	0.0351497327617594	AGIWUR7984;AGIWUR8402;AGIWUR9547;AGIWUR44930
CB4856	CT	I	10048087	10050770	WBGene00008667	F10G8.9	F10G8.9	-0.00686983234939476	AGIWUR18885;AGIWUR29836;AGIWUR32672
CB4856	CT	I	10050691	10062026	WBGene00008666	F10G8.8	F10G8.8	0.421428999431759	AGIWUR15028;AGIWUR31677
CB4856	CT	I	10072578	10074399	WBGene00007159	B0379.7	B0379.7	0.265282261762451	AGIWUR21196
CB4856	CT	I	10074952	10077749	WBGene00007155	B0379.1	B0379.1	0.271415397871813	AGIWUR38309;AGIWUR41296;AGIWUR44354
CB4856	CT	I	10080159	10085777	WBGene00003508	mut-16	B0379.3	-0.1121600621995	AGIWUR788;AGIWUR33569
CB4856	CT	I	10085713	10102012	WBGene00007054	scpl-1	B0379.4	0.0554639571006561	AGIWUR35678
CB4856	CT	I	10107393	10109000	WBGene00013698	Y106G6D.1	Y106G6D.1	0.00183849116992008	AGIWUR15930
CB4856	CT	I	10110982	10111916	WBGene00013699	Y106G6D.2	Y106G6D.2	-0.0939297924792974	AGIWUR24239
CB4856	CT	I	10112088	10114256	WBGene00013700	Y106G6D.3	Y106G6D.3	0.0556358953823137	AGIWUR43241
CB4856	CT	I	10114759	10116583	WBGene00013701	Y106G6D.4	Y106G6D.4	-0.455565480246421	AGIWUR7232;AGIWUR36951
CB4856	CT	I	10117061	10118134	WBGene00013704	Y106G6D.8	Y106G6D.8	-0.0508204710487608	AGIWUR6334;AGIWUR8836;AGIWUR9264;AGIWUR27542;AGIWUR35825
CB4856	CT	I	10119209	10122171	WBGene00014965	Y106G6D.5	Y106G6D.5	-0.159773939653425	AGIWUR20599;AGIWUR43526
CB4856	CT	I	10122459	10130215	WBGene00013703	Y106G6D.7	Y106G6D.7	0.0559350331915107	AGIWUR14206;AGIWUR26940
CB4856	CT	I	10147325	10149888	WBGene00008885	F16C3.1	F16C3.1	-0.118492191956229	AGIWUR6363
CB4856	CT	I	10150280	10155131	WBGene00008886	F16C3.2	F16C3.2	1.14247407345722	AGIWUR7129
CB4856	CT	I	10155161	10158558	WBGene00023415	F16C3.3	F16C3.3	0.0690570457885809	AGIWUR25814;AGIWUR41744
CB4856	CT	I	10158951	10160034	WBGene00044243	F16C3.4	F16C3.4	-0.096131772362094	

AGIWUR37530;AGIWUR41968;AGIWUR44496
CB4856 CT I 10159982 10161883 WBGene00006447 tag-72 C25A1.3 0.114430835486949
AGIWUR27730
CB4856 CT I 10162379 10163578 WBGene00007705 C25A1.1 C25A1.1 -0.0291183064815144
AGIWUR38730
CB4856 CT I 10163475 10165135 WBGene00001442 fkh-10 C25A1.2 -0.113556521911631
AGIWUR5426
CB4856 CT I 10169639 10172645 WBGene00007706 C25A1.4 C25A1.4 -0.0660454856223787
AGIWUR7340;AGIWUR20206;AGIWUR39825
CB4856 CT I 10177181 10178921 WBGene00007707 C25A1.5 C25A1.5 0.166540526155341
AGIWUR9829
CB4856 CT I 10179832 10184075 WBGene00002153 iars-2 C25A1.7 -0.180791382673277
AGIWUR4515;AGIWUR17911;AGIWUR22885;AGIWUR24106;AGIWUR27797;AGIWUR32150
CB4856 CT I 10184478 10185470 WBGene00007709 clec-87 C25A1.8 0.0288723877824228
AGIWUR10786;AGIWUR12722;AGIWUR14448;AGIWUR17486;AGIWUR31726;AGIWUR32435
CB4856 CT I 10185780 10187550 WBGene00007710 rsa-1 C25A1.9 -0.041094706892439
AGIWUR24491
CB4856 CT I 10187515 10190896 WBGene00000931 dao-5 C25A1.10
0.0828564195156263 AGIWUR2193;AGIWUR14692;AGIWUR38010;AGIWUR41462
CB4856 CT I 10191273 10192894 WBGene00007711 lid-1 C25A1.12
-0.108237541479006
AGIWUR7759;AGIWUR12214;AGIWUR12894;AGIWUR18762;AGIWUR24648;AGIWUR30354;AGIWUR37784;AGIWUR42516;AGIWUR43176
CB4856 CT I 10193242 10193999 WBGene00007712 mrpl-34 C25A1.13
-0.0731643821759675 AGIWUR6533
CB4856 CT I 10193925 10197000 WBGene00000095 aha-1 C25A1.11
0.250178717021619 AGIWUR44293
CB4856 CT I 10197321 10197596 WBGene00007713 C25A1.15 C25A1.15
0.0739371935631681 AGIWUR19876
CB4856 CT I 10198286 10200440 WBGene00013705 Y106G6E.1 Y106G6E.1
0.0511606583147639 AGIWUR11466
CB4856 CT I 10221635 10222778 WBGene00013708 Y106G6E.4 Y106G6E.4
0.190158989949642 AGIWUR36816;AGIWUR38525
CB4856 CT I 10222881 10227009 WBGene00000426 ced-12 Y106G6E.5
0.267456120939263 AGIWUR6640;AGIWUR10649;AGIWUR39379
CB4856 CT I 10227210 10231214 WBGene00013709 csnk-1 Y106G6E.6
0.0892976056587091 AGIWUR6259;AGIWUR41120
CB4856 CT I 10248256 10255327 WBGene00003000 lin-11 ZC247.3 0.0786241154278446
AGIWUR5317;AGIWUR13003;AGIWUR24260;AGIWUR25289;AGIWUR33061
CB4856 CT I 10255632 10262250 WBGene00013860 ZC247.2 ZC247.2 -0.0377264024501152

AGIWUR1179
CB4856	CT	I	10264314	10279234	WBGene00013859	ZC247.1	ZC247.1	0.525049586345348	AGIWUR19141
CB4856	CT	I	10291870	10297927	WBGene00007047	wts-1	T20F10.1	-0.200887966955001	AGIWUR24166
CB4856	CT	I	10298140	10300598	WBGene00011864	T20F10.2	T20F10.2	-0.16148651092626	AGIWUR17094;AGIWUR17549;AGIWUR23612;AGIWUR43299
CB4856	CT	I	10300573	10302945	WBGene00011866	T20F10.5	T20F10.5	-0.0195424436222651	AGIWUR5199;AGIWUR5957;AGIWUR9005;AGIWUR20137;AGIWUR28724;AGIWUR36038
CB4856	CT	I	10309757	10311591	WBGene00045057	Y106G6G.7	Y106G6G.7	0.0317609427436221	AGIWUR20883
CB4856	CT	I	10319669	10321493	WBGene00013715	Y106G6G.6	Y106G6G.6	0.196684034330012	AGIWUR13079;AGIWUR28186
CB4856	CT	I	10321711	10323398	WBGene00013713	Y106G6G.4	Y106G6G.4	0.28737365409896	AGIWUR1645
CB4856	CT	I	10323511	10324145	WBGene00013712	dlc-6	Y106G6G.3	0.0400116819389629	AGIWUR14226
CB4856	CT	I	10328984	10330370	WBGene00013710	Y106G6G.1	Y106G6G.1	-0.0576163981573448	AGIWUR3549
CB4856	CT	I	10330879	10331808	WBGene00004476	rps-7	ZC434.2	-0.0414489559023323	AGIWUR1411;AGIWUR7247;AGIWUR25024
CB4856	CT	I	10331945	10333082	WBGene00013892	ZC434.4	ZC434.4	0.0296785762384104	AGIWUR35993
CB4856	CT	I	10333471	10334590	WBGene00220261	ZC434.10	ZC434.10	-0.426126458985402	AGIWUR43270
CB4856	CT	I	10334661	10335739	WBGene00013891	ZC434.3	ZC434.3	-0.0571527405140042	AGIWUR2990;AGIWUR17062;AGIWUR22980;AGIWUR26531
CB4856	CT	I	10336395	10341178	WBGene00001337	ears-1	ZC434.5	-0.431479185696245	AGIWUR436;AGIWUR496;AGIWUR43053
CB4856	CT	I	10340997	10345648	WBGene00000148	aph-2	ZC434.6	-0.205778047428261	AGIWUR28276
CB4856	CT	I	10345736	10348061	WBGene00013893	ZC434.7	ZC434.7	-0.26343393953781	AGIWUR7669;AGIWUR26724;AGIWUR33950;AGIWUR39834;AGIWUR42693;AGIWUR43556
CB4856	CT	I	10348055	10349379	WBGene00013894	ZC434.8	ZC434.8	-0.131780119762715	AGIWUR17368;AGIWUR26084
CB4856	CT	I	10356251	10360890	WBGene00013895	ZC434.9	ZC434.9	-0.112494907714943	AGIWUR44177
CB4856	CT	I	10366621	10371699	WBGene00009743	sptf-1	F45H11.1	0.593302361981447	AGIWUR2830;AGIWUR23379;AGIWUR41497

CB4856 CT I 10373368 10373862 WBGene00003587 ned-8 F45H11.2
-0.174752055777981 AGIWUR23345
CB4856 CT I 10374809 10379631 WBGene00009744 hpo-35 F45H11.3
-0.280287796729953 AGIWUR4287;AGIWUR33299;AGIWUR33740;AGIWUR42031
CB4856 CT I 10381082 10382040 WBGene00009745 F45H11.5 F45H11.5
-0.070211248948242 AGIWUR3840;AGIWUR9168;AGIWUR22568;AGIWUR39623
CB4856 CT I 10382746 10384222 WBGene00077521 maf-1 F45H11.6
0.0459818660027533
AGIWUR1078;AGIWUR6836;AGIWUR12363;AGIWUR12557;AGIWUR12681;AGIWUR25932;AGIWUR34710;AGIWUR39209;AGIWUR40992;A
GIWUR43296;AGIWUR43530
CB4856 CT I 10384219 10391388 WBGene00003233 mgl-2 F45H11.4
0.155688615855307 AGIWUR4879;AGIWUR20282
CB4856 CT I 10398944 10400195 WBGene00000843 cup-2 F25D7.1 -0.19625150483372
AGIWUR4401;AGIWUR20500
CB4856 CT I 10401065 10402774 WBGene00009112 tag-353 F25D7.2
-0.00673958142708919 AGIWUR6582
CB4856 CT I 10409302 10414707 WBGene00003847 blmp-1 F25D7.3 0.0788064372446962
AGIWUR1162;AGIWUR2993;AGIWUR8630;AGIWUR9914;AGIWUR13060;AGIWUR35588;AGIWUR36795;AGIWUR37572
CB4856 CT I 10422958 10429026 WBGene00009114 F25D7.5 F25D7.5
-0.00990503377677587 AGIWUR14538;AGIWUR16277;AGIWUR26686
CB4856 CT I 10429629 10432336 WBGene00013716 Y106G6H.1 Y106G6H.1
-0.308396244124841 AGIWUR3844
CB4856 CT I 10432815 10435882 WBGene00003902 pab-1 Y106G6H.2
-0.150676501774311
AGIWUR551;AGIWUR1625;AGIWUR4054;AGIWUR8505;AGIWUR14804;AGIWUR15583;AGIWUR16120;AGIWUR24249;AGIWUR29890;AGIW
UR36402
CB4856 CT I 10436254 10436884 WBGene00004444 rpl-30 Y106G6H.3
0.0826164847729878 AGIWUR24641
CB4856 CT I 10436895 10437708 WBGene00013717 madf-10 Y106G6H.4
0.630542898310765 AGIWUR8428
CB4856 CT I 10438075 10443563 WBGene00013718 pdpr-1 Y106G6H.5
-0.15829946276514 AGIWUR3494;AGIWUR4149;AGIWUR31485;AGIWUR40774
CB4856 CT I 10443668 10446133 WBGene00013719 Y106G6H.6 Y106G6H.6
-0.36454959147701 AGIWUR7986
CB4856 CT I 10446661 10452071 WBGene00004753 sec-8 Y106G6H.7
-0.0288572352058551 AGIWUR20305
CB4856 CT I 10452178 10452895 WBGene00013720 Y106G6H.8 Y106G6H.8
-0.0532363616529388 AGIWUR6889;AGIWUR24705;AGIWUR26727;AGIWUR31225
CB4856 CT I 10464328 10471056 WBGene00001112 duo-3 Y106G6H.12

-0.191058740930197	AGIWUR27835
CB4856	CT	I	10472196	10473389	WBGene00013724	Y106G6H.14	Y106G6H.14
-0.207914861786872	AGIWUR4557;AGIWUR19187;AGIWUR19475
CB4856	CT	I	10473513	10474449	WBGene00013725	ska-1	Y106G6H.15
0.157923637087279	AGIWUR32773
CB4856	CT	I	10474835	10475525	WBGene00013726	Y106G6H.16	Y106G6H.16
-0.0392976364112858	AGIWUR31191
CB4856	CT	I	10475522	10478129	WBGene00009508	row-1	F37D6.2	0.437793791688634
AGIWUR11222
CB4856	CT	I	10479283	10484969	WBGene00009507	mus-101	F37D6.1	0.116944366632971
AGIWUR201;AGIWUR29531;AGIWUR41907
CB4856	CT	I	10486714	10487322	WBGene00009509	F37D6.3	F37D6.3	-0.134973943185486
AGIWUR26244
CB4856	CT	I	10488648	10489569	WBGene00009510	F37D6.4	F37D6.4	0.04457786603334
AGIWUR11781;AGIWUR16130;AGIWUR23068;AGIWUR24394;AGIWUR24669;AGIWUR39352
CB4856	CT	I	10501585	10505949	WBGene00006445	tag-68	F37D6.6	0.0527390566030363
AGIWUR2748;AGIWUR11690;AGIWUR14931;AGIWUR27423;AGIWUR41788;AGIWUR43215
CB4856	CT	I	10507000	10515506	WBGene00000492	che-13	F59C6.7	0.39172702022546
AGIWUR10651
CB4856	CT	I	10516036	10516432	WBGene00206464	F59C6.18	F59C6.18
-0.372118910412375	AGIWUR38691
CB4856	CT	I	10518379	10519263	WBGene00010325	exos-3	F59C6.4	-0.183631787042358
AGIWUR35660
CB4856	CT	I	10519360	10520761	WBGene00010326	F59C6.5	F59C6.5	0.0739382277058578
AGIWUR2505
CB4856	CT	I	10520863	10521986	WBGene00044251	F59C6.12	F59C6.12
0.0711574414654841	AGIWUR25235;AGIWUR38986
CB4856	CT	I	10522091	10523996	WBGene00010323	dhhc-12	F59C6.2	-0.18851580205428
AGIWUR23798
CB4856	CT	I	10524424	10525509	WBGene00010324	F59C6.3	F59C6.3	0.0415372828488088
AGIWUR7210;AGIWUR25869
CB4856	CT	I	10526533	10530095	WBGene00044792	F59C6.14	F59C6.14
0.163246596264003
AGIWUR2011;AGIWUR7131;AGIWUR11982;AGIWUR22037;AGIWUR24402;AGIWUR24720;AGIWUR25617;AGIWUR28187;AGIWUR28827;A
GIWUR36554;AGIWUR38820;AGIWUR39490;AGIWUR43929
CB4856	CT	I	10530339	10531547	WBGene00014789	F59C6.10	F59C6.10
-0.126244517782972	AGIWUR29319
CB4856	CT	I	10532667	10534484	WBGene00010328	F59C6.11	F59C6.11
-0.558732947962259	AGIWUR14721

CB4856	CT	I	10543024	10546435	WBGene00009116	snx-27	F25H2.2	-0.224761998002366	AGIWUR29978
CB4856	CT	I	10547721	10549876	WBGene00009117	F25H2.3	F25H2.3	-0.248849991757946	AGIWUR40211
CB4856	CT	I	10551972	10553035	WBGene00009118	F25H2.4	F25H2.4	-0.233620733717901	AGIWUR22807
CB4856	CT	I	10553116	10553913	WBGene00009119	ndk-1	F25H2.5	-0.00571806185154244	AGIWUR1428;AGIWUR4491;AGIWUR7177;AGIWUR8978;AGIWUR10087;AGIWUR11437;AGIWUR13155;AGIWUR13297;AGIWUR14349;AGIWUR23883;AGIWUR25992;AGIWUR32023;AGIWUR32499;AGIWUR33225;AGIWUR36407;AGIWUR39612;AGIWUR41122;AGIWUR42164;AGIWUR44511;AGIWUR45155
CB4856	CT	I	10554366	10557838	WBGene00009120	F25H2.6	F25H2.6	0.0938884191276781	AGIWUR32606
CB4856	CT	I	10558323	10560006	WBGene00009121	F25H2.7	F25H2.7	-0.152201274704263	AGIWUR30852
CB4856	CT	I	10562968	10564914	WBGene00006720	ubc-25	F25H2.8	0.129331901563821	AGIWUR14963;AGIWUR16386;AGIWUR38751
CB4856	CT	I	10566350	10567300	WBGene00003926	pas-5	F25H2.9	-0.298897175354938	AGIWUR16852
CB4856	CT	I	10567894	10569019	WBGene00004408	rla-0	F25H2.10	-0.15863993279315	AGIWUR3634;AGIWUR4230;AGIWUR5948;AGIWUR12550;AGIWUR14129;AGIWUR17615;AGIWUR31740
CB4856	CT	I	10569112	10570030	WBGene00009122	tct-1	F25H2.11	0.0692412661273103	AGIWUR34683
CB4856	CT	I	10570079	10571916	WBGene00009123	F25H2.12	F25H2.12	-0.0593431887278477	AGIWUR42316
CB4856	CT	I	10571954	10577135	WBGene00009124	rtel-1	F25H2.13	-0.216735614942866	AGIWUR14329
CB4856	CT	I	10579413	10580890	WBGene00009243	F29C6.1	F29C6.1	-0.41964510547117	AGIWUR1176;AGIWUR28962
CB4856	CT	I	10581538	10582609	WBGene00011906	hsp-12.1	T22A3.2	0.47842393039042	AGIWUR22241
CB4856	CT	I	10588177	10590464	WBGene00003083	lst-1	T22A3.3	-0.140424257032498	AGIWUR4603
CB4856	CT	I	10590678	10593972	WBGene00044070	set-18	T22A3.4	-0.431450777110815	AGIWUR474
CB4856	CT	I	10595545	10598490	WBGene00011909	T22A3.6	T22A3.6	-0.166848596108249	AGIWUR40214
CB4856	CT	I	10598673	10606620	WBGene00011908	pash-1	T22A3.5	-0.268738812917681	AGIWUR31209

CB4856	CT	I	10608466	10621433	WBGene00002248	lam-3	T22A3.8	0.550685928477949	AGIWUR5500;AGIWUR37672
CB4856	CT	I	10622952	10624361	WBGene00016622	mafr-1	C43H8.2	0.270751118430026	AGIWUR45017
CB4856	CT	I	10624464	10625387	WBGene00016621	arch-1	C43H8.1	-0.245133486166667	AGIWUR41519
CB4856	CT	I	10625388	10628387	WBGene00015232	B0511.6	B0511.6	0.132641196282066	AGIWUR3687;AGIWUR4208;AGIWUR4272;AGIWUR4753;AGIWUR45010
CB4856	CT	I	10628712	10631674	WBGene00015231	cutl-14	B0511.5	0.166853109976367	AGIWUR12758
CB4856	CT	I	10631665	10633150	WBGene00015233	B0511.7	B0511.7	-0.0206744893119293	AGIWUR29044
CB4856	CT	I	10633279	10634078	WBGene00255714	B0511.19	B0511.19	-0.350542059522331	AGIWUR2149
CB4856	CT	I	10636692	10638977	WBGene00044321	mrps-30	B0511.8	-0.11392926707985	AGIWUR24656
CB4856	CT	I	10639079	10640230	WBGene00015235	cdc-26	B0511.9	-0.154598637020798	AGIWUR4793;AGIWUR28476;AGIWUR31998;AGIWUR37074;AGIWUR37096;AGIWUR40826
CB4856	CT	I	10640465	10642281	WBGene00001228	eif-3.E	B0511.10	0.0457430355724924	AGIWUR37070
CB4856	CT	I	10644245	10645617	WBGene00015230	tag-344	B0511.4	0.0160574472507609	AGIWUR24553
CB4856	CT	I	10646762	10647777	WBGene00015229	fbxa-125	B0511.3	-0.0920607058741609	AGIWUR14685
CB4856	CT	I	10648606	10654179	WBGene00015236	B0511.11	B0511.11	-0.00982527415533241	AGIWUR44598
CB4856	CT	I	10654298	10666933	WBGene00015237	B0511.12	B0511.12	-0.0437554138112368	AGIWUR11649;AGIWUR13533;AGIWUR15421
CB4856	CT	I	10667808	10671301	WBGene00001432	fkb-7	B0511.1	0.559740860401194	AGIWUR39506
CB4856	CT	I	10671460	10672589	WBGene00016389	C34B2.4	C34B2.4	-0.108583226998904	AGIWUR41157
CB4856	CT	I	10675167	10676811	WBGene00016390	C34B2.5	C34B2.5	-0.297147958736656	AGIWUR7716;AGIWUR29153
CB4856	CT	I	10676922	10682374	WBGene00016391	lonp-1	C34B2.6	-0.0320720442023292	AGIWUR943;AGIWUR2718;AGIWUR5681
CB4856	CT	I	10682472	10683155	WBGene00016395	C34B2.10	C34B2.10	-0.41977329530481	AGIWUR14716;AGIWUR15847
CB4856	CT	I	10683270	10685664	WBGene00016392	sdha-2	C34B2.7	0.0332733453174647	

AGIWUR38680
CB4856 CT I 10685825 10687058 WBGene00016393 C34B2.8 C34B2.8 -0.0546233931933049
AGIWUR1458;AGIWUR3758;AGIWUR26926;AGIWUR36412;AGIWUR43567;AGIWUR44915
CB4856 CT I 10687363 10688068 WBGene00016387 kbp-5 C34B2.2 -0.0573079398100091
AGIWUR11015
CB4856 CT I 10688332 10690790 WBGene00016386 tag-272 C34B2.1 0.0517378128668309
AGIWUR21234
CB4856 CT I 10691345 10696783 WBGene00016396 C34B2.11 C34B2.11
-0.0983375111577747 AGIWUR2570;AGIWUR10840;AGIWUR43723
CB4856 CT I 10699562 10703801 WBGene00016394 C34B2.9 C34B2.9 -0.233529841957084
AGIWUR34791
CB4856 CT I 10708363 10710376 WBGene00015024 B0205.10 B0205.10
-0.177253740946503 AGIWUR13757
CB4856 CT I 10713505 10718201 WBGene00015017 B0205.1 B0205.1 -0.0689260607047046
AGIWUR28744
CB4856 CT I 10720813 10723496 WBGene00015023 B0205.9 B0205.9 -0.087887687606429
AGIWUR7558
CB4856 CT I 10724161 10726136 WBGene00015018 srz-85 B0205.2 0.0854523976499237
AGIWUR40535
CB4856 CT I 10726903 10727364 WBGene00044646 B0205.14 B0205.14
-0.427231464488038 AGIWUR19150;AGIWUR26793;AGIWUR29668;AGIWUR31946;AGIWUR42563
CB4856 CT I 10727755 10728808 WBGene00044644 B0205.13 B0205.13
-1.05847362406007 AGIWUR18822;AGIWUR35516;AGIWUR42241
CB4856 CT I 10728959 10730287 WBGene00015022 B0205.8 B0205.8 -0.0920172338082779
AGIWUR1980
CB4856 CT I 10730467 10730743 WBGene00044413 B0205.12 B0205.12
0.0994985390136272 AGIWUR32246;AGIWUR34973;AGIWUR40595;AGIWUR42095;AGIWUR44666
CB4856 CT I 10730712 10732448 WBGene00004466 rpn-10 B0205.3 -0.238576788056143
AGIWUR1283;AGIWUR18646;AGIWUR19121;AGIWUR19902;AGIWUR37875
CB4856 CT I 10732543 10733686 WBGene00015025 mrpl-9 B0205.11
-0.135115556374533 AGIWUR17494
CB4856 CT I 10734022 10736615 WBGene00002191 kin-3 B0205.7
-0.00315339732642261 AGIWUR6814;AGIWUR14979;AGIWUR16721;AGIWUR31425;AGIWUR35891;AGIWUR36826
CB4856 CT I 10736697 10739911 WBGene00015021 B0205.6 B0205.6 -0.233655362221736
AGIWUR33097
CB4856 CT I 10740218 10740590 WBGene00015020 B0205.5 B0205.5 -0.190881742638758
AGIWUR43771
CB4856 CT I 10741368 10744445 WBGene00015019 B0205.4 B0205.4 0.627788316518761
AGIWUR6510;AGIWUR34542

CB4856 CT I 10750343 10776700 WBGene00000912 daf-16 R13H8.1 0.0656835679501295
AGIWUR15683;AGIWUR17069;AGIWUR19627;AGIWUR26737;AGIWUR27096
CB4856 CT I 10781442 10782890 WBGene00018850 gpx-8 F55A3.5 -0.194946202732729
AGIWUR1381
CB4856 CT I 10785128 10787863 WBGene00018852 F55A3.7 F55A3.7 -0.229293690377512
AGIWUR35755
CB4856 CT I 10789130 10793152 WBGene00018849 F55A3.3 F55A3.3
-0.00532318268524978 AGIWUR21198;AGIWUR26112;AGIWUR28742;AGIWUR36072;AGIWUR43803;AGIWUR44363
CB4856 CT I 10793161 10798059 WBGene00018848 F55A3.2 F55A3.2 -0.0486149148742641
AGIWUR32325
CB4856 CT I 10799623 10806901 WBGene00018847 marc-6 F55A3.1 -0.182753525961932
AGIWUR19410
CB4856 CT I 10806922 10808238 WBGene00016461 C35E7.9 C35E7.9 0.126845449994869
AGIWUR15468;AGIWUR34153
CB4856 CT I 10808590 10810497 WBGene00016460 C35E7.8 C35E7.8 -0.397669201377564
AGIWUR12957
CB4856 CT I 10810648 10812923 WBGene00016462 C35E7.10 C35E7.10
-0.104046451199075 AGIWUR1500
CB4856 CT I 10813815 10814904 WBGene00016459 C35E7.7 C35E7.7 -0.0481934128196428
AGIWUR27260
CB4856 CT I 10817352 10819963 WBGene00016458 C35E7.6 C35E7.6 0.521152771073229
AGIWUR19288
CB4856 CT I 10823363 10828454 WBGene00016457 C35E7.5 C35E7.5 0.0745361493326415
AGIWUR221;AGIWUR3071;AGIWUR7500;AGIWUR13387;AGIWUR13605;AGIWUR15219;AGIWUR35549
CB4856 CT I 10831881 10834812 WBGene00016456 C35E7.4 C35E7.4 0.578068016503412
AGIWUR26878
CB4856 CT I 10836985 10838877 WBGene00016455 C35E7.3 C35E7.3 0.0107805374910726
AGIWUR38197
CB4856 CT I 10841114 10844647 WBGene00016454 C35E7.2 C35E7.2 0.131750439236914
AGIWUR9766
CB4856 CT I 10844772 10845922 WBGene00016463 C35E7.11 C35E7.11
-0.0242412509819712 AGIWUR28616
CB4856 CT I 10846777 10850103 WBGene00016453 vet-2 C35E7.1 0.0921622213113498
AGIWUR10906
CB4856 CT I 10855935 10857620 WBGene00021383 Y37F4.2 Y37F4.2 0.0616028145465926
AGIWUR3056
CB4856 CT I 10859534 10860769 WBGene00021382 Y37F4.1 Y37F4.1 -0.133557614307193
AGIWUR7087
CB4856 CT I 10861215 10863453 WBGene00021386 Y37F4.5 Y37F4.5 0.370427156580222

AGIWUR44323
CB4856 CT I 10863880 10866495 WBGene00021388 Y37F4.7 Y37F4.7 -0.425648644784092
AGIWUR1404;AGIWUR3734
CB4856 CT I 10867554 10868530 WBGene00044289 Y37F4.8 Y37F4.8 1.28027960428838
AGIWUR36705
CB4856 CT I 10870023 10875046 WBGene00021387 Y37F4.6 Y37F4.6 -0.273199638742438
AGIWUR44270
CB4856 CT I 10878047 10878378 WBGene00020860 nspd-10 T27C10.7
-0.0880002532370234 AGIWUR23135
CB4856 CT I 10879138 10879572 WBGene00269435 T27C10.9 T27C10.9
0.11507522100411 AGIWUR40191
CB4856 CT I 10879769 10894239 WBGene00003068 lrk-1 T27C10.6
0.240153457420273 AGIWUR10723
CB4856 CT I 10899110 10902024 WBGene00020857 srt-62 T27C10.2
0.0307234824357288 AGIWUR18376;AGIWUR33397
CB4856 CT I 10906812 10912203 WBGene00018630 F49D11.6 F49D11.6
0.00405097578870262 AGIWUR9641
CB4856 CT I 10912913 10915796 WBGene00018628 F49D11.4 F49D11.4
0.0493148121005333 AGIWUR33044
CB4856 CT I 10917064 10919095 WBGene00018627 F49D11.3 F49D11.3
-0.346392969682515 AGIWUR7428
CB4856 CT I 10921416 10923484 WBGene00018626 F49D11.2 F49D11.2
-0.156350408043203 AGIWUR4503;AGIWUR16229;AGIWUR16872
CB4856 CT I 10923574 10923944 WBGene00018631 F49D11.7 F49D11.7
0.105338756689388 AGIWUR2557;AGIWUR8303;AGIWUR9080;AGIWUR9566;AGIWUR38563;AGIWUR40010
CB4856 CT I 10924240 10925753 WBGene00000780 cpn-4 F49D11.8
-0.123235964346208 AGIWUR2714;AGIWUR20006;AGIWUR29506
CB4856 CT I 10926883 10933705 WBGene00045433 F49D11.10 F49D11.10
-0.169685490362001 AGIWUR42404
CB4856 CT I 10933810 10935712 WBGene00018632 tag-296 F49D11.9
0.4551816883152 AGIWUR11932
CB4856 CT I 10936819 10940521 WBGene00018625 prp-17 F49D11.1
-0.167788442374261 AGIWUR16544
CB4856 CT I 10940685 10943922 WBGene00045434 Y95D11A.3 Y95D11A.3
-0.0847591598708348 AGIWUR10746;AGIWUR23575
CB4856 CT I 10944210 10945690 WBGene00013605 Y95D11A.1 Y95D11A.1
0.234562608965922 AGIWUR11591
CB4856 CT I 10958297 10959906 WBGene00044262 Y95D11A.2 Y95D11A.2
0.156238237515171 AGIWUR38881

CB4856	CT	I	10964473	10966348	WBGene00013121	spe-38	Y52B11A.1	-0.147078353115207	AGIWUR10936;AGIWUR13151;AGIWUR29137
CB4856	CT	I	10966394	10970166	WBGene00013122	impt-1	Y52B11A.2	-0.223934465932739	AGIWUR5756;AGIWUR11019;AGIWUR12958;AGIWUR13000;AGIWUR18911;AGIWUR29124
CB4856	CT	I	10970493	10980111	WBGene00013123	Y52B11A.3	Y52B11A.3	-0.0433611685615309	AGIWUR948;AGIWUR8774;AGIWUR10299;AGIWUR30518;AGIWUR35426;AGIWUR35479
CB4856	CT	I	11011435	11014274	WBGene00219422	Y52B11A.20	Y52B11A.20	0.0162194521375438	AGIWUR3523;AGIWUR43245
CB4856	CT	I	11012959	11014739	WBGene00077539	Y52B11A.12	Y52B11A.12	0.632291807256584	AGIWUR8711;AGIWUR18783
CB4856	CT	I	11015486	11017497	WBGene00013125	clec-92	Y52B11A.5	-0.436234163573509	AGIWUR15108
CB4856	CT	I	11018571	11025653	WBGene00013126	mltn-2	Y52B11A.7	-0.0536476408351354	AGIWUR13171;AGIWUR21642
CB4856	CT	I	11032339	11034055	WBGene00013127	Y52B11A.8	Y52B11A.8	-0.0614152766066436	AGIWUR32358
CB4856	CT	I	11034057	11038857	WBGene00013128	dxbp-1	Y52B11A.9	-0.00933916921782973	AGIWUR22113
CB4856	CT	I	11039056	11052494	WBGene00013129	Y52B11A.10	Y52B11A.10	0.0114302751149893	AGIWUR18772;AGIWUR35839;AGIWUR36487
CB4856	CT	I	11067699	11069996	WBGene00014914	Y52B11A.11	Y52B11A.11	-0.181064297751257	AGIWUR356;AGIWUR941;AGIWUR13335;AGIWUR18309;AGIWUR23163
CB4856	CT	I	11075758	11134667	WBGene00001980	hmr-1	W02B9.1	0.184601369495883	AGIWUR2464;AGIWUR4079;AGIWUR7642;AGIWUR14037;AGIWUR25685;AGIWUR25807
CB4856	CT	I	11142113	11144090	WBGene00013929	clec-93	ZK39.4	0.262756086931308	AGIWUR16569
CB4856	CT	I	11144652	11145696	WBGene00044266	ZK39.9	ZK39.9	0.0385011632994612	AGIWUR3953;AGIWUR18942;AGIWUR31124;AGIWUR35697;AGIWUR38465
CB4856	CT	I	11146448	11148309	WBGene00013928	clec-94	ZK39.3	-0.0377526911271673	AGIWUR7109
CB4856	CT	I	11148973	11150295	WBGene00013927	clec-95	ZK39.2	-0.498145350279138	AGIWUR14880
CB4856	CT	I	11151241	11151972	WBGene00013930	clec-96	ZK39.5	-0.828952375839542	AGIWUR38744
CB4856	CT	I	11152704	11154329	WBGene00013931	clec-97	ZK39.6	-0.411563179192968	AGIWUR3379;AGIWUR10205
CB4856	CT	I	11156745	11158831	WBGene00013933	clec-99	ZK39.8	-0.514207856432543	AGIWUR13126
CB4856	CT	I	11160034	11162602	WBGene00050881	ZK39.10	ZK39.10	-0.0703612748255349

AGIWUR15295;AGIWUR25506
CB4856 CT I 11163811 11168736 WBGene00013131 Y52B11C.1 Y52B11C.1
-0.0451375644205338 AGIWUR30055
CB4856 CT I 11177805 11182115 WBGene00004706 rsr-1 F28D9.1 0.0913660656365884
AGIWUR25663
CB4856 CT I 11182817 11186137 WBGene00005517 sri-5 F28D9.2 -0.296896803420363
AGIWUR20194
CB4856 CT I 11206922 11211842 WBGene00012317 ztf-6 W06H12.1
0.45078534985709 AGIWUR4883;AGIWUR5680;AGIWUR8022;AGIWUR21252;AGIWUR28412;AGIWUR42642
CB4856 CT I 11212426 11215091 WBGene00002119 ins-36 Y53H1A.4
-0.0852252966284137 AGIWUR17879;AGIWUR21406;AGIWUR34002;AGIWUR36162;AGIWUR38489
CB4856 CT I 11216118 11221995 WBGene00013177 rsy-1 Y53H1A.1
-0.0623119555957325 AGIWUR974;AGIWUR14921
CB4856 CT I 11222326 11224400 WBGene00013178 Y53H1A.2 Y53H1A.2
-0.214860327473099 AGIWUR14334;AGIWUR20569;AGIWUR21366
CB4856 CT I 11224836 11233334 WBGene00043056 nfya-2 Y53H1A.5
-0.0412819413533505 AGIWUR33672
CB4856 CT I 11234302 11235176 WBGene00013179 clec-100 Y53H1A.3
-0.21251613258959 AGIWUR16219
CB4856 CT I 11235385 11235594 WBGene00044781 Y53H1A.6 Y53H1A.6
-0.0265497411570781 AGIWUR13135
CB4856 CT I 11243836 11245003 WBGene00009749 F46A8.6 F46A8.6 -0.433169932535407
AGIWUR32965
CB4856 CT I 11245823 11246765 WBGene00014760 F46A8.2 F46A8.2 0.0641116411896534
AGIWUR32278;AGIWUR33361
CB4856 CT I 11248270 11249952 WBGene00014761 F46A8.11 F46A8.11
0.0842814722143048 AGIWUR14328;AGIWUR40171
CB4856 CT I 11250139 11251602 WBGene00014759 F46A8.1 F46A8.1 0.851120187198253
AGIWUR15744;AGIWUR23653;AGIWUR27635
CB4856 CT I 11252238 11252981 WBGene00009752 F46A8.9 F46A8.9 0.069563859639695
AGIWUR9494
CB4856 CT I 11253256 11256022 WBGene00009753 scrm-6 F46A8.10
0.0403783846108787 AGIWUR31148
CB4856 CT I 11256550 11259225 WBGene00009467 F36D1.8 F36D1.8 0.176452709798728
AGIWUR29021
CB4856 CT I 11263066 11263661 WBGene00009466 F36D1.7 F36D1.7 1.01021491558952
AGIWUR2766;AGIWUR3959;AGIWUR7252;AGIWUR15487;AGIWUR32950;AGIWUR45156
CB4856 CT I 11265523 11265714 WBGene00077524 F36D1.11 F36D1.11
0.0281884264411087 AGIWUR3397;AGIWUR28134

CB4856	CT	I	11266070	11266474	WBGene00009464	F36D1.5	F36D1.5	-0.192201115563904	AGIWUR19361
CB4856	CT	I	11268030	11268409	WBGene00044246	F36D1.10	F36D1.10	-0.01015576297356	AGIWUR2407;AGIWUR4902;AGIWUR6043;AGIWUR20336;AGIWUR24359
CB4856	CT	I	11269946	11271226	WBGene00009465	F36D1.6	F36D1.6	-0.0188870097922846	AGIWUR5059;AGIWUR16986;AGIWUR23497
CB4856	CT	I	11271708	11272346	WBGene00009463	F36D1.4	F36D1.4	-0.0724648561294501	AGIWUR27994
CB4856	CT	I	11274899	11276346	WBGene00009468	F36D1.9	F36D1.9	-0.0776310503808482	AGIWUR28466;AGIWUR30243;AGIWUR30436
CB4856	CT	I	11276451	11280018	WBGene00009461	madf-2	F36D1.1	-0.0657237152248111	AGIWUR18025
CB4856	CT	I	11282473	11286290	WBGene00009462	sre-22	F36D1.2	-0.0433708746744657	AGIWUR8829;AGIWUR32183
CB4856	CT	I	11289044	11291868	WBGene00005710	sru-47	F36D1.3	0.148050701073606	AGIWUR34147
CB4856	CT	I	11292662	11294197	WBGene00013181	Y53H1B.2	Y53H1B.2	0.549788987012956	AGIWUR1839
CB4856	CT	I	11294593	11297206	WBGene00013180	cutl-10	Y53H1B.1	0.526968901592901	AGIWUR9275
CB4856	CT	I	11300743	11301662	WBGene00013182	Y53H1B.4	Y53H1B.4	0.322906071191033	AGIWUR40711
CB4856	CT	I	11305882	11307450	WBGene00219319	H25P06.5	H25P06.5	0.0969570820374894	AGIWUR14863;AGIWUR22874;AGIWUR29330;AGIWUR31715;AGIWUR39430
CB4856	CT	I	11311516	11323110	WBGene00010416	hxk-2	H25P06.1	-0.177197375813924	AGIWUR19499
CB4856	CT	I	11323527	11333152	WBGene00000410	cdk-9	H25P06.2	-0.239859735983972	AGIWUR2599;AGIWUR5704;AGIWUR16319;AGIWUR18803;AGIWUR22679
CB4856	CT	I	11334223	11337431	WBGene00014807	M04D5.1	M04D5.1	-0.289211883793441	AGIWUR1348;AGIWUR28722
CB4856	CT	I	11339787	11341521	WBGene00077770	M04D5.3	M04D5.3	-0.0949833802620148	AGIWUR20198
CB4856	CT	I	11361090	11362900	WBGene00044250	F56G4.7	F56G4.7	-0.139161512478967	AGIWUR219;AGIWUR6931;AGIWUR14752;AGIWUR17788;AGIWUR20636
CB4856	CT	I	11364321	11374334	WBGene00010161	F56G4.6	F56G4.6	0.109980189764155	AGIWUR42088
CB4856	CT	I	11374620	11380127	WBGene00010159	F56G4.4	F56G4.4	-0.202375025433255	AGIWUR21621
CB4856	CT	I	11380231	11388751	WBGene00010160	png-1	F56G4.5	-0.103781908677327	

AGIWUR20127
CB4856 CT I 11388689 11396660 WBGene00000010 aat-9 Y53H1C.1
-0.171394850891557 AGIWUR16336;AGIWUR17335;AGIWUR18837
CB4856 CT I 11400612 11416427 WBGene00001215 ego-2 Y53H1C.2
-0.149197103390792 AGIWUR2467;AGIWUR2804;AGIWUR4341;AGIWUR10016;AGIWUR25366;AGIWUR45207
CB4856 CT I 11417844 11421236 WBGene00013186 Y53H1C.3 Y53H1C.3
-0.110983329754552 AGIWUR20556
CB4856 CT I 11422190 11423600 WBGene00007769 C27C7.7 C27C7.7 -0.245769263422937
AGIWUR13993
CB4856 CT I 11423993 11426054 WBGene00007768 C27C7.5 C27C7.5 0.0263323122385941
AGIWUR1894
CB4856 CT I 11429029 11432145 WBGene00003664 nhr-74 C27C7.3 -0.282634535820938
AGIWUR43950
CB4856 CT I 11443452 11446644 WBGene00014992 ZK1025.1 ZK1025.1
0.00718508150039643 AGIWUR9385
CB4856 CT I 11460780 11463074 WBGene00014183 ZK1025.3 ZK1025.3
-0.217173352311953 AGIWUR9770
CB4856 CT I 11473218 11475677 WBGene00003703 nhr-113 ZK1025.9
-0.0185078216405508 AGIWUR19572
CB4856 CT I 11483392 11484571 WBGene00010652 K08C9.5 K08C9.5 0.362784494120348
AGIWUR40298
CB4856 CT I 11484778 11486598 WBGene00010650 K08C9.1 K08C9.1 -0.1337575146208
AGIWUR24763
CB4856 CT I 11486895 11488471 WBGene00010651 K08C9.2 K08C9.2 0.0760394803732645
AGIWUR16644
CB4856 CT I 11489158 11490630 WBGene00000641 col-65 K08C9.4 -0.192477877290241
AGIWUR17873
CB4856 CT I 11493036 11495080 WBGene00045218 K08C9.8 K08C9.8 -0.178887242159234
AGIWUR14097;AGIWUR39873
CB4856 CT I 11498914 11499655 WBGene00010653 K08C9.6 K08C9.6
-0.00954491015330738 AGIWUR354;AGIWUR5663;AGIWUR10669;AGIWUR15581;AGIWUR43275
CB4856 CT I 11501625 11507129 WBGene00009318 F32B4.8 F32B4.8 0.175329271143692
AGIWUR31965
CB4856 CT I 11510066 11512769 WBGene00009312 F32B4.1 F32B4.1 -0.0882477757437261
AGIWUR10881;AGIWUR12015;AGIWUR29788
CB4856 CT I 11512840 11513853 WBGene00009313 F32B4.2 F32B4.2 -0.309915693187989
AGIWUR23120;AGIWUR37167;AGIWUR44941
CB4856 CT I 11515127 11517108 WBGene00009316 abhd-11.1 F32B4.6
0.188505817592647 AGIWUR39042

CB4856 CT I 11517558 11526962 WBGene00009314 F32B4.4 F32B4.4 -0.0457168489520408
AGIWUR25980;AGIWUR30552
CB4856 CT I 11526918 11557851 WBGene00001131 dys-1 F15D3.1 0.0899899897266447
AGIWUR12141;AGIWUR22309;AGIWUR24862;AGIWUR34445;AGIWUR40088
CB4856 CT I 11557986 11558671 WBGene00077557 F15D3.9 F15D3.9 -0.0980554935351368
AGIWUR2903;AGIWUR3479;AGIWUR5850;AGIWUR17559;AGIWUR22084
CB4856 CT I 11560637 11564387 WBGene00008853 clec-101 F15D3.2
0.220107591406723 AGIWUR11727;AGIWUR34026
CB4856 CT I 11565238 11570803 WBGene00008854 F15D3.4 F15D3.4 -0.2408236830247
AGIWUR1550
CB4856 CT I 11570886 11571178 WBGene00008855 F15D3.5 F15D3.5 0.0666121049470645
AGIWUR38955
CB4856 CT I 11572121 11577488 WBGene00008856 F15D3.6 F15D3.6 0.292039390175924
AGIWUR36119;AGIWUR37869
CB4856 CT I 11578641 11579612 WBGene00008857 timm-23 F15D3.7 0.111587835079581
AGIWUR37010
CB4856 CT I 11579861 11581986 WBGene00008858 pals-1 F15D3.8 -0.0112714381360224
AGIWUR20103
CB4856 CT I 11582743 11586069 WBGene00004963 spe-9 C17D12.6
0.275947200870283 AGIWUR2003
CB4856 CT I 11585922 11587294 WBGene00007639 C17D12.5 C17D12.5
-0.319386182519327 AGIWUR29807
CB4856 CT I 11592302 11602095 WBGene00006807 unc-75 C17D12.2
0.464469273189947 AGIWUR17392;AGIWUR20095
CB4856 CT I 11604027 11611314 WBGene00007640 C17D12.7 C17D12.7
-0.213470372610457 AGIWUR32357
CB4856 CT I 11611672 11616244 WBGene00007637 dhhc-7 C17D12.1
0.104130452373312 AGIWUR38592
CB4856 CT I 11616816 11620263 WBGene00007638 C17D12.3 C17D12.3
0.555814495483506 AGIWUR9660
CB4856 CT I 11630064 11632879 WBGene00012265 W04G5.4 W04G5.4 0.0977657247878465
AGIWUR32019;AGIWUR32119;AGIWUR43756
CB4856 CT I 11633002 11638491 WBGene00004275 rab-11.2 W04G5.2
0.0363129109538365 AGIWUR6515;AGIWUR17616;AGIWUR24210
CB4856 CT I 11638482 11640414 WBGene00012270 W04G5.10 W04G5.10
0.136469247891621 AGIWUR4128;AGIWUR31161;AGIWUR34859
CB4856 CT I 11640687 11642206 WBGene00002205 kin-23 W04G5.6 0.0505087884052431
AGIWUR14720
CB4856 CT I 11642625 11645420 WBGene00012269 W04G5.9 W04G5.9 -0.246332689964252

AGIWUR26630
CB4856 CT I 11646525 11647020 WBGene00045075 W04G5.12 W04G5.12
0.144063055444611 AGIWUR863;AGIWUR7332;AGIWUR13915
CB4856 CT I 11647753 11648428 WBGene00045076 W04G5.13 W04G5.13
-0.056781719231684 AGIWUR11630;AGIWUR42286;AGIWUR42442
CB4856 CT I 11649982 11651912 WBGene00012268 W04G5.8 W04G5.8 0.205769951156471
AGIWUR29292
CB4856 CT I 11652789 11654254 WBGene00012267 W04G5.7 W04G5.7 0.123026824740883
AGIWUR227;AGIWUR4758;AGIWUR23631
CB4856 CT I 11675850 11680975 WBGene00002232 kpc-1 F11A6.1 0.447608259182147
AGIWUR6129;AGIWUR35941;AGIWUR40036;AGIWUR40532;AGIWUR41868;AGIWUR43223;AGIWUR44060
CB4856 CT I 11680913 11682441 WBGene00008681 scrm-4 F11A6.2
-0.00251908320094074 AGIWUR12708;AGIWUR15152;AGIWUR37914
CB4856 CT I 11687811 11688522 WBGene00077549 F11A6.3 F11A6.3 -0.0847745766481953
AGIWUR5970
CB4856 CT I 11688680 11690830 WBGene00011935 scrm-1 T22H2.5 0.0599576674197184
AGIWUR22686;AGIWUR42928
CB4856 CT I 11694722 11694997 WBGene00011934 T22H2.4 T22H2.4 -0.215258887271228
AGIWUR33144
CB4856 CT I 11695339 11697943 WBGene00005523 sri-11 T22H2.3 0.214595639870494
AGIWUR24552
CB4856 CT I 11699169 11701178 WBGene00005524 sri-12 T22H2.1 -0.0427959403663518
AGIWUR26364
CB4856 CT I 11701631 11703698 WBGene00011936 pgrn-1 T22H2.6 -0.111263560300892
AGIWUR27058;AGIWUR43309
CB4856 CT I 11703718 11706612 WBGene00011933 oac-47 T22H2.2 0.146778428561538
AGIWUR23499
CB4856 CT I 11707818 11712197 WBGene00009413 oac-19 F35E2.6 -0.26222280108917
AGIWUR25636
CB4856 CT I 11714956 11717616 WBGene00009414 F35E2.7 F35E2.7 -0.380703581252723
AGIWUR21665
CB4856 CT I 11718864 11719790 WBGene00009415 F35E2.8 F35E2.8 0.264671223945662
AGIWUR4814
CB4856 CT I 11719902 11722936 WBGene00044270 F35E2.10 F35E2.10
-0.227745633395248 AGIWUR4911;AGIWUR28015;AGIWUR32014;AGIWUR44280
CB4856 CT I 11723427 11726326 WBGene00009416 F35E2.9 F35E2.9 -0.441810346856808
AGIWUR15496
CB4856 CT I 11733940 11735964 WBGene00009411 F35E2.3 F35E2.3 -0.266849815353072
AGIWUR643;AGIWUR24810;AGIWUR32708;AGIWUR40664;AGIWUR43891

CB4856	CT	I	11736637	11737699	WBGene00009410	F35E2.2	F35E2.2	0.344816769636671	AGIWUR38091
CB4856	CT	I	11741603	11743951	WBGene00009412	F35E2.5	F35E2.5	-0.135459118299369	AGIWUR19040;AGIWUR31412
CB4856	CT	I	11744172	11747451	WBGene00012946	Y47H9B.2	Y47H9B.2	-0.00289846661698334	AGIWUR3183;AGIWUR27320;AGIWUR39374;AGIWUR42978
CB4856	CT	I	11747428	11792118	WBGene00006876	vab-10	ZK1151.1	0.0109205573916391	AGIWUR5594;AGIWUR6483;AGIWUR14405;AGIWUR15572;AGIWUR17308;AGIWUR19666;AGIWUR20230;AGIWUR25708;AGIWUR38637
CB4856	CT	I	11797032	11797947	WBGene00011384	T02G6.1	T02G6.1	-0.164917544180385	AGIWUR30583
CB4856	CT	I	11800433	11804991	WBGene00011385	T02G6.2	T02G6.2	-0.466824717287789	AGIWUR1183;AGIWUR9681;AGIWUR13350;AGIWUR33790;AGIWUR38283;AGIWUR38543
CB4856	CT	I	11809132	11811266	WBGene00011387	T02G6.4	T02G6.4	0.0644301540870318	AGIWUR25717
CB4856	CT	I	11813779	11814451	WBGene00011386	T02G6.3	T02G6.3	0.0262794993473783	AGIWUR33810
CB4856	CT	I	11815910	11819277	WBGene00011388	T02G6.5	T02G6.5	-0.278018358874604	AGIWUR1129
CB4856	CT	I	11820688	11823459	WBGene00086562	T02G6.11	T02G6.11	-0.0293247546269355	AGIWUR4176;AGIWUR10405;AGIWUR11978;AGIWUR13971;AGIWUR40335;AGIWUR44344
CB4856	CT	I	11827549	11828619	WBGene00011390	T02G6.7	T02G6.7	-0.130981266658198	AGIWUR6776
CB4856	CT	I	11830985	11833166	WBGene00012947	Y47H9C.1	Y47H9C.1	-0.097835044078467	AGIWUR41747
CB4856	CT	I	11836460	11849251	WBGene00012948	dhhc-2	Y47H9C.2	0.0310460431030624	AGIWUR13146
CB4856	CT	I	11851566	11868105	WBGene00000415	ced-1	Y47H9C.4	0.191872926701646	AGIWUR17222;AGIWUR19662;AGIWUR22787;AGIWUR40343
CB4856	CT	I	11888501	11890558	WBGene00000821	csp-3	Y47H9C.6	0.0581992494147627	AGIWUR42697
CB4856	CT	I	11891549	11893727	WBGene00012950	eif-2Bbeta	Y47H9C.7	0.0264840526788284	AGIWUR36725
CB4856	CT	I	11893881	11896284	WBGene00012951	Y47H9C.8	Y47H9C.8	-0.0444216975775849	AGIWUR13628
CB4856	CT	I	11898789	11901476	WBGene00012952	Y47H9C.9	Y47H9C.9	-0.115612387469815	AGIWUR19718
CB4856	CT	I	11901608	11903305	WBGene00012953	fbxa-216	Y47H9C.10	-0.100692914316882	AGIWUR18820

CB4856	CT	I	11903734	11905148	WBGene00012954	fbxa-217	Y47H9C.11	-0.253771208133251	AGIWUR28607
CB4856	CT	I	11905957	11907484	WBGene00014820	R09B3.6	R09B3.6	0.0566369958839519	AGIWUR33163;AGIWUR44264
CB4856	CT	I	11907571	11909280	WBGene00001372	exo-3	R09B3.1	-0.329506657359028	AGIWUR18555
CB4856	CT	I	11909366	11910104	WBGene00011155	R09B3.2	R09B3.2	-0.116515634114545	AGIWUR1991;AGIWUR2354;AGIWUR5088;AGIWUR17198;AGIWUR19184;AGIWUR19657;AGIWUR32430;AGIWUR32996;AGIWUR36348;AGIWUR38502
CB4856	CT	I	11910328	11911021	WBGene00011156	R09B3.3	R09B3.3	-0.0732003377033443	AGIWUR19760
CB4856	CT	I	11911324	11912052	WBGene00006707	ubc-12	R09B3.4	-0.279423829464525	AGIWUR27816
CB4856	CT	I	11912791	11917719	WBGene00001836	hda-3	R06C1.1	-0.0202952015535338	AGIWUR26718
CB4856	CT	I	11917834	11923915	WBGene00011058	fdps-1	R06C1.2	-0.00685518677132357	AGIWUR5488
CB4856	CT	I	11924785	11929511	WBGene00006958	wve-1	R06C1.3	-0.159600913136368	AGIWUR2816;AGIWUR28617;AGIWUR38538
CB4856	CT	I	11930704	11931757	WBGene00011059	R06C1.4	R06C1.4	-0.163209839566765	AGIWUR13265;AGIWUR15867;AGIWUR18035;AGIWUR19895;AGIWUR36452;AGIWUR40223
CB4856	CT	I	11947001	11953126	WBGene00011060	R06C1.6	R06C1.6	0.298522597009535	AGIWUR16528
CB4856	CT	I	11953512	11961984	WBGene00002004	hsf-1	Y53C10A.12	-0.113720764623167	AGIWUR5003
CB4856	CT	I	11966271	11967591	WBGene00014915	Y53C10A.1	Y53C10A.1	-0.130210399810029	AGIWUR3407;AGIWUR13951;AGIWUR27588;AGIWUR39138;AGIWUR40433
CB4856	CT	I	11968022	11969847	WBGene00013134	hsf-2	Y53C10A.3	-0.0855745106183554	AGIWUR14077;AGIWUR30255
CB4856	CT	I	11971179	11971797	WBGene00044805	Y53C10A.15	Y53C10A.15	-0.0985896667421944	AGIWUR5972;AGIWUR10075;AGIWUR17097;AGIWUR25230;AGIWUR41924
CB4856	CT	I	11979401	11985612	WBGene00013135	rga-2	Y53C10A.4	0.650139201797206	AGIWUR7868
CB4856	CT	I	11998781	12002643	WBGene00013136	Y53C10A.5	Y53C10A.5	-0.0328701259408088	AGIWUR35184
CB4856	CT	I	12002552	12004997	WBGene00013137	Y53C10A.6	Y53C10A.6	-0.0945753562562111	AGIWUR28508
CB4856	CT	I	12005177	12006296	WBGene00014916	fbxa-124	Y53C10A.7	-0.0957968501670852	AGIWUR32469

CB4856	CT	I	12006911	12008040	WBGene00014917	fbxa-122	Y53C10A.13	0.0190573768860283	AGIWUR4146;AGIWUR13689;AGIWUR26279;AGIWUR27755;AGIWUR38162
CB4856	CT	I	12008625	12009625	WBGene00014918	fbxa-123	Y53C10A.14	-0.434947258685556	AGIWUR4517
CB4856	CT	I	12011195	12018668	WBGene00000023	abt-5	Y53C10A.9	0.264318979084372	AGIWUR9468
CB4856	CT	I	12020756	12028313	WBGene00013138	Y53C10A.10	Y53C10A.10	-0.173575439226553	AGIWUR3846;AGIWUR7803;AGIWUR19726;AGIWUR30908;AGIWUR33643;AGIWUR37169;AGIWUR42595
CB4856	CT	I	12029927	12031762	WBGene00010247	F58D5.8	F58D5.8	0.00213226519735521	AGIWUR33736
CB4856	CT	I	12032289	12033706	WBGene00010246	F58D5.7	F58D5.7	-0.0865047297804615	AGIWUR18434
CB4856	CT	I	12034072	12037230	WBGene00010245	F58D5.6	F58D5.6	0.253604596922878	AGIWUR31967
CB4856	CT	I	12041035	12045200	WBGene00010244	F58D5.5	F58D5.5	0.135599689044094	AGIWUR541;AGIWUR6183;AGIWUR18314;AGIWUR21111;AGIWUR28012
CB4856	CT	I	12045201	12046617	WBGene00010248	F58D5.9	F58D5.9	0.1394939574662	AGIWUR21103
CB4856	CT	I	12047469	12059798	WBGene00002240	ksr-2	F58D5.4	0.027564299005105	AGIWUR8113
CB4856	CT	I	12060271	12064897	WBGene00002000	hrp-2	F58D5.1	0.429922034335371	AGIWUR10039
CB4856	CT	I	12066010	12068668	WBGene00010242	F58D5.2	F58D5.2	0.105463082309392	AGIWUR41355
CB4856	CT	I	12069806	12072878	WBGene00010243	F58D5.3	F58D5.3	-0.0247703723476267	AGIWUR42289
CB4856	CT	I	12084721	12087830	WBGene00012958	Y47H10A.2	Y47H10A.2	0.156681954108164	AGIWUR9810;AGIWUR11617;AGIWUR34817
CB4856	CT	I	12088120	12089969	WBGene00012959	Y47H10A.3	Y47H10A.3	0.446854777817085	AGIWUR19345
CB4856	CT	I	12091571	12094740	WBGene00012960	Y47H10A.4	Y47H10A.4	-0.0144240165853162	AGIWUR7903
CB4856	CT	I	12095777	12097987	WBGene00012961	Y47H10A.5	Y47H10A.5	0.554707981229863	AGIWUR33342
CB4856	CT	I	12098671	12106112	WBGene00004509	rrf-2	M01G12.12	0.304190584935153	AGIWUR44168
CB4856	CT	I	12106912	12108503	WBGene00010825	M01G12.14	M01G12.14	0.407811152429301	AGIWUR13895;AGIWUR22435
CB4856	CT	I	12109096	12112505	WBGene00010822	M01G12.9	M01G12.9		

-0.461924542855879	AGIWUR10130;AGIWUR16412;AGIWUR27321
CB4856	CT	I	12114064	12116304	WBGene00010821	M01G12.7	M01G12.7
0.251158400737308	AGIWUR27585
CB4856	CT	I	12117997	12122282	WBGene00010820	M01G12.6	M01G12.6
0.111056201075913	AGIWUR15151
CB4856	CT	I	12122811	12125586	WBGene00005858	srw-111 M01G12.4
-0.0500796107153274	AGIWUR20447
CB4856	CT	I	12126455	12128965	WBGene00010818	srt-21	M01G12.3
0.0599237738220788	AGIWUR8394
CB4856	CT	I	12144007	12146977	WBGene00005529	sri-17	F15H9.3 0.213613578405649
AGIWUR6764;AGIWUR41347
CB4856	CT	I	12148080	12150741	WBGene00005528	sri-16	F15H9.4 0.173426755797498
AGIWUR44834
CB4856	CT	I	12151021	12154333	WBGene00005527	sri-15	F15H9.2 0.103307145947786
AGIWUR8549
CB4856	CT	I	12157151	12158712	WBGene00008870	F15H9.1 F15H9.1 0.0349666691442909
AGIWUR434;AGIWUR33445
CB4856	CT	I	12160100	12162091	WBGene00077535	F15H9.7 F15H9.7 -0.0772369301719578
AGIWUR16743;AGIWUR44656
CB4856	CT	I	12162399	12163703	WBGene00011026	R05D7.1 R05D7.1 -0.351654406918152
AGIWUR27624
CB4856	CT	I	12165729	12168022	WBGene00011027	R05D7.2 R05D7.2 -0.113711054433969
AGIWUR10353;AGIWUR10361;AGIWUR19900
CB4856	CT	I	12168238	12177782	WBGene00011028	R05D7.3 R05D7.3 0.179045632593249
AGIWUR20284
CB4856	CT	I	12181046	12183297	WBGene00011029	abhd-11.2	R05D7.4
0.144092213942526	AGIWUR12358
CB4856	CT	I	12183688	12189007	WBGene00011030	R05D7.5 R05D7.5 -0.0740726978404336
AGIWUR7362
CB4856	CT	I	12189521	12190218	WBGene00077559	R05D7.7 R05D7.7 -0.145432569484885
AGIWUR3082;AGIWUR11879;AGIWUR19486;AGIWUR35942;AGIWUR38583
CB4856	CT	I	12202232	12203717	WBGene00007600	C15C6.1 C15C6.1 -0.113436296396499
AGIWUR33988
CB4856	CT	I	12203800	12205809	WBGene00007601	C15C6.2 C15C6.2 0.251796492539699
AGIWUR7410;AGIWUR19117
CB4856	CT	I	12207097	12212206	WBGene00007602	C15C6.3 C15C6.3 -0.224179510972412
AGIWUR13087
CB4856	CT	I	12213109	12215184	WBGene00007603	C15C6.4 C15C6.4 -0.20766016013728
AGIWUR2360

CB4856 CT I 12218433 12222298 WBGene00008783 F14B6.2 F14B6.2 0.0296053567942938
AGIWUR18283
CB4856 CT I 12230418 12232303 WBGene00008784 F14B6.3 F14B6.3 0.0379332449004508
AGIWUR26961
CB4856 CT I 12232689 12235193 WBGene00008787 F14B6.6 F14B6.6 -0.146793204401037
AGIWUR15381
CB4856 CT I 12236018 12238252 WBGene00008785 F14B6.4 F14B6.4 0.0976321829287476
AGIWUR38387
CB4856 CT I 12239160 12241808 WBGene00008786 oac-16 F14B6.5 -0.0695658999238473
AGIWUR42049
CB4856 CT I 12245456 12246751 WBGene00003672 nhr-82 F41D3.1 -0.811390013244522
AGIWUR30564
CB4856 CT I 12247771 12250210 WBGene00009607 oac-26 F41D3.2 0.0528660637139007
AGIWUR21424
CB4856 CT I 12251906 12253554 WBGene00009608 nhr-265 F41D3.3 -0.169389277437932
AGIWUR20066
CB4856 CT I 12254506 12257180 WBGene00009609 oac-27 F41D3.4 -0.0671761556831347
AGIWUR10798;AGIWUR12390;AGIWUR20094;AGIWUR38069;AGIWUR43226
CB4856 CT I 12264521 12266267 WBGene00009611 F41D3.6 F41D3.6 -0.122337411264632
AGIWUR37864
CB4856 CT I 12267045 12267491 WBGene00009612 F41D3.7 F41D3.7 0.125619646265072
AGIWUR35359
CB4856 CT I 12267499 12268128 WBGene00009613 F41D3.8 F41D3.8 -0.401212668606602
AGIWUR42139
CB4856 CT I 12272981 12274636 WBGene00009614 F41D3.9 F41D3.9 -0.393309799010649
AGIWUR39869
CB4856 CT I 12276893 12278775 WBGene00009616 F41D3.11 F41D3.11
-0.0139100404487209 AGIWUR44485
CB4856 CT I 12281221 12282615 WBGene00010162 F56H6.1 F56H6.1 0.0229113850040967
AGIWUR18565
CB4856 CT I 12283270 12285384 WBGene00010173 F56H6.13 F56H6.13
-0.0196503389922063 AGIWUR13846
CB4856 CT I 12287199 12289307 WBGene00010163 F56H6.2 F56H6.2 -0.248126572596535
AGIWUR39362
CB4856 CT I 12289552 12290191 WBGene00010164 F56H6.3 F56H6.3 0.0238713043270344
AGIWUR16604
CB4856 CT I 12290449 12292212 WBGene00010165 F56H6.4 F56H6.4 0.168453725799809
AGIWUR18750
CB4856 CT I 12292269 12293936 WBGene00010166 gmd-2 F56H6.5 0.532906209282479

AGIWUR7165
CB4856 CT I 12294380 12296263 WBGene00010167 bgnt-1.6 F56H6.6
-0.243575043033808 AGIWUR963
CB4856 CT I 12297734 12300817 WBGene00010168 F56H6.7 F56H6.7 0.729583195897479
AGIWUR17051
CB4856 CT I 12301424 12303157 WBGene00010169 clec-18 F56H6.8
-0.00286634468391345 AGIWUR41003
CB4856 CT I 12303599 12306820 WBGene00010170 F56H6.9 F56H6.9 0.255731654997862
AGIWUR1010;AGIWUR14730
CB4856 CT I 12307731 12310406 WBGene00010171 oac-35 F56H6.11
0.522871669664356 AGIWUR41513
CB4856 CT I 12312335 12314895 WBGene00010172 oac-36 F56H6.12
0.644310123727334 AGIWUR24225;AGIWUR26226;AGIWUR40420
CB4856 CT I 12315193 12316688 WBGene00008159 C47F8.3 C47F8.3 0.00350877372818026
AGIWUR13904
CB4856 CT I 12317321 12318735 WBGene00008160 glct-4 C47F8.4 0.559759113631349
AGIWUR15638
CB4856 CT I 12319350 12320854 WBGene00008162 C47F8.6 C47F8.6 0.712067163252534
AGIWUR31086
CB4856 CT I 12321937 12324062 WBGene00008161 C47F8.5 C47F8.5 -0.0877118823601739
AGIWUR37700
CB4856 CT I 12326441 12327030 WBGene00008163 C47F8.7 C47F8.7 0.0896788991473141
AGIWUR4906
CB4856 CT I 12328100 12329508 WBGene00003671 nhr-81 C47F8.8 0.974533811229529
AGIWUR10579;AGIWUR25388;AGIWUR39749
CB4856 CT I 12331085 12333413 WBGene00008158 nhr-165 C47F8.2 0.810495011284606
AGIWUR37607
CB4856 CT I 12335035 12336605 WBGene00008157 C47F8.1 C47F8.1 -0.202310467544722
AGIWUR45
CB4856 CT I 12337968 12339239 WBGene00011650 glct-1 T09E11.1
-0.0567728007025377 AGIWUR41546
CB4856 CT I 12341145 12342826 WBGene00011651 nhr-217 T09E11.2
-0.0467783359707532 AGIWUR38513
CB4856 CT I 12344167 12345702 WBGene00011660 T09E11.11 T09E11.11
2.01517181131606 AGIWUR9231
CB4856 CT I 12345819 12347265 WBGene00011659 T09E11.10 T09E11.10
0.306646205029448 AGIWUR9137;AGIWUR26055;AGIWUR36895
CB4856 CT I 12347700 12349819 WBGene00011658 T09E11.9 T09E11.9
0.0639729161453175 AGIWUR36326

CB4856	CT	I	12350692	12352724	WBGene00011657	T09E11.8	T09E11.8	0.130782737740363	AGIWUR32745
CB4856	CT	I	12353506	12356337	WBGene00011656	oac-45	T09E11.7	0.748977512438973	AGIWUR34978
CB4856	CT	I	12356942	12359341	WBGene00011654	oac-44	T09E11.5	0.544063531686849	AGIWUR16338;AGIWUR30786
CB4856	CT	I	12359942	12363416	WBGene00011653	oac-43	T09E11.4	0.0184777419185265	AGIWUR1706
CB4856	CT	I	12364718	12365948	WBGene00045103	T09E11.12	T09E11.12	0.463786081944525	AGIWUR13986;AGIWUR27610
CB4856	CT	I	12366335	12368619	WBGene00011655	T09E11.6	T09E11.6	0.0945272088870244	AGIWUR2283;AGIWUR21346;AGIWUR43303
CB4856	CT	I	12374205	12376649	WBGene00011779	bgnt-1.7	T15D6.4	-0.245742133495401	AGIWUR15806;AGIWUR17941
CB4856	CT	I	12376904	12378674	WBGene00001642	gly-17	T15D6.3	-0.070649047711635	AGIWUR4623
CB4856	CT	I	12379335	12381032	WBGene00001641	gly-16	T15D6.2	-0.179967048576367	AGIWUR19223
CB4856	CT	I	12381808	12383259	WBGene00011780	T15D6.5	T15D6.5	-0.0685356210330443	AGIWUR15742
CB4856	CT	I	12384277	12385731	WBGene00003667	nhr-77	T15D6.6	-0.123298920114302	AGIWUR37750
CB4856	CT	I	12385766	12388791	WBGene00011781	glct-3	T15D6.7	0.0333116857530058	AGIWUR2441
CB4856	CT	I	12392009	12393585	WBGene00011783	T15D6.9	T15D6.9	0.119660837637976	AGIWUR21864
CB4856	CT	I	12394120	12396322	WBGene00011784	T15D6.10	T15D6.10	0.153386988012871	AGIWUR795
CB4856	CT	I	12396644	12398267	WBGene00011785	T15D6.11	T15D6.11	0.708365833627942	AGIWUR40226
CB4856	CT	I	12399323	12401206	WBGene00011786	T15D6.12	T15D6.12	0.44251184959449	AGIWUR7270
CB4856	CT	I	12403110	12404489	WBGene00008470	E03H4.2	E03H4.2	0.167456642202371	AGIWUR40136
CB4856	CT	I	12405525	12406946	WBGene00008471	E03H4.3	E03H4.3	0.316266467060151	AGIWUR39310
CB4856	CT	I	12407564	12411435	WBGene00008472	E03H4.4	E03H4.4	0.362786873188178	AGIWUR38190
CB4856	CT	I	12413809	12415745	WBGene00008473	E03H4.5	E03H4.5	0.237933896412622	

AGIWUR5938
CB4856 CT I 12417386 12418800 WBGene00008474 nhr-174 E03H4.6 0.135045633173321
AGIWUR19291
CB4856 CT I 12421239 12424411 WBGene00008475 oac-13 E03H4.7 0.0449311007842113
AGIWUR4799
CB4856 CT I 12424555 12426850 WBGene00008476 E03H4.8 E03H4.8 -0.101756587445055
AGIWUR40478
CB4856 CT I 12427388 12429094 WBGene00014729 E03H4.9 E03H4.9 0.194729076495917
AGIWUR6347;AGIWUR40147
CB4856 CT I 12430028 12431500 WBGene00008477 clec-17 E03H4.10
1.45743408170223 AGIWUR22801
CB4856 CT I 12433061 12434886 WBGene00008478 E03H4.11 E03H4.11
0.483512216387402 AGIWUR5267
CB4856 CT I 12435726 12437285 WBGene00008479 glct-2 E03H4.12
0.121686063616135 AGIWUR34058
CB4856 CT I 12439936 12441253 WBGene00003679 nhr-89 E03H4.13
-0.020441708036242 AGIWUR39890
CB4856 CT I 12442299 12443728 WBGene00008289 nhr-169 C54C8.1 -0.143943419259516
AGIWUR43082
CB4856 CT I 12444587 12446502 WBGene00008290 bgnt-1.8 C54C8.2
0.335970970163281 AGIWUR19147
CB4856 CT I 12447167 12448421 WBGene00008291 C54C8.3 C54C8.3 0.0264887460150369
AGIWUR7375
CB4856 CT I 12449977 12452027 WBGene00008292 C54C8.4 C54C8.4 0.797436818699401
AGIWUR35933
CB4856 CT I 12453389 12454764 WBGene00008293 glct-5 C54C8.5 -0.494863017601157
AGIWUR25504
CB4856 CT I 12454853 12455826 WBGene00003098 lys-9 C54C8.6 0.254781158545948
AGIWUR2072
CB4856 CT I 12456918 12457209 WBGene00044242 C54C8.12 C54C8.12
3.19259183115844 AGIWUR19622;AGIWUR21351;AGIWUR24934;AGIWUR29563;AGIWUR35527
CB4856 CT I 12458028 12458996 WBGene00014714 C54C8.8 C54C8.8 0.310195640918717
AGIWUR2522
CB4856 CT I 12459499 12460903 WBGene00008294 clec-11 C54C8.7 0.23346194366955
AGIWUR6618;AGIWUR36802
CB4856 CT I 12461645 12461925 WBGene00008295 nlp-39 C54C8.9 4.531731879701
AGIWUR2285
CB4856 CT I 12462176 12465831 WBGene00001640 gly-15 C54C8.11
0.173147258604664 AGIWUR4226;AGIWUR19199;AGIWUR41172

CB4856	CT	I	12470620	12473404	WBGene00012102	T27F6.1	T27F6.1	-0.172097593747891	AGIWUR6133
CB4856	CT	I	12474620	12475934	WBGene00077508	T27F6.10	T27F6.10	-0.0497800151804473	AGIWUR40057
CB4856	CT	I	12476685	12478588	WBGene00012103	clec-12	T27F6.2	0.0524483847124859	AGIWUR32740
CB4856	CT	I	12483809	12486235	WBGene00012104	sygl-1	T27F6.4	-0.0644247875989024	AGIWUR43852
CB4856	CT	I	12486872	12490931	WBGene00004190	pars-2	T27F6.5	-0.189148516750583	AGIWUR1856;AGIWUR2356;AGIWUR8002;AGIWUR8434;AGIWUR11790;AGIWUR11802;AGIWUR15588;AGIWUR16087;AGIWUR28139;AGIWUR29090;AGIWUR41244;AGIWUR43781;AGIWUR45063
CB4856	CT	I	12490976	12494530	WBGene00012105	T27F6.6	T27F6.6	-0.380360232846385	AGIWUR3497
CB4856	CT	I	12494826	12497354	WBGene00012106	T27F6.7	T27F6.7	0.0217399988292435	AGIWUR36447
CB4856	CT	I	12497467	12505010	WBGene00010768	K11D2.1	K11D2.1	-0.0710158825098501	AGIWUR17267;AGIWUR40837
CB4856	CT	I	12505028	12508064	WBGene00010769	asah-1	K11D2.2	1.30208799377506	AGIWUR37730
CB4856	CT	I	12508299	12513419	WBGene00006829	unc-101	K11D2.3	-0.246856772298813	AGIWUR417;AGIWUR2271;AGIWUR7234;AGIWUR18404;AGIWUR20120;AGIWUR22240;AGIWUR26389;AGIWUR38381;AGIWUR40794
CB4856	CT	I	12513516	12518694	WBGene00010771	K11D2.5	K11D2.5	0.0594687980044384	AGIWUR11668
CB4856	CT	I	12518787	12527906	WBGene00010770	K11D2.4	K11D2.4	-0.268455967581212	AGIWUR1842
CB4856	CT	I	12528312	12532182	WBGene00004181	pri-2	W02D9.1	0.0329283549074226	AGIWUR3305
CB4856	CT	I	12532669	12534400	WBGene00012208	W02D9.2	W02D9.2	0.310810691286946	AGIWUR32177
CB4856	CT	I	12535312	12537671	WBGene00012209	hmg-20	W02D9.3	-0.176321859933061	AGIWUR2684;AGIWUR36464
CB4856	CT	I	12551537	12552375	WBGene00012216	W02D9.10	W02D9.10	0.829530721833958	AGIWUR8060
CB4856	CT	I	12557164	12557563	WBGene00012215	W02D9.9	W02D9.9	-0.257141654993621	AGIWUR19044
CB4856	CT	I	12558547	12558944	WBGene00012214	W02D9.8	W02D9.8	0.159472240843774	AGIWUR38870
CB4856	CT	I	12559245	12559674	WBGene00012213	W02D9.7	W02D9.7	-0.0934945037684187	AGIWUR19286

CB4856 CT I 12560292 12560714 WBGene00012212 W02D9.6 W02D9.6 -0.023717361465895
AGIWUR5048
CB4856 CT I 12561040 12561560 WBGene00012211 ssp-37 W02D9.5 0.195740818261074
AGIWUR14307
CB4856 CT I 12562756 12564210 WBGene00045474 C49G9.2 C49G9.2 -0.433732158897236
AGIWUR20355;AGIWUR23937;AGIWUR31109;AGIWUR35803;AGIWUR35835
CB4856 CT I 12564356 12565893 WBGene00008216 mec-19 C49G9.1 -0.432289810580198
AGIWUR41066
CB4856 CT I 12565804 12572801 WBGene00001008 dlk-1 F33E2.2 0.00125461468172938
AGIWUR40022;AGIWUR42874
CB4856 CT I 12578108 12579205 WBGene00077769 F33E2.10 F33E2.10
0.0298527708891498 AGIWUR5546
CB4856 CT I 12582074 12584711 WBGene00009360 F33E2.4 F33E2.4 -0.168958071517721
AGIWUR43140
CB4856 CT I 12583786 12585119 WBGene00045254 F33E2.8 F33E2.8 0.372280539749473
AGIWUR28003;AGIWUR30710
CB4856 CT I 12585382 12585971 WBGene00009363 F33E2.7 F33E2.7 -0.0329542377554555
AGIWUR16591
CB4856 CT I 12587242 12591091 WBGene00009362 F33E2.6 F33E2.6 0.048670846245777
AGIWUR22599
CB4856 CT I 12591116 12597442 WBGene00009361 F33E2.5 F33E2.5 0.387274238092093
AGIWUR2198;AGIWUR32087;AGIWUR32401
CB4856 CT I 12603772 12606248 WBGene00011581 T07D10.1 T07D10.1
0.233341196929074 AGIWUR33134
CB4856 CT I 12621171 12623084 WBGene00011582 ntr-1 T07D10.2
-0.167310022701932 AGIWUR20900
CB4856 CT I 12624235 12625238 WBGene00011586 flp-33 T07D10.6
0.665975605050637 AGIWUR26500
CB4856 CT I 12626742 12628997 WBGene00011583 T07D10.3 T07D10.3
0.541231827217887 AGIWUR36305
CB4856 CT I 12640720 12641501 WBGene00195255 H16D19.7 H16D19.7
-0.0865547173410945 AGIWUR43884
CB4856 CT I 12647376 12652087 WBGene00010420 mcrs-1 H28O16.2
-0.139510239744897 AGIWUR40821
CB4856 CT I 12652277 12654646 WBGene00010419 H28O16.1 H28O16.1
-0.0144541185824192
AGIWUR1002;AGIWUR6017;AGIWUR9225;AGIWUR15093;AGIWUR18400;AGIWUR19137;AGIWUR20909;AGIWUR21162;AGIWUR22474;AG
IWUR25477;AGIWUR30636;AGIWUR30684;AGIWUR33565;AGIWUR37320;AGIWUR37771;AGIWUR38251;AGIWUR40047;AGIWUR42542
CB4856 CT I 12655449 12655970 WBGene00012040 T26E3.7 T26E3.7 -0.0729272518537325

AGIWUR36996
CB4856 CT I 12657762 12659446 WBGene00012041 T26E3.8 T26E3.8 -0.206622631419782
AGIWUR37661
CB4856 CT I 12659729 12660647 WBGene00044905 T26E3.11 T26E3.11
0.0808058749386937 AGIWUR41658
CB4856 CT I 12661254 12661729 WBGene00012039 T26E3.6 T26E3.6 -0.0797928785089344
AGIWUR36512
CB4856 CT I 12662938 12663833 WBGene00012038 T26E3.5 T26E3.5 0.154544814954296
AGIWUR1201
CB4856 CT I 12665067 12668893 WBGene00012037 T26E3.4 T26E3.4 -0.156863491048281
AGIWUR2560;AGIWUR11531;AGIWUR28485;AGIWUR30882;AGIWUR32370;AGIWUR41162
CB4856 CT I 12669380 12674447 WBGene00003921 par-6 T26E3.3 -0.0498621659667897
AGIWUR4153;AGIWUR21135;AGIWUR24877
CB4856 CT I 12679207 12681816 WBGene00003578 ndx-1 T26E3.2 0.202633415110892
AGIWUR36323
CB4856 CT I 12681883 12682681 WBGene00012042 T26E3.10 T26E3.10
-0.167541029547844 AGIWUR2023
CB4856 CT I 12683238 12684359 WBGene00012036 clec-103 T26E3.1
-0.22865147732205 AGIWUR36031
CB4856 CT I 12686116 12688206 WBGene00005051 sra-25 T26E3.9 -0.0478292978855723
AGIWUR23081
CB4856 CT I 12688727 12690572 WBGene00005043 sra-17 F28C12.1
-0.205997978339377 AGIWUR103
CB4856 CT I 12692169 12693724 WBGene00005044 sra-18 F28C12.2
0.156688185815693 AGIWUR40855
CB4856 CT I 12694406 12695934 WBGene00005045 sra-19 F28C12.3
-0.0793514532749864 AGIWUR2298;AGIWUR5976;AGIWUR10097;AGIWUR12389;AGIWUR26934;AGIWUR30505;AGIWUR39618
CB4856 CT I 12696558 12698214 WBGene00005046 sra-20 F28C12.4
0.207631704879591 AGIWUR24117
CB4856 CT I 12698904 12699200 WBGene00077555 F28C12.8 F28C12.8
-0.0438523639524115 AGIWUR4271;AGIWUR9750;AGIWUR13760;AGIWUR22255;AGIWUR25005
CB4856 CT I 12699430 12701093 WBGene00005047 sra-21 F28C12.5
-0.179393491846798 AGIWUR44687
CB4856 CT I 12701439 12702886 WBGene00005048 sra-22 F28C12.7
-0.0546058840902131 AGIWUR34243
CB4856 CT I 12703435 12704727 WBGene00005049 sra-23 T06G6.1 -0.0798042516471771
AGIWUR6499
CB4856 CT I 12705097 12706983 WBGene00005050 sra-24 T06G6.2 -0.0137383833305345
AGIWUR44570

CB4856	CT	I	12707513	12708691	WBGene00011550	T06G6.11	T06G6.11	-0.135963228555717	AGIWUR6450
CB4856	CT	I	12708863	12711596	WBGene00011545	T06G6.3	T06G6.3	-0.0962604765817954	AGIWUR36658
CB4856	CT	I	12712158	12714722	WBGene00011546	T06G6.4	T06G6.4	-0.177442548642704	AGIWUR7160
CB4856	CT	I	12715648	12716583	WBGene00011547	T06G6.5	T06G6.5	0.206687611610455	AGIWUR30650
CB4856	CT	I	12718296	12719795	WBGene00011548	T06G6.6	T06G6.6	-0.749475902059697	AGIWUR17689
CB4856	CT	I	12720205	12722036	WBGene00005835	srw-88	T06G6.7	-0.167675365588426	AGIWUR1507
CB4856	CT	I	12722045	12722883	WBGene00011551	T06G6.12	T06G6.12	0.0529060396383993	AGIWUR32980
CB4856	CT	I	12724751	12726206	WBGene00011549	T06G6.8	T06G6.8	-0.195522604693216	AGIWUR34628
CB4856	CT	I	12728137	12729870	WBGene00006889	pfd-3	T06G6.9	-0.236904682238132	AGIWUR1885;AGIWUR2341;AGIWUR7063;AGIWUR17219;AGIWUR19489;AGIWUR31484
CB4856	CT	I	12730011	12732753	WBGene00012192	W02A11.1	W02A11.1	0.0204776625130947	AGIWUR8401
CB4856	CT	I	12733287	12741275	WBGene00012193	vps-25	W02A11.2	-0.208127801682362	AGIWUR17203
CB4856	CT	I	12741588	12749318	WBGene00006700	uba-2	W02A11.4	-0.297037897881072	AGIWUR31511
CB4856	CT	I	12749890	12752087	WBGene00012195	bath-34	W02A11.5	-0.0967294462795176	AGIWUR9884;AGIWUR28264
CB4856	CT	I	12758433	12759572	WBGene00012196	bath-35	W02A11.8	0.150620386311925	AGIWUR37753
CB4856	CT	I	12759545	12764960	WBGene00000138	amx-2	B0019.1	0.0417397211766044	AGIWUR19037
CB4856	CT	I	12773659	12782282	WBGene00007094	B0019.2	B0019.2	0.191653350982253	AGIWUR9959;AGIWUR22906
CB4856	CT	I	12783358	12784978	WBGene00023459	Y18D10A.26	Y18D10A.26	0.319353750247379	AGIWUR24786;AGIWUR33759
CB4856	CT	I	12787944	12806581	WBGene00012474	attf-6	Y18D10A.1	0.0786624673657315	AGIWUR42137
CB4856	CT	I	12807586	12809682	WBGene00012475	Y18D10A.2	Y18D10A.2	-0.043344212766927	AGIWUR13443
CB4856	CT	I	12809999	12815641	WBGene00012476	Y18D10A.3	Y18D10A.3		

-0.143534200373995 AGIWUR36104
CB4856 CT I 12815904 12816428 WBGene00012477 Y18D10A.4 Y18D10A.4
-0.0188612054188511 AGIWUR2617
CB4856 CT I 12819216 12825562 WBGene00001746 gsk-3 Y18D10A.5
-0.16148552868218 AGIWUR44507
CB4856 CT I 12825664 12834143 WBGene00003735 nhx-8 Y18D10A.6
-0.351377997843757 AGIWUR10024;AGIWUR18724;AGIWUR35964
CB4856 CT I 12834665 12843233 WBGene00004231 ptr-17 Y18D10A.7
0.00088960883762379 AGIWUR2500;AGIWUR17127;AGIWUR35830;AGIWUR38073
CB4856 CT I 12844269 12853105 WBGene00012478 mxt-1 Y18D10A.8
0.0360082050056896 AGIWUR4070
CB4856 CT I 12853088 12858272 WBGene00012479 Y18D10A.9 Y18D10A.9
-0.0800431974225635 AGIWUR18598
CB4856 CT I 12859499 12861197 WBGene00012480 clec-104 Y18D10A.10
-0.438655613472344 AGIWUR26490
CB4856 CT I 12861951 12863544 WBGene00012481 Y18D10A.11 Y18D10A.11
-0.105570432947795 AGIWUR18588
CB4856 CT I 12864230 12865568 WBGene00012488 clec-105 Y18D10A.24
-0.276607302304612 AGIWUR22133
CB4856 CT I 12866120 12866896 WBGene00012482 clec-106 Y18D10A.12
-0.606102306680178 AGIWUR40586
CB4856 CT I 12866991 12869556 WBGene00012487 Y18D10A.23 Y18D10A.23
0.114593997474621 AGIWUR21563
CB4856 CT I 12878595 12902806 WBGene00003905 pad-1 Y18D10A.13
0.0414342873498335 AGIWUR16264
CB4856 CT I 12903059 12904072 WBGene00012483 Y18D10A.16 Y18D10A.16
-0.053731585745096 AGIWUR10347;AGIWUR19850;AGIWUR33702
CB4856 CT I 12904470 12906212 WBGene00012484 car-1 Y18D10A.17
-0.0366652106912655 AGIWUR9634;AGIWUR11573;AGIWUR13065;AGIWUR30240;AGIWUR35163;AGIWUR41272
CB4856 CT I 12906462 12907754 WBGene00012485 fbxa-121 Y18D10A.18
0.0875111134666895 AGIWUR33138
CB4856 CT I 12908304 12909436 WBGene00077556 Y18D10A.27 Y18D10A.27
0.101874904226078 AGIWUR5790;AGIWUR7354;AGIWUR9630
CB4856 CT I 12916441 12918108 WBGene00001433 fkb-8 Y18D10A.25
-0.113421887172871 AGIWUR3419;AGIWUR11778
CB4856 CT I 12918722 12919278 WBGene00001427 fkb-2 Y18D10A.19
-0.115981811055168 AGIWUR1517;AGIWUR18093;AGIWUR43780
CB4856 CT I 12922004 12923548 WBGene00003989 pfn-1 Y18D10A.20
-0.258648900759659 AGIWUR749;AGIWUR10648;AGIWUR13965;AGIWUR14413;AGIWUR19323;AGIWUR22987

CB4856	CT	I	12929372	12931069	WBGene00012486	Y18D10A.21	Y18D10A.21	-0.350867137532958	AGIWUR15932
CB4856	CT	I	12932037	12933751	WBGene00014864	Y18D10A.22	Y18D10A.22	0.225853236396162	AGIWUR28353
CB4856	CT	I	12934767	12937252	WBGene00006484	tag-138	F08A8.6	0.101784753073133	AGIWUR2434
CB4856	CT	I	12938555	12942224	WBGene00008564	acox-1.1	F08A8.1	-0.130475574618449	AGIWUR34581
CB4856	CT	I	12942280	12946562	WBGene00008565	acox-1.2	F08A8.2	-0.351479122481448	AGIWUR825
CB4856	CT	I	12951025	12954015	WBGene00008567	acox-1.4	F08A8.4	-0.186557025165775	AGIWUR1174;AGIWUR16821
CB4856	CT	I	12953969	12955322	WBGene00045468	F08A8.8	F08A8.8	-0.301478788725285	AGIWUR441
CB4856	CT	I	12955328	12957902	WBGene00008568	F08A8.5	F08A8.5	0.0274673368921885	AGIWUR26122
CB4856	CT	I	12958639	12959955	WBGene00008569	fbxa-101	F08A8.7	-0.0601564514103163	AGIWUR11695
CB4856	CT	I	12960313	12961606	WBGene00008130	fbxa-140	C47B2.1	-0.0764238276177913	AGIWUR4596
CB4856	CT	I	12962042	12964579	WBGene00008131	C47B2.2	C47B2.2	-0.253807718533238	AGIWUR30169
CB4856	CT	I	12964674	12966876	WBGene00006529	tba-2	C47B2.3	-0.00189258843614137	AGIWUR4560;AGIWUR7861;AGIWUR17254;AGIWUR33286
CB4856	CT	I	12967216	12968280	WBGene00003948	pbs-2	C47B2.4	-0.074487256964078	AGIWUR41108
CB4856	CT	I	12968921	12970238	WBGene00001234	eif-6	C47B2.5	-0.126679972255074	AGIWUR1942;AGIWUR2030;AGIWUR4142;AGIWUR21916;AGIWUR24490
CB4856	CT	I	12971120	12972347	WBGene00004196	prx-11	C47B2.8	0.15225504435945	AGIWUR1599
CB4856	CT	I	12975183	12978749	WBGene00008132	gale-1	C47B2.6	-0.00611099397985715	AGIWUR10738;AGIWUR34894
CB4856	CT	I	12978839	12982452	WBGene00008133	efsc-1	C47B2.7	0.153073540441405	AGIWUR34915
CB4856	CT	I	12983004	12983974	WBGene00008134	C47B2.9	C47B2.9	-0.0346726278294458	AGIWUR36090
CB4856	CT	I	12988223	12995475	WBGene00003753	nlp-15	CC4.2	0.346252132073609	AGIWUR1711
CB4856	CT	I	12995803	13012859	WBGene00004895	smu-1	CC4.3	-0.129732636399428	

AGIWUR14067
CB4856 CT I 13004437 13023625 WBGene00004063 pmr-1 ZK256.1 0.0923972034437259
AGIWUR12760;AGIWUR19470;AGIWUR21490;AGIWUR37538
CB4856 CT I 13033483 13045555 WBGene00012274 W05B5.1 W05B5.1 0.0865108647328365
AGIWUR130;AGIWUR13029;AGIWUR16053;AGIWUR21284;AGIWUR34592;AGIWUR42290
CB4856 CT I 13050445 13057435 WBGene00012275 npr-14 W05B5.2 -0.0738800676199567
AGIWUR22861
CB4856 CT I 13066924 13069038 WBGene00012500 hpo-2 Y26D4A.2
0.247684220584606 AGIWUR24458
CB4856 CT I 13069529 13071190 WBGene00012502 clec-107 Y26D4A.4
0.14390507690095 AGIWUR8324
CB4856 CT I 13073837 13075124 WBGene00044706 Y26D4A.15 Y26D4A.15
0.0212874503332796 AGIWUR1641;AGIWUR27615
CB4856 CT I 13075122 13075381 WBGene00044710 Y26D4A.16 Y26D4A.16
-0.095234787057823 AGIWUR73
CB4856 CT I 13077994 13078880 WBGene00045235 Y26D4A.18 Y26D4A.18
-0.398886437575074 AGIWUR39678
CB4856 CT I 13079742 13080633 WBGene00045236 Y26D4A.19 Y26D4A.19
-0.0753590286867034 AGIWUR40017
CB4856 CT I 13081448 13082166 WBGene00012504 clec-108 Y26D4A.6
0.0740250840397891 AGIWUR7770
CB4856 CT I 13082681 13082966 WBGene00012509 Y26D4A.12 Y26D4A.12
0.489215517781814 AGIWUR41342
CB4856 CT I 13083321 13085505 WBGene00012505 pals-13 Y26D4A.8
-0.0649998611131926 AGIWUR17033
CB4856 CT I 13085106 13103119 WBGene00007655 C17H1.2 C17H1.2 0.139242171575823
AGIWUR9507;AGIWUR23034;AGIWUR29977;AGIWUR37866;AGIWUR42970
CB4856 CT I 13104253 13106298 WBGene00007656 pals-2 C17H1.3 1.21204841837648
AGIWUR5622;AGIWUR17946;AGIWUR35948
CB4856 CT I 13107140 13107457 WBGene00044711 pals-10 C17H1.11
0.833167950892225 AGIWUR9138;AGIWUR27537;AGIWUR32329;AGIWUR37188;AGIWUR42291
CB4856 CT I 13107492 13112443 WBGene00007657 pals-3 C17H1.4 0.801951100935907
AGIWUR37391
CB4856 CT I 13113487 13116434 WBGene00007658 pals-4 C17H1.5 0.647226746788915
AGIWUR37559
CB4856 CT I 13117629 13120324 WBGene00007654 C17H1.1 C17H1.1 0.300087322347126
AGIWUR17604;AGIWUR25487;AGIWUR28593
CB4856 CT I 13121092 13123513 WBGene00007662 pals-8 C17H1.9 1.17835217317214
AGIWUR32197

CB4856	CT	I	13124081	13126650	WBGene00044708	pals-11	C17H1.13
1.30104391016556	AGIWUR17715
CB4856	CT	I	13127152	13127876	WBGene00044722	C17H1.12	C17H1.12
0.020478359771777	AGIWUR6757;AGIWUR7071;AGIWUR11735;AGIWUR13554
CB4856	CT	I	13128470	13130409	WBGene00007661	pals-7	C17H1.8	0.894518787235526
AGIWUR41771
CB4856	CT	I	13130808	13132464	WBGene00007659	pals-5	C17H1.6	0.65482123406365
AGIWUR7049
CB4856	CT	I	13133638	13136156	WBGene00044237	pals-9	C17H1.10
2.20776642852047	AGIWUR18891
CB4856	CT	I	13136467	13138521	WBGene00007660	pals-6	C17H1.7	2.13760744786407
AGIWUR2479
CB4856	CT	I	13139132	13141789	WBGene00009061	pals-14	F22G12.1
1.83288374845099	AGIWUR8641;AGIWUR17238
CB4856	CT	I	13144912	13146453	WBGene00044712	pals-15	F22G12.8
-0.77029007185345	AGIWUR2413;AGIWUR10665;AGIWUR28917
CB4856	CT	I	13152575	13153706	WBGene00009063	F22G12.3	F22G12.3
0.208419720688018	AGIWUR38346
CB4856	CT	I	13155239	13161508	WBGene00009064	F22G12.4	F22G12.4
-0.0133071385463648	AGIWUR15762;AGIWUR18080;AGIWUR18882
CB4856	CT	I	13162492	13182725	WBGene00009065	F22G12.5	F22G12.5
-0.0129347361210037	AGIWUR21603
CB4856	CT	I	13184852	13188234	WBGene00008905	F17B5.1	F17B5.1	1.85307399699067
AGIWUR11554
CB4856	CT	I	13189792	13190583	WBGene00138716	F17B5.10	F17B5.10
-0.270759403763518	AGIWUR9777
CB4856	CT	I	13190578	13191921	WBGene00045458	F17B5.8	F17B5.8	-0.0161790420059278
AGIWUR1859;AGIWUR10263;AGIWUR21965;AGIWUR23242;AGIWUR29567
CB4856	CT	I	13197179	13198374	WBGene00008907	clec-109	F17B5.3
-0.103915207281909	AGIWUR6963;AGIWUR18308;AGIWUR20621;AGIWUR21937;AGIWUR41207
CB4856	CT	I	13199359	13200980	WBGene00008908	F17B5.4	F17B5.4	-0.112855011373654
AGIWUR11419;AGIWUR39916
CB4856	CT	I	13201710	13204293	WBGene00044058	F17B5.6	F17B5.6	0.442391425478152
AGIWUR5127
CB4856	CT	I	13204497	13205403	WBGene00008909	clec-110	F17B5.5
-0.45999729670808	AGIWUR4687;AGIWUR17223;AGIWUR17887;AGIWUR29019
CB4856	CT	I	13205664	13207759	WBGene00014235	ZK1225.1	ZK1225.1
0.310711014688418	AGIWUR129
CB4856	CT	I	13208441	13210462	WBGene00014236	ZK1225.2	ZK1225.2

0.0339858631838323	AGIWUR9579;AGIWUR13739;AGIWUR37030
CB4856	CT	I	13210585	13211352	WBGene00044713	ZK1225.9	ZK1225.9
-0.0723337050590116	AGIWUR719;AGIWUR16159;AGIWUR31446
CB4856	CT	I	13211826	13213806	WBGene00044714	ZK1225.10	ZK1225.10
-0.246670481036293	AGIWUR37508;AGIWUR38006;AGIWUR42906
CB4856	CT	I	13214095	13216230	WBGene00014238	ZK1225.4	ZK1225.4
-0.237170983226386	AGIWUR23716
CB4856	CT	I	13216605	13218867	WBGene00014239	ZK1225.5	ZK1225.5
-0.0967066191374535	AGIWUR1722
CB4856	CT	I	13219056	13219545	WBGene00006048	ssp-31	ZK1225.6
0.121955997637836	AGIWUR4043;AGIWUR11256;AGIWUR18577;AGIWUR20890;AGIWUR21770;AGIWUR43896
CB4856	CT	I	13227995	13229751	WBGene00014196	ZK1053.1	ZK1053.1
0.310056547406004	AGIWUR8882
CB4856	CT	I	13232121	13234686	WBGene00014197	ZK1053.2	ZK1053.2
0.466994915878891	AGIWUR12680
CB4856	CT	I	13235367	13236662	WBGene00014200	scrm-2	ZK1053.5
0.138385580797465	AGIWUR23919
CB4856	CT	I	13237054	13238302	WBGene00044786	ZK1053.7	ZK1053.7
0.090371684291445	AGIWUR29991;AGIWUR34385
CB4856	CT	I	13238540	13240726	WBGene00014201	ZK1053.6	ZK1053.6
-0.247173554373208	AGIWUR20160
CB4856	CT	I	13241645	13244797	WBGene00014199	ZK1053.4	ZK1053.4
0.264201168958474	AGIWUR9930
CB4856	CT	I	13246853	13249789	WBGene00014198	ZK1053.3	ZK1053.3
0.727115749260678	AGIWUR10322
CB4856	CT	I	13250519	13252902	WBGene00009693	F44F1.1	F44F1.1	-0.130426058783291
AGIWUR11569;AGIWUR12707;AGIWUR35981
CB4856	CT	I	13254141	13257729	WBGene00009695	F44F1.3	F44F1.3	-0.0241304169641623
AGIWUR36983
CB4856	CT	I	13258381	13260217	WBGene00009697	F44F1.5	F44F1.5	-0.0434240740211598
AGIWUR24484
CB4856	CT	I	13260933	13262727	WBGene00009698	F44F1.6	F44F1.6	0.433882968344873
AGIWUR2878;AGIWUR9405;AGIWUR14989;AGIWUR18297;AGIWUR19140;AGIWUR22641;AGIWUR25795;AGIWUR33450;AGIWUR43720
CB4856	CT	I	13263803	13265570	WBGene00009696	F44F1.4	F44F1.4	0.00763632335372311
AGIWUR33801
CB4856	CT	I	13269984	13272784	WBGene00006902	vet-6	F44F1.7	-0.135086603043309
AGIWUR16532
CB4856	CT	I	13274111	13276462	WBGene00010804	M01E5.1	M01E5.1	-0.0209011941066848
AGIWUR23175;AGIWUR33900;AGIWUR42322

CB4856	CT	I	13277061	13280175	WBGene00010805	M01E5.2	M01E5.2	0.0854037935624667	AGIWUR39432
CB4856	CT	I	13280892	13286497	WBGene00010806	M01E5.3	M01E5.3	0.0481914766588369	AGIWUR24647;AGIWUR27603;AGIWUR30431;AGIWUR31429
CB4856	CT	I	13287551	13289542	WBGene00010807	M01E5.4	M01E5.4	-0.104813791305279	AGIWUR20792;AGIWUR38781
CB4856	CT	I	13292506	13297555	WBGene00006595	top-1	M01E5.5	0.248936649518747	AGIWUR1777;AGIWUR15898;AGIWUR16737;AGIWUR17593;AGIWUR26904;AGIWUR32658;AGIWUR39976
CB4856	CT	I	13298209	13300716	WBGene00010808	sepa-1	M01E5.6	0.158232018333928	AGIWUR6207
CB4856	CT	I	13302759	13304311	WBGene00011431	T04D3.1	T04D3.1	-0.225526795299075	AGIWUR30784
CB4856	CT	I	13307031	13308787	WBGene00011432	sdz-30	T04D3.2	0.491466622178553	AGIWUR10330;AGIWUR32859;AGIWUR44585
CB4856	CT	I	13309994	13310715	WBGene00011435	T04D3.8	T04D3.8	0.0645585110557025	AGIWUR31158
CB4856	CT	I	13310915	13318704	WBGene00011433	pde-1	T04D3.3	-0.256930015647184	AGIWUR2048
CB4856	CT	I	13325230	13333508	WBGene00001555	gcy-35	T04D3.4	-0.115012770958765	AGIWUR30654
CB4856	CT	I	13333380	13336146	WBGene00012343	W08E3.2	W08E3.2	0.117643433658998	AGIWUR23946
CB4856	CT	I	13337460	13338921	WBGene00012345	W08E3.4	W08E3.4	-0.420738400779746	AGIWUR15170
CB4856	CT	I	13339397	13340224	WBGene00004915	snr-2	W08E3.1	-0.121403088854091	AGIWUR2020;AGIWUR17872;AGIWUR37716
CB4856	CT	I	13340327	13343940	WBGene00012344	ola-1	W08E3.3	0.0662314777397626	AGIWUR20746
CB4856	CT	I	13344044	13346966	WBGene00012732	Y40B1A.1	Y40B1A.1	0.13908341431205	AGIWUR8122;AGIWUR14845;AGIWUR22783;AGIWUR24127;AGIWUR30754;AGIWUR33337
CB4856	CT	I	13347068	13349259	WBGene00012733	Y40B1A.2	Y40B1A.2	0.000259478981242012	AGIWUR39339
CB4856	CT	I	13349410	13358674	WBGene00012734	Y40B1A.3	Y40B1A.3	-0.416401317407434	AGIWUR7751;AGIWUR14091
CB4856	CT	I	13360401	13366888	WBGene00012735	sptf-3	Y40B1A.4	-0.33612228332496	AGIWUR4406
CB4856	CT	I	13379917	13384371	WBGene00007218	C01A2.6	C01A2.6	0.190109611523932	AGIWUR11252
CB4856	CT	I	13384752	13387084	WBGene00007217	tads-1	C01A2.5	-0.220257994044365	

AGIWUR15887
CB4856	CT	I	13387819	13391450	WBGene00007215	oxa-1	C01A2.3	-0.190632948487913	AGIWUR15608;AGIWUR16802;AGIWUR22209;AGIWUR26992;AGIWUR29372;AGIWUR30785
CB4856	CT	I	13392118	13394001	WBGene00007216	C01A2.4	C01A2.4	0.255077206238242	AGIWUR11598
CB4856	CT	I	13397270	13398529	WBGene00007214	C01A2.2	C01A2.2	0.124066391641927	AGIWUR43823
CB4856	CT	I	13402447	13404106	WBGene00007219	nlp-38	C01A2.7	0.103503737423838	AGIWUR11225;AGIWUR16600;AGIWUR17922;AGIWUR36114
CB4856	CT	I	13414273	13414465	WBGene00077561	W05H12.3	W05H12.3	-0.0628406084768862	AGIWUR9577;AGIWUR17249;AGIWUR24012;AGIWUR29770;AGIWUR38825
CB4856	CT	I	13418458	13423379	WBGene00012289	W05H12.1	W05H12.1	-0.153753534594246	AGIWUR2538;AGIWUR7093;AGIWUR42065
CB4856	CT	I	13423994	13425148	WBGene00044066	fbxb-66	Y40B1B.3	0.567588368295242	AGIWUR5934
CB4856	CT	I	13426454	13428471	WBGene00012739	Y40B1B.7	Y40B1B.7	0.068344882187521	AGIWUR19958
CB4856	CT	I	13428913	13434717	WBGene00012740	slc-25A46	Y40B1B.8	0.0222524087721796	AGIWUR4532;AGIWUR25826;AGIWUR38819;AGIWUR40634
CB4856	CT	I	13435489	13437965	WBGene00012738	eif-3.J	Y40B1B.5	-0.135306720044451	AGIWUR5279
CB4856	CT	I	13438058	13450216	WBGene00005010	spr-5	Y40B1B.6	0.0503205745570163	AGIWUR25838;AGIWUR27172;AGIWUR43055
CB4856	CT	I	13451463	13452841	WBGene00013023	Y48G10A.6	Y48G10A.6	0.113687754739193	AGIWUR1449;AGIWUR19843;AGIWUR21746;AGIWUR24663;AGIWUR26086;AGIWUR30523
CB4856	CT	I	13455356	13470439	WBGene00013021	Y48G10A.4	Y48G10A.4	-0.0215235063261705	AGIWUR4727;AGIWUR25796;AGIWUR35731;AGIWUR39194;AGIWUR41307;AGIWUR44612
CB4856	CT	I	13471683	13474185	WBGene00013020	Y48G10A.3	Y48G10A.3	0.168713666247063	AGIWUR36511
CB4856	CT	I	13477214	13487404	WBGene00013019	Y48G10A.2	Y48G10A.2	-0.010602414235569	AGIWUR14937
CB4856	CT	I	13489552	13491226	WBGene00013018	Y48G10A.1	Y48G10A.1	-0.535757017371479	AGIWUR2483
CB4856	CT	I	13491425	13493317	WBGene00013603	soem-1	Y87G2A.17	-0.0566337808174762	AGIWUR34580
CB4856	CT	I	13503045	13507064	WBGene00003585	ndx-8	Y87G2A.14	-0.102776657064254	AGIWUR42138
CB4856	CT	I	13508785	13512026	WBGene00044260	Y87G2A.19	Y87G2A.19	0.269016112421728	AGIWUR38809

CB4856 CT I 13512313 13514320 WBGene00013604 Y87G2A.18 Y87G2A.18
0.164426230414801 AGIWUR37837
CB4856 CT I 13514550 13526254 WBGene00013593 Y87G2A.1 Y87G2A.1
-0.0667485003084298 AGIWUR10029
CB4856 CT I 13528706 13531444 WBGene00044261 Y87G2A.20 Y87G2A.20
0.170759928576697 AGIWUR10888;AGIWUR20595;AGIWUR33352;AGIWUR35074;AGIWUR44230
CB4856 CT I 13534718 13537014 WBGene00013594 Y87G2A.2 Y87G2A.2
0.291806619117011 AGIWUR11310
CB4856 CT I 13536892 13542703 WBGene00013595 atg-4.1 Y87G2A.3
0.0165651325586467 AGIWUR21404
CB4856 CT I 13543079 13545188 WBGene00000089 aex-6 Y87G2A.4
0.160000530298115 AGIWUR6216;AGIWUR10423;AGIWUR15952;AGIWUR20591;AGIWUR26131;AGIWUR43384
CB4856 CT I 13547898 13559361 WBGene00006936 glp-4 Y87G2A.5
-0.0262200383009308 AGIWUR33323
CB4856 CT I 13560447 13563465 WBGene00013602 Y87G2A.16 Y87G2A.16
-0.345843749806056 AGIWUR9625
CB4856 CT I 13564567 13566362 WBGene00001704 grd-15 Y87G2A.15
-0.166758505679052 AGIWUR1040
CB4856 CT I 13566258 13572063 WBGene00000891 cyn-15 Y87G2A.6
0.250374320382999 AGIWUR11314
CB4856 CT I 13572379 13575805 WBGene00013596 nyn-2 Y87G2A.7
0.13385271413398 AGIWUR32583;AGIWUR42958
CB4856 CT I 13576148 13581585 WBGene00013597 gpi-1 Y87G2A.8
-0.0734095742848728 AGIWUR4888;AGIWUR42506
CB4856 CT I 13581698 13585295 WBGene00006709 ubc-14 Y87G2A.9
-0.0737439563850195 AGIWUR13481
CB4856 CT I 13586037 13588831 WBGene00013598 vps-28 Y87G2A.10
-0.0832421532318204 AGIWUR4233;AGIWUR7451;AGIWUR18989;AGIWUR19541;AGIWUR22158;AGIWUR22997;AGIWUR26235
CB4856 CT I 13589099 13598505 WBGene00013599 eipr-1 Y87G2A.11
-0.256926547841207 AGIWUR26147
CB4856 CT I 13598618 13603393 WBGene00013601 Y87G2A.13 Y87G2A.13
-0.322809588058782 AGIWUR15125
CB4856 CT I 13603885 13626225 WBGene00012386 agef-1 Y6B3A.1 -0.041055502093049
AGIWUR25300;AGIWUR33210
CB4856 CT I 13626522 13628447 WBGene00012351 W09C5.1 W09C5.1 0.0754768009212921
AGIWUR17955;AGIWUR23847;AGIWUR32726
CB4856 CT I 13629227 13633494 WBGene00006793 unc-59 W09C5.2 0.117069517686403
AGIWUR44683
CB4856 CT I 13635042 13635958 WBGene00014858 W09C5.3 W09C5.3 -0.0497467724556788

AGIWUR3493;AGIWUR4777
CB4856 CT I 13637845 13639311 WBGene00002116 ins-33 W09C5.4 0.0686797985513437
AGIWUR10812
CB4856 CT I 13640297 13648405 WBGene00012352 dkf-1 W09C5.5 -0.0228833947266907
AGIWUR22471
CB4856 CT I 13652939 13653704 WBGene00004445 rpl-31 W09C5.6 -0.0624129633711732
AGIWUR3290;AGIWUR3645;AGIWUR5928;AGIWUR8527;AGIWUR12729;AGIWUR14128;AGIWUR19084;AGIWUR22677;AGIWUR26990;AGI
WUR30940;AGIWUR31232;AGIWUR42891
CB4856 CT I 13654009 13658167 WBGene00012353 W09C5.7 W09C5.7 0.0622541608257414
AGIWUR16306
CB4856 CT I 13658171 13659128 WBGene00012354 W09C5.8 W09C5.8 -0.0882395544557735
AGIWUR1781;AGIWUR3960;AGIWUR9938;AGIWUR16533;AGIWUR18251;AGIWUR25895
CB4856 CT I 13663462 13674997 WBGene00012234 W04A4.5 W04A4.5 -0.158694869058671
AGIWUR2477
CB4856 CT I 13676255 13677792 WBGene00012233 W04A4.4 W04A4.4 0.40728091253085
AGIWUR39892
CB4856 CT I 13680415 13681366 WBGene00012232 W04A4.3 W04A4.3 -0.407039528599432
AGIWUR12765
CB4856 CT I 13682137 13684417 WBGene00012231 W04A4.2 W04A4.2 -0.0914069344260433
AGIWUR12922;AGIWUR28553;AGIWUR29183
CB4856 CT I 13685303 13687582 WBGene00012235 W04A4.6 W04A4.6 0.253960650639955
AGIWUR30052;AGIWUR32598
CB4856 CT I 13692929 13702954 WBGene00012387 Y6B3B.1 Y6B3B.1 -0.239493278197706
AGIWUR2983
CB4856 CT I 13709779 13719528 WBGene00012388 Y6B3B.3 Y6B3B.3 -0.0835772019271879
AGIWUR35375
CB4856 CT I 13720125 13724453 WBGene00012394 hsd-1 Y6B3B.11
-0.917529822605113 AGIWUR42334
CB4856 CT I 13724577 13732046 WBGene00006505 lagr-1 Y6B3B.10
0.173953705813187 AGIWUR14852
CB4856 CT I 13734958 13751124 WBGene00012393 Y6B3B.9 Y6B3B.9 -0.230094737801723
AGIWUR2653
CB4856 CT I 13751234 13756354 WBGene00012390 gpap-1 Y6B3B.5 -0.208485962930197
AGIWUR18956
CB4856 CT I 13756730 13771773 WBGene00012389 Y6B3B.4 Y6B3B.4 -0.177470347204816
AGIWUR1970;AGIWUR5684;AGIWUR19132;AGIWUR32826
CB4856 CT I 13773067 13778782 WBGene00012576 Y37H9A.1 Y37H9A.1
-0.141280010312496 AGIWUR36695
CB4856 CT I 13778899 13792165 WBGene00012578 Y37H9A.3 Y37H9A.3

-0.038187460173944 AGIWUR7845
CB4856 CT I 13792645 13794152 WBGene00003581 ndx-4 Y37H9A.6
0.172046527798557 AGIWUR33795
CB4856 CT I 13795277 13795867 WBGene00014870 Y37H9A.4 Y37H9A.4
-0.321067928980291 AGIWUR24358
CB4856 CT I 13802390 13806801 WBGene00044065 tag-229 W09G3.3 -0.215342761547864
AGIWUR9860
CB4856 CT I 13806917 13812268 WBGene00012366 maea-1 W09G3.2 0.252559031527644
AGIWUR20326;AGIWUR24050;AGIWUR35534
CB4856 CT I 13814756 13827988 WBGene00012369 W09G3.6 W09G3.6 -0.0306178576826071
AGIWUR9505
CB4856 CT I 13828102 13832146 WBGene00012365 W09G3.1 W09G3.1 0.0521679569093152
AGIWUR21708;AGIWUR21737;AGIWUR23876;AGIWUR31807;AGIWUR34127;AGIWUR37740
CB4856 CT I 13835292 13840047 WBGene00012236 W04A8.1 W04A8.1 -0.17240062135043
AGIWUR8302
CB4856 CT I 13840706 13841846 WBGene00012237 W04A8.2 W04A8.2 -0.173439100762569
AGIWUR13981;AGIWUR15281;AGIWUR17643;AGIWUR36920;AGIWUR42788;AGIWUR44916
CB4856 CT I 13841895 13843480 WBGene00012239 W04A8.4 W04A8.4 0.674116307326618
AGIWUR3994;AGIWUR38834;AGIWUR41125;AGIWUR42044
CB4856 CT I 13844729 13846957 WBGene00012238 W04A8.3 W04A8.3 0.054190533450913
AGIWUR41862
CB4856 CT I 13849599 13850893 WBGene00012240 W04A8.5 W04A8.5 -0.288057510952688
AGIWUR25404
CB4856 CT I 13854632 13859699 WBGene00012241 W04A8.6 W04A8.6 -0.121346372297557
AGIWUR43163
CB4856 CT I 13861472 13887468 WBGene00006382 taf-1 W04A8.7 -0.0503457034159034
AGIWUR29095
CB4856 CT I 13899085 13913854 WBGene00006616 trp-4 Y71A12B.4
0.0433151323316405 AGIWUR41758
CB4856 CT I 13915664 13917809 WBGene00013502 clec-111 Y71A12B.5
-0.225288738511022 AGIWUR36981
CB4856 CT I 13919375 13922122 WBGene00013503 clec-112 Y71A12B.6
-0.0487055930344287 AGIWUR23899
CB4856 CT I 13938185 13942016 WBGene00013505 Y71A12B.8 Y71A12B.8
0.203569956576731 AGIWUR3434
CB4856 CT I 13943079 13951628 WBGene00013506 usp-3 Y71A12B.9
-0.431956386434489 AGIWUR41370
CB4856 CT I 13952159 13953194 WBGene00004475 rps-6 Y71A12B.1
-0.0334022598539718 AGIWUR4211;AGIWUR15277;AGIWUR23183;AGIWUR37410;AGIWUR42537

CB4856	CT	I	13957199	13960440	WBGene00013507	Y71A12B.10	Y71A12B.10	0.013636293440439	AGIWUR3766
CB4856	CT	I	13978430	13980144	WBGene00013508	Y71A12B.11	Y71A12B.11	0.167039257926625	AGIWUR15619
CB4856	CT	I	13984308	13989588	WBGene00045058	gadr-5	Y71A12B.17	0.275685320353978	AGIWUR11183;AGIWUR29400
CB4856	CT	I	13992582	13996221	WBGene00013511	Y71A12B.15	Y71A12B.15	0.133904950528404	AGIWUR23417;AGIWUR23555
CB4856	CT	I	13997363	14007078	WBGene00045077	Y71A12B.18	Y71A12B.18	0.130644783666547	AGIWUR33089
CB4856	CT	I	14004412	14008484	WBGene00013509	Y71A12B.12	Y71A12B.12	0.346417150591069	AGIWUR15382;AGIWUR42964
CB4856	CT	I	14009871	14011521	WBGene00013865	ZC334.7	ZC334.7	0.0210972581729258	AGIWUR38733
CB4856	CT	I	14012369	14013629	WBGene00002111	ins-28	ZC334.9	0.206732908019008	AGIWUR19714
CB4856	CT	I	14018785	14019759	WBGene00002112	ins-29	ZC334.10	-0.0899278465633095	AGIWUR5899;AGIWUR9154;AGIWUR12469;AGIWUR28025;AGIWUR32013
CB4856	CT	I	14020364	14021155	WBGene00002108	ins-25	ZC334.8	-0.143875331072248	AGIWUR2131;AGIWUR3614;AGIWUR5931;AGIWUR17706;AGIWUR32860
CB4856	CT	I	14027236	14027588	WBGene00002110	ins-27	ZC334.11	-0.342543932975202	AGIWUR1125;AGIWUR12173;AGIWUR16115;AGIWUR19429;AGIWUR27925
CB4856	CT	I	14028997	14029212	WBGene00045396	ZC334.12	ZC334.12	-0.645096121308493	AGIWUR3784;AGIWUR6250;AGIWUR31903;AGIWUR38530;AGIWUR42619
CB4856	CT	I	14031147	14031667	WBGene00002107	ins-24	ZC334.3	0.31687218684403	AGIWUR3689
CB4856	CT	I	14035096	14036057	WBGene00002113	ins-30	ZC334.2	-0.023776749646256	AGIWUR40182
CB4856	CT	I	14037151	14037803	WBGene00002109	ins-26	ZC334.1	-0.29353815233465	AGIWUR2102
CB4856	CT	I	14041627	14043478	WBGene00013512	nhr-276	Y71A12C.1	-0.0456297914715469	AGIWUR30870
CB4856	CT	I	14043531	14045694	WBGene00050878	Y71A12C.3	Y71A12C.3	-0.0892494644478603	AGIWUR19098
CB4856	CT	I	14046234	14048017	WBGene00013513	Y71A12C.2	Y71A12C.2	-0.161972133416777	AGIWUR9179;AGIWUR15094
CB4856	CT	I	14048085	14051003	WBGene00004875	smd-1	F47G4.7	-0.982011233212525	AGIWUR33737
CB4856	CT	I	14052136	14054154	WBGene00009828	F47G4.8	F47G4.8	0.00584023320836824	

AGIWUR4404
CB4856 CT I 14054593 14056894 WBGene00009822 clec-113 F47G4.1
0.0630351138680894 AGIWUR33408
CB4856 CT I 14059411 14060829 WBGene00009827 hmg-6 F47G4.6 0.758777970062559
AGIWUR8349
CB4856 CT I 14060876 14062137 WBGene00009826 F47G4.5 F47G4.5 0.365747034625168
AGIWUR13877
CB4856 CT I 14062749 14068803 WBGene00009825 F47G4.4 F47G4.4 0.0660957855125588
AGIWUR5650;AGIWUR9090;AGIWUR19297;AGIWUR26208;AGIWUR33296;AGIWUR39500
CB4856 CT I 14075836 14078211 WBGene00009824 gpdh-1 F47G4.3 -0.524863629967681
AGIWUR20253
CB4856 CT I 14078193 14082643 WBGene00009823 gadr-6 F47G4.2 0.149786786835412
AGIWUR14336;AGIWUR14614
CB4856 CT I 14088094 14089858 WBGene00013404 fbxb-101 Y63D3A.3
1.04903661861518 AGIWUR20759
CB4856 CT I 14090738 14096346 WBGene00013403 fbxb-100 Y63D3A.2
0.555713703529772 AGIWUR42011
CB4856 CT I 14097492 14101090 WBGene00013405 tdpt-1 Y63D3A.4
0.159014990747149 AGIWUR8766
CB4856 CT I 14101236 14104475 WBGene00006565 tfg-1 Y63D3A.5
-0.170934283733445 AGIWUR3031
CB4856 CT I 14104429 14113179 WBGene00001047 dnj-29 Y63D3A.6
-0.0841409411687337
AGIWUR2497;AGIWUR2707;AGIWUR8430;AGIWUR9345;AGIWUR14768;AGIWUR14847;AGIWUR15853;AGIWUR20958;AGIWUR23646;AGI
WUR25975;AGIWUR26974;AGIWUR26995;AGIWUR28054;AGIWUR28176;AGIWUR32588;AGIWUR35335;AGIWUR35775
CB4856 CT I 14113636 14114085 WBGene00013406 Y63D3A.7 Y63D3A.7
-0.275714001728915
AGIWUR7230;AGIWUR16354;AGIWUR21021;AGIWUR25749;AGIWUR29079;AGIWUR34860;AGIWUR35530;AGIWUR35904;AGIWUR43091;
AGIWUR44857
CB4856 CT I 14114194 14118650 WBGene00013407 Y63D3A.8 Y63D3A.8
-0.040198075892123 AGIWUR5018;AGIWUR21326;AGIWUR39732
CB4856 CT I 14118753 14127199 WBGene00010537 mys-2 K03D10.3
-0.10354350792998 AGIWUR15721;AGIWUR29820
CB4856 CT I 14128594 14142207 WBGene00002181 kal-1 K03D10.1
0.444549686290014 AGIWUR36370
CB4856 CT I 14150731 14151041 WBGene00014700 C37A5.6 C37A5.6 0.213411428775162
AGIWUR31033
CB4856 CT I 14154769 14155249 WBGene00014698 C37A5.3 C37A5.3 0.0874527969783889
AGIWUR138;AGIWUR9321;AGIWUR10493;AGIWUR12985;AGIWUR39666

CB4856	CT	I	14157611	14158076	WBGene00007992	fipr-24	C37A5.8	0.741908858441018	AGIWUR23130
CB4856	CT	I	14158506	14161718	WBGene00007988	best-8	C37A5.1	0.173253006634702	AGIWUR7467
CB4856	CT	I	14163447	14168309	WBGene00007991	C37A5.7	C37A5.7	0.137347571927148	AGIWUR43696
CB4856	CT	I	14171864	14172786	WBGene00077520	C37A5.11	C37A5.11	-0.0903895141116333	AGIWUR202;AGIWUR14942;AGIWUR22282;AGIWUR26996;AGIWUR33327
CB4856	CT	I	14172840	14179360	WBGene00004202	pry-1	C37A5.9	-0.212984277459164	AGIWUR33395
CB4856	CT	I	14182787	14185717	WBGene00014100	ZK849.1	ZK849.1	-0.0779402411110777	AGIWUR13863
CB4856	CT	I	14185647	14196295	WBGene00014101	gopc-1	ZK849.2	0.201389700203916	AGIWUR26761;AGIWUR33441;AGIWUR34814;AGIWUR41624;AGIWUR42335;AGIWUR42882
CB4856	CT	I	14198612	14199099	WBGene00014104	ZK849.6	ZK849.6	0.0774580975046568	AGIWUR16143
CB4856	CT	I	14200020	14204687	WBGene00014103	best-26	ZK849.5	-0.402861852712379	AGIWUR7283;AGIWUR24096;AGIWUR30353
CB4856	CT	I	14205717	14208581	WBGene00014102	best-25	ZK849.4	-0.189970153534963	AGIWUR15418
CB4856	CT	I	14209215	14212501	WBGene00008185	best-10	C49A1.2	0.0685204719938177	AGIWUR31693
CB4856	CT	I	14213681	14215790	WBGene00008184	C49A1.1	C49A1.1	0.196284321070939	AGIWUR10091
CB4856	CT	I	14216756	14220624	WBGene00008186	best-11	C49A1.3	-0.256932882010082	AGIWUR23910
CB4856	CT	I	14220783	14231360	WBGene00001377	eya-1	C49A1.4	0.467121425048354	AGIWUR4852
CB4856	CT	I	14237359	14241188	WBGene00008187	C49A1.5	C49A1.5	0.170847544269416	AGIWUR33564;AGIWUR41607
CB4856	CT	I	14241898	14244452	WBGene00008191	C49A1.10	C49A1.10	-0.0174730345836629	AGIWUR23897
CB4856	CT	I	14246228	14247314	WBGene00008188	clec-115	C49A1.6	0.0668020322773337	AGIWUR35266
CB4856	CT	I	14251061	14252362	WBGene00010576	clec-116	K04H8.1	-0.144311389249481	AGIWUR39037
CB4856	CT	I	14252957	14255510	WBGene00010577	K04H8.2	K04H8.2	-0.319200782053446	AGIWUR7769
CB4856	CT	I	14255684	14256970	WBGene00010578	K04H8.3	K04H8.3	-0.133839273832716	

									AGIWUR23282
CB4856	CT	I	14263301	14271321	WBGene00003980	pes-7	F09C3.1	-0.0400846173493079	AGIWUR25123
CB4856	CT	I	14271893	14274213	WBGene00008610	F09C3.2	F09C3.2	0.409302925469689	AGIWUR42136
CB4856	CT	I	14283464	14285319	WBGene00008613	fbxb-69	F09C3.5	0.313855214778605	AGIWUR25537
CB4856	CT	I	14287872	14295898	WBGene00008612	fbxa-103	F09C3.4	0.362941001607139	AGIWUR3613
CB4856	CT	I	14297287	14298265	WBGene00009862	fbxb-67	F49B2.2	-0.113976635320451	AGIWUR34725
CB4856	CT	I	14305070	14307134	WBGene00086555	F49B2.7	F49B2.7	0.299925718680753	AGIWUR6124
CB4856	CT	I	14311009	14312871	WBGene00009863	F49B2.3	F49B2.3	0.225672518668175	AGIWUR21423
CB4856	CT	I	14319114	14319913	WBGene00268216	F49B2.9	F49B2.9	-0.113516498548234	AGIWUR17709;AGIWUR21668;AGIWUR35873
CB4856	CT	I	14322237	14328965	WBGene00005078	src-2	F49B2.5	0.128306267418584	AGIWUR33270
CB4856	CT	I	14331085	14339831	WBGene00009865	F49B2.6	F49B2.6	0.17857940661803	AGIWUR8730;AGIWUR31462;AGIWUR36353
CB4856	CT	I	14341571	14343815	WBGene00013666	Y105E8A.1	Y105E8A.1	0.0507648929858142	AGIWUR26600
CB4856	CT	I	14347514	14358326	WBGene00013667	Y105E8A.2	Y105E8A.2	-0.091389319640415	AGIWUR28921
CB4856	CT	I	14358502	14367594	WBGene00013668	Y105E8A.3	Y105E8A.3	-0.041761495037024	AGIWUR38906
CB4856	CT	I	14367608	14369773	WBGene00001156	ech-7	Y105E8A.4	0.168024155059211	AGIWUR4586
CB4856	CT	I	14371142	14376025	WBGene00000241	bbs-1	Y105E8A.5	0.108825632711024	AGIWUR133
CB4856	CT	I	14378386	14379425	WBGene00044264	Y105E8A.32	Y105E8A.32	0.139479716957929	AGIWUR17134
CB4856	CT	I	14381344	14384737	WBGene00006824	unc-95	Y105E8A.6	-0.184178975879302	AGIWUR23275
CB4856	CT	I	14390333	14400222	WBGene00002977	lev-10	Y105E8A.7	-0.0730492823944342	AGIWUR23424;AGIWUR35694;AGIWUR38498
CB4856	CT	I	14400679	14405649	WBGene00013669	Y105E8A.8	Y105E8A.8	-0.205218195274547	AGIWUR12986

CB4856	CT	I	14406028	14417942	WBGene00000158	apg-1	Y105E8A.9
0.0325098362915632	AGIWUR1586;AGIWUR14637
CB4856	CT	I	14418197	14431806	WBGene00013670	hpo-13	Y105E8A.10
0.0666809076984336	AGIWUR42701
CB4856	CT	I	14432793	14433841	WBGene00013671	Y105E8A.11	Y105E8A.11
-0.0614078639610867	AGIWUR15864
CB4856	CT	I	14436810	14451880	WBGene00013672	catp-1	Y105E8A.12
0.0371621735915824	AGIWUR4487
CB4856	CT	I	14465380	14468214	WBGene00013674	Y105E8A.14	Y105E8A.14
-0.156648948543437
AGIWUR918;AGIWUR7311;AGIWUR12133;AGIWUR12400;AGIWUR29395;AGIWUR31736;AGIWUR37639;AGIWUR43745
CB4856	CT	I	14468321	14469354	WBGene00004489	rps-20	Y105E8A.16
-0.0907677651381071	AGIWUR26559
CB4856	CT	I	14469506	14476790	WBGene00013676	ekl-4	Y105E8A.17
-0.00885651595846593	AGIWUR43665;AGIWUR43829
CB4856	CT	I	14476674	14486767	WBGene00013677	yars-1	Y105E8A.19
-0.0206610895460519	AGIWUR18952;AGIWUR25570
CB4856	CT	I	14486878	14496560	WBGene00013678	Y105E8A.20	Y105E8A.20
0.414555706015156	AGIWUR11146
CB4856	CT	I	14496909	14498291	WBGene00013685	Y105E8A.28	Y105E8A.28
-0.216848255229378	AGIWUR6382
CB4856	CT	I	14499727	14510552	WBGene00013679	Y105E8A.21	Y105E8A.21
-0.208156994052329	AGIWUR3433
CB4856	CT	I	14511431	14518719	WBGene00001365	exc-4	Y105E8A.22
-0.280430176688474	AGIWUR16297;AGIWUR28292
CB4856	CT	I	14529531	14546634	WBGene00013683	zoo-1	Y105E8A.26
0.162926228918636	AGIWUR38954
CB4856	CT	I	14552030	14565853	WBGene00013680	rpom-1	Y105E8A.23
-0.0230037657898698	AGIWUR36449
CB4856	CT	I	14567169	14616524	WBGene00013682	Y105E8A.25	Y105E8A.25
-0.0174628644061841	AGIWUR1343;AGIWUR2683;AGIWUR4821;AGIWUR7929;AGIWUR18859;AGIWUR19624;AGIWUR36825
CB4856	CT	I	14620388	14620632	WBGene00044265	Y105E8B.11	Y105E8B.11
0.212975901569259	AGIWUR949;AGIWUR4198;AGIWUR10506;AGIWUR29150;AGIWUR40348
CB4856	CT	I	14621173	14631254	WBGene00002978	lev-11	Y105E8B.1
0.216714705013759	AGIWUR10189;AGIWUR13276;AGIWUR26792;AGIWUR32066
CB4856	CT	I	14634456	14642653	WBGene00013687	exoc-8	Y105E8B.2
-0.0417153288425371	AGIWUR34821;AGIWUR40365;AGIWUR40499;AGIWUR43251
CB4856	CT	I	14642714	14646600	WBGene00013692	Y105E8B.7	Y105E8B.7
-0.00243054523795116	AGIWUR3209

CB4856	CT	I	14647782	14651456	WBGene00013689	bath-40	Y105E8B.4
-0.243857041913413	AGIWUR35669
CB4856	CT	I	14652116	14657275	WBGene00013688	riok-2	Y105E8B.3
-0.0395073680439832	AGIWUR17763;AGIWUR26680;AGIWUR41638
CB4856	CT	I	14657355	14661414	WBGene00013690	hprt-1	Y105E8B.5
-0.350604974599932	AGIWUR26599
CB4856	CT	I	14662524	14662873	WBGene00013691	Y105E8B.6	Y105E8B.6
-0.0138021642971817	AGIWUR6708;AGIWUR11489;AGIWUR23025;AGIWUR33975;AGIWUR37197;AGIWUR39910
CB4856	CT	I	14663209	14674822	WBGene00001334	ero-1	Y105E8B.8
0.0578724868111706	AGIWUR15790;AGIWUR38676
CB4856	CT	I	14675600	14682566	WBGene00013693	Y105E8B.9	Y105E8B.9
0.0451353701204766	AGIWUR1425;AGIWUR6556;AGIWUR10139;AGIWUR24148
CB4856	CT	I	14688639	14691736	WBGene00013197	ttm-5	Y54E5A.1
-0.0372209260909671	AGIWUR43809
CB4856	CT	I	14694154	14700392	WBGene00013198	Y54E5A.2	Y54E5A.2
-0.0717153932938741	AGIWUR12835;AGIWUR39872
CB4856	CT	I	14701122	14706522	WBGene00003790	npp-4	Y54E5A.4
-0.193627351278694	AGIWUR665;AGIWUR10793;AGIWUR11371;AGIWUR15099;AGIWUR25502;AGIWUR33722
CB4856	CT	I	14707059	14707875	WBGene00013200	Y54E5A.5	Y54E5A.5
-0.090115915125601	AGIWUR39609
CB4856	CT	I	14708195	14710961	WBGene00013201	dus-2	Y54E5A.6
-0.15238939266186	AGIWUR32804
CB4856	CT	I	14711081	14715030	WBGene00013202	Y54E5A.7	Y54E5A.7
0.103875687981521	AGIWUR27553
CB4856	CT	I	14716528	14719027	WBGene00013203	Y54E5A.8	Y54E5A.8
-0.0221195447974223	AGIWUR31127;AGIWUR33181
CB4856	CT	I	14719167	14720477	WBGene00003951	pbs-5	K05C4.1	-0.38825743888145
AGIWUR4323;AGIWUR6843
CB4856	CT	I	14720588	14721979	WBGene00010579	K05C4.2	K05C4.2	-0.155997788071971
AGIWUR97;AGIWUR12763;AGIWUR19503;AGIWUR28718;AGIWUR37026;AGIWUR39116;AGIWUR39497
CB4856	CT	I	14725296	14726131	WBGene00010586	K05C4.10	K05C4.10
-0.0235847424066602	AGIWUR6961;AGIWUR7840;AGIWUR25950
CB4856	CT	I	14726804	14730719	WBGene00010585	K05C4.9	K05C4.9	0.0490351720935447
AGIWUR29746
CB4856	CT	I	14732426	14733549	WBGene00010584	K05C4.8	K05C4.8	0.0841779045100139
AGIWUR39087
CB4856	CT	I	14735236	14736945	WBGene00010581	K05C4.4	K05C4.4	-0.099469947372242
AGIWUR43308
CB4856	CT	I	14737702	14738796	WBGene00010583	K05C4.7	K05C4.7	-0.182863011902459

AGIWUR26735
CB4856 CT I 14738882 14742657 WBGene00001979 hmp-2 K05C4.6 -0.212454591735703
AGIWUR5348
CB4856 CT I 14742765 14747789 WBGene00010582 K05C4.5 K05C4.5 0.143720347560595
AGIWUR38011
CB4856 CT I 14755154 14756432 WBGene00009559 mtx-1 F39B2.11
-0.192791628736368 AGIWUR191;AGIWUR5779;AGIWUR11203;AGIWUR43974
CB4856 CT I 14756858 14759354 WBGene00001030 dnj-12 F39B2.10
-0.349476678281095 AGIWUR13540;AGIWUR15860;AGIWUR19114;AGIWUR25936;AGIWUR32563;AGIWUR40305
CB4856 CT I 14759918 14760654 WBGene00004495 rps-26 F39B2.6 -0.0640087266997806
AGIWUR5600;AGIWUR11547;AGIWUR15702;AGIWUR16030;AGIWUR26227;AGIWUR35000;AGIWUR36505;AGIWUR42692
CB4856 CT I 14760644 14768980 WBGene00009558 F39B2.8 F39B2.8 0.386283435902026
AGIWUR40829
CB4856 CT I 14770689 14775007 WBGene00009557 mtcu-1 F39B2.7 0.183637381481372
AGIWUR33237
CB4856 CT I 14775184 14776663 WBGene00009556 F39B2.5 F39B2.5 -0.121067470055637
AGIWUR3717
CB4856 CT I 14777139 14787804 WBGene00006349 sur-2 F39B2.4 -0.0757108030995752
AGIWUR2835
CB4856 CT I 14788125 14789517 WBGene00009554 F39B2.3 F39B2.3 -0.119221287088255
AGIWUR9553;AGIWUR10012;AGIWUR18635;AGIWUR19118;AGIWUR28859;AGIWUR38962
CB4856 CT I 14790244 14790934 WBGene00006730 uev-1 F39B2.2 0.242348395237476
AGIWUR32262
CB4856 CT I 14791040 14794371 WBGene00009553 hinf-1 F39B2.1 -0.0629228791141765
AGIWUR523;AGIWUR1351;AGIWUR6152;AGIWUR7485;AGIWUR15969;AGIWUR17279;AGIWUR21722;AGIWUR25305
CB4856 CT I 14794899 14797166 WBGene00010766 K11B4.1 K11B4.1 -0.285560063535225
AGIWUR14744
CB4856 CT I 14797660 14798705 WBGene00010767 blos-9 K11B4.2 -0.140004242478602
AGIWUR7970
CB4856 CT I 14807467 14815725 WBGene00004889 smp-1 Y54E5B.1
0.154216722014497 AGIWUR30467;AGIWUR30814
CB4856 CT I 14815706 14820222 WBGene00013204 Y54E5B.2 Y54E5B.2
-0.030212362169165 AGIWUR11700;AGIWUR39232
CB4856 CT I 14820381 14821651 WBGene00002324 let-49 Y54E5B.3
-0.425675677050426 AGIWUR23956
CB4856 CT I 14822050 14823996 WBGene00006711 ubc-16 Y54E5B.4
0.0149102384495992 AGIWUR12386;AGIWUR26881;AGIWUR26984;AGIWUR40178
CB4856 CT I 14824493 14825952 WBGene00014921 Y54E5B.5 Y54E5B.5
-0.366014593144688 AGIWUR12811;AGIWUR14671

CB4856 CT I 14826558 14827779 WBGene00005839 srw-92 F32A7.1 -0.0151891285749368
AGIWUR1679;AGIWUR23528;AGIWUR25181;AGIWUR30153;AGIWUR41623;AGIWUR41896
CB4856 CT I 14828200 14829594 WBGene00005840 srw-93 F32A7.2 0.223744820631736
AGIWUR1982;AGIWUR19601;AGIWUR32483;AGIWUR33418
CB4856 CT I 14829667 14834839 WBGene00009304 eva-1 F32A7.3 0.0623972865589746
AGIWUR1324;AGIWUR10089;AGIWUR20986;AGIWUR37717;AGIWUR43383
CB4856 CT I 14838917 14839138 WBGene00044245 F32A7.8 F32A7.8 -0.0243731268969762
AGIWUR10354;AGIWUR11237;AGIWUR18460;AGIWUR40339;AGIWUR41657
CB4856 CT I 14839856 14843128 WBGene00009305 F32A7.4 F32A7.4 -0.0522639365612356
AGIWUR13160
CB4856 CT I 14846476 14850137 WBGene00009306 maph-1.1 F32A7.5
0.0590037382142867 AGIWUR5224;AGIWUR15454;AGIWUR20881;AGIWUR24422;AGIWUR29527
CB4856 CT I 14850054 14855827 WBGene00000088 aex-5 F32A7.6 0.0855629417824222
AGIWUR36674
CB4856 CT I 14855901 14863573 WBGene00006789 unc-54 F11C3.3 0.256600122641911
AGIWUR25719;AGIWUR36183;AGIWUR43581
CB4856 CT I 14867924 14871467 WBGene00008695 F11C3.1 F11C3.1 -0.207775523902773
AGIWUR21606
CB4856 CT I 14872112 14875378 WBGene00006845 unc-122 F11C3.2 -0.392410552636367
AGIWUR5296
CB4856 CT I 14875784 14900213 WBGene00001488 frm-1 ZK270.2 -0.279910233363569
AGIWUR29071;AGIWUR39050;AGIWUR42633
CB4856 CT I 14909240 14913083 WBGene00004237 ptr-23 ZK270.1 0.412894905680346
AGIWUR19053
CB4856 CT I 14913623 14915571 WBGene00009022 F21F12.1 F21F12.1
0.0151383318122695 AGIWUR3298
CB4856 CT I 14919533 14951924 WBGene00002189 kin-1 ZK909.2 -0.0532367783382712
AGIWUR2274;AGIWUR5154;AGIWUR8830;AGIWUR9986;AGIWUR11088;AGIWUR14732;AGIWUR18535;AGIWUR27166;AGIWUR34274;AGI
WUR35252;AGIWUR38388;AGIWUR40098;AGIWUR41578;AGIWUR42171;AGIWUR43175
CB4856 CT I 14953490 14955492 WBGene00014148 ZK909.3 ZK909.3 -0.315799033696319
AGIWUR12225;AGIWUR23203;AGIWUR29149;AGIWUR39359;AGIWUR39889
CB4856 CT I 14955647 14957097 WBGene00014149 fbxb-70 ZK909.5 0.31901930174361
AGIWUR10119;AGIWUR38208;AGIWUR44401
CB4856 CT I 14961501 14965361 WBGene00000469 ces-2 ZK909.4 1.50360257572717
AGIWUR13093
CB4856 CT I 14967301 14968540 WBGene00014150 ZK909.6 ZK909.6 -0.315758368536903
AGIWUR20349
CB4856 CT I 14975419 14980586 WBGene00013970 klu-1 ZK337.2 -0.0893644594190874
AGIWUR38347

CB4856	CT	I	14984178	14987658	WBGene00006399	tag-4	ZK337.4	0.128531109886122	AGIWUR16061
CB4856	CT	I	14993821	15001682	WBGene00013969	tep-1	ZK337.1	0.464774782218051	AGIWUR25691
CB4856	CT	I	15001854	15005608	WBGene00003471	mtd-1	ZK337.5	0.0170433518247908	AGIWUR33354
CB4856	CT	I	15005603	15013904	WBGene00009368	pole-1	F33H2.5	0.157441528038897	AGIWUR7795
CB4856	CT	I	15014851	15016595	WBGene00009369	F33H2.6	F33H2.6	-0.281231519950051	AGIWUR26108;AGIWUR28125;AGIWUR29986;AGIWUR34837;AGIWUR37775;AGIWUR38817
CB4856	CT	I	15017283	15023017	WBGene00001049	dog-1	F33H2.1	-0.230768849957791	AGIWUR16013
CB4856	CT	I	15023092	15027761	WBGene00009366	F33H2.2	F33H2.2	-0.212587564816896	AGIWUR41707
CB4856	CT	I	15027717	15030666	WBGene00009370	set-10	F33H2.7	-0.170303047533546	AGIWUR21015;AGIWUR27125
CB4856	CT	I	15031145	15033039	WBGene00009371	F33H2.8	F33H2.8	0.716130661065259	AGIWUR34249
CB4856	CT	I	15034517	15035467	WBGene00009367	F33H2.3	F33H2.3	0.194867395693166	AGIWUR26011
CB4856	CT	I	15036373	15040487	WBGene00009284	F31C3.2	F31C3.2	-0.174811658028622	AGIWUR3373
CB4856	CT	I	15040427	15041274	WBGene00000881	cyn-5	F31C3.1	0.0258078891172975	AGIWUR8073;AGIWUR36731
CB4856	CT	I	15041433	15050700	WBGene00009285	F31C3.3	F31C3.3	0.147027943901143	AGIWUR40656
CB4856	CT	I	15050797	15052420	WBGene00009286	zipt-1	F31C3.4	0.0111173144045988	AGIWUR32215;AGIWUR36866;AGIWUR41938
CB4856	CT	I	15053025	15053670	WBGene00009287	psf-2	F31C3.5	-0.162836934291672	AGIWUR7586
CB4856	CT	I	15053617	15059003	WBGene00009288	F31C3.6	F31C3.6	0.194862335548302	AGIWUR4435;AGIWUR6596;AGIWUR10855;AGIWUR23668;AGIWUR30942;AGIWUR43814
CB4856	CT	I	15064301	15064453	WBGene00004567	rrn-2.1	F31C3.11	-0.0532743465654022	AGIWUR3226;AGIWUR10771;AGIWUR23918;AGIWUR33092;AGIWUR34754
CB4856	CT	I	15064838	15068346	WBGene00004622	rrn-3.1	F31C3.9	0.0367613485213045	AGIWUR1682;AGIWUR9181;AGIWUR9854;AGIWUR13031;AGIWUR16753;AGIWUR20764;AGIWUR24344;AGIWUR25995;AGIWUR26866;AGIWUR28966;AGIWUR31062;AGIWUR40604;AGIWUR42613
CB4856	CT	II	1848	4717	WBGene00007063	2L52.1	2L52.1	-0.159705177811992	AGIWUR7229
CB4856	CT	II	5129	5483	WBGene00022904	C01B12.9	C01B12.9	0.075794631840342	

AGIWUR3983;AGIWUR13294;AGIWUR20192;AGIWUR35059;AGIWUR42377
CB4856 CT II 6664 9486 WBGene00015287 osta-1 C01B12.4 0.0623477043898291
AGIWUR4697;AGIWUR29006;AGIWUR40725
CB4856 CT II 9485 11846 WBGene00015288 best-4 C01B12.5 0.0533387828059713
AGIWUR7492;AGIWUR13932;AGIWUR42239
CB4856 CT II 12985 16001 WBGene00015286 best-3 C01B12.3 0.230813058583877
AGIWUR14530
CB4856 CT II 19538 22169 WBGene00015285 gmeb-1 C01B12.2 -0.0655132188890706
AGIWUR30642;AGIWUR31147;AGIWUR31577;AGIWUR43552
CB4856 CT II 23328 24458 WBGene00005017 sqt-2 C01B12.1 -0.410984643087556
AGIWUR13749;AGIWUR14151;AGIWUR38043
CB4856 CT II 24666 25446 WBGene00015291 C01B12.8 C01B12.8 -0.295526475155633
AGIWUR4616
CB4856 CT II 25382 28423 WBGene00017745 F23F1.4 F23F1.4 0.166779164687463
AGIWUR29868;AGIWUR33772
CB4856 CT II 30061 31584 WBGene00017746 F23F1.5 F23F1.5 -0.229366342721875 AGIWUR40336
CB4856 CT II 31768 33610 WBGene00017744 fbxc-54 F23F1.3 -0.0553286464017511 AGIWUR18725
CB4856 CT II 34204 35262 WBGene00017743 F23F1.2 F23F1.2 -0.697957564797708 AGIWUR15988
CB4856 CT II 35202 37313 WBGene00017747 F23F1.6 F23F1.6 0.539013548244773 AGIWUR34202
CB4856 CT II 38558 39537 WBGene00017748 F23F1.7 F23F1.7 -0.443849393535093 AGIWUR1202
CB4856 CT II 41471 41982 WBGene00017750 F23F1.10 F23F1.10 -0.264053578927959
AGIWUR3668;AGIWUR12855;AGIWUR14753;AGIWUR19238;AGIWUR28736
CB4856 CT II 42181 46216 WBGene00004504 rpt-4 F23F1.8 -0.148742442775799
AGIWUR4840;AGIWUR5595;AGIWUR18020;AGIWUR20985;AGIWUR35442
CB4856 CT II 45040 47479 WBGene00017749 rpoa-49 F23F1.9 -0.138799287649613 AGIWUR41889
CB4856 CT II 47580 50303 WBGene00016021 C23H3.5 C23H3.5 -0.286943163534993 AGIWUR14601
CB4856 CT II 51072 57576 WBGene00016020 sptl-1 C23H3.4 -0.46170027184624
AGIWUR15255;AGIWUR28293;AGIWUR41602
CB4856 CT II 57670 60040 WBGene00016019 C23H3.3 C23H3.3 -0.0824328095523565
AGIWUR17330;AGIWUR18749;AGIWUR41138
CB4856 CT II 61302 64340 WBGene00016018 C23H3.2 C23H3.2 -0.0416656166429394
AGIWUR1906;AGIWUR13184;AGIWUR26164;AGIWUR32918
CB4856 CT II 69654 71279 WBGene00001193 egl-26 C23H3.1 -0.0446226702012815 AGIWUR31601
CB4856 CT II 71672 79266 WBGene00016022 C23H3.9 C23H3.9 0.365769986585541
AGIWUR6544;AGIWUR37497;AGIWUR38816
CB4856 CT II 84434 87823 WBGene00016809 C50D2.6 C50D2.6 0.538738796664287 AGIWUR28282
CB4856 CT II 88221 91519 WBGene00016812 C50D2.9 C50D2.9 -0.0976749559758296 AGIWUR14627
CB4856 CT II 92169 95277 WBGene00016810 C50D2.7 C50D2.7 0.00542502751848772 AGIWUR26625
CB4856 CT II 96127 98027 WBGene00016811 C50D2.8 C50D2.8 -0.405304267144158 AGIWUR13595

CB4856 CT II 98404 98961 WBGene00016808 C50D2.5 C50D2.5 -0.17238322617161
AGIWUR3897;AGIWUR20416;AGIWUR21779;AGIWUR31226
CB4856 CT II 99647 101061 WBGene00044420 C50D2.10 C50D2.10
-0.000838827049761903 AGIWUR3739;AGIWUR5801;AGIWUR7174;AGIWUR29349;AGIWUR42173
CB4856 CT II 104534 105833 WBGene00000644 col-68 C50D2.4 -0.287810701873884 AGIWUR5687
CB4856 CT II 106189 107521 WBGene00016807 C50D2.3 C50D2.3 -0.0758804485258775 AGIWUR39774
CB4856 CT II 109165 111429 WBGene00016806 C50D2.2 C50D2.2 -0.12396705388705
AGIWUR1716;AGIWUR23387;AGIWUR40635
CB4856 CT II 111621 114454 WBGene00016805 C50D2.1 C50D2.1 0.18479873592053 AGIWUR33829
CB4856 CT II 114429 121222 WBGene00019615 K10B4.3 K10B4.3 0.491392720270211 AGIWUR40135
CB4856 CT II 123270 125004 WBGene00019616 nmur-2 K10B4.4 0.0663382762766327 AGIWUR4619
CB4856 CT II 126938 128706 WBGene00005081 srd-3 K10B4.5 0.172464439840318 AGIWUR20269
CB4856 CT II 130983 133598 WBGene00000857 cwn-1 K10B4.6 0.154178292903982 AGIWUR16463
CB4856 CT II 135466 139999 WBGene00019613 K10B4.1 K10B4.1 -0.14976427488136 AGIWUR11600
CB4856 CT II 147241 152825 WBGene00020798 T25D3.4 T25D3.4 -0.19733235062114 AGIWUR37414
CB4856 CT II 152525 154936 WBGene00020797 T25D3.3 T25D3.3 0.108858334151407 AGIWUR40922
CB4856 CT II 154875 156043 WBGene00020796 mrpl-28 T25D3.2 -0.202914928551017
AGIWUR5393;AGIWUR23054;AGIWUR24701;AGIWUR35171;AGIWUR35915;AGIWUR40250;AGIWUR43080
CB4856 CT II 162374 170628 WBGene00001340 etr-1 T01D1.2 0.0686789370364735
AGIWUR4015;AGIWUR8140;AGIWUR15274;AGIWUR21571;AGIWUR31029;AGIWUR31047;AGIWUR31656;AGIWUR32715;AGIWUR36529;A
GIWUR42342
CB4856 CT II 171386 173779 WBGene00044428 T01D1.7 T01D1.7 0.0733802104590149
AGIWUR33551;AGIWUR37702
CB4856 CT II 174401 175642 WBGene00020148 T01D1.3 T01D1.3 -0.35962132628099 AGIWUR6933
CB4856 CT II 176997 179093 WBGene00020149 T01D1.4 T01D1.4 -0.385668851520668 AGIWUR11472
CB4856 CT II 179261 192078 WBGene00044641 T01D1.8 T01D1.8 0.1841647538851
AGIWUR3097;AGIWUR10524;AGIWUR10527;AGIWUR16181;AGIWUR19838;AGIWUR28896
CB4856 CT II 192531 194558 WBGene00000034 abu-11 T01D1.6 -0.738196380218374 AGIWUR5572
CB4856 CT II 196985 211941 WBGene00000497 chs-2 F48A11.1 0.0572146205870281
AGIWUR6866;AGIWUR9413;AGIWUR12416;AGIWUR12580;AGIWUR13068;AGIWUR28174;AGIWUR32227;AGIWUR38725
CB4856 CT II 213169 216621 WBGene00018585 F48A11.4 F48A11.4 0.12370108120211
AGIWUR38865
CB4856 CT II 217049 220667 WBGene00018586 ubxn-3 F48A11.5 0.027310532039472
AGIWUR38277
CB4856 CT II 222700 232739 WBGene00021545 Y43H11AL.1 Y43H11AL.1 -0.320789024343473
AGIWUR31822
CB4856 CT II 232852 237821 WBGene00021546 laat-1 Y43H11AL.2 -0.0719338470013824
AGIWUR382;AGIWUR607;AGIWUR4019;AGIWUR31192;AGIWUR32769;AGIWUR41132
CB4856 CT II 240722 255182 WBGene00004166 pqn-85 Y43H11AL.3 0.441956931726084

AGIWUR10541
CB4856 CT II 257819 261063 WBGene00000296 cat-2 B0432.5 0.0617109222286869
AGIWUR7552;AGIWUR31219
CB4856 CT II 264353 266077 WBGene00044640 B0432.14 B0432.14 0.251870498434124
AGIWUR7508;AGIWUR7619;AGIWUR9732;AGIWUR17555;AGIWUR23611;AGIWUR35504;AGIWUR36059;AGIWUR38673;AGIWUR40321
CB4856 CT II 266301 273966 WBGene00015188 B0432.7 B0432.7 -0.111885910916659
AGIWUR4743;AGIWUR10714
CB4856 CT II 276212 278736 WBGene00015189 B0432.8 B0432.8 -0.190380918551821 AGIWUR8831
CB4856 CT II 278621 282576 WBGene00015194 B0432.13 B0432.13 -0.244036919730259
AGIWUR20880
CB4856 CT II 282576 283694 WBGene00015190 B0432.9 B0432.9 -0.0244060875440368 AGIWUR23197
CB4856 CT II 283887 286275 WBGene00015191 B0432.10 B0432.10 -0.141175282334103
AGIWUR20057
CB4856 CT II 286625 288850 WBGene00015186 misc-1 B0432.4 0.0964205821362274 AGIWUR20234
CB4856 CT II 288939 290391 WBGene00015185 mrpl-41 B0432.3 -0.11576943630628 AGIWUR1350
CB4856 CT II 290582 291861 WBGene00015184 djr-1.1 B0432.2 0.452529042136639 AGIWUR11594
CB4856 CT II 293864 294706 WBGene00015192 B0432.11 B0432.11 0.0961025118916599
AGIWUR35985
CB4856 CT II 296291 297010 WBGene00015193 clec-117 B0432.12 0.271545686661192
AGIWUR35170
CB4856 CT II 297399 299735 WBGene00015183 B0432.1 B0432.1 0.00761661128076341 AGIWUR9466
CB4856 CT II 302405 303659 WBGene00019280 K01A2.5 K01A2.5 0.0671250045785392 AGIWUR41704
CB4856 CT II 303924 305530 WBGene00000645 col-69 K01A2.7 0.0668075931419558 AGIWUR24367
CB4856 CT II 307545 313969 WBGene00019279 K01A2.4 K01A2.4 0.209337433108502
AGIWUR1000;AGIWUR1739
CB4856 CT II 319785 321445 WBGene00001391 far-7 K01A2.2 2.41776848458096
AGIWUR24256;AGIWUR29060;AGIWUR40355
CB4856 CT II 321442 323098 WBGene00019284 K01A2.10 K01A2.10 0.940295065738761
AGIWUR2318
CB4856 CT II 326262 328224 WBGene00019277 sgcb-1 K01A2.1 -0.245070419133592
AGIWUR7196;AGIWUR43662
CB4856 CT II 328564 332513 WBGene00019285 cbn-1 K01A2.11 0.518112661655986
AGIWUR15086;AGIWUR18315;AGIWUR42119
CB4856 CT II 337339 339248 WBGene00000778 cpn-2 D1069.2 0.216313947100499 AGIWUR32382
CB4856 CT II 341982 347891 WBGene00017037 D1069.3 D1069.3 0.161770630645082
AGIWUR2482;AGIWUR11734;AGIWUR22668;AGIWUR31920
CB4856 CT II 348266 357024 WBGene00017038 dmsr-4 D1069.4 -0.193325190501895
AGIWUR1416;AGIWUR19580
CB4856 CT II 362495 367555 WBGene00004218 ptr-3 C41D7.2 1.65213052526235 AGIWUR13713

CB4856	CT	II	375250	375835	WBGene00015406	C03H5.4	C03H5.4	0.00900637379663394	AGIWUR16004;AGIWUR28127;AGIWUR29633
CB4856	CT	II	380573	382257	WBGene00015409	C03H5.7	C03H5.7	0.24016527537914	AGIWUR1131;AGIWUR3098;AGIWUR42721
CB4856	CT	II	382309	385358	WBGene00015407	C03H5.5	C03H5.5	0.154553142210888	AGIWUR9543
CB4856	CT	II	386134	387949	WBGene00015405	C03H5.3	C03H5.3	-0.187620239926472	AGIWUR35781
CB4856	CT	II	388833	392732	WBGene00015404	nstp-4	C03H5.2	0.352966732287026	AGIWUR10131
CB4856	CT	II	402580	404235	WBGene00015403	clec-10	C03H5.1	-0.659014487293917	AGIWUR17547
CB4856	CT	II	406141	410961	WBGene00016074	C24H12.5	C24H12.5	0.152250855926011	AGIWUR38684
CB4856	CT	II	411222	416620	WBGene00016073	C24H12.4	C24H12.4	-0.126195215775828	AGIWUR8344;AGIWUR26796;AGIWUR27112;AGIWUR35447;AGIWUR44221
CB4856	CT	II	417349	418882	WBGene00016075	C24H12.6	C24H12.6	0.132364797071994	AGIWUR12274
CB4856	CT	II	419178	420568	WBGene00016076	C24H12.7	C24H12.7	0.248798562094976	AGIWUR2158
CB4856	CT	II	422319	423142	WBGene00016077	C24H12.8	C24H12.8	-0.00160999092605962	AGIWUR66
CB4856	CT	II	423624	427019	WBGene00016078	C24H12.9	C24H12.9	0.236970902255886	AGIWUR781;AGIWUR7070;AGIWUR11382;AGIWUR12130;AGIWUR15508;AGIWUR37267;AGIWUR45078
CB4856	CT	II	427603	429152	WBGene00016080	C24H12.11	C24H12.11	0.393383220238086	AGIWUR31524
CB4856	CT	II	436867	438748	WBGene00016072	C24H12.2	C24H12.2	0.0647163085499312	AGIWUR8039
CB4856	CT	II	444132	446928	WBGene00235338	C24H12.19	C24H12.19	0.559809382368138	AGIWUR25847
CB4856	CT	II	448346	453156	WBGene00021130	W10D9.2	W10D9.2	0.317660879837966	AGIWUR34864
CB4856	CT	II	456113	457886	WBGene00185079	W10D9.6	W10D9.6	-0.0312718726569159	AGIWUR5554
CB4856	CT	II	461667	467365	WBGene00021132	nfyb-1	W10D9.4	-0.27536225365046	AGIWUR25019
CB4856	CT	II	468374	470475	WBGene00021133	tomm-22	W10D9.5	-0.121881965322327	AGIWUR7361
CB4856	CT	II	471247	475940	WBGene00021073	nol-1	W07E6.1	-0.147364362403858	AGIWUR4767;AGIWUR18751;AGIWUR29814;AGIWUR30790;AGIWUR35336;AGIWUR42846
CB4856	CT	II	476222	478063	WBGene00021076	W07E6.5	W07E6.5	-0.040975461784722	AGIWUR697;AGIWUR10017;AGIWUR17438;AGIWUR35291;AGIWUR42424
CB4856	CT	II	480722	484386	WBGene00021074	W07E6.2	W07E6.2	0.0859038817040378	AGIWUR43424
CB4856	CT	II	484866	490403	WBGene00004188	prp-21	W07E6.4	-0.0265113608155687	AGIWUR25405
CB4856	CT	II	495424	498659	WBGene00021075	W07E6.3	W07E6.3	0.163157527147798	AGIWUR28387
CB4856	CT	II	503229	506014	WBGene00005324	T27A1.1	T27A1.1	0.162696776482034	AGIWUR10735;AGIWUR24620

CB4856	CT	II	508245	509326	WBGene00005512	srh-105	T27A1.7	0.272287262815652	AGIWUR40738
CB4856	CT	II	516014	521605	WBGene00003106	mab-9	T27A1.6	0.197963840758682	AGIWUR7631;AGIWUR14622;AGIWUR24220
CB4856	CT	II	522594	525983	WBGene00020837	slc-36.2	T27A1.5	-0.0519289068498132	AGIWUR8375
CB4856	CT	II	526574	530374	WBGene00020836	lgc-34	T27A1.4	0.00977556572315486	AGIWUR23718
CB4856	CT	II	535578	546944	WBGene00020835	T27A1.3	T27A1.3	-0.00786805329369855	AGIWUR41722
CB4856	CT	II	546705	552031	WBGene00015646	mlt-10	C09E8.3	0.357787040513132	AGIWUR13193;AGIWUR31746
CB4856	CT	II	553086	554789	WBGene00015645	lips-7	C09E8.2	-0.234727409843124	AGIWUR27124;AGIWUR27958;AGIWUR30248
CB4856	CT	II	556485	564215	WBGene00015644	C09E8.1	C09E8.1	-0.0915628129593342	AGIWUR16148;AGIWUR22381;AGIWUR24838;AGIWUR43911
CB4856	CT	II	569236	572110	WBGene00021095	mlt-8	W08F4.6	0.500901909541019	AGIWUR39199
CB4856	CT	II	574872	577732	WBGene00021096	W08F4.7	W08F4.7	0.202197683323581	AGIWUR15585;AGIWUR17009;AGIWUR42523
CB4856	CT	II	577839	580593	WBGene00021097	cdc-37	W08F4.8	0.0854375463466415	AGIWUR41954;AGIWUR45168
CB4856	CT	II	580602	582014	WBGene00021093	W08F4.3	W08F4.3	-0.159022375919124	AGIWUR6026
CB4856	CT	II	584798	586241	WBGene00044733	W08F4.13	W08F4.13	0.515545945431847	AGIWUR8104
CB4856	CT	II	589161	589993	WBGene00268207	W08F4.20	W08F4.20	0.311908826153829	AGIWUR27767
CB4856	CT	II	590578	591210	WBGene00021099	W08F4.10	W08F4.10	0.112203223359797	AGIWUR29;AGIWUR12451;AGIWUR19061;AGIWUR26023;AGIWUR36121
CB4856	CT	II	595207	602120	WBGene00003599	nhl-3	W04H10.3	0.34070791960799	AGIWUR39114
CB4856	CT	II	606350	606735	WBGene00206416	W04H10.5	W04H10.5	0.528171806956115	AGIWUR34751
CB4856	CT	II	614767	624543	WBGene00020207	T04B8.5	T04B8.5	-0.0766143381880857	AGIWUR9756;AGIWUR21808;AGIWUR36894;AGIWUR41802;AGIWUR41815
CB4856	CT	II	627506	628662	WBGene00020205	arrd-1	T04B8.3	0.525506747661082	AGIWUR21856
CB4856	CT	II	630075	631980	WBGene00020204	T04B8.2	T04B8.2	0.00695755295858547	AGIWUR16818
CB4856	CT	II	637346	639488	WBGene00019497	K07E8.6	K07E8.6	0.0870625023064999	AGIWUR26831;AGIWUR32972
CB4856	CT	II	641010	650367	WBGene00019498	K07E8.7	K07E8.7	-0.0774307534577314	AGIWUR8822
CB4856	CT	II	650964	654062	WBGene00019496	frpr-14	K07E8.5	0.074814140744329	AGIWUR41435
CB4856	CT	II	658669	659867	WBGene00019495	sdz-24	K07E8.3	-0.406489416034006	AGIWUR38836
CB4856	CT	II	660091	661796	WBGene00005557	sri-45	K07E8.9	-0.36409353693651	AGIWUR3487

CB4856	CT	II	667755	668444	WBGene00019494	K07E8.1	K07E8.1	-0.109996619452242	AGIWUR39454
CB4856	CT	II	669506	671125	WBGene00005558	sri-46	K07E8.11	-0.232752120317928
AGIWUR22123
CB4856	CT	II	671542	673844	WBGene00005556	sri-44	K07E8.12	0.042215376762877
AGIWUR6105;AGIWUR19807
CB4856	CT	II	675603	678347	WBGene00021457	Y39F10C.1	Y39F10C.1	-0.0905242598757905
AGIWUR14888
CB4856	CT	II	679025	679714	WBGene00020179	T02H6.9	T02H6.9	-0.0130133803981511	AGIWUR35739
CB4856	CT	II	679777	680712	WBGene00020180	T02H6.10	T02H6.10	-0.0606571306446382
AGIWUR41544
CB4856	CT	II	680832	681516	WBGene00020178	T02H6.8	T02H6.8	0.00824815409225188	AGIWUR11615
CB4856	CT	II	682626	683320	WBGene00020177	T02H6.7	T02H6.7	-0.181074569533342
AGIWUR2807;AGIWUR31704
CB4856	CT	II	683767	684116	WBGene00020176	T02H6.6	T02H6.6	-0.0214962930765168	AGIWUR30315
CB4856	CT	II	685234	689014	WBGene00020175	T02H6.5	T02H6.5	0.00197133807237293	AGIWUR10088
CB4856	CT	II	690049	691084	WBGene00020174	T02H6.4	T02H6.4	-0.069870513314668	AGIWUR35824
CB4856	CT	II	691881	693456	WBGene00020173	T02H6.3	T02H6.3	0.0807739269705863
AGIWUR16045;AGIWUR20376;AGIWUR25904;AGIWUR30689;AGIWUR39982
CB4856	CT	II	700482	701125	WBGene00020181	T02H6.11	T02H6.11	-0.0501044108885056
AGIWUR22052;AGIWUR36290
CB4856	CT	II	702107	708608	WBGene00020172	thoc-1	T02H6.2	-0.0856984870920865
AGIWUR13124;AGIWUR24834;AGIWUR29393;AGIWUR34043;AGIWUR39938;AGIWUR39989
CB4856	CT	II	709381	723699	WBGene00020171	T02H6.1	T02H6.1	-0.00925696776886209
AGIWUR1925;AGIWUR31002
CB4856	CT	II	723788	732513	WBGene00021456	Y39F10B.1	Y39F10B.1	0.132263189964799
AGIWUR578
CB4856	CT	II	734607	738666	WBGene00015614	C08G5.1	C08G5.1	-0.0525615106635762
AGIWUR7483;AGIWUR22598;AGIWUR26950
CB4856	CT	II	739598	740852	WBGene00015615	fbxc-48	C08G5.2	0.0770981409533089	AGIWUR33530
CB4856	CT	II	741595	745128	WBGene00015616	C08G5.3	C08G5.3	0.111863401788365	AGIWUR34991
CB4856	CT	II	745418	750164	WBGene00004926	snt-6	C08G5.4	0.160249031043101	AGIWUR20804
CB4856	CT	II	756195	765317	WBGene00044418	C08G5.7	C08G5.7	-0.00508095360047633
AGIWUR1376;AGIWUR2926;AGIWUR3173;AGIWUR7059;AGIWUR8584;AGIWUR11962;AGIWUR14089;AGIWUR15395;AGIWUR16770;AGIW
UR16869;AGIWUR17068;AGIWUR17422;AGIWUR31479;AGIWUR32296;AGIWUR33655;AGIWUR35357;AGIWUR38066;AGIWUR38457;AGI
WUR40764
CB4856	CT	II	770553	772117	WBGene00015618	C08G5.6	C08G5.6	0.420931893787442
AGIWUR31084;AGIWUR39491
CB4856	CT	II	775333	776780	WBGene00021452	Y39F10A.1	Y39F10A.1	0.280476761298953
AGIWUR13624

CB4856	CT	II	779380	779882	WBGene00269331	Y39F10A.5	Y39F10A.5	-0.417531158179417	AGIWUR43684
CB4856	CT	II	781884	783001	WBGene00021454	Y39F10A.3	Y39F10A.3	0.17152554999959	AGIWUR5346;AGIWUR32953;AGIWUR43193
CB4856	CT	II	787312	787994	WBGene00018507	F46F5.16	F46F5.16	-0.249044244538252	AGIWUR29948
CB4856	CT	II	788378	789175	WBGene00018506	F46F5.15	F46F5.15	0.667873901791052	AGIWUR11301
CB4856	CT	II	8e+05	801454	WBGene00018505	F46F5.14	F46F5.14	-0.325592217701328	AGIWUR12850
CB4856	CT	II	801728	802693	WBGene00023287	F46F5.3	F46F5.3	-0.23760399403936	AGIWUR4664;AGIWUR27371
CB4856	CT	II	803309	803717	WBGene00018504	F46F5.13	F46F5.13	-0.151438380988505	AGIWUR26178
CB4856	CT	II	805028	806438	WBGene00018495	F46F5.4	F46F5.4	-0.0547776495902177	AGIWUR10079
CB4856	CT	II	807372	808070	WBGene00018503	F46F5.12	F46F5.12	0.00109383988834377	AGIWUR5055
CB4856	CT	II	808757	811887	WBGene00018502	F46F5.11	F46F5.11	-0.101236700516459	AGIWUR38924
CB4856	CT	II	812141	813708	WBGene00018496	F46F5.5	F46F5.5	-0.274530106207715	AGIWUR5636
CB4856	CT	II	814452	814866	WBGene00018497	F46F5.6	F46F5.6	0.169783803264027	AGIWUR40706
CB4856	CT	II	818578	820683	WBGene00018498	F46F5.7	F46F5.7	-0.580467681924735	AGIWUR4943;AGIWUR18449
CB4856	CT	II	821408	821614	WBGene00018499	F46F5.8	F46F5.8	-0.137526352461494	AGIWUR38844
CB4856	CT	II	823829	825245	WBGene00018501	F46F5.10	F46F5.10	-0.11239145747261	AGIWUR22274
CB4856	CT	II	825848	827500	WBGene00017869	F28A10.1	F28A10.1	0.0147326987466077	AGIWUR9597
CB4856	CT	II	827891	830267	WBGene00017870	F28A10.2	F28A10.2	-0.460229503963373	AGIWUR44947
CB4856	CT	II	831069	831871	WBGene00017878	F28A10.10	F28A10.10	0.051386580342113	AGIWUR22041
CB4856	CT	II	833607	835270	WBGene00017877	F28A10.9	F28A10.9	0.0551641042720683	AGIWUR6656;AGIWUR38454
CB4856	CT	II	837719	838470	WBGene00017876	F28A10.8	F28A10.8	-0.0300675897102805	AGIWUR7741
CB4856	CT	II	840567	841113	WBGene00017871	F28A10.3	F28A10.3	-0.39659700187992	AGIWUR37007
CB4856	CT	II	844274	845085	WBGene00017872	F28A10.4	F28A10.4	0.224165936285893	

AGIWUR21076

CB4856	CT	II	845288	845686	WBGene00017875	F28A10.7	F28A10.7	0.123488610426794	AGIWUR26593
CB4856	CT	II	846864	848603	WBGene00017874	acdh-9	F28A10.6	-0.496887460360753	AGIWUR29074
CB4856	CT	II	849678	850654	WBGene00017873	F28A10.5	F28A10.5	0.216351999706752	AGIWUR39524
CB4856	CT	II	862927	866901	WBGene00021578	Y46B2A.3	Y46B2A.3	0.174007538360438	AGIWUR35944
CB4856	CT	II	868814	871699	WBGene00021576	fbxc-51	Y46B2A.1	0.846442661594544	AGIWUR29811
CB4856	CT	II	873176	879001	WBGene00000106	alg-2	T07D3.7	0.306905273896222	AGIWUR35217;AGIWUR37697;AGIWUR40213
CB4856	CT	II	881576	890710	WBGene00020307	T07D3.4	T07D3.4	-0.356707334217787	AGIWUR7775;AGIWUR43697
CB4856	CT	II	893863	897379	WBGene00020306	T07D3.3	T07D3.3	-0.0408123035466668	AGIWUR20372
CB4856	CT	II	898043	901164	WBGene00020311	T07D3.9	T07D3.9	-0.368715199214717	AGIWUR4965;AGIWUR39971
CB4856	CT	II	904425	906039	WBGene00020304	fbxa-200	T07D3.1	-0.372467739446639	AGIWUR27126
CB4856	CT	II	907535	909631	WBGene00019927	fbxc-24	R07C3.11	0.920164634416851	AGIWUR15211;AGIWUR21913;AGIWUR26453
CB4856	CT	II	909884	911592	WBGene00019926	fbxc-27	R07C3.10	0.316799147297163	AGIWUR10868
CB4856	CT	II	912003	913030	WBGene00019925	fbxc-31	R07C3.9	0.0253277727308969	AGIWUR14658
CB4856	CT	II	913680	915219	WBGene00019924	fbxc-33	R07C3.8	-0.346994577186848	AGIWUR1761
CB4856	CT	II	916051	917464	WBGene00019923	fbxc-29	R07C3.7	0.732030903122026	AGIWUR5918;AGIWUR10571
CB4856	CT	II	917624	919417	WBGene00045302	fbxc-30	R07C3.15	0.0241826166327588	AGIWUR27335
CB4856	CT	II	919575	920407	WBGene00269338	R07C3.16	R07C3.16	-0.413211611675887	AGIWUR17863
CB4856	CT	II	923821	925192	WBGene00019922	fbxc-28	R07C3.6	-0.0156664363853771	AGIWUR564;AGIWUR11358;AGIWUR20652
CB4856	CT	II	926496	927431	WBGene00019929	R07C3.13	R07C3.13	0.0661659288430592	AGIWUR7319
CB4856	CT	II	931171	932327	WBGene00019921	fbxc-34	R07C3.5	0.206999776828495	AGIWUR16572
CB4856	CT	II	933018	936989	WBGene00019920	acs-15	R07C3.4	-0.00225571152519599	AGIWUR7727
CB4856	CT	II	938849	940683	WBGene00019919	R07C3.3	R07C3.3	-0.241157965347004	AGIWUR9697

CB4856	CT	II	940983	943162	WBGene00019918	fbxc-36	R07C3.2	0.319461050739857	AGIWUR14198
CB4856	CT	II	944066	946157	WBGene00019917	clec-43	R07C3.1	-0.202144869920026	AGIWUR36973
CB4856	CT	II	946485	948191	WBGene00016303	fbxc-32	C32B5.10	-0.448039162851749	AGIWUR7472
CB4856	CT	II	948432	950507	WBGene00016304	fbxc-41	C32B5.11	0.070354439214289	AGIWUR28739
CB4856	CT	II	950842	952843	WBGene00016305	fbxc-57	C32B5.12	0.110609390290775	AGIWUR2248;AGIWUR3184;AGIWUR30209;AGIWUR31810
CB4856	CT	II	954953	956337	WBGene00016302	fbxc-7	C32B5.9	0.509319480197551	AGIWUR10362
CB4856	CT	II	956674	957961	WBGene00016301	fbxc-6	C32B5.8	0.156009847177177	AGIWUR33819
CB4856	CT	II	958918	960179	WBGene00016309	sdz-4	C32B5.16	0.0553080042877951	AGIWUR17898
CB4856	CT	II	960528	964260	WBGene00016300	C32B5.7	C32B5.7	-0.0222604535355231	AGIWUR15819;AGIWUR28262;AGIWUR37767
CB4856	CT	II	965576	967062	WBGene00016307	fbxc-18	C32B5.14	0.318320604312882	AGIWUR34121
CB4856	CT	II	968406	970523	WBGene00016299	C32B5.6	C32B5.6	0.101236301998927	AGIWUR6752
CB4856	CT	II	970645	972950	WBGene00016308	C32B5.15	C32B5.15	0.017438551746018	AGIWUR41227
CB4856	CT	II	974290	975511	WBGene00016298	fbxc-14	C32B5.5	0.413292552348002	AGIWUR22506
CB4856	CT	II	976208	978951	WBGene00044419	fbxc-17	C32B5.17	0.146316294104176	AGIWUR25275;AGIWUR40429
CB4856	CT	II	979423	980825	WBGene00016297	fbxc-15	C32B5.4	0.509046786870813	AGIWUR41541
CB4856	CT	II	981282	982435	WBGene00016296	fbxc-16	C32B5.3	-0.036049683188712	AGIWUR13034;AGIWUR18974;AGIWUR22521
CB4856	CT	II	986003	988666	WBGene00016295	fbxc-40	C32B5.1	0.469092972912702	AGIWUR33276;AGIWUR33872;AGIWUR41781
CB4856	CT	II	988737	991074	WBGene00018456	fbxc-39	F45D11.10	0.352881086604416	AGIWUR14227
CB4856	CT	II	991600	993638	WBGene00018455	fbxc-42	F45D11.9	-0.402292309741096	AGIWUR39826
CB4856	CT	II	993293	994189	WBGene00018454	fbxb-28	F45D11.8	0.228946416643701	AGIWUR11045;AGIWUR15570
CB4856	CT	II	1000437	1001449	WBGene00018458	fbxb-30	F45D11.13	0.400558324333689	AGIWUR44765
CB4856	CT	II	1001703	1002437	WBGene00018452	F45D11.5	F45D11.5	0.295438657755804	AGIWUR28527
CB4856	CT	II	1021505	1023528	WBGene00018448	F45D11.1	F45D11.1	-0.549203486179022	AGIWUR11280;AGIWUR21120

CB4856	CT	II	1023693	1024784	WBGene00019700	btb-11	M01D1.3	0.17863709234965	AGIWUR26307
CB4856	CT	II	1028987	1033664	WBGene00019699	math-34	M01D1.2	1.86336697401181	AGIWUR42003
CB4856	CT	II	1039697	1040564	WBGene00019702	fbxb-39	M01D1.7	-0.0704317590335144	AGIWUR12176
CB4856	CT	II	1041321	1042327	WBGene00019703	fbxb-41	M01D1.8	-0.141164011005287	AGIWUR34886
CB4856	CT	II	1042969	1044025	WBGene00019705	fbxb-45	M01D1.10	0.0266131767371487	AGIWUR40701
CB4856	CT	II	1046505	1048276	WBGene00006195	str-148	M01D1.1	-0.0780754689142622	AGIWUR17949
CB4856	CT	II	1048863	1049949	WBGene00019704	fbxb-40	M01D1.9	0.310228318676804	AGIWUR9353;AGIWUR20714
CB4856	CT	II	1057212	1059280	WBGene00019311	K02E7.6	K02E7.6	-0.389008832267538	AGIWUR13530;AGIWUR14487
CB4856	CT	II	1059664	1060802	WBGene00019313	btb-10	K02E7.9	-0.0902363555163523	AGIWUR2350
CB4856	CT	II	1065566	1066998	WBGene00019314	K02E7.10	K02E7.10	0.0258859892620293	AGIWUR12881
CB4856	CT	II	1070640	1071490	WBGene00019316	K02E7.12	K02E7.12	-0.0903916650388386	AGIWUR28681
CB4856	CT	II	1074751	1075505	WBGene00019315	K02E7.11	K02E7.11	0.00400905655453343	AGIWUR7722
CB4856	CT	II	1083159	1083929	WBGene00019306	K02E7.1	K02E7.1	0.57491714339109	AGIWUR12424;AGIWUR33478;AGIWUR36865
CB4856	CT	II	1092006	1097899	WBGene00004902	snf-3	T13B5.1	0.142839766100209	AGIWUR19715
CB4856	CT	II	1097926	1100819	WBGene00020471	pho-14	T13B5.3	-0.0545520426478223	AGIWUR10194;AGIWUR28391
CB4856	CT	II	1101371	1104509	WBGene00020476	T13B5.9	T13B5.9	-0.142838897673819	AGIWUR26618
CB4856	CT	II	1104945	1106925	WBGene00000617	col-40	T13B5.4	0.0785952107731704	AGIWUR17459
CB4856	CT	II	1108841	1110992	WBGene00020472	lips-11	T13B5.5	0.0403510348260658	AGIWUR21783
CB4856	CT	II	1112189	1114226	WBGene00020473	lips-12	T13B5.6	-0.101236500886156	AGIWUR1562
CB4856	CT	II	1115641	1118033	WBGene00020474	lips-13	T13B5.7	0.0831398618595669	AGIWUR32051
CB4856	CT	II	1121018	1123259	WBGene00020475	sut-1	T13B5.8	0.0682070298079882	AGIWUR17492
CB4856	CT	II	1123534	1127791	WBGene00021103	eri-3	W09B6.3	-0.167481899017279	AGIWUR1524
CB4856	CT	II	1127976	1132352	WBGene00006387	taf-6.1	W09B6.2	-0.120767898313879	AGIWUR14620
CB4856	CT	II	1132302	1134240	WBGene00021104	W09B6.4	W09B6.4	-0.0446319456618873	AGIWUR11511;AGIWUR20518;AGIWUR21674;AGIWUR23256;AGIWUR28565;AGIWUR34057;AGIWUR36897
CB4856	CT	II	1136781	1152701	WBGene00004076	pod-2	W09B6.1	0.330004736365201	AGIWUR4199;AGIWUR14789
CB4856	CT	II	1163575	1165488	WBGene00020740	T23F4.2	T23F4.2	0.418021786065866	AGIWUR1509
CB4856	CT	II	1166490	1169168	WBGene00020739	T23F4.1	T23F4.1	-0.498231120045105	AGIWUR11835
CB4856	CT	II	1169546	1172130	WBGene00020741	dlhd-1	T23F4.3	1.27113684727707	AGIWUR44663
CB4856	CT	II	1175976	1179323	WBGene00003545	nas-27	T23F4.4	0.0737969225308088	AGIWUR14074

CB4856	CT	II	1197374	1230782	WBGene00018734	F53A10.2	F53A10.2	0.0256924690625464	AGIWUR30798;AGIWUR41285;AGIWUR43186
CB4856	CT	II	1233236	1252359	WBGene00018572	lin-42	F47F6.1	-0.0255505624969365	AGIWUR15290;AGIWUR36002
CB4856	CT	II	1255937	1258353	WBGene00018575	clec-119	F47F6.5	-0.22899171668816	AGIWUR24296;AGIWUR41961
CB4856	CT	II	1259365	1262998	WBGene00023410	F47F6.9	F47F6.9	0.0465483481439537	AGIWUR32467;AGIWUR38054
CB4856	CT	II	1262987	1266890	WBGene00018573	oac-30	F47F6.3	0.0661973597184451	AGIWUR11993;AGIWUR35046
CB4856	CT	II	1269540	1271914	WBGene00005813	srw-66	Y57G7A.4	0.0396426830900936	AGIWUR7731;AGIWUR8027
CB4856	CT	II	1273169	1275539	WBGene00021968	Y57G7A.5	Y57G7A.5	0.129646903328439	AGIWUR11546
CB4856	CT	II	1275752	1276872	WBGene00021974	Y57G7A.11	Y57G7A.11	-0.145756973100027	AGIWUR17548
CB4856	CT	II	1278650	1281039	WBGene00023499	srz-63	Y57G7A.12	0.000385831153408844	AGIWUR2017;AGIWUR41865;AGIWUR43322
CB4856	CT	II	1281974	1285614	WBGene00021969	Y57G7A.6	Y57G7A.6	0.341394794125341	AGIWUR17694
CB4856	CT	II	1285797	1286371	WBGene00021967	Y57G7A.3	Y57G7A.3	-0.311007179848529	AGIWUR9015;AGIWUR19309;AGIWUR20491;AGIWUR25151;AGIWUR25756;AGIWUR39388
CB4856	CT	II	1288735	1293257	WBGene00021970	srz-64	Y57G7A.7	0.137980511814238	AGIWUR34238
CB4856	CT	II	1296078	1296746	WBGene00021966	Y57G7A.2	Y57G7A.2	0.0240489291046853	AGIWUR8814
CB4856	CT	II	1298469	1299716	WBGene00021965	Y57G7A.1	Y57G7A.1	-0.292830503127295	AGIWUR20526
CB4856	CT	II	1300064	1301641	WBGene00021972	glb-31	Y57G7A.9	-0.0559714687974004	AGIWUR8263
CB4856	CT	II	1302497	1306136	WBGene00021973	emc-2	Y57G7A.10	-0.0983347225853173	AGIWUR4278
CB4856	CT	II	1306240	1310133	WBGene00000112	alh-6	F56D12.1	-0.274311304012134	AGIWUR21026;AGIWUR21258;AGIWUR24232;AGIWUR28867;AGIWUR34752;AGIWUR36615;AGIWUR43212
CB4856	CT	II	1310518	1320437	WBGene00018974	fcho-1	F56D12.6	0.155710909461497	AGIWUR3322;AGIWUR10578
CB4856	CT	II	1321037	1323170	WBGene00006924	vig-1	F56D12.5	0.276398985384995	AGIWUR2756;AGIWUR18673;AGIWUR22674;AGIWUR30958;AGIWUR33919;AGIWUR35778;AGIWUR35851;AGIWUR41568
CB4856	CT	II	1326995	1350136	WBGene00002176	jip-1	F56D12.4	0.40558795924319	

AGIWUR11259
CB4856 CT II 1350720 1355641 WBGene00015728 C13A10.1 C13A10.1 0.0960196648548202
AGIWUR42053
CB4856 CT II 1357177 1358005 WBGene00015729 C13A10.2 C13A10.2 -0.0108845100162611
AGIWUR20549
CB4856 CT II 1368103 1374988 WBGene00018819 rog-1 F54D12.6 0.0963879696719272
AGIWUR42436;AGIWUR43632
CB4856 CT II 1377628 1378359 WBGene00044424 F54D12.10 F54D12.10 -0.137726071550282
AGIWUR38508
CB4856 CT II 1380944 1381713 WBGene00018822 F54D12.9 F54D12.9 -0.404815910201867
AGIWUR30305
CB4856 CT II 1381807 1385380 WBGene00018820 F54D12.7 F54D12.7 -0.0289448885583514
AGIWUR17467
CB4856 CT II 1386888 1391567 WBGene00000008 aat-7 F54D12.3 -0.461223775205037
AGIWUR12832
CB4856 CT II 1393488 1394105 WBGene00044425 F54D12.11 F54D12.11 -0.0468385982981143
AGIWUR1470;AGIWUR23949;AGIWUR28151;AGIWUR37605;AGIWUR41989
CB4856 CT II 1395004 1398038 WBGene00018821 F54D12.8 F54D12.8 0.0390018078276118
AGIWUR36291
CB4856 CT II 1398761 1400030 WBGene00018816 F54D12.2 F54D12.2 -0.109643405249264
AGIWUR41051
CB4856 CT II 1400405 1401371 WBGene00018815 F54D12.1 F54D12.1 -0.0696553619293399
AGIWUR2284
CB4856 CT II 1401774 1402134 WBGene00021786 Y51H7C.8 Y51H7C.8 -0.0704261110357933
AGIWUR23325
CB4856 CT II 1402569 1403215 WBGene00021785 Y51H7C.7 Y51H7C.7 -0.189416482307173
AGIWUR3019
CB4856 CT II 1403321 1409118 WBGene00021784 cogc-4 Y51H7C.6 -0.190374224747954
AGIWUR9683;AGIWUR21974;AGIWUR26256;AGIWUR35503;AGIWUR35651;AGIWUR37749;AGIWUR41187
CB4856 CT II 1409184 1411472 WBGene00021787 Y51H7C.9 Y51H7C.9 -0.1725328424557
AGIWUR30133
CB4856 CT II 1412409 1420150 WBGene00021783 Y51H7C.5 Y51H7C.5 -0.250913061230882
AGIWUR13720;AGIWUR20788;AGIWUR28870
CB4856 CT II 1420422 1423668 WBGene00021788 Y51H7C.10 Y51H7C.10 0.570964208996573
AGIWUR18507;AGIWUR32455;AGIWUR33386;AGIWUR37153;AGIWUR38549
CB4856 CT II 1425758 1437499 WBGene00021789 nol-6 Y51H7C.11 0.0225843655920421
AGIWUR11653;AGIWUR20656
CB4856 CT II 1438490 1439697 WBGene00021781 Y51H7C.3 Y51H7C.3 -0.125740171360114
AGIWUR8694;AGIWUR17307;AGIWUR19226;AGIWUR22706;AGIWUR27660;AGIWUR42418

CB4856	CT	II	1440066 1446746 WBGene00021790	Y51H7C.12	Y51H7C.12	0.538591655929382
AGIWUR27053
CB4856	CT	II	1459140 1461307 WBGene00044439	Y51H7C.15	Y51H7C.15	0.0493620361079028
AGIWUR793;AGIWUR1636;AGIWUR35924;AGIWUR36322;AGIWUR41340
CB4856	CT	II	1463268 1466546 WBGene00021791	Y51H7C.13	Y51H7C.13	-0.49808122275723
AGIWUR16739
CB4856	CT	II	1467727 1469604 WBGene00021779	Y51H7C.1	Y51H7C.1	-0.760004236722761
AGIWUR40157
CB4856	CT	II	1472625 1473581 WBGene00044643	H17B01.5	H17B01.5	0.452203624045288
AGIWUR42937
CB4856	CT	II	1484094 1485764 WBGene00019208	lips-14 H17B01.3	-0.878618564643556
AGIWUR28063
CB4856	CT	II	1489930 1500330 WBGene00019209	emc-1	H17B01.4	-0.08410429016336
AGIWUR5311;AGIWUR12574;AGIWUR14027
CB4856	CT	II	1504566 1509831 WBGene00019207	fgt-1	H17B01.1	0.194481895864577
AGIWUR3961;AGIWUR9439;AGIWUR14822;AGIWUR17175;AGIWUR35685;AGIWUR37584
CB4856	CT	II	1510288 1517454 WBGene00021772	math-45 Y51H7BM.1	0.376184800144836
AGIWUR22347
CB4856	CT	II	1519222 1520328 WBGene00044645	Y51H7BR.8	Y51H7BR.8	0.0357349242838083
AGIWUR13474;AGIWUR17530;AGIWUR23980;AGIWUR31130;AGIWUR37906
CB4856	CT	II	1520384 1521842 WBGene00005704	sru-41	Y51H7BR.6	-0.0335212554863471
AGIWUR12775;AGIWUR36218
CB4856	CT	II	1523565 1525598 WBGene00021778	Y51H7BR.7	Y51H7BR.7	0.00998475768819951
AGIWUR41969
CB4856	CT	II	1529359 1535208 WBGene00021776	Y51H7BR.4	Y51H7BR.4	-0.0821401142124394
AGIWUR5447;AGIWUR16636;AGIWUR18616;AGIWUR23149;AGIWUR24516;AGIWUR25783;AGIWUR31085;AGIWUR38742;AGIWUR39425
CB4856	CT	II	1536231 1537275 WBGene00021774	fbxb-43 Y51H7BR.2	0.14087635076005
AGIWUR17877
CB4856	CT	II	1537621 1538666 WBGene00021773	fbxb-42 Y51H7BR.1	0.0690292928741973
AGIWUR31502
CB4856	CT	II	1538891 1539901 WBGene00019416	fbxb-44 K05F6.5 0.116565717948097
AGIWUR4279;AGIWUR17677
CB4856	CT	II	1543482 1545415 WBGene00019415	K05F6.4 K05F6.4 -0.140459518387803	AGIWUR24379
CB4856	CT	II	1545925 1546991 WBGene00045486	K05F6.12	K05F6.12	0.226837280813927
AGIWUR28538
CB4856	CT	II	1547060 1548182 WBGene00269420	K05F6.16	K05F6.16	-0.0513440701672749
AGIWUR7272
CB4856	CT	II	1555257 1556335 WBGene00019418	fbxb-54 K05F6.7 -0.0344559838511875	AGIWUR5734
CB4856	CT	II	1567324 1567746 WBGene00019419	K05F6.8 K05F6.8 -0.0565696507288354

AGIWUR4026;AGIWUR22442;AGIWUR26126;AGIWUR30746;AGIWUR36813;AGIWUR44179
CB4856 CT II 1570065 1571134 WBGene00019420 fbxb-46 K05F6.9 -0.0379270764438431 AGIWUR5730
CB4856 CT II 1572522 1573300 WBGene00269419 K05F6.15 K05F6.15 0.143341787307428
AGIWUR20317
CB4856 CT II 1574557 1576115 WBGene00019421 K05F6.10 K05F6.10 0.303970985525398
AGIWUR3384;AGIWUR5668;AGIWUR9394;AGIWUR24867
CB4856 CT II 1577471 1579986 WBGene00020326 math-38 T07H3.3 1.99427903978718 AGIWUR28995
CB4856 CT II 1582876 1584240 WBGene00020327 clec-21 T07H3.4 -0.200182882964563
AGIWUR7239;AGIWUR35909
CB4856 CT II 1588542 1589945 WBGene00044429 T07H3.7 T07H3.7 -0.124850615198759
AGIWUR11204;AGIWUR19451;AGIWUR40079
CB4856 CT II 1593480 1595237 WBGene00020328 clec-20 T07H3.5 0.265150200273092 AGIWUR37368
CB4856 CT II 1598029 1599488 WBGene00020325 bath-46 T07H3.2 0.271948416331888 AGIWUR648
CB4856 CT II 1599471 1600714 WBGene00020329 bath-26 T07H3.6 -0.417224013276943 AGIWUR27574
CB4856 CT II 1601789 1604618 WBGene00006464 bath-47 T07H3.1 0.16455773037525 AGIWUR1261
CB4856 CT II 1604666 1606143 WBGene00015595 bath-33 C08E3.3 0.193286656065786 AGIWUR35175
CB4856 CT II 1606595 1607603 WBGene00015596 fbxa-161 C08E3.4 1.43149092186227
AGIWUR37564
CB4856 CT II 1607869 1609014 WBGene00015597 fbxa-162 C08E3.5 0.864771217137826
AGIWUR13440
CB4856 CT II 1609256 1610715 WBGene00015598 fbxa-163 C08E3.6 1.13378308408527
AGIWUR10816;AGIWUR28346;AGIWUR37259
CB4856 CT II 1611331 1612510 WBGene00015594 btb-15 C08E3.2 0.79389285087373 AGIWUR30529
CB4856 CT II 1612788 1614059 WBGene00015599 fbxa-164 C08E3.7 0.840872973713616
AGIWUR28121;AGIWUR30670;AGIWUR34271
CB4856 CT II 1614793 1616463 WBGene00015600 fbxa-165 C08E3.8 -0.0109280079247451
AGIWUR14879
CB4856 CT II 1616912 1619004 WBGene00015601 fbxa-166 C08E3.9 0.104718091188016
AGIWUR21789
CB4856 CT II 1619894 1621261 WBGene00015602 fbxa-158 C08E3.10 -0.0818205548469031
AGIWUR1075;AGIWUR11977;AGIWUR27950
CB4856 CT II 1623907 1624672 WBGene00015604 fbxa-160 C08E3.12 0.263512776083937
AGIWUR26010
CB4856 CT II 1625966 1626883 WBGene00044415 C08E3.14 C08E3.14 -0.0547717853931387
AGIWUR16413;AGIWUR35631
CB4856 CT II 1627479 1627883 WBGene00044416 C08E3.15 C08E3.15 1.15047335326541
AGIWUR12234;AGIWUR14033;AGIWUR20688
CB4856 CT II 1639213 1640596 WBGene00005293 srh-72 ZC204.5 0.22206814655444 AGIWUR31572
CB4856 CT II 1644249 1644814 WBGene00022556 ZC204.6 ZC204.6 -0.20910242065102 AGIWUR18821

CB4856	CT	II	1645410	1646299	WBGene00194714	ZC204.17	ZC204.17	-0.0277100545372795	AGIWUR23907
CB4856	CT	II	1646597	1647568	WBGene00044695	fbxb-27	ZC204.16	-0.035388533499539	AGIWUR5799;AGIWUR21143
CB4856	CT	II	1647908	1648865	WBGene00022558	fbxb-21	ZC204.8	-0.247561861084572	AGIWUR3003;AGIWUR17859
CB4856	CT	II	1649138	1650197	WBGene00022559	fbxb-20	ZC204.9	0.0188327419915995	AGIWUR31394
CB4856	CT	II	1650819	1651580	WBGene00022560	fbxb-16	ZC204.10	0.347918682454507	AGIWUR12940
CB4856	CT	II	1652510	1653497	WBGene00022557	fbxb-15	ZC204.7	0.091067050986763	AGIWUR27611
CB4856	CT	II	1653775	1654732	WBGene00022561	btb-13	ZC204.11	0.126572602219703	AGIWUR16801
CB4856	CT	II	1655217	1656148	WBGene00022555	btb-12	ZC204.3	0.224198944083456	AGIWUR30043
CB4856	CT	II	1656263	1657302	WBGene00022562	ZC204.12	ZC204.12	-0.221636923243166	AGIWUR4325;AGIWUR23512;AGIWUR25253;AGIWUR38231
CB4856	CT	II	1663699	1665764	WBGene00022554	duxl-1	ZC204.2	-0.210707708777929	AGIWUR8677;AGIWUR19314
CB4856	CT	II	1666325	1667482	WBGene00022553	ZC204.1	ZC204.1	0.069990530293742	AGIWUR14813;AGIWUR30212;AGIWUR36411;AGIWUR42000
CB4856	CT	II	1667522	1669013	WBGene00005844	srw-97	ZC204.15	0.0304769945195149	AGIWUR4173
CB4856	CT	II	1670833	1674337	WBGene00003734	nhx-6	F58E1.6	-0.979745530480876	AGIWUR15556
CB4856	CT	II	1676388	1677439	WBGene00019037	fbxb-17	F58E1.5	0.140690331463693	AGIWUR9617
CB4856	CT	II	1677449	1679252	WBGene00019038	irld-37	F58E1.7	0.23027494960402	AGIWUR21516
CB4856	CT	II	1679381	1682134	WBGene00019036	irld-36	F58E1.4	-0.419122184013254	AGIWUR38369
CB4856	CT	II	1682518	1683655	WBGene00019035	fbxb-26	F58E1.3	0.298586603842937	AGIWUR8917
CB4856	CT	II	1684048	1685091	WBGene00019034	fbxb-25	F58E1.2	-0.0148067545235495	AGIWUR21302
CB4856	CT	II	1688235	1689172	WBGene00019040	fbxb-19	F58E1.9	0.275830414062585	AGIWUR2980;AGIWUR24623;AGIWUR33051
CB4856	CT	II	1689696	1691077	WBGene00019041	fbxc-22	F58E1.10	0.204348715983402	AGIWUR38601
CB4856	CT	II	1691557	1692907	WBGene00019042	fbxc-21	F58E1.11	-0.0120977869793144	AGIWUR28792
CB4856	CT	II	1693296	1694615	WBGene00019043	fbxc-26	F58E1.12	-0.324350370235129	AGIWUR27871
CB4856	CT	II	1694815	1695827	WBGene00019044	F58E1.13	F58E1.13	-0.257718152540821	AGIWUR42519
CB4856	CT	II	1702384	1703463	WBGene00019045	fbxb-47	F58E1.14	-0.13312579789851	AGIWUR1950

CB4856 CT II 1704904 1706476 WBGene00018442 sdz-18 F45C12.11 0.136661257140728
AGIWUR31803
CB4856 CT II 1706709 1707720 WBGene00018441 math-29 F45C12.10 0.00382233357562578
AGIWUR29511;AGIWUR39167
CB4856 CT II 1708432 1709696 WBGene00018440 F45C12.9 F45C12.9 -0.117880193577627
AGIWUR37829
CB4856 CT II 1710259 1711417 WBGene00018439 fbxa-184 F45C12.8 -0.323791547880374
AGIWUR11427;AGIWUR15075;AGIWUR21331;AGIWUR23042;AGIWUR42225
CB4856 CT II 1713238 1714397 WBGene00018443 btb-7 F45C12.12 -0.427258192659925
AGIWUR35441
CB4856 CT II 1715162 1716497 WBGene00018438 btb-6 F45C12.7 -0.194960814396124
AGIWUR9254
CB4856 CT II 1718175 1719253 WBGene00018444 fbxb-48 F45C12.13 0.0265316854999531
AGIWUR32498
CB4856 CT II 1721323 1722423 WBGene00018445 fbxb-5 F45C12.14 0.221565625535629
AGIWUR6059;AGIWUR14552;AGIWUR42708
CB4856 CT II 1722770 1724186 WBGene00018437 btb-8 F45C12.6 0.185065994788189
AGIWUR19396
CB4856 CT II 1726141 1727106 WBGene00269388 F45C12.17 F45C12.17 0.0789983419328335
AGIWUR7469;AGIWUR23367
CB4856 CT II 1728506 1730065 WBGene00018435 btb-9 F45C12.4 0.0392665507757339
AGIWUR20385;AGIWUR29933;AGIWUR32157
CB4856 CT II 1730403 1732046 WBGene00018434 ceh-81 F45C12.3 0.0808468787569598
AGIWUR25864
CB4856 CT II 1732276 1733291 WBGene00018433 ceh-82 F45C12.2 -0.125470481992716
AGIWUR16980
CB4856 CT II 1733718 1736533 WBGene00018446 ceh-83 F45C12.15 -0.172522067664016
AGIWUR43133
CB4856 CT II 1736866 1738944 WBGene00018447 irld-9 F45C12.16 -0.150874417954765
AGIWUR1756
CB4856 CT II 1739076 1741013 WBGene00018432 irld-33 F45C12.1 0.270396484900003
AGIWUR35733
CB4856 CT II 1744483 1745859 WBGene00018105 clec-19 F36H5.6 0.0777628686961158 AGIWUR34948
CB4856 CT II 1746189 1747582 WBGene00006223 str-179 F36H5.7 0.0332570315773104
AGIWUR6697;AGIWUR7711;AGIWUR18141;AGIWUR18259;AGIWUR19978;AGIWUR25714;AGIWUR34971;AGIWUR42875;AGIWUR43866
CB4856 CT II 1748378 1749943 WBGene00206519 F36H5.13 F36H5.13 0.335713235064251
AGIWUR24315
CB4856 CT II 1752749 1753090 WBGene00018107 F36H5.9 F36H5.9 -0.224647969606178 AGIWUR28515
CB4856 CT II 1753526 1754439 WBGene00018109 fbxb-12 F36H5.11 0.013667934883161

AGIWUR21376
CB4856	CT	II	1756820 1757761 WBGene00018108	F36H5.10	F36H5.10	0.0670546603415882
AGIWUR4473;AGIWUR14070;AGIWUR15873;AGIWUR17539;AGIWUR21671;AGIWUR22520;AGIWUR33979;AGIWUR38675;AGIWUR39220
CB4856	CT	II	1758326 1760667 WBGene00018104	fbxb-53 F36H5.5 0.945400305606904	AGIWUR9607
CB4856	CT	II	1761839 1762906 WBGene00018103	F36H5.4 F36H5.4 0.056396703001214
AGIWUR19793;AGIWUR21756;AGIWUR22590;AGIWUR25767;AGIWUR34490
CB4856	CT	II	1763372 1766555 WBGene00018102	math-28 F36H5.3 -0.13321418282052
AGIWUR2347;AGIWUR15007;AGIWUR27128
CB4856	CT	II	1767802 1777394 WBGene00018101	math-27 F36H5.2 -0.241382042030857
AGIWUR11186;AGIWUR32824
CB4856	CT	II	1772085 1778526 WBGene00015608	math-3	C08F1.4 -0.36350698784335
AGIWUR32321;AGIWUR38372
CB4856	CT	II	1778772 1782409 WBGene00015609	math-4	C08F1.5 -0.745286859406396	AGIWUR13924
CB4856	CT	II	1783451 1784481 WBGene00044417	C08F1.11	C08F1.11	0.646547256240657
AGIWUR27089
CB4856	CT	II	1785270 1786309 WBGene00015613	C08F1.10	C08F1.10	0.227085627122595
AGIWUR3444;AGIWUR13602;AGIWUR23231;AGIWUR23253;AGIWUR27163;AGIWUR40491
CB4856	CT	II	1787222 1788187 WBGene00015610	C08F1.6 C08F1.6 0.874123328146414	AGIWUR11918
CB4856	CT	II	1789181 1790217 WBGene00015607	fbxb-13 C08F1.3 0.493866813801359	AGIWUR38059
CB4856	CT	II	1791981 1792962 WBGene00006088	str-21	C08F1.2 0.206111012915491	AGIWUR15309
CB4856	CT	II	1797852 1801377 WBGene00015606	math-2	C08F1.1 -0.114916199563689	AGIWUR37774
CB4856	CT	II	1802107 1803302 WBGene00006089	str-22	C08F1.7 0.24174690181645
AGIWUR10773;AGIWUR24537
CB4856	CT	II	1817281 1818400 WBGene00020362	fbxb-10 T08E11.6	0.322354446901032
AGIWUR7747
CB4856	CT	II	1818889 1820094 WBGene00020363	fbxa-3	T08E11.7	-0.0109330382694468
AGIWUR29418
CB4856	CT	II	1821026 1823034 WBGene00020361	fbxc-19 T08E11.5	-0.0904753355779775
AGIWUR22962;AGIWUR26801;AGIWUR29434;AGIWUR38598;AGIWUR39671;AGIWUR42018
CB4856	CT	II	1824629 1830195 WBGene00020360	math-41 T08E11.4	-0.49065001007758
AGIWUR14454
CB4856	CT	II	1830632 1831789 WBGene00020359	math-40 T08E11.3	-0.545656122500995
AGIWUR21133
CB4856	CT	II	1832343 1833518 WBGene00020358	math-39 T08E11.2	-0.101079631683898
AGIWUR14134
CB4856	CT	II	1833627 1835783 WBGene00020364	T08E11.8	T08E11.8	-0.0322601122479833
AGIWUR7322
CB4856	CT	II	1840975 1842942 WBGene00016884	C52E2.5 C52E2.5 0.243180739001517	AGIWUR28213
CB4856	CT	II	1843165 1846234 WBGene00016883	C52E2.4 C52E2.4 0.139583801940655	AGIWUR9343

CB4856 CT II 1846990 1848154 WBGene00016885 fbxb-97 C52E2.6 -0.0982679323511189 AGIWUR4720
CB4856 CT II 1849430 1850403 WBGene00016886 fbxb-96 C52E2.7 -0.0931266347459854 AGIWUR33334
CB4856 CT II 1852803 1853092 WBGene00016881 C52E2.2 C52E2.2 -2.24159443132771 AGIWUR33694
CB4856 CT II 1854394 1855360 WBGene00016880 fbxb-95 C52E2.1 -0.252489450614335
AGIWUR16918;AGIWUR31962;AGIWUR36994
CB4856 CT II 1862634 1863782 WBGene00015830 fbxb-98 C16C4.6 -0.0961067112579608 AGIWUR26970
CB4856 CT II 1864578 1864884 WBGene00044359 C16C4.17 C16C4.17 0.0795526712207266
AGIWUR5683;AGIWUR26687;AGIWUR28800;AGIWUR32417;AGIWUR33559
CB4856 CT II 1866386 1867894 WBGene00015829 math-15 C16C4.5 -0.737425564892327 AGIWUR14519
CB4856 CT II 1869410 1870803 WBGene00015828 math-14 C16C4.4 -0.536009019817301 AGIWUR20950
CB4856 CT II 1873480 1875106 WBGene00015840 math-11 C16C4.16 0.230656023167878
AGIWUR33822
CB4856 CT II 1875958 1877237 WBGene00015827 math-13 C16C4.3 -0.128059416677485 AGIWUR291
CB4856 CT II 1877579 1878763 WBGene00015832 math-16 C16C4.8 -0.0332037788874491 AGIWUR13056
CB4856 CT II 1879590 1880708 WBGene00015833 math-17 C16C4.9 -0.131370911918383 AGIWUR27825
CB4856 CT II 1881611 1882805 WBGene00015834 math-5 C16C4.10 -0.132893667337727
AGIWUR44984
CB4856 CT II 1883596 1884735 WBGene00015835 math-6 C16C4.11 -0.0944832129889717
AGIWUR35980
CB4856 CT II 1885439 1886479 WBGene00015836 math-7 C16C4.12 -0.324998832491334
AGIWUR25687
CB4856 CT II 1887209 1889171 WBGene00015837 math-8 C16C4.13 0.149657708814011
AGIWUR20908
CB4856 CT II 1889909 1890782 WBGene00015838 math-9 C16C4.14 -0.0339022155418984
AGIWUR34335
CB4856 CT II 1890851 1891699 WBGene00015826 math-12 C16C4.2 0.149186437156372 AGIWUR17591
CB4856 CT II 1900238 1901921 WBGene00016719 math-24 C46F9.3 0.0893050845853658 AGIWUR32651
CB4856 CT II 1902281 1904446 WBGene00016718 math-23 C46F9.2 0.168139176488204 AGIWUR5837
CB4856 CT II 1905429 1907775 WBGene00016717 math-22 C46F9.1 -0.310095981052247
AGIWUR15993;AGIWUR33102
CB4856 CT II 1908161 1909735 WBGene00018662 math-30 F52C6.5 0.0788657501136178 AGIWUR14125
CB4856 CT II 1912450 1912841 WBGene00018663 math-31 F52C6.6 0.0766980620191546 AGIWUR328
CB4856 CT II 1913605 1916376 WBGene00018664 bath-11 F52C6.7 -0.161045110909466 AGIWUR41813
CB4856 CT II 1916713 1917958 WBGene00018665 bath-4 F52C6.8 -0.349646590015915 AGIWUR32571
CB4856 CT II 1918319 1919439 WBGene00018666 bath-6 F52C6.9 -0.266920299357505
AGIWUR1207;AGIWUR16786;AGIWUR29145;AGIWUR41161
CB4856 CT II 1919926 1921111 WBGene00018667 bath-7 F52C6.10 -0.233001748534825
AGIWUR5473
CB4856 CT II 1921452 1922744 WBGene00018668 bath-2 F52C6.11 -0.352106272054796

AGIWUR14381
CB4856 CT II 1923140 1923827 WBGene00018661 F52C6.4 F52C6.4 -0.0196480400073549
AGIWUR2439;AGIWUR6117;AGIWUR13431;AGIWUR24905;AGIWUR32132
CB4856 CT II 1924759 1925752 WBGene00018660 F52C6.3 F52C6.3 -0.345910000803287
AGIWUR10072;AGIWUR19332
CB4856 CT II 1926462 1927347 WBGene00018659 F52C6.2 F52C6.2 -0.143440370176056 AGIWUR29257
CB4856 CT II 1927879 1928734 WBGene00018658 bath-22 F52C6.1 -0.0756073858170563
AGIWUR7050;AGIWUR16188;AGIWUR18552;AGIWUR42126
CB4856 CT II 1929149 1930086 WBGene00018669 F52C6.12 F52C6.12 -0.308214875485298
AGIWUR9215;AGIWUR11588;AGIWUR21193;AGIWUR26570;AGIWUR42282
CB4856 CT II 1930190 1930747 WBGene00018670 F52C6.13 F52C6.13 -0.163151920163379
AGIWUR2092
CB4856 CT II 1930991 1934507 WBGene00018671 F52C6.14 F52C6.14 -0.0427389652969348
AGIWUR11418
CB4856 CT II 1937063 1940147 WBGene00235352 ZK250.13 ZK250.13 0.416620408039096
AGIWUR42114
CB4856 CT II 1944058 1945168 WBGene00235354 ZK250.14 ZK250.14 0.136382262584445
AGIWUR9209;AGIWUR37083
CB4856 CT II 1953680 1955668 WBGene00022690 math-49 ZK250.7 -0.0537818580110976 AGIWUR24489
CB4856 CT II 1956050 1958255 WBGene00022691 math-50 ZK250.8 -0.256063940175725 AGIWUR41755
CB4856 CT II 1987285 1990667 WBGene00021193 math-44 Y16E11A.1 0.188943331793729
AGIWUR42798
CB4856 CT II 1994047 1995701 WBGene00016556 math-21 C40D2.3 -0.0386448406161604
AGIWUR9190;AGIWUR26554
CB4856 CT II 1996537 1998531 WBGene00016555 math-20 C40D2.2 -0.0914659503497024 AGIWUR12840
CB4856 CT II 1999743 2000820 WBGene00016557 ceh-84 C40D2.4 -0.148541972242278 AGIWUR25294
CB4856 CT II 2011485 2012838 WBGene00019137 ceh-85 F59H6.6 0.0351351343922284 AGIWUR34788
CB4856 CT II 2012998 2013386 WBGene00269394 F59H6.15 F59H6.15 -0.327260744004256
AGIWUR9029
CB4856 CT II 2019654 2022441 WBGene00019133 F59H6.2 F59H6.2 0.108441991184561 AGIWUR29740
CB4856 CT II 2025642 2026610 WBGene00019138 bath-21 F59H6.8 0.103442944772407 AGIWUR24725
CB4856 CT II 2027612 2028812 WBGene00019139 bath-1 F59H6.9 -0.22225435351733 AGIWUR35302
CB4856 CT II 2029232 2030467 WBGene00019140 bath-3 F59H6.10 -0.343025396862504
AGIWUR1254
CB4856 CT II 2030750 2031952 WBGene00019141 bath-5 F59H6.11 -0.398583763646235
AGIWUR2269
CB4856 CT II 2032569 2033243 WBGene00019142 btb-4 F59H6.12 -0.124073400412437
AGIWUR41441;AGIWUR44999
CB4856 CT II 2033586 2034812 WBGene00206415 F59H6.14 F59H6.14 -0.0988641651918112

AGIWUR1220
CB4856 CT II 2035702 2039388 WBGene00019132 bath-19 F59H6.1 -0.28979682691987 AGIWUR32883
CB4856 CT II 2040368 2041629 WBGene00015012 bath-20 B0047.1 -0.387675104035614
AGIWUR19234;AGIWUR42677;AGIWUR44896
CB4856 CT II 2043089 2044087 WBGene00015013 btb-3 B0047.2 -0.0444526056396528 AGIWUR36170
CB4856 CT II 2044619 2045601 WBGene00015014 bath-24 B0047.3 -0.314926493848384 AGIWUR9357
CB4856 CT II 2048061 2049175 WBGene00015016 bath-14 B0047.5 -0.371910231750734
AGIWUR22179;AGIWUR23543
CB4856 CT II 2050023 2051679 WBGene00017209 F07E5.4 F07E5.4 -0.172303489949218 AGIWUR13648
CB4856 CT II 2053919 2059072 WBGene00017210 F07E5.5 F07E5.5 0.0637338634027902 AGIWUR43260
CB4856 CT II 2059869 2064018 WBGene00017211 fbxb-36 F07E5.6 0.0841342482460412 AGIWUR21594
CB4856 CT II 2068354 2068971 WBGene00219315 F07E5.12 F07E5.12 -0.492986371232211
AGIWUR2322
CB4856 CT II 2070593 2071516 WBGene00017212 F07E5.7 F07E5.7 0.0948901150333601
AGIWUR2175;AGIWUR23158;AGIWUR36248
CB4856 CT II 2072221 2075830 WBGene00017213 F07E5.8 F07E5.8 0.0787899433824135 AGIWUR11692
CB4856 CT II 2076291 2080526 WBGene00017214 F07E5.9 F07E5.9 1.1279444307913 AGIWUR21892
CB4856 CT II 2081557 2082016 WBGene00020535 T16A1.4 T16A1.4 -0.150529514949524 AGIWUR35506
CB4856 CT II 2083595 2083826 WBGene00020536 T16A1.5 T16A1.5 -0.536229213384987 AGIWUR15560
CB4856 CT II 2084129 2084891 WBGene00020534 fbxc-49 T16A1.3 -0.0606476017037423 AGIWUR3770
CB4856 CT II 2091772 2092111 WBGene00269431 T16A1.10 T16A1.10 0.562441368978304
AGIWUR5174
CB4856 CT II 2095304 2098671 WBGene00020533 T16A1.2 T16A1.2 -0.150272132815742 AGIWUR25813
CB4856 CT II 2098871 2104149 WBGene00020532 math-42 T16A1.1 -0.020152446289865
AGIWUR4912;AGIWUR5197;AGIWUR24871;AGIWUR27173;AGIWUR31259
CB4856 CT II 2104524 2106866 WBGene00020075 math-35 R52.3 0.0637141045668113 AGIWUR19446
CB4856 CT II 2107197 2108395 WBGene00020076 R52.4 R52.4 0.163574811386621 AGIWUR29766
CB4856 CT II 2108787 2110043 WBGene00020077 R52.5 R52.5 0.16331680488244 AGIWUR35621
CB4856 CT II 2111227 2113048 WBGene00020078 R52.6 R52.6 0.0600472992432444 AGIWUR17386
CB4856 CT II 2114554 2116894 WBGene00005408 srh-195 R52.7 0.153377545217452
AGIWUR25884;AGIWUR27171;AGIWUR30449
CB4856 CT II 2117276 2122181 WBGene00020080 math-37 R52.9 0.223424132572545
AGIWUR22322;AGIWUR38481
CB4856 CT II 2123687 2129281 WBGene00020081 sdz-29 R52.10 -0.0210786772677025
AGIWUR997;AGIWUR7585;AGIWUR9725;AGIWUR17929;AGIWUR24130;AGIWUR36877;AGIWUR39906
CB4856 CT II 2131070 2132475 WBGene00020073 sdz-28 R52.1 0.192836523767019 AGIWUR26196
CB4856 CT II 2133666 2135704 WBGene00016548 hpo-7 C40A11.5 0.102702203399412
AGIWUR40590
CB4856 CT II 2136822 2136909 WBGene00016553 C40A11.10 C40A11.10 -0.137686010851374

AGIWUR29844
CB4856 CT II 2137422 2138951 WBGene00016547 C40A11.4 C40A11.4 0.143098717546897
AGIWUR17400
CB4856 CT II 2144292 2145666 WBGene00016549 C40A11.6 C40A11.6 -0.0282042264121038
AGIWUR8253;AGIWUR18636;AGIWUR31194;AGIWUR37868;AGIWUR39471;AGIWUR42954
CB4856 CT II 2146950 2148775 WBGene00016545 C40A11.2 C40A11.2 0.0532553430618314
AGIWUR19003
CB4856 CT II 2148732 2150363 WBGene00016550 C40A11.7 C40A11.7
-0.00665148756149847 AGIWUR18171
CB4856 CT II 2150552 2151290 WBGene00016551 C40A11.8 C40A11.8 0.0389674625642105
AGIWUR23141;AGIWUR29012;AGIWUR41581
CB4856 CT II 2154008 2155220 WBGene00016544 math-18 C40A11.1 -0.32082387467313
AGIWUR19325
CB4856 CT II 2163341 2163832 WBGene00044360 C33C12.11 C33C12.11 -0.17467213956394
AGIWUR26375
CB4856 CT II 2165440 2166498 WBGene00016336 C33C12.4 C33C12.4 0.327315498898308
AGIWUR11200
CB4856 CT II 2170325 2173219 WBGene00016338 srb-14 C33C12.6 -0.0727889190390751
AGIWUR212;AGIWUR2539;AGIWUR6574;AGIWUR16245;AGIWUR18339;AGIWUR37823
CB4856 CT II 2174586 2175289 WBGene00016339 C33C12.7 C33C12.7 -0.33228461949076
AGIWUR41219
CB4856 CT II 2176741 2181990 WBGene00016335 gba-1 C33C12.3 0.140652117546971
AGIWUR19527
CB4856 CT II 2182503 2186434 WBGene00016340 gba-2 C33C12.8 0.210954558950267
AGIWUR32624
CB4856 CT II 2187149 2189457 WBGene00016341 mtq-2 C33C12.9 0.237949895511401
AGIWUR36138
CB4856 CT II 2192339 2194114 WBGene00016342 C33C12.10 C33C12.10 0.203777662703265
AGIWUR44353
CB4856 CT II 2197846 2198675 WBGene00016334 C33C12.1 C33C12.1 0.156061821990484
AGIWUR20259
CB4856 CT II 2199143 2204139 WBGene00021985 Y59C2A.2 Y59C2A.2 -0.354674093912147
AGIWUR13216
CB4856 CT II 2208112 2212863 WBGene00021984 Y59C2A.1 Y59C2A.1 0.562056200564275
AGIWUR9506
CB4856 CT II 2215476 2219921 WBGene00021986 Y59C2A.3 Y59C2A.3 0.119524410278617
AGIWUR15526
CB4856 CT II 2224359 2225708 WBGene00018784 F54A3.5 F54A3.5 -0.254036269983141 AGIWUR36961
CB4856 CT II 2225817 2232470 WBGene00018785 F54A3.6 F54A3.6 -0.0112254567629311 AGIWUR44438

CB4856	CT	II	2233540 2243539 WBGene00018782	cct-3	F54A3.3 -0.549666540935
AGIWUR2521;AGIWUR9717;AGIWUR20960;AGIWUR24186;AGIWUR32791
CB4856	CT	II	2255172 2261101 WBGene00019586	K09F6.3 K09F6.3 -0.306003210987136
AGIWUR31678;AGIWUR37475
CB4856	CT	II	2262128 2265768 WBGene00019587	K09F6.4 K09F6.4 -0.093716973522282	AGIWUR10760
CB4856	CT	II	2274876 2279146 WBGene00019589	K09F6.6 K09F6.6 -0.120009873824127	AGIWUR31027
CB4856	CT	II	2280139 2280580 WBGene00044361	K09F6.11	K09F6.11	0.129863912833969
AGIWUR660;AGIWUR7865;AGIWUR12531;AGIWUR38262
CB4856	CT	II	2287030 2290226 WBGene00019593	K09F6.10	K09F6.10	-0.249307379371418
AGIWUR39646
CB4856	CT	II	2290896 2292184 WBGene00019590	K09F6.7 K09F6.7 -0.0676846386678021	AGIWUR35281
CB4856	CT	II	2294693 2296265 WBGene00019591	fbxc-25 K09F6.8 -0.0792194186324142
AGIWUR7503;AGIWUR25600
CB4856	CT	II	2297847 2298793 WBGene00015111	B0281.4 B0281.4 -0.364623625047344	AGIWUR32252
CB4856	CT	II	2299854 2300946 WBGene00015112	B0281.5 B0281.5 -0.886612437770207	AGIWUR17596
CB4856	CT	II	2301308 2302229 WBGene00015113	B0281.6 B0281.6 0.446204866256156	AGIWUR30003
CB4856	CT	II	2307041 2308361 WBGene00015110	B0281.3 B0281.3 -0.342994524747096
AGIWUR10890;AGIWUR22129;AGIWUR38792
CB4856	CT	II	2310071 2311074 WBGene00023225	B0281.7 B0281.7 0.0639856141098782	AGIWUR19027
CB4856	CT	II	2313067 2314927 WBGene00015108	B0281.1 B0281.1 -0.174355799799762	AGIWUR29422
CB4856	CT	II	2315084 2315622 WBGene00022869	ZK1240.4	ZK1240.4	-0.0158250713114496
AGIWUR40750
CB4856	CT	II	2316163 2317877 WBGene00022870	ZK1240.5	ZK1240.5	0.217722389231931
AGIWUR35040
CB4856	CT	II	2318189 2320134 WBGene00022873	ZK1240.9	ZK1240.9	0.14207393852713
AGIWUR6381;AGIWUR34515;AGIWUR37334
CB4856	CT	II	2320153 2321951 WBGene00022868	ZK1240.3	ZK1240.3	-0.346112545122635
AGIWUR2960
CB4856	CT	II	2322367 2322907 WBGene00022871	ZK1240.6	ZK1240.6	0.286690425948246
AGIWUR5666
CB4856	CT	II	2323376 2325215 WBGene00022867	ZK1240.2	ZK1240.2	-0.592007563975276
AGIWUR14511
CB4856	CT	II	2326995 2329053 WBGene00022872	ZK1240.8	ZK1240.8	-0.254274842317395
AGIWUR42400
CB4856	CT	II	2329355 2331792 WBGene00022866	ZK1240.1	ZK1240.1	0.0183998712842843
AGIWUR11319
CB4856	CT	II	2332520 2334338 WBGene00018385	F43C11.8	F43C11.8	0.179486765332876
AGIWUR12050
CB4856	CT	II	2335933 2337696 WBGene00018384	F43C11.7	F43C11.7	1.77181167872715

AGIWUR27872
CB4856 CT II 2337897 2340746 WBGene00018386 F43C11.9 F43C11.9 -0.0583328277453106
AGIWUR20373
CB4856 CT II 2342433 2343925 WBGene00018383 F43C11.6 F43C11.6 -0.209388184789903
AGIWUR25498
CB4856 CT II 2345118 2346028 WBGene00018382 F43C11.5 F43C11.5 -0.354842785501338
AGIWUR45203
CB4856 CT II 2347015 2350030 WBGene00018381 F43C11.4 F43C11.4 0.230442957898209
AGIWUR19877
CB4856 CT II 2350216 2351657 WBGene00018387 F43C11.10 F43C11.10 0.0716075085468155
AGIWUR2098;AGIWUR5664;AGIWUR17935
CB4856 CT II 2354776 2355972 WBGene00018379 F43C11.2 F43C11.2 -0.492624705393064
AGIWUR27877
CB4856 CT II 2357095 2358278 WBGene00018378 F43C11.1 F43C11.1 0.00202690660144311
AGIWUR35747;AGIWUR36438
CB4856 CT II 2358801 2363773 WBGene00018388 F43C11.11 F43C11.11 0.052781864810628
AGIWUR6497
CB4856 CT II 2365573 2366720 WBGene00018389 F43C11.12 F43C11.12 -0.0692104733479475
AGIWUR28662
CB4856 CT II 2367528 2368708 WBGene00017520 F16G10.5 F16G10.5 -0.418882417641859
AGIWUR7640
CB4856 CT II 2369256 2370089 WBGene00017519 hpo-25 F16G10.4 0.105625560943625
AGIWUR33028
CB4856 CT II 2372390 2374340 WBGene00017518 F16G10.3 F16G10.3 0.0657749798269069
AGIWUR8438
CB4856 CT II 2375273 2376411 WBGene00017517 F16G10.2 F16G10.2 0.0356162204295556
AGIWUR3215
CB4856 CT II 2377199 2377851 WBGene00017521 F16G10.6 F16G10.6 0.338814583720482
AGIWUR12468
CB4856 CT II 2379579 2380274 WBGene00017522 F16G10.7 F16G10.7 0.113548382407339
AGIWUR29225
CB4856 CT II 2381949 2383434 WBGene00017523 F16G10.8 F16G10.8 -0.0574048171089885
AGIWUR20430
CB4856 CT II 2384186 2385595 WBGene00017524 F16G10.9 F16G10.9 0.275333941715273
AGIWUR11291;AGIWUR33652;AGIWUR45181
CB4856 CT II 2394916 2395964 WBGene00017527 F16G10.13 F16G10.13 0.150878165599027
AGIWUR12983
CB4856 CT II 2404284 2404659 WBGene00017516 F16G10.1 F16G10.1 -0.0831517500738634
AGIWUR30351

CB4856 CT II 2409643 2412741 WBGene00018353 fbxa-182 F42G2.4 -0.160735173714297
AGIWUR13568;AGIWUR39835
CB4856 CT II 2414119 2415531 WBGene00044672 fbxa-4 F42G2.8 -0.246453727458412
AGIWUR27382;AGIWUR33844
CB4856 CT II 2416101 2417971 WBGene00018354 F42G2.5 F42G2.5 -0.414710416698022
AGIWUR9576;AGIWUR25080;AGIWUR33778
CB4856 CT II 2418448 2419025 WBGene00044362 F42G2.7 F42G2.7 0.0221200042375926 AGIWUR35918
CB4856 CT II 2419472 2420794 WBGene00018352 fbxc-20 F42G2.3 -0.188547046977243 AGIWUR7061
CB4856 CT II 2426372 2429944 WBGene00018355 ceh-86 F42G2.6 -0.0523175910454547 AGIWUR1758
CB4856 CT II 2443279 2451130 WBGene00006882 vab-19 T22D2.1 0.21360507124795
AGIWUR22528;AGIWUR42638
CB4856 CT II 2452970 2458981 WBGene00004129 pqn-42 F53G2.4 0.151571230173229
AGIWUR2755;AGIWUR27831
CB4856 CT II 2460866 2472862 WBGene00006647 tsr-1 F53G2.6 0.0352151212541543 AGIWUR16260
CB4856 CT II 2480399 2481312 WBGene00018768 F53G2.3 F53G2.3 -0.195474308138602 AGIWUR11911
CB4856 CT II 2481921 2485922 WBGene00018767 F53G2.2 F53G2.2 0.00141759686159837 AGIWUR37648
CB4856 CT II 2488754 2496641 WBGene00018766 F53G2.1 F53G2.1 0.218030376080311 AGIWUR38040
CB4856 CT II 2504878 2514846 WBGene00019130 gbas-1 F59H5.1 -0.13444457973614 AGIWUR23587
CB4856 CT II 2514977 2516097 WBGene00019131 bath-12 F59H5.3 -0.107984884737387
AGIWUR865;AGIWUR4761;AGIWUR18764
CB4856 CT II 2520965 2522044 WBGene00018223 bath-13 F40B1.1 -0.0885498436337384 AGIWUR15079
CB4856 CT II 2522628 2524475 WBGene00018224 bath-18 F40B1.2 0.058657827332766 AGIWUR28245
CB4856 CT II 2525220 2529255 WBGene00019341 K02F6.7 K02F6.7 -0.0337132936625064 AGIWUR38714
CB4856 CT II 2530817 2531476 WBGene00019340 K02F6.6 K02F6.6 -0.224189652174944 AGIWUR20611
CB4856 CT II 2546225 2553572 WBGene00019337 K02F6.3 K02F6.3 0.0439722740750552
AGIWUR13728;AGIWUR21707;AGIWUR42219
CB4856 CT II 2553962 2556313 WBGene00019335 K02F6.1 K02F6.1 -0.0445728368903726 AGIWUR16350
CB4856 CT II 2557781 2563677 WBGene00019343 nep-18 K02F6.9 0.0454662553362706
AGIWUR27404;AGIWUR31183;AGIWUR32099
CB4856 CT II 2565275 2575930 WBGene00017280 usp-39 F09D1.1 -0.251971982233076 AGIWUR22184
CB4856 CT II 2578536 2579595 WBGene00005587 sri-75 F33H12.3 -0.165554445556115
AGIWUR5883
CB4856 CT II 2580901 2583346 WBGene00005586 sri-74 F33H12.4 0.0130052593340491
AGIWUR38546
CB4856 CT II 2583782 2585593 WBGene00005548 sri-36 F33H12.5 0.0392530289379076
AGIWUR29515
CB4856 CT II 2586078 2586911 WBGene00045457 F33H12.7 F33H12.7 0.294943087477567
AGIWUR10211;AGIWUR11014;AGIWUR14494;AGIWUR20112;AGIWUR21173;AGIWUR27869
CB4856 CT II 2587504 2589754 WBGene00005551 sri-39 F33H12.2 -0.0347277077190271

AGIWUR2569;AGIWUR9926
CB4856 CT II 2600553 2601779 WBGene00018019 F33H12.1 F33H12.1 0.0348519855963785
AGIWUR33272
CB4856 CT II 2603208 2605419 WBGene00005589 sri-77 D2062.9 0.240994727537387 AGIWUR10343
CB4856 CT II 2610862 2612359 WBGene00005550 sri-38 D2062.8 -0.163134057805788
AGIWUR18254;AGIWUR39361
CB4856 CT II 2616905 2617374 WBGene00017059 D2062.7 D2062.7 0.129800349715357 AGIWUR30414
CB4856 CT II 2619419 2619789 WBGene00017058 D2062.6 D2062.6 -0.259272276262383
AGIWUR152;AGIWUR6988;AGIWUR17021;AGIWUR28623;AGIWUR37201;AGIWUR42200
CB4856 CT II 2623402 2624530 WBGene00017056 D2062.4 D2062.4 -0.213969851182825
AGIWUR11959;AGIWUR21825;AGIWUR31235;AGIWUR43927
CB4856 CT II 2625092 2627070 WBGene00044301 lgc-28 D2062.12 0.661175926648455
AGIWUR30690;AGIWUR43966
CB4856 CT II 2627749 2629196 WBGene00005583 sri-71 D2062.3 -0.0563820065752463 AGIWUR35298
CB4856 CT II 2629570 2631231 WBGene00005549 sri-37 D2062.2 0.0017994834742634 AGIWUR17145
CB4856 CT II 2631473 2633054 WBGene00017055 D2062.1 D2062.1 0.0174023133914041 AGIWUR15096
CB4856 CT II 2634210 2635321 WBGene00005553 sri-41 F22E5.10 -0.212553141523916
AGIWUR35927;AGIWUR43074
CB4856 CT II 2635360 2637608 WBGene00044363 F22E5.21 F22E5.21 0.149506020109079
AGIWUR6996;AGIWUR13791
CB4856 CT II 2638392 2639353 WBGene00017708 F22E5.9 F22E5.9 0.0110040613910591 AGIWUR8605
CB4856 CT II 2640081 2641897 WBGene00017707 F22E5.8 F22E5.8 -0.224510771027802 AGIWUR29034
CB4856 CT II 2642090 2642884 WBGene00017706 F22E5.7 F22E5.7 -0.212885735303771 AGIWUR2135
CB4856 CT II 2643685 2645703 WBGene00017709 F22E5.11 F22E5.11 0.253252599719974
AGIWUR16622
CB4856 CT II 2647637 2649639 WBGene00017711 F22E5.13 F22E5.13 -0.160473057650268
AGIWUR15725;AGIWUR33628
CB4856 CT II 2649870 2650715 WBGene00017705 F22E5.6 F22E5.6 0.227444465217088 AGIWUR39051
CB4856 CT II 2652841 2654464 WBGene00005561 sri-49 F22E5.14 -0.0285211204650233
AGIWUR283;AGIWUR4661;AGIWUR8245;AGIWUR13251;AGIWUR28279;AGIWUR32069;AGIWUR33970;AGIWUR35353;AGIWUR37519
CB4856 CT II 2659670 2665022 WBGene00001546 gcy-21 F22E5.3 0.0595656546086722 AGIWUR44744
CB4856 CT II 2666283 2668162 WBGene00005567 sri-55 F22E5.15 0.0396026403281429
AGIWUR3285;AGIWUR11082
CB4856 CT II 2672632 2673634 WBGene00017713 F22E5.20 F22E5.20 0.346693115708323
AGIWUR8172
CB4856 CT II 2673908 2677588 WBGene00017703 F22E5.1 F22E5.1 -0.605934828152904 AGIWUR18331
CB4856 CT II 2678041 2679162 WBGene00017712 F22E5.17 F22E5.17 0.022918666565495
AGIWUR11407
CB4856 CT II 2679163 2681218 WBGene00018021 F34D6.1 F34D6.1 0.249578264862235 AGIWUR32910

CB4856	CT	II	2683329	2684308	WBGene00018022	ceh-87	F34D6.2	-0.138334610226845	AGIWUR1083
CB4856	CT	II	2684669	2686977	WBGene00006318	sup-9	F34D6.3	0.0239552430421099	AGIWUR35930
CB4856	CT	II	2688916	2689179	WBGene00044365	F34D6.8	F34D6.8	-0.288034665469258	AGIWUR3570;AGIWUR14833;AGIWUR16546;AGIWUR25095;AGIWUR30598
CB4856	CT	II	2690472	2694311	WBGene00018023	set-11	F34D6.4	-0.571982218588863	AGIWUR35159
CB4856	CT	II	2696939	2698478	WBGene00005574	sri-62	F34D6.5	-0.296766164997219	AGIWUR37243
CB4856	CT	II	2698865	2700329	WBGene00005573	sri-61	F34D6.6	0.0746990377979522	AGIWUR26167
CB4856	CT	II	2701389	2702857	WBGene00005572	sri-60	Y47G7B.3	-0.288207272872416	AGIWUR4730
CB4856	CT	II	2703371	2705316	WBGene00005345	srh-128	Y47G7B.1	0.16575826951555	AGIWUR42635
CB4856	CT	II	2705996	2707982	WBGene00021654	Y47G7B.2	Y47G7B.2	0.164966980281962	AGIWUR5884
CB4856	CT	II	2708438	2710096	WBGene00020227	T05A8.1	T05A8.1	0.652590807750144	AGIWUR29682
CB4856	CT	II	2709942	2711019	WBGene00020228	T05A8.2	T05A8.2	0.149386221963198	AGIWUR23765
CB4856	CT	II	2714647	2717435	WBGene00020229	T05A8.3	T05A8.3	-0.297981494641412	AGIWUR2639;AGIWUR12034
CB4856	CT	II	2717522	2725541	WBGene00020230	nep-2	T05A8.4	-0.0069666413308056	AGIWUR4193
CB4856	CT	II	2731319	2735250	WBGene00020231	T05A8.5	T05A8.5	0.0852997766941286	AGIWUR33951
CB4856	CT	II	2736678	2737556	WBGene00044367	T05A8.8	T05A8.8	0.0754619755031918	AGIWUR6273;AGIWUR21950;AGIWUR29441;AGIWUR34602;AGIWUR42076
CB4856	CT	II	2738523	2739642	WBGene00020232	T05A8.6	T05A8.6	0.177366942278203	AGIWUR36070
CB4856	CT	II	2740403	2742341	WBGene00020233	T05A8.7	T05A8.7	0.461067989524302	AGIWUR12867
CB4856	CT	II	2747393	2747728	WBGene00017915	F29A7.5	F29A7.5	0.232227703109107	AGIWUR5403
CB4856	CT	II	2748588	2751573	WBGene00017914	F29A7.4	F29A7.4	0.112739426645954	AGIWUR35213
CB4856	CT	II	2751741	2752611	WBGene00017916	F29A7.6	F29A7.6	-0.164431099046658	AGIWUR1148;AGIWUR13132
CB4856	CT	II	2753299	2754379	WBGene00017913	F29A7.3	F29A7.3	0.00981734409001955	AGIWUR10580;AGIWUR15672;AGIWUR31599;AGIWUR38911;AGIWUR39414
CB4856	CT	II	2756021	2757018	WBGene00017917	clec-120	F29A7.7	0.0416207799466619	AGIWUR37125
CB4856	CT	II	2757147	2758054	WBGene00017918	F29A7.8	F29A7.8	0.179168707150166	AGIWUR26911
CB4856	CT	II	2758625	2761856	WBGene00017912	fbxb-107	F29A7.2	0.424532321569222	AGIWUR35880
CB4856	CT	II	2764964	2767230	WBGene00017249	fbxb-108	F08D12.6	0.0729726764412995	AGIWUR27513
CB4856	CT	II	2767250	2769121	WBGene00017250	F08D12.7	F08D12.7	-0.202050157547106	AGIWUR13638
CB4856	CT	II	2769670	2770888	WBGene00017251	fbxb-105	F08D12.8	0.448376521963695	

AGIWUR32943
CB4856 CT II 2771082 2772266 WBGene00017252 sdz-10 F08D12.9 0.0401115033034412
AGIWUR22665
CB4856 CT II 2772389 2773634 WBGene00017253 sdz-9 F08D12.10 0.279489556778506
AGIWUR14124
CB4856 CT II 2773954 2775042 WBGene00017254 fbxb-111 F08D12.11 0.256647166591264
AGIWUR23089
CB4856 CT II 2778146 2779282 WBGene00017248 F08D12.4 F08D12.4 0.115127875209211
AGIWUR34662
CB4856 CT II 2779544 2780838 WBGene00017247 F08D12.3 F08D12.3 0.321317860595101
AGIWUR11622;AGIWUR14891;AGIWUR21016
CB4856 CT II 2781628 2783083 WBGene00017246 F08D12.2 F08D12.2 -0.0428187930303139
AGIWUR20210
CB4856 CT II 2783102 2787089 WBGene00017255 F08D12.12 F08D12.12 0.147189889699707
AGIWUR24811
CB4856 CT II 2787481 2793471 WBGene00017245 srpa-72 F08D12.1 0.286134168280714
AGIWUR36556
CB4856 CT II 2794974 2800600 WBGene00003709 nhr-119 K12H6.1 0.0730372548073368
AGIWUR24562;AGIWUR32874
CB4856 CT II 2808167 2812860 WBGene00019691 K12H6.12 K12H6.12 0.0511508350484497
AGIWUR21824
CB4856 CT II 2812872 2815544 WBGene00019683 K12H6.2 K12H6.2 -0.0442553524229048 AGIWUR31778
CB4856 CT II 2816976 2819123 WBGene00019690 set-13 K12H6.11 -0.148843439387354
AGIWUR6625
CB4856 CT II 2819435 2822490 WBGene00001507 fut-4 K12H6.3 0.0305563793267325 AGIWUR42179
CB4856 CT II 2823707 2824269 WBGene00019684 K12H6.4 K12H6.4 -0.174631739944646 AGIWUR42778
CB4856 CT II 2825662 2826598 WBGene00019685 K12H6.5 K12H6.5 -0.410910555471863 AGIWUR12317
CB4856 CT II 2827897 2828603 WBGene00019688 K12H6.8 K12H6.8 -0.52084078063961 AGIWUR35848
CB4856 CT II 2828594 2831233 WBGene00019686 K12H6.6 K12H6.6 0.180448998210703
AGIWUR10125;AGIWUR17611
CB4856 CT II 2834953 2836780 WBGene00019687 K12H6.7 K12H6.7 -0.118527571915857 AGIWUR41986
CB4856 CT II 2839304 2840386 WBGene00022441 Y110A2AL.4 Y110A2AL.4 0.213949499901843
AGIWUR2788;AGIWUR19080;AGIWUR19398;AGIWUR38238
CB4856 CT II 2841010 2841629 WBGene00022443 Y110A2AL.6 Y110A2AL.6 0.134668770310181
AGIWUR18505
CB4856 CT II 2842608 2843188 WBGene00022444 Y110A2AL.7 Y110A2AL.7 -0.281115157990161
AGIWUR361
CB4856 CT II 2845384 2852593 WBGene00004210 ptc-3 Y110A2AL.8 0.220911496680456
AGIWUR20873;AGIWUR36226;AGIWUR36414;AGIWUR44866

CB4856	CT	II	2864529 2865363 WBGene00022442	Y110A2AL.5	Y110A2AL.5	-0.227722937607771
AGIWUR26948
CB4856	CT	II	2865971 2866655 WBGene00022445	Y110A2AL.9	Y110A2AL.9	0.606618472023563
AGIWUR3686;AGIWUR15915;AGIWUR18333;AGIWUR20884;AGIWUR37977;AGIWUR39130
CB4856	CT	II	2867074 2867779 WBGene00022440	Y110A2AL.3	Y110A2AL.3	0.448909068689045
AGIWUR8722
CB4856	CT	II	2867993 2870758 WBGene00022439	Y110A2AL.2	Y110A2AL.2	-0.0153287785123268
AGIWUR5974;AGIWUR13320;AGIWUR16794;AGIWUR16833;AGIWUR30884
CB4856	CT	II	2871377 2871914 WBGene00022446	Y110A2AL.10	Y110A2AL.10	0.240984872210037
AGIWUR28531
CB4856	CT	II	2875101 2876618 WBGene00022438	Y110A2AL.1	Y110A2AL.1	-0.458073615033703
AGIWUR6925
CB4856	CT	II	2878729 2884910 WBGene00022447	hpo-20	Y110A2AL.12	-0.152902869066681
AGIWUR1875;AGIWUR11336;AGIWUR14290;AGIWUR30668;AGIWUR40005;AGIWUR44573
CB4856	CT	II	2886317 2889016 WBGene00022448	pinn-1	Y110A2AL.13	-0.024301505873332
AGIWUR13612
CB4856	CT	II	2889104 2894395 WBGene00005020	sqv-2	Y110A2AL.14	-0.0438694390955501
AGIWUR42414
CB4856	CT	II	2896737 2900348 WBGene00022450	ubc-26	Y110A2AM.3	-0.0477423091045317
AGIWUR5889;AGIWUR23227;AGIWUR30571;AGIWUR38183;AGIWUR42075
CB4856	CT	II	2902962 2904001 WBGene00022449	Y110A2AM.1	Y110A2AM.1	0.136488911289959
AGIWUR17641
CB4856	CT	II	2913705 2919314 WBGene00022453	Y110A2AR.3	Y110A2AR.3	-0.201647708599213
AGIWUR20065;AGIWUR38218;AGIWUR40062
CB4856	CT	II	2919710 2920904 WBGene00022452	Y110A2AR.1	Y110A2AR.1	-0.0861857161485489
AGIWUR30954
CB4856	CT	II	2923899 2926533 WBGene00022712	ZK355.2 ZK355.2 0.026391441847375
AGIWUR36579;AGIWUR38145
CB4856	CT	II	2927017 2927633 WBGene00022713	ZK355.3 ZK355.3 0.662552423073585	AGIWUR16281
CB4856	CT	II	2928221 2930599 WBGene00022714	irld-64 ZK355.4 0.0245072106589106	AGIWUR25390
CB4856	CT	II	2930728 2933463 WBGene00022711	irld-63 ZK355.1 0.140928666074688	AGIWUR574
CB4856	CT	II	2933521 2934454 WBGene00044369	irld-67 ZK355.7 -0.0703147047475268
AGIWUR7665;AGIWUR17850;AGIWUR29654
CB4856	CT	II	2934805 2937952 WBGene00022715	irld-65 ZK355.5 0.335466800491084	AGIWUR18946
CB4856	CT	II	2938143 2940847 WBGene00022716	irld-66 ZK355.6 -0.0395397017253654	AGIWUR36728
CB4856	CT	II	2940996 2944077 WBGene00020431	irld-50 T11F1.7 0.0384658389433898	AGIWUR1396
CB4856	CT	II	2944176 2946533 WBGene00020430	irld-49 T11F1.6 -0.0727279908248804	AGIWUR11744
CB4856	CT	II	2946738 2948434 WBGene00005826	srw-79	T11F1.5 0.148896082977976
AGIWUR11043;AGIWUR16519;AGIWUR19279;AGIWUR29750

CB4856	CT	II	2948929	2952729	WBGene00077759	irld-51	T11F1.9	0.183828572508192	AGIWUR3293
CB4856	CT	II	2956065	2958191	WBGene00020428	irld-48	T11F1.2	-0.118726381727313	AGIWUR5921
CB4856	CT	II	2958608	2961883	WBGene00005807	srw-60	T11F1.1	0.181270048571114	AGIWUR20994
CB4856	CT	II	2976639	2987023	WBGene00003678	nhr-88	K08A2.5	0.155893493828904	AGIWUR9908;AGIWUR25658;AGIWUR25973;AGIWUR31000;AGIWUR33208
CB4856	CT	II	2988307	2991238	WBGene00019513	K08A2.4	K08A2.4	-0.0155290029175976	AGIWUR23537
CB4856	CT	II	2991613	2994313	WBGene00019512	K08A2.2	K08A2.2	-0.201311356109027	AGIWUR16689
CB4856	CT	II	2997021	3000653	WBGene00019511	K08A2.1	K08A2.1	0.136424702747981	AGIWUR37176
CB4856	CT	II	3000768	3002411	WBGene00016576	C41H7.6	C41H7.6	-0.202598294282799	AGIWUR20719
CB4856	CT	II	3002513	3003827	WBGene00016575	C41H7.5	C41H7.5	-0.164992918984588	AGIWUR269
CB4856	CT	II	3003969	3005573	WBGene00016574	C41H7.4	C41H7.4	-0.159814037884409	AGIWUR2896
CB4856	CT	II	3005799	3007506	WBGene00016573	C41H7.3	C41H7.3	-0.284289668042243	AGIWUR27129
CB4856	CT	II	3014835	3015961	WBGene00016572	C41H7.2	C41H7.2	0.00339867267714311	AGIWUR19017
CB4856	CT	II	3016068	3017471	WBGene00016571	C41H7.1	C41H7.1	-0.200561310492045	AGIWUR18416
CB4856	CT	II	3017482	3019907	WBGene00016577	clec-3	C41H7.7	-1.13337958292903	AGIWUR41295
CB4856	CT	II	3032780	3036122	WBGene00015197	B0454.6	B0454.6	0.0741840634673263	AGIWUR13089
CB4856	CT	II	3038859	3040822	WBGene00015196	B0454.5	B0454.5	-0.0796189244946017	AGIWUR14221;AGIWUR34522
CB4856	CT	II	3040943	3043406	WBGene00015199	B0454.8	B0454.8	-0.164635513209486	AGIWUR43750;AGIWUR44012;AGIWUR44391
CB4856	CT	II	3044323	3046115	WBGene00005541	sri-29	B0454.4	-0.0614222334783259	AGIWUR2475
CB4856	CT	II	3046219	3048154	WBGene00005542	sri-30	B0454.10	0.10819779656341	AGIWUR8463
CB4856	CT	II	3048678	3050435	WBGene00005540	sri-28	B0454.3	-0.0114782036865941	AGIWUR12561
CB4856	CT	II	3051003	3052322	WBGene00005543	sri-31	B0454.2	0.217878165270324	AGIWUR14825
CB4856	CT	II	3052597	3054348	WBGene00015200	B0454.9	B0454.9	0.0864765202983548	AGIWUR25688
CB4856	CT	II	3056822	3060207	WBGene00002997	lin-8	B0454.1	0.484056363082338	AGIWUR39951
CB4856	CT	II	3061082	3062112	WBGene00021293	Y25C1A.6	Y25C1A.6	-0.0551533853511046	AGIWUR19149
CB4856	CT	II	3062542	3071381	WBGene00021294	Y25C1A.7	Y25C1A.7	-0.0258329306740643	AGIWUR3099;AGIWUR9962;AGIWUR12918;AGIWUR14161;AGIWUR14668;AGIWUR18126;AGIWUR23317;AGIWUR23438;AGIWUR36817;AGIWUR38161;AGIWUR42714;AGIWUR43276
CB4856	CT	II	3071164	3076953	WBGene00021295	Y25C1A.8	Y25C1A.8	-0.0141740012168192	AGIWUR29733
CB4856	CT	II	3078796	3087682	WBGene00021292	copb-1	Y25C1A.5	0.182053053892878	AGIWUR44602
CB4856	CT	II	3088474	3091296	WBGene00005178	srg-21	Y25C1A.9	0.230610513194359	AGIWUR38380
CB4856	CT	II	3091503	3093452	WBGene00005179	srg-22	Y25C1A.10	-0.101336034664714	

AGIWUR15037
CB4856	CT	II	3093902	3096160	WBGene00005180	srg-23	Y25C1A.11	-0.0216305987649914
AGIWUR11917
CB4856	CT	II	3098403	3100646	WBGene00005181	srg-24	Y25C1A.12	-0.0283350348468758
AGIWUR14814
CB4856	CT	II	3102318	3104736	WBGene00021291	clec-121	Y25C1A.4	-0.375865672754031
AGIWUR11656
CB4856	CT	II	3106065	3108032	WBGene00021290	clec-122	Y25C1A.3	-0.0701699457040874
AGIWUR39278
CB4856	CT	II	3109368	3111247	WBGene00021296	Y25C1A.13	Y25C1A.13	-0.0868891593215597
AGIWUR14960;AGIWUR25497
CB4856	CT	II	3112613	3113337	WBGene00021289	Y25C1A.2	Y25C1A.2	-0.148163546720866
AGIWUR33174
CB4856	CT	II	3114234	3116758	WBGene00021288	clec-123	Y25C1A.1	0.457894456239325
AGIWUR10375
CB4856	CT	II	3117771	3118451	WBGene00020409	T10D4.7	T10D4.7	-0.199898599341394	AGIWUR8077
CB4856	CT	II	3119332	3123192	WBGene00020408	T10D4.6	T10D4.6	-0.0799354675134527	AGIWUR6156
CB4856	CT	II	3123506	3124899	WBGene00005292	srh-71	T10D4.5	-0.0999574932483815	AGIWUR36043
CB4856	CT	II	3126037	3126441	WBGene00002102	ins-19	T10D4.13	-1.08718993519153
AGIWUR11814;AGIWUR14823;AGIWUR29537;AGIWUR31712;AGIWUR36904
CB4856	CT	II	3127041	3127790	WBGene00002114	ins-31	T10D4.4	0.0716780813778328
AGIWUR11011;AGIWUR28105;AGIWUR30924;AGIWUR31563;AGIWUR40948
CB4856	CT	II	3129179	3131288	WBGene00020407	chil-24	T10D4.3	0.060774122070276	AGIWUR34237
CB4856	CT	II	3134099	3135283	WBGene00005568	sri-56	T10D4.2	-0.00433571053900161
AGIWUR16953;AGIWUR32344
CB4856	CT	II	3135581	3137904	WBGene00005566	sri-54	T10D4.8	0.0819854656816261	AGIWUR3261
CB4856	CT	II	3140918	3142955	WBGene00005554	sri-42	T10D4.9	0.00623181233213987	AGIWUR31287
CB4856	CT	II	3143394	3144652	WBGene00005555	sri-43	T10D4.10	0.094915284146655
AGIWUR8985
CB4856	CT	II	3146142	3146992	WBGene00077522	T10D4.15	T10D4.15	0.0304854517828343
AGIWUR19568;AGIWUR37353
CB4856	CT	II	3148504	3149621	WBGene00020410	T10D4.11	T10D4.11	-0.743422028979799
AGIWUR41738
CB4856	CT	II	3150140	3152575	WBGene00020406	T10D4.1	T10D4.1	0.300810230303775	AGIWUR10791
CB4856	CT	II	3153050	3154483	WBGene00005544	sri-32	T10D4.12	0.0599949679163316
AGIWUR39838
CB4856	CT	II	3155167	3156849	WBGene00005545	sri-33	Y27F2A.4	-0.0288626903552114
AGIWUR38115
CB4856	CT	II	3157491	3159085	WBGene00005546	sri-34	Y27F2A.7	0.0536560224931666

AGIWUR36938
CB4856 CT II 3168767 3170189 WBGene00021297 fbxc-55 Y27F2A.5 -0.00867290046260205
AGIWUR9674
CB4856 CT II 3171468 3172276 WBGene00045303 Y27F2A.10 Y27F2A.10 0.193500536768218
AGIWUR17309;AGIWUR17803
CB4856 CT II 3177963 3180476 WBGene00005552 sri-40 Y27F2A.3 -0.00544612926117161
AGIWUR21152;AGIWUR38695
CB4856 CT II 3180479 3182676 WBGene00021298 Y27F2A.6 Y27F2A.6 0.131412240569139
AGIWUR12463
CB4856 CT II 3185158 3186790 WBGene00005590 sri-78 Y27F2A.2 0.262290869870419
AGIWUR16945
CB4856 CT II 3197613 3199098 WBGene00005563 sri-51 ZC239.8 0.217984632976224 AGIWUR36077
CB4856 CT II 3206020 3206541 WBGene00044371 ZC239.20 ZC239.20 -0.296036813789113
AGIWUR6160;AGIWUR8135;AGIWUR9712;AGIWUR18739;AGIWUR26353;AGIWUR35448;AGIWUR39029
CB4856 CT II 3206939 3207388 WBGene00044372 ZC239.21 ZC239.21 -0.267217985361752
AGIWUR7815;AGIWUR14066;AGIWUR22921
CB4856 CT II 3207683 3208751 WBGene00022570 sdz-35 ZC239.12 -0.0238325127113761
AGIWUR30050
CB4856 CT II 3209478 3211181 WBGene00022569 ZC239.6 ZC239.6 -0.493419672888789 AGIWUR1572
CB4856 CT II 3212371 3213208 WBGene00022568 ZC239.5 ZC239.5 -0.430718277288369 AGIWUR27087
CB4856 CT II 3214452 3215565 WBGene00022567 ZC239.4 ZC239.4 -0.177221680962273
AGIWUR1430;AGIWUR11052;AGIWUR38797
CB4856 CT II 3216081 3216600 WBGene00022566 ZC239.3 ZC239.3 -0.106887534573158 AGIWUR30364
CB4856 CT II 3217907 3218990 WBGene00022565 ZC239.2 ZC239.2 -0.101599085043953 AGIWUR11486
CB4856 CT II 3221126 3222010 WBGene00022572 ZC239.14 ZC239.14 0.174241193438885
AGIWUR28231
CB4856 CT II 3222406 3222827 WBGene00022571 ZC239.13 ZC239.13 -0.253555057382357
AGIWUR7714
CB4856 CT II 3224770 3225285 WBGene00022574 ZC239.16 ZC239.16 -0.348978383206476
AGIWUR28996
CB4856 CT II 3225348 3225968 WBGene00022575 ZC239.17 ZC239.17 -0.0860557060003666
AGIWUR8136
CB4856 CT II 3228885 3230522 WBGene00005501 srh-297 C17F4.4 0.13680696034703
AGIWUR26598;AGIWUR31997
CB4856 CT II 3230817 3234813 WBGene00015914 C17F4.8 C17F4.8 -0.0673788517163167 AGIWUR3081
CB4856 CT II 3235956 3237138 WBGene00023479 srz-67 C17F4.10 -0.132747702683307
AGIWUR21449;AGIWUR34302;AGIWUR41495
CB4856 CT II 3237471 3238179 WBGene00044373 C17F4.11 C17F4.11 -0.118212858724438
AGIWUR8847;AGIWUR26875;AGIWUR29109

CB4856	CT	II	3238169	3240043	WBGene00023480	srz-68	C17F4.9	-0.0028808574364998	AGIWUR40543
CB4856	CT	II	3240545	3241726	WBGene00015911	C17F4.3	C17F4.3	0.186982425406472	AGIWUR13213
CB4856	CT	II	3243676	3244162	WBGene00044374	C17F4.12	C17F4.12	-0.0322155279072849	AGIWUR946;AGIWUR4095;AGIWUR14240;AGIWUR31293;AGIWUR35584
CB4856	CT	II	3245087	3246015	WBGene00015913	C17F4.7	C17F4.7	-0.266101168398482	AGIWUR43953
CB4856	CT	II	3246316	3248174	WBGene00015912	fbxc-50	C17F4.5	-0.270429425874589	AGIWUR5090
CB4856	CT	II	3248623	3249386	WBGene00015910	C17F4.2	C17F4.2	-0.752309023156691	AGIWUR23272
CB4856	CT	II	3250313	3250856	WBGene00015909	clec-124	C17F4.1	-0.399067006937894	AGIWUR1933
CB4856	CT	II	3250985	3259354	WBGene00001544	gcy-19	C17F4.6	-0.228209912692106	AGIWUR2396
CB4856	CT	II	3267663	3271505	WBGene00195073	F02E11.7	F02E11.7	0.590053769594455	AGIWUR11844
CB4856	CT	II	3272349	3275023	WBGene00017181	F02E11.3	F02E11.3	-0.0387806062602305	AGIWUR41749
CB4856	CT	II	3276256	3277305	WBGene00017182	F02E11.4	F02E11.4	-0.182358317175233	AGIWUR37077
CB4856	CT	II	3278987	3281686	WBGene00017179	wht-4	F02E11.1	-0.0473167407435335	AGIWUR987;AGIWUR15251;AGIWUR17304;AGIWUR24953;AGIWUR30539;AGIWUR35672
CB4856	CT	II	3282653	3287158	WBGene00018196	nep-13	F39E9.4	-0.102838350070357	AGIWUR35454
CB4856	CT	II	3287373	3289031	WBGene00005575	sri-63	F39E9.3	0.13706540301075	AGIWUR37501
CB4856	CT	II	3290486	3290644	WBGene00044375	F39E9.14	F39E9.14	-0.0738314534282862	AGIWUR1826
CB4856	CT	II	3292264	3293472	WBGene00023281	F39E9.8	F39E9.8	0.065689258116743	AGIWUR33135
CB4856	CT	II	3294021	3295068	WBGene00023282	F39E9.9	F39E9.9	0.56060930248152	AGIWUR28424
CB4856	CT	II	3295414	3296582	WBGene00018200	btb-17	F39E9.11	0.0906709458308952	AGIWUR14277;AGIWUR18377
CB4856	CT	II	3296908	3298027	WBGene00018195	btb-16	F39E9.2	0.737740104909882	AGIWUR26250
CB4856	CT	II	3299825	3300676	WBGene00269387	btb-22	F39E9.27	-0.107935710490747	AGIWUR3343
CB4856	CT	II	3302761	3303750	WBGene00023036	F39E9.10	F39E9.10	0.212870703458442	AGIWUR23026;AGIWUR23331;AGIWUR23832;AGIWUR27066;AGIWUR32012
CB4856	CT	II	3324021	3326431	WBGene00021589	Y46D2A.1	Y46D2A.1	-0.194748079950582	AGIWUR28811
CB4856	CT	II	3326492	3327197	WBGene00021591	Y46D2A.3	Y46D2A.3	-0.297033252611197	AGIWUR26527
CB4856	CT	II	3331889	3334205	WBGene00077751	irld-27	F14D2.17	0.201740603991466	AGIWUR2293
CB4856	CT	II	3335422	3337139	WBGene00206522	F14D2.19	F14D2.19	-0.17848436120645	AGIWUR31445

CB4856	CT	II	3340556	3341260	WBGene00017453	F14D2.5	F14D2.5	0.0505311640388282	AGIWUR12906
CB4856	CT	II	3341268	3342104	WBGene00017459	F14D2.11	F14D2.11	-0.11654259403284	AGIWUR40910
CB4856	CT	II	3343710	3344967	WBGene00017461	bath-28	F14D2.13	-0.0708363884473666	AGIWUR23578;AGIWUR35439;AGIWUR38267;AGIWUR40583
CB4856	CT	II	3345218	3346378	WBGene00017460	bath-30	F14D2.12	-0.161950353484816	AGIWUR37539
CB4856	CT	II	3346745	3347519	WBGene00017462	F14D2.14	F14D2.14	-0.306679658926695	AGIWUR23383;AGIWUR28782;AGIWUR38077
CB4856	CT	II	3347574	3349870	WBGene00017456	F14D2.8	F14D2.8	-0.0263717277663496	AGIWUR8496;AGIWUR16471;AGIWUR17696;AGIWUR37394;AGIWUR38928
CB4856	CT	II	3350252	3358981	WBGene00017452	bath-29	F14D2.4	-0.189053120782699	AGIWUR8805;AGIWUR22859;AGIWUR23701;AGIWUR25164;AGIWUR32290
CB4856	CT	II	3359139	3360255	WBGene00017451	F14D2.2	F14D2.2	-0.256395118847504	AGIWUR24777
CB4856	CT	II	3361899	3365961	WBGene00021728	Y49F6C.6	Y49F6C.6	-0.184605813419997	AGIWUR8396
CB4856	CT	II	3367258	3368197	WBGene00021729	Y49F6C.7	Y49F6C.7	0.100712738202548	AGIWUR17291
CB4856	CT	II	3369175	3370111	WBGene00021730	Y49F6C.8	Y49F6C.8	-0.344511313415799	AGIWUR7299;AGIWUR13872;AGIWUR34677
CB4856	CT	II	3371310	3372145	WBGene00021727	bath-23	Y49F6C.5	-0.228850568549796	AGIWUR11832
CB4856	CT	II	3373169	3374185	WBGene00219758	linc-11	Y49F6C.11	-0.189809831467703	AGIWUR23245;AGIWUR36507
CB4856	CT	II	3375079	3376714	WBGene00021726	bath-10	Y49F6C.4	0.51474345355313	AGIWUR38786
CB4856	CT	II	3377111	3378726	WBGene00021725	bath-9	Y49F6C.3	0.0635852461899873	AGIWUR26956
CB4856	CT	II	3378575	3379551	WBGene00021724	Y49F6C.2	Y49F6C.2	0.0503210916535339	AGIWUR42499
CB4856	CT	II	3380288	3381403	WBGene00021723	bath-8	Y49F6C.1	-0.0754051424631328	AGIWUR16875;AGIWUR21492;AGIWUR35386
CB4856	CT	II	3381836	3384017	WBGene00020290	T06D4.1	T06D4.1	0.0172169171897245	AGIWUR42268
CB4856	CT	II	3388280	3391586	WBGene00020292	nep-19	T06D4.3	0.144582179934923	AGIWUR35625
CB4856	CT	II	3392036	3395533	WBGene00020293	nep-20	T06D4.4	-0.552169045901339	AGIWUR13318
CB4856	CT	II	3395821	3399303	WBGene00017554	nep-9	F18A12.5	-0.194920863605539	AGIWUR32423;AGIWUR41571;AGIWUR43562
CB4856	CT	II	3399754	3402925	WBGene00017555	nep-10	F18A12.6	-0.701094895267688	AGIWUR28365

CB4856	CT	II	3403835	3406838	WBGene00017553	nep-8	F18A12.4	-0.370707688599877	AGIWUR7990
CB4856	CT	II	3406951	3409774	WBGene00017552	nep-7	F18A12.3	0.0814829384620022	AGIWUR5802;AGIWUR20138
CB4856	CT	II	3411425	3414469	WBGene00017556	F18A12.7	F18A12.7	0.327222628390482	AGIWUR10742
CB4856	CT	II	3415005	3416250	WBGene00017551	F18A12.2	F18A12.2	-0.469932350503925	AGIWUR39979
CB4856	CT	II	3416838	3420671	WBGene00017550	nep-6	F18A12.1	0.0106083018918506	AGIWUR29679;AGIWUR39896
CB4856	CT	II	3420653	3425533	WBGene00017557	nep-11	F18A12.8	0.593705854981336	AGIWUR10985;AGIWUR21992
CB4856	CT	II	3429406	3431303	WBGene00015855	C16C8.18	C16C8.18	-0.0784880667667061	AGIWUR10454
CB4856	CT	II	3431286	3436512	WBGene00015841	skpo-2	C16C8.2	0.167017866886198	AGIWUR13637
CB4856	CT	II	3440827	3442445	WBGene00015854	C16C8.17	C16C8.17	-0.101676064029792	AGIWUR17336
CB4856	CT	II	3443488	3444380	WBGene00015842	C16C8.4	C16C8.4	0.0164875512463212	AGIWUR11008
CB4856	CT	II	3444689	3445381	WBGene00015843	C16C8.5	C16C8.5	0.238272891429803	AGIWUR10128
CB4856	CT	II	3448206	3448759	WBGene00195071	C16C8.21	C16C8.21	0.071633665371472	AGIWUR6579
CB4856	CT	II	3449562	3450994	WBGene00015852	C16C8.14	C16C8.14	-0.011199423765424	AGIWUR21154
CB4856	CT	II	3452721	3453602	WBGene00015850	C16C8.12	C16C8.12	0.0118878934201412	AGIWUR31623
CB4856	CT	II	3453708	3454688	WBGene00015849	C16C8.11	C16C8.11	0.122071156918633	AGIWUR34563
CB4856	CT	II	3458501	3459023	WBGene00015856	C16C8.19	C16C8.19	-0.519671966968039	AGIWUR34765
CB4856	CT	II	3459619	3460314	WBGene00015845	C16C8.7	C16C8.7	-0.25120911847262	AGIWUR32514
CB4856	CT	II	3461112	3461681	WBGene00015846	C16C8.8	C16C8.8	-0.35007964724028	AGIWUR41834
CB4856	CT	II	3462570	3463194	WBGene00015847	C16C8.9	C16C8.9	-0.37928558465315	AGIWUR25088
CB4856	CT	II	3464155	3464680	WBGene00015848	C16C8.10	C16C8.10	-0.159187746626415	AGIWUR44392
CB4856	CT	II	3470060	3471703	WBGene00044377	Y49F6B.13	Y49F6B.13	-0.0671877824324691	AGIWUR18112;AGIWUR21244;AGIWUR23083;AGIWUR31974;AGIWUR35691;AGIWUR36406
CB4856	CT	II	3472075	3472430	WBGene00044376	Y49F6B.12	Y49F6B.12	-0.150166701812994	AGIWUR8840;AGIWUR19052;AGIWUR20133
CB4856	CT	II	3472879	3473289	WBGene00021718	Y49F6B.6	Y49F6B.6	-0.37189881209787	

AGIWUR26738
CB4856 CT II 3474050 3474729 WBGene00021717 sdz-32 Y49F6B.5 0.134404554556638
AGIWUR40496
CB4856 CT II 3475644 3477271 WBGene00021719 fbxb-104 Y49F6B.7 0.147560835325512
AGIWUR1658
CB4856 CT II 3479473 3481685 WBGene00021720 Y49F6B.8 Y49F6B.8 -0.21230773766268
AGIWUR15937
CB4856 CT II 3482408 3490143 WBGene00004896 smu-2 Y49F6B.4 -0.146891327625697
AGIWUR14962;AGIWUR16930;AGIWUR17405;AGIWUR39392
CB4856 CT II 3491179 3492881 WBGene00021716 fbxb-112 Y49F6B.3 0.168037943414637
AGIWUR39175
CB4856 CT II 3493934 3499426 WBGene00021721 Y49F6B.9 Y49F6B.9 -0.0876636639646513
AGIWUR7086;AGIWUR11562;AGIWUR16713;AGIWUR26276;AGIWUR36914;AGIWUR39027
CB4856 CT II 3500077 3502449 WBGene00000647 col-71 Y49F6B.10 -0.227403214067406
AGIWUR4133
CB4856 CT II 3502968 3504910 WBGene00021715 Y49F6B.2 Y49F6B.2 -0.294536272991479
AGIWUR6288
CB4856 CT II 3505017 3509956 WBGene00021714 cyh-1 Y49F6B.1 -0.404725581444943
AGIWUR13832
CB4856 CT II 3512690 3514731 WBGene00021722 srz-5 Y49F6B.11 -0.539263902986174
AGIWUR17341
CB4856 CT II 3514963 3515484 WBGene00044378 Y49F6B.14 Y49F6B.14 -0.0268935927092766
AGIWUR5485;AGIWUR5867;AGIWUR23289
CB4856 CT II 3516008 3518898 WBGene00021118 clec-125 W09G10.6 -0.587384010401889
AGIWUR25512
CB4856 CT II 3519494 3520421 WBGene00021117 clec-126 W09G10.5 -0.114105012998087
AGIWUR40239
CB4856 CT II 3520703 3524180 WBGene00000648 col-72 W09G10.1 -0.294321071467004
AGIWUR26693
CB4856 CT II 3525172 3528516 WBGene00021116 ncs-6 W09G10.3 -0.102494873693342
AGIWUR29131
CB4856 CT II 3532527 3546431 WBGene00000162 apd-3 W09G10.4 0.0629474925288456
AGIWUR18273
CB4856 CT II 3550651 3553437 WBGene00021148 W10G11.19 W10G11.19 0.171190320608982
AGIWUR32549
CB4856 CT II 3553724 3554618 WBGene00021149 dnc-3 W10G11.20 -0.0921330044556825
AGIWUR15212
CB4856 CT II 3557474 3567490 WBGene00021146 lgc-45 W10G11.16 -0.234078519357228
AGIWUR3670;AGIWUR23297;AGIWUR31206;AGIWUR38708

CB4856	CT	II	3568117	3569218	WBGene00021138	clec-127	W10G11.5	0.0714505293117336	AGIWUR29457
CB4856	CT	II	3569430	3571027	WBGene00021139	clec-128	W10G11.6	0.223192006813474	AGIWUR17823
CB4856	CT	II	3571491	3572883	WBGene00021145	clec-129	W10G11.15	-0.228491282005591	AGIWUR20030
CB4856	CT	II	3573556	3574652	WBGene00021144	clec-130	W10G11.14	0.0535665956889981	AGIWUR4511;AGIWUR10186;AGIWUR26533;AGIWUR28817
CB4856	CT	II	3574699	3576201	WBGene00021140	clec-131	W10G11.7	0.115461750150692	AGIWUR21113
CB4856	CT	II	3577045	3578110	WBGene00021143	clec-132	W10G11.13	0.206502047793515	AGIWUR29293
CB4856	CT	II	3578421	3579031	WBGene00021142	clec-133	W10G11.12	0.0357519758777762	AGIWUR40246
CB4856	CT	II	3580083	3581433	WBGene00021141	clec-134	W10G11.11	-0.0836217568611985	AGIWUR34394
CB4856	CT	II	3582052	3583505	WBGene00005281	srh-59	W10G11.10	0.386246124643564	AGIWUR25922
CB4856	CT	II	3583987	3586063	WBGene00005282	srh-60	W10G11.9	-0.174723708012309	AGIWUR8096
CB4856	CT	II	3603387	3607384	WBGene00021711	wago-11	Y49F6A.1	-0.279992559109852	AGIWUR4569;AGIWUR22972;AGIWUR38323
CB4856	CT	II	3607937	3609862	WBGene00021713	Y49F6A.5	Y49F6A.5	-0.270906715078143	AGIWUR38967
CB4856	CT	II	3610409	3615352	WBGene00018235	F40E12.2	F40E12.2	-0.101787498740383	AGIWUR15978
CB4856	CT	II	3617812	3618621	WBGene00018234	F40E12.1	F40E12.1	-0.0356485818996841	AGIWUR20499
CB4856	CT	II	3624198	3626950	WBGene00019796	M151.4	M151.4	-0.12465442377718	AGIWUR7391
CB4856	CT	II	3629295	3630860	WBGene00219238	M151.9	M151.9	0.0341467939995397	AGIWUR19947
CB4856	CT	II	3631444	3633875	WBGene00019794	M151.2	M151.2	-0.23933453663665	AGIWUR40444
CB4856	CT	II	3637811	3639338	WBGene00019797	fbxb-31	M151.5	-0.0197141342151408	AGIWUR21468
CB4856	CT	II	3641500	3644082	WBGene00019793	M151.1	M151.1	0.125583932643688	AGIWUR37972
CB4856	CT	II	3645427	3646982	WBGene00019798	fbxb-32	M151.6	-0.0507356929316281	AGIWUR21526
CB4856	CT	II	3647411	3648599	WBGene00044314	fbxb-33	M151.8	-0.0309114456274848	AGIWUR37742
CB4856	CT	II	3651256	3654276	WBGene00017590	F19B10.11	F19B10.11	-0.19011078160943	AGIWUR19558
CB4856	CT	II	3655678	3657732	WBGene00017587	F19B10.5	F19B10.5	-0.312515672514387	AGIWUR136

CB4856	CT	II	3662204	3662618	WBGene00194713	F19B10.13	F19B10.13	0.195387118587346	AGIWUR21305
CB4856	CT	II	3663509	3663896	WBGene00017588	F19B10.6	F19B10.6	0.088846137349643	AGIWUR32405
CB4856	CT	II	3664404	3665120	WBGene00017585	F19B10.3	F19B10.3	-0.111178052505167	AGIWUR9260
CB4856	CT	II	3667179	3668664	WBGene00005988	srx-97	F19B10.7	-0.0730008912408628	AGIWUR11010
CB4856	CT	II	3669321	3671056	WBGene00005989	srx-98	F19B10.8	-0.198061059605937	AGIWUR27383
CB4856	CT	II	3671908	3673593	WBGene00004750	sea-1	F19B10.9	-0.194188434568165	AGIWUR13545
CB4856	CT	II	3675624	3676733	WBGene00017584	F19B10.2	F19B10.2	-0.283128084371689	AGIWUR1693;AGIWUR12007;AGIWUR13921;AGIWUR30059;AGIWUR30085;AGIWUR31919
CB4856	CT	II	3677535	3681415	WBGene00017589	F19B10.10	F19B10.10	-0.36700836600653	AGIWUR2171
CB4856	CT	II	3683177	3684996	WBGene00017583	F19B10.1	F19B10.1	-0.302737634498488	AGIWUR4480;AGIWUR5713;AGIWUR20096
CB4856	CT	II	3685952	3687133	WBGene00005990	srx-99	F19B10.12	-0.0205644441621451	AGIWUR110;AGIWUR17748
CB4856	CT	II	3687791	3688691	WBGene00044379	F40H7.12	F40H7.12	0.0997654228072307	AGIWUR26635
CB4856	CT	II	3689467	3690746	WBGene00005992	srx-101	F40H7.4	0.00212898296207342	AGIWUR24580;AGIWUR30666
CB4856	CT	II	3691784	3693231	WBGene00005991	srx-100	F40H7.3	-0.184315415757319	AGIWUR33715
CB4856	CT	II	3693401	3694764	WBGene00005993	srx-102	F40H7.5	0.0948838347054162	AGIWUR22545
CB4856	CT	II	3695383	3698162	WBGene00005994	srx-103	F40H7.11	-0.0947528724744231	AGIWUR8577
CB4856	CT	II	3698599	3699691	WBGene00005995	srx-104	F40H7.7	0.176676086190374	AGIWUR19918;AGIWUR37276
CB4856	CT	II	3699834	3701405	WBGene00005996	srx-105	F40H7.8	0.100200380404568	AGIWUR30107
CB4856	CT	II	3701708	3703250	WBGene00005997	srx-106	F40H7.9	0.133105942191522	AGIWUR13997
CB4856	CT	II	3703721	3704945	WBGene00005998	srx-107	F40H7.10	0.179302812496315	AGIWUR31004
CB4856	CT	II	3705620	3707065	WBGene00005999	srx-108	F40H7.2	-0.139440732999617	AGIWUR15821
CB4856	CT	II	3707610	3709221	WBGene00006000	srx-109	F40H7.1	0.156553367823361	AGIWUR710;AGIWUR29482
CB4856	CT	II	3709555	3710952	WBGene00006001	srx-110	C46E10.6	0.377387235667445	AGIWUR33826

CB4856	CT	II	3711293	3713048	WBGene00016711	fbxc-53	C46E10.5	0.4258677098788	AGIWUR35329
CB4856	CT	II	3713188	3714695	WBGene00005319	srh-100	C46E10.10	-0.123931234608407	AGIWUR15850
CB4856	CT	II	3715000	3716555	WBGene00005318	srh-99	C46E10.7	0.395269602866556	AGIWUR7220
CB4856	CT	II	3717138	3719768	WBGene00016710	fbxc-52	C46E10.4	-0.064106955700737	AGIWUR29686;AGIWUR42598
CB4856	CT	II	3721316	3723084	WBGene00016709	C46E10.3	C46E10.3	0.152677280328518	AGIWUR4335
CB4856	CT	II	3723378	3723896	WBGene00016708	C46E10.2	C46E10.2	-0.0145639775504378	AGIWUR1714
CB4856	CT	II	3724225	3725909	WBGene00016707	C46E10.1	C46E10.1	-0.50107631626908	AGIWUR15609
CB4856	CT	II	3725906	3727479	WBGene00016712	C46E10.8	C46E10.8	-0.178090374113316	AGIWUR300;AGIWUR7910;AGIWUR9883;AGIWUR13329;AGIWUR14299;AGIWUR34806
CB4856	CT	II	3727690	3729707	WBGene00016713	C46E10.9	C46E10.9	-0.228426664816011	AGIWUR9856
CB4856	CT	II	3730152	3731548	WBGene00017406	sdz-12	F12E12.5	-0.264962651117028	AGIWUR42036
CB4856	CT	II	3731973	3733203	WBGene00017405	bath-31	F12E12.4	-0.0309145109865732	AGIWUR7461;AGIWUR23673;AGIWUR27896
CB4856	CT	II	3735929	3736943	WBGene00017407	F12E12.6	F12E12.6	-0.359323160901429	AGIWUR2208
CB4856	CT	II	3737334	3738282	WBGene00017404	F12E12.3	F12E12.3	-0.340301553703777	AGIWUR1974
CB4856	CT	II	3739947	3740837	WBGene00017403	F12E12.2	F12E12.2	-0.092213798518717	AGIWUR28417
CB4856	CT	II	3741884	3742509	WBGene00017402	F12E12.1	F12E12.1	-0.0979784902192335	AGIWUR38937
CB4856	CT	II	3742465	3743819	WBGene00044811	F12E12.11	F12E12.11	-0.0308671913400573	AGIWUR40004
CB4856	CT	II	3746474	3747459	WBGene00044693	fbxb-92	F12E12.9	0.0535063893013392	AGIWUR35746
CB4856	CT	II	3747679	3748819	WBGene00044694	fbxb-90	F12E12.10	0.103790564495965	AGIWUR34471
CB4856	CT	II	3749061	3750785	WBGene00044692	fbxb-91	F12E12.8	0.0736689468768277	AGIWUR19698;AGIWUR21274;AGIWUR30396;AGIWUR32964;AGIWUR40368;AGIWUR41591
CB4856	CT	II	3751261	3752284	WBGene00017408	sdz-11	F12E12.7	-0.35202611187886	AGIWUR23637;AGIWUR41398

CB4856 CT II 3753921 3757428 WBGene00020774 T24E12.5 T24E12.5 -0.342411437619464
AGIWUR16825;AGIWUR41472
CB4856 CT II 3757799 3759252 WBGene00006002 srx-111 T24E12.4 0.157755202324282
AGIWUR28133
CB4856 CT II 3759581 3760036 WBGene00044380 T24E12.12 T24E12.12 -0.0283053864848064
AGIWUR2239;AGIWUR5391;AGIWUR24035;AGIWUR25488;AGIWUR25844
CB4856 CT II 3760300 3761699 WBGene00020775 T24E12.6 T24E12.6 0.0430433458369741
AGIWUR13969;AGIWUR17251;AGIWUR23816;AGIWUR33656;AGIWUR36990;AGIWUR38176;AGIWUR40059
CB4856 CT II 3763447 3764586 WBGene00006003 srx-112 T24E12.8 0.0397392190020578
AGIWUR14156;AGIWUR23415
CB4856 CT II 3765087 3773191 WBGene00020777 T24E12.10 T24E12.10 0.187331106583484
AGIWUR10671;AGIWUR13962
CB4856 CT II 3776019 3777459 WBGene00020773 T24E12.3 T24E12.3 -0.300348802014009
AGIWUR9708
CB4856 CT II 3778406 3779563 WBGene00020778 T24E12.11 T24E12.11 -0.214261397653242
AGIWUR41241
CB4856 CT II 3782950 3784156 WBGene00020772 T24E12.2 T24E12.2 -0.157649212683532
AGIWUR44647
CB4856 CT II 3787451 3788479 WBGene00195077 T24E12.13 T24E12.13 0.244853970134344
AGIWUR25725
CB4856 CT II 3790750 3791395 WBGene00021173 Y8A9A.4 Y8A9A.4 -0.29553286697491 AGIWUR33339
CB4856 CT II 3791534 3792102 WBGene00021172 Y8A9A.3 Y8A9A.3 0.0936119784737226 AGIWUR18649
CB4856 CT II 3792524 3793425 WBGene00021174 fbxb-99 Y8A9A.5 0.153839962146009 AGIWUR23693
CB4856 CT II 3795623 3800608 WBGene00021171 Y8A9A.2 Y8A9A.2 1.37818001216684 AGIWUR2883
CB4856 CT II 3801171 3802589 WBGene00005380 srh-164 Y8A9A.1 -0.183310934527498
AGIWUR8919;AGIWUR15796
CB4856 CT II 3802959 3803324 WBGene00002115 ins-32 Y8A9A.6 -0.489478674684762 AGIWUR20661
CB4856 CT II 3805493 3807053 WBGene00018805 F54D10.4 F54D10.4 -0.036774551278838
AGIWUR4615;AGIWUR13344;AGIWUR27279;AGIWUR40304
CB4856 CT II 3807096 3808223 WBGene00018806 F54D10.5 F54D10.5 -0.183338578147021
AGIWUR8260
CB4856 CT II 3812284 3813426 WBGene00018804 F54D10.3 F54D10.3 0.0811516565971466
AGIWUR4759
CB4856 CT II 3814376 3815458 WBGene00018807 fbxa-146 F54D10.6 0.333687926489327
AGIWUR5537;AGIWUR8359;AGIWUR25939
CB4856 CT II 3816686 3818238 WBGene00018803 fbxa-24 F54D10.2 3.38858573187989
AGIWUR14133
CB4856 CT II 3820152 3821312 WBGene00018808 F54D10.7 F54D10.7 -0.739556255012965
AGIWUR22587

CB4856	CT	II	3824148	3825614	WBGene00004821	skr-15	F54D10.1	-0.0774067332965691	AGIWUR10492;AGIWUR17038;AGIWUR17734;AGIWUR27405;AGIWUR28048;AGIWUR29669
CB4856	CT	II	3825661	3827879	WBGene00018809	F54D10.8	F54D10.8	-0.152574230272222	AGIWUR13369
CB4856	CT	II	3829584	3831914	WBGene00018810	F54D10.9	F54D10.9	-0.184688597281174	AGIWUR17912
CB4856	CT	II	3833250	3836015	WBGene00015873	oac-4	C17A2.5	-0.109069861080251	AGIWUR26649
CB4856	CT	II	3837338	3838948	WBGene00015874	C17A2.6	C17A2.6	0.237231670919456	AGIWUR38752
CB4856	CT	II	3839862	3841272	WBGene00015872	C17A2.4	C17A2.4	0.252145605810545	AGIWUR34717
CB4856	CT	II	3841914	3843405	WBGene00015875	C17A2.7	C17A2.7	0.13433486787511	AGIWUR18139
CB4856	CT	II	3843609	3845509	WBGene00015871	C17A2.3	C17A2.3	-0.147926275205925	AGIWUR3940
CB4856	CT	II	3846328	3848143	WBGene00015870	C17A2.2	C17A2.2	0.0611162720945106	AGIWUR43109
CB4856	CT	II	3849053	3850725	WBGene00003662	nhr-72	C17A2.8	-0.0663174052032423	AGIWUR32794
CB4856	CT	II	3852954	3854615	WBGene00015869	nhr-257	C17A2.1	-0.212840131534396	AGIWUR8067
CB4856	CT	II	3855921	3856588	WBGene00016775	C49D10.7	C49D10.7	0.0500025233893728	AGIWUR8239
CB4856	CT	II	3858142	3860926	WBGene00016776	oac-11	C49D10.8	-0.0417448492368022	AGIWUR15000
CB4856	CT	II	3863084	3864752	WBGene00016777	nhr-261	C49D10.9	-0.295818570345523	AGIWUR1635
CB4856	CT	II	3866798	3868243	WBGene00003665	nhr-75	C49D10.6	-0.353676522264339	AGIWUR6203
CB4856	CT	II	3869901	3873010	WBGene00016773	oac-10	C49D10.4	-0.0105797837609405	AGIWUR323;AGIWUR3711;AGIWUR5110;AGIWUR38159
CB4856	CT	II	3873541	3877227	WBGene00016778	nep-3	C49D10.10	-0.135742099293683	AGIWUR25432;AGIWUR28939;AGIWUR31245
CB4856	CT	II	3877249	3877974	WBGene00016779	sdz-7	C49D10.11	0.284473951286259	AGIWUR11675
CB4856	CT	II	3878630	3879891	WBGene00005456	srh-250	C49D10.3	-0.109828698884474	AGIWUR11362
CB4856	CT	II	3880245	3882182	WBGene00016772	nhr-166	C49D10.2	-0.212126578763311	AGIWUR42489
CB4856	CT	II	3885032	3887995	WBGene00005311	srh-90	F53C3.10	0.205952284642416	AGIWUR14811;AGIWUR30096;AGIWUR37788
CB4856	CT	II	3891424	3893574	WBGene00018752	F53C3.8	F53C3.8	0.365735619058167	AGIWUR22070
CB4856	CT	II	3894841	3896239	WBGene00018751	F53C3.7	F53C3.7	-0.0340444181148456	AGIWUR24285
CB4856	CT	II	3898142	3899663	WBGene00018750	F53C3.6	F53C3.6	0.465959837035128	AGIWUR1696;AGIWUR34501
CB4856	CT	II	3899851	3900783	WBGene00018749	F53C3.5	F53C3.5	0.281079378054942	AGIWUR22853

CB4856	CT	II	3900788 3902773 WBGene00018748	F53C3.4 F53C3.4 -0.172194414011067
AGIWUR7950;AGIWUR9230;AGIWUR14856;AGIWUR22223;AGIWUR35451;AGIWUR35695
CB4856	CT	II	3902860 3904794 WBGene00018747	F53C3.3 F53C3.3 -0.683504225787367	AGIWUR37908
CB4856	CT	II	3904816 3906620 WBGene00018754	F53C3.11	F53C3.11	-0.236376560759474
AGIWUR18190
CB4856	CT	II	3907140 3908478 WBGene00018746	fbxb-103	F53C3.2 0.0868422951708361
AGIWUR2972
CB4856	CT	II	3908491 3910837 WBGene00018755	bcmo-2	F53C3.12	0.609302565320197
AGIWUR28605
CB4856	CT	II	3912003 3913385 WBGene00018745	F53C3.1 F53C3.1 -0.00113814695027692	AGIWUR32457
CB4856	CT	II	3919794 3927982 WBGene00018756	F53C3.13	F53C3.13	0.134284257052219
AGIWUR8437;AGIWUR8647;AGIWUR8821;AGIWUR10822;AGIWUR13228;AGIWUR16084;AGIWUR17645;AGIWUR19318;AGIWUR24545;AGIWUR27913;AGIWUR30509;AGIWUR33161;AGIWUR39802
CB4856	CT	II	3932178 3933740 WBGene00021192	Y14H12B.2	Y14H12B.2	-0.0900320258928051
AGIWUR14725
CB4856	CT	II	3934451 3936008 WBGene00021191	Y14H12B.1	Y14H12B.1	0.0314811001914572
AGIWUR6516;AGIWUR9325
CB4856	CT	II	3950145 3972137 WBGene00004751	sea-2	K10G6.3 -0.072875002334273
AGIWUR6369;AGIWUR29408
CB4856	CT	II	3972820 3974968 WBGene00019641	K10G6.4 K10G6.4 0.83174377329337	AGIWUR42914
CB4856	CT	II	3976246 3977969 WBGene00019640	dos-2	K10G6.2 -0.031729249073571	AGIWUR16014
CB4856	CT	II	3982549 3986944 WBGene00003017	lin-31	K10G6.1 -0.193692496886764
AGIWUR37327;AGIWUR44727
CB4856	CT	II	3987281 3988185 WBGene00044381	K10G6.5 K10G6.5 -0.365209995653031
AGIWUR15812;AGIWUR23686;AGIWUR40325
CB4856	CT	II	3988700 3989677 WBGene00044382	Y14H12A.2	Y14H12A.2	0.0275365598494366
AGIWUR5795;AGIWUR13726;AGIWUR24093;AGIWUR28723;AGIWUR29110;AGIWUR34219;AGIWUR36017;AGIWUR38335;AGIWUR43600;AGIWUR45160
CB4856	CT	II	3993210 3993887 WBGene00021190	Y14H12A.1	Y14H12A.1	-0.434907956701691
AGIWUR37602
CB4856	CT	II	4004885 4016072 WBGene00019487	ephx-1	K07D4.7 0.37933837010879	AGIWUR1973
CB4856	CT	II	4016827 4018701 WBGene00019486	K07D4.6 K07D4.6 0.151555054325877	AGIWUR37055
CB4856	CT	II	4020482 4025562 WBGene00019485	K07D4.5 K07D4.5 0.137796601784257
AGIWUR4273;AGIWUR23840;AGIWUR27598;AGIWUR27867;AGIWUR39078
CB4856	CT	II	4028577 4029985 WBGene00019488	K07D4.9 K07D4.9 0.722464019798139	AGIWUR12587
CB4856	CT	II	4031214 4032387 WBGene00019484	K07D4.4 K07D4.4 -0.0531143971873478	AGIWUR27836
CB4856	CT	II	4038868 4043070 WBGene00004135	pqn-48	K07D4.8 0.230966193520766	AGIWUR44569
CB4856	CT	II	4043059 4044330 WBGene00004467	rpn-11	K07D4.3 -0.152484822462595
AGIWUR2574;AGIWUR13756;AGIWUR14661;AGIWUR25654;AGIWUR35845;AGIWUR37390

CB4856	CT	II	4044771	4047247	WBGene00019483	K07D4.2	K07D4.2	-0.282604331122764	AGIWUR20974
CB4856	CT	II	4048525	4049129	WBGene00023318	K07D4.1	K07D4.1	-0.497838129848281	AGIWUR22099
CB4856	CT	II	4055939	4057707	WBGene00021055	W06A11.4	W06A11.4	-0.199290931113829	AGIWUR4127;AGIWUR22591;AGIWUR24503;AGIWUR43789
CB4856	CT	II	4063008	4064057	WBGene00021054	fbxb-118	W06A11.3	0.0683895668798309	AGIWUR5599;AGIWUR16342;AGIWUR21363;AGIWUR28791
CB4856	CT	II	4067558	4069771	WBGene00021052	W06A11.1	W06A11.1	-0.159492323658324	AGIWUR2631;AGIWUR13752;AGIWUR14821
CB4856	CT	II	4070009	4072920	WBGene00020009	R11F4.3	R11F4.3	-0.153984313328527	AGIWUR29471;AGIWUR29931
CB4856	CT	II	4083065	4086226	WBGene00020008	R11F4.2	R11F4.2	-0.0478171338887918	AGIWUR4756;AGIWUR10772;AGIWUR13044;AGIWUR25629;AGIWUR33062;AGIWUR35536
CB4856	CT	II	4091837	4094349	WBGene00020007	R11F4.1	R11F4.1	1.02738751987807	AGIWUR5157;AGIWUR18910;AGIWUR23303;AGIWUR32277
CB4856	CT	II	4100788	4127849	WBGene00006476	rhgf-2	T08H4.1	0.385317907382378	AGIWUR5268;AGIWUR12668;AGIWUR13047;AGIWUR20456;AGIWUR23101;AGIWUR31904;AGIWUR40324
CB4856	CT	II	4127848	4134867	WBGene00020368	ast-1	T08H4.3	0.9316331514981	AGIWUR8003;AGIWUR11487
CB4856	CT	II	4140901	4141105	WBGene00021822	Y54C5A.1	Y54C5A.1	0.187886542263729	AGIWUR32585
CB4856	CT	II	4142816	4149678	WBGene00018969	F56D3.1	F56D3.1	0.205128400338574	AGIWUR37694
CB4856	CT	II	4155773	4167368	WBGene00019855	R03H10.2	R03H10.2	0.751918872173722	AGIWUR7979
CB4856	CT	II	4167555	4168500	WBGene00019856	R03H10.4	R03H10.4	0.177267250081837	AGIWUR36427
CB4856	CT	II	4169723	4170608	WBGene00019857	R03H10.5	R03H10.5	0.224994702417584	AGIWUR35423
CB4856	CT	II	4171294	4173875	WBGene00019858	R03H10.6	R03H10.6	0.340041260997092	AGIWUR30977
CB4856	CT	II	4180271	4183545	WBGene00019854	R03H10.1	R03H10.1	0.457129466008154	AGIWUR32688
CB4856	CT	II	4186666	4208499	WBGene00006495	cpna-1	F31D5.3	0.442920544922485	AGIWUR1322;AGIWUR15868;AGIWUR24333
CB4856	CT	II	4211425	4213335	WBGene00206414	F31D5.7	F31D5.7	0.412716189604965	AGIWUR22076
CB4856	CT	II	4215605	4218243	WBGene00017945	F31D5.2	F31D5.2	-0.269367832535767	AGIWUR31542
CB4856	CT	II	4223309	4226330	WBGene00015818	C16A11.5	C16A11.5	-0.263463077250709	AGIWUR789;AGIWUR26752;AGIWUR44848
CB4856	CT	II	4227046	4229157	WBGene00015817	C16A11.4	C16A11.4	-0.224501355073958	AGIWUR4751;AGIWUR14422;AGIWUR22204;AGIWUR24954;AGIWUR35304;AGIWUR42962

CB4856	CT	II	4234950	4235982	WBGene00045297	fbxc-43	C16A11.9	0.320240034945668	AGIWUR12352
CB4856	CT	II	4236504	4237751	WBGene00015819	fbxc-44	C16A11.6	-0.0570527700693626	AGIWUR11430;AGIWUR38404
CB4856	CT	II	4238159	4244830	WBGene00015815	C16A11.2	C16A11.2	-0.0475485854354893	AGIWUR22166
CB4856	CT	II	4247197	4255281	WBGene00001673	gpa-11	C16A11.1	0.27651276887824	AGIWUR6819
CB4856	CT	II	4255848	4256989	WBGene00015820	C16A11.7	C16A11.7	0.0872954744671697	AGIWUR34626
CB4856	CT	II	4257092	4258781	WBGene00015821	clec-135	C16A11.8	-0.294900798857165	AGIWUR36533
CB4856	CT	II	4276209	4279268	WBGene00015299	C01F1.4	C01F1.4	-0.137401401075637	AGIWUR35062
CB4856	CT	II	4279424	4281967	WBGene00015300	C01F1.5	C01F1.5	-0.344979793053277	AGIWUR3406
CB4856	CT	II	4282321	4288211	WBGene00015301	C01F1.6	C01F1.6	0.137462129425213	AGIWUR39703
CB4856	CT	II	4295101	4299006	WBGene00015298	C01F1.3	C01F1.3	0.161948762851958	AGIWUR11357
CB4856	CT	II	4299653	4300919	WBGene00015297	sco-1	C01F1.2	-0.400862154566042	AGIWUR1126
CB4856	CT	II	4301607	4305287	WBGene00015296	C01F1.1	C01F1.1	-0.102547346015035	AGIWUR3997
CB4856	CT	II	4305262	4307004	WBGene00021796	Y52E8A.3	Y52E8A.3	0.11063625330384	AGIWUR25352
CB4856	CT	II	4309725	4312215	WBGene00021797	plep-1	Y52E8A.4	-1.06064866086971	AGIWUR31602
CB4856	CT	II	4314417	4316111	WBGene00005787	srw-40	Y52E8A.5	0.147387121691035	AGIWUR31610
CB4856	CT	II	4316629	4319969	WBGene00021798	Y52E8A.6	Y52E8A.6	-0.0902396045380058	AGIWUR8856
CB4856	CT	II	4320836	4321654	WBGene00021795	Y52E8A.2	Y52E8A.2	0.0970879166145142	AGIWUR13205
CB4856	CT	II	4321663	4322616	WBGene00021794	Y52E8A.1	Y52E8A.1	-0.549901675934173	AGIWUR2488
CB4856	CT	II	4323256	4324187	WBGene00019221	H20J04.6	H20J04.6	-0.331896100494106	AGIWUR29158
CB4856	CT	II	4324361	4326089	WBGene00003390	mog-2	H20J04.8	-0.100239878478377	AGIWUR24324;AGIWUR26270
CB4856	CT	II	4326720	4328284	WBGene00019220	pfd-2	H20J04.5	-0.101619277515681	AGIWUR28829
CB4856	CT	II	4329023	4334484	WBGene00019219	H20J04.4	H20J04.4	-0.0882864554825105	AGIWUR12223;AGIWUR19295;AGIWUR19960
CB4856	CT	II	4335707	4338424	WBGene00019218	madf-3	H20J04.3	-0.0362166068387495	

AGIWUR41477
CB4856 CT II 4340151 4350984 WBGene00019217 athp-2 H20J04.2 0.0898239666588765
AGIWUR13157
CB4856 CT II 4351188 4353159 WBGene00044310 H20J04.9 H20J04.9 -0.0349588276751236
AGIWUR27110;AGIWUR39688
CB4856 CT II 4353221 4355317 WBGene00019222 H20J04.7 H20J04.7
0.000931581112936897 AGIWUR32453
CB4856 CT II 4355633 4358844 WBGene00019216 H20J04.1 H20J04.1 -0.342212589420789
AGIWUR42306
CB4856 CT II 4359082 4368332 WBGene00002252 lat-2 B0286.2 -0.219102567268648 AGIWUR17581
CB4856 CT II 4368490 4369692 WBGene00023425 try-9 B0286.6 -0.225754475770398 AGIWUR47
CB4856 CT II 4369625 4372597 WBGene00015116 pacs-1 B0286.3 -0.510745656464245 AGIWUR37001
CB4856 CT II 4372740 4376742 WBGene00003825 ntl-2 B0286.4 0.106966820088207
AGIWUR9456;AGIWUR25085;AGIWUR30605;AGIWUR31866;AGIWUR32151
CB4856 CT II 4375504 4386343 WBGene00015115 B0286.1 B0286.1 0.292539118229319 AGIWUR29486
CB4856 CT II 4392800 4404850 WBGene00022743 mlt-7 ZK430.8 -0.0793018184831844 AGIWUR39940
CB4856 CT II 4405864 4416553 WBGene00022741 ZK430.5 ZK430.5 0.0773740277959316 AGIWUR214
CB4856 CT II 4417683 4419131 WBGene00007036 sod-5 ZK430.3 -0.0486076299874956 AGIWUR15490
CB4856 CT II 4420255 4422319 WBGene00044063 bpnt-1 ZK430.2 -0.242220803303738 AGIWUR8553
CB4856 CT II 4422463 4424902 WBGene00022742 ZK430.7 ZK430.7 -0.00905125146632635 AGIWUR22253
CB4856 CT II 4425167 4432613 WBGene00022739 toe-1 ZK430.1 -0.239312790216443 AGIWUR7011
CB4856 CT II 4433332 4435169 WBGene00003699 nhr-109 T12C9.5 0.0666946411700717
AGIWUR376;AGIWUR15168;AGIWUR17704;AGIWUR19399;AGIWUR35164
CB4856 CT II 4435300 4436995 WBGene00020460 nhr-273 T12C9.1 -0.169507095671909 AGIWUR25363
CB4856 CT II 4438867 4440867 WBGene00003615 nhr-16 T12C9.6 -0.195371047331759 AGIWUR908
CB4856 CT II 4441265 4453151 WBGene00006657 twk-2 T12C9.3 -0.139000177377725
AGIWUR27308;AGIWUR39529
CB4856 CT II 4458263 4460404 WBGene00020461 T12C9.7 T12C9.7 -0.286633165890612
AGIWUR2066;AGIWUR5575;AGIWUR24733
CB4856 CT II 4461130 4467895 WBGene00021058 vps-18 W06B4.3 -0.339267009702759 AGIWUR2565
CB4856 CT II 4468169 4471038 WBGene00021057 W06B4.2 W06B4.2 -0.0362049410375676 AGIWUR716
CB4856 CT II 4471401 4473553 WBGene00021056 W06B4.1 W06B4.1 -0.327893824324331 AGIWUR16691
CB4856 CT II 4478613 4480948 WBGene00020248 T05C1.1 T05C1.1 -0.0801770771258052 AGIWUR5570
CB4856 CT II 4481025 4483722 WBGene00020249 T05C1.2 T05C1.2 -0.371721389735598 AGIWUR6661
CB4856 CT II 4487694 4489388 WBGene00020250 T05C1.3 T05C1.3 0.413854651471993 AGIWUR7186
CB4856 CT II 4490408 4490865 WBGene00020252 T05C1.5 T05C1.5 -0.250263178117222 AGIWUR15261
CB4856 CT II 4491152 4505147 WBGene00020251 camt-1 T05C1.4 0.0206005819876918
AGIWUR5533;AGIWUR37397;AGIWUR42799
CB4856 CT II 4512211 4516115 WBGene00015135 cyp-23A1 B0304.3 0.300288957327224

AGIWUR14628
CB4856 CT II 4518560 4522523 WBGene00001948 hlh-1 B0304.1 0.481706162329973
AGIWUR17528;AGIWUR23856
CB4856 CT II 4522465 4523222 WBGene00015136 B0304.4 B0304.4 -0.104880107149405
AGIWUR1375;AGIWUR6577;AGIWUR19962;AGIWUR37814
CB4856 CT II 4523622 4524589 WBGene00015134 B0304.2 B0304.2 -0.241357882865916 AGIWUR28156
CB4856 CT II 4524634 4527900 WBGene00005061 sra-35 B0304.8 -0.222803836326669
AGIWUR7786;AGIWUR36780;AGIWUR38769
CB4856 CT II 4529636 4531964 WBGene00005058 sra-32 B0304.5 -0.34256734698141 AGIWUR14583
CB4856 CT II 4534742 4537241 WBGene00005059 sra-33 B0304.6 0.248501840694893 AGIWUR42367
CB4856 CT II 4538988 4540731 WBGene00005062 sra-36 B0304.9 -0.0457827398938218 AGIWUR37011
CB4856 CT II 4541865 4543668 WBGene00005060 sra-34 B0304.7 0.285655718137043 AGIWUR11853
CB4856 CT II 4550894 4552177 WBGene00019489 K07E1.1 K07E1.1 0.0558658846215661
AGIWUR7894;AGIWUR11475;AGIWUR11642;AGIWUR17958;AGIWUR35065;AGIWUR35352;AGIWUR38739
CB4856 CT II 4557787 4559573 WBGene00019746 M03A1.3 M03A1.3 -0.331758878090975
AGIWUR16157;AGIWUR20492
CB4856 CT II 4560397 4562618 WBGene00077490 M03A1.8 M03A1.8 0.269608588708018 AGIWUR33318
CB4856 CT II 4562853 4563546 WBGene00000928 dao-2 M03A1.7 0.681587069252558 AGIWUR19408
CB4856 CT II 4572000 4590970 WBGene00006868 vab-1 M03A1.1 0.108686086063275
AGIWUR14824;AGIWUR44039
CB4856 CT II 4590952 4601354 WBGene00019747 ipla-1 M03A1.6 -0.00855150545083649
AGIWUR4878;AGIWUR35821
CB4856 CT II 4601355 4602633 WBGene00018046 F35D11.5 F35D11.5 -0.171525630366638
AGIWUR22207
CB4856 CT II 4603406 4604161 WBGene00018045 F35D11.4 F35D11.4
-0.000209959489108513 AGIWUR14130
CB4856 CT II 4608372 4612082 WBGene00018044 F35D11.3 F35D11.3 0.159083086017057
AGIWUR43345
CB4856 CT II 4612989 4626107 WBGene00004122 pqn-35 F35D11.2 0.101213731162728
AGIWUR28360
CB4856 CT II 4626906 4627805 WBGene00018043 F35D11.1 F35D11.1 -0.426262980265643
AGIWUR1128
CB4856 CT II 4627811 4638384 WBGene00018051 che-10 F35D11.11 -0.242810436424885
AGIWUR23841;AGIWUR35432
CB4856 CT II 4649085 4653280 WBGene00022283 lgc-27 Y74E4A.1 0.413730629174222
AGIWUR12849;AGIWUR33651
CB4856 CT II 4653189 4655934 WBGene00020222 fut-6 T05A7.5 -0.136355065843957
AGIWUR17270;AGIWUR39398
CB4856 CT II 4665893 4667588 WBGene00043986 fut-5 T05A7.10 -0.184894375974385

AGIWUR9107;AGIWUR32803
CB4856	CT	II	4668644	4671774	WBGene00020223	T05A7.6	T05A7.6	0.0584017875557983	AGIWUR15019
CB4856	CT	II	4672798	4673746	WBGene00001976	hmg-11	T05A7.4	0.705951112579653
AGIWUR31120;AGIWUR38858
CB4856	CT	II	4674420	4675729	WBGene00020220	clec-140	T05A7.2	0.45269560363095
AGIWUR14366
CB4856	CT	II	4675779	4676250	WBGene00020221	T05A7.3	T05A7.3	1.28859418797845	AGIWUR18858
CB4856	CT	II	4678026	4679638	WBGene00020219	T05A7.1	T05A7.1	-0.0196197341183613	AGIWUR40718
CB4856	CT	II	4679667	4681324	WBGene00020224	T05A7.7	T05A7.7	0.0330100972740563	AGIWUR18383
CB4856	CT	II	4681764	4683728	WBGene00020225	sre-40	T05A7.8	0.137964846032048	AGIWUR18657
CB4856	CT	II	4687241	4689392	WBGene00017371	sre-39	F10G7.7	-0.295248024049968
AGIWUR4968;AGIWUR29347
CB4856	CT	II	4689777	4690484	WBGene00044744	F10G7.12	F10G7.12	-0.0685290176729598
AGIWUR12475
CB4856	CT	II	4691227	4693075	WBGene00017370	F10G7.6	F10G7.6	0.333483476776712	AGIWUR6909
CB4856	CT	II	4693414	4696408	WBGene00017369	F10G7.5	F10G7.5	-0.0119087428884637	AGIWUR25609
CB4856	CT	II	4696419	4698427	WBGene00004461	rpn-5	F10G7.8	-0.290026164031568	AGIWUR22904
CB4856	CT	II	4698721	4700994	WBGene00004737	scc-1	F10G7.4	-0.106446105698033	AGIWUR4631
CB4856	CT	II	4702069	4703219	WBGene00006817	unc-85	F10G7.3	-0.139314558836315	AGIWUR32576
CB4856	CT	II	4703254	4706498	WBGene00017372	F10G7.9	F10G7.9	-0.0585800652567627	AGIWUR38407
CB4856	CT	II	4706821	4710052	WBGene00006626	tsn-1	F10G7.2	-0.193312699739112	AGIWUR860
CB4856	CT	II	4710277	4713690	WBGene00006497	tag-151	F10G7.1	-0.0302226531168195
AGIWUR3622;AGIWUR3894;AGIWUR14759;AGIWUR19454;AGIWUR25721;AGIWUR34601;AGIWUR41035
CB4856	CT	II	4713674	4726264	WBGene00017373	F10G7.10	F10G7.10	0.0817994249823921
AGIWUR1239;AGIWUR33197
CB4856	CT	II	4727326	4727869	WBGene00017374	ttr-41	F10G7.11	-0.294490667390146
AGIWUR30715
CB4856	CT	II	4731178	4733949	WBGene00018275	F41C3.11	F41C3.11	-0.126270398304168
AGIWUR43633
CB4856	CT	II	4735113	4736850	WBGene00018272	F41C3.6	F41C3.6	0.0829825018110668	AGIWUR15758
CB4856	CT	II	4737617	4739906	WBGene00018271	F41C3.5	F41C3.5	-0.149758111516162	AGIWUR38501
CB4856	CT	II	4740412	4741616	WBGene00018270	F41C3.4	F41C3.4	-0.181824641307873	AGIWUR32312
CB4856	CT	II	4742128	4745280	WBGene00018269	acs-11	F41C3.3	0.978530353359609	AGIWUR34073
CB4856	CT	II	4749968	4751822	WBGene00018268	F41C3.2	F41C3.2	0.447999934812865
AGIWUR23211;AGIWUR35292;AGIWUR38772
CB4856	CT	II	4752388	4752942	WBGene00018267	F41C3.1	F41C3.1	1.91813690659794	AGIWUR4862
CB4856	CT	II	4753413	4755595	WBGene00016163	C27D9.1	C27D9.1	-0.00873803200499523	AGIWUR8054
CB4856	CT	II	4755589	4756851	WBGene00016164	C27D9.2	C27D9.2	0.0365936171811909
AGIWUR26144;AGIWUR26845

CB4856	CT	II	4758762	4759289	WBGene00017839	F26G1.3	F26G1.3	-0.940968146387144	AGIWUR40801
CB4856	CT	II	4763081	4764174	WBGene00017838	F26G1.2	F26G1.2	0.824845050152478	AGIWUR7212;AGIWUR31440;AGIWUR34452
CB4856	CT	II	4764159	4766384	WBGene00017840	ttm-2	F26G1.4	1.29809911768535	AGIWUR36669
CB4856	CT	II	4770534	4771382	WBGene00017841	F26G1.5	F26G1.5	-0.530497587722628	AGIWUR2996
CB4856	CT	II	4772835	4773991	WBGene00044662	F26G1.9	F26G1.9	0.100688064088216	AGIWUR6370;AGIWUR16681;AGIWUR26642;AGIWUR37047;AGIWUR37704;AGIWUR41281
CB4856	CT	II	4774382	4774883	WBGene00044663	F26G1.10	F26G1.10	0.170975232678201	AGIWUR6035;AGIWUR18054;AGIWUR22730;AGIWUR24550
CB4856	CT	II	4775295	4781347	WBGene00017842	nep-12	F26G1.6	-0.0412221050726291	AGIWUR16205
CB4856	CT	II	4785861	4786316	WBGene00003424	msp-3	F26G1.7	0.202633518437954	AGIWUR39844
CB4856	CT	II	4791620	4795643	WBGene00017837	F26G1.1	F26G1.1	0.118638958288513	AGIWUR6838;AGIWUR8224;AGIWUR11071;AGIWUR12821;AGIWUR17678;AGIWUR38146
CB4856	CT	II	4795761	4797216	WBGene00016353	C33F10.4	C33F10.4	-0.17148363053814	AGIWUR2568;AGIWUR20383;AGIWUR20844;AGIWUR21310
CB4856	CT	II	4797371	4798176	WBGene00050941	C33F10.14	C33F10.14	-0.077008497264309	AGIWUR4432
CB4856	CT	II	4798154	4812136	WBGene00016354	rig-6	C33F10.5	-0.0276192455290696	AGIWUR8328;AGIWUR10082;AGIWUR34741;AGIWUR37698;AGIWUR37741;AGIWUR40358
CB4856	CT	II	4818662	4821564	WBGene00016355	lact-5	C33F10.7	0.0862855931731428	AGIWUR440;AGIWUR17904;AGIWUR32830
CB4856	CT	II	4823381	4825196	WBGene00016356	C33F10.8	C33F10.8	-0.183399998411094	AGIWUR3888
CB4856	CT	II	4826507	4827884	WBGene00016359	fbxb-88	C33F10.13	0.907154301443596	AGIWUR8862
CB4856	CT	II	4828560	4832469	WBGene00016352	tbck-1	C33F10.2	0.181566270545748	AGIWUR11843
CB4856	CT	II	4836304	4837759	WBGene00016358	C33F10.12	C33F10.12	-0.162373241252944	AGIWUR20730
CB4856	CT	II	4850185	4851441	WBGene00044767	T23B7.3	T23B7.3	-0.177223674987626	AGIWUR18343;AGIWUR37964;AGIWUR43618
CB4856	CT	II	4852056	4857880	WBGene00001027	dnj-9	F11G11.7	-0.334361421400089	AGIWUR1344;AGIWUR27861
CB4856	CT	II	4857923	4859185	WBGene00017385	F11G11.5	F11G11.5	0.176624730413733	AGIWUR30732
CB4856	CT	II	4860064	4860411	WBGene00017386	nspd-5	F11G11.8	0.237830243873617	AGIWUR6006
CB4856	CT	II	4860594	4861696	WBGene00017384	F11G11.4	F11G11.4	0.39155359207621	AGIWUR9900

CB4856	CT	II	4862318	4863457	WBGene00017387	mpst-4	F11G11.9	-0.721354834673969	AGIWUR35205
CB4856	CT	II	4866436	4867576	WBGene00000606	col-17	F11G11.10	-0.502846201942985	AGIWUR20968
CB4856	CT	II	4872098	4873127	WBGene00000649	col-73	F11G11.12	-0.196798764231513	AGIWUR5980
CB4856	CT	II	4875280	4875666	WBGene00017388	F11G11.13	F11G11.13	0.0143193668429951	AGIWUR13442;AGIWUR18299;AGIWUR24022;AGIWUR39742;AGIWUR43786;AGIWUR44698
CB4856	CT	II	4879885	4881105	WBGene00001754	gst-6	F11G11.3	-0.181852121037847	AGIWUR24152
CB4856	CT	II	4882062	4883358	WBGene00001755	gst-7	F11G11.2	-0.0675475971697992	AGIWUR29841
CB4856	CT	II	4883749	4884602	WBGene00001756	gst-8	F11G11.1	-0.0291553423697408	AGIWUR30738
CB4856	CT	II	4886276	4888211	WBGene00019891	R05F9.7	R05F9.7	-0.00539996014583455	AGIWUR42366
CB4856	CT	II	4889437	4891427	WBGene00019890	R05F9.6	R05F9.6	-0.289326677686137	AGIWUR15697;AGIWUR19070;AGIWUR25990;AGIWUR32241
CB4856	CT	II	4891573	4893573	WBGene00001757	gst-9	R05F9.5	-0.841504334613205	AGIWUR26662
CB4856	CT	II	4899859	4902100	WBGene00019892	R05F9.9	R05F9.9	-0.244341061622917	AGIWUR32806;AGIWUR38871
CB4856	CT	II	4902288	4903796	WBGene00019893	sgt-1	R05F9.10	-0.103269045312892	AGIWUR8466
CB4856	CT	II	4905008	4911835	WBGene00019888	btbd-10	R05F9.1	-0.0148436903895428	AGIWUR2401;AGIWUR8282;AGIWUR8547;AGIWUR11478;AGIWUR12754;AGIWUR16406;AGIWUR28362;AGIWUR33870;AGIWUR35150;AGIWUR36112;AGIWUR38514;AGIWUR44911
CB4856	CT	II	4911765	4914218	WBGene00019894	R05F9.11	R05F9.11	0.280895383293018	AGIWUR11820
CB4856	CT	II	4914494	4925248	WBGene00019895	aagr-2	R05F9.12	0.105532387538432	AGIWUR6585;AGIWUR22779;AGIWUR27231
CB4856	CT	II	4930873	4932114	WBGene00022763	ZK546.7	ZK546.7	-0.214513877162707	AGIWUR33470
CB4856	CT	II	4932991	4933450	WBGene00003470	msp-152	ZK546.6	0.20007397815874	AGIWUR34259
CB4856	CT	II	4933883	4935038	WBGene00001778	gst-30	ZK546.11	-0.125921354405669	AGIWUR12388
CB4856	CT	II	4935563	4937956	WBGene00022762	ZK546.5	ZK546.5	-0.0520883500015064	AGIWUR12473
CB4856	CT	II	4938004	4938602	WBGene00022761	ZK546.4	ZK546.4	0.0652750178244368	AGIWUR18441
CB4856	CT	II	4938599	4941150	WBGene00022766	cblc-1	ZK546.17	0.114087951916484	AGIWUR34186;AGIWUR38869
CB4856	CT	II	4944042	4945838	WBGene00022765	ZK546.14	ZK546.14	-0.19103276485385	AGIWUR6967;AGIWUR30274

CB4856	CT	II	4946025 4948070 WBGene00022759	ZK546.2 ZK546.2 -0.232783983366719	AGIWUR28619
CB4856	CT	II	4948154 4949527 WBGene00006619	try-1	ZK546.15	-0.306798039210271
AGIWUR1091
CB4856	CT	II	4949902 4953488 WBGene00006997	zyg-12	ZK546.1 -0.0209635994709725
AGIWUR32075;AGIWUR42593
CB4856	CT	II	4953589 4954503 WBGene00003580	ndx-3	Y38A8.1 0.0994472617856399	AGIWUR15903
CB4856	CT	II	4954480 4955427 WBGene00003949	pbs-3	Y38A8.2 -0.191303833585142
AGIWUR4506;AGIWUR7923
CB4856	CT	II	4955646 4959876 WBGene00006737	ulp-2	Y38A8.3 0.254810559949977	AGIWUR9197
CB4856	CT	II	4969186 4988934 WBGene00020867	shc-2	T27F7.2 0.248861170590092
AGIWUR4201;AGIWUR16312;AGIWUR17522;AGIWUR25678
CB4856	CT	II	4990831 4993580 WBGene00020868	eif-1	T27F7.3 -0.0288605714775976
AGIWUR8012;AGIWUR8543;AGIWUR26232;AGIWUR26466
CB4856	CT	II	4994002 4995607 WBGene00020866	vps-24	T27F7.1 -0.0133232610825278
AGIWUR197;AGIWUR18963;AGIWUR28840
CB4856	CT	II	4995551 4998035 WBGene00004382	rnh-1.0 F59A6.6 0.274312715815472
AGIWUR10797;AGIWUR42154
CB4856	CT	II	4998007 4998757 WBGene00044664	F59A6.11	F59A6.11	-0.274842611357338
AGIWUR3207;AGIWUR22750;AGIWUR28950;AGIWUR37911;AGIWUR43828
CB4856	CT	II	4999520 5003806 WBGene00019087	F59A6.5 F59A6.5 0.0442742660858713	AGIWUR27627
CB4856	CT	II	5003960 5004849 WBGene00044385	F59A6.10	F59A6.10	0.0264239657681768
AGIWUR1690;AGIWUR3674;AGIWUR12293;AGIWUR17634;AGIWUR18276;AGIWUR24820;AGIWUR29279;AGIWUR29498;AGIWUR31744;A
GIWUR32928;AGIWUR42515
CB4856	CT	II	5005081 5008068 WBGene00019086	F59A6.4 F59A6.4 0.175840054740394	AGIWUR44174
CB4856	CT	II	5008341 5011179 WBGene00019085	F59A6.3 F59A6.3 -0.00943039686033043
AGIWUR14498;AGIWUR29885;AGIWUR44263
CB4856	CT	II	5011523 5012078 WBGene00044665	F59A6.12	F59A6.12	0.284997035025223
AGIWUR6143;AGIWUR21365
CB4856	CT	II	5014699 5015324 WBGene00019084	F59A6.2 F59A6.2 0.101325962412886	AGIWUR27186
CB4856	CT	II	5022764 5029982 WBGene00003822	nsy-1	F59A6.1 0.0952933999740804	AGIWUR7127
CB4856	CT	II	5029942 5031712 WBGene00018014	F33G12.2	F33G12.2	-0.25963538987006
AGIWUR1431;AGIWUR6223;AGIWUR12852;AGIWUR14295;AGIWUR14922;AGIWUR15737;AGIWUR24470;AGIWUR34893;AGIWUR41448;A
GIWUR44513
CB4856	CT	II	5031757 5034035 WBGene00018015	F33G12.3	F33G12.3	-0.313451399568059
AGIWUR559;AGIWUR909;AGIWUR6263;AGIWUR23996;AGIWUR37724
CB4856	CT	II	5034142 5036439 WBGene00018016	lrr-1	F33G12.4	0.00182640504433529
AGIWUR20211
CB4856	CT	II	5036561 5040196 WBGene00018017	golg-2	F33G12.5	-0.159080353962301
AGIWUR38302

CB4856	CT	II	5040208	5040681	WBGene00044666	F33G12.7	F33G12.7	-0.155216491575954	AGIWUR11782;AGIWUR22608
CB4856	CT	II	5049915	5053966	WBGene00018018	F33G12.6	F33G12.6	0.28739621165446	AGIWUR7871;AGIWUR12854;AGIWUR35039;AGIWUR35780
CB4856	CT	II	5054084	5055338	WBGene00002068	ify-1	C27A2.3	-0.200632936863085	AGIWUR37903
CB4856	CT	II	5055616	5056214	WBGene00004434	rpl-22	C27A2.2	0.0590952910562645	AGIWUR8338;AGIWUR19216;AGIWUR25761;AGIWUR35665;AGIWUR36144;AGIWUR39113;AGIWUR39990;AGIWUR40550
CB4856	CT	II	5056211	5058113	WBGene00206373	C27A2.12	C27A2.12	-0.215999377064701	AGIWUR25367
CB4856	CT	II	5062129	5067116	WBGene00016151	smc-5	C27A2.1	-0.0757465280141938	AGIWUR13886;AGIWUR34831;AGIWUR36227
CB4856	CT	II	5067088	5067980	WBGene00044387	C27A2.8	C27A2.8	0.612325936774715	AGIWUR9687;AGIWUR23394;AGIWUR34194;AGIWUR44428
CB4856	CT	II	5068648	5069625	WBGene00016153	C27A2.5	C27A2.5	-0.124946372089235	AGIWUR20299
CB4856	CT	II	5069764	5071365	WBGene00044386	C27A2.7	C27A2.7	-0.0624484261534137	AGIWUR320;AGIWUR7549;AGIWUR32727
CB4856	CT	II	5078235	5081161	WBGene00001102	dsh-2	C27A2.6	-0.142499897387845	AGIWUR6526
CB4856	CT	II	5081490	5083441	WBGene00015454	C04G6.4	C04G6.4	0.0604443657494702	AGIWUR13458
CB4856	CT	II	5083848	5091690	WBGene00004040	pld-1	C04G6.3	0.0867303901705458	AGIWUR8787;AGIWUR15467
CB4856	CT	II	5093214	5094263	WBGene00015453	C04G6.2	C04G6.2	-0.743562487393706	AGIWUR3836;AGIWUR20839
CB4856	CT	II	5094245	5095370	WBGene00077489	C04G6.13	C04G6.13	-0.172747922696745	AGIWUR41830
CB4856	CT	II	5098770	5100242	WBGene00015456	C04G6.6	C04G6.6	-0.416704106086792	AGIWUR35921
CB4856	CT	II	5100412	5101801	WBGene00015457	C04G6.7	C04G6.7	0.0956333211621949	AGIWUR1649
CB4856	CT	II	5103341	5103834	WBGene00015459	C04G6.11	C04G6.11	0.325166157183588	AGIWUR4781
CB4856	CT	II	5104118	5108901	WBGene00003402	mpk-2	C04G6.1	0.291238732950926	AGIWUR2806;AGIWUR28220
CB4856	CT	II	5110029	5112232	WBGene00017277	F09C12.2	F09C12.2	1.35233020299139	AGIWUR15962
CB4856	CT	II	5112382	5114153	WBGene00017279	F09C12.8	F09C12.8	0.328885630613879	AGIWUR10339
CB4856	CT	II	5116033	5118276	WBGene00017278	F09C12.6	F09C12.6	-0.539150524244826	AGIWUR25931
CB4856	CT	II	5130384	5133215	WBGene00001588	ggr-3	F09C12.1	-0.153459680228117	AGIWUR28057
CB4856	CT	II	5136717	5138568	WBGene00019023	F58A6.2	F58A6.2	-0.0795314062068373	

									AGIWUR11752;AGIWUR14733;AGIWUR23481;AGIWUR27769;AGIWUR37991
CB4856	CT	II	5138648	5142036	WBGene00003547	nas-29	F58A6.4	-0.110117994630066	AGIWUR2730;AGIWUR7599;AGIWUR17429;AGIWUR25274;AGIWUR27316;AGIWUR34128
CB4856	CT	II	5142343	5144021	WBGene00019022	F58A6.1	F58A6.1	0.227146964384762	AGIWUR19021
CB4856	CT	II	5146967	5148465	WBGene00019024	F58A6.5	F58A6.5	-0.0147261344993872	AGIWUR18647;AGIWUR28907;AGIWUR29036
CB4856	CT	II	5148530	5150853	WBGene00019025	srb-16	F58A6.6	0.110675957246714	AGIWUR31347
CB4856	CT	II	5153119	5154638	WBGene00019027	srb-12	F58A6.10	0.130432363262938	AGIWUR35711
CB4856	CT	II	5155436	5157625	WBGene00019028	srb-13	F58A6.11	-0.0362208499441685	AGIWUR31294
CB4856	CT	II	5158566	5158988	WBGene00019026	F58A6.9	F58A6.9	0.209072897890755	AGIWUR42574
CB4856	CT	II	5159717	5161130	WBGene00044388	C27D6.11	C27D6.11	-0.107152394569399	AGIWUR40723
CB4856	CT	II	5161995	5163383	WBGene00005070	srb-5	C27D6.6	-0.186663838066308	AGIWUR40918
CB4856	CT	II	5163868	5165020	WBGene00005069	srb-4	C27D6.7	-0.449042755207639	AGIWUR34426
CB4856	CT	II	5165315	5166838	WBGene00005068	srb-3	C27D6.8	0.046879792363594	AGIWUR21541
CB4856	CT	II	5168110	5169324	WBGene00005067	srb-2	C27D6.9	0.274609173034396	AGIWUR22739
CB4856	CT	II	5169726	5171032	WBGene00005066	srb-1	C27D6.10	0.103629824110303	AGIWUR9291
CB4856	CT	II	5171910	5177440	WBGene00016160	C27D6.1	C27D6.1	0.042036991217465	AGIWUR9081
CB4856	CT	II	5179034	5187569	WBGene00016162	crh-2	C27D6.4	0.0573126523312055	AGIWUR6531;AGIWUR9803;AGIWUR31953
CB4856	CT	II	5195804	5199257	WBGene00016416	C34F11.5	C34F11.5	0.550965180304669	AGIWUR10404
CB4856	CT	II	5199763	5200223	WBGene00003443	msp-50	C34F11.4	0.355680376504711	AGIWUR44471
CB4856	CT	II	5203322	5209799	WBGene00016415	ampd-1	C34F11.3	-0.0373953185787617	AGIWUR3205;AGIWUR3377;AGIWUR8618;AGIWUR26632;AGIWUR28743;AGIWUR29238;AGIWUR30148
CB4856	CT	II	5209780	5210978	WBGene00016417	C34F11.8	C34F11.8	0.342657446205521	AGIWUR22884
CB4856	CT	II	5212270	5212789	WBGene00016414	C34F11.2	C34F11.2	0.408435886386066	AGIWUR12844
CB4856	CT	II	5212932	5213932	WBGene00016413	C34F11.1	C34F11.1	-0.129574464922538	AGIWUR34064
CB4856	CT	II	5214441	5225377	WBGene00001101	dsh-1	C34F11.9	0.335851563107213	AGIWUR2992;AGIWUR5381;AGIWUR18371;AGIWUR19496;AGIWUR37180;AGIWUR42341;AGIWUR43485;AGIWUR43880
CB4856	CT	II	5230081	5230501	WBGene00017148	EGAP2.2	EGAP2.2	0.149776743372737	AGIWUR38655
CB4856	CT	II	5231424	5233273	WBGene00017147	EGAP2.1	EGAP2.1	0.00237296945462973	AGIWUR19126

CB4856	CT	II	5233154	5235926	WBGene00004020	pho-1	EGAP2.3	-0.649372078425198	AGIWUR29936
CB4856	CT	II	5236258	5238514	WBGene00004023	pho-4	T16D1.2	0.56245544292058	AGIWUR3712;AGIWUR12698;AGIWUR37571;AGIWUR39791
CB4856	CT	II	5241233	5241660	WBGene00020539	T16D1.1	T16D1.1	0.22476404064137	AGIWUR41030
CB4856	CT	II	5242452	5246837	WBGene00004221	ptr-6	C54A12.1	0.0752868107860634	AGIWUR4575;AGIWUR29782;AGIWUR33750;AGIWUR35269;AGIWUR36517;AGIWUR42135
CB4856	CT	II	5251121	5255529	WBGene00016909	frpr-4	C54A12.2	0.00392840172357012	AGIWUR34619
CB4856	CT	II	5270406	5271845	WBGene00016911	drn-1	C54A12.4	0.0185769696294775	AGIWUR19420
CB4856	CT	II	5278137	5282525	WBGene00022781	pmt-1	ZK622.3	0.894029495677628	AGIWUR3942;AGIWUR11991;AGIWUR15724
CB4856	CT	II	5285329	5288908	WBGene00001806	gur-5	ZK622.2	0.0869523890108486	AGIWUR8811
CB4856	CT	II	5289442	5290326	WBGene00044667	ZK622.5	ZK622.5	-0.177701478502534	AGIWUR12678;AGIWUR42990
CB4856	CT	II	5292683	5293306	WBGene00022782	ZK622.4	ZK622.4	0.0911381142695808	AGIWUR12395;AGIWUR36745;AGIWUR38572;AGIWUR41852
CB4856	CT	II	5293800	5295612	WBGene00022780	ZK622.1	ZK622.1	0.216772328835809	AGIWUR44091
CB4856	CT	II	5312046	5319234	WBGene00001538	gcy-12	F08B1.2	-0.12373545602992	AGIWUR12960;AGIWUR42287
CB4856	CT	II	5323005	5340826	WBGene00006923	vhp-1	F08B1.1	0.171718327926342	AGIWUR5649;AGIWUR6552;AGIWUR7813;AGIWUR10624;AGIWUR11567;AGIWUR13821;AGIWUR15922;AGIWUR18468;AGIWUR20242;AGIWUR21499;AGIWUR26859;AGIWUR29335;AGIWUR29995;AGIWUR38653;AGIWUR41812;AGIWUR42052;AGIWUR44227
CB4856	CT	II	5342663	5344875	WBGene00017291	F09E5.14	F09E5.14	0.395395225832306	AGIWUR32159
CB4856	CT	II	5350669	5351993	WBGene00017286	F09E5.8	F09E5.8	-0.135241932277165	AGIWUR5675
CB4856	CT	II	5352087	5353759	WBGene00017285	F09E5.7	F09E5.7	-0.169999016993989	AGIWUR39499
CB4856	CT	II	5355712	5357341	WBGene00005226	srg-69	F09E5.4	0.262155195324008	AGIWUR24261
CB4856	CT	II	5357308	5358187	WBGene00017289	F09E5.11	F09E5.11	-0.1605550698686	AGIWUR3030
CB4856	CT	II	5358695	5359469	WBGene00017293	bmy-1	F09E5.17	-0.138541943279549	AGIWUR7837
CB4856	CT	II	5359552	5372122	WBGene00017284	sec-6	F09E5.5	0.0190225572849734	AGIWUR1999
CB4856	CT	II	5371980	5373433	WBGene00000094	agt-2	F09E5.13	-0.381825437873715	AGIWUR5697
CB4856	CT	II	5373997	5375177	WBGene00017283	F09E5.3	F09E5.3	-0.28492831739287	AGIWUR233
CB4856	CT	II	5375267	5377111	WBGene00017282	algn-2	F09E5.2	-0.0642207199056539	AGIWUR12610;AGIWUR34310
CB4856	CT	II	5378068	5379632	WBGene00006434	prdx-2	F09E5.15	-0.0509470122650448	

AGIWUR2121
CB4856 CT II 5380027 5384169 WBGene00004034 pkc-3 F09E5.1 0.115360910311361 AGIWUR11029
CB4856 CT II 5384137 5387317 WBGene00017136 ccpp-6 EEED8.6 0.16416572223282 AGIWUR7326
CB4856 CT II 5387411 5388830 WBGene00004701 rsp-4 EEED8.7 0.037368670019422
AGIWUR14445;AGIWUR27407;AGIWUR28507
CB4856 CT II 5388829 5390104 WBGene00003583 ndx-6 EEED8.8 0.0768913778556275
AGIWUR13822;AGIWUR15361;AGIWUR34198;AGIWUR39738
CB4856 CT II 5390233 5393190 WBGene00017144 brap-2 EEED8.16 -0.0643939226282484
AGIWUR33548
CB4856 CT II 5393293 5395694 WBGene00017137 pink-1 EEED8.9 -0.172991685129864 AGIWUR43636
CB4856 CT II 5395889 5398902 WBGene00017138 EEED8.10 EEED8.10 -0.114697451613418
AGIWUR13646;AGIWUR29548;AGIWUR32140
CB4856 CT II 5399538 5405810 WBGene00003393 mog-5 EEED8.5 -0.128322521241364 AGIWUR17482
CB4856 CT II 5405819 5407634 WBGene00017139 clec-141 EEED8.11 -0.570782464595225
AGIWUR19066
CB4856 CT II 5408260 5409024 WBGene00017140 EEED8.12 EEED8.12 0.448608036265835
AGIWUR11005
CB4856 CT II 5410467 5411235 WBGene00017135 EEED8.4 EEED8.4 -0.0434094311103568 AGIWUR16324
CB4856 CT II 5411542 5412174 WBGene00017141 EEED8.13 EEED8.13 -0.226855950104097
AGIWUR306
CB4856 CT II 5412196 5412610 WBGene00194696 EEED8.18 EEED8.18 -0.19325777231684
AGIWUR43040
CB4856 CT II 5414207 5415580 WBGene00017134 EEED8.3 EEED8.3 -0.198253371147683 AGIWUR17225
CB4856 CT II 5416714 5418377 WBGene00017133 EEED8.2 EEED8.2 1.1254086193987 AGIWUR40222
CB4856 CT II 5419233 5421093 WBGene00017143 EEED8.15 EEED8.15 0.303981412818679
AGIWUR2513
CB4856 CT II 5421248 5422737 WBGene00017132 tofu-6 EEED8.1 -0.248159784011058 AGIWUR23738
CB4856 CT II 5422735 5423858 WBGene00017219 F07F6.7 F07F6.7 -0.17160943146984 AGIWUR20957
CB4856 CT II 5424141 5425733 WBGene00017220 F07F6.8 F07F6.8 0.0628931986151713 AGIWUR16885
CB4856 CT II 5425750 5429572 WBGene00017217 F07F6.4 F07F6.4 -0.138993164951167
AGIWUR4465;AGIWUR5507;AGIWUR9414;AGIWUR22035;AGIWUR28235;AGIWUR34889
CB4856 CT II 5430392 5431422 WBGene00017216 F07F6.2 F07F6.2 -0.137914963307854
AGIWUR3809;AGIWUR9682;AGIWUR16521;AGIWUR28895;AGIWUR36864;AGIWUR42556
CB4856 CT II 5431476 5433048 WBGene00006005 srx-114 F07F6.9 -0.29016623468029 AGIWUR27966
CB4856 CT II 5435724 5436933 WBGene00017218 dct-5 F07F6.5 -0.246876372192885
AGIWUR15543;AGIWUR21500;AGIWUR27471
CB4856 CT II 5437891 5440256 WBGene00017215 F07F6.1 F07F6.1 0.166844373136088 AGIWUR12415
CB4856 CT II 5440243 5446705 WBGene00003774 nmr-1 F07F6.6 0.126597045609448 AGIWUR35436
CB4856 CT II 5447681 5448634 WBGene00023407 cex-1 F56D1.6 0.102239232525019 AGIWUR41097

CB4856	CT	II	5453847	5455977	WBGene00000969	dhs-5	F56D1.5	-0.0939083096496311	AGIWUR3066
CB4856	CT	II	5456305	5458609	WBGene00000935	daz-1	F56D1.7	-0.100373030120801	AGIWUR29003
CB4856	CT	II	5466363	5473812	WBGene00000548	clr-1	F56D1.4	0.0945593132880518	AGIWUR15780;AGIWUR32701;AGIWUR40029
CB4856	CT	II	5474373	5475475	WBGene00018961	mrps-16	F56D1.3	-0.135403812597931	AGIWUR20329;AGIWUR22842;AGIWUR30295
CB4856	CT	II	5475409	5478524	WBGene00018960	ilcr-2	F56D1.2	-0.414651414579522	AGIWUR3916
CB4856	CT	II	5478578	5480490	WBGene00018959	F56D1.1	F56D1.1	-0.344341245993649	AGIWUR12541;AGIWUR32062;AGIWUR39277
CB4856	CT	II	5480591	5482041	WBGene00017394	cal-8	F12A10.5	-0.185093695346366	AGIWUR35593
CB4856	CT	II	5488151	5490606	WBGene00017393	nep-5	F12A10.4	-0.416209063117526	AGIWUR35809
CB4856	CT	II	5491111	5493192	WBGene00017392	basl-2	F12A10.3	-0.0257776831730965	AGIWUR23641
CB4856	CT	II	5494085	5494505	WBGene00017395	F12A10.6	F12A10.6	-1.26633580665519	AGIWUR41057
CB4856	CT	II	5497285	5498256	WBGene00017396	F12A10.7	F12A10.7	-0.491678096305119	AGIWUR25210
CB4856	CT	II	5498851	5501409	WBGene00017397	F12A10.8	F12A10.8	0.000541551817999419	AGIWUR33792
CB4856	CT	II	5501633	5503908	WBGene00001511	fzy-1	ZK177.6	-0.231338314736789	AGIWUR9934
CB4856	CT	II	5503966	5506115	WBGene00000375	cyp-44A1	ZK177.5	-0.0279808546835465	AGIWUR8962;AGIWUR13879;AGIWUR35034;AGIWUR38693;AGIWUR43217
CB4856	CT	II	5505870	5509056	WBGene00022672	ZK177.4	ZK177.4	0.0233414414614428	AGIWUR12615;AGIWUR36298
CB4856	CT	II	5509028	5513775	WBGene00022673	ZK177.8	ZK177.8	-0.26656796973749	AGIWUR15416;AGIWUR18492;AGIWUR22202;AGIWUR33128
CB4856	CT	II	5515861	5517133	WBGene00022671	ZK177.3	ZK177.3	-0.00528644312632842	AGIWUR21731
CB4856	CT	II	5517157	5517743	WBGene00022674	ZK177.9	ZK177.9	0.459433441032379	AGIWUR36184
CB4856	CT	II	5522937	5523488	WBGene00044390	ZK177.11	ZK177.11	0.092280118646352	AGIWUR11132;AGIWUR14057;AGIWUR25550;AGIWUR38479;AGIWUR44677
CB4856	CT	II	5524200	5525379	WBGene00006554	tbx-35	ZK177.10	0.0313502687790388	AGIWUR8139;AGIWUR44660
CB4856	CT	II	5528141	5529637	WBGene00022669	ZK177.1	ZK177.1	-0.417161714240283	AGIWUR3590
CB4856	CT	II	5530501	5530928	WBGene00002121	ins-38	C17C3.20	-0.0362572447190219	AGIWUR10095;AGIWUR17770;AGIWUR24592;AGIWUR26179;AGIWUR31150
CB4856	CT	II	5531313	5532259	WBGene00015892	C17C3.9	C17C3.9	-0.148978119929717	AGIWUR3551
CB4856	CT	II	5532857	5533738	WBGene00001965	hlh-26	C17C3.8	-0.0992945470953934	AGIWUR33494

CB4856 CT II 5537835 5539004 WBGene00015893 C17C3.11 C17C3.11 -0.173406131956584
AGIWUR2886
CB4856 CT II 5544585 5544851 WBGene00002096 ins-13 C17C3.18 -0.335971015716062
AGIWUR11025;AGIWUR15472
CB4856 CT II 5544781 5547492 WBGene00015894 acdh-2 C17C3.12 -0.630497510635818
AGIWUR10216;AGIWUR11158;AGIWUR11868;AGIWUR39769;AGIWUR44925
CB4856 CT II 5551833 5552335 WBGene00015890 C17C3.5 C17C3.5 -0.245903983598696 AGIWUR20466
CB4856 CT II 5554617 5555306 WBGene00002095 ins-12 C17C3.19 0.113398571607346
AGIWUR1112;AGIWUR11658;AGIWUR22944;AGIWUR26429;AGIWUR44694
CB4856 CT II 5556761 5557088 WBGene00002094 ins-11 C17C3.4 1.80603255441713 AGIWUR24660
CB4856 CT II 5561763 5563523 WBGene00015889 C17C3.3 C17C3.3 0.594985173322758 AGIWUR13473
CB4856 CT II 5570572 5574943 WBGene00015887 C17C3.1 C17C3.1 -0.292746342371787
AGIWUR655;AGIWUR1027;AGIWUR2276;AGIWUR3952;AGIWUR5419;AGIWUR9121;AGIWUR18932;AGIWUR33510;AGIWUR36819;AGIWUR37926;AGIWUR38475;AGIWUR42256
CB4856 CT II 5578318 5579965 WBGene00020803 T25E4.1 T25E4.1 -0.221703195697863
AGIWUR17629;AGIWUR39775
CB4856 CT II 5580125 5582409 WBGene00020804 T25E4.2 T25E4.2 -0.0976497715514739 AGIWUR34051
CB4856 CT II 5586165 5587651 WBGene00003097 lys-8 C17G10.5 0.00485265965128434
AGIWUR8095
CB4856 CT II 5587614 5589755 WBGene00270322 C17G10.13 C17G10.13 -0.293301965893264
AGIWUR40743
CB4856 CT II 5589186 5593628 WBGene00000383 cdc-14 C17G10.4 0.0546726217464446
AGIWUR4060;AGIWUR6178;AGIWUR9427;AGIWUR23768;AGIWUR27886;AGIWUR37473;AGIWUR44066
CB4856 CT II 5594714 5596463 WBGene00015916 C17G10.2 C17G10.2 -0.253405128968124
AGIWUR5214
CB4856 CT II 5596547 5598449 WBGene00015915 C17G10.1 C17G10.1 -0.122265897432685
AGIWUR10468;AGIWUR25020;AGIWUR29338
CB4856 CT II 5599930 5603294 WBGene00015918 C17G10.6 C17G10.6 0.0903655898832776
AGIWUR4653;AGIWUR5160;AGIWUR9532;AGIWUR11414;AGIWUR33077;AGIWUR39820
CB4856 CT II 5606542 5607856 WBGene00015919 C17G10.7 C17G10.7 0.139406773133127
AGIWUR34206
CB4856 CT II 5617122 5619319 WBGene00000970 dhs-6 C17G10.8 -0.000763500560227115
AGIWUR41943
CB4856 CT II 5619590 5622483 WBGene00015920 eif-3.L C17G10.9 -0.409400412013074
AGIWUR6407
CB4856 CT II 5626316 5627263 WBGene00235269 F59E12.15 F59E12.15 -0.400017225185093
AGIWUR35828
CB4856 CT II 5634528 5636926 WBGene00019123 F59E12.8 F59E12.8 -0.446988703566336
AGIWUR11995

CB4856 CT   II   5638381 5639752 WBGene00019119 F59E12.3   F59E12.3
-0.00210465665991943   AGIWUR18907;AGIWUR24208;AGIWUR40233;AGIWUR42077
CB4856 CT   II   5640063 5647042 WBGene00019124 F59E12.9   F59E12.9   -0.164375448199609
AGIWUR6420
CB4856 CT   II   5647154 5647884 WBGene00019125 ddl-1   F59E12.10   0.0866988173044163
AGIWUR42386
CB4856 CT   II   5647986 5649472 WBGene00019126 sam-4   F59E12.11   -0.0921725258232424
AGIWUR3369
CB4856 CT   II   5649737 5654422 WBGene00006988 zyg-1   F59E12.2   -0.370769162977048
AGIWUR21062
CB4856 CT   II   5654515 5657371 WBGene00019118 F59E12.1   F59E12.1   -0.0582667966190087
AGIWUR27965
CB4856 CT   II   5657710 5659854 WBGene00006402 fut-3   F59E12.13   -0.100770018580398
AGIWUR857
CB4856 CT   II   5659937 5660786 WBGene00016121 C25H3.12   C25H3.12   -0.0465153468255384
AGIWUR7009;AGIWUR11907;AGIWUR20481;AGIWUR34018
CB4856 CT   II   5661167 5665243 WBGene00016115 mdt-26 C25H3.6 -0.043581713765537
AGIWUR1167;AGIWUR3321;AGIWUR12078;AGIWUR15580;AGIWUR22399;AGIWUR26846;AGIWUR31427;AGIWUR38315;AGIWUR40073;A
GIWUR41078;AGIWUR41188;AGIWUR42279;AGIWUR44075
CB4856 CT   II   5665408 5666278 WBGene00044391 C25H3.15   C25H3.15   0.210205792752075
AGIWUR31701;AGIWUR41371
CB4856 CT   II   5666291 5666850 WBGene00044392 C25H3.16   C25H3.16   -0.239686777861999
AGIWUR1916;AGIWUR4138
CB4856 CT   II   5666756 5668930 WBGene00016116 C25H3.7 C25H3.7 0.139490844469247   AGIWUR35607
CB4856 CT   II   5669788 5679276 WBGene00016117 C25H3.8 C25H3.8 0.0722277713821784
AGIWUR9610;AGIWUR19861;AGIWUR23395;AGIWUR35785;AGIWUR44190;AGIWUR44837
CB4856 CT   II   5680980 5682667 WBGene00016114 flp-27 C25H3.5 0.397125102148228   AGIWUR40585
CB4856 CT   II   5684479 5689030 WBGene00016120 C25H3.11   C25H3.11   -0.286444261081717
AGIWUR29501
CB4856 CT   II   5690137 5691355 WBGene00016118 C25H3.9 C25H3.9 -0.0218407815317416
AGIWUR1206;AGIWUR40397
CB4856 CT   II   5691662 5693939 WBGene00016113 eif-2D C25H3.4 -0.54337102792554   AGIWUR25622
CB4856 CT   II   5694195 5695237 WBGene00016112 C25H3.3 C25H3.3 -0.202614187309182   AGIWUR18537
CB4856 CT   II   5695383 5696358 WBGene00016123 C25H3.14   C25H3.14   0.167005999926623
AGIWUR41054
CB4856 CT   II   5696585 5698969 WBGene00016119 C25H3.10   C25H3.10   1.87066038449854
AGIWUR16686
CB4856 CT   II   5700170 5701408 WBGene00016111 C25H3.1 C25H3.1 0.0667592797664689
AGIWUR34742;AGIWUR36546

CB4856	CT	II	5707691	5712351	WBGene00015942	osta-2	C18A3.4	0.0706226723387653	AGIWUR3856;AGIWUR8859;AGIWUR9550;AGIWUR16022;AGIWUR17152;AGIWUR18066;AGIWUR25677;AGIWUR26532
CB4856	CT	II	5712351	5714823	WBGene00015943	tiar-1	C18A3.5	-0.152442171452543	AGIWUR9718;AGIWUR16952;AGIWUR31759;AGIWUR34523;AGIWUR37236
CB4856	CT	II	5714924	5716250	WBGene00015945	C18A3.9	C18A3.9	-0.113618275153396	AGIWUR43356
CB4856	CT	II	5716307	5718038	WBGene00015941	C18A3.3	C18A3.3	-0.225136846817064	AGIWUR23308
CB4856	CT	II	5718129	5720052	WBGene00015940	zipt-3	C18A3.2	0.00830352391560041	AGIWUR30196
CB4856	CT	II	5720148	5721791	WBGene00015939	C18A3.1	C18A3.1	0.112931075834998	AGIWUR17352;AGIWUR27379;AGIWUR35544
CB4856	CT	II	5721791	5727651	WBGene00004267	rab-3	C18A3.6	-0.0802317450294545	AGIWUR6991;AGIWUR23755;AGIWUR42885
CB4856	CT	II	5729597	5730443	WBGene00015944	C18A3.7	C18A3.7	-0.171781930120831	AGIWUR39731
CB4856	CT	II	5731033	5731741	WBGene00044668	C18A3.11	C18A3.11	-0.0603205792196246	AGIWUR3854;AGIWUR14342
CB4856	CT	II	5737790	5738888	WBGene00001958	hlh-14	C18A3.8	0.666748033976377	AGIWUR37206
CB4856	CT	II	5742971	5745284	WBGene00044296	F10C1.9	F10C1.9	-0.0740841160586463	AGIWUR25000;AGIWUR40035
CB4856	CT	II	5750190	5754140	WBGene00002054	ifb-2	F10C1.7	-0.0352182546174209	AGIWUR2064;AGIWUR4264;AGIWUR9690;AGIWUR16751;AGIWUR17005;AGIWUR20723;AGIWUR23981;AGIWUR25070;AGIWUR25591;AGIWUR32916;AGIWUR36312;AGIWUR36639;AGIWUR39866
CB4856	CT	II	5754468	5756684	WBGene00017327	F10C1.8	F10C1.8	0.0642226256564609	AGIWUR11828;AGIWUR20400;AGIWUR23510
CB4856	CT	II	5756891	5758421	WBGene00017325	F10C1.3	F10C1.3	0.124673512552727	AGIWUR28265
CB4856	CT	II	5765618	5772084	WBGene00002053	ifb-1	F10C1.2	-0.19733674834802	AGIWUR3359;AGIWUR9541;AGIWUR22649;AGIWUR27085;AGIWUR32186;AGIWUR35412
CB4856	CT	II	5772065	5774220	WBGene00017326	dmd-5	F10C1.5	0.977669776770973	AGIWUR29796
CB4856	CT	II	5774229	5774420	WBGene00017324	F10C1.1	F10C1.1	0.177908606259731	AGIWUR41388
CB4856	CT	II	5782524	5787658	WBGene00019408	K05F1.6	K05F1.6	0.0315485305981333	AGIWUR25660
CB4856	CT	II	5790726	5794402	WBGene00019407	lect-2	K05F1.5	0.371764692877655	AGIWUR42763
CB4856	CT	II	5794495	5796185	WBGene00019406	acdh-8	K05F1.3	0.40308127549714	AGIWUR44040
CB4856	CT	II	5797893	5798341	WBGene00003469	msp-142	K05F1.2	0.21010370944645	AGIWUR20776
CB4856	CT	II	5799321	5799642	WBGene00019409	K05F1.8	K05F1.8	-0.0238568153647805	AGIWUR38573
CB4856	CT	II	5800067	5801175	WBGene00019410	K05F1.9	K05F1.9	0.0955239296113906	AGIWUR22440
CB4856	CT	II	5801382	5803680	WBGene00019405	K05F1.1	K05F1.1	-0.284030587220965	AGIWUR16912
CB4856	CT	II	5804717	5805475	WBGene00019411	K05F1.10	K05F1.10	0.259608806724815	AGIWUR32783
CB4856	CT	II	5810514	5811749	WBGene00022876	ZK1248.5	ZK1248.5	0.23580662987726	AGIWUR38632
CB4856	CT	II	5812375	5815482	WBGene00022877	wago-5	ZK1248.7	-0.156555171466424	

AGIWUR11337;AGIWUR33360;AGIWUR40670
CB4856 CT II 5816573 5817306 WBGene00044393 ZK1248.20 ZK1248.20 -0.301354559574445
AGIWUR2122;AGIWUR2856;AGIWUR13420;AGIWUR13878;AGIWUR19625
CB4856 CT II 5820712 5823919 WBGene00001224 ehs-1 ZK1248.3 -0.0278264196759599
AGIWUR724;AGIWUR6312;AGIWUR10853;AGIWUR12575;AGIWUR17892;AGIWUR33127;AGIWUR37745;AGIWUR37955;AGIWUR41914;AGIWUR42738
CB4856 CT II 5827587 5828586 WBGene00022881 ZK1248.11 ZK1248.11 -0.040740506372952
AGIWUR34944;AGIWUR43813
CB4856 CT II 5828683 5833232 WBGene00022882 ZK1248.13 ZK1248.13 -0.22266186854678
AGIWUR29446
CB4856 CT II 5833328 5836203 WBGene00001509 fzo-1 ZK1248.14 -0.0406305822993343
AGIWUR6222;AGIWUR26024;AGIWUR34210
CB4856 CT II 5836777 5838553 WBGene00022883 ZK1248.15 ZK1248.15 -0.179263118706606
AGIWUR3262
CB4856 CT II 5838663 5839414 WBGene00022885 ZK1248.19 ZK1248.19 -0.0586639684586761
AGIWUR11421;AGIWUR22217;AGIWUR44302
CB4856 CT II 5839639 5841134 WBGene00002268 lec-5 ZK1248.16 -0.251734314753733
AGIWUR24252
CB4856 CT II 5842243 5844575 WBGene00022874 nep-25 ZK1248.1 -0.36137985417151
AGIWUR2152
CB4856 CT II 5845254 5845541 WBGene00022884 ZK1248.17 ZK1248.17 -0.477058581285281
AGIWUR39346
CB4856 CT II 5845777 5850308 WBGene00019270 H41C03.3 H41C03.3 -0.347211171646379
AGIWUR11954
CB4856 CT II 5850362 5852038 WBGene00019269 H41C03.2 H41C03.2 -0.208671322042891
AGIWUR25991
CB4856 CT II 5854422 5857172 WBGene00019268 H41C03.1 H41C03.1 -0.119311540314722
AGIWUR44238
CB4856 CT II 5857080 5858422 WBGene00018871 F55C12.4 F55C12.4 -0.204925349975843
AGIWUR14984
CB4856 CT II 5864182 5867041 WBGene00018874 tag-234 F55C12.7 -0.0115859763532185
AGIWUR1948;AGIWUR3530;AGIWUR13556;AGIWUR25750;AGIWUR29295;AGIWUR32862;AGIWUR33273;AGIWUR35149
CB4856 CT II 5867268 5873614 WBGene00018872 F55C12.5 F55C12.5 -0.348391582818826
AGIWUR8048;AGIWUR12256;AGIWUR13416;AGIWUR22582;AGIWUR25368;AGIWUR29569;AGIWUR29585;AGIWUR32447;AGIWUR37960
CB4856 CT II 5874507 5876101 WBGene00018873 F55C12.6 F55C12.6 -0.0156202538656718
AGIWUR10598
CB4856 CT II 5884552 5892664 WBGene00018869 rfip-1 F55C12.1 0.136484791303824
AGIWUR14819;AGIWUR33856
CB4856 CT II 5896170 5904588 WBGene00019128 F59G1.4 F59G1.4 0.0278339766224951

AGIWUR4397;AGIWUR8473;AGIWUR22602;AGIWUR24898;AGIWUR27600;AGIWUR29800;AGIWUR33108;AGIWUR36179
CB4856 CT II 5904552 5906308 WBGene00006644 tsp-18 F59G1.2 -0.119785213794966 AGIWUR36316
CB4856 CT II 5906545 5907613 WBGene00019129 F59G1.8 F59G1.8 0.0640596489648928 AGIWUR13867
CB4856 CT II 5907794 5911222 WBGene00006933 vps-35 F59G1.3 -0.228032361620973
AGIWUR7216;AGIWUR8177;AGIWUR32419;AGIWUR39608
CB4856 CT II 5911324 5916910 WBGene00019127 cgt-3 F59G1.1 -0.0713383087053954
AGIWUR12481;AGIWUR21844;AGIWUR24749;AGIWUR30188;AGIWUR36709;AGIWUR43677
CB4856 CT II 5917422 5918564 WBGene00001486 frh-1 F59G1.7 -0.21150299357565 AGIWUR21616
CB4856 CT II 5918626 5921617 WBGene00004214 ptp-2 F59G1.5 0.0589866459117898
AGIWUR4553;AGIWUR27592
CB4856 CT II 5921967 5924838 WBGene00017895 vrk-1 F28B12.3 -0.00668448024891077
AGIWUR36435
CB4856 CT II 5933493 5940815 WBGene00001208 egl-44 F28B12.2 0.264623937142493
AGIWUR7961;AGIWUR17363;AGIWUR42207
CB4856 CT II 5942927 5943990 WBGene00017894 F28B12.1 F28B12.1 0.0664378137000341
AGIWUR3006
CB4856 CT II 5945418 5956683 WBGene00000403 casy-1 B0034.3 0.187049880263179
AGIWUR3914;AGIWUR14297;AGIWUR18652;AGIWUR18713;AGIWUR34228;AGIWUR37542;AGIWUR44343
CB4856 CT II 5961677 5962024 WBGene00044394 B0034.7 B0034.7 -0.209391049343385
AGIWUR5321;AGIWUR14988;AGIWUR15791;AGIWUR20321;AGIWUR43460
CB4856 CT II 5968079 5969058 WBGene00015004 B0034.4 B0034.4 -0.0348749586068253 AGIWUR14408
CB4856 CT II 5970105 5972658 WBGene00015002 B0034.1 B0034.1 -0.272883083007674 AGIWUR972
CB4856 CT II 5988526 5989394 WBGene00002085 ins-2 ZK75.2 0.810427382012787 AGIWUR17557
CB4856 CT II 5990103 5990818 WBGene00002086 ins-3 ZK75.3 -0.00753505165825885
AGIWUR17233;AGIWUR19321;AGIWUR27154;AGIWUR37216
CB4856 CT II 5996370 5997007 WBGene00002087 ins-4 ZK75.1 -0.022051783266976 AGIWUR27264
CB4856 CT II 5997803 5999019 WBGene00002088 ins-5 ZK84.3 -0.100217930208269
AGIWUR3314;AGIWUR4383;AGIWUR12709;AGIWUR18430;AGIWUR32680;AGIWUR37567;AGIWUR41810
CB4856 CT II 6000421 6001009 WBGene00002089 ins-6 ZK84.6 -0.0936901587943382 AGIWUR2430
CB4856 CT II 6010844 6011321 WBGene00002103 ins-20 ZK84.7 1.77343593011383
AGIWUR16068;AGIWUR20676;AGIWUR23288;AGIWUR27852;AGIWUR27895;AGIWUR32162;AGIWUR38494;AGIWUR41736;AGIWUR44906
CB4856 CT II 6014357 6015891 WBGene00022650 ZK84.2 ZK84.2 -0.15126432749746 AGIWUR25039
CB4856 CT II 6016229 6016561 WBGene00022652 ZK84.5 ZK84.5 0.139184815780308 AGIWUR12563
CB4856 CT II 6020653 6023349 WBGene00022649 ZK84.1 ZK84.1 -0.123407581629762 AGIWUR20753
CB4856 CT II 6023323 6025035 WBGene00020029 R12C12.9 R12C12.9 -0.367739942066855
AGIWUR15643
CB4856 CT II 6029514 6031206 WBGene00020023 frpr-16 R12C12.3 -0.0489002596002628
AGIWUR26305
CB4856 CT II 6039344 6039650 WBGene00020024 R12C12.4 R12C12.4 0.106787377411232

AGIWUR16314
CB4856	CT	II	6042797 6043266 WBGene00044395	R12C12.10	R12C12.10	0.103216214583685
AGIWUR4656;AGIWUR11468;AGIWUR14971;AGIWUR28761;AGIWUR34529
CB4856	CT	II	6044145 6045218 WBGene00020025	R12C12.5	R12C12.5	-0.215271411174182
AGIWUR4312
CB4856	CT	II	6046286 6047867 WBGene00020026	R12C12.6	R12C12.6	-0.141870525411008
AGIWUR2250;AGIWUR7065;AGIWUR32439;AGIWUR39270
CB4856	CT	II	6048109 6049686 WBGene00004306	ran-5	R12C12.2	0.0167276500143991
AGIWUR19265;AGIWUR21585;AGIWUR31081
CB4856	CT	II	6050332 6054307 WBGene00020022	gldc-1	R12C12.1	-0.39565660415027
AGIWUR2327;AGIWUR4982;AGIWUR19292;AGIWUR33072
CB4856	CT	II	6055039 6056388 WBGene00020027	R12C12.7	R12C12.7	0.0454976208475699
AGIWUR16099
CB4856	CT	II	6057379 6061520 WBGene00020028	R12C12.8	R12C12.8	-0.0555633398265245
AGIWUR4081;AGIWUR13422;AGIWUR28711;AGIWUR30011;AGIWUR31749;AGIWUR44325
CB4856	CT	II	6061977 6067166 WBGene00019190	H12I13.1	H12I13.1	0.0228415502517567
AGIWUR31215;AGIWUR31279
CB4856	CT	II	6071386 6073251 WBGene00019192	H12I13.3	H12I13.3	0.158376174554371
AGIWUR33937
CB4856	CT	II	6077700 6078230 WBGene00019193	H12I13.5	H12I13.5	-0.0806410710390928
AGIWUR4388;AGIWUR15709;AGIWUR23079;AGIWUR35963;AGIWUR38074
CB4856	CT	II	6077904 6078235 WBGene00019194	H12I13.6	H12I13.6	-0.467814858816997
AGIWUR13378
CB4856	CT	II	6078265 6080843 WBGene00001401	fbf-1	H12I13.4	0.410137239900433
AGIWUR10379
CB4856	CT	II	6083060 6083232 WBGene00201452	F21H12.9	F21H12.9	-0.227523288139282
AGIWUR20248
CB4856	CT	II	6088008 6088202 WBGene00017684	F21H12.2	F21H12.2	-0.503132998570528
AGIWUR3403
CB4856	CT	II	6088866 6091480 WBGene00001402	fbf-2	F21H12.5	-0.102210115397151
AGIWUR38480
CB4856	CT	II	6091820 6092789 WBGene00044287	F21H12.7	F21H12.7	0.347183586237112
AGIWUR13232;AGIWUR24259
CB4856	CT	II	6092766 6097597 WBGene00017686	tpp-2	F21H12.6	-0.325339607032896
AGIWUR1355;AGIWUR10802;AGIWUR13569;AGIWUR39948
CB4856	CT	II	6100060 6110122 WBGene00004346	rgs-3	C29H12.3	-0.0543005920129938
AGIWUR13208;AGIWUR30201;AGIWUR31773;AGIWUR42877
CB4856	CT	II	6110452 6113194 WBGene00016236	cec-9	C29H12.5	0.00505529699341167
AGIWUR31509

CB4856	CT	II	6114423	6118263	WBGene00016237	C29H12.6	C29H12.6	0.368883380060993	AGIWUR19780
CB4856	CT	II	6123809	6125438	WBGene00016235	C29H12.2	C29H12.2	0.0664967438949251	AGIWUR7944;AGIWUR11819;AGIWUR29712;AGIWUR41243
CB4856	CT	II	6125559	6127942	WBGene00004680	rars-2	C29H12.1	0.0900928077002696	AGIWUR265
CB4856	CT	II	6136094	6157176	WBGene00019018	abts-3	F57F10.1	0.245135449984776	AGIWUR36284
CB4856	CT	II	6157167	6161094	WBGene00001373	exp-1	H35N03.1	-0.111778410978484	AGIWUR28654
CB4856	CT	II	6165721	6169231	WBGene00002030	hst-3.1	F40H3.5	0.174626244843568	AGIWUR1423;AGIWUR14920;AGIWUR33288;AGIWUR34425;AGIWUR35649;AGIWUR36354;AGIWUR38927;AGIWUR43602
CB4856	CT	II	6171507	6173159	WBGene00018251	F40H3.3	F40H3.3	0.0139094483264106	AGIWUR15632
CB4856	CT	II	6173568	6174863	WBGene00018250	F40H3.2	F40H3.2	-0.455544456464246	AGIWUR22105
CB4856	CT	II	6179867	6184684	WBGene00018249	F40H3.1	F40H3.1	-0.243563131205047	AGIWUR2525;AGIWUR3859;AGIWUR26674
CB4856	CT	II	6187010	6187722	WBGene00018252	F40H3.6	F40H3.6	-0.654054252053816	AGIWUR7675
CB4856	CT	II	6189580	6191402	WBGene00001440	fkh-8	F40H3.4	0.3928170045365	AGIWUR19154
CB4856	CT	II	6192784	6195170	WBGene00000771	cpb-2	C30B5.3	-0.0161697740298177	AGIWUR9692;AGIWUR28077;AGIWUR33384
CB4856	CT	II	6195813	6196959	WBGene00016245	C30B5.4	C30B5.4	-0.0168082066986166	AGIWUR20508
CB4856	CT	II	6197180	6198308	WBGene00016244	C30B5.2	C30B5.2	-0.0995777386267331	AGIWUR1981;AGIWUR4452;AGIWUR6505;AGIWUR36902;AGIWUR41508;AGIWUR42485;AGIWUR43717
CB4856	CT	II	6198696	6201628	WBGene00077732	szy-4	C30B5.1	-0.103302810148719	AGIWUR38892
CB4856	CT	II	6219526	6222658	WBGene00016247	C30B5.6	C30B5.6	-0.0723710880442194	AGIWUR8793;AGIWUR34758;AGIWUR36628;AGIWUR38666
CB4856	CT	II	6224892	6227095	WBGene00016248	C30B5.7	C30B5.7	0.23182645203787	AGIWUR14114
CB4856	CT	II	6234483	6244287	WBGene00020784	tat-4	T24H7.5	0.134041527705206	AGIWUR10956;AGIWUR15388;AGIWUR17815;AGIWUR27470;AGIWUR28746
CB4856	CT	II	6245748	6246257	WBGene00020783	blos-4	T24H7.4	-0.209923645124039	AGIWUR43685
CB4856	CT	II	6246354	6247334	WBGene00020782	T24H7.3	T24H7.3	-0.119936467128422	AGIWUR2657;AGIWUR5181;AGIWUR13345;AGIWUR28033;AGIWUR30272
CB4856	CT	II	6247311	6250841	WBGene00020781	T24H7.2	T24H7.2	-0.0503232597613881	AGIWUR14735;AGIWUR28910;AGIWUR33691
CB4856	CT	II	6251411	6252797	WBGene00004015	phb-2	T24H7.1	-0.286240411209953	AGIWUR1862
CB4856	CT	II	6252774	6256124	WBGene00017438	F13H8.5	F13H8.5	0.0171097896652332	AGIWUR39790
CB4856	CT	II	6257628	6260021	WBGene00017443	F13H8.11	F13H8.11	0.470513849864957	AGIWUR32496
CB4856	CT	II	6260857	6262524	WBGene00017437	nmgp-1	F13H8.4	-0.04011632907355	AGIWUR27005

CB4856 CT II 6262522 6263325 WBGene00017444 F13H8.12 F13H8.12
-0.00530582825153392 AGIWUR31676
CB4856 CT II 6264050 6265502 WBGene00017436 F13H8.3 F13H8.3 -0.634347331650503 AGIWUR33928
CB4856 CT II 6265612 6268083 WBGene00017441 F13H8.8 F13H8.8 0.0567254818732446
AGIWUR9280;AGIWUR18592;AGIWUR29470;AGIWUR42923
CB4856 CT II 6270370 6274005 WBGene00017435 F13H8.2 F13H8.2 -0.214602485307413
AGIWUR6887;AGIWUR11903;AGIWUR22150;AGIWUR23635
CB4856 CT II 6274115 6279699 WBGene00000259 bpl-1 F13H8.10 -0.0476549405518831
AGIWUR5304;AGIWUR14955;AGIWUR24067;AGIWUR41077;AGIWUR44743
CB4856 CT II 6279676 6281102 WBGene00017442 F13H8.9 F13H8.9 0.0362436615700759 AGIWUR41405
CB4856 CT II 6283386 6284724 WBGene00017434 F13H8.1 F13H8.1 0.583076031223214 AGIWUR4875
CB4856 CT II 6284817 6286869 WBGene00017439 F13H8.6 F13H8.6 -0.237478580594725 AGIWUR37478
CB4856 CT II 6288413 6290097 WBGene00017440 upb-1 F13H8.7 0.289359761739336
AGIWUR9195;AGIWUR17843;AGIWUR21426;AGIWUR32208;AGIWUR41349;AGIWUR41634
CB4856 CT II 6290652 6292524 WBGene00016212 C29F5.3 C29F5.3 0.0550614812987575 AGIWUR44222
CB4856 CT II 6292652 6294356 WBGene00003403 mps-1 C29F5.4 0.157697437663371 AGIWUR17874
CB4856 CT II 6297243 6298675 WBGene00016213 C29F5.5 C29F5.5 0.0507405373452592 AGIWUR11702
CB4856 CT II 6299502 6301398 WBGene00016211 sdz-3 C29F5.2 -0.019366032736885 AGIWUR5265
CB4856 CT II 6303424 6306283 WBGene00016210 C29F5.1 C29F5.1 0.124339071616109 AGIWUR39617
CB4856 CT II 6306429 6308073 WBGene00044589 C29F5.8 C29F5.8 -0.0587519984492388
AGIWUR12713;AGIWUR30405
CB4856 CT II 6308114 6309298 WBGene00016215 glb-10 C29F5.7 -0.426099111142193 AGIWUR22465
CB4856 CT II 6318087 6321159 WBGene00016311 C32D5.3 C32D5.3 0.0775596986898837
AGIWUR24680;AGIWUR44562
CB4856 CT II 6321340 6323079 WBGene00016312 C32D5.4 C32D5.4 0.0252400514950439 AGIWUR36431
CB4856 CT II 6324484 6327355 WBGene00004860 sma-6 C32D5.2 0.00258495318181851
AGIWUR49;AGIWUR39534
CB4856 CT II 6329850 6331341 WBGene00016313 set-4 C32D5.5 0.211387423959753 AGIWUR32781
CB4856 CT II 6331899 6333987 WBGene00016314 C32D5.6 C32D5.6 -0.00916391247076831 AGIWUR23810
CB4856 CT II 6335478 6336334 WBGene00044755 C32D5.14 C32D5.14 0.0336275128448218
AGIWUR1397;AGIWUR12037;AGIWUR13449;AGIWUR17048;AGIWUR39841
CB4856 CT II 6336345 6339557 WBGene00016315 C32D5.7 C32D5.7 -0.118455846402886 AGIWUR24724
CB4856 CT II 6343058 6345925 WBGene00016316 C32D5.8 C32D5.8 -0.0367870505491932
AGIWUR20118;AGIWUR36064;AGIWUR41359;AGIWUR41461
CB4856 CT II 6346960 6347719 WBGene00002980 lgg-1 C32D5.9 0.826743750047477
AGIWUR21157;AGIWUR39449;AGIWUR39870
CB4856 CT II 6349207 6351987 WBGene00016317 C32D5.10 C32D5.10 -0.132796338283189
AGIWUR18554
CB4856 CT II 6352542 6355911 WBGene00016318 C32D5.11 C32D5.11 0.126874325211751

AGIWUR20770;AGIWUR26037;AGIWUR28595;AGIWUR31352;AGIWUR38108;AGIWUR38590
CB4856	CT	II	6356416 6359005 WBGene00016319	C32D5.12	C32D5.12	0.276931503952493
AGIWUR30696
CB4856	CT	II	6360331 6360959 WBGene00019607	K10B2.4 K10B2.4 -0.0441529853865923	AGIWUR3646
CB4856	CT	II	6361159 6365367 WBGene00019608	ani-2	K10B2.5 0.0127158365712727	AGIWUR26224
CB4856	CT	II	6365722 6367002 WBGene00019606	clec-88 K10B2.3 -0.0693439126319362
AGIWUR692;AGIWUR9567;AGIWUR33254;AGIWUR42994
CB4856	CT	II	6366990 6369210 WBGene00019605	ctsa-1	K10B2.2 -0.41669597052906
AGIWUR12427;AGIWUR18699;AGIWUR19182;AGIWUR31523;AGIWUR41372
CB4856	CT	II	6371295 6374544 WBGene00003009	lin-23	K10B2.1 0.0305400240309606	AGIWUR16062
CB4856	CT	II	6383645 6384692 WBGene00019061	F58F12.1	F58F12.1	0.10428449897053
AGIWUR9471;AGIWUR23871
CB4856	CT	II	6386767 6387568 WBGene00044756	F58F12.4	F58F12.4	-0.0389935709866158
AGIWUR4742;AGIWUR26851;AGIWUR28350
CB4856	CT	II	6387525 6388242 WBGene00019062	F58F12.2	F58F12.2	-0.243899476736955
AGIWUR32553
CB4856	CT	II	6389275 6391295 WBGene00019063	F58F12.3	F58F12.3	0.0332917210158659
AGIWUR1198;AGIWUR7481;AGIWUR10443;AGIWUR30768;AGIWUR37971;AGIWUR43033
CB4856	CT	II	6398706 6400940 WBGene00020800	zig-10	T25D10.2	0.383329930420361
AGIWUR4485;AGIWUR9846;AGIWUR26601;AGIWUR27441;AGIWUR36937
CB4856	CT	II	6401949 6403102 WBGene00020802	btb-2	T25D10.5	0.0827546732437794
AGIWUR34805
CB4856	CT	II	6403401 6405040 WBGene00020799	T25D10.1	T25D10.1	0.0726929455248619
AGIWUR43640
CB4856	CT	II	6405391 6407346 WBGene00004996	spp-11	T25D10.3	0.33265030270004
AGIWUR32974
CB4856	CT	II	6407537 6410281 WBGene00020801	T25D10.4	T25D10.4	0.747261781440208
AGIWUR42618
CB4856	CT	II	6423112 6424925 WBGene00019372	K03H9.3 K03H9.3 -0.135550862212487	AGIWUR5579
CB4856	CT	II	6426293 6427513 WBGene00000651	col-75	K03H9.2 -0.132677776197468	AGIWUR25302
CB4856	CT	II	6431261 6431875 WBGene00019371	K03H9.1 K03H9.1 -0.144046051459522	AGIWUR43614
CB4856	CT	II	6441428 6443921 WBGene00019426	cutl-16 K06A1.3 0.506532578808205	AGIWUR25549
CB4856	CT	II	6444010 6446134 WBGene00019425	K06A1.2 K06A1.2 0.0842865460582507	AGIWUR15893
CB4856	CT	II	6446079 6449819 WBGene00003621	nhr-22	K06A1.4 0.0223073702607532	AGIWUR30016
CB4856	CT	II	6450889 6453391 WBGene00019427	atg-16.2	K06A1.5 -0.00262665664126072
AGIWUR13723;AGIWUR19569;AGIWUR29959;AGIWUR42434
CB4856	CT	II	6454974 6456440 WBGene00019424	aptf-1	K06A1.1 0.255278702869273	AGIWUR35064
CB4856	CT	II	6456522 6467642 WBGene00019428	dgk-5	K06A1.6 0.164419815512622	AGIWUR36916
CB4856	CT	II	6470095 6470506 WBGene00044669	T28D9.12	T28D9.12	0.0796877046772249

AGIWUR1436;AGIWUR13032;AGIWUR16320;AGIWUR20238;AGIWUR35214
CB4856 CT II 6474338 6482125 WBGene00020897 del-10 T28D9.7 -0.362951294646864 AGIWUR19065
CB4856 CT II 6483953 6484967 WBGene00020898 T28D9.9 T28D9.9 0.053815812275797 AGIWUR13709
CB4856 CT II 6485677 6488861 WBGene00020895 T28D9.3 T28D9.3 0.0596159078831171
AGIWUR31909;AGIWUR38236;AGIWUR43877
CB4856 CT II 6488830 6489761 WBGene00004916 snr-3 T28D9.10 -0.286640504160705
AGIWUR4000;AGIWUR15440;AGIWUR22068;AGIWUR22293;AGIWUR29265;AGIWUR37787;AGIWUR42229
CB4856 CT II 6490158 6491340 WBGene00004702 rsp-5 T28D9.2 -0.126680566257656
AGIWUR5146;AGIWUR5155;AGIWUR10143;AGIWUR12090;AGIWUR17860;AGIWUR27025;AGIWUR28242;AGIWUR40736
CB4856 CT II 6491469 6491993 WBGene00020899 T28D9.11 T28D9.11 0.0776917752376787
AGIWUR21047;AGIWUR34760
CB4856 CT II 6492402 6493778 WBGene00020894 T28D9.1 T28D9.1 -0.488281140283362 AGIWUR5642
CB4856 CT II 6493720 6498633 WBGene00019904 twk-49 R05G9.2 -0.241522321392173 AGIWUR26459
CB4856 CT II 6500397 6501520 WBGene00019905 R05G9.3 R05G9.3 -0.118682896325774 AGIWUR11317
CB4856 CT II 6503360 6526333 WBGene00016970 C56E6.2 C56E6.2 -0.371399458022197
AGIWUR8020;AGIWUR8720;AGIWUR8781;AGIWUR28926
CB4856 CT II 6526372 6528801 WBGene00016972 C56E6.4 C56E6.4 0.0369690078458138
AGIWUR11683;AGIWUR17423;AGIWUR23906
CB4856 CT II 6528933 6531610 WBGene00016973 abch-1 C56E6.5 -0.00363405337868983 AGIWUR23341
CB4856 CT II 6536380 6554846 WBGene00007050 utp-20 F18C5.3 0.00682615529337216
AGIWUR10754;AGIWUR11524;AGIWUR42658;AGIWUR43051
CB4856 CT II 6554977 6559267 WBGene00006944 wrn-1 F18C5.2 0.0235458298427034 AGIWUR29106
CB4856 CT II 6559258 6561583 WBGene00017563 F18C5.10 F18C5.10 0.73205908273662
AGIWUR207;AGIWUR5082;AGIWUR13583;AGIWUR14924;AGIWUR17027;AGIWUR27722;AGIWUR36568;AGIWUR37877
CB4856 CT II 6563796 6565154 WBGene00017559 mpz-3 F18C5.4 -0.114709514357465 AGIWUR16305
CB4856 CT II 6567134 6567870 WBGene00017562 F18C5.9 F18C5.9 1.23308225274401 AGIWUR27361
CB4856 CT II 6569014 6570434 WBGene00005053 sra-27 F18C5.1 0.173074534883368 AGIWUR39606
CB4856 CT II 6570547 6573777 WBGene00005054 sra-28 F18C5.6 0.832070592277251 AGIWUR37994
CB4856 CT II 6574378 6575747 WBGene00005055 sra-29 F18C5.8 -0.188406283540717 AGIWUR25113
CB4856 CT II 6576974 6578478 WBGene00005057 sra-31 C56C10.5 -0.0966574159954234
AGIWUR32720
CB4856 CT II 6583039 6584514 WBGene00016962 C56C10.4 C56C10.4 0.341205009693954
AGIWUR12231
CB4856 CT II 6584409 6587569 WBGene00016964 C56C10.7 C56C10.7 -0.172522676314308
AGIWUR10789;AGIWUR13070;AGIWUR19595;AGIWUR43494;AGIWUR44239
CB4856 CT II 6587577 6588679 WBGene00002045 icd-1 C56C10.8 -0.0119723291463778
AGIWUR11449;AGIWUR16844;AGIWUR16923;AGIWUR19543;AGIWUR37015;AGIWUR43221
CB4856 CT II 6589290 6591081 WBGene00016961 vps-32.1 C56C10.3 0.0547035803003695
AGIWUR32474

CB4856	CT	II	6591048	6592351	WBGene00016965	C56C10.9	C56C10.9	-0.0949351414543731	AGIWUR19535
CB4856	CT	II	6592440	6594251	WBGene00016966	aipr-1	C56C10.10	0.078084277644443	AGIWUR35463
CB4856	CT	II	6594382	6598080	WBGene00001026	dnj-8	C56C10.13	-0.00285975086386614	AGIWUR18501
CB4856	CT	II	6598349	6601119	WBGene00016967	C56C10.11	C56C10.11	0.342456928089689	AGIWUR37942
CB4856	CT	II	6601216	6603449	WBGene00016960	vps-33.2	C56C10.1	-0.328250684210701	AGIWUR11698
CB4856	CT	II	6603441	6609356	WBGene00016968	epg-5	C56C10.12	-0.124981250170375	AGIWUR42321
CB4856	CT	II	6618218	6622168	WBGene00003480	klf-3	F54H5.4	0.176193296423587	AGIWUR40709
CB4856	CT	II	6622189	6623500	WBGene00018840	F54H5.3	F54H5.3	0.212534647804109	AGIWUR32792
CB4856	CT	II	6623423	6625389	WBGene00255600	F54H5.14	F54H5.14	-0.283827578912893	AGIWUR17284
CB4856	CT	II	6631105	6637099	WBGene00020579	T19D12.4	T19D12.4	0.0224630168520541	AGIWUR31097;AGIWUR40733
CB4856	CT	II	6637093	6643117	WBGene00020581	T19D12.6	T19D12.6	-0.0805738507659592	AGIWUR5677;AGIWUR11762;AGIWUR34722;AGIWUR35376
CB4856	CT	II	6647166	6649126	WBGene00020582	oig-8	T19D12.7	-0.0544171653111212	AGIWUR13576
CB4856	CT	II	6649789	6651819	WBGene00005052	sra-26	T19D12.8	-0.445508155387919	AGIWUR43747
CB4856	CT	II	6652339	6654384	WBGene00020583	T19D12.9	T19D12.9	0.100630079612513	AGIWUR13667
CB4856	CT	II	6655096	6658866	WBGene00020584	T19D12.10	T19D12.10	0.0997164301126343	AGIWUR11924
CB4856	CT	II	6660274	6660994	WBGene00020578	T19D12.3	T19D12.3	1.09731431152954	AGIWUR14410
CB4856	CT	II	6661335	6665206	WBGene00020577	T19D12.2	T19D12.2	0.203276698333201	AGIWUR3051;AGIWUR10166;AGIWUR16961;AGIWUR29672
CB4856	CT	II	6666898	6673752	WBGene00020576	T19D12.1	T19D12.1	0.256197442649075	AGIWUR44211
CB4856	CT	II	6674268	6679363	WBGene00019273	H43E16.1	H43E16.1	-0.079531870736159	AGIWUR6136;AGIWUR30081
CB4856	CT	II	6679545	6682971	WBGene00016005	ift-74	C18H9.8	0.184427546951519	AGIWUR238
CB4856	CT	II	6684236	6691841	WBGene00016002	C18H9.3	C18H9.3	-0.318304130472987	AGIWUR206;AGIWUR13368;AGIWUR18047;AGIWUR23125;AGIWUR37969;AGIWUR38975;AGIWUR41873

CB4856 CT II 6692748 6695566 WBGene00016003 C18H9.5 C18H9.5 0.230239501502972
AGIWUR4158;AGIWUR8718;AGIWUR38275
CB4856 CT II 6699084 6700482 WBGene00016000 C18H9.1 C18H9.1 0.0684286220379011 AGIWUR2090
CB4856 CT II 6700660 6701625 WBGene00016004 C18H9.6 C18H9.6 -0.127112625345825 AGIWUR41807
CB4856 CT II 6702233 6705643 WBGene00004507 rpy-1 C18H9.7 -0.00609290587608768 AGIWUR37590
CB4856 CT II 6710156 6712931 WBGene00001072 dpy-10 T14B4.7 0.301767358429888
AGIWUR5189;AGIWUR7679;AGIWUR8899;AGIWUR9246
CB4856 CT II 6714292 6715971 WBGene00001064 dpy-2 T14B4.6 1.0246157862209 AGIWUR7774
CB4856 CT II 6716863 6717868 WBGene00020501 T14B4.5 T14B4.5 -0.118856572354421 AGIWUR38846
CB4856 CT II 6718532 6720294 WBGene00006636 tsp-10 T14B4.4 -0.196343950493175 AGIWUR4910
CB4856 CT II 6720177 6729302 WBGene00020502 T14B4.8 T14B4.8 -0.16449819851807 AGIWUR25786
CB4856 CT II 6730207 6733795 WBGene00020503 T14B4.9 T14B4.9 -0.220724395143901
AGIWUR6731;AGIWUR12919;AGIWUR20426
CB4856 CT II 6734907 6736110 WBGene00020500 T14B4.3 T14B4.3 -0.250268453819123 AGIWUR16797
CB4856 CT II 6736203 6736793 WBGene00020499 T14B4.2 T14B4.2 -0.278825618230803 AGIWUR20086
CB4856 CT II 6736904 6741482 WBGene00020498 T14B4.1 T14B4.1 0.000322586868339858
AGIWUR1770;AGIWUR25503;AGIWUR37363;AGIWUR39875
CB4856 CT II 6741663 6742819 WBGene00018305 exos-8 F41G3.14 0.0332396154881103
AGIWUR34684
CB4856 CT II 6742914 6744267 WBGene00018302 F41G3.6 F41G3.6 -0.183647390602237 AGIWUR4505
CB4856 CT II 6744339 6744735 WBGene00002098 ins-15 F41G3.17 0.133879293483541
AGIWUR6727;AGIWUR34476;AGIWUR39415
CB4856 CT II 6745435 6745960 WBGene00002097 ins-14 F41G3.16 -0.0584532639699491
AGIWUR3224;AGIWUR9228;AGIWUR29224;AGIWUR40668;AGIWUR40844
CB4856 CT II 6746704 6748586 WBGene00018301 F41G3.5 F41G3.5 0.193356045546508 AGIWUR8791
CB4856 CT II 6748632 6749522 WBGene00018303 F41G3.10 F41G3.10 0.351308445703236
AGIWUR22145
CB4856 CT II 6749793 6751029 WBGene00044315 F41G3.20 F41G3.20 0.150528552801662
AGIWUR22149;AGIWUR35617
CB4856 CT II 6751624 6752127 WBGene00044317 F41G3.19 F41G3.19 0.140270425109321
AGIWUR18381;AGIWUR41751
CB4856 CT II 6752352 6752950 WBGene00044316 F41G3.21 F41G3.21 -0.371586727268484
AGIWUR6402;AGIWUR13731;AGIWUR34977
CB4856 CT II 6753126 6754774 WBGene00005986 srx-95 F41G3.11 0.131032943403284
AGIWUR36254
CB4856 CT II 6755136 6755965 WBGene00001424 fis-1 F41G3.4 0.296547682947759 AGIWUR36073
CB4856 CT II 6756476 6758934 WBGene00018300 F41G3.3 F41G3.3 0.179169671714439 AGIWUR26073
CB4856 CT II 6758885 6760385 WBGene00018299 F41G3.2 F41G3.2 0.303565513988927 AGIWUR26874
CB4856 CT II 6760684 6775176 WBGene00018304 agr-1 F41G3.12 0.741408891868233

AGIWUR11141;AGIWUR41695
CB4856 CT II 6777076 6777889 WBGene00020480 ssup-72 T13C2.4 -0.304798632775158 AGIWUR39899
CB4856 CT II 6779464 6781686 WBGene00020482 T13C2.7 T13C2.7 -0.220961728863626 AGIWUR34416
CB4856 CT II 6783427 6788255 WBGene00020481 T13C2.6 T13C2.6 0.0183466476154397 AGIWUR25670
CB4856 CT II 6789248 6791782 WBGene00020479 T13C2.3 T13C2.3 0.0264753545261049 AGIWUR27932
CB4856 CT II 6799114 6800154 WBGene00017954 F31E8.4 F31E8.4 -0.264888032107772 AGIWUR13901
CB4856 CT II 6803758 6804863 WBGene00006380 tab-1 F31E8.3 0.426731887736192
AGIWUR2387;AGIWUR23684
CB4856 CT II 6805022 6807262 WBGene00017955 F31E8.5 F31E8.5 0.197392222352715 AGIWUR41168
CB4856 CT II 6810963 6816443 WBGene00004921 snt-1 F31E8.2 -0.144765092964351
AGIWUR14122;AGIWUR14322;AGIWUR16861;AGIWUR34583;AGIWUR41369
CB4856 CT II 6817750 6819328 WBGene00017956 F31E8.6 F31E8.6 0.0413350560700909
AGIWUR2540;AGIWUR4379;AGIWUR9978;AGIWUR12041;AGIWUR20457;AGIWUR24482
CB4856 CT II 6828119 6830456 WBGene00017953 F31E8.1 F31E8.1 -0.0529919459752559 AGIWUR8839
CB4856 CT II 6835021 6847906 WBGene00001194 egl-27 C04A2.3 0.0128549294137663
AGIWUR11721;AGIWUR12570;AGIWUR20852;AGIWUR21383;AGIWUR35051;AGIWUR36106;AGIWUR43838
CB4856 CT II 6848060 6851531 WBGene00015410 tbc-6 C04A2.1 0.0607350371848341
AGIWUR21486;AGIWUR36834
CB4856 CT II 6853464 6858298 WBGene00001023 dnj-5 C04A2.7 -0.028887635529312
AGIWUR4098;AGIWUR14610;AGIWUR24347;AGIWUR32490;AGIWUR43650
CB4856 CT II 6860582 6863752 WBGene00016625 aff-1 C44B7.3 0.363985226472161 AGIWUR33412
CB4856 CT II 6868946 6871039 WBGene00016626 clhm-1 C44B7.4 -0.0848569854076722 AGIWUR13362
CB4856 CT II 6871376 6872593 WBGene00016627 C44B7.5 C44B7.5 0.00705441308827304 AGIWUR26083
CB4856 CT II 6872829 6877089 WBGene00016628 slc-36.5 C44B7.6 0.681896700159005
AGIWUR8784;AGIWUR36690
CB4856 CT II 6879185 6880180 WBGene00016629 C44B7.7 C44B7.7 -0.322706008459274 AGIWUR28103
CB4856 CT II 6880701 6883754 WBGene00004058 pmp-1 C44B7.8 -0.311693518466788 AGIWUR27618
CB4856 CT II 6884049 6887805 WBGene00004059 pmp-2 C44B7.9 -0.115739696670378
AGIWUR16453;AGIWUR17155;AGIWUR31533;AGIWUR41323
CB4856 CT II 6888553 6890418 WBGene00016630 acer-1 C44B7.10 0.816217283436901
AGIWUR7035;AGIWUR9317;AGIWUR30334;AGIWUR34052;AGIWUR44700
CB4856 CT II 6891709 6893639 WBGene00016632 C44B7.12 C44B7.12 -0.217419887140803
AGIWUR40790
CB4856 CT II 6894179 6897540 WBGene00016631 C44B7.11 C44B7.11 -0.260305781093649
AGIWUR3641;AGIWUR13332
CB4856 CT II 6898736 6901792 WBGene00016624 C44B7.2 C44B7.2 -0.0307100260759814
AGIWUR11113;AGIWUR17944;AGIWUR27708
CB4856 CT II 6902095 6902937 WBGene00016623 psmd-9 C44B7.1 -0.0791453425375188 AGIWUR30804
CB4856 CT II 6902853 6907757 WBGene00015088 B0252.3 B0252.3 -0.133862884639875

AGIWUR18014;AGIWUR18631;AGIWUR31815;AGIWUR42012
CB4856 CT II 6910031 6912697 WBGene00000211 asm-1 B0252.2 0.17679155622681 AGIWUR20342
CB4856 CT II 6912543 6913365 WBGene00000886 cyn-10 B0252.4 -0.150685699918407 AGIWUR2938
CB4856 CT II 6916110 6916963 WBGene00044615 B0252.8 B0252.8 0.217823864170988
AGIWUR6502;AGIWUR23113;AGIWUR26771;AGIWUR28445;AGIWUR32697
CB4856 CT II 6917032 6918922 WBGene00015089 B0252.5 B0252.5 0.0777605488231146 AGIWUR35704
CB4856 CT II 6919680 6923665 WBGene00015087 B0252.1 B0252.1 -0.259882602805737 AGIWUR6212
CB4856 CT II 6924115 6925951 WBGene00017700 F22D3.4 F22D3.4 -0.0347819064616552
AGIWUR14957;AGIWUR25809;AGIWUR26039;AGIWUR30794
CB4856 CT II 6928770 6947899 WBGene00017699 flcn-1 F22D3.2 -0.00964713230331871
AGIWUR26716;AGIWUR31344;AGIWUR32445;AGIWUR42782
CB4856 CT II 6947855 6951300 WBGene00017702 F22D3.6 F22D3.6 0.0178825162674716
AGIWUR15369;AGIWUR32339;AGIWUR36734;AGIWUR42314
CB4856 CT II 6951640 6955381 WBGene00000459 ceh-38 F22D3.1 0.153801606823095 AGIWUR24803
CB4856 CT II 6955402 6965687 WBGene00006605 tra-2 C15F1.3 0.147446049300132
AGIWUR13352;AGIWUR31857
CB4856 CT II 6965800 6967648 WBGene00004090 eif-2Bgamma C15F1.4 -0.164219250259293
AGIWUR19676
CB4856 CT II 6967900 6970615 WBGene00015796 C15F1.5 C15F1.5 -0.173913257152963 AGIWUR28615
CB4856 CT II 6970668 6972235 WBGene00000198 art-1 C15F1.6 -0.238285945923842
AGIWUR4636;AGIWUR10000;AGIWUR14253;AGIWUR15413;AGIWUR17324;AGIWUR25159;AGIWUR31374;AGIWUR45180
CB4856 CT II 6972712 6973892 WBGene00004930 sod-1 C15F1.7 -0.167128877781398
AGIWUR2953;AGIWUR3132;AGIWUR7934;AGIWUR9931;AGIWUR10748;AGIWUR12065;AGIWUR14775;AGIWUR21755;AGIWUR23619;AGIWUR42900;AGIWUR44763
CB4856 CT II 6976031 6977790 WBGene00015797 C15F1.8 C15F1.8 0.148917415948079 AGIWUR9736
CB4856 CT II 6979953 6988629 WBGene00015795 C15F1.2 C15F1.2 0.433701889328505 AGIWUR8419
CB4856 CT II 6988778 6991797 WBGene00015794 C15F1.1 C15F1.1 -0.245119552562274 AGIWUR39010
CB4856 CT II 6994987 7010624 WBGene00006831 unc-104 C52E12.2 -0.0893013350030847
AGIWUR5540;AGIWUR13662;AGIWUR16843
CB4856 CT II 7010798 7012690 WBGene00005025 sqv-7 C52E12.3 -0.053362791669354
AGIWUR39600
CB4856 CT II 7012787 7022509 WBGene00016889 lst-6 C52E12.4 0.202101305318868
AGIWUR35200
CB4856 CT II 7022913 7024930 WBGene00016890 lst-5 C52E12.6 -0.17990327902956
AGIWUR36634
CB4856 CT II 7025802 7030152 WBGene00016888 C52E12.1 C52E12.1 -0.0239921908576939
AGIWUR28857
CB4856 CT II 7038292 7041685 WBGene00022854 cin-4 ZK1127.7 -0.00470005768038817
AGIWUR8703

CB4856 CT II 7046041 7050071 WBGene00022855 tcer-1 ZK1127.9 0.792642125623502
AGIWUR38935
CB4856 CT II 7050672 7052001 WBGene00022852 ZK1127.5 ZK1127.5
-0.00840357274279313 AGIWUR12643
CB4856 CT II 7053949 7055208 WBGene00022851 ZK1127.4 ZK1127.4 -0.334378625729912
AGIWUR22920
CB4856 CT II 7055287 7056185 WBGene00022850 ZK1127.3 ZK1127.3 -0.237079206161164
AGIWUR20341
CB4856 CT II 7056173 7058014 WBGene00022856 cth-2 ZK1127.10 -0.262640376365283
AGIWUR40731
CB4856 CT II 7061999 7064777 WBGene00022849 acs-6 ZK1127.2 0.200989037989222
AGIWUR7902;AGIWUR26493;AGIWUR32029;AGIWUR35905
CB4856 CT II 7065020 7069966 WBGene00001872 him-14 ZK1127.11 -0.066057765613273
AGIWUR32896
CB4856 CT II 7070183 7072415 WBGene00002238 kars-1 T02G5.9 -0.235245362821638
AGIWUR8558;AGIWUR8762;AGIWUR9241;AGIWUR9623;AGIWUR14316;AGIWUR16111;AGIWUR16580;AGIWUR16967;AGIWUR17504;AGI
WUR19670;AGIWUR19785;AGIWUR20560;AGIWUR24332;AGIWUR25804;AGIWUR29473
CB4856 CT II 7073796 7075551 WBGene00002183 kat-1 T02G5.8 -0.502395625645882 AGIWUR30245
CB4856 CT II 7075614 7077188 WBGene00020166 T02G5.7 T02G5.7 0.0683433468881542 AGIWUR17844
CB4856 CT II 7089674 7093102 WBGene00020170 T02G5.14 T02G5.14 -0.0672901733322835
AGIWUR23431
CB4856 CT II 7094159 7099348 WBGene00020163 T02G5.3 T02G5.3 -0.0869628509916351
AGIWUR16849;AGIWUR21511;AGIWUR23413
CB4856 CT II 7099587 7101163 WBGene00020169 mmaa-1 T02G5.13 -0.137872084518673
AGIWUR11168;AGIWUR18345;AGIWUR31200
CB4856 CT II 7101218 7105030 WBGene00017349 farl-11 F10E7.8 -0.204283047861846
AGIWUR11964;AGIWUR23533;AGIWUR42834
CB4856 CT II 7105556 7106462 WBGene00004447 rpl-33 F10E7.7 0.107240012191314
AGIWUR11876;AGIWUR26736
CB4856 CT II 7106412 7110056 WBGene00017350 F10E7.9 F10E7.9 0.0838249690921664
AGIWUR16688;AGIWUR32795;AGIWUR33226
CB4856 CT II 7111978 7112982 WBGene00017347 F10E7.5 F10E7.5 -0.045916928837066
AGIWUR4400;AGIWUR7502;AGIWUR24469;AGIWUR42014
CB4856 CT II 7113080 7113732 WBGene00017348 F10E7.6 F10E7.6 -0.201097350301882
AGIWUR1488;AGIWUR7762;AGIWUR13851;AGIWUR26019;AGIWUR37859
CB4856 CT II 7113683 7115906 WBGene00017351 cutl-5 F10E7.10 -0.335439288003474
AGIWUR13722;AGIWUR22484
CB4856 CT II 7116557 7120558 WBGene00006893 spon-1 F10E7.4 -0.304939692768283 AGIWUR20876
CB4856 CT II 7122242 7124386 WBGene00017344 F10E7.2 F10E7.2 -0.441995091769435 AGIWUR15200

CB4856	CT	II	7126757 7129015 WBGene00017343	F10E7.1 F10E7.1 0.337250562264213	AGIWUR10452
CB4856	CT	II	7132643 7137522 WBGene00001597	gld-3	T07F8.3 -0.158214521969873
AGIWUR22578;AGIWUR44550
CB4856	CT	II	7144607 7147253 WBGene00020319	npr-31	T07F8.2 -0.0684447005819244
AGIWUR9277;AGIWUR13635
CB4856	CT	II	7149248 7151760 WBGene00020318	T07F8.1 T07F8.1 0.247660617268329	AGIWUR36349
CB4856	CT	II	7152566 7155474 WBGene00016173	fust-1	C27H5.3 0.0185284038931517
AGIWUR14809;AGIWUR16435;AGIWUR22532;AGIWUR27041;AGIWUR27422;AGIWUR32056
CB4856	CT	II	7155581 7160950 WBGene00016172	C27H5.2 C27H5.2 0.284687843576211
AGIWUR2348;AGIWUR28082;AGIWUR37493
CB4856	CT	II	7167513 7168662 WBGene00000613	col-36	C27H5.5 -0.125230402781437	AGIWUR24577
CB4856	CT	II	7168817 7171355 WBGene00001129	dyf-13	C27H5.7 0.462565194340073	AGIWUR24706
CB4856	CT	II	7172204 7174936 WBGene00001594	glc-4	C27H5.8 -0.377523602437497	AGIWUR4113
CB4856	CT	II	7183806 7185192 WBGene00003966	pdl-1	C27H5.1 0.292781869325184	AGIWUR41484
CB4856	CT	II	7185277 7187210 WBGene00016175	C27H5.6 C27H5.6 0.225319698483427	AGIWUR5202
CB4856	CT	II	7189420 7192507 WBGene00017124	E04F6.4 E04F6.4 0.279004423809011	AGIWUR27694
CB4856	CT	II	7192436 7194815 WBGene00017125	acdh-12 E04F6.5 -0.173103205572319
AGIWUR7890;AGIWUR8115
CB4856	CT	II	7196276 7197495 WBGene00017123	maoc-1	E04F6.3 0.436256036094528	AGIWUR36730
CB4856	CT	II	7197415 7199565 WBGene00017126	E04F6.6 E04F6.6 0.0589433932633381
AGIWUR3875;AGIWUR9971
CB4856	CT	II	7200233 7200418 WBGene00045095	mrpr-1	E04F6.16	0.318044317147873
AGIWUR7702;AGIWUR18469;AGIWUR20475;AGIWUR43358;AGIWUR43666
CB4856	CT	II	7200611 7201880 WBGene00017122	pcrg-1	E04F6.2 0.0161403742213633	AGIWUR38515
CB4856	CT	II	7202016 7203210 WBGene00005134	srd-56	E04F6.14	-0.148668238310587
AGIWUR7886
CB4856	CT	II	7203911 7205051 WBGene00005135	srd-58	E04F6.13	0.212038435718576
AGIWUR34211
CB4856	CT	II	7205688 7206892 WBGene00005136	srd-59	E04F6.1 0.136604044870433	AGIWUR2527
CB4856	CT	II	7206990 7208460 WBGene00000971	dhs-7	E04F6.7 -0.569228648668249	AGIWUR40494
CB4856	CT	II	7208773 7210287 WBGene00017131	E04F6.15	E04F6.15	-0.293841356835291
AGIWUR13348
CB4856	CT	II	7210731 7211380 WBGene00017127	E04F6.8 E04F6.8 -0.11243534426815
AGIWUR14338;AGIWUR16369;AGIWUR26017;AGIWUR26803;AGIWUR27394;AGIWUR44297
CB4856	CT	II	7211625 7212159 WBGene00017128	E04F6.9 E04F6.9 0.167999579465318
AGIWUR19581;AGIWUR30555;AGIWUR35295
CB4856	CT	II	7214055 7216957 WBGene00017129	E04F6.10	E04F6.10
-0.00520811748914873	AGIWUR3141;AGIWUR7507;AGIWUR15684;AGIWUR26762
CB4856	CT	II	7217076 7217478 WBGene00017130	E04F6.12	E04F6.12	-0.103985363967617

AGIWUR22286
CB4856 CT II 7217955 7225648 WBGene00000530 clh-3 E04F6.11 -0.11849478457875
AGIWUR15731;AGIWUR24176;AGIWUR25909;AGIWUR41732
CB4856 CT II 7228784 7230616 WBGene00017970 F32A5.4 F32A5.4 0.34050757833058
AGIWUR1286;AGIWUR8256;AGIWUR16085;AGIWUR39884
CB4856 CT II 7232853 7235920 WBGene00017969 F32A5.3 F32A5.3 -0.377575341805485
AGIWUR12121;AGIWUR13016;AGIWUR27135;AGIWUR36318
CB4856 CT II 7235834 7238349 WBGene00000169 aqp-1 F32A5.5 1.4403775274477
AGIWUR4628;AGIWUR5582
CB4856 CT II 7245876 7250181 WBGene00017968 skpo-3 F32A5.2 -0.16659824167325
AGIWUR884;AGIWUR9594;AGIWUR11173;AGIWUR29384;AGIWUR34437;AGIWUR41351
CB4856 CT II 7250151 7252010 WBGene00004198 prx-13 F32A5.6 0.190994207891816 AGIWUR18591
CB4856 CT II 7252259 7255735 WBGene00017967 ada-2 F32A5.1 -0.167615377324051 AGIWUR15959
CB4856 CT II 7256112 7258112 WBGene00017971 F32A5.8 F32A5.8 0.00607008702426359
AGIWUR24468;AGIWUR31093
CB4856 CT II 7258389 7258829 WBGene00023021 F32A5.9 F32A5.9 -0.0154839810993135
AGIWUR9798;AGIWUR18140;AGIWUR30385
CB4856 CT II 7261648 7263654 WBGene00016276 C30G12.4 C30G12.4 -0.0581739853233278
AGIWUR13871;AGIWUR19260;AGIWUR31662;AGIWUR32334
CB4856 CT II 7274732 7277223 WBGene00016275 C30G12.3 C30G12.3 0.0549938906869114
AGIWUR23627
CB4856 CT II 7290373 7291882 WBGene00016274 C30G12.2 C30G12.2 -0.794038922101063
AGIWUR32700
CB4856 CT II 7292192 7296015 WBGene00016277 C30G12.6 C30G12.6 -0.0734961426731111
AGIWUR13314
CB4856 CT II 7296110 7298247 WBGene00004244 puf-8 C30G12.7 0.00864957672300637
AGIWUR29920;AGIWUR31561;AGIWUR36265
CB4856 CT II 7299514 7301632 WBGene00016273 C30G12.1 C30G12.1 -0.0843629736744003
AGIWUR26118
CB4856 CT II 7302101 7305760 WBGene00000839 cul-4 F45E12.3 -0.0544715157518532
AGIWUR495;AGIWUR13180;AGIWUR36169
CB4856 CT II 7305861 7308738 WBGene00000271 brf-1 F45E12.2 -0.451212125593813
AGIWUR18584
CB4856 CT II 7308702 7310115 WBGene00018475 mrpl-14 F45E12.5 -0.0839586405099766
AGIWUR11390;AGIWUR37904
CB4856 CT II 7310348 7313787 WBGene00018474 cnep-1 F45E12.1 0.034628293188435
AGIWUR35826
CB4856 CT II 7313774 7314181 WBGene00018476 F45E12.6 F45E12.6
-0.00467195645196661 AGIWUR27968

CB4856 CT II 7318684 7319153 WBGene00017662 F21D12.2 F21D12.2 -0.0149186072650895
AGIWUR25324
CB4856 CT II 7319910 7323200 WBGene00017663 F21D12.3 F21D12.3 0.603551024073656
AGIWUR34329
CB4856 CT II 7328736 7331324 WBGene00017664 npax-1 F21D12.5 -0.0909671970058623
AGIWUR29093
CB4856 CT II 7339229 7349124 WBGene00003620 nhr-21 F21D12.1 1.64421188353225
AGIWUR32353;AGIWUR34044;AGIWUR38422
CB4856 CT II 7350121 7352636 WBGene00002008 hsp-4 F43E2.8 -0.456416650111485 AGIWUR16929
CB4856 CT II 7353576 7356426 WBGene00001812 haf-2 F43E2.4 -0.122364722091067 AGIWUR37462
CB4856 CT II 7358913 7359504 WBGene00018396 F43E2.9 F43E2.9 0.227847285510749 AGIWUR10546
CB4856 CT II 7359961 7363247 WBGene00018392 insc-1 F43E2.3 0.153129237220604 AGIWUR35459
CB4856 CT II 7363216 7364107 WBGene00018393 msra-1 F43E2.5 -0.851943144692235 AGIWUR8710
CB4856 CT II 7366591 7369049 WBGene00018394 F43E2.6 F43E2.6 -0.197970244246253
AGIWUR14479;AGIWUR28754;AGIWUR30906;AGIWUR36753;AGIWUR37020
CB4856 CT II 7369992 7371275 WBGene00018397 F43E2.11 F43E2.11 0.155875792080939
AGIWUR28564
CB4856 CT II 7372594 7374172 WBGene00018395 mtch-1 F43E2.7 -0.0178685861037762
AGIWUR157;AGIWUR28677
CB4856 CT II 7374501 7375237 WBGene00018391 rpb-4 F43E2.2 0.0337371097712964 AGIWUR21679
CB4856 CT II 7379177 7379612 WBGene00044673 C07D10.6 C07D10.6 0.099077007128324
AGIWUR13270;AGIWUR14746;AGIWUR30620;AGIWUR31090;AGIWUR44996
CB4856 CT II 7381561 7383499 WBGene00015569 C07D10.5 C07D10.5 0.544235698439332
AGIWUR44000
CB4856 CT II 7387162 7389142 WBGene00015568 sre-3 C07D10.3 0.148129631937995
AGIWUR45007
CB4856 CT II 7394765 7397351 WBGene00015567 bath-44 C07D10.2 0.00186499627607972
AGIWUR36096
CB4856 CT II 7399012 7400540 WBGene00015566 C07D10.1 C07D10.1 -0.0322228314167639
AGIWUR18473
CB4856 CT II 7400544 7402724 WBGene00003526 nas-7 C07D10.4 -0.603713807908431
AGIWUR3626;AGIWUR16232;AGIWUR39221
CB4856 CT II 7408492 7410747 WBGene00005133 srd-55 K02A2.2 0.256377196060638 AGIWUR21751
CB4856 CT II 7411075 7412030 WBGene00019290 K02A2.5 K02A2.5 0.139747545550073
AGIWUR3083;AGIWUR17628;AGIWUR20926
CB4856 CT II 7425935 7431390 WBGene00019289 kcc-3 K02A2.3 0.0195949878524599 AGIWUR7656
CB4856 CT II 7432696 7434216 WBGene00005132 srd-54 K02A2.7 -0.278431583016248
AGIWUR9242;AGIWUR28397
CB4856 CT II 7435852 7436999 WBGene00005130 srd-52 K02A2.4 0.288828626289649

AGIWUR5866;AGIWUR13963;AGIWUR24611;AGIWUR39878
CB4856 CT II 7456242 7457380 WBGene00017023 D1022.4 D1022.4 -0.114157706577103 AGIWUR19967
CB4856 CT II 7457667 7458806 WBGene00017024 D1022.5 D1022.5 0.216449640748247
AGIWUR30740;AGIWUR45185
CB4856 CT II 7459531 7460076 WBGene00017021 D1022.2 D1022.2 0.134905640054071 AGIWUR8681
CB4856 CT II 7460110 7463614 WBGene00005641 sro-1 D1022.6 -0.0731123377037335 AGIWUR15307
CB4856 CT II 7472729 7478959 WBGene00000101 aka-1 D1022.7 -0.151399827064945
AGIWUR3114;AGIWUR3158;AGIWUR6829;AGIWUR12669;AGIWUR17212;AGIWUR18049;AGIWUR24183;AGIWUR40155
CB4856 CT II 7479064 7480713 WBGene00006703 ubc-6 D1022.1 -0.141182438975349 AGIWUR40011
CB4856 CT II 7480751 7483109 WBGene00000280 cah-2 D1022.8 -0.0315667987999166 AGIWUR1356
CB4856 CT II 7488242 7489661 WBGene00044300 D1022.9 D1022.9 0.145950105124017
AGIWUR33482;AGIWUR42517
CB4856 CT II 7491399 7493699 WBGene00003542 nas-23 R10H1.5 -0.0316607242008304
AGIWUR4989;AGIWUR8815;AGIWUR12650;AGIWUR18219;AGIWUR22957;AGIWUR30287
CB4856 CT II 7494460 7496174 WBGene00019998 R10H1.1 R10H1.1 0.480518463256153 AGIWUR6877
CB4856 CT II 7496247 7497369 WBGene00023416 spi-1 R10H1.4 0.0977034524402018
AGIWUR2722;AGIWUR5494;AGIWUR8413;AGIWUR33980;AGIWUR34800;AGIWUR41809
CB4856 CT II 7497286 7499475 WBGene00019999 srab-14 R10H1.2 0.188059549584549
AGIWUR20225;AGIWUR41600
CB4856 CT II 7505351 7506595 WBGene00016183 C28F5.1 C28F5.1 -0.288461714834931 AGIWUR23368
CB4856 CT II 7507319 7513975 WBGene00016184 glb-9 C28F5.2 0.0431340549772692
AGIWUR3730;AGIWUR40072
CB4856 CT II 7519860 7523368 WBGene00016185 C28F5.4 C28F5.4 -0.0623967576033362 AGIWUR19483
CB4856 CT II 7523581 7524421 WBGene00044775 ZK1290.15 ZK1290.15 -0.406837532554825
AGIWUR634;AGIWUR9068;AGIWUR12802;AGIWUR26783;AGIWUR31840
CB4856 CT II 7524467 7525558 WBGene00022889 ZK1290.7 ZK1290.7 -0.425832085610483
AGIWUR43100
CB4856 CT II 7525738 7528241 WBGene00022888 rnh-1.1 ZK1290.6 -0.0219835255903451
AGIWUR18436
CB4856 CT II 7529643 7530991 WBGene00022887 ZK1290.5 ZK1290.5 -0.045203463657483
AGIWUR13403
CB4856 CT II 7531145 7533246 WBGene00006947 wrt-1 ZK1290.12 0.444595683356443
AGIWUR18642
CB4856 CT II 7533300 7534563 WBGene00006956 wrt-10 ZK1290.8 0.939125709919111
AGIWUR42222
CB4856 CT II 7535006 7536989 WBGene00022893 ZK1290.13 ZK1290.13 0.308799999175855
AGIWUR16643
CB4856 CT II 7538187 7543000 WBGene00003592 nfi-1 ZK1290.4 -0.155912426810319
AGIWUR3009;AGIWUR17692;AGIWUR20277;AGIWUR43039

CB4856 CT II 7544069 7545335 WBGene00004398 rol-8 ZK1290.3 -0.347653301012324 AGIWUR40755;AGIWUR41897
CB4856 CT II 7546807 7548129 WBGene00022890 fbxa-224 ZK1290.9 0.221382324724594 AGIWUR8162;AGIWUR22574;AGIWUR33222
CB4856 CT II 7549335 7551977 WBGene00006600 tph-1 ZK1290.2 0.0649599591221275 AGIWUR15624;AGIWUR34300;AGIWUR45200
CB4856 CT II 7551951 7553906 WBGene00022891 ZK1290.10 ZK1290.10 -0.233601006252089 AGIWUR20209
CB4856 CT II 7554092 7554844 WBGene00044396 ZK1290.14 ZK1290.14 0.609885154946095 AGIWUR31327
CB4856 CT II 7560925 7561594 WBGene00022892 ZK1290.11 ZK1290.11 -0.154572041050878 AGIWUR11550
CB4856 CT II 7561753 7562701 WBGene00022886 ZK1290.1 ZK1290.1 0.029892028523598 AGIWUR14827
CB4856 CT II 7570605 7572444 WBGene00018041 F35D2.3 F35D2.3 0.811194679210292 AGIWUR25303
CB4856 CT II 7574431 7575830 WBGene00018040 F35D2.2 F35D2.2 0.254879412178354 AGIWUR28100
CB4856 CT II 7577723 7580244 WBGene00194916 F35D2.6 F35D2.6 -0.0509314007057906 AGIWUR5613
CB4856 CT II 7580270 7583557 WBGene00018042 mks-3 F35D2.4 0.392638733883182 AGIWUR1760;AGIWUR12494;AGIWUR15947
CB4856 CT II 7585731 7594912 WBGene00006363 syd-1 F35D2.5 0.376231865410277 AGIWUR5413;AGIWUR13745;AGIWUR15444;AGIWUR20159;AGIWUR24267;AGIWUR33701
CB4856 CT II 7597545 7598752 WBGene00019941 pgam-5 R07G3.5 -0.139058064756186 AGIWUR8443;AGIWUR16940;AGIWUR37105;AGIWUR38237
CB4856 CT II 7599657 7608014 WBGene00019940 npp-21 R07G3.3 0.0195105310422505 AGIWUR8835;AGIWUR16750;AGIWUR44811
CB4856 CT II 7608253 7610986 WBGene00019939 lips-17 R07G3.2 -0.333005446178445 AGIWUR37450
CB4856 CT II 7610986 7613730 WBGene00019943 R07G3.7 R07G3.7 -0.164621604122603 AGIWUR29343
CB4856 CT II 7613541 7614434 WBGene00043050 oig-4 R07G3.9 0.337857583541263 AGIWUR43422;AGIWUR44530
CB4856 CT II 7616452 7618245 WBGene00000390 cdc-42 R07G3.1 0.0879079018603655 AGIWUR19545;AGIWUR38027
CB4856 CT II 7618238 7620513 WBGene00019944 R07G3.8 R07G3.8 0.157937054481567 AGIWUR9490;AGIWUR14987;AGIWUR16684;AGIWUR38424
CB4856 CT II 7627250 7651295 WBGene00020114 pde-4 R153.1 0.073422056851256 AGIWUR19305;AGIWUR24964;AGIWUR29067;AGIWUR34488;AGIWUR36443;AGIWUR36503;AGIWUR36847;AGIWUR39101;AGIWUR40129;AGIWUR40871;AGIWUR41328
CB4856 CT II 7658324 7662522 WBGene00017547 alfa-1 F18A1.6 0.0209943639079846 AGIWUR39387
CB4856 CT II 7662706 7663754 WBGene00017548 F18A1.7 F18A1.7 -0.181184213977573 AGIWUR4578;AGIWUR9651;AGIWUR31096

CB4856	CT	II	7664582	7667110	WBGene00017546	rpa-1	F18A1.5	-0.240781266690581	AGIWUR6934
CB4856	CT	II	7667993	7671905	WBGene00003045	lir-2	F18A1.4	-0.163555251557295	AGIWUR26034
CB4856	CT	II	7672010	7682359	WBGene00003044	lir-1	F18A1.3	-0.119166069224583	AGIWUR17589;AGIWUR35849;AGIWUR42838
CB4856	CT	II	7682198	7684760	WBGene00003012	lin-26	F18A1.2	0.11905450624284	AGIWUR3543;AGIWUR19668;AGIWUR28083;AGIWUR31105;AGIWUR32625
CB4856	CT	II	7684889	7689689	WBGene00017545	F18A1.1	F18A1.1	0.0523876963968011	AGIWUR23601;AGIWUR25978;AGIWUR40021
CB4856	CT	II	7689710	7690572	WBGene00017549	pid-1	F18A1.8	-0.208879251190794	AGIWUR21471
CB4856	CT	II	7690711	7693957	WBGene00003730	nhx-2	B0495.4	-0.179800678633336	AGIWUR12604
CB4856	CT	II	7695935	7698506	WBGene00015204	B0495.5	B0495.5	0.046808925867813	AGIWUR31611
CB4856	CT	II	7698613	7699046	WBGene00015205	moa-2	B0495.6	0.0177903661330028	AGIWUR24473
CB4856	CT	II	7699254	7702012	WBGene00015203	cdk-11.1	B0495.2	-0.0236689063483624	AGIWUR6112;AGIWUR35553;AGIWUR42351
CB4856	CT	II	7701948	7705276	WBGene00015206	B0495.7	B0495.7	0.0271306690270015	AGIWUR15900;AGIWUR33473
CB4856	CT	II	7705536	7707483	WBGene00015207	B0495.8	B0495.8	0.012317322678848	AGIWUR1814;AGIWUR6376;AGIWUR14232;AGIWUR18839;AGIWUR31518;AGIWUR45176
CB4856	CT	II	7707586	7708725	WBGene00015208	B0495.9	B0495.9	-0.114723810006893	AGIWUR13090
CB4856	CT	II	7709789	7711444	WBGene00005149	sre-1	B0495.1	0.0297622074207187	AGIWUR10241
CB4856	CT	II	7711871	7718591	WBGene00007044	cpna-5	B0495.10	0.326706749331787	AGIWUR11799;AGIWUR15380;AGIWUR25684
CB4856	CT	II	7724903	7752715	WBGene00015061	cpna-2	B0228.4	-0.099954412904415	AGIWUR6619;AGIWUR9887;AGIWUR16731;AGIWUR25986;AGIWUR31582;AGIWUR34570;AGIWUR38002
CB4856	CT	II	7752637	7753607	WBGene00015062	trx-1	B0228.5	0.229819401494149	AGIWUR23893;AGIWUR30447;AGIWUR42639
CB4856	CT	II	7754147	7755156	WBGene00015063	B0228.6	B0228.6	0.16287074921245	AGIWUR23399
CB4856	CT	II	7755997	7756907	WBGene00015059	B0228.1	B0228.1	-0.128041626621943	AGIWUR36033;AGIWUR39436;AGIWUR45163
CB4856	CT	II	7757190	7758922	WBGene00015064	B0228.7	B0228.7	0.198160183826797	AGIWUR30631
CB4856	CT	II	7759710	7761540	WBGene00015065	B0228.8	B0228.8	0.0963439658737819	AGIWUR32882
CB4856	CT	II	7762062	7763669	WBGene00015066	B0228.9	B0228.9	-0.273616563991671	AGIWUR2478;AGIWUR10101
CB4856	CT	II	7763937	7765516	WBGene00005262	srh-39	C06A8.7	0.239654059525781	AGIWUR17170;AGIWUR21579;AGIWUR36225
CB4856	CT	II	7765545	7772017	WBGene00015517	C06A8.8	C06A8.8	0.027838805230215	AGIWUR13385;AGIWUR31560
CB4856	CT	II	7772499	7773914	WBGene00015516	C06A8.6	C06A8.6	-0.0358797345457518	AGIWUR378
CB4856	CT	II	7773880	7777827	WBGene00015512	mthf-1	C06A8.1	0.0496672201019877	AGIWUR23482

CB4856	CT	II	7779297	7781301	WBGene00015513	snpc-1.1	C06A8.2	-0.10036968478401	AGIWUR28830
CB4856	CT	II	7781566	7783354	WBGene00015515	spdl-1	C06A8.5	-0.0310099404887829	AGIWUR5128
CB4856	CT	II	7783829	7784654	WBGene00004823	skr-17	C06A8.4	-0.0983815584401864	AGIWUR37247
CB4856	CT	II	7785100	7794319	WBGene00001615	glr-4	C06A8.9	-0.174941232679579	AGIWUR6451;AGIWUR19537;AGIWUR21410
CB4856	CT	II	7811240	7812105	WBGene00000516	cki-1	T05A6.1	1.01374067557076	AGIWUR39862
CB4856	CT	II	7816509	7820301	WBGene00000517	cki-2	T05A6.2	-0.0462823257435954	AGIWUR15835;AGIWUR32206
CB4856	CT	II	7820333	7822554	WBGene00011458	irld-47	T05A6.4	0.160382748482809	AGIWUR4446
CB4856	CT	II	7822749	7825196	WBGene00011459	T05A6.5	T05A6.5	-0.0455015442136038	AGIWUR10914
CB4856	CT	II	7825407	7826694	WBGene00005809	srw-62	T05A6.6	0.0211055023606339	AGIWUR14075;AGIWUR40775
CB4856	CT	II	7832391	7832668	WBGene00011640	T09A5.14	T09A5.14	-0.314207003224835	AGIWUR35
CB4856	CT	II	7832844	7835414	WBGene00023408	cex-2	T09A5.1	0.721711645960058	AGIWUR34455
CB4856	CT	II	7842353	7845653	WBGene00002216	klp-3	T09A5.2	-0.396984848239372	AGIWUR4382;AGIWUR20513;AGIWUR29273;AGIWUR36971
CB4856	CT	II	7845662	7849797	WBGene00000046	acr-7	T09A5.3	-0.141219141859576	AGIWUR3882
CB4856	CT	II	7852821	7853779	WBGene00011634	T09A5.5	T09A5.5	-0.290137442219628	AGIWUR19073
CB4856	CT	II	7853880	7855042	WBGene00007014	mdt-10	T09A5.6	-0.0357238315702588	AGIWUR41745
CB4856	CT	II	7855135	7855719	WBGene00011635	T09A5.7	T09A5.7	-0.356202322910551	AGIWUR7525
CB4856	CT	II	7855814	7857600	WBGene00011636	cec-3	T09A5.8	0.0185082503758096	AGIWUR33974
CB4856	CT	II	7857811	7858334	WBGene00011641	T09A5.15	T09A5.15	-0.226257495695817	AGIWUR23369
CB4856	CT	II	7858401	7859781	WBGene00011637	sds-22	T09A5.9	-0.131555884308128	AGIWUR24015;AGIWUR38639;AGIWUR40986
CB4856	CT	II	7860574	7863563	WBGene00002994	lin-5	T09A5.10	-0.20668743589489	AGIWUR1605;AGIWUR16198;AGIWUR34726
CB4856	CT	II	7864074	7866256	WBGene00011638	ostb-1	T09A5.11	-0.441001413428692	AGIWUR20189;AGIWUR29057;AGIWUR41636
CB4856	CT	II	7866256	7870162	WBGene00011639	ztf-17	T09A5.12	-0.0389657452959211	AGIWUR4682;AGIWUR13291;AGIWUR19500;AGIWUR22289
CB4856	CT	II	7870881	7873893	WBGene00011349	T01H3.3	T01H3.3	-0.0628049767555474	AGIWUR23452
CB4856	CT	II	7874242	7878097	WBGene00011348	T01H3.2	T01H3.2	-0.115912395038638	AGIWUR24353
CB4856	CT	II	7878083	7879406	WBGene00006913	vha-4	T01H3.1	-0.295828399684397	AGIWUR1184;AGIWUR10620;AGIWUR20756;AGIWUR24307
CB4856	CT	II	7879720	7880464	WBGene00011351	T01H3.5	T01H3.5	-0.278609268351197	AGIWUR24996
CB4856	CT	II	7880812	7882767	WBGene00011350	perm-1	T01H3.4	-0.285967991719929	AGIWUR7835

CB4856 CT II 7893229 7900510 WBGene00004208 ptc-1 ZK675.1 0.0715546335147943
AGIWUR3463;AGIWUR7183;AGIWUR24141;AGIWUR37844;AGIWUR40130;AGIWUR41556
CB4856 CT II 7900647 7904636 WBGene00014066 rev-1 ZK675.2 0.0586619100150632 AGIWUR37852
CB4856 CT II 7904639 7907589 WBGene00014989 best-23 ZK675.3 0.299167815143618
AGIWUR3527;AGIWUR8672;AGIWUR13711;AGIWUR14386;AGIWUR17626;AGIWUR32827;AGIWUR39001
CB4856 CT II 7911323 7912113 WBGene00014067 ZK675.4 ZK675.4 1.11565596327376 AGIWUR20932
CB4856 CT II 7912093 7928694 WBGene00014051 spv-1 ZK669.1 -0.0253763359512256
AGIWUR26977;AGIWUR30516;AGIWUR36509
CB4856 CT II 7936592 7938583 WBGene00014052 ZK669.2 ZK669.2 -0.165381647697272 AGIWUR4723
CB4856 CT II 7942463 7945206 WBGene00014054 dbt-1 ZK669.4 -0.0948760014669325 AGIWUR37925
CB4856 CT II 7945501 7946894 WBGene00014055 ZK669.5 ZK669.5 0.0788378084811754
AGIWUR4467;AGIWUR5732;AGIWUR27011;AGIWUR33319;AGIWUR33597
CB4856 CT II 7958844 7960237 WBGene00007311 C04H4.1 C04H4.1 -0.0119215090663349 AGIWUR17895
CB4856 CT II 7976614 7994206 WBGene00008438 DH11.5 DH11.5 0.133085649257535
AGIWUR8512;AGIWUR15852;AGIWUR17295;AGIWUR34568
CB4856 CT II 7995587 8003152 WBGene00004005 pgp-11 DH11.3 -0.0722510249370885 AGIWUR43425
CB4856 CT II 8008619 8011633 WBGene00008435 glna-2 DH11.1 0.179548395594326 AGIWUR29345
CB4856 CT II 8012816 8015264 WBGene00008436 DH11.2 DH11.2 0.67460245819014 AGIWUR42760
CB4856 CT II 8017669 8020785 WBGene00006996 zyg-11 C08B11.1 -0.245862505005209
AGIWUR3394;AGIWUR3850;AGIWUR10462;AGIWUR19652
CB4856 CT II 8020780 8022561 WBGene00001835 hda-2 C08B11.2 -0.265765299249367
AGIWUR26485
CB4856 CT II 8022649 8027756 WBGene00007433 swsn-7 C08B11.3 -0.0646145633805837
AGIWUR43476
CB4856 CT II 8028953 8032742 WBGene00003813 nrf-6 C08B11.4 -0.258325154059527
AGIWUR7101
CB4856 CT II 8034250 8035767 WBGene00004723 sap-49 C08B11.5 -0.0750339409946434
AGIWUR2998;AGIWUR3824;AGIWUR4991;AGIWUR5242;AGIWUR6636;AGIWUR32094;AGIWUR42094;AGIWUR43153
CB4856 CT II 8035869 8037346 WBGene00007434 arp-6 C08B11.6 0.0942478036376544
AGIWUR5868;AGIWUR37186;AGIWUR38567;AGIWUR42177
CB4856 CT II 8037316 8038167 WBGene00007436 C08B11.9 C08B11.9 -0.0303556577150026
AGIWUR14890
CB4856 CT II 8038271 8039597 WBGene00006724 ubh-4 C08B11.7 -0.111729898023903
AGIWUR8971;AGIWUR33365;AGIWUR43766
CB4856 CT II 8040006 8041732 WBGene00007435 C08B11.8 C08B11.8 -0.117615131522607
AGIWUR31312
CB4856 CT II 8042589 8047452 WBGene00011507 T05H10.1 T05H10.1 -0.17280581170974
AGIWUR7238;AGIWUR35900;AGIWUR42683
CB4856 CT II 8047564 8049105 WBGene00000151 apn-1 T05H10.2 -0.0731485159467233

AGIWUR5389;AGIWUR33387;AGIWUR40600
CB4856 CT II 8049049 8050564 WBGene00011508 T05H10.3 T05H10.3 1.26465944016232
AGIWUR35674
CB4856 CT II 8053137 8054965 WBGene00011509 T05H10.4 T05H10.4 -0.129024931694516
AGIWUR18325
CB4856 CT II 8059069 8061464 WBGene00011512 T05H10.8 T05H10.8 -0.501182527662656
AGIWUR2912
CB4856 CT II 8062741 8066193 WBGene00006734 ufd-2 T05H10.5 -0.104720450971747
AGIWUR6799
CB4856 CT II 8066111 8068184 WBGene00011510 pdha-1 T05H10.6 0.103965282158553
AGIWUR31011
CB4856 CT II 8067990 8073372 WBGene00011511 gpcp-2 T05H10.7 -0.124382216252427
AGIWUR983;AGIWUR13509;AGIWUR13688;AGIWUR15497
CB4856 CT II 8075760 8076836 WBGene00010501 K02C4.2 K02C4.2 0.286956907283906
AGIWUR7423;AGIWUR23396;AGIWUR28188
CB4856 CT II 8078457 8080847 WBGene00010503 K02C4.5 K02C4.5 0.438557655571275 AGIWUR17113
CB4856 CT II 8080789 8086849 WBGene00010502 K02C4.3 K02C4.3 -0.0595418067267897
AGIWUR5833;AGIWUR12426;AGIWUR31499
CB4856 CT II 8088427 8092754 WBGene00003089 ltd-1 K02C4.4 0.603623829187951 AGIWUR7221
CB4856 CT II 8100375 8102141 WBGene00005150 sre-2 C41C4.2 -0.268571915579969 AGIWUR22986
CB4856 CT II 8102108 8105052 WBGene00044049 sfxn-5 C41C4.10 -0.0894288527506415
AGIWUR5877;AGIWUR16602;AGIWUR26988
CB4856 CT II 8105614 8106967 WBGene00008050 chst-1 C41C4.1 0.136914893184478 AGIWUR7477
CB4856 CT II 8107645 8108954 WBGene00008054 C41C4.9 C41C4.9 0.0961310007720613 AGIWUR16390
CB4856 CT II 8108941 8112163 WBGene00008051 lron-4 C41C4.3 -0.00715620531788019 AGIWUR2733
CB4856 CT II 8112262 8124910 WBGene00002147 ire-1 C41C4.4 -0.00815289383342588 AGIWUR29507
CB4856 CT II 8129960 8134742 WBGene00006739 ulp-4 C41C4.6 -0.0344188555911371
AGIWUR2044;AGIWUR6926
CB4856 CT II 8134765 8136916 WBGene00008052 ctns-1 C41C4.7 -0.0148804559319942
AGIWUR22871;AGIWUR30307;AGIWUR39329
CB4856 CT II 8137880 8141276 WBGene00008053 cdc-48.2 C41C4.8 -0.310966243746437
AGIWUR22177;AGIWUR23786
CB4856 CT II 8141213 8143478 WBGene00008642 F10B5.8 F10B5.8 0.282129066645346 AGIWUR42674
CB4856 CT II 8143863 8144671 WBGene00004421 rpl-10 F10B5.1 0.10282212731758
AGIWUR15626;AGIWUR19210;AGIWUR25935;AGIWUR27480;AGIWUR38463;AGIWUR42597;AGIWUR44548
CB4856 CT II 8144762 8147236 WBGene00008639 F10B5.2 F10B5.2 0.00882504837381504 AGIWUR23843
CB4856 CT II 8147190 8150030 WBGene00008640 F10B5.3 F10B5.3 -0.173119075116162 AGIWUR14621
CB4856 CT II 8154096 8156937 WBGene00006655 tub-1 F10B5.4 -0.218984154680566 AGIWUR23466
CB4856 CT II 8157362 8158045 WBGene00008643 tmem-138 F10B5.9 0.00706614670029371

AGIWUR3504
CB4856	CT	II	8158349 8159925 WBGene00008641	pch-2	F10B5.5 -0.172119863421013	AGIWUR2022
CB4856	CT	II	8159865 8162170 WBGene00001281	emb-27	F10B5.6 -0.249659441056602	AGIWUR21615
CB4856	CT	II	8162465 8168644 WBGene00004510	rrf-3	F10B5.7 0.23612304695912	AGIWUR12684
CB4856	CT	II	8168682 8170358 WBGene00011473	T05C12.11	T05C12.11	0.0653902574988479
AGIWUR37097
CB4856	CT	II	8172229 8173562 WBGene00011467	decr-1.3	T05C12.3	0.0331073339260808
AGIWUR7309
CB4856	CT	II	8173680 8174866 WBGene00011466	T05C12.1	T05C12.1	-0.1186469398183
AGIWUR8960;AGIWUR11009;AGIWUR38979
CB4856	CT	II	8174845 8177195 WBGene00000053	acr-14	T05C12.2	0.235197435637587
AGIWUR222;AGIWUR34520;AGIWUR36830
CB4856	CT	II	8177578 8180381 WBGene00011468	T05C12.4	T05C12.4	0.0439022117753884
AGIWUR8536
CB4856	CT	II	8181737 8182309 WBGene00011469	dylt-3	T05C12.5	-0.198869947535974
AGIWUR19498
CB4856	CT	II	8182323 8185023 WBGene00003241	mig-5	T05C12.6	-0.0661192939819149
AGIWUR41036
CB4856	CT	II	8185443 8187601 WBGene00000377	cct-1	T05C12.7	-0.298274792834574
AGIWUR2764;AGIWUR11724;AGIWUR14998;AGIWUR15680;AGIWUR20879;AGIWUR28442;AGIWUR29277
CB4856	CT	II	8187607 8190528 WBGene00011471	T05C12.8	T05C12.8	0.31208285912609
AGIWUR13267
CB4856	CT	II	8191773 8193329 WBGene00011472	T05C12.9	T05C12.9	-0.0892583903893378
AGIWUR22375
CB4856	CT	II	8198803 8204001 WBGene00004264	qua-1	T05C12.10	0.0284395130305288
AGIWUR18808
CB4856	CT	II	8204341 8207491 WBGene00000652	col-76	M110.1	0.108057810564031	AGIWUR5673
CB4856	CT	II	8208000 8209444 WBGene00010916	M110.8	M110.8	0.0910535411940874	AGIWUR18001
CB4856	CT	II	8210662 8212751 WBGene00006658	twk-3	M110.2	-0.224494372376594	AGIWUR10521
CB4856	CT	II	8214575 8216616 WBGene00010913	M110.3	M110.3	-0.0646573937742065	AGIWUR23590
CB4856	CT	II	8217231 8222395 WBGene00002066	ifg-1	M110.4	-0.18706307193607
AGIWUR3842;AGIWUR23909;AGIWUR24055
CB4856	CT	II	8226132 8229122 WBGene00000894	dab-1	M110.5	-0.0668444908728976
AGIWUR2336;AGIWUR6350;AGIWUR30204;AGIWUR38810
CB4856	CT	II	8231239 8232769 WBGene00010917	M110.9	M110.9	-0.129255366093142	AGIWUR35202
CB4856	CT	II	8232789 8237202 WBGene00010915	M110.7	M110.7	-0.152122228733454	AGIWUR27439
CB4856	CT	II	8238455 8243747 WBGene00003743	nlp-5	F35C11.1	0.322605152582911
AGIWUR18693
CB4856	CT	II	8246776 8248263 WBGene00009402	F35C11.3	F35C11.3	-0.301953590407965

AGIWUR20539
CB4856	CT	II	8249879	8251980	WBGene00009403	F35C11.4	F35C11.4	-0.234444938772723
AGIWUR37143
CB4856	CT	II	8253740	8255319	WBGene00009404	F35C11.5	F35C11.5	0.139355829786805
AGIWUR44340
CB4856	CT	II	8256313	8257605	WBGene00009406	F35C11.7	F35C11.7	0.0550340680607873
AGIWUR7569
CB4856	CT	II	8258074	8259210	WBGene00009405	F35C11.6	F35C11.6	0.191518275042139
AGIWUR32766
CB4856	CT	II	8264661	8267360	WBGene00010456	K01C8.1	K01C8.1	-0.286446794390876	AGIWUR25064
CB4856	CT	II	8267253	8269914	WBGene00010457	K01C8.2	K01C8.2	0.00482071726198718
AGIWUR195;AGIWUR41552
CB4856	CT	II	8270567	8273611	WBGene00006562	tdc-1	K01C8.3	-0.104482132034164
AGIWUR2061;AGIWUR34794
CB4856	CT	II	8273543	8276231	WBGene00001571	gei-14	K01C8.5	-0.191588057400057	AGIWUR1841
CB4856	CT	II	8276298	8277611	WBGene00010459	slc-25A32	K01C8.7	-0.134346078754847
AGIWUR8565;AGIWUR13542;AGIWUR37798;AGIWUR41814;AGIWUR44670
CB4856	CT	II	8277531	8278515	WBGene00010458	mrpl-10	K01C8.6	0.136725149850415	AGIWUR38766
CB4856	CT	II	8278686	8281035	WBGene00010460	clec-142	K01C8.8	-0.126182628786442
AGIWUR9226;AGIWUR13203;AGIWUR43626
CB4856	CT	II	8280855	8283208	WBGene00003821	nst-1	K01C8.9	0.08514903670644	AGIWUR40653
CB4856	CT	II	8283667	8285711	WBGene00000379	cct-4	K01C8.10	0.0447681086391745
AGIWUR13181;AGIWUR32010
CB4856	CT	II	8294829	8295396	WBGene00008820	nlp-55	F14F11.2	0.56015490037605
AGIWUR1677
CB4856	CT	II	8305745	8321471	WBGene00008819	shw-1	F14F11.1	-0.0669552382935139
AGIWUR4986;AGIWUR9350;AGIWUR21060;AGIWUR29497;AGIWUR34911;AGIWUR42810;AGIWUR45012
CB4856	CT	II	8322612	8325052	WBGene00004304	ran-3	C26D10.1	0.115493425494257
AGIWUR40833
CB4856	CT	II	8325386	8329658	WBGene00007744	C26D10.3	C26D10.3	-0.201772835514978
AGIWUR19644
CB4856	CT	II	8329758	8333481	WBGene00007745	C26D10.4	C26D10.4	0.15060580477357
AGIWUR16715;AGIWUR19903;AGIWUR40329;AGIWUR43871
CB4856	CT	II	8334094	8336503	WBGene00023420	C26D10.7	C26D10.7	0.127496651479849
AGIWUR16828
CB4856	CT	II	8346690	8350557	WBGene00001159	eff-1	C26D10.5	0.0401804430997825
AGIWUR22682;AGIWUR29270
CB4856	CT	II	8350535	8354700	WBGene00007746	C26D10.6	C26D10.6	-0.332448417285505
AGIWUR25563

CB4856	CT	II	8358228	8359381	WBGene00000653	col-77	M195.1	-0.40717272422447	AGIWUR23505
CB4856	CT	II	8359712	8363196	WBGene00010948	M195.2	M195.2	-0.507561931727109	AGIWUR30560
CB4856	CT	II	8363196	8369159	WBGene00000135	amt-3	M195.3	-0.0627733201440321	AGIWUR26721
CB4856	CT	II	8376611	8377357	WBGene00010949	M195.4	M195.4	-0.303375731693904	AGIWUR139
CB4856	CT	II	8381497	8383905	WBGene00009904	F49E12.12	F49E12.12	0.118434513535442	AGIWUR41004
CB4856	CT	II	8388051	8389891	WBGene00009902	drd-1	F49E12.9	-0.139686951608141	AGIWUR3152;AGIWUR16685;AGIWUR28016;AGIWUR44394
CB4856	CT	II	8391088	8392281	WBGene00009903	F49E12.10	F49E12.10	0.696599592809967	AGIWUR8922
CB4856	CT	II	8392571	8394202	WBGene00009901	F49E12.8	F49E12.8	-0.139656567962318	AGIWUR7589
CB4856	CT	II	8395276	8396141	WBGene00009900	F49E12.7	F49E12.7	0.0357745662523721	AGIWUR21880
CB4856	CT	II	8398161	8399160	WBGene00009898	dod-23	F49E12.2	-0.143429658950373	AGIWUR23090
CB4856	CT	II	8400299	8404848	WBGene00009897	skpo-1	F49E12.1	-0.306441459353351	AGIWUR22459
CB4856	CT	II	8405603	8406924	WBGene00006719	ubc-24	F49E12.4	-0.265219829483556	AGIWUR30944
CB4856	CT	II	8407685	8409237	WBGene00005039	sra-13	F49E12.5	-0.0326981431400677	AGIWUR5312;AGIWUR21380;AGIWUR38003
CB4856	CT	II	8412671	8416857	WBGene00009899	efl-3	F49E12.6	0.667904279906851	AGIWUR13219;AGIWUR15145;AGIWUR32279;AGIWUR38296;AGIWUR43804;AGIWUR44927
CB4856	CT	II	8417618	8419758	WBGene00008799	F14E5.1	F14E5.1	-0.329960542453048	AGIWUR18246
CB4856	CT	II	8423951	8428855	WBGene00008800	F14E5.2	F14E5.2	-0.0966601634324658	AGIWUR3167;AGIWUR17171;AGIWUR35433
CB4856	CT	II	8429959	8432054	WBGene00008802	acp-2	F14E5.4	-0.448330830162509	AGIWUR23377
CB4856	CT	II	8432773	8435105	WBGene00008804	acp-4	F14E5.6	-0.233003972130508	AGIWUR21718
CB4856	CT	II	8435347	8437135	WBGene00008801	acp-3	F14E5.3	-0.0517932774056202	AGIWUR26233
CB4856	CT	II	8439118	8442151	WBGene00008803	lips-10	F14E5.5	0.106925594539714	AGIWUR4094
CB4856	CT	II	8442765	8444239	WBGene00001230	eif-3.G	F22B5.2	-0.0841861747746435	AGIWUR9270;AGIWUR19095;AGIWUR24859;AGIWUR29721;AGIWUR29987;AGIWUR38375
CB4856	CT	II	8444307	8445177	WBGene00001358	evl-20	F22B5.1	0.113466213356752	AGIWUR34115
CB4856	CT	II	8445168	8446831	WBGene00009041	cut-3	F22B5.3	1.48994325735261	AGIWUR30519
CB4856	CT	II	8449701	8452739	WBGene00009042	F22B5.4	F22B5.4	0.287755917655332	AGIWUR24088
CB4856	CT	II	8453254	8454613	WBGene00009043	F22B5.5	F22B5.5	-0.303207598631928	AGIWUR2516
CB4856	CT	II	8454612	8456194	WBGene00009044	F22B5.6	F22B5.6	0.0863501783001048	AGIWUR11320
CB4856	CT	II	8457818	8464517	WBGene00006994	zyg-9	F22B5.7	0.000709492827102309	AGIWUR36027

CB4856	CT	II	8464848	8467072	WBGene00001498	fars-3	F22B5.9	0.0331027441188638	AGIWUR34112
CB4856	CT	II	8467174	8468010	WBGene00009045	F22B5.10	F22B5.10	-0.0417467219738783	AGIWUR6122;AGIWUR11334;AGIWUR27711;AGIWUR28547
CB4856	CT	II	8468109	8470162	WBGene00010875	M05D6.2	M05D6.2	0.0781885820188961	AGIWUR31827
CB4856	CT	II	8470079	8471217	WBGene00010880	M05D6.8	M05D6.8	-0.184861729765128	AGIWUR15604
CB4856	CT	II	8471880	8473522	WBGene00044168	M05D6.10	M05D6.10	0.264348200453795	AGIWUR11206;AGIWUR35547
CB4856	CT	II	8473819	8475568	WBGene00010874	M05D6.1	M05D6.1	-0.176282893024487	AGIWUR35206
CB4856	CT	II	8478010	8480478	WBGene00010877	lact-4	M05D6.4	-0.244393798095832	AGIWUR13800;AGIWUR34952
CB4856	CT	II	8480457	8482216	WBGene00010878	M05D6.5	M05D6.5	-0.141062786452347	AGIWUR3221;AGIWUR10469;AGIWUR10734;AGIWUR41286
CB4856	CT	II	8482376	8483872	WBGene00010879	M05D6.6	M05D6.6	-0.0947601750406672	AGIWUR33291
CB4856	CT	II	8485879	8487571	WBGene00001523	gbh-2	M05D6.7	0.391162384704863	AGIWUR2809;AGIWUR14517;AGIWUR29356;AGIWUR29456;AGIWUR39469;AGIWUR39551
CB4856	CT	II	8487571	8509055	WBGene00011755	T13H5.1	T13H5.1	0.0457092208917445	AGIWUR6797;AGIWUR8690;AGIWUR15118;AGIWUR15740;AGIWUR16271;AGIWUR21225;AGIWUR27017;AGIWUR30143;AGIWUR33771
CB4856	CT	II	8514118	8516891	WBGene00011756	ctg-2	T13H5.2	-0.0451648316964741	AGIWUR9249;AGIWUR11176;AGIWUR25165;AGIWUR25634;AGIWUR26294;AGIWUR32840
CB4856	CT	II	8517150	8518412	WBGene00004383	rnh-2	T13H5.7	-0.170747203609167	AGIWUR12815
CB4856	CT	II	8518510	8520700	WBGene00011761	T13H5.8	T13H5.8	-0.0363844465905583	AGIWUR1284
CB4856	CT	II	8520905	8523490	WBGene00011757	cht-2	T13H5.3	-0.0694988003141397	AGIWUR8100
CB4856	CT	II	8523494	8525396	WBGene00011758	T13H5.4	T13H5.4	0.189012085976248	AGIWUR4164;AGIWUR25257;AGIWUR28277;AGIWUR39003
CB4856	CT	II	8525685	8526804	WBGene00011759	mrps-18B	T13H5.5	-0.10280468759975	AGIWUR26447
CB4856	CT	II	8526799	8531222	WBGene00011760	T13H5.6	T13H5.6	-0.180597920845388	AGIWUR15371
CB4856	CT	II	8533095	8534363	WBGene00012175	W01C9.1	W01C9.1	0.248870925790223	AGIWUR17469
CB4856	CT	II	8536133	8538603	WBGene00012176	W01C9.2	W01C9.2	-0.418798046615693	AGIWUR13147
CB4856	CT	II	8540720	8545452	WBGene00004155	pqn-73	W01C9.3	1.83980033277039	AGIWUR70
CB4856	CT	II	8545652	8546854	WBGene00012177	decr-1.2	W01C9.4	0.171419849648037	AGIWUR35156
CB4856	CT	II	8547339	8556258	WBGene00012178	glb-27	W01C9.5	-0.306146056719781	AGIWUR20195
CB4856	CT	II	8559394	8561352	WBGene00002065	iff-2	F54C9.1	-0.224993300120214	AGIWUR1041;AGIWUR13119;AGIWUR18172;AGIWUR31956;AGIWUR34294;AGIWUR35827;AGIWUR38745
CB4856	CT	II	8563714	8566139	WBGene00006059	stc-1	F54C9.2	-0.0813128845380415	AGIWUR27084
CB4856	CT	II	8566508	8566857	WBGene00010041	F54C9.3	F54C9.3	0.44097741297913	AGIWUR35414
CB4856	CT	II	8568035	8569089	WBGene00000615	col-38	F54C9.4	-0.625895386165906	AGIWUR17395
CB4856	CT	II	8569927	8571100	WBGene00004416	rpl-5	F54C9.5	0.0164623601471181	

AGIWUR9145;AGIWUR16011;AGIWUR23458
CB4856 CT II 8571085 8572904 WBGene00010042 bcs-1 F54C9.6 -0.187333340772603
AGIWUR6986;AGIWUR16748;AGIWUR26834;AGIWUR38862
CB4856 CT II 8573355 8575045 WBGene00010043 F54C9.7 F54C9.7 0.159464685558726
AGIWUR42579;AGIWUR43932
CB4856 CT II 8575541 8577732 WBGene00004241 puf-5 F54C9.8 -0.0730814493470362 AGIWUR17244
CB4856 CT II 8578049 8581513 WBGene00010044 F54C9.9 F54C9.9 -0.164343379232854
AGIWUR1419;AGIWUR4848;AGIWUR15723
CB4856 CT II 8581945 8583080 WBGene00000187 arl-1 F54C9.10 -0.103427229383737
AGIWUR21476
CB4856 CT II 8583034 8585086 WBGene00010045 F54C9.11 F54C9.11 0.202268796278423
AGIWUR34137
CB4856 CT II 8590984 8593010 WBGene00009202 aptf-4 F28C6.1 0.0337029288476955
AGIWUR10388;AGIWUR16446;AGIWUR39555
CB4856 CT II 8594190 8596294 WBGene00009203 aptf-3 F28C6.2 0.00660454861266579
AGIWUR12578;AGIWUR32441;AGIWUR42969
CB4856 CT II 8596286 8600265 WBGene00009204 F28C6.4 F28C6.4 0.0557828424827758
AGIWUR10920;AGIWUR17476;AGIWUR38808
CB4856 CT II 8599508 8600506 WBGene00009205 F28C6.5 F28C6.5 -0.100398367181061 AGIWUR22408
CB4856 CT II 8600435 8602479 WBGene00000773 cpf-1 F28C6.3 -0.147200506315237 AGIWUR30019
CB4856 CT II 8602787 8603581 WBGene00004440 rpl-26 F28C6.7 0.0428263995849658
AGIWUR22308;AGIWUR42774
CB4856 CT II 8603846 8606744 WBGene00006307 suf-1 F28C6.6 0.0168048635321707
AGIWUR15406;AGIWUR42122;AGIWUR43954
CB4856 CT II 8606849 8607749 WBGene00009207 F28C6.8 F28C6.8 -0.313812687708637 AGIWUR35865
CB4856 CT II 8607889 8608865 WBGene00009209 F28C6.10 F28C6.10 -0.446640547000614
AGIWUR30551
CB4856 CT II 8608876 8610110 WBGene00009208 F28C6.9 F28C6.9 -0.103967999867639 AGIWUR41437
CB4856 CT II 8612770 8614426 WBGene00011922 T22C8.1 T22C8.1 -0.167023096029947 AGIWUR25354
CB4856 CT II 8614546 8617603 WBGene00011923 chhy-1 T22C8.2 -0.408582237822626 AGIWUR21816
CB4856 CT II 8620397 8621978 WBGene00011924 T22C8.3 T22C8.3 -0.0940343006540818 AGIWUR1762
CB4856 CT II 8622447 8624254 WBGene00011925 T22C8.4 T22C8.4 -0.226528326048738
AGIWUR1196;AGIWUR12798;AGIWUR30886
CB4856 CT II 8625728 8627620 WBGene00011926 sptf-2 T22C8.5 -0.0421031654967798 AGIWUR21226
CB4856 CT II 8627573 8630239 WBGene00011927 T22C8.6 T22C8.6 0.519073370787243 AGIWUR8779
CB4856 CT II 8630710 8634464 WBGene00011928 cutl-12 T22C8.7 0.0210201161814161
AGIWUR11556;AGIWUR39206
CB4856 CT II 8636233 8637711 WBGene00006875 vab-9 T22C8.8 -0.220556487338343 AGIWUR25044
CB4856 CT II 8641381 8648227 WBGene00008432 D2085.7 D2085.7 -0.291386531080098 AGIWUR40175

CB4856	CT	II	8651109 8658808 WBGene00004259	pyr-1	D2085.1 -0.0552146735034715	AGIWUR22823;AGIWUR41113
CB4856	CT	II	8659135 8659800 WBGene00008427	atg-10	D2085.2 -0.0765918724689598	AGIWUR42128
CB4856	CT	II	8659782 8661712 WBGene00008431	piga-1	D2085.6 -0.186063989166417	AGIWUR5013
CB4856	CT	II	8661869 8664721 WBGene00008428	eif-2Bepsilon	D2085.3 -0.288151296195212	AGIWUR41224
CB4856	CT	II	8664833 8669154 WBGene00008429	D2085.4 D2085.4 -0.082386453518243	AGIWUR36593
CB4856	CT	II	8669133 8684897 WBGene00008430	hgap-2	D2085.5 0.0485686755328638	AGIWUR23447;AGIWUR45090
CB4856	CT	II	8691789 8694180 WBGene00007916	C34C6.3 C34C6.3 0.787179091886938	AGIWUR9910
CB4856	CT	II	8696591 8698928 WBGene00007917	C34C6.4 C34C6.4 -0.0918742782172526	AGIWUR465
CB4856	CT	II	8699045 8702193 WBGene00007918	sphk-1	C34C6.5 -0.0204817961685633	AGIWUR4466;AGIWUR18165;AGIWUR24595;AGIWUR30041
CB4856	CT	II	8703368 8703829 WBGene00000432	ceh-7	C34C6.8 0.224355496659337	AGIWUR28771
CB4856	CT	II	8704824 8706768 WBGene00004194	prx-5	C34C6.6 0.146355348136233	AGIWUR1248;AGIWUR5786;AGIWUR10847;AGIWUR40056
CB4856	CT	II	8707155 8708860 WBGene00007919	C34C6.7 C34C6.7 2.16331197139565	AGIWUR29170
CB4856	CT	II	8709069 8710413 WBGene00005172	srg-15	C34C6.1 0.0195115665026595	AGIWUR33606
CB4856	CT	II	8711589 8712035 WBGene00014827	T01B7.1 T01B7.1 0.0206642821282284	AGIWUR3554;AGIWUR8918;AGIWUR20861;AGIWUR27845;AGIWUR35574
CB4856	CT	II	8712068 8713412 WBGene00005175	srg-18	T01B7.2 -0.331732617088994	AGIWUR21000
CB4856	CT	II	8713992 8715013 WBGene00004279	rab-21	T01B7.3 -0.139780278564025	AGIWUR4900
CB4856	CT	II	8715132 8716281 WBGene00000887	cyn-11	T01B7.4 0.00232931370270251	AGIWUR26435
CB4856	CT	II	8716834 8721691 WBGene00011311	T01B7.5 T01B7.5 -0.0132352349255491	AGIWUR20070;AGIWUR27309;AGIWUR31333;AGIWUR43034
CB4856	CT	II	8722474 8725522 WBGene00011312	trcs-2	T01B7.6 -0.00289735128052371	AGIWUR22900
CB4856	CT	II	8725533 8729777 WBGene00011314	T01B7.9 T01B7.9 0.168829682021022	AGIWUR6767
CB4856	CT	II	8733050 8734314 WBGene00004397	rol-6	T01B7.7 -0.217023519716941	AGIWUR25131
CB4856	CT	II	8736726 8737556 WBGene00011313	T01B7.8 T01B7.8 -0.270847785245576	AGIWUR14303
CB4856	CT	II	8743725 8747234 WBGene00010681	mak-1	K08F8.1 0.429346575777007	AGIWUR4577;AGIWUR13470;AGIWUR14854;AGIWUR18734;AGIWUR19560;AGIWUR20333;AGIWUR20930;AGIWUR21944;AGIWUR22982;AGIWUR25100;AGIWUR30687;AGIWUR31970;AGIWUR32850;AGIWUR34735;AGIWUR37918;AGIWUR40571;AGIWUR42916
CB4856	CT	II	8751986 8753939 WBGene00000220	atf-2	K08F8.2 1.18335178634244	AGIWUR24767
CB4856	CT	II	8754113 8755248 WBGene00010683	K08F8.7 K08F8.7 0.133771779874772	AGIWUR32053
CB4856	CT	II	8756291 8758723 WBGene00001505	fut-1	K08F8.3 -0.0640805459172178	AGIWUR29855
CB4856	CT	II	8762668 8766922 WBGene00000240	pah-1	K08F8.4 -0.426572733790336	AGIWUR3575;AGIWUR4523;AGIWUR22284;AGIWUR29197;AGIWUR45094
CB4856	CT	II	8767185 8769307 WBGene00010682	K08F8.5 K08F8.5 -0.318087056359932	AGIWUR39492
CB4856	CT	II	8770975 8782020 WBGene00002295	let-19	K08F8.6 0.100176831289569

									AGIWUR4540;AGIWUR10212;AGIWUR18372;AGIWUR25471;AGIWUR28734;AGIWUR42522
CB4856	CT	II	8782029	8783670	WBGene00004790	sgn-1	F07H5.2	-0.0987032688015077	AGIWUR29530
CB4856	CT	II	8784535	8785513	WBGene00014307	F07H5.3	F07H5.3	-0.0781542111772549	AGIWUR41665
CB4856	CT	II	8786457	8787119	WBGene00014308	F07H5.4	F07H5.4	0.500588783129331	AGIWUR18538
CB4856	CT	II	8787770	8789716	WBGene00014309	F07H5.5	F07H5.5	0.387875905786171	AGIWUR17372;AGIWUR24243;AGIWUR28524;AGIWUR32492;AGIWUR39338;AGIWUR44699
CB4856	CT	II	8790558	8791318	WBGene00008563	F07H5.13	F07H5.13	0.807221691325029	AGIWUR7414;AGIWUR39817
CB4856	CT	II	8791927	8792740	WBGene00008557	F07H5.6	F07H5.6	-0.207733556071314	AGIWUR4059;AGIWUR10978;AGIWUR39366
CB4856	CT	II	8792912	8794162	WBGene00008558	F07H5.7	F07H5.7	-0.0939744779576674	AGIWUR34161
CB4856	CT	II	8801130	8804889	WBGene00008559	F07H5.8	F07H5.8	-0.163231089000488	AGIWUR9599;AGIWUR12195;AGIWUR14389;AGIWUR26173;AGIWUR29115
CB4856	CT	II	8804835	8807021	WBGene00008560	pho-13	F07H5.9	-0.887799132140714	AGIWUR21693
CB4856	CT	II	8807133	8808582	WBGene00008561	F07H5.10	F07H5.10	-0.01938262494809	AGIWUR28131
CB4856	CT	II	8808636	8810729	WBGene00009784	F46C5.9	F46C5.9	0.216038267894495	AGIWUR9698
CB4856	CT	II	8811353	8812633	WBGene00009783	rer-1	F46C5.8	-0.162590162664713	AGIWUR2840;AGIWUR4345;AGIWUR39178
CB4856	CT	II	8817593	8818426	WBGene00009785	F46C5.10	F46C5.10	0.0410612939376023	AGIWUR30007
CB4856	CT	II	8820149	8821248	WBGene00009778	F46C5.1	F46C5.1	5.75103865089278	AGIWUR19741
CB4856	CT	II	8826069	8828215	WBGene00003544	nas-25	F46C5.3	0.0985634352349876	AGIWUR2053;AGIWUR14007;AGIWUR15314;AGIWUR18326;AGIWUR22006;AGIWUR25476
CB4856	CT	II	8828196	8829287	WBGene00009779	F46C5.2	F46C5.2	0.146838482571045	AGIWUR36211
CB4856	CT	II	8836175	8839313	WBGene00009780	F46C5.4	F46C5.4	-0.0521349365374757	AGIWUR4321
CB4856	CT	II	8839325	8840243	WBGene00009782	F46C5.7	F46C5.7	-0.0132265084699128	AGIWUR12915
CB4856	CT	II	8840200	8844515	WBGene00009781	F46C5.6	F46C5.6	-0.206160199070267	AGIWUR15946
CB4856	CT	II	8845598	8848902	WBGene00011767	agxt-1	T14D7.1	0.0521957626025369	AGIWUR23608;AGIWUR43545
CB4856	CT	II	8848843	8852096	WBGene00011768	oac-46	T14D7.2	0.20248358716078	AGIWUR22936;AGIWUR29185;AGIWUR31496;AGIWUR34912;AGIWUR35792;AGIWUR37935
CB4856	CT	II	8859074	8859696	WBGene00044062	snb-6	T14D7.3	0.294221012842762	AGIWUR10821
CB4856	CT	II	8860913	8865173	WBGene00011578	npr-20	T07D4.1	0.233334760013316	AGIWUR14786
CB4856	CT	II	8869549	8874020	WBGene00011579	T07D4.2	T07D4.2	0.17471227409022	AGIWUR34616
CB4856	CT	II	8878496	8883659	WBGene00004355	rha-1	T07D4.3	-0.117828497242794	AGIWUR8834
CB4856	CT	II	8883763	8884418	WBGene00045407	T07D4.5	T07D4.5	-0.295947723130628	AGIWUR2542;AGIWUR42808
CB4856	CT	II	8884901	8889673	WBGene00011580	ddx-19	T07D4.4	-0.15303832305786	

AGIWUR1657;AGIWUR3284;AGIWUR7547
CB4856 CT II 8896841 8908666 WBGene00002251 lat-1 B0457.1 -0.0310661759104729
AGIWUR4436;AGIWUR10719;AGIWUR13483;AGIWUR16585;AGIWUR16626;AGIWUR21725;AGIWUR23036;AGIWUR29098;AGIWUR29801;
AGIWUR36610;AGIWUR38597;AGIWUR44924
CB4856 CT II 8909008 8909994 WBGene00014660 B0457.3 B0457.3 -0.0442383401636239
AGIWUR20307;AGIWUR37158
CB4856 CT II 8910913 8912029 WBGene00007179 B0457.4 B0457.4 -0.0719346425412555 AGIWUR5875
CB4856 CT II 8922423 8924373 WBGene00007180 B0457.6 B0457.6 -0.166481523292056 AGIWUR30915
CB4856 CT II 8924572 8926678 WBGene00007178 B0457.2 B0457.2 0.0898067363210693 AGIWUR32452
CB4856 CT II 8927741 8929820 WBGene00000378 cct-2 T21B10.7 -0.243034692408413
AGIWUR1976;AGIWUR8551;AGIWUR9167;AGIWUR14051;AGIWUR22008;AGIWUR22119;AGIWUR23132;AGIWUR25480;AGIWUR29685;AG
IWUR30090;AGIWUR34863;AGIWUR35363;AGIWUR40687;AGIWUR42308;AGIWUR42836
CB4856 CT II 8929841 8930937 WBGene00011883 mrpl-50 T21B10.1 -0.515297532747756
AGIWUR14079
CB4856 CT II 8930985 8933833 WBGene00011884 enol-1 T21B10.2 -0.0675065661123746
AGIWUR5548;AGIWUR7435;AGIWUR9458;AGIWUR12492;AGIWUR12639;AGIWUR30121;AGIWUR31823
CB4856 CT II 8934382 8939033 WBGene00011885 T21B10.3 T21B10.3 0.0310692666483501
AGIWUR25642
CB4856 CT II 8939204 8940488 WBGene00011887 set-17 T21B10.5 -0.093919963499058
AGIWUR5839
CB4856 CT II 8940894 8942718 WBGene00011886 T21B10.4 T21B10.4 0.00456950503994236
AGIWUR40409
CB4856 CT II 8942674 8947688 WBGene00011888 cutl-15 T21B10.6 0.0375539747636209
AGIWUR8379;AGIWUR37786
CB4856 CT II 8955240 8964149 WBGene00007690 sftd-3 C18E9.10 0.0875069573418266
AGIWUR39676
CB4856 CT II 8964238 8964901 WBGene00007684 C18E9.4 C18E9.4 -0.309730900497735 AGIWUR2027
CB4856 CT II 8965006 8966919 WBGene00007683 C18E9.2 C18E9.2 -0.520790811803314 AGIWUR19427
CB4856 CT II 8966921 8969473 WBGene00004105 szy-20 C18E9.3 -0.270000136414033 AGIWUR232
CB4856 CT II 8969980 8971967 WBGene00007685 C18E9.5 C18E9.5 -0.555521185971848 AGIWUR9657
CB4856 CT II 8971965 8973566 WBGene00007686 tomm-40 C18E9.6 -0.133914648076285 AGIWUR15552
CB4856 CT II 8978475 8982678 WBGene00007687 C18E9.7 C18E9.7 -0.133432343667465 AGIWUR29598
CB4856 CT II 8985900 8988062 WBGene00007688 C18E9.8 C18E9.8 -0.139461267046305 AGIWUR27516
CB4856 CT II 8988359 8989544 WBGene00007689 C18E9.9 C18E9.9 0.0901145920565849 AGIWUR34240
CB4856 CT II 8993169 8994533 WBGene00003870 ooc-5 C18E9.11 -0.10154578985567
AGIWUR34657
CB4856 CT II 8994636 8996269 WBGene00009711 F44G4.1 F44G4.1 -0.0624149309073552 AGIWUR36515
CB4856 CT II 8996364 8997037 WBGene00009712 F44G4.2 F44G4.2 -0.0510557575355648
AGIWUR26656;AGIWUR29476;AGIWUR37899

CB4856	CT	II	8997027	8997662	WBGene00009713	F44G4.3	F44G4.3	-0.305938176222082	AGIWUR39850
CB4856	CT	II	8997827	8999722	WBGene00006514	tdp-1	F44G4.4	-0.0916903350191136	AGIWUR6241;AGIWUR23305;AGIWUR31169;AGIWUR40138;AGIWUR43127;AGIWUR43210
CB4856	CT	II	8999727	9000431	WBGene00009714	F44G4.5	F44G4.5	0.231658245639387	AGIWUR28065
CB4856	CT	II	9000872	9001793	WBGene00009715	F44G4.6	F44G4.6	-0.110766408912465	AGIWUR23769
CB4856	CT	II	9002527	9005167	WBGene00009716	F44G4.7	F44G4.7	0.167854770542527	AGIWUR41942
CB4856	CT	II	9005796	9019775	WBGene00009717	dep-1	F44G4.8	0.464681592929534	AGIWUR4980;AGIWUR14304;AGIWUR27995
CB4856	CT	II	9026968	9029263	WBGene00009504	F37B12.1	F37B12.1	0.00596581065751423	AGIWUR28129
CB4856	CT	II	9035562	9038301	WBGene00001527	gcs-1	F37B12.2	-0.159209885535451	AGIWUR36032;AGIWUR39622
CB4856	CT	II	9039820	9041406	WBGene00009505	F37B12.3	F37B12.3	-0.0708633725461763	AGIWUR6571
CB4856	CT	II	9042180	9053824	WBGene00011980	T24B8.7	T24B8.7	-0.142067329211564	AGIWUR3801;AGIWUR7738;AGIWUR38682
CB4856	CT	II	9054972	9056504	WBGene00011976	T24B8.2	T24B8.2	0.330424919369918	AGIWUR38510
CB4856	CT	II	9056499	9057111	WBGene00004446	rpl-32	T24B8.1	-0.0268485206179383	AGIWUR17408;AGIWUR18356;AGIWUR19434;AGIWUR19524;AGIWUR26213;AGIWUR26733;AGIWUR34869;AGIWUR36838
CB4856	CT	II	9059389	9061138	WBGene00011977	T24B8.3	T24B8.3	0.243120169963777	AGIWUR8853;AGIWUR15171;AGIWUR15912;AGIWUR22651;AGIWUR28452;AGIWUR38847
CB4856	CT	II	9063687	9070904	WBGene00011978	T24B8.4	T24B8.4	0.174605419372091	AGIWUR11315;AGIWUR11955;AGIWUR12228;AGIWUR35707
CB4856	CT	II	9082626	9083146	WBGene00011979	T24B8.5	T24B8.5	-0.738032263262415	AGIWUR4785;AGIWUR5986;AGIWUR16640;AGIWUR24375;AGIWUR34447;AGIWUR40012
CB4856	CT	II	9086608	9087917	WBGene00001950	hlh-3	T24B8.6	0.341589521942197	AGIWUR14966
CB4856	CT	II	9091109	9093696	WBGene00007421	C07H4.1	C07H4.1	-0.390375450866358	AGIWUR20829
CB4856	CT	II	9094710	9098605	WBGene00000532	clh-5	C07H4.2	0.128757170235346	AGIWUR9749
CB4856	CT	II	9099371	9100639	WBGene00012000	T24H10.1	T24H10.1	-0.133630430152476	AGIWUR27507
CB4856	CT	II	9101098	9105451	WBGene00012003	T24H10.5	T24H10.5	0.180128489144053	AGIWUR7016
CB4856	CT	II	9106515	9107145	WBGene00012004	dyrb-1	T24H10.6	-0.112628883188655	AGIWUR14292
CB4856	CT	II	9107382	9108600	WBGene00001041	dnj-23	T24H10.3	-0.00240997483164662	AGIWUR28826
CB4856	CT	II	9109534	9111240	WBGene00012002	T24H10.4	T24H10.4	0.0356364379991572	AGIWUR39308
CB4856	CT	II	9113219	9132213	WBGene00012005	jun-1	T24H10.7	-0.0239221613320515	

AGIWUR15041;AGIWUR15175;AGIWUR39207;AGIWUR43228
CB4856 CT II 9131663 9134543 WBGene00007633 C16D2.1 C16D2.1 0.132453930260589
AGIWUR3556;AGIWUR11436;AGIWUR25167;AGIWUR25792;AGIWUR35378;AGIWUR43292
CB4856 CT II 9139651 9145352 WBGene00012421 Y9C2UA.1 Y9C2UA.1 0.264110176823669
AGIWUR28136;AGIWUR44101
CB4856 CT II 9146261 9146641 WBGene00012422 Y9C2UA.2 Y9C2UA.2 -0.0317710214763682
AGIWUR34874
CB4856 CT II 9148356 9152311 WBGene00012322 W07A12.4 W07A12.4 0.0333454275765384
AGIWUR14184
CB4856 CT II 9152531 9154310 WBGene00000654 col-78 W07A12.5 -0.179900876678156
AGIWUR17535
CB4856 CT II 9158108 9159874 WBGene00012323 oac-54 W07A12.6 -0.334135754924986
AGIWUR12447
CB4856 CT II 9161379 9163448 WBGene00012324 rhy-1 W07A12.7 -0.464678813494519
AGIWUR26959
CB4856 CT II 9163368 9165800 WBGene00012325 W07A12.8 W07A12.8 -0.79474779533335
AGIWUR12569
CB4856 CT II 9165929 9167830 WBGene00011966 T23G7.3 T23G7.3 0.183725273903569 AGIWUR30241
CB4856 CT II 9167933 9172991 WBGene00011965 T23G7.2 T23G7.2 0.0542672966090372 AGIWUR12448
CB4856 CT II 9173175 9177152 WBGene00004752 sec-5 T23G7.4 -0.0877219176436626 AGIWUR6458
CB4856 CT II 9177300 9178585 WBGene00011967 pir-1 T23G7.5 -0.0119777196563184
AGIWUR12789;AGIWUR28676;AGIWUR35975
CB4856 CT II 9178514 9188480 WBGene00006367 sym-2 ZK1067.6 0.263393936813921
AGIWUR16239
CB4856 CT II 9197518 9207210 WBGene00002299 let-23 ZK1067.1 0.269578408698784
AGIWUR38958
CB4856 CT II 9210376 9213100 WBGene00014210 ZK1067.4 ZK1067.4 -0.148900483459515
AGIWUR18196
CB4856 CT II 9213658 9222798 WBGene00014208 ZK1067.2 ZK1067.2 -0.233537207597936
AGIWUR280;AGIWUR30742;AGIWUR36429;AGIWUR38565
CB4856 CT II 9223269 9225176 WBGene00014209 ZK1067.3 ZK1067.3 -0.122306081589183
AGIWUR11545
CB4856 CT II 9225186 9227261 WBGene00004174 abu-14 ZK1067.7 -0.0774412430626779
AGIWUR14252;AGIWUR36345;AGIWUR43716
CB4856 CT II 9231457 9232340 WBGene00001761 gst-13 T26C5.1 -0.405680255245793 AGIWUR6051
CB4856 CT II 9233223 9236202 WBGene00012032 T26C5.2 T26C5.2 0.298237933840935
AGIWUR22340;AGIWUR30572;AGIWUR39818
CB4856 CT II 9236583 9249123 WBGene00012033 T26C5.3 T26C5.3 0.0677078675256784 AGIWUR43937
CB4856 CT II 9249048 9250522 WBGene00012034 T26C5.4 T26C5.4 0.232351203800871 AGIWUR5499

CB4856 CT II 9251788 9254312 WBGene00044006 H32K21.1 H32K21.1 0.224006300234066 AGIWUR9374;AGIWUR14634;AGIWUR29552;AGIWUR32187;AGIWUR33749
CB4856 CT II 9258913 9261994 WBGene00000170 aqp-2 C01G6.1 1.18952880981641 AGIWUR6503;AGIWUR16119;AGIWUR16505;AGIWUR16669;AGIWUR38863;AGIWUR42186;AGIWUR43505
CB4856 CT II 9267336 9268303 WBGene00007224 C01G6.2 C01G6.2 -0.406088197498356 AGIWUR904
CB4856 CT II 9269888 9271267 WBGene00007225 C01G6.3 C01G6.3 0.703627963828103 AGIWUR10148
CB4856 CT II 9271265 9273042 WBGene00007226 C01G6.4 C01G6.4 0.134904083670236 AGIWUR4309
CB4856 CT II 9275024 9280098 WBGene00007227 C01G6.5 C01G6.5 0.0155009516672306 AGIWUR7512;AGIWUR15656;AGIWUR20663;AGIWUR34921
CB4856 CT II 9285932 9289136 WBGene00006510 mtrr-1 C01G6.6 -0.304716570515659 AGIWUR4599;AGIWUR5472;AGIWUR6166;AGIWUR8083;AGIWUR10918;AGIWUR36993
CB4856 CT II 9289506 9291539 WBGene00014663 C01G6.10 C01G6.10 -0.17092399975022 AGIWUR10768;AGIWUR33227
CB4856 CT II 9292017 9294050 WBGene00007228 acs-7 C01G6.7 0.177875396295684 AGIWUR16134;AGIWUR20204;AGIWUR41951
CB4856 CT II 9294029 9295753 WBGene00007229 C01G6.9 C01G6.9 0.780282076477057 AGIWUR9968
CB4856 CT II 9295918 9310304 WBGene00000289 cam-1 C01G6.8 0.185098169775369 AGIWUR22200
CB4856 CT II 9317551 9319627 WBGene00008403 csa-1 D2013.3 -0.250775697509816 AGIWUR20055
CB4856 CT II 9319865 9321888 WBGene00008402 wdfy-2 D2013.2 -0.156453962107001 AGIWUR11794
CB4856 CT II 9321858 9322916 WBGene00004286 rab-39 D2013.1 -0.00419200376854256 AGIWUR37896
CB4856 CT II 9323955 9324873 WBGene00006520 tag-175 D2013.10 -0.0217967671089063 AGIWUR7498;AGIWUR13845;AGIWUR37776
CB4856 CT II 9324874 9329665 WBGene00001134 eat-3 D2013.5 -0.344920525574132 AGIWUR5032;AGIWUR9059;AGIWUR14969
CB4856 CT II 9329757 9331510 WBGene00008404 D2013.6 D2013.6 0.224040098643528 AGIWUR9103
CB4856 CT II 9331643 9333925 WBGene00001229 eif-3.F D2013.7 -0.283591671551143 AGIWUR26638
CB4856 CT II 9334014 9338896 WBGene00004744 scp-1 D2013.8 -0.0983549821623253 AGIWUR31494
CB4856 CT II 9338890 9341394 WBGene00008405 ttll-12 D2013.9 0.0180609002671339 AGIWUR4157;AGIWUR10715;AGIWUR42764
CB4856 CT II 9341397 9346590 WBGene00009626 F42A8.1 F42A8.1 -0.673053095019888 AGIWUR629
CB4856 CT II 9350648 9352801 WBGene00006433 sdhb-1 F42A8.2 0.0900390743756923 AGIWUR10676;AGIWUR41853
CB4856 CT II 9353000 9354327 WBGene00009627 F42A8.3 F42A8.3 -0.266452610282165 AGIWUR6749
CB4856 CT II 9358531 9369098 WBGene00003196 mel-11 C06C3.1 0.0332911555282234 AGIWUR488;AGIWUR8877;AGIWUR12482;AGIWUR18840;AGIWUR22152;AGIWUR27473;AGIWUR28945;AGIWUR31426;AGIWUR32983;AGIWUR35345;AGIWUR41789
CB4856 CT II 9369178 9372485 WBGene00007374 C06C3.3 C06C3.3 0.21040237783724 AGIWUR42015
CB4856 CT II 9375845 9377258 WBGene00044169 C06C3.10 C06C3.10 -0.187769804287017 AGIWUR7373;AGIWUR19914;AGIWUR21399;AGIWUR24535;AGIWUR35394;AGIWUR42353;AGIWUR44149

CB4856 CT II 9378557 9379596 WBGene00045404 C06C3.11 C06C3.11 -0.380112314400556
AGIWUR14892;AGIWUR22768
CB4856 CT II 9380106 9381355 WBGene00007376 C06C3.5 C06C3.5 -0.306143249773133
AGIWUR7789;AGIWUR43989
CB4856 CT II 9380822 9384995 WBGene00005271 srh-48 C06C3.6 0.0530426249626945
AGIWUR777;AGIWUR30993;AGIWUR41635
CB4856 CT II 9385541 9386537 WBGene00007377 C06C3.7 C06C3.7 -0.0839249911527435 AGIWUR9039
CB4856 CT II 9386584 9390900 WBGene00007378 C06C3.8 C06C3.8 0.560631321954553 AGIWUR24997
CB4856 CT II 9395493 9397596 WBGene00013091 Y51B9A.9 Y51B9A.9 0.671941949004276
AGIWUR15463
CB4856 CT II 9398866 9399828 WBGene00013090 Y51B9A.8 Y51B9A.8 0.159080976846226
AGIWUR38154
CB4856 CT II 9400053 9400567 WBGene00013089 Y51B9A.7 Y51B9A.7
0.000114448694899849 AGIWUR18686
CB4856 CT II 9400717 9403187 WBGene00013088 Y51B9A.6 Y51B9A.6 0.0977577015916467
AGIWUR41585
CB4856 CT II 9404146 9404779 WBGene00013087 Y51B9A.5 Y51B9A.5 0.0701785304744611
AGIWUR8521
CB4856 CT II 9405539 9407114 WBGene00013085 mpz-6 Y51B9A.3 0.0211745197588016
AGIWUR30524
CB4856 CT II 9407270 9407547 WBGene00014909 Y51B9A.2 Y51B9A.2 -0.188733631861202
AGIWUR27725
CB4856 CT II 9407769 9409017 WBGene00013086 arrd-2 Y51B9A.4 -0.143556444520073
AGIWUR1161;AGIWUR16056;AGIWUR19379
CB4856 CT II 9409341 9411597 WBGene00014813 M176.9 M176.9 -0.0803976041279917
AGIWUR5800;AGIWUR17079;AGIWUR29430;AGIWUR30418;AGIWUR33537;AGIWUR38628;AGIWUR42401
CB4856 CT II 9412565 9414144 WBGene00006429 arrd-3 M176.1 -0.634883329972665 AGIWUR39634
CB4856 CT II 9414559 9416431 WBGene00010941 gss-1 M176.2 -0.179202439364856
AGIWUR3744;AGIWUR7901;AGIWUR17536;AGIWUR39771
CB4856 CT II 9417013 9418316 WBGene00010942 chch-3 M176.3 -0.0143443225182835
AGIWUR14876;AGIWUR32165
CB4856 CT II 9420178 9422196 WBGene00010943 M176.4 M176.4 0.449657899077268 AGIWUR16670
CB4856 CT II 9422269 9423296 WBGene00010947 M176.11 M176.11 -0.389428091799003 AGIWUR6138
CB4856 CT II 9423331 9426173 WBGene00010946 M176.10 M176.10 -0.00299425076349458 AGIWUR8173
CB4856 CT II 9426229 9427744 WBGene00010944 M176.5 M176.5 -0.140937546483254 AGIWUR437
CB4856 CT II 9431383 9433709 WBGene00002199 kin-15 M176.6 -0.830242147364668 AGIWUR9251
CB4856 CT II 9434021 9436120 WBGene00002200 kin-16 M176.7 -0.137845734175526 AGIWUR37351
CB4856 CT II 9436649 9438327 WBGene00010945 chil-13 M176.8 -0.0459583330217317
AGIWUR14242;AGIWUR18118;AGIWUR20828;AGIWUR22939;AGIWUR39368

CB4856 CT II 9438609 9440160 WBGene00011159 chil-17 R09D1.3 0.0581729490039216 AGIWUR9661
CB4856 CT II 9440441 9441959 WBGene00011158 chil-16 R09D1.2 0.0648244800376432 AGIWUR12002
CB4856 CT II 9442168 9443629 WBGene00011157 chil-15 R09D1.1 0.184580232484664 AGIWUR22130
CB4856 CT II 9443827 9445468 WBGene00011169 R09D1.13 R09D1.13 -0.202199929480916
AGIWUR9592
CB4856 CT II 9446090 9447705 WBGene00011160 R09D1.4 R09D1.4 0.22017425057087 AGIWUR21760
CB4856 CT II 9448273 9449726 WBGene00011161 chil-18 R09D1.5 0.192801671801053 AGIWUR17840
CB4856 CT II 9450028 9452263 WBGene00011168 R09D1.12 R09D1.12 0.0681779907342652
AGIWUR2982;AGIWUR9608;AGIWUR10424;AGIWUR16494
CB4856 CT II 9455417 9456617 WBGene00011163 chil-20 R09D1.7 0.00438647673367143 AGIWUR41221
CB4856 CT II 9462537 9464093 WBGene00011165 R09D1.9 R09D1.9 -0.156407372330615
AGIWUR13346;AGIWUR21482;AGIWUR21719;AGIWUR24513
CB4856 CT II 9464470 9466195 WBGene00011166 chil-22 R09D1.10 0.000572242079774909
AGIWUR31774
CB4856 CT II 9466960 9468477 WBGene00011167 chil-23 R09D1.11 0.458556529372663
AGIWUR22536
CB4856 CT II 9469119 9470082 WBGene00044807 T19H5.6 T19H5.6 0.610755705728327 AGIWUR34993
CB4856 CT II 9470521 9471924 WBGene00044806 T19H5.5 T19H5.5 0.0405448815540455 AGIWUR44827
CB4856 CT II 9472691 9474467 WBGene00011846 chil-25 T19H5.1 -0.152310207996719
AGIWUR41436;AGIWUR41503
CB4856 CT II 9475055 9476375 WBGene00011847 chil-26 T19H5.2 0.18137823519305 AGIWUR15275
CB4856 CT II 9477867 9479536 WBGene00011848 chil-27 T19H5.3 -0.241274934533485 AGIWUR1801
CB4856 CT II 9479641 9480106 WBGene00045414 T19H5.7 T19H5.7 -0.156685383114791
AGIWUR267;AGIWUR4245;AGIWUR12414;AGIWUR16867;AGIWUR18186;AGIWUR18396;AGIWUR19091;AGIWUR23500;AGIWUR26261;AG
IWUR36288
CB4856 CT II 9486377 9488260 WBGene00011849 T19H5.4 T19H5.4 0.807536955158259 AGIWUR33194
CB4856 CT II 9502757 9507281 WBGene00001530 gcy-3 R134.1 -0.0408547751936119 AGIWUR20905
CB4856 CT II 9507604 9512095 WBGene00001529 gcy-2 R134.2 0.194724489796297 AGIWUR21956
CB4856 CT II 9512490 9516936 WBGene00001528 gcy-1 AH6.1 0.260842328570684 AGIWUR34842
CB4856 CT II 9517567 9518876 WBGene00007080 sfxn-1.1 AH6.2 -0.203749188099923
AGIWUR30366
CB4856 CT II 9519055 9520205 WBGene00007081 AH6.3 AH6.3 0.0407244547949368 AGIWUR10180
CB4856 CT II 9520200 9521670 WBGene00005027 sra-1 AH6.4 0.107870838423366 AGIWUR40761
CB4856 CT II 9524174 9526390 WBGene00003231 mex-6 AH6.5 0.0503980856305652 AGIWUR26335
CB4856 CT II 9527031 9528253 WBGene00005041 sra-15 AH6.16 0.0566840052627775
AGIWUR9384;AGIWUR33043;AGIWUR38123
CB4856 CT II 9528780 9529862 WBGene00005028 sra-2 AH6.6 -0.0684170252177836 AGIWUR45064
CB4856 CT II 9530517 9531253 WBGene00005042 sra-16 AH6.13 -0.0970113442561029
AGIWUR12292;AGIWUR13397;AGIWUR19197;AGIWUR27224;AGIWUR30160;AGIWUR39965;AGIWUR43396;AGIWUR44805

CB4856	CT	II	9532299	9533974	WBGene00005035	sra-9	AH6.14	-0.79634081362544	AGIWUR20488
CB4856	CT	II	9537729	9539085	WBGene00005034	sra-8	AH6.12	-0.0555638842899535	AGIWUR26993
CB4856	CT	II	9539274	9540823	WBGene00005029	sra-3	AH6.7	-0.0879004408960462	AGIWUR22844
CB4856	CT	II	9541601	9542885	WBGene00005030	sra-4	AH6.8	0.0542726651620029	AGIWUR22321
CB4856	CT	II	9544686	9546208	WBGene00005033	sra-7	AH6.11	-0.565947040074713	AGIWUR39696
CB4856	CT	II	9546438	9547647	WBGene00005031	sra-5	AH6.9	-0.081062818657379	AGIWUR15593;AGIWUR21584
CB4856	CT	II	9548405	9549698	WBGene00005032	sra-6	AH6.10	0.00104016084499126	AGIWUR7915
CB4856	CT	II	9552631	9553279	WBGene00009446	F35H8.1	F35H8.1	0.173085345358563	AGIWUR10695;AGIWUR11849;AGIWUR16382
CB4856	CT	II	9555503	9557532	WBGene00009447	F35H8.2	F35H8.2	-0.094254675775737	AGIWUR43323
CB4856	CT	II	9557646	9559516	WBGene00009448	zfp-2	F35H8.3	0.0213573289556273	AGIWUR44357
CB4856	CT	II	9559497	9560029	WBGene00009449	F35H8.4	F35H8.4	0.161106647744997	AGIWUR31921
CB4856	CT	II	9575971	9581490	WBGene00001368	exc-7	F35H8.5	0.39593869604073	AGIWUR723;AGIWUR8197;AGIWUR12981;AGIWUR13353;AGIWUR20665;AGIWUR38098
CB4856	CT	II	9581756	9583548	WBGene00006938	wee-1.1	F35H8.7	0.0948853111983974	AGIWUR8384;AGIWUR24870;AGIWUR25452
CB4856	CT	II	9584384	9586815	WBGene00009450	ugt-58	F35H8.6	0.186828570452954	AGIWUR29447
CB4856	CT	II	9588212	9589767	WBGene00008456	E02H1.2	E02H1.2	-0.456158471129973	AGIWUR5067
CB4856	CT	II	9589865	9591221	WBGene00008455	E02H1.1	E02H1.1	-0.100176991722233	AGIWUR4770;AGIWUR6968;AGIWUR20250
CB4856	CT	II	9591292	9592936	WBGene00006473	tag-124	E02H1.3	-0.260150714015012	AGIWUR30273
CB4856	CT	II	9593037	9595128	WBGene00004050	parp-2	E02H1.4	0.0752468516679556	AGIWUR16193;AGIWUR17717;AGIWUR35231;AGIWUR43516
CB4856	CT	II	9595224	9596598	WBGene00008457	E02H1.5	E02H1.5	-0.249429070570741	AGIWUR4642
CB4856	CT	II	9596701	9597528	WBGene00008458	E02H1.6	E02H1.6	-0.133659640631718	AGIWUR24417
CB4856	CT	II	9597622	9598201	WBGene00008459	mrpl-53	E02H1.8	0.245075043170676	AGIWUR10408
CB4856	CT	II	9598197	9605526	WBGene00003618	nhr-19	E02H1.7	1.43788242479012	AGIWUR17743
CB4856	CT	II	9607533	9609396	WBGene00011791	T15H9.6	T15H9.6	-0.239615077994326	AGIWUR2463
CB4856	CT	II	9610678	9611372	WBGene00011790	T15H9.5	T15H9.5	-0.153911504996101	AGIWUR29738
CB4856	CT	II	9611873	9613403	WBGene00011789	T15H9.4	T15H9.4	-0.103562477697558	AGIWUR14684;AGIWUR16096;AGIWUR22993
CB4856	CT	II	9613463	9614812	WBGene00001952	hlh-6	T15H9.3	0.711849538953427	AGIWUR7175
CB4856	CT	II	9615800	9617125	WBGene00011788	T15H9.2	T15H9.2	-0.0103693403849956	AGIWUR40983
CB4856	CT	II	9617426	9624176	WBGene00001038	dnj-20	T15H9.7	-0.0298194899205608	AGIWUR32249;AGIWUR37878;AGIWUR41905;AGIWUR44083
CB4856	CT	II	9624590	9626641	WBGene00014249	ZK1307.8	ZK1307.8	-0.299419401091736	AGIWUR2824
CB4856	CT	II	9636628	9640170	WBGene00014248	ZK1307.7	ZK1307.7	-0.0909809502843144	

AGIWUR17502;AGIWUR21999;AGIWUR22593
CB4856 CT II 9640167 9644466 WBGene00001510 fzr-1 ZK1307.6 -0.0108462155608038
AGIWUR711;AGIWUR5820;AGIWUR6478;AGIWUR13776;AGIWUR16291;AGIWUR25244;AGIWUR44524
CB4856 CT II 9647887 9649367 WBGene00005026 sqv-8 ZK1307.5 -0.0397835617217943
AGIWUR27876
CB4856 CT II 9649491 9650959 WBGene00014250 ZK1307.9 ZK1307.9 0.145970173539055
AGIWUR32506
CB4856 CT II 9652308 9653019 WBGene00014246 ZK1307.3 ZK1307.3 -0.184969004776516
AGIWUR3583
CB4856 CT II 9653109 9653952 WBGene00014245 ZK1307.2 ZK1307.2 0.0932544485401134
AGIWUR13506;AGIWUR21858;AGIWUR23126;AGIWUR32393;AGIWUR33914
CB4856 CT II 9654345 9655616 WBGene00014244 ZK1307.1 ZK1307.1 0.16310118969253
AGIWUR3698;AGIWUR8636;AGIWUR32380
CB4856 CT II 9656689 9657719 WBGene00014251 gstk-1 ZK1320.1 0.125931641828814
AGIWUR2086;AGIWUR2871;AGIWUR24119;AGIWUR28278;AGIWUR28822;AGIWUR32853
CB4856 CT II 9657876 9659002 WBGene00014252 ZK1320.2 ZK1320.2 -0.121642038199788
AGIWUR25137
CB4856 CT II 9659246 9660442 WBGene00014253 ZK1320.3 ZK1320.3 -0.401407503512386
AGIWUR6554;AGIWUR24271;AGIWUR40507
CB4856 CT II 9660839 9663007 WBGene00014254 cyp-13A10 ZK1320.4 0.565953509392308
AGIWUR16893
CB4856 CT II 9663616 9665664 WBGene00014255 ZK1320.5 ZK1320.5 -0.0520483902396096
AGIWUR36576
CB4856 CT II 9667624 9668555 WBGene00014259 ZK1320.11 ZK1320.11 0.152019391936513
AGIWUR41760
CB4856 CT II 9669324 9671499 WBGene00006390 taf-8 ZK1320.12 -0.192586227563815
AGIWUR7931
CB4856 CT II 9671608 9673263 WBGene00014256 ZK1320.7 ZK1320.7 -0.177195485022219
AGIWUR28766
CB4856 CT II 9674978 9677210 WBGene00000180 arc-1 ZK1320.6 -0.00302915774382839
AGIWUR30528
CB4856 CT II 9680798 9683089 WBGene00044007 ZK1320.13 ZK1320.13 -0.274298102148848
AGIWUR2819;AGIWUR11856;AGIWUR29269;AGIWUR29780;AGIWUR42776
CB4856 CT II 9684088 9686713 WBGene00014258 ZK1320.9 ZK1320.9 0.281527442384952
AGIWUR8317;AGIWUR20469;AGIWUR20978;AGIWUR24292;AGIWUR33498
CB4856 CT II 9689664 9691939 WBGene00003749 nlp-11 ZK1320.10 0.275734734634019
AGIWUR11616
CB4856 CT II 9698281 9700463 WBGene00044158 Y53C12A.8 Y53C12A.8 -0.0116310698906146
AGIWUR32060;AGIWUR36415

CB4856	CT	II	9700543	9701839	WBGene00013142	Y53C12A.7	Y53C12A.7	0.113804773319536	AGIWUR2909
CB4856	CT	II	9701402	9703732	WBGene00013140	mop-25.2	Y53C12A.4	-0.129047856852601	AGIWUR20398
CB4856	CT	II	9703812	9704789	WBGene00175030	Y53C12A.10	Y53C12A.10	-0.0706930774442256	AGIWUR40049
CB4856	CT	II	9706483	9708885	WBGene00001623	glt-5	Y53C12A.2	2.41519879216727	AGIWUR40808
CB4856	CT	II	9713737	9716467	WBGene00006940	wee-1.3	Y53C12A.1	-0.017493110379478	AGIWUR7842;AGIWUR12273;AGIWUR14315;AGIWUR16110;AGIWUR25779;AGIWUR30829;AGIWUR39441
CB4856	CT	II	9717478	9717775	WBGene00013141	Y53C12A.6	Y53C12A.6	-0.461780051139423	AGIWUR20674
CB4856	CT	II	9721828	9724526	WBGene00013145	cutl-2	Y53C12B.6	0.924352014662027	AGIWUR17058
CB4856	CT	II	9732137	9738035	WBGene00003100	mab-3	Y53C12B.5	-0.00191596012821904	AGIWUR19751;AGIWUR21247;AGIWUR42412
CB4856	CT	II	9738982	9742884	WBGene00013144	Y53C12B.2	Y53C12B.2	-0.327042566552343	AGIWUR29533
CB4856	CT	II	9742982	9746074	WBGene00013143	Y53C12B.1	Y53C12B.1	-0.194021890058801	AGIWUR19337
CB4856	CT	II	9746316	9750454	WBGene00003785	nos-3	Y53C12B.3	-0.189089996379936	AGIWUR1727;AGIWUR1776;AGIWUR3921;AGIWUR9435;AGIWUR15558;AGIWUR20432;AGIWUR20708;AGIWUR27214;AGIWUR31897;AGIWUR31923;AGIWUR37671;AGIWUR39396
CB4856	CT	II	9750442	9752317	WBGene00013146	Y53C12B.7	Y53C12B.7	0.796163232092113	AGIWUR35367
CB4856	CT	II	9754030	9754967	WBGene00014260	ZK1321.1	ZK1321.1	0.131963978743483	AGIWUR35488
CB4856	CT	II	9758234	9768265	WBGene00014261	shk-1	ZK1321.2	0.0470160700632825	AGIWUR5056;AGIWUR8325;AGIWUR11748;AGIWUR15319;AGIWUR22352;AGIWUR25944;AGIWUR35532;AGIWUR37189;AGIWUR38830
CB4856	CT	II	9768357	9769847	WBGene00000178	aqp-10	ZK1321.3	-0.239890440578405	AGIWUR6508;AGIWUR9418;AGIWUR15485;AGIWUR21502;AGIWUR23911;AGIWUR30029;AGIWUR33320;AGIWUR44993
CB4856	CT	II	9770759	9774292	WBGene00014262	ZK1321.4	ZK1321.4	0.0254353701136338	AGIWUR28204
CB4856	CT	II	9775146	9775883	WBGene00011678	T10B9.9	T10B9.9	0.402239862426699	AGIWUR43510
CB4856	CT	II	9779182	9781950	WBGene00011677	cyp-13A1	T10B9.8	1.66396853889234	AGIWUR10407
CB4856	CT	II	9783403	9787419	WBGene00011676	cyp-13A2	T10B9.7	-0.233631031239533	AGIWUR9781;AGIWUR17049;AGIWUR32347
CB4856	CT	II	9788801	9793284	WBGene00011675	cyp-13A3	T10B9.5	0.402435193212535	

AGIWUR19245;AGIWUR23212;AGIWUR33620
CB4856 CT II 9793754 9795541 WBGene00014838 cyp-13A9 T10B9.6 -0.540704268799906 AGIWUR6915;AGIWUR29141
CB4856 CT II 9795848 9797740 WBGene00011671 cyp-13A4 T10B9.1 0.472265615584345 AGIWUR2689;AGIWUR6539
CB4856 CT II 9798277 9800363 WBGene00011672 cyp-13A5 T10B9.2 0.786991800902216 AGIWUR2802;AGIWUR22628;AGIWUR37154
CB4856 CT II 9800761 9802721 WBGene00011673 cyp-13A6 T10B9.3 0.170103653621948 AGIWUR22808
CB4856 CT II 9803620 9805510 WBGene00000372 cyp-13A7 T10B9.10 0.891043074353036 AGIWUR29039
CB4856 CT II 9805627 9807532 WBGene00011674 cyp-13A8 T10B9.4 0.0956849500985513 AGIWUR14110
CB4856 CT II 9826513 9827926 WBGene00013147 eyg-1 Y53C12C.1 0.931122765243805 AGIWUR28836
CB4856 CT II 9829408 9830753 WBGene00014158 ZK938.1 ZK938.1 0.219955155405928 AGIWUR42641
CB4856 CT II 9831648 9834364 WBGene00014160 ZK938.3 ZK938.3 -0.432090739483874 AGIWUR21525
CB4856 CT II 9835221 9836925 WBGene00014161 arrd-5 ZK938.4 -0.0194926992550428 AGIWUR12573;AGIWUR33602;AGIWUR41879
CB4856 CT II 9837089 9838305 WBGene00014159 arrd-4 ZK938.2 0.401182246445169 AGIWUR18357
CB4856 CT II 9839191 9840288 WBGene00270290 ZK938.11 ZK938.11 -0.157768976337026 AGIWUR4886
CB4856 CT II 9843380 9847006 WBGene00014162 chil-9 ZK938.6 0.0234428100019368 AGIWUR32304
CB4856 CT II 9847483 9849597 WBGene00003863 old-2 ZK938.5 -0.10597668492214 AGIWUR2536
CB4856 CT II 9851471 9853394 WBGene00007468 C08H9.8 C08H9.8 -0.0633644686393193 AGIWUR35968
CB4856 CT II 9855936 9857857 WBGene00007471 chil-6 C08H9.12 0.174772502342556 AGIWUR26676
CB4856 CT II 9858110 9859639 WBGene00007470 chil-5 C08H9.11 0.401881938234487 AGIWUR37372
CB4856 CT II 9859784 9861473 WBGene00007467 chil-3 C08H9.7 0.43622517636109 AGIWUR7823
CB4856 CT II 9862431 9866404 WBGene00007465 chil-1 C08H9.4 -0.298495936604716 AGIWUR1457;AGIWUR28590;AGIWUR41487
CB4856 CT II 9869456 9871172 WBGene00007472 chil-7 C08H9.13 0.285965874871247 AGIWUR8656
CB4856 CT II 9871608 9872986 WBGene00007473 chil-8 C08H9.14 0.457321339226849 AGIWUR37468
CB4856 CT II 9873249 9874521 WBGene00007466 chil-2 C08H9.6 -0.251099142322873 AGIWUR604
CB4856 CT II 9875179 9878008 WBGene00007462 C08H9.1 C08H9.1 0.0500951586564362 AGIWUR39315
CB4856 CT II 9881193 9882530 WBGene00007474 C08H9.15 C08H9.15 -0.217582818610284

AGIWUR35591
CB4856	CT	II	9882728 9886442 WBGene00007464	C08H9.3 C08H9.3 0.0549456179197882
AGIWUR38534;AGIWUR38815;AGIWUR42777
CB4856	CT	II	9886557 9891081 WBGene00007463	vgln-1	C08H9.2 -0.170908603198111
AGIWUR2728;AGIWUR6936;AGIWUR12035;AGIWUR13504;AGIWUR14455;AGIWUR19542;AGIWUR20507;AGIWUR25206;AGIWUR25934;AGIWUR32289;AGIWUR34288;AGIWUR34470;AGIWUR35940;AGIWUR40340;AGIWUR43517
CB4856	CT	II	9895538 9897351 WBGene00009146	F26C11.1	F26C11.1	-0.72704750677773
AGIWUR8276
CB4856	CT	II	9897754 9900376 WBGene00006744	unc-4	F26C11.2	0.158870357191349
AGIWUR19606;AGIWUR34527;AGIWUR35185
CB4856	CT	II	9903059 9915099 WBGene00009147	F26C11.3	F26C11.3	0.128667841086425
AGIWUR33285
CB4856	CT	II	9915438 9916289 WBGene00044159	F26C11.4	F26C11.4	-0.375594775996032
AGIWUR26120;AGIWUR28479;AGIWUR32920;AGIWUR35033
CB4856	CT	II	9917122 9917943 WBGene00086567	C05C10.8	C05C10.8	0.0972671149746986
AGIWUR38038
CB4856	CT	II	9922248 9930830 WBGene00007329	C05C10.2	C05C10.2	-0.0741016538207901
AGIWUR6878;AGIWUR29477;AGIWUR44420
CB4856	CT	II	9931845 9934443 WBGene00007331	pho-11	C05C10.4	0.0647953347834846
AGIWUR2532;AGIWUR6049;AGIWUR22087;AGIWUR30141;AGIWUR34634;AGIWUR34659;AGIWUR36019
CB4856	CT	II	9934403 9936893 WBGene00007330	C05C10.3	C05C10.3	-0.321075873301957
AGIWUR2054;AGIWUR3537;AGIWUR18491;AGIWUR21068;AGIWUR21521;AGIWUR25993;AGIWUR37963
CB4856	CT	II	9938106 9938971 WBGene00007334	C05C10.7	C05C10.7	-0.233747602445252
AGIWUR37632
CB4856	CT	II	9939040 9940432 WBGene00007332	C05C10.5	C05C10.5	-0.109383937187754
AGIWUR22090;AGIWUR32414
CB4856	CT	II	9940758 9944006 WBGene00007333	ufd-3	C05C10.6	0.0213542463329368
AGIWUR3612;AGIWUR5667;AGIWUR10944;AGIWUR24102;AGIWUR24734;AGIWUR35125;AGIWUR39205;AGIWUR41062;AGIWUR42078;AGIWUR42640
CB4856	CT	II	9944026 9947121 WBGene00011271	flad-1	R53.1	0.14152333951419
AGIWUR12784;AGIWUR21392;AGIWUR30921;AGIWUR33515
CB4856	CT	II	9955185 9962344 WBGene00001207	egl-43	R53.3	0.208963092964085	AGIWUR28128
CB4856	CT	II	9962966 9967132 WBGene00011272	dtmk-1	R53.2	-0.00818073873636038
AGIWUR14400;AGIWUR44983
CB4856	CT	II	9967277 9968049 WBGene00011273	R53.4	R53.4	0.00241021939185693
AGIWUR274;AGIWUR839;AGIWUR1651;AGIWUR2205;AGIWUR2273;AGIWUR19713;AGIWUR21938;AGIWUR30159;AGIWUR30299;AGIWUR30811;AGIWUR33954
CB4856	CT	II	9968659 9969631 WBGene00011275	psf-1	R53.6	-0.399691686538666
AGIWUR651;AGIWUR4183;AGIWUR19144;AGIWUR19922

CB4856	CT	II	9969620	9970515	WBGene00011274	R53.5	R53.5	0.344273230596233	AGIWUR39788
CB4856	CT	II	9971035	9975125	WBGene00011276	aakg-5	R53.7	0.0750001507935991	AGIWUR19477
CB4856	CT	II	9976705	9977877	WBGene00011277	R53.8	R53.8	-0.34299350200204	AGIWUR38025
CB4856	CT	II	9978765	9980076	WBGene00014130	ZK892.6	ZK892.6	-0.401146898051055	AGIWUR41533
CB4856	CT	II	9980886	9981752	WBGene00014131	sdz-38	ZK892.7	0.0216445616082215	AGIWUR12129
CB4856	CT	II	9991160	10000350	WBGene00002266	lec-3	ZK892.1	0.220287820107725	AGIWUR8893;AGIWUR9049;AGIWUR11331;AGIWUR11760;AGIWUR36080
CB4856	CT	II	10000335	10001198	WBGene00003773	nlt-1	ZK892.2	0.0852446080808775	AGIWUR9175;AGIWUR15509;AGIWUR22828;AGIWUR25447;AGIWUR39257;AGIWUR39674;AGIWUR42147
CB4856	CT	II	10001846	10003987	WBGene00014127	ZK892.3	ZK892.3	0.23323241377719	AGIWUR26549
CB4856	CT	II	10006111	10006535	WBGene00044037	ZK892.8	ZK892.8	-0.285830160999364	AGIWUR23204
CB4856	CT	II	10006642	10008089	WBGene00014128	ZK892.4	ZK892.4	0.103098016307394	AGIWUR41562
CB4856	CT	II	10008217	10009401	WBGene00014129	ZK892.5	ZK892.5	-0.132094745531351	AGIWUR7848;AGIWUR20371;AGIWUR34857
CB4856	CT	II	10009608	10016183	WBGene00002265	lec-2	F52H3.7	0.922170582566734	AGIWUR7658;AGIWUR10730;AGIWUR27476;AGIWUR34199
CB4856	CT	II	10030195	10030970	WBGene00009947	F52H3.5	F52H3.5	0.010243462477284	AGIWUR26508
CB4856	CT	II	10030895	10033213	WBGene00009944	mtcu-2	F52H3.2	0.166522458979479	AGIWUR8504
CB4856	CT	II	10033309	10039980	WBGene00002497	let-268	F52H3.1	-0.0186560884113174	AGIWUR26464
CB4856	CT	II	10041134	10042515	WBGene00009946	F52H3.4	F52H3.4	-0.086449665706585	AGIWUR8233
CB4856	CT	II	10042780	10045270	WBGene00009945	bath-38	F52H3.3	0.26612065884078	AGIWUR38471
CB4856	CT	II	10054623	10055594	WBGene00001447	flp-4	C18D1.3	0.237603332553846	AGIWUR27802
CB4856	CT	II	10056311	10060582	WBGene00007670	stg-1	C18D1.4	0.393498334542087	AGIWUR14704
CB4856	CT	II	10061287	10074443	WBGene00000995	die-1	C18D1.1	0.0932716804201057	AGIWUR1596;AGIWUR11535;AGIWUR23819;AGIWUR27718;AGIWUR27931;AGIWUR31103;AGIWUR42675
CB4856	CT	II	10094873	10098785	WBGene00014164	lact-2	ZK945.1	0.649679648226005	AGIWUR39450
CB4856	CT	II	10098782	10099867	WBGene00003928	pas-7	ZK945.2	-0.0916774343049189	AGIWUR12828;AGIWUR14425

CB4856	CT	II	10100310	10103059	WBGene00014165	puf-12	ZK945.3	-0.171095206344882	AGIWUR15434
CB4856	CT	II	10102975	10105704	WBGene00014166	ZK945.4	ZK945.4	-0.312240031127647	AGIWUR3719;AGIWUR12972;AGIWUR17154;AGIWUR23722;AGIWUR35881
CB4856	CT	II	10106080	10106970	WBGene00014168	ZK945.6	ZK945.6	0.0925489296094124	AGIWUR30388
CB4856	CT	II	10107473	10108477	WBGene00014169	ZK945.7	ZK945.7	0.320420836987967	AGIWUR37463
CB4856	CT	II	10108702	10109081	WBGene00014170	ZK945.8	ZK945.8	-0.223073062286962	AGIWUR37452
CB4856	CT	II	10109279	10121825	WBGene00003058	lov-1	ZK945.9	0.112672391047518	AGIWUR1185
CB4856	CT	II	10122765	10125015	WBGene00044035	F27E5.7	F27E5.7	-0.24788888199291	AGIWUR32500
CB4856	CT	II	10125898	10127455	WBGene00044027	F27E5.8	F27E5.8	-0.0862385397810517	AGIWUR25811
CB4856	CT	II	10128254	10131860	WBGene00009194	F27E5.5	F27E5.5	-0.0910800753405967	AGIWUR7390
CB4856	CT	II	10137658	10139216	WBGene00004017	phg-1	F27E5.4	-0.0530027552344752	AGIWUR40529
CB4856	CT	II	10139590	10141406	WBGene00009192	asah-2	F27E5.1	0.320473713712017	AGIWUR18928;AGIWUR22197;AGIWUR33073
CB4856	CT	II	10141457	10144192	WBGene00009193	F27E5.3	F27E5.3	0.0982323242510912	AGIWUR44100
CB4856	CT	II	10146746	10152445	WBGene00003939	pax-3	F27E5.2	0.335506422901423	AGIWUR18917
CB4856	CT	II	10155413	10156220	WBGene00044028	F33H1.6	F33H1.6	-0.420089193643058	AGIWUR5814;AGIWUR10380;AGIWUR29406;AGIWUR40440;AGIWUR44519
CB4856	CT	II	10157537	10169346	WBGene00000914	daf-19	F33H1.1	0.507392231528197	AGIWUR10712;AGIWUR11493;AGIWUR14380
CB4856	CT	II	10172923	10174346	WBGene00009364	F33H1.3	F33H1.3	0.152076509543543	AGIWUR16485
CB4856	CT	II	10174572	10180183	WBGene00009365	F33H1.4	F33H1.4	-0.168760579872088	AGIWUR21472;AGIWUR27653;AGIWUR34930
CB4856	CT	II	10180199	10182734	WBGene00005079	srd-1	F33H1.5	-0.0491855769713858	AGIWUR18723
CB4856	CT	II	10185789	10187908	WBGene00005080	srd-2	R05H5.1	0.0354077995161886	AGIWUR14572
CB4856	CT	II	10188263	10189469	WBGene00000389	cdc-25.4	R05H5.2		

-0.00849393626851214	AGIWUR15999
CB4856	CT	II	10192529	10193451	WBGene00011041	R05H5.7	R05H5.7	-0.0416842112261486	AGIWUR6013;AGIWUR11895;AGIWUR21955;AGIWUR26462
CB4856	CT	II	10194905	10196835	WBGene00005071	srb-6	R05H5.6	-0.150781847161512	AGIWUR32602
CB4856	CT	II	10197625	10198324	WBGene00011038	R05H5.3	R05H5.3	0.0569536974980345	AGIWUR6581;AGIWUR9461;AGIWUR24214;AGIWUR29342;AGIWUR31178;AGIWUR37075
CB4856	CT	II	10198309	10199768	WBGene00011039	R05H5.4	R05H5.4	0.477709240996044	AGIWUR10312
CB4856	CT	II	10199810	10200819	WBGene00011040	R05H5.5	R05H5.5	0.441841270307531	AGIWUR12341
CB4856	CT	II	10201125	10202230	WBGene00006651	tts-2	R05H5.8	1.22435609121238	AGIWUR23370
CB4856	CT	II	10206715	10209391	WBGene00011331	T01E8.1	T01E8.1	0.396908824914171	AGIWUR29024
CB4856	CT	II	10218471	10220025	WBGene00004334	ref-1	T01E8.2	0.0647468862110756	AGIWUR8907
CB4856	CT	II	10222877	10229440	WBGene00004038	plc-3	T01E8.3	0.0549123918401575	AGIWUR14929;AGIWUR18912;AGIWUR22081;AGIWUR24082;AGIWUR28683;AGIWUR42469
CB4856	CT	II	10231272	10236014	WBGene00011335	T01E8.8	T01E8.8	-0.136428944353082	AGIWUR33309
CB4856	CT	II	10236936	10239695	WBGene00003177	mec-15	T01E8.4	-0.221487682310699	AGIWUR1407;AGIWUR9774;AGIWUR10357;AGIWUR34006
CB4856	CT	II	10240277	10242490	WBGene00011334	mrps-14	T01E8.6	-0.218567355532018	AGIWUR1632;AGIWUR18563;AGIWUR25105
CB4856	CT	II	10242595	10247638	WBGene00011333	nrde-2	T01E8.5	-0.00575316300126705	AGIWUR1484;AGIWUR28560
CB4856	CT	II	10248991	10251165	WBGene00010015	atad-3	F54B3.3	-0.245721486839628	AGIWUR20261;AGIWUR36134;AGIWUR44377
CB4856	CT	II	10251266	10268168	WBGene00010013	F54B3.1	F54B3.1	-0.0879096202365837	AGIWUR11170;AGIWUR12967;AGIWUR28208
CB4856	CT	II	10268143	10270152	WBGene00010014	F54B3.2	F54B3.2	-0.0924617434183759	AGIWUR39268
CB4856	CT	II	10278732	10280459	WBGene00014175	ZK971.1	ZK971.1	1.09673868947924	AGIWUR39627;AGIWUR39902
CB4856	CT	II	10283466	10300132	WBGene00014171	nep-26	ZK970.1	0.280358730383576	AGIWUR41540
CB4856	CT	II	10300789	10302859	WBGene00014172	clpp-1	ZK970.2	0.0747265178685215	AGIWUR39591

CB4856	CT	II	10303007	10303713	WBGene00006918	vha-9	ZK970.4	-0.0102527491628077	AGIWUR29615
CB4856	CT	II	10304139	10308826	WBGene00001531	gcy-4	ZK970.5	-0.723026508012637	AGIWUR24821
CB4856	CT	II	10309418	10310068	WBGene00014174	ZK970.8	ZK970.8	-0.239897627044067	AGIWUR15415
CB4856	CT	II	10310400	10316219	WBGene00001532	gcy-5	ZK970.6	-0.217841773374404	AGIWUR35870
CB4856	CT	II	10318228	10319970	WBGene00010832	M02G9.3	M02G9.3	-0.289772862171883	AGIWUR754
CB4856	CT	II	10321460	10324850	WBGene00010831	M02G9.2	M02G9.2	-0.35801630430529	AGIWUR29671
CB4856	CT	II	10326041	10335807	WBGene00010830	M02G9.1	M02G9.1	0.157677775522664	AGIWUR21007
CB4856	CT	II	10339692	10345112	WBGene00007413	pro-2	C07E3.2	-0.0855874933497383	AGIWUR25738
CB4856	CT	II	10346375	10347001	WBGene00007420	C07E3.10	C07E3.10	-0.330443593299412	AGIWUR7074
CB4856	CT	II	10346981	10350007	WBGene00007412	stip-1	C07E3.1	-0.177466848520652	AGIWUR16235;AGIWUR18632;AGIWUR37575
CB4856	CT	II	10350760	10351613	WBGene00007419	C07E3.9	C07E3.9	0.000560842365347369	AGIWUR41793
CB4856	CT	II	10353584	10357518	WBGene00007414	C07E3.3	C07E3.3	0.739925398559485	AGIWUR42376
CB4856	CT	II	10363584	10365709	WBGene00007416	ceh-57	C07E3.5	0.118942191753002	AGIWUR12801
CB4856	CT	II	10367741	10369597	WBGene00007417	ceh-58	C07E3.6	-0.116878487317519	AGIWUR5364;AGIWUR14869
CB4856	CT	II	10385333	10388133	WBGene00011661	ztf-27	T09F3.1	0.336699675723377	AGIWUR9306
CB4856	CT	II	10391154	10393614	WBGene00011662	T09F3.2	T09F3.2	-0.0206195136736946	AGIWUR3756;AGIWUR5121;AGIWUR16551;AGIWUR28159;AGIWUR35682;AGIWUR38281;AGIWUR39478
CB4856	CT	II	10394114	10395349	WBGene00001683	gpd-1	T09F3.3	0.0366186445505505	AGIWUR11243
CB4856	CT	II	10395707	10396756	WBGene00011663	T09F3.4	T09F3.4	0.0237879208977398	AGIWUR881;AGIWUR42449
CB4856	CT	II	10396878	10399320	WBGene00011664	T09F3.5	T09F3.5	-0.0809954097766017	AGIWUR17018
CB4856	CT	II	10411289	10412670	WBGene00008484	E04D5.5	E04D5.5	-0.223814599738297	

AGIWUR2989
CB4856	CT	II	10416786	10419237	WBGene00008480	eif-2A	E04D5.1	0.0504300787505425
AGIWUR21663;AGIWUR24337;AGIWUR42583
CB4856	CT	II	10419230	10422026	WBGene00008481	E04D5.2	E04D5.2	0.112783540930799
AGIWUR1199
CB4856	CT	II	10440286	10442515	WBGene00008483	E04D5.4	E04D5.4	-0.0681110223981065
AGIWUR3398;AGIWUR37195;AGIWUR39288
CB4856	CT	II	10443327	10445233	WBGene00014057	ZK673.1	ZK673.1	0.415529801653605
AGIWUR43812
CB4856	CT	II	10448266	10450222	WBGene00014058	ZK673.2	ZK673.2	0.609203364664687
AGIWUR4328;AGIWUR10658;AGIWUR17853;AGIWUR37680
CB4856	CT	II	10450607	10452004	WBGene00003038	lin-56	ZK673.3	-0.335052236288465
AGIWUR25094
CB4856	CT	II	10452920	10455200	WBGene00014060	ZK673.4	ZK673.4	-0.0916922101818169
AGIWUR34906
CB4856	CT	II	10457786	10462672	WBGene00014065	ZK673.11	ZK673.11
-0.0611017173053234	AGIWUR1953
CB4856	CT	II	10462870	10464330	WBGene00014062	ZK673.6	ZK673.6	-0.329342385196716
AGIWUR41397
CB4856	CT	II	10464870	10465298	WBGene00014064	sdz-37	ZK673.10
-0.620382122753901	AGIWUR36805
CB4856	CT	II	10466852	10468344	WBGene00006583	tnc-2	ZK673.7	0.481829765591325
AGIWUR23392
CB4856	CT	II	10468351	10470297	WBGene00014063	clec-143	ZK673.9
0.022444998698802	AGIWUR27744
CB4856	CT	II	10470934	10471347	WBGene00014041	ZK666.1	ZK666.1	-0.124825957734455
AGIWUR24121
CB4856	CT	II	10471696	10471890	WBGene00014042	ZK666.2	ZK666.2	-0.124126529786881
AGIWUR4815;AGIWUR39373
CB4856	CT	II	10472439	10473726	WBGene00005520	sri-8	ZK666.10
0.0914986173988783	AGIWUR876;AGIWUR12313;AGIWUR40432
CB4856	CT	II	10474221	10476139	WBGene00014043	clec-58	ZK666.3	0.0126100503022721
AGIWUR14034;AGIWUR18180;AGIWUR26359
CB4856	CT	II	10478022	10479448	WBGene00014045	clec-59	ZK666.5	0.150397915257392
AGIWUR30540
CB4856	CT	II	10479920	10480081	WBGene00044046	ZK666.13	ZK666.13
-0.0178361049267091	AGIWUR12895;AGIWUR23306;AGIWUR31826;AGIWUR35627;AGIWUR39741
CB4856	CT	II	10480152	10481846	WBGene00014046	clec-60	ZK666.6	3.10438525101614
AGIWUR2509;AGIWUR31381;AGIWUR35871

CB4856	CT	II	10488747	10490514	WBGene00014047	clec-61	ZK666.7	1.23320760332113	AGIWUR38848
CB4856	CT	II	10491662	10493134	WBGene00014048	ZK666.8	ZK666.8	-0.063214916564263	AGIWUR3288
CB4856	CT	II	10496212	10496703	WBGene00014050	ZK666.12	ZK666.12	0.166586531615586	AGIWUR8874
CB4856	CT	II	10496987	10502541	WBGene00004134	myrf-1	F59B10.1	0.0296479200536943	AGIWUR131
CB4856	CT	II	10507269	10509932	WBGene00010318	F59B10.2	F59B10.2	0.309622710962357	AGIWUR23868
CB4856	CT	II	10510542	10512991	WBGene00010319	F59B10.3	F59B10.3	-0.350491894605304	AGIWUR38198
CB4856	CT	II	10513827	10519108	WBGene00010320	F59B10.4	F59B10.4	-0.0163806036921876	AGIWUR38327
CB4856	CT	II	10521788	10523191	WBGene00010321	F59B10.5	F59B10.5	-0.123774453902853	AGIWUR6528
CB4856	CT	II	10523274	10524474	WBGene00010322	F59B10.6	F59B10.6	-0.0205320959697772	AGIWUR5739;AGIWUR23280;AGIWUR29381;AGIWUR36335;AGIWUR40116;AGIWUR44732
CB4856	CT	II	10528084	10533880	WBGene00003107	mab-10	R166.1	-0.517644356671362	AGIWUR1300
CB4856	CT	II	10534332	10537671	WBGene00011302	R166.2	R166.2	0.0781412991967017	AGIWUR39289
CB4856	CT	II	10537799	10539332	WBGene00011303	R166.3	R166.3	-0.154951592542613	AGIWUR36948
CB4856	CT	II	10539438	10541996	WBGene00004185	pro-1	R166.4	-0.281279757717396	AGIWUR31553
CB4856	CT	II	10542428	10555044	WBGene00011304	mnk-1	R166.5	0.264224652431729	AGIWUR34539
CB4856	CT	II	10560018	10563407	WBGene00007352	cdc-48.1	C06A1.1	-0.273443295648981	AGIWUR11455;AGIWUR13116;AGIWUR21063;AGIWUR26985;AGIWUR43969
CB4856	CT	II	10570602	10572530	WBGene00007353	C06A1.2	C06A1.2	0.270313816742629	AGIWUR9060
CB4856	CT	II	10572247	10574067	WBGene00007354	C06A1.3	C06A1.3	-0.0369822886477742	AGIWUR6893
CB4856	CT	II	10574842	10578193	WBGene00014667	C06A1.4	C06A1.4	-0.349501540804823	AGIWUR25976;AGIWUR30602
CB4856	CT	II	10579366	10579955	WBGene00007355	rpb-6	C06A1.5	-0.0308052072052725	AGIWUR5821
CB4856	CT	II	10579955	10581045	WBGene00007555	dohh-1	C14A4.1	0.13175253077382	

AGIWUR2970;AGIWUR7187;AGIWUR19048
CB4856 CT II 10581133 10582519 WBGene00000933 dap-3 C14A4.2 -0.309310587286713
AGIWUR24770;AGIWUR27331;AGIWUR27823;AGIWUR32317
CB4856 CT II 10582618 10584236 WBGene00007564 mrps-22 C14A4.14
-0.0522411507709455 AGIWUR11916;AGIWUR14585;AGIWUR17314;AGIWUR19277;AGIWUR22350;AGIWUR33834;AGIWUR36123
CB4856 CT II 10584336 10587928 WBGene00007556 C14A4.3 C14A4.3 -0.201008515234353
AGIWUR41411
CB4856 CT II 10588042 10592652 WBGene00000796 crn-3 C14A4.4 -0.0734036194933765
AGIWUR23554;AGIWUR44213
CB4856 CT II 10592752 10593591 WBGene00000798 crn-5 C14A4.5 -0.0636574277171249
AGIWUR22345;AGIWUR34177;AGIWUR35482
CB4856 CT II 10593529 10594776 WBGene00007557 C14A4.6 C14A4.6 0.0749710988268983
AGIWUR4051;AGIWUR21431;AGIWUR24456
CB4856 CT II 10595266 10596562 WBGene00007558 C14A4.7 C14A4.7 -0.215114704933695
AGIWUR15049;AGIWUR25017;AGIWUR26477;AGIWUR39292
CB4856 CT II 10597174 10598100 WBGene00007559 C14A4.8 C14A4.8 -0.328661098481822
AGIWUR22102
CB4856 CT II 10599337 10600529 WBGene00007560 C14A4.9 C14A4.9 0.564026766235617
AGIWUR25127
CB4856 CT II 10600599 10602276 WBGene00005713 srv-2 C14A4.15
0.0624941723868844 AGIWUR22923
CB4856 CT II 10605030 10605612 WBGene00006397 taf-13 C14A4.10
0.0977762742166153 AGIWUR24634
CB4856 CT II 10605717 10607275 WBGene00007561 ccm-3 C14A4.11
-0.159980806423341 AGIWUR27434
CB4856 CT II 10613020 10618332 WBGene00007562 C14A4.12 C14A4.12
0.0775204258591238 AGIWUR29508
CB4856 CT II 10618471 10620503 WBGene00007563 C14A4.13 C14A4.13
-0.0157995272437681 AGIWUR32603
CB4856 CT II 10622265 10625943 WBGene00010893 cutl-9 M28.1 0.887213519679328
AGIWUR4593;AGIWUR17552;AGIWUR21126;AGIWUR23189;AGIWUR36371;AGIWUR36670;AGIWUR40197
CB4856 CT II 10628263 10633216 WBGene00010899 M28.8 M28.8 0.691060744988931
AGIWUR44648
CB4856 CT II 10633476 10639112 WBGene00010894 M28.2 M28.2 0.00524871877183131
AGIWUR2316;AGIWUR15524;AGIWUR29354
CB4856 CT II 10639410 10643536 WBGene00010900 M28.9 M28.9 0.0323969423635084
AGIWUR4478
CB4856 CT II 10643703 10646978 WBGene00010895 M28.4 M28.4 0.498972504337816
AGIWUR26377

CB4856 CT II 10647480 10648676 WBGene00010901 M28.10 M28.10 -0.328361565101957
AGIWUR21717
CB4856 CT II 10648938 10652256 WBGene00010898 nphp-1 M28.7 0.613839464512935
AGIWUR6755
CB4856 CT II 10652353 10657841 WBGene00010897 lact-3 M28.6 0.327275109750587
AGIWUR25318
CB4856 CT II 10658027 10658626 WBGene00010896 M28.5 M28.5 -0.109654363146138
AGIWUR25451;AGIWUR33760
CB4856 CT II 10659206 10662317 WBGene00004704 rsp-7 D2089.1 -0.0102412306426052
AGIWUR4646;AGIWUR11930;AGIWUR13996;AGIWUR17240;AGIWUR21144;AGIWUR29901;AGIWUR38441;AGIWUR40407;AGIWUR43998
CB4856 CT II 10662256 10665131 WBGene00008434 D2089.3 D2089.3 0.0485692155001245
AGIWUR26579;AGIWUR27148;AGIWUR28093;AGIWUR33867
CB4856 CT II 10675602 10690271 WBGene00004207 ptb-1 D2089.4 0.0217816228965901
AGIWUR6285;AGIWUR12901;AGIWUR21246
CB4856 CT II 10690452 10693863 WBGene00001522 gbh-1 D2089.5 -0.261263809378027
AGIWUR19935
CB4856 CT II 10695013 10702524 WBGene00001586 ggr-1 C09G5.1 -0.121380035512618
AGIWUR2899;AGIWUR11942
CB4856 CT II 10702509 10714297 WBGene00007488 dph-2 C09G5.2 -0.037098155095725
AGIWUR23621
CB4856 CT II 10714886 10716416 WBGene00007489 C09G5.7 C09G5.7 -0.214849011169579
AGIWUR23618
CB4856 CT II 10720231 10729252 WBGene00007490 mks-5 C09G5.8 0.814587678267283
AGIWUR34107
CB4856 CT II 10729804 10731627 WBGene00008256 glb-12 C52A11.2
0.195202719684954 AGIWUR27689
CB4856 CT II 10737402 10738628 WBGene00008257 C52A11.3 C52A11.3
0.114389376349225 AGIWUR42803
CB4856 CT II 10738635 10775061 WBGene00003404 mpz-1 C52A11.4
0.105631986469411
AGIWUR6677;AGIWUR9212;AGIWUR16617;AGIWUR19487;AGIWUR20489;AGIWUR20775;AGIWUR25775;AGIWUR27445;AGIWUR28484;AGIWUR30306;AGIWUR32704;AGIWUR32857;AGIWUR39509;AGIWUR40262
CB4856 CT II 10778389 10783962 WBGene00002297 ect-2 T19E10.1
0.0719200119238312 AGIWUR1328;AGIWUR5981;AGIWUR9727;AGIWUR21337;AGIWUR24688;AGIWUR44022
CB4856 CT II 10785452 10787272 WBGene00000411 cdl-1 R06F6.1 -0.122888810254172
AGIWUR1579;AGIWUR4225
CB4856 CT II 10787312 10787914 WBGene00011072 tag-209 R06F6.11
0.0939751481221087 AGIWUR5104
CB4856 CT II 10788106 10793493 WBGene00011067 vps-11 R06F6.2 0.235948765069535

AGIWUR9444
CB4856 CT II 10793575 10796294 WBGene00011068 set-14 R06F6.4 -0.192591108089484
AGIWUR9082
CB4856 CT II 10796646 10798479 WBGene00003805 npp-19 R06F6.5 -0.0935570544880342
AGIWUR27834;AGIWUR30360
CB4856 CT II 10809279 10809847 WBGene00269421 R06F6.14 R06F6.14
0.419036655584126 AGIWUR20089
CB4856 CT II 10811016 10812188 WBGene00011070 R06F6.7 R06F6.7 0.730988713827317
AGIWUR37319
CB4856 CT II 10812949 10819978 WBGene00011071 R06F6.8 R06F6.8 -0.0383518754078658
AGIWUR13413
CB4856 CT II 10821015 10822443 WBGene00001153 ech-4 R06F6.9 0.0226804253555635
AGIWUR32397
CB4856 CT II 10822328 10824081 WBGene00023418 R06F6.12 R06F6.12
-0.2980526319323 AGIWUR23823
CB4856 CT II 10824294 10841424 WBGene00003367 mix-1 M106.1 -0.0765835026068711
AGIWUR7474;AGIWUR7521;AGIWUR30139;AGIWUR39827
CB4856 CT II 10855642 10857177 WBGene00044924 M106.7 M106.7 0.0627761474660602
AGIWUR22794
CB4856 CT II 10857473 10860909 WBGene00010911 M106.3 M106.3 -0.205906138548009
AGIWUR25515;AGIWUR38047
CB4856 CT II 10863682 10868714 WBGene00010912 gmps-1 M106.4 -0.267062039232098
AGIWUR4592;AGIWUR25800;AGIWUR26865;AGIWUR30939;AGIWUR33439
CB4856 CT II 10869196 10870827 WBGene00000293 cap-2 M106.5 -0.0703918777862542
AGIWUR17286;AGIWUR17665;AGIWUR38899;AGIWUR40831
CB4856 CT II 10870779 10872611 WBGene00000880 cyn-4 F59E10.2
0.0656792044197267 AGIWUR17570;AGIWUR29891;AGIWUR33211
CB4856 CT II 10872879 10873796 WBGene00010333 copz-1 F59E10.3
0.0908058066856512 AGIWUR33271
CB4856 CT II 10873931 10877934 WBGene00003882 orc-2 F59E10.1
-0.067880378769031 AGIWUR32871;AGIWUR40159
CB4856 CT II 10880925 10882427 WBGene00009700 lurp-1 F44F4.1 0.182148613598642
AGIWUR27458
CB4856 CT II 10882408 10884462 WBGene00009701 egg-3 F44F4.2 -0.251127896211333
AGIWUR20772
CB4856 CT II 10884705 10885878 WBGene00009702 F44F4.3 F44F4.3 0.077277486206178
AGIWUR21067
CB4856 CT II 10889279 10893288 WBGene00004222 ptr-8 F44F4.4 0.0245318153316774
AGIWUR37161

CB4856	CT	II	10893275	10895088	WBGene00005036	sra-10	F44F4.5	0.181716160516702	AGIWUR12637
CB4856	CT	II	10900042	10902204	WBGene00001626	gly-1	F44F4.6	-0.268791480837275	AGIWUR1846
CB4856	CT	II	10902756	10904479	WBGene00005038	sra-12	F44F4.7	-0.185913598143162	AGIWUR16841;AGIWUR26876
CB4856	CT	II	10908808	10910319	WBGene00005037	sra-11	F44F4.13	0.39336845645153	AGIWUR9493;AGIWUR17231;AGIWUR40553
CB4856	CT	II	10915901	10917274	WBGene00009705	F44F4.10	F44F4.10	0.0374289312585461	AGIWUR15150
CB4856	CT	II	10918706	10920202	WBGene00006530	tba-4	F44F4.11	-0.607539976724353	AGIWUR1211;AGIWUR23556
CB4856	CT	II	10922672	10922974	WBGene00012489	Y19D2B.1	Y19D2B.1	-0.437883134621632	AGIWUR43206
CB4856	CT	II	10923716	10924595	WBGene00012490	Y19D2B.2	Y19D2B.2	-0.065307241048326	AGIWUR25225
CB4856	CT	II	10926561	10931075	WBGene00010988	metr-1	R03D7.1	-0.0219582997803389	AGIWUR5757;AGIWUR6884;AGIWUR43211
CB4856	CT	II	10935726	10942198	WBGene00014814	R03D7.3	R03D7.3	-0.147972373303486	AGIWUR4633;AGIWUR35952
CB4856	CT	II	10943390	10945660	WBGene00010990	R03D7.4	R03D7.4	-0.0959042997566257	AGIWUR31576
CB4856	CT	II	10947610	10949184	WBGene00010991	R03D7.5	R03D7.5	0.0191043029601303	AGIWUR30739
CB4856	CT	II	10950350	10951604	WBGene00001753	gst-5	R03D7.6	-0.064084138877206	AGIWUR32044
CB4856	CT	II	10951879	10953165	WBGene00003783	nos-1	R03D7.7	-0.127305833176788	AGIWUR19424
CB4856	CT	II	10953594	10956878	WBGene00010992	R03D7.8	R03D7.8	0.0138172574125974	AGIWUR44506
CB4856	CT	II	10968386	11012247	WBGene00004215	ptp-3	C09D8.1	0.0939962477513632	AGIWUR3429;AGIWUR6662;AGIWUR13959;AGIWUR34474;AGIWUR35314;AGIWUR37980
CB4856	CT	II	11012397	11013820	WBGene00000657	col-81	F38A3.1	-0.065566175201246	AGIWUR3425
CB4856	CT	II	11015588	11016881	WBGene00004300	ram-2	F38A3.2	-0.259343184368195	AGIWUR4854;AGIWUR6326;AGIWUR11647
CB4856	CT	II	11022370	11027909	WBGene00002957	let-858	F33A8.1	0.0338679067909844	AGIWUR12874;AGIWUR29582
CB4856	CT	II	11027962	11030404	WBGene00003756	nlp-18	F33A8.2	0.203609297534961	

AGIWUR5506;AGIWUR38400
CB4856	CT	II	11036074	11039471	WBGene00009356	F33A8.10	F33A8.10
-0.571890973944088	AGIWUR20869
CB4856	CT	II	11039348	11041206	WBGene00000472	cey-1	F33A8.3 0.329679610012002
AGIWUR534;AGIWUR9013;AGIWUR15336;AGIWUR29341;AGIWUR30653
CB4856	CT	II	11044135	11045968	WBGene00000658	col-83	F33A8.9 0.275797687092383
AGIWUR40812
CB4856	CT	II	11045786	11049030	WBGene00009352	F33A8.4 F33A8.4 -0.181880007240824
AGIWUR17337
CB4856	CT	II	11049144	11049913	WBGene00009353	sdhd-1	F33A8.5 0.0792100374920608
AGIWUR2216
CB4856	CT	II	11058096	11089456	WBGene00006788	unc-53	F45E10.1
0.284449956193341	AGIWUR14085
CB4856	CT	II	11092713	11093871	WBGene00004455	rpl-42	C09H10.1
0.116548719290702	AGIWUR24645
CB4856	CT	II	11095048	11096475	WBGene00007504	C09H10.10	C09H10.10
-0.217519736481258	AGIWUR31480
CB4856	CT	II	11096542	11097204	WBGene00004454	rpl-36.A	C09H10.2
0.117671498795179	AGIWUR20646
CB4856	CT	II	11097841	11100146	WBGene00003831	nuo-1	C09H10.3
0.0846967608163824	AGIWUR11037;AGIWUR44982
CB4856	CT	II	11100141	11100462	WBGene00043120	C09H10.4	C09H10.4
-0.0504567141994565	AGIWUR35898
CB4856	CT	II	11100479	11103779	WBGene00007499	C09H10.5	C09H10.5
-0.409603465173209	AGIWUR19231
CB4856	CT	II	11104462	11106046	WBGene00007503	C09H10.9	C09H10.9
0.112799382106729	AGIWUR20653
CB4856	CT	II	11106616	11108070	WBGene00007500	nasp-1	C09H10.6
-0.0789467436443445	AGIWUR24585
CB4856	CT	II	11108159	11109583	WBGene00007501	apc-17	C09H10.7
-0.333385970963137	AGIWUR44608
CB4856	CT	II	11109584	11111169	WBGene00007502	glb-4	C09H10.8
-0.137183511360593	AGIWUR34694
CB4856	CT	II	11112719	11120788	WBGene00003133	mat-2	W10C6.1 0.00834411117612843
AGIWUR1142;AGIWUR11753;AGIWUR12091;AGIWUR12124;AGIWUR13946;AGIWUR26553
CB4856	CT	II	11120882	11121900	WBGene00044160	W10C6.2 W10C6.2 0.0163538918405148
AGIWUR3254;AGIWUR7000;AGIWUR13656;AGIWUR14327;AGIWUR41178
CB4856	CT	II	11125371	11126362	WBGene00004478	rps-9	F40F8.10
0.00922073467106799	AGIWUR13095;AGIWUR27232;AGIWUR44243

CB4856	CT	II	11126932	11127530	WBGene00003076	lsm-1	F40F8.9	-0.148219779575292
AGIWUR21932
CB4856	CT	II	11127634	11129256	WBGene00009575	F40F8.1	F40F8.1	0.0945147452284871
AGIWUR30901
CB4856	CT	II	11129289	11130187	WBGene00009576	F40F8.3	F40F8.3	0.154344118451633
AGIWUR8310;AGIWUR20158;AGIWUR29689;AGIWUR34395;AGIWUR34689
CB4856	CT	II	11130275	11130698	WBGene00044917	F40F8.12	F40F8.12
-0.0956191055908455	AGIWUR40447
CB4856	CT	II	11130690	11132648	WBGene00044916	F40F8.11	F40F8.11
-0.0609394766429219	AGIWUR7480;AGIWUR13537;AGIWUR13618;AGIWUR40789;AGIWUR42894
CB4856	CT	II	11132861	11135140	WBGene00009578	F40F8.5	F40F8.5	0.0713271247437746
AGIWUR4445;AGIWUR33682
CB4856	CT	II	11139407	11140275	WBGene00009577	F40F8.4	F40F8.4	0.0577824480895694
AGIWUR8970;AGIWUR28271;AGIWUR29838;AGIWUR35430
CB4856	CT	II	11142751	11150381	WBGene00009579	arrd-6	F40F8.8	0.360510116923485
AGIWUR36363
CB4856	CT	II	11150299	11152600	WBGene00004096	pqm-1	F40F8.7	0.871819074196218
AGIWUR28055
CB4856	CT	II	11157094	11162481	WBGene00004227	ptr-13	K07C10.1
-0.179425574488696	AGIWUR42577
CB4856	CT	II	11171446	11175479	WBGene00009511	F37H8.2	F37H8.2	-0.32136930707979
AGIWUR28562
CB4856	CT	II	11181654	11183147	WBGene00009512	F37H8.3	F37H8.3	0.688218615681407
AGIWUR7905
CB4856	CT	II	11183075	11184807	WBGene00003046	lir-3	F37H8.1	-0.327339617904023
AGIWUR1936
CB4856	CT	II	11186028	11188080	WBGene00009513	sfxn-1.2	F37H8.4
0.145473088469101	AGIWUR24608
CB4856	CT	II	11188206	11189437	WBGene00009514	F37H8.5	F37H8.5	-0.404542175509544
AGIWUR7339
CB4856	CT	II	11203221	11220642	WBGene00011522	srap-1	T06D8.1	0.562154128868578
AGIWUR17063;AGIWUR23459;AGIWUR36933
CB4856	CT	II	11222305	11226548	WBGene00011524	T06D8.3	T06D8.3	0.177934895172917
AGIWUR21094
CB4856	CT	II	11226679	11232835	WBGene00011523	T06D8.2	T06D8.2	0.0136858272904696
AGIWUR9844;AGIWUR12839;AGIWUR36573
CB4856	CT	II	11232756	11234952	WBGene00011526	cox-15	T06D8.5	0.144632347418234
AGIWUR17356
CB4856	CT	II	11235053	11236450	WBGene00011527	cchl-1	T06D8.6	0.00619834987342482

AGIWUR20738;AGIWUR29488
CB4856 CT II 11236624 11237323 WBGene00011528 T06D8.7 T06D8.7 0.0047504857807823
AGIWUR41769
CB4856 CT II 11237447 11239092 WBGene00004465 rpn-9 T06D8.8 -0.256501663218558
AGIWUR3086;AGIWUR16274
CB4856 CT II 11239407 11240988 WBGene00011529 T06D8.9 T06D8.9 -0.135010827520206
AGIWUR7515;AGIWUR38105
CB4856 CT II 11240932 11247514 WBGene00011530 T06D8.10 T06D8.10
0.101983959175874 AGIWUR28942
CB4856 CT II 11257081 11258506 WBGene00011465 T05B9.2 T05B9.2 0.15783734986092
AGIWUR1889;AGIWUR27281;AGIWUR29593;AGIWUR34102;AGIWUR38410
CB4856 CT II 11260087 11261256 WBGene00011464 T05B9.1 T05B9.1 -0.321542450895194
AGIWUR5135
CB4856 CT II 11269124 11271599 WBGene00000851 cut-1 C47G2.1 -0.268005273263924
AGIWUR10983
CB4856 CT II 11281986 11283953 WBGene00006853 unc-130 C47G2.2 0.638605053656773
AGIWUR15293
CB4856 CT II 11288690 11290159 WBGene00008164 C47G2.3 C47G2.3 -0.0682500437583609
AGIWUR4536;AGIWUR5872
CB4856 CT II 11290843 11293606 WBGene00008165 C47G2.4 C47G2.4 -0.124474611801999
AGIWUR17801
CB4856 CT II 11293997 11294579 WBGene00044038 C47G2.6 C47G2.6 -0.202680338523835
AGIWUR25886
CB4856 CT II 11295563 11296019 WBGene00044039 C47G2.7 C47G2.7 -0.331180411137996
AGIWUR23223
CB4856 CT II 11297700 11301254 WBGene00008166 saps-1 C47G2.5 -0.145603320491862
AGIWUR26416;AGIWUR31317;AGIWUR39412
CB4856 CT II 11301359 11302783 WBGene00006414 raga-1 T24F1.1 -0.0778855130332907
AGIWUR32876
CB4856 CT II 11302891 11305683 WBGene00011994 samp-1 T24F1.2
-0.00474855177360212 AGIWUR30
CB4856 CT II 11306399 11311940 WBGene00011995 rsf-1 T24F1.3 0.476759891852721
AGIWUR16058
CB4856 CT II 11312460 11313346 WBGene00011999 T24F1.7 T24F1.7 -0.119167535945727
AGIWUR545
CB4856 CT II 11313331 11314265 WBGene00011996 T24F1.4 T24F1.4 -0.103727222485379
AGIWUR38428
CB4856 CT II 11319241 11319691 WBGene00011997 nlp-51 T24F1.5 1.02300043786755
AGIWUR32939

CB4856	CT	II	11323051	11329429	WBGene00007041	tag-180	T24F1.6	-0.124109017894769	AGIWUR3735;AGIWUR11884;AGIWUR43713
CB4856	CT	II	11336693	11337837	WBGene00005016	sqt-1	B0491.2	-0.329674927639315	AGIWUR5183
CB4856	CT	II	11338451	11339620	WBGene00007190	rmd-3	B0491.3	-0.0739488124093661	AGIWUR3162;AGIWUR18812;AGIWUR39848
CB4856	CT	II	11340134	11343530	WBGene00007191	lgc-20	B0491.4	-0.0818068601980748	AGIWUR19561
CB4856	CT	II	11343716	11345743	WBGene00007192	B0491.5	B0491.5	0.107624600174896	AGIWUR6670;AGIWUR9367
CB4856	CT	II	11345943	11348248	WBGene00007193	B0491.6	B0491.6	-0.095842434979275	AGIWUR12652
CB4856	CT	II	11348430	11349946	WBGene00007189	B0491.1	B0491.1	0.280051016129633	AGIWUR37232
CB4856	CT	II	11350192	11351373	WBGene00007194	B0491.7	B0491.7	-0.21749667789036	AGIWUR41619
CB4856	CT	II	11358447	11370311	WBGene00000529	clh-2	B0491.8	0.0116372731865373	AGIWUR16507;AGIWUR21676;AGIWUR31905;AGIWUR39721
CB4856	CT	II	11370315	11372145	WBGene00007891	C33B4.2	C33B4.2	-0.532818592823276	AGIWUR36998
CB4856	CT	II	11375608	11376269	WBGene00007892	C33B4.4	C33B4.4	-0.120674916163303	AGIWUR17076
CB4856	CT	II	11383327	11384570	WBGene00007893	C33B4.5	C33B4.5	-0.155957735105075	AGIWUR19815
CB4856	CT	II	11384594	11390862	WBGene00006444	shn-1	C33B4.3	0.789816048237403	AGIWUR34943
CB4856	CT	II	11410091	11412280	WBGene00044029	C05D12.7	C05D12.7	-0.146431338508973	AGIWUR1320;AGIWUR20111;AGIWUR26683;AGIWUR26954;AGIWUR33534
CB4856	CT	II	11412232	11415820	WBGene00007339	C05D12.1	C05D12.1	0.00251446225627738	AGIWUR12511
CB4856	CT	II	11418314	11423393	WBGene00007340	C05D12.2	C05D12.2	0.640762805237713	AGIWUR10538
CB4856	CT	II	11424830	11430607	WBGene00014666	C05D12.3	C05D12.3	-0.697386358516767	AGIWUR21963;AGIWUR23327
CB4856	CT	II	11431709	11432766	WBGene00007341	C05D12.4	C05D12.4	-0.274980878353002	AGIWUR6395
CB4856	CT	II	11434221	11434958	WBGene00007342	C05D12.5	C05D12.5	-0.0538845995246717	AGIWUR19686;AGIWUR25321;AGIWUR39345
CB4856	CT	II	11435212	11438464	WBGene00006915	vha-6	VW02B12L.1		

-0.549690364713518	AGIWUR891;AGIWUR8238;AGIWUR15957
CB4856	CT	II	11439177	11439762	WBGene00012155	VW02B12L.2	VW02B12L.2
0.0669136822644489	AGIWUR3395
CB4856	CT	II	11442693	11444009	WBGene00012156	ebp-2	VW02B12L.3
0.000511073297119857	AGIWUR7374;AGIWUR30744
CB4856	CT	II	11444162	11445255	WBGene00012157	adbp-1	VW02B12L.4
-0.227081684643979	AGIWUR1736
CB4856	CT	II	11445289	11448895	WBGene00043534	W02B12.13	W02B12.13
0.0680834527577865	AGIWUR23311;AGIWUR26362;AGIWUR40669
CB4856	CT	II	11448965	11451445	WBGene00012201	W02B12.1	W02B12.1
-0.0435154445697385	AGIWUR3778
CB4856	CT	II	11451444	11452955	WBGene00004699	rsp-2	W02B12.2
-0.139021989495667	AGIWUR7342;AGIWUR16886;AGIWUR19469;AGIWUR24287;AGIWUR28283
CB4856	CT	II	11453362	11455864	WBGene00004698	rsp-1	W02B12.3
-0.109886777321221	AGIWUR8808;AGIWUR19633;AGIWUR44613
CB4856	CT	II	11455759	11459356	WBGene00012202	W02B12.4	W02B12.4
0.0570994892806329	AGIWUR9639;AGIWUR25066;AGIWUR32512
CB4856	CT	II	11460215	11464129	WBGene00000196	aars-1	W02B12.6
-0.244604574469632	AGIWUR42057
CB4856	CT	II	11464892	11468653	WBGene00012203	rga-1	W02B12.8
0.0582861818473885	AGIWUR9836;AGIWUR17737;AGIWUR20910;AGIWUR37766
CB4856	CT	II	11468552	11469624	WBGene00044026	cisd-1	W02B12.15
-0.0487471663008154	AGIWUR9645;AGIWUR11244;AGIWUR30877;AGIWUR36766;AGIWUR43387
CB4856	CT	II	11470010	11472033	WBGene00012204	mfn-1	W02B12.9
0.252194887882492	AGIWUR7193;AGIWUR40221
CB4856	CT	II	11472135	11473571	WBGene00012205	W02B12.10	W02B12.10
-0.193786671125521	AGIWUR25960
CB4856	CT	II	11473861	11476019	WBGene00012206	W02B12.11	W02B12.11
-0.00924773521167666	AGIWUR16635;AGIWUR22977;AGIWUR44267
CB4856	CT	II	11476757	11479176	WBGene00012207	W02B12.12	W02B12.12
0.0728653150860001	AGIWUR13834;AGIWUR34835;AGIWUR35852
CB4856	CT	II	11480432	11482385	WBGene00007147	B0334.9	B0334.9	-0.45831029035898
AGIWUR3847
CB4856	CT	II	11483201	11485359	WBGene00006660	twk-5	B0334.2	-0.0278391468659631
AGIWUR24833
CB4856	CT	II	11485510	11486774	WBGene00007142	ttr-18	B0334.1	0.0883257602942162
AGIWUR35879
CB4856	CT	II	11488302	11489619	WBGene00007149	B0334.13	B0334.13
-0.625264743307392	AGIWUR2411

CB4856	CT	II	11489605	11493039	WBGene00007143	B0334.3	B0334.3	0.642767612361084
AGIWUR8770
CB4856	CT	II	11493231	11495321	WBGene00003868	ooc-3	B0334.11
-0.107339880815433	AGIWUR41973
CB4856	CT	II	11495692	11496663	WBGene00007144	B0334.4	B0334.4	-0.162276514130035
AGIWUR44324
CB4856	CT	II	11497665	11499620	WBGene00007145	B0334.5	B0334.5	0.0211421495619604
AGIWUR1708
CB4856	CT	II	11510673	11513890	WBGene00007146	B0334.6	B0334.6	-0.0337105318955579
AGIWUR18391
CB4856	CT	II	11515075	11518154	WBGene00005267	srh-44	B0334.7	0.0635457446197387
AGIWUR18781
CB4856	CT	II	11519323	11527062	WBGene00000090	age-1	B0334.8	-0.0604253382482351
AGIWUR6065;AGIWUR20151;AGIWUR40421
CB4856	CT	II	11527172	11533814	WBGene00007013	mdt-8	Y62F5A.1
-0.0111545422260253	AGIWUR10085;AGIWUR18350
CB4856	CT	II	11533925	11534479	WBGene00077453	Y62F5A.12	Y62F5A.12
-0.138100204445138	AGIWUR15635;AGIWUR22508;AGIWUR29852;AGIWUR30561;AGIWUR32531
CB4856	CT	II	11535165	11538841	WBGene00013387	Y62F5A.9	Y62F5A.9
0.383511860771884	AGIWUR2621
CB4856	CT	II	11538904	11540630	WBGene00010055	F54D5.12	F54D5.12
-0.253315991052034	AGIWUR4071;AGIWUR32091;AGIWUR43219
CB4856	CT	II	11540772	11542579	WBGene00010054	F54D5.11	F54D5.11
-0.0700842832638688	AGIWUR23057
CB4856	CT	II	11542636	11544875	WBGene00010053	F54D5.9	F54D5.9	0.0578223286531241
AGIWUR20809
CB4856	CT	II	11545563	11547366	WBGene00001031	dnj-13	F54D5.8	-0.0596768986873553
AGIWUR32433
CB4856	CT	II	11547481	11549313	WBGene00010052	F54D5.7	F54D5.7
-0.00950984543136801	AGIWUR16917;AGIWUR18203
CB4856	CT	II	11558715	11564227	WBGene00010057	F54D5.15	F54D5.15
-0.292301772493318	AGIWUR23389
CB4856	CT	II	11564385	11567020	WBGene00010051	F54D5.5	F54D5.5	0.0489456153947536
AGIWUR4180;AGIWUR8631
CB4856	CT	II	11569818	11571002	WBGene00010050	F54D5.4	F54D5.4	-0.376390328901243
AGIWUR25511;AGIWUR35726;AGIWUR43286
CB4856	CT	II	11571805	11573025	WBGene00010049	F54D5.3	F54D5.3	0.2209453743602
AGIWUR31038
CB4856	CT	II	11573180	11575000	WBGene00010048	F54D5.2	F54D5.2	-0.055481181442671

AGIWUR14382
CB4856 CT II 11574940 11577258 WBGene00003960 pcs-1 F54D5.1 0.0264200787844651
AGIWUR12093;AGIWUR26991;AGIWUR27917
CB4856 CT II 11578716 11582758 WBGene00010056 smc-6 F54D5.14
0.255588642744191 AGIWUR9293;AGIWUR28985;AGIWUR42540
CB4856 CT II 11582849 11588536 WBGene00008361 D1043.1 D1043.1 -0.0250310151548376
AGIWUR31695
CB4856 CT II 11588464 11592060 WBGene00008545 F07A11.1 F07A11.1
0.337510287867546 AGIWUR540
CB4856 CT II 11592392 11596590 WBGene00008546 gfat-1 F07A11.2
-0.229294812603545 AGIWUR7924
CB4856 CT II 11596767 11599809 WBGene00003791 npp-5 F07A11.3
-0.105878496220692 AGIWUR15789
CB4856 CT II 11603956 11611532 WBGene00008547 F07A11.4 F07A11.4
-0.25979697779643 AGIWUR16553
CB4856 CT II 11612257 11614391 WBGene00008548 F07A11.5 F07A11.5
-0.0811193270614829 AGIWUR34591
CB4856 CT II 11614636 11633609 WBGene00008549 din-1 F07A11.6
0.316429718340214
AGIWUR2384;AGIWUR5574;AGIWUR20351;AGIWUR26081;AGIWUR29000;AGIWUR30375;AGIWUR35211;AGIWUR35355;AGIWUR40727;A
GIWUR42354
CB4856 CT II 11640454 11641778 WBGene00002194 kin-6 ZK20.2 -0.294824568078583
AGIWUR20410
CB4856 CT II 11646747 11649139 WBGene00013923 ghi-1 ZK20.1 0.146367605721906
AGIWUR21946
CB4856 CT II 11649096 11651213 WBGene00013925 ZK20.4 ZK20.4 -0.354847807763061
AGIWUR28663
CB4856 CT II 11651261 11652906 WBGene00013924 rad-23 ZK20.3 -0.19739255365459
AGIWUR7698;AGIWUR12477;AGIWUR32141;AGIWUR39417
CB4856 CT II 11653096 11654194 WBGene00004468 rpn-12 ZK20.5 -0.225148258270472
AGIWUR27674
CB4856 CT II 11654214 11657172 WBGene00013926 nep-1 ZK20.6 -0.0920171124591356
AGIWUR2710
CB4856 CT II 11661583 11675927 WBGene00007028 trr-1 C47D12.1
0.174257986914302 AGIWUR5325;AGIWUR12751;AGIWUR31535
CB4856 CT II 11676306 11679197 WBGene00008136 C47D12.2 C47D12.2
-0.26042630091448 AGIWUR5784
CB4856 CT II 11679091 11681068 WBGene00008137 sfxn-1.4 C47D12.3
0.0929767924035936 AGIWUR19634

CB4856	CT	II	11681825	11682881	WBGene00008138	C47D12.4	C47D12.4
0.349823974488894	AGIWUR23593
CB4856	CT	II	11683479	11685289	WBGene00008139	C47D12.5	C47D12.5
0.177286022078842	AGIWUR40843
CB4856	CT	II	11696171	11705371	WBGene00008140	xpf-1	C47D12.8
0.183539353041411	AGIWUR41402
CB4856	CT	II	11705989	11713610	WBGene00012149	VF13D12L.3	VF13D12L.3
-0.012645926407167	AGIWUR3208
CB4856	CT	II	11713721	11716785	WBGene00008744	F13D12.10	F13D12.10
0.359962272369884	AGIWUR10883
CB4856	CT	II	11720444	11722308	WBGene00008739	F13D12.3	F13D12.3
-0.0516414446262772	AGIWUR43065
CB4856	CT	II	11724204	11726533	WBGene00002262	ldh-1	F13D12.2
-0.0229112397595905	AGIWUR13529
CB4856	CT	II	11726814	11728541	WBGene00008740	F13D12.5	F13D12.5
-0.173166224221461	AGIWUR26392
CB4856	CT	II	11728949	11731226	WBGene00000114	alh-8	F13D12.4
-0.0453022262559291
AGIWUR1224;AGIWUR5891;AGIWUR12029;AGIWUR14165;AGIWUR15757;AGIWUR16773;AGIWUR17567;AGIWUR18235;AGIWUR20123;A
GIWUR20401;AGIWUR22020;AGIWUR24172;AGIWUR24755;AGIWUR29296;AGIWUR34759;AGIWUR38206;AGIWUR41118;AGIWUR41506;
AGIWUR42887;AGIWUR43612;AGIWUR44212
CB4856	CT	II	11731165	11732594	WBGene00008742	F13D12.8	F13D12.8
-0.0484239026182287	AGIWUR8447
CB4856	CT	II	11739456	11742876	WBGene00008741	F13D12.6	F13D12.6
-0.0884592442686518	AGIWUR13795
CB4856	CT	II	11742814	11748611	WBGene00001679	gpb-1	F13D12.7
-0.0396966838324655
AGIWUR1044;AGIWUR6942;AGIWUR12617;AGIWUR16349;AGIWUR22503;AGIWUR22766;AGIWUR27763;AGIWUR37448;AGIWUR38234;A
GIWUR39837
CB4856	CT	II	11762166	11765662	WBGene00009690	F44E5.3	F44E5.3	0.562445141432045
AGIWUR39880
CB4856	CT	II	11771978	11774039	WBGene00206377	F44E5.15	F44E5.15
0.273892763746858	AGIWUR4924
CB4856	CT	II	11774144	11774559	WBGene00009688	F44E5.1	F44E5.1	-0.121382694687721
AGIWUR4219;AGIWUR6573;AGIWUR8599;AGIWUR12421;AGIWUR14398;AGIWUR25459;AGIWUR41823
CB4856	CT	II	11774669	11777943	WBGene00009661	patr-1	F43G6.9	-0.0294239858381289
AGIWUR8301;AGIWUR9463;AGIWUR35105
CB4856	CT	II	11778435	11782231	WBGene00001016	dna-2	F43G6.1	0.0476838464018279
AGIWUR42987

CB4856	CT	II	11782987	11784086	WBGene00009656	F43G6.3	F43G6.3	0.0613751633288686	AGIWUR7939;AGIWUR9994;AGIWUR39798
CB4856	CT	II	11790759	11797322	WBGene00009657	F43G6.4	F43G6.4	0.555538376979085	AGIWUR3169;AGIWUR32275
CB4856	CT	II	11806567	11808805	WBGene00005013	jmjd-1.1	F43G6.6	-0.0131081189596571	AGIWUR21925
CB4856	CT	II	11812294	11814113	WBGene00009660	F43G6.8	F43G6.8	0.784694697758962	AGIWUR840
CB4856	CT	II	11814839	11816706	WBGene00009658	F43G6.5	F43G6.5	-0.0385467064804832	AGIWUR34108
CB4856	CT	II	11818248	11819924	WBGene00012396	Y6D1A.2	Y6D1A.2	0.422376890099708	AGIWUR3568
CB4856	CT	II	11827933	11830020	WBGene00003979	pes-5	T27D12.4	-0.196516341492419	AGIWUR42789
CB4856	CT	II	11831062	11832575	WBGene00012086	clec-144	T27D12.3	-0.0616245911291529	AGIWUR3999
CB4856	CT	II	11842617	11846026	WBGene00012085	T27D12.1	T27D12.1	0.27261260279208	AGIWUR43883
CB4856	CT	II	11846508	11854743	WBGene00000528	clh-1	T27D12.2	-0.0329852142040021	AGIWUR11100;AGIWUR27732;AGIWUR44823
CB4856	CT	II	11861029	11863420	WBGene00010787	K12D12.5	K12D12.5	-0.384235178944323	AGIWUR27714
CB4856	CT	II	11863525	11865506	WBGene00010786	K12D12.4	K12D12.4	0.132343370930258	AGIWUR32968
CB4856	CT	II	11865523	11874702	WBGene00003789	npp-3	K12D12.2	0.0822334747446654	AGIWUR28569
CB4856	CT	II	11875145	11880588	WBGene00010785	top-2	K12D12.1	0.0788807703235843	AGIWUR7051;AGIWUR16104;AGIWUR21962;AGIWUR37958;AGIWUR39538;AGIWUR40938;AGIWUR41350
CB4856	CT	II	11882614	11883685	WBGene00014155	ZK930.5	ZK930.5	-0.232432965229151	AGIWUR19690
CB4856	CT	II	11885588	11886601	WBGene00014156	ZK930.6	ZK930.6	0.332503583149688	AGIWUR43651
CB4856	CT	II	11890992	11893423	WBGene00014152	ZK930.2	ZK930.2	1.16998925716826	AGIWUR42824
CB4856	CT	II	11897365	11901210	WBGene00014153	vab-23	ZK930.3	0.357918966498268	AGIWUR3989;AGIWUR8215;AGIWUR15786
CB4856	CT	II	11906517	11908601	WBGene00014154	ZK930.4	ZK930.4	0.041219716841308	AGIWUR34030
CB4856	CT	II	11908714	11915852	WBGene00014151	vps-15	ZK930.1	0.0227906770264035	

AGIWUR6792
CB4856 CT II 11916513 11917643 WBGene00001977 hmg-12 Y17G7A.1
-0.24633439978076 AGIWUR3120;AGIWUR4269;AGIWUR7020;AGIWUR18198;AGIWUR21727;AGIWUR26983
CB4856 CT II 11917642 11935343 WBGene00003015 lin-29 W03C9.4 -0.220260598132854
AGIWUR21089;AGIWUR27180
CB4856 CT II 11948727 11949474 WBGene00012223 W03C9.8 W03C9.8 -0.115674396215272
AGIWUR29013
CB4856 CT II 11953239 11960743 WBGene00012222 W03C9.6 W03C9.6
-0.00911019189793194 AGIWUR32907
CB4856 CT II 11960989 11962128 WBGene00012219 W03C9.1 W03C9.1 -0.372350417300325
AGIWUR38306
CB4856 CT II 11962111 11965258 WBGene00012220 W03C9.2 W03C9.2 -0.139971217134752
AGIWUR29253
CB4856 CT II 11965339 11966931 WBGene00004271 rab-7 W03C9.3 -0.0446119556365989
AGIWUR4192;AGIWUR4526;AGIWUR8098;AGIWUR17129;AGIWUR22447;AGIWUR23927;AGIWUR25730;AGIWUR33425
CB4856 CT II 11967048 11967533 WBGene00012221 W03C9.5 W03C9.5 0.0629488376381282
AGIWUR2780;AGIWUR8954;AGIWUR14848;AGIWUR18824;AGIWUR24306;AGIWUR42497
CB4856 CT II 11968637 11972334 WBGene00003228 mex-1 W03C9.7 0.172899014293485
AGIWUR43188
CB4856 CT II 11972310 11973732 WBGene00012457 acbp-6 Y17G7B.1
0.107290185321343 AGIWUR920;AGIWUR15194;AGIWUR16203;AGIWUR37119;AGIWUR38415;AGIWUR39380
CB4856 CT II 11975190 11977659 WBGene00012472 Y17G7B.21 Y17G7B.21
-0.052892009700539 AGIWUR31694
CB4856 CT II 11977784 11979859 WBGene00012471 Y17G7B.20 Y17G7B.20
0.0855146924943355 AGIWUR10903
CB4856 CT II 11979838 11989017 WBGene00012458 ash-2 Y17G7B.2
0.0288362499889558 AGIWUR10526;AGIWUR16389;AGIWUR28205;AGIWUR36922;AGIWUR39383
CB4856 CT II 11989024 11992823 WBGene00012459 Y17G7B.3 Y17G7B.3
-0.0482874183248896 AGIWUR1664
CB4856 CT II 11993001 11998009 WBGene00012460 dhps-1 Y17G7B.4
-0.0839755893151057 AGIWUR8348
CB4856 CT II 11999260 12001422 WBGene00044030 Y17G7B.23 Y17G7B.23
-0.222215376754164 AGIWUR22822
CB4856 CT II 12001510 12006625 WBGene00003154 mcm-2 Y17G7B.5
0.0458627398521605
AGIWUR6431;AGIWUR14362;AGIWUR22034;AGIWUR22863;AGIWUR24320;AGIWUR27510;AGIWUR31581;AGIWUR43987
CB4856 CT II 12009077 12023825 WBGene00012461 glb-29 Y17G7B.6
-0.120705868993364 AGIWUR14390;AGIWUR23983;AGIWUR25092;AGIWUR26552;AGIWUR34236;AGIWUR39129
CB4856 CT II 12023861 12025932 WBGene00012465 Y17G7B.12 Y17G7B.12

0.0324751828180609 AGIWUR29467
CB4856 CT II 12028763 12036742 WBGene00000892 cyn-16 Y17G7B.9
-0.221428373820528 AGIWUR4024;AGIWUR23109
CB4856 CT II 12036842 12039139 WBGene00006601 tpi-1 Y17G7B.7
0.0619059708824059 AGIWUR1217;AGIWUR2688;AGIWUR4294;AGIWUR7287;AGIWUR8391;AGIWUR11326
CB4856 CT II 12042910 12049260 WBGene00012466 ippk-1 Y17G7B.13
0.158756035745583 AGIWUR17013;AGIWUR20129;AGIWUR22244;AGIWUR29786;AGIWUR35371;AGIWUR35857
CB4856 CT II 12049583 12059866 WBGene00012463 Y17G7B.10 Y17G7B.10
0.137949578593215 AGIWUR3151;AGIWUR18392;AGIWUR21813;AGIWUR22929;AGIWUR30218;AGIWUR30266
CB4856 CT II 12060228 12062621 WBGene00012464 arrd-7 Y17G7B.11
-0.345585166647515 AGIWUR8573
CB4856 CT II 12065085 12067568 WBGene00012467 arrd-8 Y17G7B.14
-0.0448876127911069 AGIWUR8442
CB4856 CT II 12068099 12070675 WBGene00012473 Y17G7B.22 Y17G7B.22
0.156006920766412 AGIWUR5117;AGIWUR30056;AGIWUR39044
CB4856 CT II 12072082 12088868 WBGene00000565 cnt-1 Y17G7B.15
0.18093677211632 AGIWUR32973
CB4856 CT II 12092422 12099989 WBGene00012468 Y17G7B.17 Y17G7B.17
0.0502810524892894 AGIWUR39390
CB4856 CT II 12103331 12107857 WBGene00012469 Y17G7B.18 Y17G7B.18
0.0811748193484298 AGIWUR21167;AGIWUR32919
CB4856 CT II 12108273 12112552 WBGene00012470 Y17G7B.19 Y17G7B.19
0.12760126967977 AGIWUR15320
CB4856 CT II 12114971 12130968 WBGene00012845 set-24 Y43F11A.5
-0.318900482484249 AGIWUR1402;AGIWUR10026;AGIWUR26909;AGIWUR33032;AGIWUR43098
CB4856 CT II 12131530 12132894 WBGene00014576 Y43F11A.6 Y43F11A.6
-0.0104204509066292 AGIWUR3471;AGIWUR10296
CB4856 CT II 12133535 12147134 WBGene00013246 Y57A10A.1 Y57A10A.1
-0.35794612029801 AGIWUR4370
CB4856 CT II 12147633 12150216 WBGene00013247 Y57A10A.2 Y57A10A.2
-0.0759177926008725 AGIWUR26512
CB4856 CT II 12154486 12156812 WBGene00013250 Y57A10A.5 Y57A10A.5
-0.13200812735629 AGIWUR27774
CB4856 CT II 12156940 12159252 WBGene00013251 Y57A10A.6 Y57A10A.6
0.104132997820146 AGIWUR7730
CB4856 CT II 12159472 12162671 WBGene00013252 Y57A10A.7 Y57A10A.7
0.0713924706922295 AGIWUR6939
CB4856 CT II 12164839 12170762 WBGene00013253 Y57A10A.8 Y57A10A.8
-0.131834717176886 AGIWUR22872

CB4856	CT	II	12172571	12176069	WBGene00004394	rol-1	Y57A10A.11
-0.212194411459134	AGIWUR34088
CB4856	CT	II	12176880	12184875	WBGene00013256	Y57A10A.13	Y57A10A.13
-0.321037471991614	AGIWUR5797
CB4856	CT	II	12186007	12189265	WBGene00013257	Y57A10A.14	Y57A10A.14
-0.239478583586387	AGIWUR43593
CB4856	CT	II	12189795	12199959	WBGene00013258	polg-1	Y57A10A.15
-0.107741361657723	AGIWUR23407
CB4856	CT	II	12200607	12202381	WBGene00013259	trpp-5	Y57A10A.16
-0.0200919371417392	AGIWUR40094
CB4856	CT	II	12203309	12209160	WBGene00013272	aqp-12	Y57A10A.35
0.0789557730154726	AGIWUR11399
CB4856	CT	II	12209228	12225086	WBGene00004167	pqn-87	Y57A10A.18
0.0285963525231807	AGIWUR40602
CB4856	CT	II	12225356	12227265	WBGene00013260	rsr-2	Y57A10A.19
-0.374467538843003	AGIWUR30536
CB4856	CT	II	12228305	12237051	WBGene00013261	sinh-1	Y57A10A.20
-0.10079243296202	AGIWUR11106;AGIWUR29352;AGIWUR30256;AGIWUR35620;AGIWUR42257
CB4856	CT	II	12240388	12242567	WBGene00013263	Y57A10A.23	Y57A10A.23
-0.0738228051579597	AGIWUR11981
CB4856	CT	II	12242946	12247611	WBGene00013264	Y57A10A.24	Y57A10A.24
0.303457216276298	AGIWUR40090
CB4856	CT	II	12248125	12249135	WBGene00013254	tag-276	Y57A10A.9
-0.35670416762601	AGIWUR11305
CB4856	CT	II	12251667	12256628	WBGene00013265	parn-2	Y57A10A.25
-0.158114684241358	AGIWUR36791
CB4856	CT	II	12256950	12269706	WBGene00013255	Y57A10A.10	Y57A10A.10
-0.324219552119153	AGIWUR16522
CB4856	CT	II	12269815	12275044	WBGene00013267	Y57A10A.27	Y57A10A.27
-0.0196750967649066	AGIWUR37762
CB4856	CT	II	12275936	12279383	WBGene00013268	Y57A10A.28	Y57A10A.28
-0.299313278831161	AGIWUR8915
CB4856	CT	II	12279561	12280595	WBGene00013269	Y57A10A.29	Y57A10A.29
-0.058506962934251	AGIWUR12646
CB4856	CT	II	12280662	12284133	WBGene00002063	ife-5	Y57A10A.30
-0.28492103142916	AGIWUR30348
CB4856	CT	II	12289385	12300573	WBGene00013270	Y57A10A.31	Y57A10A.31
0.0661033143718535	AGIWUR15857;AGIWUR44592
CB4856	CT	II	12301077	12302429	WBGene00016825	C50E10.1	C50E10.1

0.524096778565177	AGIWUR13445
CB4856	CT	II	12303525	12305132	WBGene00016834	sre-50	C50E10.11
0.116776733149591	AGIWUR26514
CB4856	CT	II	12311860	12313660	WBGene00016833	sre-51	C50E10.10
0.000927466039651215	AGIWUR26368
CB4856	CT	II	12318841	12321070	WBGene00016832	sre-49	C50E10.9
-0.0161876399811067	AGIWUR24431
CB4856	CT	II	12321193	12323405	WBGene00016827	sre-53	C50E10.3
-0.206925217972323	AGIWUR15154
CB4856	CT	II	12325544	12327514	WBGene00016831	sre-56	C50E10.8
0.122382608091829	AGIWUR15130
CB4856	CT	II	12329294	12331122	WBGene00016830	sre-55	C50E10.7
0.308226606465343	AGIWUR35147
CB4856	CT	II	12332924	12335044	WBGene00016829	sre-54	C50E10.6
-0.167633883312847	AGIWUR21803
CB4856	CT	II	12335730	12338093	WBGene00016828	sre-52	C50E10.5
0.0481690877492111	AGIWUR10613
CB4856	CT	II	12339944	12344795	WBGene00004945	sop-2	C50E10.4
0.30308383843413	AGIWUR34187
CB4856	CT	II	12345285	12352243	WBGene00004310	ras-1	C44C11.1
0.267236570551194	AGIWUR23461;AGIWUR41923
CB4856	CT	II	12358756	12367023	WBGene00013273	marc-5	Y57A10B.1
0.319515663465468	AGIWUR22652
CB4856	CT	II	12373522	12379511	WBGene00013274	Y57A10B.2	Y57A10B.2
0.416429067135167	AGIWUR19618
CB4856	CT	II	12383463	12384692	WBGene00013275	btb-14	Y57A10B.3
-0.409744345753099	AGIWUR16330;AGIWUR18091;AGIWUR33933
CB4856	CT	II	12384593	12386881	WBGene00013276	sre-42	Y57A10B.4
0.28901090036837	AGIWUR5074
CB4856	CT	II	12388293	12389839	WBGene00013277	sre-41	Y57A10B.5
-0.285701286025458	AGIWUR11779
CB4856	CT	II	12390083	12392662	WBGene00013278	Y57A10B.6	Y57A10B.6
-0.197501079761604	AGIWUR7958
CB4856	CT	II	12392701	12393743	WBGene00013279	Y57A10B.7	Y57A10B.7
0.0520339665668992	AGIWUR36071
CB4856	CT	II	12394856	12397756	WBGene00044048	F57G9.7	F57G9.7	0.14601174087336
AGIWUR25016;AGIWUR42871
CB4856	CT	II	12399399	12401891	WBGene00010220	sre-29	F57G9.4	0.180177617810646
AGIWUR10397

CB4856	CT	II	12402434	12404887	WBGene00044041	F57G9.6	F57G9.6	-0.0858700124489005	AGIWUR4855;AGIWUR19927;AGIWUR33180
CB4856	CT	II	12404951	12406494	WBGene00010218	sre-30	F57G9.2	-0.147764954036277	AGIWUR12686
CB4856	CT	II	12406822	12408817	WBGene00010217	sre-31	F57G9.1	-0.456863644944494	AGIWUR19812
CB4856	CT	II	12409320	12410207	WBGene00010219	F57G9.3	F57G9.3	0.159733184049536	AGIWUR30827
CB4856	CT	II	12411622	12412517	WBGene00013280	Y57A10C.1	Y57A10C.1	0.288024980432661	AGIWUR20533
CB4856	CT	II	12412723	12412957	WBGene00044052	Y57A10C.12	Y57A10C.12	0.0269833457935551	AGIWUR4626;AGIWUR12188;AGIWUR26299;AGIWUR31376;AGIWUR36187
CB4856	CT	II	12414036	12414905	WBGene00044047	Y57A10C.11	Y57A10C.11	0.310317383837636	AGIWUR3841;AGIWUR14115
CB4856	CT	II	12415527	12417527	WBGene00013281	sre-27	Y57A10C.3	0.152062631666794	AGIWUR5752
CB4856	CT	II	12419133	12420586	WBGene00013282	sre-26	Y57A10C.4	0.153303584697735	AGIWUR220
CB4856	CT	II	12421204	12422623	WBGene00013283	sre-25	Y57A10C.5	0.648532273492434	AGIWUR38853
CB4856	CT	II	12423514	12425694	WBGene00013284	daf-22	Y57A10C.6	0.117312135319258	AGIWUR5216
CB4856	CT	II	12427986	12429946	WBGene00013286	Y57A10C.8	Y57A10C.8	-0.110250233627951	AGIWUR24902
CB4856	CT	II	12430788	12432311	WBGene00013285	dct-12	Y57A10C.7	-0.254200307162172	AGIWUR17519
CB4856	CT	II	12433608	12435846	WBGene00013287	Y57A10C.9	Y57A10C.9	0.437990243052599	AGIWUR21893
CB4856	CT	II	12442142	12442367	WBGene00012284	W05H5.2	W05H5.2	-0.0717272903758506	AGIWUR19090
CB4856	CT	II	12443119	12444779	WBGene00012283	W05H5.1	W05H5.1	-0.0464580382553592	AGIWUR12242
CB4856	CT	II	12445332	12447769	WBGene00012285	pitr-6	W05H5.3	0.525989061400125	AGIWUR13105;AGIWUR15362;AGIWUR30498
CB4856	CT	II	12448541	12449887	WBGene00005250	srh-27	W05H5.4	-0.454787030228223	AGIWUR3233
CB4856	CT	II	12450149	12451407	WBGene00043058	sre-34	W05H5.8	-0.154150557613578	AGIWUR10523
CB4856	CT	II	12453302	12454693	WBGene00012287	sre-33	W05H5.6	0.226144995745845	

AGIWUR3814
CB4856	CT	II	12454954	12456733	WBGene00012288	sre-32	W05H5.7	0.301403280134472
AGIWUR4582
CB4856	CT	II	12457575	12459510	WBGene00008837	sre-38	F15A4.1	-0.0415923888647276
AGIWUR35287
CB4856	CT	II	12459772	12460551	WBGene00044323	tag-281	F15A4.11
-0.26642306324479	AGIWUR7257;AGIWUR25748;AGIWUR28954;AGIWUR31949
CB4856	CT	II	12460641	12461471	WBGene00044322	tag-280	F15A4.12
0.0457532292917868	AGIWUR18364
CB4856	CT	II	12462418	12464078	WBGene00008838	F15A4.2	F15A4.2	-0.131533516417184
AGIWUR36102
CB4856	CT	II	12465872	12467519	WBGene00008839	sre-37	F15A4.3	0.118521322833242
AGIWUR20272
CB4856	CT	II	12467732	12469369	WBGene00005173	srg-16	F15A4.4	-0.0845360566029605
AGIWUR3092
CB4856	CT	II	12469374	12471211	WBGene00008840	F15A4.5	F15A4.5	-0.0446825793626289
AGIWUR32638
CB4856	CT	II	12471805	12473084	WBGene00008841	F15A4.6	F15A4.6	0.318094305142871
AGIWUR44991
CB4856	CT	II	12473706	12475234	WBGene00005174	srg-17	F15A4.7	-0.0852607616903915
AGIWUR23534
CB4856	CT	II	12475514	12478917	WBGene00008842	chil-28	F15A4.8	-0.0735010721184048
AGIWUR2093;AGIWUR3251;AGIWUR33896
CB4856	CT	II	12479590	12480910	WBGene00008843	arrd-9	F15A4.9	-0.232092389383959
AGIWUR34011
CB4856	CT	II	12482931	12484279	WBGene00008847	fbxb-102	F15A4.13
0.322510465372073	AGIWUR8569
CB4856	CT	II	12484544	12485160	WBGene00008844	F15A4.10	F15A4.10
-0.169532057677456	AGIWUR7846
CB4856	CT	II	12490214	12491815	WBGene00043054	T21B4.15	T21B4.15
0.152544505192157	AGIWUR19166;AGIWUR22620;AGIWUR28953
CB4856	CT	II	12492398	12493623	WBGene00011876	srz-6	T21B4.1	0.410278158498484
AGIWUR12887
CB4856	CT	II	12493800	12495106	WBGene00000660	col-85	T21B4.2	-0.340738153402167
AGIWUR28426
CB4856	CT	II	12495487	12496387	WBGene00011877	T21B4.3	T21B4.3	0.0944280894192486
AGIWUR379
CB4856	CT	II	12497217	12502878	WBGene00011878	ador-1	T21B4.4	0.430306328286047
AGIWUR925;AGIWUR19024

CB4856 CT II 12507338 12508452 WBGene00005289 srh-68 T21B4.5 0.120092711370486
AGIWUR27004
CB4856 CT II 12508950 12510135 WBGene00005288 srh-67 T21B4.6 -0.0773581794742855
AGIWUR19619
CB4856 CT II 12510682 12511814 WBGene00005287 srh-66 T21B4.10
-0.204542527589734 AGIWUR21685
CB4856 CT II 12512166 12513316 WBGene00005294 srh-73 T21B4.14
-0.147202552166259 AGIWUR29018
CB4856 CT II 12513618 12514715 WBGene00005290 srh-69 T21B4.7 0.0186381878272082
AGIWUR18370
CB4856 CT II 12514972 12516413 WBGene00005283 srh-61 T21B4.8 0.067695363201123
AGIWUR32493
CB4856 CT II 12516753 12517858 WBGene00005291 srh-70 T21B4.9
-0.00476347053308235 AGIWUR1997
CB4856 CT II 12520004 12524478 WBGene00044032 ceh-99 T21B4.17
-0.356474125168024 AGIWUR22947
CB4856 CT II 12525828 12527868 WBGene00006009 srx-118 T21B4.12
0.126270218331655 AGIWUR15515
CB4856 CT II 12528247 12529746 WBGene00006010 srx-119 F49C5.1 -0.105490121185622
AGIWUR7947
CB4856 CT II 12531258 12532977 WBGene00006011 srx-120 F49C5.2 0.00761201021403313
AGIWUR44058
CB4856 CT II 12535760 12542569 WBGene00009867 F49C5.4 F49C5.4 -0.456742529058517
AGIWUR1250;AGIWUR15649;AGIWUR16749;AGIWUR42084
CB4856 CT II 12542754 12544633 WBGene00044031 F49C5.9 F49C5.9 -0.445445921808039
AGIWUR19466;AGIWUR27810
CB4856 CT II 12546030 12546513 WBGene00009868 F49C5.5 F49C5.5 -0.126394571794195
AGIWUR5768
CB4856 CT II 12546761 12548249 WBGene00044162 F49C5.10 F49C5.10
0.128940878648759 AGIWUR8807;AGIWUR9074
CB4856 CT II 12555884 12557129 WBGene00044163 F49C5.11 F49C5.11
-0.357491791133206
AGIWUR1347;AGIWUR3489;AGIWUR3872;AGIWUR3883;AGIWUR6795;AGIWUR7306;AGIWUR11761;AGIWUR12963;AGIWUR20946;AGIWU
R22899;AGIWUR26088;AGIWUR31262;AGIWUR39486;AGIWUR44001
CB4856 CT II 12564185 12565943 WBGene00006254 str-223 F49C5.6 -0.681664404385632
AGIWUR36858
CB4856 CT II 12566165 12568297 WBGene00009869 F49C5.7 F49C5.7 0.119059937888439
AGIWUR10009
CB4856 CT II 12570198 12570623 WBGene00012579 Y38E10A.1 Y38E10A.1

-0.395013378177461	AGIWUR18619
CB4856	CT	II	12571572	12573529	WBGene00012580	Y38E10A.2	Y38E10A.2
0.118299042523545	AGIWUR4355
CB4856	CT	II	12573940	12574768	WBGene00012581	Y38E10A.3	Y38E10A.3
-0.437430382276853	AGIWUR18800
CB4856	CT	II	12575691	12577748	WBGene00012582	clec-8	Y38E10A.4
0.242088541481242	AGIWUR5171;AGIWUR8397;AGIWUR26584;AGIWUR28388;AGIWUR36068;AGIWUR41478
CB4856	CT	II	12578356	12581098	WBGene00012583	clec-4	Y38E10A.5
-0.72848939156248	AGIWUR23230
CB4856	CT	II	12582179	12603680	WBGene00012584	ceh-100	Y38E10A.6
0.0429346805090933	AGIWUR17963
CB4856	CT	II	12605968	12607027	WBGene00012585	lips-15	Y38E10A.7
0.181944156992646	AGIWUR14642;AGIWUR19800
CB4856	CT	II	12607200	12608788	WBGene00012586	Y38E10A.8	Y38E10A.8
0.14295486340329	AGIWUR40371
CB4856	CT	II	12608953	12609630	WBGene00012587	Y38E10A.9	Y38E10A.9
0.77000166510281	AGIWUR17764
CB4856	CT	II	12610313	12612357	WBGene00012588	lips-16	Y38E10A.10
-0.499880400851234	AGIWUR39472
CB4856	CT	II	12613564	12613939	WBGene00012589	nspe-4	Y38E10A.11
0.298870989518484	AGIWUR11054;AGIWUR13607;AGIWUR20167;AGIWUR22819;AGIWUR26536;AGIWUR41154
CB4856	CT	II	12615897	12616166	WBGene00012603	nspe-6	Y38E10A.25
0.524929812148802	AGIWUR27393
CB4856	CT	II	12617886	12618209	WBGene00012590	nspe-3	Y38E10A.12
-0.812410949181482	AGIWUR8742
CB4856	CT	II	12620548	12620888	WBGene00012591	nspe-1	Y38E10A.13
-0.439908700970829	AGIWUR7082
CB4856	CT	II	12623215	12624506	WBGene00012592	Y38E10A.14	Y38E10A.14
-0.667000100075629	AGIWUR14522;AGIWUR18602;AGIWUR33881
CB4856	CT	II	12627187	12627570	WBGene00012593	nspe-7	Y38E10A.15
-1.31237411626242	AGIWUR13856
CB4856	CT	II	12628936	12629380	WBGene00012594	nspe-5	Y38E10A.16
-0.47144790585833	AGIWUR8167
CB4856	CT	II	12629687	12631995	WBGene00044034	Y38E10A.28	Y38E10A.28
0.228640418923192	AGIWUR22063
CB4856	CT	II	12632593	12634799	WBGene00012595	Y38E10A.17	Y38E10A.17
-0.0643359322834627	AGIWUR10282
CB4856	CT	II	12636699	12638448	WBGene00012596	nhr-234	Y38E10A.18
0.269626147262684	AGIWUR6486;AGIWUR13527;AGIWUR40928

CB4856	CT	II	12640670	12642740	WBGene00012597	nhr-235	Y38E10A.19	-0.0601387885811242	AGIWUR37120
CB4856	CT	II	12662759	12671920	WBGene00004347	rgs-4	Y38E10A.21	0.110798241373951	AGIWUR13627
CB4856	CT	II	12673166	12677803	WBGene00012600	Y38E10A.22	Y38E10A.22	-0.593342374397254	AGIWUR14395;AGIWUR15734
CB4856	CT	II	12679035	12679413	WBGene00012602	Y38E10A.24	Y38E10A.24	0.0828637569222792	AGIWUR20712
CB4856	CT	II	12679954	12686713	WBGene00012895	cox-10	Y46G5A.2	-0.414709046846129	AGIWUR34531
CB4856	CT	II	12694412	12725483	WBGene00012894	tbc-17	Y46G5A.1	0.0726520271242652	AGIWUR733;AGIWUR2958;AGIWUR13448
CB4856	CT	II	12726963	12736910	WBGene00012896	snrp-200	Y46G5A.4	0.237305076767861	AGIWUR35032
CB4856	CT	II	12737032	12738334	WBGene00012897	pisy-1	Y46G5A.5	-0.151126014803735	AGIWUR12144
CB4856	CT	II	12742546	12744256	WBGene00077636	Y46G5A.43	Y46G5A.43	0.102467461116868	AGIWUR35875
CB4856	CT	II	12752854	12753813	WBGene00044164	Y46G5A.39	Y46G5A.39	-0.151774500088401	AGIWUR7494;AGIWUR40442
CB4856	CT	II	12754610	12754877	WBGene00077634	Y46G5A.41	Y46G5A.41	0.0872206587433695	AGIWUR17785
CB4856	CT	II	12756353	12757677	WBGene00012900	Y46G5A.8	Y46G5A.8	-0.0440355375884964	AGIWUR8892
CB4856	CT	II	12760797	12767232	WBGene00012901	cnp-2	Y46G5A.10	-0.146192070356431	AGIWUR11892;AGIWUR14337
CB4856	CT	II	12767221	12768217	WBGene00012903	vps-2	Y46G5A.12	0.0582268233414496	AGIWUR25006
CB4856	CT	II	12768686	12773903	WBGene00012904	tiar-2	Y46G5A.13	-0.125690036283459	AGIWUR40126
CB4856	CT	II	12774646	12781705	WBGene00012919	Y46G5A.34	Y46G5A.34	0.0354505258688533	AGIWUR34502
CB4856	CT	II	12782751	12785412	WBGene00012906	Y46G5A.15	Y46G5A.15	0.0249413437207515	AGIWUR19252
CB4856	CT	II	12786322	12788153	WBGene00012920	Y46G5A.35	Y46G5A.35	-0.060814175554583	AGIWUR1477
CB4856	CT	II	12788252	12796452	WBGene00012907	cpt-1	Y46G5A.17	0.336614828579864	AGIWUR9426;AGIWUR17814;AGIWUR24235;AGIWUR34373
CB4856	CT	II	12800449	12805383	WBGene00012908	Y46G5A.18	Y46G5A.18		

0.0468480991110667 AGIWUR2765;AGIWUR7180;AGIWUR7666;AGIWUR13153;AGIWUR35086;AGIWUR44753
CB4856 CT II 12804862 12810872 WBGene00012909 spds-1 Y46G5A.19
-0.210935599336418 AGIWUR24011
CB4856 CT II 12811009 12811934 WBGene00012910 Y46G5A.20 Y46G5A.20
0.678457822385603 AGIWUR1526;AGIWUR11175;AGIWUR20331;AGIWUR20731;AGIWUR23358;AGIWUR29839
CB4856 CT II 12811802 12816462 WBGene00012911 acl-7 Y46G5A.21
0.62673026333891 AGIWUR8222
CB4856 CT II 12816820 12818588 WBGene00012912 Y46G5A.22 Y46G5A.22
-0.0252875187322661 AGIWUR29260;AGIWUR39522
CB4856 CT II 12823649 12824177 WBGene00012921 Y46G5A.36 Y46G5A.36
0.057701432857451 AGIWUR40412
CB4856 CT II 12825546 12825833 WBGene00044042 Y46G5A.37 Y46G5A.37
-0.151955078787184 AGIWUR4500;AGIWUR9007;AGIWUR9678;AGIWUR17026;AGIWUR33153
CB4856 CT II 12827032 12827519 WBGene00044043 Y46G5A.38 Y46G5A.38
0.0944361654844252 AGIWUR15102;AGIWUR25500;AGIWUR37081;AGIWUR40350;AGIWUR44577
CB4856 CT II 12830707 12832720 WBGene00012914 bcmo-1 Y46G5A.24
0.130035124257255 AGIWUR15666
CB4856 CT II 12832915 12835824 WBGene00004903 snf-4 Y46G5A.25
0.154621499138738 AGIWUR35822
CB4856 CT II 12836548 12840157 WBGene00012915 lgc-35 Y46G5A.26
-0.330746867129071 AGIWUR22436;AGIWUR38636
CB4856 CT II 12846203 12847836 WBGene00003983 pes-10 Y46G5A.27
-0.0926971655196187 AGIWUR14951
CB4856 CT II 12856200 12862058 WBGene00012917 Y46G5A.29 Y46G5A.29
-0.728542527988185 AGIWUR21330
CB4856 CT II 12861963 12865883 WBGene00004904 snf-5 Y46G5A.30
0.143399294918385 AGIWUR7331
CB4856 CT II 12866448 12878116 WBGene00001793 gsy-1 Y46G5A.31
-0.0695295131732472 AGIWUR37228
CB4856 CT II 12880815 12881968 WBGene00009390 F35C5.1 F35C5.1 0.165142132888576
AGIWUR36217
CB4856 CT II 12885276 12888606 WBGene00005040 sra-14 F35C5.2 -0.121839424502172
AGIWUR40027
CB4856 CT II 12888762 12890460 WBGene00009391 F35C5.3 F35C5.3 0.111333103617195
AGIWUR32174
CB4856 CT II 12890630 12892024 WBGene00009392 F35C5.4 F35C5.4 -0.0751331210420894
AGIWUR25875
CB4856 CT II 12893065 12895098 WBGene00009393 clec-62 F35C5.5 0.252376410259016
AGIWUR31973

CB4856	CT	II	12895638	12897462	WBGene00009394	clec-63 F35C5.6 0.0783123854252352
AGIWUR2709;AGIWUR9580;AGIWUR9637;AGIWUR12263;AGIWUR24969;AGIWUR26563;AGIWUR27914;AGIWUR39707;AGIWUR40319
CB4856	CT	II	12898242	12900435	WBGene00009395	clec-64 F35C5.7 -0.255849819115014
AGIWUR25751
CB4856	CT	II	12901264	12903336	WBGene00009396	clec-65 F35C5.8 -0.081868778852316
AGIWUR13763
CB4856	CT	II	12904398	12906686	WBGene00009397	clec-66 F35C5.9 -0.29314884908596
AGIWUR15704;AGIWUR25952
CB4856	CT	II	12907370	12907783	WBGene00009398	nspb-11 F35C5.10
-0.296456144618105	AGIWUR40518
CB4856	CT	II	12909079	12910334	WBGene00009399	F35C5.11	F35C5.11
0.227948786646347	AGIWUR13730;AGIWUR21453;AGIWUR45080
CB4856	CT	II	12910229	12914603	WBGene00009400	F35C5.12	F35C5.12
-0.134303088388116	AGIWUR17427;AGIWUR23921;AGIWUR31371;AGIWUR33008
CB4856	CT	II	12915417	12919018	WBGene00010264	F58G1.2 F58G1.2 -0.163449158178569
AGIWUR4611
CB4856	CT	II	12919551	12923377	WBGene00010263	wago-4	F58G1.1 0.164473365626413
AGIWUR13287;AGIWUR32641
CB4856	CT	II	12924026	12925241	WBGene00010271	F58G1.9 F58G1.9
-0.00375894415050126	AGIWUR28772
CB4856	CT	II	12925304	12925602	WBGene00010270	F58G1.8 F58G1.8 -0.159165777096848
AGIWUR43158
CB4856	CT	II	12925981	12928341	WBGene00010265	F58G1.3 F58G1.3 -0.307124341823467
AGIWUR22297
CB4856	CT	II	12931873	12933515	WBGene00010266	dct-18	F58G1.4 -0.247254973232394
AGIWUR44081
CB4856	CT	II	12933854	12935362	WBGene00010267	lips-9	F58G1.5 -0.121741893181382
AGIWUR25581
CB4856	CT	II	12938869	12943867	WBGene00010268	arrd-10 F58G1.6 -0.702705187193027
AGIWUR3865
CB4856	CT	II	12943862	12948143	WBGene00010269	F58G1.7 F58G1.7 0.111357978547239
AGIWUR3737
CB4856	CT	II	12952490	12954832	WBGene00012605	Y38F1A.1	Y38F1A.1
0.251155112775706	AGIWUR25647
CB4856	CT	II	12954962	12958018	WBGene00012606	Y38F1A.2	Y38F1A.2
-0.12899586175099	AGIWUR36625
CB4856	CT	II	12965546	12978168	WBGene00004232	ptr-18	Y38F1A.3
0.0207482532425205	AGIWUR718
CB4856	CT	II	12980591	12993118	WBGene00000870	cyd-1	Y38F1A.5

-0.0240463348547451 AGIWUR2519;AGIWUR4218;AGIWUR4651;AGIWUR8981;AGIWUR24138;AGIWUR33027;AGIWUR42526
CB4856 CT II 12994358 12996571 WBGene00012608 hphd-1 Y38F1A.6
-0.550206270127447 AGIWUR17199;AGIWUR23064;AGIWUR41550
CB4856 CT II 12996921 12997804 WBGene00012609 Y38F1A.7 Y38F1A.7
0.0121335927153883 AGIWUR12368
CB4856 CT II 12997986 13000853 WBGene00012610 Y38F1A.8 Y38F1A.8
0.215218845108085 AGIWUR15880
CB4856 CT II 13006610 13008164 WBGene00003860 oig-2 Y38F1A.9
-0.334623752662297 AGIWUR43827
CB4856 CT II 13014331 13025838 WBGene00003144 max-2 Y38F1A.10
0.0207992326335003 AGIWUR11822;AGIWUR27909;AGIWUR29030;AGIWUR41651;AGIWUR42104
CB4856 CT II 13032778 13036505 WBGene00008929 F18A11.2 F18A11.2
0.0429473393579816 AGIWUR33087
CB4856 CT II 13047941 13050807 WBGene00009641 F42G4.2 F42G4.2 0.0013609248163533
AGIWUR32122
CB4856 CT II 13056814 13071530 WBGene00006999 zyx-1 F42G4.3 0.0347638592640265
AGIWUR13740;AGIWUR22966;AGIWUR35979;AGIWUR44121
CB4856 CT II 13073493 13076223 WBGene00009642 F42G4.5 F42G4.5 0.0276502767880444
AGIWUR61;AGIWUR9936;AGIWUR27632
CB4856 CT II 13081776 13082322 WBGene00195150 Y81G3A.6 Y81G3A.6
-0.307379906842635 AGIWUR7372
CB4856 CT II 13083989 13091483 WBGene00013591 gcn-2 Y81G3A.3
-0.208851863630048 AGIWUR1733;AGIWUR23403;AGIWUR36942
CB4856 CT II 13091587 13094241 WBGene00013592 Y81G3A.4 Y81G3A.4
0.100279316091498 AGIWUR27760
CB4856 CT II 13096009 13098275 WBGene00000661 col-86 Y81G3A.5
-0.0704593479437285 AGIWUR17449
CB4856 CT II 13098249 13116806 WBGene00009245 rict-1 F29C12.3
-0.115816805493773 AGIWUR30701;AGIWUR40860
CB4856 CT II 13117483 13122085 WBGene00009246 gfm-1 F29C12.4
0.0427507166052035 AGIWUR22803
CB4856 CT II 13123038 13124789 WBGene00009247 bath-45 F29C12.5
-0.296552374620418 AGIWUR30834
CB4856 CT II 13129902 13130189 WBGene00009248 F29C12.6 F29C12.6
-0.0359363562337212 AGIWUR16796
CB4856 CT II 13131009 13134212 WBGene00009310 F32A11.5 F32A11.5
-0.103377197624556 AGIWUR15642;AGIWUR20935;AGIWUR21948;AGIWUR22348;AGIWUR36357;AGIWUR36804
CB4856 CT II 13139465 13145225 WBGene00009311 mltn-1 F32A11.7
0.581460930367153 AGIWUR6903

CB4856 CT II 13151459 13153399 WBGene00009308 F32A11.3 F32A11.3
-0.0838969444692879 AGIWUR807
CB4856 CT II 13153461 13157093 WBGene00001998 hpr-17 F32A11.2
0.0113772119730271 AGIWUR7794
CB4856 CT II 13157333 13160462 WBGene00009307 F32A11.1 F32A11.1
0.0065609676057079 AGIWUR31770
CB4856 CT II 13160545 13165733 WBGene00003388 moe-3 F32A11.6
0.266965280896858 AGIWUR6208
CB4856 CT II 13175857 13181561 WBGene00012372 W09H1.1 W09H1.1 0.0392122405735087
AGIWUR5978
CB4856 CT II 13183450 13183845 WBGene00001947 his-73 W09H1.2 0.117088016624225
AGIWUR31054
CB4856 CT II 13184429 13185023 WBGene00012373 W09H1.3 W09H1.3 -0.0167544747662252
AGIWUR24666
CB4856 CT II 13185092 13185914 WBGene00012374 W09H1.4 W09H1.4 0.0314936114041071
AGIWUR21145
CB4856 CT II 13185987 13187351 WBGene00012375 mecr-1 W09H1.5 0.0904590597955917
AGIWUR34282
CB4856 CT II 13195549 13199603 WBGene00002264 lec-1 W09H1.6 0.0675209957134957
AGIWUR18206;AGIWUR41352;AGIWUR41572
CB4856 CT II 13200165 13215701 WBGene00000274 btf-1 F15D4.1 0.0425094081676018
AGIWUR12208;AGIWUR39267
CB4856 CT II 13216092 13216764 WBGene00008859 F15D4.2 F15D4.2 0.216626636168067
AGIWUR8652;AGIWUR29706;AGIWUR37514
CB4856 CT II 13235306 13237417 WBGene00001459 flp-16 F15D4.8 0.101717537878957
AGIWUR3652;AGIWUR16501;AGIWUR36244;AGIWUR39440
CB4856 CT II 13239249 13243121 WBGene00008861 F15D4.4 F15D4.4 0.443351506261437
AGIWUR28570;AGIWUR37736
CB4856 CT II 13245348 13247774 WBGene00008862 F15D4.5 F15D4.5 -0.401449772804833
AGIWUR1585
CB4856 CT II 13249189 13249895 WBGene00008863 F15D4.6 F15D4.6 -0.269403915606533
AGIWUR859;AGIWUR4033;AGIWUR13762;AGIWUR13873;AGIWUR18842;AGIWUR26669;AGIWUR33094;AGIWUR43311
CB4856 CT II 13260495 13261201 WBGene00012985 Y48C3A.1 Y48C3A.1
-0.0176012878788869 AGIWUR22693
CB4856 CT II 13272634 13304229 WBGene00012989 Y48C3A.5 Y48C3A.5
0.413487441270693 AGIWUR11657
CB4856 CT II 13309290 13311488 WBGene00012987 Y48C3A.3 Y48C3A.3
0.0249695857202603 AGIWUR12236;AGIWUR12626;AGIWUR21275
CB4856 CT II 13311459 13320911 WBGene00003119 mac-1 Y48C3A.7

-0.00781651527238569	AGIWUR9960;AGIWUR9979;AGIWUR21632;AGIWUR43284
CB4856	CT	II	13321954	13330825	WBGene00012990	sam-10	Y48C3A.8
0.028581147746249	AGIWUR19200
CB4856	CT	II	13338281	13339961	WBGene00012992	mrpl-20	Y48C3A.10
-0.29950643093977	AGIWUR16972
CB4856	CT	II	13340644	13347884	WBGene00012993	dmsr-3	Y48C3A.11
-0.0159253538915349	AGIWUR8773
CB4856	CT	II	13369607	13375290	WBGene00012998	Y48C3A.20	Y48C3A.20
-0.132242109229191	AGIWUR6602;AGIWUR34545
CB4856	CT	II	13376412	13388879	WBGene00012994	Y48C3A.12	Y48C3A.12
-0.307353667129369	AGIWUR4627;AGIWUR21344
CB4856	CT	II	13389761	13401992	WBGene00012995	Y48C3A.14	Y48C3A.14
-0.0883811509943957	AGIWUR13908
CB4856	CT	II	13402980	13404629	WBGene00012996	pinn-4	Y48C3A.16
-0.0327853647367462	AGIWUR26311
CB4856	CT	II	13406160	13411483	WBGene00001162	efl-2	Y48C3A.17
-0.206111783943474	AGIWUR25742;AGIWUR26124;AGIWUR26605
CB4856	CT	II	13412635	13414906	WBGene00012997	Y48C3A.18	Y48C3A.18
0.0881076856535907	AGIWUR8883;AGIWUR13097;AGIWUR13111;AGIWUR16520;AGIWUR24033;AGIWUR25801
CB4856	CT	II	13415024	13424446	WBGene00013014	Y48E1C.1	Y48E1C.1
-0.00209790741647176	AGIWUR2649;AGIWUR5616;AGIWUR12338;AGIWUR16989;AGIWUR19071;AGIWUR32260;AGIWUR41231
CB4856	CT	II	13424506	13428970	WBGene00013015	Y48E1C.2	Y48E1C.2
-0.0703609514332904	AGIWUR13917
CB4856	CT	II	13433880	13436825	WBGene00013017	Y48E1C.4	Y48E1C.4
0.353530985249371	AGIWUR10395
CB4856	CT	II	13437579	13447593	WBGene00008444	tbc-14	E01G4.1	0.00875785463767986
AGIWUR5556;AGIWUR44407
CB4856	CT	II	13448835	13460642	WBGene00004116	pqn-27	E01G4.4	-0.169039543480423
AGIWUR25;AGIWUR10877;AGIWUR44806
CB4856	CT	II	13460991	13469602	WBGene00008446	E01G4.3	E01G4.3	-0.103472576246553
AGIWUR10416;AGIWUR28784;AGIWUR33582;AGIWUR41256;AGIWUR44661
CB4856	CT	II	13472401	13473995	WBGene00008447	E01G4.5	E01G4.5	-0.195492029177736
AGIWUR4017;AGIWUR39799
CB4856	CT	II	13477471	13478929	WBGene00045410	E01G4.7	E01G4.7	-0.201944789540908
AGIWUR4005;AGIWUR8184;AGIWUR21131;AGIWUR27985;AGIWUR29964;AGIWUR39135
CB4856	CT	II	13482405	13485780	WBGene00008448	E01G4.6	E01G4.6	-0.180621545478852
AGIWUR7121
CB4856	CT	II	13485792	13491786	WBGene00000113	alh-7	F45H10.1
-0.147247862012762	AGIWUR37785

CB4856	CT	II	13491803	13493050	WBGene00009739	F45H10.2	F45H10.2
-0.0183666326827971	AGIWUR16910
CB4856	CT	II	13493181	13494600	WBGene00009740	F45H10.3	F45H10.3
-0.103219609030748	AGIWUR19029;AGIWUR22747
CB4856	CT	II	13495050	13495900	WBGene00009741	drr-1	F45H10.4
-0.106340875316633	AGIWUR8290
CB4856	CT	II	13501877	13503822	WBGene00009742	F45H10.5	F45H10.5
0.138141222437207
AGIWUR5292;AGIWUR9571;AGIWUR12387;AGIWUR12768;AGIWUR21572;AGIWUR25012;AGIWUR29346;AGIWUR34331;AGIWUR35280;AGIWUR39653;AGIWUR40900
CB4856	CT	II	13504082	13506905	WBGene00010069	F54F11.1	F54F11.1
0.566734268727075	AGIWUR34937
CB4856	CT	II	13507806	13516479	WBGene00010070	nep-17	F54F11.2
-0.209630336759538	AGIWUR31258
CB4856	CT	II	13517912	13522925	WBGene00010071	sre-45	F54F11.3
-0.245543754447986	AGIWUR27402
CB4856	CT	II	13522919	13531218	WBGene00013011	snx-14	Y48E1B.14
-0.125286781120552	AGIWUR11452;AGIWUR18223;AGIWUR20153;AGIWUR36079;AGIWUR41053
CB4856	CT	II	13533486	13541084	WBGene00013001	Y48E1B.2	Y48E1B.2
-0.260051985915316	AGIWUR24617;AGIWUR29266
CB4856	CT	II	13542007	13547645	WBGene00013000	ddl-2	Y48E1B.1
-0.0330963944987817	AGIWUR8887;AGIWUR16973
CB4856	CT	II	13547739	13567999	WBGene00013002	Y48E1B.3	Y48E1B.3
-0.149441769397809
AGIWUR15123;AGIWUR19802;AGIWUR31063;AGIWUR31692;AGIWUR33404;AGIWUR34595;AGIWUR44162;AGIWUR44251
CB4856	CT	II	13568077	13575068	WBGene00013004	mrpl-37	Y48E1B.5
-0.270266297112356	AGIWUR2143
CB4856	CT	II	13575925	13584732	WBGene00013006	lin-38	Y48E1B.7
-0.0485488602870303	AGIWUR2850;AGIWUR25413
CB4856	CT	II	13585705	13589033	WBGene00013007	Y48E1B.8	Y48E1B.8
-0.566704663580037	AGIWUR15064
CB4856	CT	II	13589036	13590552	WBGene00013013	clec-145	Y48E1B.16
0.824041865613665	AGIWUR7883
CB4856	CT	II	13590640	13593797	WBGene00013008	clec-146	Y48E1B.9
0.346299802580504	AGIWUR7912;AGIWUR21835;AGIWUR41929
CB4856	CT	II	13595006	13596551	WBGene00013009	fbxc-23	Y48E1B.11
0.105194805183565
AGIWUR11623;AGIWUR14076;AGIWUR30753;AGIWUR33903;AGIWUR35723;AGIWUR38420;AGIWUR39312;AGIWUR42220
CB4856	CT	II	13596955	13598068	WBGene00000804	csc-1	Y48E1B.12

-0.0430469273772909 AGIWUR7181;AGIWUR27268
CB4856 CT II 13601204 13605057 WBGene00000819 csp-1 Y48E1B.13
0.257814636180474 AGIWUR13033
CB4856 CT II 13608154 13609824 WBGene00001768 gst-20 Y48E1B.10
-0.147346532649948 AGIWUR21650
CB4856 CT II 13610443 13611349 WBGene00001772 gst-24 F37B1.1 -0.46440590412942
AGIWUR15934
CB4856 CT II 13611771 13613196 WBGene00001763 gst-15 F37B1.4 0.683413031348527
AGIWUR43213
CB4856 CT II 13614788 13616100 WBGene00001762 gst-14 F37B1.3 0.199867347016651
AGIWUR27122
CB4856 CT II 13616685 13618061 WBGene00001760 gst-12 F37B1.2 0.421659717249078
AGIWUR11751
CB4856 CT II 13622967 13624502 WBGene00001764 gst-16 F37B1.5 -0.52581245627468
AGIWUR31264
CB4856 CT II 13625544 13627141 WBGene00001767 gst-19 F37B1.8 0.209455143653454
AGIWUR3777;AGIWUR36800
CB4856 CT II 13628017 13628774 WBGene00001765 gst-17 F37B1.6 0.0689685981734627
AGIWUR36475
CB4856 CT II 13629799 13630887 WBGene00001766 gst-18 F37B1.7 0.0214660467911134
AGIWUR12330;AGIWUR24948;AGIWUR32902;AGIWUR38232;AGIWUR44742
CB4856 CT II 13632655 13636986 WBGene00006404 tag-10 C31C9.1 0.0962853553723466
AGIWUR37940
CB4856 CT II 13636929 13638480 WBGene00007836 C31C9.2 C31C9.2 0.316511069114546
AGIWUR3095;AGIWUR8721;AGIWUR10783;AGIWUR21035;AGIWUR28229;AGIWUR36644
CB4856 CT II 13641495 13643821 WBGene00007837 fbxa-167 C31C9.3
-0.112437368797412 AGIWUR44122
CB4856 CT II 13645781 13648850 WBGene00007838 fbxa-168 C31C9.4
0.0354875518751401 AGIWUR1946
CB4856 CT II 13648530 13655347 WBGene00045289 fbxa-169 C31C9.8
-0.133685014715725 AGIWUR7582
CB4856 CT II 13669104 13669713 WBGene00007841 C31C9.7 C31C9.7 0.0964845557610374
AGIWUR36149
CB4856 CT II 13670633 13694787 WBGene00012999 rpoa-1 Y48E1A.1
0.218847701207896 AGIWUR5492;AGIWUR11083
CB4856 CT II 13703877 13712911 WBGene00002235 kqt-3 Y54G9A.3
-0.248348032450044 AGIWUR17672;AGIWUR41149
CB4856 CT II 13712921 13715893 WBGene00013207 zipt-2.3 Y54G9A.4
-0.398078491158051 AGIWUR31247

CB4856	CT	II	13718185	13721318	WBGene00044084	sre-46	Y54G9A.10	-0.0619031910901473	AGIWUR9584;AGIWUR39918
CB4856	CT	II	13722964	13725037	WBGene00013208	Y54G9A.5	Y54G9A.5	0.22910397130418	AGIWUR38181
CB4856	CT	II	13724978	13725805	WBGene00013210	Y54G9A.7	Y54G9A.7	0.0307273914211361	AGIWUR8650
CB4856	CT	II	13726062	13729610	WBGene00013209	bub-3	Y54G9A.6	-0.0732937296939298	AGIWUR27299
CB4856	CT	II	13729716	13731543	WBGene00013211	Y54G9A.9	Y54G9A.9	-0.596769664817304	AGIWUR6367
CB4856	CT	II	13736349	13736933	WBGene00011056	R06B9.5	R06B9.5	-0.0352453610071661	AGIWUR29910
CB4856	CT	II	13741742	13749983	WBGene00003246	mig-14	R06B9.6	0.0238137830801151	AGIWUR3974
CB4856	CT	II	13752316	13756267	WBGene00011055	arrd-14	R06B9.4	0.497595967902921	AGIWUR11577
CB4856	CT	II	13758408	13761233	WBGene00011054	arrd-13	R06B9.3	0.0183952681791943	AGIWUR21367
CB4856	CT	II	13792229	13817398	WBGene00013934	ZK131.11	ZK131.11	0.573294310397299	AGIWUR2862;AGIWUR10799
CB4856	CT	II	13821778	13822333	WBGene00001885	his-11	ZK131.5	-0.519454398991123	AGIWUR9952;AGIWUR15972;AGIWUR16772;AGIWUR23210;AGIWUR36528
CB4856	CT	II	13828287	13828697	WBGene00001916	his-42	F08G2.3	0.170150260230956	AGIWUR34096
CB4856	CT	II	13830592	13831213	WBGene00008576	F08G2.4	F08G2.4	0.33998647109615	AGIWUR31364
CB4856	CT	II	13832870	13833653	WBGene00008577	F08G2.5	F08G2.5	0.00654747334990891	AGIWUR43189
CB4856	CT	II	13835103	13835709	WBGene00002120	ins-37	F08G2.6	0.539403820350175	AGIWUR44380
CB4856	CT	II	13836241	13841139	WBGene00008578	F08G2.7	F08G2.7	0.104274701986469	AGIWUR28240
CB4856	CT	II	13852311	13855290	WBGene00008579	F08G2.8	F08G2.8	0.161418318821287	AGIWUR5634
CB4856	CT	II	13855788	13872315	WBGene00013093	Y51H1A.2	Y51H1A.2	-0.0217938546171697	AGIWUR10118;AGIWUR12948;AGIWUR15961;AGIWUR23741;AGIWUR29105;AGIWUR32778;AGIWUR41195
CB4856	CT	II	13872869	13875423	WBGene00013094	Y51H1A.3	Y51H1A.3	-0.0684697654671993	AGIWUR11740;AGIWUR14779;AGIWUR19083;AGIWUR23307;AGIWUR31503;AGIWUR38345;AGIWUR41980
CB4856	CT	II	13876034	13882585	WBGene00013095	ing-3	Y51H1A.4		

-0.130528373931181 AGIWUR539
CB4856 CT II 13882705 13888031 WBGene00001838 hda-10 Y51H1A.5
0.211384513424143 AGIWUR33508
CB4856 CT II 13888583 13898394 WBGene00013096 mcd-1 Y51H1A.6
0.172250496514147 AGIWUR8763;AGIWUR38762
CB4856 CT II 13898366 13902114 WBGene00012197 W02B8.1 W02B8.1 0.0785567345679716
AGIWUR36040
CB4856 CT II 13908921 13915254 WBGene00012198 W02B8.2 W02B8.2 0.362381395820417
AGIWUR13508
CB4856 CT II 13916340 13919838 WBGene00012199 mltn-3 W02B8.3 0.100460287070804
AGIWUR4662;AGIWUR18634;AGIWUR35203
CB4856 CT II 13920555 13924724 WBGene00012200 mltn-4 W02B8.4 0.109390403376743
AGIWUR36510;AGIWUR40714
CB4856 CT II 13925268 13930516 WBGene00044074 mltn-5 W02B8.6 -0.234434912464453
AGIWUR39189
CB4856 CT II 13930648 13931997 WBGene00001988 hot-3 W02B8.5 0.126031776026044
AGIWUR31128;AGIWUR40137
CB4856 CT II 13937995 13939631 WBGene00012328 W07G1.1 W07G1.1 -0.0948303266093653
AGIWUR33574
CB4856 CT II 13949590 13951869 WBGene00012329 sre-44 W07G1.2 -0.274248273381597
AGIWUR23234;AGIWUR24414;AGIWUR34747
CB4856 CT II 13951906 13957543 WBGene00012330 zip-3 W07G1.3 -0.136322386907479
AGIWUR18520;AGIWUR40639
CB4856 CT II 13959239 13962417 WBGene00012332 rpi-1 W07G1.5 -0.0698744310644441
AGIWUR29081;AGIWUR34293;AGIWUR44572
CB4856 CT II 13964891 13966333 WBGene00012334 W07G1.7 W07G1.7 0.0905420734202398
AGIWUR15265
CB4856 CT II 13966534 13969079 WBGene00012333 sre-43 W07G1.6 0.127064033780981
AGIWUR27997
CB4856 CT II 13970584 13972970 WBGene00012722 Y39G8B.1 Y39G8B.1
-0.1609487820089 AGIWUR15112;AGIWUR16525
CB4856 CT II 13973690 13976221 WBGene00012723 Y39G8B.2 Y39G8B.2
-0.248862987515832 AGIWUR6405
CB4856 CT II 13977164 13979883 WBGene00012724 sre-48 Y39G8B.3
0.308626222874724 AGIWUR23507
CB4856 CT II 13981330 13981785 WBGene00012728 Y39G8B.9 Y39G8B.9
-0.158127528322101 AGIWUR1280;AGIWUR18970;AGIWUR25187;AGIWUR37328;AGIWUR41482;AGIWUR44093
CB4856 CT II 13982048 13983115 WBGene00012729 Y39G8B.10 Y39G8B.10
0.329731754011471 AGIWUR44923

CB4856	CT	II	13983397	13984651	WBGene00012725	sre-47	Y39G8B.4	-0.0352529599448168	AGIWUR14222
CB4856	CT	II	13987616	13991838	WBGene00012726	Y39G8B.5	Y39G8B.5	0.358119994125827	AGIWUR5997;AGIWUR29173
CB4856	CT	II	13993009	13993527	WBGene00014885	Y39G8B.8	Y39G8B.8	-0.16570332545157	AGIWUR4346;AGIWUR37185;AGIWUR43820
CB4856	CT	II	13994411	13995045	WBGene00012727	Y39G8B.7	Y39G8B.7	-0.105005295836486	AGIWUR25154
CB4856	CT	II	13995401	13995876	WBGene00008501	fbxa-177	F01D5.11	-0.153086874945207	AGIWUR13019
CB4856	CT	II	13996958	13997606	WBGene00008492	F01D5.1	F01D5.1	-0.81008251679909	AGIWUR10090
CB4856	CT	II	13998497	13999064	WBGene00008493	F01D5.2	F01D5.2	-0.699773988626923	AGIWUR45174
CB4856	CT	II	13999059	14000144	WBGene00008494	F01D5.3	F01D5.3	-0.501802926517758	AGIWUR16727
CB4856	CT	II	14001686	14002306	WBGene00008495	F01D5.5	F01D5.5	-0.50948883280053	AGIWUR8659
CB4856	CT	II	14006511	14008514	WBGene00008496	F01D5.6	F01D5.6	0.352702676337541	AGIWUR18402
CB4856	CT	II	14008666	14010583	WBGene00008497	F01D5.7	F01D5.7	-0.225865170873444	AGIWUR41689
CB4856	CT	II	14010706	14013826	WBGene00008498	F01D5.8	F01D5.8	-0.238733631523015	AGIWUR10871
CB4856	CT	II	14013825	14016965	WBGene00008499	cyp-37A1	F01D5.9	0.504218958753301	AGIWUR11139;AGIWUR13421;AGIWUR31528;AGIWUR34500
CB4856	CT	II	14017839	14021330	WBGene00008500	F01D5.10	F01D5.10	0.0563877396939469	AGIWUR9014
CB4856	CT	II	14031402	14032644	WBGene00014851	W01G7.2	W01G7.2	0.0989039108031653	AGIWUR33995
CB4856	CT	II	14033233	14037721	WBGene00000901	daf-5	W01G7.1	-0.0350311718035998	AGIWUR2777;AGIWUR3143;AGIWUR4552;AGIWUR23271;AGIWUR42691;AGIWUR43981
CB4856	CT	II	14060061	14060624	WBGene00012187	rpb-11	W01G7.3	-0.182666646386672	AGIWUR1799
CB4856	CT	II	14060739	14062960	WBGene00012188	W01G7.4	W01G7.4	-0.0172194608659728	AGIWUR6293;AGIWUR13407;AGIWUR25133;AGIWUR39814;AGIWUR41628;AGIWUR42037
CB4856	CT	II	14063450	14066170	WBGene00002275	lem-2	W01G7.5	-0.1096912411251	AGIWUR36856
CB4856	CT	II	14068907	14089119	WBGene00012730	xrn-1	Y39G8C.1		

-0.162034589941259	AGIWUR3029;AGIWUR27428
CB4856	CT	II	14090075	14094690	WBGene00000662	col-87	Y39G8C.4
0.0691337865288215	AGIWUR21941
CB4856	CT	II	14095447	14097390	WBGene00012731	Y39G8C.2	Y39G8C.2
-0.320104292417062	AGIWUR18523
CB4856	CT	II	14101263	14102961	WBGene00014569	bkip-1	Y39G8C.3
0.123756405260076
AGIWUR2676;AGIWUR3891;AGIWUR12056;AGIWUR14174;AGIWUR15841;AGIWUR15877;AGIWUR16335;AGIWUR23672;AGIWUR35130;A
GIWUR37737;AGIWUR38371;AGIWUR40259;AGIWUR41719;AGIWUR43332
CB4856	CT	II	14104773	14108072	WBGene00001805	gur-4	K09E4.5	-0.0338546467803758
AGIWUR819
CB4856	CT	II	14108016	14109635	WBGene00010723	cpg-7	K09E4.6	0.780389593204319
AGIWUR861
CB4856	CT	II	14114213	14122165	WBGene00010722	K09E4.4	K09E4.4	0.224821094033725
AGIWUR36499
CB4856	CT	II	14123945	14126941	WBGene00010721	K09E4.3	K09E4.3	0.0897184553057144
AGIWUR12853
CB4856	CT	II	14127687	14132833	WBGene00010720	K09E4.2	K09E4.2	-0.115616917243354
AGIWUR6171
CB4856	CT	II	14133397	14142289	WBGene00012230	cacn-1	W03H9.4	-0.18322281073425
AGIWUR27840
CB4856	CT	II	14142949	14143874	WBGene00012228	W03H9.2	W03H9.2	-0.0244234528438541
AGIWUR7297;AGIWUR20495
CB4856	CT	II	14151040	14154373	WBGene00012978	Y48B6A.1	Y48B6A.1
0.0164220705239062	AGIWUR7453;AGIWUR18959
CB4856	CT	II	14155280	14155660	WBGene00004456	rpl-43	Y48B6A.2
-0.168588254104838
AGIWUR3534;AGIWUR4568;AGIWUR11626;AGIWUR13510;AGIWUR21750;AGIWUR21886;AGIWUR26463;AGIWUR33415;AGIWUR36339;A
GIWUR38880
CB4856	CT	II	14155768	14166018	WBGene00006964	xrn-2	Y48B6A.3
0.0475500014694461	AGIWUR40875
CB4856	CT	II	14172833	14175174	WBGene00012979	Y48B6A.5	Y48B6A.5
-0.32579568580479	AGIWUR8342
CB4856	CT	II	14178814	14189917	WBGene00012980	efhd-1	Y48B6A.6
0.0162538013250653	AGIWUR14476;AGIWUR16862;AGIWUR19411;AGIWUR21777
CB4856	CT	II	14205633	14210214	WBGene00000037	ace-3	Y48B6A.8
0.386218277894496	AGIWUR33827
CB4856	CT	II	14211439	14213123	WBGene00001992	hot-7	Y48B6A.9
-0.26096859985952	AGIWUR20114;AGIWUR22691

CB4856	CT	II	14214409	14217139	WBGene00012981	Y48B6A.10	Y48B6A.10
0.00408643048661439	AGIWUR21643
CB4856	CT	II	14217107	14218064	WBGene00050948	Y48B6A.16	Y48B6A.16
-0.0481226787250542	AGIWUR4514;AGIWUR21323;AGIWUR24184;AGIWUR35402;AGIWUR42266
CB4856	CT	II	14220938	14226287	WBGene00012984	Y48B6A.13	Y48B6A.13
-0.13878951867005	AGIWUR31036
CB4856	CT	II	14226377	14234120	WBGene00012983	men-1	Y48B6A.12
0.162049424472329	AGIWUR33695
CB4856	CT	II	14234972	14243603	WBGene00012982	jmjd-2	Y48B6A.11
-0.0424777210525846
AGIWUR484;AGIWUR4107;AGIWUR9451;AGIWUR12375;AGIWUR17242;AGIWUR26629;AGIWUR28009;AGIWUR35789;AGIWUR40210
CB4856	CT	II	14250683	14251448	WBGene00001971	hmg-1.1	Y48B6A.14
0.308356011576062	AGIWUR2307;AGIWUR6488;AGIWUR12701;AGIWUR18413;AGIWUR19350;AGIWUR40655
CB4856	CT	II	14252357	14255705	WBGene00013213	Y54G11A.2	Y54G11A.2
0.0609549878714688	AGIWUR11933
CB4856	CT	II	14256610	14256987	WBGene00235256	Y54G11A.19	Y54G11A.19
-0.0931951402934565	AGIWUR5247
CB4856	CT	II	14276621	14283973	WBGene00013214	Y54G11A.3	Y54G11A.3
-0.0836020257296752	AGIWUR26523
CB4856	CT	II	14285780	14287377	WBGene00005264	srh-41	Y54G11A.12
0.189306821047776	AGIWUR23858
CB4856	CT	II	14287632	14289068	WBGene00005265	srh-42	Y54G11A.15
0.111557117600976	AGIWUR36608
CB4856	CT	II	14289696	14290875	WBGene00005266	srh-43	Y54G11A.16
0.132674279416165	AGIWUR8458
CB4856	CT	II	14292824	14296055	WBGene00013215	Y54G11A.4	Y54G11A.4
-0.0796021790302373	AGIWUR27775
CB4856	CT	II	14297305	14299633	WBGene00013216	Y54G11A.7	Y54G11A.7
-0.0278401665003827	AGIWUR2686
CB4856	CT	II	14299553	14302083	WBGene00000831	ctl-2	Y54G11A.5
0.253551611563111	AGIWUR8751
CB4856	CT	II	14318158	14320917	WBGene00013221	Y54G11A.14	Y54G11A.14
0.000482129746892213	AGIWUR43300
CB4856	CT	II	14340068	14344454	WBGene00013217	ddl-3	Y54G11A.8
-0.292801599784121	AGIWUR17300;AGIWUR32646
CB4856	CT	II	14346877	14348792	WBGene00002996	lin-7	Y54G11A.10
-0.217909050725456	AGIWUR14816
CB4856	CT	II	14349783	14350795	WBGene00013219	Y54G11A.11	Y54G11A.11
-0.0366965858028898	AGIWUR20166;AGIWUR32611

CB4856	CT	II	14350719	14358113	WBGene00011050	agl-1	R06A4.8	-0.0824040965587542
AGIWUR23864
CB4856	CT	II	14358366	14361701	WBGene00011051	pfs-2	R06A4.9	-0.0876896852803757
AGIWUR11966
CB4856	CT	II	14362019	14365177	WBGene00011047	R06A4.2	R06A4.2	0.0440471137022307
AGIWUR31139
CB4856	CT	II	14365705	14373899	WBGene00002076	imb-2	R06A4.4	-0.0219613925449202
AGIWUR3727;AGIWUR8967;AGIWUR15087;AGIWUR16722;AGIWUR22315
CB4856	CT	II	14373831	14377890	WBGene00000059	acr-20	R06A4.10
0.0619770373696618	AGIWUR3520
CB4856	CT	II	14381772	14383376	WBGene00011049	sdz-26	R06A4.6	-0.0732333658391582
AGIWUR11439
CB4856	CT	II	14383973	14388454	WBGene00003220	mes-2	R06A4.7	0.11355144797038
AGIWUR7454
CB4856	CT	II	14389236	14390116	WBGene00009179	kbp-3	F26H11.1
0.04499058391918	AGIWUR37325
CB4856	CT	II	14390718	14407905	WBGene00009180	nurf-1	F26H11.2
0.0514876042855903
AGIWUR1055;AGIWUR3235;AGIWUR7486;AGIWUR10576;AGIWUR22291;AGIWUR22413;AGIWUR23878;AGIWUR26556;AGIWUR26933;AGIWUR27114;AGIWUR29545;AGIWUR33257;AGIWUR38259;AGIWUR40676
CB4856	CT	II	14411095	14412218	WBGene00194892	dsb-2	F26H11.6
-0.21211994122001	AGIWUR17446
CB4856	CT	II	14412201	14413516	WBGene00001371	exl-1	F26H11.5
0.123527936807655	AGIWUR41988
CB4856	CT	II	14417889	14424891	WBGene00004048	plx-2	K04B12.1
0.457503236159883	AGIWUR4508;AGIWUR33866;AGIWUR38759;AGIWUR44043
CB4856	CT	II	14434419	14438977	WBGene00010550	K04B12.2	K04B12.2
0.451912321807303	AGIWUR8514
CB4856	CT	II	14441409	14447219	WBGene00010551	smg-8	K04B12.3
-0.145684841480451	AGIWUR21796
CB4856	CT	II	14455632	14464911	WBGene00013222	dmsr-6	Y54G11B.1
0.316369764267714	AGIWUR16816
CB4856	CT	II	14470371	14479627	WBGene00007545	C13B4.1	C13B4.1	0.0486465940115117
AGIWUR7062
CB4856	CT	II	14485624	14488688	WBGene00006856	usp-14	C13B4.2	0.140986950878151
AGIWUR9141;AGIWUR11923;AGIWUR27616;AGIWUR28554;AGIWUR34325;AGIWUR36729;AGIWUR43933
CB4856	CT	II	14489802	14492901	WBGene00010758	vnut-1	K10H10.1
-0.169884339853644	AGIWUR5410
CB4856	CT	II	14492826	14494514	WBGene00010759	cysl-2	K10H10.2

-0.0799149350825274	AGIWUR25107
CB4856	CT	II	14498026	14499755	WBGene00000972	dhs-8	K10H10.3
0.58305996539525	AGIWUR22194
CB4856	CT	II	14500422	14501663	WBGene00010760	K10H10.4	K10H10.4
-0.834394100370833	AGIWUR44601
CB4856	CT	II	14501747	14502543	WBGene00010761	K10H10.5	K10H10.5
-0.0147152909944541	AGIWUR33435
CB4856	CT	II	14505015	14506409	WBGene00045403	K10H10.12	K10H10.12
0.109121841082077
AGIWUR871;AGIWUR3702;AGIWUR4293;AGIWUR13405;AGIWUR18072;AGIWUR18318;AGIWUR20971;AGIWUR23433;AGIWUR28318;AGIWUR29134
CB4856	CT	II	14507092	14507663	WBGene00010763	K10H10.7	K10H10.7
-0.421601365129124	AGIWUR17523
CB4856	CT	II	14508068	14510162	WBGene00010765	K10H10.10	K10H10.10
0.56529854335918	AGIWUR37841
CB4856	CT	II	14513191	14515117	WBGene00010764	K10H10.9	K10H10.9
-0.00699217484717352	AGIWUR826
CB4856	CT	II	14524173	14525112	WBGene00010195	pot-2	F57C2.3	-0.450792052692753
AGIWUR14166
CB4856	CT	II	14525312	14526031	WBGene00010196	F57C2.4	F57C2.4	0.227712316850154
AGIWUR38942
CB4856	CT	II	14526213	14528296	WBGene00010197	F57C2.5	F57C2.5	0.0277331813096318
AGIWUR20898
CB4856	CT	II	14528754	14534173	WBGene00010198	spat-1	F57C2.6	-0.0380263526324907
AGIWUR21485
CB4856	CT	II	14534300	14535365	WBGene00007312	C04H5.1	C04H5.1	-0.544429908493974
AGIWUR6314
CB4856	CT	II	14535923	14537579	WBGene00007313	clec-147	C04H5.2
0.346661029251528	AGIWUR37021
CB4856	CT	II	14537946	14539162	WBGene00007315	C04H5.7	C04H5.7	-0.031627670486321
AGIWUR11910;AGIWUR32330;AGIWUR41692;AGIWUR42941
CB4856	CT	II	14540895	14548791	WBGene00007314	gcy-29	C04H5.3	0.244890684568294
AGIWUR12134
CB4856	CT	II	14550913	14552476	WBGene00007316	nlp-41	C04H5.8	0.126723760955628
AGIWUR1866;AGIWUR5601;AGIWUR7581;AGIWUR9920;AGIWUR14170;AGIWUR37811
CB4856	CT	II	14552501	14561599	WBGene00003392	mog-4	C04H5.6	-0.283918917109002
AGIWUR18263
CB4856	CT	II	14573615	14576558	WBGene00007242	madf-5	C01G12.1
0.465163740080701	AGIWUR44209

CB4856	CT	II	14576957	14578075	WBGene00044333	C01G12.13	C01G12.13	-0.173884994580499	AGIWUR14353;AGIWUR44551
CB4856	CT	II	14579016	14580124	WBGene00007249	C01G12.9	C01G12.9	-0.17089266734559	AGIWUR34770
CB4856	CT	II	14580254	14581043	WBGene00007250	nspb-8	C01G12.10	-0.107295245609707	AGIWUR8498;AGIWUR34778;AGIWUR36101
CB4856	CT	II	14581377	14582361	WBGene00007244	C01G12.3	C01G12.3	-0.103681553057385	AGIWUR28563
CB4856	CT	II	14583706	14585733	WBGene00006939	wee-1.2	C01G12.4	0.0530962503877173	AGIWUR12799;AGIWUR41845
CB4856	CT	II	14586527	14587507	WBGene00007245	C01G12.5	C01G12.5	0.16212018103976	AGIWUR21158
CB4856	CT	II	14587957	14588241	WBGene00007246	nspb-10	C01G12.6	-0.164969989041856	AGIWUR22159
CB4856	CT	II	14588621	14591893	WBGene00007247	C01G12.7	C01G12.7	0.0673752228344693	AGIWUR6995
CB4856	CT	II	14593581	14597657	WBGene00007248	catp-4	C01G12.8	0.166966837873129	AGIWUR22850;AGIWUR29822
CB4856	CT	II	14600902	14610554	WBGene00006603	tps-2	F19H8.1	0.036316637980228	AGIWUR35888
CB4856	CT	II	14616068	14622612	WBGene00008964	mltn-9	F19H8.4	0.288662385111964	AGIWUR22280
CB4856	CT	II	14627630	14630121	WBGene00007999	tag-297	C38C6.6	0.338286190745622	AGIWUR1156
CB4856	CT	II	14630553	14632636	WBGene00007998	C38C6.5	C38C6.5	-0.0776061669611776	AGIWUR2409;AGIWUR16695
CB4856	CT	II	14633123	14634657	WBGene00007996	C38C6.3	C38C6.3	0.0903356684569125	AGIWUR29542
CB4856	CT	II	14634656	14636837	WBGene00007997	sre-13	C38C6.4	-0.0181110007913343	AGIWUR30223
CB4856	CT	II	14640096	14644083	WBGene00000225	atgp-2	C38C6.2	0.0178561027596027	AGIWUR3765;AGIWUR21389;AGIWUR21804;AGIWUR28905
CB4856	CT	II	14647325	14666943	WBGene00006787	unc-52	ZC101.2	0.265915725270055	AGIWUR18169;AGIWUR31295;AGIWUR36135
CB4856	CT	II	14675263	14684296	WBGene00013851	ZC101.1	ZC101.1	0.358538249149058	AGIWUR35897
CB4856	CT	II	14694192	14695299	WBGene00010491	K02B7.3	K02B7.3	0.078439546958168	AGIWUR13831;AGIWUR17035;AGIWUR22360;AGIWUR27365;AGIWUR31050;AGIWUR37046
CB4856	CT	II	14698701	14699362	WBGene00007478	C09F9.1	C09F9.1	-0.334650962939714	

AGIWUR371
CB4856 CT II 14705512 14712800 WBGene00007479 C09F9.2 C09F9.2 0.396586835621514
AGIWUR3438
CB4856 CT II 14713340 14718225 WBGene00045322 C09F9.7 C09F9.7
-0.00775940441966938 AGIWUR30576
CB4856 CT II 14734410 14739243 WBGene00013187 npr-34 Y54E2A.1
-0.0639529564875954 AGIWUR27662
CB4856 CT II 14746890 14752489 WBGene00013188 smg-9 Y54E2A.2
-0.191510604611903 AGIWUR8749;AGIWUR25196;AGIWUR38912
CB4856 CT II 14752891 14753907 WBGene00006381 tac-1 Y54E2A.3
-0.0683258731481544 AGIWUR18103
CB4856 CT II 14754622 14778149 WBGene00013189 Y54E2A.4 Y54E2A.4
-0.0578446989956962 AGIWUR9156;AGIWUR23342
CB4856 CT II 14778345 14782723 WBGene00013193 Y54E2A.8 Y54E2A.8
0.101094530485835 AGIWUR8170
CB4856 CT II 14785184 14785995 WBGene00013194 Y54E2A.9 Y54E2A.9
-0.191070178901018 AGIWUR1495
CB4856 CT II 14786198 14786512 WBGene00044171 Y54E2A.13 Y54E2A.13
-0.0826694293323289 AGIWUR8457;AGIWUR19675;AGIWUR30747;AGIWUR37073;AGIWUR43089
CB4856 CT II 14791101 14792554 WBGene00013195 Y54E2A.10 Y54E2A.10
0.203190964345925 AGIWUR30769
CB4856 CT II 14795404 14798224 WBGene00001225 eif-3.B Y54E2A.11
-0.0637176887309607 AGIWUR3619;AGIWUR7825;AGIWUR11053;AGIWUR11745;AGIWUR15942
CB4856 CT II 14798472 14800971 WBGene00013196 tbc-20 Y54E2A.12
0.0398948701543613 AGIWUR13816;AGIWUR17503;AGIWUR33262
CB4856 CT II 14800918 14804309 WBGene00012180 W01D2.3 W01D2.3 -0.336785872805887
AGIWUR5729
CB4856 CT II 14805986 14808087 WBGene00012181 srt-11 W01D2.4 0.283172895037477
AGIWUR13564
CB4856 CT II 14808922 14809204 WBGene00012183 W01D2.6 W01D2.6 0.076892836822692
AGIWUR19703
CB4856 CT II 14812182 14814889 WBGene00012182 osta-3 W01D2.5 -0.114588251719731
AGIWUR28930
CB4856 CT II 14816136 14840005 WBGene00003651 nhr-61 W01D2.2 0.0683158364835617
AGIWUR20157;AGIWUR25353;AGIWUR29518;AGIWUR30187
CB4856 CT II 14843177 14850905 WBGene00000399 cdh-7 R05H10.6
0.00959243669713139 AGIWUR6629
CB4856 CT II 14852430 14853594 WBGene00011042 R05H10.1 R05H10.1
0.391737439105842 AGIWUR42783

CB4856	CT	II	14855529	14857010	WBGene00011046	R05H10.7	R05H10.7
0.108992257473199	AGIWUR24851
CB4856	CT	II	14857259	14866636	WBGene00011043	rbm-28	R05H10.2
-0.345951962966684	AGIWUR4595
CB4856	CT	II	14866716	14869577	WBGene00011045	gpx-2	R05H10.5
-0.0516799981105613	AGIWUR24659
CB4856	CT	II	14869944	14883054	WBGene00011044	R05H10.3	R05H10.3
0.10201492308693	AGIWUR38905
CB4856	CT	II	14902493	14907533	WBGene00000584	cog-1	R03C1.3 0.164829684151371
AGIWUR6298;AGIWUR13164;AGIWUR13360;AGIWUR13897;AGIWUR23597;AGIWUR32475
CB4856	CT	II	14907508	14910405	WBGene00010987	R03C1.1 R03C1.1 -0.315167282104676
AGIWUR36717;AGIWUR40106
CB4856	CT	II	14919705	14953312	WBGene00008443	pde-3	E01F3.1 0.0516051829383901
AGIWUR2735;AGIWUR5932;AGIWUR6278;AGIWUR7192;AGIWUR10161;AGIWUR10894;AGIWUR16006;AGIWUR25584;AGIWUR29713;AGIWUR40204;AGIWUR41494
CB4856	CT	II	14954278	14958189	WBGene00013149	Y53F4B.1	Y53F4B.1
0.216104345392923	AGIWUR36946
CB4856	CT	II	14959675	14964108	WBGene00001247	elo-9	Y53F4B.2
-0.737208374315749	AGIWUR40927
CB4856	CT	II	14968633	14969354	WBGene00013150	Y53F4B.3	Y53F4B.3
0.0845631265319913	AGIWUR40915
CB4856	CT	II	14969617	14977005	WBGene00013151	nsun-5	Y53F4B.4
-0.128202343716527	AGIWUR13954;AGIWUR28834
CB4856	CT	II	14982592	14984009	WBGene00013152	Y53F4B.5	Y53F4B.5
-0.498442132916393	AGIWUR25839
CB4856	CT	II	14985806	14987225	WBGene00013153	Y53F4B.6	Y53F4B.6
0.0302641093110783	AGIWUR3788;AGIWUR23842;AGIWUR26105;AGIWUR36212;AGIWUR36748
CB4856	CT	II	14988471	14992667	WBGene00044726	Y53F4B.43	Y53F4B.43
0.185872689320963	AGIWUR13396;AGIWUR22421;AGIWUR27105
CB4856	CT	II	14994197	14997444	WBGene00013155	Y53F4B.8	Y53F4B.8
0.172009894974296	AGIWUR38021
CB4856	CT	II	15001896	15003510	WBGene00013157	Y53F4B.10	Y53F4B.10
0.161190738006901	AGIWUR34468
CB4856	CT	II	15004869	15010416	WBGene00013156	Y53F4B.9	Y53F4B.9
0.265452828768325	AGIWUR36328
CB4856	CT	II	15010748	15013408	WBGene00013158	Y53F4B.11	Y53F4B.11
-0.153212224519558	AGIWUR2609
CB4856	CT	II	15019223	15025768	WBGene00013159	Y53F4B.12	Y53F4B.12
-0.0696426514174391	AGIWUR38902

CB4856	CT	II	15026137	15037807	WBGene00013160	Y53F4B.13	Y53F4B.13	0.0214010294677909	AGIWUR26575
CB4856	CT	II	15037991	15039297	WBGene00013161	Y53F4B.14	Y53F4B.14	0.133019070373211	AGIWUR12472;AGIWUR18353
CB4856	CT	II	15039404	15048904	WBGene00000208	asc-1	Y53F4B.15	0.288885507810342	AGIWUR15313;AGIWUR41331;AGIWUR44728
CB4856	CT	II	15052085	15055718	WBGene00013162	Y53F4B.16	Y53F4B.16	-0.017231399638562	AGIWUR16810
CB4856	CT	II	15064267	15071671	WBGene00013164	Y53F4B.18	Y53F4B.18	0.00353901064692863	AGIWUR33080
CB4856	CT	II	15077218	15079475	WBGene00013165	Y53F4B.19	Y53F4B.19	0.0816894386889429	AGIWUR8305;AGIWUR20252;AGIWUR43799
CB4856	CT	II	15084476	15089626	WBGene00013166	Y53F4B.20	Y53F4B.20	-0.0126871509749451	AGIWUR10663
CB4856	CT	II	15090172	15109042	WBGene00013167	Y53F4B.21	Y53F4B.21	-0.215745513934703	AGIWUR3656;AGIWUR23425;AGIWUR24369;AGIWUR38305;AGIWUR42729
CB4856	CT	II	15110144	15116306	WBGene00013176	Y53F4B.39	Y53F4B.39	0.111303046956872	AGIWUR38178
CB4856	CT	II	15117686	15117963	WBGene00044729	Y53F4B.46	Y53F4B.46	0.0139806772211095	AGIWUR5825;AGIWUR8271;AGIWUR12857;AGIWUR33283;AGIWUR37234
CB4856	CT	II	15118282	15118951	WBGene00014920	Y53F4B.38	Y53F4B.38	-0.11764036074347	AGIWUR17970;AGIWUR35908
CB4856	CT	II	15120301	15123345	WBGene00013168	arp-1	Y53F4B.22	-0.189836958005641	AGIWUR3603
CB4856	CT	II	15124504	15126365	WBGene00013169	Y53F4B.23	Y53F4B.23	0.0575041648068799	AGIWUR31793;AGIWUR32003
CB4856	CT	II	15136186	15145370	WBGene00013171	Y53F4B.25	Y53F4B.25	0.283872999157982	AGIWUR5360
CB4856	CT	II	15145894	15151934	WBGene00013173	Y53F4B.27	Y53F4B.27	0.0795286937251456	AGIWUR27560
CB4856	CT	II	15158180	15163614	WBGene00004234	ptr-20	Y53F4B.28	0.181943332861598	AGIWUR24814
CB4856	CT	II	15163808	15164646	WBGene00001774	gst-26	Y53F4B.29	-0.60473434746473	AGIWUR6018
CB4856	CT	II	15164890	15165731	WBGene00001775	gst-27	Y53F4B.30	-0.551402692138595	AGIWUR31989
CB4856	CT	II	15166194	15167406	WBGene00001776	gst-28	Y53F4B.31	-0.223744881249475	AGIWUR1127
CB4856	CT	II	15168538	15169860	WBGene00001777	gst-29	Y53F4B.32		

0.463053512561	AGIWUR31465
CB4856	CT	II	15171163	15171912	WBGene00001787	gst-39	Y53F4B.33
-0.182736374743982	AGIWUR6423
CB4856	CT	II	15174271	15174965	WBGene00001779	gst-31	Y53F4B.35
0.134264363914876	AGIWUR37191
CB4856	CT	II	15175943	15177271	WBGene00013175	Y53F4B.36	Y53F4B.36
-0.0202601480210927	AGIWUR4348;AGIWUR23408
CB4856	CT	II	15177372	15178594	WBGene00001780	gst-32	Y53F4B.37
0.0269013645331952	AGIWUR32214
CB4856	CT	II	15179971	15183232	WBGene00001782	gst-34	Y1H11.1	-0.288967458574227
AGIWUR779;AGIWUR18828;AGIWUR37638
CB4856	CT	II	15183801	15185385	WBGene00001783	gst-35	Y1H11.2	0.583768194320731
AGIWUR2855
CB4856	CT	II	15196214	15198075	WBGene00021598	Y46E12BR.1	Y46E12BR.1
1.33201616629279	AGIWUR26873
CB4856	CT	II	15207443	15229725	WBGene00021597	spsb-1	Y46E12BL.4
0.32491557709869	AGIWUR15839
CB4856	CT	II	15230343	15232803	WBGene00021596	spsb-2	Y46E12BL.3
0.055735621588882	AGIWUR17487
CB4856	CT	II	15233153	15244931	WBGene00021595	Y46E12BL.2	Y46E12BL.2
0.192407523896552	AGIWUR12187
CB4856	CT	II	15268212	15275689	WBGene00007064	2RSSE.1	2RSSE.1	0.133898320762639
AGIWUR40832;AGIWUR43546
CB4856	CT	III	1271	3092	WBGene00008352	fbxa-6	cTel54X.1	-0.171066142426852
AGIWUR922;AGIWUR23412;AGIWUR38130
CB4856	CT	III	3321	11940	WBGene00019183	npr-30	H10E21.2	-0.281522536766681
AGIWUR33824
CB4856	CT	III	12170	14869	WBGene00003670	nhr-80	H10E21.3	0.084027507651486
AGIWUR301
CB4856	CT	III	15720	17332	WBGene00019182	H10E21.1	H10E21.1	-0.065834163897561
AGIWUR6087;AGIWUR27387
CB4856	CT	III	17316	19675	WBGene00019184	H10E21.4	H10E21.4	-0.363566058963649
AGIWUR9512
CB4856	CT	III	20531	23962	WBGene00019185	H10E21.5	H10E21.5	0.271425034865935
AGIWUR42375
CB4856	CT	III	35454	39462	WBGene00021041	W05G11.4	W05G11.4	0.25579970472377
AGIWUR21738
CB4856	CT	III	41061	42021	WBGene00000663	col-88	W05G11.3	-0.445090519216041
AGIWUR40441

CB4856	CT	III	57702	58682	WBGene00021040	W05G11.2	W05G11.2	-0.145343078182268	AGIWUR3886
CB4856	CT	III	62888	66261	WBGene00021043	pck-1	W05G11.6	-0.0126830378163927	AGIWUR11857;AGIWUR14639;AGIWUR19103;AGIWUR23802;AGIWUR24065;AGIWUR25842;AGIWUR34655;AGIWUR38529;AGIWUR41918
CB4856	CT	III	69073	69817	WBGene00005014	spt-4	F54C4.2	0.176785939876853	AGIWUR11937
CB4856	CT	III	70442	86384	WBGene00018794	attf-3	F54C4.3	-0.148381975972772	AGIWUR6291
CB4856	CT	III	86677	87381	WBGene00018795	F54C4.4	F54C4.4	0.449491576459593	AGIWUR11681
CB4856	CT	III	87559	89243	WBGene00018793	mrpl-40	F54C4.1	-0.098480963225537	AGIWUR18193
CB4856	CT	III	89453	94809	WBGene00003931	pat-4	C29F9.7	-0.0426199339455806	AGIWUR26748
CB4856	CT	III	95394	97726	WBGene00016222	C29F9.8	C29F9.8	-0.0679077805472392	AGIWUR17287
CB4856	CT	III	106824	107899	WBGene00016224	fbxa-57	C29F9.10	-0.205994098250439	AGIWUR32662
CB4856	CT	III	109322	110185	WBGene00016225	fbxa-58	C29F9.11	0.177711186177678	AGIWUR32908
CB4856	CT	III	114903	116619	WBGene00016221	C29F9.6	C29F9.6	0.0623317828332786	AGIWUR41447
CB4856	CT	III	117707	118767	WBGene00016220	C29F9.5	C29F9.5	-1.01778667213528	AGIWUR24053
CB4856	CT	III	120492	124641	WBGene00016219	pals-24	C29F9.4	-0.250519379003546	AGIWUR14875;AGIWUR40400
CB4856	CT	III	124778	126527	WBGene00016218	pals-23	C29F9.3	-0.535061157913591	AGIWUR245;AGIWUR5444;AGIWUR26093;AGIWUR30406;AGIWUR30704;AGIWUR34729;AGIWUR34764
CB4856	CT	III	130180	130750	WBGene00016227	nhr-280	C29F9.13	0.0212801162877509	AGIWUR8933;AGIWUR19189;AGIWUR27290;AGIWUR38196;AGIWUR39253
CB4856	CT	III	130947	131751	WBGene00016217	C29F9.2	C29F9.2	-0.205934686351929	AGIWUR9509
CB4856	CT	III	132418	134401	WBGene00016216	pals-22	C29F9.1	-0.215174579079147	AGIWUR40864
CB4856	CT	III	134494	137975	WBGene00020540	pals-25	T17A3.2	-0.035351271538751	AGIWUR36367
CB4856	CT	III	142121	143217	WBGene00020542	fbxb-82	T17A3.4	0.333074261263603	AGIWUR11919
CB4856	CT	III	143631	144792	WBGene00020543	fbxb-83	T17A3.6	-0.0133842120007639	AGIWUR12633
CB4856	CT	III	144994	145299	WBGene00020548	T17A3.12	T17A3.12	-0.187328222124609	AGIWUR35103
CB4856	CT	III	145360	146491	WBGene00020544	fbxb-84	T17A3.7	-0.0658605514980994	AGIWUR4909
CB4856	CT	III	146917	147607	WBGene00269434	sqst-5	T17A3.13	-0.228108552905274	AGIWUR8165;AGIWUR15973;AGIWUR24457
CB4856	CT	III	150481	151805	WBGene00020547	srz-7	T17A3.11	-0.0350774073281315	AGIWUR1435
CB4856	CT	III	152572	153418	WBGene00020545	adbp-2	T17A3.9	0.182878807584201	AGIWUR16101
CB4856	CT	III	154475	156674	WBGene00020546	T17A3.10	T17A3.10	0.0875111643557123	AGIWUR19180
CB4856	CT	III	156824	163542	WBGene00006894	ver-1	T17A3.1	0.313606354818529	AGIWUR29063
CB4856	CT	III	163598	164875	WBGene00018244	F40G9.8	F40G9.8	0.0442192991209591	AGIWUR31936

CB4856	CT	III	165690	166285	WBGene00018243	F40G9.7	F40G9.7	-0.456896960239241	AGIWUR25649
CB4856	CT	III	167385	169556	WBGene00189954	F40G9.18	F40G9.18	-0.0322599176047369	AGIWUR35541
CB4856	CT	III	173797	174558	WBGene00189955	F40G9.19	F40G9.19	0.0577627405254209	AGIWUR17083
CB4856	CT	III	174609	175833	WBGene00018246	clec-148	F40G9.10	0.18261590736724	AGIWUR4961
CB4856	CT	III	176111	177815	WBGene00018248	F40G9.14	F40G9.14	-0.152642063624723	AGIWUR9016
CB4856	CT	III	179542	180140	WBGene00018242	F40G9.6	F40G9.6	-0.0129009013224355	AGIWUR33631
CB4856	CT	III	181561	182981	WBGene00077531	F40G9.15	F40G9.15	-0.416710157889188	AGIWUR32497;AGIWUR36577;AGIWUR39549
CB4856	CT	III	183231	184915	WBGene00018241	F40G9.5	F40G9.5	-0.142802726027236	AGIWUR37387
CB4856	CT	III	185652	186922	WBGene00001963	btb-1	F40G9.4	-0.134736609071621	AGIWUR9514
CB4856	CT	III	186951	188131	WBGene00003510	mxl-2	F40G9.11	0.224776551642647	AGIWUR14877
CB4856	CT	III	188838	190974	WBGene00006715	ubc-20	F40G9.3	-0.0241101120519431	AGIWUR5501;AGIWUR11356;AGIWUR36186;AGIWUR37947;AGIWUR38131
CB4856	CT	III	190986	191981	WBGene00018240	cox-17	F40G9.2	-0.242959010973559	AGIWUR31562
CB4856	CT	III	194460	210063	WBGene00185078	F40G9.17	F40G9.17	-0.173201672730205	AGIWUR33957
CB4856	CT	III	209992	230450	WBGene00002230	klp-20	Y50D7A.6	-0.000291987493225676	AGIWUR17698
CB4856	CT	III	230429	235121	WBGene00021755	hpo-38	Y50D7A.5	0.282968855077666	AGIWUR10164
CB4856	CT	III	235844	254067	WBGene00021754	hpo-29	Y50D7A.4	-0.398509631633999	AGIWUR23049
CB4856	CT	III	254535	264636	WBGene00021753	Y50D7A.3	Y50D7A.3	-0.195321837738902	AGIWUR3020;AGIWUR32669;AGIWUR45172
CB4856	CT	III	265090	266593	WBGene00021759	Y50D7A.11	Y50D7A.11	-0.175875546812971	AGIWUR17396
CB4856	CT	III	268458	283009	WBGene00021752	xpd-1	Y50D7A.2	-0.322164179830665	AGIWUR16560
CB4856	CT	III	284117	289444	WBGene00021757	taco-1	Y50D7A.9	-0.390114569129768	AGIWUR6437
CB4856	CT	III	292640	314266	WBGene00021751	Y50D7A.1	Y50D7A.1	-0.041787710170663	AGIWUR30024
CB4856	CT	III	315460	321721	WBGene00021758	Y50D7A.10	Y50D7A.10	-0.0385453824725195	AGIWUR2997;AGIWUR9957;AGIWUR40637

CB4856 CT III 335956 343241 WBGene00021756 Y50D7A.8 Y50D7A.8 -0.0117433771069173
AGIWUR15243
CB4856 CT III 344307 378250 WBGene00001561 gei-4 W07B3.2 0.276152071213056
AGIWUR34;AGIWUR2203;AGIWUR4322;AGIWUR9947;AGIWUR12564;AGIWUR37982;AGIWUR38490;AGIWUR43785
CB4856 CT III 378004 381298 WBGene00019636 gsto-3 K10F12.4 0.24803250469509
AGIWUR38396
CB4856 CT III 381900 382570 WBGene00019639 K10F12.7 K10F12.7 -0.034757916263878
AGIWUR31688
CB4856 CT III 383771 384827 WBGene00023104 K10F12.8 K10F12.8 0.0591554237874075
AGIWUR25628;AGIWUR32699;AGIWUR33484;AGIWUR37265;AGIWUR42159
CB4856 CT III 398730 399392 WBGene00019638 K10F12.6 K10F12.6 0.0601756951814565
AGIWUR36075
CB4856 CT III 402384 405589 WBGene00021909 Y55B1BL.1 Y55B1BL.1 0.42436074973592
AGIWUR8894;AGIWUR10666;AGIWUR13026;AGIWUR20424
CB4856 CT III 408094 418738 WBGene00021910 stim-1 Y55B1BM.1 -0.217347589411425
AGIWUR37725
CB4856 CT III 429300 431750 WBGene00021913 cec-8 Y55B1BR.3 0.48158597788988
AGIWUR21073
CB4856 CT III 434891 440142 WBGene00021912 Y55B1BR.2 Y55B1BR.2 -0.0814783558120755
AGIWUR10365
CB4856 CT III 453785 463811 WBGene00044401 Y55B1BR.6 Y55B1BR.6 0.113034025670586
AGIWUR16016;AGIWUR19782;AGIWUR25028;AGIWUR28804;AGIWUR32799
CB4856 CT III 464715 471702 WBGene00021914 magu-1 Y55B1BR.4 -0.157774474901204
AGIWUR9636;AGIWUR33177;AGIWUR35853
CB4856 CT III 472301 475349 WBGene00017328 F10C5.2 F10C5.2 0.0608271515370937 AGIWUR41874
CB4856 CT III 475454 482322 WBGene00003134 mat-3 F10C5.1 0.0231545196576519 AGIWUR30332
CB4856 CT III 486123 486547 WBGene00003770 nlp-32 F30H5.2 0.147043100537493 AGIWUR5416
CB4856 CT III 488764 489452 WBGene00044288 F30H5.5 F30H5.5 -0.0895332617160909
AGIWUR1090;AGIWUR12017;AGIWUR13475;AGIWUR25827
CB4856 CT III 491547 502625 WBGene00006781 unc-45 F30H5.1 -0.464482039007077 AGIWUR15042
CB4856 CT III 505241 512217 WBGene00017937 F30H5.3 F30H5.3 0.55947730422269 AGIWUR28952
CB4856 CT III 513720 516614 WBGene00020704 T22F7.5 T22F7.5 -0.12660065163419 AGIWUR18109
CB4856 CT III 518356 524044 WBGene00020702 T22F7.3 T22F7.3 0.100959981107196 AGIWUR35438
CB4856 CT III 525234 526924 WBGene00020703 T22F7.4 T22F7.4 -0.80458631586461 AGIWUR20184
CB4856 CT III 542066 546537 WBGene00020701 T22F7.1 T22F7.1 0.230712735814501 AGIWUR2200
CB4856 CT III 557518 572137 WBGene00015155 B0353.1 B0353.1 -0.00831584104965266
AGIWUR21682;AGIWUR27532;AGIWUR33521
CB4856 CT III 573984 576447 WBGene00021903 Y55B1AL.1 Y55B1AL.1 0.289315420514834
AGIWUR5302

CB4856	CT	III	576956	577567	WBGene00021904	Y55B1AL.2	Y55B1AL.2	0.0950196165765576	AGIWUR5763;AGIWUR12164;AGIWUR16417;AGIWUR23626;AGIWUR25079;AGIWUR43817
CB4856	CT	III	577684	587105	WBGene00021905	helq-1	Y55B1AL.3	-0.0325124899546374	AGIWUR20903
CB4856	CT	III	587244	598117	WBGene00021906	Y55B1AR.2	Y55B1AR.2	-0.159082912003569	AGIWUR1743;AGIWUR21403;AGIWUR32389
CB4856	CT	III	599509	600366	WBGene00002269	lec-6	Y55B1AR.1	-0.326216832811707	AGIWUR670;AGIWUR6324;AGIWUR18866;AGIWUR21263;AGIWUR33575;AGIWUR43375
CB4856	CT	III	602708	603822	WBGene00021907	Y55B1AR.3	Y55B1AR.3	-0.26858880981003	AGIWUR22782
CB4856	CT	III	604003	605515	WBGene00021908	Y55B1AR.4	Y55B1AR.4	-0.178977358441969	AGIWUR6153
CB4856	CT	III	606004	606994	WBGene00020566	ttr-7	T19C3.9	0.141686571609679	AGIWUR12291
CB4856	CT	III	607045	609973	WBGene00020559	best-17	T19C3.1	-0.255020949163535	AGIWUR246
CB4856	CT	III	610881	614547	WBGene00020561	T19C3.3	T19C3.3	-0.0918668543866197	AGIWUR28405
CB4856	CT	III	614707	617447	WBGene00020560	T19C3.2	T19C3.2	-0.28144147764403	AGIWUR27733;AGIWUR37800
CB4856	CT	III	618246	620254	WBGene00020562	T19C3.4	T19C3.4	0.206071170169038	AGIWUR19194
CB4856	CT	III	624607	627938	WBGene00020563	T19C3.5	T19C3.5	-0.00865403471339832	AGIWUR17749
CB4856	CT	III	628175	630987	WBGene00020564	T19C3.6	T19C3.6	-0.0450993578164134	AGIWUR11209
CB4856	CT	III	631938	634645	WBGene00001412	fem-2	T19C3.8	0.151517941988608	AGIWUR12509
CB4856	CT	III	634893	636167	WBGene00020565	T19C3.7	T19C3.7	0.394177364555407	AGIWUR7024
CB4856	CT	III	636479	638419	WBGene00021063	sbds-1	W06E11.4	-0.133648787291804	AGIWUR37227
CB4856	CT	III	638531	640226	WBGene00044319	tag-266	W06E11.5	-0.105431196043861	AGIWUR19835;AGIWUR27606
CB4856	CT	III	640790	641625	WBGene00044318	tag-267	W06E11.2	-0.0672094576417059	AGIWUR22516
CB4856	CT	III	642059	645613	WBGene00021061	W06E11.1	W06E11.1	-0.150183237225118	AGIWUR14099;AGIWUR36340
CB4856	CT	III	646754	654463	WBGene00015648	orai-1	C09F5.2	-0.261572045766735	AGIWUR14468;AGIWUR23243
CB4856	CT	III	658709	662732	WBGene00015647	C09F5.1	C09F5.1	0.456341749272227	AGIWUR8004
CB4856	CT	III	662847	666265	WBGene00015649	C09F5.3	C09F5.3	0.292406883228913	AGIWUR9052
CB4856	CT	III	668098	668433	WBGene00020923	W02B3.3	W02B3.3	0.123875420664643	AGIWUR26697
CB4856	CT	III	676122	680253	WBGene00001709	grk-2	W02B3.2	-0.0149925660711219	AGIWUR2530;AGIWUR28189
CB4856	CT	III	680634	683418	WBGene00020924	W02B3.4	W02B3.4	-0.0166931747695802	AGIWUR42698
CB4856	CT	III	686237	692033	WBGene00020927	W02B3.7	W02B3.7	-0.0618967631954428	AGIWUR7724

CB4856 CT III 688739 715823 WBGene00016029 elf-1 C24A1.2 0.232495413929518 AGIWUR13322
CB4856 CT III 725022 726155 WBGene00016028 flp-24 C24A1.1 0.145044584948083
AGIWUR8330;AGIWUR16177
CB4856 CT III 727211 752426 WBGene00003936 pat-12 T17H7.4 -0.118779118345472
AGIWUR17747;AGIWUR24713;AGIWUR43935
CB4856 CT III 755298 758080 WBGene00020551 T17H7.7 T17H7.7 0.239993852136539 AGIWUR17028
CB4856 CT III 761779 762392 WBGene00018363 nlp-52 F42G9.4 0.635202990406633 AGIWUR13691
CB4856 CT III 763810 771366 WBGene00004212 ptl-1 F42G9.9 -0.0596700847627796
AGIWUR17260;AGIWUR23112;AGIWUR30034;AGIWUR30114;AGIWUR31642;AGIWUR43414
CB4856 CT III 771577 772819 WBGene00018365 tpst-2 F42G9.8 0.387848802849148 AGIWUR18955
CB4856 CT III 773403 774159 WBGene00000882 cyn-6 F42G9.2 -0.0741075179593227
AGIWUR2632;AGIWUR7089;AGIWUR13686;AGIWUR21840;AGIWUR23853;AGIWUR42813
CB4856 CT III 774321 778871 WBGene00000117 alh-11 F42G9.5 0.00845264716776439
AGIWUR25312;AGIWUR36861
CB4856 CT III 783714 786092 WBGene00018362 F42G9.1 F42G9.1 -0.0938956708824854
AGIWUR2021;AGIWUR26590;AGIWUR41729
CB4856 CT III 786532 793733 WBGene00004922 snt-2 F42G9.7 -0.342213402997301
AGIWUR6947;AGIWUR14223;AGIWUR27057;AGIWUR28596;AGIWUR35258;AGIWUR42534
CB4856 CT III 794300 795120 WBGene00004498 rps-29 B0412.4 -0.141674193641126
AGIWUR4823;AGIWUR17511;AGIWUR19190;AGIWUR30443;AGIWUR33838;AGIWUR34596;AGIWUR44337
CB4856 CT III 795136 809439 WBGene00015173 trpp-11 B0412.3 -0.0470536136633939 AGIWUR28634
CB4856 CT III 811828 813773 WBGene00000903 daf-7 B0412.2 0.19600735202555 AGIWUR34983
CB4856 CT III 816145 821730 WBGene00000895 dac-1 B0412.1 -0.300694477486022 AGIWUR21958
CB4856 CT III 823222 826408 WBGene00019328 clec-149 K02F3.5 -0.151518755167496
AGIWUR8225;AGIWUR30863
CB4856 CT III 828590 832277 WBGene00019329 ceeh-1 K02F3.6 0.143710411145002
AGIWUR5535;AGIWUR20354;AGIWUR29930;AGIWUR42060
CB4856 CT III 833858 835808 WBGene00019327 zip-2 K02F3.4 0.220399112776811 AGIWUR37051
CB4856 CT III 835815 839591 WBGene00019331 dos-3 K02F3.8 0.204473007947374
AGIWUR14933;AGIWUR21593
CB4856 CT III 840460 841366 WBGene00019332 K02F3.9 K02F3.9 0.209887600555263 AGIWUR30193
CB4856 CT III 842935 843802 WBGene00001392 far-8 K02F3.3 -0.020538710382187 AGIWUR16155
CB4856 CT III 845842 850586 WBGene00019326 K02F3.2 K02F3.2 -0.106092357056539 AGIWUR6966
CB4856 CT III 851328 852396 WBGene00019333 moma-1 K02F3.10 0.114858322609837
AGIWUR42016
CB4856 CT III 852801 853909 WBGene00004388 rnp-5 K02F3.11 0.0284949621753707
AGIWUR17078
CB4856 CT III 854219 856535 WBGene00006430 tag-51 K02F3.1 -0.321828780112877
AGIWUR1565;AGIWUR16267;AGIWUR32843

CB4856	CT	III	856560	860090	WBGene00019334	K02F3.12	K02F3.12	-0.0917045778707175	AGIWUR15344;AGIWUR19706;AGIWUR40168
CB4856	CT	III	860198	861324	WBGene00000202	exos-4.2	Y6D11A.1	0.0112878015025935	AGIWUR23712;AGIWUR35800
CB4856	CT	III	862306	865399	WBGene00021170	arx-4	Y6D11A.2	-0.0339770341926062	AGIWUR30094
CB4856	CT	III	867512	869987	WBGene00020758	T24C4.2	T24C4.2	0.178335786500624	AGIWUR25065;AGIWUR38112;AGIWUR42624
CB4856	CT	III	871783	872385	WBGene00020759	T24C4.3	T24C4.3	0.0375191036351867	AGIWUR14423;AGIWUR16344;AGIWUR41032;AGIWUR42660
CB4856	CT	III	872622	873531	WBGene00020760	T24C4.4	T24C4.4	-0.0599508885673088	AGIWUR2930;AGIWUR34970;AGIWUR38120;AGIWUR39190
CB4856	CT	III	874310	875325	WBGene00044681	T24C4.8	T24C4.8	-0.248205941507763	AGIWUR2329;AGIWUR6257;AGIWUR26491
CB4856	CT	III	877435	879734	WBGene00020761	T24C4.5	T24C4.5	-0.341307689854169	AGIWUR2668
CB4856	CT	III	879860	881506	WBGene00020757	ucr-2.3	T24C4.1	-0.0237952138949122	AGIWUR35884
CB4856	CT	III	881443	886789	WBGene00020762	zer-1	T24C4.6	0.322693303339131	AGIWUR39357;AGIWUR44580
CB4856	CT	III	889955	895634	WBGene00020763	ztf-18	T24C4.7	0.203324376171936	AGIWUR8590;AGIWUR22251;AGIWUR24829;AGIWUR30563;AGIWUR41360;AGIWUR43319
CB4856	CT	III	895786	900158	WBGene00017758	F23H11.2	F23H11.2	-0.0206139024240919	AGIWUR11974
CB4856	CT	III	900138	901444	WBGene00017759	sucl-2	F23H11.3	-0.0823609142528881	AGIWUR17484
CB4856	CT	III	902151	907539	WBGene00017760	F23H11.4	F23H11.4	-0.123602819512195	AGIWUR6869;AGIWUR39276
CB4856	CT	III	908328	909902	WBGene00017763	crls-1	F23H11.9	0.151951182175329	AGIWUR8468
CB4856	CT	III	909957	910589	WBGene00000263	F23H11.5	F23H11.5	-0.00955183768296308	AGIWUR6932;AGIWUR8088;AGIWUR33553;AGIWUR37413;AGIWUR37756;AGIWUR41694
CB4856	CT	III	911261	912243	WBGene00017757	bra-2	F23H11.1	-0.186786705542904	AGIWUR1147
CB4856	CT	III	913248	916997	WBGene00017761	F23H11.6	F23H11.6	-0.105423150156428	AGIWUR33605
CB4856	CT	III	917088	919422	WBGene00017762	F23H11.7	F23H11.7	0.220721762810337	AGIWUR13839
CB4856	CT	III	919528	926314	WBGene00003969	pef-1	F23H11.8	0.638921338030487	AGIWUR10891;AGIWUR36664
CB4856	CT	III	933585	936067	WBGene00020453	fbxa-55	T12B5.6	0.0151226635165003	AGIWUR1047

CB4856	CT	III	937302	938061	WBGene00020454	fbxa-56	T12B5.7	-0.403841922262373	AGIWUR34001
CB4856	CT	III	938869	939437	WBGene00020452	fbxa-22	T12B5.5	-0.847039514795992	AGIWUR25671
CB4856	CT	III	940879	943469	WBGene00020455	fbxa-59	T12B5.8	-0.0877378881079203	AGIWUR41567
CB4856	CT	III	944795	946298	WBGene00020456	fbxa-60	T12B5.10	-0.629957676224571	AGIWUR20061
CB4856	CT	III	947848	949092	WBGene00020459	fbxa-23	T12B5.13	2.36330694025098	AGIWUR31728
CB4856	CT	III	950869	952264	WBGene00020451	fbxa-11	T12B5.4	-0.429566134660313	AGIWUR23723
CB4856	CT	III	952419	953803	WBGene00020450	fbxa-10	T12B5.3	0.0840645524683783	AGIWUR38989
CB4856	CT	III	954291	954653	WBGene00050914	T12B5.15	T12B5.15	-0.973942800319897	AGIWUR1132;AGIWUR1851;AGIWUR22450;AGIWUR38458;AGIWUR39446
CB4856	CT	III	954873	955295	WBGene00050913	T12B5.14	T12B5.14	-0.76650079402279	AGIWUR18737;AGIWUR28984;AGIWUR31470;AGIWUR32166;AGIWUR41902
CB4856	CT	III	956334	957947	WBGene00020449	fbxa-54	T12B5.2	-0.338576831919813	AGIWUR27557
CB4856	CT	III	960268	961347	WBGene00020458	fbxa-70	T12B5.12	-0.0684744034939495	AGIWUR18579
CB4856	CT	III	962304	963679	WBGene00020448	fbxa-51	T12B5.1	0.0456263917342392	AGIWUR21655
CB4856	CT	III	966654	972269	WBGene00019912	R06B10.1	R06B10.1	-0.188864502458175	AGIWUR22141
CB4856	CT	III	973311	980451	WBGene00019913	R06B10.2	R06B10.2	-0.405334555940555	AGIWUR38698
CB4856	CT	III	977386	988446	WBGene00019915	tbc-10	R06B10.5	-0.0946647106107026	AGIWUR23747;AGIWUR27330;AGIWUR30813
CB4856	CT	III	991881	1001907	WBGene00006615	trp-2	R06B10.4	0.0557504336208612	AGIWUR18580;AGIWUR36258
CB4856	CT	III	1002707	1006298	WBGene00019914	clec-150	R06B10.3	-0.212024340772274	AGIWUR43057
CB4856	CT	III	1010535	1011385	WBGene00021337	Y34F4.2	Y34F4.2	-0.331271289422034	AGIWUR37101
CB4856	CT	III	1015680	1016136	WBGene00021338	Y34F4.3	Y34F4.3	-0.0811518334574828	AGIWUR27784
CB4856	CT	III	1020954	1021876	WBGene00021336	Y34F4.1	Y34F4.1	0.441921047492465	AGIWUR24935;AGIWUR25182
CB4856	CT	III	1023221	1024561	WBGene00021339	Y34F4.4	Y34F4.4	-0.369886100231311	AGIWUR4205
CB4856	CT	III	1026916	1031065	WBGene00021340	Y34F4.5	Y34F4.5	0.353637560539305	AGIWUR17466
CB4856	CT	III	1031324	1037212	WBGene00015267	chin-1	BE0003N10.2	-0.0224318386409337	AGIWUR40526
CB4856	CT	III	1037492	1046261	WBGene00015266	BE0003N10.1	BE0003N10.1	-0.108404850213457	AGIWUR20325;AGIWUR32995
CB4856	CT	III	1045809	1053246	WBGene00015268	BE0003N10.3	BE0003N10.3	0.191398791345984	AGIWUR996

CB4856	CT	III	1062793	1076555	WBGene00006697	uaf-1	Y92C3B.2	0.0965594384298107	AGIWUR8964;AGIWUR25966
CB4856	CT	III	1078150	1080775	WBGene00004277	rab-18	Y92C3B.3	-0.180466304060765	AGIWUR11765;AGIWUR17720;AGIWUR21390;AGIWUR30018;AGIWUR33466;AGIWUR38575
CB4856	CT	III	1080901	1081300	WBGene00022357	kbp-4	Y92C3B.1	0.0649383277289974	AGIWUR33018
CB4856	CT	III	1081402	1095073	WBGene00003917	par-2	F58B6.3	-0.027386975938879	AGIWUR17088;AGIWUR25963
CB4856	CT	III	1103224	1111923	WBGene00019030	exc-6	F58B6.2	0.0311392816580529	AGIWUR41199
CB4856	CT	III	1112279	1116374	WBGene00019029	F58B6.1	F58B6.1	-0.340654167601494	AGIWUR6761;AGIWUR17185;AGIWUR25640;AGIWUR33066;AGIWUR42074
CB4856	CT	III	1116424	1142927	WBGene00022104	acsd-1	Y71D11A.3	0.205231792596983	AGIWUR10744
CB4856	CT	III	1143172	1145211	WBGene00004891	smr-1	Y71D11A.2	0.134513025769241	AGIWUR35021;AGIWUR39134
CB4856	CT	III	1152044	1181137	WBGene00022103	cdh-12	Y71D11A.1	0.00530612907720318	AGIWUR9139;AGIWUR11394;AGIWUR17116;AGIWUR32780;AGIWUR32922
CB4856	CT	III	1181990	1184942	WBGene00022490	Y119D3B.13	Y119D3B.13	-0.169870179880835	AGIWUR31799
CB4856	CT	III	1185248	1193812	WBGene00022491	Y119D3B.14	Y119D3B.14	0.00052440916746832	AGIWUR26380
CB4856	CT	III	1194367	1196292	WBGene00022492	dss-1	Y119D3B.15	0.416502223776467	AGIWUR8941
CB4856	CT	III	1196560	1202008	WBGene00022493	mrpl-45	Y119D3B.16	-0.594686606108064	AGIWUR3926
CB4856	CT	III	1202548	1207346	WBGene00022489	Y119D3B.12	Y119D3B.12	-0.12196506170258	AGIWUR3387;AGIWUR12866;AGIWUR16578;AGIWUR21705;AGIWUR27552;AGIWUR35374;AGIWUR36012
CB4856	CT	III	1208104	1213650	WBGene00022488	orc-3	Y119D3B.11	-0.0256139603550594	AGIWUR15068
CB4856	CT	III	1214951	1215293	WBGene00022497	Y119D3B.21	Y119D3B.21	-0.20542684213882	AGIWUR32793
CB4856	CT	III	1216116	1222072	WBGene00003978	pes-4	Y119D3B.17	0.356586758664875	AGIWUR25541
CB4856	CT	III	1225459	1226065	WBGene00023205	Y119D3B.10	Y119D3B.10	0.235185363688966	AGIWUR12248
CB4856	CT	III	1236015	1237142	WBGene00022487	fbxa-21	Y119D3B.9	1.1894837375987	AGIWUR39017
CB4856	CT	III	1239260	1242780	WBGene00022486	fbxa-61	Y119D3B.8	0.259039152805098	AGIWUR8976;AGIWUR34220;AGIWUR38464
CB4856	CT	III	1243513	1245283	WBGene00022485	fbxa-63	Y119D3B.7	-0.362558614481774	

AGIWUR29215
CB4856 CT III 1247618 1249153 WBGene00044675 fbxa-76 Y119D3B.22 -0.150589021016208
AGIWUR7976;AGIWUR28206
CB4856 CT III 1249493 1251838 WBGene00022484 fbxa-77 Y119D3B.6 -0.474666167243864
AGIWUR5949
CB4856 CT III 1253384 1254401 WBGene00022494 fbxa-91 Y119D3B.18 -0.65574634159217
AGIWUR20168
CB4856 CT III 1267338 1269256 WBGene00013839 fbxa-32 ZC47.6 -0.239701141492231 AGIWUR6737
CB4856 CT III 1269558 1271451 WBGene00013838 fbxa-31 ZC47.5 -0.367775370363571 AGIWUR2303
CB4856 CT III 1272474 1273676 WBGene00013836 fbxa-29 ZC47.3 -0.157292086152107 AGIWUR25699
CB4856 CT III 1276249 1277360 WBGene00014979 ZC47.11 ZC47.11 -0.113834959228476 AGIWUR3704
CB4856 CT III 1277960 1278991 WBGene00014978 ZC47.8 ZC47.8 0.193402524931065 AGIWUR7537
CB4856 CT III 1279627 1281725 WBGene00013841 fbxa-33 ZC47.9 -0.0185495981526351 AGIWUR29339
CB4856 CT III 1282401 1284144 WBGene00013842 fbxa-34 ZC47.10 -0.012311523796626 AGIWUR33731
CB4856 CT III 1285016 1287119 WBGene00013845 fbxa-37 ZC47.14 -0.344174435756956 AGIWUR16699
CB4856 CT III 1287299 1288523 WBGene00013844 fbxa-50 ZC47.13 -0.229228297630845 AGIWUR40231
CB4856 CT III 1289747 1290938 WBGene00013843 fbxa-49 ZC47.12 -0.0359466487266715 AGIWUR28291
CB4856 CT III 1292051 1293215 WBGene00022479 fbxa-36 Y119D3A.4 -0.566868288980688
AGIWUR23896
CB4856 CT III 1297904 1299484 WBGene00022477 fbxa-28 Y119D3A.2 0.0111378836996892
AGIWUR43481
CB4856 CT III 1303714 1305461 WBGene00022476 fbxa-75 Y119D3A.1 -0.63627085817204
AGIWUR17956
CB4856 CT III 1305716 1308294 WBGene00022329 fbxa-79 Y82E9BL.13 0.0542380785708936
AGIWUR43569
CB4856 CT III 1309187 1311226 WBGene00022328 Y82E9BL.12 Y82E9BL.12 -0.0378128836937082
AGIWUR33175
CB4856 CT III 1311228 1313148 WBGene00022332 fbxa-20 Y82E9BL.16 -0.398650800324925
AGIWUR37149
CB4856 CT III 1313297 1315229 WBGene00022330 fbxa-80 Y82E9BL.14 -0.155715171026083
AGIWUR6535
CB4856 CT III 1315264 1316665 WBGene00022331 fbxa-19 Y82E9BL.15 -0.380165638975599
AGIWUR35810
CB4856 CT III 1319016 1321671 WBGene00022327 fbxa-15 Y82E9BL.11 -0.701003680317646
AGIWUR44682
CB4856 CT III 1322133 1324633 WBGene00022326 fbxa-14 Y82E9BL.10 -0.528999795988538
AGIWUR6137
CB4856 CT III 1332016 1333708 WBGene00194735 Y82E9BL.19 Y82E9BL.19 0.306779661495528
AGIWUR2791;AGIWUR6751

CB4856	CT	III	1333921	1335467	WBGene00023395	Y82E9BL.9	Y82E9BL.9	-0.259941388958341	AGIWUR32587
CB4856	CT	III	1336707	1340333	WBGene00022325	fbxa-5	Y82E9BL.8	0.018994051323963	AGIWUR16200
CB4856	CT	III	1340597	1342494	WBGene00022324	fbxa-26	Y82E9BL.7	0.103023878950628	AGIWUR41167
CB4856	CT	III	1342975	1344692	WBGene00022333	fbxa-27	Y82E9BL.17	0.292494127124786	AGIWUR35505
CB4856	CT	III	1344874	1345794	WBGene00022323	Y82E9BL.6	Y82E9BL.6	0.404849736029637	AGIWUR17019
CB4856	CT	III	1346315	1347128	WBGene00022322	Y82E9BL.5	Y82E9BL.5	0.066158038372496	AGIWUR43463
CB4856	CT	III	1347787	1349400	WBGene00022321	fbxa-25	Y82E9BL.4	0.398158570246363	AGIWUR40811
CB4856	CT	III	1349752	1350836	WBGene00022320	Y82E9BL.3	Y82E9BL.3	0.233001847741829	AGIWUR12446
CB4856	CT	III	1351216	1353371	WBGene00022319	Y82E9BL.2	Y82E9BL.2	0.0338538778091504	AGIWUR708
CB4856	CT	III	1354997	1355420	WBGene00022318	Y82E9BL.1	Y82E9BL.1	0.187865294020827	AGIWUR13081
CB4856	CT	III	1357080	1359135	WBGene00022341	Y82E9BR.8	Y82E9BR.8	0.12896228902328	AGIWUR34134
CB4856	CT	III	1359254	1359769	WBGene00022342	Y82E9BR.9	Y82E9BR.9	-0.244614762281882	AGIWUR2133
CB4856	CT	III	1360507	1362676	WBGene00022340	Y82E9BR.7	Y82E9BR.7	-0.0108483531239605	AGIWUR31551
CB4856	CT	III	1374152	1376542	WBGene00022352	Y82E9BR.20	Y82E9BR.20	0.226207651487564	AGIWUR2628;AGIWUR9977
CB4856	CT	III	1376945	1378003	WBGene00022344	Y82E9BR.11	Y82E9BR.11	1.59884726961734	AGIWUR18767
CB4856	CT	III	1379140	1380663	WBGene00255597	pals-21	Y82E9BR.32	0.142258102536085	AGIWUR43451
CB4856	CT	III	1390450	1391852	WBGene00022346	pals-17	Y82E9BR.13	-0.421968581074882	AGIWUR19418
CB4856	CT	III	1399972	1401305	WBGene00022337	pals-16	Y82E9BR.4	-0.43612221364117	AGIWUR19480;AGIWUR25923
CB4856	CT	III	1401946	1403460	WBGene00044761	pals-19	Y82E9BR.23	-0.459889549725011	AGIWUR1993
CB4856	CT	III	1404792	1406230	WBGene00022353	pals-18	Y82E9BR.21	-0.0684284025978776	

AGIWUR7285
CB4856 CT III 1406463 1409239 WBGene00022354 Y82E9BR.22 Y82E9BR.22 0.135811971858548
AGIWUR4189;AGIWUR14797;AGIWUR32752;AGIWUR42719;AGIWUR45183
CB4856 CT III 1409243 1414116 WBGene00022347 Y82E9BR.14 Y82E9BR.14 0.0296842744822925
AGIWUR7123;AGIWUR10710;AGIWUR14676;AGIWUR29289
CB4856 CT III 1415152 1417188 WBGene00001236 elc-1 Y82E9BR.15 -0.24079231281935
AGIWUR1874
CB4856 CT III 1419196 1419914 WBGene00022336 Y82E9BR.3 Y82E9BR.3
-0.00989169089857711 AGIWUR2471;AGIWUR6917;AGIWUR26470;AGIWUR28885;AGIWUR32675;AGIWUR33117;AGIWUR38718
CB4856 CT III 1419863 1427228 WBGene00022348 Y82E9BR.16 Y82E9BR.16 0.0645681283486592
AGIWUR3732;AGIWUR21213;AGIWUR28839;AGIWUR29791;AGIWUR32813;AGIWUR36733;AGIWUR40929
CB4856 CT III 1438832 1443190 WBGene00022335 Y82E9BR.2 Y82E9BR.2 0.0864173599112146
AGIWUR14741
CB4856 CT III 1447495 1455770 WBGene00022349 Y82E9BR.17 Y82E9BR.17 0.618128297444505
AGIWUR27623
CB4856 CT III 1458536 1466732 WBGene00022334 Y82E9BR.1 Y82E9BR.1 0.597783765438445
AGIWUR31260
CB4856 CT III 1467876 1483507 WBGene00022350 Y82E9BR.18 Y82E9BR.18 -0.106810229166598
AGIWUR44514
CB4856 CT III 1468643 1517993 WBGene00019716 M01G5.3 M01G5.3 0.147787733147883 AGIWUR43524
CB4856 CT III 1520224 1532768 WBGene00004905 snf-6 M01G5.5 -0.151679518151786
AGIWUR10711;AGIWUR30660
CB4856 CT III 1533774 1538198 WBGene00022054 Y67D2.4 Y67D2.4 -0.121248205758146
AGIWUR16840;AGIWUR18256;AGIWUR19412;AGIWUR33162;AGIWUR38801;AGIWUR39025
CB4856 CT III 1538472 1540244 WBGene00022055 Y67D2.5 Y67D2.5 -0.213984122370497
AGIWUR4238;AGIWUR4934;AGIWUR24164;AGIWUR41948
CB4856 CT III 1555395 1558529 WBGene00022057 Y67D2.7 Y67D2.7 0.0805476593869727 AGIWUR7019
CB4856 CT III 1558800 1570129 WBGene00022052 Y67D2.2 Y67D2.2 0.14781094011974 AGIWUR42933
CB4856 CT III 1570306 1576638 WBGene00022051 rep-1 Y67D2.1 -0.176988135494021
AGIWUR13469;AGIWUR21879
CB4856 CT III 1576751 1580857 WBGene00021246 Y22D7AL.7 Y22D7AL.7 -0.0995928604769958
AGIWUR30553
CB4856 CT III 1583155 1601140 WBGene00004894 sms-3 Y22D7AL.8 0.107235151970434
AGIWUR22781
CB4856 CT III 1604955 1606850 WBGene00021245 Y22D7AL.6 Y22D7AL.6 0.016531905298371
AGIWUR11587;AGIWUR13083
CB4856 CT III 1606829 1614033 WBGene00021247 Y22D7AL.9 Y22D7AL.9 0.248690230415753
AGIWUR10604;AGIWUR11815;AGIWUR13461;AGIWUR20754
CB4856 CT III 1617151 1618954 WBGene00021248 Y22D7AL.10 Y22D7AL.10 -0.443520668186854

AGIWUR3499;AGIWUR30303
CB4856	CT	III	1619742	1625797	WBGene00002025	hsp-60	Y22D7AL.5	-0.256856478821283
AGIWUR10628;AGIWUR19794;AGIWUR20849;AGIWUR20967;AGIWUR21651;AGIWUR26390;AGIWUR33280;AGIWUR34824;AGIWUR42555;AGIWUR43856
CB4856	CT	III	1626632	1637561	WBGene00021244	Y22D7AL.4	Y22D7AL.4	-0.0117439481695092
AGIWUR9679;AGIWUR24031
CB4856	CT	III	1641463	1641735	WBGene00021243	Y22D7AL.3	Y22D7AL.3	-0.159443686011503
AGIWUR6951
CB4856	CT	III	1642649	1644563	WBGene00021250	Y22D7AL.12	Y22D7AL.12	-0.220838972646666
AGIWUR44260
CB4856	CT	III	1648621	1648806	WBGene00021251	Y22D7AL.13	Y22D7AL.13	-0.130824244352084
AGIWUR43504
CB4856	CT	III	1650302	1652669	WBGene00021241	Y22D7AL.1	Y22D7AL.1	-0.125393700196448
AGIWUR26222
CB4856	CT	III	1653731	1657480	WBGene00021252	cht-4	Y22D7AL.14	0.216409233459411
AGIWUR39024
CB4856	CT	III	1658207	1659863	WBGene00021253	Y22D7AL.15	Y22D7AL.15	0.741749950723381
AGIWUR28095
CB4856	CT	III	1662104	1665152	WBGene00021254	Y22D7AL.16	Y22D7AL.16	-0.0570798912942161
AGIWUR14048;AGIWUR28872;AGIWUR29282;AGIWUR35626;AGIWUR42920;AGIWUR43018
CB4856	CT	III	1665493	1665793	WBGene00021257	Y22D7AR.3	Y22D7AR.3	-0.26124091653512
AGIWUR37407
CB4856	CT	III	1665913	1668634	WBGene00021259	glb-30	Y22D7AR.5	0.087737025714417
AGIWUR3197;AGIWUR7436;AGIWUR12048;AGIWUR14806;AGIWUR16613;AGIWUR17397;AGIWUR19490;AGIWUR21379;AGIWUR26038;AGIWUR36213;AGIWUR38898;AGIWUR40232;AGIWUR41393
CB4856	CT	III	1672517	1676721	WBGene00021260	Y22D7AR.6	Y22D7AR.6	0.0810208460769726
AGIWUR542;AGIWUR6397
CB4856	CT	III	1683630	1688099	WBGene00021261	Y22D7AR.7	Y22D7AR.7	0.181237999471951
AGIWUR43214
CB4856	CT	III	1688144	1691770	WBGene00021266	Y22D7AR.14	Y22D7AR.14	-0.313488414473197
AGIWUR43757
CB4856	CT	III	1692081	1695106	WBGene00005141	srd-64	Y22D7AR.8	0.020419611149733
AGIWUR1225
CB4856	CT	III	1697221	1698986	WBGene00021262	fbxa-74	Y22D7AR.9	-0.315325397951728
AGIWUR2493
CB4856	CT	III	1701714	1702437	WBGene00021263	Y22D7AR.10	Y22D7AR.10	-0.23039232632296
AGIWUR35366
CB4856	CT	III	1703500	1705899	WBGene00021264	fbxa-13	Y22D7AR.11	-0.254466526302055
AGIWUR33164

CB4856	CT	III	1705946	1713729	WBGene00021256	Y22D7AR.2	Y22D7AR.2	-0.191855160426591	AGIWUR21273
CB4856	CT	III	1716527	1722605	WBGene00021265	Y22D7AR.12	Y22D7AR.12	0.213460590393902	AGIWUR30137
CB4856	CT	III	1723194	1733101	WBGene00004779	ser-4	Y22D7AR.13	0.410340430808802	AGIWUR29875;AGIWUR39631;AGIWUR41915
CB4856	CT	III	1752183	1754942	WBGene00021593	Y46E12A.3	Y46E12A.3	0.333009273104539	AGIWUR10721
CB4856	CT	III	1758721	1759783	WBGene00021592	Y46E12A.2	Y46E12A.2	-0.0946154033069107	AGIWUR26043
CB4856	CT	III	1763524	1764836	WBGene00000560	cnc-6	Y46E12A.1	0.837665033132154	AGIWUR12088
CB4856	CT	III	1765458	1770091	WBGene00044798	tbx-43	Y46E12A.4	0.18850874425202	AGIWUR9296;AGIWUR9677
CB4856	CT	III	1772531	1778069	WBGene00021442	Y39A3CL.4	Y39A3CL.4	-0.0307346350878781	AGIWUR34744
CB4856	CT	III	1777802	1778309	WBGene00021441	Y39A3CL.3	Y39A3CL.3	-0.00871497950828015	AGIWUR43170
CB4856	CT	III	1789084	1806859	WBGene00006513	rimb-1	Y39A3CL.2	-0.227813795133658	AGIWUR16195
CB4856	CT	III	1807740	1818323	WBGene00000545	clp-4	Y39A3CL.5	0.191576307871915	AGIWUR8475;AGIWUR36088
CB4856	CT	III	1821150	1824180	WBGene00004249	pvf-1	Y39A3CL.6	0.128124948535147	AGIWUR2416
CB4856	CT	III	1824919	1834347	WBGene00021443	Y39A3CL.7	Y39A3CL.7	-0.327847459421504	AGIWUR20246;AGIWUR32428
CB4856	CT	III	1834513	1846758	WBGene00021440	Y39A3CL.1	Y39A3CL.1	-0.0702684016583408	AGIWUR36420
CB4856	CT	III	1849047	1852902	WBGene00004163	pqn-82	Y39A3CR.7	-0.124023416932518	AGIWUR18098
CB4856	CT	III	1855390	1858884	WBGene00021446	hlh-33	Y39A3CR.6	0.258728717328893	AGIWUR4579
CB4856	CT	III	1858913	1859569	WBGene00021445	Y39A3CR.5	Y39A3CR.5	0.115585532278796	AGIWUR25527
CB4856	CT	III	1859888	1860381	WBGene00000941	ddp-1	Y39A3CR.4	-0.113817659228586	AGIWUR11023;AGIWUR17766
CB4856	CT	III	1860429	1877514	WBGene00021444	ttc-7	Y39A3CR.3	0.137523103715582	AGIWUR13456
CB4856	CT	III	1878365	1880866	WBGene00004886	smi-1	Y39A3CR.1	-0.171374489758647	

AGIWUR12074;AGIWUR28726
CB4856	CT	III	1881158	1883017	WBGene00021447	Y39A3CR.8	Y39A3CR.8	-0.283242951100426	AGIWUR17516
CB4856	CT	III	1883607	1886218	WBGene00015243	B0524.4	B0524.4	0.196529613893007	AGIWUR13914
CB4856	CT	III	1887558	1889691	WBGene00015242	B0524.3	B0524.3	-0.0838781930793324	AGIWUR39402
CB4856	CT	III	1891383	1893842	WBGene00015244	B0524.5	B0524.5	0.145758606133474	AGIWUR13306
CB4856	CT	III	1894057	1899856	WBGene00015241	B0524.2	B0524.2	0.0146487785251974	AGIWUR35356
CB4856	CT	III	1902193	1905640	WBGene00015245	B0524.6	B0524.6	-0.450158204710821	AGIWUR23257
CB4856	CT	III	1905962	1939568	WBGene00004128	pqn-41	F53A3.4	0.0369718741353835	AGIWUR1642;AGIWUR6490;AGIWUR7208;AGIWUR8911;AGIWUR9570;AGIWUR11776;AGIWUR13585;AGIWUR18268;AGIWUR19636;AGIWUR19977;AGIWUR22432;AGIWUR27099;AGIWUR34458;AGIWUR40424
CB4856	CT	III	1939546	1943653	WBGene00018720	F53A3.1	F53A3.1	-0.066605443322107	AGIWUR44653
CB4856	CT	III	1945058	1950270	WBGene00018721	polh-1	F53A3.2	0.112706970635227	AGIWUR34536
CB4856	CT	III	1950492	1951426	WBGene00004491	rps-22	F53A3.3	-0.125770997932015	AGIWUR12230;AGIWUR28690;AGIWUR34975;AGIWUR38589;AGIWUR39036;AGIWUR41473;AGIWUR43706
CB4856	CT	III	1951526	1952032	WBGene00018723	F53A3.7	F53A3.7	0.119847287267325	AGIWUR7627
CB4856	CT	III	1952014	1961945	WBGene00020115	mboa-6	R155.1	-0.126387590946434	AGIWUR26427
CB4856	CT	III	1963566	1969902	WBGene00020116	moa-1	R155.2	0.0202472197823225	AGIWUR40050
CB4856	CT	III	1971182	1975709	WBGene00020117	R155.3	R155.3	-0.00630254968785771	AGIWUR28349
CB4856	CT	III	1977315	1980462	WBGene00020118	R155.4	R155.4	0.268252695181991	AGIWUR10910
CB4856	CT	III	1980823	1982647	WBGene00005143	srd-66	Y39A3B.4	0.0830350493121124	AGIWUR24540
CB4856	CT	III	1985109	1987466	WBGene00021438	Y39A3B.3	Y39A3B.3	-0.0525142152270179	AGIWUR29085
CB4856	CT	III	1997841	2002438	WBGene00021437	lgc-42	Y39A3B.2	0.0217637101718955	AGIWUR39318
CB4856	CT	III	2006029	2009196	WBGene00021436	Y39A3B.1	Y39A3B.1	0.113387326248748	AGIWUR42754
CB4856	CT	III	2009302	2021589	WBGene00021439	ckr-2	Y39A3B.5	0.224924429310689	AGIWUR1930;AGIWUR17361;AGIWUR30236;AGIWUR38253;AGIWUR44531
CB4856	CT	III	2035036	2048007	WBGene00001063	dpy-1	M01E10.2	0.497057130092969	AGIWUR23010
CB4856	CT	III	2053872	2054449	WBGene00019709	M01E10.3	M01E10.3	-0.618419644756337	AGIWUR23384
CB4856	CT	III	2056730	2057670	WBGene00021433	Y39A3A.2	Y39A3A.2	-0.259153938521694	AGIWUR20632
CB4856	CT	III	2060673	2060957	WBGene00002099	ins-16	Y39A3A.5	-0.0295352185422981	AGIWUR14112;AGIWUR31180;AGIWUR32785;AGIWUR34303;AGIWUR38456
CB4856	CT	III	2063442	2065855	WBGene00023471	srz-86	Y39A3A.7	0.0379707097127073

AGIWUR3884;AGIWUR32374
CB4856 CT III 2066640 2068350 WBGene00021434 Y39A3A.3 Y39A3A.3 0.165889980867066
AGIWUR13923
CB4856 CT III 2071513 2072347 WBGene00023470 srz-87 Y39A3A.6 0.0839707546093833
AGIWUR7389;AGIWUR23252
CB4856 CT III 2073009 2076520 WBGene00021435 Y39A3A.4 Y39A3A.4 0.109035391570101
AGIWUR36719
CB4856 CT III 2082819 2088248 WBGene00021703 cpt-3 Y48G9A.10 -0.00869110515684568
AGIWUR30430
CB4856 CT III 2092709 2099881 WBGene00021702 Y48G9A.9 Y48G9A.9 -0.0283476979434862
AGIWUR713
CB4856 CT III 2102295 2107497 WBGene00004088 ppk-2 Y48G9A.8 0.0882438519348803
AGIWUR28088
CB4856 CT III 2111802 2112427 WBGene00021701 Y48G9A.7 Y48G9A.7 0.0850297112251432
AGIWUR5690
CB4856 CT III 2112416 2116897 WBGene00021704 Y48G9A.11 Y48G9A.11 -0.0115349267184595
AGIWUR39784
CB4856 CT III 2117842 2119091 WBGene00021700 Y48G9A.6 Y48G9A.6 0.11663404806454
AGIWUR31978
CB4856 CT III 2124137 2151550 WBGene00021698 frl-1 Y48G9A.4 0.000357617704429103
AGIWUR33287
CB4856 CT III 2151968 2197908 WBGene00021697 gcn-1 Y48G9A.3 -0.456146707849782
AGIWUR2499;AGIWUR24890;AGIWUR39089
CB4856 CT III 2198006 2206882 WBGene00016524 C39B5.6 C39B5.6 -0.0648662331127561 AGIWUR32372
CB4856 CT III 2211420 2217095 WBGene00189950 C39B5.14 C39B5.14 1.40697943871104
AGIWUR9309;AGIWUR17915;AGIWUR33312
CB4856 CT III 2217173 2219494 WBGene00016526 fbxa-8 C39B5.8 0.376502463679369 AGIWUR33756
CB4856 CT III 2219797 2222365 WBGene00016525 fbxa-12 C39B5.7 -0.387389874495716 AGIWUR19075
CB4856 CT III 2222438 2224000 WBGene00005142 srd-65 C39B5.12 -0.0631631855787944
AGIWUR31417
CB4856 CT III 2224672 2227198 WBGene00016522 fbxa-38 C39B5.4 0.0130176648702793 AGIWUR24971
CB4856 CT III 2227707 2229228 WBGene00016521 fbxa-62 C39B5.3 -0.0868588987361321 AGIWUR15201
CB4856 CT III 2229992 2232460 WBGene00016527 fbxa-47 C39B5.9 -0.00597581506421196 AGIWUR41580
CB4856 CT III 2233888 2236987 WBGene00016520 C39B5.2 C39B5.2 0.533948293542064 AGIWUR33275
CB4856 CT III 2238967 2240172 WBGene00016528 C39B5.10 C39B5.10 -0.25228892760455
AGIWUR18177
CB4856 CT III 2240771 2243843 WBGene00016519 srz-56 C39B5.1 0.244259787071643 AGIWUR37544
CB4856 CT III 2244165 2245827 WBGene00005144 srd-67 C39B5.11 0.177855039082816
AGIWUR34479

CB4856	CT	III	2246016	2249404	WBGene00020642	T20H9.6	T20H9.6	0.146705086884818	AGIWUR25745
CB4856	CT	III	2249932	2251448	WBGene00020637	fbxa-7	T20H9.1	0.68469754420541	AGIWUR37127
CB4856	CT	III	2251957	2252989	WBGene00020641	fbxa-68	T20H9.5	1.00941614132621	AGIWUR3648
CB4856	CT	III	2253449	2254783	WBGene00020640	fbxa-73	T20H9.4	0.12043843731721	AGIWUR32193
CB4856	CT	III	2257554	2258941	WBGene00020638	fbxa-43	T20H9.2	-0.073577922438572	AGIWUR4357;AGIWUR25103;AGIWUR25937;AGIWUR41236;AGIWUR44639
CB4856	CT	III	2260004	2261445	WBGene00044676	fbxa-42	Y54F10BL.1	0.123099644228919	AGIWUR13364
CB4856	CT	III	2266980	2272175	WBGene00021862	fbxa-48	Y54F10BM.7	0.660647915577655	AGIWUR4973
CB4856	CT	III	2272453	2276965	WBGene00021859	fbxa-39	Y54F10BM.4	0.432077547039436	AGIWUR40392
CB4856	CT	III	2277279	2289967	WBGene00021858	Y54F10BM.3	Y54F10BM.3	-0.173275438802743	AGIWUR2267
CB4856	CT	III	2291419	2294124	WBGene00044403	fbxa-219	Y54F10BM.15	-0.0102830542451819	AGIWUR28098
CB4856	CT	III	2295344	2304920	WBGene00021863	Y54F10BM.9	Y54F10BM.9	-0.168088288472163	AGIWUR29475
CB4856	CT	III	2305942	2308008	WBGene00021864	fbxa-1	Y54F10BM.10	0.0362677486724099	AGIWUR24508
CB4856	CT	III	2308256	2309744	WBGene00206486	Y54F10BM.20	Y54F10BM.20	-0.102008829761578	AGIWUR32548
CB4856	CT	III	2309924	2314073	WBGene00021865	fbxa-66	Y54F10BM.11	0.49242749931515	AGIWUR7609
CB4856	CT	III	2314436	2326901	WBGene00021857	iffb-1	Y54F10BM.2	-0.266972874446303	AGIWUR5626;AGIWUR10411;AGIWUR23707
CB4856	CT	III	2326921	2329644	WBGene00021866	Y54F10BM.12	Y54F10BM.12	-0.0676377027080077	AGIWUR29344
CB4856	CT	III	2329577	2331183	WBGene00021867	Y54F10BM.13	Y54F10BM.13	-0.153525112052323	AGIWUR19905;AGIWUR36281
CB4856	CT	III	2331572	2342184	WBGene00021856	Y54F10BM.1	Y54F10BM.1	-0.234267150937381	AGIWUR20766
CB4856	CT	III	2343573	2347329	WBGene00004016	phf-5	Y54F10BM.14	-0.0835631183121315	AGIWUR869;AGIWUR3182;AGIWUR4589;AGIWUR5858;AGIWUR6693;AGIWUR10165;AGIWUR12970;AGIWUR22995;AGIWUR41822
CB4856	CT	III	2347591	2362056	WBGene00001589	gip-1	H04J21.3	-0.074749312638418	AGIWUR2869;AGIWUR12151;AGIWUR21514
CB4856	CT	III	2362948	2366278	WBGene00005146	srd-69	H04J21.2	0.0117654726920229	AGIWUR37224
CB4856	CT	III	2366884	2372655	WBGene00019150	H04J21.1	H04J21.1	0.181637920122878	

AGIWUR14406
CB4856 CT III 2376193 2384840 WBGene00019199 H14E04.2 H14E04.2 -0.107834177017127
AGIWUR2073;AGIWUR27203
CB4856 CT III 2384892 2386579 WBGene00019200 H14E04.3 H14E04.3 -0.366391116518717
AGIWUR20025
CB4856 CT III 2390203 2392116 WBGene00019201 H14E04.4 H14E04.4 -0.142938040912144
AGIWUR24047
CB4856 CT III 2394325 2401308 WBGene00000506 cic-1 H14E04.5 0.215479448033699
AGIWUR12520
CB4856 CT III 2402070 2406332 WBGene00019198 strm-1 H14E04.1 0.382525040358551
AGIWUR40650
CB4856 CT III 2406308 2410985 WBGene00021021 mrpl-30 W04B5.4 -0.302190871408119 AGIWUR43598
CB4856 CT III 2411090 2418205 WBGene00021022 W04B5.5 W04B5.5 -0.261291446422277 AGIWUR8262
CB4856 CT III 2418345 2423912 WBGene00021020 W04B5.3 W04B5.3 -0.146299790664086
AGIWUR42343;AGIWUR43483
CB4856 CT III 2424355 2427738 WBGene00021019 W04B5.2 W04B5.2 0.159769126893012 AGIWUR18604
CB4856 CT III 2436105 2442328 WBGene00017104 E02H9.6 E02H9.6 0.00343245668509518 AGIWUR23811
CB4856 CT III 2445228 2446142 WBGene00017105 E02H9.7 E02H9.7 -0.199486530141016
AGIWUR25516;AGIWUR35139;AGIWUR40542
CB4856 CT III 2446223 2448696 WBGene00017103 klo-2 E02H9.5 -0.299965823504461
AGIWUR3705;AGIWUR22017;AGIWUR43846
CB4856 CT III 2449382 2454949 WBGene00003711 nhr-121 E02H9.8 0.834750969953756 AGIWUR34209
CB4856 CT III 2461911 2465079 WBGene00017101 E02H9.3 E02H9.3 -0.183472357984456 AGIWUR12571
CB4856 CT III 2465474 2466800 WBGene00017100 E02H9.2 E02H9.2 0.252389378746218 AGIWUR12410
CB4856 CT III 2467347 2468302 WBGene00017106 E02H9.9 E02H9.9 -0.0875477568589931 AGIWUR3644
CB4856 CT III 2468486 2469443 WBGene00017099 E02H9.1 E02H9.1 -0.126548191120202
AGIWUR672;AGIWUR6778;AGIWUR8337;AGIWUR36905
CB4856 CT III 2473868 2486620 WBGene00004884 smg-6 Y54F10AL.2 -0.0602829432039833
AGIWUR11295;AGIWUR20339;AGIWUR22367;AGIWUR22837;AGIWUR26612;AGIWUR28035;AGIWUR28767
CB4856 CT III 2487769 2494340 WBGene00021847 Y54F10AL.1 Y54F10AL.1
-0.000716233607325201 AGIWUR905;AGIWUR13399;AGIWUR33083;AGIWUR33626;AGIWUR33848;AGIWUR42816
CB4856 CT III 2503448 2507972 WBGene00021852 Y54F10AM.8 Y54F10AM.8 -0.171700747749215
AGIWUR2335;AGIWUR25672;AGIWUR32210;AGIWUR35648;AGIWUR36425
CB4856 CT III 2508848 2511802 WBGene00021850 Y54F10AM.6 Y54F10AM.6 0.0724682561229204
AGIWUR27666;AGIWUR38314
CB4856 CT III 2512283 2528628 WBGene00004314 rbc-2 Y54F10AM.10 -0.261739511536099
AGIWUR29963;AGIWUR39097
CB4856 CT III 2529063 2530502 WBGene00021849 Y54F10AM.5 Y54F10AM.5 0.0383924111844287
AGIWUR19611

CB4856	CT	III	2530615	2546420	WBGene00000464	ceh-44	Y54F10AM.4	-0.253264528109339	AGIWUR4705;AGIWUR16518;AGIWUR24433
CB4856	CT	III	2549927	2560684	WBGene00001410	feh-1	Y54F10AM.2	0.0678617121708225	AGIWUR4773
CB4856	CT	III	2561710	2566589	WBGene00021848	nhr-239	Y54F10AM.1	0.0291742355557072	AGIWUR14239;AGIWUR19746;AGIWUR25060;AGIWUR30065
CB4856	CT	III	2567404	2571547	WBGene00021855	Y54F10AR.2	Y54F10AR.2	0.162447174365049	AGIWUR40408
CB4856	CT	III	2572031	2575723	WBGene00021854	Y54F10AR.1	Y54F10AR.1	-0.169508689895865	AGIWUR16208
CB4856	CT	III	2579314	2580664	WBGene00022197	Y71H2B.8	Y71H2B.8	0.18880955320597	AGIWUR12764
CB4856	CT	III	2583953	2597034	WBGene00000160	apb-1	Y71H2B.10	-0.0443794882620523	AGIWUR28860;AGIWUR38318
CB4856	CT	III	2598057	2603299	WBGene00007019	mdt-19	Y71H2B.6	0.00331150655346235	AGIWUR9849
CB4856	CT	III	2604236	2606541	WBGene00022198	Y71H2B.11	Y71H2B.11	-0.0431884887290854	AGIWUR3768;AGIWUR39503
CB4856	CT	III	2608017	2618856	WBGene00022195	Y71H2B.5	Y71H2B.5	-0.0457379082679434	AGIWUR16225
CB4856	CT	III	2618963	2619375	WBGene00022194	Y71H2B.4	Y71H2B.4	-0.29017870463432	AGIWUR6650
CB4856	CT	III	2619936	2622709	WBGene00022193	ppfr-4	Y71H2B.3	0.27772264111195	AGIWUR44844
CB4856	CT	III	2623170	2627771	WBGene00022192	Y71H2B.2	Y71H2B.2	0.100897240718131	AGIWUR42251
CB4856	CT	III	2629013	2631439	WBGene00022191	Y71H2B.1	Y71H2B.1	-0.0972609975293874	AGIWUR20164;AGIWUR34065;AGIWUR41211
CB4856	CT	III	2636405	2642848	WBGene00019212	zmp-2	H19M22.3	0.21748125386429	AGIWUR10196;AGIWUR17797;AGIWUR22540;AGIWUR22981;AGIWUR30381;AGIWUR30911;AGIWUR35936;AGIWUR39133;AGIWUR42071
CB4856	CT	III	2650851	2651565	WBGene00023084	H19M22.4	H19M22.4	-0.0556106397564218	AGIWUR7023;AGIWUR25650;AGIWUR36609;AGIWUR42415;AGIWUR43101
CB4856	CT	III	2658083	2681112	WBGene00002915	let-805	H19M22.2	-0.0724498167526741	AGIWUR2534;AGIWUR15885;AGIWUR16395;AGIWUR16752;AGIWUR18969;AGIWUR21421;AGIWUR25034;AGIWUR30471;AGIWUR34160;AGIWUR35690;AGIWUR40260;AGIWUR42896
CB4856	CT	III	2681451	2686463	WBGene00003941	pbo-1	Y71H2AL.1	-0.0594937227627472	AGIWUR16024
CB4856	CT	III	2686791	2688997	WBGene00022165	Y71H2AL.2	Y71H2AL.2	0.117852329498094	AGIWUR36616

CB4856 CT III 2690160 2698817 WBGene00022174 Y71H2AM.9 Y71H2AM.9 -0.3057641610215
AGIWUR44591
CB4856 CT III 2700937 2707818 WBGene00022173 set-27 Y71H2AM.8 -0.552640521004481
AGIWUR19608
CB4856 CT III 2707737 2716447 WBGene00022175 Y71H2AM.10 Y71H2AM.10 -0.161232727590056
AGIWUR31620
CB4856 CT III 2718158 2725092 WBGene00022176 Y71H2AM.11 Y71H2AM.11 0.0084655156565514
AGIWUR34264
CB4856 CT III 2734080 2739531 WBGene00022178 Y71H2AM.13 Y71H2AM.13 -0.186623464775037
AGIWUR29754
CB4856 CT III 2743719 2746653 WBGene00022179 Y71H2AM.14 Y71H2AM.14 0.237501624636012
AGIWUR1759
CB4856 CT III 2747130 2756512 WBGene00022180 Y71H2AM.15 Y71H2AM.15 0.521090154983805
AGIWUR14045
CB4856 CT III 2757087 2760816 WBGene00022181 pho-9 Y71H2AM.16 0.182225247444292
AGIWUR1582;AGIWUR7980;AGIWUR12398;AGIWUR14707;AGIWUR22714
CB4856 CT III 2764037 2768675 WBGene00022182 swsn-3 Y71H2AM.17 -0.203137953617545
AGIWUR22386;AGIWUR25232
CB4856 CT III 2769358 2779053 WBGene00002244 laf-1 Y71H2AM.19 -0.161091695065299
AGIWUR3116;AGIWUR3798;AGIWUR9004;AGIWUR23603;AGIWUR24974;AGIWUR25228;AGIWUR30455
CB4856 CT III 2781039 2782817 WBGene00022171 Y71H2AM.6 Y71H2AM.6
-0.000916145160324788 AGIWUR24357
CB4856 CT III 2782733 2785993 WBGene00022185 Y71H2AM.20 Y71H2AM.20 0.254672280650635
AGIWUR33509;AGIWUR38426
CB4856 CT III 2786117 2786859 WBGene00022170 Y71H2AM.5 Y71H2AM.5 -0.0853562992825481
AGIWUR2693;AGIWUR6440;AGIWUR17716;AGIWUR32055;AGIWUR36884;AGIWUR38719;AGIWUR41121
CB4856 CT III 2786854 2788028 WBGene00022169 Y71H2AM.4 Y71H2AM.4 0.0900727213940273
AGIWUR36947;AGIWUR43443
CB4856 CT III 2788441 2792973 WBGene00044609 tbcc-1 Y71H2AM.24 -0.135211440885456
AGIWUR23229;AGIWUR33566
CB4856 CT III 2793323 2801900 WBGene00006695 twk-45 Y71H2AM.22 -0.0173246535306681
AGIWUR3799;AGIWUR4712;AGIWUR14874;AGIWUR21233;AGIWUR27245;AGIWUR35937
CB4856 CT III 2802450 2803681 WBGene00044760 Y71H2AM.25 Y71H2AM.25 -0.0366633673744117
AGIWUR36266
CB4856 CT III 2805430 2811096 WBGene00022168 Y71H2AM.3 Y71H2AM.3 -0.217906658143534
AGIWUR192
CB4856 CT III 2811870 2814969 WBGene00007000 tufm-1 Y71H2AM.23 -0.0201854933100876
AGIWUR44719
CB4856 CT III 2819280 2826153 WBGene00022167 Y71H2AM.2 Y71H2AM.2 -0.412390994377107

AGIWUR9342
CB4856 CT III 2827070 2833824 WBGene00022166 Y71H2AM.1 Y71H2AM.1 -0.0595857904467101
AGIWUR25268;AGIWUR25695
CB4856 CT III 2834663 2836261 WBGene00022188 Y71H2AR.1 Y71H2AR.1 0.0321592393667309
AGIWUR8952
CB4856 CT III 2836639 2838453 WBGene00022190 josd-1 Y71H2AR.3 0.0305003366942258
AGIWUR10191;AGIWUR14524;AGIWUR20442;AGIWUR42988
CB4856 CT III 2838763 2840927 WBGene00022189 Y71H2AR.2 Y71H2AR.2 -0.106510007488839
AGIWUR36642
CB4856 CT III 2842044 2845197 WBGene00019261 H34I24.2 H34I24.2 -0.0749830466169037
AGIWUR22219
CB4856 CT III 2847016 2848582 WBGene00019260 H34I24.1 H34I24.1 0.0466556236542015
AGIWUR16301
CB4856 CT III 2849066 2855419 WBGene00018992 trpl-1 F56F11.5 0.264353019083474
AGIWUR13328;AGIWUR37474;AGIWUR40427
CB4856 CT III 2855554 2865497 WBGene00018991 F56F11.4 F56F11.4 -0.480226520512279
AGIWUR5816
CB4856 CT III 2874818 2880705 WBGene00018990 klf-1 F56F11.3 0.092759854235962
AGIWUR37613
CB4856 CT III 2882304 2884967 WBGene00018989 F56F11.2 F56F11.2 -0.451285377348626
AGIWUR6833;AGIWUR13653;AGIWUR40840
CB4856 CT III 2891762 2892613 WBGene00018988 F56F11.1 F56F11.1 -0.371125010109775
AGIWUR13941
CB4856 CT III 2893433 2897183 WBGene00020598 T20B6.2 T20B6.2 -0.329266419190534 AGIWUR17017
CB4856 CT III 2897711 2898802 WBGene00020597 T20B6.1 T20B6.1 0.116214071191877 AGIWUR19617
CB4856 CT III 2899441 2900402 WBGene00020599 T20B6.3 T20B6.3 -0.223561433058428 AGIWUR28148
CB4856 CT III 2910910 2935740 WBGene00019994 cdh-1 R10F2.1 0.113961304392181 AGIWUR5450
CB4856 CT III 2937103 2944044 WBGene00019997 R10F2.6 R10F2.6 0.120147246711027 AGIWUR7457
CB4856 CT III 2947091 2960574 WBGene00019157 H05C05.1 H05C05.1 -0.297490236512088
AGIWUR1750;AGIWUR25924
CB4856 CT III 2962170 2965634 WBGene00019158 H05C05.2 H05C05.2 0.024391913996042
AGIWUR33855;AGIWUR33898
CB4856 CT III 2970677 2975785 WBGene00021917 Y55D5A.3 Y55D5A.3 -0.127497035373796
AGIWUR1723
CB4856 CT III 2982414 2988150 WBGene00021915 Y55D5A.1 Y55D5A.1 0.688874153328412
AGIWUR10708;AGIWUR39069
CB4856 CT III 2988081 2990057 WBGene00021918 Y55D5A.4 Y55D5A.4
3.98584217693454e-05 AGIWUR22699;AGIWUR34129
CB4856 CT III 2995487 3040860 WBGene00000898 daf-2 Y55D5A.5 0.250493615749537

AGIWUR7133;AGIWUR13223;AGIWUR19374;AGIWUR30665;AGIWUR30985;AGIWUR34221;AGIWUR38552
CB4856 CT III 3042726 3047125 WBGene00021919 cutl-25 Y55D5A.6 0.306201047499117
AGIWUR4266
CB4856 CT III 3052916 3057550 WBGene00019169 H06I04.5 H06I04.5 -0.0327907776378753
AGIWUR17811
CB4856 CT III 3059598 3059943 WBGene00019171 fipr-29 H06I04.7 0.349594786260597
AGIWUR3286
CB4856 CT III 3061192 3064328 WBGene00019170 H06I04.6 H06I04.6 0.225177244025776
AGIWUR17697;AGIWUR28831
CB4856 CT III 3068306 3069210 WBGene00006725 ubl-1 H06I04.4 0.105333077287174
AGIWUR40097
CB4856 CT III 3070523 3085268 WBGene00019168 H06I04.3 H06I04.3 -0.121078236850699
AGIWUR19571;AGIWUR26916;AGIWUR43041;AGIWUR43553
CB4856 CT III 3086693 3091738 WBGene00004787 sft-1 H06I04.2 -0.191418679305091
AGIWUR34593
CB4856 CT III 3091850 3100468 WBGene00019167 H06I04.1 H06I04.1 0.120923990316387
AGIWUR8697;AGIWUR11825;AGIWUR19377;AGIWUR24950;AGIWUR25370;AGIWUR27115;AGIWUR30206;AGIWUR37893
CB4856 CT III 3101267 3103955 WBGene00021818 Y53G8B.2 Y53G8B.2 0.0165589490418974
AGIWUR23098
CB4856 CT III 3105668 3111828 WBGene00021819 Y53G8B.3 Y53G8B.3 0.00787324811215884
AGIWUR20240;AGIWUR39483
CB4856 CT III 3112428 3127124 WBGene00021820 nipa-1 Y53G8B.4 0.0107899348121245
AGIWUR13380;AGIWUR15191;AGIWUR20673;AGIWUR22382;AGIWUR27257;AGIWUR28555
CB4856 CT III 3129277 3131399 WBGene00021817 Y53G8B.1 Y53G8B.1 0.0172065940575372
AGIWUR8460
CB4856 CT III 3131574 3139234 WBGene00003175 mec-12 C44B11.3 0.0618429278147445
AGIWUR9745
CB4856 CT III 3142198 3143757 WBGene00016634 C44B11.4 C44B11.4 0.209693153596845
AGIWUR35263
CB4856 CT III 3152722 3153471 WBGene00016635 C44B11.6 C44B11.6 0.0503804318599883
AGIWUR35468
CB4856 CT III 3155268 3158393 WBGene00016633 C44B11.1 C44B11.1 -0.379955943167092
AGIWUR38618
CB4856 CT III 3166285 3171198 WBGene00020104 R148.5 R148.5 0.477193490014084
AGIWUR3695;AGIWUR8574;AGIWUR10518;AGIWUR10971;AGIWUR23515;AGIWUR35283;AGIWUR40568
CB4856 CT III 3171592 3174845 WBGene00020103 R148.4 R148.4 -0.114527007654727
AGIWUR2853;AGIWUR12459;AGIWUR13273;AGIWUR20228;AGIWUR20610;AGIWUR30751;AGIWUR38883
CB4856 CT III 3175339 3183498 WBGene00020102 R148.3 R148.3 -0.112772161748353
AGIWUR12884;AGIWUR22527

CB4856	CT	III	3183677	3185237	WBGene00020101	lmtr-5	R148.2	-0.272853361936603	AGIWUR96;AGIWUR32957
CB4856	CT	III	3186700	3192206	WBGene00020100	mks-1	R148.1	0.182980049496274	AGIWUR15590;AGIWUR23354
CB4856	CT	III	3195561	3198407	WBGene00020105	R148.7	R148.7	0.22570995935456	AGIWUR42091
CB4856	CT	III	3198477	3199516	WBGene00006452	heh-1	R148.6	-0.16591647131373	AGIWUR16994
CB4856	CT	III	3200795	3219150	WBGene00001494	frm-8	H09G03.2	0.0153730978949316	AGIWUR6857
CB4856	CT	III	3226352	3230652	WBGene00019174	H09G03.1	H09G03.1	-0.0610131206356654	AGIWUR20902;AGIWUR38539
CB4856	CT	III	3233102	3241263	WBGene00021799	Y53G8AL.1	Y53G8AL.1	0.0661763790266852	AGIWUR3746;AGIWUR9132;AGIWUR27505
CB4856	CT	III	3241717	3248575	WBGene00021800	Y53G8AL.2	Y53G8AL.2	-0.106279824241081	AGIWUR11969
CB4856	CT	III	3249346	3250538	WBGene00050917	Y53G8AL.4	Y53G8AL.4	-0.109338349791056	AGIWUR98;AGIWUR2795;AGIWUR8581;AGIWUR11247;AGIWUR12880;AGIWUR27907;AGIWUR29640;AGIWUR31983;AGIWUR32616
CB4856	CT	III	3251461	3253143	WBGene00021801	Y53G8AL.3	Y53G8AL.3	0.113191049536578	AGIWUR3567
CB4856	CT	III	3253969	3255619	WBGene00021804	Y53G8AM.4	Y53G8AM.4	0.0747939028824574	AGIWUR11618;AGIWUR14433;AGIWUR15534
CB4856	CT	III	3256730	3258395	WBGene00021805	Y53G8AM.5	Y53G8AM.5	-0.198371410712506	AGIWUR6953
CB4856	CT	III	3259331	3260882	WBGene00021806	Y53G8AM.6	Y53G8AM.6	0.135932814185802	AGIWUR37373
CB4856	CT	III	3263682	3266608	WBGene00021807	Y53G8AM.7	Y53G8AM.7	0.278929806005808	AGIWUR31082
CB4856	CT	III	3267178	3278710	WBGene00021808	Y53G8AM.8	Y53G8AM.8	0.197356372286436	AGIWUR2613;AGIWUR10843
CB4856	CT	III	3282308	3290215	WBGene00021814	Y53G8AR.7	Y53G8AR.7	-0.427924083149289	AGIWUR17390;AGIWUR29595;AGIWUR30914
CB4856	CT	III	3290117	3294330	WBGene00021815	Y53G8AR.8	Y53G8AR.8	-0.298355713370409	AGIWUR37995
CB4856	CT	III	3294743	3297115	WBGene00021813	Y53G8AR.6	Y53G8AR.6	-0.189845254614664	AGIWUR4561;AGIWUR24284;AGIWUR24788;AGIWUR38114
CB4856	CT	III	3297454	3302151	WBGene00021812	Y53G8AR.5	Y53G8AR.5	-0.0303082244246191	AGIWUR41086
CB4856	CT	III	3311423	3317713	WBGene00021811	ral-1	Y53G8AR.3	-0.341546438499714	AGIWUR39100
CB4856	CT	III	3317737	3321245	WBGene00021816	Y53G8AR.9	Y53G8AR.9	-0.167827848191251	

AGIWUR331
CB4856	CT	III	3322962 3331727 WBGene00021810	phf-15	Y53G8AR.2	0.0325776110594624
AGIWUR35836
CB4856	CT	III	3333036 3333957 WBGene00021809	Y53G8AR.1	Y53G8AR.1	-0.29829449240545
AGIWUR12431;AGIWUR18982;AGIWUR22098
CB4856	CT	III	3344875 3363409 WBGene00001559	gei-1	F45H7.2 0.201333040098491
AGIWUR6128;AGIWUR42668
CB4856	CT	III	3363668 3370209 WBGene00004183	prk-2	F45H7.4 0.102256577223908	AGIWUR39213
CB4856	CT	III	3377408 3389970 WBGene00009738	hecw-1	F45H7.6 0.0318605341570266
AGIWUR2775;AGIWUR13999
CB4856	CT	III	3389894 3392525 WBGene00008348	C56G7.3 C56G7.3 0.122623378607411	AGIWUR39672
CB4856	CT	III	3392514 3393787 WBGene00003372	mlc-4	C56G7.1 0.225651178269349
AGIWUR30999;AGIWUR42250;AGIWUR42527
CB4856	CT	III	3396585 3397675 WBGene00010303	cri-3	F59A2.3 -0.277861375914956
AGIWUR13117;AGIWUR25564;AGIWUR26708;AGIWUR37141;AGIWUR43589
CB4856	CT	III	3397743 3400644 WBGene00010304	clpf-1	F59A2.4 -0.157836726244156	AGIWUR17293
CB4856	CT	III	3400644 3404479 WBGene00003795	npp-9	F59A2.1 -0.103685182458459
AGIWUR8688;AGIWUR12216;AGIWUR18359;AGIWUR29274;AGIWUR32884;AGIWUR34620;AGIWUR35151
CB4856	CT	III	3404863 3405469 WBGene00010305	F59A2.5 F59A2.5 -0.15514407192456	AGIWUR18938
CB4856	CT	III	3405576 3411624 WBGene00010302	F59A2.2 F59A2.2 0.0106000068502246	AGIWUR22160
CB4856	CT	III	3415552 3425447 WBGene00010306	golg-4	F59A2.6 -0.22737677971605	AGIWUR43196
CB4856	CT	III	3425736 3428094 WBGene00010450	K01A11.2	K01A11.2
-0.00644969391999162	AGIWUR41248
CB4856	CT	III	3428080 3431366 WBGene00010449	K01A11.1	K01A11.1	-0.441024621749118
AGIWUR37728
CB4856	CT	III	3439023 3444207 WBGene00004976	spe-41	K01A11.4	0.123136572750539
AGIWUR42753
CB4856	CT	III	3447140 3452613 WBGene00010451	K01A11.3	K01A11.3	-0.243196715584444
AGIWUR12199
CB4856	CT	III	3454345 3455850 WBGene00007920	C34C12.1	C34C12.1	0.104050610908299
AGIWUR8950
CB4856	CT	III	3456435 3460106 WBGene00007921	C34C12.2	C34C12.2
-0.00429668951898776	AGIWUR33347
CB4856	CT	III	3460406 3461590 WBGene00007927	C34C12.8	C34C12.8	-0.15486814899354
AGIWUR23839
CB4856	CT	III	3461703 3467687 WBGene00007922	pph-6	C34C12.3	-0.035833747654042
AGIWUR15982
CB4856	CT	III	3467664 3469827 WBGene00007923	C34C12.4	C34C12.4	-0.400325108539085
AGIWUR4591;AGIWUR11505;AGIWUR14624;AGIWUR27106;AGIWUR34870

CB4856 CT III 3472003 3473718 WBGene00007924 rsu-1 C34C12.5 -0.212501138763576 AGIWUR68;AGIWUR4021;AGIWUR7201;AGIWUR8236;AGIWUR24702;AGIWUR27827;AGIWUR32997;AGIWUR37412;AGIWUR44729
CB4856 CT III 3474019 3475450 WBGene00007928 C34C12.9 C34C12.9 0.455099367806769 AGIWUR12359
CB4856 CT III 3482591 3484843 WBGene00007925 C34C12.6 C34C12.6 0.132798373038506 AGIWUR31348
CB4856 CT III 3484813 3488341 WBGene00007926 C34C12.7 C34C12.7 0.456064344138261 AGIWUR32822
CB4856 CT III 3490363 3493234 WBGene00001639 gly-14 M01F1.1 -0.0393222694479745 AGIWUR22529
CB4856 CT III 3493811 3499057 WBGene00010815 M01F1.9 M01F1.9 -0.123818104311285 AGIWUR15859;AGIWUR37865;AGIWUR43079
CB4856 CT III 3499375 3502547 WBGene00010814 M01F1.8 M01F1.8 -0.310416077916948 AGIWUR4692
CB4856 CT III 3502841 3503724 WBGene00004428 rpl-16 M01F1.2 -0.0721067240409017 AGIWUR5475;AGIWUR9044;AGIWUR10626;AGIWUR28475;AGIWUR28655;AGIWUR34090
CB4856 CT III 3503710 3505355 WBGene00010809 M01F1.3 M01F1.3 0.275936145746938 AGIWUR14202
CB4856 CT III 3505649 3510033 WBGene00010810 M01F1.4 M01F1.4 0.243520642920361 AGIWUR40566
CB4856 CT III 3510237 3511011 WBGene00010812 mrpl-35 M01F1.6 -0.120431674776139 AGIWUR6204
CB4856 CT III 3510964 3519607 WBGene00010811 hmit-1.3 M01F1.5 0.505274035127115 AGIWUR26004
CB4856 CT III 3521133 3529838 WBGene00010813 pitp-1 M01F1.7 0.0383369060518817 AGIWUR42568
CB4856 CT III 3534660 3537496 WBGene00008288 C54C6.6 C54C6.6 -0.0764382748511547 AGIWUR25423
CB4856 CT III 3537688 3541628 WBGene00000248 ben-1 C54C6.2 -0.13346929348123 AGIWUR19529
CB4856 CT III 3545740 3546419 WBGene00044166 C54C6.7 C54C6.7 0.523493593992102 AGIWUR16866
CB4856 CT III 3549410 3549850 WBGene00008287 C54C6.5 C54C6.5 -0.0932089392151791 AGIWUR3248;AGIWUR7930;AGIWUR11537;AGIWUR12523;AGIWUR12823;AGIWUR23202
CB4856 CT III 3553649 3556627 WBGene00008286 C54C6.4 C54C6.4 -0.10043563067922 AGIWUR2455;AGIWUR20439;AGIWUR35219
CB4856 CT III 3573585 3578771 WBGene00003605 nhr-6 C48D5.1 0.467538214024033 AGIWUR30336
CB4856 CT III 3578716 3581736 WBGene00008180 C48D5.3 C48D5.3 -0.0982129999065711 AGIWUR23414
CB4856 CT III 3590734 3611993 WBGene00004213 ptp-1 C48D5.2 0.236907730299451 AGIWUR6891;AGIWUR25035;AGIWUR35090
CB4856 CT III 3612951 3627142 WBGene00004765 sel-8 C32A3.1 -0.552267599509204 AGIWUR25533
CB4856 CT III 3627986 3630368 WBGene00007859 mrps-31 C32A3.2 -0.533891378481854 AGIWUR2977
CB4856 CT III 3635876 3643670 WBGene00007860 rilp-1 C32A3.3 0.0985566852805597 AGIWUR12909
CB4856 CT III 3644375 3648822 WBGene00006822 unc-93 C46F11.1 -0.368391511535481 AGIWUR19227
CB4856 CT III 3648792 3650832 WBGene00008118 madf-8 C46F11.3 -0.0614240934972266 AGIWUR4896
CB4856 CT III 3650905 3652973 WBGene00008117 gsr-1 C46F11.2 -0.0413709508606424

AGIWUR3922;AGIWUR4457;AGIWUR11413;AGIWUR16538;AGIWUR21036;AGIWUR25473;AGIWUR30459
CB4856 CT III 3652866 3657602 WBGene00008120 C46F11.5 C46F11.5 -0.144113744993801
AGIWUR2103;AGIWUR17982;AGIWUR19818
CB4856 CT III 3657716 3664494 WBGene00008119 C46F11.4 C46F11.4 0.418996406829848
AGIWUR9635
CB4856 CT III 3667022 3670436 WBGene00008121 C46F11.6 C46F11.6 0.106893532006399
AGIWUR5194
CB4856 CT III 3683253 3685808 WBGene00012084 npr-15 T27D1.3 0.222320701104628 AGIWUR45091
CB4856 CT III 3697821 3699640 WBGene00000885 cyn-9 T27D1.1 -0.0351776973209343
AGIWUR5691;AGIWUR22866
CB4856 CT III 3699740 3701476 WBGene00003962 pdi-1 C14B1.1 -0.0321821924439583 AGIWUR26524
CB4856 CT III 3702260 3703172 WBGene00007573 C14B1.2 C14B1.2 -0.00147796585911308 AGIWUR35229
CB4856 CT III 3703564 3705046 WBGene00007574 C14B1.3 C14B1.3 0.176164005998903 AGIWUR36098
CB4856 CT III 3705265 3706784 WBGene00006474 wdr-5.1 C14B1.4 0.191318470598032 AGIWUR43195
CB4856 CT III 3706886 3709225 WBGene00007576 dph-1 C14B1.5 0.204501436993318 AGIWUR42170
CB4856 CT III 3709330 3713282 WBGene00007581 alkb-8 C14B1.10 -0.105468286309739
AGIWUR43295
CB4856 CT III 3713463 3718745 WBGene00007577 nrde-1 C14B1.6 0.0336390018266535 AGIWUR30630
CB4856 CT III 3718655 3719722 WBGene00007579 C14B1.8 C14B1.8 -0.242507461787583
AGIWUR1479;AGIWUR19965;AGIWUR23671;AGIWUR36535
CB4856 CT III 3719904 3723597 WBGene00007578 C14B1.7 C14B1.7 1.77635683940025e-15
AGIWUR3899;AGIWUR7683;AGIWUR11406;AGIWUR14763;AGIWUR18063;AGIWUR23634;AGIWUR27561
CB4856 CT III 3724222 3724980 WBGene00184990 C14B1.12 C14B1.12 -0.0646010862545747
AGIWUR40467
CB4856 CT III 3726683 3729570 WBGene00045484 F34D10.9 F34D10.9 0.108626272966394
AGIWUR26415
CB4856 CT III 3729493 3735039 WBGene00009372 evl-18 F34D10.2 -0.0425616251996441
AGIWUR31255;AGIWUR41733
CB4856 CT III 3736602 3739765 WBGene00009374 F34D10.4 F34D10.4 -0.0389955107774931
AGIWUR6706
CB4856 CT III 3745461 3747451 WBGene00003033 lin-48 F34D10.5 0.138030220613489
AGIWUR8297;AGIWUR33973
CB4856 CT III 3747560 3759412 WBGene00009375 F34D10.6 F34D10.6 -0.0767906827263012
AGIWUR24321;AGIWUR27132
CB4856 CT III 3760378 3765556 WBGene00008091 C44F1.1 C44F1.1 0.0164179239371851 AGIWUR34530
CB4856 CT III 3768500 3770321 WBGene00008092 gmeb-3 C44F1.2 -0.109766952705904
AGIWUR17407;AGIWUR28024;AGIWUR36161
CB4856 CT III 3771425 3773394 WBGene00002267 lec-4 C44F1.3 0.0940151461115146 AGIWUR34709
CB4856 CT III 3774896 3786640 WBGene00000070 acy-3 C44F1.5 0.0464898104498204 AGIWUR26920

CB4856	CT	III	3789982	3800547	WBGene00003476	mtm-3	T24A11.1	0.0719335871575746	AGIWUR9591;AGIWUR13492
CB4856	CT	III	3802452	3804438	WBGene00011974	xbx-5	T24A11.2	0.130572568218177	AGIWUR16614;AGIWUR30028
CB4856	CT	III	3804772	3809030	WBGene00006591	toh-1	T24A11.3	0.0159086069627499	AGIWUR2545;AGIWUR10073;AGIWUR17610;AGIWUR19116;AGIWUR29103;AGIWUR29370;AGIWUR36519
CB4856	CT	III	3813519	3819679	WBGene00001086	dpy-27	R13G10.1	0.14659389470514	AGIWUR14664
CB4856	CT	III	3822403	3826261	WBGene00000137	amx-1	R13G10.2	0.116272477051865	AGIWUR18369
CB4856	CT	III	3826378	3829205	WBGene00011260	cnnm-5	R13G10.4	-0.34460914674502	AGIWUR24264
CB4856	CT	III	3832591	3834522	WBGene00007963	cyp-25A1	C36A4.1	-0.0764435765317772	AGIWUR27481;AGIWUR44867
CB4856	CT	III	3835221	3837211	WBGene00007964	cyp-25A2	C36A4.2	0.02514027707638	AGIWUR18068
CB4856	CT	III	3837551	3839477	WBGene00014697	cyp-25A3	C36A4.3	0.407682302163542	AGIWUR33808
CB4856	CT	III	3841262	3843614	WBGene00007965	C36A4.4	C36A4.4	-0.0482415499281679	AGIWUR21773
CB4856	CT	III	3853026	3862958	WBGene00000264	brc-1	C36A4.8	-0.27400821530086	AGIWUR18213
CB4856	CT	III	3864036	3867098	WBGene00007969	acs-19	C36A4.9	0.471512422626265	AGIWUR5743;AGIWUR8131;AGIWUR18162;AGIWUR24888;AGIWUR42768
CB4856	CT	III	3867852	3869587	WBGene00004414	rpl-3	F13B10.2	-0.0197683874176606	AGIWUR4750;AGIWUR29310
CB4856	CT	III	3869591	3901466	WBGene00006575	tir-1	F13B10.1	0.613742815436205	AGIWUR2597;AGIWUR3084;AGIWUR3265;AGIWUR5178;AGIWUR9784;AGIWUR15222;AGIWUR19101;AGIWUR21064;AGIWUR30859;AGIWUR30875;AGIWUR34308;AGIWUR38888;AGIWUR39275;AGIWUR40787
CB4856	CT	III	3903114	3907687	WBGene00014202	mmcm-1	ZK1058.1	0.10802546188165	AGIWUR1795;AGIWUR2675;AGIWUR8381;AGIWUR12443;AGIWUR14231;AGIWUR16597;AGIWUR17881;AGIWUR33357;AGIWUR39146;AGIWUR41856
CB4856	CT	III	3908929	3914149	WBGene00003930	pat-3	ZK1058.2	0.228891754965687	AGIWUR35961
CB4856	CT	III	3916933	3918809	WBGene00014204	ccdc-47	ZK1058.4	0.0244848961141493	AGIWUR44755
CB4856	CT	III	3918871	3920700	WBGene00014203	ZK1058.3	ZK1058.3	-0.0193846311899453	AGIWUR18587
CB4856	CT	III	3920626	3921711	WBGene00014205	ZK1058.5	ZK1058.5	-0.0649149307204322	AGIWUR39458
CB4856	CT	III	3922207	3923869	WBGene00014206	nit-1	ZK1058.6	0.146015092741983	

AGIWUR42259
CB4856 CT III 3923792 3926238 WBGene00014207 ZK1058.9 ZK1058.9 0.20289311243447
AGIWUR27933
CB4856 CT III 3929530 3931523 WBGene00009502 F37A8.2 F37A8.2 -0.0315393191272335 AGIWUR14427
CB4856 CT III 3933577 3935908 WBGene00003748 nlp-10 F37A8.4 0.13084284714586 AGIWUR40346
CB4856 CT III 3956624 3958161 WBGene00009503 F37A8.5 F37A8.5 1.21893993371745 AGIWUR6158
CB4856 CT III 3960258 3961825 WBGene00011215 R10E9.3 R10E9.3 0.191926681523745 AGIWUR21427
CB4856 CT III 3962731 3964158 WBGene00011214 R10E9.2 R10E9.2 0.0230300292883676 AGIWUR23439
CB4856 CT III 3964663 3976391 WBGene00003423 msi-1 R10E9.1 0.235215116174531
AGIWUR32590;AGIWUR33464
CB4856 CT III 3984367 3984670 WBGene00012380 Y1A5A.2 Y1A5A.2 0.0576969901335449 AGIWUR12504
CB4856 CT III 3985565 3986809 WBGene00012379 Y1A5A.1 Y1A5A.1 -0.0439709276840983
AGIWUR35450;AGIWUR37937
CB4856 CT III 3988059 3988575 WBGene00044230 C36E8.6 C36E8.6 -0.126793927059177 AGIWUR30001
CB4856 CT III 3989298 3992174 WBGene00007981 glb-11 C36E8.2 0.204187238419749 AGIWUR33067
CB4856 CT III 3999286 4004143 WBGene00007980 C36E8.1 C36E8.1 0.0812572489223591
AGIWUR1345;AGIWUR21250;AGIWUR23164
CB4856 CT III 4006142 4009960 WBGene00007982 pxd-1 C36E8.3 0.105027805134997 AGIWUR29639
CB4856 CT III 4012336 4015763 WBGene00007983 C36E8.4 C36E8.4 0.358263361324443 AGIWUR41273
CB4856 CT III 4015769 4017643 WBGene00006537 tbb-2 C36E8.5 -0.0736284385952573
AGIWUR668;AGIWUR1222;AGIWUR16069;AGIWUR18887;AGIWUR19179;AGIWUR42288
CB4856 CT III 4020037 4021867 WBGene00011367 snpc-3.4 T02C12.2 -0.174665813030963
AGIWUR24584
CB4856 CT III 4022166 4025658 WBGene00011368 tftc-5 T02C12.3 -0.0604859873184047
AGIWUR29547
CB4856 CT III 4029115 4036840 WBGene00002038 hum-5 T02C12.1 0.00488316737435568
AGIWUR9502
CB4856 CT III 4052807 4057976 WBGene00004322 rcq-5 E03A3.2 -0.11922380449235
AGIWUR3697;AGIWUR26154;AGIWUR31769
CB4856 CT III 4058272 4058743 WBGene00001943 his-69 E03A3.3 -0.0184533130750637 AGIWUR40118
CB4856 CT III 4059647 4060172 WBGene00001944 his-70 E03A3.4 -0.325867652683824 AGIWUR14280
CB4856 CT III 4060591 4062822 WBGene00008465 E03A3.5 E03A3.5 -0.295743008851659 AGIWUR14656
CB4856 CT III 4066717 4082794 WBGene00006811 unc-79 E03A3.6 -0.0320515052106387
AGIWUR4423;AGIWUR5142;AGIWUR15798;AGIWUR15963;AGIWUR18293;AGIWUR20493;AGIWUR28967;AGIWUR39605;AGIWUR41832
CB4856 CT III 4084003 4086636 WBGene00002202 kin-19 C03C10.1 0.0765261333601082
AGIWUR1828;AGIWUR5712;AGIWUR8099;AGIWUR15304;AGIWUR16042;AGIWUR21790;AGIWUR22763;AGIWUR30118;AGIWUR32074;AG
IWUR33100;AGIWUR34225;AGIWUR34996;AGIWUR37313;AGIWUR37347
CB4856 CT III 4090291 4092120 WBGene00007271 C03C10.5 C03C10.5 1.25832891010337
AGIWUR25475

CB4856	CT	III	4091126	4093311	WBGene00007269	C03C10.2	C03C10.2	-0.0076831776770474	AGIWUR111
CB4856	CT	III	4093306	4094921	WBGene00004392	rnr-2	C03C10.3	-0.214955084629947	AGIWUR10602;AGIWUR20109;AGIWUR25130;AGIWUR33669;AGIWUR38097;AGIWUR44770
CB4856	CT	III	4096045	4096915	WBGene00007273	C03C10.7	C03C10.7	-0.0198253540183888	AGIWUR44164
CB4856	CT	III	4096896	4098190	WBGene00007270	rei-1	C03C10.4	-0.0115029756944267	AGIWUR20043
CB4856	CT	III	4115226	4147255	WBGene00006830	unc-103	C30D11.1	0.119787520523774	AGIWUR8148;AGIWUR9828;AGIWUR16874;AGIWUR24520;AGIWUR31702;AGIWUR35136;AGIWUR35765;AGIWUR38831;AGIWUR41941
CB4856	CT	III	4147425	4151598	WBGene00007631	wht-3	C16C10.12	-0.328559534260307	AGIWUR39934
CB4856	CT	III	4152054	4152915	WBGene00007630	har-1	C16C10.11	-0.439572336835602	AGIWUR2630;AGIWUR3696;AGIWUR4247;AGIWUR8756;AGIWUR24873;AGIWUR36702
CB4856	CT	III	4153599	4154650	WBGene00006448	glod-4	C16C10.10	-0.221749279257132	AGIWUR16289
CB4856	CT	III	4155388	4157394	WBGene00007629	C16C10.9	C16C10.9	0.0968076204948574	AGIWUR38186
CB4856	CT	III	4161951	4164171	WBGene00007628	C16C10.8	C16C10.8	-0.128626030328167	AGIWUR15159
CB4856	CT	III	4164275	4166845	WBGene00004381	rnf-5	C16C10.7	0.320626647353222	AGIWUR10393
CB4856	CT	III	4166944	4168765	WBGene00007627	ccdc-55	C16C10.6	-0.0178365760431358	AGIWUR21267
CB4856	CT	III	4168797	4170949	WBGene00007626	rnf-121	C16C10.5	-0.115302927990351	AGIWUR2199
CB4856	CT	III	4171336	4172223	WBGene00007625	C16C10.4	C16C10.4	0.0340280890414384	AGIWUR17931
CB4856	CT	III	4172337	4176159	WBGene00007624	hrde-1	C16C10.3	-0.0266445429602111	AGIWUR20823
CB4856	CT	III	4176678	4177578	WBGene00007632	C16C10.13	C16C10.13	-0.178598218636603	AGIWUR1567
CB4856	CT	III	4177532	4178664	WBGene00007623	C16C10.2	C16C10.2	-0.0646537271436429	AGIWUR10585;AGIWUR12996;AGIWUR21393;AGIWUR27638;AGIWUR30103
CB4856	CT	III	4178783	4181438	WBGene00007622	C16C10.1	C16C10.1	0.0581560497855591	AGIWUR31291
CB4856	CT	III	4181543	4186825	WBGene00003073	lars-1	R74.1	-0.139726967546684	AGIWUR30809
CB4856	CT	III	4188160	4189285	WBGene00011278	R74.2	R74.2	-0.548129148281859	AGIWUR27575;AGIWUR27987;AGIWUR31182;AGIWUR31227;AGIWUR43478

CB4856	CT	III	4189380	4190341	WBGene00206525	R74.10	R74.10	-0.0713102393256762	AGIWUR44068
CB4856	CT	III	4191141	4193427	WBGene00001034	dnj-16	R74.4	-0.318531810829245
AGIWUR19228;AGIWUR30349
CB4856	CT	III	4193809	4195358	WBGene00006959	xbp-1	R74.3	-0.084840535377392
AGIWUR16166;AGIWUR22379;AGIWUR37406
CB4856	CT	III	4197285	4202289	WBGene00011279	asd-1	R74.5	0.000975754766546544
AGIWUR33402;AGIWUR35999
CB4856	CT	III	4202253	4204269	WBGene00011280	R74.6	R74.6	-0.284111386205872
AGIWUR1687;AGIWUR16800;AGIWUR35134
CB4856	CT	III	4204429	4205814	WBGene00011281	R74.7	R74.7	-0.202914851527103	AGIWUR28021
CB4856	CT	III	4206253	4208265	WBGene00011282	R74.8	R74.8	-0.163068253590007
AGIWUR5769;AGIWUR22059
CB4856	CT	III	4210562	4216724	WBGene00009647	F43C1.1	F43C1.1	0.598213225109276	AGIWUR35794
CB4856	CT	III	4216764	4228108	WBGene00003401	mpk-1	F43C1.2	-0.0979806524537379
AGIWUR1958;AGIWUR9733;AGIWUR14898;AGIWUR14905;AGIWUR17406;AGIWUR40763
CB4856	CT	III	4229141	4230427	WBGene00009649	F43C1.5	F43C1.5	-0.335536836528298	AGIWUR1306
CB4856	CT	III	4230436	4233219	WBGene00003619	nhr-20	F43C1.4	0.0737823990593842	AGIWUR42423
CB4856	CT	III	4241477	4242593	WBGene00023423	F43C1.7	F43C1.7	-0.17267051605681
AGIWUR8452;AGIWUR19266;AGIWUR33069
CB4856	CT	III	4242672	4243561	WBGene00023422	mrpl-21	F43C1.6	-0.0807728142961537	AGIWUR5851
CB4856	CT	III	4243674	4244385	WBGene00009648	zhit-2	F43C1.3	0.0567175435839062
AGIWUR3825;AGIWUR22128;AGIWUR36936;AGIWUR44159
CB4856	CT	III	4244858	4247671	WBGene00012858	Y44F5A.1	Y44F5A.1	-0.20838580927059
AGIWUR13909
CB4856	CT	III	4248155	4263048	WBGene00011604	T08A11.1	T08A11.1	0.0206921938630571
AGIWUR8299;AGIWUR14489;AGIWUR20691;AGIWUR32732;AGIWUR33366;AGIWUR37156
CB4856	CT	III	4263785	4269270	WBGene00011605	sftb-1	T08A11.2	-0.191596174376222
AGIWUR1553;AGIWUR11891;AGIWUR19820;AGIWUR25548
CB4856	CT	III	4269743	4272506	WBGene00011198	R10E4.1	R10E4.1	0.013308491697396
AGIWUR7076;AGIWUR9157;AGIWUR18471
CB4856	CT	III	4276266	4280217	WBGene00006331	sup-26	R10E4.2	0.712304109783651
AGIWUR6823;AGIWUR16442;AGIWUR16959;AGIWUR20757;AGIWUR24961;AGIWUR41251
CB4856	CT	III	4285861	4287472	WBGene00011200	R10E4.3	R10E4.3	0.0431644814828838	AGIWUR36049
CB4856	CT	III	4288264	4291283	WBGene00003157	mcm-5	R10E4.4	0.0205620429455777
AGIWUR11433;AGIWUR14883;AGIWUR23176;AGIWUR25320;AGIWUR33448
CB4856	CT	III	4291543	4293413	WBGene00011201	nth-1	R10E4.5	-0.136618743271047	AGIWUR25315
CB4856	CT	III	4293408	4295142	WBGene00011202	R10E4.6	R10E4.6	0.234600727737837	AGIWUR16345
CB4856	CT	III	4295224	4296599	WBGene00011203	R10E4.7	R10E4.7	0.400266425619629	AGIWUR34301
CB4856	CT	III	4297239	4301039	WBGene00011206	R10E4.11	R10E4.11	-0.339928655800399

AGIWUR3790
CB4856	CT	III	4301620 4302393 WBGene00011205	R10E4.9 R10E4.9 0.0459315931593647	AGIWUR33427
CB4856	CT	III	4303096 4313128 WBGene00001352	evl-14	H38K22.1	-0.0382007705079133
AGIWUR33989
CB4856	CT	III	4313927 4317608 WBGene00010428	dcn-1	H38K22.2	-0.151800754184164
AGIWUR30845
CB4856	CT	III	4317702 4320341 WBGene00006478	tag-131 H38K22.3	0.128441557310353
AGIWUR20634
CB4856	CT	III	4320389 4321348 WBGene00044911	H38K22.6	H38K22.6	-0.238096774773156
AGIWUR13317;AGIWUR17809;AGIWUR26894;AGIWUR40933;AGIWUR41799
CB4856	CT	III	4321748 4322867 WBGene00044912	H38K22.7	H38K22.7	-0.173372198667662
AGIWUR917;AGIWUR9416;AGIWUR19948;AGIWUR20893;AGIWUR30626
CB4856	CT	III	4324280 4331227 WBGene00001631	gly-6	H38K22.5	0.0706090144165543
AGIWUR11179;AGIWUR26035;AGIWUR27415;AGIWUR29155;AGIWUR39994
CB4856	CT	III	4332996 4340464 WBGene00007135	cdk-12	B0285.1 -0.115838290638708	AGIWUR15012
CB4856	CT	III	4341983 4343336 WBGene00007136	B0285.3 B0285.3 -0.0121462086975708	AGIWUR31964
CB4856	CT	III	4343446 4345366 WBGene00007137	B0285.4 B0285.4 0.291998300412345	AGIWUR9788
CB4856	CT	III	4345339 4350079 WBGene00002003	hse-5	B0285.5 -0.0111737284884583	AGIWUR25929
CB4856	CT	III	4352246 4355888 WBGene00007138	B0285.6 B0285.6 -0.0762984507237157	AGIWUR18506
CB4856	CT	III	4356539 4364974 WBGene00007139	mnp-1	B0285.7 0.405748826079963	AGIWUR19898
CB4856	CT	III	4365285 4367469 WBGene00000511	ckb-1	B0285.8 -0.390007863366122	AGIWUR25566
CB4856	CT	III	4367832 4370160 WBGene00000512	ckb-2	B0285.9 -0.951254959914598	AGIWUR19613
CB4856	CT	III	4370885 4372820 WBGene00000513	ckb-3	B0285.10	-0.122721866959082
AGIWUR12272
CB4856	CT	III	4373179 4375323 WBGene00045188	B0284.5 B0284.5 0.155354907072569
AGIWUR6647;AGIWUR44597
CB4856	CT	III	4376503 4379200 WBGene00007131	pals-26 B0284.1 1.11866494551985
AGIWUR659;AGIWUR34401
CB4856	CT	III	4379368 4381041 WBGene00007133	B0284.3 B0284.3 -0.162891821107074	AGIWUR22476
CB4856	CT	III	4381739 4384141 WBGene00007132	pals-27 B0284.2 0.939023885803728	AGIWUR2667
CB4856	CT	III	4384787 4387757 WBGene00007134	pals-28 B0284.4 0.462170980565065	AGIWUR31960
CB4856	CT	III	4394796 4396617 WBGene00011112	R07E5.4 R07E5.4 -0.462156958030345	AGIWUR14612
CB4856	CT	III	4397415 4398576 WBGene00011113	R07E5.5 R07E5.5 -0.0997334428771381	AGIWUR38210
CB4856	CT	III	4398763 4400942 WBGene00011114	R07E5.6 R07E5.6 0.0462817334631396	AGIWUR35081
CB4856	CT	III	4401442 4402505 WBGene00011120	R07E5.15	R07E5.15	-0.225347588494837
AGIWUR37803
CB4856	CT	III	4402595 4404436 WBGene00011121	R07E5.17	R07E5.17	0.312912328334975
AGIWUR2552;AGIWUR38683
CB4856	CT	III	4404786 4406285 WBGene00011111	snfc-5	R07E5.3 -0.192862021021273

AGIWUR11492;AGIWUR14649;AGIWUR22515;AGIWUR25831;AGIWUR28126;AGIWUR28837;AGIWUR29804;AGIWUR32754;AGIWUR35093
CB4856 CT III 4406286 4407326 WBGene00004387 rnp-4 R07E5.14 -0.0938343450226355
AGIWUR11861;AGIWUR14460;AGIWUR19160;AGIWUR24049;AGIWUR25412;AGIWUR25790;AGIWUR27926
CB4856 CT III 4407444 4408437 WBGene00011110 prdx-3 R07E5.2 -0.17258584873211
AGIWUR25524;AGIWUR35814;AGIWUR37579;AGIWUR41024
CB4856 CT III 4408551 4412149 WBGene00011109 R07E5.1 R07E5.1 -0.224855030291346 AGIWUR8228
CB4856 CT III 4413457 4414748 WBGene00011119 mpc-1 R07E5.13 -0.118378393557611
AGIWUR5754;AGIWUR9776;AGIWUR19771;AGIWUR33099;AGIWUR40240
CB4856 CT III 4414732 4415235 WBGene00011117 R07E5.11 R07E5.11 -0.390529318070355
AGIWUR32637
CB4856 CT III 4415520 4418058 WBGene00011116 pdcd-2 R07E5.10 -0.0671905714915191
AGIWUR4236;AGIWUR6736;AGIWUR6974;AGIWUR15557;AGIWUR19842;AGIWUR20601;AGIWUR20807;AGIWUR36005;AGIWUR38031;AG
IWUR38783
CB4856 CT III 4418343 4419167 WBGene00011115 R07E5.7 R07E5.7 -0.1323338073647
AGIWUR1971;AGIWUR7078;AGIWUR8963;AGIWUR28166;AGIWUR32271
CB4856 CT III 4419848 4423368 WBGene00000520 cku-80 R07E5.8 -0.246155247371554 AGIWUR29231
CB4856 CT III 4423711 4429499 WBGene00007791 C28A5.6 C28A5.6 -0.106194736692498 AGIWUR37808
CB4856 CT III 4442081 4444237 WBGene00003590 nex-3 C28A5.3 -0.384087073217355 AGIWUR30120
CB4856 CT III 4446637 4449943 WBGene00000463 ceh-43 C28A5.4 1.04037616270002 AGIWUR11902
CB4856 CT III 4462833 4467377 WBGene00003577 ndg-4 F56F3.2 -0.412601777363854
AGIWUR18135;AGIWUR26646
CB4856 CT III 4468878 4470808 WBGene00010155 F56F3.4 F56F3.4 0.11980801570312 AGIWUR33529
CB4856 CT III 4471407 4475150 WBGene00004132 ifet-1 F56F3.1 -0.221485350159389
AGIWUR13827;AGIWUR40868;AGIWUR42975
CB4856 CT III 4475918 4476887 WBGene00004470 rps-1 F56F3.5 -0.0903020525944083
AGIWUR7578;AGIWUR16537;AGIWUR18534;AGIWUR24392;AGIWUR34321;AGIWUR36331
CB4856 CT III 4476888 4477583 WBGene00002100 ins-17 F56F3.6 0.402112386784972
AGIWUR7314;AGIWUR9236;AGIWUR11108;AGIWUR13900;AGIWUR16963;AGIWUR24675
CB4856 CT III 4489359 4491392 WBGene00000465 cpg-1 C07G2.1 -0.0465916228419267
AGIWUR3126;AGIWUR4450;AGIWUR6962;AGIWUR9701;AGIWUR25056;AGIWUR28301;AGIWUR33939
CB4856 CT III 4491627 4499043 WBGene00000223 atf-7 C07G2.2 0.0920190031170129
AGIWUR4927;AGIWUR5314;AGIWUR14974;AGIWUR20820;AGIWUR24837;AGIWUR29854;AGIWUR31507;AGIWUR34947;AGIWUR35619;A
GIWUR37894;AGIWUR42736
CB4856 CT III 4499191 4508114 WBGene00000380 cct-5 C07G2.3 -0.223986571485724
AGIWUR3833;AGIWUR4408;AGIWUR31324;AGIWUR40854
CB4856 CT III 4508855 4512162 WBGene00009115 F25F2.1 F25F2.1 -0.0217380998210324 AGIWUR307
CB4856 CT III 4520363 4536991 WBGene00000396 cdh-4 F25F2.2 0.206983308538145 AGIWUR20794
CB4856 CT III 4537277 4540644 WBGene00010904 ugt-62 M88.1 -0.67877988516757 AGIWUR13910
CB4856 CT III 4541326 4542452 WBGene00010905 mrps-34 M88.2 -0.165142143293703 AGIWUR38242

CB4856	CT	III	4542881 4546025 WBGene00010909	cisd-3.1	M88.7	-0.145234517870154
AGIWUR21078
CB4856	CT	III	4549063 4551567 WBGene00010907	M88.4	M88.4	-0.171853378648883	AGIWUR44096
CB4856	CT	III	4553618 4560350 WBGene00010908	zbp-1	M88.5	0.10225025359976
AGIWUR6561;AGIWUR6686;AGIWUR36835
CB4856	CT	III	4562738 4566276 WBGene00003915	pan-1	M88.6	0.241267788537851	AGIWUR26141
CB4856	CT	III	4566279 4568144 WBGene00009439	mlcd-1	F35G12.1	0.0911847162136068
AGIWUR2770;AGIWUR2817;AGIWUR13489;AGIWUR27496;AGIWUR33025;AGIWUR39632
CB4856	CT	III	4567325 4571835 WBGene00009440	idhg-1	F35G12.2	0.234626711097922
AGIWUR3341;AGIWUR9882;AGIWUR23281
CB4856	CT	III	4571753 4578437 WBGene00004762	sel-5	F35G12.3	-0.0743842570831887
AGIWUR4191;AGIWUR10977;AGIWUR11373;AGIWUR15605;AGIWUR17298;AGIWUR22658;AGIWUR30054;AGIWUR38843
CB4856	CT	III	4578855 4581696 WBGene00009441	wdr-48	F35G12.4	-0.0618972745360074
AGIWUR16083;AGIWUR31303;AGIWUR33315
CB4856	CT	III	4581795 4583893 WBGene00009445	F35G12.12	F35G12.12	-0.19504035395261
AGIWUR19409
CB4856	CT	III	4584583 4586753 WBGene00009442	F35G12.5	F35G12.5	0.168821914337773
AGIWUR40495
CB4856	CT	III	4586628 4587330 WBGene00009443	F35G12.7	F35G12.7	-0.103230761492174
AGIWUR3544
CB4856	CT	III	4587415 4593124 WBGene00004874	smc-4	F35G12.8	0.0295275465801019
AGIWUR39484
CB4856	CT	III	4593176 4593832 WBGene00009444	F35G12.11	F35G12.11	-0.372168771141032
AGIWUR25108
CB4856	CT	III	4593769 4594426 WBGene00000145	apc-11	F35G12.9	0.0919696626975028
AGIWUR11099
CB4856	CT	III	4594543 4595944 WBGene00000206	asb-1	F35G12.10	-0.137580669778377
AGIWUR14757;AGIWUR15446;AGIWUR18553
CB4856	CT	III	4596134 4612384 WBGene00004760	sel-2	F10F2.1 0.0675584570429244	AGIWUR7358
CB4856	CT	III	4616479 4618893 WBGene00008655	lips-3	F10F2.3 -0.13326172455269
AGIWUR2515;AGIWUR12349;AGIWUR13166;AGIWUR21826;AGIWUR35962;AGIWUR38897
CB4856	CT	III	4618853 4643305 WBGene00008654	F10F2.2 F10F2.2 -0.0394842352166993	AGIWUR33800
CB4856	CT	III	4643999 4646346 WBGene00004118	pqn-29	F10F2.9 0.0068495010455738	AGIWUR32867
CB4856	CT	III	4646507 4647854 WBGene00011957	T23F11.6	T23F11.6	0.0193465768755914
AGIWUR29047
CB4856	CT	III	4648591 4653929 WBGene00011953	ppm-2	T23F11.1	0.0625418877875319
AGIWUR2592;AGIWUR7677;AGIWUR16510;AGIWUR23088;AGIWUR31765
CB4856	CT	III	4658739 4662697 WBGene00011955	cdka-1	T23F11.3	0.145164017520031
AGIWUR5542;AGIWUR14846;AGIWUR38432;AGIWUR39512

CB4856 CT III 4663185 4665368 WBGene00011956 T23F11.4 T23F11.4 -0.435466026319924
AGIWUR1001
CB4856 CT III 4672249 4674207 WBGene00005170 srg-13 T23F11.5 0.235943369032393
AGIWUR40917
CB4856 CT III 4677766 4679663 WBGene00005168 srg-10 T04A8.1 0.127760552495555 AGIWUR27750
CB4856 CT III 4680013 4681562 WBGene00005169 srg-11 T04A8.2 -0.140034081571085 AGIWUR13241
CB4856 CT III 4682774 4685533 WBGene00011405 clec-155 T04A8.3 -0.116504639619258
AGIWUR11086
CB4856 CT III 4686088 4688210 WBGene00044078 tag-243 T04A8.4 0.668533492482755
AGIWUR18509;AGIWUR24487
CB4856 CT III 4689280 4692166 WBGene00011407 ppat-1 T04A8.5 -0.400852358328862 AGIWUR40473
CB4856 CT III 4692098 4693303 WBGene00011408 T04A8.6 T04A8.6 -0.171883806199698 AGIWUR18609
CB4856 CT III 4694003 4695270 WBGene00011410 T04A8.8 T04A8.8 0.123093207372696 AGIWUR12099
CB4856 CT III 4695361 4698010 WBGene00011409 T04A8.7 T04A8.7 -0.0432620638657575
AGIWUR21833;AGIWUR25655;AGIWUR27695;AGIWUR37122
CB4856 CT III 4697987 4699453 WBGene00001036 dnj-18 T04A8.9 0.0206006774340466
AGIWUR2611;AGIWUR38656;AGIWUR41916
CB4856 CT III 4699407 4700224 WBGene00011411 sel-13 T04A8.10 -0.0133166901235988
AGIWUR32048
CB4856 CT III 4700325 4704054 WBGene00000543 clp-2 T04A8.16 -0.115234129640072
AGIWUR21921
CB4856 CT III 4704404 4705404 WBGene00011412 mrpl-16 T04A8.11 -0.099026057871896
AGIWUR8495;AGIWUR22631;AGIWUR34099
CB4856 CT III 4705413 4706949 WBGene00007045 tag-189 T04A8.12 -0.154649515305186
AGIWUR964;AGIWUR10737;AGIWUR33930
CB4856 CT III 4709248 4712547 WBGene00119203 T04A8.18 T04A8.18 -0.283756767198725
AGIWUR29768
CB4856 CT III 4712572 4715188 WBGene00011415 him-18 T04A8.15 -0.0132775739561666
AGIWUR14976
CB4856 CT III 4715293 4720670 WBGene00001259 emb-5 T04A8.14 0.0543403446893134
AGIWUR10332;AGIWUR10446;AGIWUR22030;AGIWUR23199;AGIWUR23677;AGIWUR30711;AGIWUR33433;AGIWUR34113;AGIWUR36496
CB4856 CT III 4722126 4730919 WBGene00000068 acy-1 F17C8.1 0.0598613172689291 AGIWUR11552
CB4856 CT III 4734356 4736065 WBGene00185014 F17C8.9 F17C8.9 -0.36191449858312
AGIWUR14597;AGIWUR41893
CB4856 CT III 4736050 4737258 WBGene00008912 F17C8.7 F17C8.7 -0.414347170751377 AGIWUR19939
CB4856 CT III 4737589 4740075 WBGene00004311 ras-2 F17C8.4 -0.140278591046241
AGIWUR2834;AGIWUR17837;AGIWUR26706;AGIWUR32802
CB4856 CT III 4748127 4749164 WBGene00000664 col-89 F17C8.2 -0.15358260753003 AGIWUR18994
CB4856 CT III 4752998 4754083 WBGene00004469 rps-0 B0393.1 -0.0669748963977668

AGIWUR193;AGIWUR6226;AGIWUR6783
CB4856 CT III 4754432 4757017 WBGene00007167 rbg-3 B0393.2 -0.156786114798692 AGIWUR3240
CB4856 CT III 4757201 4759728 WBGene00007168 B0393.3 B0393.3 -0.0631100439854357 AGIWUR43498
CB4856 CT III 4759744 4763609 WBGene00007169 B0393.4 B0393.4 -0.221549634915846 AGIWUR6601
CB4856 CT III 4765833 4776342 WBGene00007170 B0393.5 B0393.5 0.527524113430539 AGIWUR34080
CB4856 CT III 4776264 4778065 WBGene00007172 B0393.7 B0393.7 0.0681605938512142 AGIWUR36678
CB4856 CT III 4778390 4780157 WBGene00044636 B0393.9 B0393.9 -0.0850210568774461
AGIWUR1374;AGIWUR3283;AGIWUR40762
CB4856 CT III 4780130 4781987 WBGene00007171 B0393.6 B0393.6 0.382596345342403 AGIWUR45184
CB4856 CT III 4786080 4792391 WBGene00003210 mel-28 C38D4.3 -0.100090065558859 AGIWUR40516
CB4856 CT III 4793086 4795423 WBGene00008005 C38D4.4 C38D4.4 -0.242024953440968 AGIWUR5766
CB4856 CT III 4796839 4802907 WBGene00008006 tag-325 C38D4.5 -0.152886762323877 AGIWUR17735
CB4856 CT III 4803007 4804753 WBGene00008008 C38D4.9 C38D4.9 0.0742855198983499
AGIWUR4922;AGIWUR26328;AGIWUR39586
CB4856 CT III 4804708 4809489 WBGene00003912 pal-1 C38D4.6 -0.0165130023294351
AGIWUR3503;AGIWUR8986;AGIWUR17483;AGIWUR25142;AGIWUR26070;AGIWUR26964;AGIWUR29813;AGIWUR34020
CB4856 CT III 4810031 4811162 WBGene00008007 C38D4.7 C38D4.7 -0.192743015153388 AGIWUR275
CB4856 CT III 4813309 4814814 WBGene00000193 arl-6 C38D4.8 0.443387716466138 AGIWUR6297
CB4856 CT III 4816040 4817078 WBGene00017804 F26A1.6 F26A1.6 -0.028612030743405 AGIWUR20049
CB4856 CT III 4818042 4819073 WBGene00017803 F26A1.4 F26A1.4 -0.212518718413031 AGIWUR21527
CB4856 CT III 4819328 4819943 WBGene00017805 F26A1.7 F26A1.7 0.0602120988148407 AGIWUR34354
CB4856 CT III 4820670 4822226 WBGene00017806 F26A1.8 F26A1.8 -0.510585161022332 AGIWUR25170
CB4856 CT III 4825345 4829509 WBGene00017807 F26A1.9 F26A1.9 -0.352797521349032 AGIWUR16711
CB4856 CT III 4830010 4830349 WBGene00017808 nspd-9 F26A1.10 0.020601183688747
AGIWUR5361
CB4856 CT III 4837731 4839931 WBGene00270319 F26A1.19 F26A1.19 0.0970427906880094
AGIWUR745
CB4856 CT III 4840187 4841024 WBGene00044603 acbp-7 F26A1.15 0.0369689467527605
AGIWUR8465;AGIWUR28608
CB4856 CT III 4841039 4842273 WBGene00017809 clec-156 F26A1.11 -0.367748842156153
AGIWUR40380
CB4856 CT III 4843493 4844410 WBGene00017810 clec-157 F26A1.12 -0.309564630844259
AGIWUR41337
CB4856 CT III 4844662 4846546 WBGene00001437 fkh-5 F26A1.2 0.0723379136420288
AGIWUR10841;AGIWUR35894;AGIWUR40674
CB4856 CT III 4847072 4848967 WBGene00017811 F26A1.13 F26A1.13 -0.260632980341396
AGIWUR9823;AGIWUR14986;AGIWUR25874
CB4856 CT III 4849266 4849850 WBGene00017812 F26A1.14 F26A1.14 -0.377981365285153
AGIWUR21644;AGIWUR25758;AGIWUR38856

CB4856	CT	III	4850507	4851910	WBGene00017801	F26A1.1	F26A1.1	-0.0602845091505397	AGIWUR13881
CB4856	CT	III	4852313	4855177	WBGene00000418	ced-4	C35D10.9	-0.0802187175870639	AGIWUR5354;AGIWUR5654;AGIWUR41670
CB4856	CT	III	4855250	4856101	WBGene00016445	C35D10.8	C35D10.8	0.136610696833902	AGIWUR5688
CB4856	CT	III	4856193	4859240	WBGene00016444	C35D10.7	C35D10.7	-0.155811632213147	AGIWUR24486;AGIWUR38743
CB4856	CT	III	4859339	4860772	WBGene00016443	C35D10.6	C35D10.6	-0.131163214326204	AGIWUR6719
CB4856	CT	III	4860669	4861794	WBGene00016446	C35D10.10	C35D10.10	-0.28118827782618	AGIWUR44106
CB4856	CT	III	4862351	4863426	WBGene00016442	C35D10.5	C35D10.5	-0.240889977219608	AGIWUR10145;AGIWUR15024;AGIWUR19643;AGIWUR27288
CB4856	CT	III	4863427	4866949	WBGene00000767	coq-8	C35D10.4	-0.181065676930576	AGIWUR31304;AGIWUR41201;AGIWUR43913
CB4856	CT	III	4867062	4867469	WBGene00016452	C35D10.17	C35D10.17	0.154887702993191	AGIWUR26941
CB4856	CT	III	4868333	4868769	WBGene00016447	msd-4	C35D10.11	0.0476941577456422	AGIWUR19153
CB4856	CT	III	4868921	4870106	WBGene00016441	C35D10.3	C35D10.3	-0.225216292426769	AGIWUR25259
CB4856	CT	III	4870762	4872046	WBGene00016440	gipc-1	C35D10.2	0.156485747208706	AGIWUR18831;AGIWUR36466
CB4856	CT	III	4872501	4874751	WBGene00016448	C35D10.12	C35D10.12	0.02609038259091	AGIWUR21860
CB4856	CT	III	4877526	4878311	WBGene00016449	C35D10.13	C35D10.13	-0.0227950338079754	AGIWUR28892
CB4856	CT	III	4878658	4879643	WBGene00000204	arx-6	C35D10.16	0.0299448577435193	AGIWUR41105
CB4856	CT	III	4879745	4881497	WBGene00016439	C35D10.1	C35D10.1	-0.0422786735928309	AGIWUR11893;AGIWUR14564
CB4856	CT	III	4881909	4883606	WBGene00016450	clec-5	C35D10.14	-0.655218017138772	AGIWUR14533
CB4856	CT	III	4885697	4887442	WBGene00016451	clec-6	C35D10.15	-0.201666848784583	AGIWUR7022
CB4856	CT	III	4887921	4891252	WBGene00017827	F26F4.5	F26F4.5	-0.259040618022647	AGIWUR19753
CB4856	CT	III	4891609	4893409	WBGene00017828	F26F4.6	F26F4.6	0.151645969089735	AGIWUR3837;AGIWUR40615
CB4856	CT	III	4894647	4899085	WBGene00003598	nhl-2	F26F4.7	0.104386558980915	AGIWUR5479

CB4856	CT	III	4900073	4903143	WBGene00017826	tag-340	F26F4.4	-0.136568795545816	AGIWUR3465
CB4856	CT	III	4903177	4903884	WBGene00017832	kbp-2	F26F4.13	-0.172838233324086
AGIWUR33560
CB4856	CT	III	4903997	4905473	WBGene00005011	F26F4.8	F26F4.8	-0.00433714554875486	AGIWUR28628
CB4856	CT	III	4907217	4908968	WBGene00004400	rom-1	F26F4.3	-0.143301667088792	AGIWUR39550
CB4856	CT	III	4908960	4910038	WBGene00017831	F26F4.12	F26F4.12	-0.110679714175941
AGIWUR21349;AGIWUR24383;AGIWUR27298;AGIWUR33398
CB4856	CT	III	4910475	4912521	WBGene00017829	F26F4.9	F26F4.9	-0.217259268472421
AGIWUR42549;AGIWUR44256
CB4856	CT	III	4912207	4914133	WBGene00017825	cee-1	F26F4.1	-0.307821795381111
AGIWUR2541;AGIWUR6238;AGIWUR9786;AGIWUR15199;AGIWUR42030
CB4856	CT	III	4914096	4917129	WBGene00004679	rars-1	F26F4.10	-0.131052111793039
AGIWUR12;AGIWUR6520;AGIWUR9621;AGIWUR21567;AGIWUR22168;AGIWUR24027;AGIWUR30044;AGIWUR36014;AGIWUR36740
CB4856	CT	III	4917104	4918008	WBGene00017830	rpb-8	F26F4.11	-0.304049348503353
AGIWUR18230;AGIWUR19441;AGIWUR25054;AGIWUR29048
CB4856	CT	III	4918277	4920319	WBGene00006735	ula-1	C26E6.8	-0.0298907555667842
AGIWUR12372;AGIWUR16420;AGIWUR26326
CB4856	CT	III	4920442	4921747	WBGene00016144	mmab-1	C26E6.11	0.243795008842998
AGIWUR11339
CB4856	CT	III	4922446	4930204	WBGene00004782	set-2	C26E6.9	-0.0750602425019755
AGIWUR1720;AGIWUR9964;AGIWUR18640;AGIWUR33687;AGIWUR35092;AGIWUR36053
CB4856	CT	III	4930602	4932253	WBGene00016145	C26E6.12	C26E6.12	-0.145781412718764
AGIWUR1262
CB4856	CT	III	4932450	4934945	WBGene00016143	eri-9	C26E6.7	0.0218578341520743	AGIWUR36611
CB4856	CT	III	4935578	4937223	WBGene00016142	mrps-18C	C26E6.6	-0.216655668390788
AGIWUR8152;AGIWUR10057;AGIWUR17312;AGIWUR26927;AGIWUR38877;AGIWUR40105
CB4856	CT	III	4937322	4939453	WBGene00001499	fsn-1	C26E6.5	-0.173372456627272	AGIWUR21648
CB4856	CT	III	4939567	4944404	WBGene00016140	rpb-2	C26E6.4	0.0192467189242187
AGIWUR1292;AGIWUR7484;AGIWUR13354;AGIWUR26461;AGIWUR26734;AGIWUR28828;AGIWUR31075;AGIWUR31332;AGIWUR34633;A
GIWUR36394;AGIWUR38499
CB4856	CT	III	4944513	4947317	WBGene00016139	ntl-9	C26E6.3	-0.211997450625226
AGIWUR4765;AGIWUR6733;AGIWUR29362
CB4856	CT	III	4952124	4955171	WBGene00016138	flh-2	C26E6.2	0.188678856474585
AGIWUR7622;AGIWUR8591;AGIWUR17173;AGIWUR26133;AGIWUR26422;AGIWUR33680
CB4856	CT	III	4956891	4957539	WBGene00016137	C26E6.1	C26E6.1	0.200365237849195	AGIWUR6750
CB4856	CT	III	4958329	4960305	WBGene00016166	C27F2.4	C27F2.4	-0.0906903099563046	AGIWUR5822
CB4856	CT	III	4960390	4961715	WBGene00016167	vps-22	C27F2.5	-0.155963291101841
AGIWUR27289;AGIWUR30151;AGIWUR37552
CB4856	CT	III	4961817	4963092	WBGene00043156	C27F2.9	C27F2.9	-0.271393729806995

AGIWUR5606;AGIWUR22616;AGIWUR34511;AGIWUR35346;AGIWUR35703;AGIWUR36189
CB4856 CT III 4965571 4979729 WBGene00003558 nca-2 C27F2.2 -0.0764024410106501 AGIWUR38222
CB4856 CT III 4979796 4980418 WBGene00016168 C27F2.6 C27F2.6 -0.0139527790832261 AGIWUR5107
CB4856 CT III 4980644 4984474 WBGene00016169 C27F2.7 C27F2.7 -0.205168639560497
AGIWUR3709;AGIWUR13500;AGIWUR26613;AGIWUR44036
CB4856 CT III 4985019 4986931 WBGene00016171 C27F2.10 C27F2.10 0.0272430298440263
AGIWUR8510
CB4856 CT III 4987209 4991450 WBGene00016165 C27F2.1 C27F2.1 -0.100012915538091 AGIWUR8370
CB4856 CT III 4991439 4998161 WBGene00016170 C27F2.8 C27F2.8 -0.0517647213879151
AGIWUR7004;AGIWUR15438;AGIWUR21077;AGIWUR22621;AGIWUR35360
CB4856 CT III 4999379 5001336 WBGene00020095 R144.5 R144.5 0.415445388123389 AGIWUR12094
CB4856 CT III 5001297 5002331 WBGene00020098 R144.10 R144.10 -0.389780513435044 AGIWUR808
CB4856 CT III 5003197 5004721 WBGene00020096 R144.6 R144.6 0.0323074017796365 AGIWUR16813
CB4856 CT III 5004820 5006981 WBGene00043060 R144.12 R144.12 -0.189263452468017 AGIWUR1049
CB4856 CT III 5007100 5008830 WBGene00020099 R144.11 R144.11 -0.213840005429958 AGIWUR11854
CB4856 CT III 5008933 5017860 WBGene00020097 larp-1 R144.7 -0.0553372069379137
AGIWUR5277;AGIWUR8701;AGIWUR34641
CB4856 CT III 5018645 5021118 WBGene00020094 wip-1 R144.4 0.00405149749026634
AGIWUR10954;AGIWUR22155;AGIWUR23908
CB4856 CT III 5021224 5021910 WBGene00007015 mdt-11 R144.9 0.00116498674956844 AGIWUR43452
CB4856 CT III 5022047 5023200 WBGene00020093 R144.3 R144.3 -0.109079711255667
AGIWUR20707;AGIWUR39793
CB4856 CT III 5023264 5024064 WBGene00044648 R144.13 R144.13 -0.237332887056082 AGIWUR7341
CB4856 CT III 5024966 5029540 WBGene00020092 pcf-11 R144.2 -0.0979885210834865
AGIWUR6885;AGIWUR28587;AGIWUR41768
CB4856 CT III 5030929 5036548 WBGene00002218 klp-6 R144.1 0.0450399955580978
AGIWUR6002;AGIWUR10422;AGIWUR27584;AGIWUR31010
CB4856 CT III 5036381 5040413 WBGene00016677 C45G9.6 C45G9.6 -0.00691360118743667
AGIWUR2247;AGIWUR31234
CB4856 CT III 5042412 5044672 WBGene00016684 nlp-43 C45G9.13 0.349017326823478
AGIWUR41739
CB4856 CT III 5045009 5045667 WBGene00044409 C45G9.14 C45G9.14
-0.00185604471611889 AGIWUR6835;AGIWUR12672;AGIWUR13383;AGIWUR37679
CB4856 CT III 5046170 5047161 WBGene00016678 C45G9.7 C45G9.7 -0.214426668144743 AGIWUR3268
CB4856 CT III 5048117 5050158 WBGene00016676 C45G9.5 C45G9.5 -0.257239359197738 AGIWUR13838
CB4856 CT III 5050107 5052068 WBGene00050919 C45G9.15 C45G9.15 -0.0163210475630446
AGIWUR35385
CB4856 CT III 5052529 5053341 WBGene00016679 C45G9.8 C45G9.8 0.0783282274772796
AGIWUR5777;AGIWUR13001;AGIWUR13513;AGIWUR30470;AGIWUR36583

CB4856	CT	III	5056847	5061888	WBGene00016681	C45G9.10	C45G9.10	-0.145569611411895	AGIWUR12766
CB4856	CT	III	5063993	5068725	WBGene00016682	C45G9.11	C45G9.11	-0.080216057353617	AGIWUR36530
CB4856	CT	III	5069001	5070418	WBGene00016674	C45G9.2	C45G9.2	-0.10866778516979	AGIWUR38185;AGIWUR39760
CB4856	CT	III	5070431	5071597	WBGene00016683	C45G9.12	C45G9.12	-0.261056413354783	AGIWUR39034
CB4856	CT	III	5074698	5075968	WBGene00016673	C45G9.1	C45G9.1	-0.290751799615458	AGIWUR22776
CB4856	CT	III	5077765	5080665	WBGene00000107	alh-1	F54D8.3	0.552691302859747	AGIWUR22754;AGIWUR36700
CB4856	CT	III	5081726	5082995	WBGene00006519	tag-174	F54D8.2	-0.031773711968567	AGIWUR58;AGIWUR4165;AGIWUR4356;AGIWUR6331;AGIWUR6584;AGIWUR26746;AGIWUR28653;AGIWUR34083
CB4856	CT	III	5083206	5085289	WBGene00000279	cah-1	F54D8.4	-0.0322710808914906	AGIWUR22257
CB4856	CT	III	5102991	5106955	WBGene00018802	F54D8.6	F54D8.6	-0.293335620645742	AGIWUR15008;AGIWUR28987
CB4856	CT	III	5107330	5108497	WBGene00001076	dpy-17	F54D8.1	1.50966149019313	AGIWUR26534;AGIWUR31818
CB4856	CT	III	5109701	5130673	WBGene00004728	sax-2	F21H11.2	0.0370268567478487	AGIWUR6192;AGIWUR7162;AGIWUR12049;AGIWUR17202;AGIWUR32090
CB4856	CT	III	5130923	5136319	WBGene00006543	tbx-2	F21H11.3	0.466900259145097	AGIWUR14269
CB4856	CT	III	5137252	5149578	WBGene00021900	Y54H5A.2	Y54H5A.2	-0.123362633990848	AGIWUR5297;AGIWUR36403;AGIWUR39420
CB4856	CT	III	5151907	5153229	WBGene00021901	tag-262	Y54H5A.3	0.0938285613494863	AGIWUR36811
CB4856	CT	III	5153406	5155465	WBGene00021899	Y54H5A.1	Y54H5A.1	-0.108392302218355	AGIWUR27003
CB4856	CT	III	5155472	5158774	WBGene00021902	oxy-4	Y54H5A.4	-0.13957660879225	AGIWUR33031
CB4856	CT	III	5158873	5159605	WBGene00044604	T10F2.5	T10F2.5	-0.140278961417076	AGIWUR16189;AGIWUR34183;AGIWUR37130
CB4856	CT	III	5159729	5163627	WBGene00001744	gars-1	T10F2.1	-0.246575462130618	AGIWUR8674;AGIWUR17893
CB4856	CT	III	5163766	5165311	WBGene00020422	T10F2.2	T10F2.2	-0.193282851597987	AGIWUR5009
CB4856	CT	III	5165659	5169799	WBGene00006736	ulp-1	T10F2.3	0.0172311836210746	AGIWUR17583;AGIWUR39466;AGIWUR39693
CB4856	CT	III	5169933	5172045	WBGene00020423	prp-19	T10F2.4	0.338355835711513	AGIWUR8511;AGIWUR11498;AGIWUR12677;AGIWUR16041;AGIWUR16667;AGIWUR42332

CB4856 CT III 5172720 5173338 WBGene00019631 K10D2.5 K10D2.5 0.0766618690475065
AGIWUR12667;AGIWUR13199;AGIWUR17632;AGIWUR36997
CB4856 CT III 5173439 5173809 WBGene00019630 emb-1 K10D2.4 -0.120686064448838 AGIWUR2253
CB4856 CT III 5173922 5180507 WBGene00019629 cid-1 K10D2.3 0.0918445827883492 AGIWUR29111
CB4856 CT III 5185920 5188646 WBGene00019628 pup-2 K10D2.2 0.108296041836724
AGIWUR6106;AGIWUR24493;AGIWUR28776;AGIWUR33868;AGIWUR45004;AGIWUR45089
CB4856 CT III 5191131 5196028 WBGene00019627 K10D2.1 K10D2.1 -0.198325912603839
AGIWUR535;AGIWUR40874
CB4856 CT III 5196027 5196709 WBGene00019633 K10D2.7 K10D2.7 -0.0326407203053911
AGIWUR525;AGIWUR7607;AGIWUR14899
CB4856 CT III 5196678 5199688 WBGene00001262 emb-8 K10D2.6 0.00832432894537405
AGIWUR318;AGIWUR7707;AGIWUR14019;AGIWUR19026;AGIWUR23518;AGIWUR30421;AGIWUR38124
CB4856 CT III 5203713 5206911 WBGene00022531 morc-1 ZC155.3 -0.147688451908405 AGIWUR31982
CB4856 CT III 5207567 5209557 WBGene00022534 syx-16 ZC155.7 -0.093275893251783
AGIWUR5863;AGIWUR22835;AGIWUR32681
CB4856 CT III 5211781 5215512 WBGene00022532 ZC155.4 ZC155.4 -0.443927094362859 AGIWUR17968
CB4856 CT III 5215949 5217604 WBGene00022533 cutl-19 ZC155.5 -0.102970606635386 AGIWUR43291
CB4856 CT III 5219280 5220093 WBGene00022530 ZC155.2 ZC155.2 0.0398572675990154 AGIWUR24241
CB4856 CT III 5225621 5226801 WBGene00003588 nex-1 ZC155.1 -0.0668301872097947
AGIWUR20142;AGIWUR20359
CB4856 CT III 5226818 5227884 WBGene00016411 C34E10.10 C34E10.10 -0.269358778368256
AGIWUR29112
CB4856 CT III 5228417 5230536 WBGene00000229 atp-2 C34E10.6 -0.026208190098707
AGIWUR5070;AGIWUR12032;AGIWUR19582;AGIWUR21091;AGIWUR26182;AGIWUR37558
CB4856 CT III 5230555 5234530 WBGene00016408 prmt-5 C34E10.5 -0.179557510058624
AGIWUR31212;AGIWUR33990
CB4856 CT III 5234617 5236364 WBGene00016412 mrps-26 C34E10.11 -0.0750779926707708
AGIWUR42475
CB4856 CT III 5236410 5240055 WBGene00006946 prx-10 C34E10.4 -0.111318606459026
AGIWUR4880
CB4856 CT III 5240593 5242108 WBGene00000561 cnd-1 C34E10.7 0.495816258690501
AGIWUR4625
CB4856 CT III 5249075 5254187 WBGene00016409 sumv-1 C34E10.8 -0.268382612558629
AGIWUR6645
CB4856 CT III 5254438 5258922 WBGene00001660 gop-1 C34E10.3 -0.205472182567998
AGIWUR33981
CB4856 CT III 5259023 5260512 WBGene00001661 gop-2 C34E10.2 -0.00857267684516483
AGIWUR3830;AGIWUR7839;AGIWUR12289;AGIWUR26150
CB4856 CT III 5260693 5263837 WBGene00001662 gop-3 C34E10.1 -0.16612908516613

AGIWUR3380;AGIWUR23142;AGIWUR26880
CB4856 CT III 5263943 5264996 WBGene00001823 hap-1 ZC395.7 -0.333152640847334 AGIWUR27062
CB4856 CT III 5265113 5267204 WBGene00001740 gro-1 ZC395.6 -0.148907822314371 AGIWUR27294
CB4856 CT III 5267809 5268581 WBGene00022597 ZC395.5 ZC395.5 1.11155641595043 AGIWUR28768
CB4856 CT III 5268682 5273471 WBGene00022598 ztf-8 ZC395.8 -0.0519542064519438 AGIWUR13066
CB4856 CT III 5273800 5274787 WBGene00022599 ZC395.10 ZC395.10 -0.269946200134028
AGIWUR16003;AGIWUR22790
CB4856 CT III 5275225 5277797 WBGene00006590 toc-1 ZC395.3 -0.340590090456439 AGIWUR21186
CB4856 CT III 5277894 5279344 WBGene00000536 clk-1 ZC395.2 -0.0339998316960166 AGIWUR12724
CB4856 CT III 5286086 5286305 WBGene00022600 ZC395.11 ZC395.11 0.0416518694030019
AGIWUR24858
CB4856 CT III 5288180 5298361 WBGene00020530 T15B12.1 T15B12.1 0.138452989558525
AGIWUR295;AGIWUR35679
CB4856 CT III 5300008 5301886 WBGene00020531 T15B12.2 T15B12.2 -0.287198377295406
AGIWUR13248
CB4856 CT III 5302218 5306324 WBGene00018675 F52C9.5 F52C9.5 0.452772734831289 AGIWUR11007
CB4856 CT III 5308600 5311140 WBGene00003391 mog-3 F52C9.7 -0.136807875954219 AGIWUR40263
CB4856 CT III 5311246 5321732 WBGene00004095 pqe-1 F52C9.8 -0.0702619284435695
AGIWUR2071;AGIWUR19151;AGIWUR21084;AGIWUR30521;AGIWUR39453;AGIWUR40792;AGIWUR40889
CB4856 CT III 5321885 5326345 WBGene00018674 F52C9.3 F52C9.3 -0.322158544376226 AGIWUR18125
CB4856 CT III 5326623 5333620 WBGene00018672 sorf-2 F52C9.1 0.135679105825695
AGIWUR14541;AGIWUR27235;AGIWUR30565
CB4856 CT III 5341682 5344273 WBGene00022664 ZK121.2 ZK121.2 0.0526826040001982
AGIWUR10875;AGIWUR28689;AGIWUR36103
CB4856 CT III 5345252 5346117 WBGene00022663 glrx-21 ZK121.1 -0.217021825241808
AGIWUR4869;AGIWUR43468
CB4856 CT III 5345982 5349401 WBGene00021311 Y32H12A.2 Y32H12A.2 -0.260488995146073
AGIWUR28001
CB4856 CT III 5362209 5364372 WBGene00021310 Y32H12A.1 Y32H12A.1 0.288906759894997
AGIWUR5008
CB4856 CT III 5364512 5368045 WBGene00000973 dhs-9 Y32H12A.3 0.43795945815756
AGIWUR9949;AGIWUR33255
CB4856 CT III 5368967 5371274 WBGene00021312 szy-2 Y32H12A.4 0.0201487891844554
AGIWUR12268;AGIWUR40185
CB4856 CT III 5375698 5379929 WBGene00021313 paqr-2 Y32H12A.5 -0.000446652433035766
AGIWUR28786
CB4856 CT III 5380906 5382173 WBGene00021314 Y32H12A.6 Y32H12A.6
-0.00623447047234649 AGIWUR12120
CB4856 CT III 5382288 5385415 WBGene00021315 Y32H12A.7 Y32H12A.7 0.186246286326512

AGIWUR41756
CB4856 CT III 5385617 5406037 WBGene00021316 Y32H12A.8 Y32H12A.8 -0.0387723457696083
AGIWUR44435
CB4856 CT III 5406102 5407968 WBGene00020967 W03A5.2 W03A5.2 -0.519875561171458 AGIWUR4501
CB4856 CT III 5407888 5413177 WBGene00020966 W03A5.1 W03A5.1 -0.267883250677626 AGIWUR1467
CB4856 CT III 5413137 5413993 WBGene00001731 grl-22 W03A5.3 -0.275618557707012 AGIWUR36919
CB4856 CT III 5415556 5419565 WBGene00001042 dnj-24 W03A5.7 -0.0603737028027753
AGIWUR24399;AGIWUR33765
CB4856 CT III 5431407 5434467 WBGene00020968 W03A5.4 W03A5.4 -0.0788777353902645 AGIWUR14281
CB4856 CT III 5438826 5440632 WBGene00020970 W03A5.6 W03A5.6 -0.186869429321187
AGIWUR6394;AGIWUR38349
CB4856 CT III 5440862 5442761 WBGene00020779 T24G10.2 T24G10.2 0.147625367585984
AGIWUR29411
CB4856 CT III 5443167 5446506 WBGene00018612 disl-2 F48E8.6 -0.304742255926434
AGIWUR13480;AGIWUR16272;AGIWUR25335;AGIWUR32043
CB4856 CT III 5446510 5448419 WBGene00018613 skpt-1 F48E8.7 -0.0871882580537253 AGIWUR12645
CB4856 CT III 5448838 5449868 WBGene00044410 F48E8.8 F48E8.8 -0.0991343593368774
AGIWUR7055;AGIWUR24195;AGIWUR37290;AGIWUR42009
CB4856 CT III 5450106 5453137 WBGene00003901 paa-1 F48E8.5 0.032540365576267
AGIWUR21505;AGIWUR27982;AGIWUR29246
CB4856 CT III 5453590 5459516 WBGene00018611 F48E8.4 F48E8.4 -0.366790522210094
AGIWUR12928;AGIWUR13899;AGIWUR38555
CB4856 CT III 5461379 5464382 WBGene00018610 F48E8.3 F48E8.3 0.142095454887524
AGIWUR5788;AGIWUR6747;AGIWUR21003;AGIWUR34646;AGIWUR36155;AGIWUR36413;AGIWUR40162
CB4856 CT III 5464381 5467992 WBGene00018609 F48E8.2 F48E8.2 -0.137153987159756
AGIWUR16941;AGIWUR22846
CB4856 CT III 5474478 5478005 WBGene00003055 lon-1 F48E8.1 -0.420372899301409 AGIWUR36140
CB4856 CT III 5478682 5478924 WBGene00019835 R02F2.6 R02F2.6 -0.321517803173423
AGIWUR13517;AGIWUR30140
CB4856 CT III 5479318 5480278 WBGene00019834 R02F2.5 R02F2.5 -0.212069622944197 AGIWUR23390
CB4856 CT III 5480262 5483784 WBGene00019836 R02F2.7 R02F2.7 -0.314906416598491
AGIWUR16860;AGIWUR18493;AGIWUR20223;AGIWUR37735
CB4856 CT III 5484200 5486005 WBGene00019833 R02F2.4 R02F2.4 -0.0032378500946173 AGIWUR13548
CB4856 CT III 5487642 5498943 WBGene00019832 osg-1 R02F2.2 0.502098657405725 AGIWUR4638
CB4856 CT III 5499190 5499976 WBGene00019838 R02F2.9 R02F2.9 -0.178105142138111 AGIWUR37966
CB4856 CT III 5501861 5506392 WBGene00019831 R02F2.1 R02F2.1 0.337487660542351
AGIWUR11309;AGIWUR20593;AGIWUR33029;AGIWUR36550;AGIWUR37726;AGIWUR40000;AGIWUR40694
CB4856 CT III 5507050 5508738 WBGene00017032 D1044.7 D1044.7 0.132279980364773 AGIWUR24507
CB4856 CT III 5512927 5517523 WBGene00017033 nekl-4 D1044.8 -0.0990639613104625 AGIWUR2553

CB4856	CT	III	5524181	5524439	WBGene00017030	D1044.5	D1044.5	-0.0322838310874092	AGIWUR8736
CB4856	CT	III	5526071	5535481	WBGene00017028	dex-1	D1044.2	0.596404840669006	AGIWUR21827;AGIWUR23018;AGIWUR23267
CB4856	CT	III	5535660	5543218	WBGene00017031	D1044.6	D1044.6	-0.128437119988686	AGIWUR15083
CB4856	CT	III	5544304	5546287	WBGene00017027	D1044.1	D1044.1	-0.0390310234593656	AGIWUR40892
CB4856	CT	III	5546987	5551231	WBGene00017302	F09F7.5	F09F7.5	-0.0378360713536718	AGIWUR4529;AGIWUR6227;AGIWUR12534;AGIWUR18161;AGIWUR23373;AGIWUR25157;AGIWUR35306;AGIWUR40309
CB4856	CT	III	5551232	5552131	WBGene00017303	F09F7.6	F09F7.6	2.029032077614	AGIWUR225
CB4856	CT	III	5553489	5554934	WBGene00017304	F09F7.7	F09F7.7	0.644876706455944	AGIWUR5737;AGIWUR9245;AGIWUR35372;AGIWUR35725
CB4856	CT	III	5555404	5557318	WBGene00017301	hach-1	F09F7.4	-0.636700166282542	AGIWUR605;AGIWUR4972;AGIWUR14052;AGIWUR26931
CB4856	CT	III	5557344	5564837	WBGene00017300	rpc-2	F09F7.3	-0.0724154702822348	AGIWUR897;AGIWUR31854;AGIWUR34704;AGIWUR43566
CB4856	CT	III	5565025	5567382	WBGene00003371	mlc-3	F09F7.2	0.125372369903964	AGIWUR3571;AGIWUR7969;AGIWUR9563;AGIWUR9855;AGIWUR23139;AGIWUR26212;AGIWUR31710
CB4856	CT	III	5568213	5568779	WBGene00017305	nspb-12	F09F7.8	-0.13498965044927	AGIWUR36124
CB4856	CT	III	5573994	5576684	WBGene00017299	molo-1	F09F7.1	-0.104271465904897	AGIWUR5547
CB4856	CT	III	5578435	5581343	WBGene00018966	F56D2.5	F56D2.5	-0.303764677160615	AGIWUR36564
CB4856	CT	III	5581582	5582411	WBGene00018968	F56D2.8	F56D2.8	-0.272518403593867	AGIWUR43983
CB4856	CT	III	5583135	5584213	WBGene00006731	uev-2	F56D2.4	0.118825774362755	AGIWUR10384
CB4856	CT	III	5584687	5587110	WBGene00018965	F56D2.3	F56D2.3	0.632144283723009	AGIWUR32222
CB4856	CT	III	5587263	5589095	WBGene00018964	F56D2.2	F56D2.2	-0.175617600406019	AGIWUR12096;AGIWUR16038;AGIWUR18564
CB4856	CT	III	5589036	5591718	WBGene00018967	ddx-15	F56D2.6	0.016299083474216	AGIWUR1234;AGIWUR6210;AGIWUR11625;AGIWUR28848;AGIWUR44724
CB4856	CT	III	5592135	5594785	WBGene00018963	ucr-1	F56D2.1	-0.0803767852503645	AGIWUR396;AGIWUR17595;AGIWUR18618;AGIWUR33070;AGIWUR33417;AGIWUR39900
CB4856	CT	III	5595771	5598066	WBGene00000420	ced-6	F56D2.7	0.276963808146437	AGIWUR21338;AGIWUR38804
CB4856	CT	III	5597947	5600058	WBGene00015470	C05D2.10	C05D2.10	0.153224280458003	AGIWUR30330
CB4856	CT	III	5600393	5603656	WBGene00015468	madf-11	C05D2.6	-0.077140926340558	AGIWUR20578
CB4856	CT	III	5604460	5607549	WBGene00001514	xnd-1	C05D2.5	-0.136697773749578	AGIWUR12205
CB4856	CT	III	5607509	5610346	WBGene00015469	C05D2.8	C05D2.8	-0.157140546528661	AGIWUR22365
CB4856	CT	III	5612274	5614700	WBGene00000239	bas-1	C05D2.4	-0.50023421934406	AGIWUR11865;AGIWUR22234;AGIWUR25155
CB4856	CT	III	5614972	5617257	WBGene00015467	basl-1	C05D2.3	0.0806902724966871	AGIWUR5686;AGIWUR25855;AGIWUR41332;AGIWUR44004

CB4856	CT	III	5625014 5632322 WBGene00000900	daf-4	C05D2.1 0.0420462131991783
AGIWUR741;AGIWUR4499;AGIWUR13629;AGIWUR26026;AGIWUR28955
CB4856	CT	III	5635479 5654558 WBGene00000518	ckk-1	C05H8.1 0.0195809722472311
AGIWUR35270;AGIWUR44158
CB4856	CT	III	5654984 5656994 WBGene00018828	sdz-21	F54E7.5 0.387844660540403	AGIWUR8561
CB4856	CT	III	5659889 5661085 WBGene00018829	F54E7.6 F54E7.6 -0.216937166679628	AGIWUR2470
CB4856	CT	III	5664234 5676732 WBGene00003918	par-3	F54E7.3 0.0829825849510604
AGIWUR5765;AGIWUR39210
CB4856	CT	III	5676612 5677890 WBGene00018831	F54E7.9 F54E7.9 0.00150658048278274
AGIWUR7591;AGIWUR27414;AGIWUR31239;AGIWUR42631
CB4856	CT	III	5677953 5679038 WBGene00018830	ska-3	F54E7.8 -0.303681335270429	AGIWUR242
CB4856	CT	III	5679298 5680187 WBGene00004481	rps-12	F54E7.2 0.0596382181905959	AGIWUR34405
CB4856	CT	III	5680433 5682326 WBGene00018827	pst-2	F54E7.1 -0.220816144959532	AGIWUR2823
CB4856	CT	III	5682310 5684098 WBGene00004321	rcan-1	F54E7.7 0.399474516723636
AGIWUR2604;AGIWUR22461
CB4856	CT	III	5690107 5692740 WBGene00015146	abi-1	B0336.6 -0.0489826376529194	AGIWUR34075
CB4856	CT	III	5692896 5693938 WBGene00015145	B0336.5 B0336.5 -0.157742633889965	AGIWUR40695
CB4856	CT	III	5694036 5694784 WBGene00015150	B0336.13	B0336.13	-0.185048295862911
AGIWUR6621
CB4856	CT	III	5694735 5697366 WBGene00015147	B0336.7 B0336.7 -0.209890789895448
AGIWUR24198;AGIWUR34686
CB4856	CT	III	5697477 5698013 WBGene00002982	lgg-3	B0336.8 0.158406357792309	AGIWUR43374
CB4856	CT	III	5698430 5702304 WBGene00004348	rgs-5	B0336.4 -0.0292921168035605
AGIWUR7851;AGIWUR7993;AGIWUR20937
CB4856	CT	III	5702248 5705464 WBGene00006359	swp-1	B0336.9 -0.124836117083119
AGIWUR12277;AGIWUR15861;AGIWUR22510;AGIWUR29765;AGIWUR33611;AGIWUR35913;AGIWUR40245
CB4856	CT	III	5705521 5706129 WBGene00004435	rpl-23	B0336.10	-0.0614934149222428
AGIWUR5646;AGIWUR25265
CB4856	CT	III	5706718 5709329 WBGene00015148	hpo-28	B0336.11	-0.0497998424472708
AGIWUR204;AGIWUR5819;AGIWUR30382;AGIWUR44654
CB4856	CT	III	5710062 5710509 WBGene00015149	B0336.12	B0336.12	-0.0221560804241072
AGIWUR43192
CB4856	CT	III	5711548 5714846 WBGene00015143	B0336.3 B0336.3 0.0947683738587113	AGIWUR38024
CB4856	CT	III	5714861 5717585 WBGene00000182	arf-1.2 B0336.2 -0.147031126727751
AGIWUR6417;AGIWUR10593;AGIWUR23258;AGIWUR26259
CB4856	CT	III	5722824 5727115 WBGene00006943	wrm-1	B0336.1 0.042484640786105	AGIWUR16540
CB4856	CT	III	5728180 5730159 WBGene00015083	egg-1	B0244.8 -0.0917502127974608
AGIWUR973;AGIWUR2213;AGIWUR8633;AGIWUR12270;AGIWUR15368;AGIWUR28877;AGIWUR30186;AGIWUR35008;AGIWUR44144
CB4856	CT	III	5730118 5731030 WBGene00015084	B0244.9 B0244.9 -0.278066453322703	AGIWUR29575

CB4856	CT	III	5731501 5732854 WBGene00015082	B0244.7 B0244.7 0.101302821084131	AGIWUR27688
CB4856	CT	III	5733997 5737481 WBGene00015085	B0244.10	B0244.10	0.0215465877213585
AGIWUR3640;AGIWUR11691;AGIWUR22636
CB4856	CT	III	5742556 5744820 WBGene00206513	B0244.15	B0244.15	0.0373901552389952
AGIWUR27006
CB4856	CT	III	5744870 5747195 WBGene00015080	B0244.5 B0244.5 -0.0144276789201694	AGIWUR35844
CB4856	CT	III	5747485 5751637 WBGene00015079	B0244.4 B0244.4 -0.685564059497652	AGIWUR28304
CB4856	CT	III	5755496 5765815 WBGene00002048	ida-1	B0244.2 0.40913676039786	AGIWUR14657
CB4856	CT	III	5767882 5768665 WBGene00015086	B0244.11	B0244.11	0.122548436478708
AGIWUR6678
CB4856	CT	III	5770185 5774703 WBGene00021343	cutl-23 Y37B11A.1	0.673721788633871
AGIWUR41663
CB4856	CT	III	5774679 5790707 WBGene00021344	Y37B11A.2	Y37B11A.2	0.464172100872347
AGIWUR10288
CB4856	CT	III	5791171 5803042 WBGene00020964	polq-1	W03A3.2 -0.0819814129581697
AGIWUR5084;AGIWUR19096
CB4856	CT	III	5803260 5805112 WBGene00001869	him-10	R12B2.4 -0.105058083982549	AGIWUR38298
CB4856	CT	III	5805563 5806975 WBGene00020019	R12B2.3 R12B2.3 -0.0263723979182267	AGIWUR18560
CB4856	CT	III	5807052 5809206 WBGene00020018	R12B2.2 R12B2.2 0.0742547556434729	AGIWUR31538
CB4856	CT	III	5815045 5815513 WBGene00020021	R12B2.7 R12B2.7 -0.035740689614693	AGIWUR42938
CB4856	CT	III	5816203 5820200 WBGene00004858	sma-4	R12B2.1 0.334795061072894
AGIWUR15423;AGIWUR34046;AGIWUR34858;AGIWUR38559
CB4856	CT	III	5823957 5828651 WBGene00044411	R12B2.8 R12B2.8 0.218817032839056
AGIWUR7783;AGIWUR23563;AGIWUR28200;AGIWUR28780;AGIWUR28810
CB4856	CT	III	5828603 5833745 WBGene00007016	mdt-15	R12B2.5 0.2161346109196 AGIWUR4471
CB4856	CT	III	5834022 5835756 WBGene00020020	R12B2.6 R12B2.6 0.0200374248211759	AGIWUR33024
CB4856	CT	III	5836926 5837183 WBGene00021029	W04D12.1	W04D12.1	0.0642247444060926
AGIWUR33001
CB4856	CT	III	5848838 5853269 WBGene00017162	ddx-23	F01F1.7 -0.100835453567932	AGIWUR17455
CB4856	CT	III	5853532 5855687 WBGene00000381	cct-6	F01F1.8 -0.205185790733379
AGIWUR6023;AGIWUR22509;AGIWUR24670;AGIWUR31718;AGIWUR33596;AGIWUR43085
CB4856	CT	III	5856018 5859186 WBGene00000115	alh-9	F01F1.6 -0.245318552724003
AGIWUR7791;AGIWUR17410;AGIWUR18827;AGIWUR41022;AGIWUR44087
CB4856	CT	III	5859308 5861907 WBGene00001057	dpf-4	F01F1.5 0.157100660954843
AGIWUR11864;AGIWUR27021;AGIWUR45153
CB4856	CT	III	5861855 5863546 WBGene00017163	dnpp-1	F01F1.9 -0.0671142923504924
AGIWUR4140;AGIWUR30765;AGIWUR43330
CB4856	CT	III	5864500 5866541 WBGene00017161	rabn-5	F01F1.4 0	AGIWUR7226
CB4856	CT	III	5866495 5868501 WBGene00017164	eng-1	F01F1.10	-0.0415189907247733

AGIWUR8283
CB4856 CT III 5868587 5869620 WBGene00017169 F01F1.15 F01F1.15
-0.00645301622409633 AGIWUR26321;AGIWUR33269
CB4856 CT III 5869685 5872396 WBGene00017165 F01F1.11 F01F1.11 -0.0602213063860297
AGIWUR12560
CB4856 CT III 5873025 5874680 WBGene00017160 F01F1.3 F01F1.3 0.253569530287759 AGIWUR13608
CB4856 CT III 5874672 5876625 WBGene00017166 aldo-2 F01F1.12 0.0665714903497596
AGIWUR12260;AGIWUR18058;AGIWUR25115;AGIWUR25603;AGIWUR31691;AGIWUR40498
CB4856 CT III 5877020 5878419 WBGene00003252 mig-21 F01F1.13 -0.138606163605514
AGIWUR44500
CB4856 CT III 5878521 5879415 WBGene00017159 F01F1.2 F01F1.2 -0.0316533914861736
AGIWUR400;AGIWUR17259;AGIWUR18227;AGIWUR28252;AGIWUR33591;AGIWUR38436
CB4856 CT III 5879489 5882428 WBGene00017158 hpo-10 F01F1.1 -0.17850964867524
AGIWUR1028;AGIWUR18342;AGIWUR23790
CB4856 CT III 5882319 5882923 WBGene00017168 F01F1.14 F01F1.14 0.129990472237127
AGIWUR11771
CB4856 CT III 5884307 5885848 WBGene00016196 C28H8.5 C28H8.5 -0.354679386525973 AGIWUR19907
CB4856 CT III 5885847 5886956 WBGene00016198 C28H8.7 C28H8.7 0.157510062872126 AGIWUR7242
CB4856 CT III 5887104 5888775 WBGene00016199 C28H8.8 C28H8.8 0.120493963112355 AGIWUR21123
CB4856 CT III 5889721 5896013 WBGene00016197 pxl-1 C28H8.6 -0.0935880414120085
AGIWUR2647;AGIWUR6131;AGIWUR10300;AGIWUR12567;AGIWUR13793;AGIWUR15805;AGIWUR18173;AGIWUR21049;AGIWUR24758;A
GIWUR30067;AGIWUR33589;AGIWUR42592
CB4856 CT III 5903576 5906122 WBGene00016200 dpff-1 C28H8.9 0.118687883262805
AGIWUR5400;AGIWUR12108;AGIWUR23801
CB4856 CT III 5907044 5908457 WBGene00016195 C28H8.4 C28H8.4 -0.197952486763201
AGIWUR5053;AGIWUR10268;AGIWUR28529
CB4856 CT III 5908700 5914734 WBGene00016194 C28H8.3 C28H8.3 -0.0649394012988873
AGIWUR4907;AGIWUR5521;AGIWUR5753;AGIWUR8116;AGIWUR15977;AGIWUR18776;AGIWUR38328;AGIWUR38602;AGIWUR41317;AGI
WUR44843
CB4856 CT III 5917628 5920086 WBGene00016201 tdo-2 C28H8.11 -0.218665017671864
AGIWUR189;AGIWUR9055;AGIWUR37615;AGIWUR44015
CB4856 CT III 5923187 5924431 WBGene00016193 C28H8.2 C28H8.2 0.117100863757397 AGIWUR33140
CB4856 CT III 5924394 5925754 WBGene00001018 dnc-2 C28H8.12 -0.0569902098999258
AGIWUR7289;AGIWUR28714;AGIWUR32259
CB4856 CT III 5926086 5926987 WBGene00016192 bcl-7 C28H8.1 -0.0963321905817036 AGIWUR14967
CB4856 CT III 5926898 5932953 WBGene00006987 zmp-1 EGAP1.3 -0.0867493884140802
AGIWUR7925;AGIWUR17769
CB4856 CT III 5935749 5937220 WBGene00017146 EGAP1.1 EGAP1.1 0.412606466099057 AGIWUR25306
CB4856 CT III 5940244 5943835 WBGene00006727 ubq-1 F25B5.4 0.0397828343142166

AGIWUR8687;AGIWUR12178;AGIWUR21911;AGIWUR22522;AGIWUR22721;AGIWUR23032;AGIWUR27981;AGIWUR32258;AGIWUR40838
CB4856 CT III 5943833 5946124 WBGene00017776 F25B5.5 F25B5.5 -0.338035699618452 AGIWUR17556
CB4856 CT III 5946217 5948711 WBGene00017777 F25B5.6 F25B5.6 -0.0545287960051919
AGIWUR4867;AGIWUR36391;AGIWUR42299
CB4856 CT III 5948890 5953534 WBGene00017775 F25B5.3 F25B5.3 -0.176781050924423
AGIWUR1552;AGIWUR4298;AGIWUR15197;AGIWUR41985
CB4856 CT III 5953526 5958246 WBGene00017778 nono-1 F25B5.7 -0.182663966885217
AGIWUR5396;AGIWUR8117;AGIWUR13268;AGIWUR16611;AGIWUR18055;AGIWUR20561;AGIWUR37674;AGIWUR38427
CB4856 CT III 5958722 5962322 WBGene00017774 nop-1 F25B5.2 -0.0624539076392754 AGIWUR31282
CB4856 CT III 5966413 5971510 WBGene00017773 dct-6 F25B5.1 -0.555180831011427 AGIWUR14598
CB4856 CT III 5971530 5973664 WBGene00003375 mlp-1 T04C9.4 -0.487869665438068
AGIWUR33014;AGIWUR37421
CB4856 CT III 5976781 5982126 WBGene00001489 frm-2 T04C9.6 0.362186257952995 AGIWUR3828
CB4856 CT III 5985640 5985858 WBGene00020211 T04C9.3 T04C9.3 0.239105686145749 AGIWUR38664
CB4856 CT III 5991880 5992230 WBGene00020210 T04C9.2 T04C9.2 -0.158953794159043 AGIWUR36256
CB4856 CT III 5993219 6006360 WBGene00020209 T04C9.1 T04C9.1 0.109929552964468
AGIWUR11458;AGIWUR16358;AGIWUR30105;AGIWUR35380;AGIWUR39302;AGIWUR42067
CB4856 CT III 6006344 6009084 WBGene00022694 gcna-1 ZK328.4 -0.220690631326754 AGIWUR18288
CB4856 CT III 6009550 6012839 WBGene00001166 eftu-2 ZK328.2 -0.0792798883407428
AGIWUR1543;AGIWUR1621;AGIWUR23124;AGIWUR33224;AGIWUR33784;AGIWUR39115;AGIWUR42275
CB4856 CT III 6013536 6019562 WBGene00003796 npp-10 ZK328.5 -0.146828311929048
AGIWUR17451;AGIWUR18497;AGIWUR19359;AGIWUR19986;AGIWUR31346;AGIWUR35634;AGIWUR38716
CB4856 CT III 6020165 6024659 WBGene00000874 cyk-3 ZK328.1 -0.258855958157956
AGIWUR587;AGIWUR7409
CB4856 CT III 6025090 6028532 WBGene00006544 tbx-7 ZK328.8 0.400988847441734 AGIWUR35350
CB4856 CT III 6028790 6030583 WBGene00022695 ZK328.6 ZK328.6 0.117357269786238 AGIWUR13455
CB4856 CT III 6032696 6039475 WBGene00022696 ZK328.7 ZK328.7 -0.0142645947324191
AGIWUR21256;AGIWUR23219
CB4856 CT III 6040334 6045125 WBGene00018254 F40H6.2 F40H6.2 0.13447247342131 AGIWUR42043
CB4856 CT III 6045771 6046981 WBGene00006547 tbx-11 F40H6.4 0.216746258412556
AGIWUR31993;AGIWUR39421
CB4856 CT III 6049316 6055448 WBGene00018255 F40H6.5 F40H6.5 0.112087945208404
AGIWUR16778;AGIWUR34353
CB4856 CT III 6057055 6058444 WBGene00018253 F40H6.1 F40H6.1 0.0702061418150741 AGIWUR16150
CB4856 CT III 6065139 6066217 WBGene00044412 F40H6.6 F40H6.6 0.262772622357009 AGIWUR6163
CB4856 CT III 6067857 6072076 WBGene00015478 C05D10.2 C05D10.2 -0.0969849762646513
AGIWUR106;AGIWUR11939;AGIWUR23889;AGIWUR29361;AGIWUR43021
CB4856 CT III 6080563 6084748 WBGene00015477 attf-4 C05D10.1 0.124482906013192
AGIWUR35938

CB4856	CT	III	6084665 6087721 WBGene00015479	wht-1	C05D10.3	0.00811878630754137
AGIWUR18060
CB4856	CT	III	6088684 6102862 WBGene00015480	C05D10.4	C05D10.4	0.154007893583375
AGIWUR2123;AGIWUR4314;AGIWUR4703;AGIWUR8809;AGIWUR10254;AGIWUR27781;AGIWUR42360
CB4856	CT	III	6114482 6116331 WBGene00020549	nmt-1	T17E9.2 0.0389012709459795
AGIWUR27138;AGIWUR31541;AGIWUR41341
CB4856	CT	III	6118627 6124055 WBGene00002201	kin-18	T17E9.1 0.00234100274204518
AGIWUR1170;AGIWUR5256
CB4856	CT	III	6124184 6136276 WBGene00021534	mvk-1	Y42G9A.4	-0.0677125738862667
AGIWUR12699;AGIWUR40028
CB4856	CT	III	6142123 6149590 WBGene00021535	wht-7	Y42G9A.6	0.00694828976784656
AGIWUR12629
CB4856	CT	III	6149961 6154933 WBGene00021531	Y42G9A.1	Y42G9A.1	0.284927301667857
AGIWUR36020
CB4856	CT	III	6154844 6157758 WBGene00018340	anmt-2	F42A10.3	-0.00847987240529946
AGIWUR34997
CB4856	CT	III	6159842 6164588 WBGene00003593	nfm-1	F42A10.2	0.232408831271
AGIWUR1316;AGIWUR1497;AGIWUR7359;AGIWUR41587
CB4856	CT	III	6164645 6171165 WBGene00023489	F42A10.9	F42A10.9	-0.646581703124987
AGIWUR19772;AGIWUR40858
CB4856	CT	III	6171147 6172800 WBGene00018341	F42A10.5	F42A10.5	-0.400907614357701
AGIWUR24355;AGIWUR30556
CB4856	CT	III	6173305 6176171 WBGene00018339	abcf-3	F42A10.1	0.0157180046903935
AGIWUR32812
CB4856	CT	III	6176148 6176861 WBGene00018342	F42A10.6	F42A10.6	-0.224533560804758
AGIWUR14546
CB4856	CT	III	6177916 6178686 WBGene00018343	F42A10.7	F42A10.7	0.0556879385395836
AGIWUR20743;AGIWUR44549
CB4856	CT	III	6181078 6183218 WBGene00003546	nas-28	F42A10.8	0.203068673850265
AGIWUR10042;AGIWUR38272
CB4856	CT	III	6191110 6192081 WBGene00016012	C23G10.5	C23G10.5
-0.00384337605488039	AGIWUR42032
CB4856	CT	III	6192116 6194580 WBGene00016013	C23G10.6	C23G10.6	1.2842315163522
AGIWUR42473
CB4856	CT	III	6195984 6198414 WBGene00016014	C23G10.7	C23G10.7	-0.283075390568312
AGIWUR1598;AGIWUR10377;AGIWUR17777;AGIWUR19282
CB4856	CT	III	6198902 6202594 WBGene00004459	rpn-2	C23G10.4	-0.409495591971142
AGIWUR2174;AGIWUR13729;AGIWUR23688;AGIWUR24196;AGIWUR24746;AGIWUR38535;AGIWUR43344
CB4856	CT	III	6202619 6206211 WBGene00016015	C23G10.8	C23G10.8	0.0833976461015169

AGIWUR39422
CB4856 CT III 6206803 6207971 WBGene00004472 rps-3 C23G10.3 0.00993160876055299
AGIWUR7661;AGIWUR40667
CB4856 CT III 6208064 6209140 WBGene00016011 C23G10.2 C23G10.2 -0.0572078697762102
AGIWUR31032
CB4856 CT III 6209284 6212904 WBGene00016010 C23G10.1 C23G10.1 -0.320991347602228
AGIWUR19899
CB4856 CT III 6213112 6213923 WBGene00016016 C23G10.10 C23G10.10 1.24585087760602
AGIWUR15153
CB4856 CT III 6215164 6215576 WBGene00016017 C23G10.11 C23G10.11 5.80553719785489
AGIWUR44734
CB4856 CT III 6220537 6224451 WBGene00001989 hot-4 T12A2.16 0.28208529257042
AGIWUR4554;AGIWUR11038;AGIWUR14225;AGIWUR34548;AGIWUR41379
CB4856 CT III 6224491 6229403 WBGene00020443 esyt-2 T12A2.15 -0.275637652922914
AGIWUR11091;AGIWUR17234;AGIWUR18813;AGIWUR22069;AGIWUR27544
CB4856 CT III 6233718 6239336 WBGene00020439 T12A2.5 T12A2.5 -0.159730249569995 AGIWUR41851
CB4856 CT III 6240775 6241581 WBGene00020438 T12A2.3 T12A2.3 -0.256703718275206
AGIWUR17752;AGIWUR29138;AGIWUR37203;AGIWUR43278
CB4856 CT III 6241850 6244383 WBGene00020440 T12A2.6 T12A2.6 0.0612756923203674
AGIWUR10318;AGIWUR21837;AGIWUR24481
CB4856 CT III 6244672 6246039 WBGene00020441 T12A2.7 T12A2.7 -0.29857655772711 AGIWUR27093
CB4856 CT III 6245932 6248234 WBGene00020442 gen-1 T12A2.8 -0.0678020802716937
AGIWUR5759;AGIWUR14650;AGIWUR23445
CB4856 CT III 6248317 6251607 WBGene00020437 stt-3 T12A2.2 -0.024999336688142
AGIWUR3513;AGIWUR9746;AGIWUR13447;AGIWUR19992;AGIWUR22561;AGIWUR28326;AGIWUR35902;AGIWUR38028;AGIWUR39066;A
GIWUR41574
CB4856 CT III 6251811 6271282 WBGene00020436 T12A2.1 T12A2.1 -0.36156589522659 AGIWUR4320
CB4856 CT III 6271561 6272959 WBGene00015979 C18F10.9 C18F10.9 -0.268102255286175
AGIWUR9386
CB4856 CT III 6273160 6276016 WBGene00015977 C18F10.2 C18F10.2 0.332084587924762
AGIWUR41470
CB4856 CT III 6276048 6278351 WBGene00015978 C18F10.7 C18F10.7 -0.084258371120054
AGIWUR5641;AGIWUR11223;AGIWUR19375;AGIWUR26373;AGIWUR27221;AGIWUR27295;AGIWUR29666;AGIWUR29857;AGIWUR30170;
AGIWUR32153;AGIWUR36457;AGIWUR40713
CB4856 CT III 6280380 6282411 WBGene00001972 hmg-1.2 F47D12.4 -0.0336774908063742
AGIWUR26400;AGIWUR26979
CB4856 CT III 6282444 6286231 WBGene00018561 gadr-3 F47D12.5 -0.0237683427843702
AGIWUR26317
CB4856 CT III 6286629 6287293 WBGene00018562 F47D12.6 F47D12.6 0.153613531431118

AGIWUR17571
CB4856	CT	III	6293247 6296022 WBGene00018563	F47D12.7	F47D12.7	0.0416987508314719
AGIWUR28697
CB4856	CT	III	6298461 6299338 WBGene00018560	F47D12.3	F47D12.3	-0.234709154922552
AGIWUR29555
CB4856	CT	III	6299331 6303217 WBGene00018564	F47D12.9	F47D12.9	-0.162985121109861
AGIWUR6888;AGIWUR10228;AGIWUR20703;AGIWUR25146;AGIWUR30129;AGIWUR30161
CB4856	CT	III	6309378 6320872 WBGene00001518	gar-2	F47D12.1	-0.0584175481409233
AGIWUR3654;AGIWUR4396;AGIWUR18671;AGIWUR18967;AGIWUR29649;AGIWUR38247;AGIWUR38822
CB4856	CT	III	6321176 6324239 WBGene00016980	C56G2.5 C56G2.5 -0.263018049665663
AGIWUR2514;AGIWUR14213;AGIWUR15494;AGIWUR37144;AGIWUR40562;AGIWUR43714
CB4856	CT	III	6331732 6335829 WBGene00016979	C56G2.4 C56G2.4 0.316038054977216	AGIWUR28406
CB4856	CT	III	6338508 6340228 WBGene00002891	let-767 C56G2.6 -0.0958557829654225
AGIWUR23719;AGIWUR39408
CB4856	CT	III	6342481 6343287 WBGene00016982	C56G2.9 C56G2.9 -0.0622162914894	AGIWUR27081
CB4856	CT	III	6346285 6348121 WBGene00016978	C56G2.3 C56G2.3 -0.433487785064989	AGIWUR7248
CB4856	CT	III	6353384 6366660 WBGene00016977	akap-1	C56G2.1 0.0873854675206531
AGIWUR630;AGIWUR2907;AGIWUR8486;AGIWUR8983;AGIWUR15005;AGIWUR20081;AGIWUR29898;AGIWUR38741
CB4856	CT	III	6366582 6367899 WBGene00016983	C56G2.15	C56G2.15	-0.0787625721918577
AGIWUR37593
CB4856	CT	III	6368042 6373904 WBGene00015813	thoc-2	C16A3.8 0.131559344145197	AGIWUR35223
CB4856	CT	III	6374048 6374997 WBGene00004482	rps-13	C16A3.9 0.0291373512532793
AGIWUR19240;AGIWUR42951
CB4856	CT	III	6375379 6379190 WBGene00007048	nfx-1	C16A3.7 0.0081546420696057	AGIWUR32264
CB4856	CT	III	6379449 6380671 WBGene00015811	C16A3.6 C16A3.6 0.399179136436224
AGIWUR10740;AGIWUR41642
CB4856	CT	III	6380695 6381517 WBGene00015810	C16A3.5 C16A3.5 -0.0874310231377642	AGIWUR18873
CB4856	CT	III	6381634 6383024 WBGene00015809	C16A3.4 C16A3.4 -0.0640041599671406	AGIWUR8037
CB4856	CT	III	6383552 6389401 WBGene00002850	let-716 C16A3.3 -0.117126265510308	AGIWUR5062
CB4856	CT	III	6389833 6390841 WBGene00015807	C16A3.2 C16A3.2 -0.361503006994864	AGIWUR37881
CB4856	CT	III	6390946 6393843 WBGene00015806	smrc-1	C16A3.1 0.0404785670444312
AGIWUR10053;AGIWUR20541;AGIWUR34378
CB4856	CT	III	6394223 6396182 WBGene00015814	oatr-1	C16A3.10	-0.304453901643893
AGIWUR29129;AGIWUR41871;AGIWUR42636
CB4856	CT	III	6407129 6410018 WBGene00002881	let-756 C05D11.4	0.0647342256411809
AGIWUR36676;AGIWUR40092;AGIWUR44667
CB4856	CT	III	6410033 6412014 WBGene00015483	C05D11.5	C05D11.5	0.35544737871512
AGIWUR25683;AGIWUR32018;AGIWUR33469;AGIWUR36116
CB4856	CT	III	6411816 6414993 WBGene00003523	nas-4	C05D11.6	-0.0891223610968837

AGIWUR26546;AGIWUR27915;AGIWUR35771
CB4856 CT III 6415774 6421885 WBGene00015484 atgl-1 C05D11.7 0.138979862843456
AGIWUR16740;AGIWUR21361;AGIWUR28752
CB4856 CT III 6422459 6427305 WBGene00015485 C05D11.8 C05D11.8 -0.0456223914543905
AGIWUR13966
CB4856 CT III 6427529 6430934 WBGene00015486 C05D11.9 C05D11.9 -0.175017408739006
AGIWUR7701
CB4856 CT III 6431153 6431792 WBGene00015487 mrps-17 C05D11.10 -0.247550249351793
AGIWUR30900
CB4856 CT III 6432178 6433145 WBGene00006515 txdc-9 C05D11.3 -0.598115518774463
AGIWUR13727
CB4856 CT III 6433239 6436502 WBGene00006516 vps-16 C05D11.2 -0.103414497644959
AGIWUR12562
CB4856 CT III 6436608 6439959 WBGene00015481 C05D11.1 C05D11.1 -0.0514946155383944
AGIWUR20542;AGIWUR28535
CB4856 CT III 6440145 6443000 WBGene00003214 mel-32 C05D11.11 -0.408631881927219
AGIWUR4122;AGIWUR20891;AGIWUR29394;AGIWUR41655
CB4856 CT III 6443044 6444079 WBGene00015488 C05D11.13 C05D11.13 0.105290051401585
AGIWUR6024
CB4856 CT III 6444693 6446943 WBGene00002855 let-721 C05D11.12 0.0668636721078002
AGIWUR6107;AGIWUR19355;AGIWUR41545
CB4856 CT III 6448075 6450696 WBGene00020822 T26A5.6 T26A5.6 -0.225522079292983 AGIWUR16995
CB4856 CT III 6454483 6460014 WBGene00020821 jhdm-1 T26A5.5 -0.195375166791113
AGIWUR22295;AGIWUR37371
CB4856 CT III 6460120 6462222 WBGene00020820 algn-1 T26A5.4 -0.0940286906554082 AGIWUR10349
CB4856 CT III 6462216 6462856 WBGene00020823 T26A5.8 T26A5.8 -0.156164813200617 AGIWUR29912
CB4856 CT III 6462907 6463801 WBGene00001005 dlc-1 T26A5.9 -0.235791606582657
AGIWUR2065;AGIWUR7456;AGIWUR14904;AGIWUR27849
CB4856 CT III 6465157 6467208 WBGene00006463 nduf-2.2 T26A5.3 -0.28086414925497
AGIWUR33657
CB4856 CT III 6467382 6471510 WBGene00020819 T26A5.2 T26A5.2 0.498402191005264 AGIWUR11165
CB4856 CT III 6471720 6475246 WBGene00007060 wht-6 T26A5.1 0.199833383462103 AGIWUR31046
CB4856 CT III 6478961 6481226 WBGene00017755 zip-8 F23F12.9 0.678891361167593
AGIWUR18229;AGIWUR25746
CB4856 CT III 6483218 6488875 WBGene00017754 F23F12.8 F23F12.8 0.441327832790623
AGIWUR7260;AGIWUR7807;AGIWUR20366
CB4856 CT III 6489793 6491282 WBGene00004503 rpt-3 F23F12.6 -0.112526361452044
AGIWUR5122;AGIWUR30499;AGIWUR41391
CB4856 CT III 6491368 6492907 WBGene00005076 srb-11 F23F12.10 0.0931547028516881

AGIWUR30246
CB4856 CT III 6493553 6494781 WBGene00005075 srb-10 F23F12.11 -0.18310322765671
AGIWUR24014
CB4856 CT III 6495677 6498198 WBGene00044455 F23F12.13 F23F12.13 0.405789683300342
AGIWUR33864;AGIWUR45117
CB4856 CT III 6498476 6500023 WBGene00017752 sdz-15 F23F12.4 -0.231209061780169
AGIWUR867;AGIWUR22937;AGIWUR25799
CB4856 CT III 6500514 6507717 WBGene00017751 F23F12.3 F23F12.3 -0.499891500560967
AGIWUR12746;AGIWUR22390;AGIWUR33264;AGIWUR43307
CB4856 CT III 6508132 6509499 WBGene00017756 F23F12.12 F23F12.12 0.363925623104489
AGIWUR24827
CB4856 CT III 6509699 6514768 WBGene00000044 acr-5 K03F8.2 -0.0404687143167184
AGIWUR945;AGIWUR2385;AGIWUR14698;AGIWUR31814
CB4856 CT III 6518714 6520049 WBGene00044295 C09E7.10 C09E7.10 -0.0371334793799694
AGIWUR9196;AGIWUR10502;AGIWUR25243
CB4856 CT III 6524051 6525660 WBGene00003859 oig-1 C09E7.3 -0.0725078015342122 AGIWUR44575
CB4856 CT III 6528202 6532088 WBGene00004102 pqn-10 C09E7.2 0.0265952876476128 AGIWUR41366
CB4856 CT III 6532560 6533819 WBGene00015637 srxa-5 C09E7.1 -0.168593901209842 AGIWUR26008
CB4856 CT III 6534467 6536995 WBGene00015638 C09E7.4 C09E7.4 0.11302691458037 AGIWUR8849
CB4856 CT III 6538101 6541312 WBGene00015639 C09E7.5 C09E7.5 0.0718362660475265 AGIWUR9379
CB4856 CT III 6541550 6546425 WBGene00015640 C09E7.6 C09E7.6 -0.210560171343311
AGIWUR1061;AGIWUR30995
CB4856 CT III 6553575 6558575 WBGene00015642 C09E7.8 C09E7.8 -0.343537426181033 AGIWUR2078
CB4856 CT III 6559097 6563666 WBGene00017849 F27B3.7 F27B3.7 -0.327402054988543 AGIWUR40502
CB4856 CT III 6577827 6578584 WBGene00017848 F27B3.6 F27B3.6 -0.256692259333217 AGIWUR29969
CB4856 CT III 6579698 6579877 WBGene00044454 F27B3.8 F27B3.8 0.299900881870606 AGIWUR13094
CB4856 CT III 6583461 6585617 WBGene00000060 acr-21 F27B3.2 0.2894746407021 AGIWUR28720
CB4856 CT III 6587864 6589398 WBGene00017648 ddo-3 F20H11.5 0.138382316692431
AGIWUR24363;AGIWUR41686
CB4856 CT III 6589377 6592175 WBGene00017646 F20H11.1 F20H11.1 0.0927820409275419
AGIWUR29171
CB4856 CT III 6592567 6601171 WBGene00002889 let-765 F20H11.2 -0.232526890097155
AGIWUR17640;AGIWUR25140;AGIWUR27253;AGIWUR43209
CB4856 CT III 6606247 6608106 WBGene00003162 mdh-2 F20H11.3 0.0454892282654491
AGIWUR5742;AGIWUR9105;AGIWUR11499;AGIWUR32191;AGIWUR32225;AGIWUR43317
CB4856 CT III 6608622 6610455 WBGene00017647 F20H11.4 F20H11.4 0.13852114909033
AGIWUR25769
CB4856 CT III 6610822 6612263 WBGene00005263 srh-40 Y40D12A.3 -0.0612630420263294
AGIWUR28492

CB4856	CT	III	6612516	6615134	WBGene00021503	ctsa-2	Y40D12A.2	0.207829899524281	AGIWUR11341
CB4856	CT	III	6615414	6620051	WBGene00021502	Y40D12A.1	Y40D12A.1	-0.0355773273895927	AGIWUR14140;AGIWUR35368;AGIWUR38777
CB4856	CT	III	6620254	6622265	WBGene00015733	C13B9.2	C13B9.2	0.104224465250422	AGIWUR32233
CB4856	CT	III	6622676	6624972	WBGene00015734	copd-1	C13B9.3	-0.110898895594767	AGIWUR24038
CB4856	CT	III	6632841	6652083	WBGene00015735	pdfr-1	C13B9.4	0.0260649681361302	AGIWUR2877;AGIWUR3657;AGIWUR9278;AGIWUR12756;AGIWUR39212
CB4856	CT	III	6664960	6665374	WBGene00019714	M01G4.1	M01G4.1	0.365932328486666	AGIWUR17124
CB4856	CT	III	6675801	6703974	WBGene00004316	rbf-1	F37A4.7	0.197036310211272	AGIWUR809;AGIWUR10220;AGIWUR21533;AGIWUR33340;AGIWUR35132
CB4856	CT	III	6703993	6710084	WBGene00002169	isw-1	F37A4.8	-0.0706473469211919	AGIWUR21624;AGIWUR25898;AGIWUR35837;AGIWUR38143
CB4856	CT	III	6711869	6713191	WBGene00018135	F37A4.5	F37A4.5	0.0705588444048324	AGIWUR10785
CB4856	CT	III	6713583	6718022	WBGene00018134	F37A4.4	F37A4.4	0.24850592927652	AGIWUR38765
CB4856	CT	III	6718570	6720487	WBGene00018133	F37A4.3	F37A4.3	0.144932570082862	AGIWUR28310
CB4856	CT	III	6720415	6722258	WBGene00018137	bath-41	F37A4.9	-0.213211184672058	AGIWUR38646
CB4856	CT	III	6722454	6723284	WBGene00018132	F37A4.2	F37A4.2	-0.164461471933999	AGIWUR15914
CB4856	CT	III	6723389	6725711	WBGene00018131	F37A4.1	F37A4.1	0.0302486341103041	AGIWUR31813
CB4856	CT	III	6725789	6728058	WBGene00022420	wdr-4	Y102E9.2	-0.12925627482015	AGIWUR29143
CB4856	CT	III	6728155	6730170	WBGene00044452	Y102E9.5	Y102E9.5	-0.289064084145547	AGIWUR3681;AGIWUR14452
CB4856	CT	III	6730781	6733935	WBGene00003851	odr-4	Y102E9.1	-0.173582003637573	AGIWUR7032;AGIWUR24539;AGIWUR26097;AGIWUR33738
CB4856	CT	III	6733992	6734520	WBGene00022421	Y102E9.3	Y102E9.3	0.156110104293129	AGIWUR36461
CB4856	CT	III	6735968	6736642	WBGene00044453	Y102E9.6	Y102E9.6	0.0691921314746295	AGIWUR2619;AGIWUR10795;AGIWUR16286;AGIWUR21903;AGIWUR28604
CB4856	CT	III	6737271	6737783	WBGene00019501	K07E12.2	K07E12.2	0.0212883376785129	AGIWUR36836
CB4856	CT	III	6746308	6794764	WBGene00000998	dig-1	K07E12.1	0.116982719778113	AGIWUR27360
CB4856	CT	III	6795626	6797774	WBGene00019907	R05H11.1	R05H11.1	-0.296064665095841	AGIWUR12904;AGIWUR43298
CB4856	CT	III	6798299	6800489	WBGene00019908	R05H11.2	R05H11.2	0.0887305510907508	AGIWUR9915
CB4856	CT	III	6802788	6816132	WBGene00017904	lim-8	F28F5.3	0.205895419631497	AGIWUR16870;AGIWUR44041

CB4856	CT	III	6816643 6816880 WBGene00043297	F28F5.4 F28F5.4 0.244209691732924
AGIWUR16112;AGIWUR20630;AGIWUR25870;AGIWUR27417;AGIWUR29321;AGIWUR35286
CB4856	CT	III	6817316 6819372 WBGene00017905	F28F5.6 F28F5.6 -0.152515191383728	AGIWUR24831
CB4856	CT	III	6820935 6847283 WBGene00006498	ten-1	R13F6.4 0.134779934697137
AGIWUR35629;AGIWUR38757;AGIWUR41531
CB4856	CT	III	6847390 6849055 WBGene00020066	dhhc-5	R13F6.5 -0.333480091043551	AGIWUR6886
CB4856	CT	III	6851254 6853130 WBGene00005712	srv-1	R13F6.3 -0.0570952619928811	AGIWUR24472
CB4856	CT	III	6853113 6856804 WBGene00006971	zak-1	R13F6.6 -0.123551913373959	AGIWUR29484
CB4856	CT	III	6858793 6859571 WBGene00020067	clec-158	R13F6.8 -0.101376165018585
AGIWUR19949;AGIWUR39355
CB4856	CT	III	6859863 6860786 WBGene00020065	clec-159	R13F6.2 0.0935184823375312
AGIWUR5341
CB4856	CT	III	6860939 6863968 WBGene00004857	sma-3	R13F6.9 0.313376874604566	AGIWUR29198
CB4856	CT	III	6865207 6869460 WBGene00020068	cra-1	R13F6.10	-0.157316576564632
AGIWUR6115
CB4856	CT	III	6869634 6870298 WBGene00020064	kbp-1	R13F6.1 0.0357187736454243	AGIWUR9178
CB4856	CT	III	6870296 6871171 WBGene00044312	K04C2.8 K04C2.8 -0.00502459790898335
AGIWUR8796;AGIWUR11686;AGIWUR12275;AGIWUR21315;AGIWUR28990;AGIWUR41699;AGIWUR44588
CB4856	CT	III	6873048 6873729 WBGene00019381	K04C2.3 K04C2.3 0.115475048660677
AGIWUR13400;AGIWUR14947;AGIWUR25514;AGIWUR26287;AGIWUR31355;AGIWUR34016;AGIWUR34276;AGIWUR34672;AGIWUR40758
CB4856	CT	III	6880947 6883853 WBGene00044311	K04C2.7 K04C2.7 -0.148664821919835
AGIWUR7543;AGIWUR16799;AGIWUR39321
CB4856	CT	III	6885204 6889523 WBGene00000265	brd-1	K04C2.4 -0.0319415014707616	AGIWUR29695
CB4856	CT	III	6889749 6895089 WBGene00019380	K04C2.2 K04C2.2 0.276305908179769	AGIWUR9457
CB4856	CT	III	6898016 6900605 WBGene00020315	T07E3.4 T07E3.4 -0.0557802256251598	AGIWUR17150
CB4856	CT	III	6900568 6903106 WBGene00020316	brc-2	T07E3.5 -0.263118414542854	AGIWUR23409
CB4856	CT	III	6903895 6905170 WBGene00020314	T07E3.3 T07E3.3 -0.0517469424379096
AGIWUR6606;AGIWUR7386
CB4856	CT	III	6907441 6908012 WBGene00020313	T07E3.2 T07E3.2 -0.188173876872191	AGIWUR40870
CB4856	CT	III	6909438 6912481 WBGene00020317	pdf-1	T07E3.6 0.421709432324626	AGIWUR21194
CB4856	CT	III	6924097 6928817 WBGene00018998	flap-1	F57B9.7 -0.130063045635431	AGIWUR38961
CB4856	CT	III	6930039 6931953 WBGene00002083	inf-1	F57B9.6 -0.0290558129938923	AGIWUR32955
CB4856	CT	III	6932001 6935862 WBGene00000276	byn-1	F57B9.5 0.0109312505041768
AGIWUR2887;AGIWUR9859;AGIWUR13290
CB4856	CT	III	6935967 6939443 WBGene00000762	coq-2	F57B9.4 0.0428003380578827
AGIWUR7144;AGIWUR9523;AGIWUR12892;AGIWUR31473;AGIWUR38173;AGIWUR43114
CB4856	CT	III	6939888 6941736 WBGene00018997	F57B9.3 F57B9.3 -0.0672095726510076
AGIWUR23882;AGIWUR25698;AGIWUR38042
CB4856	CT	III	6941889 6945895 WBGene00004133	abu-13	F57B9.9 0.550524830418556	AGIWUR40209

CB4856	CT	III	6946718	6958882	WBGene00002845	let-711	F57B9.2	0.244702975887858	AGIWUR10152
CB4856	CT	III	6959110	6962027	WBGene00004462	rpn-6.1	F57B9.10	-0.0304193968702702	AGIWUR2602;AGIWUR16836;AGIWUR22959
CB4856	CT	III	6962090	6963319	WBGene00018996	F57B9.1	F57B9.1	0.0238110230943356	AGIWUR37456
CB4856	CT	III	6963620	6968130	WBGene00017951	panl-2	F31E3.4	-0.092652712413603	AGIWUR3878
CB4856	CT	III	6970026	6972003	WBGene00001168	eef-1A.1	F31E3.5	-0.00645151711195879	AGIWUR14568;AGIWUR18769;AGIWUR22122;AGIWUR25445;AGIWUR25772;AGIWUR41301;AGIWUR42112
CB4856	CT	III	6972210	6973717	WBGene00004340	rfc-4	F31E3.3	-0.0976877023356906	AGIWUR37637
CB4856	CT	III	6974338	6978025	WBGene00017950	F31E3.2	F31E3.2	0.112607293713749	AGIWUR6472;AGIWUR9830;AGIWUR20256;AGIWUR21734;AGIWUR27342;AGIWUR35926;AGIWUR37116;AGIWUR37915;AGIWUR39344
CB4856	CT	III	6979884	6981785	WBGene00000443	ceh-20	F31E3.1	0.400360210051286	AGIWUR8244
CB4856	CT	III	6990168	7002310	WBGene00004182	prk-1	C06E8.3	0.141962178623142	AGIWUR1009;AGIWUR20404;AGIWUR20919;AGIWUR43839
CB4856	CT	III	7002301	7005450	WBGene00015544	C06E8.5	C06E8.5	-0.32365366783257	AGIWUR44652
CB4856	CT	III	7009136	7019574	WBGene00017389	lgc-38	F11H8.2	0.356556721994527	AGIWUR127;AGIWUR44935
CB4856	CT	III	7019583	7020600	WBGene00000597	col-8	F11H8.3	-0.190713213553401	AGIWUR25771
CB4856	CT	III	7021167	7023652	WBGene00004341	rfl-1	F11H8.1	-0.00952146011353605	AGIWUR25920
CB4856	CT	III	7023642	7029203	WBGene00000872	cyk-1	F11H8.4	0.042512961919166	AGIWUR2929;AGIWUR7188;AGIWUR9447;AGIWUR31305;AGIWUR34755;AGIWUR36247
CB4856	CT	III	7035432	7045724	WBGene00023414	R01H2.7	R01H2.7	-0.0116467472147943	AGIWUR10603;AGIWUR22901;AGIWUR23233;AGIWUR41509
CB4856	CT	III	7056541	7058526	WBGene00019810	R01H2.2	R01H2.2	0.0897509290525971	AGIWUR6400;AGIWUR27609;AGIWUR33698
CB4856	CT	III	7058516	7060597	WBGene00019811	egg-2	R01H2.3	-0.0101099019067536	AGIWUR39983
CB4856	CT	III	7061111	7064275	WBGene00019812	R01H2.4	R01H2.4	0.0918395408458661	AGIWUR17584
CB4856	CT	III	7065439	7067477	WBGene00019813	ger-1	R01H2.5	-0.167404519792338	AGIWUR8609;AGIWUR22100;AGIWUR34387
CB4856	CT	III	7067686	7068570	WBGene00006713	ubc-18	R01H2.6	-0.0400102861660052	AGIWUR20704;AGIWUR29116;AGIWUR36145;AGIWUR38951
CB4856	CT	III	7069103	7069580	WBGene00045190	R01H2.8	R01H2.8	0.663522111138806	AGIWUR613;AGIWUR4684;AGIWUR11900;AGIWUR31809;AGIWUR39639
CB4856	CT	III	7070800	7072552	WBGene00003022	lin-37	ZK418.4	-0.222370188129821	AGIWUR4353
CB4856	CT	III	7072613	7073851	WBGene00022734	ZK418.5	ZK418.5	-0.149947682446586	AGIWUR5961
CB4856	CT	III	7074250	7076349	WBGene00220259	ZK418.13	ZK418.13	0.0962085472369196	AGIWUR31633;AGIWUR36398;AGIWUR37087
CB4856	CT	III	7079077	7080789	WBGene00022736	ZK418.7	ZK418.7	-0.373335965943893	AGIWUR15775;AGIWUR28330;AGIWUR32337
CB4856	CT	III	7083684	7085088	WBGene00022737	tofu-7	ZK418.8	-0.385158433854761	AGIWUR1384

CB4856 CT III 7085197 7087741 WBGene00022738 ZK418.9 ZK418.9 -0.263775465251776
AGIWUR5350;AGIWUR11967;AGIWUR12779;AGIWUR22635;AGIWUR44900
CB4856 CT III 7088644 7090132 WBGene00044353 ZK418.10 ZK418.10 0.121890494534649
AGIWUR33945
CB4856 CT III 7091330 7093408 WBGene00022732 ZK418.2 ZK418.2 -0.0899429020653386
AGIWUR26774;AGIWUR38970;AGIWUR43419
CB4856 CT III 7095553 7097079 WBGene00003608 nhr-9 ZK418.1 0.0214604656566522 AGIWUR40203
CB4856 CT III 7100482 7102033 WBGene00044354 ZK418.11 ZK418.11 -0.134567245434448
AGIWUR12692;AGIWUR40894
CB4856 CT III 7102086 7103990 WBGene00015102 cpg-2 B0280.5 0.0439267139543205
AGIWUR14788;AGIWUR26818
CB4856 CT III 7108322 7109741 WBGene00003845 odd-1 B0280.4 0.114311661907712 AGIWUR21681
CB4856 CT III 7110302 7111893 WBGene00015103 B0280.7 B0280.7 -0.290519306258521 AGIWUR8179
CB4856 CT III 7112300 7115438 WBGene00003609 nhr-10 B0280.8 0.0251300641220826 AGIWUR15057
CB4856 CT III 7120905 7122895 WBGene00015104 B0280.9 B0280.9 0.204373439191732 AGIWUR38406
CB4856 CT III 7123128 7124329 WBGene00015101 rpia-1 B0280.3 -0.200148846755051
AGIWUR24377;AGIWUR29690;AGIWUR34957;AGIWUR43357
CB4856 CT III 7132038 7133674 WBGene00015105 pot-1 B0280.10 -0.0902415112753392
AGIWUR17608
CB4856 CT III 7133766 7136460 WBGene00015099 ggtb-1 B0280.1 -0.129398815864413
AGIWUR26;AGIWUR16425;AGIWUR21558;AGIWUR29845
CB4856 CT III 7136510 7138278 WBGene00015106 B0280.11 B0280.11 -0.0797243303230668
AGIWUR27947;AGIWUR34409
CB4856 CT III 7138683 7144832 WBGene00001613 glr-2 B0280.12 0.486920097366682
AGIWUR21369
CB4856 CT III 7145816 7153795 WBGene00003858 ogt-1 K04G7.3 0.0857335464206637
AGIWUR5550;AGIWUR36960
CB4856 CT III 7156032 7158809 WBGene00019401 nuo-4 K04G7.4 -0.066859377118865
AGIWUR1349;AGIWUR3103;AGIWUR11949;AGIWUR15549;AGIWUR17066;AGIWUR21918;AGIWUR42710;AGIWUR44306
CB4856 CT III 7158912 7161157 WBGene00004390 rnp-7 K04G7.10 -0.193454775485536
AGIWUR27127
CB4856 CT III 7161157 7162115 WBGene00019402 K04G7.11 K04G7.11 -0.0655635893989448
AGIWUR41126
CB4856 CT III 7162694 7165261 WBGene00019400 K04G7.1 K04G7.1 -0.0741312978692239 AGIWUR11536
CB4856 CT III 7167838 7170990 WBGene00018152 acs-4 F37C12.7 0.0185064232669783
AGIWUR33729;AGIWUR35716
CB4856 CT III 7177530 7178853 WBGene00005072 srb-7 F37C12.17 -0.319620559733675
AGIWUR8880
CB4856 CT III 7178984 7179606 WBGene00004483 rps-14 F37C12.9 0.048645736899287

AGIWUR2374;AGIWUR32041
CB4856 CT III 7180249 7180677 WBGene00004450 rpl-36 F37C12.4 -0.0138508950022214
AGIWUR3760;AGIWUR5590;AGIWUR25437
CB4856 CT III 7180730 7181925 WBGene00018151 F37C12.3 F37C12.3 -0.441685840148705
AGIWUR5900;AGIWUR21081
CB4856 CT III 7181970 7184049 WBGene00018150 epg-4 F37C12.2 0.00273232232475706
AGIWUR42197
CB4856 CT III 7184348 7187868 WBGene00018149 F37C12.1 F37C12.1 0.0839225936722414
AGIWUR4488
CB4856 CT III 7187841 7188430 WBGene00018153 F37C12.10 F37C12.10
-0.00625430031176677 AGIWUR22078
CB4856 CT III 7189142 7189631 WBGene00004490 rps-21 F37C12.11 -0.00110256067023329
AGIWUR12006;AGIWUR31276;AGIWUR34444
CB4856 CT III 7190207 7192992 WBGene00003176 mec-14 F37C12.12 -0.0611575040554819
AGIWUR9445;AGIWUR17804;AGIWUR28649;AGIWUR39147
CB4856 CT III 7194094 7195425 WBGene00044005 F37C12.18 F37C12.18 0.0679455306829819
AGIWUR11612
CB4856 CT III 7196225 7232172 WBGene00018154 exos-9 F37C12.13 -0.339612915856772
AGIWUR7392;AGIWUR7814;AGIWUR20734;AGIWUR21953;AGIWUR33274
CB4856 CT III 7232926 7234202 WBGene00020636 T20H4.5 T20H4.5 -0.192238851172772
AGIWUR19610;AGIWUR21920;AGIWUR24242;AGIWUR33423
CB4856 CT III 7238607 7241284 WBGene00004189 pars-1 T20H4.3 0.0556096717426033
AGIWUR4343;AGIWUR9909;AGIWUR36586
CB4856 CT III 7241365 7242950 WBGene00020635 T20H4.2 T20H4.2 -0.00683651249520523 AGIWUR15565
CB4856 CT III 7243305 7245743 WBGene00003890 osm-10 T20H4.1 0.0964584821549916 AGIWUR39413
CB4856 CT III 7245703 7247937 WBGene00020646 zmp-6 T21D11.1 -0.261084724853456
AGIWUR9527
CB4856 CT III 7268414 7275396 WBGene00015159 psd-1 B0361.5 0.325393767858048
AGIWUR9371;AGIWUR25166
CB4856 CT III 7275502 7277274 WBGene00015160 B0361.6 B0361.6 -0.27308497180427 AGIWUR42062
CB4856 CT III 7277604 7281217 WBGene00015165 B0361.11 B0361.11 0.0758963673414712
AGIWUR44378
CB4856 CT III 7281377 7283959 WBGene00015157 B0361.3 B0361.3 -0.197835806180901 AGIWUR22638
CB4856 CT III 7283915 7285883 WBGene00015161 pho-5 B0361.7 0.0519100192696542
AGIWUR4284;AGIWUR4841;AGIWUR6453;AGIWUR19407;AGIWUR27150;AGIWUR32167
CB4856 CT III 7285983 7287603 WBGene00015164 ykt-6 B0361.10 -0.163937733385586
AGIWUR22374
CB4856 CT III 7287848 7290562 WBGene00015162 algn-11 B0361.8 -0.0624180893717714 AGIWUR25298
CB4856 CT III 7293632 7308364 WBGene00015156 B0361.2 B0361.2 -0.240577305493993 AGIWUR464

CB4856	CT	III	7308354 7308975 WBGene00018949	acbp-4	F56C9.5 0.153912452909136	AGIWUR42872
CB4856	CT	III	7308998 7310773 WBGene00018950	F56C9.6 F56C9.6 -0.0254236705621729	AGIWUR36977
CB4856	CT	III	7311290 7313195 WBGene00018948	F56C9.3 F56C9.3 -0.207853259447571	AGIWUR2658
CB4856	CT	III	7313149 7314977 WBGene00018951	F56C9.7 F56C9.7 -0.522753616011371	AGIWUR21582
CB4856	CT	III	7315903 7319595 WBGene00018952	F56C9.8 F56C9.8 0.449522597009953	AGIWUR35334
CB4856	CT	III	7324940 7329649 WBGene00018954	F56C9.11	F56C9.11	-0.360994430568528
AGIWUR145
CB4856	CT	III	7329714 7335316 WBGene00018953	F56C9.10	F56C9.10	-0.0502094163789675
AGIWUR4974;AGIWUR6930;AGIWUR7997;AGIWUR15213;AGIWUR25399;AGIWUR25573;AGIWUR40411;AGIWUR43493;AGIWUR43851
CB4856	CT	III	7336587 7338765 WBGene00001748	gsp-2	F56C9.1 0.0272409710466448	AGIWUR26543
CB4856	CT	III	7339001 7343626 WBGene00002182	kap-1	F08F8.3 0.206063894744429
AGIWUR23181;AGIWUR29263
CB4856	CT	III	7343675 7345855 WBGene00017271	F08F8.6 F08F8.6 -0.0235714703366643	AGIWUR6914
CB4856	CT	III	7345861 7347149 WBGene00017272	F08F8.7 F08F8.7 -0.114332342973833
AGIWUR3256;AGIWUR12445;AGIWUR15029;AGIWUR17210;AGIWUR17376;AGIWUR43081
CB4856	CT	III	7347237 7349175 WBGene00017273	gos-28	F08F8.8 -0.0308101904671076	AGIWUR19819
CB4856	CT	III	7349291 7354090 WBGene00017274	F08F8.9 F08F8.9 -0.0517804544839287
AGIWUR6831;AGIWUR31117;AGIWUR42085
CB4856	CT	III	7354159 7357395 WBGene00017275	F08F8.10	F08F8.10	-0.142536173040956
AGIWUR3820
CB4856	CT	III	7357663 7359183 WBGene00017269	F08F8.4 F08F8.4 -0.227501344605896	AGIWUR6271
CB4856	CT	III	7359498 7362132 WBGene00017268	hmgr-1	F08F8.2 -0.139096914666034
AGIWUR15001;AGIWUR25419;AGIWUR25776
CB4856	CT	III	7365902 7366448 WBGene00269381	F08F8.11	F08F8.11	-0.265713072189804
AGIWUR5929
CB4856	CT	III	7366717 7367112 WBGene00020603	T20B12.5	T20B12.5	0.105970550230921
AGIWUR45073
CB4856	CT	III	7367710 7369538 WBGene00020602	T20B12.4	T20B12.4	-0.115386470453973
AGIWUR29818
CB4856	CT	III	7370131 7374731 WBGene00003378	mml-1	T20B12.6	0.135854286324516
AGIWUR277;AGIWUR27528;AGIWUR27964
CB4856	CT	III	7377854 7379045 WBGene00020604	T20B12.7	T20B12.7	0.0466870957437973
AGIWUR17909;AGIWUR18286;AGIWUR31122;AGIWUR35623;AGIWUR36772;AGIWUR43617
CB4856	CT	III	7379143 7381609 WBGene00001974	hmg-4	T20B12.8	-0.222007522146818
AGIWUR13021;AGIWUR13254;AGIWUR15814;AGIWUR16997;AGIWUR37862
CB4856	CT	III	7382115 7383906 WBGene00020601	T20B12.3	T20B12.3	-0.0246090500733089
AGIWUR22514
CB4856	CT	III	7384033 7386132 WBGene00006542	tbp-1	T20B12.2	-0.193998936886938
AGIWUR1258;AGIWUR1790;AGIWUR12732;AGIWUR19590;AGIWUR40034

CB4856	CT	III	7386229	7391135	WBGene00020600	trd-1	T20B12.1	-0.00768515895678235	AGIWUR24657;AGIWUR32413;AGIWUR36042
CB4856	CT	III	7391482	7395454	WBGene00020605	lgc-50	T20B12.9	0.0437304507714185	AGIWUR5207
CB4856	CT	III	7454012	7455980	WBGene00000118	alh-12	Y69F12A.2	-0.351768073798326	AGIWUR29059;AGIWUR34442
CB4856	CT	III	7456734	7458544	WBGene00022102	Y69F12A.1	Y69F12A.1	0.0884503233826681	AGIWUR11528;AGIWUR13958;AGIWUR44146
CB4856	CT	III	7460341	7461931	WBGene00003376	mls-1	H14A12.4	0.0671056194605022	AGIWUR13598
CB4856	CT	III	7463797	7465238	WBGene00019196	H14A12.3	H14A12.3	-0.213919811129779	AGIWUR2821
CB4856	CT	III	7465605	7467633	WBGene00001503	fum-1	H14A12.2	-0.0124986561353779	AGIWUR21960;AGIWUR44920
CB4856	CT	III	7467574	7468676	WBGene00019197	H14A12.5	H14A12.5	0.0652519721362577	AGIWUR34074
CB4856	CT	III	7479680	7489075	WBGene00003497	mup-4	K07D8.1	-0.337359123983074	AGIWUR31451
CB4856	CT	III	7495320	7497832	WBGene00000479	cgh-1	C07H6.5	-0.0424774367524741	AGIWUR3774;AGIWUR4099;AGIWUR15533;AGIWUR16780;AGIWUR28635;AGIWUR38324;AGIWUR42561
CB4856	CT	III	7498819	7499447	WBGene00015582	C07H6.9	C07H6.9	0.943130309872066	AGIWUR10743
CB4856	CT	III	7499677	7503241	WBGene00015581	C07H6.4	C07H6.4	0.12259347726746	AGIWUR706;AGIWUR875;AGIWUR5973;AGIWUR7898;AGIWUR15254;AGIWUR15464;AGIWUR21636;AGIWUR23127;AGIWUR33052;AGIWUR33165;AGIWUR38055
CB4856	CT	III	7508178	7515285	WBGene00015580	cls-1	C07H6.3	0.0403945313290102	AGIWUR6187;AGIWUR26175;AGIWUR28060;AGIWUR38483
CB4856	CT	III	7515223	7518524	WBGene00000537	clk-2	C07H6.6	-0.0284677175213304	AGIWUR5586;AGIWUR7836
CB4856	CT	III	7518628	7520845	WBGene00000854	cux-7	C07H6.8	-0.0296821909524265	AGIWUR12788;AGIWUR23553;AGIWUR39468;AGIWUR43244
CB4856	CT	III	7521004	7524836	WBGene00002986	lig-4	C07H6.1	0.343777785896473	AGIWUR12502
CB4856	CT	III	7528641	7536632	WBGene00003024	lin-39	C07H6.7	0.0112059208465478	AGIWUR2784
CB4856	CT	III	7555617	7558208	WBGene00000437	ceh-13	R13A5.5	0.324683235816028	AGIWUR6653
CB4856	CT	III	7562172	7564529	WBGene00020048	lgc-12	R13A5.4	-0.0170387339709137	AGIWUR22283
CB4856	CT	III	7564500	7565347	WBGene00020049	ttr-8	R13A5.6	0.225719113943709	AGIWUR10718
CB4856	CT	III	7566411	7567486	WBGene00020047	ttr-32	R13A5.3	0.0908523054963002	AGIWUR44767
CB4856	CT	III	7567475	7570133	WBGene00020050	R13A5.7	R13A5.7	-0.111070656623047	AGIWUR8137
CB4856	CT	III	7572105	7572831	WBGene00004420	rpl-9	R13A5.8	0.0821245265796122	AGIWUR14686
CB4856	CT	III	7573350	7575263	WBGene00003063	lpd-7	R13A5.12	0.297737308929857	AGIWUR3802;AGIWUR36129;AGIWUR45192

CB4856 CT III 7575274 7579411 WBGene00020051 R13A5.9 R13A5.9 -0.199794778962852 AGIWUR25499
CB4856 CT III 7582033 7582999 WBGene00020052 R13A5.10 R13A5.10 -0.525861195521266
AGIWUR14155
CB4856 CT III 7584181 7591570 WBGene00000846 cup-5 R13A5.1 0.129446349859252
AGIWUR8333;AGIWUR37240
CB4856 CT III 7605949 7608290 WBGene00020202 T04A6.3 T04A6.3 -0.338057318419614 AGIWUR19994
CB4856 CT III 7614862 7615699 WBGene00020201 T04A6.2 T04A6.2 0.122604828369287
AGIWUR4917;AGIWUR9169;AGIWUR22596;AGIWUR28490
CB4856 CT III 7616528 7617590 WBGene00020200 T04A6.1 T04A6.1 0.508166495619652
AGIWUR22210;AGIWUR23187;AGIWUR23913;AGIWUR27973
CB4856 CT III 7625376 7641053 WBGene00022816 fbn-1 ZK783.1 0.282266209059654
AGIWUR3202;AGIWUR4339;AGIWUR28751;AGIWUR30718;AGIWUR36180;AGIWUR43017
CB4856 CT III 7641034 7643101 WBGene00022817 upp-1 ZK783.2 0.0220250922259977
AGIWUR33056;AGIWUR36827;AGIWUR44756;AGIWUR44950
CB4856 CT III 7648788 7650005 WBGene00044448 ZK783.6 ZK783.6 -0.400376842707932 AGIWUR41156
CB4856 CT III 7650549 7656032 WBGene00001470 baz-2 ZK783.4 -0.19030664545866 AGIWUR33055
CB4856 CT III 7662428 7672153 WBGene00015988 C18H2.1 C18H2.1 -0.254583394779768 AGIWUR27375
CB4856 CT III 7684357 7688917 WBGene00015990 C18H2.3 C18H2.3 -0.145010971700794 AGIWUR18472
CB4856 CT III 7689356 7694398 WBGene00015992 C18H2.5 C18H2.5 0.198579348049262 AGIWUR36085
CB4856 CT III 7694554 7700381 WBGene00015991 C18H2.4 C18H2.4 0.0705148530501916 AGIWUR28887
CB4856 CT III 7700338 7709491 WBGene00003002 lin-13 C03B8.4 0.231865200851821
AGIWUR1136;AGIWUR13351;AGIWUR42089
CB4856 CT III 7712309 7717314 WBGene00015386 C03B8.3 C03B8.3 0.0333959491890159
AGIWUR4334;AGIWUR7654;AGIWUR10933;AGIWUR12045;AGIWUR13588;AGIWUR22325;AGIWUR26403
CB4856 CT III 7719791 7738105 WBGene00003559 ncl-1 ZK112.2 -0.0589347215322995
AGIWUR29632;AGIWUR30707;AGIWUR44134
CB4856 CT III 7738085 7739656 WBGene00022661 ZK112.5 ZK112.5 0.41697962766249 AGIWUR8890
CB4856 CT III 7739855 7740541 WBGene00022662 ZK112.6 ZK112.6 -0.03661575340894 AGIWUR34004
CB4856 CT III 7741326 7754448 WBGene00000395 cdh-3 ZK112.7 0.199824351418311 AGIWUR26163
CB4856 CT III 7758730 7761273 WBGene00003956 pcp-1 ZK112.1 -0.32555678266996 AGIWUR32149
CB4856 CT III 7761977 7763603 WBGene00022516 mtx-2 ZC97.1 -0.298183934867133 AGIWUR33978
CB4856 CT III 7763698 7766052 WBGene00022792 ZK686.2 ZK686.2 -0.150673984101585 AGIWUR29287
CB4856 CT III 7766389 7767515 WBGene00022795 ZK686.5 ZK686.5 0.113812021378746 AGIWUR15013
CB4856 CT III 7767571 7769063 WBGene00022793 ZK686.3 ZK686.3 -0.305354751350229
AGIWUR1330;AGIWUR17964
CB4856 CT III 7769165 7770149 WBGene00022794 snu-23 ZK686.4 0.0464880605693159
AGIWUR31585;AGIWUR44773
CB4856 CT III 7771591 7772559 WBGene00044799 ZK686.6 ZK686.6 -0.294977762605783
AGIWUR10543;AGIWUR38278

CB4856 CT III 7773134 7773849 WBGene00022791 ZK686.1 ZK686.1 -0.235714808932987 AGIWUR3542;AGIWUR27040;AGIWUR33797
CB4856 CT III 7774778 7776039 WBGene00015590 bath-15 C08C3.2 -0.135705218953824 AGIWUR30918
CB4856 CT III 7776835 7783454 WBGene00003102 mab-5 C08C3.3 -0.106349061854151 AGIWUR524
CB4856 CT III 7793446 7795409 WBGene00015591 cyk-7 C08C3.4 0.0148325136276171 AGIWUR39828
CB4856 CT III 7814380 7817127 WBGene00001174 egl-5 C08C3.1 0.170801339229997 AGIWUR9619;AGIWUR10136;AGIWUR23410;AGIWUR31673;AGIWUR34828;AGIWUR41220
CB4856 CT III 7824377 7828626 WBGene00001209 egl-45 C27D11.1 -0.0172256719254307 AGIWUR16837
CB4856 CT III 7829411 7832503 WBGene00022785 ZK652.6 ZK652.6 -0.112647128600273 AGIWUR22114;AGIWUR26296;AGIWUR34734
CB4856 CT III 7839594 7841491 WBGene00000446 ceh-23 ZK652.5 0.0208361673957826 AGIWUR4590
CB4856 CT III 7842381 7843636 WBGene00022786 ZK652.8 ZK652.8 -0.00985311840708469 AGIWUR21230
CB4856 CT III 7855118 7855680 WBGene00004449 rpl-35 ZK652.4 -0.0370563933227004 AGIWUR1318;AGIWUR7271;AGIWUR13592;AGIWUR15530;AGIWUR19789;AGIWUR21396;AGIWUR22629;AGIWUR23860;AGIWUR27111;AGIWUR36006;AGIWUR41499
CB4856 CT III 7855840 7857242 WBGene00022787 tag-307 ZK652.10 -0.198899678558687 AGIWUR7350
CB4856 CT III 7857315 7858457 WBGene00000765 coq-5 ZK652.9 -0.376054469226386 AGIWUR16679
CB4856 CT III 7858979 7859512 WBGene00044324 ufm-1 ZK652.3 -0.0373899060488356 AGIWUR7099;AGIWUR15272
CB4856 CT III 7859613 7860159 WBGene00022783 tomm-7 ZK652.2 0.0986190502583426 AGIWUR10295;AGIWUR25626;AGIWUR26385;AGIWUR36387;AGIWUR38431;AGIWUR41648
CB4856 CT III 7860466 7861004 WBGene00000835 cuc-1 ZK652.11 -0.524209340280038 AGIWUR21223
CB4856 CT III 7862070 7862577 WBGene00004918 snr-5 ZK652.1 -0.13654226589067 AGIWUR7207;AGIWUR8160;AGIWUR18084;AGIWUR22192;AGIWUR27998;AGIWUR30639
CB4856 CT III 7862583 7865306 WBGene00015333 slc-17.3 C02C2.4 -0.230669241665804 AGIWUR31202
CB4856 CT III 7866509 7868732 WBGene00000845 cup-4 C02C2.3 -0.162665507827402 AGIWUR37722
CB4856 CT III 7873781 7877194 WBGene00015332 tyr-1 C02C2.1 0.00852442039088608 AGIWUR41080
CB4856 CT III 7877176 7878839 WBGene00015334 sup-18 C02C2.5 0.0946858567701341 AGIWUR39722
CB4856 CT III 7881113 7883003 WBGene00022803 ZK688.9 ZK688.9 0.192775643106813 AGIWUR43699
CB4856 CT III 7883574 7884963 WBGene00044762 ZK688.11 ZK688.11 0.0997323217283679 AGIWUR19621;AGIWUR27683
CB4856 CT III 7885088 7891615 WBGene00022800 ZK688.5 ZK688.5 -0.124629343436407 AGIWUR14059;AGIWUR42115
CB4856 CT III 7892256 7894625 WBGene00022798 fahd-1 ZK688.3 0.529685420616794 AGIWUR9390
CB4856 CT III 7895024 7895270 WBGene00022799 ZK688.4 ZK688.4 -0.0318400589317971

AGIWUR13823;AGIWUR16019;AGIWUR16901;AGIWUR19883;AGIWUR19970;AGIWUR22563;AGIWUR28504;AGIWUR37484;AGIWUR37746;AGIWUR38268;AGIWUR40686

CB4856	CT	III	7895752	7897025	WBGene00044447	ZK688.10	ZK688.10	-0.111237897873445	AGIWUR2695;AGIWUR9783
CB4856	CT	III	7901033	7904473	WBGene00022797	best-24	ZK688.2	0.118459458326516	AGIWUR3194;AGIWUR10548;AGIWUR22051
CB4856	CT	III	7904945	7907843	WBGene00022801	pcp-5	ZK688.6	-0.213713669807342	AGIWUR20771
CB4856	CT	III	7909563	7910758	WBGene00022796	ZK688.1	ZK688.1	-0.101088708608733	AGIWUR6214
CB4856	CT	III	7910798	7911630	WBGene00022802	ZK688.7	ZK688.7	0.366970415273776	AGIWUR42017
CB4856	CT	III	7912698	7913600	WBGene00044763	ZK688.12	ZK688.12	-0.131560687717759	AGIWUR36930;AGIWUR43391;AGIWUR44954
CB4856	CT	III	7914314	7917767	WBGene00001628	gly-3	ZK688.8	0.0706211577499918	AGIWUR13062;AGIWUR36778;AGIWUR36892
CB4856	CT	III	7917987	7924917	WBGene00016203	tag-250	C29E4.5	-0.0861440662115909	AGIWUR16151;AGIWUR24388;AGIWUR44595;AGIWUR44893
CB4856	CT	III	7926999	7928734	WBGene00043051	C29E4.13	C29E4.13	-0.313865803515079	AGIWUR727;AGIWUR15228
CB4856	CT	III	7929013	7932993	WBGene00003801	npp-15	C29E4.4	-0.178225461556998	AGIWUR31543
CB4856	CT	III	7933535	7937708	WBGene00004303	ran-2	C29E4.3	0.0447869815905939	AGIWUR24556
CB4856	CT	III	7937827	7940931	WBGene00016207	C29E4.10	C29E4.10	-0.166039577766197	AGIWUR39117
CB4856	CT	III	7941057	7941614	WBGene00016209	C29E4.12	C29E4.12	0.511033415994051	AGIWUR10229
CB4856	CT	III	7941717	7945593	WBGene00016202	kle-2	C29E4.2	-0.0620051685789331	AGIWUR24070
CB4856	CT	III	7945544	7947018	WBGene00016204	gsto-1	C29E4.7	-0.0518069834862089	AGIWUR31884
CB4856	CT	III	7948838	7950068	WBGene00000665	col-90	C29E4.1	-0.424579184320803	AGIWUR12003;AGIWUR43808
CB4856	CT	III	7949975	7951915	WBGene00002879	let-754	C29E4.8	-0.172619409134959	AGIWUR3078;AGIWUR6977;AGIWUR16207;AGIWUR24532
CB4856	CT	III	7952623	7955816	WBGene00045246	C29E4.15	C29E4.15	-0.312283430085294	AGIWUR7251;AGIWUR10062;AGIWUR39986
CB4856	CT	III	7955828	7958105	WBGene00016206	C29E4.9	C29E4.9	0.158465863568932	AGIWUR3333;AGIWUR5912;AGIWUR14429;AGIWUR23987;AGIWUR24372;AGIWUR27083;AGIWUR27187;AGIWUR37254;AGIWUR40677
CB4856	CT	III	7961596	7962741	WBGene00018844	F54H12.4	F54H12.4	0.278409805963856	AGIWUR22335
CB4856	CT	III	7966601	7969221	WBGene00045247	F54H12.8	F54H12.8	-0.174287134555211	AGIWUR229
CB4856	CT	III	7969319	7970283	WBGene00044633	F54H12.7	F54H12.7	-0.0693591697353249	AGIWUR4710;AGIWUR17387;AGIWUR38904

CB4856	CT	III	7970263	7972161	WBGene00018845	F54H12.5	F54H12.5	-0.460003724041846	AGIWUR34607
CB4856	CT	III	7972382	7973386	WBGene00018846	eef-1B.1	F54H12.6	0.0507376555296783	AGIWUR27194;AGIWUR33538
CB4856	CT	III	7973717	7977355	WBGene00000041	aco-2	F54H12.1	0.0256971281001652	AGIWUR550;AGIWUR666;AGIWUR7264;AGIWUR7765;AGIWUR11526;AGIWUR32513;AGIWUR33054;AGIWUR39786
CB4856	CT	III	7977216	7977803	WBGene00015558	C06G4.4	C06G4.4	0.0976706975074793	AGIWUR12183;AGIWUR18220;AGIWUR45108
CB4856	CT	III	7980913	7986327	WBGene00000542	clp-1	C06G4.2	0.31582112792276	AGIWUR2782;AGIWUR10623;AGIWUR24515
CB4856	CT	III	7987121	7990613	WBGene00015557	C06G4.1	C06G4.1	-0.182960172530455	AGIWUR11719
CB4856	CT	III	7990789	7996438	WBGene00015559	npr-17	C06G4.5	-0.147306000656654	AGIWUR34939
CB4856	CT	III	8004397	8005887	WBGene00044446	C06G4.6	C06G4.6	-0.110112986706384	AGIWUR299
CB4856	CT	III	8005935	8009821	WBGene00000507	cit-1.1	F44B9.4	-0.0620940927571674	AGIWUR4816;AGIWUR18943;AGIWUR28638
CB4856	CT	III	8010778	8013774	WBGene00000508	cit-1.2	F44B9.3	-0.196726887619379	AGIWUR8542;AGIWUR15822;AGIWUR17269
CB4856	CT	III	8013753	8016538	WBGene00018408	F44B9.5	F44B9.5	-0.1906713906123	AGIWUR17897
CB4856	CT	III	8016648	8020873	WBGene00003021	lin-36	F44B9.6	0.0821773806739499	AGIWUR12990;AGIWUR14761;AGIWUR20035;AGIWUR29449;AGIWUR36188;AGIWUR44489;AGIWUR45031
CB4856	CT	III	8022463	8025195	WBGene00018407	F44B9.2	F44B9.2	-0.00787540153280375	AGIWUR33109
CB4856	CT	III	8025210	8028042	WBGene00004125	mdt-30	F44B9.7	-0.242154616756235	AGIWUR34503
CB4856	CT	III	8028133	8029649	WBGene00018409	F44B9.8	F44B9.8	-0.370154203349732	AGIWUR1353;AGIWUR8841;AGIWUR14148;AGIWUR15772
CB4856	CT	III	8029858	8031063	WBGene00018410	F44B9.9	F44B9.9	0.122493905721524	AGIWUR21414
CB4856	CT	III	8031413	8033727	WBGene00018411	F44B9.10	F44B9.10	0.143934457384516	AGIWUR27975
CB4856	CT	III	8038241	8041374	WBGene00001059	dpf-6	F44B9.1	0.28563025680555	AGIWUR786;AGIWUR7937;AGIWUR10600;AGIWUR12968;AGIWUR15899;AGIWUR39780
CB4856	CT	III	8042577	8042875	WBGene00019681	K12H4.6	K12H4.6	-0.0271682299438432	AGIWUR27362
CB4856	CT	III	8044481	8044916	WBGene00019680	K12H4.5	K12H4.5	-0.295889524470933	AGIWUR19414
CB4856	CT	III	8045017	8045931	WBGene00019679	K12H4.4	K12H4.4	-0.113217981587765	AGIWUR20073
CB4856	CT	III	8046034	8047612	WBGene00019678	K12H4.3	K12H4.3	-0.253454752186201	AGIWUR25530
CB4856	CT	III	8047712	8048461	WBGene00019677	K12H4.2	K12H4.2	-0.0204906538226446	AGIWUR20148
CB4856	CT	III	8048424	8051155	WBGene00019682	K12H4.7	K12H4.7	-0.341144118043609	AGIWUR10819;AGIWUR15259;AGIWUR34223
CB4856	CT	III	8067289	8071455	WBGene00000448	pros-1	K12H4.1	0.83779019461387	AGIWUR34802
CB4856	CT	III	8071916	8080281	WBGene00000939	dcr-1	K12H4.8	-0.163846617541775	AGIWUR15164
CB4856	CT	III	8080570	8083701	WBGene00000143	apc-2	K06H7.6	-0.0423624140341659	AGIWUR44183

CB4856 CT III 8083692 8085538 WBGene00019458 K06H7.7 K06H7.7 0.0115579763648537 AGIWUR13531
CB4856 CT III 8085705 8088449 WBGene00001743 grp-1 K06H7.4 -0.11536455565558 AGIWUR12843;AGIWUR21602;AGIWUR32200
CB4856 CT III 8088482 8091730 WBGene00019457 vms-1 K06H7.3 -0.239069712917223 AGIWUR34576
CB4856 CT III 8091708 8093042 WBGene00019460 idi-1 K06H7.9 -0.0328042314452546 AGIWUR11791
CB4856 CT III 8093570 8096311 WBGene00019456 K06H7.2 K06H7.2 0.10614244439933 AGIWUR11874
CB4856 CT III 8096736 8098216 WBGene00019459 K06H7.8 K06H7.8 -0.0150566288810126 AGIWUR25434
CB4856 CT III 8099327 8100638 WBGene00019455 K06H7.1 K06H7.1 -0.0260971228925833 AGIWUR23564
CB4856 CT III 8101348 8104076 WBGene00004042 plk-1 C14B9.4 -0.0378633620383493 AGIWUR7884;AGIWUR22550
CB4856 CT III 8108703 8111245 WBGene00015753 C14B9.3 C14B9.3 0.0776925372215214 AGIWUR5914;AGIWUR11983;AGIWUR39945;AGIWUR43369;AGIWUR44024;AGIWUR44415
CB4856 CT III 8111166 8126222 WBGene00001565 gei-8 C14B9.6 0.0334944830491004 AGIWUR22239;AGIWUR29182;AGIWUR35088
CB4856 CT III 8129551 8130227 WBGene00004433 rpl-21 C14B9.7 0.153103511370155 AGIWUR13555
CB4856 CT III 8130852 8132436 WBGene00015755 C14B9.10 C14B9.10 -0.060350182896892 AGIWUR9726;AGIWUR20003;AGIWUR24913;AGIWUR25956;AGIWUR27149;AGIWUR34179
CB4856 CT III 8133665 8136649 WBGene00015752 C14B9.2 C14B9.2 0.148460599360593 AGIWUR8508;AGIWUR11821;AGIWUR19513;AGIWUR30168;AGIWUR41633
CB4856 CT III 8138090 8139645 WBGene00002011 hsp-12.2 C14B9.1 0.161550575774574 AGIWUR13046;AGIWUR29749
CB4856 CT III 8139610 8144629 WBGene00015754 C14B9.8 C14B9.8 -0.0420893282309542 AGIWUR1876
CB4856 CT III 8145015 8145974 WBGene00017044 mrpl-18 D2007.4 -0.18754838719023 AGIWUR3327;AGIWUR16310;AGIWUR26805
CB4856 CT III 8148967 8154619 WBGene00017042 D2007.2 D2007.2 -0.00397813068381225 AGIWUR24883;AGIWUR30242
CB4856 CT III 8155470 8155989 WBGene00017041 D2007.1 D2007.1 -0.175776716733516 AGIWUR34639
CB4856 CT III 8155928 8158245 WBGene00017045 atg-13 D2007.5 -0.0207895561017928 AGIWUR4063
CB4856 CT III 8159155 8161338 WBGene00086546 cil-1 C50C3.7 -0.166137262152128 AGIWUR24538
CB4856 CT III 8161449 8163383 WBGene00016803 bath-42 C50C3.8 0.0456354023791068 AGIWUR3146;AGIWUR20862;AGIWUR21544;AGIWUR34384
CB4856 CT III 8164373 8172702 WBGene00004187 prp-8 C50C3.6 0.116037773319264 AGIWUR12014;AGIWUR25090;AGIWUR31758
CB4856 CT III 8176484 8177243 WBGene00016804 C50C3.12 C50C3.12 -0.0212887881239805 AGIWUR1160;AGIWUR13818;AGIWUR27621
CB4856 CT III 8179224 8180549 WBGene00016801 C50C3.5 C50C3.5 -0.365108611472411 AGIWUR2594;AGIWUR18155;AGIWUR27487
CB4856 CT III 8181881 8189372 WBGene00016800 C50C3.2 C50C3.2 -0.0487831109879249 AGIWUR16251
CB4856 CT III 8191817 8193847 WBGene00016799 C50C3.1 C50C3.1 0.0150508991086067 AGIWUR10522

CB4856	CT	III	8193844	8201206	WBGene00006772	unc-36	C50C3.9	0.0673414985607952	AGIWUR10266;AGIWUR11145;AGIWUR18480
CB4856	CT	III	8205124	8209945	WBGene00016242	C30A5.10	C30A5.10	0.0488444774813601	AGIWUR5775;AGIWUR37751;AGIWUR40981
CB4856	CT	III	8212750	8216336	WBGene00006818	unc-86	C30A5.7	0.584668565731276	AGIWUR37956
CB4856	CT	III	8225097	8226386	WBGene00016238	C30A5.3	C30A5.3	-0.0562701432182529	AGIWUR1588;AGIWUR5145;AGIWUR13733;AGIWUR20984;AGIWUR34110;AGIWUR39012
CB4856	CT	III	8226478	8227650	WBGene00004342	rfs-1	C30A5.2	-0.174093183436796	AGIWUR40272
CB4856	CT	III	8227362	8232660	WBGene00015350	fbxl-1	C02F5.7	0.707656043860791	AGIWUR32117;AGIWUR40156;AGIWUR40691;AGIWUR41092
CB4856	CT	III	8232651	8234591	WBGene00015349	henn-1	C02F5.6	-0.110689925865014	AGIWUR13862;AGIWUR17509;AGIWUR20220;AGIWUR33609;AGIWUR35010
CB4856	CT	III	8234526	8235543	WBGene00044445	C02F5.14	C02F5.14	-0.27071833682503	AGIWUR3399;AGIWUR8250;AGIWUR16249;AGIWUR19857;AGIWUR33544;AGIWUR37801
CB4856	CT	III	8236126	8236908	WBGene00015348	C02F5.5	C02F5.5	0.190855067791496	AGIWUR24696
CB4856	CT	III	8236984	8237881	WBGene00006628	tsp-2	C02F5.11	0.62197688111778	AGIWUR37744;AGIWUR39045
CB4856	CT	III	8237995	8239423	WBGene00006627	tsp-1	C02F5.8	1.38859030981279	AGIWUR1230
CB4856	CT	III	8240872	8243029	WBGene00015352	C02F5.12	C02F5.12	0.0389214914950813	AGIWUR8531
CB4856	CT	III	8243122	8244092	WBGene00003952	pbs-6	C02F5.9	-0.23310062770028	AGIWUR1154;AGIWUR35929
CB4856	CT	III	8244445	8245794	WBGene00015347	cids-1	C02F5.4	-0.0406615796612044	AGIWUR42649
CB4856	CT	III	8245891	8247258	WBGene00015346	C02F5.3	C02F5.3	-0.187033505352931	AGIWUR2618
CB4856	CT	III	8247351	8248814	WBGene00015353	C02F5.13	C02F5.13	0.277589767502629	AGIWUR12182
CB4856	CT	III	8248685	8249512	WBGene00015351	C02F5.10	C02F5.10	-0.0824947016097362	AGIWUR11304
CB4856	CT	III	8249619	8250193	WBGene00015345	C02F5.2	C02F5.2	-0.121785825435737	AGIWUR134;AGIWUR4847;AGIWUR16620;AGIWUR19511;AGIWUR28543;AGIWUR31316
CB4856	CT	III	8250636	8254286	WBGene00002231	knl-1	C02F5.1	-0.198735609160918	AGIWUR21785
CB4856	CT	III	8254975	8257638	WBGene00017320	F09G8.5	F09G8.5	-0.540759275821362	AGIWUR1808
CB4856	CT	III	8258345	8265805	WBGene00003562	ncr-2	F09G8.4	0.361401305189373	AGIWUR10674
CB4856	CT	III	8266062	8267841	WBGene00017319	mrps-9	F09G8.3	-0.056283094820234	AGIWUR33013
CB4856	CT	III	8267921	8268355	WBGene00017321	F09G8.7	F09G8.7	-0.200239670281897	AGIWUR5913;AGIWUR16944;AGIWUR22226;AGIWUR22263;AGIWUR22551;AGIWUR34486
CB4856	CT	III	8268609	8270680	WBGene00007056	crn-7	F09G8.2	-0.581824638040562	AGIWUR29025
CB4856	CT	III	8272960	8275971	WBGene00017322	clec-160	F09G8.8	-0.158903202646844	AGIWUR31776

CB4856	CT	III	8279108	8305832	WBGene00003243	mig-10	F10E9.6	0.377999530240515	AGIWUR9097;AGIWUR10503;AGIWUR26773
CB4856	CT	III	8305733	8326818	WBGene00017358	F10E9.7	F10E9.7	-0.219451709086169	AGIWUR4437
CB4856	CT	III	8326516	8330947	WBGene00022580	iglr-2	ZC262.3	0.00838870512743384	AGIWUR8953;AGIWUR24237;AGIWUR26998
CB4856	CT	III	8331047	8331919	WBGene00022583	mrps-18A	ZC262.8	-0.301129791773647	AGIWUR27397
CB4856	CT	III	8332223	8333572	WBGene00022579	ZC262.2	ZC262.2	0.137284402547658	AGIWUR6868;AGIWUR9361;AGIWUR12810;AGIWUR18704;AGIWUR34135;AGIWUR42356
CB4856	CT	III	8333663	8334233	WBGene00022581	ZC262.4	ZC262.4	0.199613832536796	AGIWUR15636
CB4856	CT	III	8351249	8355174	WBGene00006840	unc-116	R05D3.7	-0.0683771679295573	AGIWUR1060;AGIWUR21329
CB4856	CT	III	8356887	8358844	WBGene00019881	R05D3.8	R05D3.8	0.0677034675571635	AGIWUR1460;AGIWUR10567
CB4856	CT	III	8358625	8362496	WBGene00007008	rfp-1	R05D3.4	0.137677028888247	AGIWUR37669
CB4856	CT	III	8362408	8368848	WBGene00019882	R05D3.9	R05D3.9	0.0746034270063198	AGIWUR23033;AGIWUR30286
CB4856	CT	III	8372255	8375175	WBGene00019877	R05D3.2	R05D3.2	0.0971055162260761	AGIWUR4020;AGIWUR13106;AGIWUR43656
CB4856	CT	III	8375300	8380833	WBGene00019883	met-2	R05D3.11	-0.137106789816254	AGIWUR27065
CB4856	CT	III	8380943	8387529	WBGene00019884	R05D3.12	R05D3.12	-0.0799501379829808	AGIWUR9424;AGIWUR40415
CB4856	CT	III	8388466	8389639	WBGene00022700	ZK353.4	ZK353.4	0.276170111512128	AGIWUR10980
CB4856	CT	III	8389882	8394424	WBGene00044677	ZK353.10	ZK353.10	-0.238075654143987	AGIWUR43758
CB4856	CT	III	8395985	8396727	WBGene00022699	ZK353.3	ZK353.3	0.0391008367747334	AGIWUR7042;AGIWUR10899;AGIWUR11298;AGIWUR16527;AGIWUR22949
CB4856	CT	III	8398711	8399240	WBGene00022698	ZK353.2	ZK353.2	0.166022759208952	AGIWUR43243
CB4856	CT	III	8399190	8401928	WBGene00002249	lap-1	ZK353.6	-0.0703801625783118	AGIWUR19824;AGIWUR21981;AGIWUR31549
CB4856	CT	III	8402049	8403607	WBGene00022702	cutc-1	ZK353.7	-0.0491413809088677	AGIWUR11611;AGIWUR15249;AGIWUR19716;AGIWUR22359;AGIWUR28520;AGIWUR30902;AGIWUR34334;AGIWUR35842;AGIWUR37404
CB4856	CT	III	8403715	8405562	WBGene00022703	ubxn-4	ZK353.8	-0.0623330275403582	AGIWUR17659
CB4856	CT	III	8405874	8409406	WBGene00022697	cyy-1	ZK353.1	-0.0302940634152034	AGIWUR158;AGIWUR1847;AGIWUR4594;AGIWUR6679;AGIWUR26066;AGIWUR33796
CB4856	CT	III	8409496	8412017	WBGene00022704	ZK353.9	ZK353.9	0.0995073603912395	AGIWUR9955
CB4856	CT	III	8416227	8422650	WBGene00023405	sor-1	ZK1236.3	-0.15749652988848	AGIWUR14064

CB4856	CT	III	8426334	8428583	WBGene00000414	cec-1	ZK1236.2	0.438232897384452	AGIWUR35155
CB4856	CT	III	8429307	8431586	WBGene00022862	ZK1236.1	ZK1236.1	0.231856353924103	AGIWUR10904
CB4856	CT	III	8431552	8432214	WBGene00022864	ZK1236.5	ZK1236.5	-0.059525622318974	AGIWUR44607
CB4856	CT	III	8432469	8435174	WBGene00044588	ZK1236.9	ZK1236.9	-0.0859247062361841	AGIWUR16787;AGIWUR29697
CB4856	CT	III	8436700	8437811	WBGene00023223	ZK1236.8	ZK1236.8	0.0963499362333051	AGIWUR5337;AGIWUR11160;AGIWUR24137;AGIWUR26002;AGIWUR37523
CB4856	CT	III	8438061	8439412	WBGene00004175	pqn-96	ZK1236.6	0.0926793742718601	AGIWUR25763
CB4856	CT	III	8439468	8443607	WBGene00022865	ufbp-1	ZK1236.7	0.09271532293711	AGIWUR21809;AGIWUR40300;AGIWUR43834
CB4856	CT	III	8443917	8446920	WBGene00016250	hsp-110	C30C11.4	-0.235036504671239	AGIWUR13298;AGIWUR24695;AGIWUR24876
CB4856	CT	III	8447353	8449346	WBGene00004460	rpn-3	C30C11.2	0.0977871718322056	AGIWUR40229
CB4856	CT	III	8449757	8450519	WBGene00016249	mrpl-32	C30C11.1	-0.232139002790792	AGIWUR42312
CB4856	CT	III	8467035	8478377	WBGene00018369	mig-39	F42H10.5	0.155439148021767	AGIWUR20369
CB4856	CT	III	8478382	8480455	WBGene00018370	F42H10.6	F42H10.6	-0.12592755463413	AGIWUR13388
CB4856	CT	III	8484153	8488365	WBGene00018367	F42H10.3	F42H10.3	-0.0870423587048927	AGIWUR11127;AGIWUR40428
CB4856	CT	III	8488344	8490347	WBGene00018371	ess-2	F42H10.7	-0.146704613461033	AGIWUR20825
CB4856	CT	III	8490624	8491097	WBGene00018366	F42H10.2	F42H10.2	-0.218089035928868	AGIWUR6373
CB4856	CT	III	8493878	8518879	WBGene00015418	pac-1	C04D8.1	-0.050263572733463	AGIWUR8499;AGIWUR21807
CB4856	CT	III	8518904	8519945	WBGene00022507	ZC21.8	ZC21.8	-0.153391185290253	AGIWUR36373
CB4856	CT	III	8525073	8528650	WBGene00022505	ZC21.3	ZC21.3	-0.261959520478501	AGIWUR6744;AGIWUR18379;AGIWUR21048;AGIWUR26702;AGIWUR33777;AGIWUR41319
CB4856	CT	III	8530077	8531123	WBGene00044443	ZC21.10	ZC21.10	-0.146954676636517	AGIWUR25900;AGIWUR30228;AGIWUR41931
CB4856	CT	III	8535131	8542748	WBGene00006614	trp-1	ZC21.2	0.0713608736524147	AGIWUR12908
CB4856	CT	III	8542952	8545608	WBGene00022506	ZC21.6	ZC21.6	0.209233199340861	AGIWUR43550

CB4856 CT III 8552555 8553839 WBGene00043097 C02D5.4 C02D5.4 0.549269275215629
AGIWUR11444;AGIWUR40140;AGIWUR42459
CB4856 CT III 8554772 8555821 WBGene00015337 gsto-2 C02D5.3 0.238459929445915
AGIWUR16755;AGIWUR36587;AGIWUR38455
CB4856 CT III 8557335 8559194 WBGene00015336 C02D5.2 C02D5.2 -0.0243953166847675 AGIWUR27961
CB4856 CT III 8561136 8563026 WBGene00015335 acdh-6 C02D5.1 -0.167884595672222 AGIWUR2682
CB4856 CT III 8581102 8583928 WBGene00015519 doxa-1 C06E1.3 -0.340814476392248 AGIWUR20889
CB4856 CT III 8584186 8588642 WBGene00001612 glr-1 C06E1.4 -0.283525362980424 AGIWUR5066
CB4856 CT III 8589336 8589488 WBGene00015520 fip-3 C06E1.5 -0.312399721883958 AGIWUR5323
CB4856 CT III 8590833 8590982 WBGene00015521 fipr-16 C06E1.6 -0.215947429002758
AGIWUR15253;AGIWUR15387;AGIWUR16496;AGIWUR18026;AGIWUR21580;AGIWUR34124
CB4856 CT III 8591239 8593720 WBGene00015522 C06E1.7 C06E1.7 0.17344517966872 AGIWUR10879
CB4856 CT III 8595339 8597870 WBGene00015523 ztf-30 C06E1.8 0.431372080901649 AGIWUR16028
CB4856 CT III 8605832 8606622 WBGene00015518 C06E1.1 C06E1.1 0.203882992099898
AGIWUR2461;AGIWUR14714;AGIWUR20169;AGIWUR35713
CB4856 CT III 8609882 8614569 WBGene00015525 rha-2 C06E1.10 -0.235058330616274
AGIWUR2692;AGIWUR15564;AGIWUR31321
CB4856 CT III 8614762 8616381 WBGene00015526 C06E1.11 C06E1.11 -0.054394633688263
AGIWUR14844
CB4856 CT III 8616781 8626917 WBGene00015747 C13G5.2 C13G5.2 -0.300628848131916 AGIWUR17169
CB4856 CT III 8626291 8628571 WBGene00017696 polk-1 F22B7.6 -0.051565745688162
AGIWUR4510;AGIWUR37658;AGIWUR39072
CB4856 CT III 8631165 8633549 WBGene00001028 dnj-10 F22B7.5 -0.204774386757177
AGIWUR815;AGIWUR6507;AGIWUR26820;AGIWUR28613
CB4856 CT III 8634078 8643739 WBGene00006662 twk-7 F22B7.7 0.17927574083604
AGIWUR395;AGIWUR39525
CB4856 CT III 8648889 8649477 WBGene00017694 F22B7.3 F22B7.3 -0.242153141337561 AGIWUR43782
CB4856 CT III 8650764 8652190 WBGene00017693 flp-23 F22B7.2 0.0144410499696219
AGIWUR4182;AGIWUR16416;AGIWUR16449;AGIWUR17359;AGIWUR25271;AGIWUR29653;AGIWUR30956;AGIWUR37005;AGIWUR42211;
AGIWUR43285
CB4856 CT III 8652578 8653925 WBGene00017698 F22B7.9 F22B7.9 0.916843923406269
AGIWUR3277;AGIWUR19821;AGIWUR23277;AGIWUR25110;AGIWUR27887;AGIWUR28497
CB4856 CT III 8659872 8667482 WBGene00001078 dpy-19 F22B7.10 -0.131415701598321
AGIWUR34986
CB4856 CT III 8670543 8675051 WBGene00003993 pgl-2 B0523.3 -0.129623084872701 AGIWUR18234
CB4856 CT III 8675297 8683732 WBGene00001443 fli-1 B0523.5 -0.0526777672836438
AGIWUR4414;AGIWUR25594;AGIWUR32485
CB4856 CT III 8686118 8688457 WBGene00015125 B0303.3 B0303.3 -0.156836068738555
AGIWUR2262;AGIWUR10093;AGIWUR21243;AGIWUR36276

CB4856 CT III 8689850 8693473 WBGene00015126 B0303.4 B0303.4 0.0829211943850119 AGIWUR16137
CB4856 CT III 8693638 8699497 WBGene00015128 B0303.7 B0303.7 0.0230456941933674 AGIWUR39808
CB4856 CT III 8699481 8700333 WBGene00015129 sdz-1 B0303.8 -0.0658260925627392 AGIWUR26442
CB4856 CT III 8700927 8703341 WBGene00015130 vps-33.1 B0303.9 -0.195210841024672
AGIWUR13675
CB4856 CT III 8703300 8710410 WBGene00015131 B0303.11 B0303.11 -0.0204903358009982
AGIWUR39482
CB4856 CT III 8713993 8716184 WBGene00015132 B0303.14 B0303.14 -0.27425954476417
AGIWUR29492
CB4856 CT III 8717644 8718567 WBGene00015133 mrpl-11 B0303.15 0.077609350952244
AGIWUR38117;AGIWUR41229
CB4856 CT III 8720201 8725334 WBGene00022717 hipr-1 ZK370.3 -0.0876784388120111 AGIWUR1623
CB4856 CT III 8725323 8731373 WBGene00022718 ZK370.4 ZK370.4 0.165039014486545
AGIWUR393;AGIWUR13330;AGIWUR23503;AGIWUR25689;AGIWUR29681;AGIWUR34771;AGIWUR35829
CB4856 CT III 8737686 8741412 WBGene00022719 pdhk-2 ZK370.5 0.382116844465431 AGIWUR9815
CB4856 CT III 8742007 8743842 WBGene00022720 ZK370.6 ZK370.6 -0.168589947628759 AGIWUR11574
CB4856 CT III 8746216 8748701 WBGene00022721 ugtp-1 ZK370.7 -0.196829464788337 AGIWUR25896
CB4856 CT III 8749763 8757069 WBGene00004856 sma-2 ZK370.2 0.396405437555204 AGIWUR33081
CB4856 CT III 8756995 8761101 WBGene00019801 aak-1 PAR2.3 -0.0530295317794547
AGIWUR5774;AGIWUR7517;AGIWUR34094
CB4856 CT III 8763291 8767342 WBGene00003253 mig-22 PAR2.4 -0.214146642544425
AGIWUR7118;AGIWUR40525
CB4856 CT III 8768311 8769252 WBGene00019800 mtss-1 PAR2.1 -0.133302863767792 AGIWUR33250
CB4856 CT III 8769502 8776141 WBGene00019301 K02D10.1 K02D10.1 -0.0962424189689495
AGIWUR3144;AGIWUR5099;AGIWUR7470;AGIWUR11325;AGIWUR18329;AGIWUR25715;AGIWUR39553;AGIWUR42939
CB4856 CT III 8776183 8777895 WBGene00019305 snap-29 K02D10.5 -0.212566751181695
AGIWUR2012
CB4856 CT III 8777890 8780012 WBGene00019304 K02D10.4 K02D10.4 0.0670521032769749
AGIWUR11635;AGIWUR12464;AGIWUR16357
CB4856 CT III 8787092 8792346 WBGene00018833 ztf-1 F54F2.5 -0.225051006403996
AGIWUR12987;AGIWUR35878
CB4856 CT III 8792847 8806305 WBGene00006975 zfp-1 F54F2.2 0.152886494146654
AGIWUR16269;AGIWUR28175
CB4856 CT III 8806231 8807346 WBGene00018835 F54F2.7 F54F2.7 -0.0772992284910128 AGIWUR45086
CB4856 CT III 8807482 8809329 WBGene00004201 prx-19 F54F2.8 -0.0498259627586588 AGIWUR28794
CB4856 CT III 8818899 8825270 WBGene00003929 pat-2 F54F2.1 0.129358841344086 AGIWUR44985
CB4856 CT III 8825243 8827228 WBGene00018836 F54F2.9 F54F2.9 -0.382233413036374 AGIWUR21876
CB4856 CT III 8827358 8829305 WBGene00018417 F44E2.3 F44E2.3 -0.118618064326534
AGIWUR13313;AGIWUR22083

CB4856	CT	III	8829493	8831109	WBGene00018423	F44E2.10	F44E2.10	-0.376270328056739	AGIWUR14243
CB4856	CT	III	8832479	8838424	WBGene00018418	F44E2.4	F44E2.4	0.586962036087304	AGIWUR21160
CB4856	CT	III	8844494	8845214	WBGene00018419	F44E2.6	F44E2.6	-0.138224281367251	AGIWUR15400;AGIWUR16092;AGIWUR16254;AGIWUR16985;AGIWUR34165;AGIWUR38620
CB4856	CT	III	8845856	8848486	WBGene00018420	F44E2.7	F44E2.7	-0.163107668831437	AGIWUR16197;AGIWUR37883;AGIWUR40627
CB4856	CT	III	8848582	8849877	WBGene00018421	F44E2.8	F44E2.8	-0.14024390369098	AGIWUR26871
CB4856	CT	III	8849994	8850532	WBGene00018422	F44E2.9	F44E2.9	-0.289827355781744	AGIWUR30697
CB4856	CT	III	8852264	8864451	WBGene00004041	plg-1	F44E2.11	-0.646108507352702	AGIWUR5095;AGIWUR10027;AGIWUR20923;AGIWUR36446
CB4856	CT	III	8870099	8877128	WBGene00010475	K01F9.2	K01F9.2	0.784918730300634	AGIWUR30346
CB4856	CT	III	8882707	8885482	WBGene00014021	svop-1	ZK637.1	0.238863689303805	AGIWUR11249
CB4856	CT	III	8887751	8890143	WBGene00014022	ZK637.2	ZK637.2	0.467657884844252	AGIWUR45186
CB4856	CT	III	8890231	8894208	WBGene00014023	lnkn-1	ZK637.3	0.144002539748611	AGIWUR10243
CB4856	CT	III	8894209	8894691	WBGene00014024	ZK637.4	ZK637.4	0.0565341304860603	AGIWUR37235
CB4856	CT	III	8895082	8896654	WBGene00014025	asna-1	ZK637.5	0.00909315412664569	AGIWUR17798
CB4856	CT	III	8898622	8899530	WBGene00014987	ZK637.6	ZK637.6	-0.0927188271595369	AGIWUR11772;AGIWUR16118
CB4856	CT	III	8900237	8904160	WBGene00002998	lin-9	ZK637.7	0.009245488482442	AGIWUR7682;AGIWUR11606;AGIWUR14623
CB4856	CT	III	8905615	8911749	WBGene00006768	unc-32	ZK637.8	0.0402806950995078	AGIWUR30237;AGIWUR33688;AGIWUR37194
CB4856	CT	III	8912144	8913632	WBGene00014027	tpk-1	ZK637.9	-0.00657117156886144	AGIWUR23783;AGIWUR40284;AGIWUR42847
CB4856	CT	III	8913769	8916450	WBGene00014028	trxr-2	ZK637.10	-0.151548592072499	AGIWUR2437
CB4856	CT	III	8916351	8917929	WBGene00000388	cdc-25.3	ZK637.11	-0.407650715373222	AGIWUR1926
CB4856	CT	III	8922292	8925302	WBGene00014029	ZK637.12	ZK637.12	0.041905072155739	AGIWUR11063
CB4856	CT	III	8925607	8926529	WBGene00014038	ZK643.7	ZK643.7	0.000753513084105428	AGIWUR30593
CB4856	CT	III	8930036	8936682	WBGene00014033	arrd-15	ZK643.1	-0.148900430325043	AGIWUR3131
CB4856	CT	III	8944686	8952953	WBGene00014035	seb-2	ZK643.3	0.117999660808312	AGIWUR21977;AGIWUR41246;AGIWUR42198
CB4856	CT	III	8952886	8956216	WBGene00014036	ZK643.5	ZK643.5	-0.104156943815017	AGIWUR1264
CB4856	CT	III	8956459	8957731	WBGene00014034	ZK643.2	ZK643.2	-0.315221066315583	AGIWUR7832
CB4856	CT	III	8957704	8960693	WBGene00014037	ZK643.6	ZK643.6	0.0722259601802842	AGIWUR15236
CB4856	CT	III	8962295	8965638	WBGene00001734	grl-25	ZK643.8	-0.274943078334777	AGIWUR45029

CB4856	CT	III	8966022	8967727	WBGene00011142	R08D7.1	R08D7.1	-0.199745017355692	AGIWUR42741
CB4856	CT	III	8967917	8969658	WBGene00011143	R08D7.2	R08D7.2	-0.0897431703603182	AGIWUR1150
CB4856	CT	III	8969638	8971578	WBGene00001227	eif-3.D	R08D7.3	-0.0307766494494341	AGIWUR1208;AGIWUR4378;AGIWUR22575;AGIWUR33307;AGIWUR34559;AGIWUR43455
CB4856	CT	III	8971892	8973672	WBGene00011144	R08D7.4	R08D7.4	0.00177131638936867	AGIWUR13868;AGIWUR41913
CB4856	CT	III	8974126	8975061	WBGene00011145	R08D7.5	R08D7.5	0.0177644429206758	AGIWUR41124
CB4856	CT	III	8975070	8981897	WBGene00011146	pde-2	R08D7.6	-0.0202638796529238	AGIWUR20156;AGIWUR22060;AGIWUR24440;AGIWUR33393
CB4856	CT	III	8992205	8994827	WBGene00011147	R08D7.7	R08D7.7	0.104659683225476	AGIWUR24736
CB4856	CT	III	8996418	8999785	WBGene00010308	F59B2.3	F59B2.3	-0.0509831251027348	AGIWUR2749;AGIWUR3789;AGIWUR8329;AGIWUR22566;AGIWUR25535;AGIWUR35192
CB4856	CT	III	8999828	9001973	WBGene00010309	rpn-6.2	F59B2.5	0.234130775498981	AGIWUR44151
CB4856	CT	III	9002779	9004839	WBGene00006977	zif-1	F59B2.6	-0.0974584593795598	AGIWUR43029
CB4856	CT	III	9004821	9005955	WBGene00004269	rab-6.1	F59B2.7	-0.211107403073349	AGIWUR5493;AGIWUR6537;AGIWUR18805;AGIWUR36400;AGIWUR44152
CB4856	CT	III	9007974	9010121	WBGene00010310	F59B2.8	F59B2.8	0.969571276805722	AGIWUR43386
CB4856	CT	III	9010676	9012409	WBGene00010311	F59B2.9	F59B2.9	0.326148958501548	AGIWUR8241;AGIWUR25616;AGIWUR28441
CB4856	CT	III	9013003	9016961	WBGene00010313	F59B2.11	F59B2.11	-0.0218559491785717	AGIWUR5273;AGIWUR14502;AGIWUR15535
CB4856	CT	III	9017607	9020960	WBGene00010314	F59B2.12	F59B2.12	-0.357323673963178	AGIWUR35085
CB4856	CT	III	9021370	9022635	WBGene00050931	F59B2.14	F59B2.14	-0.0390997696768167	AGIWUR25458;AGIWUR30439;AGIWUR39247
CB4856	CT	III	9023964	9027044	WBGene00010315	F59B2.13	F59B2.13	-0.120642756809595	AGIWUR39438
CB4856	CT	III	9038779	9043300	WBGene00003518	nac-2	R107.1	0.219364304523605	AGIWUR17074
CB4856	CT	III	9043886	9045938	WBGene00011298	R107.2	R107.2	0.0344042015499575	AGIWUR29941
CB4856	CT	III	9046055	9050693	WBGene00011299	ikke-1	R107.4	0.094744682869802	AGIWUR24852;AGIWUR26951
CB4856	CT	III	9051592	9053793	WBGene00011300	R107.5	R107.5	-0.332228267070503	AGIWUR19646;AGIWUR26520
CB4856	CT	III	9055052	9058882	WBGene00000549	cls-2	R107.6	0.103127794898356	AGIWUR39897
CB4856	CT	III	9059314	9060181	WBGene00001749	gst-1	R107.7	-0.138254960400229	AGIWUR24754
CB4856	CT	III	9060096	9071760	WBGene00003001	lin-12	R107.8	0.463336403056337	AGIWUR31911
CB4856	CT	III	9074872	9075639	WBGene00044406	F02A9.7	F02A9.7	0.0937190213191439	AGIWUR800
CB4856	CT	III	9075726	9077804	WBGene00008513	F02A9.1	F02A9.1	0.111889691453148	AGIWUR6275;AGIWUR14081;AGIWUR19721;AGIWUR31244;AGIWUR37134

CB4856 CT III 9080371 9081179 WBGene00001385 far-1 F02A9.2 -0.0816083453095402
AGIWUR9075;AGIWUR11257;AGIWUR19678;AGIWUR20921;AGIWUR23169;AGIWUR26703;AGIWUR42791
CB4856 CT III 9081679 9082511 WBGene00001386 far-2 F02A9.3 -0.00518242626671928
AGIWUR6957;AGIWUR13264;AGIWUR15268;AGIWUR31229
CB4856 CT III 9084306 9089386 WBGene00008514 F02A9.4 F02A9.4 -0.156522683124837
AGIWUR2606;AGIWUR4621;AGIWUR4931;AGIWUR5859;AGIWUR8562;AGIWUR27392;AGIWUR31943;AGIWUR43479
CB4856 CT III 9092241 9099698 WBGene00001609 glp-1 F02A9.6 0.259160263302787 AGIWUR41673
CB4856 CT III 9101367 9103095 WBGene00013979 ZK507.3 ZK507.3 0.0706631124706965 AGIWUR8624
CB4856 CT III 9103943 9106805 WBGene00013980 dos-1 ZK507.4 0.0185226432731511
AGIWUR121;AGIWUR22323;AGIWUR25063
CB4856 CT III 9106702 9109272 WBGene00000863 cya-1 ZK507.6 0.0456308896591118 AGIWUR31862
CB4856 CT III 9115082 9117270 WBGene00013982 ZK512.1 ZK512.1 0.765729283377523 AGIWUR11510
CB4856 CT III 9117349 9118364 WBGene00001720 grl-11 ZK512.9 -0.184445190388938 AGIWUR20150
CB4856 CT III 9119559 9125153 WBGene00000425 ced-11 ZK512.3 0.21856486533998 AGIWUR9162
CB4856 CT III 9125641 9130807 WBGene00013985 sec-16 ZK512.5 0.0585113907993229
AGIWUR8065;AGIWUR12160;AGIWUR25926;AGIWUR42645
CB4856 CT III 9130844 9131360 WBGene00013988 ZK512.10 ZK512.10 0.18495127603288
AGIWUR14773
CB4856 CT III 9136916 9141822 WBGene00001135 eat-4 ZK512.6 -0.249653580154712 AGIWUR15306
CB4856 CT III 9141789 9144794 WBGene00013983 ZK512.2 ZK512.2 0.0314790129813503
AGIWUR2192;AGIWUR3650;AGIWUR7066
CB4856 CT III 9145325 9145976 WBGene00013984 ZK512.4 ZK512.4 -0.180276576189476 AGIWUR25270
CB4856 CT III 9146031 9147411 WBGene00013987 ZK512.8 ZK512.8 0.00307763110250825 AGIWUR44998
CB4856 CT III 9147395 9153909 WBGene00003597 nhl-1 F54G8.4 0.102900451926843 AGIWUR7367
CB4856 CT III 9155116 9157165 WBGene00010074 irld-34 F54G8.1 0.094519973484724 AGIWUR18148
CB4856 CT III 9157243 9161653 WBGene00004223 ptr-9 F54G8.5 0.368157528152789 AGIWUR15185
CB4856 CT III 9163077 9168027 WBGene00000960 dgk-3 F54G8.2 0.031278230474868 AGIWUR30352
CB4856 CT III 9168040 9172802 WBGene00002081 ina-1 F54G8.3 -0.0639340428713222 AGIWUR3362
CB4856 CT III 9181550 9185649 WBGene00006526 tax-4 ZC84.2 0.0397556499794973 AGIWUR15160
CB4856 CT III 9186077 9192951 WBGene00013850 ZC84.7 ZC84.7 0.160423601311256 AGIWUR31325
CB4856 CT III 9192983 9199507 WBGene00013849 ZC84.6 ZC84.6 0.495233027310569 AGIWUR6565
CB4856 CT III 9199450 9206285 WBGene00013846 ZC84.1 ZC84.1 0.0721870400744122
AGIWUR1559;AGIWUR10301;AGIWUR12527;AGIWUR22077
CB4856 CT III 9206966 9207854 WBGene00014980 ZC84.5 ZC84.5 0.197583086805194 AGIWUR15239
CB4856 CT III 9208288 9215440 WBGene00013848 npr-29 ZC84.4 0.00565881677864866 AGIWUR25356
CB4856 CT III 9225374 9228786 WBGene00004391 rnr-1 T23G5.1 -0.115236798528166
AGIWUR21235;AGIWUR28652;AGIWUR34624
CB4856 CT III 9229235 9231210 WBGene00011963 T23G5.3 T23G5.3 0.323620967731536 AGIWUR12934
CB4856 CT III 9231421 9236322 WBGene00011962 T23G5.2 T23G5.2 -0.199361662322975

AGIWUR21176;AGIWUR33460;AGIWUR36304
CB4856 CT III 9240337 9242514 WBGene00011964 saeg-2 T23G5.6 0.0333008526328502
AGIWUR6898;AGIWUR13321;AGIWUR38443
CB4856 CT III 9242914 9246802 WBGene00000934 dat-1 T23G5.5 0.129307002607715 AGIWUR22310
CB4856 CT III 9247422 9248618 WBGene00011365 T02C1.1 T02C1.1 -0.0199172926506108 AGIWUR26963
CB4856 CT III 9251029 9253126 WBGene00011366 T02C1.2 T02C1.2 -0.0103577726008215 AGIWUR13854
CB4856 CT III 9257259 9260222 WBGene00010795 M01A8.1 M01A8.1 0.196743226521609
AGIWUR13143;AGIWUR15928;AGIWUR16043;AGIWUR38473;AGIWUR38524;AGIWUR45013
CB4856 CT III 9260191 9269062 WBGene00010796 clip-1 M01A8.2 0.0618720208698704
AGIWUR9838;AGIWUR11840;AGIWUR19871
CB4856 CT III 9281785 9296668 WBGene00010453 fozi-1 K01B6.1 0.320209538313224 AGIWUR1508
CB4856 CT III 9296627 9297394 WBGene00010455 K01B6.4 K01B6.4 -0.0121529929849142 AGIWUR5362
CB4856 CT III 9300219 9303928 WBGene00010454 K01B6.3 K01B6.3 -0.2786605386656 AGIWUR27244
CB4856 CT III 9304034 9306696 WBGene00005936 srx-45 K01B6.2 0.170703102926369
AGIWUR8476;AGIWUR18281;AGIWUR39530
CB4856 CT III 9314714 9317365 WBGene00008043 C40H1.8 C40H1.8 -0.261914809972364 AGIWUR23496
CB4856 CT III 9318044 9319271 WBGene00008038 C40H1.2 C40H1.2 -0.170043411034736 AGIWUR40443
CB4856 CT III 9320040 9321663 WBGene00008044 C40H1.9 C40H1.9 0.153017058143647 AGIWUR14266
CB4856 CT III 9322316 9324733 WBGene00000770 cpb-1 C40H1.1 0.0953918380402303 AGIWUR13573
CB4856 CT III 9325048 9327031 WBGene00008042 C40H1.7 C40H1.7 0.316824108997338 AGIWUR16820
CB4856 CT III 9327307 9329366 WBGene00008039 C40H1.3 C40H1.3 0.3560006038723 AGIWUR27686
CB4856 CT III 9330316 9330928 WBGene00008040 ttr-5 C40H1.5 0.718923278938862 AGIWUR40524
CB4856 CT III 9331514 9332284 WBGene00008041 ufc-1 C40H1.6 0.0959095315812171 AGIWUR41584
CB4856 CT III 9332269 9334274 WBGene00001242 elo-4 C40H1.4 -0.125155871439709 AGIWUR23388
CB4856 CT III 9336881 9344447 WBGene00001263 emb-9 K04H4.1 0.169488429038626
AGIWUR9539;AGIWUR9984
CB4856 CT III 9347714 9355480 WBGene00010573 K04H4.2 K04H4.2 0.392766159319166
AGIWUR7753;AGIWUR12418;AGIWUR14842;AGIWUR21373;AGIWUR22356;AGIWUR23321;AGIWUR25593;AGIWUR36790;AGIWUR39493;
AGIWUR40643;AGIWUR41480;AGIWUR42869;AGIWUR43596
CB4856 CT III 9360269 9361333 WBGene00010574 K04H4.5 K04H4.5 0.121960558685714 AGIWUR28137
CB4856 CT III 9361528 9365380 WBGene00010575 flp-25 K04H4.7 -0.149567753419237
AGIWUR2661;AGIWUR28217;AGIWUR28904;AGIWUR38699
CB4856 CT III 9367238 9370385 WBGene00000799 crn-6 K04H4.6 0.240737795556649
AGIWUR3728;AGIWUR24581
CB4856 CT III 9383796 9387605 WBGene00006576 tkr-1 C38C10.1 -0.0808364573599132
AGIWUR1367
CB4856 CT III 9387601 9390107 WBGene00008000 slc-17.2 C38C10.2 -0.121016111605044
AGIWUR8053;AGIWUR12304;AGIWUR18680;AGIWUR21362;AGIWUR22003;AGIWUR28566
CB4856 CT III 9393981 9404532 WBGene00004343 rgr-1 C38C10.5 0.157959665547333

AGIWUR31939
CB4856 CT III 9404484 9406641 WBGene00012059 T26G10.1 T26G10.1 -0.0838737305863457
AGIWUR39909
CB4856 CT III 9412854 9413440 WBGene00012060 T26G10.3 T26G10.3 -0.23741021079693
AGIWUR18817
CB4856 CT III 9420531 9421574 WBGene00012061 T26G10.4 T26G10.4 0.107363520145187
AGIWUR40905
CB4856 CT III 9425536 9426674 WBGene00012062 T26G10.5 T26G10.5 0.203943575904242
AGIWUR17340
CB4856 CT III 9434057 9435100 WBGene00010036 cpar-1 F54C8.2 -0.257381094254402 AGIWUR14509
CB4856 CT III 9435083 9441539 WBGene00001284 emb-30 F54C8.3 -0.0387110693429022
AGIWUR12925;AGIWUR43849
CB4856 CT III 9441557 9443634 WBGene00010037 F54C8.4 F54C8.4 -0.0412078310823043
AGIWUR12673;AGIWUR18539;AGIWUR19722;AGIWUR26404
CB4856 CT III 9444360 9449931 WBGene00010039 F54C8.6 F54C8.6 0.273085707371233 AGIWUR35169
CB4856 CT III 9451595 9454062 WBGene00010040 F54C8.7 F54C8.7 -0.0327141646871247 AGIWUR30464
CB4856 CT III 9454163 9456030 WBGene00010038 rheb-1 F54C8.5 -0.271310114227083 AGIWUR34422
CB4856 CT III 9456563 9463004 WBGene00004980 spk-1 B0464.5 -0.102629159325225
AGIWUR2559;AGIWUR3910;AGIWUR29612
CB4856 CT III 9462989 9465965 WBGene00007186 B0464.6 B0464.6 -0.0603961401255702
AGIWUR956;AGIWUR34359
CB4856 CT III 9465767 9467177 WBGene00007188 B0464.9 B0464.9 0.356703187894908 AGIWUR10494
CB4856 CT III 9467464 9468058 WBGene00000235 baf-1 B0464.7 -0.0514381489892166
AGIWUR652;AGIWUR2228;AGIWUR3386;AGIWUR20361;AGIWUR21114;AGIWUR22416;AGIWUR27119;AGIWUR28155;AGIWUR29069;AGI
WUR31986;AGIWUR33673
CB4856 CT III 9468157 9469899 WBGene00007187 tag-342 B0464.8 -0.0573936398331059
AGIWUR1236;AGIWUR26705
CB4856 CT III 9469920 9485451 WBGene00000268 bre-3 B0464.4 0.380373483950072 AGIWUR9948
CB4856 CT III 9488731 9489188 WBGene00007185 nlp-36 B0464.3 -0.439253153432354 AGIWUR14705
CB4856 CT III 9489176 9491236 WBGene00001094 dars-1 B0464.1 -0.148902793525876
AGIWUR10366;AGIWUR28818;AGIWUR37587
CB4856 CT III 9491640 9497044 WBGene00007184 ctr-9 B0464.2 -0.0655995653500883
AGIWUR4327;AGIWUR4818;AGIWUR5388;AGIWUR16073;AGIWUR26585;AGIWUR26624
CB4856 CT III 9497019 9500202 WBGene00004933 sod-4 F55H2.1 0.34131824443552
AGIWUR16498;AGIWUR25425;AGIWUR32411;AGIWUR40264;AGIWUR40842
CB4856 CT III 9504764 9507081 WBGene00003067 lrg-1 F55H2.4 -0.150039040814476
AGIWUR20208;AGIWUR34899
CB4856 CT III 9508074 9509806 WBGene00010130 vha-14 F55H2.2 -0.269899650352842
AGIWUR3972;AGIWUR25785;AGIWUR39739

CB4856 CT III 9511470 9513375 WBGene00010131 F55H2.5 F55H2.5 -0.107354897987589
AGIWUR15384;AGIWUR16468;AGIWUR17057;AGIWUR18183;AGIWUR21757;AGIWUR32281;AGIWUR39797
CB4856 CT III 9513348 9516248 WBGene00010132 F55H2.7 F55H2.7 -0.397212826146522 AGIWUR21357
CB4856 CT III 9516631 9521460 WBGene00000550 clu-1 F55H2.6 -0.211762178710961
AGIWUR6012;AGIWUR12781;AGIWUR24985;AGIWUR25703
CB4856 CT III 9522075 9525437 WBGene00014218 ZK1098.1 ZK1098.1 -0.0204558520492988
AGIWUR40242
CB4856 CT III 9525552 9527068 WBGene00014222 trpp-3 ZK1098.5 -0.147451166261787
AGIWUR27234;AGIWUR28384;AGIWUR35407;AGIWUR36975
CB4856 CT III 9527151 9528689 WBGene00014223 ZK1098.6 ZK1098.6 -0.114787972127454
AGIWUR23405
CB4856 CT III 9529166 9531888 WBGene00014219 ZK1098.2 ZK1098.2 -0.136280759888237
AGIWUR25200
CB4856 CT III 9532427 9538700 WBGene00014220 ZK1098.3 ZK1098.3 -0.17418947513074
AGIWUR7676;AGIWUR9497;AGIWUR42452
CB4856 CT III 9539031 9542380 WBGene00003504 mut-7 ZK1098.8 -0.193187015009897
AGIWUR25701
CB4856 CT III 9543069 9544404 WBGene00014221 eif-2Balpha ZK1098.4 -0.358913500538147
AGIWUR18997
CB4856 CT III 9545120 9545897 WBGene00194691 ZK1098.12 ZK1098.12 0.124881773343944
AGIWUR12008
CB4856 CT III 9546258 9556514 WBGene00006755 unc-16 ZK1098.10 0.091430083126081
AGIWUR5621;AGIWUR33196;AGIWUR42349
CB4856 CT III 9556507 9557454 WBGene00014226 ZK1098.11 ZK1098.11 -0.0964927662016031
AGIWUR25774
CB4856 CT III 9557448 9558563 WBGene00008169 C48B4.6 C48B4.6 -0.150039018996507 AGIWUR1145
CB4856 CT III 9558669 9559427 WBGene00008170 C48B4.7 C48B4.7 0.208460575950536 AGIWUR8640
CB4856 CT III 9559831 9561258 WBGene00008171 C48B4.8 C48B4.8 -0.020205594342892 AGIWUR29752
CB4856 CT III 9562336 9563335 WBGene00008172 C48B4.9 C48B4.9 -0.0320495505356373 AGIWUR12846
CB4856 CT III 9563437 9564510 WBGene00008173 C48B4.10 C48B4.10 -0.366682746672861
AGIWUR29101
CB4856 CT III 9564605 9566115 WBGene00008174 C48B4.11 C48B4.11 -0.235153757067067
AGIWUR9676
CB4856 CT III 9568751 9570460 WBGene00008176 C48B4.13 C48B4.13 -0.133752459608441
AGIWUR5852
CB4856 CT III 9570394 9580081 WBGene00000421 ced-7 C48B4.4 0.0701157861245356
AGIWUR15134;AGIWUR15652;AGIWUR41526
CB4856 CT III 9581220 9582361 WBGene00008168 C48B4.3 C48B4.3 0.579941806963447 AGIWUR39224
CB4856 CT III 9586617 9592192 WBGene00008167 acox-1.5 C48B4.1 0.380118453190001

AGIWUR40965
CB4856 CT III 9592282 9595667 WBGene00004401 rom-2 C48B4.2 -0.425372472480992 AGIWUR30006
CB4856 CT III 9597359 9598361 WBGene00008175 C48B4.12 C48B4.12 -0.152037595253134
AGIWUR33971
CB4856 CT III 9598475 9600049 WBGene00010226 F58A4.1 F58A4.1 -0.227686093930632 AGIWUR44129
CB4856 CT III 9600038 9600745 WBGene00010227 F58A4.2 F58A4.2 -0.121459133749489 AGIWUR17790
CB4856 CT III 9603030 9608688 WBGene00010228 clec-161 F58A4.5 -0.125252028683303
AGIWUR38854
CB4856 CT III 9609568 9612191 WBGene00043992 bbs-4 F58A4.14 0.170173456000177
AGIWUR13700;AGIWUR30878
CB4856 CT III 9614425 9615332 WBGene00010229 F58A4.6 F58A4.6 0.0430740347581402 AGIWUR11369
CB4856 CT III 9615306 9616470 WBGene00001831 hcp-3 F58A4.3 -0.243768092662101 AGIWUR41202
CB4856 CT III 9619711 9626537 WBGene00001955 hlh-11 F58A4.7 0.130937443464207
AGIWUR1204;AGIWUR18661;AGIWUR29302
CB4856 CT III 9626532 9629154 WBGene00006540 tbg-1 F58A4.8 -0.0309587032211898
AGIWUR5583;AGIWUR6671;AGIWUR8319;AGIWUR23541;AGIWUR24863;AGIWUR27460;AGIWUR31856;AGIWUR39229;AGIWUR39539
CB4856 CT III 9629246 9629917 WBGene00010230 rpac-19 F58A4.9 -0.0955319327342616
AGIWUR15916;AGIWUR37629
CB4856 CT III 9630085 9631167 WBGene00006704 ubc-7 F58A4.10 0.0165506627201459
AGIWUR18406;AGIWUR20044;AGIWUR40951;AGIWUR43784
CB4856 CT III 9631994 9637116 WBGene00001570 gei-13 F58A4.11 0.551024255069697
AGIWUR30688
CB4856 CT III 9650422 9651971 WBGene00007610 C15H7.3 C15H7.3 0.0420677723618956 AGIWUR41659
CB4856 CT III 9652313 9653641 WBGene00014678 C15H7.2 C15H7.2 -0.0930281488754696 AGIWUR3998
CB4856 CT III 9654409 9655859 WBGene00007611 C15H7.4 C15H7.4 -0.117121253831409 AGIWUR12873
CB4856 CT III 9663290 9667640 WBGene00007405 C07A9.9 C07A9.9 0.10109744776752
AGIWUR2793;AGIWUR21425;AGIWUR40053;AGIWUR40663
CB4856 CT III 9676320 9679051 WBGene00007402 ugt-60 C07A9.6 -0.584527259731409 AGIWUR2544
CB4856 CT III 9679698 9681269 WBGene00044227 C07A9.13 C07A9.13 0.450502071228646
AGIWUR9129
CB4856 CT III 9681524 9682829 WBGene00007401 C07A9.5 C07A9.5 0.06852303228455
AGIWUR35061;AGIWUR41297
CB4856 CT III 9686990 9690253 WBGene00007404 best-5 C07A9.8 1.71290077563258 AGIWUR16656
CB4856 CT III 9691030 9694572 WBGene00007403 set-3 C07A9.7 -0.0802571470534232
AGIWUR6874;AGIWUR16066
CB4856 CT III 9694668 9696517 WBGene00007400 C07A9.2 C07A9.2 -0.137756295790616
AGIWUR2106;AGIWUR8528;AGIWUR23710;AGIWUR30448;AGIWUR44233
CB4856 CT III 9699876 9710268 WBGene00006579 tlk-1 C07A9.3 0.170803326243686
AGIWUR28026;AGIWUR28045;AGIWUR37049

CB4856	CT	III	9710312	9713928	WBGene00003571	ncx-6	C07A9.4	0.106897546525243	AGIWUR5692
CB4856	CT	III	9715883	9720291	WBGene00003572	ncx-7	C07A9.11	-0.229393653288692	AGIWUR41131
CB4856	CT	III	9723282	9725214	WBGene00007399	clec-162	C07A9.1	-0.0758186781844197	AGIWUR18;AGIWUR21283;AGIWUR43658
CB4856	CT	III	9725285	9727539	WBGene00043994	C07A9.12	C07A9.12	0.351951764086297	AGIWUR23015;AGIWUR42131
CB4856	CT	III	9735265	9736057	WBGene00000932	dao-6	T05G5.12	0.186320886137014	AGIWUR8538;AGIWUR16163;AGIWUR36321
CB4856	CT	III	9736030	9736869	WBGene00004097	pqm-96	T05G5.11	-0.0106081871156753	AGIWUR4087;AGIWUR11001;AGIWUR19161;AGIWUR25569;AGIWUR34670
CB4856	CT	III	9737469	9738839	WBGene00001951	hlh-4	T05G5.2	0.149635563830703	AGIWUR41294
CB4856	CT	III	9745864	9747193	WBGene00002064	iff-1	T05G5.10	-0.143624439995765	AGIWUR21180
CB4856	CT	III	9747432	9748852	WBGene00000405	cdk-1	T05G5.3	-0.120725648909669	AGIWUR18204;AGIWUR25840;AGIWUR26140
CB4856	CT	III	9748863	9749673	WBGene00011499	T05G5.4	T05G5.4	0.326182623326908	AGIWUR44760
CB4856	CT	III	9751440	9752922	WBGene00001155	ech-6	T05G5.6	-0.487104918923645	AGIWUR4728;AGIWUR22276;AGIWUR32179;AGIWUR36972;AGIWUR39062
CB4856	CT	III	9753672	9757514	WBGene00011502	vps-53	T05G5.8	-0.251708410724333	AGIWUR12730
CB4856	CT	III	9757616	9762143	WBGene00011503	gcc-2	T05G5.9	-0.12649307632449	AGIWUR26271
CB4856	CT	III	9766516	9774825	WBGene00000366	cbp-1	R10E11.1	0.0744088227605344	AGIWUR3680;AGIWUR20510;AGIWUR23302;AGIWUR36765
CB4856	CT	III	9776532	9778850	WBGene00011216	usp-46	R10E11.3	-0.156681114355276	AGIWUR828;AGIWUR9155;AGIWUR27956;AGIWUR35299
CB4856	CT	III	9778782	9780350	WBGene00005021	sqv-3	R10E11.4	0.00389138330452177	AGIWUR4791
CB4856	CT	III	9781243	9782057	WBGene00006910	vha-1	R10E11.8	-0.100452659429301	AGIWUR12283;AGIWUR14952;AGIWUR20193;AGIWUR21407;AGIWUR26765;AGIWUR32083
CB4856	CT	III	9782164	9783212	WBGene00006911	vha-2	R10E11.2	-0.0304093234972971	AGIWUR28465
CB4856	CT	III	9783325	9786038	WBGene00011218	R10E11.6	R10E11.6	0.0228806797408518	AGIWUR4535;AGIWUR8860
CB4856	CT	III	9785957	9789657	WBGene00011217	R10E11.5	R10E11.5	-0.330419353309813	AGIWUR32181;AGIWUR38562;AGIWUR42235
CB4856	CT	III	9792548	9793642	WBGene00011220	R10E11.9	R10E11.9	0.0655740083594525	AGIWUR44902
CB4856	CT	III	9794367	9798643	WBGene00003158	mcm-6	ZK632.1	-0.068581963428394	AGIWUR3400;AGIWUR44759

CB4856	CT	III	9799107	9802333	WBGene00014011	ZK632.2	ZK632.2	-0.126931994683336	AGIWUR31557
CB4856	CT	III	9802354	9805093	WBGene00014012	riok-3	ZK632.3	0.000197968117412017	AGIWUR17835
CB4856	CT	III	9805515	9807454	WBGene00014013	ZK632.4	ZK632.4	-0.0168011331574931	AGIWUR36478
CB4856	CT	III	9808544	9812639	WBGene00014014	ZK632.5	ZK632.5	-0.14944675878758	AGIWUR40919
CB4856	CT	III	9813446	9814424	WBGene00014020	ZK632.14	ZK632.14	-0.0838255182990308	AGIWUR10501;AGIWUR29646;AGIWUR30662;AGIWUR44427
CB4856	CT	III	9814364	9817137	WBGene00000567	cnx-1	ZK632.6	0.23281698958737	AGIWUR9035
CB4856	CT	III	9819294	9823120	WBGene00014015	panl-3	ZK632.7	0.0571786955286946	AGIWUR30337
CB4856	CT	III	9823242	9824132	WBGene00000189	arl-5	ZK632.8	0.0519843448762352	AGIWUR25912
CB4856	CT	III	9824877	9825445	WBGene00014016	ZK632.9	ZK632.9	0.114172938983189	AGIWUR16999;AGIWUR17140;AGIWUR17442;AGIWUR20289;AGIWUR29675;AGIWUR30283
CB4856	CT	III	9826130	9827178	WBGene00014017	ZK632.10	ZK632.10	0.213044440284726	AGIWUR30752
CB4856	CT	III	9827915	9829719	WBGene00014018	ZK632.11	ZK632.11	-0.393495509328346	AGIWUR24674
CB4856	CT	III	9830792	9832430	WBGene00014019	ZK632.12	ZK632.12	0.094683959512416	AGIWUR42050
CB4856	CT	III	9835192	9838311	WBGene00185002	K11H3.8	K11H3.8	-0.0297753337693027	AGIWUR26726
CB4856	CT	III	9843782	9845733	WBGene00010782	K11H3.5	K11H3.5	0.242140422206103	AGIWUR35882
CB4856	CT	III	9846393	9847521	WBGene00010779	K11H3.2	K11H3.2	-0.213866269503305	AGIWUR4923
CB4856	CT	III	9847626	9848116	WBGene00010783	mrpl-36	K11H3.6	0.13281322766414	AGIWUR40294
CB4856	CT	III	9851854	9854102	WBGene00010780	K11H3.3	K11H3.3	0.090060118341885	AGIWUR32929
CB4856	CT	III	9854077	9859183	WBGene00010778	gpdh-2	K11H3.1	0.031965502792012	AGIWUR18384;AGIWUR23070
CB4856	CT	III	9860730	9863302	WBGene00010784	twk-48	K11H3.7	-0.0155473725904578	AGIWUR9189;AGIWUR22564;AGIWUR37335
CB4856	CT	III	9863251	9867786	WBGene00014075	dhhc-4	ZK757.4	-0.237061809835012	AGIWUR2140;AGIWUR3995;AGIWUR20382;AGIWUR33633
CB4856	CT	III	9868594	9871124	WBGene00014073	ZK757.1	ZK757.1	0.00323011905531878	AGIWUR24918
CB4856	CT	III	9874142	9877476	WBGene00014074	ZK757.2	ZK757.2	0.265502653727638	AGIWUR40144
CB4856	CT	III	9887210	9891458	WBGene00006449	alg-4	ZK757.3	0.0364785792805291	AGIWUR21301;AGIWUR22137
CB4856	CT	III	9893155	9898426	WBGene00009593	mam-7	F40F12.4	0.17928105369258	AGIWUR30722
CB4856	CT	III	9898602	9910658	WBGene00009594	cyld-1	F40F12.5	0.192140909821262	AGIWUR11679;AGIWUR20472
CB4856	CT	III	9913021	9914075	WBGene00043995	F40F12.9	F40F12.9	0.536887690979757	AGIWUR3535;AGIWUR7077;AGIWUR10893;AGIWUR13925;AGIWUR16426;AGIWUR21462;AGIWUR23195;AGIWUR30134;AGIWUR31059;AGIWUR34499

CB4856	CT	III	9918645	9920864	WBGene00009592	F40F12.3	F40F12.3	0.0918762797052466	AGIWUR20178
CB4856	CT	III	9921200	9921892	WBGene00014755	F40F12.8	F40F12.8	0.0865921600311994	AGIWUR19378
CB4856	CT	III	9923174	9925443	WBGene00010545	cbp-2	K03H1.10	0.635684560732788	AGIWUR37099
CB4856	CT	III	9927732	9929004	WBGene00010539	ttr-2	K03H1.4	-0.229286213714719	AGIWUR29522
CB4856	CT	III	9929058	9930413	WBGene00010541	ttr-1	K03H1.6	0.0883073659269797	AGIWUR9772;AGIWUR14636;AGIWUR19049;AGIWUR34888
CB4856	CT	III	9932398	9939712	WBGene00010540	K03H1.5	K03H1.5	0.0251840247677357	AGIWUR34316
CB4856	CT	III	9940771	9941464	WBGene00010544	K03H1.9	K03H1.9	0.299107017024856	AGIWUR31829
CB4856	CT	III	9941624	9942345	WBGene00010547	K03H1.12	K03H1.12	0.0472991682512056	AGIWUR11705
CB4856	CT	III	9943243	9944823	WBGene00001603	gln-2	K03H1.1	-0.011407887319244	AGIWUR42968
CB4856	CT	III	9945395	9946663	WBGene00010543	K03H1.8	K03H1.8	-0.366783988889759	AGIWUR23294
CB4856	CT	III	9950381	9951964	WBGene00010546	K03H1.11	K03H1.11	-0.128386489234295	AGIWUR27779;AGIWUR29848;AGIWUR39811
CB4856	CT	III	9952056	9956271	WBGene00003389	mog-1	K03H1.2	-0.0602902032574484	AGIWUR16381
CB4856	CT	III	9957357	9963171	WBGene00007010	alx-1	R10E12.1	-0.00873638541744448	AGIWUR17080;AGIWUR21528;AGIWUR23807;AGIWUR38367;AGIWUR41652;AGIWUR44473
CB4856	CT	III	9963121	9973865	WBGene00011221	R10E12.2	R10E12.2	-0.0985429935867135	AGIWUR40690
CB4856	CT	III	9987367	9987728	WBGene00194886	C05B5.12	C05B5.12	0.0410576892631438	AGIWUR29906
CB4856	CT	III	9993885	9996235	WBGene00007321	C05B5.4	C05B5.4	0.52379468411346	AGIWUR11599
CB4856	CT	III	9996205	9997955	WBGene00004100	pqn-8	C05B5.3	-0.205242126732776	AGIWUR38225
CB4856	CT	III	9998542	9999335	WBGene00219314	C05B5.17	C05B5.17	0.112517167615053	AGIWUR44836
CB4856	CT	III	9999819	10000528	WBGene00007320	C05B5.2	C05B5.2	0.379852855042268	AGIWUR23685
CB4856	CT	III	10001400	10003856	WBGene00007322	C05B5.5	C05B5.5	0.0905667530116361	AGIWUR28548
CB4856	CT	III	10005459	10006784	WBGene00007324	C05B5.8	C05B5.8	0.0140337344865769	AGIWUR10471;AGIWUR21687;AGIWUR31806
CB4856	CT	III	10007966	10010177	WBGene00007323	fbxa-155	C05B5.6	0.0720277347941121	AGIWUR33096
CB4856	CT	III	10010843	10011065	WBGene00044130	C05B5.10	C05B5.10	-0.221761979578691	AGIWUR30379
CB4856	CT	III	10014188	10016558	WBGene00004344	rgs-1	C05B5.7	-0.0898297001724213

AGIWUR23156
CB4856	CT	III	10023804	10024120	WBGene00002104	ins-21	M04D8.1	-0.276718686822625
AGIWUR44786
CB4856	CT	III	10026289	10026673	WBGene00002105	ins-22	M04D8.2	0.330897456958338
AGIWUR4200
CB4856	CT	III	10028247	10028600	WBGene00002106	ins-23	M04D8.3	1.84330980036527
AGIWUR37017
CB4856	CT	III	10029991	10030600	WBGene00010862	M04D8.4	M04D8.4	-0.331754791787038
AGIWUR2895
CB4856	CT	III	10032344	10033313	WBGene00010863	M04D8.5	M04D8.5	0.175404906296891
AGIWUR3713;AGIWUR20119;AGIWUR22864;AGIWUR36190
CB4856	CT	III	10034181	10035102	WBGene00010866	M04D8.8	M04D8.8	-0.355963998305223
AGIWUR36738
CB4856	CT	III	10036004	10036796	WBGene00010864	xbx-3	M04D8.6	0.00923997936631604
AGIWUR28393
CB4856	CT	III	10037450	10038247	WBGene00010865	M04D8.7	M04D8.7	0.519263283934695
AGIWUR9287
CB4856	CT	III	10038458	10040113	WBGene00011808	T16G12.7	T16G12.7
-0.0434270513407586	AGIWUR12066;AGIWUR16783;AGIWUR24474
CB4856	CT	III	10040191	10049857	WBGene00011803	T16G12.1	T16G12.1
-0.583120266230363	AGIWUR9573;AGIWUR22085;AGIWUR24630
CB4856	CT	III	10055604	10056133	WBGene00044222	T16G12.10	T16G12.10
-0.432118318850828	AGIWUR15645;AGIWUR32508
CB4856	CT	III	10057822	10058684	WBGene00011805	T16G12.4	T16G12.4
0.15239433120915	AGIWUR43984
CB4856	CT	III	10059302	10061982	WBGene00011809	T16G12.8	T16G12.8
-0.221148613588186	AGIWUR26835
CB4856	CT	III	10064337	10069201	WBGene00011806	ekl-6	T16G12.5
-0.262992307133706	AGIWUR57
CB4856	CT	III	10069564	10075995	WBGene00011807	T16G12.6	T16G12.6
-0.101044050598585	AGIWUR23002
CB4856	CT	III	10076387	10078707	WBGene00011810	T16G12.9	T16G12.9
-0.235894846289311	AGIWUR25375
CB4856	CT	III	10081443	10083286	WBGene00011813	T16H12.3	T16H12.3
-0.235650403422241	AGIWUR17981
CB4856	CT	III	10085403	10086064	WBGene00194725	T16H12.13	T16H12.13
-0.204331021169859	AGIWUR21108
CB4856	CT	III	10086303	10087171	WBGene00011812	T16H12.2	T16H12.2
-0.0981956808059685	AGIWUR2932

CB4856 CT III 10087223 10088673 WBGene00011819 hal-2 T16H12.11
-0.316503837464403 AGIWUR1595;AGIWUR7708;AGIWUR15104
CB4856 CT III 10088757 10090800 WBGene00011814 T16H12.4 T16H12.4
0.174486927324357 AGIWUR28361;AGIWUR38433
CB4856 CT III 10090862 10098081 WBGene00011815 bath-43 T16H12.5
0.0573307019899332 AGIWUR20784
CB4856 CT III 10098469 10101608 WBGene00002186 kel-10 T16H12.6
-0.0923701137843285 AGIWUR6246;AGIWUR10107;AGIWUR40683
CB4856 CT III 10104841 10106490 WBGene00011816 srt-55 T16H12.8
-0.0983581778722531 AGIWUR16209
CB4856 CT III 10109423 10112108 WBGene00011817 T16H12.9 T16H12.9
0.578561099910187 AGIWUR24769;AGIWUR33645;AGIWUR40685
CB4856 CT III 10112100 10113046 WBGene00014234 vps-29 ZK1128.8
0.099837865383547 AGIWUR6205;AGIWUR11659
CB4856 CT III 10113146 10114826 WBGene00014227 ZK1128.1 ZK1128.1
-0.159531230598688 AGIWUR23994;AGIWUR27881;AGIWUR29799
CB4856 CT III 10115042 10116760 WBGene00014228 mett-10 ZK1128.2
-0.30221001536289 AGIWUR25608
CB4856 CT III 10116967 10118918 WBGene00014229 ZK1128.3 ZK1128.3
0.13179390325514 AGIWUR10949;AGIWUR26661;AGIWUR29008
CB4856 CT III 10118961 10120148 WBGene00014230 ZK1128.4 ZK1128.4
0.00672292656320295 AGIWUR26863
CB4856 CT III 10120781 10122928 WBGene00044072 ham-3 ZK1128.5
0.0620217392462212 AGIWUR31504
CB4856 CT III 10124183 10132411 WBGene00014232 ttll-4 ZK1128.6
0.155416523609874 AGIWUR8363;AGIWUR19920;AGIWUR35531
CB4856 CT III 10136982 10139241 WBGene00014233 ZK1128.7 ZK1128.7
0.689288610815501 AGIWUR2705;AGIWUR17993;AGIWUR31730
CB4856 CT III 10141573 10146801 WBGene00014801 K08E5.1 K08E5.1 -0.284587928124455
AGIWUR5036
CB4856 CT III 10152795 10157923 WBGene00003519 nac-3 K08E5.2 0.141733641134458
AGIWUR13516;AGIWUR34351
CB4856 CT III 10159027 10160482 WBGene00077703 K08E5.5 K08E5.5 0.0283155442506731
AGIWUR38527;AGIWUR38946;AGIWUR39699
CB4856 CT III 10160697 10183529 WBGene00003482 mua-3 K08E5.3 -0.242465328385862
AGIWUR10672
CB4856 CT III 10183794 10186390 WBGene00006783 unc-47 T20G5.6 0.039265112774773
AGIWUR12361
CB4856 CT III 10189218 10195399 WBGene00004255 epac-1 T20G5.5 -0.140222241331328

AGIWUR25467
CB4856 CT III 10197716 10198352 WBGene00011869 dod-6 T20G5.7 0.528129675075681
AGIWUR14195;AGIWUR22878;AGIWUR26343;AGIWUR29516
CB4856 CT III 10199337 10199859 WBGene00011870 T20G5.8 T20G5.8 0.0308328725315477
AGIWUR6691;AGIWUR18399;AGIWUR22022;AGIWUR40445
CB4856 CT III 10199894 10201326 WBGene00011874 T20G5.13 T20G5.13
-0.0622331239322715 AGIWUR48;AGIWUR21088
CB4856 CT III 10201623 10202528 WBGene00011873 T20G5.12 T20G5.12
-0.259343154991146 AGIWUR37044
CB4856 CT III 10203821 10209704 WBGene00011867 chc-1 T20G5.1 -0.0197883681736206
AGIWUR23019;AGIWUR39562;AGIWUR40241
CB4856 CT III 10209840 10211093 WBGene00011871 T20G5.9 T20G5.9 -0.342242588917477
AGIWUR1557
CB4856 CT III 10211041 10211946 WBGene00011872 blos-1 T20G5.10
-0.0307896484661114 AGIWUR28631
CB4856 CT III 10212396 10214825 WBGene00011868 best-18 T20G5.4 0.0019120744424912
AGIWUR1668;AGIWUR12462;AGIWUR19724;AGIWUR27812;AGIWUR32888;AGIWUR33217;AGIWUR41702
CB4856 CT III 10214814 10216635 WBGene00000833 cts-1 T20G5.2 0.203073256872992
AGIWUR6067;AGIWUR20186;AGIWUR25038;AGIWUR27140;AGIWUR44892
CB4856 CT III 10217302 10218646 WBGene00004326 rde-4 T20G5.11
-0.395999415612788 AGIWUR1070
CB4856 CT III 10218943 10222935 WBGene00011875 T20G5.14 T20G5.14
-0.196691811774183 AGIWUR13544;AGIWUR16763;AGIWUR17186;AGIWUR21489;AGIWUR33131
CB4856 CT III 10223524 10224162 WBGene00014946 Y70G10A.1 Y70G10A.1
-0.1094506285287 AGIWUR14372
CB4856 CT III 10228164 10234079 WBGene00013498 Y70G10A.2 Y70G10A.2
0.171524687612844 AGIWUR17841;AGIWUR20918;AGIWUR22136;AGIWUR38703;AGIWUR41816
CB4856 CT III 10238672 10244810 WBGene00013499 Y70G10A.3 Y70G10A.3
0.807855854581852 AGIWUR42684
CB4856 CT III 10245056 10248905 WBGene00001002 div-1 R01H10.1
0.174132609177121 AGIWUR32960
CB4856 CT III 10249111 10254709 WBGene00000768 cor-1 R01H10.3
-0.115909803578307 AGIWUR4531;AGIWUR25414;AGIWUR37845;AGIWUR40559
CB4856 CT III 10254800 10257221 WBGene00010972 R01H10.4 R01H10.4
-0.162912056676285 AGIWUR39750
CB4856 CT III 10257181 10258736 WBGene00010973 rip-1 R01H10.5
0.113780197894746 AGIWUR2959;AGIWUR5367;AGIWUR14748;AGIWUR29061;AGIWUR36636;AGIWUR38963
CB4856 CT III 10258856 10261679 WBGene00010974 bbs-5 R01H10.6
0.0919289864904589 AGIWUR28666;AGIWUR30482

CB4856	CT	III	10261799	10267381	WBGene00010975	R01H10.7	R01H10.7	-0.0380501346734121	AGIWUR23476
CB4856	CT	III	10271257	10278579	WBGene00000564	cnk-1	R01H10.8	-0.140903088381895	AGIWUR31863
CB4856	CT	III	10279127	10281476	WBGene00010757	pad-2	K10G9.3	-0.178544303183877	AGIWUR15507
CB4856	CT	III	10285923	10288621	WBGene00010756	K10G9.2	K10G9.2	0.0268939814443439	AGIWUR11899
CB4856	CT	III	10295137	10298687	WBGene00010755	vglu-2	K10G9.1	0.0928516342670189	AGIWUR9593
CB4856	CT	III	10299164	10299949	WBGene00011557	ostf-4	T07A5.4	-0.314075597237041	AGIWUR24598
CB4856	CT	III	10300293	10303423	WBGene00011556	vglu-3	T07A5.3	-0.565717872668504	AGIWUR19587
CB4856	CT	III	10303368	10306188	WBGene00011554	T07A5.1	T07A5.1	0.153291899876149	AGIWUR10153
CB4856	CT	III	10306756	10311100	WBGene00045475	T07A5.7	T07A5.7	0.00624216219882801	AGIWUR11178;AGIWUR39351;AGIWUR42277;AGIWUR43222
CB4856	CT	III	10314490	10318162	WBGene00006785	unc-50	T07A5.2	-0.142834133927688	AGIWUR8024;AGIWUR9434;AGIWUR11973;AGIWUR25507;AGIWUR29014;AGIWUR32340
CB4856	CT	III	10318939	10320021	WBGene00006802	unc-69	T07A5.6	0.115401156407842	AGIWUR30280;AGIWUR32788;AGIWUR33203;AGIWUR41320
CB4856	CT	III	10321019	10325675	WBGene00011562	T07C4.10	T07C4.10	-0.0469794512919819	AGIWUR29783
CB4856	CT	III	10325613	10330422	WBGene00003589	nex-2	T07C4.9	0.368423441930391	AGIWUR2964;AGIWUR5102;AGIWUR5328;AGIWUR10659;AGIWUR41869;AGIWUR42232
CB4856	CT	III	10331551	10332480	WBGene00044891	T07C4.12	T07C4.12	-0.0774899474028601	AGIWUR11953
CB4856	CT	III	10334267	10335171	WBGene00003225	mev-1	T07C4.7	0.0829042417471486	AGIWUR10210;AGIWUR33889
CB4856	CT	III	10335172	10337536	WBGene00000423	ced-9	T07C4.8	-0.0439390359057352	AGIWUR286;AGIWUR6854;AGIWUR9572;AGIWUR16216;AGIWUR23511;AGIWUR33329
CB4856	CT	III	10337840	10339440	WBGene00011561	ttr-15	T07C4.5	-0.0798489734697405	AGIWUR12663
CB4856	CT	III	10339984	10341858	WBGene00006546	tbx-9	T07C4.6	-0.428567398332884	AGIWUR5722
CB4856	CT	III	10347575	10349740	WBGene00006545	tbx-8	T07C4.2	0.344042509231922	AGIWUR29064
CB4856	CT	III	10352157	10355117	WBGene00011560	T07C4.3	T07C4.3		

-0.00230449700773683 AGIWUR20793;AGIWUR29934;AGIWUR31453;AGIWUR31839
CB4856 CT III 10355122 10355982 WBGene00004986 spp-1 T07C4.4 0.677787444118479
AGIWUR237;AGIWUR7718;AGIWUR8291;AGIWUR10149;AGIWUR21291;AGIWUR35483
CB4856 CT III 10355799 10357761 WBGene00011559 umps-1 T07C4.1 0.0671991303786985
AGIWUR7994;AGIWUR33842
CB4856 CT III 10364951 10373231 WBGene00008018 tbc-8 C38H2.1 0.0952049439889056
AGIWUR4274;AGIWUR22656;AGIWUR25009;AGIWUR35189;AGIWUR38326;AGIWUR43448
CB4856 CT III 10381740 10385335 WBGene00008019 C38H2.2 C38H2.2 0.0893401106011726
AGIWUR1650;AGIWUR12814;AGIWUR18659;AGIWUR32985;AGIWUR34960;AGIWUR36869
CB4856 CT III 10385313 10386572 WBGene00008020 C38H2.3 C38H2.3 0.142369449221723
AGIWUR33304
CB4856 CT III 10393389 10396081 WBGene00010838 M03C11.1 M03C11.1
0.0892317748246239 AGIWUR36458
CB4856 CT III 10399174 10405240 WBGene00010839 chl-1 M03C11.2
0.18240981302788 AGIWUR44839
CB4856 CT III 10405070 10415150 WBGene00010840 M03C11.3 M03C11.3
-0.547611126501421 AGIWUR3706
CB4856 CT III 10416069 10421785 WBGene00010842 ymel-1 M03C11.5
0.024664994714966 AGIWUR16409;AGIWUR23999
CB4856 CT III 10421781 10424006 WBGene00010843 M03C11.6 M03C11.6
-0.0267646728293691 AGIWUR23661
CB4856 CT III 10424112 10427982 WBGene00010844 prp-3 M03C11.7
0.00719411111644064 AGIWUR586;AGIWUR12218
CB4856 CT III 10428334 10435988 WBGene00010845 M03C11.8 M03C11.8
-0.228449736601743 AGIWUR27739
CB4856 CT III 10445805 10451112 WBGene00010365 H04D03.4 H04D03.4
0.058241226538235 AGIWUR7036
CB4856 CT III 10461389 10468051 WBGene00000231 atx-2 D2045.1 0.132688903195101
AGIWUR11012;AGIWUR23942;AGIWUR24350;AGIWUR38378;AGIWUR40827;AGIWUR43539
CB4856 CT III 10471237 10475245 WBGene00000836 cul-1 D2045.6 0.0876928936361701
AGIWUR42378
CB4856 CT III 10475245 10479758 WBGene00008426 D2045.9 D2045.9 0.0915490766253058
AGIWUR4407;AGIWUR25018;AGIWUR30597;AGIWUR35709;AGIWUR36584;AGIWUR39005
CB4856 CT III 10479807 10482285 WBGene00008424 D2045.7 D2045.7 0.0753844459554731
AGIWUR14104
CB4856 CT III 10482288 10483926 WBGene00008425 D2045.8 D2045.8 0.225714598964752
AGIWUR39626
CB4856 CT III 10488128 10500267 WBGene00009653 F43D9.1 F43D9.1 0.911803997764949
AGIWUR22640

CB4856	CT	III	10500284	10503853	WBGene00004283	rab-33	F43D9.2	-0.100743812231176	AGIWUR9622;AGIWUR24413;AGIWUR29010;AGIWUR31447;AGIWUR32558;AGIWUR42267
CB4856	CT	III	10503965	10505250	WBGene00006395	taf-11.3	F43D9.5	-0.206107865301334	AGIWUR20041
CB4856	CT	III	10506106	10506801	WBGene00004798	sip-1	F43D9.4	0.206562791734463	AGIWUR7686;AGIWUR13613;AGIWUR38909
CB4856	CT	III	10507058	10518391	WBGene00009654	sly-1	F43D9.3	-0.170604476805829	AGIWUR8732;AGIWUR19708;AGIWUR29661;AGIWUR35705
CB4856	CT	III	10520676	10532705	WBGene00006784	unc-49	T21C12.1	0.12054980252200 5	AGIWUR1626;AGIWUR2943;AGIWUR6217;AGIWUR7139;AGIWUR8761;AGIWUR13176;AGIWUR14305;AGIWUR15017;AGIWUR15943;AGIWUR16705;AGIWUR21882;AGIWUR26074;AGIWUR26395;AGIWUR29204;AGIWUR33200;AGIWUR36268
CB4856	CT	III	10535526	10537682	WBGene00001993	hpd-1	T21C12.2	-0.532955393760485	AGIWUR15910;AGIWUR42722;AGIWUR43555
CB4856	CT	III	10538081	10539163	WBGene00011899	T21C12.3	T21C12.3	0.0450825782218911	AGIWUR24461;AGIWUR33959;AGIWUR35118;AGIWUR39927
CB4856	CT	III	10551102	10552955	WBGene00043998	T21C12.8	T21C12.8	-0.133794073512897	AGIWUR4565;AGIWUR7449;AGIWUR8399;AGIWUR15232;AGIWUR17836;AGIWUR21442;AGIWUR42310;AGIWUR43489;AGIWUR44757
CB4856	CT	III	10553005	10558009	WBGene00011900	T21C12.4	T21C12.4	-0.23096974383134	AGIWUR7867;AGIWUR22646;AGIWUR32575
CB4856	CT	III	10558189	10561948	WBGene00012859	Y45F3A.1	Y45F3A.1	0.104655277531904	AGIWUR32679
CB4856	CT	III	10563026	10565603	WBGene00004282	rab-30	Y45F3A.2	-0.193499075750154	AGIWUR1388;AGIWUR2446;AGIWUR25760;AGIWUR28411;AGIWUR29148;AGIWUR37905
CB4856	CT	III	10566181	10571606	WBGene00012860	acdh-11	Y45F3A.3	-0.0494386237811604	AGIWUR23517
CB4856	CT	III	10572475	10575420	WBGene00012861	Y45F3A.4	Y45F3A.4	0.0343880386410849	AGIWUR20000;AGIWUR21324;AGIWUR36197
CB4856	CT	III	10585848	10586343	WBGene00012862	Y45F3A.5	Y45F3A.5	-0.30240556343611	AGIWUR39377
CB4856	CT	III	10586407	10587706	WBGene00014898	Y45F3A.6	Y45F3A.6	-0.115298221089564	AGIWUR12638
CB4856	CT	III	10589865	10592801	WBGene00012863	Y45F3A.8	Y45F3A.8	0.348253017249558	AGIWUR28002
CB4856	CT	III	10592754	10595166	WBGene00012864	Y45F3A.9	Y45F3A.9	0.309811975591565	AGIWUR12357
CB4856	CT	III	10595258	10596487	WBGene00012643	Y39A1A.3	Y39A1A.3	-0.155973051319215	AGIWUR113

CB4856	CT	III	10596615	10600184	WBGene00012641	epg-6	Y39A1A.1	0.039746423797375	AGIWUR29369
CB4856	CT	III	10601905	10603566	WBGene00012642	Y39A1A.2	Y39A1A.2	-0.0189740712806223	AGIWUR308;AGIWUR29627;AGIWUR38468;AGIWUR41990
CB4856	CT	III	10605349	10613294	WBGene00012644	rabx-5	Y39A1A.5	-0.420978096648986	AGIWUR5300
CB4856	CT	III	10613567	10614931	WBGene00012660	Y39A1A.24	Y39A1A.24	-0.0232815237049486	AGIWUR9172
CB4856	CT	III	10615466	10617862	WBGene00012645	mrpl-22	Y39A1A.6	-0.0810402547632734	AGIWUR27121
CB4856	CT	III	10617863	10621369	WBGene00012646	lron-10	Y39A1A.7	0.136667611584093	AGIWUR6665;AGIWUR11602;AGIWUR35533;AGIWUR39777
CB4856	CT	III	10630070	10633501	WBGene00012648	Y39A1A.9	Y39A1A.9	0.11494459083311	AGIWUR11161
CB4856	CT	III	10640828	10642872	WBGene00000974	dhs-11	Y39A1A.11	-0.0558215288496804	AGIWUR10943
CB4856	CT	III	10643002	10646107	WBGene00012650	orc-1	Y39A1A.12	-0.0202754323855707	AGIWUR7399
CB4856	CT	III	10646170	10648861	WBGene00012651	orc-4	Y39A1A.13	-0.0315255448300409	AGIWUR18023;AGIWUR45072
CB4856	CT	III	10649519	10650908	WBGene00012652	Y39A1A.14	Y39A1A.14	-0.219121011662509	AGIWUR5717
CB4856	CT	III	10655018	10677521	WBGene00000566	cnt-2	Y39A1A.15	0.0948630623601581	AGIWUR9480;AGIWUR11595;AGIWUR17227;AGIWUR22673
CB4856	CT	III	10679474	10682212	WBGene00012654	Y39A1A.16	Y39A1A.16	-0.134592677962636	AGIWUR3345
CB4856	CT	III	10687127	10691101	WBGene00012655	Y39A1A.17	Y39A1A.17	0.111498166060504	AGIWUR3054
CB4856	CT	III	10692221	10694391	WBGene00012656	Y39A1A.18	Y39A1A.18	-0.350099423160501	AGIWUR6126
CB4856	CT	III	10694590	10698861	WBGene00001478	fmo-3	Y39A1A.19	-0.151481715658537	AGIWUR32081
CB4856	CT	III	10699216	10700560	WBGene00012657	Y39A1A.20	Y39A1A.20	-0.377349805564153	AGIWUR2587
CB4856	CT	III	10700652	10707024	WBGene00012658	Y39A1A.21	Y39A1A.21	-0.0880327252311641	AGIWUR23804;AGIWUR30508;AGIWUR38450
CB4856	CT	III	10707761	10708518	WBGene00012361	mrpl-12	W09D10.3	-0.167330494388262	AGIWUR11263;AGIWUR27960;AGIWUR37422
CB4856	CT	III	10708484	10711527	WBGene00012363	W09D10.5	W09D10.5		

-0.0545926416032803	AGIWUR24376
CB4856	CT	III	10711724	10716191	WBGene00012362	W09D10.4	W09D10.4
0.0104240491690391	AGIWUR23890
CB4856	CT	III	10722148	10730705	WBGene00012360	tat-3	W09D10.2
-0.0517110960506564	AGIWUR23128
CB4856	CT	III	10736893	10739824	WBGene00012359	W09D10.1	W09D10.1
0.411922002188424	AGIWUR9099
CB4856	CT	III	10740280	10740772	WBGene00010479	K01G5.8	K01G5.8	0.0341714789941427
AGIWUR1395;AGIWUR8920;AGIWUR21836;AGIWUR27800;AGIWUR30550;AGIWUR36735;AGIWUR42856
CB4856	CT	III	10740868	10742461	WBGene00006536	tbb-1	K01G5.7	0.0511485985609621
AGIWUR12340;AGIWUR24533
CB4856	CT	III	10742777	10746675	WBGene00004361	rib-2	K01G5.6	0.208180025678248
AGIWUR18008
CB4856	CT	III	10746581	10747728	WBGene00004302	ran-1	K01G5.4	-0.132471382349475
AGIWUR4222;AGIWUR5342;AGIWUR31873;AGIWUR37645;AGIWUR37954;AGIWUR44185
CB4856	CT	III	10748429	10750080	WBGene00010478	K01G5.5	K01G5.5	-0.0415525255894202
AGIWUR2813
CB4856	CT	III	10752254	10755523	WBGene00001996	hpl-2	K01G5.2	-0.0748766631589071
AGIWUR26193
CB4856	CT	III	10755624	10757640	WBGene00010476	rnf-113	K01G5.1	-0.209256659965462
AGIWUR5623;AGIWUR16936;AGIWUR23877;AGIWUR30190;AGIWUR33377
CB4856	CT	III	10757641	10758522	WBGene00010481	K01G5.10	K01G5.10
-0.102555580356295	AGIWUR42363
CB4856	CT	III	10759290	10763006	WBGene00010480	K01G5.9	K01G5.9
-0.00943322427416504	AGIWUR2342;AGIWUR26214;AGIWUR43246
CB4856	CT	III	10763131	10777343	WBGene00001087	dpy-28	Y39A1B.3
0.259207980873416	AGIWUR19814;AGIWUR31824;AGIWUR37311;AGIWUR39594;AGIWUR42588;AGIWUR44785;AGIWUR44913
CB4856	CT	III	10781853	10791252	WBGene00004233	ptr-19	Y39A1B.2
-0.224418467664648	AGIWUR2310
CB4856	CT	III	10794349	10796369	WBGene00012661	clec-163	Y39A1B.1
-0.0644012356936328	AGIWUR4091;AGIWUR16942;AGIWUR30849
CB4856	CT	III	10796711	10802212	WBGene00010772	K11D9.3	K11D9.3	0.0785546250518827
AGIWUR43139
CB4856	CT	III	10805930	10810999	WBGene00002219	klp-7	K11D9.1	0.174669845424949
AGIWUR11370;AGIWUR12933;AGIWUR13168;AGIWUR23162;AGIWUR34095;AGIWUR34776;AGIWUR35301
CB4856	CT	III	10811906	10816844	WBGene00004736	sca-1	K11D9.2	0.538005826351862
AGIWUR12804;AGIWUR13224;AGIWUR18711;AGIWUR22264;AGIWUR22479;AGIWUR26781;AGIWUR32116;AGIWUR40601
CB4856	CT	III	10832936	10839451	WBGene00011269	R17.3	R17.3	-0.613582870220877
AGIWUR22883

CB4856	CT	III	10840023	10845020	WBGene00011268	pde-12	R17.2	0.0196149895236477	AGIWUR23043
CB4856	CT	III	10847619	10848670	WBGene00011267	fbxb-85	R17.1	-0.0101278175256763	AGIWUR2734
CB4856	CT	III	10849222	10854561	WBGene00012663	hex-3	Y39A1C.4	-0.223241167492347	AGIWUR15260;AGIWUR27664;AGIWUR37209
CB4856	CT	III	10856776	10858186	WBGene00012662	Y39A1C.1	Y39A1C.1	0.0395856049826264	AGIWUR27789
CB4856	CT	III	10858284	10866994	WBGene00003898	oxi-1	Y39A1C.2	0.0786211111862443	AGIWUR24661
CB4856	CT	III	10866870	10868727	WBGene00000475	cey-4	Y39A1C.3	-0.137157608699078	AGIWUR12140;AGIWUR16283;AGIWUR18401;AGIWUR22576;AGIWUR38439;AGIWUR42063
CB4856	CT	III	10869193	10874528	WBGene00008078	vps-50	C44B9.1	-0.0972620104601596	AGIWUR35718
CB4856	CT	III	10875459	10878061	WBGene00008079	C44B9.2	C44B9.2	-0.19971444977765	AGIWUR32805
CB4856	CT	III	10878191	10880183	WBGene00008080	C44B9.3	C44B9.3	0.00226925251257271	AGIWUR18192
CB4856	CT	III	10882345	10893672	WBGene00008081	athp-1	C44B9.4	-0.0865623869277456	AGIWUR37057
CB4856	CT	III	10894520	10895732	WBGene00043996	fbxb-87	C44B9.6	0.22627609901799	AGIWUR31035;AGIWUR40977
CB4856	CT	III	10896363	10901836	WBGene00008082	com-1	C44B9.5	0.0587986924072332	AGIWUR17334;AGIWUR29610;AGIWUR37809
CB4856	CT	III	10902648	10908927	WBGene00006843	unc-119	M142.1	0.234914082633587	AGIWUR42584
CB4856	CT	III	10911460	10916567	WBGene00000853	cut-6	M142.2	0.834716731185803	AGIWUR20219;AGIWUR21209;AGIWUR27188;AGIWUR37502
CB4856	CT	III	10920336	10926041	WBGene00006873	vab-7	M142.4	-0.0114198507314125	AGIWUR44250
CB4856	CT	III	10927093	10928909	WBGene00014809	M142.3	M142.3	0.242366597736094	AGIWUR38763
CB4856	CT	III	10931208	10932226	WBGene00010922	M142.5	M142.5	-0.197538207822259	AGIWUR29358
CB4856	CT	III	10933631	10936129	WBGene00043057	M142.8	M142.8	0.0951330761355109	AGIWUR36829
CB4856	CT	III	10936246	10946834	WBGene00010923	rle-1	M142.6	-0.385481068761737	AGIWUR4139
CB4856	CT	III	10947446	10951169	WBGene00013132	strd-1	Y52D3.1	0.0528839020709739	

AGIWUR11800;AGIWUR26861;AGIWUR34611
CB4856 CT III 10951225 10953505 WBGene00006982 zig-5 Y48A6A.1
0.00887476806203757 AGIWUR7315;AGIWUR13367;AGIWUR16414;AGIWUR26225;AGIWUR43305
CB4856 CT III 10960243 10966996 WBGene00012271 W05B2.2 W05B2.2 0.157381794089271
AGIWUR10851;AGIWUR17598
CB4856 CT III 10967779 10969472 WBGene00014854 W05B2.3 W05B2.3 -0.0917079150410824
AGIWUR18948
CB4856 CT III 10969944 10981848 WBGene00012272 dhc-4 W05B2.4 0.0763285011100203
AGIWUR14770
CB4856 CT III 10982166 10983253 WBGene00000667 col-92 W05B2.6 -0.215001892914252
AGIWUR6521
CB4856 CT III 10988133 10991912 WBGene00012273 W05B2.7 W05B2.7 -0.522904580550698
AGIWUR7734;AGIWUR9067;AGIWUR9729;AGIWUR17657;AGIWUR26332;AGIWUR29538;AGIWUR33745;AGIWUR37673;AGIWUR38793
CB4856 CT III 10992129 10995237 WBGene00012962 dmsr-5 Y48A6B.1
0.0885106833034746 AGIWUR32538;AGIWUR40488;AGIWUR43570
CB4856 CT III 11007415 11007620 WBGene00012965 fipr-17 Y48A6B.4
-0.233100922922118 AGIWUR446;AGIWUR13076;AGIWUR15516;AGIWUR36422;AGIWUR41315
CB4856 CT III 11008222 11008934 WBGene00012964 Y48A6B.3 Y48A6B.3
-0.238256137997127 AGIWUR29078;AGIWUR40689
CB4856 CT III 11009043 11010595 WBGene00012966 exos-1 Y48A6B.5
-0.145628199515881 AGIWUR7404
CB4856 CT III 11012566 11025292 WBGene00012967 kvs-4 Y48A6B.6
0.0403438047143467 AGIWUR4045
CB4856 CT III 11033172 11036080 WBGene00012970 Y48A6B.9 Y48A6B.9
0.632104671002493 AGIWUR8842
CB4856 CT III 11036943 11063027 WBGene00012973 spat-2 Y48A6B.13
0.0310239153531802 AGIWUR11863;AGIWUR15358;AGIWUR26237;AGIWUR29100
CB4856 CT III 11063479 11068753 WBGene00012972 rsa-2 Y48A6B.11
-0.0586417749500203 AGIWUR29460
CB4856 CT III 11068858 11072981 WBGene00012356 W09D6.1 W09D6.1 0.203326158751482
AGIWUR7033
CB4856 CT III 11074536 11077494 WBGene00006287 str-261 W09D6.2 0.0795344372866644
AGIWUR26409
CB4856 CT III 11077704 11079446 WBGene00006273 str-246 W09D6.3 0.287149096931958
AGIWUR13621
CB4856 CT III 11079745 11080413 WBGene00012357 W09D6.4 W09D6.4 0.0517428160387112
AGIWUR2936
CB4856 CT III 11088487 11091616 WBGene00012358 W09D6.5 W09D6.5 0.155506478796866
AGIWUR27195

CB4856 CT III 11091548 11099964 WBGene00001815 hmt-1 W09D6.6 0.165657172101426
AGIWUR10993;AGIWUR34799;AGIWUR37401
CB4856 CT III 11101268 11107206 WBGene00012974 Y48A6C.1 Y48A6C.1
-0.385891302975542 AGIWUR35097
CB4856 CT III 11116994 11119222 WBGene00012976 sup-35 Y48A6C.3
-0.166387558590166 AGIWUR22107
CB4856 CT III 11131775 11136380 WBGene00004010 pha-1 Y48A6C.5
-0.116180571611496 AGIWUR37170
CB4856 CT III 11136802 11138905 WBGene00012923 fbxa-128 Y47D3A.2
-0.0812613177881181 AGIWUR43741
CB4856 CT III 11139241 11141825 WBGene00012922 Y47D3A.1 Y47D3A.1
-0.0574195935435879 AGIWUR18483
CB4856 CT III 11142448 11154287 WBGene00012936 Y47D3A.29 Y47D3A.29
-0.151463451344206 AGIWUR2112
CB4856 CT III 11154827 11168820 WBGene00000519 cku-70 Y47D3A.4
0.0174416039119905 AGIWUR31521
CB4856 CT III 11168923 11170865 WBGene00012924 Y47D3A.5 Y47D3A.5
-0.175919198393078 AGIWUR42591
CB4856 CT III 11170889 11195207 WBGene00006604 tra-1 Y47D3A.6
-0.194202353879837
AGIWUR1689;AGIWUR2672;AGIWUR17668;AGIWUR22313;AGIWUR25374;AGIWUR29878;AGIWUR35190;AGIWUR38829
CB4856 CT III 11216398 11218260 WBGene00006553 tbx-34 Y47D3A.10
-0.11426874655584 AGIWUR45039
CB4856 CT III 11218797 11222666 WBGene00012925 wht-8 Y47D3A.11
0.0639333113470597 AGIWUR5856;AGIWUR6471;AGIWUR18518
CB4856 CT III 11223439 11224106 WBGene00012926 Y47D3A.13 Y47D3A.13
-0.0208258142026914 AGIWUR20032;AGIWUR22061
CB4856 CT III 11224800 11227125 WBGene00006556 tbx-37 Y47D3A.12
0.0195579304513132 AGIWUR19564
CB4856 CT III 11235581 11236868 WBGene00012927 Y47D3A.14 Y47D3A.14
-0.160470649691176 AGIWUR18209;AGIWUR21353
CB4856 CT III 11237149 11238252 WBGene00012928 aakb-2 Y47D3A.15
-0.0366260945755137 AGIWUR26897
CB4856 CT III 11245120 11252281 WBGene00012929 rsks-1 Y47D3A.16
-0.198911370583157 AGIWUR17817;AGIWUR19720
CB4856 CT III 11252391 11256646 WBGene00012938 Y47D3A.32 Y47D3A.32
0.281817577757721 AGIWUR9222
CB4856 CT III 11257307 11270875 WBGene00012930 obr-1 Y47D3A.17
-0.0248773133419959 AGIWUR4034

CB4856 CT III 11274901 11276032 WBGene00012931 Y47D3A.20 Y47D3A.20
-0.00369467202297358 AGIWUR17656
CB4856 CT III 11276292 11278917 WBGene00012932 Y47D3A.21 Y47D3A.21
-0.0398231726040983 AGIWUR43113
CB4856 CT III 11279021 11290580 WBGene00012934 gly-9 Y47D3A.23
-0.297924915790283 AGIWUR10346
CB4856 CT III 11290911 11306145 WBGene00004873 smc-3 Y47D3A.26
0.245106102816056 AGIWUR11729
CB4856 CT III 11306560 11311566 WBGene00004284 rab-35 Y47D3A.25
-0.0722840985383506 AGIWUR2957;AGIWUR6937;AGIWUR24812;AGIWUR25485;AGIWUR28738;AGIWUR36629
CB4856 CT III 11312251 11317055 WBGene00006563 teg-1 Y47D3A.27
-0.110579184760079 AGIWUR30380
CB4856 CT III 11317570 11326664 WBGene00012935 mcm-10 Y47D3A.28
0.0201364032022617 AGIWUR37595
CB4856 CT III 11326654 11343108 WBGene00012126 T28D6.6 T28D6.6
-0.000811595414276667 AGIWUR8625
CB4856 CT III 11343272 11344147 WBGene00003975 pen-2 T28D6.9 0.0101130137170724
AGIWUR8411
CB4856 CT III 11344255 11346789 WBGene00012127 T28D6.7 T28D6.7 -0.0489922386342627
AGIWUR10769
CB4856 CT III 11360456 11366214 WBGene00012124 T28D6.4 T28D6.4 0.352505881594336
AGIWUR36605
CB4856 CT III 11366618 11368435 WBGene00012123 T28D6.3 T28D6.3 -0.115296367999898
AGIWUR3710;AGIWUR7468;AGIWUR14018;AGIWUR35762;AGIWUR40316;AGIWUR41376
CB4856 CT III 11368415 11371775 WBGene00006533 tba-7 T28D6.2 -0.0563522264607119
AGIWUR715;AGIWUR5371;AGIWUR29133;AGIWUR32168;AGIWUR42111
CB4856 CT III 11372886 11377980 WBGene00001077 dpy-18 Y47D3B.10
0.143861624413716 AGIWUR12436;AGIWUR13772
CB4856 CT III 11382222 11388669 WBGene00012939 Y47D3B.1 Y47D3B.1
0.476171814968513 AGIWUR1218;AGIWUR5068;AGIWUR16542
CB4856 CT III 11388606 11393261 WBGene00003759 nlp-21 Y47D3B.2
-0.197689784790373 AGIWUR821;AGIWUR1951;AGIWUR3467;AGIWUR16106;AGIWUR18641;AGIWUR34936;AGIWUR40877
CB4856 CT III 11403017 11404197 WBGene00185055 Y47D3B.12 Y47D3B.12
-0.381840457063403 AGIWUR10572;AGIWUR15106;AGIWUR27216
CB4856 CT III 11405361 11409425 WBGene00012941 Y47D3B.4 Y47D3B.4
-0.0167845272398424 AGIWUR6626;AGIWUR12982;AGIWUR13920;AGIWUR22355;AGIWUR36034
CB4856 CT III 11410712 11424655 WBGene00006683 twk-31 Y47D3B.5
0.240108007738542 AGIWUR26655
CB4856 CT III 11427135 11428414 WBGene00012942 Y47D3B.6 Y47D3B.6

-0.0585999441739151	AGIWUR2691;AGIWUR6711;AGIWUR8090;AGIWUR16063;AGIWUR19274;AGIWUR21033;AGIWUR42739
CB4856	CT	III	11428367	11442566	WBGene00004735	sbp-1	Y47D3B.7
0.329457709308787	AGIWUR35305
CB4856	CT	III	11456877	11462897	WBGene00023404	plr-1	Y47D3B.11
0.0845879822820166	AGIWUR38850
CB4856	CT	III	11463665	11470805	WBGene00012943	bed-2	Y47D3B.9
0.184565694451567	AGIWUR34421
CB4856	CT	III	11482603	11484244	WBGene00013421	fbxa-220	Y66A7A.1
0.194108468325247	AGIWUR9425
CB4856	CT	III	11485474	11487345	WBGene00013422	Y66A7A.2	Y66A7A.2
-0.220376135889801	AGIWUR6555
CB4856	CT	III	11488355	11500747	WBGene00013447	nars-2	Y66D12A.23
-0.348240441221611	AGIWUR8032;AGIWUR18572;AGIWUR36913
CB4856	CT	III	11500867	11502852	WBGene00006573	tin-10	Y66D12A.22
-0.348529492102957	AGIWUR23071
CB4856	CT	III	11502963	11505291	WBGene00013446	Y66D12A.21	Y66D12A.21
-0.0555814692081755	AGIWUR236;AGIWUR33308
CB4856	CT	III	11505535	11508919	WBGene00004960	spe-6	Y66D12A.20
-0.222982686023858	AGIWUR19592
CB4856	CT	III	11509081	11511467	WBGene00013445	Y66D12A.19	Y66D12A.19
-0.321874333556411	AGIWUR6432;AGIWUR11711
CB4856	CT	III	11512860	11523759	WBGene00013443	such-1	Y66D12A.17
0.177527254050588	AGIWUR42355
CB4856	CT	III	11524322	11526564	WBGene00013442	Y66D12A.16	Y66D12A.16
-0.232878568763034	AGIWUR24201
CB4856	CT	III	11526372	11534595	WBGene00013441	xpb-1	Y66D12A.15
-0.0125817880076369	AGIWUR3323
CB4856	CT	III	11534674	11546669	WBGene00013440	nrps-1	Y66D12A.14
0.392516107759096	AGIWUR32086
CB4856	CT	III	11548377	11552398	WBGene00013439	Y66D12A.13	Y66D12A.13
0.0418956790754041	AGIWUR43703
CB4856	CT	III	11561152	11562655	WBGene00013438	ztf-29	Y66D12A.12
-0.162475746420359	AGIWUR30629
CB4856	CT	III	11563467	11565536	WBGene00013437	Y66D12A.11	Y66D12A.11
0.0637039282068379	AGIWUR7037;AGIWUR9963;AGIWUR19649
CB4856	CT	III	11565833	11568007	WBGene00013436	Y66D12A.10	Y66D12A.10
-0.252438201811387	AGIWUR19129
CB4856	CT	III	11568137	11573343	WBGene00013435	Y66D12A.9	Y66D12A.9
0.519044544728883	AGIWUR11078

CB4856	CT	III	11574221	11578752	WBGene00013434	Y66D12A.8	Y66D12A.8
-0.0890783020614441	AGIWUR27440;AGIWUR44945
CB4856	CT	III	11579265	11580478	WBGene00013433	Y66D12A.7	Y66D12A.7
-0.0889950100169923	AGIWUR7522
CB4856	CT	III	11580694	11581402	WBGene00013432	Y66D12A.6	Y66D12A.6
0.109908339056055	AGIWUR18421
CB4856	CT	III	11581600	11589133	WBGene00013431	ceh-92	Y66D12A.5
-0.338387425018541	AGIWUR8014;AGIWUR41570
CB4856	CT	III	11589211	11592837	WBGene00013429	Y66D12A.3	Y66D12A.3
0.0791942469624516	AGIWUR32063
CB4856	CT	III	11608170	11609082	WBGene00013427	Y66D12A.1	Y66D12A.1
0.107296852861058	AGIWUR31529
CB4856	CT	III	11612607	11615707	WBGene00013424	Y66A7A.4	Y66A7A.4
0.110501794697701	AGIWUR22804
CB4856	CT	III	11616758	11623991	WBGene00013425	ceh-91	Y66A7A.5
0.0292227341434081	AGIWUR26537
CB4856	CT	III	11624991	11628238	WBGene00001633	gly-8	Y66A7A.6
-0.480153917533894	AGIWUR39593
CB4856	CT	III	11628386	11631014	WBGene00013426	Y66A7A.7	Y66A7A.7
-0.168510448896582	AGIWUR39184
CB4856	CT	III	11633216	11636840	WBGene00006552	tbx-33	Y66A7A.8
-0.0205322969644115	AGIWUR1180
CB4856	CT	III	11641461	11641861	WBGene00012760	Y41C4A.17	Y41C4A.17
-0.0321098526178059	AGIWUR15250;AGIWUR16680;AGIWUR23606;AGIWUR24686;AGIWUR39423
CB4856	CT	III	11647769	11649893	WBGene00012749	Y41C4A.1	Y41C4A.1
-0.111552107247185	AGIWUR15681
CB4856	CT	III	11650471	11656185	WBGene00012750	faah-6	Y41C4A.2
0.417857578119197	AGIWUR17139
CB4856	CT	III	11657738	11693837	WBGene00000793	crh-1	Y41C4A.4
-0.0600383431467861
AGIWUR743;AGIWUR3806;AGIWUR4913;AGIWUR12286;AGIWUR22855;AGIWUR24059;AGIWUR26704;AGIWUR27210;AGIWUR28416;AGIWUR33693;AGIWUR35499;AGIWUR40809;AGIWUR41451;AGIWUR42718;AGIWUR43847
CB4856	CT	III	11696599	11698306	WBGene00012753	Y41C4A.6	Y41C4A.6
-0.115446822971863	AGIWUR279;AGIWUR8193;AGIWUR16575;AGIWUR27230;AGIWUR37467;AGIWUR44508
CB4856	CT	III	11698869	11700087	WBGene00012754	Y41C4A.7	Y41C4A.7
-0.321103339935763	AGIWUR34332
CB4856	CT	III	11700285	11702856	WBGene00004165	pqn-84	Y41C4A.5
-0.206966550209426	AGIWUR28537
CB4856	CT	III	11703885	11705091	WBGene00000670	col-95	Y41C4A.16

0.94016343822587 AGIWUR43392
CB4856 CT III 11705713 11706644 WBGene00000671 col-96 Y41C4A.19
-0.403787655797608 AGIWUR15044
CB4856 CT III 11706969 11708820 WBGene00012755 Y41C4A.8 Y41C4A.8
-0.301838029739702 AGIWUR314
CB4856 CT III 11710827 11718741 WBGene00012756 Y41C4A.9 Y41C4A.9
0.262068449824206 AGIWUR36806
CB4856 CT III 11718834 11719450 WBGene00001235 elb-1 Y41C4A.10
-0.0343891351634689 AGIWUR44409
CB4856 CT III 11721706 11723602 WBGene00270321 Y41C4A.32 Y41C4A.32
-1.50737829347757 AGIWUR18044
CB4856 CT III 11724038 11726690 WBGene00012758 Y41C4A.12 Y41C4A.12
0.0925744181585824 AGIWUR11342;AGIWUR37933
CB4856 CT III 11730612 11731305 WBGene00012761 Y41C4A.18 Y41C4A.18
-0.0736319515455399 AGIWUR22498
CB4856 CT III 11733346 11737627 WBGene00012759 sup-1 Y41C4A.13
-0.234013469809998 AGIWUR24400
CB4856 CT III 11747042 11750160 WBGene00003417 mrt-2 Y41C4A.14
-0.145596710312331 AGIWUR6066
CB4856 CT III 11751320 11773681 WBGene00007703 gbf-1 C24H11.7
-0.252231421601378 AGIWUR21724
CB4856 CT III 11774950 11776675 WBGene00007021 immp-1 C24H11.6
-0.206904341390061 AGIWUR14285;AGIWUR14709;AGIWUR24365;AGIWUR28971;AGIWUR37179
CB4856 CT III 11777625 11779629 WBGene00007704 mdt-21 C24H11.9
-0.416431998033531 AGIWUR15738
CB4856 CT III 11780235 11781035 WBGene00007702 C24H11.5 C24H11.5
-0.0776194219862143 AGIWUR9878;AGIWUR11324;AGIWUR14573;AGIWUR14995;AGIWUR23570;AGIWUR25385
CB4856 CT III 11782112 11784074 WBGene00007701 srd-74 C24H11.4
-0.179514855850409 AGIWUR22266
CB4856 CT III 11784147 11786052 WBGene00006557 tbx-38 C24H11.3
-0.0412596695604606 AGIWUR8227
CB4856 CT III 11795748 11803243 WBGene00006690 twk-39 C24H11.8
-0.0626262974072804 AGIWUR3472;AGIWUR15834
CB4856 CT III 11804547 11806795 WBGene00044232 C18D11.7 C18D11.7
-0.196135918692066 AGIWUR6793;AGIWUR14078
CB4856 CT III 11809118 11809561 WBGene00045211 C18D11.9 C18D11.9
-0.789141760745357 AGIWUR1937;AGIWUR17901;AGIWUR26015;AGIWUR27348
CB4856 CT III 11810556 11811046 WBGene00007682 C18D11.6 C18D11.6
0.393230561492231 AGIWUR24374;AGIWUR28866

CB4856 CT III 11816569 11819806 WBGene00007679 C18D11.1 C18D11.1
-0.217302357014903 AGIWUR30952
CB4856 CT III 11819745 11822526 WBGene00007680 maa-1 C18D11.2
0.104649838124288 AGIWUR10358;AGIWUR12232;AGIWUR27929
CB4856 CT III 11823327 11824975 WBGene00007681 C18D11.3 C18D11.3
-0.056284644016408 AGIWUR37930
CB4856 CT III 11826215 11830996 WBGene00004705 rsp-8 C18D11.4
-0.228085364860508 AGIWUR491;AGIWUR962
CB4856 CT III 11831008 11846465 WBGene00003826 ntl-3 Y56A3A.1
0.159137461688866 AGIWUR15901;AGIWUR41835
CB4856 CT III 11848152 11849522 WBGene00003234 mif-1 Y56A3A.3
0.0513628186254333 AGIWUR51
CB4856 CT III 11849951 11862249 WBGene00013225 Y56A3A.2 Y56A3A.2
0.0754136267902235 AGIWUR6842;AGIWUR31751
CB4856 CT III 11862393 11869281 WBGene00006396 taf-12 Y56A3A.4
-0.055128050549949 AGIWUR32671;AGIWUR32932
CB4856 CT III 11871252 11878889 WBGene00013227 Y56A3A.6 Y56A3A.6
0.368492926487075 AGIWUR9530
CB4856 CT III 11886485 11891435 WBGene00013226 faah-5 Y56A3A.5
-0.100631281374231 AGIWUR23762
CB4856 CT III 11894784 11898269 WBGene00013231 tsen-2 Y56A3A.11
-0.0808366637506666 AGIWUR30976;AGIWUR31534;AGIWUR41043
CB4856 CT III 11899337 11907387 WBGene00013232 faah-4 Y56A3A.12
-0.259913860074402 AGIWUR22557
CB4856 CT III 11907477 11908474 WBGene00013233 sdz-33 Y56A3A.14
0.229753497925833 AGIWUR5241
CB4856 CT III 11915151 11915712 WBGene00013237 Y56A3A.19 Y56A3A.19
-0.42617561726146 AGIWUR10004;AGIWUR20598;AGIWUR30622
CB4856 CT III 11915891 11917876 WBGene00003802 npp-16 Y56A3A.17
-0.122572934197998
AGIWUR6396;AGIWUR6847;AGIWUR9066;AGIWUR15726;AGIWUR16215;AGIWUR18214;AGIWUR27133;AGIWUR28029;AGIWUR29924;AG
IWUR30267;AGIWUR33353;AGIWUR41005
CB4856 CT III 11918828 11920237 WBGene00000369 ccf-1 Y56A3A.20
-0.225837799007691
AGIWUR3851;AGIWUR4029;AGIWUR6652;AGIWUR15305;AGIWUR22050;AGIWUR33330;AGIWUR35362;AGIWUR41944
CB4856 CT III 11920716 11921590 WBGene00013238 trap-4 Y56A3A.21
0.113065948744502 AGIWUR42080
CB4856 CT III 11921692 11923825 WBGene00013239 Y56A3A.22 Y56A3A.22
-0.245091948425809 AGIWUR24269

CB4856	CT	III	11923886	11929376	WBGene00003594	nft-1	Y56A3A.13	-0.0491492350356015	AGIWUR21699
CB4856	CT	III	11929889	11933639	WBGene00044233	Y56A3A.36	Y56A3A.36	-0.327676159187463	AGIWUR5818;AGIWUR35337;AGIWUR37064
CB4856	CT	III	11934002	11947017	WBGene00013228	Y56A3A.7	Y56A3A.7	-0.0135695299143537	AGIWUR6563;AGIWUR12033
CB4856	CT	III	11949918	11960180	WBGene00006596	top-3	Y56A3A.27	-0.254408253763877	AGIWUR2678
CB4856	CT	III	11961470	11971367	WBGene00013240	Y56A3A.28	Y56A3A.28	-0.295341810692231	AGIWUR1138;AGIWUR8487
CB4856	CT	III	11971461	11974092	WBGene00013241	ung-1	Y56A3A.29	-0.0487914071801523	AGIWUR5152;AGIWUR6264;AGIWUR7720
CB4856	CT	III	11974077	11979065	WBGene00013242	Y56A3A.30	Y56A3A.30	-0.077228538999627	AGIWUR1081
CB4856	CT	III	11974477	11989885	WBGene00013243	Y56A3A.31	Y56A3A.31	-0.314365705580368	AGIWUR3812
CB4856	CT	III	11990167	11999552	WBGene00006937	wah-1	Y56A3A.32	-0.184395897817421	AGIWUR2501;AGIWUR3631;AGIWUR34419;AGIWUR35517;AGIWUR35901;AGIWUR45018
CB4856	CT	III	11999497	12002950	WBGene00013244	Y56A3A.33	Y56A3A.33	0.0333633443475536	AGIWUR10400;AGIWUR17573;AGIWUR27018
CB4856	CT	III	12020552	12025325	WBGene00000273	brp-1	Y79H2A.1	0.371968348946814	AGIWUR20615
CB4856	CT	III	12025559	12028811	WBGene00043997	Y79H2A.12	Y79H2A.12	0.160057099606504	AGIWUR31274;AGIWUR37681
CB4856	CT	III	12028969	12030769	WBGene00013579	Y79H2A.2	Y79H2A.2	0.128387743039062	AGIWUR21385;AGIWUR43928
CB4856	CT	III	12031516	12052303	WBGene00013580	Y79H2A.3	Y79H2A.3	-0.238937266087892	AGIWUR3496;AGIWUR4375;AGIWUR5043;AGIWUR7222;AGIWUR15628;AGIWUR20046;AGIWUR38151
CB4856	CT	III	12052424	12058458	WBGene00000201	arx-3	Y79H2A.6	-0.0440907599645666	AGIWUR26668
CB4856	CT	III	12058844	12072293	WBGene00006993	zyg-8	Y79H2A.11	0.127253363077275	AGIWUR36623;AGIWUR44842
CB4856	CT	III	12074290	12077556	WBGene00004024	php-3	Y75B8A.1	0.108096231912784	AGIWUR9119
CB4856	CT	III	12077788	12084320	WBGene00003779	nob-1	Y75B8A.2	0.393424393278269	AGIWUR2669;AGIWUR13194
CB4856	CT	III	12101810	12106597	WBGene00013540	Y75B8A.3	Y75B8A.3	0.187394496830876	AGIWUR41000
CB4856	CT	III	12107774	12113471	WBGene00013541	lonp-2	Y75B8A.4		

0.0921619216957712	AGIWUR44655
CB4856	CT	III	12114577	12118470	WBGene00013542	lron-12 Y75B8A.5
-0.324045461331659	AGIWUR24439
CB4856	CT	III	12123881	12130352	WBGene00013543	Y75B8A.6	Y75B8A.6
0.393553464515493	AGIWUR12891
CB4856	CT	III	12152899	12160100	WBGene00013544	Y75B8A.7	Y75B8A.7
-0.455777842656362	AGIWUR40081
CB4856	CT	III	12160758	12171356	WBGene00013545	Y75B8A.8	Y75B8A.8
0.027436849678784	AGIWUR8629
CB4856	CT	III	12171353	12177245	WBGene00001636	gly-11	Y75B8A.9
0.0949019027976461	AGIWUR185;AGIWUR4639;AGIWUR34343;AGIWUR43642
CB4856	CT	III	12180960	12183013	WBGene00013547	Y75B8A.10	Y75B8A.10
-0.302660946537172	AGIWUR41582
CB4856	CT	III	12186411	12190116	WBGene00013548	Y75B8A.11	Y75B8A.11
0.406024974561672	AGIWUR9380
CB4856	CT	III	12191056	12202450	WBGene00003892	osm-12	Y75B8A.12
0.603134718779577	AGIWUR1812;AGIWUR18881;AGIWUR24126
CB4856	CT	III	12203822	12215686	WBGene00013549	Y75B8A.13	Y75B8A.13
-0.218967668748944	AGIWUR19945;AGIWUR20922;AGIWUR30232;AGIWUR36978
CB4856	CT	III	12216779	12218634	WBGene00013550	Y75B8A.14	Y75B8A.14
0.359750591685184	AGIWUR9294
CB4856	CT	III	12218990	12223934	WBGene00013551	Y75B8A.16	Y75B8A.16
-0.254521136223051	AGIWUR33754
CB4856	CT	III	12224487	12225331	WBGene00013552	gmn-1	Y75B8A.17
0.0266412461298837	AGIWUR1240;AGIWUR23735;AGIWUR30289;AGIWUR30706;AGIWUR31045;AGIWUR33207
CB4856	CT	III	12225638	12226267	WBGene00013553	Y75B8A.18	Y75B8A.18
-0.00747760142503751	AGIWUR31363
CB4856	CT	III	12226632	12232241	WBGene00013554	Y75B8A.19	Y75B8A.19
0.095554574393768	AGIWUR15908;AGIWUR16398;AGIWUR29200
CB4856	CT	III	12232632	12235711	WBGene00001724	grl-15	Y75B8A.20
-0.11424020903838	AGIWUR20943
CB4856	CT	III	12240367	12250164	WBGene00006571	tim-1	Y75B8A.22
-0.221168377960639	AGIWUR1647;AGIWUR6048;AGIWUR11763
CB4856	CT	III	12250602	12251009	WBGene00013556	Y75B8A.23	Y75B8A.23
0.00355843790524446	AGIWUR30023
CB4856	CT	III	12251492	12273692	WBGene00013557	Y75B8A.24	Y75B8A.24
-0.142991347925468	AGIWUR17796
CB4856	CT	III	12274040	12282221	WBGene00013558	Y75B8A.25	Y75B8A.25
0.0200537630765272	AGIWUR29151

CB4856	CT	III	12283208	12303216	WBGene00003414	mrp-8	Y75B8A.26	-0.0250614006231831	AGIWUR6025;AGIWUR14868;AGIWUR30937;AGIWUR39033
CB4856	CT	III	12307771	12311924	WBGene00004172	pqn-92	Y75B8A.27	0.329852313735213	AGIWUR41336
CB4856	CT	III	12313287	12314403	WBGene00023483	Y75B8A.39	Y75B8A.39	0.848497018249763	AGIWUR2361
CB4856	CT	III	12315479	12315857	WBGene00013565	Y75B8A.37	Y75B8A.37	0.140202539670053	AGIWUR38393
CB4856	CT	III	12319108	12323771	WBGene00013559	Y75B8A.28	Y75B8A.28	-0.271616841127308	AGIWUR24263
CB4856	CT	III	12327667	12332274	WBGene00013560	zip-12	Y75B8A.29	-0.14298759349837	AGIWUR20625
CB4856	CT	III	12333745	12345347	WBGene00006986	zip-1	Y75B8A.35	0.279877864897587	AGIWUR10170
CB4856	CT	III	12345323	12351336	WBGene00004085	pph-4.1	Y75B8A.30	0.161729810887017	AGIWUR12115
CB4856	CT	III	12351642	12354925	WBGene00013561	Y75B8A.31	Y75B8A.31	-0.0331863805120207	AGIWUR12456
CB4856	CT	III	12354944	12358561	WBGene00013562	Y75B8A.32	Y75B8A.32	-0.120505829707487	AGIWUR36648
CB4856	CT	III	12359565	12361248	WBGene00013564	Y75B8A.34	Y75B8A.34	0.0379160535886758	AGIWUR19352;AGIWUR29568;AGIWUR38384
CB4856	CT	III	12361859	12367373	WBGene00013563	Y75B8A.33	Y75B8A.33	0.0794545553006838	AGIWUR20947;AGIWUR37137;AGIWUR37178
CB4856	CT	III	12367474	12369050	WBGene00001946	his-72	Y49E10.6	0.277032728569791	AGIWUR13672;AGIWUR22973;AGIWUR24494;AGIWUR28018;AGIWUR42852
CB4856	CT	III	12370456	12372625	WBGene00004506	rpt-6	Y49E10.1	0.0444643256784119	AGIWUR41530
CB4856	CT	III	12372583	12373857	WBGene00013029	glrx-5	Y49E10.2	-0.10117698856255	AGIWUR36243
CB4856	CT	III	12374126	12377248	WBGene00004086	pph-4.2	Y49E10.3	-0.124807384564722	AGIWUR6473;AGIWUR7787;AGIWUR15115;AGIWUR24606;AGIWUR27782;AGIWUR33390;AGIWUR42004;AGIWUR43090
CB4856	CT	III	12377731	12378829	WBGene00013031	Y49E10.7	Y49E10.7	0.159080314428275	AGIWUR33767
CB4856	CT	III	12378724	12380647	WBGene00013030	Y49E10.4	Y49E10.4	-0.175925692093802	AGIWUR12131;AGIWUR22301;AGIWUR32211;AGIWUR38930
CB4856	CT	III	12387891	12388985	WBGene00014904	Y49E10.26	Y49E10.26	-0.20304791741744	AGIWUR30780

CB4856 CT III 12389885 12390556 WBGene00014903 Y49E10.8 Y49E10.8
0.472026082830336 AGIWUR24628
CB4856 CT III 12398901 12421570 WBGene00013034 tat-1 Y49E10.11
-0.133672971167618 AGIWUR15617;AGIWUR18057;AGIWUR27355;AGIWUR34118
CB4856 CT III 12426862 12429195 WBGene00004027 pie-1 Y49E10.14
-0.0108675004166239 AGIWUR4842;AGIWUR43801
CB4856 CT III 12429172 12429630 WBGene00004919 snr-6 Y49E10.15
-1.77635683940025e-15 AGIWUR43554
CB4856 CT III 12430430 12433892 WBGene00013035 Y49E10.16 Y49E10.16
0.335581648414067 AGIWUR15845;AGIWUR27141;AGIWUR43362
CB4856 CT III 12436552 12441056 WBGene00013036 fbxa-218 Y49E10.17
-0.321781296003233 AGIWUR23422
CB4856 CT III 12442630 12446145 WBGene00013037 Y49E10.18 Y49E10.18
0.10956819209496 AGIWUR10529;AGIWUR24845;AGIWUR26960;AGIWUR29787;AGIWUR32268;AGIWUR34014
CB4856 CT III 12446353 12453202 WBGene00013038 ani-1 Y49E10.19
0.0737627797232641 AGIWUR4746
CB4856 CT III 12453588 12456850 WBGene00013039 scav-3 Y49E10.20
0.0727113571894638 AGIWUR22103;AGIWUR31816;AGIWUR37790;AGIWUR43918
CB4856 CT III 12457599 12465821 WBGene00013040 Y49E10.21 Y49E10.21
-0.129574201081947 AGIWUR105;AGIWUR15140;AGIWUR38802
CB4856 CT III 12466610 12468277 WBGene00013041 pstk-1 Y49E10.22
-0.00899450577567507 AGIWUR13994
CB4856 CT III 12469169 12472541 WBGene00045239 Y49E10.29 Y49E10.29
-0.00956154995777592 AGIWUR11208;AGIWUR22942;AGIWUR28929;AGIWUR33709;AGIWUR34350;AGIWUR38560
CB4856 CT III 12472868 12484651 WBGene00013042 cccp-1 Y49E10.23
-0.0791818809173641 AGIWUR13894
CB4856 CT III 12484732 12488339 WBGene00013043 arrd-16 Y49E10.24
-0.0483771259029977 AGIWUR7495;AGIWUR21391;AGIWUR42175
CB4856 CT III 12488679 12492084 WBGene00013044 Y49E10.25 Y49E10.25
-0.445137590290381 AGIWUR10386
CB4856 CT III 12492793 12495640 WBGene00013727 Y111B2A.1 Y111B2A.1
-0.250788023342642 AGIWUR15820
CB4856 CT III 12496538 12499352 WBGene00013729 Y111B2A.3 Y111B2A.3
-0.0858548866537898 AGIWUR8789
CB4856 CT III 12499557 12527924 WBGene00013730 sql-1 Y111B2A.4
-0.14204328285097 AGIWUR4403;AGIWUR5215;AGIWUR17830;AGIWUR26217;AGIWUR27811
CB4856 CT III 12528287 12536848 WBGene00014613 Y111B2A.25 Y111B2A.25
-0.0421322424312915 AGIWUR5020;AGIWUR7629;AGIWUR17399;AGIWUR32936;AGIWUR37551
CB4856 CT III 12538664 12575401 WBGene00013732 aakg-1 Y111B2A.8

0.279483902723097	AGIWUR17518;AGIWUR19289;AGIWUR39040;AGIWUR41104;AGIWUR42689
CB4856	CT	III	12595994	12598604	WBGene00013734	Y111B2A.10	Y111B2A.10
0.180453099168765	AGIWUR31114;AGIWUR32437
CB4856	CT	III	12599304	12608604	WBGene00007030	epc-1	Y111B2A.11
-0.0579887566699888	AGIWUR24947
CB4856	CT	III	12609289	12623740	WBGene00013735	Y111B2A.12	Y111B2A.12
-0.123139977692932	AGIWUR1326;AGIWUR15735;AGIWUR23494;AGIWUR25188;AGIWUR27817;AGIWUR29975
CB4856	CT	III	12624766	12625890	WBGene00013736	Y111B2A.13	Y111B2A.13
0.076405544193392	AGIWUR7917;AGIWUR20589;AGIWUR21701;AGIWUR22852;AGIWUR24926
CB4856	CT	III	12626568	12651757	WBGene00004161	pqn-80	Y111B2A.14
0.0542869069394749	AGIWUR3950;AGIWUR19275;AGIWUR36462;AGIWUR45159
CB4856	CT	III	12652344	12662286	WBGene00013737	tpst-1	Y111B2A.15
-0.140485954229032	AGIWUR30331
CB4856	CT	III	12671307	12673421	WBGene00006389	taf-7.2	Y111B2A.16
-0.17031817351989	AGIWUR31423
CB4856	CT	III	12673633	12686230	WBGene00013738	elpc-2	Y111B2A.17
0.0773947744114114	AGIWUR37084
CB4856	CT	III	12686243	12687916	WBGene00004700	rsp-3	Y111B2A.18
-0.129715819847178	AGIWUR5188;AGIWUR13561;AGIWUR19588
CB4856	CT	III	12699494	12710888	WBGene00013742	sas-1	Y111B2A.24
-0.182574208945145	AGIWUR24021
CB4856	CT	III	12711506	12713874	WBGene00013740	hut-1	Y111B2A.20
-0.335176399017795	AGIWUR14912
CB4856	CT	III	12714566	12743929	WBGene00007027	ssl-1	Y111B2A.22
0.0326573086271154	AGIWUR15732;AGIWUR31590;AGIWUR43765
CB4856	CT	III	12746450	12747621	WBGene00013741	Y111B2A.21	Y111B2A.21
0.105283954886301	AGIWUR15341
CB4856	CT	III	12747899	12750927	WBGene00013911	irld-60	ZC482.2	0.153019175697439
AGIWUR8989
CB4856	CT	III	12751109	12753634	WBGene00013915	irld-20	ZC482.7
-0.000426039884002805	AGIWUR6282
CB4856	CT	III	12755348	12756613	WBGene00005757	srw-10	ZC482.6	0.341984249518188
AGIWUR3866;AGIWUR16156;AGIWUR34190
CB4856	CT	III	12757667	12759619	WBGene00005756	srw-9	ZC482.8	-0.0930292101729204
AGIWUR4324
CB4856	CT	III	12760684	12764341	WBGene00013912	irld-19	ZC482.3	-0.0445055224973867
AGIWUR17913
CB4856	CT	III	12764553	12769048	WBGene00013913	irld-61	ZC482.4	-0.0441071206248225
AGIWUR489;AGIWUR22473;AGIWUR34972

CB4856 CT III 12769564 12771594 WBGene00005752 srw-5 ZC482.9 0.0991243599436595
AGIWUR1998;AGIWUR30084
CB4856 CT III 12771878 12779726 WBGene00001512 gab-1 ZC482.1 0.0480821754576048
AGIWUR28927;AGIWUR31006;AGIWUR38849
CB4856 CT III 12785154 12793128 WBGene00013914 lgc-37 ZC482.5 0.0195881097230264
AGIWUR454;AGIWUR11920;AGIWUR37953;AGIWUR45122
CB4856 CT III 12793386 12798157 WBGene00007210 agmo-1 BE10.2 -0.183105743043701
AGIWUR5835;AGIWUR38227
CB4856 CT III 12804599 12804761 WBGene00044128 BE10.6 BE10.6 -0.049172528467988
AGIWUR12532
CB4856 CT III 12806175 12806855 WBGene00044127 BE10.5 BE10.5 -0.0792088560269413
AGIWUR6016;AGIWUR6428;AGIWUR33191;AGIWUR37207;AGIWUR42255
CB4856 CT III 12813541 12816798 WBGene00007212 BE10.4 BE10.4 0.0403389758680182
AGIWUR5283;AGIWUR14355;AGIWUR17624;AGIWUR23051
CB4856 CT III 12816789 12818100 WBGene00000203 arx-5 Y37D8A.1
-0.242106431341367 AGIWUR8540;AGIWUR16491;AGIWUR26323;AGIWUR42562
CB4856 CT III 12818658 12820252 WBGene00012560 Y37D8A.25 Y37D8A.25
-0.309406338061388 AGIWUR1537
CB4856 CT III 12820141 12825651 WBGene00012544 Y37D8A.2 Y37D8A.2
-0.191072880264912 AGIWUR281
CB4856 CT III 12829368 12837274 WBGene00012545 Y37D8A.3 Y37D8A.3
0.0375207745451309 AGIWUR42989
CB4856 CT III 12837189 12838959 WBGene00012546 Y37D8A.4 Y37D8A.4
0.11732744913976 AGIWUR26272
CB4856 CT III 12854051 12855315 WBGene00012547 Y37D8A.5 Y37D8A.5
0.181148901518162 AGIWUR12221
CB4856 CT III 12855844 12856728 WBGene00012561 Y37D8A.26 Y37D8A.26
-0.520860216956528 AGIWUR29792
CB4856 CT III 12856833 12857911 WBGene00012548 Y37D8A.6 Y37D8A.6
-0.379178626175035 AGIWUR19054
CB4856 CT III 12864948 12867591 WBGene00012549 Y37D8A.8 Y37D8A.8
0.259183267060502 AGIWUR42474
CB4856 CT III 12871195 12874654 WBGene00003406 mrg-1 Y37D8A.9
-0.202956279616092 AGIWUR13050;AGIWUR24765;AGIWUR32619
CB4856 CT III 12875007 12875831 WBGene00012550 hpo-21 Y37D8A.10
-0.0396437759107258 AGIWUR36621
CB4856 CT III 12875771 12877577 WBGene00012551 cec-7 Y37D8A.11
-0.153446910188308 AGIWUR21609
CB4856 CT III 12881037 12891738 WBGene00006804 unc-71 Y37D8A.13

-0.0386898386141858	AGIWUR35864
CB4856	CT	III	12905491	12906619	WBGene00012553	cco-2	Y37D8A.14
-0.0764782343140702
AGIWUR611;AGIWUR5718;AGIWUR11609;AGIWUR18775;AGIWUR29351;AGIWUR29790;AGIWUR39376;AGIWUR42144
CB4856	CT	III	12910510	12914930	WBGene00001457	flp-14	Y37D8A.15
0.542840835348304	AGIWUR36571
CB4856	CT	III	12915118	12918675	WBGene00012554	Y37D8A.16	Y37D8A.16
0.705427496044261	AGIWUR1963;AGIWUR9465;AGIWUR12505;AGIWUR19886
CB4856	CT	III	12921282	12925997	WBGene00012555	Y37D8A.17	Y37D8A.17
-0.0827555350315876	AGIWUR34460
CB4856	CT	III	12926557	12927886	WBGene00012556	mrps-10	Y37D8A.18
-0.0168659785141791	AGIWUR32331
CB4856	CT	III	12928370	12931867	WBGene00012558	Y37D8A.21	Y37D8A.21
-0.157504879511073	AGIWUR8071
CB4856	CT	III	12934717	12941069	WBGene00012559	epg-3	Y37D8A.22
-0.147480831041957	AGIWUR15032
CB4856	CT	III	12942617	12949899	WBGene00006762	unc-25	Y37D8A.23
0.000426278858800089	AGIWUR1644;AGIWUR15904;AGIWUR33380
CB4856	CT	III	12951429	12952342	WBGene00012711	Y39E4A.1	Y39E4A.1
0.14515644334066	AGIWUR25309
CB4856	CT	III	12952969	12966995	WBGene00012712	ttm-1	Y39E4A.2
-0.158150020754157	AGIWUR32890
CB4856	CT	III	12967898	12972498	WBGene00012713	bckd-1A	Y39E4A.3
-0.237948698887021	AGIWUR27070;AGIWUR27757;AGIWUR28392;AGIWUR44309
CB4856	CT	III	12975068	12977862	WBGene00014176	ZK1010.2	ZK1010.2
0.158014054148239	AGIWUR29594
CB4856	CT	III	12977910	12978523	WBGene00006728	ubq-2	ZK1010.1
0.00719375016839994	AGIWUR20435;AGIWUR37225
CB4856	CT	III	12979162	12980163	WBGene00014177	frg-1	ZK1010.3
-0.228012183939659	AGIWUR3936;AGIWUR5758;AGIWUR6372;AGIWUR13721;AGIWUR15815;AGIWUR41903
CB4856	CT	III	12981070	12984423	WBGene00014178	ZK1010.4	ZK1010.4
-0.0618404228550933	AGIWUR27206
CB4856	CT	III	12984493	12987542	WBGene00014179	ZK1010.5	ZK1010.5
0.0630796690090474	AGIWUR17826
CB4856	CT	III	12987672	12992492	WBGene00014180	ZK1010.6	ZK1010.6
-0.0540375855350455	AGIWUR37174
CB4856	CT	III	12992405	12993707	WBGene00000672	col-97	ZK1010.7
-0.674327968342709	AGIWUR142;AGIWUR14870;AGIWUR16747;AGIWUR17138;AGIWUR42663
CB4856	CT	III	12995738	13000670	WBGene00014181	ZK1010.8	ZK1010.8

0.538709284703936	AGIWUR29793
CB4856	CT	III	13005461	13011130	WBGene00004906	snf-7	ZK1010.9
-0.107185838314786	AGIWUR16387
CB4856	CT	III	13018935	13020226	WBGene00000673	col-98	F14F7.1	-0.15752499627175
AGIWUR16447
CB4856	CT	III	13021009	13023949	WBGene00008809	cyp-13A11	F14F7.2
-0.0723459300389591	AGIWUR22639
CB4856	CT	III	13024547	13028012	WBGene00008810	cyp-13A12	F14F7.3
-0.380585956744056	AGIWUR44517
CB4856	CT	III	13028393	13029708	WBGene00008811	F14F7.4	F14F7.4	-0.151334952941143
AGIWUR6007
CB4856	CT	III	13032001	13036344	WBGene00008812	F14F7.5	F14F7.5	-0.423548828342327
AGIWUR42336
CB4856	CT	III	13040100	13049434	WBGene00010073	F54F12.2	F54F12.2
-0.227921264471346	AGIWUR36521
CB4856	CT	III	13049588	13055485	WBGene00010072	F54F12.1	F54F12.1
0.372449694923244	AGIWUR38600
CB4856	CT	III	13070756	13074697	WBGene00012717	Y39E4B.6	Y39E4B.6
0.227968355308535
AGIWUR1705;AGIWUR3123;AGIWUR5764;AGIWUR6739;AGIWUR9897;AGIWUR11950;AGIWUR14953;AGIWUR16922
CB4856	CT	III	13087479	13099455	WBGene00004164	pqn-83	Y39E4B.3
-0.285054107879332	AGIWUR3211;AGIWUR22318
CB4856	CT	III	13107607	13119042	WBGene00012718	dhhc-8	Y39E4B.7
-0.250734822246252	AGIWUR17580;AGIWUR37080;AGIWUR41866
CB4856	CT	III	13119820	13133938	WBGene00006985	zig-8	Y39E4B.8
0.0292969706160413	AGIWUR17456
CB4856	CT	III	13134014	13139614	WBGene00000267	bre-2	Y39E4B.9
0.0129759462271331	AGIWUR38640
CB4856	CT	III	13143373	13152858	WBGene00012719	Y39E4B.10	Y39E4B.10
-0.38797344869103	AGIWUR33059
CB4856	CT	III	13153817	13160284	WBGene00012714	abce-1	Y39E4B.1
-0.339001888736291	AGIWUR6306
CB4856	CT	III	13162475	13164864	WBGene00014884	Y39E4B.14	Y39E4B.14
0.178706023891408	AGIWUR12326;AGIWUR36130;AGIWUR36504
CB4856	CT	III	13165659	13169520	WBGene00012715	snpc-1.2	Y39E4B.2
-0.0694812527499327	AGIWUR31060
CB4856	CT	III	13169916	13173106	WBGene00012716	Y39E4B.5	Y39E4B.5
0.142368124007756	AGIWUR43541
CB4856	CT	III	13187392	13204897	WBGene00001630	gly-5	Y39E4B.12

0.0720758723446027	AGIWUR9158;AGIWUR10483;AGIWUR11255;AGIWUR15746;AGIWUR16404;AGIWUR18625
CB4856	CT	III	13204982	13207393	WBGene00012720	Y39E4B.11	Y39E4B.11
-0.24935889314734	AGIWUR445;AGIWUR13620;AGIWUR31679
CB4856	CT	III	13212666	13214371	WBGene00006629	tsp-3	Y39E4B.4
0.297110550834768	AGIWUR20275;AGIWUR27390;AGIWUR42535
CB4856	CT	III	13223072	13229465	WBGene00012796	Y43F4A.1	Y43F4A.1
0.175901413050013	AGIWUR3573;AGIWUR8389;AGIWUR13912;AGIWUR15547;AGIWUR19301;AGIWUR23348;AGIWUR38773
CB4856	CT	III	13254675	13256456	WBGene00185001	F56A8.9 F56A8.9 -0.0342263213861145
AGIWUR12170
CB4856	CT	III	13256878	13262905	WBGene00010139	F56A8.3 F56A8.3 -0.0428119040299064
AGIWUR10753;AGIWUR11459;AGIWUR13812;AGIWUR14851;AGIWUR20482;AGIWUR33389;AGIWUR34133;AGIWUR35104;AGIWUR37135
;AGIWUR37646
CB4856	CT	III	13263268	13265038	WBGene00010140	F56A8.4 F56A8.4 -0.0698672212885345
AGIWUR38160
CB4856	CT	III	13265866	13266932	WBGene00010141	F56A8.5 F56A8.5 0.0504400024826257
AGIWUR4737
CB4856	CT	III	13266874	13268897	WBGene00000774	cpf-2	F56A8.6
-0.00707718679931091	AGIWUR820;AGIWUR36539;AGIWUR44328
CB4856	CT	III	13277261	13283883	WBGene00006798	unc-64	F56A8.7 0.0504119729722774
AGIWUR4899;AGIWUR14105;AGIWUR27209;AGIWUR27682;AGIWUR28092;AGIWUR28579;AGIWUR30962;AGIWUR34538;AGIWUR37059
CB4856	CT	III	13283892	13293088	WBGene00012802	set-25	Y43F4B.3
-0.107628606382166
AGIWUR6872;AGIWUR10552;AGIWUR11103;AGIWUR20458;AGIWUR33492;AGIWUR35107;AGIWUR37662;AGIWUR39018
CB4856	CT	III	13296853	13299122	WBGene00003804	npp-18	Y43F4B.4
-0.123458023064025	AGIWUR1607;AGIWUR7364
CB4856	CT	III	13299378	13302760	WBGene00012803	Y43F4B.5	Y43F4B.5
-0.0806120717988019	AGIWUR12941;AGIWUR17119;AGIWUR18458;AGIWUR30210
CB4856	CT	III	13302743	13307259	WBGene00002229	klp-19	Y43F4B.6
-0.14990757436511	AGIWUR17050;AGIWUR26586;AGIWUR31738;AGIWUR38118;AGIWUR39195;AGIWUR39404
CB4856	CT	III	13307502	13313389	WBGene00012804	Y43F4B.7	Y43F4B.7
0.00888264738548372	AGIWUR45165
CB4856	CT	III	13314011	13314721	WBGene00012807	Y43F4B.10	Y43F4B.10
-0.059530417803753	AGIWUR3272
CB4856	CT	III	13315088	13318597	WBGene00012806	Y43F4B.9	Y43F4B.9
-0.101282652671987	AGIWUR2346;AGIWUR5832;AGIWUR10132;AGIWUR20720;AGIWUR31478;AGIWUR32629
CB4856	CT	III	13319265	13321892	WBGene00194870	F53A2.11	F53A2.11
-0.0633203251436409	AGIWUR27265
CB4856	CT	III	13334600	13336422	WBGene00009950	sdz-20	F53A2.2 0.142932954808122
AGIWUR36368

CB4856 CT III 13336940 13339768 WBGene00009951 F53A2.3 F53A2.3 -0.307206511652129
AGIWUR1393
CB4856 CT III 13341527 13342607 WBGene00003829 nud-1 F53A2.4 -0.260641925340256
AGIWUR7935;AGIWUR15431;AGIWUR29058;AGIWUR34024;AGIWUR35551;AGIWUR45037
CB4856 CT III 13342684 13343337 WBGene00001092 dro-1 F53A2.5 -0.429988922005787
AGIWUR18467
CB4856 CT III 13343574 13344616 WBGene00002059 ife-1 F53A2.6 -0.0931803381315319
AGIWUR10155;AGIWUR12605;AGIWUR23772;AGIWUR29803;AGIWUR38165
CB4856 CT III 13344917 13346866 WBGene00009952 acaa-2 F53A2.7 -0.174977916199916
AGIWUR17228;AGIWUR29233;AGIWUR44741
CB4856 CT III 13347085 13355683 WBGene00003478 mtm-6 F53A2.8 -0.0482725426055821
AGIWUR13022;AGIWUR20098;AGIWUR41009;AGIWUR43895
CB4856 CT III 13356610 13359354 WBGene00009953 F53A2.9 F53A2.9 0.168260587476682
AGIWUR15428
CB4856 CT III 13361299 13364765 WBGene00044229 T03F6.8 T03F6.8 -0.438849339195051
AGIWUR5376;AGIWUR39849
CB4856 CT III 13365425 13367747 WBGene00044228 T03F6.7 T03F6.7 -0.267484251736976
AGIWUR7179;AGIWUR21758
CB4856 CT III 13368087 13371072 WBGene00045482 T03F6.9 T03F6.9 -0.0707441665969668
AGIWUR31129;AGIWUR33220;AGIWUR43152
CB4856 CT III 13371175 13376710 WBGene00003047 lis-1 T03F6.5 0.365434339189861
AGIWUR9723
CB4856 CT III 13377830 13378342 WBGene00185005 T03F6.10 T03F6.10
-0.578022846278335 AGIWUR20586
CB4856 CT III 13386150 13388353 WBGene00011400 T03F6.4 T03F6.4 0.193953089283058
AGIWUR11909;AGIWUR29629
CB4856 CT III 13389399 13390857 WBGene00011399 T03F6.3 T03F6.3 -0.236533657139594
AGIWUR5191;AGIWUR9832;AGIWUR24981;AGIWUR25586;AGIWUR32831;AGIWUR41743
CB4856 CT III 13390850 13393339 WBGene00001035 dnj-17 T03F6.2 -0.162906219389509
AGIWUR42653
CB4856 CT III 13393910 13394979 WBGene00011398 qdpr-1 T03F6.1 0.00696674483640791
AGIWUR43190
CB4856 CT III 13395633 13400563 WBGene00008714 F11F1.1 F11F1.1 -0.336305160582603
AGIWUR36166
CB4856 CT III 13400962 13401773 WBGene00008716 F11F1.4 F11F1.4 -0.181301521840837
AGIWUR45038
CB4856 CT III 13403234 13404514 WBGene00003249 mig-18 F11F1.6 0.431358090827773
AGIWUR34265
CB4856 CT III 13404737 13405645 WBGene00008719 ttr-52 F11F1.7 0.559283951282716

AGIWUR4329;AGIWUR6840;AGIWUR26823;AGIWUR27520;AGIWUR33186;AGIWUR34462;AGIWUR38554;AGIWUR45068
CB4856 CT III 13405965 13409292 WBGene00003520 nas-1 F45G2.1 -0.271275505760485
AGIWUR7337
CB4856 CT III 13409895 13423537 WBGene00009730 myo-6 F45G2.2 0.312089807081991
AGIWUR9322
CB4856 CT III 13423514 13426204 WBGene00009731 exo-1 F45G2.3 -0.200161475840876
AGIWUR34496
CB4856 CT III 13426100 13427715 WBGene00009732 cope-1 F45G2.4 -0.225333462412923
AGIWUR20389
CB4856 CT III 13427642 13429713 WBGene00000255 bli-5 F45G2.5 -0.629229762381864
AGIWUR30064
CB4856 CT III 13430462 13433394 WBGene00006612 trf-1 F45G2.6 -0.335596392193214
AGIWUR18199
CB4856 CT III 13433447 13434504 WBGene00009733 F45G2.7 F45G2.7 0.0545743146788702
AGIWUR31301
CB4856 CT III 13434861 13435670 WBGene00009734 F45G2.8 F45G2.8 -0.140037662442428
AGIWUR8725
CB4856 CT III 13435771 13436943 WBGene00009735 F45G2.9 F45G2.9
-0.00854260689345487 AGIWUR15689;AGIWUR32039;AGIWUR40076
CB4856 CT III 13437054 13439586 WBGene00009736 F45G2.10 F45G2.10
0.162691097872703 AGIWUR41087
CB4856 CT III 13439730 13455307 WBGene00000834 cua-1 Y76A2A.2
-0.158868326247047 AGIWUR21350
CB4856 CT III 13457687 13462238 WBGene00012097 abcf-2 T27E9.7 0.0123563201288839
AGIWUR9395;AGIWUR15925;AGIWUR26658;AGIWUR29496;AGIWUR39154
CB4856 CT III 13462239 13462594 WBGene00012094 T27E9.2 T27E9.2 -0.139996487572649
AGIWUR44257
CB4856 CT III 13463163 13464657 WBGene00006439 ant-1.1 T27E9.1 0.0755889729616488
AGIWUR9898;AGIWUR15073;AGIWUR22154;AGIWUR23463;AGIWUR23557;AGIWUR24064;AGIWUR24262;AGIWUR24722;AGIWUR25282;
AGIWUR26293;AGIWUR27044;AGIWUR30394
CB4856 CT III 13464829 13471348 WBGene00002185 kel-3 T27E9.4 -0.0449452393227556
AGIWUR39681
CB4856 CT III 13471708 13475385 WBGene00012095 pssy-2 T27E9.5 -0.0557242631265744
AGIWUR30488
CB4856 CT III 13475406 13476107 WBGene00012096 T27E9.6 T27E9.6 0.0180666147309108
AGIWUR32016
CB4856 CT III 13478561 13479629 WBGene00012098 fbxb-86 T27E9.8 0.0815063406423455
AGIWUR728
CB4856 CT III 13480665 13484155 WBGene00012099 acc-4 T27E9.9 0.254559216037014

AGIWUR43609
CB4856 CT III 13486643 13492430 WBGene00006691 twk-40 T28A8.1 -0.072443814607019
AGIWUR11101;AGIWUR18718;AGIWUR21352
CB4856 CT III 13493892 13495671 WBGene00012108 T28A8.2 T28A8.2 -0.372077581097869
AGIWUR17099
CB4856 CT III 13499199 13501510 WBGene00012112 T28A8.6 T28A8.6 0.038863141564895
AGIWUR4827;AGIWUR27619;AGIWUR28503
CB4856 CT III 13502813 13503578 WBGene00194852 T28A8.8 T28A8.8 0.167166741993769
AGIWUR5587
CB4856 CT III 13503679 13505536 WBGene00012110 T28A8.4 T28A8.4 -0.224995757005015
AGIWUR32059
CB4856 CT III 13506018 13508650 WBGene00012109 T28A8.3 T28A8.3 -0.142195021432624
AGIWUR27068
CB4856 CT III 13509301 13514570 WBGene00003373 mlh-1 T28A8.7 0.338585131614892
AGIWUR32352
CB4856 CT III 13514945 13524110 WBGene00004075 pod-1 Y76A2B.1
-0.0923403818439108 AGIWUR15436;AGIWUR24420;AGIWUR33016;AGIWUR34472;AGIWUR37115;AGIWUR43659
CB4856 CT III 13527438 13539971 WBGene00013574 lron-13 Y76A2B.2
0.170404586827381 AGIWUR25269
CB4856 CT III 13540139 13543534 WBGene00013575 acs-5 Y76A2B.3
-0.222021563692172 AGIWUR9452;AGIWUR9888;AGIWUR28530;AGIWUR31495;AGIWUR36463;AGIWUR43869;AGIWUR44980
CB4856 CT III 13545245 13546777 WBGene00013576 Y76A2B.4 Y76A2B.4
0.0328108670009968 AGIWUR28901
CB4856 CT III 13546889 13549020 WBGene00013577 Y76A2B.5 Y76A2B.5
0.142493313645174 AGIWUR2571
CB4856 CT III 13550699 13555334 WBGene00013578 scav-2 Y76A2B.6
-0.353636832489613 AGIWUR3260
CB4856 CT III 13561729 13563082 WBGene00011475 T05D4.2 T05D4.2 0.389013935186192
AGIWUR7893
CB4856 CT III 13563346 13564990 WBGene00011474 aldo-1 T05D4.1 -0.11840360029409
AGIWUR4857;AGIWUR14566
CB4856 CT III 13566874 13570447 WBGene00011476 T05D4.3 T05D4.3 -0.095751123500758
AGIWUR23312
CB4856 CT III 13576188 13580662 WBGene00003887 osm-7 T05D4.4 0.0170172276482425
AGIWUR27770
CB4856 CT III 13580850 13583589 WBGene00011478 T05D4.5 T05D4.5 0.151190967212775
AGIWUR33116
CB4856 CT III 13601760 13603156 WBGene00001605 gln-4 T25C8.3 0.230537873025309
AGIWUR11582

CB4856	CT	III	13604339	13606067	WBGene00000067	act-5	T25C8.2	-0.37659395868835	AGIWUR17983;AGIWUR31487;AGIWUR45107
CB4856	CT	III	13608072	13614704	WBGene00012017	T25C8.1	T25C8.1	-0.335761954097125	AGIWUR34341
CB4856	CT	III	13614699	13622489	WBGene00011735	hip-1	T12D8.8	-0.0323158090160067	AGIWUR3805;AGIWUR19795;AGIWUR30111;AGIWUR38096
CB4856	CT	III	13623279	13624172	WBGene00011734	mlc-5	T12D8.6	-0.190218126136557	AGIWUR11723
CB4856	CT	III	13624195	13626007	WBGene00006391	taf-9	T12D8.7	-0.164974337285201	AGIWUR19328
CB4856	CT	III	13625968	13626750	WBGene00011733	T12D8.5	T12D8.5	2.75038616724689	AGIWUR44006
CB4856	CT	III	13626994	13630808	WBGene00011732	arrd-17	T12D8.4	0.205126207019357	AGIWUR9273;AGIWUR23751;AGIWUR25808;AGIWUR30362
CB4856	CT	III	13639893	13641688	WBGene00011731	acbp-5	T12D8.3	0.0448929809866891	AGIWUR15630;AGIWUR23888;AGIWUR25272;AGIWUR27449;AGIWUR38615;AGIWUR39949
CB4856	CT	III	13641795	13642327	WBGene00023450	T12D8.10	T12D8.10	0.50155154173482	AGIWUR44933
CB4856	CT	III	13642420	13643570	WBGene00011730	drr-2	T12D8.2	0.0761544754314691	AGIWUR27572;AGIWUR36635
CB4856	CT	III	13643916	13656637	WBGene00011729	set-16	T12D8.1	0.220133051723181	AGIWUR27385;AGIWUR40824
CB4856	CT	III	13663400	13667839	WBGene00001458	flp-15	ZK525.1	0.518755377524514	AGIWUR11195
CB4856	CT	III	13673280	13674746	WBGene00000179	aqp-11	ZK525.2	-0.322341088926464	AGIWUR16789
CB4856	CT	III	13674917	13686445	WBGene00001118	dyf-2	ZK520.3	0.772332499135038	AGIWUR11571
CB4856	CT	III	13687199	13692683	WBGene00000837	cul-2	ZK520.4	0.00404112822316804	AGIWUR20;AGIWUR1441;AGIWUR1540;AGIWUR13518;AGIWUR13995;AGIWUR14723;AGIWUR23792;AGIWUR28327;AGIWUR41222;AGIWUR42608
CB4856	CT	III	13692668	13693811	WBGene00000878	cyn-2	ZK520.5	0.0355019492755186	AGIWUR39498
CB4856	CT	III	13701993	13724401	WBGene00003048	lit-1	W06F12.1	0.514876183820229	AGIWUR6941;AGIWUR12354;AGIWUR32350
CB4856	CT	III	13726641	13731679	WBGene00012306	W06F12.2	W06F12.2	0.427988329100982	AGIWUR33882;AGIWUR37683;AGIWUR40574
CB4856	CT	III	13735071	13736541	WBGene00012307	W06F12.3	W06F12.3	0.157568161779992	AGIWUR25203

CB4856	CT	III	13744222	13747287	WBGene00010661	tyr-2	K08E3.1	-0.240551389670614	AGIWUR5503
CB4856	CT	III	13747472	13748686	WBGene00010662	K08E3.2	K08E3.2	0.326895895710781	AGIWUR9724
CB4856	CT	III	13752406	13755861	WBGene00023451	mlc-7	K08E3.10	0.31654011571252	AGIWUR4778;AGIWUR24245;AGIWUR35195;AGIWUR44855
CB4856	CT	III	13755896	13759434	WBGene00010663	toca-2	K08E3.3	-0.102095009650911	AGIWUR13679;AGIWUR25876;AGIWUR25927;AGIWUR33111
CB4856	CT	III	13759444	13762069	WBGene00010664	dbn-1	K08E3.4	0.0110560373211808	AGIWUR34377
CB4856	CT	III	13764030	13768092	WBGene00010665	K08E3.5	K08E3.5	-0.0926842187048447	AGIWUR9559;AGIWUR16248;AGIWUR17000;AGIWUR18925;AGIWUR25456;AGIWUR26080;AGIWUR27983;AGIWUR30514
CB4856	CT	III	13768541	13771242	WBGene00000875	cyk-4	K08E3.6	-0.0171588384460839	AGIWUR11676;AGIWUR19991;AGIWUR24305;AGIWUR25234
CB4856	CT	III	13771448	13773348	WBGene00003967	pdr-1	K08E3.7	0.0365646699522291	AGIWUR30328
CB4856	CT	III	13773456	13778896	WBGene00007025	mdt-29	K08E3.8	-0.0580452292888647	AGIWUR9180;AGIWUR10608
CB4856	CT	III	13780220	13781133	WBGene00007065	pot-3	3R5.1	0.159707689858744	AGIWUR9200
CB4856	CT	IV	32481	48880	WBGene00001492	frm-5.1	Y38C1AB.8	-0.187930714118707	AGIWUR11307;AGIWUR23830
CB4856	CT	IV	86890	90082	WBGene00015246	scl-23	B0545.3	-0.296872495655075	AGIWUR11368
CB4856	CT	IV	112408	118629	WBGene00001111	duo-2	F29C4.5	-0.115514661449216	AGIWUR31597
CB4856	CT	IV	119272	121707	WBGene00017928	tut-1	F29C4.6	-0.0077551644566034	AGIWUR33251;AGIWUR36931
CB4856	CT	IV	121797	126225	WBGene00017929	grld-1	F29C4.7	-0.0813566226550266	AGIWUR23201;AGIWUR26391
CB4856	CT	IV	126731	127640	WBGene00017926	F29C4.2	F29C4.2	-0.0644637495778966	AGIWUR6362;AGIWUR10252;AGIWUR12097;AGIWUR12529;AGIWUR16172;AGIWUR23544;AGIWUR27558;AGIWUR31184;AGIWUR37211;AGIWUR37885;AGIWUR40280
CB4856	CT	IV	132416	138076	WBGene00000897	daf-1	F29C4.1	-0.112168891610033	AGIWUR34627
CB4856	CT	IV	138346	147919	WBGene00000674	col-99	F29C4.8	0.187911211564308	AGIWUR2432;AGIWUR9402
CB4856	CT	IV	156071	157901	WBGene00021400	Y38C1AA.9	Y38C1AA.9	0.16710644009332	AGIWUR3829
CB4856	CT	IV	158962	159836	WBGene00021398	Y38C1AA.7	Y38C1AA.7	-0.0819549811516427	AGIWUR10389;AGIWUR17218;AGIWUR26513;AGIWUR30309;AGIWUR31337;AGIWUR38359
CB4856	CT	IV	162830	164814	WBGene00021397	Y38C1AA.6	Y38C1AA.6	0.393223868725451	

AGIWUR38082
CB4856 CT IV 165077 169980 WBGene00021396 cutl-24 Y38C1AA.5 0.280036985868891
AGIWUR7334;AGIWUR11652;AGIWUR32978
CB4856 CT IV 179008 183261 WBGene00006561 tcl-2 Y38C1AA.4 0.68946414354812
AGIWUR34449
CB4856 CT IV 184554 192151 WBGene00021395 pnc-1 Y38C1AA.3 -0.104926991537361
AGIWUR1594;AGIWUR5204
CB4856 CT IV 192256 194745 WBGene00021402 Y38C1AA.12 Y38C1AA.12 -0.330673013698548
AGIWUR6999;AGIWUR31095
CB4856 CT IV 194745 202586 WBGene00000815 csn-3 Y38C1AA.2 -0.0736241460440858
AGIWUR42984
CB4856 CT IV 205239 208049 WBGene00195063 Y38C1AA.14 Y38C1AA.14 -0.149589657096621
AGIWUR12592;AGIWUR34861
CB4856 CT IV 208189 217390 WBGene00021394 Y38C1AA.1 Y38C1AA.1 -0.0534385607947523
AGIWUR10055;AGIWUR19059;AGIWUR22478;AGIWUR30888;AGIWUR35437;AGIWUR44502
CB4856 CT IV 218551 220854 WBGene00021401 prdx-6 Y38C1AA.11 -0.036453420512002
AGIWUR8871;AGIWUR23470;AGIWUR26757
CB4856 CT IV 225439 231773 WBGene00019821 R02D3.3 R02D3.3 -0.110289144003556 AGIWUR3185
CB4856 CT IV 232473 237450 WBGene00019822 R02D3.4 R02D3.4 0.00794569596117967 AGIWUR39185
CB4856 CT IV 237591 240307 WBGene00019823 fnta-1 R02D3.5 -0.136593144138968
AGIWUR5534;AGIWUR12653;AGIWUR22990;AGIWUR24412;AGIWUR29214;AGIWUR29389
CB4856 CT IV 240451 244129 WBGene00019820 cogc-8 R02D3.2 -0.119492847856844 AGIWUR1137
CB4856 CT IV 245114 251925 WBGene00019819 R02D3.1 R02D3.1 -0.541419873216986
AGIWUR3486;AGIWUR11047;AGIWUR27639;AGIWUR30392;AGIWUR39481
CB4856 CT IV 251970 252554 WBGene00001728 grl-19 R02D3.6 0.156994698369447
AGIWUR9545;AGIWUR9685;AGIWUR13103;AGIWUR30596;AGIWUR32729;AGIWUR41213
CB4856 CT IV 253571 255429 WBGene00019824 R02D3.7 R02D3.7 -0.217328022723141 AGIWUR39002
CB4856 CT IV 255966 257199 WBGene00019825 R02D3.8 R02D3.8 0.115314803775263 AGIWUR22427
CB4856 CT IV 257384 259204 WBGene00001071 dpy-9 T21D12.2 -0.3349562341335
AGIWUR18790;AGIWUR27258
CB4856 CT IV 261886 264232 WBGene00003932 pat-6 T21D12.4 -0.171683732228338
AGIWUR43224
CB4856 CT IV 266448 268322 WBGene00020651 T21D12.12 T21D12.12 -0.108750722793811
AGIWUR14769;AGIWUR43154;AGIWUR43734
CB4856 CT IV 269407 272099 WBGene00020650 T21D12.11 T21D12.11 -0.0191929030113274
AGIWUR37598
CB4856 CT IV 272315 273263 WBGene00005487 srh-282 T21D12.5 0.194488911438495
AGIWUR7137
CB4856 CT IV 274570 279940 WBGene00020649 sma-10 T21D12.9 0.583088828814171

AGIWUR15332;AGIWUR23401;AGIWUR28389;AGIWUR36460
CB4856 CT IV 283647 287212 WBGene00206487 best-19 T21D12.15 -0.165398565148243
AGIWUR28322
CB4856 CT IV 287305 288127 WBGene00045177 T21D12.14 T21D12.14 1.32583169550576
AGIWUR14474
CB4856 CT IV 288803 291456 WBGene00020648 T21D12.7 T21D12.7 1.03250920622477
AGIWUR8323;AGIWUR24804
CB4856 CT IV 292429 298280 WBGene00019298 K02D7.1 K02D7.1 0.510319085431737 AGIWUR22001
CB4856 CT IV 307505 311232 WBGene00019300 swt-1 K02D7.5 1.1037803465868 AGIWUR44079
CB4856 CT IV 311512 313697 WBGene00019299 snai-1 K02D7.2 0.412907870967182 AGIWUR5231
CB4856 CT IV 315056 320605 WBGene00001099 dsc-4 K02D7.4 -0.425005105301635 AGIWUR35592
CB4856 CT IV 321571 323022 WBGene00001735 grl-26 K02D7.6 0.0674818857581583 AGIWUR36277
CB4856 CT IV 325063 330631 WBGene00017578 drl-1 F18F11.5 -0.152229947009751
AGIWUR15146
CB4856 CT IV 330823 334385 WBGene00017577 F18F11.4 F18F11.4 0.0542140701918488
AGIWUR18816;AGIWUR18855;AGIWUR32242;AGIWUR34618
CB4856 CT IV 334809 337178 WBGene00017574 F18F11.1 F18F11.1 0.0165639504008475
AGIWUR39978
CB4856 CT IV 339629 358976 WBGene00017576 cdh-8 F18F11.3 0.104407693154737
AGIWUR3867;AGIWUR7444;AGIWUR27378
CB4856 CT IV 359204 376062 WBGene00022048 fln-1 Y66H1B.2 0.188186017863162
AGIWUR6229;AGIWUR12057;AGIWUR34969
CB4856 CT IV 379898 383185 WBGene00004981 spl-1 Y66H1B.4 -0.344341589680877
AGIWUR14485
CB4856 CT IV 385894 386607 WBGene00004493 rps-24 T07A9.11 -0.0219606592073092
AGIWUR18988
CB4856 CT IV 386699 387949 WBGene00044789 T07A9.14 T07A9.14 -0.107868042502114
AGIWUR18205
CB4856 CT IV 388084 388733 WBGene00020300 tag-261 T07A9.13 -0.315347351436563
AGIWUR1038
CB4856 CT IV 388686 390468 WBGene00001338 ears-2 T07A9.2 -0.135255847965947 AGIWUR4672
CB4856 CT IV 390512 393158 WBGene00020298 T07A9.10 T07A9.10 -0.161394830185849
AGIWUR8392;AGIWUR29528;AGIWUR35867
CB4856 CT IV 393156 393546 WBGene00045249 T07A9.15 T07A9.15 -0.147353245861751
AGIWUR8754;AGIWUR11842;AGIWUR20015;AGIWUR22125;AGIWUR24642;AGIWUR30586;AGIWUR31937;AGIWUR33298;AGIWUR34076
CB4856 CT IV 393880 396554 WBGene00020297 nog-1 T07A9.9 0.257210179354955
AGIWUR8931;AGIWUR9531;AGIWUR16928;AGIWUR20629;AGIWUR28111;AGIWUR30284;AGIWUR32858;AGIWUR34292;AGIWUR35408;A
GIWUR39296;AGIWUR40218;AGIWUR41675;AGIWUR44447
CB4856 CT IV 396545 399095 WBGene00020296 T07A9.8 T07A9.8 0.142047669625082 AGIWUR13100

CB4856	CT	IV	401695	405346	WBGene00001666	gpa-4	T07A9.7	0.250943369988545	AGIWUR11240;AGIWUR23437;AGIWUR40058
CB4856	CT	IV	405621	410033	WBGene00002187	kgb-1	T07A9.3	0.0541704718352136	AGIWUR7146
CB4856	CT	IV	410831	415615	WBGene00020299	T07A9.12	T07A9.12	0.132801466552375	AGIWUR10654;AGIWUR20490;AGIWUR28756;AGIWUR37082
CB4856	CT	IV	415635	419958	WBGene00001332	eri-1	T07A9.5	-0.250012401437491	AGIWUR3022;AGIWUR3334;AGIWUR17888;AGIWUR20131;AGIWUR41811
CB4856	CT	IV	419938	425181	WBGene00000913	daf-18	T07A9.6	0.0128481087913892	AGIWUR2814;AGIWUR14949;AGIWUR15080;AGIWUR23347;AGIWUR28679;AGIWUR32747;AGIWUR39915
CB4856	CT	IV	425972	427204	WBGene00022046	Y66H1A.4	Y66H1A.4	0.00880257980811727	AGIWUR14850
CB4856	CT	IV	428070	429133	WBGene00022047	Y66H1A.5	Y66H1A.5	0.213317511020602	AGIWUR29648
CB4856	CT	IV	429214	440554	WBGene00002041	hum-8	Y66H1A.6	-0.187621288048733	AGIWUR34330
CB4856	CT	IV	443985	444783	WBGene00022045	mrpl-55	Y66H1A.3	0.0077886546582917	AGIWUR35396
CB4856	CT	IV	445677	448439	WBGene00022044	dpm-1	Y66H1A.2	-0.118849258006165	AGIWUR39955
CB4856	CT	IV	455726	457099	WBGene00019772	M04G7.1	M04G7.1	0.159081750863209	AGIWUR737
CB4856	CT	IV	458032	461029	WBGene00019773	M04G7.2	M04G7.2	-0.129653898341526	AGIWUR13061
CB4856	CT	IV	461292	481167	WBGene00019774	M04G7.3	M04G7.3	0.0473700833093496	AGIWUR16593
CB4856	CT	IV	505069	506600	WBGene00021011	W03G1.5	W03G1.5	0.70459871615348	AGIWUR7800
CB4856	CT	IV	506737	519457	WBGene00021012	pig-1	W03G1.6	-0.0437280884573088	AGIWUR1369;AGIWUR2867;AGIWUR7710;AGIWUR12794;AGIWUR15026;AGIWUR38661;AGIWUR43168
CB4856	CT	IV	520214	523809	WBGene00000213	asm-3	W03G1.7	-0.132061666489413	AGIWUR17779;AGIWUR43469
CB4856	CT	IV	524652	526479	WBGene00021010	W03G1.2	W03G1.2	-0.159956070352816	AGIWUR7043
CB4856	CT	IV	527379	528583	WBGene00021013	W03G1.8	W03G1.8	0.101443703648462	AGIWUR8211
CB4856	CT	IV	532135	535703	WBGene00001625	glt-7	W03G1.1	-0.192024476669046	AGIWUR40008
CB4856	CT	IV	543231	544086	WBGene00017276	F09C11.1	F09C11.1	0.104405896669178	AGIWUR1852
CB4856	CT	IV	548302	553231	WBGene00018924	F56A11.4	F56A11.4	0.104196298605038	AGIWUR5465
CB4856	CT	IV	559234	568076	WBGene00001165	efn-4	F56A11.3	0.186832109975992	AGIWUR31786
CB4856	CT	IV	568372	570875	WBGene00018925	F56A11.5	F56A11.5	-0.118214461407769	AGIWUR35066
CB4856	CT	IV	572723	583577	WBGene00001579	gex-2	F56A11.1	0.00670208554112506	

AGIWUR20233
CB4856	CT	IV	583813	587829	WBGene00189987	C18H7.12	C18H7.12	0.034999078107985
AGIWUR4609
CB4856	CT	IV	589030	592485	WBGene00015996	C18H7.6	C18H7.6	0.301593883797246	AGIWUR16736
CB4856	CT	IV	593329	595233	WBGene00015994	C18H7.4	C18H7.4	-0.179484920010301	AGIWUR16422
CB4856	CT	IV	595722	596419	WBGene00015997	C18H7.7	C18H7.7	0.107073737683386
AGIWUR12555;AGIWUR23432;AGIWUR33157;AGIWUR40236
CB4856	CT	IV	596864	598771	WBGene00044457	C18H7.11	C18H7.11	-0.552774524457508
AGIWUR26978
CB4856	CT	IV	599449	601695	WBGene00015998	srt-59	C18H7.8	-0.00330851788664965	AGIWUR38612
CB4856	CT	IV	602991	603643	WBGene00015999	prmt-4	C18H7.9	0.0963472986065979	AGIWUR21044
CB4856	CT	IV	604444	605876	WBGene00000676	col-102	C18H7.3	-0.0253546239837528	AGIWUR16403
CB4856	CT	IV	606355	614021	WBGene00002140	inx-18	C18H7.2	-0.238285925529973	AGIWUR28351
CB4856	CT	IV	615544	620925	WBGene00015993	C18H7.1	C18H7.1	2.79688980696768	AGIWUR4760
CB4856	CT	IV	627537	633954	WBGene00015497	nhr-76	C05G6.1	-0.211030993524879
AGIWUR2051;AGIWUR7810
CB4856	CT	IV	649665	651994	WBGene00019665	K11H12.9	K11H12.9	-0.102390168786003
AGIWUR23645
CB4856	CT	IV	652130	653421	WBGene00019658	K11H12.1	K11H12.1	-0.110226308370361
AGIWUR13212
CB4856	CT	IV	653436	654576	WBGene00004427	rpl-15	K11H12.2	-0.0115469102137746
AGIWUR18481;AGIWUR24381
CB4856	CT	IV	655353	658604	WBGene00019664	K11H12.8	K11H12.8	0.0975118420151215
AGIWUR33789;AGIWUR35461
CB4856	CT	IV	665525	666917	WBGene00019663	K11H12.7	K11H12.7	-0.177858916715092
AGIWUR16784;AGIWUR20600;AGIWUR28935;AGIWUR34976;AGIWUR35464
CB4856	CT	IV	667291	668556	WBGene00019662	K11H12.6	K11H12.6	0.138139020377153
AGIWUR21661
CB4856	CT	IV	668962	670555	WBGene00044723	K11H12.11	K11H12.11	-0.0953540059933089
AGIWUR4530;AGIWUR41020
CB4856	CT	IV	674036	675883	WBGene00019660	K11H12.4	K11H12.4	-0.519624951991041
AGIWUR12851
CB4856	CT	IV	676075	676687	WBGene00044458	K11H12.10	K11H12.10	-0.283232873544895
AGIWUR22675
CB4856	CT	IV	677616	679171	WBGene00019661	K11H12.5	K11H12.5	0.178599656881797
AGIWUR26759
CB4856	CT	IV	684497	688336	WBGene00018256	cutl-28	F41A4.1	1.1055866124634	AGIWUR7323
CB4856	CT	IV	698761	707719	WBGene00021950	cutl-26	Y55F3C.7	-0.00914352784290173
AGIWUR3838

CB4856	CT	IV	717925	719451	WBGene00044460	Y55F3C.10	Y55F3C.10	0.0938579039811973	AGIWUR29659
CB4856	CT	IV	719766	727481	WBGene00044459	Y55F3C.9	Y55F3C.9	-0.0081395283350032	AGIWUR3147;AGIWUR17197
CB4856	CT	IV	728500	731037	WBGene00021951	srt-24	Y55F3C.8	0.107921328890411	AGIWUR31555;AGIWUR35044
CB4856	CT	IV	736675	753316	WBGene00021948	kvs-5	Y55F3C.3	-0.267026442539638	AGIWUR4683;AGIWUR4981;AGIWUR12075;AGIWUR25624
CB4856	CT	IV	756721	759084	WBGene00021947	srt-23	Y55F3C.2	-0.33752613168835	AGIWUR19631;AGIWUR28671
CB4856	CT	IV	760610	762342	WBGene00001788	gst-40	F56B3.10	-0.212664813734182	AGIWUR6114
CB4856	CT	IV	762416	763680	WBGene00000677	col-103	F56B3.1	-0.0407195252068622	AGIWUR8934;AGIWUR12460
CB4856	CT	IV	764078	768584	WBGene00018928	F56B3.2	F56B3.2	0.163843690949562	AGIWUR34465
CB4856	CT	IV	773228	776655	WBGene00018932	mrpl-2	F56B3.8	-0.152637605352364	AGIWUR13207
CB4856	CT	IV	778784	784182	WBGene00018931	ugt-52	F56B3.7	0.650679528725682	AGIWUR43381
CB4856	CT	IV	786234	794326	WBGene00004824	F56B3.4	F56B3.4	-0.196105772420411	AGIWUR4977
CB4856	CT	IV	794632	795689	WBGene00018935	skr-18	F56B3.12	-0.149590707403323	AGIWUR20815
CB4856	CT	IV	795755	796567	WBGene00018934	F56B3.11	F56B3.11	-0.0374039112122801	AGIWUR10110;AGIWUR20027;AGIWUR22185;AGIWUR26451;AGIWUR30559;AGIWUR37301;AGIWUR43312
CB4856	CT	IV	797023	799922	WBGene00001154	ech-5	F56B3.5	-0.430328052672998	AGIWUR22304
CB4856	CT	IV	805102	806236	WBGene00185088	Y55F3BL.4	Y55F3BL.4	-0.0436807701502939	AGIWUR21165
CB4856	CT	IV	807439	824954	WBGene00021936	zipt-20	Y55F3BL.2	0.0510222981385802	AGIWUR5347;AGIWUR13156;AGIWUR14357
CB4856	CT	IV	830359	831896	WBGene00021942	madf-1	Y55F3BR.5	-0.0405493170104965	AGIWUR35663
CB4856	CT	IV	832362	833329	WBGene00044742	Y55F3BR.10	Y55F3BR.10	-0.257673006031275	AGIWUR13482;AGIWUR20780;AGIWUR28506;AGIWUR42338
CB4856	CT	IV	835033	842126	WBGene00021943	Y55F3BR.6	Y55F3BR.6	-0.129569248547054	AGIWUR15121;AGIWUR42889
CB4856	CT	IV	845881	848850	WBGene00021941	lgc-33	Y55F3BR.4	0.190535422699011	AGIWUR12929
CB4856	CT	IV	849269	854780	WBGene00021945	lem-4	Y55F3BR.8	-0.119872841018863	AGIWUR6239
CB4856	CT	IV	857327	874724	WBGene00021939	Y55F3BR.2	Y55F3BR.2	0.0164504711565261	AGIWUR2219;AGIWUR14690;AGIWUR43144

CB4856	CT	IV	874791	881910	WBGene00021938	Y55F3BR.1	Y55F3BR.1	-0.169690824807708	AGIWUR8321
CB4856	CT	IV	885346	890200	WBGene00007007	mak-2	C44C8.6	0.373233489953294	AGIWUR5736;AGIWUR10731;AGIWUR10982;AGIWUR40813
CB4856	CT	IV	919990	920808	WBGene00019070	F58H7.5	F58H7.5	0.0834919157226421	AGIWUR34903
CB4856	CT	IV	924936	939667	WBGene00019069	lgc-30	F58H7.3	0.207901678061712	AGIWUR10043
CB4856	CT	IV	940017	945653	WBGene00019068	faah-3	F58H7.2	0.117367658404866	AGIWUR10182
CB4856	CT	IV	959452	984392	WBGene00004047	plx-1	Y55F3AL.1	0.320455126212297	AGIWUR24475
CB4856	CT	IV	984405	990521	WBGene00043705	egrh-2	Y55F3AM.7	0.0643050666991325	AGIWUR6981
CB4856	CT	IV	992240	998348	WBGene00021924	Y55F3AM.6	Y55F3AM.6	-0.426628747819081	AGIWUR12792;AGIWUR15312;AGIWUR16365;AGIWUR19661
CB4856	CT	IV	999214	1003639	WBGene00021923	Y55F3AM.5	Y55F3AM.5	-0.120437799558569	AGIWUR1547;AGIWUR20689;AGIWUR44614
CB4856	CT	IV	1003600	1005864	WBGene00021925	immp-2	Y55F3AM.8	-0.0668611080138763	AGIWUR9588
CB4856	CT	IV	1006083	1009734	WBGene00021926	Y55F3AM.9	Y55F3AM.9	0.160174784951064	AGIWUR35970
CB4856	CT	IV	1010054	1012502	WBGene00021922	atg-3	Y55F3AM.4	0.0907450042742717	AGIWUR44533
CB4856	CT	IV	1012624	1019558	WBGene00021921	Y55F3AM.3	Y55F3AM.3	-0.0138511094872769	AGIWUR19334;AGIWUR19775;AGIWUR20515;AGIWUR20761;AGIWUR24236;AGIWUR37138;AGIWUR42157
CB4856	CT	IV	1027787	1030024	WBGene00021927	abhd-14	Y55F3AM.10	0.402640512607029	AGIWUR26712
CB4856	CT	IV	1038671	1041008	WBGene00021928	Y55F3AM.11	Y55F3AM.11	-0.0922512427736137	AGIWUR19979
CB4856	CT	IV	1045983	1049506	WBGene00005903	srx-12	Y55F3AM.2	-0.0463831022194725	AGIWUR42547
CB4856	CT	IV	1052524	1054794	WBGene00021929	dcap-1	Y55F3AM.12	0.21559209084355	AGIWUR13844;AGIWUR14536;AGIWUR15020;AGIWUR20920;AGIWUR35100;AGIWUR36637
CB4856	CT	IV	1056890	1057620	WBGene00021920	mrps-25	Y55F3AM.1	-0.218707964542158	AGIWUR31309
CB4856	CT	IV	1058695	1067525	WBGene00021931	Y55F3AM.14	Y55F3AM.14	0.0103229951654429	AGIWUR14109
CB4856	CT	IV	1077463	1087013	WBGene00000816	csn-4	Y55F3AM.15	-0.290814699937084	AGIWUR14506;AGIWUR35063;AGIWUR36445;AGIWUR38851
CB4856	CT	IV	1087095	1093920	WBGene00021934	cct-8	Y55F3AR.3	-0.47540883685401	AGIWUR7621

CB4856	CT	IV	1094545	1096723	WBGene00021933	Y55F3AR.2	Y55F3AR.2	-0.341268817398063	AGIWUR7254
CB4856	CT	IV	1097453	1102980	WBGene00021932	Y55F3AR.1	Y55F3AR.1	-0.294044210259422	AGIWUR2526
CB4856	CT	IV	1103541	1104609	WBGene00044462	C44B12.9	C44B12.9	-0.318555749617149	AGIWUR13870;AGIWUR22722;AGIWUR29440;AGIWUR34768;AGIWUR38469
CB4856	CT	IV	1107396	1110856	WBGene00003893	ost-1	C44B12.2	-0.0558079074669768	AGIWUR35036;AGIWUR44237
CB4856	CT	IV	1111462	1114820	WBGene00005905	srx-14	C44B12.8	0.0549468276846117	AGIWUR3271
CB4856	CT	IV	1114969	1118666	WBGene00005904	srx-13	C44B12.4	0.0988489065980946	AGIWUR26071
CB4856	CT	IV	1121457	1125107	WBGene00016638	perm-4	C44B12.5	0.14137749212184	AGIWUR36087
CB4856	CT	IV	1126790	1128848	WBGene00016636	perm-2	C44B12.1	0.164770053495538	AGIWUR12455;AGIWUR35647
CB4856	CT	IV	1128964	1136947	WBGene00016639	C44B12.6	C44B12.6	-0.13041610395419	AGIWUR17771
CB4856	CT	IV	1145681	1158028	WBGene00016797	C50A2.3	C50A2.3	-0.361262322752662	AGIWUR22624
CB4856	CT	IV	1159637	1160484	WBGene00016798	ets-8	C50A2.4	0.0747785246702497	AGIWUR6703
CB4856	CT	IV	1170672	1177776	WBGene00016796	cec-2	C50A2.2	-0.216659137264005	AGIWUR29859
CB4856	CT	IV	1178022	1182837	WBGene00022426	Y104H12D.2	Y104H12D.2	0.0559414543938779	AGIWUR25304
CB4856	CT	IV	1182592	1186604	WBGene00022428	Y104H12D.4	Y104H12D.4	-0.179433567532399	AGIWUR14593
CB4856	CT	IV	1187799	1192977	WBGene00007020	mdt-20	Y104H12D.1	0.329327535213652	AGIWUR13101
CB4856	CT	IV	1193065	1201846	WBGene00022427	Y104H12D.3	Y104H12D.3	0.0505401487137078	AGIWUR34307
CB4856	CT	IV	1202651	1204970	WBGene00001104	dsl-2	W09G12.1	-0.0249654850903482	AGIWUR13919
CB4856	CT	IV	1205868	1206377	WBGene00023163	W09G12.3	W09G12.3	-0.00868312826132067	AGIWUR8237;AGIWUR25731;AGIWUR35019;AGIWUR42511
CB4856	CT	IV	1207255	1208102	WBGene00001103	dsl-1	W09G12.4	0.532830369482651	AGIWUR14118
CB4856	CT	IV	1216590	1218527	WBGene00021123	W09G12.9	W09G12.9	0.0445213667819768	AGIWUR34992
CB4856	CT	IV	1219333	1221287	WBGene00044463	W09G12.10	W09G12.10	-0.272354620070089	AGIWUR6676;AGIWUR24423;AGIWUR28632;AGIWUR31254;AGIWUR40883

CB4856 CT IV 1226182 1227668 WBGene00021122 W09G12.8 W09G12.8 -0.400622124037932 AGIWUR26633
CB4856 CT IV 1229080 1230789 WBGene00021121 W09G12.7 W09G12.7 1.93108859769767 AGIWUR44787
CB4856 CT IV 1231897 1232812 WBGene00021120 W09G12.6 W09G12.6 -0.0669453581166035 AGIWUR15970
CB4856 CT IV 1232872 1239852 WBGene00009521 F38A1.8 F38A1.8 -0.392558104478304 AGIWUR1399;AGIWUR42823
CB4856 CT IV 1241332 1243402 WBGene00044908 F38A1.15 F38A1.15 0.368317881084019 AGIWUR6368
CB4856 CT IV 1244247 1245344 WBGene00009522 F38A1.9 F38A1.9 -0.114003974148899 AGIWUR39998
CB4856 CT IV 1245399 1246886 WBGene00009523 clec-165 F38A1.10 0.166518790948215 AGIWUR18756
CB4856 CT IV 1247386 1249025 WBGene00009524 F38A1.11 F38A1.11 -0.358755246714157 AGIWUR15217
CB4856 CT IV 1249018 1251366 WBGene00009518 clec-166 F38A1.5 0.330430171885325 AGIWUR3404
CB4856 CT IV 1251861 1252470 WBGene00009519 F38A1.6 F38A1.6 0.00921096503557628 AGIWUR39698
CB4856 CT IV 1253137 1255732 WBGene00009517 clec-167 F38A1.4 -0.222669381179735 AGIWUR32291
CB4856 CT IV 1256956 1259344 WBGene00009520 clec-168 F38A1.7 -0.157306541098475 AGIWUR22643
CB4856 CT IV 1261272 1264622 WBGene00009526 clec-169 F38A1.14 -0.349412226651511 AGIWUR1769
CB4856 CT IV 1265228 1265848 WBGene00009525 F38A1.13 F38A1.13 -0.034488590744111 AGIWUR34868
CB4856 CT IV 1266866 1274425 WBGene00009515 clec-170 F38A1.1 -0.144906749283287 AGIWUR120;AGIWUR3055;AGIWUR12336;AGIWUR18316;AGIWUR25656;AGIWUR28250;AGIWUR29831;AGIWUR31041;AGIWUR35445;AGIWUR35831
CB4856 CT IV 1275865 1277091 WBGene00007476 C09E9.1 C09E9.1 -0.109585518287669 AGIWUR1226;AGIWUR23153;AGIWUR24526;AGIWUR25781;AGIWUR28437;AGIWUR38895
CB4856 CT IV 1289627 1296897 WBGene00022425 plst-1 Y104H12BR.1 -0.321218626668928 AGIWUR6341;AGIWUR8186;AGIWUR19456;AGIWUR20224;AGIWUR30101;AGIWUR30260;AGIWUR33915;AGIWUR35453;AGIWUR36595;AGIWUR41949
CB4856 CT IV 1297222 1309652 WBGene00018776 F53H1.1 F53H1.1 0.0486888635995371 AGIWUR7687
CB4856 CT IV 1310166 1328241 WBGene00018778 F53H1.4 F53H1.4 -0.236068675451609 AGIWUR7608;AGIWUR20200;AGIWUR20748;AGIWUR22712
CB4856 CT IV 1329162 1339376 WBGene00018777 F53H1.3 F53H1.3 0.139520400725889 AGIWUR16858
CB4856 CT IV 1347316 1352977 WBGene00004338 rfc-2 F58F6.4 -0.111572227037204 AGIWUR19510

CB4856	CT	IV	1353636	1355305	WBGene00000678	col-104	F58F6.1	-0.0541034512821135	AGIWUR9061
CB4856	CT	IV	1357816	1359225	WBGene00000679	col-105	F58F6.2	-0.136582975517893	AGIWUR12554
CB4856	CT	IV	1375369	1376563	WBGene00019052	F58F6.3	F58F6.3	0.0253195463705227	AGIWUR5879
CB4856	CT	IV	1386045	1399538	WBGene00022423	nhr-41	Y104H12A.1	0.131605596881236	AGIWUR7746;AGIWUR10431;AGIWUR12457;AGIWUR30123
CB4856	CT	IV	1406542	1408131	WBGene00022314	Y77E11A.14	Y77E11A.14	0.942041992936394	AGIWUR40359
CB4856	CT	IV	1408582	1410041	WBGene00022309	rpc-11	Y77E11A.6	-0.0749982416373101	AGIWUR12229
CB4856	CT	IV	1410786	1419847	WBGene00022310	Y77E11A.7	Y77E11A.7	-0.151607415102919	AGIWUR268;AGIWUR9813;AGIWUR11788;AGIWUR15813;AGIWUR17418;AGIWUR22112;AGIWUR22493;AGIWUR34355
CB4856	CT	IV	1420929	1421770	WBGene00002180	jtr-1	Y77E11A.4	-0.142989391654108	AGIWUR20279
CB4856	CT	IV	1425482	1426887	WBGene00022312	clec-171	Y77E11A.9	0.0815395983698552	AGIWUR15284;AGIWUR26848;AGIWUR35309
CB4856	CT	IV	1429041	1430367	WBGene00235267	Y77E11A.23	Y77E11A.23	0.265430564781364	AGIWUR20360
CB4856	CT	IV	1435465	1446182	WBGene00000547	clp-7	Y77E11A.11	-0.0163524328059808	AGIWUR14913
CB4856	CT	IV	1447594	1448316	WBGene00022308	Y77E11A.3	Y77E11A.3	0.221523074392673	AGIWUR17454
CB4856	CT	IV	1457075	1459521	WBGene00022307	Y77E11A.2	Y77E11A.2	0.502296803096371	AGIWUR20302
CB4856	CT	IV	1459866	1460786	WBGene00000680	col-106	Y77E11A.15	0.0340174980198711	AGIWUR30247
CB4856	CT	IV	1461899	1475109	WBGene00022313	Y77E11A.12	Y77E11A.12	0.0588800455587712	AGIWUR10496
CB4856	CT	IV	1485286	1489762	WBGene00044464	Y77E11A.16	Y77E11A.16	0.0699834253208085	AGIWUR7338
CB4856	CT	IV	1490653	1496022	WBGene00003806	npp-20	Y77E11A.13	-0.0944553950447951	AGIWUR4990;AGIWUR24613;AGIWUR40679
CB4856	CT	IV	1497630	1509965	WBGene00022306	hxk-3	Y77E11A.1	0.182382707859507	AGIWUR29596
CB4856	CT	IV	1510546	1515251	WBGene00019368	K03H6.2	K03H6.2	-0.124311098345315	AGIWUR12991
CB4856	CT	IV	1515510	1516627	WBGene00044719	clec-172	K03H6.7	1.26587479967747	AGIWUR18366
CB4856	CT	IV	1517969	1521324	WBGene00019369	K03H6.4	K03H6.4	0.212463251356411	AGIWUR24843;AGIWUR40141
CB4856	CT	IV	1523718	1526599	WBGene00019367	K03H6.1	K03H6.1	-0.0347067100843299	AGIWUR37618

CB4856	CT	IV	1527221	1536398	WBGene00019370	K03H6.5	K03H6.5	0.0159749483930796	AGIWUR25528;AGIWUR39670
CB4856	CT	IV	1545296	1566192	WBGene00016427	C34H4.5	C34H4.5	0.0969455369539123	AGIWUR13;AGIWUR480;AGIWUR7571;AGIWUR10154;AGIWUR15441;AGIWUR21312;AGIWUR36966;AGIWUR38213;AGIWUR44132
CB4856	CT	IV	1567921	1595824	WBGene00003794	npp-8	Y41D4B.19	0.185026927813849	AGIWUR451;AGIWUR7749;AGIWUR13128;AGIWUR14605;AGIWUR16223;AGIWUR17183;AGIWUR19609;AGIWUR27086;AGIWUR33432;AGIWUR35153;AGIWUR37403;AGIWUR38651;AGIWUR42141
CB4856	CT	IV	1597316	1601620	WBGene00000416	ced-2	Y41D4B.13	0.0486881536919022	AGIWUR16139
CB4856	CT	IV	1602985	1605609	WBGene00021515	set-23	Y41D4B.12	0.161610322199417	AGIWUR12103
CB4856	CT	IV	1605712	1608901	WBGene00021514	Y41D4B.11	Y41D4B.11	-0.10410408994777	AGIWUR41173
CB4856	CT	IV	1610236	1612915	WBGene00001105	dsl-3	Y41D4B.10	0.913618757104445	AGIWUR20853
CB4856	CT	IV	1613897	1616766	WBGene00003712	nhr-122	Y41D4B.9	0.460725198946216	AGIWUR37999
CB4856	CT	IV	1626973	1630756	WBGene00045255	nhr-146	Y41D4B.27	0.0650637130916163	AGIWUR21898;AGIWUR30984;AGIWUR32578;AGIWUR38940;AGIWUR43528
CB4856	CT	IV	1631951	1633257	WBGene00021525	Y41D4B.26	Y41D4B.26	1.00912700649036	AGIWUR36375
CB4856	CT	IV	1633772	1637367	WBGene00021513	Y41D4B.6	Y41D4B.6	1.56287235734671	AGIWUR39537
CB4856	CT	IV	1659691	1660011	WBGene00004497	rps-28	Y41D4B.5	0.160122824256032	AGIWUR42056
CB4856	CT	IV	1660112	1669707	WBGene00021512	Y41D4B.4	Y41D4B.4	-0.159734579843656	AGIWUR30269
CB4856	CT	IV	1672706	1676209	WBGene00021511	Y41D4B.1	Y41D4B.1	-0.14137246591388	AGIWUR2885
CB4856	CT	IV	1676720	1682159	WBGene00021524	Y41D4B.24	Y41D4B.24	0.133297320043428	AGIWUR572;AGIWUR2148;AGIWUR10477;AGIWUR11515;AGIWUR12627;AGIWUR16482;AGIWUR19697;AGIWUR25338;AGIWUR29364;AGIWUR30172;AGIWUR38879
CB4856	CT	IV	1699899	1702820	WBGene00044618	bus-2	K08D12.5	-0.0685618617666099	AGIWUR6907
CB4856	CT	IV	1707296	1708691	WBGene00019538	K08D12.4	K08D12.4	-0.0223407894075258	AGIWUR41649
CB4856	CT	IV	1708804	1709745	WBGene00003947	pbs-1	K08D12.1	-0.26516173753731	AGIWUR526
CB4856	CT	IV	1710265	1711127	WBGene00019537	K08D12.3	K08D12.3	-0.0150126422607997	

AGIWUR1281;AGIWUR4072;AGIWUR7432;AGIWUR7623;AGIWUR10035;AGIWUR15285;AGIWUR19577;AGIWUR24436;AGIWUR25955;AGIWUR29099;AGIWUR32333;AGIWUR32915;AGIWUR33849;AGIWUR36200;AGIWUR42283
CB4856 CT IV 1711279 1714179 WBGene00019536 rpi-2 K08D12.2 0.065476160089716 AGIWUR9019
CB4856 CT IV 1720790 1723067 WBGene00019540 K08D12.6 K08D12.6 -0.0506290927766253 AGIWUR1415;AGIWUR2340;AGIWUR41859;AGIWUR42751
CB4856 CT IV 1723199 1724143 WBGene00044293 K08D12.7 K08D12.7 -0.08343022104558 AGIWUR27413
CB4856 CT IV 1727438 1737884 WBGene00021507 Y41D4A.5 Y41D4A.5 0.0744327550512285 AGIWUR8940
CB4856 CT IV 1740921 1745587 WBGene00021508 Y41D4A.6 Y41D4A.6 0.276315793851285 AGIWUR11580
CB4856 CT IV 1745875 1751633 WBGene00021506 Y41D4A.4 Y41D4A.4 0.292875871043982 AGIWUR9031;AGIWUR10979;AGIWUR20429
CB4856 CT IV 1752270 1761647 WBGene00021509 Y41D4A.7 Y41D4A.7 0.221709678464284 AGIWUR14956
CB4856 CT IV 1768621 1769667 WBGene00000682 col-108 Y41D4A.2 -0.32240376376042 AGIWUR23691
CB4856 CT IV 1772144 1774695 WBGene00021504 Y41D4A.1 Y41D4A.1 -0.161546596659509 AGIWUR38771
CB4856 CT IV 1775277 1781562 WBGene00021510 frpr-19 Y41D4A.8 0.0895309257124941 AGIWUR8492
CB4856 CT IV 1802026 1803982 WBGene00021410 Y38C1BA.1 Y38C1BA.1 0.355849871196472 AGIWUR12252
CB4856 CT IV 1805037 1815099 WBGene00004913 snn-1 Y38C1BA.2 0.148403192524431 AGIWUR9737
CB4856 CT IV 1817311 1820253 WBGene00000683 col-109 Y38C1BA.3 -0.310226878352427 AGIWUR41439
CB4856 CT IV 1824481 1834029 WBGene00000553 cmk-1 K07A9.2 0.174466580570758 AGIWUR6980;AGIWUR44640
CB4856 CT IV 1834628 1847336 WBGene00019462 K07A9.3 K07A9.3 -0.182306978057156 AGIWUR8587;AGIWUR9835;AGIWUR41314
CB4856 CT IV 1847968 1880587 WBGene00001173 egl-4 F55A8.2 0.102155779749111 AGIWUR6005;AGIWUR7284;AGIWUR8769;AGIWUR10842;AGIWUR11592;AGIWUR11699;AGIWUR19320;AGIWUR20152;AGIWUR29949;AGIWUR30504;AGIWUR34266;AGIWUR36671;AGIWUR37765;AGIWUR44393;AGIWUR44568
CB4856 CT IV 1902323 1917841 WBGene00001186 egl-18 F55A8.1 0.375611706221323 AGIWUR5983;AGIWUR11851;AGIWUR22220;AGIWUR24154;AGIWUR26660;AGIWUR35388
CB4856 CT IV 1917854 1921644 WBGene00001253 elt-6 F52C12.5 0.7879875429611 AGIWUR35073
CB4856 CT IV 1923939 1948040 WBGene00018681 denn-4 F52C12.4 -0.391121439409249

AGIWUR27710
CB4856 CT IV 1948191 1953322 WBGene00018680 tsen-54 F52C12.3 -0.107399348853264
AGIWUR15719
CB4856 CT IV 1953579 1956704 WBGene00018679 F52C12.2 F52C12.2 -0.135357480418321
AGIWUR30822;AGIWUR41330
CB4856 CT IV 1956711 1959749 WBGene00138717 F52C12.6 F52C12.6 0.0665512132201433
AGIWUR10670;AGIWUR13703;AGIWUR23756;AGIWUR30117;AGIWUR37019
CB4856 CT IV 1959854 1963240 WBGene00018678 F52C12.1 F52C12.1 -0.0167295665825851
AGIWUR20817
CB4856 CT IV 1963191 1964724 WBGene00006738 ulp-3 Y48A5A.2 0.0839791548622806
AGIWUR12318
CB4856 CT IV 1965481 1969650 WBGene00021655 Y48A5A.1 Y48A5A.1 0.147563176802383
AGIWUR7982;AGIWUR29240
CB4856 CT IV 1969737 1970280 WBGene00044466 Y48A5A.3 Y48A5A.3 -0.0183099643599292
AGIWUR18424;AGIWUR25118;AGIWUR36764;AGIWUR37110;AGIWUR39187
CB4856 CT IV 1970734 1972977 WBGene00022298 Y76B12C.4 Y76B12C.4 -0.176365514158965
AGIWUR11644
CB4856 CT IV 1973702 1975677 WBGene00022297 Y76B12C.3 Y76B12C.3 0.13281790416621
AGIWUR39977
CB4856 CT IV 1979988 1980363 WBGene00022302 Y76B12C.8 Y76B12C.8 -0.204423306595491
AGIWUR19264
CB4856 CT IV 1981406 1992966 WBGene00022300 Y76B12C.6 Y76B12C.6 -0.111386993625058
AGIWUR11393;AGIWUR23683;AGIWUR35565
CB4856 CT IV 1993089 2008993 WBGene00022296 xpc-1 Y76B12C.2 -0.0247761424000998
AGIWUR4472
CB4856 CT IV 2009038 2032998 WBGene00022301 cpsf-1 Y76B12C.7 -0.0580469139977637
AGIWUR32546
CB4856 CT IV 2034982 2035986 WBGene00022303 Y76B12C.9 Y76B12C.9 -0.45420071978744
AGIWUR6069
CB4856 CT IV 2037191 2043191 WBGene00022295 cng-2 Y76B12C.1 -0.00611007717301071
AGIWUR30761
CB4856 CT IV 2051500 2057079 WBGene00044467 R05C11.4 R05C11.4 -0.079734532959689
AGIWUR7195
CB4856 CT IV 2057878 2059111 WBGene00019874 R05C11.2 R05C11.2 0.00816931613336092
AGIWUR26095
CB4856 CT IV 2066200 2071741 WBGene00019873 arrd-26 R05C11.1 -0.287640685315425
AGIWUR33154
CB4856 CT IV 2084034 2091931 WBGene00019875 mca-2 R05C11.3 0.0591433478027446
AGIWUR11098;AGIWUR19693;AGIWUR30452;AGIWUR43035

CB4856 CT IV 2091929 2099890 WBGene00044649 C23H5.11 C23H5.11 -0.290494338115134
AGIWUR6993
CB4856 CT IV 2104056 2111605 WBGene00000487 che-6 C23H5.7 0.00341225035473247 AGIWUR31703
CB4856 CT IV 2111894 2113422 WBGene00016027 C23H5.8 C23H5.8 -0.80678914335792
AGIWUR13732;AGIWUR14068;AGIWUR24032;AGIWUR24904;AGIWUR36120;AGIWUR42627
CB4856 CT IV 2114626 2115625 WBGene00016025 xbx-4 C23H5.3 -0.0240710189719779
AGIWUR4935;AGIWUR5289;AGIWUR12000;AGIWUR18510;AGIWUR36867;AGIWUR37752
CB4856 CT IV 2122797 2126038 WBGene00016024 glb-7 C23H5.2 -0.0769416529255089 AGIWUR1915
CB4856 CT IV 2126145 2128852 WBGene00001729 grl-20 C23H5.9 0.127909714607135
AGIWUR3649;AGIWUR24293;AGIWUR25291;AGIWUR31492;AGIWUR35279;AGIWUR40828
CB4856 CT IV 2130965 2133025 WBGene00016023 prmt-6 C23H5.1 -0.643385467634898
AGIWUR5424;AGIWUR17274
CB4856 CT IV 2133481 2134708 WBGene00077523 C23H5.12 C23H5.12 0.0183606915041654
AGIWUR6775
CB4856 CT IV 2136895 2146461 WBGene00016918 test-1 C54E4.2 -0.366254686028306
AGIWUR1237;AGIWUR8185
CB4856 CT IV 2146672 2149297 WBGene00016920 C54E4.5 C54E4.5 -0.240882512775386 AGIWUR24380
CB4856 CT IV 2150650 2159162 WBGene00001557 gcy-37 C54E4.3 0.43340170752376 AGIWUR40786
CB4856 CT IV 2161252 2168626 WBGene00016919 C54E4.4 C54E4.4 0.283541764270128 AGIWUR28881
CB4856 CT IV 2175606 2177579 WBGene00016917 C54E4.1 C54E4.1 0.033407270851324 AGIWUR7652
CB4856 CT IV 2177900 2198871 WBGene00022134 Y71G10AL.1 Y71G10AL.1 -0.231392493492674
AGIWUR6949;AGIWUR10218;AGIWUR16899;AGIWUR23265;AGIWUR37024;AGIWUR38505;AGIWUR43268
CB4856 CT IV 2199041 2209051 WBGene00044650 Y71G10AR.4 Y71G10AR.4 -0.163884519624979
AGIWUR10691;AGIWUR30628;AGIWUR30812
CB4856 CT IV 2222828 2235795 WBGene00020208 T04C4.1 T04C4.1 -0.0900474706227365
AGIWUR5060;AGIWUR9922;AGIWUR18558;AGIWUR18666;AGIWUR22407;AGIWUR24849;AGIWUR26201;AGIWUR34832;AGIWUR35575;A
GIWUR38382
CB4856 CT IV 2237663 2242449 WBGene00019785 M70.3 M70.3 0.0829834239662901 AGIWUR21308
CB4856 CT IV 2243060 2243699 WBGene00019784 M70.2 M70.2 -0.51576166831207 AGIWUR43594
CB4856 CT IV 2245874 2252370 WBGene00019783 M70.1 M70.1 0.00965111066367541 AGIWUR44404
CB4856 CT IV 2253004 2263704 WBGene00019786 M70.4 M70.4 0.499674975541941 AGIWUR8671
CB4856 CT IV 2264309 2275622 WBGene00019787 M70.5 M70.5 -0.188002082477396 AGIWUR41044
CB4856 CT IV 2275864 2300351 WBGene00002990 lin-1 C37F5.1 0.237704546831625
AGIWUR2520;AGIWUR11089;AGIWUR14119;AGIWUR19872;AGIWUR40161;AGIWUR43471
CB4856 CT IV 2304446 2306540 WBGene00021416 mksr-2 Y38F2AL.2 0.207012143542343
AGIWUR19462
CB4856 CT IV 2307819 2314567 WBGene00006920 vha-11 Y38F2AL.3 -0.300947643350366
AGIWUR5573;AGIWUR21184;AGIWUR35616;AGIWUR38366
CB4856 CT IV 2314589 2315990 WBGene00006912 vha-3 Y38F2AL.4 -0.0508565042745559

AGIWUR8240
CB4856 CT IV 2324058 2326739 WBGene00021415 nsy-4 Y38F2AL.1 0.792990087639044
AGIWUR35241
CB4856 CT IV 2327126 2329640 WBGene00021417 nhr-236 Y38F2AL.5 0.224530304915115
AGIWUR33118
CB4856 CT IV 2330193 2331009 WBGene00021418 Y38F2AL.6 Y38F2AL.6 -0.0114693038143496
AGIWUR12369;AGIWUR40782
CB4856 CT IV 2333837 2348912 WBGene00021425 ppgn-1 Y38F2AR.7 -0.247936313438444
AGIWUR27269;AGIWUR34081
CB4856 CT IV 2349271 2382171 WBGene00021423 tftc-1 Y38F2AR.5 -0.288333311315049
AGIWUR2694;AGIWUR40174
CB4856 CT IV 2384013 2388089 WBGene00021421 Y38F2AR.3 Y38F2AR.3
-0.00816441874660434 AGIWUR26768;AGIWUR29181;AGIWUR34184
CB4856 CT IV 2388195 2388675 WBGene00021427 Y38F2AR.9 Y38F2AR.9 -0.148818511380581
AGIWUR23976;AGIWUR42445
CB4856 CT IV 2389445 2390386 WBGene00021420 trap-3 Y38F2AR.2 0.00224583930228306
AGIWUR18279
CB4856 CT IV 2390821 2396263 WBGene00021419 eri-5 Y38F2AR.1 0.035030102841116
AGIWUR30757
CB4856 CT IV 2396257 2399420 WBGene00021428 Y38F2AR.10 Y38F2AR.10 0.160992078584439
AGIWUR17742
CB4856 CT IV 2399515 2412410 WBGene00021430 Y38F2AR.12 Y38F2AR.12 0.0608799789207359
AGIWUR8407;AGIWUR30068
CB4856 CT IV 2432993 2436472 WBGene00016671 C45G7.4 C45G7.4 0.487467685322388 AGIWUR18077
CB4856 CT IV 2436505 2463405 WBGene00000402 cdh-10 C45G7.5 0.017448762243955
AGIWUR32982;AGIWUR37797
CB4856 CT IV 2464456 2465401 WBGene00016670 ilys-3 C45G7.3 3.19803961684859 AGIWUR18202
CB4856 CT IV 2467681 2468810 WBGene00016669 ilys-2 C45G7.2 -0.380165356250973 AGIWUR12281
CB4856 CT IV 2470414 2471449 WBGene00016668 ilys-1 C45G7.1 0.168774776785484 AGIWUR12322
CB4856 CT IV 2476700 2480218 WBGene00001705 grd-16 Y69A2AL.1 0.482256948667658
AGIWUR28070
CB4856 CT IV 2482255 2484218 WBGene00044468 Y69A2AL.2 Y69A2AL.2 0.924170237487498
AGIWUR4846;AGIWUR10333;AGIWUR22619
CB4856 CT IV 2488820 2494135 WBGene00022090 Y69A2AR.19 Y69A2AR.19 0.355059608830418
AGIWUR32158
CB4856 CT IV 2494513 2495174 WBGene00022092 Y69A2AR.21 Y69A2AR.21 -0.20162379303758
AGIWUR3459;AGIWUR6118;AGIWUR16638;AGIWUR29487;AGIWUR29613
CB4856 CT IV 2495735 2497683 WBGene00022089 Y69A2AR.18 Y69A2AR.18 -0.0170835674202223
AGIWUR4576;AGIWUR29217;AGIWUR34804

CB4856	CT	IV	2497801	2530296	WBGene00022087	Y69A2AR.16	Y69A2AR.16	-0.210361644327013	AGIWUR8327;AGIWUR23283;AGIWUR27495;AGIWUR40391
CB4856	CT	IV	2542326	2548719	WBGene00022086	sprr-3	Y69A2AR.15	-0.264841784995987	AGIWUR36441
CB4856	CT	IV	2549926	2551697	WBGene00022094	Y69A2AR.23	Y69A2AR.23	-0.118025745780868	AGIWUR21548
CB4856	CT	IV	2551872	2557106	WBGene00022085	Y69A2AR.14	Y69A2AR.14	0.0619562904749067	AGIWUR24129
CB4856	CT	IV	2563778	2564069	WBGene00022095	Y69A2AR.24	Y69A2AR.24	-0.0209121446743454	AGIWUR38735
CB4856	CT	IV	2565820	2568367	WBGene00022096	Y69A2AR.25	Y69A2AR.25	-0.536339042614648	AGIWUR31094
CB4856	CT	IV	2568614	2572056	WBGene00022083	Y69A2AR.12	Y69A2AR.12	-0.276273324154424	AGIWUR43136
CB4856	CT	IV	2592395	2596786	WBGene00022079	Y69A2AR.8	Y69A2AR.8	-0.381013053711029	AGIWUR43208;AGIWUR44094
CB4856	CT	IV	2597574	2597970	WBGene00235294	Y69A2AR.47	Y69A2AR.47	-0.184986742849793	AGIWUR35460
CB4856	CT	IV	2601141	2607571	WBGene00022078	epg-9	Y69A2AR.7	0.621033761705495	AGIWUR7888;AGIWUR11032;AGIWUR12009;AGIWUR21991;AGIWUR24627;AGIWUR31250;AGIWUR33586;AGIWUR44851
CB4856	CT	IV	2608051	2610200	WBGene00022077	vamp-7	Y69A2AR.6	0.185486087883277	AGIWUR41890
CB4856	CT	IV	2610408	2612435	WBGene00022101	Y69A2AR.32	Y69A2AR.32	0.162474689161956	AGIWUR1713;AGIWUR9524;AGIWUR20374;AGIWUR24017;AGIWUR36001;AGIWUR38697
CB4856	CT	IV	2614200	2617135	WBGene00022076	daao-1	Y69A2AR.5	0.092341023366405	AGIWUR1766;AGIWUR3255;AGIWUR8311;AGIWUR25091;AGIWUR35754;AGIWUR36807
CB4856	CT	IV	2619875	2625438	WBGene00004878	smf-3	Y69A2AR.4	1.41699982630244	AGIWUR7804
CB4856	CT	IV	2626132	2628271	WBGene00003595	ngn-1	Y69A2AR.29	0.577899770322628	AGIWUR28661
CB4856	CT	IV	2636228	2641117	WBGene00003161	mdf-2	Y69A2AR.30	-0.109822537148477	AGIWUR3452;AGIWUR25205;AGIWUR38116;AGIWUR39583
CB4856	CT	IV	2641310	2642113	WBGene00022075	Y69A2AR.3	Y69A2AR.3	-0.218704203238705	AGIWUR24450
CB4856	CT	IV	2642979	2650104	WBGene00004367	ric-8	Y69A2AR.2	-0.096081036149398	AGIWUR22951;AGIWUR44852
CB4856	CT	IV	2650384	2664003	WBGene00022100	Y69A2AR.31	Y69A2AR.31	0.118856937125003	AGIWUR15525
CB4856	CT	IV	2667050	2672202	WBGene00022074	Y69A2AR.1	Y69A2AR.1	0.140211837742051	

AGIWUR16654;AGIWUR29046;AGIWUR35364
CB4856 CT IV 2673098 2675015 WBGene00022377 gpx-4 Y94H6A.4 0.267022108378181
AGIWUR32332
CB4856 CT IV 2676512 2678162 WBGene00022376 Y94H6A.3 Y94H6A.3 0.0220776816076587
AGIWUR1371
CB4856 CT IV 2680098 2688808 WBGene00044651 egrh-3 Y94H6A.11 -0.0906440983323682
AGIWUR18045;AGIWUR18215
CB4856 CT IV 2688947 2703977 WBGene00022378 Y94H6A.5 Y94H6A.5 0.0325884955241165
AGIWUR39570
CB4856 CT IV 2704693 2707231 WBGene00006705 ubc-8 Y94H6A.6 0.747988288554489
AGIWUR17647
CB4856 CT IV 2709942 2711374 WBGene00022382 Y94H6A.10 Y94H6A.10 -0.671216212311773
AGIWUR41705
CB4856 CT IV 2711939 2713035 WBGene00050885 Y94H6A.12 Y94H6A.12 -0.0705074264224699
AGIWUR4052
CB4856 CT IV 2713907 2716574 WBGene00022379 Y94H6A.7 Y94H6A.7 -0.368202852542185
AGIWUR2447;AGIWUR5357;AGIWUR6386;AGIWUR7246
CB4856 CT IV 2716661 2719834 WBGene00022380 Y94H6A.8 Y94H6A.8 -0.17476539463275
AGIWUR29445
CB4856 CT IV 2720323 2725858 WBGene00022381 ubxn-2 Y94H6A.9 -0.00935937279555255
AGIWUR14157;AGIWUR22376;AGIWUR23767;AGIWUR25071;AGIWUR28469;AGIWUR30699
CB4856 CT IV 2730252 2732404 WBGene00022375 Y94H6A.2 Y94H6A.2 1.07993687346641
AGIWUR29436
CB4856 CT IV 2743007 2747476 WBGene00022374 nhr-277 Y94H6A.1 0.156549419143182
AGIWUR28763
CB4856 CT IV 2751868 2753461 WBGene00044485 Y54G2A.41 Y54G2A.41 -0.204491044915695
AGIWUR14592;AGIWUR15871;AGIWUR18786;AGIWUR23658;AGIWUR44244
CB4856 CT IV 2762680 2765612 WBGene00235350 Y54G2A.77 Y54G2A.77 0.0500447133226203
AGIWUR18732
CB4856 CT IV 2765719 2771790 WBGene00021876 Y54G2A.11 Y54G2A.11 0.931559181604933
AGIWUR7881;AGIWUR8693;AGIWUR10151;AGIWUR12762;AGIWUR13973;AGIWUR16616
CB4856 CT IV 2777232 2782843 WBGene00021877 Y54G2A.12 Y54G2A.12 -0.294777650512266
AGIWUR4693;AGIWUR23522
CB4856 CT IV 2788692 2796805 WBGene00021868 atln-1 Y54G2A.2 -0.171059515083495
AGIWUR33912
CB4856 CT IV 2797061 2798573 WBGene00045397 Y54G2A.52 Y54G2A.52 0.439120587409796
AGIWUR10865;AGIWUR18487;AGIWUR25860;AGIWUR31076;AGIWUR40561;AGIWUR45067
CB4856 CT IV 2799689 2801039 WBGene00021875 Y54G2A.10 Y54G2A.10 1.04126659516663
AGIWUR7140;AGIWUR12331;AGIWUR26440

CB4856 CT IV 2801902 2803240 WBGene00044487 Y54G2A.44 Y54G2A.44 0.0617316945128294
AGIWUR24750;AGIWUR34109
CB4856 CT IV 2803589 2804916 WBGene00021894 Y54G2A.32 Y54G2A.32 0.569361767088814
AGIWUR13210
CB4856 CT IV 2805839 2809504 WBGene00044488 Y54G2A.45 Y54G2A.45 -0.438731605958452
AGIWUR7243;AGIWUR20787
CB4856 CT IV 2814568 2819733 WBGene00021878 Y54G2A.13 Y54G2A.13 -0.0322674912278522
AGIWUR42785
CB4856 CT IV 2823746 2828069 WBGene00043062 clec-80 Y54G2A.39 0.257733492106215
AGIWUR35552
CB4856 CT IV 2831477 2832687 WBGene00044491 Y54G2A.48 Y54G2A.48 -0.0106138440964161
AGIWUR2292;AGIWUR8308;AGIWUR23599;AGIWUR35331;AGIWUR36640
CB4856 CT IV 2832877 2833480 WBGene00044489 pudl-1 Y54G2A.46 0.351414348663054
AGIWUR15460;AGIWUR22792
CB4856 CT IV 2834816 2835357 WBGene00044490 pudl-2 Y54G2A.47 0.288560788606981
AGIWUR3206;AGIWUR12409;AGIWUR21422
CB4856 CT IV 2835664 2838583 WBGene00021873 clec-82 Y54G2A.8 1.7377816158785 AGIWUR28251
CB4856 CT IV 2838608 2839422 WBGene00043702 Y54G2A.7 Y54G2A.7 0.00630069250457232
AGIWUR6401;AGIWUR8166;AGIWUR14841;AGIWUR23759;AGIWUR31835;AGIWUR32286;AGIWUR34675;AGIWUR36900;AGIWUR37090;A
GIWUR40142
CB4856 CT IV 2839375 2841434 WBGene00021879 clec-83 Y54G2A.14 0.272040512932342
AGIWUR41939
CB4856 CT IV 2842474 2845604 WBGene00021895 clec-84 Y54G2A.33 0.196501808918722
AGIWUR29146
CB4856 CT IV 2846476 2851282 WBGene00044495 Y54G2A.40 Y54G2A.40 -0.295675375411856
AGIWUR3193;AGIWUR17597
CB4856 CT IV 2851691 2854293 WBGene00044492 Y54G2A.49 Y54G2A.49 0.26751918227749
AGIWUR13172;AGIWUR17819;AGIWUR31750;AGIWUR41764;AGIWUR42281
CB4856 CT IV 2854339 2855456 WBGene00021872 clec-85 Y54G2A.6 -0.301152091473041
AGIWUR3905
CB4856 CT IV 2855607 2864843 WBGene00021881 Y54G2A.16 Y54G2A.16 -0.210082244382789
AGIWUR29314
CB4856 CT IV 2872050 2890449 WBGene00021882 nprt-1 Y54G2A.17 -0.0754948812967342
AGIWUR2046;AGIWUR6560;AGIWUR18981;AGIWUR41129
CB4856 CT IV 2890888 2891636 WBGene00235102 Y54G2A.75 Y54G2A.75
-3.5527136788005e-15 AGIWUR32909
CB4856 CT IV 2891560 2892495 WBGene00021883 Y54G2A.18 Y54G2A.18 -0.166457271196973
AGIWUR14241;AGIWUR28071
CB4856 CT IV 2893819 2894730 WBGene00021884 Y54G2A.19 Y54G2A.19 -0.173375437872863

AGIWUR2785
CB4856 CT IV 2896968 2900638 WBGene00021885 Y54G2A.20 Y54G2A.20 -0.0207325968592356
AGIWUR17990
CB4856 CT IV 2902436 2914816 WBGene00021886 Y54G2A.21 Y54G2A.21 -0.29008067670214
AGIWUR473;AGIWUR4090;AGIWUR6409;AGIWUR28153
CB4856 CT IV 2918441 2919780 WBGene00044493 Y54G2A.50 Y54G2A.50 -0.243381717767439
AGIWUR14616;AGIWUR16703
CB4856 CT IV 2919826 2922251 WBGene00021887 fbxc-45 Y54G2A.22 -0.0516808895756906
AGIWUR4439
CB4856 CT IV 2922591 2925691 WBGene00006708 ubc-13 Y54G2A.31 -0.0706457972150059
AGIWUR3658
CB4856 CT IV 2927515 2928421 WBGene00021888 Y54G2A.23 Y54G2A.23 -0.238095690654607
AGIWUR8295
CB4856 CT IV 2931403 2936843 WBGene00021870 samt-1 Y54G2A.4 -0.109192979407579
AGIWUR10505
CB4856 CT IV 2937990 2939862 WBGene00021889 Y54G2A.24 Y54G2A.24 0.0442477538982118
AGIWUR20311
CB4856 CT IV 2960123 2964395 WBGene00021897 ser-6 Y54G2A.35 0.139848257542336
AGIWUR7278
CB4856 CT IV 2964365 2978461 WBGene00002243 lad-2 Y54G2A.25 0.390351388049006
AGIWUR29465;AGIWUR35601;AGIWUR41610
CB4856 CT IV 2986104 2995573 WBGene00021890 Y54G2A.26 Y54G2A.26 -0.088748880932112
AGIWUR29419;AGIWUR31009;AGIWUR39038
CB4856 CT IV 2998877 2999485 WBGene00021891 Y54G2A.27 Y54G2A.27 -0.0025943404432569
AGIWUR32480
CB4856 CT IV 3000541 3008892 WBGene00219425 Y54G2A.73 Y54G2A.73 -0.0717095418741067
AGIWUR14853;AGIWUR19329;AGIWUR28296;AGIWUR28643;AGIWUR37339
CB4856 CT IV 3012587 3014440 WBGene00021869 vrp-1 Y54G2A.3 -0.406236780316867
AGIWUR26458
CB4856 CT IV 3019738 3022315 WBGene00003008 lin-22 Y54G2A.1 0.117882108251942
AGIWUR15555;AGIWUR17357;AGIWUR23870
CB4856 CT IV 3023403 3028942 WBGene00021893 ndnf-1 Y54G2A.29 0.116825860817181
AGIWUR24891
CB4856 CT IV 3032578 3050102 WBGene00022069 eel-1 Y67D8C.5 0.195540981332685
AGIWUR9265;AGIWUR12793;AGIWUR16370;AGIWUR19875;AGIWUR25410;AGIWUR32832;AGIWUR34877;AGIWUR36944
CB4856 CT IV 3053730 3062053 WBGene00022067 Y67D8C.3 Y67D8C.3 0.151113372069264
AGIWUR20090;AGIWUR34714
CB4856 CT IV 3062524 3063366 WBGene00022070 Y67D8C.6 Y67D8C.6 -0.276810586397012
AGIWUR18899

CB4856	CT	IV	3065568 3066934 WBGene00022071	Y67D8C.7	Y67D8C.7	-0.100957432774284
AGIWUR3480;AGIWUR14950;AGIWUR36360
CB4856	CT	IV	3067848 3069876 WBGene00022072	cpg-9	Y67D8C.8	-0.582654445572865
AGIWUR798;AGIWUR6063;AGIWUR12656;AGIWUR20171;AGIWUR23004;AGIWUR23055;AGIWUR23636;AGIWUR24578;AGIWUR25102;AGIWUR33268;AGIWUR38111;AGIWUR39434
CB4856	CT	IV	3070134 3076307 WBGene00235279	Y67D8C.22	Y67D8C.22	-0.22396238525995
AGIWUR11754
CB4856	CT	IV	3077466 3086923 WBGene00022073	Y67D8C.9	Y67D8C.9	0.147880064037387
AGIWUR128;AGIWUR18909;AGIWUR22466;AGIWUR23929;AGIWUR24530;AGIWUR24619;AGIWUR26816;AGIWUR34605;AGIWUR35226;AGIWUR38622;AGIWUR39640
CB4856	CT	IV	3103157 3133147 WBGene00003153	mca-3	Y67D8C.10	-0.0769739827742661
AGIWUR13343;AGIWUR25220
CB4856	CT	IV	3139833 3146723 WBGene00020729	T23E1.2 T23E1.2 0.0741539858265847
AGIWUR8351;AGIWUR39712
CB4856	CT	IV	3146894 3148882 WBGene00020730	T23E1.3 T23E1.3 0.0569803163427842	AGIWUR38188
CB4856	CT	IV	3151361 3155987 WBGene00020728	T23E1.1 T23E1.1 0.106227135373084
AGIWUR1768;AGIWUR6637;AGIWUR10515;AGIWUR12603;AGIWUR17243;AGIWUR18643;AGIWUR19244;AGIWUR27038;AGIWUR33531;AGIWUR33996;AGIWUR38671
CB4856	CT	IV	3169946 3172610 WBGene00022061	Y67D8B.1	Y67D8B.1
-0.00222535554991499	AGIWUR30463
CB4856	CT	IV	3173550 3176115 WBGene00022063	Y67D8B.4	Y67D8B.4	0.236310303032953
AGIWUR12437;AGIWUR15848;AGIWUR18862;AGIWUR23118;AGIWUR31340;AGIWUR32394;AGIWUR33681;AGIWUR36083;AGIWUR39191
CB4856	CT	IV	3186080 3191562 WBGene00022062	Y67D8B.2	Y67D8B.2	0.163107224299081
AGIWUR9123;AGIWUR35608
CB4856	CT	IV	3202333 3203861 WBGene00022064	Y67D8B.5	Y67D8B.5	0.253469305109507
AGIWUR29258
CB4856	CT	IV	3204559 3210582 WBGene00004240	puf-4	M4.2	0.273447869936582	AGIWUR19536
CB4856	CT	IV	3213764 3219825 WBGene00019766	M4.1	M4.1	-0.121168041639169	AGIWUR39578
CB4856	CT	IV	3222440 3252601 WBGene00022059	Y67D8A.2	Y67D8A.2	-0.17230119243877
AGIWUR4774;AGIWUR19074;AGIWUR21542;AGIWUR25290
CB4856	CT	IV	3253263 3254543 WBGene00022060	dmd-9	Y67D8A.3	0.0680288569580352
AGIWUR7989
CB4856	CT	IV	3258910 3263489 WBGene00020831	T26C12.1	T26C12.1	0.188275359774572
AGIWUR7690;AGIWUR10912;AGIWUR32472
CB4856	CT	IV	3263851 3265372 WBGene00020832	T26C12.2	T26C12.2	0.0963345118591814
AGIWUR6073
CB4856	CT	IV	3266404 3267677 WBGene00044469	clec-173	T26C12.6	-0.226487828266153
AGIWUR20423
CB4856	CT	IV	3269527 3273397 WBGene00020833	T26C12.3	T26C12.3	0.642729576825044

AGIWUR1492;AGIWUR16675;AGIWUR27212;AGIWUR38075
CB4856	CT	IV	3282885	3289337	WBGene00001548	gcy-23	T26C12.4		-0.00496207388707326	AGIWUR42079
CB4856	CT	IV	3292120	3294633	WBGene00021089	W08E12.8		W08E12.8		-0.10222805490266	AGIWUR25114
CB4856	CT	IV	3295135	3297225	WBGene00021088	W08E12.7		W08E12.7		-0.093140078488096	AGIWUR209;AGIWUR14640
CB4856	CT	IV	3298629	3299687	WBGene00021087	W08E12.6		W08E12.6		0.553844171000896	AGIWUR36833
CB4856	CT	IV	3305898	3306541	WBGene00021083	W08E12.2		W08E12.2		0.0860955492684834	AGIWUR27318
CB4856	CT	IV	3306664	3307563	WBGene00021090	W08E12.9		W08E12.9		0.324204816276395	AGIWUR887
CB4856	CT	IV	3311276	3313180	WBGene00021082	zip-11	W08E12.1		-0.114510001567748	AGIWUR24069
CB4856	CT	IV	3320013	3324950	WBGene00018325	F42A6.3	F42A6.3	-0.125072705977557	AGIWUR41089
CB4856	CT	IV	3334042	3336868	WBGene00018328	F42A6.6	F42A6.6	-0.1268014087129	AGIWUR33815
CB4856	CT	IV	3336927	3338867	WBGene00001999	hrp-1	F42A6.7	-0.103045581538375	AGIWUR11495;AGIWUR13108;AGIWUR16261;AGIWUR18729;AGIWUR24759;AGIWUR30889;AGIWUR35196;AGIWUR42971;AGIWUR43704
CB4856	CT	IV	3339792	3340953	WBGene00018329	fbxa-130		F42A6.8	-0.362067016729601	AGIWUR41895
CB4856	CT	IV	3341470	3341939	WBGene00018324	F42A6.2	F42A6.2	0.0296679887088001	AGIWUR28289
CB4856	CT	IV	3342519	3350027	WBGene00018330	elks-1	F42A6.9	0.0361795195624168	AGIWUR3955
CB4856	CT	IV	3363553	3366079	WBGene00021953	Y55H10A.2		Y55H10A.2		0.174342504806288	AGIWUR44245
CB4856	CT	IV	3369197	3373467	WBGene00021952	vha-19	Y55H10A.1		-0.123657235331949	AGIWUR27642
CB4856	CT	IV	3373387	3378130	WBGene00015249	otub-4	B0546.2	0.095359483124227	AGIWUR36668;AGIWUR40143
CB4856	CT	IV	3382949	3384147	WBGene00015251	B0546.4	B0546.4	0.322492993730206	AGIWUR8456
CB4856	CT	IV	3385903	3386904	WBGene00015248	mai-2	B0546.1	0.0143773086581298	AGIWUR23582
CB4856	CT	IV	3396581	3399123	WBGene00001119	dyf-3	C04C3.5	0.0675353235795821	AGIWUR32839
CB4856	CT	IV	3399672	3403904	WBGene00015413	pdhb-1	C04C3.3	0.212500905946641	AGIWUR9048;AGIWUR21733
CB4856	CT	IV	3415683	3419234	WBGene00015412	lgc-9	C04C3.2	-0.296480831612806	AGIWUR16793
CB4856	CT	IV	3419813	3422607	WBGene00015416	C04C3.6	C04C3.6	-0.0563764696231797	AGIWUR7655;AGIWUR10839;AGIWUR23041
CB4856	CT	IV	3428900	3430327	WBGene00015411	srz-60	C04C3.1	-0.346164861072951	AGIWUR41151
CB4856	CT	IV	3430310	3432470	WBGene00219646	linc-89	C04C3.15		-0.048220544173561

AGIWUR39779
CB4856 CT IV 3436319 3437878 WBGene00021033 srz-32 W05E7.2 0.00755327965762564
AGIWUR8896;AGIWUR37518;AGIWUR39643
CB4856 CT IV 3438312 3439503 WBGene00001702 grd-13 W05E7.3 -0.607838312814312 AGIWUR1917
CB4856 CT IV 3439655 3440453 WBGene00001692 grd-3 W05E7.1 -0.525481408736912
AGIWUR6630;AGIWUR31335
CB4856 CT IV 3441055 3441770 WBGene00001699 grd-10 F09D12.1 -0.508977978756024
AGIWUR27659
CB4856 CT IV 3442303 3444201 WBGene00017281 F09D12.2 F09D12.2 -0.110314138990613
AGIWUR38734
CB4856 CT IV 3444914 3446528 WBGene00019049 F58E2.5 F58E2.5 0.0179618013224427 AGIWUR34019
CB4856 CT IV 3447216 3448708 WBGene00023302 F58E2.6 F58E2.6 0.16921902577056 AGIWUR18347
CB4856 CT IV 3453608 3454788 WBGene00019051 srz-23 F58E2.9 0.170875786517282 AGIWUR34738
CB4856 CT IV 3455893 3457113 WBGene00019050 srz-88 F58E2.7 -0.0765572175806071
AGIWUR19533;AGIWUR37029
CB4856 CT IV 3458600 3460440 WBGene00023481 srz-22 F58E2.10 -0.0344176158916625
AGIWUR42395
CB4856 CT IV 3462027 3462929 WBGene00023303 F58E2.8 F58E2.8 -0.0256417418907695 AGIWUR12004
CB4856 CT IV 3463567 3466276 WBGene00019048 F58E2.4 F58E2.4 -0.0618127872887069
AGIWUR16957;AGIWUR18621;AGIWUR27185
CB4856 CT IV 3467430 3469828 WBGene00019047 F58E2.3 F58E2.3 0.0326382392482198 AGIWUR6323
CB4856 CT IV 3470042 3470711 WBGene00019046 F58E2.2 F58E2.2 -0.2957947942499 AGIWUR15414
CB4856 CT IV 3471563 3473584 WBGene00023301 F58E2.1 F58E2.1 -0.101200073157883
AGIWUR9201;AGIWUR12704
CB4856 CT IV 3477392 3479011 WBGene00015656 srz-70 C09G12.6 0.0485808495966822
AGIWUR23116
CB4856 CT IV 3479335 3479730 WBGene00015655 C09G12.5 C09G12.5 -0.440579893437514
AGIWUR37214
CB4856 CT IV 3480011 3481860 WBGene00023474 srz-37 C09G12.16 0.15406081152695
AGIWUR18826;AGIWUR38245
CB4856 CT IV 3482112 3482565 WBGene00023476 srz-55 C09G12.14 0.144847401817545
AGIWUR278;AGIWUR16470;AGIWUR33376;AGIWUR34469
CB4856 CT IV 3482895 3484706 WBGene00023463 srz-82 C09G12.11 0.0279392228211517
AGIWUR5025;AGIWUR33887
CB4856 CT IV 3485316 3487105 WBGene00023465 srz-81 C09G12.13 0.0644077869735269
AGIWUR5334;AGIWUR17762
CB4856 CT IV 3487524 3489089 WBGene00015654 srz-77 C09G12.4 -0.0633085041833628
AGIWUR30868
CB4856 CT IV 3489858 3491519 WBGene00023464 srz-80 C09G12.12 0.223769207466319

AGIWUR1603
CB4856 CT IV 3491777 3493216 WBGene00015653 srz-78 C09G12.3 -0.157472495684822
AGIWUR40657
CB4856 CT IV 3493610 3495179 WBGene00015652 srz-79 C09G12.2 -0.157846521913221
AGIWUR2191
CB4856 CT IV 3495296 3496440 WBGene00044470 C09G12.17 C09G12.17 0.0553438247516613
AGIWUR671;AGIWUR27248;AGIWUR36976;AGIWUR39683;AGIWUR40123
CB4856 CT IV 3498218 3503912 WBGene00015651 ceh-53 C09G12.1 0.251591335525628
AGIWUR21847
CB4856 CT IV 3504444 3506115 WBGene00023462 srz-83 C09G12.10 0.169808696997135
AGIWUR26431;AGIWUR37625
CB4856 CT IV 3506328 3509527 WBGene00000424 ced-10 C09G12.8 -0.318098955625076
AGIWUR21339;AGIWUR26580;AGIWUR27559
CB4856 CT IV 3510046 3514572 WBGene00015658 tsg-101 C09G12.9 -0.29404477390576
AGIWUR3762;AGIWUR5733;AGIWUR14214
CB4856 CT IV 3516819 3526602 WBGene00006769 unc-33 Y37E11C.1 0.0807989856723044
AGIWUR33833
CB4856 CT IV 3527354 3532521 WBGene00019778 M57.2 M57.2 0.0528743697903042 AGIWUR38368
CB4856 CT IV 3543628 3547600 WBGene00015038 nstp-6 B0212.4 0.0254244771649041 AGIWUR32216
CB4856 CT IV 3551090 3557431 WBGene00003889 osm-9 B0212.5 -0.48875092754244 AGIWUR16919
CB4856 CT IV 3565201 3567814 WBGene00015036 sre-5 B0212.2 -0.129551791501449 AGIWUR39523
CB4856 CT IV 3568979 3577630 WBGene00015035 otpl-1 B0212.1 -0.180097820259763
AGIWUR6709;AGIWUR25191
CB4856 CT IV 3578937 3581020 WBGene00021374 Y37E11B.1 Y37E11B.1 -0.105716918158095
AGIWUR42712
CB4856 CT IV 3580970 3582909 WBGene00021376 blos-7 Y37E11B.3 -0.00712970298429738
AGIWUR24972
CB4856 CT IV 3584314 3593615 WBGene00006383 taf-2 Y37E11B.4 0.00314938974839407
AGIWUR40540
CB4856 CT IV 3593960 3598552 WBGene00021377 Y37E11B.5 Y37E11B.5 -0.0587193598462208
AGIWUR27479;AGIWUR36703
CB4856 CT IV 3598656 3599299 WBGene00021378 Y37E11B.6 Y37E11B.6 -0.0297301496934192
AGIWUR32299
CB4856 CT IV 3599497 3600164 WBGene00021379 Y37E11B.7 Y37E11B.7 -0.178589758998446
AGIWUR1502;AGIWUR9722;AGIWUR10223;AGIWUR10260;AGIWUR10569;AGIWUR32668
CB4856 CT IV 3601735 3603401 WBGene00021380 Y37E11B.9 Y37E11B.9 -0.312289996806642
AGIWUR23524
CB4856 CT IV 3603550 3606466 WBGene00021381 Y37E11B.10 Y37E11B.10 -0.0658399951361912
AGIWUR4295;AGIWUR39675

CB4856	CT	IV	3606784	3609229	WBGene00002188	kgb-2	ZC416.4	0.212537019938914	AGIWUR24635
CB4856	CT	IV	3609218	3613072	WBGene00022610	ZC416.6	ZC416.6	0.208038300175602	AGIWUR9980
CB4856	CT	IV	3613934	3624697	WBGene00000481	cha-1	ZC416.8	0.0409973606343783	AGIWUR11212
CB4856	CT	IV	3633619	3635472	WBGene00022609	ZC416.2	ZC416.2	0.290076022731523	AGIWUR44932
CB4856	CT	IV	3637079	3640265	WBGene00022608	madf-9	ZC416.1	-0.24493924761131	AGIWUR25395;AGIWUR25919
CB4856	CT	IV	3645166	3648684	WBGene00016987	CC8.2	CC8.2	-0.0399030257098296	AGIWUR24618
CB4856	CT	IV	3658877	3668703	WBGene00021362	Y37E11AL.6	Y37E11AL.6	0.278856711085921	AGIWUR14654;AGIWUR28359
CB4856	CT	IV	3683537	3686865	WBGene00003129	map-1	Y37E11AL.7	-0.15598022113981	AGIWUR16605
CB4856	CT	IV	3687565	3694837	WBGene00021363	taf-6.2	Y37E11AL.8	-0.274197324481847	AGIWUR18124
CB4856	CT	IV	3694905	3698436	WBGene00021359	Y37E11AL.3	Y37E11AL.3	-0.0832433121679088	AGIWUR8174;AGIWUR10767;AGIWUR17784
CB4856	CT	IV	3698501	3699722	WBGene00021358	Y37E11AL.2	Y37E11AL.2	0.214725458588404	AGIWUR187
CB4856	CT	IV	3699804	3700654	WBGene00021364	Y37E11AL.9	Y37E11AL.9	0.118222338253073	AGIWUR38046
CB4856	CT	IV	3709515	3715381	WBGene00021357	Y37E11AL.1	Y37E11AL.1	-0.158802793228839	AGIWUR20687
CB4856	CT	IV	3715782	3722790	WBGene00021367	Y37E11AM.3	Y37E11AM.3	-0.485249773844339	AGIWUR3608;AGIWUR8546;AGIWUR9320;AGIWUR16402
CB4856	CT	IV	3722886	3725461	WBGene00021366	Y37E11AM.2	Y37E11AM.2	0.126052504421876	AGIWUR29009
CB4856	CT	IV	3726112	3735495	WBGene00021365	smgl-2	Y37E11AM.1	0.101553162904688	AGIWUR41488
CB4856	CT	IV	3735845	3738917	WBGene00021370	nape-2	Y37E11AR.3	0.292992653445446	AGIWUR1805;AGIWUR6459
CB4856	CT	IV	3739524	3740876	WBGene00021371	nape-1	Y37E11AR.4	0.102255603182497	AGIWUR1499;AGIWUR10188
CB4856	CT	IV	3744048	3747655	WBGene00021369	siah-1	Y37E11AR.2	0.391057063640174	AGIWUR32287
CB4856	CT	IV	3748210	3751101	WBGene00021372	ugt-45	Y37E11AR.5	-0.0342943447465771	AGIWUR35655
CB4856	CT	IV	3752612	3755419	WBGene00021373	Y37E11AR.7	Y37E11AR.7	-0.225622460292882	AGIWUR15651
CB4856	CT	IV	3756242	3759910	WBGene00021368	best-20	Y37E11AR.1	0.429709087074519	AGIWUR14000

CB4856	CT	IV	3761192	3766826	WBGene00006869	vab-2	Y37E11AR.6	0.367813413624127	AGIWUR5528
CB4856	CT	IV	3784358	3794044	WBGene00019546	glct-6	W07G9.2	-0.33449045685498	AGIWUR20512;AGIWUR38088
CB4856	CT	IV	3794767	3796267	WBGene00019545	K09B3.1	K09B3.1	-0.140930454110564	AGIWUR2928;AGIWUR10590;AGIWUR15949;AGIWUR18365;AGIWUR32674;AGIWUR43271
CB4856	CT	IV	3796306	3800461	WBGene00003884	osm-3	M02B7.3	0.24016922784746	AGIWUR1662
CB4856	CT	IV	3801864	3803426	WBGene00019725	algn-14	M02B7.4	-0.332552865372447	AGIWUR4303
CB4856	CT	IV	3803581	3804949	WBGene00019724	M02B7.2	M02B7.2	0.0224914333848938	AGIWUR13365;AGIWUR36768
CB4856	CT	IV	3806703	3807190	WBGene00044690	M02B7.7	M02B7.7	-0.22400238340281	AGIWUR2282
CB4856	CT	IV	3807870	3819328	WBGene00019726	bris-1	M02B7.5	-0.0910230119215356	AGIWUR3769;AGIWUR15598;AGIWUR33890
CB4856	CT	IV	3839720	3841628	WBGene00017906	F28F9.2	F28F9.2	0.0180395925696661	AGIWUR21998
CB4856	CT	IV	3855843	3861552	WBGene00006970	zag-1	F28F9.1	0.768212979638653	AGIWUR16716
CB4856	CT	IV	3862129	3865042	WBGene00000009	aat-8	F28F9.4	0.647288366643095	AGIWUR6881
CB4856	CT	IV	3867147	3871842	WBGene00018144	F37C4.4	F37C4.4	0.173549435733418	AGIWUR1949;AGIWUR44847
CB4856	CT	IV	3871829	3874416	WBGene00018145	F37C4.5	F37C4.5	-0.220228189254302	AGIWUR4389;AGIWUR8043;AGIWUR22760;AGIWUR31925;AGIWUR35017;AGIWUR38139;AGIWUR42284
CB4856	CT	IV	3875410	3878372	WBGene00018146	F37C4.6	F37C4.6	-0.217637284365662	AGIWUR24044
CB4856	CT	IV	3878547	3881187	WBGene00018148	F37C4.8	F37C4.8	0.00509147172451296	AGIWUR10242;AGIWUR16117
CB4856	CT	IV	3881852	3884683	WBGene00018143	oac-23	F37C4.3	-0.0514382679054659	AGIWUR37307
CB4856	CT	IV	3885665	3888836	WBGene00018142	oac-22	F37C4.2	0.414215184173919	AGIWUR8307
CB4856	CT	IV	3889263	3892563	WBGene00018141	oac-21	F37C4.1	0.132252603493837	AGIWUR40823
CB4856	CT	IV	3892998	3893283	WBGene00044471	F56D6.9	F56D6.9	0.568839682781924	AGIWUR9848
CB4856	CT	IV	3894058	3894430	WBGene00044292	F56D6.8	F56D6.8	0.647554671832749	AGIWUR20004
CB4856	CT	IV	3895070	3895506	WBGene00044472	dct-8	F56D6.10	0.349434055100366	AGIWUR2226
CB4856	CT	IV	3895963	3897997	WBGene00219307	F56D6.17	F56D6.17	0.0642211403187352	AGIWUR26987;AGIWUR37616
CB4856	CT	IV	3898478	3899806	WBGene00219304	F56D6.16	F56D6.16	-0.387680504924584	AGIWUR30419
CB4856	CT	IV	3900571	3902326	WBGene00023478	srz-62	F56D6.7	-0.360124986581922	AGIWUR2650;AGIWUR24689
CB4856	CT	IV	3906113	3906493	WBGene00044473	F56D6.11	F56D6.11	0.421895824607565	AGIWUR10325;AGIWUR14565;AGIWUR22261;AGIWUR29588
CB4856	CT	IV	3908107	3908509	WBGene00044474	F56D6.12	F56D6.12	0.0530045793585607	

AGIWUR39239
CB4856 CT IV 3909381 3909799 WBGene00044475 F56D6.13 F56D6.13
0.000286009849364888 AGIWUR35300
CB4856 CT IV 3915610 3916901 WBGene00018973 srz-20 F56D6.5 -0.0836865807104052 AGIWUR43400
CB4856 CT IV 3917679 3918056 WBGene00044476 F56D6.14 F56D6.14 -0.133157418122807
AGIWUR7218;AGIWUR7742;AGIWUR31197;AGIWUR39159;AGIWUR42565
CB4856 CT IV 3919806 3921613 WBGene00018972 srz-19 F56D6.4 -0.0663344860218729 AGIWUR5228
CB4856 CT IV 3926417 3929024 WBGene00018970 clec-68 F56D6.1 -0.0843832460361416
AGIWUR28660;AGIWUR41001
CB4856 CT IV 3932733 3936238 WBGene00021581 clec-70 Y46C8AL.3 0.430390440110827
AGIWUR30235
CB4856 CT IV 3937464 3939924 WBGene00021582 clec-71 Y46C8AL.4 -0.0936907063931365
AGIWUR21185
CB4856 CT IV 3940259 3941702 WBGene00021580 clec-174 Y46C8AL.2 1.83318730077063
AGIWUR33517
CB4856 CT IV 3944816 3947364 WBGene00021583 clec-72 Y46C8AL.5 -0.164415450128656
AGIWUR25323
CB4856 CT IV 3953935 3956210 WBGene00021579 clec-73 Y46C8AL.1 0.211278368426209
AGIWUR38603
CB4856 CT IV 3957005 3960139 WBGene00021585 clec-74 Y46C8AL.8 -0.777782829155694
AGIWUR2330;AGIWUR6622;AGIWUR11329
CB4856 CT IV 3960734 3964657 WBGene00021586 clec-75 Y46C8AL.9 0.0357533110522024
AGIWUR14291;AGIWUR22989;AGIWUR29616;AGIWUR30729;AGIWUR31166
CB4856 CT IV 3965246 3969025 WBGene00021587 clec-76 Y46C8AR.1 0.0181108820359341
AGIWUR23862
CB4856 CT IV 3969931 3972616 WBGene00044343 clec-77 Y46C8AR.3 1.08571889385271
AGIWUR31568
CB4856 CT IV 3973349 3975721 WBGene00018548 clec-79 F47C12.4 -0.0296845520831983
AGIWUR4310;AGIWUR7539;AGIWUR26372
CB4856 CT IV 3976429 3978015 WBGene00005430 srh-220 F47C12.5 -0.308662397129355
AGIWUR20619
CB4856 CT IV 3979825 3981205 WBGene00005432 srh-222 F47C12.10 0.143175572847675
AGIWUR24878;AGIWUR41002;AGIWUR42461
CB4856 CT IV 3981901 3983609 WBGene00005433 srh-223 F47C12.3 -0.165262641358777
AGIWUR17687
CB4856 CT IV 3989102 4014195 WBGene00018547 clec-78 F47C12.2 0.88760666018107
AGIWUR12467;AGIWUR29123;AGIWUR35382
CB4856 CT IV 4014799 4028127 WBGene00020930 hlh-30 W02C12.3 0.39515706093826
AGIWUR12568;AGIWUR14682;AGIWUR22585

CB4856 CT IV 4040308 4041532 WBGene00018065 F35F11.2 F35F11.2 0.0305552308031496
AGIWUR4078;AGIWUR9316;AGIWUR38890
CB4856 CT IV 4042575 4047786 WBGene00018064 cdc-73 F35F11.1 -0.12096503915454
AGIWUR4520;AGIWUR41674
CB4856 CT IV 4047937 4049448 WBGene00003955 pcn-1 W03D2.4 0.0789278294550861
AGIWUR1229;AGIWUR1452;AGIWUR10565;AGIWUR13258;AGIWUR27822;AGIWUR40277
CB4856 CT IV 4050049 4051639 WBGene00006951 wrt-5 W03D2.5 -0.168324827226016 AGIWUR29565
CB4856 CT IV 4056586 4058243 WBGene00020981 W03D2.6 W03D2.6 0.382779031105336 AGIWUR34941
CB4856 CT IV 4062567 4063673 WBGene00020982 ilys-6 W03D2.7 2.49903551654618 AGIWUR15927
CB4856 CT IV 4065905 4068412 WBGene00006173 str-122 W03D2.10 -0.0254715280814617
AGIWUR12209
CB4856 CT IV 4068780 4069548 WBGene00020979 fbxb-55 W03D2.2 0.444621275155057 AGIWUR30957
CB4856 CT IV 4072327 4077644 WBGene00004157 pqn-75 W03D2.1 0.803260179244017 AGIWUR43269
CB4856 CT IV 4077671 4078953 WBGene00016437 C35B1.8 C35B1.8 0.00118699071541739
AGIWUR13784;AGIWUR23914;AGIWUR29188;AGIWUR29380;AGIWUR30138;AGIWUR44411
CB4856 CT IV 4079804 4080207 WBGene00016434 C35B1.4 C35B1.4 -0.264707410681066 AGIWUR17839
CB4856 CT IV 4081136 4081348 WBGene00016433 C35B1.3 C35B1.3 -0.223986840385923
AGIWUR12609;AGIWUR19585;AGIWUR39717
CB4856 CT IV 4083603 4084209 WBGene00016435 C35B1.5 C35B1.5 0.0848834238865823 AGIWUR696
CB4856 CT IV 4086212 4102907 WBGene00016432 C35B1.2 C35B1.2 0.0155326963841809
AGIWUR34404;AGIWUR40227
CB4856 CT IV 4103020 4106220 WBGene00006701 ubc-1 C35B1.1 0.067380651990657
AGIWUR11827;AGIWUR34071
CB4856 CT IV 4109067 4110471 WBGene00016436 C35B1.7 C35B1.7 -0.194161149164188 AGIWUR19562
CB4856 CT IV 4110544 4112487 WBGene00044477 F49F1.14 F49F1.14 -0.150353803554265
AGIWUR17789;AGIWUR24698;AGIWUR25509;AGIWUR28648
CB4856 CT IV 4117732 4119179 WBGene00018643 drd-50 F49F1.1 -0.59758376002539 AGIWUR14882
CB4856 CT IV 4119460 4120399 WBGene00018645 F49F1.5 F49F1.5 1.79446721410939 AGIWUR27910
CB4856 CT IV 4121342 4123166 WBGene00018646 mul-1 F49F1.6 -0.518910920571214
AGIWUR37789;AGIWUR40067
CB4856 CT IV 4123525 4125757 WBGene00018647 F49F1.7 F49F1.7 0.000867923077039201 AGIWUR4538
CB4856 CT IV 4130503 4134631 WBGene00018648 F49F1.8 F49F1.8 -0.0698869455774673
AGIWUR23704;AGIWUR29660
CB4856 CT IV 4138408 4139122 WBGene00018650 F49F1.10 F49F1.10
-0.000924909531116569 AGIWUR29551
CB4856 CT IV 4139685 4140363 WBGene00235368 F49F1.18 F49F1.18 -0.0240823404605042
AGIWUR21703
CB4856 CT IV 4141734 4143019 WBGene00018652 F49F1.12 F49F1.12 -0.360595933927393
AGIWUR15653;AGIWUR25495

CB4856	CT	IV	4145084	4147064	WBGene00019933	R07C12.3	R07C12.3	0.173673612264241	AGIWUR35115
CB4856	CT	IV	4148141	4150055	WBGene00019932	R07C12.2	R07C12.2	-0.855203441052968	AGIWUR2270
CB4856	CT	IV	4152547	4154029	WBGene00019931	R07C12.1	R07C12.1	0.0405696529751642	AGIWUR36113
CB4856	CT	IV	4155273	4157185	WBGene00219216	K08D10.14	K08D10.14	-0.506119138393199	AGIWUR27539
CB4856	CT	IV	4157634	4161789	WBGene00019532	K08D10.9	K08D10.9	-0.406775742966835	AGIWUR39171
CB4856	CT	IV	4162517	4164481	WBGene00019531	scrm-5	K08D10.8	-0.789434320170181	AGIWUR3106
CB4856	CT	IV	4164692	4166230	WBGene00019530	scrm-8	K08D10.7	-0.501604433570459	AGIWUR9450
CB4856	CT	IV	4166541	4168120	WBGene00235369	K08D10.18	K08D10.18	0.130259520916492	AGIWUR35321
CB4856	CT	IV	4168038	4169891	WBGene00019529	K08D10.5	K08D10.5	-0.159575835895031	AGIWUR13883
CB4856	CT	IV	4172076	4173988	WBGene00004385	rnp-2	K08D10.4	-0.144221397626275	AGIWUR23011
CB4856	CT	IV	4174089	4176279	WBGene00004386	rnp-3	K08D10.3	-0.0218240781076737	AGIWUR32748
CB4856	CT	IV	4176388	4178364	WBGene00001033	dnj-15	K08D10.2	0.0412187166026321	AGIWUR41604
CB4856	CT	IV	4178463	4179531	WBGene00019535	tsen-34	K08D10.12	-0.0618863105907046	AGIWUR8924;AGIWUR19233;AGIWUR22445
CB4856	CT	IV	4196173	4196695	WBGene00044478	K06B9.6	K06B9.6	0.803358612265816	AGIWUR1235;AGIWUR5457;AGIWUR15755;AGIWUR17854;AGIWUR29162
CB4856	CT	IV	4199501	4204375	WBGene00003938	pax-2	K06B9.5	0.419046403514402	AGIWUR38588
CB4856	CT	IV	4205975	4218259	WBGene00017934	F30B5.4	F30B5.4	-0.195639969484789	AGIWUR34612
CB4856	CT	IV	4219271	4220083	WBGene00235371	F30B5.11	F30B5.11	0.0512605978516252	AGIWUR18478
CB4856	CT	IV	4222183	4223140	WBGene00044093	srbc-72	F30B5.9	0.136538907215134	AGIWUR5787;AGIWUR27677;AGIWUR41288
CB4856	CT	IV	4223857	4225946	WBGene00044092	srbc-71	F30B5.8	-0.133000995595343	AGIWUR25867;AGIWUR30376
CB4856	CT	IV	4226858	4228017	WBGene00017935	srbc-70	F30B5.6	-0.14136554910712	AGIWUR22342
CB4856	CT	IV	4229195	4231861	WBGene00017936	zipt-2.2	F30B5.7	-0.154917936800976	AGIWUR42862

CB4856	CT	IV	4235614	4236710	WBGene00001074	dpy-13	F30B5.1	-0.317734273912436	AGIWUR16246;AGIWUR43234
CB4856	CT	IV	4239853	4240855	WBGene00023279	F36A4.9	F36A4.9	-0.194166132695678	AGIWUR6068;AGIWUR29161
CB4856	CT	IV	4243548	4244610	WBGene00000611	col-34	F36A4.10	0.165812593880007	AGIWUR6058
CB4856	CT	IV	4244956	4245620	WBGene00018086	F36A4.11	F36A4.11	0.150380188109617	AGIWUR22161
CB4856	CT	IV	4246760	4247486	WBGene00018085	ttr-20	F36A4.8	0.372430644085759	AGIWUR31794
CB4856	CT	IV	4248020	4258173	WBGene00000123	ama-1	F36A4.7	0.0482513546204655	AGIWUR3117;AGIWUR13557;AGIWUR21122;AGIWUR27884;AGIWUR30437;AGIWUR40528
CB4856	CT	IV	4263293	4265202	WBGene00000610	col-33	F36A4.6	-0.349809556485967	AGIWUR7365
CB4856	CT	IV	4265524	4266598	WBGene00018084	F36A4.5	F36A4.5	0.14752401933613	AGIWUR32046
CB4856	CT	IV	4267582	4268236	WBGene00018083	F36A4.4	F36A4.4	0.364119686233902	AGIWUR41627
CB4856	CT	IV	4269608	4270186	WBGene00018082	F36A4.3	F36A4.3	0.0893698122425928	AGIWUR2033
CB4856	CT	IV	4271215	4271820	WBGene00018081	F36A4.2	F36A4.2	0.050876686474707	AGIWUR6627;AGIWUR15954;AGIWUR37357
CB4856	CT	IV	4272473	4272899	WBGene00018080	F36A4.1	F36A4.1	0.0296971493324572	AGIWUR37819
CB4856	CT	IV	4274341	4277875	WBGene00003668	nhr-78	F36A4.14	0.174338479004261	AGIWUR11194
CB4856	CT	IV	4278825	4284120	WBGene00017487	F15E6.6	F15E6.6	-0.0280526511158534	AGIWUR27430
CB4856	CT	IV	4285403	4288432	WBGene00005715	srv-4	F15E6.7	0.128945818744894	AGIWUR4587;AGIWUR9840;AGIWUR33733
CB4856	CT	IV	4289567	4290061	WBGene00017486	F15E6.5	F15E6.5	0.145946508577229	AGIWUR24506
CB4856	CT	IV	4290189	4290703	WBGene00017488	dct-7	F15E6.8	0.328892161905388	AGIWUR18791
CB4856	CT	IV	4291467	4291872	WBGene00017485	F15E6.4	F15E6.4	-0.366797244564054	AGIWUR10390;AGIWUR18519;AGIWUR29557;AGIWUR30230;AGIWUR37617;AGIWUR43915
CB4856	CT	IV	4292869	4293611	WBGene00044479	F15E6.10	F15E6.10	1.04105296133449	AGIWUR14420;AGIWUR18856;AGIWUR23310;AGIWUR31892;AGIWUR36814
CB4856	CT	IV	4293620	4299083	WBGene00017489	F15E6.9	F15E6.9	0.137300813064408	AGIWUR4362
CB4856	CT	IV	4301308	4304437	WBGene00017483	lgc-22	F15E6.2	0.762570098012153	AGIWUR23008
CB4856	CT	IV	4326656	4332018	WBGene00020972	trpl-2	W03B1.2	-0.300269003344908	AGIWUR25968
CB4856	CT	IV	4332692	4335372	WBGene00020973	W03B1.3	W03B1.3	0.31969726551064	AGIWUR32449
CB4856	CT	IV	4336834	4341304	WBGene00020976	oac-51	W03B1.7	0.0510002849189943	AGIWUR63;AGIWUR4276
CB4856	CT	IV	4342357	4346558	WBGene00020977	oac-52	W03B1.8	-0.0277772110442225	AGIWUR14080
CB4856	CT	IV	4357631	4359936	WBGene00005662	sars-2	W03B1.4	-0.0204235115662499	AGIWUR1886;AGIWUR40320;AGIWUR44856
CB4856	CT	IV	4359953	4360657	WBGene00050916	F55F10.3	F55F10.3	-0.240773556295164	

AGIWUR16657;AGIWUR29416;AGIWUR41260
CB4856 CT IV 4360837 4386533 WBGene00018898 F55F10.1 F55F10.1 -0.107249012453963
AGIWUR28847
CB4856 CT IV 4386630 4388878 WBGene00021286 tald-1 Y24D9A.8 -0.0703519608938343
AGIWUR1068;AGIWUR23705
CB4856 CT IV 4389135 4390649 WBGene00004419 rpl-7A Y24D9A.4 -0.060474001946627
AGIWUR8205
CB4856 CT IV 4390848 4393403 WBGene00021282 set-21 Y24D9A.2 -0.237388778913752
AGIWUR34746
CB4856 CT IV 4394410 4415622 WBGene00021281 ell-1 Y24D9A.1 0.0801170939939571
AGIWUR632;AGIWUR12116
CB4856 CT IV 4422339 4426566 WBGene00019950 clec-175 R08C7.6 0.0562461667330165
AGIWUR5192;AGIWUR14417;AGIWUR21071
CB4856 CT IV 4427713 4429782 WBGene00006228 str-185 R08C7.7 0.167157134410722 AGIWUR27
CB4856 CT IV 4433049 4434541 WBGene00019951 R08C7.8 R08C7.8 -0.113054253556078 AGIWUR24643
CB4856 CT IV 4435127 4437318 WBGene00019948 R08C7.4 R08C7.4 -0.0164856558048037 AGIWUR1608
CB4856 CT IV 4437786 4439290 WBGene00019952 fbxb-74 R08C7.9 0.0841660738924173
AGIWUR6404;AGIWUR13245;AGIWUR18904;AGIWUR24042;AGIWUR27521;AGIWUR30324
CB4856 CT IV 4442399 4445876 WBGene00019953 wapl-1 R08C7.10 -0.0925316016721833
AGIWUR3049;AGIWUR3502;AGIWUR5857;AGIWUR6345;AGIWUR8110;AGIWUR14201;AGIWUR16332;AGIWUR20448;AGIWUR45027
CB4856 CT IV 4445990 4446997 WBGene00019947 htz-1 R08C7.3 0.158962609710088
AGIWUR35276;AGIWUR40345
CB4856 CT IV 4447299 4449897 WBGene00019946 chat-1 R08C7.2 0.102925935205864
AGIWUR23617;AGIWUR24421
CB4856 CT IV 4450000 4460027 WBGene00019955 R08C7.12 R08C7.12 0.470419484859928
AGIWUR26172;AGIWUR28842;AGIWUR31454;AGIWUR40599;AGIWUR43621
CB4856 CT IV 4460179 4462061 WBGene00019718 M01H9.2 M01H9.2 0.32334223721897 AGIWUR8857
CB4856 CT IV 4465484 4468509 WBGene00019721 M01H9.5 M01H9.5 -0.123081624535313 AGIWUR11586
CB4856 CT IV 4468505 4477059 WBGene00019719 M01H9.3 M01H9.3 0.56665478501812
AGIWUR3461;AGIWUR16506;AGIWUR18811;AGIWUR37123;AGIWUR42973
CB4856 CT IV 4479693 4482764 WBGene00019720 M01H9.4 M01H9.4 0.525213557070143
AGIWUR1939;AGIWUR17950;AGIWUR22449;AGIWUR29216;AGIWUR30278;AGIWUR36545
CB4856 CT IV 4487028 4489000 WBGene00019717 trx-3 M01H9.1 1.46375042127451 AGIWUR33279
CB4856 CT IV 4489012 4506708 WBGene00022675 gbb-2 ZK180.1 -0.00381006262142503
AGIWUR3783;AGIWUR5291;AGIWUR12774;AGIWUR13601;AGIWUR20036;AGIWUR29856;AGIWUR31024;AGIWUR31657;AGIWUR32763;A
GIWUR33247;AGIWUR33381;AGIWUR33807;AGIWUR35502;AGIWUR41532;AGIWUR42433
CB4856 CT IV 4508499 4511320 WBGene00022677 ZK180.3 ZK180.3 0.0649324639584412
AGIWUR25175;AGIWUR32103
CB4856 CT IV 4511896 4513319 WBGene00022678 sar-1 ZK180.4 -0.156269641772131 AGIWUR17236

CB4856	CT	IV	4515044 4517169 WBGene00022679	ZK180.5 ZK180.5 -0.429680613961975
AGIWUR13104;AGIWUR17731;AGIWUR25887;AGIWUR38271
CB4856	CT	IV	4520181 4525043 WBGene00022680	ZK180.6 ZK180.6 -0.547469972564635	AGIWUR1519
CB4856	CT	IV	4528730 4530382 WBGene00022683	ZK185.3 ZK185.3 -0.304731209811184	AGIWUR18708
CB4856	CT	IV	4531222 4533121 WBGene00044480	ZK185.4 ZK185.4 -0.0977104695448827
AGIWUR32050;AGIWUR33234
CB4856	CT	IV	4536553 4541771 WBGene00022682	ZK185.2 ZK185.2 0.0732135990855012	AGIWUR38270
CB4856	CT	IV	4541769 4545918 WBGene00044481	ZK185.5 ZK185.5 -0.355537315630089
AGIWUR5901;AGIWUR10425
CB4856	CT	IV	4548553 4552742 WBGene00022681	ZK185.1 ZK185.1 -0.415422503217218	AGIWUR11021
CB4856	CT	IV	4562654 4565674 WBGene00017901	igeg-1	F28E10.2	0.522150208232652
AGIWUR35893
CB4856	CT	IV	4566018 4567443 WBGene00017902	F28E10.4	F28E10.4
-0.00380302975342417	AGIWUR500;AGIWUR10120;AGIWUR31071;AGIWUR32695;AGIWUR32757;AGIWUR32829
CB4856	CT	IV	4568841 4590297 WBGene00017900	F28E10.1	F28E10.1	0.184918146473642
AGIWUR450;AGIWUR3642;AGIWUR15545;AGIWUR26942;AGIWUR33923;AGIWUR35572;AGIWUR36232;AGIWUR41759
CB4856	CT	IV	4595052 4595530 WBGene00017596	F19C7.6 F19C7.6 0.366012903389812	AGIWUR35427
CB4856	CT	IV	4595958 4596732 WBGene00017595	F19C7.5 F19C7.5 0.433000832744231	AGIWUR33005
CB4856	CT	IV	4605786 4608216 WBGene00017591	F19C7.1 F19C7.1 -0.145837440344227	AGIWUR43965
CB4856	CT	IV	4608201 4609679 WBGene00000684	col-110 F19C7.7 -0.452655089886363
AGIWUR14499;AGIWUR24835
CB4856	CT	IV	4610576 4638095 WBGene00017597	F19C7.8 F19C7.8 0.228733658390595
AGIWUR16659;AGIWUR19393;AGIWUR23800;AGIWUR28813;AGIWUR29521
CB4856	CT	IV	4647202 4649743 WBGene00017923	F29B9.8 F29B9.8 -0.179581702897645	AGIWUR11048
CB4856	CT	IV	4649744 4650654 WBGene00017922	F29B9.7 F29B9.7 0.0894250080802106	AGIWUR18836
CB4856	CT	IV	4651044 4652468 WBGene00006706	ubc-9	F29B9.6 -0.285066363269054
AGIWUR18958;AGIWUR43174
CB4856	CT	IV	4652459 4653159 WBGene00017921	F29B9.5 F29B9.5 -0.220816566052315	AGIWUR26617
CB4856	CT	IV	4653293 4655848 WBGene00004205	psr-1	F29B9.4 -0.197911676125401	AGIWUR21294
CB4856	CT	IV	4655776 4657783 WBGene00000685	col-111 F29B9.9 -0.159105576668125
AGIWUR4023;AGIWUR27103;AGIWUR31068;AGIWUR31841;AGIWUR33074;AGIWUR37890;AGIWUR38286;AGIWUR39857;AGIWUR41390
CB4856	CT	IV	4659049 4659517 WBGene00017924	mrps-21 F29B9.10	-0.240744666511103
AGIWUR3956
CB4856	CT	IV	4659521 4660203 WBGene00017925	F29B9.11	F29B9.11	-0.0980434914137049
AGIWUR3042;AGIWUR27108;AGIWUR33457;AGIWUR35521;AGIWUR41724;AGIWUR43364
CB4856	CT	IV	4660991 4665204 WBGene00017920	jmjd-1.2	F29B9.2 -0.113109598955997
AGIWUR352;AGIWUR20412;AGIWUR21912
CB4856	CT	IV	4665251 4667293 WBGene00017919	F29B9.1 F29B9.1 0.011546542233555	AGIWUR28303
CB4856	CT	IV	4669529 4671992 WBGene00044482	F29B9.12	F29B9.12	-0.037025117192246

AGIWUR10537
CB4856 CT IV 4679018 4685168 WBGene00020678 T22B11.4 T22B11.4 0.328678320067493
AGIWUR181;AGIWUR5515;AGIWUR9417;AGIWUR13970;AGIWUR23451;AGIWUR32601;AGIWUR33091
CB4856 CT IV 4688269 4691936 WBGene00044620 bus-4 T22B11.2 0.0628409114537938
AGIWUR34594
CB4856 CT IV 4692793 4696826 WBGene00020675 T22B11.1 T22B11.1 0.137823689381416
AGIWUR44942
CB4856 CT IV 4696982 4704011 WBGene00020679 ogdh-1 T22B11.5 0.0182350393526134
AGIWUR20048;AGIWUR28410
CB4856 CT IV 4706777 4710755 WBGene00019543 cif-1 K08F11.3 0.107723853039803
AGIWUR36907;AGIWUR44735
CB4856 CT IV 4710861 4712624 WBGene00006968 yars-2 K08F11.4 -0.127492925127436
AGIWUR1336;AGIWUR23561;AGIWUR33150
CB4856 CT IV 4712825 4715807 WBGene00019544 miro-1 K08F11.5 -0.000798539025578471
AGIWUR13942;AGIWUR43333
CB4856 CT IV 4724862 4725332 WBGene00019541 K08F11.1 K08F11.1 -0.156907212641285
AGIWUR16904
CB4856 CT IV 4730636 4732750 WBGene00017118 E04A4.3 E04A4.3 1.39949552747857 AGIWUR23548
CB4856 CT IV 4734305 4735876 WBGene00017117 fbxb-80 E04A4.2 0.278683906599271 AGIWUR35293
CB4856 CT IV 4737558 4738734 WBGene00017116 fbxb-78 E04A4.1 0.472383505248546 AGIWUR23218
CB4856 CT IV 4739490 4744269 WBGene00001983 hoe-1 E04A4.4 -0.293760035256934 AGIWUR18753
CB4856 CT IV 4744377 4745492 WBGene00017119 timm-17B.1 E04A4.5 -0.0785766104786667
AGIWUR16514
CB4856 CT IV 4745825 4747832 WBGene00017120 tctn-1 E04A4.6 0.409999176539287 AGIWUR11344
CB4856 CT IV 4747901 4749113 WBGene00017121 cyc-2.1 E04A4.7 0.0906719459341616
AGIWUR322;AGIWUR10063;AGIWUR11494;AGIWUR16074;AGIWUR24498;AGIWUR26130;AGIWUR28305;AGIWUR30014;AGIWUR36824;A
GIWUR38385;AGIWUR43982
CB4856 CT IV 4749012 4749778 WBGene00004432 rpl-20 E04A4.8 0.0130668217736645
AGIWUR2616;AGIWUR4332;AGIWUR15631;AGIWUR18334;AGIWUR27175;AGIWUR37198
CB4856 CT IV 4750240 4751491 WBGene00021202 Y17G9B.5 Y17G9B.5 -0.104443778233271
AGIWUR26981
CB4856 CT IV 4752119 4753163 WBGene00021201 Y17G9B.4 Y17G9B.4 0.169946416559144
AGIWUR20135
CB4856 CT IV 4754199 4756157 WBGene00021204 fbxb-77 Y17G9B.7 -0.005815423155763
AGIWUR5085;AGIWUR28574
CB4856 CT IV 4757179 4758840 WBGene00021205 Y17G9B.8 Y17G9B.8 -0.122182669827696
AGIWUR17568
CB4856 CT IV 4758982 4760428 WBGene00003216 memi-1 Y17G9B.9 -0.310395454579808
AGIWUR30173

CB4856	CT	IV	4761049	4762856	WBGene00021200	cyp-31A3	Y17G9B.3	-0.134400041585007	AGIWUR18877
CB4856	CT	IV	4764182	4764602	WBGene00021199	Y17G9B.2	Y17G9B.2	0.579306896196368	AGIWUR35670
CB4856	CT	IV	4764748	4767839	WBGene00021198	Y17G9B.1	Y17G9B.1	0.141456291454746	AGIWUR6283;AGIWUR20407;AGIWUR41861
CB4856	CT	IV	4772092	4775133	WBGene00020004	R11E3.2	R11E3.2	0.00920193307847583	AGIWUR10352
CB4856	CT	IV	4775242	4776096	WBGene00235374	R11E3.19	R11E3.19	0.664200057854702	AGIWUR17262
CB4856	CT	IV	4784233	4788692	WBGene00003694	nhr-104	R11E3.5	0.0189268618369867	AGIWUR26636;AGIWUR33735
CB4856	CT	IV	4789503	4791983	WBGene00020003	R11E3.1	R11E3.1	0.0115858049879005	AGIWUR18867;AGIWUR23382
CB4856	CT	IV	4793202	4799378	WBGene00001324	eor-1	R11E3.6	0.0436948898995331	AGIWUR23443;AGIWUR36822
CB4856	CT	IV	4799388	4802592	WBGene00001060	dpf-7	R11E3.7	-0.238450673160796	AGIWUR20072;AGIWUR31018
CB4856	CT	IV	4802702	4806586	WBGene00001058	dpf-5	R11E3.8	-0.137479504051087	AGIWUR4930;AGIWUR6015;AGIWUR20859;AGIWUR21375
CB4856	CT	IV	4806697	4807319	WBGene00044483	H06H21.11	H06H21.11	-0.258810882947504	AGIWUR21364
CB4856	CT	IV	4807426	4809976	WBGene00019163	ubxn-6	H06H21.6	-0.246119438248249	AGIWUR12053;AGIWUR32125;AGIWUR41983;AGIWUR43407
CB4856	CT	IV	4810934	4811983	WBGene00019162	eif-1.A	H06H21.3	-0.219891144571614	AGIWUR17674
CB4856	CT	IV	4812075	4814029	WBGene00005842	srw-95	H06H21.2	0.050048113663852	AGIWUR17290
CB4856	CT	IV	4817388	4821131	WBGene00019164	H06H21.8	H06H21.8	-0.417547344095589	AGIWUR3945;AGIWUR19556
CB4856	CT	IV	4825488	4827281	WBGene00019165	mpz-4	H06H21.9	-0.152457336165888	AGIWUR14973
CB4856	CT	IV	4827853	4846893	WBGene00019166	tat-2	H06H21.10	0.0688462747907472	AGIWUR3450
CB4856	CT	IV	4854394	4856287	WBGene00021195	Y17G9A.2	Y17G9A.2	-0.136761556800611	AGIWUR6330;AGIWUR26489;AGIWUR39776
CB4856	CT	IV	4859255	4860255	WBGene00235375	Y17G9A.91	Y17G9A.91	0.112015708123084	AGIWUR30819
CB4856	CT	IV	4862185	4864141	WBGene00006217	str-173	Y17G9A.1	0.716168420118484	AGIWUR43543

CB4856	CT	IV	4864978	4867659	WBGene00006216	str-172	Y17G9A.5	0.0274494656495232	AGIWUR33017
CB4856	CT	IV	4872267	4874703	WBGene00006218	str-174	Y17G9A.7	-0.180979803679399	AGIWUR26745
CB4856	CT	IV	4877402	4880315	WBGene00006214	str-170	T08B6.7	-0.594617319442462	AGIWUR41481
CB4856	CT	IV	4884296	4886897	WBGene00006211	str-166	T08B6.6	-0.0775723527862704	AGIWUR31779
CB4856	CT	IV	4886885	4887358	WBGene00020351	T08B6.1	T08B6.1	0.0735209547909861	AGIWUR34344
CB4856	CT	IV	4889918	4890672	WBGene00020354	T08B6.5	T08B6.5	-0.0541624809073191	AGIWUR30916
CB4856	CT	IV	4890843	4894085	WBGene00020356	T08B6.9	T08B6.9	0.270466032207871	AGIWUR3587
CB4856	CT	IV	4894370	4897043	WBGene00020352	T08B6.2	T08B6.2	0.0762150774716446	AGIWUR36141
CB4856	CT	IV	4902422	4904061	WBGene00006206	str-161	T08B6.3	-0.475723718957742	AGIWUR22867
CB4856	CT	IV	4905658	4908498	WBGene00020353	T08B6.4	T08B6.4	0.0366748345559742	AGIWUR25964
CB4856	CT	IV	4909339	4910962	WBGene00006204	str-159	Y9C9A.18	0.078032738583659	AGIWUR10470;AGIWUR14777
CB4856	CT	IV	4911320	4912780	WBGene00021184	Y9C9A.17	Y9C9A.17	0.0238949367144645	AGIWUR25700
CB4856	CT	IV	4915989	4917553	WBGene00006201	str-155	Y9C9A.10	-0.0122068273029496	AGIWUR21959
CB4856	CT	IV	4918074	4919632	WBGene00006203	str-158	Y9C9A.9	-0.0909183716403774	AGIWUR11250;AGIWUR15567;AGIWUR22659
CB4856	CT	IV	4930365	4932217	WBGene00021178	Y9C9A.8	Y9C9A.8	0.0210115952213012	AGIWUR14229
CB4856	CT	IV	4934414	4936643	WBGene00006199	str-153	Y9C9A.7	0.120995673526917	AGIWUR15918
CB4856	CT	IV	4936655	4938011	WBGene00021179	fbxa-86	Y9C9A.12	-0.227831074846863	AGIWUR44600
CB4856	CT	IV	4939315	4941506	WBGene00006212	str-168	Y9C9A.6	-0.423562641897993	AGIWUR1074;AGIWUR27267;AGIWUR41906
CB4856	CT	IV	4941981	4944588	WBGene00021177	Y9C9A.5	Y9C9A.5	0.194122072383848	AGIWUR25885
CB4856	CT	IV	4946105	4948518	WBGene00006213	str-169	Y9C9A.4	0.0249371679188446	AGIWUR13121;AGIWUR15955
CB4856	CT	IV	4954187	4958206	WBGene00006208	str-163	Y9C9A.3	0.776531757169852	AGIWUR3410;AGIWUR26106;AGIWUR29004
CB4856	CT	IV	4960735	4963147	WBGene00006209	str-164	Y9C9A.2	-0.141034679759521	AGIWUR26339
CB4856	CT	IV	4963883	4965369	WBGene00021176	Y9C9A.1	Y9C9A.1	0.616765964565225	AGIWUR19033
CB4856	CT	IV	4966097	4969540	WBGene00021180	Y9C9A.13	Y9C9A.13	-0.0866931821238923	AGIWUR32536
CB4856	CT	IV	4969840	4971145	WBGene00021181	srz-27	Y9C9A.14	-0.294197754921704	AGIWUR18744
CB4856	CT	IV	4971671	4973203	WBGene00021182	srz-28	Y9C9A.15	0.053494647622216	AGIWUR11092

CB4856	CT	IV	4973622	4976089	WBGene00021183	Y9C9A.16	Y9C9A.16	-0.106186143447101	AGIWUR10233;AGIWUR14926;AGIWUR24909
CB4856	CT	IV	4977201	4978084	WBGene00017112	E03H12.5	E03H12.5	0.551003630725219	AGIWUR2197;AGIWUR17016;AGIWUR28451;AGIWUR35167;AGIWUR38500
CB4856	CT	IV	4979789	4981127	WBGene00017113	srz-24	E03H12.6	0.0891648913210634	AGIWUR17310;AGIWUR19064;AGIWUR20580
CB4856	CT	IV	4982596	4983892	WBGene00017111	E03H12.4	E03H12.4	-0.0913740459105465	AGIWUR11663
CB4856	CT	IV	4984046	4985375	WBGene00017110	clec-176	E03H12.3	0.650445754427027	AGIWUR27091
CB4856	CT	IV	4986546	4987725	WBGene00017109	clec-177	E03H12.2	0.0811203693882412	AGIWUR12062
CB4856	CT	IV	4987702	4988642	WBGene00017114	E03H12.7	E03H12.7	-0.179836831562181	AGIWUR45032
CB4856	CT	IV	4989065	4989717	WBGene00023265	E03H12.8	E03H12.8	0.0877137556289123	AGIWUR18233;AGIWUR28268;AGIWUR31646;AGIWUR32544;AGIWUR39099
CB4856	CT	IV	4990255	4991433	WBGene00017115	E03H12.9	E03H12.9	-0.0226719626522653	AGIWUR469
CB4856	CT	IV	4992389	4993857	WBGene00006207	str-162	E03H12.1	0.0946923593091968	AGIWUR15331
CB4856	CT	IV	4996142	4997559	WBGene00006051	ssq-2	T28H11.5	0.580899894104792	AGIWUR44466
CB4856	CT	IV	4999434	4999853	WBGene00006040	ssp-11	T28H11.6	-0.0312989520285107	AGIWUR6740
CB4856	CT	IV	5002752	5005418	WBGene00003976	pes-1	T28H11.4	-0.0651031369773598	AGIWUR30072
CB4856	CT	IV	5008678	5010789	WBGene00005639	srm-2	T28H11.3	0.00792212698313222	AGIWUR22655
CB4856	CT	IV	5012549	5014604	WBGene00005638	srm-1	T28H11.2	-0.309773623914369	AGIWUR3163
CB4856	CT	IV	5017989	5020654	WBGene00020906	T28H11.8	T28H11.8	0.0226106719608712	AGIWUR17781
CB4856	CT	IV	5021188	5025746	WBGene00220250	C09B9.85	C09B9.85	-0.0461666018175828	AGIWUR21248;AGIWUR39960
CB4856	CT	IV	5029250	5031046	WBGene00015627	C09B9.2	C09B9.2	0.228295911780108	AGIWUR33878
CB4856	CT	IV	5031596	5033395	WBGene00015629	C09B9.4	C09B9.4	0.139019400315489	AGIWUR41775
CB4856	CT	IV	5037815	5040495	WBGene00015626	C09B9.1	C09B9.1	0.194586557902771	AGIWUR7046;AGIWUR28974;AGIWUR34100
CB4856	CT	IV	5040859	5045485	WBGene00044484	hot-9	C09B9.8	0.0946133925664232	

AGIWUR33253;AGIWUR38760
CB4856	CT	IV	5056973 5061012 WBGene00015630	C09B9.7 C09B9.7 -0.0874180073272441	AGIWUR22172
CB4856	CT	IV	5083303 5086887 WBGene00015330	C02B10.5	C02B10.5	0.0375994700995719
AGIWUR12136;AGIWUR16791;AGIWUR30192
CB4856	CT	IV	5086839 5088883 WBGene00015326	ivd-1	C02B10.1	-0.10840159363 1103
AGIWUR9354;AGIWUR17617;AGIWUR18265;AGIWUR20484;AGIWUR26324;AGIWUR27425;AGIWUR35957;AGIWUR42479
CB4856	CT	IV	5090243 5091043 WBGene00015327	snpn-1	C02B10.2	-0.156299020389046
AGIWUR16347
CB4856	CT	IV	5091586 5092807 WBGene00015329	C02B10.4	C02B10.4	-0.172862878188257
AGIWUR7850;AGIWUR23239;AGIWUR33462
CB4856	CT	IV	5092823 5103279 WBGene00015328	C02B10.3	C02B10.3	-0.087007126985208
AGIWUR17458;AGIWUR21441;AGIWUR22579;AGIWUR29397;AGIWUR32557;AGIWUR37173
CB4856	CT	IV	5111839 5112917 WBGene00014997	AC7.3	AC7.3	-0.214225498496823	AGIWUR5318
CB4856	CT	IV	5112929 5122019 WBGene00006428	tkr-3	AC7.1	-0.372266581784759	AGIWUR3307
CB4856	CT	IV	5127006 5152485 WBGene00004929	soc-2	AC7.2	0.0035507700833346
AGIWUR7562;AGIWUR17527;AGIWUR17969;AGIWUR20865;AGIWUR22829;AGIWUR24095;AGIWUR29132
CB4856	CT	IV	5159427 5160248 WBGene00016057	C24D10.6	C24D10.6	-0.0713273331379618
AGIWUR2577
CB4856	CT	IV	5160522 5161388 WBGene00016055	C24D10.4	C24D10.4	-0.0989701254661206
AGIWUR2286
CB4856	CT	IV	5162223 5162712 WBGene00023243	C24D10.3	C24D10.3	-0.556048522115197
AGIWUR38800
CB4856	CT	IV	5162743 5163074 WBGene00016058	nspd-3	C24D10.7	0.186560706129525
AGIWUR5468
CB4856	CT	IV	5163293 5164351 WBGene00016054	C24D10.2	C24D10.2	-0.0144771314031757
AGIWUR653;AGIWUR1095;AGIWUR1370;AGIWUR2640;AGIWUR5782;AGIWUR12706;AGIWUR23865;AGIWUR26333;AGIWUR30473;AGIWU
R31756;AGIWUR34688
CB4856	CT	IV	5166330 5168427 WBGene00016053	C24D10.1	C24D10.1	-0.146313043509238
AGIWUR4016
CB4856	CT	IV	5173280 5183708 WBGene00002247	lam-1	W03F8.5 -0.0663504648449766
AGIWUR8912;AGIWUR20019;AGIWUR22298;AGIWUR26640;AGIWUR31449;AGIWUR36076;AGIWUR42853;AGIWUR43042
CB4856	CT	IV	5184123 5185240 WBGene00020994	W03F8.4 W03F8.4 -0.219845842175339	AGIWUR5806
CB4856	CT	IV	5185333 5189096 WBGene00020993	W03F8.3 W03F8.3 -0.150472330955253	AGIWUR31727
CB4856	CT	IV	5189042 5195627 WBGene00020997	gpa-18	W03F8.9 0.0132637713602635
AGIWUR32817;AGIWUR45125
CB4856	CT	IV	5204591 5207138 WBGene00006586	tni-4	W03F8.1 0.135653465346858
AGIWUR13736;AGIWUR40275
CB4856	CT	IV	5207109 5209481 WBGene00195093	Y59E9AL.36	Y59E9AL.36	0.0615784342032288
AGIWUR6575;AGIWUR10992

CB4856	CT	IV	5209501	5210690	WBGene00043064	nbet-1	Y59E9AL.7	-0.133496324980516	AGIWUR26374
CB4856	CT	IV	5221284	5222133	WBGene00021995	Y59E9AL.5	Y59E9AL.5	0.168010033861636	AGIWUR35112
CB4856	CT	IV	5223237	5224172	WBGene00044346	Y59E9AL.8	Y59E9AL.8	-0.026418859869084	AGIWUR20183;AGIWUR32714
CB4856	CT	IV	5224899	5225772	WBGene00021996	Y59E9AL.6	Y59E9AL.6	0.366903498871419	AGIWUR7006;AGIWUR23221
CB4856	CT	IV	5228919	5230083	WBGene00021992	thn-6	Y59E9AL.1	-0.243461784425275	AGIWUR7330
CB4856	CT	IV	5230830	5232479	WBGene00021999	thn-5	Y59E9AR.4	0.898979182568701	AGIWUR38274
CB4856	CT	IV	5241364	5241794	WBGene00022002	Y59E9AR.7	Y59E9AR.7	0.187651063581491	AGIWUR31205
CB4856	CT	IV	5262786	5264433	WBGene00018127	F36H12.14	F36H12.14	0.0347623547619453	AGIWUR3951;AGIWUR14943;AGIWUR16218;AGIWUR19616;AGIWUR23777;AGIWUR30541;AGIWUR36641;AGIWUR44470
CB4856	CT	IV	5268049	5278070	WBGene00018130	F36H12.17	F36H12.17	-0.211396597045786	AGIWUR29954;AGIWUR36873;AGIWUR44131
CB4856	CT	IV	5290847	5293689	WBGene00018117	nlp-47	F36H12.1	-0.0258977003810532	AGIWUR9348
CB4856	CT	IV	5295931	5297487	WBGene00022707	ZK354.6	ZK354.6	0.0358182659477517	AGIWUR43447
CB4856	CT	IV	5302141	5303078	WBGene00022708	ZK354.7	ZK354.7	-0.186285302743979	AGIWUR14632
CB4856	CT	IV	5304021	5306140	WBGene00022709	ZK354.8	ZK354.8	-0.0551709779715956	AGIWUR16814
CB4856	CT	IV	5310042	5311445	WBGene00022706	ZK354.3	ZK354.3	-0.330240877431093	AGIWUR4701
CB4856	CT	IV	5313405	5315525	WBGene00022705	ZK354.2	ZK354.2	-0.0466124549753633	AGIWUR1753;AGIWUR19004
CB4856	CT	IV	5319253	5325033	WBGene00021159	Y4C6B.5	Y4C6B.5	-0.231486925051669	AGIWUR27808
CB4856	CT	IV	5326444	5329640	WBGene00021158	Y4C6B.4	Y4C6B.4	0.467432889298407	AGIWUR21358;AGIWUR34632
CB4856	CT	IV	5332478	5336827	WBGene00021157	Y4C6B.3	Y4C6B.3	0.193366855679733	AGIWUR25646
CB4856	CT	IV	5340890	5344204	WBGene00021156	Y4C6B.2	Y4C6B.2	0.68946596102214	AGIWUR21116
CB4856	CT	IV	5344592	5350947	WBGene00021160	gba-4	Y4C6B.6	-0.187216513958623	AGIWUR20362;AGIWUR23005;AGIWUR28435;AGIWUR39463
CB4856	CT	IV	5351420	5353464	WBGene00021161	Y4C6B.7	Y4C6B.7	-0.134785485037308	AGIWUR1244;AGIWUR12051;AGIWUR17871;AGIWUR26058;AGIWUR41428;AGIWUR41479
CB4856	CT	IV	5354488	5356539	WBGene00021155	Y4C6B.1	Y4C6B.1	0.0392200512318048	AGIWUR23550
CB4856	CT	IV	5356538	5364306	WBGene00018319	hda-6	F41H10.6	-0.0632103740809278	AGIWUR3721;AGIWUR16258;AGIWUR17382;AGIWUR27562
CB4856	CT	IV	5364559	5366751	WBGene00018317	F41H10.4	F41H10.4	-0.172821696652514	

AGIWUR8752
CB4856 CT IV 5366867 5368866 WBGene00018321 sand-1 F41H10.11 0.10303766271128
AGIWUR18778;AGIWUR36540;AGIWUR44444
CB4856 CT IV 5369797 5374673 WBGene00018316 F41H10.3 F41H10.3 -0.0513705211892783
AGIWUR28505;AGIWUR32473;AGIWUR39991;AGIWUR42589
CB4856 CT IV 5374598 5377275 WBGene00001243 elo-5 F41H10.7 -0.203710831442935
AGIWUR38049
CB4856 CT IV 5381634 5382470 WBGene00018315 F41H10.2 F41H10.2
0.000794904779800376 AGIWUR10409
CB4856 CT IV 5385320 5386080 WBGene00018322 F41H10.12 F41H10.12 -0.0161578257868538
AGIWUR27702
CB4856 CT IV 5386629 5388559 WBGene00001244 elo-6 F41H10.8 -0.129164881115928
AGIWUR12198;AGIWUR38050
CB4856 CT IV 5390184 5391557 WBGene00002032 htp-1 F41H10.10 -0.243682778736765
AGIWUR17040
CB4856 CT IV 5391912 5393572 WBGene00022775 ZK616.5 ZK616.5 -0.085051155230321 AGIWUR17545
CB4856 CT IV 5393843 5394849 WBGene00022776 perm-3 ZK616.6 -0.106439580596748
AGIWUR3289;AGIWUR10280;AGIWUR14230;AGIWUR37025;AGIWUR37266;AGIWUR42327
CB4856 CT IV 5394962 5396966 WBGene00022774 swsn-6 ZK616.4 -0.0137799168398516 AGIWUR19887
CB4856 CT IV 5397069 5397694 WBGene00022773 ZK616.3 ZK616.3 -0.287361227937637 AGIWUR16868
CB4856 CT IV 5397839 5398591 WBGene00022772 ZK616.2 ZK616.2 0.166117610854851 AGIWUR16916
CB4856 CT IV 5405645 5407375 WBGene00194816 ZK616.61 ZK616.61 0.14931984383988
AGIWUR8298
CB4856 CT IV 5408426 5411166 WBGene00235383 ZK616.65 ZK616.65 -0.161272857788781
AGIWUR31061
CB4856 CT IV 5412827 5415771 WBGene00004992 spp-7 ZK616.9 1.09452271635818 AGIWUR2110
CB4856 CT IV 5416906 5419195 WBGene00001223 ehn-3 ZK616.10 0.261224093630749
AGIWUR26234
CB4856 CT IV 5423576 5450802 WBGene00021152 mgl-3 Y4C6A.2 0.0642378206555061
AGIWUR24178;AGIWUR32618
CB4856 CT IV 5450993 5452455 WBGene00021153 Y4C6A.3 Y4C6A.3 0.0682785744739345 AGIWUR10331
CB4856 CT IV 5452967 5453640 WBGene00021154 Y4C6A.4 Y4C6A.4 -0.427405568207975 AGIWUR8178
CB4856 CT IV 5458767 5460735 WBGene00021151 Y4C6A.1 Y4C6A.1 0.0735654914858115
AGIWUR37588;AGIWUR42972
CB4856 CT IV 5462501 5464448 WBGene00021793 Y52D5A.2 Y52D5A.2
0.000237172183289225 AGIWUR31886
CB4856 CT IV 5467393 5469134 WBGene00021792 Y52D5A.1 Y52D5A.1 -0.307806859462385
AGIWUR17822
CB4856 CT IV 5469961 5473174 WBGene00004374 rme-2 T11F8.3 -0.110836033489193 AGIWUR29033

CB4856	CT	IV	5474162 5476568 WBGene00020435	T11F8.4 T11F8.4 -0.0668128917187216	AGIWUR40747
CB4856	CT	IV	5477214 5478708 WBGene00020434	T11F8.2 T11F8.2 -0.21273116615803
AGIWUR8500;AGIWUR20411;AGIWUR27745;AGIWUR38023
CB4856	CT	IV	5479589 5480644 WBGene00020433	T11F8.1 T11F8.1 -0.18097967437085	AGIWUR14895
CB4856	CT	IV	5493992 5495252 WBGene00015097	B0273.1 B0273.1 0.355949762286251	AGIWUR31928
CB4856	CT	IV	5495666 5528338 WBGene00006745	unc-5	B0273.4 0.532472084734025	AGIWUR31044
CB4856	CT	IV	5533142 5537992 WBGene00001411	fem-1	F35D6.1 -0.121215971419026
AGIWUR3074;AGIWUR5563;AGIWUR31665;AGIWUR31934;AGIWUR33987;AGIWUR37509;AGIWUR44674
CB4856	CT	IV	5537981 5541365 WBGene00001093	drp-1	T12E12.4	-0.119743148909495
AGIWUR7638;AGIWUR8632;AGIWUR21497;AGIWUR26100;AGIWUR26830;AGIWUR27798
CB4856	CT	IV	5541607 5544765 WBGene00020464	T12E12.3	T12E12.3	-0.24557434492559
AGIWUR16529
CB4856	CT	IV	5544860 5548459 WBGene00020463	cec-6	T12E12.2	-0.017970601566029
AGIWUR35802
CB4856	CT	IV	5551134 5558820 WBGene00020462	T12E12.1	T12E12.1	-0.0965543614307727
AGIWUR4873;AGIWUR8315;AGIWUR18358;AGIWUR20163;AGIWUR28072;AGIWUR31360
CB4856	CT	IV	5567020 5574133 WBGene00020465	T12E12.6	T12E12.6	0.0122756141454339
AGIWUR22199;AGIWUR35240;AGIWUR35383
CB4856	CT	IV	5584661 5610368 WBGene00001439	fkh-7	F26D12.1	0.743226866126284
AGIWUR21093;AGIWUR25106;AGIWUR27035;AGIWUR32201;AGIWUR38013;AGIWUR38887;AGIWUR42087
CB4856	CT	IV	5611950 5614608 WBGene00016542	C39H7.4 C39H7.4 -0.0525847654254097	AGIWUR23749
CB4856	CT	IV	5615522 5619685 WBGene00044125	srsx-16 C39H7.9 0.208357713470819
AGIWUR32633;AGIWUR34370
CB4856	CT	IV	5622063 5623818 WBGene00044124	srsx-15 C39H7.8 0.126116613587701
AGIWUR25141;AGIWUR35563;AGIWUR45008
CB4856	CT	IV	5624855 5627156 WBGene00016543	srsx-14 C39H7.5 0.172358725148361	AGIWUR22984
CB4856	CT	IV	5628792 5630239 WBGene00005090	srd-12	C39H7.6 -0.074647158135865	AGIWUR14655
CB4856	CT	IV	5633818 5635651 WBGene00005091	srd-13	C39H7.7 0.152853262920009	AGIWUR33901
CB4856	CT	IV	5637835 5640190 WBGene00005092	srd-14	B0547.3 -0.602689679819409
AGIWUR37068;AGIWUR42849
CB4856	CT	IV	5643252 5645614 WBGene00005086	srd-8	B0547.4 0.235061637398448	AGIWUR13315
CB4856	CT	IV	5645831 5646233 WBGene00015253	B0547.2 B0547.2 -0.151059113049037	AGIWUR4167
CB4856	CT	IV	5647432 5648910 WBGene00005084	srd-6	B0547.5 -0.0639044188286606
AGIWUR15071;AGIWUR24916
CB4856	CT	IV	5649533 5651079 WBGene00000817	csn-5	B0547.1 -0.0366393407660377
AGIWUR15023;AGIWUR26838;AGIWUR30038;AGIWUR44781
CB4856	CT	IV	5651087 5660523 WBGene00004804	skn-1	T19E7.2 -0.119651931610462
AGIWUR1037;AGIWUR1729;AGIWUR7018;AGIWUR30483
CB4856	CT	IV	5661141 5664425 WBGene00000247	bec-1	T19E7.3 -0.0419861566498128	AGIWUR28646

CB4856	CT	IV	5667525	5669583	WBGene00044500	T19E7.6	T19E7.6	0.899602333723385	AGIWUR1961;AGIWUR7360;AGIWUR13107;AGIWUR18108;AGIWUR28425;AGIWUR31204;AGIWUR37038
CB4856	CT	IV	5672633	5674542	WBGene00005083	srd-5	T19E7.5	-0.251870941375832	AGIWUR18363;AGIWUR21112;AGIWUR40940
CB4856	CT	IV	5676212	5678144	WBGene00016954	C55C3.4	C55C3.4	-0.154014646438714	AGIWUR31025
CB4856	CT	IV	5678847	5682639	WBGene00043065	C55C3.7	C55C3.7	-0.184332029965662	AGIWUR20678;AGIWUR31895;AGIWUR44145
CB4856	CT	IV	5683184	5684672	WBGene00016956	C55C3.6	C55C3.6	-0.147745105276171	AGIWUR3247;AGIWUR44402
CB4856	CT	IV	5685287	5688273	WBGene00016953	C55C3.3	C55C3.3	0.0524409961948038	AGIWUR31711
CB4856	CT	IV	5691030	5694646	WBGene00044501	C55C3.8	C55C3.8	-0.000668872458147618	AGIWUR11565;AGIWUR14365;AGIWUR41535
CB4856	CT	IV	5695777	5703468	WBGene00016955	perm-5	C55C3.5	0.117252403356013	AGIWUR2558;AGIWUR12926;AGIWUR13869
CB4856	CT	IV	5709096	5714336	WBGene00016951	C55C3.1	C55C3.1	-0.116517842350488	AGIWUR33858
CB4856	CT	IV	5725600	5729727	WBGene00019263	H35B03.1	H35B03.1	0.0939692130652467	AGIWUR12168;AGIWUR15462;AGIWUR16089;AGIWUR21515;AGIWUR42466
CB4856	CT	IV	5729658	5732636	WBGene00019264	H35B03.2	H35B03.2	-0.189799913189248	AGIWUR20162;AGIWUR24408;AGIWUR43047;AGIWUR44813
CB4856	CT	IV	5735019	5737106	WBGene00016667	C45E5.4	C45E5.4	-0.106326408236479	AGIWUR28686
CB4856	CT	IV	5744336	5747110	WBGene00016664	C45E5.1	C45E5.1	0.0323932393102258	AGIWUR3135;AGIWUR7956;AGIWUR37449
CB4856	CT	IV	5747376	5757862	WBGene00003636	nhr-46	C45E5.6	0.0581999852996447	AGIWUR3975;AGIWUR15663;AGIWUR18429;AGIWUR27946;AGIWUR29904
CB4856	CT	IV	5791296	5793457	WBGene00016287	C31H1.2	C31H1.2	-0.0275352547374981	AGIWUR7772;AGIWUR9298;AGIWUR24041
CB4856	CT	IV	5794932	5796825	WBGene00016288	C31H1.5	C31H1.5	0.175471381115827	AGIWUR15801
CB4856	CT	IV	5797453	5800233	WBGene00016286	C31H1.1	C31H1.1	0.0842026475747284	AGIWUR44740
CB4856	CT	IV	5800336	5807093	WBGene00016289	lntl-1	C31H1.6	0.0106915844745137	AGIWUR1036;AGIWUR26901
CB4856	CT	IV	5811583	5814142	WBGene00016291	C31H1.8	C31H1.8	-0.111183093901218	AGIWUR28224
CB4856	CT	IV	5820370	5830199	WBGene00015680	egal-1	C10G6.1	0.0468603128304386	AGIWUR11282;AGIWUR38980
CB4856	CT	IV	5839387	5844004	WBGene00015539	C06E7.2	C06E7.2	-0.166979822332923	AGIWUR16132
CB4856	CT	IV	5843890	5846398	WBGene00015540	sams-4	C06E7.3	0.157335176263674	AGIWUR33922
CB4856	CT	IV	5848657	5850845	WBGene00015538	sams-3	C06E7.1	0.22169130704852	AGIWUR8961
CB4856	CT	IV	5850826	5859976	WBGene00015541	C06E7.4	C06E7.4	0.247729867853943	AGIWUR4608
CB4856	CT	IV	5864529	5865918	WBGene00004973	spe-27	C06E7.6	-0.105088816466942	AGIWUR7261
CB4856	CT	IV	5867689	5870172	WBGene00005728	srv-17	C06E7.7	-0.651838628070667	AGIWUR11136

CB4856	CT	IV	5871143 5871737 WBGene00005729	srv-18	H04M03.5	0.0493294860821605
AGIWUR72;AGIWUR9244;AGIWUR19701;AGIWUR19952;AGIWUR27219
CB4856	CT	IV	5871976 5874081 WBGene00005730	srv-19	H04M03.6	0.330046799245582
AGIWUR30456
CB4856	CT	IV	5874559 5875659 WBGene00005731	srv-20	H04M03.7	-0.0318280485201434
AGIWUR1978;AGIWUR4326;AGIWUR7692;AGIWUR11512;AGIWUR32527
CB4856	CT	IV	5876092 5877346 WBGene00005732	srv-21	H04M03.8	0.0399843715895791
AGIWUR15833
CB4856	CT	IV	5878470 5879953 WBGene00005733	srv-22	H04M03.9	0.126976525402885
AGIWUR31893
CB4856	CT	IV	5882707 5884529 WBGene00019155	H04M03.11	H04M03.11	-0.307808032950236
AGIWUR17441
CB4856	CT	IV	5885835 5889821 WBGene00019154	glf-1	H04M03.4	0.627315534291863
AGIWUR44615
CB4856	CT	IV	5891565 5893329 WBGene00019153	H04M03.3	H04M03.3	-0.236646684727841
AGIWUR27890
CB4856	CT	IV	5895490 5895888 WBGene00019152	nspb-6	H04M03.2	-0.315033838552571
AGIWUR11992
CB4856	CT	IV	5896623 5899112 WBGene00019151	pck-3	H04M03.1	0.0837180777417839
AGIWUR8619
CB4856	CT	IV	5899586 5903355 WBGene00019156	H04M03.12	H04M03.12	0.419529362839125
AGIWUR14194
CB4856	CT	IV	5917326 5921446 WBGene00019257	dhhc-13 H32C10.3	0.0201988491689811
AGIWUR33658
CB4856	CT	IV	5930645 5933326 WBGene00003019	lin-33	H32C10.2	-0.0693030921218583
AGIWUR31530
CB4856	CT	IV	5934346 5937497 WBGene00019255	H32C10.1	H32C10.1	-0.0164873269908812
AGIWUR5965;AGIWUR20503;AGIWUR32526
CB4856	CT	IV	5937445 5939230 WBGene00019296	mcu-1	K02B2.3 0.0400179524613691
AGIWUR5511;AGIWUR16477;AGIWUR17572
CB4856	CT	IV	5941926 5943998 WBGene00019295	pfkb-1.2	K02B2.1 0.00907705879686826
AGIWUR3026;AGIWUR7954
CB4856	CT	IV	5943981 5946754 WBGene00002129	inx-7	K02B2.4 0.102597703336542	AGIWUR7168
CB4856	CT	IV	5947036 5947607 WBGene00004494	rps-25	K02B2.5 0.0903491187640224
AGIWUR37136;AGIWUR39980
CB4856	CT	IV	5961690 5962132 WBGene00019297	K02B2.6 K02B2.6 0.225845553067778	AGIWUR24823
CB4856	CT	IV	5966656 6004857 WBGene00006780	unc-44	B0350.2 0.323122073695817
AGIWUR7735;AGIWUR8509;AGIWUR10238;AGIWUR12013;AGIWUR12342;AGIWUR14441;AGIWUR17621;AGIWUR19110;AGIWUR25248;A
GIWUR27550;AGIWUR27667;AGIWUR29096;AGIWUR32226;AGIWUR35643;AGIWUR38125;AGIWUR39293;AGIWUR44581

CB4856	CT	IV	6010352	6011645	WBGene00044502	C46G7.5	C46G7.5	0.197404868440445	AGIWUR17759
CB4856	CT	IV	6015195	6017803	WBGene00016722	C46G7.2	C46G7.2	-0.0957461367121084	AGIWUR4371
CB4856	CT	IV	6018003	6019713	WBGene00016721	C46G7.1	C46G7.1	0.0534271606181651	AGIWUR8440
CB4856	CT	IV	6019705	6033639	WBGene00004112	pqn-22	C46G7.4	-0.197131297779372	AGIWUR19188
CB4856	CT	IV	6040976	6074507	WBGene00019205	kcc-2	H16O14.1	0.129192194936153	AGIWUR1911;AGIWUR2231;AGIWUR5271;AGIWUR6446;AGIWUR7025;AGIWUR8507;AGIWUR13439;AGIWUR13842;AGIWUR14139;AGIWUR15302;AGIWUR17542;AGIWUR19667;AGIWUR21985;AGIWUR26651;AGIWUR27097;AGIWUR28020;AGIWUR29762;AGIWUR34185;AGIWUR42611;AGIWUR42648
CB4856	CT	IV	6075301	6076157	WBGene00019514	K08B4.2	K08B4.2	0.973814105283866	AGIWUR42732
CB4856	CT	IV	6076692	6079945	WBGene00019515	ugt-19	K08B4.3	-0.679262501548569	AGIWUR11886
CB4856	CT	IV	6080907	6083592	WBGene00019516	ugt-20	K08B4.4	-0.428406885797084	AGIWUR914
CB4856	CT	IV	6086350	6089404	WBGene00019517	K08B4.5	K08B4.5	0.0673509849119149	AGIWUR15458
CB4856	CT	IV	6091536	6092413	WBGene00023486	K08B4.7	K08B4.7	-0.501405609024024	AGIWUR5558;AGIWUR16663;AGIWUR19385
CB4856	CT	IV	6095411	6110639	WBGene00002245	lag-1	K08B4.1	0.100475195851597	AGIWUR1113;AGIWUR12391;AGIWUR34040
CB4856	CT	IV	6110651	6112391	WBGene00019750	M03D4.3	M03D4.3	0.668534703101926	AGIWUR34204
CB4856	CT	IV	6118219	6121775	WBGene00006974	zen-4	M03D4.1	-0.0517214274463527	AGIWUR6721;AGIWUR7368;AGIWUR10759;AGIWUR19300;AGIWUR31924
CB4856	CT	IV	6143183	6149923	WBGene00019176	H10D12.2	H10D12.2	0.199727241176793	AGIWUR2783;AGIWUR6943;AGIWUR11080;AGIWUR17501;AGIWUR23031;AGIWUR44002
CB4856	CT	IV	6152437	6154623	WBGene00015701	C11D2.3	C11D2.3	-0.0273905323328929	AGIWUR2737
CB4856	CT	IV	6154558	6155396	WBGene00015703	C11D2.7	C11D2.7	-0.0493080493418763	AGIWUR26177
CB4856	CT	IV	6155492	6156960	WBGene00015702	C11D2.4	C11D2.4	0.00514565406818246	AGIWUR9202;AGIWUR10951;AGIWUR18099;AGIWUR20572
CB4856	CT	IV	6157310	6183857	WBGene00006809	unc-77	C11D2.6	-0.0801032604924705	AGIWUR18031;AGIWUR29623;AGIWUR40642;AGIWUR43664
CB4856	CT	IV	6185888	6189205	WBGene00015700	asp-9	C11D2.2	0.229217487987194	AGIWUR3747;AGIWUR12177
CB4856	CT	IV	6202080	6205703	WBGene00016761	tkr-2	C49A9.7	0.0458997301731028	AGIWUR14708
CB4856	CT	IV	6205793	6206344	WBGene00016764	C49A9.10	C49A9.10	-0.327870124758235	AGIWUR23758
CB4856	CT	IV	6206843	6209527	WBGene00016762	ugt-24	C49A9.8	0.0789693756042027	AGIWUR14772
CB4856	CT	IV	6210936	6213181	WBGene00016760	C49A9.6	C49A9.6	-0.427472997699336	AGIWUR38078
CB4856	CT	IV	6213822	6216371	WBGene00016759	C49A9.5	C49A9.5	-0.48395853174223	AGIWUR8070
CB4856	CT	IV	6217349	6219449	WBGene00016763	C49A9.9	C49A9.9	-0.0252334941278232	AGIWUR1773;AGIWUR15196
CB4856	CT	IV	6219604	6222346	WBGene00016758	C49A9.4	C49A9.4	-0.0706269053115225	AGIWUR12726
CB4856	CT	IV	6222089	6225009	WBGene00016757	C49A9.3	C49A9.3	0.0761358118414055	AGIWUR37712

CB4856	CT	IV	6225443	6228046	WBGene00016756	C49A9.2	C49A9.2	-0.531547885462285	AGIWUR23748
CB4856	CT	IV	6228203	6230025	WBGene00016755	C49A9.1	C49A9.1	-0.0851960201384969	AGIWUR3139
CB4856	CT	IV	6231023	6232607	WBGene00019058	F58F9.4	F58F9.4	-0.392820706186345	AGIWUR41348
CB4856	CT	IV	6233246	6235666	WBGene00019057	F58F9.3	F58F9.3	-0.523775272549802	AGIWUR7538;AGIWUR20237
CB4856	CT	IV	6235977	6237543	WBGene00019059	F58F9.6	F58F9.6	0.18086089230101	AGIWUR19018
CB4856	CT	IV	6238192	6239437	WBGene00044306	F58F9.8	F58F9.8	-0.0702979092342981	AGIWUR9116;AGIWUR24570
CB4856	CT	IV	6240306	6241389	WBGene00044307	F58F9.9	F58F9.9	0.0231089374300506	AGIWUR18241;AGIWUR29280
CB4856	CT	IV	6241855	6242795	WBGene00044308	F58F9.10	F58F9.10	0.0639187002849444	AGIWUR26484;AGIWUR38991
CB4856	CT	IV	6243382	6244374	WBGene00044309	F58F9.11	F58F9.11	-0.136489110484063	AGIWUR17345
CB4856	CT	IV	6245341	6248492	WBGene00019060	acox-3	F58F9.7	0.487581328493871	AGIWUR24700
CB4856	CT	IV	6249874	6253083	WBGene00019055	F58F9.1	F58F9.1	0.113852458901037	AGIWUR12407
CB4856	CT	IV	6255071	6259858	WBGene00003754	nlp-16	T13A10.5	0.0570241300494096	AGIWUR21097;AGIWUR24636
CB4856	CT	IV	6261058	6263344	WBGene00005739	srv-28	T13A10.14	0.266360135710158	AGIWUR7801
CB4856	CT	IV	6267457	6269446	WBGene00005741	srv-30	T13A10.7	0.129874604315797	AGIWUR37058
CB4856	CT	IV	6269773	6272023	WBGene00005742	srv-31	T13A10.9	-0.114043694548509	AGIWUR40378
CB4856	CT	IV	6272529	6275713	WBGene00000005	aat-4	T13A10.10	-0.495837584181555	AGIWUR15818
CB4856	CT	IV	6277435	6279572	WBGene00006416	sams-5	T13A10.11	0.00863266769109217	AGIWUR3761;AGIWUR11823;AGIWUR19459;AGIWUR20660;AGIWUR41960
CB4856	CT	IV	6280147	6282243	WBGene00005743	srv-32	T13A10.12	-0.159655821371558	AGIWUR18255
CB4856	CT	IV	6282931	6284387	WBGene00005744	ops-1	T13A10.13	0.228050169256726	AGIWUR33785
CB4856	CT	IV	6288364	6290841	WBGene00005735	srv-24	Y73B6BL.10	0.298066808839971	AGIWUR42294
CB4856	CT	IV	6294234	6295219	WBGene00001853	hil-2	Y73B6BL.9	0.24117896952475	AGIWUR18626
CB4856	CT	IV	6296279	6298347	WBGene00005738	srv-27	Y73B6BL.41	0.0270299764285138	AGIWUR7755;AGIWUR37612;AGIWUR39406
CB4856	CT	IV	6299273	6301864	WBGene00005737	srv-26	Y73B6BL.40	0.172988954849788	

AGIWUR22999									
CB4856	CT	IV	6302159	6303742	WBGene00005736	srv-25	Y73B6BL.11	0.0206768104049058	
AGIWUR2146									
CB4856	CT	IV	6307739	6315719	WBGene00000820	csp-2	Y73B6BL.7	0.00643845406829469	
AGIWUR27137;AGIWUR37601									
CB4856	CT	IV	6321948	6323562	WBGene00022250	Y73B6BL.29	Y73B6BL.29	-0.24251793053759	
AGIWUR43135									
CB4856	CT	IV	6323979	6326421	WBGene00022235	sqd-1	Y73B6BL.6	-0.232794210688086	
AGIWUR5530;AGIWUR6062;AGIWUR6714;AGIWUR9941;AGIWUR10169;AGIWUR30227									
CB4856	CT	IV	6326526	6329461	WBGene00003082	lsm-8	Y73B6BL.32	-0.235544179548892	
AGIWUR39819									
CB4856	CT	IV	6329578	6333554	WBGene00004783	seu-1	Y73B6BL.5	-0.0445895563734435	
AGIWUR2395;AGIWUR9149;AGIWUR33136;AGIWUR35630;AGIWUR41727									
CB4856	CT	IV	6334215	6335168	WBGene00022251	blos-2	Y73B6BL.30	-0.027072687833142	
AGIWUR34101									
CB4856	CT	IV	6335249	6335994	WBGene00003237	mif-4	Y73B6BL.13	0.0850182251189793	
AGIWUR43599									
CB4856	CT	IV	6337653	6340098	WBGene00022233	ipla-6	Y73B6BL.4	0.0583929090581057	
AGIWUR9142;AGIWUR19157;AGIWUR25759;AGIWUR35569									
CB4856	CT	IV	6342471	6342976	WBGene00022237	Y73B6BL.14	Y73B6BL.14	-0.330464358432913	
AGIWUR15623									
CB4856	CT	IV	6373162	6376523	WBGene00022253	hrpf-2	Y73B6BL.33	-0.0758741585132832	
AGIWUR15470									
CB4856	CT	IV	6377065	6378195	WBGene00000256	bli-6	Y73B6BL.34	-0.44801685535904	
AGIWUR43023									
CB4856	CT	IV	6378594	6384070	WBGene00022240	shl-1	Y73B6BL.19	-0.317222291566985	
AGIWUR18216									
CB4856	CT	IV	6396582	6402458	WBGene00022242	sfrp-1	Y73B6BL.21	0.172826014964162	
AGIWUR5050									
CB4856	CT	IV	6405176	6408236	WBGene00005724	srv-13	Y73B6BL.39	0.0268374166899239	
AGIWUR3601									
CB4856	CT	IV	6409557	6413566	WBGene00022254	Y73B6BL.35	Y73B6BL.35	0.298809923575974	
AGIWUR21204;AGIWUR23050;AGIWUR23340;AGIWUR26890;AGIWUR29462;AGIWUR40052									
CB4856	CT	IV	6415478	6417457	WBGene00022232	exos-2	Y73B6BL.3	0.129381257198201	
AGIWUR10655									
CB4856	CT	IV	6417696	6419078	WBGene00022244	Y73B6BL.23	Y73B6BL.23	0.0535145860471982	
AGIWUR3816;AGIWUR6155;AGIWUR11951;AGIWUR28586;AGIWUR40745									
CB4856	CT	IV	6419500	6420898	WBGene00002033	htp-2	Y73B6BL.2	0.0311553618529175	
AGIWUR10934									

CB4856	CT	IV	6421036	6421200	WBGene00044752	Y73B6BL.45	Y73B6BL.45	0.623688950910553	AGIWUR9734
CB4856	CT	IV	6421589	6423489	WBGene00022257	puf-11	Y73B6BL.38	-0.0390249267922478	AGIWUR28069
CB4856	CT	IV	6424600	6426139	WBGene00022245	acp-6	Y73B6BL.24	-0.456863950044591	AGIWUR5531;AGIWUR20296;AGIWUR22193
CB4856	CT	IV	6428305	6431707	WBGene00022246	acp-7	Y73B6BL.25	-0.290102757418312	AGIWUR31201
CB4856	CT	IV	6438223	6440032	WBGene00043067	dlc-5	Y73B6BL.43	-0.0575980789371968	AGIWUR5344;AGIWUR7316;AGIWUR16502;AGIWUR29630
CB4856	CT	IV	6442229	6446532	WBGene00022252	Y73B6BL.31	Y73B6BL.31	0.042158017091495	AGIWUR10068;AGIWUR14224;AGIWUR30990
CB4856	CT	IV	6446573	6457543	WBGene00022247	lgc-10	Y73B6BL.26	-0.0514839302708845	AGIWUR18076
CB4856	CT	IV	6464352	6468327	WBGene00043066	acr-25	Y73B6BL.42	-0.244567141609511	AGIWUR38243
CB4856	CT	IV	6469955	6471384	WBGene00022255	Y73B6BL.36	Y73B6BL.36	0.526682218937883	AGIWUR11423;AGIWUR14886
CB4856	CT	IV	6472155	6473235	WBGene00022248	Y73B6BL.27	Y73B6BL.27	-0.240689686998826	AGIWUR42102
CB4856	CT	IV	6480521	6486971	WBGene00022231	tyr-6	Y73B6BL.1	-0.239553810487177	AGIWUR1895
CB4856	CT	IV	6487347	6489090	WBGene00022256	Y73B6BL.37	Y73B6BL.37	0.684423600418408	AGIWUR12748
CB4856	CT	IV	6493599	6497378	WBGene00044350	Y73B6BL.44	Y73B6BL.44	0.159088744915412	AGIWUR1321;AGIWUR8689
CB4856	CT	IV	6506726	6511042	WBGene00004169	pqn-89	Y73B6BR.1	0.222717306351457	AGIWUR2166;AGIWUR18201;AGIWUR33782;AGIWUR35889;AGIWUR41226
CB4856	CT	IV	6518174	6519502	WBGene00015309	C01G5.7	C01G5.7	0.00849533051939755	AGIWUR30933
CB4856	CT	IV	6519926	6522242	WBGene00015311	C01G5.9	C01G5.9	-0.233713152362228	AGIWUR16070
CB4856	CT	IV	6522874	6524568	WBGene00015308	ddi-1	C01G5.6	-0.411804689586779	AGIWUR695
CB4856	CT	IV	6524610	6526524	WBGene00015307	C01G5.5	C01G5.5	0.0540641457814051	AGIWUR13057
CB4856	CT	IV	6526729	6529802	WBGene00004179	prg-2	C01G5.2	0.0272604547095074	AGIWUR19766;AGIWUR25037;AGIWUR35662
CB4856	CT	IV	6531750	6535677	WBGene00015306	C01G5.4	C01G5.4	0.377669377577843	AGIWUR12939
CB4856	CT	IV	6548818	6552511	WBGene00015310	fan-1	C01G5.8	-0.0985267176034892	AGIWUR30930
CB4856	CT	IV	6552479	6555920	WBGene00016610	paqr-1	C43G2.1	0.113801433728209	AGIWUR6337;AGIWUR20140;AGIWUR39059
CB4856	CT	IV	6557171	6560881	WBGene00006621	try-3	C43G2.5	-0.491630271005654	AGIWUR22974

CB4856 CT IV 6560736 6565261 WBGene00016613 best-9 C43G2.4 -0.256894088532795 AGIWUR27424
CB4856 CT IV 6565384 6572338 WBGene00016611 bicd-1 C43G2.2 -0.169260368416452 AGIWUR13162
CB4856 CT IV 6578197 6579402 WBGene00016612 C43G2.3 C43G2.3 -0.083406926424539 AGIWUR21928
CB4856 CT IV 6579840 6582676 WBGene00018160 odr-8 F38A5.1 -0.0913852040577918
AGIWUR2601;AGIWUR3004;AGIWUR4843;AGIWUR31954;AGIWUR33472;AGIWUR34492;AGIWUR44384
CB4856 CT IV 6582703 6585571 WBGene00018161 F38A5.2 F38A5.2 0.0805925893485711
AGIWUR1637;AGIWUR1880;AGIWUR26971;AGIWUR31257;AGIWUR33703;AGIWUR41277;AGIWUR42545
CB4856 CT IV 6585871 6588186 WBGene00001029 dnj-11 F38A5.13 -0.152171222798669
AGIWUR26044;AGIWUR32851;AGIWUR33015;AGIWUR41606
CB4856 CT IV 6588184 6590307 WBGene00002274 lec-11 F38A5.3 -0.0201401313586675
AGIWUR6492;AGIWUR16105;AGIWUR16475;AGIWUR27350
CB4856 CT IV 6592666 6594529 WBGene00018168 irld-7 F38A5.11 -0.334479023590408
AGIWUR3776
CB4856 CT IV 6596108 6597398 WBGene00018163 F38A5.6 F38A5.6 0.0972602713792199 AGIWUR30197
CB4856 CT IV 6599882 6600491 WBGene00018165 F38A5.8 F38A5.8 -0.269077577660956 AGIWUR19468
CB4856 CT IV 6601678 6602443 WBGene00018164 sup-36 F38A5.7 0.592657280267902 AGIWUR35277
CB4856 CT IV 6602680 6603966 WBGene00017481 F15B10.3 F15B10.3 0.486651050673022
AGIWUR29802
CB4856 CT IV 6604001 6606188 WBGene00017480 nstp-2 F15B10.1 -0.193627179522833
AGIWUR9501
CB4856 CT IV 6607376 6613353 WBGene00001090 drh-1 F15B10.2 -0.200767868562894
AGIWUR1034;AGIWUR9440;AGIWUR42915
CB4856 CT IV 6613918 6618498 WBGene00001091 drh-2 C01B10.1 -0.523531780131787
AGIWUR7926;AGIWUR18407
CB4856 CT IV 6619468 6621352 WBGene00015278 C01B10.3 C01B10.3 -0.278517619637618
AGIWUR34609
CB4856 CT IV 6624528 6627992 WBGene00015284 C01B10.10 C01B10.10 -0.0934431004684648
AGIWUR10607;AGIWUR34060
CB4856 CT IV 6628366 6631745 WBGene00015279 C01B10.4 C01B10.4 -0.336079376505992
AGIWUR31155
CB4856 CT IV 6632222 6634986 WBGene00015283 C01B10.9 C01B10.9 -0.188598857116281
AGIWUR27315
CB4856 CT IV 6635039 6638155 WBGene00015282 C01B10.8 C01B10.8 -0.104447514484214
AGIWUR40431
CB4856 CT IV 6638556 6639271 WBGene00015281 C01B10.7 C01B10.7 -0.501415400768341
AGIWUR44495
CB4856 CT IV 6640161 6645352 WBGene00015280 C01B10.6 C01B10.6 0.0105142358498931
AGIWUR35272;AGIWUR40404;AGIWUR42883
CB4856 CT IV 6646034 6646936 WBGene00001858 hil-7 C01B10.5 1.64115313901484

AGIWUR1319;AGIWUR11770;AGIWUR16609;AGIWUR22740;AGIWUR24965;AGIWUR25043;AGIWUR29174;AGIWUR31187;AGIWUR40020;AGIWUR42223
CB4856 CT IV 6657981 6660434 WBGene00044294 C01B10.11 C01B10.11 -0.499173004069565
AGIWUR27716
CB4856 CT IV 6661234 6668054 WBGene00022228 Y73B6A.1 Y73B6A.1 0.146182467151645
AGIWUR26914
CB4856 CT IV 6704925 6707052 WBGene00044503 Y73B6A.6 Y73B6A.6 -0.124494976448781
AGIWUR2811;AGIWUR15743;AGIWUR23600
CB4856 CT IV 6732980 6736110 WBGene00022229 Y73B6A.2 Y73B6A.2 -0.19782995380096
AGIWUR33883
CB4856 CT IV 6738727 6740374 WBGene00022230 Y73B6A.3 Y73B6A.3 -0.337051661660411
AGIWUR7831
CB4856 CT IV 6740496 6742443 WBGene00004885 smg-7 Y73B6A.4 -0.0951909461739096
AGIWUR5239
CB4856 CT IV 6742538 6748424 WBGene00003030 lin-45 Y73B6A.5 -0.216044764281544
AGIWUR561;AGIWUR3602;AGIWUR11674;AGIWUR12654;AGIWUR18197;AGIWUR21509;AGIWUR22879;AGIWUR32207
CB4856 CT IV 6757311 6762609 WBGene00019259 H34C03.2 H34C03.2 -0.424387521432497
AGIWUR4690;AGIWUR5240;AGIWUR6955;AGIWUR32986;AGIWUR33475
CB4856 CT IV 6762716 6765200 WBGene00004954 spd-3 H34C03.1 -0.362648103757703
AGIWUR42614
CB4856 CT IV 6770810 6779859 WBGene00015938 anat-1 C17H12.13 -0.127482769768209
AGIWUR5446;AGIWUR28862;AGIWUR30020;AGIWUR40363
CB4856 CT IV 6779848 6784340 WBGene00015927 dyci-1 C17H12.1 -0.0965104492971314
AGIWUR20301
CB4856 CT IV 6786858 6789644 WBGene00015928 C17H12.2 C17H12.2 -0.0967005985929479
AGIWUR4069
CB4856 CT IV 6791074 6792629 WBGene00006917 vha-8 C17H12.14 -0.164559479648393
AGIWUR10144;AGIWUR10801;AGIWUR12310;AGIWUR12618;AGIWUR34885
CB4856 CT IV 6793912 6795629 WBGene00015929 C17H12.3 C17H12.3 -0.0552236507869868
AGIWUR18330
CB4856 CT IV 6795913 6798789 WBGene00015937 C17H12.12 C17H12.12 -0.0216386961624302
AGIWUR20007
CB4856 CT IV 6798921 6802337 WBGene00015930 C17H12.4 C17H12.4 -0.436663589699531
AGIWUR24155;AGIWUR26596
CB4856 CT IV 6805115 6805908 WBGene00236786 C17H12.37 C17H12.37 -0.118910499816311
AGIWUR25400
CB4856 CT IV 6806810 6809630 WBGene00015935 C17H12.10 C17H12.10 -0.0628597762090806
AGIWUR32645
CB4856 CT IV 6812108 6815607 WBGene00015934 ceh-48 C17H12.9 0.246345193447334

AGIWUR28941
CB4856 CT IV 6815912 6817654 WBGene00015931 C17H12.5 C17H12.5 0.106146585498474
AGIWUR15237;AGIWUR24685;AGIWUR36468
CB4856 CT IV 6818606 6820643 WBGene00015933 C17H12.8 C17H12.8 -0.721383015540383
AGIWUR24325
CB4856 CT IV 6821077 6822506 WBGene00015932 C17H12.6 C17H12.6 -0.803989459585846
AGIWUR28820
CB4856 CT IV 6824894 6826763 WBGene00019215 H20E11.3 H20E11.3 -0.156544384635911
AGIWUR43547
CB4856 CT IV 6827777 6829490 WBGene00019214 H20E11.2 H20E11.2 0.0912097478026332
AGIWUR41422
CB4856 CT IV 6830145 6833464 WBGene00019213 H20E11.1 H20E11.1 -0.0992904344607819
AGIWUR1515;AGIWUR24083;AGIWUR24257;AGIWUR26117;AGIWUR34547
CB4856 CT IV 6839064 6839199 WBGene00202332 C05B10.21 C05B10.21 0.0793811559783282
AGIWUR14375;AGIWUR36755
CB4856 CT IV 6841485 6841609 WBGene00202097 C05B10.20 C05B10.20 0.117014836988021
AGIWUR28691
CB4856 CT IV 6842964 6845805 WBGene00003043 lip-1 C05B10.1 -0.240111821472381
AGIWUR28238
CB4856 CT IV 6846129 6859370 WBGene00020069 R13H7.2 R13H7.2 -0.0938427316104917
AGIWUR6354;AGIWUR17959;AGIWUR26946
CB4856 CT IV 6868091 6869137 WBGene00005911 srx-20 R13H7.1 -0.0503217993871345 AGIWUR15324
CB4856 CT IV 6873258 6874774 WBGene00005910 srx-19 T05A12.1 -0.210798043737137
AGIWUR25873;AGIWUR26519
CB4856 CT IV 6876737 6883461 WBGene00006608 tre-2 T05A12.2 -0.204166836460564
AGIWUR3771;AGIWUR16934;AGIWUR34702;AGIWUR35029;AGIWUR37513
CB4856 CT IV 6883576 6887662 WBGene00020234 T05A12.3 T05A12.3 -0.240241812553966
AGIWUR16023
CB4856 CT IV 6887948 6897777 WBGene00020235 T05A12.4 T05A12.4 -0.182439021984219
AGIWUR17582;AGIWUR23680;AGIWUR36204
CB4856 CT IV 6899519 6900056 WBGene00020685 T22D1.6 T22D1.6 -0.177864361672971 AGIWUR24019
CB4856 CT IV 6900326 6903624 WBGene00020686 T22D1.8 T22D1.8 -0.129885068001489
AGIWUR7978;AGIWUR22377;AGIWUR36253
CB4856 CT IV 6903950 6905546 WBGene00020684 T22D1.5 T22D1.5 -0.277928939519441 AGIWUR28419
CB4856 CT IV 6906415 6909913 WBGene00004458 rpn-1 T22D1.9 -0.137697844156509 AGIWUR2624
CB4856 CT IV 6910683 6912865 WBGene00020683 ribo-1 T22D1.4 -0.06784965052071 AGIWUR17258
CB4856 CT IV 6913536 6917110 WBGene00020682 T22D1.3 T22D1.3 0.101613647337797
AGIWUR30821;AGIWUR45070
CB4856 CT IV 6917111 6919454 WBGene00020687 ruvb-2 T22D1.10 -0.151958341583029

AGIWUR21494
CB4856 CT IV 6921855 6922852 WBGene00020681 T22D1.2 T22D1.2 -0.150328101538129 AGIWUR30873
CB4856 CT IV 6926140 6928517 WBGene00020688 T22D1.11 T22D1.11 -0.0172957575283714
AGIWUR32365
CB4856 CT IV 6928942 6937713 WBGene00020689 npr-12 T22D1.12 -0.176926391737156
AGIWUR16564
CB4856 CT IV 6957641 6963716 WBGene00002178 jnk-1 B0478.1 -0.00614914680803569
AGIWUR5169;AGIWUR22314
CB4856 CT IV 6963716 6964654 WBGene00015202 B0478.3 B0478.3 0.107988192231132 AGIWUR3282
CB4856 CT IV 6965492 6967806 WBGene00022725 ZK381.2 ZK381.2 0.165109470001375
AGIWUR31292;AGIWUR35750
CB4856 CT IV 6968209 6970280 WBGene00044352 ZK381.8 ZK381.8 -0.0537959927663736
AGIWUR21041;AGIWUR21905
CB4856 CT IV 6971898 6972527 WBGene00022726 nhr-249 ZK381.3 -0.278706953501095 AGIWUR41593
CB4856 CT IV 6974401 6977318 WBGene00003992 pgl-1 ZK381.4 0.151416911146889 AGIWUR9053
CB4856 CT IV 6977517 6978943 WBGene00001862 him-3 ZK381.1 -0.106811088027129 AGIWUR7115
CB4856 CT IV 6978775 6986935 WBGene00022727 prkl-1 ZK381.5 0.868905189506299
AGIWUR33041;AGIWUR40133
CB4856 CT IV 6999557 7001493 WBGene00016085 C25A8.5 C25A8.5 -0.0996486668740779 AGIWUR40307
CB4856 CT IV 7003623 7009268 WBGene00016084 cht-3 C25A8.4 -0.0461738287303852 AGIWUR32990
CB4856 CT IV 7009841 7010744 WBGene00016083 C25A8.2 C25A8.2 -0.0807276146970226
AGIWUR4076;AGIWUR20082
CB4856 CT IV 7011960 7016542 WBGene00015553 trxr-1 C06G3.7 -0.00151250995987873 AGIWUR31747
CB4856 CT IV 7017626 7020210 WBGene00015552 C06G3.6 C06G3.6 0.270071253663167
AGIWUR7736;AGIWUR28620;AGIWUR44593
CB4856 CT IV 7024211 7028144 WBGene00015551 C06G3.5 C06G3.5 0.79054837949727
AGIWUR30316;AGIWUR36250;AGIWUR38569
CB4856 CT IV 7028173 7030967 WBGene00015550 C06G3.4 C06G3.4 0.0685689434030792 AGIWUR44247
CB4856 CT IV 7031298 7032559 WBGene00015554 C06G3.8 C06G3.8 -0.127639368221425 AGIWUR5950
CB4856 CT IV 7032716 7036982 WBGene00015555 ufl-1 C06G3.9 -0.222927152818841 AGIWUR13922
CB4856 CT IV 7036960 7039580 WBGene00000585 cogc-2 C06G3.10 -0.543544016992012
AGIWUR23147
CB4856 CT IV 7040211 7043694 WBGene00002228 klp-18 C06G3.2 0.166055435624446
AGIWUR14117;AGIWUR15370;AGIWUR19879;AGIWUR28610;AGIWUR31615;AGIWUR38249
CB4856 CT IV 7043935 7044604 WBGene00006572 tin-9.1 C06G3.11 -0.0989791650245788
AGIWUR17497;AGIWUR24565;AGIWUR36287;AGIWUR41579
CB4856 CT IV 7049954 7053450 WBGene00003695 nhr-105 C06G3.1 0.26289926309526
AGIWUR8408;AGIWUR25401;AGIWUR27613;AGIWUR38972;AGIWUR39864
CB4856 CT IV 7057977 7059911 WBGene00005941 srx-50 Y43B11AL.2 0.0390669532214272

AGIWUR10633
CB4856 CT IV 7060192 7061778 WBGene00005811 srw-64 Y43B11AL.3 0.0123047451757174
AGIWUR25631;AGIWUR29248;AGIWUR34284;AGIWUR38179
CB4856 CT IV 7065322 7068035 WBGene00021542 Y43B11AL.1 Y43B11AL.1 0.0467474899226552
AGIWUR28733
CB4856 CT IV 7070858 7071736 WBGene00005206 srg-49 Y43B11AR.6 0.395810674561855
AGIWUR28710
CB4856 CT IV 7072410 7073625 WBGene00005205 srg-48 Y43B11AR.5 0.0382021162212958
AGIWUR9062
CB4856 CT IV 7074030 7075163 WBGene00005207 srg-50 Y43B11AR.2 0.156709554234864
AGIWUR25216
CB4856 CT IV 7081514 7082266 WBGene00021543 Y43B11AR.1 Y43B11AR.1 -0.0728151653349336
AGIWUR4163
CB4856 CT IV 7082220 7083716 WBGene00021544 Y43B11AR.3 Y43B11AR.3 0.462868359647155
AGIWUR16252
CB4856 CT IV 7083694 7084682 WBGene00004473 rps-4 Y43B11AR.4 -0.0135124965405353
AGIWUR360;AGIWUR10099
CB4856 CT IV 7085216 7094364 WBGene00000942 deb-1 ZC477.9 -0.175950321188052
AGIWUR22512;AGIWUR25757;AGIWUR32217;AGIWUR32771
CB4856 CT IV 7101375 7103261 WBGene00022620 rde-8 ZC477.5 -0.000963944100611513 AGIWUR23519
CB4856 CT IV 7103492 7105096 WBGene00022618 ZC477.3 ZC477.3 -0.237311512024752
AGIWUR6342;AGIWUR8916;AGIWUR23991;AGIWUR24712
CB4856 CT IV 7105169 7107733 WBGene00022622 ZC477.10 ZC477.10 0.218701401299451
AGIWUR38280
CB4856 CT IV 7108221 7109912 WBGene00022617 ZC477.2 ZC477.2 0.0373616737035647 AGIWUR23545
CB4856 CT IV 7114899 7119926 WBGene00003527 nas-8 C34D4.9 -0.30563730412577 AGIWUR2427
CB4856 CT IV 7119918 7121413 WBGene00016402 C34D4.10 C34D4.10 -0.286255356422082
AGIWUR17282
CB4856 CT IV 7122194 7123947 WBGene00006113 str-48 C34D4.8 0.166431855509813
AGIWUR19828;AGIWUR35417;AGIWUR42209
CB4856 CT IV 7124481 7125516 WBGene00006114 str-49 C34D4.7 -0.0225154520403237 AGIWUR31208
CB4856 CT IV 7127683 7130462 WBGene00006115 str-50 C34D4.16 -0.174525922801458
AGIWUR42325
CB4856 CT IV 7130701 7131928 WBGene00016403 grsp-3 C34D4.11 0.16788773915782
AGIWUR9753
CB4856 CT IV 7132132 7133217 WBGene00000888 cyn-12 C34D4.12 -0.190257517647549
AGIWUR701
CB4856 CT IV 7133448 7134083 WBGene00016404 mutd-1 C34D4.13 -0.218823156472777
AGIWUR16341

CB4856	CT	IV	7134347	7144898	WBGene00016405	hecd-1	C34D4.14	0.183613322823975	AGIWUR20390
CB4856	CT	IV	7149361	7150692	WBGene00016400	C34D4.4	C34D4.4	-0.131935436969354	AGIWUR3381
CB4856	CT	IV	7150663	7152501	WBGene00000687	col-113	C34D4.15	-0.355412810457901	AGIWUR33931
CB4856	CT	IV	7153623	7154193	WBGene00016399	C34D4.3	C34D4.3	0.286205440936856	AGIWUR8606
CB4856	CT	IV	7154656	7156010	WBGene00016398	C34D4.2	C34D4.2	0.0684083433911891	AGIWUR28894
CB4856	CT	IV	7156900	7160109	WBGene00016397	maph-9	C34D4.1	0.414594463875361	AGIWUR21448
CB4856	CT	IV	7160135	7165748	WBGene00004411	rpc-1	C42D4.8	0.157329362593781	AGIWUR40633
CB4856	CT	IV	7168710	7169346	WBGene00004822	skr-16	C42D4.6	-0.0922738645949668	AGIWUR44191
CB4856	CT	IV	7169931	7171322	WBGene00006251	str-220	C42D4.9	0.292843041508056	AGIWUR13622
CB4856	CT	IV	7171489	7172691	WBGene00006250	str-219	C42D4.10	-0.000299505637359143	AGIWUR984;AGIWUR38838
CB4856	CT	IV	7173789	7175008	WBGene00006069	str-1	C42D4.5	0.0185651349196867	AGIWUR28880
CB4856	CT	IV	7177187	7178423	WBGene00006109	str-44	C42D4.4	0.173284987442281	AGIWUR8855
CB4856	CT	IV	7179239	7180484	WBGene00016596	C42D4.3	C42D4.3	-0.608460539933786	AGIWUR3239
CB4856	CT	IV	7180535	7182993	WBGene00016595	C42D4.2	C42D4.2	-0.888290968553381	AGIWUR26269
CB4856	CT	IV	7183412	7184697	WBGene00016597	clec-179	C42D4.11	-0.33451562879476	AGIWUR42822
CB4856	CT	IV	7185283	7186856	WBGene00006276	str-249	C42D4.12	-0.196219585727736	AGIWUR22230;AGIWUR41333
CB4856	CT	IV	7187189	7188130	WBGene00016598	C42D4.13	C42D4.13	0.190284819622342	AGIWUR12548
CB4856	CT	IV	7189191	7190331	WBGene00016594	C42D4.1	C42D4.1	0.188133695214125	AGIWUR18620
CB4856	CT	IV	7191076	7191841	WBGene00020043	R13A1.5	R13A1.5	-0.132672398195188	AGIWUR28017
CB4856	CT	IV	7193520	7195834	WBGene00020046	best-16	R13A1.9	0.138118407093222	AGIWUR33813
CB4856	CT	IV	7197153	7203643	WBGene00006748	unc-8	R13A1.4	-0.302564521452986	AGIWUR37063
CB4856	CT	IV	7206246	7218000	WBGene00006504	kcc-1	R13A1.2	0.00239749073824758	AGIWUR8066;AGIWUR11215;AGIWUR19432;AGIWUR21562;AGIWUR24569;AGIWUR25696;AGIWUR27820;AGIWUR29249
CB4856	CT	IV	7218049	7219485	WBGene00020045	glb-23	R13A1.8	-0.0768329689605434	AGIWUR17701
CB4856	CT	IV	7220104	7220607	WBGene00020041	R13A1.1	R13A1.1	-0.170202926612516	AGIWUR17265
CB4856	CT	IV	7225416	7227172	WBGene00044504	R13A1.10	R13A1.10	0.0778731478174066	AGIWUR11055;AGIWUR15267
CB4856	CT	IV	7227274	7228306	WBGene00017053	D2024.5	D2024.5	-0.0806664828262429	AGIWUR23426;AGIWUR37950
CB4856	CT	IV	7228403	7230223	WBGene00000292	cap-1	D2024.6	0.16515959943936	AGIWUR37009
CB4856	CT	IV	7231235	7232822	WBGene00017052	D2024.4	D2024.4	-0.158854905069104	AGIWUR13170;AGIWUR14444;AGIWUR40395
CB4856	CT	IV	7237722	7239976	WBGene00001241	elo-3	D2024.3	-0.103136267312486	AGIWUR10615

CB4856	CT	IV	7240445	7242270	WBGene00017054	gstk-2	D2024.7	0.678092641342486	AGIWUR35947
CB4856	CT	IV	7244208	7245502	WBGene00017051	afmd-1	D2024.2	-0.660552190966229	AGIWUR4942;AGIWUR16746
CB4856	CT	IV	7247543	7249437	WBGene00017050	D2024.1	D2024.1	-0.304617727192529	AGIWUR40720
CB4856	CT	IV	7254990	7255909	WBGene00015534	C06E4.5	C06E4.5	0.0172238105640314	AGIWUR6487
CB4856	CT	IV	7257217	7258248	WBGene00015535	C06E4.6	C06E4.6	0.00960346934700684	AGIWUR40975
CB4856	CT	IV	7258653	7259622	WBGene00015533	C06E4.4	C06E4.4	0.343883701435776	AGIWUR32146
CB4856	CT	IV	7260875	7262132	WBGene00015532	C06E4.3	C06E4.3	0.493909784050961	AGIWUR15367
CB4856	CT	IV	7262237	7263237	WBGene00015536	glb-2	C06E4.7	-0.0271509744528924	AGIWUR22556
CB4856	CT	IV	7276961	7283086	WBGene00015531	C06E4.2	C06E4.2	0.2000435207305	AGIWUR4170
CB4856	CT	IV	7283257	7283871	WBGene00015530	C06E4.1	C06E4.1	-0.0734123647338389	AGIWUR38303
CB4856	CT	IV	7284219	7285873	WBGene00015537	C06E4.8	C06E4.8	1.24938004817737	AGIWUR19169
CB4856	CT	IV	7286182	7287526	WBGene00018345	F42C5.3	F42C5.3	2.28776209024841	AGIWUR36799
CB4856	CT	IV	7288074	7292127	WBGene00018346	F42C5.4	F42C5.4	0.709703056783319	AGIWUR22927
CB4856	CT	IV	7292536	7294221	WBGene00018347	F42C5.5	F42C5.5	0.0521854480239661	AGIWUR27276
CB4856	CT	IV	7305628	7308350	WBGene00018344	npr-27	F42C5.2	0.196845472788327	AGIWUR10249
CB4856	CT	IV	7308486	7309057	WBGene00018348	F42C5.6	F42C5.6	0.00952086721184653	AGIWUR32176
CB4856	CT	IV	7309838	7310760	WBGene00001713	grl-4	F42C5.7	-0.557596333713068	AGIWUR17540
CB4856	CT	IV	7312981	7313864	WBGene00004477	rps-8	F42C5.8	0.0358702719689319	AGIWUR14443;AGIWUR25098;AGIWUR29580;AGIWUR41164;AGIWUR41234;AGIWUR43049
CB4856	CT	IV	7314371	7317163	WBGene00018349	F42C5.9	F42C5.9	0.216633006741305	AGIWUR6426;AGIWUR34872
CB4856	CT	IV	7320734	7326663	WBGene00018350	ifo-1	F42C5.10	0.0167670153481119	AGIWUR19909
CB4856	CT	IV	7327110	7329108	WBGene00021495	sqst-2	Y40C5A.1	-0.2514081706837	AGIWUR16543
CB4856	CT	IV	7329620	7332811	WBGene00003841	ocr-4	Y40C5A.2	0.185829061061041	AGIWUR23250
CB4856	CT	IV	7349283	7365111	WBGene00021497	Y40C5A.4	Y40C5A.4	0.19028554675787	AGIWUR41795;AGIWUR44488
CB4856	CT	IV	7375147	7376635	WBGene00020446	T12B3.3	T12B3.3	0.747887292049635	AGIWUR11785
CB4856	CT	IV	7376811	7378906	WBGene00020445	T12B3.2	T12B3.2	-0.457020037401259	AGIWUR26548
CB4856	CT	IV	7382415	7384752	WBGene00020444	T12B3.1	T12B3.1	-0.548953217606575	AGIWUR11306;AGIWUR19404;AGIWUR43250
CB4856	CT	IV	7384756	7388943	WBGene00020447	T12B3.4	T12B3.4	0.000441923513115228	AGIWUR28778
CB4856	CT	IV	7393221	7397555	WBGene00016739	pitr-1	C48A7.2	-0.0690262442544611	AGIWUR21517
CB4856	CT	IV	7405460	7418733	WBGene00001187	egl-19	C48A7.1	0.140987914955023	AGIWUR364;AGIWUR4859;AGIWUR5340;AGIWUR16650;AGIWUR17147;AGIWUR31639;AGIWUR43178
CB4856	CT	IV	7419118	7419971	WBGene00015215	B0496.6	B0496.6	-0.0358662294547383	AGIWUR8028

CB4856	CT	IV	7420958	7422671	WBGene00015214	srtx-1	B0496.5	0.0388515329071115	AGIWUR6765;AGIWUR18892;AGIWUR42651
CB4856	CT	IV	7424498	7444660	WBGene00006814	unc-82	B0496.3	0.177578312882896	AGIWUR29816;AGIWUR33821
CB4856	CT	IV	7444639	7445432	WBGene00015216	valv-1	B0496.7	0.18780437817443	AGIWUR490
CB4856	CT	IV	7446390	7447466	WBGene00015210	B0496.1	B0496.1	-0.243088129955584	AGIWUR23905
CB4856	CT	IV	7447856	7449169	WBGene00015211	B0496.2	B0496.2	-0.115307432598367	AGIWUR6296;AGIWUR8092;AGIWUR26012
CB4856	CT	IV	7449067	7451445	WBGene00015217	tes-1	B0496.8	0.257836117178568	AGIWUR15116
CB4856	CT	IV	7456525	7458214	WBGene00017416	F13B6.1	F13B6.1	1.17105771533563	AGIWUR11830;AGIWUR14383;AGIWUR42047
CB4856	CT	IV	7458836	7460424	WBGene00017417	F13B6.2	F13B6.2	-0.586146581172891	AGIWUR26063
CB4856	CT	IV	7461966	7465957	WBGene00017418	F13B6.3	F13B6.3	-0.25741097465044	AGIWUR17248
CB4856	CT	IV	7466702	7469832	WBGene00018903	F55G1.7	F55G1.7	0.0335395347655822	AGIWUR216
CB4856	CT	IV	7470860	7473340	WBGene00004044	plk-3	F55G1.8	-0.00355402581010278	AGIWUR16334;AGIWUR37685
CB4856	CT	IV	7473476	7474957	WBGene00018902	F55G1.6	F55G1.6	-0.120232895371851	AGIWUR23448
CB4856	CT	IV	7475041	7476215	WBGene00018901	F55G1.5	F55G1.5	-0.416525431567512	AGIWUR31696
CB4856	CT	IV	7476171	7477344	WBGene00018904	F55G1.9	F55G1.9	-0.0503013308347189	AGIWUR9261
CB4856	CT	IV	7478171	7485523	WBGene00018900	rod-1	F55G1.4	-0.133333004556082	AGIWUR3348
CB4856	CT	IV	7486519	7486962	WBGene00001936	his-62	F55G1.3	0.0685786888881612	AGIWUR11523
CB4856	CT	IV	7487560	7488025	WBGene00001934	his-60	F55G1.11	0.00246011076877117	AGIWUR12606;AGIWUR26290
CB4856	CT	IV	7488216	7488956	WBGene00001933	his-59	F55G1.2	0.0897756135200112	AGIWUR33079
CB4856	CT	IV	7491771	7493764	WBGene00018899	F55G1.1	F55G1.1	0.023317437785277	AGIWUR18883
CB4856	CT	IV	7493843	7497850	WBGene00018906	egas-4	F55G1.13	0.0254836801059062	AGIWUR4429;AGIWUR13990
CB4856	CT	IV	7498129	7501437	WBGene00018907	F55G1.15	F55G1.15	-0.0497112921555267	AGIWUR15014
CB4856	CT	IV	7503622	7506447	WBGene00001624	glt-6	R05G6.6	-0.174452987717402	AGIWUR44783
CB4856	CT	IV	7506425	7507891	WBGene00019900	vdac-1	R05G6.7	-0.086318742660461	AGIWUR7516;AGIWUR13358;AGIWUR26690;AGIWUR34696
CB4856	CT	IV	7508514	7509475	WBGene00019899	R05G6.5	R05G6.5	0.0626015454734876	AGIWUR24234
CB4856	CT	IV	7509479	7512667	WBGene00004039	plc-4	R05G6.8	0.178017038777511	AGIWUR15511;AGIWUR15657;AGIWUR38392
CB4856	CT	IV	7513233	7514459	WBGene00019898	R05G6.4	R05G6.4	-0.259345455671077	AGIWUR24319
CB4856	CT	IV	7514366	7516267	WBGene00019901	R05G6.9	R05G6.9	0.102058736018371	AGIWUR43722
CB4856	CT	IV	7516585	7517035	WBGene00043466	R05G6.3	R05G6.3	-0.200481329222173	AGIWUR30281
CB4856	CT	IV	7517445	7542535	WBGene00019902	R05G6.10	R05G6.10	-0.0418804599989411	

									AGIWUR4083;AGIWUR9586;AGIWUR17594;AGIWUR19402;AGIWUR21095;AGIWUR22162;AGIWUR23598;AGIWUR30608;AGIWUR35767;AGIWUR37498;AGIWUR39891;AGIWUR44953
CB4856	CT	IV	7543984	7550812	WBGene00003914	pam-1	F49E8.3	-0.538306561425545	AGIWUR2787;AGIWUR8214;AGIWUR17602;AGIWUR18876;AGIWUR23932
CB4856	CT	IV	7548417	7551511	WBGene00018636	F49E8.2	F49E8.2	0.0942395011683121	AGIWUR43872
CB4856	CT	IV	7551550	7553008	WBGene00000996	dif-1	F49E8.5	0.152143650313093	AGIWUR12711;AGIWUR41882
CB4856	CT	IV	7553532	7554488	WBGene00018637	F49E8.6	F49E8.6	0.301304149902874	AGIWUR10807
CB4856	CT	IV	7554489	7556447	WBGene00018638	F49E8.7	F49E8.7	-0.325838589778158	AGIWUR26569
CB4856	CT	IV	7556637	7558759	WBGene00018635	nprl-2	F49E8.1	-0.226179597174836	AGIWUR6442;AGIWUR23027;AGIWUR37510
CB4856	CT	IV	7558726	7561664	WBGene00002074	ima-3	F32E10.4	0.098250661237735	AGIWUR4573;AGIWUR43964
CB4856	CT	IV	7562220	7563724	WBGene00043068	F32E10.9	F32E10.9	-0.127089445698727	AGIWUR22062
CB4856	CT	IV	7567607	7568507	WBGene00017995	F32E10.8	F32E10.8	0.134704180500767	AGIWUR42705
CB4856	CT	IV	7568893	7573761	WBGene00017991	clec-180	F32E10.3	-0.198767588901403	AGIWUR7560
CB4856	CT	IV	7574355	7578381	WBGene00017992	F32E10.5	F32E10.5	-0.137698724438113	AGIWUR30292
CB4856	CT	IV	7578501	7580269	WBGene00017993	cec-5	F32E10.6	0.0701104800894798	AGIWUR26501;AGIWUR38094
CB4856	CT	IV	7580597	7583726	WBGene00017989	nol-10	F32E10.1	-0.178169719690501	AGIWUR40007
CB4856	CT	IV	7588931	7628234	WBGene00018468	tag-80	F45E4.3	0.0054592198103669	AGIWUR2833;AGIWUR3482;AGIWUR15919;AGIWUR21429;AGIWUR30093;AGIWUR30681;AGIWUR33742;AGIWUR34251
CB4856	CT	IV	7628206	7629042	WBGene00018470	F45E4.5	F45E4.5	0.256875800961394	AGIWUR1969;AGIWUR7378;AGIWUR9519;AGIWUR11474;AGIWUR13230;AGIWUR18375;AGIWUR19195;AGIWUR20605;AGIWUR22249;AGIWUR35994;AGIWUR42005
CB4856	CT	IV	7633360	7634657	WBGene00004046	plp-1	F45E4.2	-0.147548456292472	AGIWUR13584;AGIWUR15669
CB4856	CT	IV	7634786	7635710	WBGene00000190	arf-1.1	F45E4.1	-0.41457935234165	AGIWUR9982;AGIWUR29192
CB4856	CT	IV	7635655	7641925	WBGene00018472	nep-16	F45E4.7	0.175095130492831	AGIWUR10429;AGIWUR19799;AGIWUR25987;AGIWUR37128;AGIWUR37568;AGIWUR39304
CB4856	CT	IV	7642052	7643096	WBGene00003758	nlp-20	F45E4.8	0.167338982019476	AGIWUR31298
CB4856	CT	IV	7644895	7646219	WBGene00001975	hmg-5	F45E4.9	-0.0227567959902135	AGIWUR23274
CB4856	CT	IV	7646200	7654230	WBGene00001584	nrde-4	F45E4.10	0.0996338522830325	

AGIWUR36051
CB4856 CT IV 7658734 7664166 WBGene00015619 C08G9.1 C08G9.1 0.343635893113181
AGIWUR8284;AGIWUR21343;AGIWUR34698
CB4856 CT IV 7664157 7672972 WBGene00015620 C08G9.2 C08G9.2 0.135717443602161
AGIWUR12102;AGIWUR35330;AGIWUR39246;AGIWUR40267;AGIWUR44579
CB4856 CT IV 7675174 7696923 WBGene00002173 itr-1 F33D4.2 -0.184348131528173
AGIWUR1811;AGIWUR9866;AGIWUR11333;AGIWUR16479;AGIWUR20010;AGIWUR24951;AGIWUR24980;AGIWUR25962;AGIWUR27452;AGIWUR27554;AGIWUR31844;AGIWUR40796
CB4856 CT IV 7696932 7698420 WBGene00001456 flp-13 F33D4.3 0.552868907804397 AGIWUR40385
CB4856 CT IV 7703873 7706905 WBGene00017996 F33D4.4 F33D4.4 0.186083867386213 AGIWUR40904
CB4856 CT IV 7707575 7709084 WBGene00017997 mrpl-1 F33D4.5 -0.0445639779444775
AGIWUR23551;AGIWUR40906;AGIWUR40985
CB4856 CT IV 7709192 7709736 WBGene00017999 emc-6 F33D4.7 -0.278697968663666 AGIWUR3453
CB4856 CT IV 7710045 7710826 WBGene00023487 mrps-24 F33D4.8 -0.0811097489349741
AGIWUR1782;AGIWUR18029;AGIWUR24110;AGIWUR41310
CB4856 CT IV 7710975 7714198 WBGene00003607 nhr-8 F33D4.1 -0.163552714407257
AGIWUR9043;AGIWUR11051;AGIWUR20855;AGIWUR37299
CB4856 CT IV 7714164 7718009 WBGene00017998 F33D4.6 F33D4.6 0.0512369113609683
AGIWUR2259;AGIWUR4489;AGIWUR4741;AGIWUR6491;AGIWUR20721;AGIWUR21072;AGIWUR30717;AGIWUR34578;AGIWUR41364
CB4856 CT IV 7720566 7723199 WBGene00016846 C50F7.6 C50F7.6 0.183263656759371 AGIWUR29864
CB4856 CT IV 7726718 7727951 WBGene00016845 C50F7.5 C50F7.5 2.07508594001138
AGIWUR5278;AGIWUR18042;AGIWUR42280
CB4856 CT IV 7728913 7730645 WBGene00016844 sucg-1 C50F7.4 -0.360684011379178 AGIWUR31058
CB4856 CT IV 7730669 7732060 WBGene00016847 C50F7.9 C50F7.9 -0.244778435670325 AGIWUR29923
CB4856 CT IV 7732329 7734210 WBGene00016843 C50F7.3 C50F7.3 0.24863566982818 AGIWUR22357
CB4856 CT IV 7734647 7736409 WBGene00000551 clx-1 C50F7.2 -0.0185675240399652 AGIWUR36592
CB4856 CT IV 7743906 7746074 WBGene00016842 npr-35 C50F7.1 -0.0301769712815059
AGIWUR5063;AGIWUR17132;AGIWUR28609
CB4856 CT IV 7745979 7748225 WBGene00016848 klo-1 C50F7.10 -0.0807432178849261
AGIWUR5356
CB4856 CT IV 7748157 7757358 WBGene00016700 C46A5.4 C46A5.4 0.269298820301726 AGIWUR5385
CB4856 CT IV 7757513 7759627 WBGene00016701 C46A5.5 C46A5.5 -0.0331109923006601 AGIWUR36380
CB4856 CT IV 7760000 7763848 WBGene00016702 C46A5.6 C46A5.6 -0.0629954780747308 AGIWUR4718
CB4856 CT IV 7763931 7767094 WBGene00016698 C46A5.1 C46A5.1 0.134227678546751 AGIWUR10952
CB4856 CT IV 7768395 7770025 WBGene00016703 rdl-1 C46A5.8 0.198113144333377 AGIWUR19222
CB4856 CT IV 7770770 7774275 WBGene00001827 hcf-1 C46A5.9 0.132396488103835
AGIWUR14670;AGIWUR29125;AGIWUR34534
CB4856 CT IV 7774411 7775956 WBGene00016374 swd-2.2 C33H5.7 -0.0348361956872818 AGIWUR13567
CB4856 CT IV 7776025 7778022 WBGene00016373 swd-2.1 C33H5.6 -0.0626942674818984 AGIWUR4839

CB4856	CT	IV	7779379 7782499 WBGene00016376	sec-10	C33H5.9 -0.25606343483684
AGIWUR24432;AGIWUR29650;AGIWUR29677;AGIWUR35758;AGIWUR37825
CB4856	CT	IV	7782599 7783851 WBGene00044325	tag-321 C33H5.19	-0.0264282918897969
AGIWUR8294
CB4856	CT	IV	7783970 7785556 WBGene00044326	tag-322 C33H5.10	-0.0362242298503883
AGIWUR5860
CB4856	CT	IV	7786000 7789613 WBGene00016378	imp-3	C33H5.11	0.038448912142055
AGIWUR351;AGIWUR4509;AGIWUR18679;AGIWUR19177;AGIWUR28518
CB4856	CT	IV	7789847 7791229 WBGene00004703	rsp-6	C33H5.12	0.019497168323598
AGIWUR2502;AGIWUR3624;AGIWUR21741;AGIWUR27504;AGIWUR32559;AGIWUR34162;AGIWUR37983;AGIWUR38089
CB4856	CT	IV	7791704 7792263 WBGene00016379	C33H5.13	C33H5.13	0.444684832003564
AGIWUR285;AGIWUR2593;AGIWUR16455;AGIWUR20732;AGIWUR31399;AGIWUR41305
CB4856	CT	IV	7792349 7794809 WBGene00016380	ntp-1	C33H5.14	0.0364960720396228
AGIWUR738;AGIWUR12371;AGIWUR31554
CB4856	CT	IV	7794871 7796376 WBGene00016381	sgo-1	C33H5.15	0.0165968276127018
AGIWUR36220
CB4856	CT	IV	7797192 7800252 WBGene00002221	klp-10	C33H5.4 -0.22288398538438
AGIWUR403;AGIWUR20408;AGIWUR24076;AGIWUR44025
CB4856	CT	IV	7801916 7803350 WBGene00016382	C33H5.16	C33H5.16	-0.204292908991571
AGIWUR39505
CB4856	CT	IV	7805040 7807948 WBGene00016371	C33H5.2 C33H5.2 0.118657825405071	AGIWUR22667
CB4856	CT	IV	7808912 7810681 WBGene00016370	C33H5.1 C33H5.1 -0.0668410669120805	AGIWUR4459
CB4856	CT	IV	7811157 7812809 WBGene00016383	zgpa-1	C33H5.17	-0.315140503445562
AGIWUR8182
CB4856	CT	IV	7812770 7815733 WBGene00016384	cdgs-1	C33H5.18	0.0449049186060324
AGIWUR10278;AGIWUR14325;AGIWUR20433;AGIWUR20750;AGIWUR21303;AGIWUR21397;AGIWUR25679;AGIWUR28313;AGIWUR28334
;AGIWUR29500;AGIWUR30310;AGIWUR36176;AGIWUR39157;AGIWUR44187
CB4856	CT	IV	7818251 7821195 WBGene00004363	ric-3	T14A8.1 0.465357572111744	AGIWUR12936
CB4856	CT	IV	7821673 7822700 WBGene00020497	T14A8.2 T14A8.2 -0.153947702921801	AGIWUR21984
CB4856	CT	IV	7826334 7829301 WBGene00015509	C06A6.4 C06A6.4 -0.106798619908625	AGIWUR17848
CB4856	CT	IV	7830525 7832401 WBGene00015508	mvb-12	C06A6.3 -0.0676280952685815	AGIWUR28526
CB4856	CT	IV	7832344 7834675 WBGene00015510	C06A6.5 C06A6.5 0.0027044924686308	AGIWUR44246
CB4856	CT	IV	7837350 7838548 WBGene00015511	C06A6.7 C06A6.7 0.0305982791870196
AGIWUR1094;AGIWUR1440;AGIWUR43137
CB4856	CT	IV	7846630 7854224 WBGene00015507	C06A6.2 C06A6.2 -0.103670442159764	AGIWUR3948
CB4856	CT	IV	7868907 7870100 WBGene00001453	flp-10	T06C10.4	0.104474302609486
AGIWUR12836;AGIWUR17073;AGIWUR17722;AGIWUR20675;AGIWUR29234
CB4856	CT	IV	7870865 7873034 WBGene00020289	T06C10.3	T06C10.3	-0.299086210339274
AGIWUR11575

CB4856	CT	IV	7875727 7877881 WBGene00002208	kin-26	T06C10.6	0.270424205091329
AGIWUR12261
CB4856	CT	IV	7879317 7881034 WBGene00020288	srt-73	T06C10.2	-0.406098503309366
AGIWUR1450
CB4856	CT	IV	7882028 7900247 WBGene00016957	atic-1	C55F2.1 -0.652321777151185
AGIWUR11020;AGIWUR15539;AGIWUR31631;AGIWUR40721
CB4856	CT	IV	7900200 7902271 WBGene00016793	arp-11	C49H3.8 -0.0681872988245535
AGIWUR22789;AGIWUR27681;AGIWUR41817
CB4856	CT	IV	7903740 7907083 WBGene00016792	C49H3.6 C49H3.6 0.140932843426418	AGIWUR35959
CB4856	CT	IV	7907093 7909474 WBGene00016794	C49H3.9 C49H3.9 0.0404209494640249
AGIWUR7966;AGIWUR10533;AGIWUR33949
CB4856	CT	IV	7910575 7915184 WBGene00003827	ntl-4	C49H3.5 -0.0698752215294114
AGIWUR1118;AGIWUR2358;AGIWUR7312;AGIWUR8161;AGIWUR8439;AGIWUR19950;AGIWUR23304
CB4856	CT	IV	7915291 7916711 WBGene00016791	C49H3.4 C49H3.4 -0.130649326138037
AGIWUR3522;AGIWUR10447;AGIWUR11519;AGIWUR14805
CB4856	CT	IV	7916699 7924129 WBGene00002080	xpo-3	C49H3.10	-0.0501385132545629
AGIWUR39598
CB4856	CT	IV	7924350 7924968 WBGene00016790	C49H3.3 C49H3.3 -0.225370721335917	AGIWUR2622
CB4856	CT	IV	7925298 7926391 WBGene00004471	rps-2	C49H3.11	-0.00954018756354102
AGIWUR7606;AGIWUR9077
CB4856	CT	IV	7926748 7927315 WBGene00016795	C49H3.12	C49H3.12	0.928438213364741
AGIWUR44761
CB4856	CT	IV	7928159 7933775 WBGene00001535	gcy-8	C49H3.1 0.272526561167263	AGIWUR30693
CB4856	CT	IV	7934097 7937746 WBGene00000242	bbs-2	F20D12.3	0.531217687995983
AGIWUR5162
CB4856	CT	IV	7937959 7938934 WBGene00017645	F20D12.7	F20D12.7	0.16215351021443
AGIWUR12975
CB4856	CT	IV	7939812 7941064 WBGene00017644	exc-9	F20D12.5	0.510437868033396
AGIWUR36421
CB4856	CT	IV	7943617 7946819 WBGene00017643	czw-1	F20D12.4	-0.332848755856204
AGIWUR16838;AGIWUR18511;AGIWUR27746;AGIWUR30226
CB4856	CT	IV	7948643 7953548 WBGene00017642	F20D12.2	F20D12.2	-0.0612280724691558
AGIWUR4597
CB4856	CT	IV	7953509 7955898 WBGene00000442	ceh-19	F20D12.6	0.236983423814078
AGIWUR15960;AGIWUR38313
CB4856	CT	IV	7957580 7962129 WBGene00017641	csr-1	F20D12.1	0.182422749997237
AGIWUR32004
CB4856	CT	IV	7967363 7969600 WBGene00019020	F57H12.5	F57H12.5	-0.340325796842201
AGIWUR37274

CB4856	CT	IV	7970525	7973363	WBGene00019019	frpr-10	F57H12.4	-0.0844418407523806	AGIWUR12162
CB4856	CT	IV	7975430	7976406	WBGene00019021	F57H12.6	F57H12.6	-0.574812939021307	AGIWUR23322
CB4856	CT	IV	7977836	7979014	WBGene00003879	ora-1	F57H12.3	-0.364490163737356	AGIWUR273
CB4856	CT	IV	7979823	7982005	WBGene00006761	unc-24	F57H12.2	0.13214062482278	AGIWUR28958
CB4856	CT	IV	7982526	7983919	WBGene00000183	arf-3	F57H12.1	0.0596937303198928	AGIWUR17772
CB4856	CT	IV	7983854	7987183	WBGene00003178	mec-17	F57H12.7	-0.136258225261171	AGIWUR6494
CB4856	CT	IV	7988685	7991425	WBGene00000689	col-115	Y42H9B.1	-0.466574840889855	AGIWUR29817
CB4856	CT	IV	7991410	8004136	WBGene00004371	rig-4	Y42H9B.2	0.14718259730698	AGIWUR2109
CB4856	CT	IV	8011031	8012430	WBGene00021541	Y42H9B.3	Y42H9B.3	-0.130243199532313	AGIWUR16920
CB4856	CT	IV	8014149	8015722	WBGene00016130	C26B2.8	C26B2.8	-0.552914348200339	AGIWUR8660;AGIWUR13048;AGIWUR25460
CB4856	CT	IV	8016153	8016882	WBGene00016129	C26B2.7	C26B2.7	-0.331886963847134	AGIWUR10974;AGIWUR14635;AGIWUR23376;AGIWUR29971
CB4856	CT	IV	8016994	8018221	WBGene00016128	elpc-4	C26B2.6	0.0319590104915406	AGIWUR32454
CB4856	CT	IV	8018130	8019808	WBGene00016124	dnc-4	C26B2.1	-0.0361348688463039	AGIWUR7415;AGIWUR34884;AGIWUR41679;AGIWUR42082;AGIWUR42711
CB4856	CT	IV	8021146	8022122	WBGene00016125	C26B2.2	C26B2.2	-0.134692950760501	AGIWUR33968
CB4856	CT	IV	8022656	8029513	WBGene00003625	nhr-31	C26B2.3	-0.414455475866733	AGIWUR5390;AGIWUR12112;AGIWUR24589;AGIWUR24612;AGIWUR28272;AGIWUR38754
CB4856	CT	IV	8045045	8047363	WBGene00016126	nhr-258	C26B2.4	-0.137408462361259	AGIWUR15286
CB4856	CT	IV	8062669	8084087	WBGene00004732	sax-7	C18F3.2	-0.200805280141292	AGIWUR1466;AGIWUR9755;AGIWUR15045;AGIWUR23315;AGIWUR30426;AGIWUR32779;AGIWUR34494
CB4856	CT	IV	8099918	8101424	WBGene00021537	Y42H9AR.2	Y42H9AR.2	-0.169952721185773	AGIWUR33662
CB4856	CT	IV	8102501	8104323	WBGene00021540	Y42H9AR.5	Y42H9AR.5	-0.00185772632331904	AGIWUR18854;AGIWUR21863;AGIWUR44955
CB4856	CT	IV	8104287	8107600	WBGene00021538	rabs-5	Y42H9AR.3	-0.295636420744104	AGIWUR18906
CB4856	CT	IV	8107766	8112241	WBGene00021539	Y42H9AR.4	Y42H9AR.4	-0.0240202777284484	AGIWUR1965;AGIWUR5037;AGIWUR6631;AGIWUR23066;AGIWUR30445;AGIWUR33465

CB4856 CT IV 8113384 8115670 WBGene00021536 Y42H9AR.1 Y42H9AR.1 0.0877148786221813
AGIWUR31877
CB4856 CT IV 8115762 8118445 WBGene00018360 irld-8 F42G8.9 -0.00966295686985319 AGIWUR18758
CB4856 CT IV 8121908 8122240 WBGene00249816 F42G8.18 F42G8.18 -0.0486501754122841
AGIWUR20867
CB4856 CT IV 8127734 8128909 WBGene00249817 F42G8.19 F42G8.19 -0.636290336388637
AGIWUR34512
CB4856 CT IV 8128940 8130256 WBGene00018361 F42G8.10 F42G8.10 -0.168733401344364
AGIWUR2781;AGIWUR14396;AGIWUR19987;AGIWUR20573;AGIWUR25865;AGIWUR33090;AGIWUR34700
CB4856 CT IV 8130561 8132213 WBGene00018357 moc-3 F42G8.6 -0.225379219447344 AGIWUR33514
CB4856 CT IV 8132175 8133692 WBGene00004979 sph-1 F42G8.11 0.0257357503381357
AGIWUR35585
CB4856 CT IV 8133789 8136055 WBGene00002162 isp-1 F42G8.12 0.285605388628062
AGIWUR2986;AGIWUR42505
CB4856 CT IV 8136325 8139087 WBGene00018356 islo-1 F42G8.5 -0.0129876851797119
AGIWUR16021;AGIWUR21631;AGIWUR44443;AGIWUR44737
CB4856 CT IV 8139219 8143151 WBGene00004057 pmk-3 F42G8.4 0.364372686356614 AGIWUR35419
CB4856 CT IV 8143500 8148647 WBGene00004056 pmk-2 F42G8.3 0.0195516408176717
AGIWUR24;AGIWUR6567;AGIWUR11118;AGIWUR28986;AGIWUR30474;AGIWUR41777;AGIWUR42922
CB4856 CT IV 8148794 8151156 WBGene00004055 pmk-1 B0218.3 0.0531025870860802
AGIWUR2001;AGIWUR5179;AGIWUR10399;AGIWUR20441;AGIWUR23734;AGIWUR34850;AGIWUR36924
CB4856 CT IV 8152445 8154857 WBGene00015048 faah-2 B0218.2 -0.028261889022545 AGIWUR16601
CB4856 CT IV 8155211 8156587 WBGene00015049 B0218.5 B0218.5 -0.251847595330585 AGIWUR3420
CB4856 CT IV 8159358 8162042 WBGene00015047 faah-1 B0218.1 0.143235055703697 AGIWUR22394
CB4856 CT IV 8161967 8163795 WBGene00015050 clec-51 B0218.6 -0.432186375287364
AGIWUR5196;AGIWUR44442
CB4856 CT IV 8164590 8166050 WBGene00015051 B0218.7 B0218.7 0.117461629997228 AGIWUR5993
CB4856 CT IV 8167164 8168813 WBGene00015052 clec-52 B0218.8 0.188737029179137 AGIWUR921
CB4856 CT IV 8176479 8188879 WBGene00003961 pct-1 C07G1.3 0.171782977408005
AGIWUR35135;AGIWUR38001
CB4856 CT IV 8188904 8198951 WBGene00006957 wsp-1 C07G1.4 -0.0655453798063821
AGIWUR1941;AGIWUR4372;AGIWUR9072;AGIWUR9845;AGIWUR15505;AGIWUR19474;AGIWUR41409
CB4856 CT IV 8200604 8201233 WBGene00044608 glrx-22 C07G1.8 -0.0174572760483329
AGIWUR2696;AGIWUR10561;AGIWUR19689;AGIWUR27723;AGIWUR33581
CB4856 CT IV 8201378 8206048 WBGene00004101 hgrs-1 C07G1.5 0.203377331310621 AGIWUR13098
CB4856 CT IV 8206614 8213723 WBGene00006620 svh-1 C07G1.1 -0.0698615013568782
AGIWUR22767;AGIWUR25262
CB4856 CT IV 8213826 8220036 WBGene00003839 ocr-2 T09A12.3 -0.409775705252835
AGIWUR7008

CB4856	CT	IV	8222202	8223459	WBGene00020373	gpx-6	T09A12.2	0.203383787317306	AGIWUR5259;AGIWUR30035
CB4856	CT	IV	8225674	8226366	WBGene00020374	T09A12.5	T09A12.5	0.0130035332743486	AGIWUR19944
CB4856	CT	IV	8226337	8236602	WBGene00003656	nhr-66	T09A12.4	-0.110154911474288	AGIWUR5339;AGIWUR18857;AGIWUR19208;AGIWUR25358;AGIWUR26631;AGIWUR30253;AGIWUR43015
CB4856	CT	IV	8259248	8259979	WBGene00019507	K07H8.7	K07H8.7	0.113470150074519	AGIWUR3973;AGIWUR19600
CB4856	CT	IV	8261134	8266462	WBGene00006930	vit-6	K07H8.6	0.0390003084246668	AGIWUR8837;AGIWUR35220
CB4856	CT	IV	8266752	8268435	WBGene00019508	K07H8.8	K07H8.8	0.175750750496698	AGIWUR19784
CB4856	CT	IV	8273226	8275332	WBGene00044510	K07H8.11	K07H8.11	1.07928609346465	AGIWUR24203;AGIWUR36263
CB4856	CT	IV	8275621	8276709	WBGene00044509	K07H8.12	K07H8.12	-0.0820249462542462	AGIWUR17978;AGIWUR27697;AGIWUR31520;AGIWUR37850;AGIWUR38264
CB4856	CT	IV	8285175	8286566	WBGene00019509	K07H8.9	K07H8.9	0.361182528177373	AGIWUR36215
CB4856	CT	IV	8286587	8289531	WBGene00019510	K07H8.10	K07H8.10	-0.0805288665671586	AGIWUR657;AGIWUR2486;AGIWUR8519;AGIWUR15273;AGIWUR22002;AGIWUR26379;AGIWUR28977
CB4856	CT	IV	8290273	8291496	WBGene00000923	daf-31	K07H8.3	-0.0951400866643475	AGIWUR20212
CB4856	CT	IV	8291515	8294596	WBGene00019504	K07H8.2	K07H8.2	0.010133654879156	AGIWUR2279;AGIWUR6071;AGIWUR17654;AGIWUR26947
CB4856	CT	IV	8294690	8297283	WBGene00019503	tbce-1	K07H8.1	-0.174867028335761	AGIWUR15141
CB4856	CT	IV	8297197	8299843	WBGene00018072	nprl-3	F35H10.7	-0.0384377698636538	AGIWUR4422;AGIWUR42532
CB4856	CT	IV	8299936	8300647	WBGene00018071	F35H10.6	F35H10.6	-0.0770649637663574	AGIWUR17331
CB4856	CT	IV	8300755	8301165	WBGene00018070	F35H10.5	F35H10.5	0.182905863847537	AGIWUR44449
CB4856	CT	IV	8303968	8307369	WBGene00006914	vha-5	F35H10.4	-0.19374354130251	AGIWUR21901;AGIWUR30912;AGIWUR35131
CB4856	CT	IV	8307294	8312946	WBGene00018073	F35H10.10	F35H10.10	-0.157826101303819	AGIWUR17187
CB4856	CT	IV	8325967	8327062	WBGene00018069	F35H10.3	F35H10.3	-0.105550437115159	AGIWUR9334
CB4856	CT	IV	8329205	8329956	WBGene00018068	F35H10.2	F35H10.2	0.0895924583264485	AGIWUR36343
CB4856	CT	IV	8332826	8333559	WBGene00003099	lys-10	F17E9.11	0.220489795738708	AGIWUR42956
CB4856	CT	IV	8333557	8334021	WBGene00001905	his-31	F17E9.12	0.0576867083550034	

AGIWUR22692
CB4856 CT IV 8334298 8334862 WBGene00001906 his-32 F17E9.10 0.387679948206898
AGIWUR5672;AGIWUR14633;AGIWUR15754;AGIWUR21832;AGIWUR32746
CB4856 CT IV 8336807 8338172 WBGene00023270 F17E9.14 F17E9.14 -0.299353336693732
AGIWUR44242
CB4856 CT IV 8342685 8344485 WBGene00017543 lgc-5 F17E9.7 0.265240640167049 AGIWUR8436
CB4856 CT IV 8345818 8346546 WBGene00017542 F17E9.5 F17E9.5 -0.499952596430226 AGIWUR38365
CB4856 CT IV 8348373 8349169 WBGene00017541 F17E9.4 F17E9.4 0.379637734855642 AGIWUR2460
CB4856 CT IV 8354874 8355433 WBGene00017540 F17E9.3 F17E9.3 -0.610455212614539 AGIWUR9498
CB4856 CT IV 8355892 8356503 WBGene00017539 F17E9.2 F17E9.2 -0.31896740654013 AGIWUR27596
CB4856 CT IV 8356776 8357854 WBGene00000690 col-116 F17E9.1 0.316226676838989 AGIWUR39272
CB4856 CT IV 8357938 8358592 WBGene00044953 D2096.13 D2096.13 -0.112375133086333
AGIWUR4484;AGIWUR9578;AGIWUR10373;AGIWUR22490
CB4856 CT IV 8359165 8359716 WBGene00017072 D2096.5 D2096.5 -0.593101374818906 AGIWUR14138
CB4856 CT IV 8361089 8363370 WBGene00005019 sqv-1 D2096.4 -0.122792872882918
AGIWUR5412;AGIWUR24328;AGIWUR41399
CB4856 CT IV 8363463 8366376 WBGene00017079 D2096.12 D2096.12 -0.264496683168126
AGIWUR8270
CB4856 CT IV 8366539 8375371 WBGene00017071 aagr-1 D2096.3 0.662604160459228 AGIWUR12328
CB4856 CT IV 8375720 8377698 WBGene00017074 D2096.7 D2096.7 -0.216214413416291
AGIWUR3581;AGIWUR8824;AGIWUR12898;AGIWUR41238
CB4856 CT IV 8377703 8379319 WBGene00017075 nap-1 D2096.8 -0.199225862447395 AGIWUR22992
CB4856 CT IV 8379984 8381925 WBGene00017070 praf-3 D2096.2 -0.0701344152901031
AGIWUR9038;AGIWUR18527;AGIWUR31847;AGIWUR40100
CB4856 CT IV 8381922 8382814 WBGene00017076 D2096.9 D2096.9 0.232665744594474 AGIWUR3892
CB4856 CT IV 8387626 8389239 WBGene00017077 D2096.10 D2096.10
0.000246438543156202 AGIWUR19057
CB4856 CT IV 8398923 8399762 WBGene00019789 M116.2 M116.2 0.0248824668480978 AGIWUR34103
CB4856 CT IV 8414293 8421061 WBGene00019792 M116.5 M116.5 0.0103343479843598 AGIWUR4969
CB4856 CT IV 8422053 8424146 WBGene00023488 srz-84 T26A8.5 0.0555003156813649
AGIWUR7668;AGIWUR26211;AGIWUR32005;AGIWUR37031;AGIWUR38715;AGIWUR44688
CB4856 CT IV 8424278 8428512 WBGene00020825 tmem-231 T26A8.2 0.535787402928444
AGIWUR2333
CB4856 CT IV 8428961 8431493 WBGene00020827 T26A8.4 T26A8.4 -0.12627247548399 AGIWUR31550
CB4856 CT IV 8433038 8438004 WBGene00020824 T26A8.1 T26A8.1 -0.0811000164207343 AGIWUR25557
CB4856 CT IV 8447204 8449048 WBGene00020138 T01B11.1 T01B11.1 -0.0685213452913072
AGIWUR23119
CB4856 CT IV 8449353 8451771 WBGene00020139 T01B11.2 T01B11.2 -0.283033602760121
AGIWUR1975;AGIWUR23355;AGIWUR24343;AGIWUR27969;AGIWUR37585

CB4856 CT IV 8457280 8458933 WBGene00006374 syx-4 T01B11.3 -0.0153765238913675
AGIWUR12060
CB4856 CT IV 8459178 8460327 WBGene00020140 ant-1.4 T01B11.4 -0.0504088036588963
AGIWUR8471;AGIWUR23208
CB4856 CT IV 8460999 8463816 WBGene00006220 str-176 T01B11.5 -0.0339937457330528
AGIWUR2743;AGIWUR7347;AGIWUR17297;AGIWUR25379;AGIWUR28348
CB4856 CT IV 8464752 8472605 WBGene00003837 oat-1 T01B11.7 -0.530666733863661
AGIWUR1624;AGIWUR6649;AGIWUR13136
CB4856 CT IV 8474056 8475278 WBGene00016181 C28C12.11 C28C12.11 0.213621554459438
AGIWUR8444
CB4856 CT IV 8477182 8480656 WBGene00004995 spp-10 C28C12.7 0.255493283479097
AGIWUR10234;AGIWUR13523;AGIWUR23082;AGIWUR44161
CB4856 CT IV 8481664 8483710 WBGene00004993 spp-8 C28C12.5 1.27656085922146
AGIWUR19253
CB4856 CT IV 8487497 8488481 WBGene00016180 C28C12.4 C28C12.4 0.049505749334612
AGIWUR31231
CB4856 CT IV 8490221 8490857 WBGene00016179 C28C12.3 C28C12.3 -0.14554961014859
AGIWUR40139
CB4856 CT IV 8492542 8494487 WBGene00005670 sru-7 C28C12.13 0.0947224576113772
AGIWUR1844
CB4856 CT IV 8494550 8496363 WBGene00016182 C28C12.12 C28C12.12 -0.137795465913812
AGIWUR5702;AGIWUR6603;AGIWUR6924;AGIWUR25765;AGIWUR26275;AGIWUR26545;AGIWUR29922;AGIWUR41298
CB4856 CT IV 8496486 8498871 WBGene00001566 acdh-13 C28C12.9 -0.195916011250326
AGIWUR33202
CB4856 CT IV 8499310 8500076 WBGene00016178 mesp-1 C28C12.2 0.0381105593782713
AGIWUR28639
CB4856 CT IV 8501479 8502983 WBGene00016177 C28C12.1 C28C12.1 -0.113399601913134
AGIWUR1654;AGIWUR16632;AGIWUR31146;AGIWUR32280;AGIWUR34091;AGIWUR39245
CB4856 CT IV 8503493 8510559 WBGene00006450 tag-77 C28C12.10 0.118128554500675
AGIWUR11253;AGIWUR18212;AGIWUR32240;AGIWUR36697
CB4856 CT IV 8513205 8513916 WBGene00044001 C09G4.4 C09G4.4 -0.0178099820805659
AGIWUR28241;AGIWUR42704
CB4856 CT IV 8513919 8514725 WBGene00001051 cks-1 C09G4.3 0.00246570613093766 AGIWUR43376
CB4856 CT IV 8520894 8538182 WBGene00015650 pkg-2 C09G4.2 0.0031766949588885
AGIWUR4010;AGIWUR11603;AGIWUR13590;AGIWUR16392;AGIWUR23473;AGIWUR31328;AGIWUR39930
CB4856 CT IV 8539123 8544403 WBGene00002043 hyl-1 C09G4.1 -0.00139964826285421
AGIWUR19450;AGIWUR23662;AGIWUR38311
CB4856 CT IV 8559434 8563159 WBGene00022004 npr-22 Y59H11AL.1 0.00618447062926508
AGIWUR20346;AGIWUR29725

CB4856	CT	IV	8569144	8570353	WBGene00022008	Y59H11AM.4	Y59H11AM.4	0.184015910597295	AGIWUR21150
CB4856	CT	IV	8571438	8571821	WBGene00022005	Y59H11AM.1	Y59H11AM.1	0.29724837873092	AGIWUR27952
CB4856	CT	IV	8585516	8594613	WBGene00022010	catp-7	Y59H11AR.2	0.635360602193753	AGIWUR26104
CB4856	CT	IV	8594871	8595417	WBGene00022011	Y59H11AR.3	Y59H11AR.3	-0.474174353497415	AGIWUR27882
CB4856	CT	IV	8596067	8596943	WBGene00044511	clec-181	Y59H11AR.5	0.0488567155144892	AGIWUR10135;AGIWUR12743;AGIWUR39931
CB4856	CT	IV	8597762	8598727	WBGene00077437	Y59H11AR.6	Y59H11AR.6	0.0145282843225214	AGIWUR1141;AGIWUR20626;AGIWUR26591;AGIWUR30910;AGIWUR39286;AGIWUR45101
CB4856	CT	IV	8598739	8600742	WBGene00022012	Y59H11AR.4	Y59H11AR.4	0.18570424211789	AGIWUR9861;AGIWUR14711
CB4856	CT	IV	8609239	8611595	WBGene00018335	F42A9.6	F42A9.6	-0.336174401805724	AGIWUR15055
CB4856	CT	IV	8612363	8614683	WBGene00018334	cyp-33E2	F42A9.5	-0.406236190920639	AGIWUR4458
CB4856	CT	IV	8616006	8617147	WBGene00018336	F42A9.7	F42A9.7	0.0158589839113592	AGIWUR26310
CB4856	CT	IV	8617285	8618153	WBGene00018332	F42A9.3	F42A9.3	-0.0848065657998482	AGIWUR21805
CB4856	CT	IV	8618244	8618788	WBGene00018337	F42A9.8	F42A9.8	0.0940287625386613	AGIWUR17831
CB4856	CT	IV	8620683	8626149	WBGene00003034	lin-49	F42A9.2	-0.147581595605851	AGIWUR15629
CB4856	CT	IV	8631334	8644019	WBGene00006483	dgk-4	F42A9.1	-0.191387147912216	AGIWUR31290
CB4856	CT	IV	8644732	8645345	WBGene00016767	C49C8.3	C49C8.3	0.141643124738039	AGIWUR16082
CB4856	CT	IV	8645521	8648429	WBGene00016768	cyp-33E1	C49C8.4	0.34553813406092	AGIWUR34645
CB4856	CT	IV	8648596	8653677	WBGene00016769	C49C8.5	C49C8.5	-0.00106143977793671	AGIWUR21299
CB4856	CT	IV	8654604	8656829	WBGene00016765	C49C8.1	C49C8.1	-0.352307677524256	AGIWUR6466
CB4856	CT	IV	8657455	8658184	WBGene00016770	C49C8.6	C49C8.6	-0.138335476132466	AGIWUR9489
CB4856	CT	IV	8666252	8667177	WBGene00017235	F08B4.3	F08B4.3	0.0194101007563248	AGIWUR5523
CB4856	CT	IV	8668941	8676542	WBGene00000397	cdh-5	F08B4.2	0.438033044127178	AGIWUR11543;AGIWUR30314;AGIWUR32142
CB4856	CT	IV	8677590	8678019	WBGene00255375	F08B4.8	F08B4.8	0.31031006496635	AGIWUR22110
CB4856	CT	IV	8678695	8679325	WBGene00017236	F08B4.4	F08B4.4	-0.0793657520176483	AGIWUR989
CB4856	CT	IV	8682239	8686610	WBGene00017237	pole-2	F08B4.5	-0.150563620747168	AGIWUR19783;AGIWUR31958
CB4856	CT	IV	8686756	8687810	WBGene00017238	F08B4.7	F08B4.7	-0.256208092647249	AGIWUR1268;AGIWUR30177
CB4856	CT	IV	8688273	8691513	WBGene00002028	hst-1	F08B4.6	0.189175222826943	AGIWUR10146
CB4856	CT	IV	8692086	8696553	WBGene00000994	dic-1	F08B4.1	-0.279360855309296	

AGIWUR9759;AGIWUR35715
CB4856 CT IV 8697736 8702699 WBGene00019230 ttll-11 H23L24.3 -0.132583305577096
AGIWUR37576
CB4856 CT IV 8703043 8705606 WBGene00019231 H23L24.4 H23L24.4 -0.193224217497642
AGIWUR33495
CB4856 CT IV 8712635 8715027 WBGene00004052 parg-2 H23L24.5 -0.460622056707235
AGIWUR20011
CB4856 CT IV 8715247 8717881 WBGene00019229 ipla-5 H23L24.2 -0.111319064168107
AGIWUR35798
CB4856 CT IV 8724020 8724876 WBGene00009014 F21D5.9 F21D5.9 0.476409696996114 AGIWUR22252
CB4856 CT IV 8725722 8728060 WBGene00009006 F21D5.1 F21D5.1 -0.138502281072406
AGIWUR5139;AGIWUR23171;AGIWUR26887;AGIWUR27754;AGIWUR34703;AGIWUR36880
CB4856 CT IV 8728139 8730510 WBGene00009007 otub-3 F21D5.2 -0.368428919461826
AGIWUR5723;AGIWUR5991;AGIWUR13125;AGIWUR18617;AGIWUR25556
CB4856 CT IV 8730721 8735686 WBGene00009008 F21D5.3 F21D5.3 -0.369527560332411 AGIWUR19369
CB4856 CT IV 8735695 8739394 WBGene00009010 F21D5.5 F21D5.5 -0.102038241688568 AGIWUR8645
CB4856 CT IV 8739504 8740026 WBGene00009013 mrps-33 F21D5.8 -0.0608439531719611 AGIWUR26351
CB4856 CT IV 8740140 8741388 WBGene00009011 F21D5.6 F21D5.6 -0.246578078841939 AGIWUR32885
CB4856 CT IV 8741383 8743704 WBGene00009012 F21D5.7 F21D5.7 -0.182176578995895
AGIWUR448;AGIWUR7143;AGIWUR44082
CB4856 CT IV 8744411 8745691 WBGene00008972 F20C5.7 F20C5.7 0.159273654450773 AGIWUR17508
CB4856 CT IV 8746252 8752676 WBGene00004051 parg-1 F20C5.1 -0.175895273748869
AGIWUR4893;AGIWUR38395;AGIWUR43587
CB4856 CT IV 8753195 8754823 WBGene00008968 F20C5.3 F20C5.3 0.100892234627924 AGIWUR12683
CB4856 CT IV 8755038 8762057 WBGene00002222 klp-11 F20C5.2 0.270390572320773
AGIWUR5353;AGIWUR17348;AGIWUR26051;AGIWUR27395
CB4856 CT IV 8764867 8767450 WBGene00008969 F20C5.4 F20C5.4 -0.0288469483899902 AGIWUR25943
CB4856 CT IV 8767688 8770092 WBGene00008970 F20C5.5 F20C5.5 0.28968009906243 AGIWUR33556
CB4856 CT IV 8771558 8773206 WBGene00008971 F20C5.6 F20C5.6 0.545509001724861
AGIWUR25050;AGIWUR31933;AGIWUR40251
CB4856 CT IV 8773977 8778145 WBGene00007900 C33D9.6 C33D9.6 0.450569520680256
AGIWUR13080;AGIWUR24131;AGIWUR35116;AGIWUR41899;AGIWUR42317
CB4856 CT IV 8778721 8780872 WBGene00007899 C33D9.5 C33D9.5 0.786327252763424 AGIWUR13505
CB4856 CT IV 8783271 8785254 WBGene00194651 C33D9.13 C33D9.13 0.353498835404494
AGIWUR30795
CB4856 CT IV 8787459 8788057 WBGene00044239 C33D9.10 C33D9.10 0.259009620016241
AGIWUR14289;AGIWUR29107;AGIWUR29938;AGIWUR33034;AGIWUR38129
CB4856 CT IV 8788449 8790244 WBGene00005698 sru-35 C33D9.4 -0.113276906772985 AGIWUR3912
CB4856 CT IV 8790742 8793294 WBGene00007898 C33D9.3 C33D9.3 0.257327576296435 AGIWUR40327

CB4856	CT	IV	8796881	8798355	WBGene00007897	clec-182	C33D9.2	0.029835654799963	AGIWUR38966
CB4856	CT	IV	8799089	8813539	WBGene00001366	exc-5	C33D9.1	0.172736372436076	AGIWUR702;AGIWUR39104
CB4856	CT	IV	8816335	8818525	WBGene00012118	grsp-2	T28C6.1	-0.0863869903864938	AGIWUR5776;AGIWUR8431;AGIWUR8780;AGIWUR11470;AGIWUR29223;AGIWUR29771;AGIWUR34493
CB4856	CT	IV	8818555	8819158	WBGene00045183	T28C6.10	T28C6.10	0.542736332060853	AGIWUR2357;AGIWUR9034;AGIWUR27977;AGIWUR30031;AGIWUR41136
CB4856	CT	IV	8820356	8821265	WBGene00012119	T28C6.3	T28C6.3	0.0907240657902673	AGIWUR3511
CB4856	CT	IV	8821447	8822874	WBGene00012120	T28C6.5	T28C6.5	-0.235026595644019	AGIWUR23333
CB4856	CT	IV	8840725	8842805	WBGene00011706	T11B7.1	T11B7.1	0.0964144994553369	AGIWUR462
CB4856	CT	IV	8843621	8844491	WBGene00011707	T11B7.2	T11B7.2	-0.104084160831734	AGIWUR18714
CB4856	CT	IV	8848182	8849673	WBGene00000692	col-118	T11B7.3	0.107483905084337	AGIWUR9680;AGIWUR43006
CB4856	CT	IV	8850865	8851127	WBGene00044914	T11B7.6	T11B7.6	-0.49236529436778	AGIWUR38341
CB4856	CT	IV	8852500	8862228	WBGene00001132	alp-1	T11B7.4	-0.419525713769379	AGIWUR1301;AGIWUR11637;AGIWUR14354;AGIWUR16309
CB4856	CT	IV	8862173	8865483	WBGene00007492	C09G9.1	C09G9.1	0.24070397071533	AGIWUR6290;AGIWUR24125;AGIWUR40398
CB4856	CT	IV	8868036	8869679	WBGene00007493	npp-23	C09G9.2	-0.0320612583142648	AGIWUR42471
CB4856	CT	IV	8869962	8873057	WBGene00007494	C09G9.3	C09G9.3	0.0555373551941067	AGIWUR10147;AGIWUR37282
CB4856	CT	IV	8873820	8874007	WBGene00045323	C09G9.9	C09G9.9	-0.0362314357806692	AGIWUR13666;AGIWUR14560;AGIWUR17102;AGIWUR28067;AGIWUR33063
CB4856	CT	IV	8874129	8877022	WBGene00006409	hdl-2	C09G9.4	-0.00944521660774811	AGIWUR18595
CB4856	CT	IV	8877553	8879910	WBGene00007496	npax-4	C09G9.7	-0.397688919296981	AGIWUR3477
CB4856	CT	IV	8881481	8882240	WBGene00007497	C09G9.8	C09G9.8	0.0599809971377141	AGIWUR35470
CB4856	CT	IV	8886528	8888602	WBGene00007495	C09G9.5	C09G9.5	0.0285943382139635	AGIWUR1804;AGIWUR30771
CB4856	CT	IV	8888976	8890999	WBGene00003864	oma-1	C09G9.6	0.182113583941472	AGIWUR35807
CB4856	CT	IV	8891466	8892747	WBGene00005007	spr-2	C27B7.1	-0.152329793970861	AGIWUR5937;AGIWUR16694
CB4856	CT	IV	8892858	8893854	WBGene00007760	C27B7.2	C27B7.2	-0.0643576412413758	AGIWUR23338
CB4856	CT	IV	8894083	8901386	WBGene00007761	rad-26	C27B7.4	-0.244009741373156	AGIWUR14504;AGIWUR17257;AGIWUR21870;AGIWUR23761;AGIWUR27573;AGIWUR42108
CB4856	CT	IV	8901646	8903177	WBGene00007762	C27B7.5	C27B7.5	-0.122022213503982	AGIWUR23632
CB4856	CT	IV	8903222	8906205	WBGene00007763	C27B7.6	C27B7.6	-0.173376195598119	AGIWUR44147
CB4856	CT	IV	8907047	8915383	WBGene00007764	C27B7.7	C27B7.7	0.573952387590918	AGIWUR44416
CB4856	CT	IV	8915472	8916110	WBGene00007765	C27B7.9	C27B7.9	0.105679816141228	AGIWUR39920

CB4856	CT	IV	8916408	8918890	WBGene00004307	rap-1	C27B7.8	0.132558327249638	AGIWUR9710;AGIWUR16339;AGIWUR18115;AGIWUR32787;AGIWUR38012;AGIWUR39863
CB4856	CT	IV	8924161	8926112	WBGene00008363	D1046.2	D1046.2	-0.196629818880643	AGIWUR2947;AGIWUR32878
CB4856	CT	IV	8926335	8930315	WBGene00008362	cfim-2	D1046.1	-0.124121037108353	AGIWUR43416
CB4856	CT	IV	8930442	8932791	WBGene00008364	slc-25A26	D1046.3	0.0835283683791843	AGIWUR37641
CB4856	CT	IV	8933997	8937400	WBGene00008365	D1046.4	D1046.4	-0.23225396472121	AGIWUR4421;AGIWUR21306;AGIWUR29332
CB4856	CT	IV	8956489	8965534	WBGene00008366	tpra-1	D1046.5	0.478098789261054	AGIWUR13759
CB4856	CT	IV	8966133	8967903	WBGene00008272	C53B4.2	C53B4.2	-0.242697575379971	AGIWUR12201
CB4856	CT	IV	8968290	8970565	WBGene00008271	C53B4.1	C53B4.1	0.129745556388244	AGIWUR35347
CB4856	CT	IV	8970868	8973707	WBGene00008273	C53B4.3	C53B4.3	-0.454875651338908	AGIWUR18768
CB4856	CT	IV	8975532	8984706	WBGene00008274	C53B4.4	C53B4.4	-0.00375329905910959	AGIWUR9423
CB4856	CT	IV	8984866	8986774	WBGene00008275	nstp-1	C53B4.6	-0.0630543117996041	AGIWUR16097
CB4856	CT	IV	8987130	8990444	WBGene00000266	bre-1	C53B4.7	-0.135413682325424	AGIWUR2014;AGIWUR6820;AGIWUR10995;AGIWUR14717;AGIWUR21811;AGIWUR33573
CB4856	CT	IV	8993086	9000774	WBGene00008277	mltn-12	C53B4.8	0.26897164131524	AGIWUR43232
CB4856	CT	IV	9001145	9002734	WBGene00008285	C53D6.8	C53D6.8	0.200779162119735	AGIWUR36472
CB4856	CT	IV	9002837	9005410	WBGene00006852	unc-129	C53D6.2	0.448745358254197	AGIWUR24153
CB4856	CT	IV	9010584	9012339	WBGene00044241	C53D6.11	C53D6.11	-0.128644844818019	AGIWUR9918;AGIWUR38669
CB4856	CT	IV	9016788	9019101	WBGene00008280	acc-2	C53D6.3	-0.0164867087485163	AGIWUR5422;AGIWUR31435;AGIWUR43909
CB4856	CT	IV	9019175	9020940	WBGene00008281	C53D6.4	C53D6.4	-0.221856097163819	AGIWUR14521
CB4856	CT	IV	9021515	9023607	WBGene00005906	srx-15	C53D6.9	0.134706983796511	AGIWUR17975;AGIWUR34822
CB4856	CT	IV	9024738	9027598	WBGene00008282	C53D6.5	C53D6.5	-0.217701172288879	AGIWUR44862
CB4856	CT	IV	9027686	9032659	WBGene00008283	C53D6.6	C53D6.6	-0.28495383928519	AGIWUR17419
CB4856	CT	IV	9033576	9034440	WBGene00023424	C53D6.10	C53D6.10	-0.0728114676892631	AGIWUR7408
CB4856	CT	IV	9034845	9037163	WBGene00008284	C53D6.7	C53D6.7	-0.373203575600398	AGIWUR3673
CB4856	CT	IV	9039575	9040504	WBGene00013516	Y73F4A.3	Y73F4A.3	0.64549140130176	AGIWUR10887
CB4856	CT	IV	9042387	9043347	WBGene00013515	Y73F4A.2	Y73F4A.2	0.691109850501157	AGIWUR3643
CB4856	CT	IV	9043938	9045369	WBGene00013514	Y73F4A.1	Y73F4A.1	0.00510994275523657	AGIWUR9218
CB4856	CT	IV	9070010	9073622	WBGene00003610	nhr-11	ZC410.1	0.0108748574076509

AGIWUR7171;AGIWUR11583;AGIWUR23108;AGIWUR39961
CB4856 CT IV 9074673 9076571 WBGene00013880 mppb-1 ZC410.2 -0.312224909465378 AGIWUR19324
CB4856 CT IV 9076501 9078807 WBGene00013881 mans-4 ZC410.3 -0.0678255586794965
AGIWUR9478;AGIWUR18890;AGIWUR19559
CB4856 CT IV 9082361 9087554 WBGene00006663 twk-8 ZC410.4 -0.332877221426467
AGIWUR23492;AGIWUR23900
CB4856 CT IV 9088594 9092193 WBGene00013882 msa-1 ZC410.5 0.0892296061818163
AGIWUR3044;AGIWUR5696;AGIWUR19247;AGIWUR31771
CB4856 CT IV 9092217 9094468 WBGene00003066 lpl-1 ZC410.7 -0.115387380296404
AGIWUR13244;AGIWUR23652;AGIWUR27179
CB4856 CT IV 9094541 9095653 WBGene00002046 icln-1 C01F6.8 -0.262852367320468
AGIWUR3303;AGIWUR13660
CB4856 CT IV 9095754 9096231 WBGene00007223 C01F6.9 C01F6.9 -0.121699887923313
AGIWUR1398;AGIWUR29038
CB4856 CT IV 9096842 9099364 WBGene00007221 cpna-3 C01F6.1 0.0222408737593112
AGIWUR2412;AGIWUR10869;AGIWUR24822
CB4856 CT IV 9099242 9101592 WBGene00007222 C01F6.2 C01F6.2 0.345911281004394 AGIWUR12163
CB4856 CT IV 9101773 9103851 WBGene00000374 cyp-31A1 C01F6.3 -0.599404914048623
AGIWUR3245
CB4856 CT IV 9104966 9107609 WBGene00001413 fem-3 C01F6.4 -0.0701307178205433 AGIWUR43635
CB4856 CT IV 9108931 9109753 WBGene00000120 aly-1 C01F6.5 0.132758511951623 AGIWUR13547
CB4856 CT IV 9119098 9130378 WBGene00006438 nrfl-1 C01F6.6 -0.16224425164638
AGIWUR548;AGIWUR8424;AGIWUR27484;AGIWUR31768;AGIWUR33238;AGIWUR34798;AGIWUR37061
CB4856 CT IV 9132600 9135477 WBGene00009073 delm-1 F23B2.3 0.0515916960420313
AGIWUR2991;AGIWUR25145;AGIWUR25402
CB4856 CT IV 9136933 9146210 WBGene00000906 daf-10 F23B2.4 -0.136501869311353 AGIWUR2652
CB4856 CT IV 9146337 9146869 WBGene00009078 rpb-12 F23B2.13 -0.189606626672905
AGIWUR18561
CB4856 CT IV 9147468 9148579 WBGene00000121 aly-2 F23B2.6 -0.315898149632986 AGIWUR995
CB4856 CT IV 9148949 9149297 WBGene00043283 F23B2.9 F23B2.9 0.0518765981179925 AGIWUR32127
CB4856 CT IV 9150491 9152043 WBGene00009075 F23B2.7 F23B2.7 -0.119590675991432 AGIWUR26059
CB4856 CT IV 9152819 9154320 WBGene00009077 F23B2.10 F23B2.10 0.133087558645226
AGIWUR26346;AGIWUR39763
CB4856 CT IV 9155079 9159019 WBGene00003958 pcp-3 F23B2.11 -0.668561778690179
AGIWUR14667
CB4856 CT IV 9160640 9165127 WBGene00003957 pcp-2 F23B2.12 -0.539439529561168
AGIWUR25329
CB4856 CT IV 9185064 9186023 WBGene00007411 C07C7.1 C07C7.1 0.162984348166098 AGIWUR28190
CB4856 CT IV 9186815 9191122 WBGene00008112 C46C2.4 C46C2.4 0.064084015661896 AGIWUR34546

CB4856	CT	IV	9191639	9192622	WBGene00008113	C46C2.5	C46C2.5	-0.742148887812096	AGIWUR15595
CB4856	CT	IV	9200102	9212235	WBGene00006941	wnk-1	C46C2.1	0.17012283143784	AGIWUR29399
CB4856	CT	IV	9212375	9214236	WBGene00008114	C46C2.6	C46C2.6	-0.302514326615415	AGIWUR1073;AGIWUR10519;AGIWUR11852;AGIWUR15701;AGIWUR29304;AGIWUR30848
CB4856	CT	IV	9216952	9220397	WBGene00008110	C46C2.2	C46C2.2	0.160722513442526	AGIWUR14022
CB4856	CT	IV	9225125	9227781	WBGene00008111	C46C2.3	C46C2.3	-0.264078947188204	AGIWUR26119
CB4856	CT	IV	9228277	9231051	WBGene00012426	Y11D7A.1	Y11D7A.1	-0.02715701031455	AGIWUR3515;AGIWUR25965
CB4856	CT	IV	9233028	9241387	WBGene00012428	Y11D7A.3	Y11D7A.3	-0.0368335327309293	AGIWUR29448
CB4856	CT	IV	9242225	9243999	WBGene00004281	rab-28	Y11D7A.4	0.70024457594412	AGIWUR8803
CB4856	CT	IV	9244520	9245523	WBGene00012429	Y11D7A.5	Y11D7A.5	-0.165568273707932	AGIWUR33747;AGIWUR38826
CB4856	CT	IV	9247156	9248088	WBGene00014863	Y11D7A.16	Y11D7A.16	-0.21990979405891	AGIWUR12333;AGIWUR24561
CB4856	CT	IV	9251379	9253913	WBGene00012432	Y11D7A.8	Y11D7A.8	-0.176194855828508	AGIWUR23676;AGIWUR37799;AGIWUR40803
CB4856	CT	IV	9253979	9256117	WBGene00012433	Y11D7A.9	Y11D7A.9	-0.108419536875227	AGIWUR1652
CB4856	CT	IV	9258913	9259764	WBGene00012434	Y11D7A.10	Y11D7A.10	-0.0349848355297304	AGIWUR39649
CB4856	CT	IV	9260610	9261787	WBGene00000694	col-120	Y11D7A.11	-0.268140131726188	AGIWUR34720
CB4856	CT	IV	9261808	9264265	WBGene00012435	flh-1	Y11D7A.12	-0.0293251075913723	AGIWUR26190
CB4856	CT	IV	9270096	9271935	WBGene00012436	flh-3	Y11D7A.13	-0.0660922345664012	AGIWUR12856
CB4856	CT	IV	9272582	9273639	WBGene00045309	srg-52	Y11D7A.18	0.0252032381154157	AGIWUR14678;AGIWUR16760
CB4856	CT	IV	9274416	9283113	WBGene00012437	hum-9	Y11D7A.14	-0.233473146474192	AGIWUR20979
CB4856	CT	IV	9292296	9293925	WBGene00008778	nhr-264	F14A5.1	-1.00333315802997	AGIWUR42246
CB4856	CT	IV	9294899	9296406	WBGene00009871	F49C12.1	F49C12.1	-0.0896419053606579	AGIWUR18302
CB4856	CT	IV	9298024	9299220	WBGene00009872	F49C12.2	F49C12.2	-0.0995697410621599	AGIWUR7653
CB4856	CT	IV	9300427	9301951	WBGene00009873	F49C12.3	F49C12.3	0.327646117779431	AGIWUR10306

CB4856	CT	IV	9302665	9304094	WBGene00009874	F49C12.4	F49C12.4	-0.381448714222574	AGIWUR44441
CB4856	CT	IV	9304917	9307165	WBGene00009875	F49C12.5	F49C12.5	-0.155522175028612	AGIWUR36045
CB4856	CT	IV	9307338	9309109	WBGene00009876	F49C12.6	F49C12.6	0.0541760825573085	AGIWUR22142
CB4856	CT	IV	9309108	9312927	WBGene00009877	F49C12.7	F49C12.7	0.808645618177717	AGIWUR25176
CB4856	CT	IV	9314810	9316215	WBGene00009878	F49C12.9	F49C12.9	0.00550652790659534	AGIWUR30770
CB4856	CT	IV	9316639	9318632	WBGene00004463	rpn-7	F49C12.8	-0.226013235010585	AGIWUR30692;AGIWUR41894
CB4856	CT	IV	9319380	9320143	WBGene00009880	F49C12.11	F49C12.11	-0.0630886593397406	AGIWUR5414
CB4856	CT	IV	9320122	9321089	WBGene00009881	F49C12.12	F49C12.12	-0.135314680966472	AGIWUR29619
CB4856	CT	IV	9321801	9322528	WBGene00009882	vha-17	F49C12.13	0.041035619066462	AGIWUR10460;AGIWUR33594
CB4856	CT	IV	9324435	9327516	WBGene00009884	F49C12.15	F49C12.15	0.206877911547029	AGIWUR1749
CB4856	CT	IV	9328010	9328745	WBGene00011855	clec-183	T20D3.1	-0.284910330460647	AGIWUR27131
CB4856	CT	IV	9328973	9330855	WBGene00011856	T20D3.2	T20D3.2	-0.280993418103209	AGIWUR37376;AGIWUR43822
CB4856	CT	IV	9333342	9335916	WBGene00011857	T20D3.3	T20D3.3	-0.21603433480195	AGIWUR640;AGIWUR2173;AGIWUR11482;AGIWUR21374;AGIWUR24100;AGIWUR25036;AGIWUR34649
CB4856	CT	IV	9337044	9338567	WBGene00011858	T20D3.5	T20D3.5	-0.100889027421871	AGIWUR6055;AGIWUR15510;AGIWUR24330
CB4856	CT	IV	9338587	9339305	WBGene00011859	T20D3.6	T20D3.6	0.0389064993016319	AGIWUR10001
CB4856	CT	IV	9339403	9341790	WBGene00006931	vps-26	T20D3.7	-0.247977688172323	AGIWUR22148
CB4856	CT	IV	9341964	9343152	WBGene00011860	T20D3.8	T20D3.8	0.0249230711624051	AGIWUR6522
CB4856	CT	IV	9343358	9366506	WBGene00007505	pezo-1	C10C5.1	-0.151322012287498	AGIWUR3741;AGIWUR15461;AGIWUR19565;AGIWUR37952
CB4856	CT	IV	9371508	9373636	WBGene00007506	C10C5.2	C10C5.2	-0.0492054365451455	AGIWUR14726;AGIWUR25513
CB4856	CT	IV	9374733	9376492	WBGene00007507	C10C5.3	C10C5.3	0.272633908259987	AGIWUR3415
CB4856	CT	IV	9378552	9380450	WBGene00007508	C10C5.4	C10C5.4	0.588525839880859	AGIWUR32390
CB4856	CT	IV	9381515	9383115	WBGene00007509	C10C5.5	C10C5.5	0.659961007110583	AGIWUR3133
CB4856	CT	IV	9385126	9385713	WBGene00007511	C10C5.7	C10C5.7	0.284963034107443	AGIWUR28883

CB4856	CT	IV	9386208	9394930	WBGene00000911	daf-15	C10C5.6	0.171996837838485
AGIWUR15043;AGIWUR24389
CB4856	CT	IV	9394934	9396218	WBGene00000695	col-121	F56D5.1	1.5431070741227	AGIWUR43719
CB4856	CT	IV	9396920	9398603	WBGene00010147	F56D5.2	F56D5.2	0.160529835625726	AGIWUR29719
CB4856	CT	IV	9399653	9401293	WBGene00010148	F56D5.3	F56D5.3	-0.595011138673566	AGIWUR947
CB4856	CT	IV	9401922	9412653	WBGene00010150	F56D5.6	F56D5.6	0.240844998969227	AGIWUR16853
CB4856	CT	IV	9418432	9422506	WBGene00010152	F56D5.9	F56D5.9	-0.473772012634928	AGIWUR9437
CB4856	CT	IV	9423395	9424415	WBGene00010153	srxa-2	F56D5.10	-0.117684921540384
AGIWUR11246
CB4856	CT	IV	9428259	9439077	WBGene00010316	F59B8.1	F59B8.1	0.0682673888768788
AGIWUR9633;AGIWUR25189;AGIWUR27633
CB4856	CT	IV	9439043	9440893	WBGene00010317	idh-1	F59B8.2	-0.104187044252082	AGIWUR21726
CB4856	CT	IV	9442877	9444516	WBGene00009544	F38E11.9	F38E11.9	-0.180115169124084
AGIWUR6817
CB4856	CT	IV	9444791	9445507	WBGene00002012	hsp-12.3	F38E11.1	0.206630482716522
AGIWUR5661;AGIWUR7122;AGIWUR9794;AGIWUR13490;AGIWUR20005;AGIWUR20124
CB4856	CT	IV	9446385	9446884	WBGene00002013	hsp-12.6	F38E11.2	0.907985627338759
AGIWUR6281;AGIWUR11904;AGIWUR17211;AGIWUR25673;AGIWUR30513;AGIWUR35348
CB4856	CT	IV	9449473	9457501	WBGene00009541	cutl-17	F38E11.4	0.125531151452811
AGIWUR12258;AGIWUR20388;AGIWUR31001;AGIWUR36293
CB4856	CT	IV	9459731	9463998	WBGene00009542	copb-2	F38E11.5	-0.0847128774477293
AGIWUR4755;AGIWUR16599;AGIWUR26454;AGIWUR34313;AGIWUR34549;AGIWUR40798
CB4856	CT	IV	9463951	9467518	WBGene00009543	F38E11.6	F38E11.6	0.277768825780099
AGIWUR2349
CB4856	CT	IV	9473680	9475365	WBGene00014752	F38E11.8	F38E11.8	0.042654815319132
AGIWUR40862
CB4856	CT	IV	9475863	9477446	WBGene00006949	wrt-3	F38E11.7	-0.518111919195475
AGIWUR3094;AGIWUR9220;AGIWUR33893;AGIWUR39063
CB4856	CT	IV	9482053	9485140	WBGene00007887	gadr-2	C33A12.12	-0.0787162873390823
AGIWUR36875
CB4856	CT	IV	9486272	9487631	WBGene00005665	sru-2	C33A12.13	0.0761526547995448
AGIWUR3401;AGIWUR6766;AGIWUR23330
CB4856	CT	IV	9488670	9490194	WBGene00005664	sru-1	C33A12.14	0.117424566748294
AGIWUR1098
CB4856	CT	IV	9490620	9491991	WBGene00005669	sru-6	C33A12.8	-0.135124742130753
AGIWUR6449
CB4856	CT	IV	9492576	9493220	WBGene00014693	C33A12.18	C33A12.18	-0.179663587205811
AGIWUR3246;AGIWUR34967
CB4856	CT	IV	9494032	9495519	WBGene00005668	sru-5	C33A12.9	0.0154074407346667

AGIWUR15262;AGIWUR38061
CB4856 CT IV 9496606 9497888 WBGene00005666 sru-3 C33A12.11 -0.225417735525701
AGIWUR19248
CB4856 CT IV 9498388 9499588 WBGene00005667 sru-4 C33A12.10 0.0419883277027138
AGIWUR18393
CB4856 CT IV 9500311 9502568 WBGene00007885 ugt-21 C33A12.6 -0.561524569778838
AGIWUR7536;AGIWUR29751
CB4856 CT IV 9502572 9505292 WBGene00007889 C33A12.16 C33A12.16 0.071169152391521
AGIWUR21842;AGIWUR26855;AGIWUR30931
CB4856 CT IV 9505615 9506326 WBGene00007888 ttr-9 C33A12.15 -0.106682473370161
AGIWUR33075
CB4856 CT IV 9506936 9508779 WBGene00007886 ethe-1 C33A12.7 -0.0605502628169496
AGIWUR27815
CB4856 CT IV 9510055 9510594 WBGene00007883 C33A12.4 C33A12.4 0.441503324989778
AGIWUR14783
CB4856 CT IV 9510748 9513002 WBGene00007882 C33A12.3 C33A12.3 0.19535668485311
AGIWUR38679
CB4856 CT IV 9516992 9518410 WBGene00007890 C33A12.19 C33A12.19 0.0143066038286044
AGIWUR12496
CB4856 CT IV 9518367 9518927 WBGene00007881 nlp-35 C33A12.2 0.0734795523651908
AGIWUR14739
CB4856 CT IV 9523764 9524895 WBGene00007880 C33A12.1 C33A12.1 0.0279561234979102
AGIWUR15621
CB4856 CT IV 9526359 9528350 WBGene00001239 elo-1 F56H11.4 -0.166360876584919
AGIWUR19591;AGIWUR20671;AGIWUR26778
CB4856 CT IV 9528355 9529569 WBGene00010176 F56H11.6 F56H11.6 -0.137252326488334
AGIWUR44359
CB4856 CT IV 9529947 9531378 WBGene00001245 elo-7 F56H11.3 0.0203132772247692
AGIWUR10588;AGIWUR38339;AGIWUR44859
CB4856 CT IV 9531357 9533171 WBGene00010175 F56H11.2 F56H11.2 0.940283652233695
AGIWUR38244
CB4856 CT IV 9540808 9546250 WBGene00001403 fbl-1 F56H11.1 -0.344943109871451
AGIWUR3088;AGIWUR21630;AGIWUR27626;AGIWUR31406;AGIWUR35333;AGIWUR35815
CB4856 CT IV 9546286 9560428 WBGene00011456 T05A1.5 T05A1.5 -0.0199669946883265
AGIWUR30574;AGIWUR36667;AGIWUR36862
CB4856 CT IV 9568828 9580071 WBGene00003808 npr-2 T05A1.1 0.491707657123477 AGIWUR9649
CB4856 CT IV 9587100 9588834 WBGene00009186 trcs-1 F27C8.6 -0.0857501823624496
AGIWUR23993;AGIWUR24542;AGIWUR43108
CB4856 CT IV 9589795 9592529 WBGene00009185 F27C8.5 F27C8.5 0.167204153850705 AGIWUR25567

CB4856	CT	IV	9593667	9594425	WBGene00005003	spp-18	F27C8.4	0.584855170547023	AGIWUR21130
CB4856	CT	IV	9595088	9595933	WBGene00009184	F27C8.3	F27C8.3	0.0692905854013981	AGIWUR17532;AGIWUR18902;AGIWUR31320;AGIWUR33452;AGIWUR35776
CB4856	CT	IV	9596497	9599081	WBGene00009183	F27C8.2	F27C8.2	-0.19455534289105	AGIWUR11750;AGIWUR19540;AGIWUR21046;AGIWUR26418
CB4856	CT	IV	9598986	9601695	WBGene00000002	aat-1	F27C8.1	0.0569794378772936	AGIWUR12999
CB4856	CT	IV	9614970	9620140	WBGene00001249	elt-1	W09C2.1	0.0548981661042713	AGIWUR18559
CB4856	CT	IV	9625910	9634948	WBGene00003151	mca-1	W09C2.3	0.152528400851368	AGIWUR8635;AGIWUR13889;AGIWUR15173;AGIWUR23235;AGIWUR27015;AGIWUR29670
CB4856	CT	IV	9636980	9642003	WBGene00000953	del-2	F58G6.6	0.368690950098481	AGIWUR33261
CB4856	CT	IV	9643422	9644268	WBGene00010278	F58G6.8	F58G6.8	0.372709180091459	AGIWUR25483
CB4856	CT	IV	9644245	9647934	WBGene00010272	amph-1	F58G6.1	-0.0523108914553845	AGIWUR8192;AGIWUR9748;AGIWUR15924;AGIWUR32381;AGIWUR43634
CB4856	CT	IV	9649043	9650490	WBGene00010273	srm-3	F58G6.2	-0.346820052344594	AGIWUR28922;AGIWUR39645
CB4856	CT	IV	9651394	9652484	WBGene00044107	F58G6.9	F58G6.9	-0.934690871860061	AGIWUR735;AGIWUR3220;AGIWUR14260;AGIWUR23851;AGIWUR29022
CB4856	CT	IV	9653535	9654175	WBGene00010274	F58G6.3	F58G6.3	-1.23832447805706	AGIWUR40504
CB4856	CT	IV	9654317	9655588	WBGene00010277	F58G6.7	F58G6.7	-0.780966059564973	AGIWUR22873
CB4856	CT	IV	9658053	9663941	WBGene00010275	acc-1	F58G6.4	-0.154097341544784	AGIWUR29262
CB4856	CT	IV	9664365	9675672	WBGene00003627	nhr-34	F58G6.5	-0.0843529228659774	AGIWUR3607;AGIWUR9087;AGIWUR9865;AGIWUR17700;AGIWUR20213;AGIWUR38487;AGIWUR39401;AGIWUR43484
CB4856	CT	IV	9680387	9680941	WBGene00002091	ins-8	ZK1251.11	0.53517216227487	AGIWUR12964
CB4856	CT	IV	9682013	9682826	WBGene00002090	ins-7	ZK1251.2	-0.211088341947743	AGIWUR35925
CB4856	CT	IV	9682984	9683533	WBGene00014240	htas-1	ZK1251.1	0.280460638320688	AGIWUR13934
CB4856	CT	IV	9683617	9684473	WBGene00014241	ZK1251.3	ZK1251.3	-0.396144042662621	AGIWUR19628
CB4856	CT	IV	9687049	9688392	WBGene00014996	ZK1251.5	ZK1251.5	0.0162719608157187	AGIWUR16631;AGIWUR36524
CB4856	CT	IV	9690932	9691465	WBGene00014242	sdz-36	ZK1251.7	-0.0582195370678491	AGIWUR42072
CB4856	CT	IV	9693202	9697005	WBGene00006664	twk-9	ZK1251.8	-0.0385786300457323	AGIWUR5316;AGIWUR12010;AGIWUR41514
CB4856	CT	IV	9696989	9706723	WBGene00014243	dcaf-1	ZK1251.9	0.154994314002291	AGIWUR40370
CB4856	CT	IV	9707148	9707764	WBGene00010484	dph-3	K01H12.1	0.190379009216441	

AGIWUR11984;AGIWUR15311;AGIWUR21907;AGIWUR29882;AGIWUR39442;AGIWUR44342
CB4856 CT IV 9708170 9709305 WBGene00010485 ant-1.3 K01H12.2 0.0761298975109366
AGIWUR22865
CB4856 CT IV 9710202 9712419 WBGene00003629 nhr-38 K01H12.3 0.134212589922729
AGIWUR15627;AGIWUR30641;AGIWUR42905
CB4856 CT IV 9713082 9714886 WBGene00010486 K01H12.4 K01H12.4 0.285311202145067
AGIWUR42932
CB4856 CT IV 9725251 9728576 WBGene00003728 nhr-138 C28D4.9 0.0178638835712288 AGIWUR5703
CB4856 CT IV 9728676 9731314 WBGene00003690 nhr-100 C28D4.1 -0.0307843284231772 AGIWUR14259
CB4856 CT IV 9731737 9737621 WBGene00000509 cka-1 C28D4.2 0.136995285830402 AGIWUR39127
CB4856 CT IV 9738510 9739220 WBGene00007796 C28D4.10 C28D4.10 0.0670981832218578
AGIWUR28516
CB4856 CT IV 9739219 9740908 WBGene00001607 gln-6 C28D4.3 -0.0315598734377467
AGIWUR8252;AGIWUR27183;AGIWUR32269
CB4856 CT IV 9741875 9743147 WBGene00007794 C28D4.7 C28D4.7 -0.00362338507431836 AGIWUR7363
CB4856 CT IV 9743765 9745003 WBGene00007792 C28D4.4 C28D4.4 -0.0590712435928555 AGIWUR43339
CB4856 CT IV 9745555 9746862 WBGene00007793 C28D4.5 C28D4.5 -0.244999354121661
AGIWUR8420;AGIWUR34963
CB4856 CT IV 9757648 9764494 WBGene00003687 nhr-97 H27C11.1 0.0355881443517809
AGIWUR10437;AGIWUR25208;AGIWUR26811;AGIWUR42455;AGIWUR44637
CB4856 CT IV 9765669 9766436 WBGene00011749 ssp-36 T13F2.12 0.270372182805916
AGIWUR11597
CB4856 CT IV 9772510 9773470 WBGene00000301 cav-1 T13F2.8 0.590279016484306
AGIWUR3025;AGIWUR6303;AGIWUR17426;AGIWUR24603;AGIWUR43155
CB4856 CT IV 9774563 9777634 WBGene00011747 sna-2 T13F2.7 -0.0140703807063698 AGIWUR32315
CB4856 CT IV 9778288 9781203 WBGene00011746 T13F2.6 T13F2.6 -0.0478882112769359 AGIWUR39516
CB4856 CT IV 9789808 9791917 WBGene00011744 T13F2.4 T13F2.4 0.282264050674569 AGIWUR39540
CB4856 CT IV 9791945 9796592 WBGene00004031 pis-1 T13F2.3 -0.0486466566662997 AGIWUR17460
CB4856 CT IV 9796688 9797354 WBGene00011743 T13F2.2 T13F2.2 -0.163083523529689
AGIWUR2648;AGIWUR40457
CB4856 CT IV 9799429 9802638 WBGene00001396 fat-4 T13F2.1 0.596829988341856
AGIWUR13144;AGIWUR23506;AGIWUR33115
CB4856 CT IV 9803023 9806119 WBGene00001395 fat-3 W08D2.4 1.73596006476699 AGIWUR19711
CB4856 CT IV 9806416 9810733 WBGene00012340 dct-15 W08D2.3 -0.0955598001187337 AGIWUR20257
CB4856 CT IV 9812444 9815526 WBGene00001188 egl-20 W08D2.1 0.011239329782557
AGIWUR16385;AGIWUR23531;AGIWUR27673;AGIWUR33623
CB4856 CT IV 9817412 9824632 WBGene00012341 catp-6 W08D2.5 -0.0490028025269464
AGIWUR6997;AGIWUR17095;AGIWUR22958;AGIWUR43986
CB4856 CT IV 9825694 9830620 WBGene00012342 mtr-4 W08D2.7 0.02739390360437 AGIWUR36980

CB4856 CT IV 9832935 9834822 WBGene00002204 kin-21 W08D2.8 -0.256635262273399 AGIWUR37340
CB4856 CT IV 9839057 9841084 WBGene00002206 kin-24 K07F5.4 -0.043683869102435 AGIWUR26938
CB4856 CT IV 9842371 9842992 WBGene00045180 K07F5.17 K07F5.17 -0.0477268008378502
AGIWUR7217;AGIWUR9095;AGIWUR18583
CB4856 CT IV 9844014 9844344 WBGene00010633 nspd-2 K07F5.5 0.359555081652086 AGIWUR17317
CB4856 CT IV 9844989 9846674 WBGene00010634 K07F5.6 K07F5.6 -0.0378862003090319 AGIWUR23014
CB4856 CT IV 9847724 9848175 WBGene00010635 K07F5.7 K07F5.7 -0.035796420800323 AGIWUR25972
CB4856 CT IV 9848491 9849796 WBGene00010636 K07F5.8 K07F5.8 -0.373826230552573
AGIWUR2188;AGIWUR2399;AGIWUR24222
CB4856 CT IV 9852550 9853609 WBGene00006050 ssq-1 K07F5.11 0.614772120132525
AGIWUR10720
CB4856 CT IV 9856012 9858067 WBGene00010637 K07F5.12 K07F5.12 -0.436994962467346
AGIWUR15523;AGIWUR22601
CB4856 CT IV 9858488 9862118 WBGene00003787 npp-1 K07F5.13 -0.207533740428705
AGIWUR8201;AGIWUR16295;AGIWUR35932
CB4856 CT IV 9862221 9864076 WBGene00010638 K07F5.14 K07F5.14 -0.163989831681565
AGIWUR39713
CB4856 CT IV 9864179 9864954 WBGene00010640 K07F5.16 K07F5.16 0.268288590057027
AGIWUR43013
CB4856 CT IV 9865078 9866163 WBGene00010639 K07F5.15 K07F5.15 0.011997429669421
AGIWUR2940;AGIWUR3047;AGIWUR17907;AGIWUR19206;AGIWUR41539
CB4856 CT IV 9879329 9882152 WBGene00003603 nhr-4 F32B6.1 0.00686852236504798 AGIWUR40095
CB4856 CT IV 9882132 9884383 WBGene00009320 F32B6.3 F32B6.3 -0.216447320249662 AGIWUR38724
CB4856 CT IV 9884556 9887150 WBGene00009319 mccc-1 F32B6.2 -0.274731693280894
AGIWUR14175;AGIWUR25051;AGIWUR30913;AGIWUR44689
CB4856 CT IV 9887190 9889929 WBGene00009324 F32B6.10 F32B6.10 0.039579178578582
AGIWUR12556
CB4856 CT IV 9890585 9892038 WBGene00009321 F32B6.4 F32B6.4 -0.0304920319852311 AGIWUR10939
CB4856 CT IV 9892739 9893779 WBGene00006056 sss-1 F32B6.5 0.308894708874313 AGIWUR38254
CB4856 CT IV 9895388 9896147 WBGene00006049 ssp-32 F32B6.7 -0.211248765138771 AGIWUR1266
CB4856 CT IV 9899012 9902513 WBGene00009322 tbc-3 F32B6.8 -0.115532872727195 AGIWUR19284
CB4856 CT IV 9903908 9905903 WBGene00009323 best-13 F32B6.9 -0.152347208188525 AGIWUR3199
CB4856 CT IV 9916530 9921996 WBGene00009133 bed-3 F25H8.6 0.0275089104308925 AGIWUR41049
CB4856 CT IV 9921955 9928223 WBGene00001113 dur-1 F25H8.5 1.41091814586444
AGIWUR18088;AGIWUR37175;AGIWUR44758
CB4856 CT IV 9930899 9932240 WBGene00009131 F25H8.1 F25H8.1 -0.192286177963172
AGIWUR3558;AGIWUR13798;AGIWUR38273
CB4856 CT IV 9932235 9933879 WBGene00009132 F25H8.2 F25H8.2 -0.249602719566797 AGIWUR23509
CB4856 CT IV 9933910 9934285 WBGene00004974 spe-29 F25H8.7 -0.274401664576876

AGIWUR12135;AGIWUR14054;AGIWUR16647;AGIWUR19696;AGIWUR25093;AGIWUR25417
CB4856	CT	IV	9934767 9953433 WBGene00001650	gon-1	F25H8.3 -0.267822416483993
AGIWUR15816;AGIWUR29887;AGIWUR40536
CB4856	CT	IV	9964245 9966630 WBGene00011762	T13H10.2	T13H10.2	0.181297816234586
AGIWUR3038;AGIWUR9024;AGIWUR17513;AGIWUR18569
CB4856	CT	IV	9967095 9969181 WBGene00002193	kin-5	T13H10.1	0.0357069294388488
AGIWUR4224
CB4856	CT	IV	9969400 9971200 WBGene00008154	C47E12.11	C47E12.11	-0.207193262390433
AGIWUR13467
CB4856	CT	IV	9972037 9972652 WBGene00008155	C47E12.12	C47E12.12	0.0598154868784171
AGIWUR27207
CB4856	CT	IV	9972800 9973984 WBGene00008153	C47E12.10	C47E12.10
-0.00433469414117482	AGIWUR34366
CB4856	CT	IV	9974672 9975484 WBGene00008152	C47E12.9	C47E12.9	0.323082835037491
AGIWUR29301
CB4856	CT	IV	9975496 9976384 WBGene00044240	C47E12.14	C47E12.14	0.10001475463429
AGIWUR30214;AGIWUR33392;AGIWUR34516
CB4856	CT	IV	9978122 9980770 WBGene00000964	dhp-2	C47E12.8	0.368827603274539
AGIWUR34299
CB4856	CT	IV	9980724 9982276 WBGene00008151	C47E12.7	C47E12.7	-0.208991456869782
AGIWUR18656
CB4856	CT	IV	9983195 9989678 WBGene00008150	mam-2	C47E12.6	-0.24054910473718
AGIWUR29556
CB4856	CT	IV	9989670 9995034 WBGene00006699	uba-1	C47E12.5	-0.272725805204569
AGIWUR10534;AGIWUR11941;AGIWUR17729;AGIWUR18720;AGIWUR28621;AGIWUR29207;AGIWUR32686;AGIWUR34505;AGIWUR35578
;AGIWUR41605
CB4856	CT	IV	9995472 9999032 WBGene00008149	pyp-1	C47E12.4	0.175609883090253
AGIWUR3161;AGIWUR26414
CB4856	CT	IV	9999047 10001846	WBGene00008148	C47E12.3	C47E12.3
-0.246066689902245	AGIWUR23502
CB4856	CT	IV	10001972	10003534	WBGene00008147	C47E12.2	C47E12.2
-0.2227367644272	AGIWUR44403
CB4856	CT	IV	10003726	10005599	WBGene00005663	sars-1	C47E12.1
-0.215548763165321	AGIWUR1796;AGIWUR5322;AGIWUR11890;AGIWUR31242
CB4856	CT	IV	10009354	10014187	WBGene00009678	magu-4	F44D12.1
-0.0393247768952101	AGIWUR3382
CB4856	CT	IV	10013958	10016398	WBGene00009679	F44D12.2	F44D12.2
0.10106074805703	AGIWUR6307
CB4856	CT	IV	10017425	10021286	WBGene00009686	ent-7	F44D12.9

-0.594526353835699	AGIWUR21559
CB4856	CT	IV	10023291	10025226	WBGene00009685	F44D12.8	F44D12.8
0.299431041394049	AGIWUR19688
CB4856	CT	IV	10026413	10027666	WBGene00009681	gipc-2	F44D12.4
-0.112635558238733	AGIWUR34255
CB4856	CT	IV	10032972	10033155	WBGene00045002	F44D12.12	F44D12.12
0.260307075338369	AGIWUR20102
CB4856	CT	IV	10033363	10034820	WBGene00009683	F44D12.6	F44D12.6
-0.0629347244607459	AGIWUR8676;AGIWUR30156;AGIWUR38109
CB4856	CT	IV	10036627	10037066	WBGene00009687	F44D12.10	F44D12.10
0.00387716518522652
AGIWUR7081;AGIWUR7294;AGIWUR15394;AGIWUR18459;AGIWUR19867;AGIWUR22633;AGIWUR27241;AGIWUR33146;AGIWUR34661;AGIWUR40121;AGIWUR40462
CB4856	CT	IV	10037452	10039043	WBGene00014375	F44D12.11	F44D12.11
-0.0846580560570942	AGIWUR25592
CB4856	CT	IV	10039773	10040903	WBGene00011424	dhs-31	T04B2.6	-0.0716075374717313
AGIWUR19849;AGIWUR41560
CB4856	CT	IV	10041130	10042432	WBGene00011421	T04B2.1	T04B2.1	0.092615958117658
AGIWUR7713;AGIWUR10342;AGIWUR36383;AGIWUR38194
CB4856	CT	IV	10043183	10045739	WBGene00001487	frk-1	T04B2.2	-0.2662836843358
AGIWUR40759
CB4856	CT	IV	10047408	10050554	WBGene00011422	T04B2.3	T04B2.3	-0.466116082135006
AGIWUR18224
CB4856	CT	IV	10050581	10051188	WBGene00044018	T04B2.8	T04B2.8	-0.0547034128458783
AGIWUR4874;AGIWUR8285;AGIWUR24191;AGIWUR35191;AGIWUR38908
CB4856	CT	IV	10051286	10053650	WBGene00005723	srv-12	T04B2.4	0.0495630986020146
AGIWUR15022
CB4856	CT	IV	10054036	10056250	WBGene00011425	T04B2.7	T04B2.7	0.00735935797628606
AGIWUR36502
CB4856	CT	IV	10056591	10062849	WBGene00011423	ipla-7	T04B2.5	0.204932657875299
AGIWUR712;AGIWUR11375;AGIWUR21307;AGIWUR21939;AGIWUR30091;AGIWUR33006;AGIWUR41031;AGIWUR44901
CB4856	CT	IV	10067824	10070251	WBGene00000858	cwn-2	W01B6.1	0.58269048795794
AGIWUR9085;AGIWUR12598;AGIWUR15419;AGIWUR28973;AGIWUR37317
CB4856	CT	IV	10070270	10072626	WBGene00012170	W01B6.3	W01B6.3	-0.272532635113917
AGIWUR22414
CB4856	CT	IV	10075414	10075775	WBGene00012171	W01B6.4	W01B6.4	-0.306094920186791
AGIWUR5168
CB4856	CT	IV	10076325	10078705	WBGene00012172	W01B6.5	W01B6.5	-0.0446795415171888
AGIWUR27903

CB4856 CT IV 10081155 10082610 WBGene00012173 W01B6.6 W01B6.6 -0.100653038536117
AGIWUR5015;AGIWUR41383
CB4856 CT IV 10082867 10082962 WBGene00045182 W01B6.10 W01B6.10
0.0952616193471947 AGIWUR29759
CB4856 CT IV 10083491 10084440 WBGene00012174 W01B6.8 W01B6.8 -0.257078066557929
AGIWUR34753
CB4856 CT IV 10084454 10085438 WBGene00000593 col-2 W01B6.7 -0.0988088022672349
AGIWUR30833
CB4856 CT IV 10086137 10088437 WBGene00003576 ndc-80 W01B6.9 -0.17786582034876
AGIWUR293;AGIWUR3177;AGIWUR28049
CB4856 CT IV 10088367 10089894 WBGene00007304 ttr-39 C04G2.1 0.164743205443784
AGIWUR35819
CB4856 CT IV 10090658 10092379 WBGene00007305 C04G2.2 C04G2.2 -0.0832703418002758
AGIWUR3541
CB4856 CT IV 10094198 10095259 WBGene00007306 C04G2.5 C04G2.5 0.114494953412301
AGIWUR33346
CB4856 CT IV 10097196 10106556 WBGene00001001 dis-3 C04G2.6 -0.26195733555234
AGIWUR33485
CB4856 CT IV 10106917 10110412 WBGene00001204 egl-38 C04G2.7 -0.147915584573702
AGIWUR43499
CB4856 CT IV 10110347 10111577 WBGene00007307 spch-1 C04G2.8 0.1188196214943
AGIWUR2944
CB4856 CT IV 10111721 10112028 WBGene00045104 C04G2.12 C04G2.12
-0.023728039615198 AGIWUR10102;AGIWUR30391
CB4856 CT IV 10112870 10114287 WBGene00007308 C04G2.9 C04G2.9 0.253144068432023
AGIWUR3561
CB4856 CT IV 10115389 10118084 WBGene00007309 C04G2.10 C04G2.10
-0.354601733669889 AGIWUR41110
CB4856 CT IV 10119460 10122305 WBGene00007310 irld-21 C04G2.11
-0.278932088575456 AGIWUR43001
CB4856 CT IV 10122120 10126675 WBGene00010676 K08F4.1 K08F4.1 -0.247530484701823
AGIWUR6421;AGIWUR24373;AGIWUR44754
CB4856 CT IV 10126749 10128956 WBGene00010677 gtbp-1 K08F4.2 -0.122538786325057
AGIWUR444;AGIWUR1140;AGIWUR31820;AGIWUR42248
CB4856 CT IV 10129257 10130244 WBGene00010678 K08F4.3 K08F4.3 -0.32459239658731
AGIWUR6612
CB4856 CT IV 10130257 10133283 WBGene00001621 glt-3 K08F4.4 0.150217431367217
AGIWUR13198
CB4856 CT IV 10134932 10135887 WBGene00010679 K08F4.5 K08F4.5 0.202937720587256

AGIWUR28659
CB4856 CT IV 10138124 10139104 WBGene00010680 K08F4.12 K08F4.12
-0.0747475510592075 AGIWUR43473
CB4856 CT IV 10139380 10140285 WBGene00001751 gst-3 K08F4.11
0.269850178805253 AGIWUR15799
CB4856 CT IV 10145249 10146222 WBGene00000975 dhs-12 K08F4.9 -0.318028531925419
AGIWUR15183;AGIWUR15296;AGIWUR17821;AGIWUR17973
CB4856 CT IV 10146226 10147105 WBGene00011777 T14G10.8 T14G10.8
0.0445819712560134 AGIWUR34844
CB4856 CT IV 10147031 10149038 WBGene00011776 hpo-5 T14G10.7
-0.0493683798773041 AGIWUR43385
CB4856 CT IV 10149166 10150602 WBGene00006638 tsp-12 T14G10.6
0.157674145327379 AGIWUR33375
CB4856 CT IV 10151949 10155636 WBGene00011775 T14G10.5 T14G10.5
-0.0281463900030339 AGIWUR16222;AGIWUR42213;AGIWUR44173
CB4856 CT IV 10155775 10156771 WBGene00011774 ttr-54 T14G10.4
0.22410252723239 AGIWUR25565
CB4856 CT IV 10158038 10159243 WBGene00011773 ttr-53 T14G10.3
-0.102203370373903 AGIWUR43146
CB4856 CT IV 10162008 10164390 WBGene00004091 pps-1 T14G10.1
0.148422091075497 AGIWUR14039
CB4856 CT IV 10164331 10174341 WBGene00004254 pxf-1 T14G10.2
0.0120843124493426 AGIWUR18067;AGIWUR19827;AGIWUR21600;AGIWUR27870;AGIWUR36386
CB4856 CT IV 10176680 10178099 WBGene00010556 rack-1 K04D7.1 0.0369119133759845
AGIWUR2243;AGIWUR11655;AGIWUR14450;AGIWUR16648;AGIWUR24842;AGIWUR43131
CB4856 CT IV 10177968 10180304 WBGene00010557 mspn-1 K04D7.2 -0.250501949971635
AGIWUR29722;AGIWUR30885
CB4856 CT IV 10180660 10183939 WBGene00001794 gta-1 K04D7.3 -0.382528274611879
AGIWUR3779;AGIWUR5118;AGIWUR12585
CB4856 CT IV 10183851 10194424 WBGene00010558 ptp-4 K04D7.4 0.131791716797194
AGIWUR7030;AGIWUR11134;AGIWUR22388;AGIWUR24476;AGIWUR27283;AGIWUR40206;AGIWUR41498;AGIWUR42425
CB4856 CT IV 10197820 10202993 WBGene00001653 gon-4 K04D7.5 -0.197867615844459
AGIWUR17084
CB4856 CT IV 10203729 10203964 WBGene00045022 K04D7.7 K04D7.7 0.148621415734717
AGIWUR28428;AGIWUR30611
CB4856 CT IV 10204185 10206073 WBGene00010559 K04D7.6 K04D7.6 0.312905649946554
AGIWUR27252;AGIWUR44586
CB4856 CT IV 10206189 10214911 WBGene00000019 abt-1 C24F3.5 -0.230823880531471
AGIWUR25441

CB4856	CT	IV	10214986	10219308	WBGene00007698	qns-1	C24F3.4 0.00649010128977334
AGIWUR200;AGIWUR9742;AGIWUR16857;AGIWUR29306
CB4856	CT	IV	10219373	10221281	WBGene00007696	tram-1	C24F3.1 -0.170612115941881
AGIWUR296;AGIWUR3729;AGIWUR4771;AGIWUR13767;AGIWUR14881;AGIWUR20042;AGIWUR32443;AGIWUR32814
CB4856	CT	IV	10222472	10223745	WBGene00007697	C24F3.2 C24F3.2 0.0406942132409895
AGIWUR16729
CB4856	CT	IV	10224023	10226342	WBGene00003531	nas-12	C24F3.3 -0.197653207751657
AGIWUR9183;AGIWUR18486;AGIWUR23887;AGIWUR39103;AGIWUR42188;AGIWUR44445
CB4856	CT	IV	10226334	10227639	WBGene00008509	F01G10.6	F01G10.6
-0.0523693722001646	AGIWUR38080
CB4856	CT	IV	10227987	10231060	WBGene00008508	F01G10.5	F01G10.5
-0.27871418429032	AGIWUR18121
CB4856	CT	IV	10231375	10232552	WBGene00008507	F01G10.4	F01G10.4
0.480792887993564	AGIWUR16428
CB4856	CT	IV	10233321	10235329	WBGene00001158	ech-9	F01G10.3
0.334782804664945	AGIWUR33647
CB4856	CT	IV	10236626	10239176	WBGene00001157	ech-8	F01G10.2
0.0872941380804839	AGIWUR30960
CB4856	CT	IV	10239278	10241859	WBGene00008510	lipl-7	F01G10.7
0.144829748278471	AGIWUR3315;AGIWUR11327
CB4856	CT	IV	10243591	10245752	WBGene00008506	tkt-1	F01G10.1
-0.239374101200148	AGIWUR22815;AGIWUR24020;AGIWUR41119;AGIWUR43337
CB4856	CT	IV	10246160	10249785	WBGene00008512	F01G10.10	F01G10.10
-0.113912359034574	AGIWUR10003
CB4856	CT	IV	10250646	10255844	WBGene00000910	daf-14	F01G10.8
0.0748951062868919	AGIWUR20358
CB4856	CT	IV	10255755	10259657	WBGene00008511	F01G10.9	F01G10.9
0.00761818001983094	AGIWUR6188;AGIWUR9546;AGIWUR10240;AGIWUR14914;AGIWUR44928
CB4856	CT	IV	10268882	10269228	WBGene00012781	nspd-7	Y43C5A.1
-0.680914847741382	AGIWUR14919
CB4856	CT	IV	10269883	10272691	WBGene00012782	Y43C5A.2	Y43C5A.2
-0.314728079283247	AGIWUR16256;AGIWUR17505;AGIWUR22870;AGIWUR44812
CB4856	CT	IV	10274036	10275411	WBGene00012783	Y43C5A.3	Y43C5A.3
0.959935560242528	AGIWUR4497;AGIWUR10434;AGIWUR13683
CB4856	CT	IV	10277264	10277918	WBGene00044922	Y43C5A.7	Y43C5A.7
-0.718081832662628	AGIWUR34336
CB4856	CT	IV	10277989	10279501	WBGene00012784	Y43C5A.4	Y43C5A.4
-0.0198741556589592	AGIWUR28573
CB4856	CT	IV	10279457	10281582	WBGene00006568	thk-1	Y43C5A.5

0.0162680644455264	AGIWUR5016
CB4856	CT	IV	10281637	10284244	WBGene00004297	rad-51	Y43C5A.6
-0.0490388260688412	AGIWUR18645;AGIWUR33535;AGIWUR36015
CB4856	CT	IV	10286955	10293013	WBGene00004140	ebax-1	R09E10.7
-0.0052006276595371	AGIWUR43680
CB4856	CT	IV	10292928	10294130	WBGene00011176	R09E10.6	R09E10.6
0.48680901562161	AGIWUR21841
CB4856	CT	IV	10296046	10302310	WBGene00011175	R09E10.5	R09E10.5
0.748290320905231	AGIWUR27517
CB4856	CT	IV	10304845	10307880	WBGene00011174	acs-23	R09E10.4
-0.138024909656156	AGIWUR1309;AGIWUR21443;AGIWUR21599
CB4856	CT	IV	10310116	10310429	WBGene00011177	R09E10.8	R09E10.8
-0.747680303907057	AGIWUR29195
CB4856	CT	IV	10310772	10313670	WBGene00011173	acs-18	R09E10.3
0.561393460630732	AGIWUR13284
CB4856	CT	IV	10314139	10314576	WBGene00044254	R09E10.10	R09E10.10
0.0541950835970031	AGIWUR31311;AGIWUR33716;AGIWUR36979
CB4856	CT	IV	10314989	10317377	WBGene00011178	R09E10.9	R09E10.9
-0.256961374934295	AGIWUR3962
CB4856	CT	IV	10317558	10318293	WBGene00011172	R09E10.2	R09E10.2
-0.114011687728065	AGIWUR38681
CB4856	CT	IV	10319351	10321170	WBGene00011171	R09E10.1	R09E10.1
0.201810223510869	AGIWUR35003
CB4856	CT	IV	10324255	10350217	WBGene00006779	unc-43	K11E8.1
-0.00429559648718225
AGIWUR4340;AGIWUR6349;AGIWUR7878;AGIWUR7921;AGIWUR8651;AGIWUR9769;AGIWUR9871;AGIWUR10142;AGIWUR10176;AGIWUR
11503;AGIWUR12155;AGIWUR18114;AGIWUR20811;AGIWUR30344;AGIWUR37623;AGIWUR39280;AGIWUR39753;AGIWUR41763;AGIWU
R42419;AGIWUR44510
CB4856	CT	IV	10351445	10352343	WBGene00012785	Y43C5B.2	Y43C5B.2
-0.450348000756339	AGIWUR22600
CB4856	CT	IV	10353167	10355120	WBGene00012786	Y43C5B.3	Y43C5B.3
0.20572864354893	AGIWUR29043
CB4856	CT	IV	10355046	10357170	WBGene00011236	R11A8.1	R11A8.1	-0.148962700601377
AGIWUR20547
CB4856	CT	IV	10358141	10360219	WBGene00011237	gkow-1	R11A8.2	-0.20480850869097
AGIWUR16364
CB4856	CT	IV	10360800	10363331	WBGene00011238	ugt-59	R11A8.3	0.209940395557769
AGIWUR31384
CB4856	CT	IV	10366333	10367790	WBGene00011239	pges-2	R11A8.5	-0.378191805731291

AGIWUR18136
CB4856 CT IV 10368607 10373136 WBGene00002152 iars-1 R11A8.6 -0.278456307107291
AGIWUR822;AGIWUR2745;AGIWUR7114
CB4856 CT IV 10372638 10373821 WBGene00011241 mpz-5 R11A8.8 0.181228973797215
AGIWUR16355;AGIWUR25461
CB4856 CT IV 10374905 10387318 WBGene00011240 R11A8.7 R11A8.7 -0.117280321513599
AGIWUR902;AGIWUR3127;AGIWUR9042;AGIWUR10806;AGIWUR18248;AGIWUR18961;AGIWUR24502;AGIWUR29166;AGIWUR43502
CB4856 CT IV 10387413 10388345 WBGene00003064 lpd-8 R10H10.1
-0.357890811808865 AGIWUR20350
CB4856 CT IV 10388483 10393294 WBGene00011222 R10H10.3 R10H10.3
-0.438147319993888 AGIWUR6660;AGIWUR15226;AGIWUR31267
CB4856 CT IV 10394591 10394958 WBGene00011223 R10H10.4 R10H10.4
-0.14254085364123 AGIWUR22287
CB4856 CT IV 10404012 10408530 WBGene00001669 gpa-7 R10H10.5
-0.0476801405071186 AGIWUR26769
CB4856 CT IV 10408703 10409477 WBGene00011224 R10H10.6 R10H10.6
-0.134860841870243 AGIWUR6359
CB4856 CT IV 10409985 10413928 WBGene00011225 R10H10.7 R10H10.7
0.0989432282267444 AGIWUR12860
CB4856 CT IV 10414030 10417881 WBGene00008733 F13B12.6 F13B12.6
0.0903381023309926 AGIWUR8525;AGIWUR10975;AGIWUR19192
CB4856 CT IV 10422610 10424635 WBGene00008729 F13B12.1 F13B12.1
-0.0421012968385526 AGIWUR21607;AGIWUR33993;AGIWUR37529
CB4856 CT IV 10426231 10429110 WBGene00008730 F13B12.2 F13B12.2
-0.147950753983215 AGIWUR28816;AGIWUR37040;AGIWUR38086
CB4856 CT IV 10436708 10438410 WBGene00002084 ins-1 F13B12.5
0.146081900730819 AGIWUR33522;AGIWUR34508;AGIWUR37691
CB4856 CT IV 10439123 10443819 WBGene00008731 F13B12.3 F13B12.3
-0.0174774147412711 AGIWUR6185;AGIWUR43439
CB4856 CT IV 10444094 10444584 WBGene00008734 F13B12.7 F13B12.7
0.134057821289621 AGIWUR41881
CB4856 CT IV 10444583 10449899 WBGene00008732 F13B12.4 F13B12.4
-0.0552907803844036 AGIWUR16795
CB4856 CT IV 10450802 10453098 WBGene00008490 F01D4.8 F01D4.8
-0.00827539921689624 AGIWUR41212
CB4856 CT IV 10454432 10457237 WBGene00008489 srb-17 F01D4.7 0.153332640536146
AGIWUR4263;AGIWUR34342;AGIWUR35770
CB4856 CT IV 10458863 10461766 WBGene00003167 mec-3 F01D4.6 0.496099554234113
AGIWUR1570;AGIWUR4280;AGIWUR9851;AGIWUR12694

CB4856	CT	IV	10464588	10467240	WBGene00008485	ugt-43	F01D4.1	0.134319640829393	AGIWUR3944;AGIWUR9096;AGIWUR16273;AGIWUR17471;AGIWUR27317;AGIWUR30495
CB4856	CT	IV	10469741	10471899	WBGene00008486	ugt-44	F01D4.2	-0.281181496902118	AGIWUR4885
CB4856	CT	IV	10472778	10478168	WBGene00008491	bgnt-1.1	F01D4.9	0.0114587864700635	AGIWUR42817
CB4856	CT	IV	10478450	10481844	WBGene00001189	egl-21	F01D4.4	0.323321785872096	AGIWUR782;AGIWUR10945;AGIWUR21638;AGIWUR30012
CB4856	CT	IV	10482629	10483655	WBGene00008488	F01D4.5	F01D4.5	-0.0397695544008361	AGIWUR31233;AGIWUR39452
CB4856	CT	IV	10484110	10492952	WBGene00000419	ced-5	C02F4.1	-0.0481984785785068	AGIWUR14004;AGIWUR17614;AGIWUR20229;AGIWUR21408;AGIWUR36560;AGIWUR37218
CB4856	CT	IV	10493071	10501599	WBGene00006527	tax-6	C02F4.2	0.0885774114143922	AGIWUR16185;AGIWUR24485
CB4856	CT	IV	10513887	10515228	WBGene00007264	C02F4.4	C02F4.4	0.952463582973866	AGIWUR24466
CB4856	CT	IV	10521422	10530597	WBGene00000288	cal-4	T07G12.1	-0.0874263450543777	AGIWUR9574;AGIWUR15671;AGIWUR30680
CB4856	CT	IV	10533943	10536442	WBGene00011594	T07G12.3	T07G12.3	0.186815102714821	AGIWUR2713
CB4856	CT	IV	10541495	10545091	WBGene00011596	T07G12.5	T07G12.5	4.58730438037668	AGIWUR14244
CB4856	CT	IV	10547088	10555442	WBGene00011597	zim-1	T07G12.6	-0.108255275878657	AGIWUR9315;AGIWUR9412;AGIWUR20517;AGIWUR36426;AGIWUR43050;AGIWUR45065
CB4856	CT	IV	10555247	10558221	WBGene00011600	zim-2	T07G12.10	0.00853378355870049	AGIWUR17323
CB4856	CT	IV	10558721	10561130	WBGene00011601	zim-3	T07G12.11	-0.107331637301954	AGIWUR20579
CB4856	CT	IV	10561905	10563947	WBGene00001867	him-8	T07G12.12	-0.125590021605396	AGIWUR11669;AGIWUR17932;AGIWUR29184
CB4856	CT	IV	10576350	10578288	WBGene00001163	efn-2	C43F9.8	-0.128167746955947	AGIWUR576
CB4856	CT	IV	10578667	10581786	WBGene00008076	lgc-43	C43F9.9	-0.13007506761339	AGIWUR35328
CB4856	CT	IV	10584537	10587400	WBGene00008077	oac-8	C43F9.10	-0.167774515344175	AGIWUR11560
CB4856	CT	IV	10587553	10589147	WBGene00008072	C43F9.4	C43F9.4	-0.437219132365875	AGIWUR21238;AGIWUR31087;AGIWUR37630
CB4856	CT	IV	10589294	10590749	WBGene00008073	C43F9.5	C43F9.5	0.121913352089525	

AGIWUR26479
CB4856 CT IV 10590925 10593042 WBGene00008074 nkb-2 C43F9.6 0.0621897895846946
AGIWUR40207
CB4856 CT IV 10593068 10593575 WBGene00008075 C43F9.7 C43F9.7 -0.213648681192034
AGIWUR30846
CB4856 CT IV 10596805 10600030 WBGene00045024 C43F9.11 C43F9.11
-0.215559721665701
AGIWUR2244;AGIWUR3422;AGIWUR3911;AGIWUR5466;AGIWUR11308;AGIWUR17380;AGIWUR17551;AGIWUR20450;AGIWUR20607;AGIWUR29357;AGIWUR34540;AGIWUR36202;AGIWUR36488;AGIWUR40584
CB4856 CT IV 10600314 10601852 WBGene00011179 R09H10.1 R09H10.1
-0.125954846748993 AGIWUR43641
CB4856 CT IV 10602944 10606894 WBGene00011180 R09H10.2 R09H10.2
-0.528564409319588 AGIWUR447
CB4856 CT IV 10606902 10608861 WBGene00011181 R09H10.3 R09H10.3
-0.559766654862736 AGIWUR27996
CB4856 CT IV 10608575 10609744 WBGene00011183 R09H10.6 R09H10.6
-0.5365564890393 AGIWUR20822
CB4856 CT IV 10615427 10620958 WBGene00004228 ptr-14 R09H10.4
0.0543480382254415 AGIWUR26151
CB4856 CT IV 10621111 10621832 WBGene00011184 R09H10.7 R09H10.7
-0.702366089915818 AGIWUR33527
CB4856 CT IV 10621684 10628861 WBGene00011182 R09H10.5 R09H10.5
-0.27385857854617 AGIWUR7981;AGIWUR11151;AGIWUR18797
CB4856 CT IV 10629431 10631245 WBGene00044977 F56C4.4 F56C4.4 0.448685079928656
AGIWUR4833;AGIWUR11064;AGIWUR20553;AGIWUR32137
CB4856 CT IV 10637060 10639213 WBGene00010144 F56C4.1 F56C4.1 0.0435000161701264
AGIWUR2736
CB4856 CT IV 10639277 10642690 WBGene00010145 F56C4.2 F56C4.2 0.113289322046863
AGIWUR21153
CB4856 CT IV 10642912 10654147 WBGene00010146 glb-19 F56C4.3 -0.263459699637252
AGIWUR12731
CB4856 CT IV 10656874 10660774 WBGene00010646 K08C7.1 K08C7.1 -0.25707769154927
AGIWUR7095
CB4856 CT IV 10661857 10664404 WBGene00001476 fmo-1 K08C7.2 0.22072998085708
AGIWUR44143
CB4856 CT IV 10664412 10665685 WBGene00010649 K08C7.7 K08C7.7 -0.234274248631245
AGIWUR3375
CB4856 CT IV 10666827 10679289 WBGene00001328 epi-1 K08C7.3 -0.110871251103887
AGIWUR26700

CB4856	CT	IV	10679383	10680475	WBGene00010647	K08C7.4	K08C7.4	0.158455960279908	AGIWUR4296;AGIWUR4337;AGIWUR23573;AGIWUR30664;AGIWUR41434;AGIWUR43000
CB4856	CT	IV	10682524	10684385	WBGene00001477	fmo-2	K08C7.5	4.50470774961473	AGIWUR10619;AGIWUR14319
CB4856	CT	IV	10684432	10685894	WBGene00010648	K08C7.6	K08C7.6	0.272120951099035	AGIWUR34138
CB4856	CT	IV	10687592	10688135	WBGene00011289	R102.2	R102.2	0.0235599199272869	AGIWUR40102
CB4856	CT	IV	10689640	10690309	WBGene00194984	R102.15	R102.15	-0.0503587497941229	AGIWUR2156;AGIWUR35917;AGIWUR40196
CB4856	CT	IV	10690444	10691789	WBGene00011290	R102.3	R102.3	-0.0122776402702822	AGIWUR24495;AGIWUR25359;AGIWUR30903
CB4856	CT	IV	10691991	10695303	WBGene00011291	R102.4	R102.4	-0.421026639454215	AGIWUR37470
CB4856	CT	IV	10695309	10697071	WBGene00011292	R102.5	R102.5	0.11598337888327	AGIWUR29945
CB4856	CT	IV	10705463	10707313	WBGene00011297	R102.10	R102.10	-0.494148220690211	AGIWUR149
CB4856	CT	IV	10710057	10711794	WBGene00011296	glb-21	R102.9	0.0344900551780922	AGIWUR23699
CB4856	CT	IV	10716648	10721755	WBGene00003568	ncx-3	ZC168.1	0.257353569291922	AGIWUR15067
CB4856	CT	IV	10728572	10729003	WBGene00013856	ZC168.2	ZC168.2	-0.0781921147092426	AGIWUR30191
CB4856	CT	IV	10732832	10734632	WBGene00013857	orc-5	ZC168.3	-0.0706823366105684	AGIWUR27343
CB4856	CT	IV	10735259	10737177	WBGene00000865	cyb-1	ZC168.4	0.0719905398386835	AGIWUR26407
CB4856	CT	IV	10737474	10738189	WBGene00001730	grl-21	ZC168.5	-0.0120303350085145	AGIWUR10750;AGIWUR29076;AGIWUR30387;AGIWUR33665;AGIWUR33768
CB4856	CT	IV	10738204	10738658	WBGene00013858	ssp-34	ZC168.6	0.392073573564938	AGIWUR33627
CB4856	CT	IV	10744779	10748275	WBGene00012009	T25B9.3	T25B9.3	0.608577950930121	AGIWUR11156
CB4856	CT	IV	10748385	10750153	WBGene00012007	T25B9.1	T25B9.1	-0.578893207345313	AGIWUR23959
CB4856	CT	IV	10755760	10757716	WBGene00012010	T25B9.4	T25B9.4	0.198904040789835	AGIWUR34909
CB4856	CT	IV	10758126	10760795	WBGene00012012	T25B9.6	T25B9.6	0.301898908651586	

AGIWUR38356
CB4856 CT IV 10761597 10764496 WBGene00012013 ugt-54 T25B9.7 0.908296367697988
AGIWUR36767
CB4856 CT IV 10764520 10765297 WBGene00044990 T25B9.11 T25B9.11
0.0783096091805202 AGIWUR1346;AGIWUR13843;AGIWUR27155;AGIWUR34391;AGIWUR44154
CB4856 CT IV 10765441 10766067 WBGene00044991 T25B9.12 T25B9.12
-0.149939034240558 AGIWUR9993;AGIWUR39829;AGIWUR41583;AGIWUR42368;AGIWUR44664
CB4856 CT IV 10766481 10768429 WBGene00012014 T25B9.8 T25B9.8
-0.00752889898771691 AGIWUR21253
CB4856 CT IV 10769653 10777755 WBGene00012015 T25B9.9 T25B9.9 -0.205047316040849
AGIWUR19060
CB4856 CT IV 10778985 10780711 WBGene00007384 swt-3 C06G8.1 -0.0726798384114886
AGIWUR12296;AGIWUR16026;AGIWUR27123
CB4856 CT IV 10785024 10786831 WBGene00005085 srd-7 C06G8.4 -0.039678895994685
AGIWUR8806;AGIWUR11850;AGIWUR19272;AGIWUR35602
CB4856 CT IV 10790598 10794597 WBGene00003876 pept-2 C06G8.2 -0.129453504729705
AGIWUR6846;AGIWUR24610;AGIWUR25825
CB4856 CT IV 10799614 10802250 WBGene00014669 zipt-2.1 C06G8.3
0.0848531921111144 AGIWUR20694;AGIWUR24626;AGIWUR26420
CB4856 CT IV 10812409 10825953 WBGene00011259 miz-1 R13.4 0.09333080363119
AGIWUR2408;AGIWUR6546;AGIWUR11507;AGIWUR15807;AGIWUR19435;AGIWUR19882;AGIWUR41489
CB4856 CT IV 10826028 10828021 WBGene00011257 npax-3 R13.2 0.0306270002794031
AGIWUR30327
CB4856 CT IV 10829549 10832248 WBGene00011258 best-15 R13.3 -0.212888190475795
AGIWUR38177
CB4856 CT IV 10834363 10835130 WBGene00014825 R13.5 R13.5 0.305580661165637
AGIWUR1203;AGIWUR28576;AGIWUR40220
CB4856 CT IV 10844594 10845620 WBGene00011721 T11G6.7 T11G6.7 0.112502621272004
AGIWUR499
CB4856 CT IV 10849749 10851933 WBGene00011719 T11G6.4 T11G6.4 -0.321194644583626
AGIWUR11558
CB4856 CT IV 10852669 10855366 WBGene00011718 T11G6.3 T11G6.3 0.0691265221920254
AGIWUR28703
CB4856 CT IV 10855485 10858559 WBGene00011717 T11G6.2 T11G6.2 -0.0347800276460113
AGIWUR12551
CB4856 CT IV 10858957 10861295 WBGene00002001 hars-1 T11G6.1 -0.171133804327731
AGIWUR14510;AGIWUR14542;AGIWUR22495
CB4856 CT IV 10861402 10864232 WBGene00011722 T11G6.8 T11G6.8 0.0712769490593601
AGIWUR34117

CB4856	CT	IV	10864331	10873632	WBGene00008750	F13E9.1	F13E9.1	-0.0175533539196486	AGIWUR12104;AGIWUR13811;AGIWUR16606
CB4856	CT	IV	10873673	10874627	WBGene00008761	F13E9.12	F13E9.12	-0.130725972587248	AGIWUR31437
CB4856	CT	IV	10877144	10877296	WBGene00008751	fipr-14	F13E9.2	-0.231030400544235	AGIWUR28316
CB4856	CT	IV	10877836	10879727	WBGene00008755	sdz-13	F13E9.6	0.28130340892847	AGIWUR10549
CB4856	CT	IV	10881549	10882523	WBGene00044797	F13E9.13	F13E9.13	0.36069966610404	AGIWUR13634
CB4856	CT	IV	10882972	10884363	WBGene00008754	F13E9.5	F13E9.5	-0.0783083987913997	AGIWUR4319
CB4856	CT	IV	10885238	10886015	WBGene00008759	F13E9.10	F13E9.10	-0.559101809260694	AGIWUR20583
CB4856	CT	IV	10889471	10889878	WBGene00045208	F13E9.15	F13E9.15	0.771762656556945	AGIWUR34664
CB4856	CT	IV	10891333	10892093	WBGene00045207	F13E9.14	F13E9.14	0.164765448380423	AGIWUR7566;AGIWUR9516;AGIWUR42487
CB4856	CT	IV	10893648	10894355	WBGene00008757	F13E9.8	F13E9.8	0.411034629340611	AGIWUR24368
CB4856	CT	IV	10895225	10896612	WBGene00008753	F13E9.4	F13E9.4	0.446049815753266	AGIWUR12837
CB4856	CT	IV	10897655	10899180	WBGene00008758	F13E9.9	F13E9.9	-0.107693874451718	AGIWUR22978
CB4856	CT	IV	10900370	10901386	WBGene00014006	ZK596.1	ZK596.1	0.689691696959802	AGIWUR11142
CB4856	CT	IV	10901913	10904382	WBGene00014008	ZK596.3	ZK596.3	0.324738204877901	AGIWUR13858
CB4856	CT	IV	10908131	10909306	WBGene00014007	ZK596.2	ZK596.2	0.0787460558182413	AGIWUR43993
CB4856	CT	IV	10909915	10913078	WBGene00014001	pyk-2	ZK593.1	0.0571543073923557	AGIWUR19204
CB4856	CT	IV	10913361	10914582	WBGene00014002	ZK593.2	ZK593.2	0.589542262759995	AGIWUR208
CB4856	CT	IV	10916597	10917718	WBGene00014003	ZK593.3	ZK593.3	-0.913419979659107	AGIWUR15333
CB4856	CT	IV	10920075	10926807	WBGene00004319	rbr-2	ZK593.4	0.263620731422317	AGIWUR10324;AGIWUR33503
CB4856	CT	IV	10926730	10933203	WBGene00001017	dnc-1	ZK593.5	-0.331583643329544	

AGIWUR4036;AGIWUR15883;AGIWUR30150
CB4856 CT IV 10933502 10934200 WBGene00003081 lsm-7 ZK593.7 -0.249971847362307
AGIWUR7816
CB4856 CT IV 10934186 10935914 WBGene00002981 lgg-2 ZK593.6 0.466992716674209
AGIWUR5996;AGIWUR9128;AGIWUR16430;AGIWUR18825;AGIWUR28298;AGIWUR28809;AGIWUR32345;AGIWUR36092;AGIWUR37432
CB4856 CT IV 10937454 10939935 WBGene00014004 fic-1 ZK593.8 0.336097254246466
AGIWUR10636
CB4856 CT IV 10940959 10942869 WBGene00014005 ZK593.9 ZK593.9 0.309037324957949
AGIWUR27344
CB4856 CT IV 10949767 10951133 WBGene00013474 Y69E1A.2 Y69E1A.2
0.319807345551702 AGIWUR34021
CB4856 CT IV 10956863 10957698 WBGene00013477 Y69E1A.5 Y69E1A.5
-0.0528148508516217 AGIWUR15234
CB4856 CT IV 10958372 10961208 WBGene00005531 sri-19 Y69E1A.6
0.138238999260959 AGIWUR24828;AGIWUR40891
CB4856 CT IV 10961355 10966459 WBGene00000171 aqp-3 Y69E1A.7
-0.136668399620335 AGIWUR16033
CB4856 CT IV 10966590 10968166 WBGene00013478 Y69E1A.8 Y69E1A.8
-0.296124864931821 AGIWUR36844
CB4856 CT IV 10982068 10985030 WBGene00012794 Y43E12A.2 Y43E12A.2
0.330599161675011 AGIWUR19508;AGIWUR21438
CB4856 CT IV 10990999 10994137 WBGene00008776 F13H10.5 F13H10.5
0.706363563515417 AGIWUR23815;AGIWUR29827;AGIWUR37699
CB4856 CT IV 11002119 11003801 WBGene00045056 F13H10.9 F13H10.9
-0.103995615138381 AGIWUR837;AGIWUR25734;AGIWUR43446
CB4856 CT IV 11004573 11005070 WBGene00008773 F13H10.1 F13H10.1
0.281648700175761 AGIWUR8767
CB4856 CT IV 11009296 11010103 WBGene00008777 F13H10.6 F13H10.6
0.246566463449735 AGIWUR18010
CB4856 CT IV 11009981 11012020 WBGene00003586 ndx-9 F13H10.2
0.0175670445792449 AGIWUR43255
CB4856 CT IV 11012119 11015883 WBGene00008774 F13H10.3 F13H10.3
-0.308375517795518 AGIWUR736
CB4856 CT IV 11016229 11021902 WBGene00008775 mogs-1 F13H10.4
0.0929879920717145 AGIWUR8586;AGIWUR8727;AGIWUR15668;AGIWUR24442;AGIWUR44599
CB4856 CT IV 11022603 11025870 WBGene00000109 alh-3 F36H1.6 0.167932915923824
AGIWUR5136;AGIWUR12312;AGIWUR17773;AGIWUR30861;AGIWUR35885;AGIWUR37536
CB4856 CT IV 11028477 11029105 WBGene00009494 hrg-5 F36H1.9 0.24087287806271
AGIWUR23826

CB4856	CT	IV	11030160	11031082	WBGene00009493	hrg-4	F36H1.5	-0.651619582031183	AGIWUR2400
CB4856	CT	IV	11031171	11033581	WBGene00009495	hrg-6	F36H1.10	-0.780209386668265	AGIWUR18494
CB4856	CT	IV	11036548	11038148	WBGene00001426	fkb-1	F36H1.1	-0.233601639771237	AGIWUR3040;AGIWUR12315;AGIWUR21537;AGIWUR37162
CB4856	CT	IV	11038662	11047422	WBGene00006490	kdin-1	F36H1.2	-0.0654598854643513	AGIWUR7040;AGIWUR17878;AGIWUR22372;AGIWUR28170;AGIWUR36853;AGIWUR43480
CB4856	CT	IV	11049171	11049632	WBGene00009496	F36H1.11	F36H1.11	0.501383403871836	AGIWUR32032
CB4856	CT	IV	11050128	11052430	WBGene00009492	F36H1.3	F36H1.3	-0.456056281076123	AGIWUR19348
CB4856	CT	IV	11052816	11053634	WBGene00009497	F36H1.12	F36H1.12	0.213715695429972	AGIWUR34392
CB4856	CT	IV	11054308	11063613	WBGene00002992	lin-3	F36H1.4	-0.0562250272851967	AGIWUR54;AGIWUR748;AGIWUR2711;AGIWUR5458;AGIWUR5804;AGIWUR6726;AGIWUR7349;AGIWUR8580;AGIWUR16415;AGIWUR26047;AGIWUR26767;AGIWUR40552;AGIWUR42101
CB4856	CT	IV	11063512	11064152	WBGene00012382	ttr-16	Y5F2A.1	0.00605189838661957	AGIWUR35777
CB4856	CT	IV	11066018	11067072	WBGene00012383	ttr-17	Y5F2A.2	0.28410175003517	AGIWUR20365;AGIWUR35910;AGIWUR38875
CB4856	CT	IV	11069387	11070950	WBGene00012384	Y5F2A.3	Y5F2A.3	-0.0342846063821867	AGIWUR8611;AGIWUR10963;AGIWUR19805;AGIWUR20173;AGIWUR21012;AGIWUR29326;AGIWUR35458;AGIWUR43963
CB4856	CT	IV	11076085	11078582	WBGene00012385	Y5F2A.4	Y5F2A.4	0.0057331889219725	AGIWUR43902
CB4856	CT	IV	11078677	11081055	WBGene00010882	atg-7	M7.5	0.05804406554227	AGIWUR36657;AGIWUR43525
CB4856	CT	IV	11080989	11082253	WBGene00002344	let-70	M7.1	0.104685669571815	AGIWUR11476;AGIWUR18418;AGIWUR19817;AGIWUR24836;AGIWUR40767;AGIWUR43507
CB4856	CT	IV	11083735	11086050	WBGene00002214	klc-1	M7.2	-0.308711621969004	AGIWUR2976;AGIWUR21767;AGIWUR40967;AGIWUR41854
CB4856	CT	IV	11086074	11092552	WBGene00000246	bcc-1	M7.3	-0.0371581970958523	AGIWUR38663
CB4856	CT	IV	11093843	11095085	WBGene00010884	M7.8	M7.8	0.724681938846732	AGIWUR42600
CB4856	CT	IV	11096724	11097982	WBGene00010887	M7.12	M7.12	-0.250552378377927	AGIWUR32734
CB4856	CT	IV	11098448	11100371	WBGene00006071	str-3	M7.13	0.25130125853916	AGIWUR28180

CB4856	CT	IV	11100631	11101478	WBGene00010885	M7.9	M7.9	-0.364045419559977	AGIWUR41741
CB4856	CT	IV	11101962	11102695	WBGene00010886	M7.10	M7.10	0.361929145111777	AGIWUR13450
CB4856	CT	IV	11103255	11104262	WBGene00011483	T05E11.7	T05E11.7	-0.251625446112613	AGIWUR19919
CB4856	CT	IV	11104743	11105820	WBGene00011484	T05E11.8	T05E11.8	0.0604812708902962	AGIWUR34023
CB4856	CT	IV	11108494	11110017	WBGene00011482	hpo-4	T05E11.6	-0.45231112679795	AGIWUR2772
CB4856	CT	IV	11110102	11111045	WBGene00004474	rps-5	T05E11.1	-0.00759276709664292	AGIWUR15009;AGIWUR19637
CB4856	CT	IV	11112999	11116754	WBGene00011479	T05E11.2	T05E11.2	0.330523672302212	AGIWUR5936
CB4856	CT	IV	11116732	11118887	WBGene00011481	imp-2	T05E11.5	0.078009838453454	AGIWUR34176
CB4856	CT	IV	11119298	11123155	WBGene00011480	enpl-1	T05E11.3	0.329580650698777	AGIWUR8786
CB4856	CT	IV	11123133	11124862	WBGene00004985	spo-11	T05E11.4	-0.233523794090724	AGIWUR29114
CB4856	CT	IV	11126024	11131162	WBGene00004204	swsn-4	F01G4.1	0.119727041479679	AGIWUR11401;AGIWUR19740;AGIWUR26530;AGIWUR29970;AGIWUR39043;AGIWUR39371
CB4856	CT	IV	11131279	11133785	WBGene00000181	ard-1	F01G4.2	-0.0728957804461317	AGIWUR9929;AGIWUR40487;AGIWUR41787
CB4856	CT	IV	11135566	11142360	WBGene00008502	skih-2	F01G4.3	-0.0941350647774897	AGIWUR33640
CB4856	CT	IV	11143345	11145923	WBGene00008503	F01G4.4	F01G4.4	-0.0528522855381617	AGIWUR41438
CB4856	CT	IV	11146106	11147247	WBGene00008504	F01G4.5	F01G4.5	0.195142694822682	AGIWUR42508
CB4856	CT	IV	11146940	11149504	WBGene00008505	F01G4.6	F01G4.6	0.146757294975663	AGIWUR886;AGIWUR1602;AGIWUR3823;AGIWUR7003;AGIWUR7501;AGIWUR15287;AGIWUR15503;AGIWUR17652;AGIWUR23154;AGIWUR25021;AGIWUR29635;AGIWUR34643;AGIWUR36572
CB4856	CT	IV	11149189	11154334	WBGene00007444	C08F8.2	C08F8.2	-0.0471090926708797	AGIWUR32022
CB4856	CT	IV	11155029	11156634	WBGene00007445	C08F8.3	C08F8.3	-0.243614452515882	AGIWUR20028
CB4856	CT	IV	11157102	11159492	WBGene00007446	mboa-4	C08F8.4	-0.0552092037815326	AGIWUR35424

CB4856 CT IV 11159570 11160807 WBGene00219217 C08F8.15 C08F8.15
0.349066742555019 AGIWUR36439
CB4856 CT IV 11162768 11164622 WBGene00007448 C08F8.6 C08F8.6 0.777162912821842
AGIWUR10293
CB4856 CT IV 11169834 11174783 WBGene00003657 nhr-67 C08F8.8 -0.0358056194191949
AGIWUR32104
CB4856 CT IV 11177286 11178200 WBGene00004309 rap-3 C08F8.7 -0.241438231606892
AGIWUR40303
CB4856 CT IV 11179645 11183001 WBGene00011122 cpt-2 R07H5.2 0.0126386642929894
AGIWUR7927
CB4856 CT IV 11183132 11184310 WBGene00004199 prx-14 R07H5.1 0.061319828038588
AGIWUR7532
CB4856 CT IV 11184243 11186000 WBGene00011123 nuaf-3 R07H5.3 0.185433984493971
AGIWUR41396
CB4856 CT IV 11187012 11188264 WBGene00011124 sdz-27 R07H5.4 -0.265972979715174
AGIWUR7288
CB4856 CT IV 11188891 11189613 WBGene00011127 fbxb-79 R07H5.7 0.0131186249121651
AGIWUR16050
CB4856 CT IV 11207531 11209359 WBGene00011129 R07H5.9 R07H5.9 0.295689121927772
AGIWUR30176
CB4856 CT IV 11209309 11210831 WBGene00011128 R07H5.8 R07H5.8 -0.197522491584249
AGIWUR8968;AGIWUR15795;AGIWUR30053
CB4856 CT IV 11213651 11218137 WBGene00011130 zip-6 R07H5.10
0.13670795969666 AGIWUR28427
CB4856 CT IV 11220727 11221755 WBGene00045197 C29F4.4 C29F4.4 0.13801056638351
AGIWUR19584;AGIWUR24136
CB4856 CT IV 11222048 11223936 WBGene00007809 C29F4.3 C29F4.3 -0.0665912452333215
AGIWUR2326;AGIWUR18475;AGIWUR33132
CB4856 CT IV 11226502 11230307 WBGene00007808 best-7 C29F4.2 0.536177610245961
AGIWUR9612
CB4856 CT IV 11231670 11233124 WBGene00007338 C05C12.6 C05C12.6
-0.3988292317497 AGIWUR18612;AGIWUR23713
CB4856 CT IV 11246451 11248081 WBGene00007336 C05C12.4 C05C12.4
0.0476621881335886 AGIWUR5435;AGIWUR7534;AGIWUR8031;AGIWUR9057;AGIWUR27100;AGIWUR28815
CB4856 CT IV 11248884 11259535 WBGene00001795 gtl-1 C05C12.3
0.153469140335575 AGIWUR22273
CB4856 CT IV 11260412 11261185 WBGene00007337 C05C12.5 C05C12.5
0.260732402118265 AGIWUR44003
CB4856 CT IV 11261844 11263075 WBGene00010046 F54D1.1 F54D1.1 0.416273173056089

AGIWUR12500
CB4856 CT IV 11266290 11271352 WBGene00003606 nhr-7 F54D1.4 0.0478506784708781
AGIWUR30721
CB4856 CT IV 11273483 11281924 WBGene00001796 gtl-2 F54D1.5 0.0269310245554166
AGIWUR24761
CB4856 CT IV 11287811 11294913 WBGene00010047 F54D1.6 F54D1.6 0.259397520934009
AGIWUR32639
CB4856 CT IV 11295074 11296887 WBGene00007114 mttu-1 B0035.16
0.108599747286211 AGIWUR8709
CB4856 CT IV 11296952 11298388 WBGene00007113 B0035.15 B0035.15
-0.242321532609278 AGIWUR19989
CB4856 CT IV 11299008 11304789 WBGene00001019 dnj-1 B0035.14
-0.0748404002128957 AGIWUR32054;AGIWUR41709
CB4856 CT IV 11304721 11306762 WBGene00007112 B0035.13 B0035.13
0.0370789723207663 AGIWUR33214;AGIWUR41564;AGIWUR42935
CB4856 CT IV 11310972 11312515 WBGene00007105 B0035.1 B0035.1 -0.0963716931623164
AGIWUR6910;AGIWUR24062;AGIWUR36481;AGIWUR37302
CB4856 CT IV 11312437 11313980 WBGene00001020 dnj-2 B0035.2 -0.178265919300571
AGIWUR39962
CB4856 CT IV 11314078 11315116 WBGene00007106 B0035.3 B0035.3 -0.223582368732238
AGIWUR7573
CB4856 CT IV 11315142 11315877 WBGene00007107 pfd-4 B0035.4 -0.175901752449519
AGIWUR34374;AGIWUR43014;AGIWUR44476
CB4856 CT IV 11317419 11319951 WBGene00007108 gspd-1 B0035.5 0.0108750588528874
AGIWUR239;AGIWUR15655;AGIWUR34155
CB4856 CT IV 11320320 11320697 WBGene00044290 B0035.18 B0035.18
0.17302492759835 AGIWUR42866
CB4856 CT IV 11320800 11323634 WBGene00007109 B0035.6 B0035.6 -0.0497247511250425
AGIWUR28931
CB4856 CT IV 11323885 11324470 WBGene00001921 his-47 B0035.7 -0.585385796601091
AGIWUR2738;AGIWUR21949;AGIWUR27672;AGIWUR30476;AGIWUR39690
CB4856 CT IV 11327356 11327569 WBGene00235095 B0035.21 B0035.21
-0.164268377712681 AGIWUR7626
CB4856 CT IV 11327576 11329369 WBGene00007110 leo-1 B0035.11
-0.240581444391117 AGIWUR878;AGIWUR4853;AGIWUR30506;AGIWUR39319
CB4856 CT IV 11329466 11332969 WBGene00007111 sart-3 B0035.12
-0.0905539890557172 AGIWUR40276
CB4856 CT IV 11333079 11337184 WBGene00010061 F54E12.2 F54E12.2
-0.142289276725751 AGIWUR14228;AGIWUR28750

CB4856	CT	IV	11341325	11359863	WBGene00002223	klp-12	T01G1.1	-0.0398871260606182
AGIWUR4114;AGIWUR19671;AGIWUR23903
CB4856	CT	IV	11359864	11361509	WBGene00044025	T01G1.4	T01G1.4	-0.129595429715025
AGIWUR18422
CB4856	CT	IV	11365605	11371836	WBGene00011338	sec-31	T01G1.3	0.0164654120844503
AGIWUR30781
CB4856	CT	IV	11372991	11377627	WBGene00010351	cbd-1	H02I12.1
-0.154163100308921	AGIWUR1977
CB4856	CT	IV	11378174	11379348	WBGene00010352	fbxb-76	H02I12.2
0.275210453797033	AGIWUR41180
CB4856	CT	IV	11394543	11396728	WBGene00006459	tag-89	H02I12.3
0.183817691862686	AGIWUR8649;AGIWUR21996;AGIWUR42002
CB4856	CT	IV	11396788	11398317	WBGene00001108	dsl-6	H02I12.4
1.19395146885749	AGIWUR3759
CB4856	CT	IV	11401884	11403338	WBGene00010353	memi-3	H02I12.5
-0.00885821356874139	AGIWUR44329
CB4856	CT	IV	11403961	11406066	WBGene00010354	cyp-31A2	H02I12.8
-0.590650555652925	AGIWUR1332
CB4856	CT	IV	11410228	11413001	WBGene00009035	gfat-2	F22B3.4	-0.232085423206367
AGIWUR910;AGIWUR3963;AGIWUR6608;AGIWUR8102;AGIWUR15198;AGIWUR18863;AGIWUR32594;AGIWUR40623;AGIWUR43992
CB4856	CT	IV	11413208	11415020	WBGene00045122	F22B3.10	F22B3.10
0.50398726324385	AGIWUR37720
CB4856	CT	IV	11415598	11416824	WBGene00009036	F22B3.5	F22B3.5	0.556682010946142
AGIWUR8998;AGIWUR21522;AGIWUR32760;AGIWUR38677
CB4856	CT	IV	11417581	11421796	WBGene00009038	F22B3.7	F22B3.7	-0.379911086437989
AGIWUR44684
CB4856	CT	IV	11422711	11425251	WBGene00009039	F22B3.8	F22B3.8	-0.0352652049753495
AGIWUR9347;AGIWUR15133;AGIWUR19339;AGIWUR27538
CB4856	CT	IV	11425742	11447060	WBGene00002192	kin-4	C10C6.1	-0.0266663379290715
AGIWUR13495;AGIWUR36397
CB4856	CT	IV	11459984	11463195	WBGene00007006	npr-3	C10C6.2	-0.326525945742027
AGIWUR4533
CB4856	CT	IV	11463609	11465189	WBGene00007512	C10C6.3	C10C6.3	-0.389412728595176
AGIWUR17673;AGIWUR28120
CB4856	CT	IV	11465433	11467239	WBGene00007515	C10C6.7	C10C6.7	-0.43515205864319
AGIWUR1267;AGIWUR1584;AGIWUR20291
CB4856	CT	IV	11470185	11473419	WBGene00007513	wht-2	C10C6.5	0.0148394748597358
AGIWUR1848;AGIWUR11284;AGIWUR22623;AGIWUR29007;AGIWUR39331;AGIWUR45150
CB4856	CT	IV	11473363	11479251	WBGene00007514	catp-8	C10C6.6	-0.0717768393036113

AGIWUR10656;AGIWUR17810;AGIWUR31938
CB4856 CT IV 11479358 11480369 WBGene00003148 mbf-1 H21P03.1
-0.161611500503001 AGIWUR28512
CB4856 CT IV 11480846 11482437 WBGene00010409 H21P03.2 H21P03.2
-0.169361619619478 AGIWUR27589
CB4856 CT IV 11482668 11498167 WBGene00004892 sms-1 H21P03.3
-0.0355596471949386 AGIWUR6928;AGIWUR20047;AGIWUR27142;AGIWUR32836;AGIWUR41669;AGIWUR44350
CB4856 CT IV 11504716 11509723 WBGene00006678 twk-25 M04B2.5 -0.0255141478650138
AGIWUR24160
CB4856 CT IV 11509726 11510764 WBGene00010848 M04B2.6 M04B2.6 0.39343383280071
AGIWUR43084
CB4856 CT IV 11517186 11517620 WBGene00045319 M04B2.8 M04B2.8 0.028522322737647
AGIWUR8716;AGIWUR25069;AGIWUR38609;AGIWUR40846
CB4856 CT IV 11518422 11521031 WBGene00010847 M04B2.4 M04B2.4 -0.273427831096537
AGIWUR40957
CB4856 CT IV 11521290 11522214 WBGene00001585 gfl-1 M04B2.3 -0.0142374310759692
AGIWUR38859
CB4856 CT IV 11524642 11527954 WBGene00003218 mep-1 M04B2.1 0.108101131932608
AGIWUR26739
CB4856 CT IV 11528052 11530715 WBGene00010846 M04B2.2 M04B2.2 -0.105329099082116
AGIWUR2719
CB4856 CT IV 11530650 11548586 WBGene00010849 M04B2.7 M04B2.7 -0.0178444813145902
AGIWUR40341
CB4856 CT IV 11550002 11551987 WBGene00000702 col-128 F12F6.9 -0.428080769445899
AGIWUR26388
CB4856 CT IV 11554489 11558261 WBGene00008723 F12F6.8 F12F6.8 0.136879464804415
AGIWUR31855
CB4856 CT IV 11558737 11561891 WBGene00008722 F12F6.7 F12F6.7 -0.107151574834129
AGIWUR18979
CB4856 CT IV 11561986 11566194 WBGene00004755 sec-24.1 F12F6.6
-0.368728597584896 AGIWUR11185;AGIWUR12547;AGIWUR14071;AGIWUR40393
CB4856 CT IV 11570500 11578699 WBGene00006406 srgp-1 F12F6.5 0.213936519527699
AGIWUR5747
CB4856 CT IV 11578757 11584066 WBGene00004360 rib-1 F12F6.3 0.0853195307021175
AGIWUR21192;AGIWUR34748
CB4856 CT IV 11585224 11588321 WBGene00008720 F12F6.1 F12F6.1 0.0633484865367109
AGIWUR20751
CB4856 CT IV 11588263 11592240 WBGene00009589 F40F11.4 F40F11.4
0.0848534467393236 AGIWUR22717

CB4856 CT IV 11592351 11594003 WBGene00009588 F40F11.3 F40F11.3
0.00819831348385769 AGIWUR10426
CB4856 CT IV 11594244 11601695 WBGene00009587 mig-38 F40F11.2
0.266406482354309 AGIWUR15204
CB4856 CT IV 11602641 11603283 WBGene00004480 rps-11 F40F11.1
0.0311363048140549 AGIWUR3267;AGIWUR24771;AGIWUR29833;AGIWUR41945;AGIWUR42716
CB4856 CT IV 11603613 11604927 WBGene00012497 ragc-1 Y24F12A.2
0.199742985480341 AGIWUR10140
CB4856 CT IV 11605254 11607457 WBGene00012496 Y24F12A.1 Y24F12A.1
-0.0796624087542881 AGIWUR38440
CB4856 CT IV 11619502 11620938 WBGene00001107 dsl-5 F58B3.8 0.222169789180601
AGIWUR547;AGIWUR15690
CB4856 CT IV 11621443 11623174 WBGene00003093 lys-4 F58B3.1 0.259982213795988
AGIWUR39966
CB4856 CT IV 11624255 11625582 WBGene00003094 lys-5 F58B3.2 0.287000445246344
AGIWUR19394
CB4856 CT IV 11626910 11627947 WBGene00003095 lys-6 F58B3.3 0.0139338669492339
AGIWUR36906
CB4856 CT IV 11628757 11631850 WBGene00010231 F58B3.4 F58B3.4 -0.296558552830557
AGIWUR6455
CB4856 CT IV 11631941 11635487 WBGene00003415 mars-1 F58B3.5 -0.170507262384865
AGIWUR12127;AGIWUR12383;AGIWUR19727
CB4856 CT IV 11636648 11638208 WBGene00010232 F58B3.6 F58B3.6 -0.0233689380023065
AGIWUR1821;AGIWUR13189;AGIWUR24826
CB4856 CT IV 11638316 11640097 WBGene00010233 F58B3.7 F58B3.7 -0.13239759202569
AGIWUR25433
CB4856 CT IV 11640047 11641015 WBGene00010234 ttr-50 F58B3.9 0.958287035358051
AGIWUR38959
CB4856 CT IV 11645597 11648893 WBGene00014088 ZK809.8 ZK809.8 0.312966382892402
AGIWUR6247
CB4856 CT IV 11650715 11652033 WBGene00006491 acl-3 ZK809.2 -0.140265890723597
AGIWUR44861
CB4856 CT IV 11652403 11653461 WBGene00014086 ZK809.3 ZK809.3 0.0553284163090995
AGIWUR10668;AGIWUR32126;AGIWUR34914;AGIWUR37719
CB4856 CT IV 11653422 11655470 WBGene00001320 ent-1 ZK809.4 -0.203932148527796
AGIWUR14683
CB4856 CT IV 11656730 11659425 WBGene00014087 ZK809.5 ZK809.5 -0.280927013308419
AGIWUR4363
CB4856 CT IV 11659800 11665203 WBGene00014076 ZK792.1 ZK792.1

-0.00304307266750925 AGIWUR15773
CB4856 CT IV 11665310 11667989 WBGene00194934 ZK809.9 ZK809.9 0.304679460608726
AGIWUR32942
CB4856 CT IV 11668024 11671505 WBGene00014080 atg-4.2 ZK792.8 0.0757373287702769
AGIWUR33183
CB4856 CT IV 11673816 11675635 WBGene00002130 inx-8 ZK792.2 0.133919318859999
AGIWUR42581
CB4856 CT IV 11676354 11678497 WBGene00002131 inx-9 ZK792.3 -0.124695447530886
AGIWUR16911
CB4856 CT IV 11678440 11682005 WBGene00014077 ZK792.4 ZK792.4 -0.064160207897153
AGIWUR14826
CB4856 CT IV 11684975 11688118 WBGene00014078 ZK792.5 ZK792.5 -0.295303074197101
AGIWUR21899
CB4856 CT IV 11688227 11691088 WBGene00002335 let-60 ZK792.6 0.108080160745073
AGIWUR34480
CB4856 CT IV 11694359 11696464 WBGene00014079 ZK792.7 ZK792.7 0.289054734156323
AGIWUR44766
CB4856 CT IV 11696443 11699895 WBGene00001079 dpy-20 T22B3.1 -0.682573387594021
AGIWUR28561
CB4856 CT IV 11703185 11704016 WBGene00011911 T22B3.3 T22B3.3 0.170874662332576
AGIWUR45199
CB4856 CT IV 11704246 11708264 WBGene00011910 alg-3 T22B3.2 0.38007702985362
AGIWUR42929
CB4856 CT IV 11715039 11729276 WBGene00010444 magi-1 K01A6.2 0.094459557254484
AGIWUR7822;AGIWUR13519;AGIWUR17756;AGIWUR24742;AGIWUR27030;AGIWUR34487;AGIWUR43990
CB4856 CT IV 11735325 11742305 WBGene00010448 K01A6.6 K01A6.6 -0.062237512161996
AGIWUR29459
CB4856 CT IV 11742706 11743789 WBGene00010447 K01A6.5 K01A6.5 -0.108634623840716
AGIWUR16888
CB4856 CT IV 11745640 11748640 WBGene00001577 gem-4 T12A7.1 0.574050977467868
AGIWUR14415
CB4856 CT IV 11749154 11751074 WBGene00011727 T12A7.6 T12A7.6 1.34714993080132
AGIWUR14379
CB4856 CT IV 11751872 11752945 WBGene00011728 T12A7.7 T12A7.7 -0.0930950566319364
AGIWUR3767
CB4856 CT IV 11752987 11754387 WBGene00011724 scl-18 T12A7.3 0.147398012726036
AGIWUR26905
CB4856 CT IV 11755274 11755970 WBGene00185100 T12A7.9 T12A7.9 -0.0474106935388567
AGIWUR37943

CB4856	CT	IV	11756381	11761064	WBGene00011723	T12A7.2	T12A7.2	0.374478696947726	AGIWUR3478;AGIWUR5238;AGIWUR7874;AGIWUR16576;AGIWUR22434;AGIWUR34793;AGIWUR37106
CB4856	CT	IV	11768639	11773742	WBGene00011725	lips-5	T12A7.4	0.134696658158397	AGIWUR10418;AGIWUR26358;AGIWUR39694
CB4856	CT	IV	11779216	11786969	WBGene00011726	lpr-2	T12A7.5	0.436098745927458	AGIWUR8854;AGIWUR12875
CB4856	CT	IV	11783941	11792593	WBGene00010350	H01G02.3	H01G02.3	-0.141638457283999	AGIWUR13502;AGIWUR21251;AGIWUR40362
CB4856	CT	IV	11798667	11801088	WBGene00010349	dyf-18	H01G02.2	0.614169077051296	AGIWUR32927
CB4856	CT	IV	11801697	11803144	WBGene00045108	H01G02.4	H01G02.4	-0.311604350015662	AGIWUR9688;AGIWUR24665
CB4856	CT	IV	11803273	11804152	WBGene00010348	H01G02.1	H01G02.1	-0.300836788907076	AGIWUR1707;AGIWUR8257;AGIWUR23953;AGIWUR25577
CB4856	CT	IV	11814247	11819612	WBGene00010918	M117.1	M117.1	-0.0381416874113381	AGIWUR3124
CB4856	CT	IV	11820505	11821917	WBGene00003920	par-5	M117.2	-0.0436380959495466	AGIWUR5263;AGIWUR5896;AGIWUR26028;AGIWUR34257
CB4856	CT	IV	11823516	11823938	WBGene00010919	M117.3	M117.3	-0.230993974841954	AGIWUR43702
CB4856	CT	IV	11828973	11830446	WBGene00010921	fbxb-4	M117.5	0.0569567249987442	AGIWUR5287;AGIWUR17122;AGIWUR27546
CB4856	CT	IV	11830799	11833556	WBGene00010920	M117.4	M117.4	0.000589748221987207	AGIWUR6805
CB4856	CT	IV	11834390	11836020	WBGene00044768	M117.7	M117.7	0.174762550750077	AGIWUR38728
CB4856	CT	IV	11838980	11840423	WBGene00009546	fbxb-3	F38H4.2	0.407194207524054	AGIWUR38113
CB4856	CT	IV	11842421	11846350	WBGene00009547	F38H4.3	F38H4.3	-0.138572794122172	AGIWUR6905
CB4856	CT	IV	11847009	11847871	WBGene00009550	F38H4.6	F38H4.6	0.0597087299660544	AGIWUR21232
CB4856	CT	IV	11848232	11850309	WBGene00009548	F38H4.4	F38H4.4	0.137054792690565	AGIWUR3661
CB4856	CT	IV	11850950	11855159	WBGene00006415	tag-30	F38H4.7	-0.199523695382297	AGIWUR2752
CB4856	CT	IV	11854828	11856376	WBGene00001151	ech-2	F38H4.8	-0.397740959924583	AGIWUR23837
CB4856	CT	IV	11856629	11860460	WBGene00002363	let-92	F38H4.9	0.0267759919212951	

AGIWUR5253;AGIWUR5272;AGIWUR5474;AGIWUR5480;AGIWUR11755;AGIWUR36054
CB4856 CT IV 11860555 11862498 WBGene00009551 F38H4.10 F38H4.10 0.210009752733573 AGIWUR7377
CB4856 CT IV 11864532 11875738 WBGene00007801 trpa-1 C29E6.2 -0.16927066647229 AGIWUR16343;AGIWUR19719;AGIWUR26134;AGIWUR40717
CB4856 CT IV 11878549 11883407 WBGene00007802 cutl-27 C29E6.4 0.0499739320311399 AGIWUR40702
CB4856 CT IV 11885530 11891081 WBGene00002827 let-653 C29E6.1 0.308291571742934 AGIWUR20827
CB4856 CT IV 11899683 11902910 WBGene00003633 nhr-43 C29E6.5 0.308812904172761 AGIWUR3447
CB4856 CT IV 11912005 11913245 WBGene00012163 VZK822L.2 VZK822L.2 -0.567918887522467 AGIWUR895
CB4856 CT IV 11913691 11915796 WBGene00001398 fat-6 VZK822L.1 0.727680756459513 AGIWUR11968;AGIWUR32999;AGIWUR39182
CB4856 CT IV 11922358 11923174 WBGene00014090 ZK822.2 ZK822.2 0.183681372258803 AGIWUR13802
CB4856 CT IV 11924685 11925397 WBGene00044009 ZK822.6 ZK822.6 0.143074778571178 AGIWUR16060;AGIWUR25195;AGIWUR36482
CB4856 CT IV 11926023 11928415 WBGene00014091 ZK822.4 ZK822.4 0.0190202641388346 AGIWUR4838
CB4856 CT IV 11930872 11935718 WBGene00003736 nhx-9 ZK822.3 0.262697300689059 AGIWUR30705;AGIWUR32431;AGIWUR41714
CB4856 CT IV 11937384 11940604 WBGene00014092 ZK822.5 ZK822.5 -0.168983548418179 AGIWUR14263;AGIWUR25076;AGIWUR28171
CB4856 CT IV 11940603 11942385 WBGene00014093 ZK829.1 ZK829.1 -0.275824409660116 AGIWUR980;AGIWUR26330;AGIWUR43730
CB4856 CT IV 11943314 11945721 WBGene00014094 ZK829.3 ZK829.3 -0.112198319918261 AGIWUR18368
CB4856 CT IV 11946106 11949817 WBGene00001839 hdl-1 ZK829.2 -0.180025123386744 AGIWUR17660
CB4856 CT IV 11950570 11952208 WBGene00006555 tbx-36 ZK829.5 -0.251257341874908 AGIWUR30920
CB4856 CT IV 11953249 11955541 WBGene00014095 gdh-1 ZK829.4 -0.0277403506160532 AGIWUR3935;AGIWUR10461;AGIWUR14208;AGIWUR20797;AGIWUR40982
CB4856 CT IV 11957547 11959804 WBGene00006566 tgt-1 ZK829.6 -0.0251888681711332 AGIWUR1767
CB4856 CT IV 11959823 11961535 WBGene00014096 ZK829.7 ZK829.7 -0.0196664426016238 AGIWUR14518

CB4856	CT	IV	11963910	11965427	WBGene00005591	srj-1	ZK829.8	-0.220679108230225	AGIWUR2300
CB4856	CT	IV	11965665	11968703	WBGene00004907	snf-8	ZK829.10	-0.0865824209863586	AGIWUR38948
CB4856	CT	IV	11970534	11972789	WBGene00014097	ZK829.9	ZK829.9	-0.233866091036699	AGIWUR8490
CB4856	CT	IV	11972697	12011007	WBGene00006759	unc-22	ZK617.1	-0.0810860559313031	AGIWUR3562;AGIWUR13699;AGIWUR28418;AGIWUR39579
CB4856	CT	IV	12013420	12014045	WBGene00004971	spe-17	ZK617.3	-0.135469492945429	AGIWUR42758
CB4856	CT	IV	12014189	12015686	WBGene00014009	lips-6	ZK617.2	0.294100432363511	AGIWUR5897
CB4856	CT	IV	12026882	12029174	WBGene00011742	tgn-38	T12G3.7	-0.0216849205941179	AGIWUR3714;AGIWUR14906;AGIWUR17093;AGIWUR20016;AGIWUR24430;AGIWUR44919
CB4856	CT	IV	12030047	12031271	WBGene00011741	T12G3.6	T12G3.6	-0.30329276315895	AGIWUR28845
CB4856	CT	IV	12031314	12032154	WBGene00011740	mrpl-51	T12G3.5	-0.0815005096858066	AGIWUR4307;AGIWUR6927;AGIWUR43099
CB4856	CT	IV	12034252	12037051	WBGene00011737	sqst-1	T12G3.1	0.914627661744831	AGIWUR1200
CB4856	CT	IV	12038985	12046967	WBGene00011738	T12G3.2	T12G3.2	0.214325539819239	AGIWUR16765;AGIWUR33601;AGIWUR44249
CB4856	CT	IV	12048256	12049943	WBGene00011739	T12G3.4	T12G3.4	0.243461314890498	AGIWUR36660
CB4856	CT	IV	12049862	12051226	WBGene00000270	bre-5	T12G3.8	-0.127695894861096	AGIWUR24329
CB4856	CT	IV	12051484	12055785	WBGene00005015	spt-5	K08E4.1	0.0326425595577113	AGIWUR6774
CB4856	CT	IV	12055670	12056656	WBGene00010666	K08E4.2	K08E4.2	-0.272052721517689	AGIWUR24105
CB4856	CT	IV	12057626	12059951	WBGene00010667	K08E4.3	K08E4.3	-0.201550722870572	AGIWUR26770
CB4856	CT	IV	12060397	12061329	WBGene00045283	K08E4.8	K08E4.8	-0.109288102627281	AGIWUR35311;AGIWUR41068
CB4856	CT	IV	12066572	12066884	WBGene00010668	K08E4.4	K08E4.4	-0.85547593017049	AGIWUR16321
CB4856	CT	IV	12068890	12074914	WBGene00010669	bath-37	K08E4.5	0.0803248502618281	AGIWUR5644;AGIWUR10958
CB4856	CT	IV	12075744	12076950	WBGene00045036	K08E4.7	K08E4.7	0.254256723775846

AGIWUR10309
CB4856 CT IV 12077107 12078162 WBGene00010670 K08E4.6 K08E4.6 0.00516388894799924
AGIWUR20378
CB4856 CT IV 12079463 12084362 WBGene00008682 lex-1 F11A10.1
-0.0401203616920061 AGIWUR18425;AGIWUR36995
CB4856 CT IV 12084478 12085320 WBGene00008683 repo-1 F11A10.2
-0.0533629026458442 AGIWUR23785
CB4856 CT IV 12085423 12087060 WBGene00008688 F11A10.7 F11A10.7
0.0885242895539449 AGIWUR34296
CB4856 CT IV 12087017 12089095 WBGene00008687 F11A10.6 F11A10.6
-0.112405180839367 AGIWUR28237
CB4856 CT IV 12089247 12092213 WBGene00008684 mig-32 F11A10.3
0.00745584737904181 AGIWUR38996
CB4856 CT IV 12092322 12093472 WBGene00044329 cpsf-4 F11A10.8
-0.162608592821378 AGIWUR15808;AGIWUR22988
CB4856 CT IV 12094372 12096692 WBGene00008686 F11A10.5 F11A10.5
-0.109122587847619 AGIWUR17214
CB4856 CT IV 12096779 12105762 WBGene00008685 mon-2 F11A10.4
0.109043118795888 AGIWUR9219;AGIWUR38240;AGIWUR38547
CB4856 CT IV 12105858 12108388 WBGene00010889 M18.3 M18.3 -0.0948757562601727
AGIWUR41362
CB4856 CT IV 12108672 12110275 WBGene00000703 col-129 M18.1 0.101025198020132
AGIWUR8374
CB4856 CT IV 12110586 12111577 WBGene00010888 dlc-2 M18.2 -0.121745608010205
AGIWUR14688;AGIWUR15276;AGIWUR15586
CB4856 CT IV 12114810 12121183 WBGene00010890 ddb-1 M18.5 0.393012878573041
AGIWUR10292
CB4856 CT IV 12121266 12122881 WBGene00010891 M18.6 M18.6 0.0981991383921486
AGIWUR34671;AGIWUR40569;AGIWUR42513
CB4856 CT IV 12123155 12124807 WBGene00000122 aly-3 M18.7 -0.147157507133027
AGIWUR20179;AGIWUR27251
CB4856 CT IV 12124952 12126785 WBGene00010892 dhhc-6 M18.8
-0.00667757944055936 AGIWUR26917
CB4856 CT IV 12129504 12135096 WBGene00003010 lin-24 B0001.1 -0.142590533417263
AGIWUR998
CB4856 CT IV 12135074 12138193 WBGene00007089 B0001.4 B0001.4 0.0890879276150223
AGIWUR34195
CB4856 CT IV 12138847 12141057 WBGene00007093 B0001.8 B0001.8 -0.0320154756262845
AGIWUR38687

CB4856	CT	IV	12141161	12143331	WBGene00007090	B0001.5	B0001.5	-0.0105442793212323	AGIWUR38103
CB4856	CT	IV	12143431	12145731	WBGene00007088	B0001.3	B0001.3	0.0124416440239656	AGIWUR14977;AGIWUR37338;AGIWUR44425
CB4856	CT	IV	12146486	12150975	WBGene00007087	B0001.2	B0001.2	-0.448662110845419	AGIWUR25713
CB4856	CT	IV	12151170	12154245	WBGene00007092	B0001.7	B0001.7	0.0658075076359719	AGIWUR41334
CB4856	CT	IV	12156838	12164266	WBGene00007091	eri-12	B0001.6	0.389940848050745	AGIWUR36393
CB4856	CT	IV	12168244	12169216	WBGene00008967	F20B10.3	F20B10.3	-0.364795356966123	AGIWUR43417
CB4856	CT	IV	12170824	12178335	WBGene00003772	nlr-1	F20B10.1	0.447736228064352	AGIWUR2056
CB4856	CT	IV	12200978	12207956	WBGene00009435	F35G2.1	F35G2.1	0.378708795151027	AGIWUR2732;AGIWUR28383;AGIWUR39547
CB4856	CT	IV	12208192	12209010	WBGene00009436	marb-1	F35G2.2	-0.0657438888417756	AGIWUR18307;AGIWUR25328;AGIWUR26517;AGIWUR34553;AGIWUR42977
CB4856	CT	IV	12209708	12210737	WBGene00009437	F35G2.3	F35G2.3	-0.317684293797943	AGIWUR26562
CB4856	CT	IV	12211098	12215987	WBGene00004025	phy-2	F35G2.4	-0.00966133642535283	AGIWUR7106
CB4856	CT	IV	12219609	12222889	WBGene00009438	F35G2.5	F35G2.5	0.110203536299187	AGIWUR10427;AGIWUR24979;AGIWUR25805
CB4856	CT	IV	12222872	12227112	WBGene00012704	mlt-3	Y39C12A.1	-0.244677113963295	AGIWUR25518
CB4856	CT	IV	12227910	12229779	WBGene00012705	sre-18	Y39C12A.3	0.0436687518179912	AGIWUR22014
CB4856	CT	IV	12231310	12231631	WBGene00045314	Y39C12A.10	Y39C12A.10	-0.184957219939187	AGIWUR4014;AGIWUR15890;AGIWUR23137;AGIWUR25704;AGIWUR38992
CB4856	CT	IV	12232489	12233408	WBGene00012706	sre-17	Y39C12A.4	-0.0943329418394043	AGIWUR6305;AGIWUR6896
CB4856	CT	IV	12234139	12236111	WBGene00012707	sre-16	Y39C12A.5	0.100268704971816	AGIWUR7617;AGIWUR22914;AGIWUR39281
CB4856	CT	IV	12237175	12238951	WBGene00012708	sre-19	Y39C12A.6	-0.0589681721600748	AGIWUR11239
CB4856	CT	IV	12241363	12244806	WBGene00012709	sre-15	Y39C12A.7	-0.0193883169818232	AGIWUR8094
CB4856	CT	IV	12251111	12252663	WBGene00001044	dnj-26	Y39C12A.8

-0.203997492977912	AGIWUR15323
CB4856	CT	IV	12257637	12259192	WBGene00012710	Y39C12A.9	Y39C12A.9
0.276696337730824	AGIWUR9675
CB4856	CT	IV	12259797	12261449	WBGene00016579	sre-14	C42C1.1	0.185519553726563
AGIWUR9188
CB4856	CT	IV	12265526	12268306	WBGene00016580	C42C1.2	C42C1.2	-0.433419568729271
AGIWUR27865
CB4856	CT	IV	12268995	12269533	WBGene00004448	rpl-34	C42C1.14
0.10032155912684	AGIWUR8628
CB4856	CT	IV	12269594	12270492	WBGene00016581	C42C1.3	C42C1.3	-0.286334631490963
AGIWUR17440
CB4856	CT	IV	12270490	12275992	WBGene00016582	C42C1.4	C42C1.4	-0.0930691600628464
AGIWUR1169
CB4856	CT	IV	12276083	12278059	WBGene00016583	tag-335	C42C1.5	-0.23439689768184
AGIWUR1600
CB4856	CT	IV	12278729	12279546	WBGene00016591	C42C1.13	C42C1.13
-0.0281091640955751	AGIWUR38119
CB4856	CT	IV	12279659	12281917	WBGene00016589	C42C1.11	C42C1.11
-0.178359980587343	AGIWUR9093;AGIWUR25940;AGIWUR37458;AGIWUR41625
CB4856	CT	IV	12282739	12287820	WBGene00016588	hpo-12	C42C1.10
0.0534920941217347	AGIWUR12191;AGIWUR14023;AGIWUR25224;AGIWUR43531
CB4856	CT	IV	12287928	12289392	WBGene00016592	erl-1	C42C1.15
0.291113783790022	AGIWUR8866
CB4856	CT	IV	12289449	12290994	WBGene00016587	C42C1.9	C42C1.9	0.0976539231567912
AGIWUR18105
CB4856	CT	IV	12291046	12293317	WBGene00016586	C42C1.8	C42C1.8	-0.0555298849282195
AGIWUR41507
CB4856	CT	IV	12293234	12301280	WBGene00016585	oac-59	C42C1.7	-0.0152239262033853
AGIWUR32855;AGIWUR37511
CB4856	CT	IV	12304462	12307820	WBGene00019579	oac-57	K09E10.1
0.115005075067994	AGIWUR4366
CB4856	CT	IV	12308293	12311606	WBGene00019580	oac-58	K09E10.2
-0.0554985335467246	AGIWUR12521;AGIWUR19198
CB4856	CT	IV	12327190	12328718	WBGene00008949	F19B6.3	F19B6.3	0.18185110068603
AGIWUR9173
CB4856	CT	IV	12329407	12331054	WBGene00006733	ufd-1	F19B6.2	-0.334966704632323
AGIWUR14233;AGIWUR22664
CB4856	CT	IV	12330984	12336041	WBGene00008948	F19B6.1	F19B6.1	-0.117728236256159
AGIWUR7069;AGIWUR8219;AGIWUR16393;AGIWUR19254;AGIWUR20477;AGIWUR24504;AGIWUR37312

CB4856	CT	IV	12337577	12340436	WBGene00008950	wht-5	F19B6.4	0.0543048238232462	AGIWUR16742
CB4856	CT	IV	12349946	12355724	WBGene00013921	best-22	ZC518.1	0.118633057952412	AGIWUR7513;AGIWUR23782;AGIWUR28936;AGIWUR30454;AGIWUR37902;AGIWUR39697;AGIWUR40257
CB4856	CT	IV	12355604	12360792	WBGene00004756	sec-24.2	ZC518.2	-0.104215076114728	AGIWUR2099;AGIWUR8661
CB4856	CT	IV	12361542	12371852	WBGene00000376	ccr-4	ZC518.3	-0.284711028758494	AGIWUR10657;AGIWUR14172;AGIWUR15335;AGIWUR16124;AGIWUR26381;AGIWUR34290;AGIWUR39356
CB4856	CT	IV	12372253	12374908	WBGene00009211	wdr-46	F28D1.1	-0.297829625474115	AGIWUR3214;AGIWUR10643;AGIWUR30836;AGIWUR37610;AGIWUR39901
CB4856	CT	IV	12374709	12376121	WBGene00009212	F28D1.2	F28D1.2	0.215259017375307	AGIWUR1712;AGIWUR9248;AGIWUR12535;AGIWUR43721
CB4856	CT	IV	12376869	12378050	WBGene00044285	thn-8	F28D1.12	0.158276794920505	AGIWUR7166;AGIWUR25211
CB4856	CT	IV	12380104	12380857	WBGene00009213	thn-1	F28D1.3	0.360111256854225	AGIWUR40381
CB4856	CT	IV	12381592	12382339	WBGene00009214	thn-3	F28D1.4	0.181400838125114	AGIWUR32234
CB4856	CT	IV	12383170	12384019	WBGene00009215	thn-2	F28D1.5	0.504331816448261	AGIWUR9895
CB4856	CT	IV	12384507	12385161	WBGene00009216	F28D1.6	F28D1.6	-0.113413661613858	AGIWUR43733
CB4856	CT	IV	12387720	12390095	WBGene00009217	spe-45	F28D1.8	-0.367015464664773	AGIWUR28423
CB4856	CT	IV	12390264	12391396	WBGene00004492	rps-23	F28D1.7	-0.014944320266272	AGIWUR15756;AGIWUR39218
CB4856	CT	IV	12391591	12392120	WBGene00009219	dpm-3	F28D1.11	-0.173464810374008	AGIWUR3122;AGIWUR6771;AGIWUR17201;AGIWUR23209;AGIWUR32254;AGIWUR34150
CB4856	CT	IV	12392121	12398706	WBGene00009218	acs-20	F28D1.9	-0.110993872995385	AGIWUR6360;AGIWUR11480;AGIWUR27506;AGIWUR34666;AGIWUR37887;AGIWUR43532
CB4856	CT	IV	12404374	12408697	WBGene00001580	gex-3	F28D1.10	0.017062111938694	AGIWUR23995;AGIWUR30797;AGIWUR40839
CB4856	CT	IV	12408895	12410468	WBGene00008580	dsb-1	F08G5.1	0.144331876876286	AGIWUR7802
CB4856	CT	IV	12410467	12412219	WBGene00008581	acl-13	F08G5.2	0.0438760139420005	AGIWUR15747
CB4856	CT	IV	12412923	12417582	WBGene00008582	F08G5.3	F08G5.3	0.0779178398539164	AGIWUR1489;AGIWUR11150;AGIWUR22785;AGIWUR39630
CB4856	CT	IV	12425217	12432533	WBGene00008583	ugt-65	F08G5.5	-0.130487025453038	

AGIWUR12087;AGIWUR17157;AGIWUR24039;AGIWUR37416
CB4856 CT IV 12432640 12435001 WBGene00023502 clec-184 F08G5.7
-0.296697339417824 AGIWUR18437
CB4856 CT IV 12435802 12438274 WBGene00008584 irg-4 F08G5.6 -0.364904069241021
AGIWUR38591
CB4856 CT IV 12442068 12443035 WBGene00255469 C25G4.17 C25G4.17
-0.139490334693111 AGIWUR25856
CB4856 CT IV 12443236 12444046 WBGene00007730 C25G4.2 C25G4.2 0.344839658392779
AGIWUR13600
CB4856 CT IV 12444450 12445884 WBGene00007731 C25G4.3 C25G4.3 0.250579001103882
AGIWUR12772
CB4856 CT IV 12445801 12451299 WBGene00001085 dpy-26 C25G4.5 -0.0128748429573999
AGIWUR4086;AGIWUR33667;AGIWUR34816
CB4856 CT IV 12452066 12454215 WBGene00007732 spe-44 C25G4.4 -0.0919879559244325
AGIWUR9240
CB4856 CT IV 12455097 12456175 WBGene00007733 smz-1 C25G4.6
-0.00116696610670353 AGIWUR21117
CB4856 CT IV 12456913 12458561 WBGene00007734 C25G4.7 C25G4.7 -0.169055868898353
AGIWUR28094
CB4856 CT IV 12459819 12463412 WBGene00007735 C25G4.8 C25G4.8 0.0761883127621212
AGIWUR12518;AGIWUR34070
CB4856 CT IV 12468658 12474889 WBGene00007736 igdb-2 C25G4.10
0.0492431118150662 AGIWUR26780
CB4856 CT IV 12487579 12489721 WBGene00005689 sru-26 T04A11.12
0.0136324191918966 AGIWUR37757
CB4856 CT IV 12490791 12495298 WBGene00001865 him-6 T04A11.6
0.249278809421861 AGIWUR44976
CB4856 CT IV 12496065 12497844 WBGene00005683 sru-20 T04A11.7
0.053098077117391 AGIWUR43611
CB4856 CT IV 12498538 12500413 WBGene00005682 sru-19 T04A11.8
0.122393104938023 AGIWUR38884
CB4856 CT IV 12502256 12504055 WBGene00005681 sru-18 T04A11.9
-0.00548136614345562 AGIWUR22213
CB4856 CT IV 12507409 12507566 WBGene00045131 T04A11.14 T04A11.14
0.00165599815832707 AGIWUR14695
CB4856 CT IV 12517110 12518128 WBGene00009954 F53B2.1 F53B2.1 0.0875358759871894
AGIWUR10830
CB4856 CT IV 12521239 12522621 WBGene00005676 sru-13 F53B2.4 -0.0441839688632299
AGIWUR38780

CB4856 CT IV 12524478 12525509 WBGene00009955 eak-4 F53B2.3 0.173923544981795
AGIWUR40563
CB4856 CT IV 12526260 12527339 WBGene00009957 F53B2.8 F53B2.8 1.33405871545876
AGIWUR11000
CB4856 CT IV 12528529 12531166 WBGene00009956 F53B2.5 F53B2.5 0.0200357334773988
AGIWUR2861;AGIWUR19916;AGIWUR28988
CB4856 CT IV 12542034 12545729 WBGene00001820 ham-1 F53B2.6 0.100784436009416
AGIWUR1006;AGIWUR4237;AGIWUR6168;AGIWUR14548;AGIWUR16905;AGIWUR17346;AGIWUR23386;AGIWUR25307;AGIWUR33707;AG
IWUR34203;AGIWUR41967
CB4856 CT IV 12548129 12554681 WBGene00014081 ZK795.1 ZK795.1 0.0338571903394094
AGIWUR38827
CB4856 CT IV 12557072 12558975 WBGene00014082 ZK795.2 ZK795.2 0.0471654145563782
AGIWUR8412;AGIWUR17586;AGIWUR33634
CB4856 CT IV 12559365 12560719 WBGene00014083 ZK795.3 ZK795.3 0.0434848139993314
AGIWUR11867
CB4856 CT IV 12560749 12561201 WBGene00014084 snb-7 ZK795.4 -0.291945337740421
AGIWUR15016;AGIWUR39153
CB4856 CT IV 12563775 12566111 WBGene00010671 eak-7 K08E7.1 0.0107851559742151
AGIWUR8409
CB4856 CT IV 12565983 12567226 WBGene00002002 hsb-1 K08E7.2 0.255330857422111
AGIWUR9703
CB4856 CT IV 12567842 12570622 WBGene00002368 let-99 K08E7.3 0.00480636647344923
AGIWUR12809
CB4856 CT IV 12573370 12578870 WBGene00010673 K08E7.5 K08E7.5 0.0622595989107619
AGIWUR8564;AGIWUR19836;AGIWUR31015;AGIWUR31638;AGIWUR38386;AGIWUR39800;AGIWUR40401
CB4856 CT IV 12579055 12581128 WBGene00010674 K08E7.6 K08E7.6 0.292277272575372
AGIWUR39385
CB4856 CT IV 12585192 12586216 WBGene00010675 K08E7.8 K08E7.8 -0.48203065853823
AGIWUR21419
CB4856 CT IV 12586511 12590428 WBGene00000841 cul-6 K08E7.7 0.157965861623792
AGIWUR28440
CB4856 CT IV 12591697 12599169 WBGene00003995 pgp-1 K08E7.9 -0.196475538468981
AGIWUR12495
CB4856 CT IV 12601126 12605425 WBGene00013224 Y55D9A.2 Y55D9A.2
0.00195240416484754 AGIWUR8804;AGIWUR26146
CB4856 CT IV 12605700 12614723 WBGene00013223 efa-6 Y55D9A.1
0.133078728633313
AGIWUR231;AGIWUR5658;AGIWUR8719;AGIWUR14800;AGIWUR29089;AGIWUR32656;AGIWUR33205;AGIWUR34962;AGIWUR36389;AGI
WUR45118

CB4856	CT	IV	12622093	12631022	WBGene00000186	ark-1	C01C7.1	-0.0549242046578371	AGIWUR6011
CB4856	CT	IV	12640447	12650652	WBGene00006916	vha-7	C26H9A.1	-0.11784383211768	AGIWUR10849;AGIWUR20516;AGIWUR33193;AGIWUR40517;AGIWUR43760
CB4856	CT	IV	12653403	12674773	WBGene00007752	wdfy-3	C26H9A.2	-0.13908383362317	AGIWUR6728;AGIWUR14116;AGIWUR23638;AGIWUR41522;AGIWUR41680
CB4856	CT	IV	12675207	12676000	WBGene00011941	T23B5.4	T23B5.4	-0.14889332686592	AGIWUR7224
CB4856	CT	IV	12677398	12684378	WBGene00011939	prmt-9	T23B5.1	0.104623436418709	AGIWUR15729;AGIWUR21183;AGIWUR36147
CB4856	CT	IV	12688655	12703991	WBGene00011940	T23B5.3	T23B5.3	0.167216563464402	AGIWUR2290;AGIWUR2511;AGIWUR8373;AGIWUR17180;AGIWUR29168;AGIWUR32930;AGIWUR32938
CB4856	CT	IV	12704111	12706755	WBGene00007780	C27D8.4	C27D8.4	-0.184601573239039	AGIWUR3968
CB4856	CT	IV	12706714	12711744	WBGene00007779	C27D8.3	C27D8.3	0.0469516037932713	AGIWUR7885
CB4856	CT	IV	12712765	12714771	WBGene00007778	C27D8.2	C27D8.2	0.144045400784357	AGIWUR37386
CB4856	CT	IV	12717831	12719391	WBGene00007777	C27D8.1	C27D8.1	-0.111772838569117	AGIWUR19597
CB4856	CT	IV	12719960	12720997	WBGene00011949	T23F6.1	T23F6.1	-0.507160266511918	AGIWUR555;AGIWUR21553;AGIWUR25722;AGIWUR27233;AGIWUR42033;AGIWUR44778
CB4856	CT	IV	12721356	12723015	WBGene00011950	T23F6.2	T23F6.2	0.161424540322086	AGIWUR6919
CB4856	CT	IV	12723103	12724651	WBGene00011951	T23F6.3	T23F6.3	-0.445412596324431	AGIWUR6892
CB4856	CT	IV	12724948	12730095	WBGene00004315	rbd-1	T23F6.4	-0.17741400166045 8	AGIWUR7447;AGIWUR20940;AGIWUR22173;AGIWUR25199;AGIWUR27648;AGIWUR29331;AGIWUR34066;AGIWUR35698;AGIWUR39765
CB4856	CT	IV	12734262	12734895	WBGene00045184	T23F6.7	T23F6.7	0.0920595739706602	AGIWUR9615;AGIWUR15641;AGIWUR31164;AGIWUR31497;AGIWUR33297
CB4856	CT	IV	12735187	12741523	WBGene00011952	T23F6.5	T23F6.5	0.0266513280532976	AGIWUR36912
CB4856	CT	IV	12743039	12744813	WBGene00007987	C36H8.1	C36H8.1	0.280376421359048	AGIWUR11471;AGIWUR16086;AGIWUR26266
CB4856	CT	IV	12746584	12749721	WBGene00002128	inx-6	C36H8.2	0.350149859225084	AGIWUR8891;AGIWUR37533;AGIWUR44695
CB4856	CT	IV	12754558	12757673	WBGene00001452	flp-9	C36H8.3	0.531288662300115	AGIWUR15622;AGIWUR17526;AGIWUR21930;AGIWUR25621;AGIWUR28951;AGIWUR31273;AGIWUR34191;AGIWUR34712
CB4856	CT	IV	12773862	12787513	WBGene00006767	unc-31	ZK897.1	-0.0800375158235873	

AGIWUR40041
CB4856 CT IV 12789041 12790512 WBGene00008554 F07C6.3 F07C6.3 -0.0299616594499756
AGIWUR872
CB4856 CT IV 12791171 12791685 WBGene00008553 F07C6.2 F07C6.2 -0.158840136837707
AGIWUR329;AGIWUR28099;AGIWUR28140;AGIWUR32182;AGIWUR39821
CB4856 CT IV 12792795 12793274 WBGene00045067 F07C6.6 F07C6.6 -0.125871610789046
AGIWUR17651;AGIWUR27327;AGIWUR27461;AGIWUR32761;AGIWUR42888
CB4856 CT IV 12793816 12796164 WBGene00004030 pin-2 F07C6.1 0.239132035283102
AGIWUR12942
CB4856 CT IV 12805931 12830112 WBGene00008555 F07C6.4 F07C6.4
-0.000853337215861671 AGIWUR5109;AGIWUR10157;AGIWUR13724;AGIWUR35786
CB4856 CT IV 12836510 12840674 WBGene00010828 M02B1.3 M02B1.3 0.0852736843901418
AGIWUR28765;AGIWUR40819;AGIWUR42206
CB4856 CT IV 12845696 12848428 WBGene00010827 M02B1.2 M02B1.2 -0.0449589550545078
AGIWUR34338
CB4856 CT IV 12849506 12854408 WBGene00005153 srf-3 M02B1.1 -0.19231567114755
AGIWUR41492
CB4856 CT IV 12855549 12859928 WBGene00014139 nstp-5 ZK896.9 -0.289348888865669
AGIWUR18936
CB4856 CT IV 12860137 12865935 WBGene00001543 gcy-18 ZK896.8 -0.0384877577778511
AGIWUR29403
CB4856 CT IV 12869270 12871513 WBGene00014137 clec-187 ZK896.6
-0.0924833707291519 AGIWUR9398
CB4856 CT IV 12872829 12875360 WBGene00014132 ZK896.1 ZK896.1 -0.146289295009965
AGIWUR32204
CB4856 CT IV 12876965 12879667 WBGene00014136 ZK896.5 ZK896.5 -0.263619057013665
AGIWUR21817
CB4856 CT IV 12880634 12883146 WBGene00014135 ZK896.4 ZK896.4 0.0284385394797795
AGIWUR34775
CB4856 CT IV 12883505 12886127 WBGene00014134 ZK896.3 ZK896.3 -0.595806231183887
AGIWUR4993;AGIWUR16444;AGIWUR24848
CB4856 CT IV 12887102 12890508 WBGene00010660 K08D8.6 K08D8.6 -0.501009008520004
AGIWUR3610;AGIWUR16326;AGIWUR20149;AGIWUR20866;AGIWUR21444
CB4856 CT IV 12892513 12893283 WBGene00010655 K08D8.1 K08D8.1 0.238506557179294
AGIWUR25160;AGIWUR34928;AGIWUR42392;AGIWUR43870;AGIWUR44833
CB4856 CT IV 12893773 12894724 WBGene00194926 K08D8.12 K08D8.12
0.268810152530097 AGIWUR11140;AGIWUR27285;AGIWUR30099;AGIWUR39581;AGIWUR39736
CB4856 CT IV 12903785 12906659 WBGene00010657 K08D8.3 K08D8.3 -0.472818519435515
AGIWUR5630

CB4856	CT	IV	12907469	12909998	WBGene00010658	K08D8.4	K08D8.4	0.276915352048412
AGIWUR633;AGIWUR4055;AGIWUR20476
CB4856	CT	IV	12910647	12912145	WBGene00010659	K08D8.5	K08D8.5	-0.54828448634856
AGIWUR5854;AGIWUR9526;AGIWUR32040;AGIWUR33244;AGIWUR40070
CB4856	CT	IV	12912790	12914969	WBGene00007864	irg-6	C32H11.1
-0.0114597062319	AGIWUR10713;AGIWUR12335;AGIWUR31481
CB4856	CT	IV	12916043	12917293	WBGene00007865	srz-15	C32H11.2
-0.108555654786269	AGIWUR4336
CB4856	CT	IV	12918611	12920393	WBGene00007866	C32H11.3	C32H11.3
-0.415218081800031	AGIWUR563
CB4856	CT	IV	12921264	12923288	WBGene00007867	C32H11.4	C32H11.4
-0.105406108034806	AGIWUR12257
CB4856	CT	IV	12923525	12924432	WBGene00007868	C32H11.5	C32H11.5
0.0199872265455436	AGIWUR971;AGIWUR25407
CB4856	CT	IV	12925866	12926805	WBGene00007869	C32H11.6	C32H11.6
-0.24068367710536	AGIWUR15139
CB4856	CT	IV	12930707	12931514	WBGene00007870	C32H11.7	C32H11.7
-0.2062541143003	AGIWUR18444
CB4856	CT	IV	12940106	12941405	WBGene00007875	dod-24	C32H11.12
-0.249924091172915	AGIWUR33110
CB4856	CT	IV	12942933	12944526	WBGene00007876	dct-19	C32H11.13
-0.657724294368196	AGIWUR9992
CB4856	CT	IV	12944887	12946577	WBGene00010122	F55G11.1	F55G11.1
-0.477158758947976	AGIWUR12449
CB4856	CT	IV	12950315	12957128	WBGene00000021	abt-3	F55G11.9
-0.137572072591349	AGIWUR38787
CB4856	CT	IV	12958030	12959594	WBGene00010126	F55G11.6	F55G11.6
-0.123939584035345	AGIWUR8760
CB4856	CT	IV	12960436	12962120	WBGene00010127	F55G11.7	F55G11.7
0.154565575630489	AGIWUR9714
CB4856	CT	IV	12962808	12964625	WBGene00010128	F55G11.8	F55G11.8
-0.700256169830112	AGIWUR22605
CB4856	CT	IV	12965387	12967318	WBGene00010125	dod-22	F55G11.5
0.443304361720593	AGIWUR25463
CB4856	CT	IV	12967448	12969289	WBGene00010123	F55G11.2	F55G11.2
-1.24200797098225	AGIWUR27920
CB4856	CT	IV	12970270	12971974	WBGene00014771	F55G11.3	F55G11.3
0.03894004962895	AGIWUR2906;AGIWUR28495;AGIWUR33446;AGIWUR34281;AGIWUR34577;AGIWUR40510
CB4856	CT	IV	12973624	12975591	WBGene00010124	F55G11.4	F55G11.4

-0.713927769265357	AGIWUR16709
CB4856	CT	IV	12976231	12978343	WBGene00010745	dod-17	K10D11.1
-0.904398807310997	AGIWUR40735
CB4856	CT	IV	12979017	12981940	WBGene00010746	K10D11.2	K10D11.2
0.774653269139086	AGIWUR16720;AGIWUR27060
CB4856	CT	IV	12982119	12986088	WBGene00010747	K10D11.3	K10D11.3
0.0889126538739315	AGIWUR375
CB4856	CT	IV	12986726	12989455	WBGene00010750	K10D11.6	K10D11.6
-0.21703723201947	AGIWUR38923
CB4856	CT	IV	12989685	12991843	WBGene00010749	K10D11.5	K10D11.5
-0.165377798711482	AGIWUR32026
CB4856	CT	IV	12992157	12994462	WBGene00010748	K10D11.4	K10D11.4
-0.0216909879495351	AGIWUR23605
CB4856	CT	IV	12994676	12995924	WBGene00045198	K10D11.7	K10D11.7
0.35628328201453	AGIWUR9382;AGIWUR15214;AGIWUR16890;AGIWUR33905;AGIWUR34781
CB4856	CT	IV	13009116	13035955	WBGene00003150	mbk-2	F49E11.1
-0.00782862732995815
AGIWUR1792;AGIWUR5161;AGIWUR6014;AGIWUR7031;AGIWUR7450;AGIWUR7488;AGIWUR8472;AGIWUR8614;AGIWUR9221;AGIWUR11544;AGIWUR14795;AGIWUR17676;AGIWUR17802;AGIWUR18717;AGIWUR18810;AGIWUR19747;AGIWUR20031;AGIWUR20460;AGIWUR21573;AGIWUR21792;AGIWUR22205;AGIWUR25369;AGIWUR26815;AGIWUR28415;AGIWUR30531;AGIWUR31681;AGIWUR33258;AGIWUR34682;AGIWUR35026;AGIWUR44228
CB4856	CT	IV	13035956	13039021	WBGene00009889	F49E11.2	F49E11.2
-0.066181494388414	AGIWUR885;AGIWUR18578;AGIWUR28259;AGIWUR32335
CB4856	CT	IV	13042391	13046858	WBGene00000134	amt-2	F49E11.3
0.166328227256352	AGIWUR13890
CB4856	CT	IV	13047197	13048262	WBGene00009890	scl-9	F49E11.4
-0.609133333820659	AGIWUR5966
CB4856	CT	IV	13048457	13049835	WBGene00009891	scl-10	F49E11.5
-0.514896356617442	AGIWUR31865
CB4856	CT	IV	13051377	13052149	WBGene00009892	scl-11	F49E11.6
-0.232584692064226	AGIWUR843
CB4856	CT	IV	13053297	13055154	WBGene00009893	F49E11.7	F49E11.7
-0.171707770790976	AGIWUR44526
CB4856	CT	IV	13055357	13055768	WBGene00009894	F49E11.8	F49E11.8
0.0799992095286761	AGIWUR30147
CB4856	CT	IV	13056376	13057271	WBGene00004742	scl-1	F49E11.9
0.336233917610283	AGIWUR25578
CB4856	CT	IV	13057678	13059085	WBGene00009895	scl-2	F49E11.10
3.02214621700526	AGIWUR22496

CB4856	CT	IV	13059618	13060695	WBGene00009896	scl-3	F49E11.11	0.0131506345802386	AGIWUR1835
CB4856	CT	IV	13061774	13063205	WBGene00008026	scl-4	C39E9.1	0.20935183951463	AGIWUR40780
CB4856	CT	IV	13064003	13065159	WBGene00008027	scl-5	C39E9.2	0.776670544616103	AGIWUR16531
CB4856	CT	IV	13065766	13067300	WBGene00000705	col-131	C39E9.3	0.335055775680716	AGIWUR12644
CB4856	CT	IV	13068751	13070290	WBGene00008028	scl-6	C39E9.4	-0.70417751056952	AGIWUR458
CB4856	CT	IV	13070479	13071221	WBGene00008029	scl-7	C39E9.5	0.217666536641762	AGIWUR12072
CB4856	CT	IV	13071754	13072478	WBGene00008030	scl-8	C39E9.6	0.146125940395804	AGIWUR44929
CB4856	CT	IV	13072660	13076720	WBGene00008031	C39E9.7	C39E9.7	-0.161450936331986	AGIWUR36436
CB4856	CT	IV	13079446	13082781	WBGene00008032	C39E9.8	C39E9.8	-0.200395836600086	AGIWUR612;AGIWUR22734
CB4856	CT	IV	13087005	13089619	WBGene00008033	spin-2	C39E9.10	0.200828824030346	AGIWUR29849
CB4856	CT	IV	13089578	13091067	WBGene00000706	col-132	C39E9.9	0.0670745099196131	AGIWUR15066
CB4856	CT	IV	13091187	13093853	WBGene00008034	C39E9.11	C39E9.11	-0.139388341847857	AGIWUR17054
CB4856	CT	IV	13094007	13095490	WBGene00008035	C39E9.12	C39E9.12	-0.132734479275594	AGIWUR24925
CB4856	CT	IV	13095762	13097065	WBGene00004339	rfc-3	C39E9.13	-0.153594770329535	AGIWUR144;AGIWUR10555;AGIWUR13424;AGIWUR40682
CB4856	CT	IV	13096958	13098836	WBGene00001007	dli-1	C39E9.14	-0.225010176030646	AGIWUR3418;AGIWUR7426;AGIWUR35722;AGIWUR37928
CB4856	CT	IV	13099378	13100608	WBGene00007201	exos-4.1	B0564.1	0.00123658018284711	AGIWUR27813;AGIWUR33869
CB4856	CT	IV	13100715	13104641	WBGene00007207	B0564.7	B0564.7	-0.392080737965244	AGIWUR2841
CB4856	CT	IV	13105323	13106927	WBGene00023421	rde-11	B0564.11	-0.0740544537814927	AGIWUR1580
CB4856	CT	IV	13107659	13111500	WBGene00007202	B0564.2	B0564.2	0.0416267576980367	AGIWUR9047;AGIWUR11732
CB4856	CT	IV	13119096	13124387	WBGene00006766	unc-30	B0564.10		

0.334280008637236 AGIWUR1590;AGIWUR3331;AGIWUR27447;AGIWUR33252;AGIWUR35405;AGIWUR36465
CB4856 CT IV 13138991 13142354 WBGene00008623 F09E8.1 F09E8.1 -0.41212231447251
AGIWUR42795
CB4856 CT IV 13149576 13150569 WBGene00008626 F09E8.8 F09E8.8 0.219000565873021
AGIWUR38329
CB4856 CT IV 13150552 13154441 WBGene00008624 F09E8.2 F09E8.2 -0.0773071626684505
AGIWUR3879;AGIWUR41374;AGIWUR44977
CB4856 CT IV 13155228 13164056 WBGene00003421 msh-5 F09E8.3 -0.153742979001924
AGIWUR6808;AGIWUR7907;AGIWUR22916
CB4856 CT IV 13165516 13166815 WBGene00008625 scl-14 F09E8.5 0.793223904256964
AGIWUR19778;AGIWUR27818
CB4856 CT IV 13170544 13173694 WBGene00003533 nas-14 F09E8.6
-0.00994380326434907 AGIWUR5805;AGIWUR19870;AGIWUR28940
CB4856 CT IV 13174157 13179457 WBGene00002974 lev-1 F09E8.7 -0.446229630884293
AGIWUR20176
CB4856 CT IV 13180179 13180911 WBGene00044978 F58D2.3 F58D2.3 0.322749224372728
AGIWUR31280
CB4856 CT IV 13183072 13186984 WBGene00010240 gadr-1 F58D2.1 0.130002770133126
AGIWUR28122
CB4856 CT IV 13193563 13197382 WBGene00008177 C48D1.1 C48D1.1 -0.0328975939409801
AGIWUR2280
CB4856 CT IV 13206783 13207278 WBGene00044909 C48D1.7 C48D1.7 -0.0351864654156824
AGIWUR16247;AGIWUR19522;AGIWUR20384;AGIWUR37541;AGIWUR42217
CB4856 CT IV 13209729 13214566 WBGene00000501 cho-1 C48D1.3 -0.0997677085512514
AGIWUR26521
CB4856 CT IV 13221961 13232974 WBGene00008178 C48D1.5 C48D1.5 0.0354802878680758
AGIWUR9021;AGIWUR9601;AGIWUR33314
CB4856 CT IV 13232943 13236585 WBGene00010435 JC8.2 JC8.2 -0.166493635228999
AGIWUR24175
CB4856 CT IV 13237281 13239441 WBGene00006466 tag-115 JC8.13 0.121133214059309
AGIWUR9056
CB4856 CT IV 13239588 13240651 WBGene00004424 rpl-12 JC8.3 0.0089114623710902
AGIWUR4632;AGIWUR6964;AGIWUR7060;AGIWUR9613;AGIWUR11217;AGIWUR13131
CB4856 CT IV 13241051 13243401 WBGene00003037 lin-54 JC8.6 -0.202340836959905
AGIWUR35358
CB4856 CT IV 13243673 13244296 WBGene00010436 JC8.4 JC8.4 -0.346729269231783
AGIWUR18188
CB4856 CT IV 13244324 13247160 WBGene00010437 cox-11 JC8.5 -0.0533421562779868
AGIWUR39629

CB4856	CT	IV	13247261	13250699	WBGene00010439	JC8.7	JC8.7	-0.0950861900938555	AGIWUR3986;AGIWUR23796;AGIWUR29016
CB4856	CT	IV	13250648	13252311	WBGene00010440	ttr-51	JC8.8	0.269997941376658	AGIWUR1763;AGIWUR9407;AGIWUR17465;AGIWUR24987;AGIWUR39926;AGIWUR40897
CB4856	CT	IV	13254225	13255538	WBGene00044737	ttr-45	JC8.14	0.204373310749318	AGIWUR7141;AGIWUR15386;AGIWUR23834;AGIWUR25802;AGIWUR36581
CB4856	CT	IV	13256051	13259076	WBGene00010442	bus-12	JC8.12	0.58598553977345	AGIWUR24223
CB4856	CT	IV	13261556	13272514	WBGene00006763	unc-26	JC8.10	-0.0862961135495572	AGIWUR3166;AGIWUR31782;AGIWUR33371
CB4856	CT	IV	13273911	13282878	WBGene00013460	cpsf-3	Y67H2A.1	-0.273572805781718	AGIWUR210
CB4856	CT	IV	13283002	13289536	WBGene00013465	Y67H2A.10	Y67H2A.10	-0.00497215255850847	AGIWUR36969
CB4856	CT	IV	13289776	13297869	WBGene00013461	Y67H2A.2	Y67H2A.2	-0.157367067324438	AGIWUR41344
CB4856	CT	IV	13297926	13300066	WBGene00013462	micu-1	Y67H2A.4	-0.115471712101317	AGIWUR5915;AGIWUR6075
CB4856	CT	IV	13300179	13300720	WBGene00013463	kdp-1	Y67H2A.5	-0.421664805896816	AGIWUR747
CB4856	CT	IV	13300828	13304145	WBGene00000818	csn-6	Y67H2A.6	0.124434434417555	AGIWUR12038
CB4856	CT	IV	13303746	13310818	WBGene00013464	Y67H2A.7	Y67H2A.7	0.0542035603868847	AGIWUR15280
CB4856	CT	IV	13315644	13318811	WBGene00001393	fat-1	Y67H2A.8	0.484807439014423	AGIWUR12237;AGIWUR36063
CB4856	CT	IV	13320158	13321831	WBGene00014941	Y67H2A.9	Y67H2A.9	-0.412635477765644	AGIWUR1097;AGIWUR1417;AGIWUR12240;AGIWUR18964;AGIWUR22391;AGIWUR43346
CB4856	CT	IV	13323850	13326394	WBGene00001394	fat-2	W02A2.1	0.73380288640935	AGIWUR34363
CB4856	CT	IV	13328359	13329497	WBGene00001390	far-6	W02A2.2	-0.0179605518854515	AGIWUR29425
CB4856	CT	IV	13329508	13331406	WBGene00004156	pqn-74	W02A2.3	-0.525368076969675	AGIWUR7098
CB4856	CT	IV	13342858	13346189	WBGene00012190	W02A2.5	W02A2.5	0.147492613605933	AGIWUR2236;AGIWUR38536
CB4856	CT	IV	13347665	13348599	WBGene00044280	W02A2.9	W02A2.9	0.876383161607258	AGIWUR39519
CB4856	CT	IV	13348870	13353193	WBGene00004333	rec-8	W02A2.6	-0.128853111267166	

AGIWUR6839
CB4856 CT IV 13353723 13356104 WBGene00003230 mex-5 W02A2.7 0.0048076562899606
AGIWUR24629
CB4856 CT IV 13356113 13358601 WBGene00012191 W02A2.8 W02A2.8 0.154377584114449
AGIWUR29239
CB4856 CT IV 13365169 13368435 WBGene00013375 srsx-25 Y62E10A.4
-0.208332913464444 AGIWUR37939
CB4856 CT IV 13368368 13369417 WBGene00000093 agt-1 Y62E10A.5
-0.138227070441506 AGIWUR14160;AGIWUR35728;AGIWUR37488
CB4856 CT IV 13369424 13370285 WBGene00004410 rla-2 Y62E10A.1
0.0629808277885395 AGIWUR9256;AGIWUR30171;AGIWUR41654
CB4856 CT IV 13370653 13371613 WBGene00013373 Y62E10A.2 Y62E10A.2
-0.0797621003734594 AGIWUR32819
CB4856 CT IV 13371999 13377383 WBGene00013376 Y62E10A.6 Y62E10A.6
-0.119507488815028 AGIWUR36222
CB4856 CT IV 13378016 13380407 WBGene00003142 mau-8 Y62E10A.8
0.0829796319899856 AGIWUR11971
CB4856 CT IV 13380387 13381923 WBGene00013378 emc-3 Y62E10A.10
0.107627147676002 AGIWUR43645
CB4856 CT IV 13382055 13384316 WBGene00004278 rab-19 Y62E10A.9
0.0917044466194952 AGIWUR22027;AGIWUR43076;AGIWUR44348
CB4856 CT IV 13383905 13386091 WBGene00044745 Y62E10A.20 Y62E10A.20
-0.19281034745856 AGIWUR15148;AGIWUR29097
CB4856 CT IV 13386294 13387358 WBGene00014938 mdt-9 Y62E10A.11
-0.414861453454665 AGIWUR19242;AGIWUR26065
CB4856 CT IV 13387469 13389209 WBGene00003077 lsm-3 Y62E10A.12
-0.164841599383585 AGIWUR5031;AGIWUR29539;AGIWUR33021;AGIWUR35748;AGIWUR38348;AGIWUR40337
CB4856 CT IV 13389146 13398799 WBGene00013379 Y62E10A.13 Y62E10A.13
0.0169022684609601
AGIWUR1459;AGIWUR1661;AGIWUR21061;AGIWUR32539;AGIWUR32818;AGIWUR34098;AGIWUR35714;AGIWUR43565
CB4856 CT IV 13399068 13400506 WBGene00013380 memi-2 Y62E10A.14
-0.346503821492144 AGIWUR5865;AGIWUR34235
CB4856 CT IV 13401306 13403831 WBGene00013381 cyp-31A5 Y62E10A.15
-0.402157932111667 AGIWUR28184
CB4856 CT IV 13403769 13408306 WBGene00013385 Y62E10A.19 Y62E10A.19
-0.346079968416612 AGIWUR10040;AGIWUR18675;AGIWUR20403;AGIWUR23523;AGIWUR39058;AGIWUR42855;AGIWUR44771
CB4856 CT IV 13408606 13412895 WBGene00013382 gcl-1 Y62E10A.16
0.0148345525320579 AGIWUR39074
CB4856 CT IV 13413560 13415954 WBGene00013383 aptf-2 Y62E10A.17

-0.152940693494739	AGIWUR2952;AGIWUR10679
CB4856	CT	IV	13416041	13421927	WBGene00004029	pik-1	K09B11.1
0.114702013963393	AGIWUR40481
CB4856	CT	IV	13422500	13429379	WBGene00010709	nol-9	K09B11.2
-0.0230746919711571	AGIWUR32343
CB4856	CT	IV	13431288	13434068	WBGene00010712	K09B11.5	K09B11.5
-0.0409570499251051	AGIWUR1133;AGIWUR37524
CB4856	CT	IV	13434188	13445375	WBGene00010714	mam-3	K09B11.10
0.162151620165908	AGIWUR34784
CB4856	CT	IV	13445819	13454954	WBGene00010713	uso-1	K09B11.9
-0.454085507192405	AGIWUR22969
CB4856	CT	IV	13456976	13458841	WBGene00045062	Y38H8A.8	Y38H8A.8
-0.549985797430509	AGIWUR24056
CB4856	CT	IV	13466139	13469402	WBGene00012639	fezf-1	Y38H8A.5
0.193575453468823	AGIWUR31080
CB4856	CT	IV	13474432	13476225	WBGene00012636	Y38H8A.2	Y38H8A.2
-0.0367333597053614	AGIWUR3266;AGIWUR12111;AGIWUR37091;AGIWUR38610;AGIWUR44125;AGIWUR44481
CB4856	CT	IV	13476935	13502852	WBGene00012868	tbc-9	Y45F10A.6
-0.157931675475801	AGIWUR31457
CB4856	CT	IV	13505270	13505762	WBGene00003755	nlp-17	Y45F10A.5
-0.0951609273510714	AGIWUR8556;AGIWUR23648
CB4856	CT	IV	13506079	13509225	WBGene00012867	seld-1	Y45F10A.4
0.191323187468743	AGIWUR10820
CB4856	CT	IV	13509224	13513426	WBGene00012866	afmd-2	Y45F10A.3
0.139853470545528	AGIWUR33223
CB4856	CT	IV	13513369	13521870	WBGene00003582	dcap-2	F52G2.1	0.0984929064925524
AGIWUR14135;AGIWUR28797;AGIWUR33187
CB4856	CT	IV	13521970	13540271	WBGene00004681	rsd-2	F52G2.2	0.0235902556834198
AGIWUR42570
CB4856	CT	IV	13543402	13548230	WBGene00009943	F52G2.3	F52G2.3	-0.145642242240099
AGIWUR43070
CB4856	CT	IV	13549605	13561729	WBGene00012875	Y45F10B.13	Y45F10B.13
-0.547065250198428	AGIWUR1433;AGIWUR1775;AGIWUR16615;AGIWUR37079
CB4856	CT	IV	13561861	13574440	WBGene00004265	qui-1	Y45F10B.10
-0.0729062588348253	AGIWUR414;AGIWUR5539;AGIWUR38295
CB4856	CT	IV	13575058	13576740	WBGene00005088	srd-10	Y45F10B.14
0.0564091869866923	AGIWUR31343
CB4856	CT	IV	13579253	13582112	WBGene00005679	sru-16	Y45F10B.4
-0.0329175079429822	AGIWUR16693;AGIWUR38014

CB4856 CT IV 13583209 13584479 WBGene00005675 sru-12 Y45F10B.5
-0.0780359054873916 AGIWUR43576
CB4856 CT IV 13584946 13587288 WBGene00005674 sru-11 Y45F10B.6
-0.0525034293805886 AGIWUR21838
CB4856 CT IV 13587475 13588237 WBGene00012874 Y45F10B.12 Y45F10B.12
0.147840148056256 AGIWUR35600
CB4856 CT IV 13588993 13590337 WBGene00005672 sru-9 Y45F10B.11
-0.148730604364004 AGIWUR34417
CB4856 CT IV 13594517 13596279 WBGene00012870 Y45F10B.2 Y45F10B.2
-0.158392352210636 AGIWUR36105;AGIWUR41860
CB4856 CT IV 13598755 13600436 WBGene00045045 Y45F10B.17 Y45F10B.17
0.160494044385675 AGIWUR21237
CB4856 CT IV 13603549 13604197 WBGene00012876 Y45F10B.15 Y45F10B.15
-0.0942136565006013 AGIWUR4125
CB4856 CT IV 13604969 13607263 WBGene00045059 Y45F10B.18 Y45F10B.18
0.107614753718542 AGIWUR6109;AGIWUR28546
CB4856 CT IV 13607436 13609892 WBGene00007460 C08F11.13 C08F11.13
0.871403830220175 AGIWUR8724;AGIWUR37159
CB4856 CT IV 13611328 13611579 WBGene00045286 C08F11.19 C08F11.19
0.0460246434914762 AGIWUR5449;AGIWUR14418;AGIWUR21976;AGIWUR24286;AGIWUR27726
CB4856 CT IV 13611812 13612210 WBGene00045285 C08F11.18 C08F11.18
0.0138945032190634 AGIWUR5645;AGIWUR11696;AGIWUR15085;AGIWUR23111;AGIWUR33000
CB4856 CT IV 13612805 13618061 WBGene00007461 C08F11.14 C08F11.14
-0.282064239097057 AGIWUR961
CB4856 CT IV 13619508 13621513 WBGene00007452 C08F11.3 C08F11.3
0.514941210262745 AGIWUR13220;AGIWUR13316;AGIWUR20736;AGIWUR33351
CB4856 CT IV 13621987 13623134 WBGene00007451 C08F11.2 C08F11.2
0.0627922968207741 AGIWUR638;AGIWUR12018;AGIWUR15459;AGIWUR39370
CB4856 CT IV 13633499 13635179 WBGene00007453 fbxa-98 C08F11.5
-0.245566210522751 AGIWUR5958
CB4856 CT IV 13637288 13638525 WBGene00007454 C08F11.7 C08F11.7
0.173291590744823 AGIWUR19769
CB4856 CT IV 13639260 13644402 WBGene00007455 ugt-22 C08F11.8
-0.621790910210464 AGIWUR2529
CB4856 CT IV 13644463 13645923 WBGene00007456 srz-29 C08F11.9
0.0948843750310715 AGIWUR41021
CB4856 CT IV 13646735 13647658 WBGene00007458 ule-4 C08F11.11
-0.218450518717678 AGIWUR11720
CB4856 CT IV 13648039 13649335 WBGene00007457 C08F11.10 C08F11.10

-0.0386988663190024 AGIWUR11031
CB4856 CT IV 13649836 13650338 WBGene00045069 C08F11.17 C08F11.17
-0.0765197301491591 AGIWUR16107;AGIWUR16421;AGIWUR19761;AGIWUR37431
CB4856 CT IV 13651014 13651512 WBGene00007459 C08F11.12 C08F11.12
-0.600318319323328 AGIWUR9420
CB4856 CT IV 13653955 13655851 WBGene00044256 Y45F10C.6 Y45F10C.6
-0.22700620733871 AGIWUR23085
CB4856 CT IV 13662116 13662671 WBGene00012878 Y45F10C.2 Y45F10C.2
-0.330945667004098 AGIWUR37828
CB4856 CT IV 13663398 13664190 WBGene00012880 Y45F10C.4 Y45F10C.4
-0.463568161406119 AGIWUR23361
CB4856 CT IV 13665345 13666909 WBGene00012879 fbxa-215 Y45F10C.3
-0.166818405723095 AGIWUR42106
CB4856 CT IV 13673804 13677823 WBGene00011960 oac-48 T23G4.4 -0.0913922085903547
AGIWUR10707;AGIWUR24857;AGIWUR40749
CB4856 CT IV 13678951 13680333 WBGene00011961 T23G4.5 T23G4.5 -0.517322391857038
AGIWUR35188
CB4856 CT IV 13685076 13687016 WBGene00011959 nyn-1 T23G4.3 -0.244095664800879
AGIWUR1504
CB4856 CT IV 13688324 13689131 WBGene00011958 T23G4.2 T23G4.2 -0.0486233408864818
AGIWUR41972
CB4856 CT IV 13701240 13704082 WBGene00006580 tlp-1 T23G4.1 0.466149453839806
AGIWUR28090
CB4856 CT IV 13726930 13732829 WBGene00008123 ent-4 C47A4.2 -0.363571970850289
AGIWUR21058
CB4856 CT IV 13740951 13742344 WBGene00008124 C47A4.3 C47A4.3 -0.211887688972615
AGIWUR42090
CB4856 CT IV 13742550 13742985 WBGene00045287 C47A4.7 C47A4.7 -0.0427668978514273
AGIWUR25662
CB4856 CT IV 13744155 13744988 WBGene00014703 C47A4.4 C47A4.4 -0.277396146459407
AGIWUR17605
CB4856 CT IV 13745709 13745967 WBGene00008125 C47A4.5 C47A4.5 0.328667991725982
AGIWUR36297
CB4856 CT IV 13750334 13770495 WBGene00012891 sorb-1 Y45F10D.13
-0.0828557565634895 AGIWUR13231;AGIWUR15599;AGIWUR20295;AGIWUR24204;AGIWUR26844
CB4856 CT IV 13773548 13774678 WBGene00012883 Y45F10D.2 Y45F10D.2
0.211573182569122 AGIWUR13053
CB4856 CT IV 13776415 13777149 WBGene00044257 Y45F10D.16 Y45F10D.16
0.101044301908061 AGIWUR729;AGIWUR6269;AGIWUR9214

CB4856 CT IV 13777808 13778812 WBGene00004430 rpl-18 Y45F10D.12
0.0208459243905903 AGIWUR319;AGIWUR4112;AGIWUR9238;AGIWUR37931;AGIWUR41838;AGIWUR44465
CB4856 CT IV 13778866 13782396 WBGene00001635 gly-10 Y45F10D.3
0.0487068631923062 AGIWUR9113
CB4856 CT IV 13782385 13785646 WBGene00012890 lact-9 Y45F10D.11
0.0501436467406187 AGIWUR40217
CB4856 CT IV 13785743 13786961 WBGene00012889 Y45F10D.10 Y45F10D.10
-0.067473810195728 AGIWUR23138;AGIWUR33058;AGIWUR36898;AGIWUR39358
CB4856 CT IV 13786945 13788727 WBGene00012888 sas-6 Y45F10D.9
0.494523536442156 AGIWUR12327
CB4856 CT IV 13788936 13796224 WBGene00012887 Y45F10D.7 Y45F10D.7
-0.216093144489555 AGIWUR26040
CB4856 CT IV 13796255 13797904 WBGene00012886 Y45F10D.6 Y45F10D.6
0.773536916334349 AGIWUR33489
CB4856 CT IV 13804206 13807777 WBGene00012892 Y45F10D.14 Y45F10D.14
-0.327960105080655 AGIWUR25961
CB4856 CT IV 13817487 13820962 WBGene00012893 Y45F10D.15 Y45F10D.15
-0.039426701977094 AGIWUR3598
CB4856 CT IV 13827886 13844997 WBGene00010374 rga-5 H08M01.2
-0.058484678039072
AGIWUR1256;AGIWUR6570;AGIWUR6804;AGIWUR16603;AGIWUR18694;AGIWUR19134;AGIWUR25806;AGIWUR34783;AGIWUR35545;AG
IWUR39495;AGIWUR43718;AGIWUR44172
CB4856 CT IV 13847777 13852712 WBGene00007199 B0513.7 B0513.7 -0.100052978281303
AGIWUR5510;AGIWUR40527
CB4856 CT IV 13880138 13881546 WBGene00007196 B0513.4 B0513.4 0.478974788531691
AGIWUR10382
CB4856 CT IV 13882225 13882620 WBGene00004443 rpl-29 B0513.3 0.0434302112713922
AGIWUR3930;AGIWUR14691;AGIWUR15375;AGIWUR30112;AGIWUR30805;AGIWUR35741;AGIWUR42921
CB4856 CT IV 13882734 13884177 WBGene00007195 B0513.2 B0513.2 -0.185551863795599
AGIWUR4762;AGIWUR15258;AGIWUR30424;AGIWUR32810
CB4856 CT IV 13888089 13893985 WBGene00001562 lin-66 B0513.1 0.150981212252221
AGIWUR19479;AGIWUR20559;AGIWUR42857;AGIWUR43601
CB4856 CT IV 13897817 13902874 WBGene00012536 Y37A1A.3 Y37A1A.3
-0.559865685391782 AGIWUR7072
CB4856 CT IV 13905991 13912297 WBGene00012535 Y37A1A.2 Y37A1A.2
-0.225018867674926 AGIWUR23675;AGIWUR39180;AGIWUR42520
CB4856 CT IV 13934445 13964001 WBGene00045063 Y37A1A.4 Y37A1A.4
-0.225378466662089
AGIWUR6496;AGIWUR7639;AGIWUR12903;AGIWUR16192;AGIWUR20473;AGIWUR20664;AGIWUR21287;AGIWUR22079;AGIWUR26424;A

GIWUR28173;AGIWUR29843;AGIWUR38442;AGIWUR38689
CB4856 CT IV 13964359 13979847 WBGene00044989 Y37A1B.17 Y37A1B.17
-0.0437669570704706 AGIWUR3355;AGIWUR8863;AGIWUR21633;AGIWUR27631;AGIWUR29167
CB4856 CT IV 13981087 13982525 WBGene00001575 gei-18 Y37A1B.15
-0.308466309570403 AGIWUR19287;AGIWUR27437
CB4856 CT IV 13982636 13984141 WBGene00006598 tor-2 Y37A1B.13
-0.448724128583145 AGIWUR21555
CB4856 CT IV 13989094 14012026 WBGene00001190 egl-23 Y37A1B.11
0.0697979674542992 AGIWUR25296
CB4856 CT IV 14014024 14015944 WBGene00005810 srw-63 Y37A1B.10
0.0704894571596144 AGIWUR34340
CB4856 CT IV 14016099 14019983 WBGene00012542 irld-56 Y37A1B.9
-0.0657332838168507 AGIWUR18144
CB4856 CT IV 14020198 14024628 WBGene00012541 irld-55 Y37A1B.8
-0.0397794997686649 AGIWUR38811
CB4856 CT IV 14042595 14043780 WBGene00012540 Y37A1B.7 Y37A1B.7
0.434206995748355 AGIWUR38138
CB4856 CT IV 14051200 14056676 WBGene00012538 Y37A1B.5 Y37A1B.5
0.330691332081219 AGIWUR6852;AGIWUR40734
CB4856 CT IV 14059551 14060712 WBGene00012537 Y37A1B.4 Y37A1B.4
-0.0506828551703435 AGIWUR1505;AGIWUR10911;AGIWUR16676;AGIWUR44769
CB4856 CT IV 14061005 14069854 WBGene00003086 lst-4 Y37A1B.2
0.00299614950677984 AGIWUR3964;AGIWUR20485;AGIWUR37987
CB4856 CT IV 14071503 14076743 WBGene00003085 ccar-1 Y37A1B.1
-0.0990704078978979 AGIWUR19476;AGIWUR27341
CB4856 CT IV 14077441 14083559 WBGene00009924 cfp-1 F52B11.1
0.0228276434486077 AGIWUR34871
CB4856 CT IV 14083318 14084695 WBGene00009925 F52B11.2 F52B11.2
-0.313562129288179 AGIWUR6410
CB4856 CT IV 14091254 14096617 WBGene00009926 noah-2 F52B11.3
0.931414150850465 AGIWUR42435
CB4856 CT IV 14098325 14099368 WBGene00000707 col-133 F52B11.4
-0.257587780923133 AGIWUR23056
CB4856 CT IV 14102095 14103231 WBGene00009927 F52B11.5 F52B11.5
-0.30807671963996 AGIWUR2614;AGIWUR25317;AGIWUR39751
CB4856 CT IV 14106584 14107715 WBGene00002117 ins-34 F52B11.6
0.168509720391112 AGIWUR41432
CB4856 CT IV 14107721 14116034 WBGene00012543 nkcc-1 Y37A1C.1
-0.297456277423622 AGIWUR2790;AGIWUR15781;AGIWUR21124;AGIWUR37379;AGIWUR39725

CB4856	CT	IV	14129391	14131061	WBGene00010388	srz-42	H12I19.1
0.246625142084545	AGIWUR30887
CB4856	CT	IV	14132070	14134330	WBGene00010394	srz-59	H12I19.7
-0.0609709989290224	AGIWUR36275
CB4856	CT	IV	14142081	14143873	WBGene00010390	srz-30	H12I19.3
0.0325396394067665	AGIWUR3130;AGIWUR15872;AGIWUR25242;AGIWUR29676;AGIWUR32133;AGIWUR42099
CB4856	CT	IV	14145665	14148869	WBGene00010391	oac-37	H12I19.4
0.01215283180548	AGIWUR12381
CB4856	CT	IV	14150331	14153361	WBGene00010392	oac-38	H12I19.5
0.0229793429026861	AGIWUR26448
CB4856	CT	IV	14154511	14154913	WBGene00010395	H12I19.8	H12I19.8
-0.161315666679467	AGIWUR15654
CB4856	CT	IV	14155270	14158048	WBGene00011025	R05A10.8	R05A10.8
0.534264447913082	AGIWUR20126;AGIWUR41257
CB4856	CT	IV	14160354	14162872	WBGene00011024	R05A10.7	R05A10.7
0.103787044492036	AGIWUR13597
CB4856	CT	IV	14165776	14168487	WBGene00011023	R05A10.6	R05A10.6
-0.29121504346895	AGIWUR7296
CB4856	CT	IV	14168906	14170838	WBGene00011022	R05A10.5	R05A10.5
-0.397066128363034	AGIWUR17174
CB4856	CT	IV	14172481	14173216	WBGene00011021	R05A10.4	R05A10.4
0.401711510695257	AGIWUR13880
CB4856	CT	IV	14173642	14176473	WBGene00011020	R05A10.3	R05A10.3
-0.0271622327174814	AGIWUR1079
CB4856	CT	IV	14177200	14178940	WBGene00011019	R05A10.2	R05A10.2
0.0119609212343867	AGIWUR9589
CB4856	CT	IV	14182377	14192674	WBGene00011018	R05A10.1	R05A10.1
0.0555739007339469	AGIWUR11624;AGIWUR43235
CB4856	CT	IV	14193430	14195989	WBGene00010483	K01G12.3	K01G12.3
-0.473406620776937	AGIWUR23314;AGIWUR30947
CB4856	CT	IV	14196440	14198824	WBGene00008535	F02H6.2	F02H6.2	-0.264342134747027
AGIWUR3140;AGIWUR7706
CB4856	CT	IV	14199263	14201780	WBGene00008534	F02H6.1	F02H6.1	-0.498048623402738
AGIWUR21214
CB4856	CT	IV	14203427	14205357	WBGene00008536	F02H6.3	F02H6.3	-0.278205515171344
AGIWUR10428;AGIWUR21966;AGIWUR28377;AGIWUR31985
CB4856	CT	IV	14207564	14219159	WBGene00008537	F02H6.4	F02H6.4	-0.127860772683555
AGIWUR12439
CB4856	CT	IV	14219871	14224439	WBGene00008538	sqrd-1	F02H6.5	-0.196986517407336

AGIWUR17864
CB4856 CT IV 14225918 14228169 WBGene00008540 F02H6.7 F02H6.7 0.0537645735729804
AGIWUR14179
CB4856 CT IV 14229336 14230899 WBGene00008539 F02H6.6 F02H6.6 -0.215518545351475
AGIWUR2933;AGIWUR31731;AGIWUR36044
CB4856 CT IV 14234341 14239076 WBGene00013411 Y64G10A.1 Y64G10A.1
-0.132983310893343 AGIWUR24728
CB4856 CT IV 14245439 14250213 WBGene00013417 srab-24 Y64G10A.8
-0.0872657267299255 AGIWUR31275;AGIWUR33166;AGIWUR42828
CB4856 CT IV 14276466 14285523 WBGene00013415 ilcr-1 Y64G10A.6
0.129402587594466 AGIWUR37644
CB4856 CT IV 14291901 14319564 WBGene00013416 Y64G10A.7 Y64G10A.7
0.0730913858046014 AGIWUR16745;AGIWUR24103;AGIWUR27809;AGIWUR41358;AGIWUR41377;AGIWUR42644
CB4856 CT IV 14319834 14321755 WBGene00013454 fbxa-126 Y67A10A.5
0.0420659867884385 AGIWUR8515
CB4856 CT IV 14323244 14325665 WBGene00013453 fbxa-83 Y67A10A.4
-0.332347386732767 AGIWUR15944
CB4856 CT IV 14328995 14334459 WBGene00013452 Y67A10A.3 Y67A10A.3
0.163752087802246 AGIWUR42727
CB4856 CT IV 14335194 14336491 WBGene00045099 Y67A10A.13 Y67A10A.13
0.231799658686201 AGIWUR19281;AGIWUR37071
CB4856 CT IV 14338057 14339279 WBGene00013451 Y67A10A.2 Y67A10A.2
-0.350299726760278 AGIWUR26715;AGIWUR40793
CB4856 CT IV 14339968 14342112 WBGene00044906 Y67A10A.11 Y67A10A.11
-0.224891089963484 AGIWUR5989
CB4856 CT IV 14342660 14343856 WBGene00013459 Y67A10A.10 Y67A10A.10
-0.142865853244306 AGIWUR41956
CB4856 CT IV 14345435 14350751 WBGene00013450 oac-56 Y67A10A.1
0.583309031449097 AGIWUR22040
CB4856 CT IV 14374114 14379643 WBGene00013456 Y67A10A.7 Y67A10A.7
0.320209622388672 AGIWUR10747;AGIWUR41785
CB4856 CT IV 14379953 14383701 WBGene00013457 paqr-3 Y67A10A.8
-0.181441850154608 AGIWUR12238
CB4856 CT IV 14394138 14400368 WBGene00013458 Y67A10A.9 Y67A10A.9
0.20689374102494 AGIWUR35244
CB4856 CT IV 14419711 14422038 WBGene00045060 F55B11.7 F55B11.7
-0.212081059231444 AGIWUR20609;AGIWUR37396
CB4856 CT IV 14422268 14423294 WBGene00010084 F55B11.2 F55B11.2
-0.0302427962046359 AGIWUR9340

CB4856	CT	IV	14424693	14425859	WBGene00010085	F55B11.3	F55B11.3
-0.851786293292218	AGIWUR26421
CB4856	CT	IV	14427695	14428464	WBGene00010087	F55B11.5	F55B11.5
-0.131784522275435	AGIWUR37102
CB4856	CT	IV	14429574	14430920	WBGene00010086	F55B11.4	F55B11.4
-0.114408481933465	AGIWUR5871;AGIWUR7295;AGIWUR13797;AGIWUR39876;AGIWUR41646
CB4856	CT	IV	14436367	14444183	WBGene00006606	tra-3	LLC1.1	-0.36870127445475
AGIWUR18748
CB4856	CT	IV	14456900	14464098	WBGene00010793	LLC1.2	LLC1.2	-0.545721113809655
AGIWUR19648
CB4856	CT	IV	14464517	14469136	WBGene00010794	dld-1	LLC1.3	-0.0621106899468646
AGIWUR1832;AGIWUR15974;AGIWUR42021
CB4856	CT	IV	14482334	14486439	WBGene00013292	Y57G11A.4	Y57G11A.4
-0.0204150093315398	AGIWUR25101
CB4856	CT	IV	14487086	14505166	WBGene00013289	tag-273	Y57G11A.1
-0.036430416669825	AGIWUR3903;AGIWUR7605;AGIWUR27692
CB4856	CT	IV	14511884	14512505	WBGene00013293	Y57G11A.5	Y57G11A.5
-0.0821507723754413	AGIWUR30207
CB4856	CT	IV	14514376	14515118	WBGene00044097	srh-64	Y57G11A.6
0.110903450165401	AGIWUR28692
CB4856	CT	IV	14517022	14519539	WBGene00013290	Y57G11A.2	Y57G11A.2
0.172464584603778	AGIWUR16288
CB4856	CT	IV	14519759	14520999	WBGene00012087	T27E7.1	T27E7.1	0.0578565002792857
AGIWUR4550
CB4856	CT	IV	14522464	14523677	WBGene00005286	srh-65	T27E7.2	0.264062246565034
AGIWUR5005
CB4856	CT	IV	14523868	14525197	WBGene00005285	srh-63	T27E7.8	-0.161667378254776
AGIWUR20602
CB4856	CT	IV	14527093	14527343	WBGene00045313	T27E7.12	T27E7.12
-0.146060964761862	AGIWUR32313
CB4856	CT	IV	14529018	14530809	WBGene00012088	T27E7.3	T27E7.3	0.0704629518002946
AGIWUR32906
CB4856	CT	IV	14531180	14531646	WBGene00044268	T27E7.10	T27E7.10
-0.148295457716565	AGIWUR17144;AGIWUR40545
CB4856	CT	IV	14533518	14535067	WBGene00012089	T27E7.4	T27E7.4	-0.196463925363231
AGIWUR36082
CB4856	CT	IV	14537163	14538325	WBGene00012090	T27E7.5	T27E7.5	0.0774554201105513
AGIWUR17253
CB4856	CT	IV	14539762	14542602	WBGene00012091	pals-29	T27E7.6	1.14226243067419

AGIWUR2847;AGIWUR6198;AGIWUR10921
CB4856	CT	IV	14546933	14549386	WBGene00012093	T27E7.9	T27E7.9	0.205841382214181	AGIWUR33290
CB4856	CT	IV	14549827	14551957	WBGene00013294	pals-30	Y57G11B.1	0.0834587280738726	AGIWUR43995
CB4856	CT	IV	14553598	14554883	WBGene00013298	Y57G11B.6	Y57G11B.6	-0.0799961526756476	AGIWUR21498
CB4856	CT	IV	14565330	14567331	WBGene00013295	Y57G11B.2	Y57G11B.2	0.125575393942812	AGIWUR36699
CB4856	CT	IV	14570160	14572721	WBGene00077763	Y57G11B.97	Y57G11B.97	0.14379364693074	AGIWUR12079;AGIWUR37121
CB4856	CT	IV	14574819	14576580	WBGene00013297	Y57G11B.5	Y57G11B.5	-0.150621565116452	AGIWUR33753
CB4856	CT	IV	14577004	14577273	WBGene00255522	Y57G11B.223	Y57G11B.223	0.140076366191308	AGIWUR36876
CB4856	CT	IV	14616603	14623961	WBGene00007058	dmd-6	F13G11.1	0.0152210349209989	AGIWUR18122;AGIWUR41975
CB4856	CT	IV	14633099	14634405	WBGene00008772	irld-4	F13G11.2	-0.456888608618028	AGIWUR19940
CB4856	CT	IV	14635126	14636051	WBGene00045038	ule-2	F13G11.3	0.0140540100577127	AGIWUR16361
CB4856	CT	IV	14637751	14641229	WBGene00013328	Y57G11C.39	Y57G11C.39	0.514675777169681	AGIWUR36645
CB4856	CT	IV	14643025	14653376	WBGene00013322	Y57G11C.32	Y57G11C.32	0.354427138004041	AGIWUR13955
CB4856	CT	IV	14655535	14655916	WBGene00045282	Y57G11C.56	Y57G11C.56	0.157465994550767	AGIWUR27072;AGIWUR40230
CB4856	CT	IV	14657081	14658165	WBGene00013323	Y57G11C.33	Y57G11C.33	-0.170289535738793	AGIWUR11773;AGIWUR31272
CB4856	CT	IV	14658319	14675141	WBGene00013326	anoh-2	Y57G11C.37	0.230733302398043	AGIWUR43237
CB4856	CT	IV	14683043	14687405	WBGene00013321	Y57G11C.31	Y57G11C.31	0.0250579253416561	AGIWUR12323
CB4856	CT	IV	14694032	14699940	WBGene00001818	haf-8	Y57G11C.1	0.130641160303642	AGIWUR32154
CB4856	CT	IV	14703209	14704255	WBGene00013329	Y57G11C.40	Y57G11C.40	-0.436822003280803	AGIWUR30195
CB4856	CT	IV	14705536	14706402	WBGene00013330	Y57G11C.41	Y57G11C.41	-0.766562080650253	AGIWUR28191

CB4856 CT IV 14728534 14745676 WBGene00013331 Y57G11C.42 Y57G11C.42
-0.254344100403515 AGIWUR6177;AGIWUR42172;AGIWUR44605
CB4856 CT IV 14754089 14754969 WBGene00077762 Y57G11C.499 Y57G11C.499
-0.158460277506911 AGIWUR36923
CB4856 CT IV 14758362 14760945 WBGene00013304 Y57G11C.6 Y57G11C.6
0.184151447643684 AGIWUR5415;AGIWUR6344
CB4856 CT IV 14761818 14765434 WBGene00013303 Y57G11C.5 Y57G11C.5
0.636594377072407 AGIWUR557
CB4856 CT IV 14765629 14766665 WBGene00013302 vti-1 Y57G11C.4
-0.253959331095249 AGIWUR18568
CB4856 CT IV 14766773 14767730 WBGene00013324 mrps-7 Y57G11C.34
-0.303369783476832 AGIWUR41282
CB4856 CT IV 14768155 14769282 WBGene00013301 Y57G11C.3 Y57G11C.3
-0.151327606713537 AGIWUR1560;AGIWUR3449;AGIWUR5876
CB4856 CT IV 14769441 14770719 WBGene00013332 Y57G11C.43 Y57G11C.43
0.290360808751668 AGIWUR11667
CB4856 CT IV 14770654 14771425 WBGene00013333 Y57G11C.44 Y57G11C.44
0.274066023675699 AGIWUR25285
CB4856 CT IV 14771419 14773535 WBGene00013325 Y57G11C.36 Y57G11C.36
0.260232732375183 AGIWUR44730
CB4856 CT IV 14773598 14774634 WBGene00044258 Y57G11C.51 Y57G11C.51
-0.0946437590845406 AGIWUR44818
CB4856 CT IV 14780940 14781266 WBGene00045311 Y57G11C.57 Y57G11C.57
-0.0813165615867391 AGIWUR23356
CB4856 CT IV 14782644 14783342 WBGene00194649 Y57G11C.1130 Y57G11C.1130
0.101156636496983 AGIWUR29716
CB4856 CT IV 14783341 14784252 WBGene00013334 nlp-56 Y57G11C.45
-0.0644637273317077 AGIWUR32710
CB4856 CT IV 14787168 14790843 WBGene00013307 Y57G11C.9 Y57G11C.9
-0.0201462675218123
AGIWUR12676;AGIWUR14588;AGIWUR17507;AGIWUR18185;AGIWUR18687;AGIWUR32905;AGIWUR40587;AGIWUR43681
CB4856 CT IV 14790818 14792887 WBGene00001558 gdi-1 Y57G11C.10
-0.229319827013132 AGIWUR20763;AGIWUR35736;AGIWUR44186
CB4856 CT IV 14793119 14796375 WBGene00000763 coq-3 Y57G11C.11
-0.220645575780498 AGIWUR3632;AGIWUR32574;AGIWUR39151;AGIWUR42391;AGIWUR43225
CB4856 CT IV 14796417 14797544 WBGene00013308 nuo-3 Y57G11C.12
-0.0269912303443185
AGIWUR900;AGIWUR4845;AGIWUR13485;AGIWUR14053;AGIWUR19896;AGIWUR20504;AGIWUR26997;AGIWUR35472;AGIWUR38753;AG
IWUR41292;AGIWUR42681

CB4856 CT IV 14798149 14799229 WBGene00000192 arl-8 Y57G11C.13
-0.159945480532611 AGIWUR2999;AGIWUR15427;AGIWUR15779;AGIWUR17064;AGIWUR28321;AGIWUR41048
CB4856 CT IV 14799311 14801063 WBGene00013310 Y57G11C.14 Y57G11C.14
-0.130023123593254 AGIWUR26615
CB4856 CT IV 14803103 14803480 WBGene00045029 Y57G11C.54 Y57G11C.54
-0.0602312135912566 AGIWUR4085;AGIWUR6020;AGIWUR12212;AGIWUR20576;AGIWUR28328
CB4856 CT IV 14807189 14809958 WBGene00023520 oig-5 Y57G11C.50
-0.0156942591159588 AGIWUR17302;AGIWUR29275
CB4856 CT IV 14822540 14825264 WBGene00013311 sec-61 Y57G11C.15
-0.298058049856618 AGIWUR26320
CB4856 CT IV 14825679 14826448 WBGene00004487 rps-18 Y57G11C.16
0.00598745816712665 AGIWUR31968;AGIWUR37362
CB4856 CT IV 14826449 14829115 WBGene00013312 hhat-2 Y57G11C.17
0.385437223510914 AGIWUR33555
CB4856 CT IV 14837112 14853662 WBGene00013317 Y57G11C.22 Y57G11C.22
0.258280989025247 AGIWUR3328;AGIWUR9873
CB4856 CT IV 14854003 14854426 WBGene00044741 Y57G11C.52 Y57G11C.52
-0.517812511327964 AGIWUR2076;AGIWUR10413;AGIWUR32636;AGIWUR36749;AGIWUR37684
CB4856 CT IV 14855331 14861791 WBGene00013318 Y57G11C.23 Y57G11C.23
-0.0813578842944676 AGIWUR4719
CB4856 CT IV 14862447 14869951 WBGene00013327 Y57G11C.38 Y57G11C.38
-0.232951332887088 AGIWUR15936
CB4856 CT IV 14879490 14900652 WBGene00001330 eps-8 Y57G11C.24
-0.0399290998179822 AGIWUR641;AGIWUR27191;AGIWUR32076;AGIWUR35401;AGIWUR36433
CB4856 CT IV 14904994 14909686 WBGene00013336 pnc-2 Y57G11C.47
0.133104654889477 AGIWUR24181
CB4856 CT IV 14919610 14920421 WBGene00013319 ccch-5 Y57G11C.25
-0.313936957404007 AGIWUR21538
CB4856 CT IV 14924444 14926046 WBGene00014923 Y57G11C.26 Y57G11C.26
0.163802402983126 AGIWUR14344;AGIWUR26021
CB4856 CT IV 14927059 14928623 WBGene00014925 Y57G11C.29 Y57G11C.29
0.00637581123996167 AGIWUR7530;AGIWUR39249
CB4856 CT IV 14929721 14931931 WBGene00014924 Y57G11C.28 Y57G11C.28
0.122570705373589 AGIWUR40086
CB4856 CT IV 14938896 14941898 WBGene00014926 Y57G11C.30 Y57G11C.30
0.000240575470336069 AGIWUR37285
CB4856 CT IV 14942286 14945328 WBGene00013337 Y57G11C.48 Y57G11C.48
0.195778313145182 AGIWUR33033
CB4856 CT IV 14950652 14954226 WBGene00045146 Y41E3.22 Y41E3.22

-0.147370943116879	AGIWUR11004;AGIWUR13696;AGIWUR31781;AGIWUR44633
CB4856	CT	IV	14954206	14958914	WBGene00012762	Y41E3.1	Y41E3.1	0.133022388436814
AGIWUR33798
CB4856	CT	IV	14974292	14974561	WBGene00045000	Y41E3.20	Y41E3.20
-0.21683571288725	AGIWUR5211
CB4856	CT	IV	14981609	14996306	WBGene00012763	atln-2	Y41E3.3	0.109432983297064
AGIWUR39088
CB4856	CT	IV	15008503	15013183	WBGene00001336	qars-1	Y41E3.4	0.0365369246391403
AGIWUR33851
CB4856	CT	IV	15014042	15025513	WBGene00012765	Y41E3.7	Y41E3.7	0.0428512067201492
AGIWUR7796;AGIWUR32310;AGIWUR32659;AGIWUR37732
CB4856	CT	IV	15025710	15037036	WBGene00012767	fcd-2	Y41E3.9	-0.0915882148919938
AGIWUR24036
CB4856	CT	IV	15037321	15039551	WBGene00012768	eef-1B.2	Y41E3.10
-0.0744042993143896	AGIWUR22719;AGIWUR28123;AGIWUR37620
CB4856	CT	IV	15039619	15049273	WBGene00012769	Y41E3.11	Y41E3.11
0.0404739500375837	AGIWUR7425;AGIWUR12350;AGIWUR28909;AGIWUR36823
CB4856	CT	IV	15049411	15053371	WBGene00012770	srt-47	Y41E3.12
0.0338655594030719	AGIWUR5695
CB4856	CT	IV	15056131	15057889	WBGene00044979	Y41E3.19	Y41E3.19
0.154303482559436	AGIWUR38414
CB4856	CT	IV	15058294	15058694	WBGene00012771	Y41E3.13	Y41E3.13
-0.0616331953008231	AGIWUR39152
CB4856	CT	IV	15061929	15065748	WBGene00044003	Y41E3.18	Y41E3.18
-0.0594261529946181	AGIWUR10034;AGIWUR15352
CB4856	CT	IV	15065866	15068918	WBGene00012773	srt-48	Y41E3.15
-0.17414049711241	AGIWUR37577;AGIWUR44448
CB4856	CT	IV	15074692	15088581	WBGene00012775	srt-50	Y41E3.17
-0.0806716449784139	AGIWUR4268;AGIWUR5689;AGIWUR13957;AGIWUR21801;AGIWUR28319;AGIWUR36582
CB4856	CT	IV	15092881	15098376	WBGene00012772	srt-49	Y41E3.14
0.0946705006790518	AGIWUR25669
CB4856	CT	IV	15103629	15106483	WBGene00010950	srt-44	M199.1	0.0644184382305237
AGIWUR28281
CB4856	CT	IV	15108775	15110915	WBGene00010951	M199.2	M199.2	-0.258031362896284
AGIWUR10267
CB4856	CT	IV	15115569	15117823	WBGene00010952	clec-188	M199.3
0.0823971193576671	AGIWUR27564
CB4856	CT	IV	15120519	15122548	WBGene00010954	clec-189	M199.6
0.188939507865435	AGIWUR32713

CB4856	CT	IV	15123182	15124393	WBGene00010955	M199.7	M199.7	0.142048587243739
AGIWUR22985
CB4856	CT	IV	15124931	15126122	WBGene00010953	clec-190	M199.4
-0.482767021034439	AGIWUR10019
CB4856	CT	IV	15127491	15127794	WBGene00044998	M199.9	M199.9	-0.388526800088384
AGIWUR14687;AGIWUR16889;AGIWUR27904;AGIWUR31600;AGIWUR42202
CB4856	CT	IV	15129666	15131797	WBGene00000708	col-135	M199.5	0.713048786934957
AGIWUR42402
CB4856	CT	IV	15139101	15140932	WBGene00005208	srg-51	Y40H7A.8
0.0655804609117174	AGIWUR28884
CB4856	CT	IV	15143773	15147204	WBGene00006138	str-78	Y40H7A.1
0.04129347785815	AGIWUR5149
CB4856	CT	IV	15167800	15175536	WBGene00012744	Y40H7A.4	Y40H7A.4
-0.187258754074667
AGIWUR1824;AGIWUR18651;AGIWUR20328;AGIWUR20976;AGIWUR27625;AGIWUR34304;AGIWUR40849;AGIWUR41662
CB4856	CT	IV	15179922	15182953	WBGene00005101	srd-23	Y40H7A.5
-0.0902431649256563	AGIWUR6509
CB4856	CT	IV	15192299	15203023	WBGene00005056	sra-30	Y40H7A.6
-0.225657584085073	AGIWUR24500
CB4856	CT	IV	15207593	15209522	WBGene00012746	Y40H7A.9	Y40H7A.9
-0.216347757595761	AGIWUR21815
CB4856	CT	IV	15210123	15215626	WBGene00012747	Y40H7A.10	Y40H7A.10
0.0359074477033197	AGIWUR12949
CB4856	CT	IV	15219686	15220346	WBGene00012748	Y40H7A.11	Y40H7A.11
-0.228053671352669	AGIWUR36967
CB4856	CT	IV	15236251	15243675	WBGene00013517	lgc-52	Y73F8A.2
0.0820713644024078	AGIWUR9569
CB4856	CT	IV	15245955	15246371	WBGene00044259	Y73F8A.36	Y73F8A.36
0.0852235223037727	AGIWUR13828;AGIWUR18916;AGIWUR29753;AGIWUR30089;AGIWUR40751
CB4856	CT	IV	15250152	15252257	WBGene00005625	srj-42	Y73F8A.3
0.0860808272225508	AGIWUR32614;AGIWUR37734;AGIWUR41029
CB4856	CT	IV	15255690	15257628	WBGene00014948	Y73F8A.4	Y73F8A.4
0.0949800763608257	AGIWUR6659;AGIWUR23773
CB4856	CT	IV	15259879	15269045	WBGene00013518	Y73F8A.5	Y73F8A.5
0.031452274651361	AGIWUR5580
CB4856	CT	IV	15270160	15275835	WBGene00000370	ccg-1	Y73F8A.6
0.594659497605406	AGIWUR40979
CB4856	CT	IV	15288019	15289505	WBGene00006079	str-11	Y73F8A.7
0.0701186283171857	AGIWUR13196;AGIWUR43672

CB4856	CT	IV	15294340	15295297	WBGene00013519	Y73F8A.10	Y73F8A.10
-0.139225865196643	AGIWUR2474
CB4856	CT	IV	15302421	15309408	WBGene00013520	best-21	Y73F8A.11
-0.0554678295212199	AGIWUR25474
CB4856	CT	IV	15312247	15313982	WBGene00013522	Y73F8A.13	Y73F8A.13
-0.242521669807525	AGIWUR16964
CB4856	CT	IV	15321579	15322183	WBGene00013524	Y73F8A.15	Y73F8A.15
-0.208164321536785	AGIWUR25245
CB4856	CT	IV	15323893	15325189	WBGene00006558	tbx-39	Y73F8A.16
0.152219687582918	AGIWUR40555
CB4856	CT	IV	15326939	15328186	WBGene00006559	tbx-40	Y73F8A.17
-0.0539153595959139	AGIWUR22353
CB4856	CT	IV	15337078	15339821	WBGene00013525	irld-59	Y73F8A.18
0.182915481854963	AGIWUR27948
CB4856	CT	IV	15361555	15368260	WBGene00006441	cpna-4	Y73F8A.19
0.287692560645624	AGIWUR10706
CB4856	CT	IV	15378460	15381686	WBGene00013526	Y73F8A.20	Y73F8A.20
0.240174269572865	AGIWUR35971
CB4856	CT	IV	15423920	15424366	WBGene00045025	Y73F8A.39	Y73F8A.39
-0.0198716496147568	AGIWUR8018;AGIWUR20983;AGIWUR30345;AGIWUR42566;AGIWUR43731
CB4856	CT	IV	15442960	15444581	WBGene00013528	Y73F8A.23	Y73F8A.23
0.00292890663704881	AGIWUR39226
CB4856	CT	IV	15445368	15453187	WBGene00013529	Y73F8A.24	Y73F8A.24
0.0216345767514454	AGIWUR6088
CB4856	CT	IV	15455137	15471488	WBGene00013530	ntl-11	Y73F8A.25
-0.362189442817641
AGIWUR1774;AGIWUR2229;AGIWUR11645;AGIWUR12402;AGIWUR18303;AGIWUR25732;AGIWUR28053;AGIWUR29737;AGIWUR35606;A
GIWUR37418;AGIWUR42623
CB4856	CT	IV	15472809	15473682	WBGene00013532	Y73F8A.27	Y73F8A.27
-0.00967698842728026	AGIWUR8366;AGIWUR15188;AGIWUR20529;AGIWUR21845;AGIWUR23023;AGIWUR31382
CB4856	CT	IV	15473660	15477512	WBGene00013531	Y73F8A.26	Y73F8A.26
-0.00255814926477971	AGIWUR6318;AGIWUR34926
CB4856	CT	IV	15497567	15510980	WBGene00013534	acr-24	Y73F8A.30
-0.394754039520862	AGIWUR3707
CB4856	CT	IV	15513786	15514464	WBGene00013535	gyg-3	Y73F8A.31
-0.0497531480603479	AGIWUR1385;AGIWUR8902;AGIWUR21518;AGIWUR34407
CB4856	CT	IV	15514537	15521258	WBGene00013536	Y73F8A.32	Y73F8A.32
-0.0447766622972265	AGIWUR2334;AGIWUR33625
CB4856	CT	IV	15533167	15539959	WBGene00013539	Y73F8A.35	Y73F8A.35

0.0360147025029409	AGIWUR42953
CB4856	CT	IV	15550854	15554721	WBGene00013632	Y105C5A.1	Y105C5A.1
-0.0220320002056482	AGIWUR3354;AGIWUR13296;AGIWUR13372;AGIWUR23799;AGIWUR23986;AGIWUR28284;AGIWUR43025
CB4856	CT	IV	15591973	15592386	WBGene00045016	Y105C5A.30	Y105C5A.30
0.0941900610929469	AGIWUR25604;AGIWUR37634;AGIWUR39230
CB4856	CT	IV	15612768	15617087	WBGene00013633	Y105C5A.8	Y105C5A.8
0.366181352647725	AGIWUR28785
CB4856	CT	IV	15646031	15646433	WBGene00185102	Y105C5A.1268	Y105C5A.1268
-0.000712178005018771	AGIWUR29947
CB4856	CT	IV	15689201	15722267	WBGene00013639	Y105C5A.15	Y105C5A.15
0.192964187359923	AGIWUR7273;AGIWUR15147;AGIWUR15364;AGIWUR16294;AGIWUR25313
CB4856	CT	IV	15820059	15825617	WBGene00013642	daf-38	Y105C5A.23
-0.234514252582912	AGIWUR18586
CB4856	CT	IV	15849875	15852905	WBGene00013643	Y105C5A.24	Y105C5A.24
-0.0998488928914352	AGIWUR15451
CB4856	CT	IV	15860221	15861442	WBGene00013644	Y105C5A.25	Y105C5A.25
-0.151316089232338	AGIWUR14488
CB4856	CT	IV	15870643	15872221	WBGene00013645	Y105C5B.1	Y105C5B.1
0.28131795275247	AGIWUR22250
CB4856	CT	IV	15872933	15882377	WBGene00001549	gcy-25	Y105C5B.2
0.238962070258736	AGIWUR4121;AGIWUR28709
CB4856	CT	IV	15899935	15901225	WBGene00013647	Y105C5B.5	Y105C5B.5
0.0470737851003413	AGIWUR10199
CB4856	CT	IV	15906582	15910091	WBGene00005726	srv-15	Y105C5B.6
-0.0697116290636348	AGIWUR43627
CB4856	CT	IV	15914997	15919234	WBGene00013648	Y105C5B.7	Y105C5B.7
0.589634592676289	AGIWUR5491
CB4856	CT	IV	15930581	15931813	WBGene00013650	Y105C5B.9	Y105C5B.9
0.340275361378586	AGIWUR9620
CB4856	CT	IV	15937112	15941491	WBGene00013651	Y105C5B.11	Y105C5B.11
-0.134882694436747	AGIWUR4903;AGIWUR15594;AGIWUR21513
CB4856	CT	IV	15942535	15944430	WBGene00013652	sams-2	Y105C5B.12
-0.0925490198064551	AGIWUR43488
CB4856	CT	IV	15946784	15947438	WBGene00004816	skr-10	Y105C5B.13
-0.0561657633401609	AGIWUR4957
CB4856	CT	IV	15949725	15950861	WBGene00013653	Y105C5B.14	Y105C5B.14
-0.0188031397090365	AGIWUR42421
CB4856	CT	IV	15951909	15954836	WBGene00013654	Y105C5B.15	Y105C5B.15
-0.563321587408295	AGIWUR569

CB4856 CT IV 15956571 15962712 WBGene00013655 lgc-19 Y105C5B.16
-0.391172556159637 AGIWUR44188
CB4856 CT IV 15963124 15964425 WBGene00013656 Y105C5B.17 Y105C5B.17
-0.154268973066952 AGIWUR25817
CB4856 CT IV 15964640 15965679 WBGene00013657 Y105C5B.18 Y105C5B.18
-0.071511302009315 AGIWUR24962;AGIWUR28919;AGIWUR31362;AGIWUR33863
CB4856 CT IV 15981575 15983970 WBGene00013658 Y105C5B.19 Y105C5B.19
-0.0450935115744144 AGIWUR13191
CB4856 CT IV 15993454 15994605 WBGene00013659 Y105C5B.20 Y105C5B.20
-0.243776089516452 AGIWUR44271
CB4856 CT IV 15997858 16026847 WBGene00002175 jac-1 Y105C5B.21
-0.179765624474919 AGIWUR19039;AGIWUR20231
CB4856 CT IV 16057283 16061358 WBGene00013661 Y105C5B.23 Y105C5B.23
0.161540220372562 AGIWUR25871
CB4856 CT IV 16063326 16064132 WBGene00005220 srg-63 Y105C5B.24
-0.0455192055286315 AGIWUR7527;AGIWUR22419;AGIWUR32294;AGIWUR38092
CB4856 CT IV 16090484 16091939 WBGene00044085 Y105C5B.30 Y105C5B.30
0.158735845949115 AGIWUR13023;AGIWUR28227
CB4856 CT IV 16132464 16134179 WBGene00013665 hlh-32 Y105C5B.29
-0.433046150194929 AGIWUR21888
CB4856 CT IV 16142544 16147155 WBGene00013662 Y105C5B.25 Y105C5B.25
-0.219093180853186 AGIWUR6295
CB4856 CT IV 16156908 16165267 WBGene00220260 Y105C5B.1420 Y105C5B.1420
0.0214253902161579 AGIWUR30234;AGIWUR35772
CB4856 CT IV 16167785 16170859 WBGene00001604 gln-3 Y105C5B.28
-0.144436300924728 AGIWUR3320;AGIWUR11501;AGIWUR13974;AGIWUR21293;AGIWUR27036;AGIWUR32466;AGIWUR38304
CB4856 CT IV 16197048 16201417 WBGene00012416 Y7A9A.1 Y7A9A.1 -0.081902067921277
AGIWUR22558
CB4856 CT IV 16202374 16205321 WBGene00010411 H25K10.1 H25K10.1
-0.78146688529645 AGIWUR28701;AGIWUR41997
CB4856 CT IV 16210723 16211013 WBGene00010412 H25K10.2 H25K10.2
-0.416237311693724 AGIWUR41059
CB4856 CT IV 16219652 16221659 WBGene00005722 srv-11 H25K10.3
-0.311144779505087 AGIWUR41993
CB4856 CT IV 16226047 16227122 WBGene00010413 H25K10.4 H25K10.4
0.194404574949683 AGIWUR19112
CB4856 CT IV 16230452 16233128 WBGene00010414 hpa-1 H25K10.5
0.06680658042864 AGIWUR1004
CB4856 CT IV 16233148 16236555 WBGene00010415 irld-39 H25K10.6

-0.0418155827756488	AGIWUR12877
CB4856	CT	IV	16236732	16238655	WBGene00005808	srw-61	H25K10.7
0.122512502541321	AGIWUR20698
CB4856	CT	IV	16247431	16251790	WBGene00009535	F38C2.1	F38C2.1	0.511087096724014
AGIWUR1967
CB4856	CT	IV	16254764	16256274	WBGene00001961	hlh-17	F38C2.2	0.155054878061728
AGIWUR39384
CB4856	CT	IV	16257668	16259417	WBGene00009540	hlh-31	F38C2.8	-0.0616351349577271
AGIWUR14645;AGIWUR37847
CB4856	CT	IV	16271263	16273187	WBGene00009536	F38C2.4	F38C2.4	-0.120780313932169
AGIWUR16881
CB4856	CT	IV	16276647	16277391	WBGene00009539	F38C2.7	F38C2.7	-0.124456372209438
AGIWUR36786
CB4856	CT	IV	16278202	16279018	WBGene00009537	ccch-2	F38C2.5	-0.166673327002397
AGIWUR2753
CB4856	CT	IV	16279634	16280714	WBGene00009538	clec-191		F38C2.6
-0.204806992975286	AGIWUR43506
CB4856	CT	IV	16281520	16283423	WBGene00045026	Y7A9C.10		Y7A9C.10
-0.0618849827661609	AGIWUR25908
CB4856	CT	IV	16286655	16287825	WBGene00023517	srz-75	Y7A9C.9	0.104784198935249
AGIWUR5516;AGIWUR11469
CB4856	CT	IV	16288113	16289310	WBGene00023518	srz-76	Y7A9C.8	-0.0205540532384738
AGIWUR29943
CB4856	CT	IV	16289558	16290835	WBGene00012419	srz-73	Y7A9C.3	0.101165151839639
AGIWUR4987;AGIWUR29474;AGIWUR31684
CB4856	CT	IV	16291988	16293070	WBGene00023510	srz-40	Y7A9C.5	0.0604406563020925
AGIWUR3818
CB4856	CT	IV	16293199	16293482	WBGene00045105	Y7A9C.11		Y7A9C.11
0.691521148628283	AGIWUR1833
CB4856	CT	IV	16293619	16293923	WBGene00045106	Y7A9C.12		Y7A9C.12
-0.140545849831011	AGIWUR18794;AGIWUR27829;AGIWUR41685
CB4856	CT	IV	16294154	16298479	WBGene00012418	Y7A9C.1	Y7A9C.1	0.0351912257846383
AGIWUR10327;AGIWUR12900;AGIWUR29256;AGIWUR40799;AGIWUR44868
CB4856	CT	IV	16298813	16299012	WBGene00045107	Y7A9C.13		Y7A9C.13
0.0474067305568893	AGIWUR10892;AGIWUR11887;AGIWUR12721;AGIWUR22530;AGIWUR23429
CB4856	CT	IV	16299151	16300272	WBGene00023514	srz-72	Y7A9C.7	0.0856633394757651
AGIWUR953;AGIWUR34466
CB4856	CT	IV	16300588	16301746	WBGene00023511	srz-41	Y7A9C.6	-0.150459091042001
AGIWUR22366;AGIWUR29053

CB4856	CT	IV	16303614	16306235	WBGene00014862	Y7A9C.2	Y7A9C.2	0.145933724781047	AGIWUR2421;AGIWUR8717
CB4856	CT	IV	16306312	16306839	WBGene00023509	srz-39	Y7A9C.4	0.0543321008664073	AGIWUR19605;AGIWUR25345;AGIWUR35351;AGIWUR39465;AGIWUR42243
CB4856	CT	IV	16308225	16309829	WBGene00004287	rac-2	K03D3.10	-0.256540641372514	AGIWUR1338
CB4856	CT	IV	16311079	16319703	WBGene00044670	rac-3	K03D3.13	-0.0778294723545749	AGIWUR12250;AGIWUR12813;AGIWUR35498;AGIWUR44020
CB4856	CT	IV	16320139	16320506	WBGene00010531	K03D3.5	K03D3.5	-0.516927014337446	AGIWUR6101
CB4856	CT	IV	16321166	16323195	WBGene00010530	srz-61	K03D3.4	-0.117153131590075	AGIWUR38770;AGIWUR43421
CB4856	CT	IV	16323673	16324973	WBGene00043052	srz-104	K03D3.11	-0.033724730850782	AGIWUR19731
CB4856	CT	IV	16325172	16326601	WBGene00044587	srz-105	K03D3.12	0.582782199602722	AGIWUR40134
CB4856	CT	IV	16326718	16327965	WBGene00010529	srz-21	K03D3.3	0.143637661002193	AGIWUR40769
CB4856	CT	IV	16328135	16328614	WBGene00010528	K03D3.2	K03D3.2	-0.20204617067182	AGIWUR13565
CB4856	CT	IV	16329459	16331059	WBGene00010527	srz-74	K03D3.1	0.154376957858573	AGIWUR13697
CB4856	CT	IV	16331365	16332611	WBGene00045100	K03D3.14	K03D3.14	-0.141440882933233	AGIWUR7604
CB4856	CT	IV	16332885	16334461	WBGene00023513	srz-71	C35D6.10	-0.0031547592442811	AGIWUR20690
CB4856	CT	IV	16335337	16336503	WBGene00023508	srz-38	C35D6.9	0.108059485958712	AGIWUR21322;AGIWUR27500
CB4856	CT	IV	16337021	16338502	WBGene00045101	C35D6.13	C35D6.13	0.145719737663689	AGIWUR22054;AGIWUR25308
CB4856	CT	IV	16338562	16339098	WBGene00045040	C35D6.12	C35D6.12	-0.0666306290612502	AGIWUR33321
CB4856	CT	IV	16341625	16345442	WBGene00005434	srh-224	C35D6.8	-0.0727016189908922	AGIWUR7329;AGIWUR13640
CB4856	CT	IV	16347168	16347430	WBGene00007960	C35D6.3	C35D6.3	0.168944187087943	AGIWUR40446
CB4856	CT	IV	16348409	16348751	WBGene00045028	C35D6.11	C35D6.11	-0.042876538761691	AGIWUR2973;AGIWUR5282;AGIWUR16135;AGIWUR20681;AGIWUR36159
CB4856	CT	IV	16350990	16351601	WBGene00007961	C35D6.4	C35D6.4	0.138202233983992	

AGIWUR5086
CB4856 CT IV 16366996 16368943 WBGene00007962 C35D6.5 C35D6.5 -0.20883913097618
AGIWUR5610
CB4856 CT IV 16381378 16382235 WBGene00012420 Y7A9D.1 Y7A9D.1 0.687820648021147
AGIWUR32721;AGIWUR33229;AGIWUR37237;AGIWUR41776;AGIWUR43445;AGIWUR45148
CB4856 CT IV 16398839 16399875 WBGene00013418 Y65A5A.1 Y65A5A.1
0.474000124007395 AGIWUR30411
CB4856 CT IV 16412020 16416808 WBGene00013419 Y65A5A.2 Y65A5A.2
-0.30383352269895 AGIWUR26456
CB4856 CT IV 16424637 16429895 WBGene00012423 Y10G11A.1 Y10G11A.1
-0.108546656807342 AGIWUR5130;AGIWUR7085
CB4856 CT IV 16432231 16437953 WBGene00012424 dlc-3 Y10G11A.2
0.285354705846508 AGIWUR19845
CB4856 CT IV 16443263 16446683 WBGene00012425 dlc-4 Y10G11A.3
0.171026340947001 AGIWUR23539
CB4856 CT IV 16454270 16454579 WBGene00045185 VY10G11R.4 VY10G11R.4
-0.0276376912169747 AGIWUR10617;AGIWUR13766;AGIWUR19304;AGIWUR37200;AGIWUR40802
CB4856 CT IV 16468723 16469064 WBGene00045097 VY10G11R.2 VY10G11R.2
0.0732253049217508 AGIWUR6952;AGIWUR14946;AGIWUR15445;AGIWUR29398;AGIWUR36369
CB4856 CT IV 16469580 16470881 WBGene00012159 VY10G11R.1 VY10G11R.1
-0.0298695998479026 AGIWUR4731
CB4856 CT IV 16474044 16477192 WBGene00013098 Y51H4A.1 Y51H4A.1
0.922636667754169 AGIWUR13939
CB4856 CT IV 16482784 16489021 WBGene00004357 rho-1 Y51H4A.3
-0.174097034632599 AGIWUR2147;AGIWUR39259
CB4856 CT IV 16493428 16494510 WBGene00013099 Y51H4A.2 Y51H4A.2
-0.870586808556121 AGIWUR5454
CB4856 CT IV 16503249 16505682 WBGene00013100 zip-7 Y51H4A.4
-0.0895819203038242 AGIWUR8385;AGIWUR41524
CB4856 CT IV 16522529 16526132 WBGene00013101 Y51H4A.5 Y51H4A.5
0.923571793818348 AGIWUR31950
CB4856 CT IV 16533661 16533962 WBGene00013102 Y51H4A.6 Y51H4A.6
-0.0906610743263245 AGIWUR42976
CB4856 CT IV 16535730 16542894 WBGene00013103 Y51H4A.7 Y51H4A.7
-0.197236299505013 AGIWUR18320
CB4856 CT IV 16551492 16554348 WBGene00000709 col-136 Y51H4A.28
-0.168323264188216 AGIWUR28306;AGIWUR33242
CB4856 CT IV 16575102 16578549 WBGene00013104 Y51H4A.8 Y51H4A.8
0.412279414888923 AGIWUR43151

CB4856	CT	IV	16601480	16602776	WBGene00000710	col-137	Y51H4A.9
-0.377558414868199	AGIWUR17787
CB4856	CT	IV	16627976	16628441	WBGene00013120	fipr-28	Y51H4A.26
-0.0821924110700749	AGIWUR12842
CB4856	CT	IV	16656613	16657359	WBGene00013109	Y51H4A.15	Y51H4A.15
-0.0518011203978119	AGIWUR26764
CB4856	CT	IV	16664320	16671467	WBGene00013111	sta-1	Y51H4A.17
0.074734105607988
AGIWUR1252;AGIWUR2004;AGIWUR3505;AGIWUR5230;AGIWUR21787;AGIWUR22093;AGIWUR26957;AGIWUR34933
CB4856	CT	IV	16720715	16722736	WBGene00013116	Y51H4A.22	Y51H4A.22
0.0128818673685238	AGIWUR3435
CB4856	CT	IV	16722916	16723892	WBGene00013117	Y51H4A.23	Y51H4A.23
0.32161951246348	AGIWUR39803
CB4856	CT	IV	16736329	16743178	WBGene00013119	Y51H4A.25	Y51H4A.25
0.599312090678024	AGIWUR25777;AGIWUR38342
CB4856	CT	IV	16758420	16758769	WBGene00045321	Y43D4A.10	Y43D4A.10
-0.478385686519992	AGIWUR15417;AGIWUR20545;AGIWUR31612;AGIWUR34955
CB4856	CT	IV	16775827	16785172	WBGene00012791	Y43D4A.5	Y43D4A.5
-0.245139713979405	AGIWUR9735
CB4856	CT	IV	16787327	16788510	WBGene00012792	Y43D4A.6	Y43D4A.6
-0.0660719031579973	AGIWUR21279;AGIWUR36471;AGIWUR42890
CB4856	CT	IV	16826324	16828020	WBGene00013776	Y116A8A.6	Y116A8A.6
0.216871281052212	AGIWUR7119
CB4856	CT	IV	16831666	16832984	WBGene00013778	clec-194	Y116A8A.8
0.0946476867345858	AGIWUR35391
CB4856	CT	IV	16847829	16854943	WBGene00003130	map-2	Y116A8A.9
0.19735873242683	AGIWUR9535
CB4856	CT	IV	16855044	16863443	WBGene00020284	mel-46	T06A10.1
-0.183133677272323	AGIWUR38585
CB4856	CT	IV	16862396	16865479	WBGene00020287	lsy-13	T06A10.4
-0.330864712811636	AGIWUR21297
CB4856	CT	IV	16865838	16866504	WBGene00020286	T06A10.3	T06A10.3
0.087950806068676	AGIWUR18852
CB4856	CT	IV	16920341	16924071	WBGene00013785	nep-23	Y116A8C.4
0.109557085398365	AGIWUR7228
CB4856	CT	IV	16928334	16929067	WBGene00014976	Y116A8C.7	Y116A8C.7
-0.190194615138876	AGIWUR6180;AGIWUR11548;AGIWUR21776;AGIWUR22996;AGIWUR37782
CB4856	CT	IV	16930837	16931177	WBGene00045128	Y116A8C.49	Y116A8C.49
-0.0116890393724187	AGIWUR4096

CB4856 CT IV 16931990 16933113 WBGene00013788 Y116A8C.8 Y116A8C.8
-0.114840540987809 AGIWUR929;AGIWUR9903
CB4856 CT IV 16934266 16939111 WBGene00013790 Y116A8C.10 Y116A8C.10
0.0432350574106382 AGIWUR1366;AGIWUR8901;AGIWUR28542;AGIWUR34521;AGIWUR40850;AGIWUR42572;AGIWUR44503
CB4856 CT IV 16939804 16946910 WBGene00013789 Y116A8C.9 Y116A8C.9
-0.244918801710723 AGIWUR37459
CB4856 CT IV 16947120 16949734 WBGene00000184 arf-6 Y116A8C.12
0.0421898145008122 AGIWUR22709
CB4856 CT IV 16950065 16962868 WBGene00013792 Y116A8C.13 Y116A8C.13
0.0856320816352856 AGIWUR12465
CB4856 CT IV 16965284 16970490 WBGene00004402 rom-3 Y116A8C.14
-0.245034157632396 AGIWUR10932
CB4856 CT IV 16988828 16989771 WBGene00013791 Y116A8C.11 Y116A8C.11
0.0102533125836453 AGIWUR673;AGIWUR30500;AGIWUR33757;AGIWUR38152;AGIWUR41182;AGIWUR42358
CB4856 CT IV 16995777 17014953 WBGene00004403 rom-4 Y116A8C.16
-0.221848942595408 AGIWUR5163;AGIWUR22741;AGIWUR30005
CB4856 CT IV 17022990 17023922 WBGene00045315 Y116A8C.52 Y116A8C.52
-0.367707809377894 AGIWUR38540
CB4856 CT IV 17024318 17025132 WBGene00013794 dct-13 Y116A8C.17
-0.00270258476593099 AGIWUR4796
CB4856 CT IV 17028931 17030594 WBGene00013795 nhr-229 Y116A8C.18
-0.0461153860506065 AGIWUR8246;AGIWUR27868
CB4856 CT IV 17038923 17039667 WBGene00013796 Y116A8C.19 Y116A8C.19
-0.191193009976576 AGIWUR10845
CB4856 CT IV 17040913 17041523 WBGene00013797 Y116A8C.20 Y116A8C.20
-0.153093547842852 AGIWUR14834
CB4856 CT IV 17046470 17048357 WBGene00013799 athp-3 Y116A8C.22
-0.497032021428563 AGIWUR26399
CB4856 CT IV 17053594 17055513 WBGene00013800 Y116A8C.23 Y116A8C.23
0.149421475829943 AGIWUR13331
CB4856 CT IV 17056746 17057831 WBGene00045280 Y116A8C.51 Y116A8C.51
0.0378069376490595 AGIWUR4143;AGIWUR15519;AGIWUR36264
CB4856 CT IV 17068795 17070903 WBGene00013801 Y116A8C.24 Y116A8C.24
0.134188474577376 AGIWUR12026
CB4856 CT IV 17079606 17087438 WBGene00013803 snx-13 Y116A8C.26
-0.263170791007052 AGIWUR1253;AGIWUR16582;AGIWUR22196;AGIWUR30792;AGIWUR39567;AGIWUR41148
CB4856 CT IV 17088058 17088834 WBGene00004914 snr-1 Y116A8C.42
-0.221600507251431
AGIWUR2910;AGIWUR2945;AGIWUR15577;AGIWUR21296;AGIWUR22559;AGIWUR27398;AGIWUR34517;AGIWUR40521

CB4856	CT	IV	17088851	17090646	WBGene00013814	Y116A8C.43	Y116A8C.43
0.131451413734247	AGIWUR9877
CB4856	CT	IV	17090630	17096286	WBGene00013804	Y116A8C.27	Y116A8C.27
-0.0616294650297959	AGIWUR14712
CB4856	CT	IV	17096386	17100246	WBGene00013805	bca-2	Y116A8C.28
0.159815442626514	AGIWUR8202;AGIWUR34891
CB4856	CT	IV	17100225	17103715	WBGene00013807	Y116A8C.30	Y116A8C.30
-0.0877514987413726	AGIWUR15694
CB4856	CT	IV	17104179	17110982	WBGene00013808	sfa-1	Y116A8C.32
-0.42093234675721	AGIWUR19437
CB4856	CT	IV	17111082	17116666	WBGene00000889	cyn-13	Y116A8C.34
-0.143270702116578	AGIWUR20202
CB4856	CT	IV	17116763	17118760	WBGene00006698	uaf-2	Y116A8C.35
-0.243370875187146	AGIWUR22742
CB4856	CT	IV	17119684	17126266	WBGene00006405	itsn-1	Y116A8C.36
-0.0394584384598708	AGIWUR25288;AGIWUR26969;AGIWUR31401;AGIWUR32319
CB4856	CT	IV	17130066	17131075	WBGene00013810	Y116A8C.37	Y116A8C.37
-0.205943952822842	AGIWUR9715
CB4856	CT	IV	17137348	17142989	WBGene00045043	Y116A8C.48	Y116A8C.48
-0.0117485849635393	AGIWUR3839;AGIWUR13359;AGIWUR17919;AGIWUR22945
CB4856	CT	IV	17144355	17150971	WBGene00013812	Y116A8C.40	Y116A8C.40
0.19340946393352	AGIWUR9796;AGIWUR10092
CB4856	CT	IV	17162024	17162634	WBGene00004819	skr-13	C52D10.8
0.437388540780494	AGIWUR30480
CB4856	CT	IV	17163554	17164226	WBGene00004815	skr-9	C52D10.7
0.062876237129629	AGIWUR42025
CB4856	CT	IV	17167253	17168051	WBGene00004818	skr-12	C52D10.6
0.551731497602486	AGIWUR11514
CB4856	CT	IV	17176477	17189805	WBGene00016877	C52D10.3	C52D10.3
-0.216291038490682	AGIWUR28085;AGIWUR36872;AGIWUR39260
CB4856	CT	IV	17190177	17191891	WBGene00001460	flp-17	C52D10.11
-0.0571908013315579	AGIWUR23495
CB4856	CT	IV	17197905	17206845	WBGene00016879	cnnm-1	C52D10.12
0.0683899596674848	AGIWUR25040
CB4856	CT	IV	17216333	17217368	WBGene00013779	Y116A8B.1	Y116A8B.1
-0.197958778541262	AGIWUR6796
CB4856	CT	IV	17223774	17225019	WBGene00013781	Y116A8B.4	Y116A8B.4
-0.19754198872458	AGIWUR5030;AGIWUR6033;AGIWUR28449;AGIWUR32021;AGIWUR35102;AGIWUR37333
CB4856	CT	IV	17226014	17231056	WBGene00013782	npr-32	Y116A8B.5

-0.109094113817313	AGIWUR8559
CB4856	CT	IV	17239941	17243212	WBGene00013995	mboa-5	ZK550.1	-0.0931204548495046
AGIWUR11027;AGIWUR21565;AGIWUR26813
CB4856	CT	IV	17245584	17249750	WBGene00013996	ZK550.2	ZK550.2	0.127466143188785
AGIWUR37560
CB4856	CT	IV	17250131	17254964	WBGene00013997	ZK550.3	ZK550.3	-0.0338203223254236
AGIWUR13238
CB4856	CT	IV	17255301	17257562	WBGene00013998	ZK550.4	ZK550.4	-0.0672647208479429
AGIWUR2456;AGIWUR42569
CB4856	CT	IV	17257504	17259410	WBGene00013999	ZK550.5	ZK550.5	0.0764907012311955
AGIWUR2165;AGIWUR11834;AGIWUR37733
CB4856	CT	IV	17260307	17262485	WBGene00014000	ZK550.6	ZK550.6	0.702027010539315
AGIWUR2507;AGIWUR7827
CB4856	CT	IV	17264297	17264814	WBGene00044244	F26D10.13	F26D10.13
-0.569067278418844	AGIWUR8280;AGIWUR10704;AGIWUR24795;AGIWUR25868
CB4856	CT	IV	17265579	17266926	WBGene00006229	str-187	F26D10.8
-0.26661917813867	AGIWUR1710
CB4856	CT	IV	17269342	17272183	WBGene00000224	atgp-1	F26D10.9
0.184422302066132	AGIWUR31914
CB4856	CT	IV	17272231	17273652	WBGene00001606	gln-5	F26D10.10
0.0950359326100454	AGIWUR34181
CB4856	CT	IV	17275581	17276864	WBGene00009155	lhfp-4	F26D10.11
0.810394385504479	AGIWUR42728
CB4856	CT	IV	17276956	17277748	WBGene00009156	clec-196	F26D10.12
0.658769333781706	AGIWUR7487
CB4856	CT	IV	17278959	17281330	WBGene00002005	hsp-1	F26D10.3
-0.159217713062279
AGIWUR6894;AGIWUR15751;AGIWUR17478;AGIWUR18500;AGIWUR21587;AGIWUR23964;AGIWUR26492;AGIWUR28010;AGIWUR33671;
AGIWUR41303;AGIWUR41534
CB4856	CT	IV	17281456	17284199	WBGene00012128	nra-1	T28F3.1	-0.219288983313682
AGIWUR2747
CB4856	CT	IV	17285733	17287454	WBGene00044067	zipt-7.1	T28F3.3
-0.084731645524176	AGIWUR20648
CB4856	CT	IV	17288171	17291733	WBGene00012130	slc-17.4	T28F3.4
-0.503281679089901	AGIWUR18244;AGIWUR18531
CB4856	CT	IV	17294793	17300629	WBGene00012131	T28F3.5	T28F3.5	0.0469853119416381
AGIWUR41791
CB4856	CT	IV	17300809	17302033	WBGene00012132	ifta-2	T28F3.6	0.247660358813837
AGIWUR19088

CB4856	CT	IV	17302242	17304370	WBGene00012133	T28F3.7	T28F3.7	-0.13278365348339	AGIWUR43806
CB4856	CT	IV	17308525	17311695	WBGene00012135	T28F3.9	T28F3.9	0.284616239714909	AGIWUR31439
CB4856	CT	IV	17312141	17315462	WBGene00004908	snf-9	C49C3.1	-0.0777070445641783	AGIWUR27569
CB4856	CT	IV	17316710	17318246	WBGene00008192	C49C3.2	C49C3.2	0.106581114114688	AGIWUR38738
CB4856	CT	IV	17319353	17320027	WBGene00008193	C49C3.3	C49C3.3	-0.225384702329817	AGIWUR29862;AGIWUR35793
CB4856	CT	IV	17320795	17328662	WBGene00008194	C49C3.4	C49C3.4	-0.215244860717279	AGIWUR38518
CB4856	CT	IV	17329192	17330737	WBGene00008195	ceh-88	C49C3.5	0.186344928364572	AGIWUR8331;AGIWUR13535;AGIWUR24087;AGIWUR35702
CB4856	CT	IV	17331527	17332447	WBGene00008196	C49C3.6	C49C3.6	0.133769006674971	AGIWUR10924
CB4856	CT	IV	17332573	17333645	WBGene00008197	C49C3.7	C49C3.7	-0.0350662063512885	AGIWUR42814
CB4856	CT	IV	17334193	17335094	WBGene00008204	C49C3.15	C49C3.15	0.166804542640804	AGIWUR31876;AGIWUR40425
CB4856	CT	IV	17335203	17336681	WBGene00008198	C49C3.8	C49C3.8	0.190350564477878	AGIWUR42771
CB4856	CT	IV	17336800	17338984	WBGene00008200	C49C3.10	C49C3.10	-0.418056982221028	AGIWUR30175
CB4856	CT	IV	17339640	17342314	WBGene00008199	C49C3.9	C49C3.9	-0.455489992233323	AGIWUR35239
CB4856	CT	IV	17343782	17344805	WBGene00008201	C49C3.11	C49C3.11	-0.320994913273333	AGIWUR37871
CB4856	CT	IV	17345568	17346695	WBGene00008202	clec-197	C49C3.12	-0.131760799300757	AGIWUR31392
CB4856	CT	IV	17347168	17351076	WBGene00008203	clec-198	C49C3.13	0.11098647236132	AGIWUR33042
CB4856	CT	IV	17351886	17357895	WBGene00007820	clec-199	C30H6.1	0.00947311682697727	AGIWUR19025;AGIWUR33156
CB4856	CT	IV	17358139	17360635	WBGene00007821	clec-201	C30H6.3	0.213172224428215	AGIWUR43767
CB4856	CT	IV	17361443	17364583	WBGene00007822	clec-202	C30H6.4	-0.128129621616308	AGIWUR8247
CB4856	CT	IV	17364791	17366491	WBGene00006487	zipt-22	C30H6.2	-0.103776505061902	

AGIWUR20952
CB4856 CT IV 17368592 17370618 WBGene00007827 nlp-54 C30H6.10
0.276578087123615 AGIWUR11946;AGIWUR16831;AGIWUR19598;AGIWUR29665
CB4856 CT IV 17371769 17375638 WBGene00007823 C30H6.5 C30H6.5 -0.64767077685565
AGIWUR18277;AGIWUR21420;AGIWUR21814
CB4856 CT IV 17376427 17379317 WBGene00001811 haf-1 C30H6.6
-0.00533199287509234 AGIWUR2043;AGIWUR5177;AGIWUR21139
CB4856 CT IV 17379247 17380188 WBGene00007826 C30H6.9 C30H6.9 -0.0890939236144419
AGIWUR21945
CB4856 CT IV 17380626 17382225 WBGene00007824 dlat-2 C30H6.7 0.354936770807578
AGIWUR33786
CB4856 CT IV 17382298 17383087 WBGene00007825 C30H6.8 C30H6.8 -0.0545206532413616
AGIWUR8005;AGIWUR25239;AGIWUR26763;AGIWUR40999
CB4856 CT IV 17383370 17386783 WBGene00044238 C30H6.12 C30H6.12
0.208656695540355 AGIWUR303;AGIWUR4607;AGIWUR11706;AGIWUR32487;AGIWUR34320
CB4856 CT IV 17386763 17387492 WBGene00011374 T02D1.8 T02D1.8 -0.380554429210221
AGIWUR20749;AGIWUR36007;AGIWUR36236
CB4856 CT IV 17393974 17395333 WBGene00005677 sru-14 T02D1.2 0.0623903823023353
AGIWUR1863;AGIWUR12530
CB4856 CT IV 17396210 17397491 WBGene00005678 sru-15 T02D1.3 0.287822803881268
AGIWUR43037
CB4856 CT IV 17399776 17402336 WBGene00011371 T02D1.4 T02D1.4 0.289025046954583
AGIWUR37043
CB4856 CT IV 17402463 17405630 WBGene00004061 pmp-4 T02D1.5 -0.136665055947619
AGIWUR9352;AGIWUR13782;AGIWUR30490;AGIWUR37445
CB4856 CT IV 17408231 17417794 WBGene00011372 npr-26 T02D1.6 0.0459347255629972
AGIWUR6871
CB4856 CT IV 17427717 17429602 WBGene00007357 C06A12.3 C06A12.3
0.0851827198793558 AGIWUR31400
CB4856 CT IV 17429637 17431968 WBGene00007358 lact-6 C06A12.5
-0.0621710008653809 AGIWUR4456;AGIWUR12935
CB4856 CT IV 17432977 17436652 WBGene00001550 gcy-27 C06A12.4
0.332332652185921 AGIWUR31564
CB4856 CT IV 17437338 17438155 WBGene00007359 C06A12.8 C06A12.8
0.223877826494328 AGIWUR32750
CB4856 CT IV 17440642 17442125 WBGene00045013 F08A7.1 F08A7.1 0.72483052921023
AGIWUR6277;AGIWUR17924;AGIWUR26032;AGIWUR39716;AGIWUR45208
CB4856 CT IV 17442827 17443738 WBGene00023501 F11E6.11 F11E6.11
2.13424510931847 AGIWUR41389

CB4856 CT IV 17444125 17450543 WBGene00008711 F11E6.8 F11E6.8 0.117716746854967
AGIWUR924
CB4856 CT IV 17457834 17461272 WBGene00008710 F11E6.7 F11E6.7 0.0948541650737376
AGIWUR37764
CB4856 CT IV 17461909 17463158 WBGene00008713 F11E6.10 F11E6.10
-0.0695728870016339 AGIWUR15528;AGIWUR19371;AGIWUR28688
CB4856 CT IV 17464451 17465986 WBGene00008709 F11E6.6 F11E6.6 0.183934278490902
AGIWUR38814
CB4856 CT IV 17466060 17467408 WBGene00001240 elo-2 F11E6.5 0.233939422015604
AGIWUR15442
CB4856 CT IV 17469347 17470387 WBGene00008708 F11E6.4 F11E6.4 -0.0378041641944336
AGIWUR25207
CB4856 CT IV 17470510 17472025 WBGene00008707 F11E6.3 F11E6.3 0.0193478565374292
AGIWUR753;AGIWUR11490
CB4856 CT IV 17473334 17479397 WBGene00008706 gba-3 F11E6.1 0.0494267743779089
AGIWUR26597;AGIWUR26714
CB4856 CT IV 17480396 17483332 WBGene00007067 4R79.2 4R79.2 0.138004342399534
AGIWUR14144
CB4856 CT IV 17486871 17490779 WBGene00003525 nas-6 4R79.1 0.150953694420762
AGIWUR30655
CB4856 CT MtDNA 113 549 WBGene00010957 nduo-6 MTCE.3 -0.320041493899401
AGIWUR24989;AGIWUR35041
CB4856 CT MtDNA 549 783 WBGene00010958 ndfl-4 MTCE.4 0.384039815012515
AGIWUR23526;AGIWUR34575
CB4856 CT MtDNA 898 1593 WBGene00014454 MTCE.7 MTCE.7 0.0668317567902701
AGIWUR7913;AGIWUR13747;AGIWUR22917;AGIWUR36868;AGIWUR41384
CB4856 CT MtDNA 1763 2635 WBGene00010959 nduo-1 MTCE.11 0.0502096988305292
AGIWUR28777;AGIWUR31173
CB4856 CT MtDNA 2634 3235 WBGene00010960 atp-6 MTCE.12 -0.0336569563901383
AGIWUR4315;AGIWUR23319
CB4856 CT MtDNA 3389 4269 WBGene00010961 nduo-2 MTCE.16 -0.152501572099116
AGIWUR13582;AGIWUR27453;AGIWUR34174
CB4856 CT MtDNA 4504 5621 WBGene00000829 ctb-1 MTCE.21 0.0962321809866733
AGIWUR13075;AGIWUR32293
CB4856 CT MtDNA 5678 6449 WBGene00010962 ctc-3 MTCE.23 0.109322290237593
AGIWUR16275;AGIWUR21151;AGIWUR33004
CB4856 CT MtDNA 6506 7808 WBGene00010963 nduo-4 MTCE.25 -0.0545372073554979
AGIWUR2185;AGIWUR25147;AGIWUR28528;AGIWUR32111;AGIWUR33776;AGIWUR38430;AGIWUR43128
CB4856 CT MtDNA 7827 9428 WBGene00010964 ctc-1 MTCE.26 0.0699131688589212

									AGIWUR14543;AGIWUR27788;AGIWUR34673;AGIWUR38561;AGIWUR40016
CB4856	CT	MtDNA	9649	10348	WBGene00010965	ctc-2	MTCE.31	0.0358483627416568	AGIWUR3817;AGIWUR15036
CB4856	CT	MtDNA	10403	11354	WBGene00014472	MTCE.33	MTCE.33	0.513349013196071	AGIWUR35310;AGIWUR39867
CB4856	CT	MtDNA	11356	11691	WBGene00010966	nduo-3	MTCE.34	0.0416723902981215	AGIWUR35958;AGIWUR36046
CB4856	CT	MtDNA	11691	13272	WBGene00010967	nduo-5	MTCE.35	-0.191409115082463	AGIWUR5384;AGIWUR5608;AGIWUR6469;AGIWUR27940;AGIWUR29535;AGIWUR34029
CB4856	CT	V	1663	6632	WBGene00015153	B0348.5	B0348.5	0.128787429675904	AGIWUR43464
CB4856	CT	V	6588	7822	WBGene00002061	ife-3	B0348.6	-0.172768851571858	AGIWUR1089;AGIWUR2583;AGIWUR3915;AGIWUR15574;AGIWUR31428;AGIWUR34420;AGIWUR35684;AGIWUR40770
CB4856	CT	V	20825	43697	WBGene00001177	egl-8	B0348.4	0.137859179070601	AGIWUR27169;AGIWUR28430;AGIWUR43926
CB4856	CT	V	45310	46916	WBGene00015152	B0348.2	B0348.2	0.518932202732218	AGIWUR29175
CB4856	CT	V	48922	49619	WBGene00015151	B0348.1	B0348.1	0.135096421723458	AGIWUR7437;AGIWUR12945;AGIWUR15241;AGIWUR19933;AGIWUR38670;AGIWUR42802
CB4856	CT	V	49956	56046	WBGene00021413	srt-52	Y38C9B.2	0.0891362428044502	AGIWUR8074
CB4856	CT	V	59239	63509	WBGene00021412	cyp-29A3	Y38C9B.1	-0.474433006147613	AGIWUR1490
CB4856	CT	V	63726	64503	WBGene00021414	Y38C9B.3	Y38C9B.3	0.150063302924089	AGIWUR15767
CB4856	CT	V	64564	83831	WBGene00018976	daam-1	F56E10.2	0.170948951251718	AGIWUR12745;AGIWUR21333
CB4856	CT	V	103394	104064	WBGene00004496	rps-27	F56E10.4	-0.157436157645474	AGIWUR15986
CB4856	CT	V	104826	118188	WBGene00018975	F56E10.1	F56E10.1	-0.177749091137073	AGIWUR30224;AGIWUR37479
CB4856	CT	V	118043	122966	WBGene00000480	cgp-1	Y38C9A.2	-0.0177257711719392	AGIWUR7387;AGIWUR17842;AGIWUR23609
CB4856	CT	V	124010	126758	WBGene00020999	W03F9.1	W03F9.1	-0.0828306056935837	AGIWUR40968
CB4856	CT	V	127000	128034	WBGene00021000	W03F9.2	W03F9.2	-0.0400843680527299	AGIWUR413;AGIWUR3259;AGIWUR9935;AGIWUR38374
CB4856	CT	V	128237	131754	WBGene00021004	sftb-2	W03F9.10	0.0409029992282406	AGIWUR1576;AGIWUR40301
CB4856	CT	V	132450	134993	WBGene00021001	W03F9.3	W03F9.3	0.145207041779164	AGIWUR9969
CB4856	CT	V	137177	141370	WBGene00044512	W03F9.11	W03F9.11	0.209048604847419	AGIWUR452

CB4856	CT	V	142449	142799	WBGene00023356	W03F9.8	W03F9.8	0.0811904119706437	AGIWUR15929;AGIWUR34727
CB4856	CT	V	143047	144617	WBGene00005302	srh-81	W03F9.7	0.0583236908589146	AGIWUR8210;AGIWUR27656;AGIWUR28230
CB4856	CT	V	145194	146931	WBGene00005303	srh-82	W03F9.6	0.246613100542577	AGIWUR27502
CB4856	CT	V	147538	152104	WBGene00021002	W03F9.4	W03F9.4	0.78459507854204	AGIWUR22684
CB4856	CT	V	156825	166866	WBGene00006648	ttb-1	W03F9.5	-0.0202562704613491	AGIWUR17082
CB4856	CT	V	177421	184966	WBGene00000838	cul-3	Y108G3AL.1	0.022069311037157	AGIWUR7760;AGIWUR19520;AGIWUR25925;AGIWUR29443;AGIWUR44731
CB4856	CT	V	186169	200777	WBGene00044689	arid-1	Y108G3AL.7	-0.0921257509979916	AGIWUR8634;AGIWUR41275
CB4856	CT	V	201600	204279	WBGene00077643	trpp-1	DC2.8	-0.246749703970618	AGIWUR13239
CB4856	CT	V	204382	209758	WBGene00017080	lec-12	DC2.3	0.0117634843827439	AGIWUR11135
CB4856	CT	V	210148	212787	WBGene00017082	DC2.5	DC2.5	-0.592674589307963	AGIWUR26784
CB4856	CT	V	217154	219473	WBGene00005299	srh-78	DC2.2	-0.246162564276292	AGIWUR34036
CB4856	CT	V	219932	222776	WBGene00005301	srh-80	DC2.1	-0.00863129454603673	AGIWUR15830
CB4856	CT	V	222995	226947	WBGene00005298	srh-77	DC2.6	0.096278744751281	AGIWUR24387
CB4856	CT	V	228858	234672	WBGene00017083	kin-33	DC2.7	0.120895723078479	AGIWUR2580;AGIWUR6254;AGIWUR15785;AGIWUR20620
CB4856	CT	V	236750	238192	WBGene00019814	R02C2.1	R02C2.1	0.099948658652055	AGIWUR19767
CB4856	CT	V	238963	240362	WBGene00019817	R02C2.6	R02C2.6	0.0228477460795746	AGIWUR5843
CB4856	CT	V	241326	242618	WBGene00019818	R02C2.7	R02C2.7	0.780397629146622	AGIWUR28807
CB4856	CT	V	244469	245846	WBGene00019815	kin-34	R02C2.2	0.0732510286720238	AGIWUR21942
CB4856	CT	V	246618	251756	WBGene00006419	oac-39	R02C2.3	0.215951376805097	AGIWUR18420
CB4856	CT	V	252364	254691	WBGene00006172	str-121	R02C2.5	-0.240684021239606	AGIWUR29847
CB4856	CT	V	254912	257992	WBGene00019816	nhr-204	R02C2.4	-0.0615973506913932	AGIWUR43394
CB4856	CT	V	268238	271419	WBGene00021981	lgc-26	Y58G8A.1	0.00370495785817138	AGIWUR1175
CB4856	CT	V	271984	272937	WBGene00044513	Y58G8A.5	Y58G8A.5	0.325783326207967	AGIWUR3273;AGIWUR7079;AGIWUR9802;AGIWUR25645;AGIWUR36238
CB4856	CT	V	276989	281716	WBGene00021983	npr-5	Y58G8A.4	-0.0812339314270076	AGIWUR6860;AGIWUR37723;AGIWUR42426
CB4856	CT	V	297949	300005	WBGene00018010	F33E11.2	F33E11.2	0	AGIWUR15878
CB4856	CT	V	300015	304713	WBGene00018011	F33E11.3	F33E11.3	-0.195376133339987	AGIWUR16662
CB4856	CT	V	305457	311565	WBGene00018013	phf-10	F33E11.6	-0.0776600076667822	AGIWUR7995;AGIWUR39428
CB4856	CT	V	320204	323647	WBGene00003614	nhr-15	F33E11.1	-0.0289980260294422	AGIWUR9738;AGIWUR22028;AGIWUR23891;AGIWUR36943;AGIWUR43837

CB4856	CT	V	323774	330725	WBGene00000022	abt-4	Y39D8C.1	-0.326985974386917	AGIWUR5072
CB4856	CT	V	334646	336861	WBGene00020708	dmd-8	T22H9.4	-0.196575597982343	AGIWUR27406
CB4856	CT	V	343428	347623	WBGene00020707	wago-10	T22H9.3	0.0406725864046127	AGIWUR2687;AGIWUR8699;AGIWUR10606;AGIWUR26893;AGIWUR28965;AGIWUR40430
CB4856	CT	V	349374	359016	WBGene00020706	atg-9	T22H9.2	0.110086897860803	AGIWUR12285;AGIWUR27158
CB4856	CT	V	359766	363705	WBGene00020705	T22H9.1	T22H9.1	0.241993480563416	AGIWUR40024
CB4856	CT	V	364950	369088	WBGene00021450	mltn-6	Y39D8B.2	0.246771248378135	AGIWUR25789
CB4856	CT	V	374408	379672	WBGene00021451	mltn-8	Y39D8B.3	-0.730141317302167	AGIWUR24991
CB4856	CT	V	381901	384688	WBGene00022643	ZK6.8	ZK6.8	0.0675764126852201	AGIWUR3417
CB4856	CT	V	385713	388090	WBGene00022642	lipl-5	ZK6.7	0.251946268225561	AGIWUR40960
CB4856	CT	V	388697	391598	WBGene00022641	ZK6.6	ZK6.6	-0.537473574481707	AGIWUR42231
CB4856	CT	V	392628	395117	WBGene00022640	nhr-254	ZK6.5	-0.309332983272659	AGIWUR28089
CB4856	CT	V	396827	398756	WBGene00022639	nhr-253	ZK6.4	-0.0640221753185504	AGIWUR19862
CB4856	CT	V	400192	401602	WBGene00022638	scl-27	ZK6.3	-0.0312093928236603	AGIWUR22678
CB4856	CT	V	401688	403128	WBGene00005284	srh-62	ZK6.9	-0.141764510019289	AGIWUR9555
CB4856	CT	V	404619	405997	WBGene00022637	nhr-252	ZK6.2	0.127823481205208	AGIWUR27857
CB4856	CT	V	408265	410080	WBGene00022636	nhr-278	ZK6.1	-0.0923685434839268	AGIWUR41645
CB4856	CT	V	411782	414760	WBGene00022644	dod-19	ZK6.10	-0.894266731795065	AGIWUR35435
CB4856	CT	V	415713	417708	WBGene00022645	ZK6.11	ZK6.11	-0.921085674130493	AGIWUR21388
CB4856	CT	V	419435	421839	WBGene00021448	Y39D8A.1	Y39D8A.1	-0.243558082671261	AGIWUR327;AGIWUR8494;AGIWUR13402;AGIWUR17855;AGIWUR18239;AGIWUR18452;AGIWUR22056;AGIWUR35812;AGIWUR41891;AGIWUR43529
CB4856	CT	V	422498	424064	WBGene00015259	dod-20	B0554.6	-0.475703073229829	AGIWUR38983
CB4856	CT	V	424440	426560	WBGene00015258	B0554.5	B0554.5	0.723628584808959	AGIWUR9976
CB4856	CT	V	429144	433574	WBGene00015257	B0554.4	B0554.4	-0.0191420484981464	AGIWUR2740;AGIWUR5771;AGIWUR24531
CB4856	CT	V	434198	436726	WBGene00015255	B0554.2	B0554.2	0.0972984046294898	AGIWUR11783;AGIWUR41253
CB4856	CT	V	437576	441023	WBGene00015260	B0554.7	B0554.7	-0.0417789503918193	AGIWUR16317
CB4856	CT	V	441820	443293	WBGene00005256	srh-33	M02H5.9	0.155799742842157	AGIWUR7920
CB4856	CT	V	444893	446674	WBGene00005257	srh-34	M02H5.10	-0.279658180925772	AGIWUR28651
CB4856	CT	V	447569	449230	WBGene00005258	srh-35	M02H5.11	0.0244654499670069	AGIWUR18900
CB4856	CT	V	449915	450438	WBGene00019744	M02H5.8	M02H5.8	0.175318960540668	AGIWUR37280

CB4856	CT	V	451451	453746	WBGene00003713	nhr-123	M02H5.7	-0.0738913432962711	AGIWUR38106
CB4856	CT	V	454009	456772	WBGene00003688	nhr-98	M02H5.6	-0.00790937685350457	AGIWUR11409
CB4856	CT	V	459734	461899	WBGene00019743	nhr-203	M02H5.5	-0.253595638045564	AGIWUR39587
CB4856	CT	V	462600	464963	WBGene00019742	nhr-202	M02H5.4	-0.221433001165732	AGIWUR16331
CB4856	CT	V	465757	467491	WBGene00019741	nhr-201	M02H5.3	0.311869627285576	AGIWUR3037
CB4856	CT	V	467567	468560	WBGene00019745	srbc-57	M02H5.12	0.0608109255979912	AGIWUR15097
CB4856	CT	V	472889	474631	WBGene00019740	srt-31	M02H5.2	0.00575762103267063	AGIWUR43022
CB4856	CT	V	474791	477150	WBGene00003689	nhr-99	M02H5.1	0.262389439533903	AGIWUR14192;AGIWUR23003;AGIWUR43277
CB4856	CT	V	478162	480710	WBGene00003645	nhr-55	T01G6.7	0.851159060913879	AGIWUR25933
CB4856	CT	V	480811	483355	WBGene00003720	nhr-130	T01G6.8	0.0450231818193298	AGIWUR34491
CB4856	CT	V	484901	486895	WBGene00020153	nhr-212	T01G6.6	0.593087898566724	AGIWUR23170
CB4856	CT	V	486990	489104	WBGene00020152	nhr-211	T01G6.5	0.346131016642238	AGIWUR16625;AGIWUR39859;AGIWUR39924
CB4856	CT	V	489647	491430	WBGene00003696	nhr-106	T01G6.4	-0.155209659309371	AGIWUR18097
CB4856	CT	V	491732	493641	WBGene00006233	str-196	T01G6.3	-0.0640646741916271	AGIWUR30475
CB4856	CT	V	494499	497287	WBGene00003721	nhr-131	T01G6.2	0.0508206430134841	AGIWUR5940
CB4856	CT	V	497839	498943	WBGene00020151	T01G6.1	T01G6.1	0.0797750762354514	AGIWUR24893
CB4856	CT	V	499770	501330	WBGene00006282	str-256	T01G6.9	0.0734191136351683	AGIWUR27783
CB4856	CT	V	502142	503223	WBGene00020154	T01G6.10	T01G6.10	0.0520447535983823	AGIWUR4126;AGIWUR17221;AGIWUR38447
CB4856	CT	V	504028	504616	WBGene00004144	pqn-60	R11G11.7	-0.369681075095334	AGIWUR13341
CB4856	CT	V	505491	506068	WBGene00020014	R11G11.6	R11G11.6	-0.440077310427837	AGIWUR21543
CB4856	CT	V	508112	509803	WBGene00005851	srw-104	R11G11.8	-0.0838195345737436	AGIWUR15163;AGIWUR43712
CB4856	CT	V	511194	512520	WBGene00005260	srh-37	R11G11.9	-0.0200661612830295	AGIWUR6632
CB4856	CT	V	513390	515226	WBGene00005852	srw-105	R11G11.10	0.289618486616847	AGIWUR41200
CB4856	CT	V	515787	517388	WBGene00005853	srw-106	R11G11.11	0.0879444732617172	AGIWUR15100
CB4856	CT	V	519852	521617	WBGene00005854	srw-107	R11G11.13	0.24830963776011	AGIWUR24048
CB4856	CT	V	521702	524218	WBGene00020016	lipl-3	R11G11.14	3.72018579076178	AGIWUR43454
CB4856	CT	V	525666	526691	WBGene00006119	str-54	R11G11.15	-0.0743836776526559	

									AGIWUR10962;AGIWUR38855
CB4856	CT	V	527902	528869	WBGene00005855	srw-108	R11G11.5	0.0131437765232256	AGIWUR42181
CB4856	CT	V	529277	530557	WBGene00005600	srj-12	R11G11.4	-0.0143279377695693	AGIWUR9716;AGIWUR30691
CB4856	CT	V	530799	532024	WBGene00020013	R11G11.3	R11G11.3	0.226241226304646	AGIWUR39705
CB4856	CT	V	532290	534064	WBGene00003648	nhr-58	R11G11.2	-0.198178638108612	AGIWUR814;AGIWUR43924
CB4856	CT	V	534446	536226	WBGene00003722	nhr-132	R11G11.1	0.323330200414125	AGIWUR30967
CB4856	CT	V	536628	537418	WBGene00015759	C14C6.5	C14C6.5	-0.824372373426195	AGIWUR33256;AGIWUR33998
CB4856	CT	V	539460	541485	WBGene00015758	nhr-155	C14C6.4	-0.257490059198716	AGIWUR27454
CB4856	CT	V	543469	545359	WBGene00015757	C14C6.3	C14C6.3	-0.417579511667713	AGIWUR38250
CB4856	CT	V	545844	548080	WBGene00015760	C14C6.6	C14C6.6	0.145082923851332	AGIWUR30450
CB4856	CT	V	548713	550619	WBGene00015761	C14C6.7	C14C6.7	0.115763394189858	AGIWUR41855
CB4856	CT	V	554374	556155	WBGene00005899	srx-8	C14C6.9	0.113085580114742	AGIWUR30075
CB4856	CT	V	560195	562956	WBGene00005898	srx-7	C14C6.1	-0.0665177647659494	AGIWUR985
CB4856	CT	V	563134	564446	WBGene00015756	C14C6.2	C14C6.2	1.03244872694961	AGIWUR44552
CB4856	CT	V	564391	566084	WBGene00005585	sri-73	C14C6.10	-0.368722670626145	AGIWUR19650
CB4856	CT	V	566719	568205	WBGene00005584	sri-72	C14C6.11	-0.564105251765247	AGIWUR20755
CB4856	CT	V	570571	571205	WBGene00015763	C14C6.12	C14C6.12	-0.229222697083953	AGIWUR2344
CB4856	CT	V	572031	574739	WBGene00015764	C14C6.13	C14C6.13	-0.0636287434393346	AGIWUR20391
CB4856	CT	V	576370	578428	WBGene00019453	K06H6.5	K06H6.5	0.0795321563879021	AGIWUR27993
CB4856	CT	V	581916	583431	WBGene00019451	nstp-7	K06H6.3	0.00627680429611566	AGIWUR29404
CB4856	CT	V	583801	585308	WBGene00019454	K06H6.6	K06H6.6	-0.0329969448095087	AGIWUR29139
CB4856	CT	V	585848	587250	WBGene00019450	K06H6.2	K06H6.2	-0.108443272260876	AGIWUR32952
CB4856	CT	V	588135	589646	WBGene00019449	K06H6.1	K06H6.1	-0.197958782050275	AGIWUR36953
CB4856	CT	V	590482	592463	WBGene00022758	ZK488.6	ZK488.6	0.184727081631352	AGIWUR10298
CB4856	CT	V	593259	595677	WBGene00022757	ZK488.5	ZK488.5	0.122263137720169	AGIWUR11121
CB4856	CT	V	600595	602482	WBGene00022756	nhr-251	ZK488.4	-0.4435318546485	AGIWUR21345
CB4856	CT	V	603665	604205	WBGene00004177	pqn-98	ZK488.7	-0.0093662979431306	AGIWUR12119
CB4856	CT	V	604723	606183	WBGene00005857	srw-110	ZK488.8	0.080929224014072	AGIWUR4227;AGIWUR21610

CB4856	CT	V	606781	608944	WBGene00005856	srw-109	ZK488.9	-0.155725965152802	AGIWUR43361
CB4856	CT	V	609144	609726	WBGene00004176	pqn-97	ZK488.10	0.129218855738223	AGIWUR11417
CB4856	CT	V	610445	612513	WBGene00003680	nhr-90	ZK488.2	0.529548180003193	AGIWUR33832
CB4856	CT	V	612781	614908	WBGene00022755	nhr-250	ZK488.1	0.171915816423036	AGIWUR20154
CB4856	CT	V	615576	616728	WBGene00018624	F48G7.13	F48G7.13	-0.0742807740876703	AGIWUR8576
CB4856	CT	V	619434	621679	WBGene00018615	F48G7.4	F48G7.4	-0.159030552327403	AGIWUR43563
CB4856	CT	V	622170	622951	WBGene00018616	F48G7.5	F48G7.5	-0.720414886882413	AGIWUR20604
CB4856	CT	V	623298	625022	WBGene00018617	srt-17	F48G7.6	0.235602976890406	AGIWUR22569
CB4856	CT	V	625752	626390	WBGene00018618	F48G7.7	F48G7.7	0.229011784808035	AGIWUR42807
CB4856	CT	V	627095	627650	WBGene00018619	F48G7.8	F48G7.8	-0.169005701926302	AGIWUR25428
CB4856	CT	V	628059	628623	WBGene00018620	F48G7.9	F48G7.9	-0.0532776135541129	AGIWUR23150
CB4856	CT	V	634142	634893	WBGene00018621	F48G7.10	F48G7.10	-0.0156485995485909	AGIWUR39400
CB4856	CT	V	636587	637501	WBGene00018614	pals-31	F48G7.2	1.00651154593172	AGIWUR42892
CB4856	CT	V	638628	639928	WBGene00005621	srj-37	F48G7.1	0.323461575378642	AGIWUR42858
CB4856	CT	V	640799	642893	WBGene00018622	nhr-190	F48G7.11	0.0197490090777634	AGIWUR20655
CB4856	CT	V	643762	646199	WBGene00018623	F48G7.12	F48G7.12	0.363528105731532	AGIWUR5201
CB4856	CT	V	647188	649741	WBGene00017959	ugt-42	F31F4.7	-0.523329320548356	AGIWUR22094
CB4856	CT	V	651837	653040	WBGene00005593	srj-5	F31F4.16	-0.156069919112609	AGIWUR33839
CB4856	CT	V	653303	654502	WBGene00005594	srj-6	F31F4.8	0.178477611425801	AGIWUR31845
CB4856	CT	V	654727	655923	WBGene00005451	srh-245	F31F4.9	-0.164142351328721	AGIWUR1794
CB4856	CT	V	656499	657778	WBGene00005452	srh-246	F31F4.18	0.478535477600463	AGIWUR12239
CB4856	CT	V	658476	660310	WBGene00017963	F31F4.17	F31F4.17	-0.0879897499702009	AGIWUR26694
CB4856	CT	V	660681	661954	WBGene00005320	srh-101	F31F4.10	-0.0994953953471933	AGIWUR14309;AGIWUR23039
CB4856	CT	V	662479	664443	WBGene00017960	F31F4.11	F31F4.11	0.144900110720882	AGIWUR28684
CB4856	CT	V	664797	665923	WBGene00005472	srh-266	F31F4.6	0.528313407240425	AGIWUR12540
CB4856	CT	V	665919	667494	WBGene00017961	nhr-180	F31F4.12	0.84307067980679	AGIWUR13553;AGIWUR38977;AGIWUR39200
CB4856	CT	V	667896	671442	WBGene00005687	sru-24	F31F4.13	0.0175380851113909	AGIWUR1691;AGIWUR6096;AGIWUR7045;AGIWUR16318;AGIWUR18111

CB4856	CT	V	672245	674313	WBGene00005688	sru-25	F31F4.14	-0.564309333321539	AGIWUR29944
CB4856	CT	V	674911	676754	WBGene00017962	fbxa-72	F31F4.15	-0.770789912631448	AGIWUR29718
CB4856	CT	V	677034	678920	WBGene00005912	srx-21	F31F4.4	-0.246939490729266	AGIWUR21802
CB4856	CT	V	679963	681465	WBGene00005913	srx-22	F31F4.3	0.201208239946715	AGIWUR42097;AGIWUR43628
CB4856	CT	V	682331	683970	WBGene00005914	srx-23	F31F4.2	-0.143355188392278	AGIWUR41650
CB4856	CT	V	684060	686289	WBGene00017957	F31F4.1	F31F4.1	-0.409667747142448	AGIWUR15879
CB4856	CT	V	687684	690004	WBGene00019244	H25P19.1	H25P19.1	-0.0828649423387482	AGIWUR40390
CB4856	CT	V	690875	692770	WBGene00005895	srx-4	Y49C4A.5	0.0643890711821902	AGIWUR19415
CB4856	CT	V	696523	699614	WBGene00021709	ugt-29	Y49C4A.8	-0.356618163791615	AGIWUR1072;AGIWUR38841
CB4856	CT	V	700079	701669	WBGene00005896	srx-5	Y49C4A.4	-0.0858581251766708	AGIWUR28320
CB4856	CT	V	702560	703745	WBGene00021708	srbc-37	Y49C4A.3	0.00909620601525152	AGIWUR3871;AGIWUR30163
CB4856	CT	V	707218	708453	WBGene00021707	srbc-38	Y49C4A.2	-0.0267474651303079	AGIWUR5061;AGIWUR12514;AGIWUR28023
CB4856	CT	V	709108	710566	WBGene00021706	srbc-39	Y49C4A.1	-0.328782178276168	AGIWUR2939
CB4856	CT	V	710642	712921	WBGene00021710	cyp-33C11	Y49C4A.9	0.0616246495440258	AGIWUR24386
CB4856	CT	V	715833	719831	WBGene00019242	srbc-40	H24O09.1	-0.0287649417086859	AGIWUR36794
CB4856	CT	V	722695	723827	WBGene00019243	fbxb-72	H24O09.2	0.496909486726286	AGIWUR15315
CB4856	CT	V	724207	728103	WBGene00001564	icl-1	C05E4.9	2.46287913154572	AGIWUR3072;AGIWUR10341;AGIWUR12073;AGIWUR15664;AGIWUR16363;AGIWUR22644;AGIWUR33040;AGIWUR42667
CB4856	CT	V	728312	734279	WBGene00005228	srh-2	C05E4.14	1.78199378919581	AGIWUR538;AGIWUR4676;AGIWUR34038
CB4856	CT	V	734420	736271	WBGene00005613	srj-25	C05E4.10	-0.0491834442579417	AGIWUR37614
CB4856	CT	V	736803	738870	WBGene00005612	srj-24	C05E4.11	-0.188352507345367	AGIWUR42876
CB4856	CT	V	742120	743273	WBGene00015489	C05E4.7	C05E4.7	0.0631373723479935	AGIWUR5225
CB4856	CT	V	743648	745475	WBGene00006182	str-134	C05E4.6	0.0954220660418077	AGIWUR31792

CB4856	CT	V	746397	747649	WBGene00006181	str-133	C05E4.5	0.119056235792366	AGIWUR2230
CB4856	CT	V	748344	750467	WBGene00005516	sri-4	C05E4.4	0.144170270649026	AGIWUR39397
CB4856	CT	V	751326	752779	WBGene00006288	str-262	C05E4.13	0.45662272612899	AGIWUR34956
CB4856	CT	V	753283	755124	WBGene00005642	srp-1	C05E4.3	0.288128194275675	AGIWUR27054
CB4856	CT	V	756469	757892	WBGene00006087	str-20	C05E4.2	0.123269544809314	AGIWUR33476
CB4856	CT	V	762734	764593	WBGene00005643	srp-2	C05E4.1	0.235678779548724	AGIWUR41978
CB4856	CT	V	765032	772585	WBGene00006266	str-238	R09E12.4	-0.104505522855353	AGIWUR4387
CB4856	CT	V	773518	774854	WBGene00019983	sti-1	R09E12.3	-0.160689599808472	AGIWUR10844;AGIWUR12820
CB4856	CT	V	774995	777272	WBGene00019982	srbc-58	R09E12.2	0.0534530909043962	AGIWUR39807
CB4856	CT	V	777571	778235	WBGene00044514	R09E12.9	R09E12.9	0.104469268124152	AGIWUR2083;AGIWUR5324;AGIWUR43324
CB4856	CT	V	778639	780223	WBGene00019981	srbc-59	R09E12.1	0.0825507229040499	AGIWUR13673;AGIWUR16438
CB4856	CT	V	780299	781803	WBGene00044091	srbc-60	R09E12.8	-0.273733659059939	AGIWUR11174
CB4856	CT	V	782732	784763	WBGene00020060	srab-16	R13D11.6	0.139681319658461	AGIWUR39334
CB4856	CT	V	787431	789888	WBGene00020059	srab-15	R13D11.5	0.161502092584874	AGIWUR26822;AGIWUR31101;AGIWUR41356
CB4856	CT	V	802550	804741	WBGene00020062	nhr-270	R13D11.8	0.388937765859344	AGIWUR7489
CB4856	CT	V	810793	814726	WBGene00020058	R13D11.4	R13D11.4	0.136043342082925	AGIWUR25263
CB4856	CT	V	815016	816873	WBGene00020057	R13D11.3	R13D11.3	-0.0401772413255195	AGIWUR20422
CB4856	CT	V	817482	818733	WBGene00020056	R13D11.1	R13D11.1	0.153577793444299	AGIWUR27687
CB4856	CT	V	823156	824972	WBGene00005921	srx-30	F41H8.3	-0.068873927556536	AGIWUR13632;AGIWUR38148
CB4856	CT	V	826411	828761	WBGene00018313	F41H8.2	F41H8.2	0.145617273213824	AGIWUR34562
CB4856	CT	V	833668	835463	WBGene00018312	F41H8.1	F41H8.1	0.235910288849212	AGIWUR32946
CB4856	CT	V	839421	839934	WBGene00044778	K09C6.10	K09C6.10	0.305102116181836	AGIWUR2115
CB4856	CT	V	844450	845373	WBGene00019560	K09C6.6	K09C6.6	-0.267328532960919	AGIWUR19380
CB4856	CT	V	846715	848858	WBGene00019559	srbc-13	K09C6.5	0.336395683718555	AGIWUR23450

CB4856	CT	V	849499	851332	WBGene00019558	srbc-12	K09C6.4	0.174423861993929	AGIWUR39150
CB4856	CT	V	852484	853126	WBGene00019557	K09C6.3	K09C6.3	-0.239256313862681	AGIWUR19218
CB4856	CT	V	853436	855082	WBGene00019563	K09C6.9	K09C6.9	0.0908768782859326	AGIWUR1931
CB4856	CT	V	856849	862061	WBGene00019556	K09C6.2	K09C6.2	-0.0339379425635453	AGIWUR23309;AGIWUR31796
CB4856	CT	V	865074	866829	WBGene00020155	T02B11.3	T02B11.3	0.211562474667897	AGIWUR9089;AGIWUR9821;AGIWUR16661;AGIWUR23990;AGIWUR26089;AGIWUR29584;AGIWUR40374;AGIWUR41779;AGIWUR44136
CB4856	CT	V	869481	871129	WBGene00020156	T02B11.4	T02B11.4	0.266462190953547	AGIWUR4354;AGIWUR20286;AGIWUR37778
CB4856	CT	V	874008	875728	WBGene00006118	str-53	T02B11.2	0.0458349733806553	AGIWUR25762;AGIWUR42040
CB4856	CT	V	876245	877265	WBGene00044777	T02B11.9	T02B11.9	0.00530596075334344	AGIWUR7027;AGIWUR16432;AGIWUR24052
CB4856	CT	V	877530	878667	WBGene00044776	T02B11.8	T02B11.8	-0.218906190345844	AGIWUR5303;AGIWUR25999;AGIWUR31851;AGIWUR40465;AGIWUR41074
CB4856	CT	V	879793	881574	WBGene00005210	srg-53	T02B11.1	0.182782839163862	AGIWUR879
CB4856	CT	V	882176	884424	WBGene00005622	srj-38	T02B11.5	-0.0464844319654256	AGIWUR650
CB4856	CT	V	884881	889274	WBGene00020157	T02B11.6	T02B11.6	0.243674842571292	AGIWUR2914;AGIWUR43173
CB4856	CT	V	894930	899187	WBGene00003550	nas-32	T02B11.7	0.0843900770944122	AGIWUR18338;AGIWUR20939;AGIWUR32505;AGIWUR38638;AGIWUR39727;AGIWUR42992
CB4856	CT	V	899310	903665	WBGene00001480	fmo-5	H24K24.5	0.511428664598725	AGIWUR16238
CB4856	CT	V	904950	911214	WBGene00019241	H24K24.4	H24K24.4	-0.432037912177096	AGIWUR38390
CB4856	CT	V	911776	916315	WBGene00019240	H24K24.3	H24K24.3	-0.281878888412924	AGIWUR3416;AGIWUR23565
CB4856	CT	V	917817	925945	WBGene00019239	H24K24.2	H24K24.2	0.453028038954082	AGIWUR26433
CB4856	CT	V	936464	939228	WBGene00021747	Y50D4C.2	Y50D4C.2	-0.0762624097312532	AGIWUR14286
CB4856	CT	V	939391	945244	WBGene00021748	Y50D4C.3	Y50D4C.3	0.204843507786499	AGIWUR37729
CB4856	CT	V	946732	949860	WBGene00021750	Y50D4C.6	Y50D4C.6	-0.124407157669911	AGIWUR25709
CB4856	CT	V	951454	964800	WBGene00005024	sqv-6	Y50D4C.4	-0.620933155994806	AGIWUR5529

CB4856	CT	V	982534	996207	WBGene00006770	unc-34	Y50D4C.1	0.00487083690952783	AGIWUR16552;AGIWUR41806
CB4856	CT	V	998289	1003753	WBGene00021749	Y50D4C.5	Y50D4C.5	-0.179926660421442	AGIWUR35566
CB4856	CT	V	1008803	1018350	WBGene00019971	ergo-1	R09A1.1	-0.0159973562837585	AGIWUR24215
CB4856	CT	V	1018337	1023144	WBGene00019972	R09A1.2	R09A1.2	-0.103628417618037	AGIWUR32045
CB4856	CT	V	1023782	1027073	WBGene00019973	R09A1.3	R09A1.3	-0.171679392573767	AGIWUR40489
CB4856	CT	V	1027904	1033385	WBGene00019975	flp-34	R09A1.5	-0.0845896127901042	AGIWUR18884;AGIWUR30787
CB4856	CT	V	1042692	1048580	WBGene00015344	nra-4	C02E11.1	-0.0848257852984009	AGIWUR11453;AGIWUR24900;AGIWUR31213;AGIWUR39654
CB4856	CT	V	1050126	1053014	WBGene00019625	K10C9.4	K10C9.4	-0.211472057094483	AGIWUR13036
CB4856	CT	V	1054602	1055773	WBGene00044515	K10C9.9	K10C9.9	-0.343790086238174	AGIWUR16400;AGIWUR22203;AGIWUR27064;AGIWUR31901;AGIWUR40912
CB4856	CT	V	1056548	1058987	WBGene00006255	str-224	K10C9.8	0.236646803511801	AGIWUR16313
CB4856	CT	V	1061595	1067908	WBGene00019624	K10C9.3	K10C9.3	0.455499257718998	AGIWUR40890;AGIWUR44492
CB4856	CT	V	1075200	1079597	WBGene00019623	K10C9.1	K10C9.1	0.31175595042657	AGIWUR25743;AGIWUR26540;AGIWUR29157;AGIWUR32093;AGIWUR32144;AGIWUR38999
CB4856	CT	V	1081483	1082372	WBGene00268189	Y50D4B.9	Y50D4B.9	0.0619277569569068	AGIWUR36491
CB4856	CT	V	1084316	1087272	WBGene00021745	Y50D4B.6	Y50D4B.6	-0.589459425049044	AGIWUR26156
CB4856	CT	V	1088283	1089891	WBGene00021744	clec-203	Y50D4B.5	-0.23330973862705	AGIWUR24505
CB4856	CT	V	1092657	1098951	WBGene00021743	Y50D4B.4	Y50D4B.4	-0.395236602600552	AGIWUR42320;AGIWUR44641
CB4856	CT	V	1101186	1104046	WBGene00021741	Y50D4B.2	Y50D4B.2	0.123319835129847	AGIWUR11172
CB4856	CT	V	1112933	1129360	WBGene00021740	Y50D4B.1	Y50D4B.1	0.114499470969116	AGIWUR41609;AGIWUR44635
CB4856	CT	V	1130088	1131942	WBGene00021072	W07B8.4	W07B8.4	0.108252928119978	AGIWUR31431
CB4856	CT	V	1132598	1134036	WBGene00000785	cpr-5	W07B8.5	-0.239937190854835	AGIWUR9037;AGIWUR27737;AGIWUR39996
CB4856	CT	V	1134485	1139172	WBGene00021070	cpr-8	W07B8.1	-0.364166215203042	AGIWUR5094
CB4856	CT	V	1141770	1146498	WBGene00020663	cutl-22	T21H3.4	0.15373012815635	AGIWUR17117
CB4856	CT	V	1156271	1158174	WBGene00000552	cmd-1	T21H3.3	0.0576600450364726	AGIWUR2590;AGIWUR9349;AGIWUR13292;AGIWUR33879;AGIWUR34932;AGIWUR37455;AGIWUR40916;AGIWUR45191
CB4856	CT	V	1165673	1174969	WBGene00004230	ptr-16	T21H3.2	-0.160326517013809	AGIWUR7971

CB4856 CT V 1176724 1178864 WBGene00020662 T21H3.1 T21H3.1 -0.0285662411914451
AGIWUR5708;AGIWUR6366;AGIWUR13610;AGIWUR28256;AGIWUR28832;AGIWUR36759
CB4856 CT V 1179604 1181548 WBGene00017764 F25A2.1 F25A2.1 0.00552866538436447
AGIWUR19707;AGIWUR22186;AGIWUR32036;AGIWUR37356
CB4856 CT V 1182806 1184644 WBGene00021737 Y50D4A.3 Y50D4A.3 0.0269629555067041
AGIWUR37975
CB4856 CT V 1196072 1207792 WBGene00021736 wrb-1 Y50D4A.2 0.112762300180551
AGIWUR29205
CB4856 CT V 1207889 1219874 WBGene00021735 Y50D4A.1 Y50D4A.1 -0.0289691121469193
AGIWUR17146
CB4856 CT V 1223901 1235445 WBGene00016539 madd-2 C39F7.2 0.479329801577002 AGIWUR12478
CB4856 CT V 1260465 1262616 WBGene00016538 C39F7.1 C39F7.1 -0.102957307462207 AGIWUR27762
CB4856 CT V 1262628 1263989 WBGene00004266 rab-1 C39F7.4 0.0123355438559116
AGIWUR8106;AGIWUR10175;AGIWUR15670;AGIWUR20977;AGIWUR30892;AGIWUR40582;AGIWUR43515
CB4856 CT V 1264773 1270160 WBGene00016540 C39F7.5 C39F7.5 0.304988332098898 AGIWUR14855
CB4856 CT V 1270911 1273043 WBGene00022288 Y75B7B.1 Y75B7B.1 -0.215647167926926
AGIWUR43411
CB4856 CT V 1274210 1276006 WBGene00022289 Y75B7B.2 Y75B7B.2 0.0143457908373641
AGIWUR15950
CB4856 CT V 1276420 1285700 WBGene00004053 tank-1 ZK1005.1 -0.0214567732425266
AGIWUR2481;AGIWUR29658;AGIWUR32914
CB4856 CT V 1288841 1299332 WBGene00001171 egl-2 F16B3.1 -0.0424368432773656 AGIWUR41827
CB4856 CT V 1315105 1315524 WBGene00017502 F16B3.2 F16B3.2 1.14134067736088 AGIWUR34396
CB4856 CT V 1315643 1317919 WBGene00044774 F16B3.3 F16B3.3 0.150593650897743
AGIWUR2659;AGIWUR2880;AGIWUR5172;AGIWUR5984;AGIWUR14866;AGIWUR18012;AGIWUR23552;AGIWUR24334;AGIWUR24824;AGIWUR30074;AGIWUR33277;AGIWUR37172
CB4856 CT V 1332275 1358164 WBGene00022285 Y75B7AL.2 Y75B7AL.2 0.267039560358764
AGIWUR8365;AGIWUR16645;AGIWUR25866;AGIWUR41586
CB4856 CT V 1373120 1383254 WBGene00022286 rga-4 Y75B7AL.4 0.0146755245613228
AGIWUR36713
CB4856 CT V 1391163 1392103 WBGene00022287 Y75B7AR.1 Y75B7AR.1 -0.27529384388297
AGIWUR8594
CB4856 CT V 1393112 1411616 WBGene00004925 snt-5 R12A1.2 -0.214381052815799
AGIWUR15426;AGIWUR26619;AGIWUR28167
CB4856 CT V 1422796 1425794 WBGene00020017 R12A1.3 R12A1.3 -0.0601228543456473
AGIWUR1907;AGIWUR36237
CB4856 CT V 1427653 1432137 WBGene00001578 ges-1 R12A1.4 -0.092476650643416 AGIWUR39263
CB4856 CT V 1434339 1435804 WBGene00019976 fbxa-195 R09B5.1 0.015100223258897
AGIWUR15706

CB4856	CT	V	1439179 1441871 WBGene00019979	R09B5.11	R09B5.11	0.189009873811916
AGIWUR21597
CB4856	CT	V	1442588 1442876 WBGene00044900	cnc-11	R09B5.13	0.585555270216959
AGIWUR2922;AGIWUR7262;AGIWUR9640;AGIWUR41712;AGIWUR43465
CB4856	CT	V	1444044 1444280 WBGene00000555	cnc-1	R09B5.2 0.0170410772735572	AGIWUR36656
CB4856	CT	V	1445381 1445832 WBGene00000559	cnc-5	R09B5.10	-0.129706424250248
AGIWUR14026
CB4856	CT	V	1447195 1447707 WBGene00000558	cnc-4	R09B5.9 0.0302281544239822	AGIWUR28592
CB4856	CT	V	1448004 1448790 WBGene00000557	cnc-3	R09B5.8 -0.231375678266884	AGIWUR39532
CB4856	CT	V	1449665 1450189 WBGene00000556	cnc-2	R09B5.3 -0.926324345174246
AGIWUR1432;AGIWUR12946;AGIWUR14737;AGIWUR38057;AGIWUR40413;AGIWUR45034
CB4856	CT	V	1451634 1454340 WBGene00005611	srj-23	R09B5.7 -0.0916750955753924
AGIWUR498;AGIWUR28473;AGIWUR36052
CB4856	CT	V	1455062 1456646 WBGene00019980	chil-14 R09B5.12	-0.000100053755134333
AGIWUR44056
CB4856	CT	V	1460369 1461613 WBGene00019978	hacd-1	R09B5.6 0.874167306048296
AGIWUR5379;AGIWUR19041;AGIWUR34145
CB4856	CT	V	1462063 1464699 WBGene00019977	fpn-1.2 R09B5.4 0.476598007748496	AGIWUR31821
CB4856	CT	V	1464990 1466360 WBGene00004139	pqn-54	R09B5.5 -0.760697458942918	AGIWUR41618
CB4856	CT	V	1467744 1471105 WBGene00018757	tag-304 F53E2.1 0.270131720238307	AGIWUR29558
CB4856	CT	V	1475072 1479576 WBGene00006794	unc-60	C38C3.5 -0.091421667473206
AGIWUR324;AGIWUR2449;AGIWUR2803;AGIWUR4564;AGIWUR7681;AGIWUR10222;AGIWUR18543;AGIWUR20925;AGIWUR26152;AGIWU
R28373;AGIWUR29218;AGIWUR39824;AGIWUR42409
CB4856	CT	V	1482042 1484015 WBGene00016513	C38C3.4 C38C3.4 0.524139097873061
AGIWUR1850;AGIWUR9335;AGIWUR14308
CB4856	CT	V	1484828 1491604 WBGene00016514	C38C3.6 C38C3.6 0.142154358462227	AGIWUR43075
CB4856	CT	V	1494769 1495453 WBGene00045390	C38C3.10	C38C3.10	0.142506164650311
AGIWUR32515
CB4856	CT	V	1495634 1497985 WBGene00016512	C38C3.3 C38C3.3 0.498550645142281	AGIWUR42559
CB4856	CT	V	1499440 1500687 WBGene00005690	sru-27	C38C3.2 0.0784875243637853	AGIWUR29744
CB4856	CT	V	1501420 1505310 WBGene00016516	C38C3.8 C38C3.8 0.00077348553917389
AGIWUR29041;AGIWUR31012
CB4856	CT	V	1510024 1511630 WBGene00016517	nhr-260 C38C3.9 0.142333662189456	AGIWUR14282
CB4856	CT	V	1518736 1519807 WBGene00023317	K02H11.8	K02H11.8	-0.487799695686156
AGIWUR4057
CB4856	CT	V	1520463 1521945 WBGene00006268	str-240 K02H11.2	-0.84232681196328
AGIWUR41821
CB4856	CT	V	1522785 1524224 WBGene00006270	str-243 K02H11.7	0.105714193329713
AGIWUR35522

CB4856 CT V 1524502 1525687 WBGene00006267 str-239 K02H11.1 0.0128512586507328
AGIWUR17216;AGIWUR44671
CB4856 CT V 1526467 1528085 WBGene00006265 str-236 K02H11.3 0.247626795389758
AGIWUR25872
CB4856 CT V 1528824 1531259 WBGene00019348 K02H11.4 K02H11.4 0.196816183794146
AGIWUR31621
CB4856 CT V 1532030 1533304 WBGene00006248 str-216 K02H11.5 -0.320396248400105
AGIWUR16290;AGIWUR33379
CB4856 CT V 1533892 1534415 WBGene00019349 trx-5 K02H11.6 0.0943346702650629
AGIWUR5711
CB4856 CT V 1535224 1537079 WBGene00019350 K02H11.9 K02H11.9 0.140718370030126
AGIWUR7169
CB4856 CT V 1538364 1541824 WBGene00018707 oac-31 F52F10.3 1.55538895133182
AGIWUR3780
CB4856 CT V 1542517 1545791 WBGene00018708 oac-32 F52F10.4 -0.354221232868326
AGIWUR37337
CB4856 CT V 1548143 1550701 WBGene00018706 F52F10.2 F52F10.2 0.18136933432609
AGIWUR30363
CB4856 CT V 1551044 1553102 WBGene00018709 srsx-8 F52F10.5 0.186832621646873
AGIWUR4739
CB4856 CT V 1567076 1585532 WBGene00000387 cdc-25.2 F16B4.8 0.217276573062147
AGIWUR22718;AGIWUR23475;AGIWUR34143;AGIWUR38017;AGIWUR40845
CB4856 CT V 1585722 1586028 WBGene00017509 F16B4.7 F16B4.7 0.649756994112585
AGIWUR2074;AGIWUR36242
CB4856 CT V 1588548 1591727 WBGene00017507 F16B4.5 F16B4.5 -0.163015472359618
AGIWUR22275;AGIWUR23024;AGIWUR35644
CB4856 CT V 1594019 1594665 WBGene00017506 F16B4.4 F16B4.4 -0.174621090866394
AGIWUR14436;AGIWUR21983;AGIWUR24255;AGIWUR25347;AGIWUR31339;AGIWUR42496
CB4856 CT V 1596144 1598689 WBGene00017505 F16B4.3 F16B4.3 -0.0993834837924172 AGIWUR20497
CB4856 CT V 1601038 1603916 WBGene00017504 F16B4.2 F16B4.2 -0.196597469264486 AGIWUR28708
CB4856 CT V 1603934 1607846 WBGene00017510 nhr-178 F16B4.9 0.296083863376385 AGIWUR31021
CB4856 CT V 1610263 1612747 WBGene00017503 nhr-177 F16B4.1 -0.189315241896422 AGIWUR1914
CB4856 CT V 1612803 1614826 WBGene00017511 srbc-41 F16B4.10 -0.145756811010344
AGIWUR42512
CB4856 CT V 1615723 1618072 WBGene00017512 nhr-179 F16B4.11 0.32210746117959
AGIWUR12425
CB4856 CT V 1619194 1621536 WBGene00003707 nhr-117 F16B4.12 0.600871090469698
AGIWUR11701;AGIWUR28007;AGIWUR41153;AGIWUR42311
CB4856 CT V 1622636 1624433 WBGene00003700 nhr-110 Y46H3D.5 0.326762325488025

AGIWUR16766
CB4856 CT V 1625225 1626818 WBGene00021610 nhr-237 Y46H3D.6 -0.212856151407593
AGIWUR3104
CB4856 CT V 1628106 1629432 WBGene00021611 nhr-238 Y46H3D.7 0.012518058068105
AGIWUR1379
CB4856 CT V 1630309 1631248 WBGene00021612 Y46H3D.8 Y46H3D.8 -0.0448712724733529
AGIWUR27368
CB4856 CT V 1632017 1635883 WBGene00021609 irld-17 Y46H3D.4 0.0700576675004854
AGIWUR27296
CB4856 CT V 1636159 1639281 WBGene00006253 str-222 Y46H3D.3 0.116775046023865
AGIWUR13494;AGIWUR21212;AGIWUR21818
CB4856 CT V 1640308 1642287 WBGene00006252 str-221 Y46H3D.2 0.0461082624585183
AGIWUR10629;AGIWUR12769;AGIWUR24631
CB4856 CT V 1642499 1643121 WBGene00021608 Y46H3D.1 Y46H3D.1 -0.511298278083675
AGIWUR2223;AGIWUR11726;AGIWUR13295;AGIWUR14828;AGIWUR29088;AGIWUR32631;AGIWUR39494
CB4856 CT V 1644169 1647728 WBGene00000108 alh-2 K04F1.15 -0.258877936489031
AGIWUR6897
CB4856 CT V 1648300 1651143 WBGene00019393 irld-45 K04F1.14 -0.075381449234488
AGIWUR7514
CB4856 CT V 1651345 1654444 WBGene00019392 irld-44 K04F1.13 -0.100081069461194
AGIWUR8683
CB4856 CT V 1655090 1657364 WBGene00019391 irld-11 K04F1.12 0.294519309979566
AGIWUR2258
CB4856 CT V 1658072 1659636 WBGene00019384 K04F1.1 K04F1.1 0.0432968603609698 AGIWUR27851
CB4856 CT V 1660297 1662150 WBGene00019390 irld-43 K04F1.11 -0.120731616670201
AGIWUR17985;AGIWUR42790
CB4856 CT V 1662570 1664503 WBGene00019389 irld-40 K04F1.10 0.0582388246835448
AGIWUR26847
CB4856 CT V 1664650 1665950 WBGene00019388 K04F1.9 K04F1.9 -0.620442932092971 AGIWUR20834
CB4856 CT V 1667204 1667813 WBGene00019387 K04F1.8 K04F1.8 -0.279535995774246 AGIWUR38947
CB4856 CT V 1668516 1670630 WBGene00019386 irld-42 K04F1.7 -0.195086751571878 AGIWUR31674
CB4856 CT V 1670973 1673439 WBGene00019385 irld-41 K04F1.6 0.09199018572623 AGIWUR21923
CB4856 CT V 1673543 1675613 WBGene00005843 srw-96 K04F1.2 0.023001476594577 AGIWUR42752
CB4856 CT V 1676474 1678328 WBGene00005845 srw-98 K04F1.3 -0.445268257091452 AGIWUR42917
CB4856 CT V 1679208 1680870 WBGene00005838 srw-91 K04F1.4 0.14179062029317 AGIWUR23336
CB4856 CT V 1682579 1685089 WBGene00005229 srh-3 K04F1.5 -0.0372964521539991 AGIWUR13668
CB4856 CT V 1685628 1686864 WBGene00005231 srh-5 K04F1.16 -0.382412204541616
AGIWUR20402
CB4856 CT V 1687495 1689687 WBGene00021604 Y46H3C.4 Y46H3C.4 0.0574326592020977

AGIWUR18415
CB4856	CT	V	1691029	1692052	WBGene00021605	Y46H3C.5	Y46H3C.5	-0.461491209020982	AGIWUR3831
CB4856	CT	V	1697889	1701617	WBGene00021606	Y46H3C.6	Y46H3C.6	0.120743831058149	AGIWUR43236
CB4856	CT	V	1702051	1704053	WBGene00005837	srw-90	Y46H3C.3	0.226160857799806	AGIWUR9837
CB4856	CT	V	1708741	1710663	WBGene00005846	srw-99	Y46H3C.2	0.0540156937887026	AGIWUR13161
CB4856	CT	V	1711209	1715399	WBGene00005847	srw-100	Y46H3C.1	0.123274774769103	AGIWUR25705
CB4856	CT	V	1717243	1719464	WBGene00005850	srw-103	ZK697.5	0.0117242423405619	AGIWUR13701
CB4856	CT	V	1720227	1721973	WBGene00005849	srw-102	ZK697.12	0.178337618958709	AGIWUR23406
CB4856	CT	V	1722266	1725261	WBGene00005848	srw-101	ZK697.13	0.204360141119221	AGIWUR6004
CB4856	CT	V	1725653	1727632	WBGene00005539	sri-27	ZK697.4	-0.0425895043569842	AGIWUR23655
CB4856	CT	V	1729116	1730649	WBGene00022806	ZK697.3	ZK697.3	-0.0562367839067122	AGIWUR32139
CB4856	CT	V	1730759	1732000	WBGene00001769	gst-21	ZK697.6	-0.381808106163009	AGIWUR36708
CB4856	CT	V	1734869	1736767	WBGene00006205	str-160	ZK697.10	0.0975470880233615	AGIWUR1209
CB4856	CT	V	1738927	1741157	WBGene00022807	srab-25	ZK697.7	0.226685779458969	AGIWUR16633
CB4856	CT	V	1743771	1747969	WBGene00022805	nhr-256	ZK697.2	-0.0477264669894648	AGIWUR7017;AGIWUR8600;AGIWUR28647
CB4856	CT	V	1748432	1749153	WBGene00022808	ZK697.8	ZK697.8	0.251989930888247	AGIWUR17448;AGIWUR25011;AGIWUR27665;AGIWUR31649
CB4856	CT	V	1750001	1751549	WBGene00005410	srh-199	ZK697.11	0.535957730668755	AGIWUR10138;AGIWUR40344;AGIWUR44497
CB4856	CT	V	1752796	1753856	WBGene00022804	ZK697.1	ZK697.1	-0.173983884655766	AGIWUR8405
CB4856	CT	V	1754238	1755689	WBGene00022809	ZK697.14	ZK697.14	-0.226744456235386	AGIWUR31379
CB4856	CT	V	1760861	1765305	WBGene00021602	clec-205	Y46H3B.1	-0.416430748737017	AGIWUR8341
CB4856	CT	V	1765311	1771734	WBGene00000976	dhs-13	F36H9.3	-0.0905692853859694	AGIWUR16906;AGIWUR23989;AGIWUR24571;AGIWUR29221;AGIWUR33084;AGIWUR37543
CB4856	CT	V	1773826	1774918	WBGene00018115	F36H9.7	F36H9.7	0.0436907987867006	AGIWUR6284;AGIWUR24760;AGIWUR25424;AGIWUR26480
CB4856	CT	V	1779657	1782022	WBGene00018113	F36H9.5	F36H9.5	-0.226325431866393	AGIWUR32450
CB4856	CT	V	1788511	1793250	WBGene00018111	F36H9.2	F36H9.2	-0.119930314261461	AGIWUR7637

CB4856 CT V 1798200 1799694 WBGene00268194 F36H9.10 F36H9.10 0.23510267044969
AGIWUR33123
CB4856 CT V 1799726 1803143 WBGene00018114 srt-41 F36H9.6 0.0669237330394825
AGIWUR1923;AGIWUR21739;AGIWUR39411
CB4856 CT V 1804334 1804971 WBGene00002016 hsp-16.2 Y46H3A.3 0.567398390425119
AGIWUR33481
CB4856 CT V 1805178 1805875 WBGene00002018 hsp-16.41 Y46H3A.2 0.512334747160087
AGIWUR5399;AGIWUR40972
CB4856 CT V 1807450 1809468 WBGene00021599 srt-42 Y46H3A.1 0.165768659009745
AGIWUR28255;AGIWUR43360
CB4856 CT V 1809560 1811135 WBGene00021600 Y46H3A.4 Y46H3A.4 0.0445435438667925
AGIWUR20038
CB4856 CT V 1811423 1814116 WBGene00021601 Y46H3A.5 Y46H3A.5 0.0119550765469203
AGIWUR26944;AGIWUR42495
CB4856 CT V 1815852 1821323 WBGene00001632 gly-7 Y46H3A.6 0.0965331517213119
AGIWUR9988
CB4856 CT V 1821579 1823096 WBGene00044344 mrpl-39 Y46H3A.7 -0.375903948347645
AGIWUR20002
CB4856 CT V 1823077 1830073 WBGene00021309 Y32G9B.1 Y32G9B.1 -0.0831079090235818
AGIWUR23123
CB4856 CT V 1842044 1844807 WBGene00020394 T10B5.8 T10B5.8 -0.0695252009642222
AGIWUR20519;AGIWUR29587
CB4856 CT V 1846201 1848015 WBGene00020393 T10B5.7 T10B5.7 -0.030790753559792 AGIWUR22131
CB4856 CT V 1848073 1851445 WBGene00020388 T10B5.2 T10B5.2 -0.116648930483843 AGIWUR27215
CB4856 CT V 1852449 1856276 WBGene00020396 T10B5.10 T10B5.10 -0.166215517782025
AGIWUR30645
CB4856 CT V 1864181 1868571 WBGene00020389 T10B5.3 T10B5.3 -0.0977577151303555
AGIWUR38;AGIWUR4999;AGIWUR9987;AGIWUR31559
CB4856 CT V 1869213 1870558 WBGene00020392 knl-3 T10B5.6 -0.279062582967544 AGIWUR5520
CB4856 CT V 1870421 1871789 WBGene00020390 T10B5.4 T10B5.4 -0.372268666991447 AGIWUR20357
CB4856 CT V 1872246 1875526 WBGene00020391 cct-7 T10B5.5 -0.152869714292683
AGIWUR3536;AGIWUR13115;AGIWUR17600;AGIWUR24679;AGIWUR37923
CB4856 CT V 1875478 1882265 WBGene00021305 zig-11 Y32G9A.8 -0.357309250644564
AGIWUR22727
CB4856 CT V 1895797 1906560 WBGene00021304 nphp-2 Y32G9A.6 0.0103488104346319
AGIWUR40867
CB4856 CT V 1907654 1908528 WBGene00021303 Y32G9A.5 Y32G9A.5 0.0433395939944488
AGIWUR12249
CB4856 CT V 1908855 1910692 WBGene00005644 srp-3 Y32G9A.4 -0.000373541149670586

AGIWUR13979
CB4856 CT V 1911402 1913572 WBGene00021302 Y32G9A.3 Y32G9A.3 0.39395327403501
AGIWUR15769
CB4856 CT V 1913627 1917986 WBGene00044517 Y32G9A.13 Y32G9A.13 -0.0206143164446022
AGIWUR6335;AGIWUR14178;AGIWUR20443;AGIWUR22055;AGIWUR33726;AGIWUR37482
CB4856 CT V 1918539 1921029 WBGene00021301 Y32G9A.2 Y32G9A.2 0.0107461367881854
AGIWUR19923
CB4856 CT V 1921621 1922381 WBGene00001785 gst-37 Y32G9A.1 0.0606499695546749
AGIWUR4468;AGIWUR15840
CB4856 CT V 1923116 1925565 WBGene00020277 T06A1.1 T06A1.1 -0.194843006997663 AGIWUR34808
CB4856 CT V 1927223 1931413 WBGene00020280 T06A1.5 T06A1.5 -0.546358038822159 AGIWUR21341
CB4856 CT V 1944955 1953349 WBGene00020279 glb-25 T06A1.4 -0.0200390917615447
AGIWUR627;AGIWUR1111;AGIWUR4223;AGIWUR7808;AGIWUR17908;AGIWUR20988;AGIWUR21891;AGIWUR23733;AGIWUR24723;AGIWUR37834;AGIWUR45105
CB4856 CT V 1953912 1956560 WBGene00005604 srj-16 T06A1.2 0.0376198905727883 AGIWUR33567
CB4856 CT V 1957417 1957932 WBGene00044518 fipr-25 T06A1.7 0.0225855406838846
AGIWUR1522;AGIWUR16811;AGIWUR24089;AGIWUR29190;AGIWUR41979
CB4856 CT V 1960137 1961403 WBGene00021494 Y40B10B.1 Y40B10B.1 1.78742410156884
AGIWUR15579
CB4856 CT V 1962815 1964906 WBGene00005603 srj-15 Y40B10B.2 -0.102453437152307
AGIWUR9421;AGIWUR25828
CB4856 CT V 1969528 1974514 WBGene00020343 acs-3 T08B1.6 0.62613636506598 AGIWUR39853
CB4856 CT V 1975418 1977261 WBGene00020342 fbxa-201 T08B1.5 -0.0988464747209452
AGIWUR23246;AGIWUR29026;AGIWUR35886
CB4856 CT V 1979435 1982664 WBGene00020341 T08B1.4 T08B1.4 -0.00433825518155162
AGIWUR13175;AGIWUR14799;AGIWUR20161;AGIWUR27409;AGIWUR38153;AGIWUR43363
CB4856 CT V 1982631 1987045 WBGene00020340 T08B1.1 T08B1.1 -0.565644689124785 AGIWUR23266
CB4856 CT V 1989476 1992918 WBGene00006589 tnt-4 T08B1.2 0.355618230598294
AGIWUR21520;AGIWUR45074
CB4856 CT V 1996100 1999581 WBGene00000111 alh-5 T08B1.3 2.0133902348546 AGIWUR37322
CB4856 CT V 2000418 2004329 WBGene00019089 clec-206 F59A7.1 0.0298505485742977
AGIWUR8715
CB4856 CT V 2005057 2006804 WBGene00019096 cysl-4 F59A7.9 -0.334082759816491 AGIWUR38987
CB4856 CT V 2007360 2007709 WBGene00023306 F59A7.10 F59A7.10 -0.158757333278913
AGIWUR28358
CB4856 CT V 2008609 2011620 WBGene00019095 F59A7.8 F59A7.8 -0.671695028824954 AGIWUR11972
CB4856 CT V 2013418 2013860 WBGene00044519 F59A7.11 F59A7.11 0.190665590135053
AGIWUR11090;AGIWUR18576
CB4856 CT V 2015836 2016541 WBGene00019090 F59A7.2 F59A7.2 0.469026342781453

AGIWUR3374;AGIWUR38564
CB4856 CT V 2022674 2024710 WBGene00019092 F59A7.5 F59A7.5 0.429795826244831
AGIWUR8988;AGIWUR21220
CB4856 CT V 2030177 2030407 WBGene00044520 F59A7.12 F59A7.12 -0.0795076684782146
AGIWUR13534
CB4856 CT V 2030619 2031475 WBGene00001857 hil-6 F59A7.4 0.407328319320007
AGIWUR4410;AGIWUR41283
CB4856 CT V 2033792 2035645 WBGene00021488 srab-23 Y40B10A.3 -0.0620545427814889
AGIWUR34938;AGIWUR35892;AGIWUR36570
CB4856 CT V 2038193 2042359 WBGene00021489 Y40B10A.4 Y40B10A.4 -0.0844291579329326
AGIWUR1482
CB4856 CT V 2043352 2046253 WBGene00021490 Y40B10A.5 Y40B10A.5 -0.0311579158489916
AGIWUR16333
CB4856 CT V 2049223 2050172 WBGene00021491 comt-4 Y40B10A.6 0.780988779695596
AGIWUR40501
CB4856 CT V 2052231 2053600 WBGene00021492 comt-5 Y40B10A.7 -0.376809123473883
AGIWUR41205
CB4856 CT V 2057537 2061129 WBGene00021487 comt-3 Y40B10A.2 -0.701215474760629
AGIWUR4448
CB4856 CT V 2061111 2066844 WBGene00003676 nhr-86 Y40B10A.8 -0.0600497012559842
AGIWUR16383
CB4856 CT V 2068449 2069143 WBGene00021486 lbp-9 Y40B10A.1 -0.122964208695053
AGIWUR759;AGIWUR42829
CB4856 CT V 2069207 2072896 WBGene00021493 Y40B10A.9 Y40B10A.9 0.683045466175507
AGIWUR9779
CB4856 CT V 2086279 2089819 WBGene00018094 F36F12.3 F36F12.3 0.0369325710346295
AGIWUR2494;AGIWUR8943;AGIWUR11717;AGIWUR13195;AGIWUR14727;AGIWUR15308;AGIWUR15958;AGIWUR17370;AGIWUR21645;AGIWUR21769;AGIWUR24549;AGIWUR30250;AGIWUR30745
CB4856 CT V 2090669 2091735 WBGene00018099 ztf-28 F36F12.8 -0.13447037981474
AGIWUR35689
CB4856 CT V 2096072 2097257 WBGene00018096 clec-207 F36F12.5 -0.517646787173264
AGIWUR13902
CB4856 CT V 2097953 2099153 WBGene00018097 clec-208 F36F12.6 -0.465954484049446
AGIWUR17623
CB4856 CT V 2099349 2100051 WBGene00018098 F36F12.7 F36F12.7 0.0511045592604535
AGIWUR13503
CB4856 CT V 2100062 2103218 WBGene00018092 F36F12.1 F36F12.1 0.483551971032971
AGIWUR13743
CB4856 CT V 2103658 2106076 WBGene00017722 F22F7.4 F22F7.4 0.312943293741526 AGIWUR23521

CB4856 CT V 2106398 2109418 WBGene00000514 ckb-4 F22F7.5 -0.395938768257247
AGIWUR17305;AGIWUR28551;AGIWUR34233
CB4856 CT V 2115490 2118644 WBGene00017723 bgnt-1.2 F22F7.6 0.294961506391655
AGIWUR5028
CB4856 CT V 2122866 2127299 WBGene00017720 F22F7.2 F22F7.2 -0.5074266639897
AGIWUR13725;AGIWUR30695
CB4856 CT V 2127535 2130100 WBGene00017719 ldp-1 F22F7.1 -0.0304503778297445 AGIWUR26701
CB4856 CT V 2131476 2140042 WBGene00017724 F22F7.7 F22F7.7 0.0666554528936238 AGIWUR38755
CB4856 CT V 2144020 2144508 WBGene00268198 F22F7.12 F22F7.12 0.071780894811651
AGIWUR29493
CB4856 CT V 2147095 2148626 WBGene00016693 srbc-20 C45H4.11 0.29482368403711
AGIWUR11502
CB4856 CT V 2151762 2153010 WBGene00016692 srbc-24 C45H4.10 -0.0309137438275995
AGIWUR29865
CB4856 CT V 2153574 2155112 WBGene00016691 srbc-23 C45H4.9 0.000753297753041338 AGIWUR35642
CB4856 CT V 2155532 2156635 WBGene00016689 srbc-21 C45H4.7 0.128582098141027 AGIWUR9672
CB4856 CT V 2157130 2158274 WBGene00016690 srbc-22 C45H4.8 0.0465002588590337 AGIWUR18608
CB4856 CT V 2158674 2159427 WBGene00016695 C45H4.14 C45H4.14 -0.316257631612597
AGIWUR5246;AGIWUR27391;AGIWUR40317
CB4856 CT V 2161500 2163835 WBGene00006281 str-255 C45H4.15 0.146957262456146
AGIWUR39636
CB4856 CT V 2164826 2166779 WBGene00016696 srbc-19 C45H4.16 0.0189703306862228
AGIWUR16474;AGIWUR35248;AGIWUR37129
CB4856 CT V 2169306 2170668 WBGene00016688 srbc-18 C45H4.6 -0.0608779928116761 AGIWUR30026
CB4856 CT V 2171418 2174412 WBGene00005709 sru-46 C45H4.5 0.245278988853958 AGIWUR11450
CB4856 CT V 2175542 2177503 WBGene00044087 C45H4.18 C45H4.18 0.0715135606629742
AGIWUR4518;AGIWUR36492
CB4856 CT V 2177714 2179116 WBGene00016687 srbc-17 C45H4.3 0.194416996626674 AGIWUR32870
CB4856 CT V 2179586 2181759 WBGene00016686 cyp-33C1 C45H4.2 0.138168478075441
AGIWUR43067
CB4856 CT V 2182038 2183191 WBGene00016685 srbc-16 C45H4.1 -0.271316394620184 AGIWUR28585
CB4856 CT V 2183521 2186535 WBGene00016697 cyp-33C2 C45H4.17 0.84792847393517
AGIWUR13520
CB4856 CT V 2188280 2189062 WBGene00021165 Y5H2B.3 Y5H2B.3 -0.0492525940127422 AGIWUR30403
CB4856 CT V 2189586 2191222 WBGene00021166 srbc-42 Y5H2B.4 0.0779400191873876 AGIWUR16020
CB4856 CT V 2192477 2196823 WBGene00003612 nhr-13 Y5H2B.2 0.0509811601032233 AGIWUR31918
CB4856 CT V 2198059 2201415 WBGene00021167 cyp-32B1 Y5H2B.5 1.34525008170031
AGIWUR20546
CB4856 CT V 2201581 2204397 WBGene00021168 cyp-33C12 Y5H2B.6 -0.00475951668510355

AGIWUR27493
CB4856 CT V 2205030 2206281 WBGene00021169 srt-40 Y5H2B.7 0.12542044394365
AGIWUR17162;AGIWUR26968;AGIWUR27794
CB4856 CT V 2207180 2210209 WBGene00021164 Y5H2B.1 Y5H2B.1 0.790531577346478 AGIWUR20260
CB4856 CT V 2210516 2212114 WBGene00003723 nhr-133 F44C8.8 0.333047058810536 AGIWUR22577
CB4856 CT V 2212899 2214460 WBGene00018415 nhr-184 F44C8.9 -0.185691893017299
AGIWUR15480;AGIWUR20409;AGIWUR32815;AGIWUR32949;AGIWUR33909
CB4856 CT V 2215724 2218091 WBGene00018414 nstp-8 F44C8.7 -0.133044930134519 AGIWUR5605
CB4856 CT V 2219004 2221305 WBGene00003646 nhr-56 F44C8.6 0.386075347382697 AGIWUR16627
CB4856 CT V 2221948 2225296 WBGene00003718 nhr-128 F44C8.5 0.0740838764815654
AGIWUR14749;AGIWUR18311;AGIWUR25768;AGIWUR26935
CB4856 CT V 2225430 2227042 WBGene00003693 nhr-103 F44C8.4 0.366725519166236 AGIWUR14265
CB4856 CT V 2227203 2228981 WBGene00003617 nhr-18 F44C8.3 0.717810769378101 AGIWUR23493
CB4856 CT V 2229081 2230984 WBGene00003716 nhr-126 F44C8.10 0.0680893998518304
AGIWUR3148;AGIWUR9146;AGIWUR11153;AGIWUR13084
CB4856 CT V 2231124 2233381 WBGene00003724 nhr-134 F44C8.2 0.492961258694713 AGIWUR787
CB4856 CT V 2233482 2235138 WBGene00003686 nhr-96 F44C8.11 0.217795426773719
AGIWUR29784
CB4856 CT V 2236162 2237937 WBGene00018413 cyp-33C4 F44C8.1 0.242081688089975
AGIWUR1806;AGIWUR8637
CB4856 CT V 2242196 2242772 WBGene00023039 F41B5.11 F41B5.11 0.0329763219453181
AGIWUR16567
CB4856 CT V 2242778 2244871 WBGene00018262 cyp-33C3 F41B5.4 0.30138558857859
AGIWUR43831
CB4856 CT V 2245015 2247015 WBGene00018261 cyp-33C5 F41B5.3 0.479042024512298
AGIWUR42271
CB4856 CT V 2247472 2249335 WBGene00018264 cyp-33C6 F41B5.7 0.167497198938484
AGIWUR36205
CB4856 CT V 2250545 2252553 WBGene00018260 cyp-33C7 F41B5.2 -0.082752534756418
AGIWUR39759
CB4856 CT V 2252804 2254603 WBGene00006279 str-253 F41B5.8 -0.104212102734246 AGIWUR43104
CB4856 CT V 2255533 2256658 WBGene00023284 F41B5.1 F41B5.1 0.0066443262450111 AGIWUR11202
CB4856 CT V 2257420 2259913 WBGene00018265 nhr-182 F41B5.9 -0.0100964195509938 AGIWUR41433
CB4856 CT V 2262489 2264634 WBGene00018266 nhr-183 F41B5.10 -0.138212663212597
AGIWUR34818;AGIWUR34908
CB4856 CT V 2264909 2267058 WBGene00021163 nhr-275 Y5H2A.2 0.505694330743055 AGIWUR10872
CB4856 CT V 2271175 2271861 WBGene00021162 Y5H2A.1 Y5H2A.1 -0.114913142068219 AGIWUR20420
CB4856 CT V 2272441 2274353 WBGene00044521 Y5H2A.4 Y5H2A.4 -0.251127308932038 AGIWUR553
CB4856 CT V 2274842 2275977 WBGene00000027 abu-4 Y5H2A.3 -0.358553566224389 AGIWUR11350

CB4856	CT	V	2276018	2278246	WBGene00003649	nhr-59	T27B7.1	0.661579311086447	AGIWUR5243
CB4856	CT	V	2278826	2281387	WBGene00020853	fbxa-137	T27B7.7	0.978914054317864	AGIWUR29600
CB4856	CT	V	2281828	2284963	WBGene00020852	nhr-228	T27B7.6	0.233910175327392	AGIWUR36659
CB4856	CT	V	2285279	2287520	WBGene00020851	nhr-227	T27B7.5	-0.184467963201612	AGIWUR14453
CB4856	CT	V	2288328	2290468	WBGene00020849	nhr-225	T27B7.2	-0.475948201491496	AGIWUR19641
CB4856	CT	V	2288487	2294798	WBGene00003705	nhr-115	T27B7.4	-0.536237403300046	AGIWUR29278
CB4856	CT	V	2294822	2296967	WBGene00020850	nhr-226	T27B7.3	-0.231449721267317	AGIWUR5248
CB4856	CT	V	2298945	2301005	WBGene00235117	Y19D10B.8	Y19D10B.8	-0.0291735092325602	AGIWUR33818
CB4856	CT	V	2301743	2303868	WBGene00021232	irld-15	Y19D10B.3	0.0236621858539081	AGIWUR1695
CB4856	CT	V	2304355	2309159	WBGene00021233	Y19D10B.4	Y19D10B.4	-0.323997122392827	AGIWUR30881
CB4856	CT	V	2311291	2313809	WBGene00021230	Y19D10B.1	Y19D10B.1	-0.982501673173429	AGIWUR23689
CB4856	CT	V	2313962	2318163	WBGene00021234	irld-16	Y19D10B.5	-0.383619253448956	AGIWUR19389
CB4856	CT	V	2324510	2326827	WBGene00017501	pud-3	F15E11.15	-0.42084311823069	AGIWUR26728
CB4856	CT	V	2326678	2327262	WBGene00017498	pud-4	F15E11.12	-0.644435930057527	AGIWUR26194
CB4856	CT	V	2339567	2341408	WBGene00017493	irld-30	F15E11.4	0.0717232274928143	AGIWUR10991
CB4856	CT	V	2342016	2344508	WBGene00017494	irld-31	F15E11.5	0.167006178348256	AGIWUR16975
CB4856	CT	V	2453414	2456147	WBGene00018911	F56A4.3	F56A4.3	-0.116285804671693	AGIWUR27891
CB4856	CT	V	2561690	2564701	WBGene00001758	gst-10	Y45G12C.2	-0.691188948854242	AGIWUR3108;AGIWUR12703;AGIWUR23562;AGIWUR42382
CB4856	CT	V	2565683	2568878	WBGene00021565	Y45G12C.1	Y45G12C.1	-0.512456400970208	AGIWUR33496
CB4856	CT	V	2568876	2570743	WBGene00005607	srj-19	Y45G12C.14	-0.0544018205314845	AGIWUR27902;AGIWUR41936
CB4856	CT	V	2571527	2573208	WBGene00005608	srj-20	Y45G12C.15	0.266493245709333	AGIWUR27705
CB4856	CT	V	2577903	2579816	WBGene00016232	srt-4	C29G2.4	0.137990246202191	AGIWUR20535
CB4856	CT	V	2580872	2582939	WBGene00016231	C29G2.3	C29G2.3	-0.0203053248510532	AGIWUR37256
CB4856	CT	V	2582867	2585908	WBGene00016234	C29G2.6	C29G2.6	-0.110656539227939	AGIWUR28534
CB4856	CT	V	2590393	2591758	WBGene00016230	C29G2.2	C29G2.2	-0.000198791710264601	

AGIWUR19539;AGIWUR20711;AGIWUR24384;AGIWUR28086;AGIWUR35312;AGIWUR36193;AGIWUR41857
CB4856 CT V 2592487 2595062 WBGene00018760 irg-3 F53E10.4 0.0303800791378688
AGIWUR34840
CB4856 CT V 2596020 2598930 WBGene00018761 F53E10.5 F53E10.5 -0.0930319080275464
AGIWUR19102
CB4856 CT V 2602153 2603063 WBGene00018762 F53E10.6 F53E10.6 -0.141876295255386
AGIWUR3618
CB4856 CT V 2608656 2615247 WBGene00000056 acr-17 F53E10.2 0.0547830376269793
AGIWUR21260
CB4856 CT V 2615790 2618554 WBGene00018758 F53E10.1 F53E10.1 0.281730104204788
AGIWUR8011;AGIWUR15797
CB4856 CT V 2618999 2620714 WBGene00005477 srh-271 W02H5.6 0.137939687705628
AGIWUR10433;AGIWUR18408;AGIWUR35984
CB4856 CT V 2638843 2646620 WBGene00020957 W02H5.2 W02H5.2 -0.0704979932975256
AGIWUR11330;AGIWUR17167;AGIWUR19063
CB4856 CT V 2647037 2652712 WBGene00020962 W02H5.8 W02H5.8 0.298343029129168 AGIWUR17071
CB4856 CT V 2653079 2662655 WBGene00020955 angl-1 W02G9.5 0.102143836464746 AGIWUR10994
CB4856 CT V 2662964 2665003 WBGene00003579 ndx-2 W02G9.1 -0.360720486186562 AGIWUR24002
CB4856 CT V 2666866 2670234 WBGene00020954 W02G9.4 W02G9.4 0.501245760261533 AGIWUR29410
CB4856 CT V 2671464 2688500 WBGene00020952 kel-8 W02G9.2 -0.0827636927979567
AGIWUR3574;AGIWUR4374;AGIWUR14073;AGIWUR18975;AGIWUR23190;AGIWUR25226;AGIWUR30569;AGIWUR37100
CB4856 CT V 2688461 2694430 WBGene00021564 Y45G12B.3 Y45G12B.3 0.0742179983245279
AGIWUR43901
CB4856 CT V 2694768 2698864 WBGene00021563 Y45G12B.2 Y45G12B.2 -0.176038469590208
AGIWUR18287;AGIWUR25218
CB4856 CT V 2698972 2707024 WBGene00021562 nuo-5 Y45G12B.1 0.0821519667898718
AGIWUR20241;AGIWUR25712;AGIWUR38658;AGIWUR42997
CB4856 CT V 2706781 2711599 WBGene00016048 C24B9.3 C24B9.3 -0.246520156508561
AGIWUR966;AGIWUR17192;AGIWUR41142;AGIWUR41995
CB4856 CT V 2712034 2713632 WBGene00016049 srt-28 C24B9.4 -0.201697000806481 AGIWUR19236
CB4856 CT V 2715274 2717000 WBGene00016047 srt-26 C24B9.2 -0.158919765393355 AGIWUR15766
CB4856 CT V 2717395 2719135 WBGene00016050 srt-29 C24B9.5 0.0649564755390486 AGIWUR16487
CB4856 CT V 2719696 2720863 WBGene00016051 srt-27 C24B9.6 0.101666141956684 AGIWUR9939
CB4856 CT V 2721290 2722655 WBGene00005217 srg-60 C24B9.15 0.0342414592664699
AGIWUR14062
CB4856 CT V 2722903 2725325 WBGene00005216 srg-59 C24B9.7 0.381287245106597 AGIWUR30333
CB4856 CT V 2725902 2727201 WBGene00006081 str-13 C24B9.8 0.142242450428917 AGIWUR5432
CB4856 CT V 2728536 2730243 WBGene00006099 str-33 C24B9.1 0.186079522191983 AGIWUR17470
CB4856 CT V 2730594 2731098 WBGene00016052 dod-3 C24B9.9 1.69646476625955 AGIWUR30647

CB4856	CT	V	2732177	2734460	WBGene00005214	srg-57	C24B9.14	-0.0892544645820266	AGIWUR28096
CB4856	CT	V	2735531	2738274	WBGene00005213	srg-56	C24B9.10	-0.431720557005776	AGIWUR39080
CB4856	CT	V	2740444	2744326	WBGene00005219	srg-62	C24B9.12	-0.572508476013203	AGIWUR2554
CB4856	CT	V	2745013	2746947	WBGene00005916	srx-25	C24B9.13	0.235257805196775	AGIWUR10481
CB4856	CT	V	2748152	2750106	WBGene00005915	srx-24	C24B9.16	0.0968259247290879	AGIWUR13684
CB4856	CT	V	2751256	2753107	WBGene00005917	srx-26	R05D8.4	0.196523196677067	AGIWUR33456
CB4856	CT	V	2753306	2755158	WBGene00005918	srx-27	R05D8.12	0.0849459287324983	AGIWUR5205;AGIWUR11166;AGIWUR30165
CB4856	CT	V	2755701	2757276	WBGene00005919	srx-28	R05D8.5	0.00881044243658646	AGIWUR31039
CB4856	CT	V	2758174	2759556	WBGene00005215	srg-58	R05D8.6	0.360642003077212	AGIWUR6399
CB4856	CT	V	2761825	2763208	WBGene00019885	R05D8.7	R05D8.7	0.507879409609895	AGIWUR20612
CB4856	CT	V	2763981	2765274	WBGene00000977	dhs-14	R05D8.8	-0.098641464811116	AGIWUR14412;AGIWUR30446
CB4856	CT	V	2766271	2767503	WBGene00019886	R05D8.9	R05D8.9	0.272726120379785	AGIWUR42273
CB4856	CT	V	2768419	2769854	WBGene00000978	dhs-15	R05D8.10	0.127100951375727	AGIWUR36965
CB4856	CT	V	2771860	2773519	WBGene00005279	srh-56	R05D8.3	0.0508024732007923	AGIWUR17184;AGIWUR31165;AGIWUR34838
CB4856	CT	V	2774342	2776258	WBGene00005606	srj-18	R05D8.2	0.0699779284354847	AGIWUR22116;AGIWUR30340;AGIWUR37772
CB4856	CT	V	2776442	2779233	WBGene00005605	srj-17	R05D8.1	-0.0558028186053283	AGIWUR18696;AGIWUR37375
CB4856	CT	V	2779384	2780845	WBGene00019887	R05D8.11	R05D8.11	0.0386946988791763	AGIWUR37072
CB4856	CT	V	2781943	2807934	WBGene00004130	ketn-1	F54E2.3	-0.086044010180454	AGIWUR1666;AGIWUR8340;AGIWUR14563;AGIWUR30694;AGIWUR35122;AGIWUR39274;AGIWUR40581
CB4856	CT	V	2810398	2812017	WBGene00018823	F54E2.1	F54E2.1	-0.724056294224891	AGIWUR29760
CB4856	CT	V	2812531	2813948	WBGene00268206	F54E2.9	F54E2.9	0.358079656738669	AGIWUR14121
CB4856	CT	V	2816883	2818217	WBGene00018826	srt-34	F54E2.6	-0.228469705513996	AGIWUR25520
CB4856	CT	V	2819581	2821668	WBGene00005259	srh-36	T03D3.4	-0.0151338093305071	AGIWUR3593;AGIWUR4361;AGIWUR7859
CB4856	CT	V	2823806	2826831	WBGene00020183	T03D3.5	T03D3.5	-0.295425320118245	AGIWUR3087
CB4856	CT	V	2832966	2834921	WBGene00005629	srj-50	T03D3.2	0.151637121719952	AGIWUR22775
CB4856	CT	V	2835411	2836601	WBGene00005627	srj-45	T03D3.6	0.0576746999061921	AGIWUR33727

CB4856	CT	V	2837634	2838805	WBGene00023335	T03D3.7	T03D3.7	-0.0409839393561677	AGIWUR15810
CB4856	CT	V	2839154	2840318	WBGene00023336	T03D3.8	T03D3.8	-0.159980465718754	AGIWUR29605
CB4856	CT	V	2841018	2842241	WBGene00023337	T03D3.9	T03D3.9	0.0488892706118782	AGIWUR19942;AGIWUR44223
CB4856	CT	V	2842946	2844240	WBGene00023338	T03D3.10	T03D3.10	-0.0289174995198973	AGIWUR12011;AGIWUR26145
CB4856	CT	V	2844563	2845871	WBGene00005626	srj-44	T03D3.11	0.384699542270153	AGIWUR10080
CB4856	CT	V	2846250	2848340	WBGene00005628	srj-49	T03D3.12	0.0123317385195758	AGIWUR8827
CB4856	CT	V	2849343	2852031	WBGene00020182	ugt-53	T03D3.1	-0.215554957688983	AGIWUR43938
CB4856	CT	V	2852425	2853605	WBGene00023339	T03D3.13	T03D3.13	0.0638477285161407	AGIWUR40784
CB4856	CT	V	2854393	2855573	WBGene00005635	srj-57	T03D3.14	0.0784940833211314	AGIWUR481
CB4856	CT	V	2856082	2857942	WBGene00005634	srj-55	Y45G12A.1	-0.266034865488831	AGIWUR42694
CB4856	CT	V	2858660	2859838	WBGene00005633	srj-54	F37B4.11	-0.0130245639894992	AGIWUR8532
CB4856	CT	V	2860130	2861605	WBGene00005476	srh-270	F37B4.5	0.127325796752364	AGIWUR30782
CB4856	CT	V	2863333	2865001	WBGene00005478	srh-272	F37B4.4	0.0262900927155068	AGIWUR6780
CB4856	CT	V	2865199	2867284	WBGene00005447	srh-240	F37B4.13	0.120732575932708	AGIWUR1223
CB4856	CT	V	2867792	2869603	WBGene00005448	srh-241	F37B4.6	-0.388507174840481	AGIWUR3234
CB4856	CT	V	2870427	2872106	WBGene00005449	srh-243	F37B4.3	-0.255620864849694	AGIWUR44846
CB4856	CT	V	2872465	2875600	WBGene00018138	folt-2	F37B4.7	-0.668591284075165	AGIWUR5700;AGIWUR9537;AGIWUR14965;AGIWUR15205;AGIWUR27435;AGIWUR30621;AGIWUR32404;AGIWUR43045
CB4856	CT	V	2875734	2877609	WBGene00018140	F37B4.10	F37B4.10	-0.19041513545889	AGIWUR38144
CB4856	CT	V	2877804	2879901	WBGene00006104	str-39	F37B4.12	0.277409703386535	AGIWUR16037
CB4856	CT	V	2881393	2883753	WBGene00002055	ifc-1	F37B4.2	0.0571372756654167	AGIWUR20904
CB4856	CT	V	2884711	2886425	WBGene00018139	sre-24	F37B4.9	-0.0556518916038256	AGIWUR25959
CB4856	CT	V	2887191	2888781	WBGene00005751	srw-4	F37B4.8	0.0335591408133808	AGIWUR17156
CB4856	CT	V	2889302	2890029	WBGene00044523	F37B4.14	F37B4.14	0.0972873138373873	AGIWUR7633;AGIWUR13078;AGIWUR13575;AGIWUR31735
CB4856	CT	V	2893276	2895584	WBGene00005344	srh-127	F37B4.1	0.217875855183724	AGIWUR1744
CB4856	CT	V	2896637	2898434	WBGene00016281	pals-32	C31B8.4	1.51885493586814	AGIWUR7768;AGIWUR16666;AGIWUR29442

CB4856	CT	V	2898436	2899967	WBGene00006111	str-46	C31B8.6	-0.0646157382174879	AGIWUR23220
CB4856	CT	V	2900448	2903355	WBGene00016282	C31B8.7	C31B8.7	-0.156342225368527	AGIWUR647
CB4856	CT	V	2905767	2909698	WBGene00016283	zmp-3	C31B8.8	-0.258004975796773	AGIWUR28295
CB4856	CT	V	2911082	2913001	WBGene00016284	C31B8.9	C31B8.9	0.01168858342617	AGIWUR31880
CB4856	CT	V	2914248	2916172	WBGene00005362	srh-146	C31B8.3	0.159211338108974	AGIWUR10530;AGIWUR20427;AGIWUR23935;AGIWUR23968;AGIWUR32765
CB4856	CT	V	2916523	2918034	WBGene00016280	C31B8.2	C31B8.2	0.124372730174795	AGIWUR4147;AGIWUR9281;AGIWUR9646;AGIWUR12282;AGIWUR15923;AGIWUR18521;AGIWUR19038;AGIWUR43160
CB4856	CT	V	2918830	2920008	WBGene00005630	srj-51	C31B8.10	0.0105973377191626	AGIWUR20464;AGIWUR28135;AGIWUR28956;AGIWUR32501;AGIWUR33894
CB4856	CT	V	2920600	2922694	WBGene00005261	srh-38	C31B8.11	0.0560829463299779	AGIWUR43647
CB4856	CT	V	2925193	2927722	WBGene00016285	C31B8.12	C31B8.12	-0.117553624351808	AGIWUR15874
CB4856	CT	V	2930053	2931170	WBGene00044602	C31B8.16	C31B8.16	-0.143266480458645	AGIWUR3699;AGIWUR42580
CB4856	CT	V	2931552	2932988	WBGene00016279	C31B8.1	C31B8.1	-0.055964103692169	AGIWUR38619
CB4856	CT	V	2933143	2934175	WBGene00005453	srh-247	C31B8.13	0.0597617959503456	AGIWUR18435
CB4856	CT	V	2935178	2936199	WBGene00005455	srh-249	C31B8.14	0.31866713230993	AGIWUR23607
CB4856	CT	V	2937311	2938597	WBGene00005888	srw-141	H27D07.2	0.133584272179507	AGIWUR4724;AGIWUR39410
CB4856	CT	V	2939603	2941505	WBGene00005890	srw-143	H27D07.3	-0.00572361646529451	AGIWUR2779
CB4856	CT	V	2942571	2943959	WBGene00005884	srw-137	H27D07.4	-0.145685090936	AGIWUR8026
CB4856	CT	V	2944379	2945682	WBGene00005308	srh-87	H27D07.6	0.0175596039861112	AGIWUR13211
CB4856	CT	V	2946813	2948416	WBGene00005875	srw-128	H27D07.1	0.174241163031309	AGIWUR26758
CB4856	CT	V	2950712	2952202	WBGene00005869	srw-122	H27D07.5	-0.0709501251869025	AGIWUR25472
CB4856	CT	V	2953193	2954289	WBGene00005873	srw-126	H05B21.1	-0.0137684527201269	AGIWUR1897;AGIWUR37067
CB4856	CT	V	2954404	2955442	WBGene00005454	srh-248	H05B21.2	-0.0838850268492237	AGIWUR39328
CB4856	CT	V	2957016	2958302	WBGene00005889	srw-142	H05B21.3	-0.146433014888894	AGIWUR29592
CB4856	CT	V	2961632	2963060	WBGene00005885	srw-138	C44C3.3	-0.0274581251453876	AGIWUR39049

CB4856	CT	V	2965422	2966702	WBGene00005863	srw-116	C44C3.10	0.140749901601881	AGIWUR13677;AGIWUR28717
CB4856	CT	V	2969349	2971164	WBGene00005307	srh-86	C44C3.4	0.0607254588115382	AGIWUR9864;AGIWUR24809
CB4856	CT	V	2971949	2973485	WBGene00005876	srw-129	C44C3.2	0.145788795121435	AGIWUR12878
CB4856	CT	V	2974029	2975385	WBGene00005870	srw-123	C44C3.11	0.0508934236778877	AGIWUR33523
CB4856	CT	V	2976587	2977886	WBGene00005871	srw-124	C44C3.1	0.0329306561471423	AGIWUR15851
CB4856	CT	V	2978760	2980078	WBGene00005865	srw-118	C44C3.5	-0.0513624777036084	AGIWUR29350
CB4856	CT	V	2982152	2983451	WBGene00005864	srw-117	C44C3.6	-0.0162478056630757	AGIWUR23329
CB4856	CT	V	2984077	2985409	WBGene00005886	srw-139	C44C3.7	-0.00146186989591746	AGIWUR23695;AGIWUR44571
CB4856	CT	V	2986192	2987507	WBGene00005306	srh-85	C44C3.8	0.163838113442241	AGIWUR31725
CB4856	CT	V	2988460	2990091	WBGene00005305	srh-84	K12D9.2	0.0118275912050301	AGIWUR2561;AGIWUR25386;AGIWUR32591
CB4856	CT	V	2990313	2991585	WBGene00005868	srw-121	K12D9.3	0.101932872966133	AGIWUR8427
CB4856	CT	V	2992622	2993916	WBGene00005862	srw-115	K12D9.4	0.23906591845598	AGIWUR18613
CB4856	CT	V	2994899	2996205	WBGene00005867	srw-120	K12D9.5	0.0343916621586473	AGIWUR28704
CB4856	CT	V	2997092	2997917	WBGene00005872	srw-125	K12D9.6	0.0556326848480175	AGIWUR18143
CB4856	CT	V	2998542	2999836	WBGene00005866	srw-119	K12D9.7	0.199920629075999	AGIWUR20695
CB4856	CT	V	3000426	3002016	WBGene00005861	srw-114	K12D9.8	-0.102270627073309	AGIWUR11996;AGIWUR30855
CB4856	CT	V	3004141	3006064	WBGene00005859	srw-112	K12D9.9	0.0270363025932667	AGIWUR21370
CB4856	CT	V	3006728	3008123	WBGene00005878	srw-131	K12D9.13	-0.103023375804805	AGIWUR23697;AGIWUR25350
CB4856	CT	V	3008907	3010568	WBGene00005883	srw-136	K12D9.10	0.0594074246953653	AGIWUR6161
CB4856	CT	V	3010927	3011671	WBGene00005882	srw-135	K12D9.11	-0.667290808507389	AGIWUR15455;AGIWUR35172
CB4856	CT	V	3014485	3016034	WBGene00019675	K12D9.1	K12D9.1	0.195333175398879	AGIWUR7110
CB4856	CT	V	3018361	3021406	WBGene00077758	irld-12	K12D9.14	0.173485101292629	AGIWUR16081
CB4856	CT	V	3024520	3027931	WBGene00019105	asp-8	F59D6.3	0.176285472228848	AGIWUR35580
CB4856	CT	V	3028294	3029583	WBGene00019104	asp-7	F59D6.2	-0.432130617362883	AGIWUR31111
CB4856	CT	V	3029655	3032443	WBGene00019106	irld-38	F59D6.4	0.220143050533664	AGIWUR24135
CB4856	CT	V	3032578	3034336	WBGene00005755	srw-8	F59D6.5	-0.0439679326451508	AGIWUR2680
CB4856	CT	V	3035130	3037689	WBGene00019107	irld-10	F59D6.6	-0.279913452654983	AGIWUR22111
CB4856	CT	V	3037812	3039382	WBGene00019108	chpf-2	F59D6.7	0.26268851736204	AGIWUR34475
CB4856	CT	V	3045144	3052149	WBGene00019103	F59D6.1	F59D6.1	0.185232873640462	AGIWUR3414

CB4856	CT	V	3054553	3055667	WBGene00016855	C50H11.8	C50H11.8	-0.146964463024789
AGIWUR27749
CB4856	CT	V	3057699	3059096	WBGene00006271	str-244	C50H11.9	0.0019010614276409
AGIWUR750
CB4856	CT	V	3059640	3062178	WBGene00016856	srt-15	C50H11.10	0.115954807883421
AGIWUR24385
CB4856	CT	V	3063160	3064518	WBGene00016857	srt-16	C50H11.11	0.241925733201827
AGIWUR21675
CB4856	CT	V	3066228	3067497	WBGene00005400	srh-185	C50H11.7	-0.0411087947907296
AGIWUR8079
CB4856	CT	V	3068135	3069201	WBGene00006105	str-40	C50H11.12	0.258931145344957
AGIWUR10195
CB4856	CT	V	3069898	3071648	WBGene00016858	C50H11.13	C50H11.13	0.0463877652931357
AGIWUR5643;AGIWUR9917;AGIWUR18242;AGIWUR24776
CB4856	CT	V	3075407	3077173	WBGene00016854	srt-10	C50H11.6	-0.0200148578071069
AGIWUR36442
CB4856	CT	V	3078072	3079300	WBGene00016853	srt-9	C50H11.5	-0.0919280856110154
AGIWUR7906
CB4856	CT	V	3079449	3080892	WBGene00016859	srt-5	C50H11.14	0.00437662183030341
AGIWUR35025
CB4856	CT	V	3081771	3083027	WBGene00016852	srt-7	C50H11.4	-0.386592869314893
AGIWUR42233
CB4856	CT	V	3083645	3085127	WBGene00016851	srt-71	C50H11.3	-0.175270547370663
AGIWUR15342;AGIWUR18706
CB4856	CT	V	3086560	3087986	WBGene00016850	srt-8	C50H11.2	-0.125962089855288
AGIWUR25845
CB4856	CT	V	3089074	3091351	WBGene00016849	acs-21	C50H11.1	-0.133805767260776
AGIWUR12607;AGIWUR13127;AGIWUR34615
CB4856	CT	V	3091815	3094501	WBGene00016860	cyp-33C9	C50H11.15	0.019508299512708
AGIWUR35734
CB4856	CT	V	3095090	3096177	WBGene00006132	str-70	C50H11.16	0.198091542499109
AGIWUR12740;AGIWUR36852
CB4856	CT	V	3097130	3098688	WBGene00016861	C50H11.17	C50H11.17	-0.10747354261014
AGIWUR26203
CB4856	CT	V	3103128	3104234	WBGene00006128	str-66	Y73C8C.5	0.0145455183345193
AGIWUR21799
CB4856	CT	V	3104790	3105910	WBGene00006131	str-69	Y73C8C.11	-5.80681446322566
AGIWUR16419
CB4856	CT	V	3106228	3107550	WBGene00006130	str-68	Y73C8C.6	-0.00461748376674187

AGIWUR38915

CB4856	CT	V	3107792	3109664	WBGene00022264	sdz-34	Y73C8C.7	-0.133455912928083	AGIWUR9429
CB4856	CT	V	3110089	3113851	WBGene00022265	Y73C8C.8	Y73C8C.8	0.129092385150642	AGIWUR8522
CB4856	CT	V	3115303	3118128	WBGene00022262	Y73C8C.3	Y73C8C.3	0.125352236693422	AGIWUR20824
CB4856	CT	V	3118377	3120323	WBGene00022261	clec-210	Y73C8C.2	-0.247464800369547	AGIWUR326
CB4856	CT	V	3122969	3124222	WBGene00006269	str-242	Y73C8C.1	0.0619918934434285	AGIWUR33082;AGIWUR37993
CB4856	CT	V	3125212	3125469	WBGene00044524	Y73C8C.12	Y73C8C.12	0.0535319749599221	AGIWUR1216;AGIWUR12325;AGIWUR26265;AGIWUR28602;AGIWUR39102
CB4856	CT	V	3125566	3127770	WBGene00022267	Y73C8C.10	Y73C8C.10	-0.0894613852821475	AGIWUR10724;AGIWUR12344;AGIWUR40970
CB4856	CT	V	3129125	3133406	WBGene00017174	tat-6	F02C9.3	0.0184421253040874	AGIWUR38702;AGIWUR40033;AGIWUR44141
CB4856	CT	V	3133705	3137382	WBGene00017173	F02C9.2	F02C9.2	-0.0273409106230398	AGIWUR2321;AGIWUR4262;AGIWUR29080
CB4856	CT	V	3137431	3138588	WBGene00017175	irld-3	F02C9.4	-0.251940523469028	AGIWUR39610
CB4856	CT	V	3138703	3139090	WBGene00017172	F02C9.1	F02C9.1	-0.130774889511411	AGIWUR34685
CB4856	CT	V	3139931	3141290	WBGene00016475	srab-9	C36C5.6	-0.0684580622815538	AGIWUR11504;AGIWUR26115;AGIWUR32463
CB4856	CT	V	3143094	3144597	WBGene00016476	srab-10	C36C5.7	-0.06259799012218	AGIWUR23471
CB4856	CT	V	3145203	3147032	WBGene00016477	srab-11	C36C5.8	0.151107471046921	AGIWUR35579
CB4856	CT	V	3148215	3149489	WBGene00016478	srt-72	C36C5.9	-0.0248515175836221	AGIWUR19941
CB4856	CT	V	3150488	3151962	WBGene00016479	srab-6	C36C5.10	0.00371321425722382	AGIWUR37693
CB4856	CT	V	3153485	3155172	WBGene00016480	srab-7	C36C5.11	0.00330133246571584	AGIWUR42020
CB4856	CT	V	3157077	3157870	WBGene00016474	C36C5.5	C36C5.5	-0.198291850593233	AGIWUR24051
CB4856	CT	V	3158406	3159165	WBGene00016481	C36C5.12	C36C5.12	-0.177535523045485	AGIWUR4426
CB4856	CT	V	3166560	3167268	WBGene00016473	C36C5.4	C36C5.4	0.345619050573467	AGIWUR29268
CB4856	CT	V	3167968	3168750	WBGene00016484	C36C5.15	C36C5.15	-0.555539922672764	AGIWUR21646
CB4856	CT	V	3170138	3171408	WBGene00016472	srbc-6	C36C5.3	0.18278372493182	AGIWUR19765
CB4856	CT	V	3176461	3177978	WBGene00016471	srab-8	C36C5.2	0.144462184239499	AGIWUR31318
CB4856	CT	V	3179527	3181064	WBGene00016470	srab-5	C36C5.1	-0.816572104919169	AGIWUR38960

CB4856	CT	V	3188350	3189841	WBGene00002246	lag-2	Y73C8B.4	0.224561602406692	AGIWUR4259
CB4856	CT	V	3191302	3193233	WBGene00022260	Y73C8B.3	Y73C8B.3	-0.0564123151467655	AGIWUR26298
CB4856	CT	V	3193394	3195002	WBGene00022259	Y73C8B.2	Y73C8B.2	0.0282559005635505	AGIWUR43261
CB4856	CT	V	3197268	3198333	WBGene00044525	Y73C8B.5	Y73C8B.5	0.00816280094100286	AGIWUR5299;AGIWUR26367;AGIWUR32194;AGIWUR34388;AGIWUR40466
CB4856	CT	V	3210189	3223218	WBGene00000168	apx-1	K08D9.3	-0.127230863047632	AGIWUR37888
CB4856	CT	V	3223612	3227058	WBGene00019526	K08D9.5	K08D9.5	0.0301924983185233	AGIWUR6970;AGIWUR8617;AGIWUR19552;AGIWUR20987
CB4856	CT	V	3227796	3230128	WBGene00019524	K08D9.2	K08D9.2	-0.219543798517775	AGIWUR21368
CB4856	CT	V	3231398	3234987	WBGene00019527	K08D9.6	K08D9.6	-0.331169118299369	AGIWUR23029
CB4856	CT	V	3236874	3240436	WBGene00006084	str-16	Y73C8A.1	-0.0221319128088489	AGIWUR24411;AGIWUR28177
CB4856	CT	V	3241424	3242636	WBGene00005599	srj-11	T28A11.12	0.0216074380038105	AGIWUR41289
CB4856	CT	V	3243287	3244495	WBGene00001771	gst-23	T28A11.11	0.210232030343482	AGIWUR10803;AGIWUR16486;AGIWUR36289;AGIWUR37107
CB4856	CT	V	3245003	3246722	WBGene00005595	srj-7	T28A11.10	-0.0306508684222759	AGIWUR19781
CB4856	CT	V	3247815	3249140	WBGene00005596	srj-8	T28A11.9	0.178240954245155	AGIWUR41430
CB4856	CT	V	3249743	3250205	WBGene00020875	T28A11.13	T28A11.13	0.139385750112817	AGIWUR12351
CB4856	CT	V	3253469	3254590	WBGene00006123	str-58	T28A11.8	0.021824836454539	AGIWUR28675;AGIWUR34848
CB4856	CT	V	3254602	3256151	WBGene00020877	srt-63	T28A11.15	0.197787657306347	AGIWUR20639
CB4856	CT	V	3257577	3258289	WBGene00044526	T28A11.22	T28A11.22	0.357660398436932	AGIWUR8845;AGIWUR24114;AGIWUR31464;AGIWUR39416
CB4856	CT	V	3258372	3259218	WBGene00020878	T28A11.16	T28A11.16	0.318036511098606	AGIWUR33578
CB4856	CT	V	3260375	3262082	WBGene00020874	srbc-5	T28A11.7	0.188599303252003	AGIWUR12723
CB4856	CT	V	3262571	3263235	WBGene00020873	T28A11.6	T28A11.6	-0.448096070859278	AGIWUR7694;AGIWUR29877;AGIWUR32742;AGIWUR38034;AGIWUR40987;AGIWUR44557
CB4856	CT	V	3266828	3268050	WBGene00020880	T28A11.18	T28A11.18	0.652668772078446	AGIWUR38778

CB4856	CT	V	3268809	3269510	WBGene00020881	T28A11.19	T28A11.19	1.94718707780296	AGIWUR7073
CB4856	CT	V	3270553	3271242	WBGene00020872	T28A11.5	T28A11.5	0.0750151657334768	AGIWUR18249;AGIWUR35583
CB4856	CT	V	3271395	3273256	WBGene00020882	T28A11.20	T28A11.20	1.36368395447741	AGIWUR17791
CB4856	CT	V	3274054	3274374	WBGene00020871	T28A11.4	T28A11.4	3.17830925242977	AGIWUR24975
CB4856	CT	V	3275333	3276110	WBGene00020870	T28A11.3	T28A11.3	0.128290049429276	AGIWUR35661
CB4856	CT	V	3276956	3278147	WBGene00020883	fbxa-64	T28A11.21	-0.155063320752394	AGIWUR21947
CB4856	CT	V	3280379	3281169	WBGene00020869	T28A11.2	T28A11.2	-0.177924301943506	AGIWUR45206
CB4856	CT	V	3281828	3282704	WBGene00269355	T28A11.25	T28A11.25	1.09321922530563	AGIWUR7972
CB4856	CT	V	3283257	3284930	WBGene00006127	str-64	T28A11.1	-0.0146628400801947	AGIWUR3736;AGIWUR6694
CB4856	CT	V	3286432	3287460	WBGene00018057	F35F10.7	F35F10.7	-0.33425631969827	AGIWUR4804
CB4856	CT	V	3287995	3289385	WBGene00018056	F35F10.6	F35F10.6	-0.298217537653054	AGIWUR20667
CB4856	CT	V	3289635	3293435	WBGene00018054	F35F10.4	F35F10.4	-0.0829251774876019	AGIWUR4521;AGIWUR5943;AGIWUR14466;AGIWUR23650;AGIWUR36797
CB4856	CT	V	3296108	3297864	WBGene00006013	srx-122	F35F10.8	-0.0500011083684306	AGIWUR6060
CB4856	CT	V	3298885	3301998	WBGene00018058	srbc-1	F35F10.9	-0.0278389730505717	AGIWUR12295
CB4856	CT	V	3302415	3307363	WBGene00018059	F35F10.10	F35F10.10	-0.125878696235307	AGIWUR44417
CB4856	CT	V	3308469	3309689	WBGene00018053	srbc-3	F35F10.2	-0.499710787458456	AGIWUR20747
CB4856	CT	V	3310123	3312117	WBGene00018060	arrd-18	F35F10.11	-0.467112874839183	AGIWUR34169
CB4856	CT	V	3313286	3315516	WBGene00018061	arrd-19	F35F10.12	-0.37948391446796	AGIWUR3862;AGIWUR22827
CB4856	CT	V	3320720	3324279	WBGene00015882	C17B7.8	C17B7.8	0.178714705853929	AGIWUR4566
CB4856	CT	V	3330915	3332113	WBGene00015883	C17B7.9	C17B7.9	-0.207295312180143	AGIWUR42983
CB4856	CT	V	3333635	3339070	WBGene00015879	C17B7.5	C17B7.5	-0.176212427050399	AGIWUR30643

CB4856	CT	V	3339348	3340092	WBGene00015878	C17B7.4	C17B7.4	0.0749622440900906	AGIWUR14925;AGIWUR28398
CB4856	CT	V	3340179	3342006	WBGene00015884	C17B7.10	C17B7.10	0.751041462580108	AGIWUR25791
CB4856	CT	V	3345307	3346484	WBGene00015885	fbxa-65	C17B7.11	-0.605194223630284	AGIWUR19694
CB4856	CT	V	3350177	3350967	WBGene00268213	C17B7.15	C17B7.15	-0.0350937902702571	AGIWUR5840
CB4856	CT	V	3354667	3355513	WBGene00015436	C04E12.6	C04E12.6	-0.0304527555384251	AGIWUR22660
CB4856	CT	V	3355848	3357193	WBGene00015437	scrm-3	C04E12.7	-0.0828849630301258	AGIWUR284;AGIWUR27159;AGIWUR40065
CB4856	CT	V	3358567	3361093	WBGene00015435	C04E12.5	C04E12.5	-0.058292098260897	AGIWUR14815
CB4856	CT	V	3362775	3368340	WBGene00015434	C04E12.4	C04E12.4	-0.230427695580671	AGIWUR40921
CB4856	CT	V	3370942	3372773	WBGene00006012	srx-121	C04E12.8	-0.169321598191596	AGIWUR41071
CB4856	CT	V	3374362	3377073	WBGene00015438	srbc-2	C04E12.9	-0.00397695777262497	AGIWUR9314;AGIWUR10084;AGIWUR15107;AGIWUR26825
CB4856	CT	V	3377536	3382850	WBGene00015439	C04E12.10	C04E12.10	-0.304810581511644	AGIWUR28568
CB4856	CT	V	3385695	3388156	WBGene00015440	arrd-20	C04E12.11	-0.193166675435107	AGIWUR9441
CB4856	CT	V	3392111	3393296	WBGene00020619	T20D4.13	T20D4.13	-0.240906995203463	AGIWUR32543;AGIWUR44356;AGIWUR44364
CB4856	CT	V	3393478	3394688	WBGene00020620	T20D4.15	T20D4.15	0.105395388502874	AGIWUR13888;AGIWUR26446;AGIWUR32049
CB4856	CT	V	3395275	3395966	WBGene00020622	T20D4.17	T20D4.17	-0.015592910819147	AGIWUR9905
CB4856	CT	V	3397065	3397800	WBGene00020618	T20D4.12	T20D4.12	0.492741738007744	AGIWUR10809
CB4856	CT	V	3398690	3399640	WBGene00020617	T20D4.11	T20D4.11	1.4784961955391	AGIWUR32038
CB4856	CT	V	3400252	3401063	WBGene00020616	T20D4.10	T20D4.10	-0.347954110473086	AGIWUR23484
CB4856	CT	V	3401645	3402356	WBGene00044718	T20D4.20	T20D4.20	0.15009849046857	AGIWUR29094
CB4856	CT	V	3405709	3407893	WBGene00020614	T20D4.8	T20D4.8	-0.196551546057442	AGIWUR34715

CB4856	CT	V	3408912	3409519	WBGene00020613	T20D4.7	T20D4.7	-0.0529271721472204	AGIWUR41765
CB4856	CT	V	3410520	3412858	WBGene00020612	arrd-22	T20D4.6	-0.563373077569679	AGIWUR24818
CB4856	CT	V	3413344	3417770	WBGene00020611	T20D4.5	T20D4.5	-0.424446495095726	AGIWUR18362;AGIWUR36984
CB4856	CT	V	3418263	3422733	WBGene00020609	T20D4.3	T20D4.3	-0.103383802293757	AGIWUR14715;AGIWUR23453
CB4856	CT	V	3422949	3424441	WBGene00020623	srab-21	T20D4.18	-0.0809157272253769	AGIWUR23103
CB4856	CT	V	3426376	3427733	WBGene00020608	srab-22	T20D4.2	0.0472122502095296	AGIWUR42868
CB4856	CT	V	3428999	3430408	WBGene00020607	srab-20	T20D4.1	-0.304180208313987	AGIWUR3585
CB4856	CT	V	3430669	3431446	WBGene00020624	T20D4.19	T20D4.19	0.0238161161820187	AGIWUR15291
CB4856	CT	V	3432259	3433658	WBGene00005597	srj-9	T20C4.1	-0.915772233569072	AGIWUR11391
CB4856	CT	V	3441504	3444877	WBGene00000478	cfz-2	F27E11.3	-0.143614136372468	AGIWUR757;AGIWUR7380;AGIWUR10239;AGIWUR15707;AGIWUR27031;AGIWUR28201;AGIWUR30282
CB4856	CT	V	3459740	3463071	WBGene00017868	slc-28.2	F27E11.2	0.482606709034837	AGIWUR6289
CB4856	CT	V	3463814	3466150	WBGene00017867	slc-28.1	F27E11.1	0.540705024406215	AGIWUR29163
CB4856	CT	V	3467453	3468545	WBGene00001781	gst-33	C02A12.1	0.270985653240094	AGIWUR37263
CB4856	CT	V	3469445	3470882	WBGene00015315	srbc-29	C02A12.2	-0.329196037304726	AGIWUR20182
CB4856	CT	V	3471870	3475121	WBGene00044088	oac-2	C02A12.8	0.121332895872224	AGIWUR9707;AGIWUR37879
CB4856	CT	V	3476405	3477596	WBGene00044089	srbc-31	C02A12.9	-0.013321765918378	AGIWUR25970
CB4856	CT	V	3478031	3480446	WBGene00015316	srbc-30	C02A12.3	0.148577579383863	AGIWUR22138;AGIWUR22731
CB4856	CT	V	3481421	3482600	WBGene00003096	lys-7	C02A12.4	0.64722502517715	AGIWUR21327
CB4856	CT	V	3483091	3484781	WBGene00044090	srbc-33	C02A12.10	-0.473940655608833	AGIWUR15981
CB4856	CT	V	3485549	3486957	WBGene00015317	srbc-32	C02A12.5	-0.112460623233296	AGIWUR13444;AGIWUR26033
CB4856	CT	V	3489228	3490535	WBGene00015318	srbc-36	C02A12.6	-0.0201092588255172	AGIWUR44918
CB4856	CT	V	3491361	3492598	WBGene00015575	srbc-35	C07G3.7	0.0361261863864879	AGIWUR14490
CB4856	CT	V	3493637	3496943	WBGene00269378	C07G3.15	C07G3.15	0.169374349954055	

AGIWUR44418
CB4856	CT	V	3502307 3503077 WBGene00015578	C07G3.10	C07G3.10	0.296641278764505
AGIWUR38399
CB4856	CT	V	3503130 3506352 WBGene00015577	ugt-64	C07G3.9 -0.235267928654476
AGIWUR4206;AGIWUR16194;AGIWUR27047
CB4856	CT	V	3507822 3509251 WBGene00006256	str-225 C07G3.6 0.0438353376673248	AGIWUR1173
CB4856	CT	V	3510178 3512038 WBGene00006259	str-228 C07G3.5 -0.363613532056281	AGIWUR27547
CB4856	CT	V	3512681 3514343 WBGene00006257	str-226 C07G3.4 -0.183550841620276	AGIWUR29424
CB4856	CT	V	3518189 3519801 WBGene00006258	str-227 C07G3.3 -0.212478833376794	AGIWUR20857
CB4856	CT	V	3522496 3523373 WBGene00044119	srh-191 C07G3.11	0.213118816797782
AGIWUR3466;AGIWUR20550
CB4856	CT	V	3523587 3524575 WBGene00015574	irg-1	C07G3.2 -0.343545847422865
AGIWUR3896;AGIWUR15501;AGIWUR26755;AGIWUR27297;AGIWUR35590;AGIWUR39514
CB4856	CT	V	3525274 3526178 WBGene00020747	T24A6.7 T24A6.7 -0.0278339757301529	AGIWUR2091
CB4856	CT	V	3527182 3530413 WBGene00006286	str-260 T24A6.6 0.0214935838930632	AGIWUR17196
CB4856	CT	V	3530703 3532380 WBGene00020746	srbc-66 T24A6.5 -0.020609137114965	AGIWUR11780
CB4856	CT	V	3533003 3535235 WBGene00020748	nhr-221 T24A6.8 0.286832758127475	AGIWUR27511
CB4856	CT	V	3536196 3538407 WBGene00003683	nhr-93	T24A6.9 -0.166827875074941
AGIWUR14893;AGIWUR35485
CB4856	CT	V	3538929 3540329 WBGene00020749	srbc-67 T24A6.10	0.0910885807527322
AGIWUR29858
CB4856	CT	V	3541586 3543444 WBGene00020750	nhr-222 T24A6.11	-0.0928008455181342
AGIWUR45167
CB4856	CT	V	3544081 3544969 WBGene00020751	srbc-69 T24A6.12	-0.0139732723565116
AGIWUR18305
CB4856	CT	V	3545677 3546617 WBGene00020752	srbc-68 T24A6.13	0.0715844210079322
AGIWUR22134
CB4856	CT	V	3546969 3549685 WBGene00005877	srw-130 T24A6.14	0.136532967847461
AGIWUR40579
CB4856	CT	V	3549853 3551269 WBGene00005533	sri-21	T24A6.4 -0.0637457393286844	AGIWUR24721
CB4856	CT	V	3551346 3551865 WBGene00001737	grl-28	T24A6.15	-0.354052650900666
AGIWUR25251
CB4856	CT	V	3553134 3554568 WBGene00020756	T24A6.20	T24A6.20	-0.345344672791426
AGIWUR993;AGIWUR6754;AGIWUR17149;AGIWUR29084
CB4856	CT	V	3554611 3555236 WBGene00001738	grl-29	T24A6.18	0.13595261405937
AGIWUR36854
CB4856	CT	V	3556286 3556804 WBGene00001739	grl-30	T24A6.3 -0.0805637086894766
AGIWUR11277;AGIWUR13308;AGIWUR16687;AGIWUR26402;AGIWUR30986;AGIWUR32654;AGIWUR40692;AGIWUR44446
CB4856	CT	V	3557206 3557778 WBGene00020755	grl-31	T24A6.19	-0.234533520644167

AGIWUR44762
CB4856	CT	V	3575055 3575874 WBGene00001736	grl-27	F40C5.3 -0.643921113074565	AGIWUR25823
CB4856	CT	V	3579023 3585042 WBGene00020696	T22F3.3 T22F3.3 0.0496310192869522
AGIWUR8623;AGIWUR10935
CB4856	CT	V	3585313 3586064 WBGene00004422	rpl-11.1	T22F3.4 -0.0234395545986352
AGIWUR6406;AGIWUR22697;AGIWUR26264
CB4856	CT	V	3586639 3589015 WBGene00020695	T22F3.2 T22F3.2 0.0124930884021985	AGIWUR7830
CB4856	CT	V	3589029 3592199 WBGene00044705	T22F3.12	T22F3.12	0.395807090701139
AGIWUR15774;AGIWUR32295
CB4856	CT	V	3592768 3594973 WBGene00005423	srh-213 T22F3.5 -0.0107345905016247	AGIWUR3939
CB4856	CT	V	3595191 3597152 WBGene00005422	srh-212 T22F3.6 0.197148858356845	AGIWUR13236
CB4856	CT	V	3597753 3601021 WBGene00020697	T22F3.7 T22F3.7 0.108639647358963	AGIWUR44509
CB4856	CT	V	3602052 3605425 WBGene00020698	T22F3.8 T22F3.8 1.59631391982166	AGIWUR21890
CB4856	CT	V	3606818 3610927 WBGene00020699	T22F3.10	T22F3.10	1.08927465183934
AGIWUR38785
CB4856	CT	V	3612173 3614551 WBGene00020700	T22F3.11	T22F3.11	3.77110473248091
AGIWUR707;AGIWUR22769
CB4856	CT	V	3622382 3623698 WBGene00005985	srx-94	F59B1.5 -0.332704650130212	AGIWUR22337
CB4856	CT	V	3625022 3627044 WBGene00019098	F59B1.4 F59B1.4 0.0130983128441713	AGIWUR36888
CB4856	CT	V	3629473 3631691 WBGene00019099	F59B1.6 F59B1.6 -0.466746927986065	AGIWUR21295
CB4856	CT	V	3633341 3634683 WBGene00019097	F59B1.2 F59B1.2 0.0341675191454502
AGIWUR6873;AGIWUR19168;AGIWUR41274;AGIWUR41970;AGIWUR43406
CB4856	CT	V	3634688 3637173 WBGene00005932	srx-41	F59B1.7 0.330550585920765	AGIWUR40658
CB4856	CT	V	3641858 3644243 WBGene00019100	F59B1.8 F59B1.8 -0.25429779502662	AGIWUR25579
CB4856	CT	V	3644745 3647954 WBGene00019101	F59B1.10	F59B1.10	-0.383321952128588
AGIWUR11522
CB4856	CT	V	3649773 3651457 WBGene00006165	str-114 F59B1.1 0.141672660160483	AGIWUR26857
CB4856	CT	V	3651686 3659499 WBGene00000062	acr-23	F59B1.9 -0.132169115648979	AGIWUR24298
CB4856	CT	V	3664871 3666987 WBGene00006164	str-113 Y39H10B.1	-0.331138436536845
AGIWUR42664
CB4856	CT	V	3668728 3670631 WBGene00044527	Y39H10B.2	Y39H10B.2	-0.379518340031707
AGIWUR1568;AGIWUR10702
CB4856	CT	V	3673380 3676632 WBGene00021081	pals-33 W08A12.4	2.59812804494683
AGIWUR25982
CB4856	CT	V	3682853 3686474 WBGene00021080	W08A12.3	W08A12.3	-0.3034351037037
AGIWUR23717
CB4856	CT	V	3687110 3701864 WBGene00021078	unc-132 W08A12.1	0.117187863420702
AGIWUR10957
CB4856	CT	V	3704988 3712796 WBGene00003025	lin-40	T27C4.4 0.233341103086545

AGIWUR6346;AGIWUR11359;AGIWUR19273;AGIWUR20650;AGIWUR24147;AGIWUR30880;AGIWUR43792
CB4856 CT V 3712779 3715969 WBGene00020854 T27C4.1 T27C4.1 0.47359859984354
AGIWUR5398;AGIWUR38936;AGIWUR42805;AGIWUR44078
CB4856 CT V 3725072 3726775 WBGene00006096 str-30 T27C4.3 0.0139662401859137 AGIWUR43466
CB4856 CT V 3727482 3739398 WBGene00020855 hpo-36 T27C4.2 0.0424404795408737
AGIWUR10356;AGIWUR40347;AGIWUR42612
CB4856 CT V 3743576 3749401 WBGene00003479 mtm-9 Y39H10A.3 -0.165185884945291
AGIWUR25442;AGIWUR36850;AGIWUR39616
CB4856 CT V 3749494 3757074 WBGene00021482 Y39H10A.6 Y39H10A.6 0.123610237152175
AGIWUR15606
CB4856 CT V 3757047 3759469 WBGene00000498 chk-1 Y39H10A.7 -0.1929498144964
AGIWUR1558;AGIWUR23006;AGIWUR31592;AGIWUR32015;AGIWUR42878
CB4856 CT V 3761139 3766306 WBGene00021479 oac-55 Y39H10A.2 -0.219374970956791
AGIWUR21286
CB4856 CT V 3767204 3767734 WBGene00021478 Y39H10A.1 Y39H10A.1 1.60403875864315
AGIWUR30517
CB4856 CT V 3768570 3769776 WBGene00019964 R08E5.4 R08E5.4 -0.093972278417878 AGIWUR19614
CB4856 CT V 3769801 3771518 WBGene00019961 R08E5.1 R08E5.1 -0.189476752660473
AGIWUR22836;AGIWUR35303;AGIWUR37563
CB4856 CT V 3772021 3775546 WBGene00019963 R08E5.3 R08E5.3 -0.487084656509298 AGIWUR19259
CB4856 CT V 3774533 3776136 WBGene00019962 cysl-3 R08E5.2 -0.279754652863989
AGIWUR7833;AGIWUR18152;AGIWUR20551;AGIWUR20799
CB4856 CT V 3777553 3784213 WBGene00019965 R08F11.1 R08F11.1 -0.293898797015409
AGIWUR4405
CB4856 CT V 3790133 3793766 WBGene00019970 R08F11.7 R08F11.7 -0.0173311452726903
AGIWUR24390
CB4856 CT V 3794733 3798587 WBGene00019969 fpn-1.3 R08F11.6 -0.152494317154488
AGIWUR18782
CB4856 CT V 3798605 3800478 WBGene00019966 srt-30 R08F11.2 -0.36512359880243
AGIWUR24318
CB4856 CT V 3800782 3803531 WBGene00019967 cyp-33C8 R08F11.3 -0.276548366721173
AGIWUR2301
CB4856 CT V 3803845 3805531 WBGene00019968 R08F11.4 R08F11.4 -0.186270832307668
AGIWUR3076
CB4856 CT V 3806261 3807752 WBGene00005754 srw-7 R08F11.5 0.131575747735026
AGIWUR27147
CB4856 CT V 3808636 3810708 WBGene00005753 srw-6 H10D18.3 0.06064685720865
AGIWUR12194
CB4856 CT V 3810786 3811634 WBGene00019179 scl-13 H10D18.4 0.139408193565602

AGIWUR5987
CB4856 CT V 3812596 3813363 WBGene00019178 scl-12 H10D18.2 0.087062536533498
AGIWUR34033
CB4856 CT V 3813737 3831659 WBGene00019180 H10D18.5 H10D18.5 0.0995133884675137
AGIWUR1798;AGIWUR17439;AGIWUR39972;AGIWUR40273;AGIWUR42309;AGIWUR43558
CB4856 CT V 3841859 3844007 WBGene00018543 ugt-32 F47C10.6 -0.0336341680828056
AGIWUR43683
CB4856 CT V 3844645 3847151 WBGene00018539 nhr-185 F47C10.1 0.325602739764356
AGIWUR24112
CB4856 CT V 3850127 3851796 WBGene00018540 btb-21 F47C10.2 0.406301920073536
AGIWUR37295
CB4856 CT V 3852286 3854947 WBGene00018541 nhr-186 F47C10.3 -0.0679467247191727
AGIWUR37709
CB4856 CT V 3856329 3857861 WBGene00018544 nhr-188 F47C10.7 0.182876778498981
AGIWUR32751
CB4856 CT V 3858461 3860243 WBGene00018545 nhr-189 F47C10.8 0.0249016516721401
AGIWUR23666
CB4856 CT V 3861089 3862933 WBGene00018542 nhr-187 F47C10.4 0.293899061287233
AGIWUR31141
CB4856 CT V 3866556 3869005 WBGene00015900 nhr-157 C17E7.5 0.0600906143952233 AGIWUR31162
CB4856 CT V 3871174 3873216 WBGene00015901 nhr-158 C17E7.6 -0.169746852897198 AGIWUR18937
CB4856 CT V 3875245 3878382 WBGene00015902 nhr-159 C17E7.7 0.0767342126569925 AGIWUR34504
CB4856 CT V 3880798 3886204 WBGene00003714 nhr-124 C17E7.8 0.241443046520111 AGIWUR12597
CB4856 CT V 3887750 3889286 WBGene00015906 C17E7.13 C17E7.13 0.545898436959519
AGIWUR26604
CB4856 CT V 3889287 3890861 WBGene00015903 C17E7.9 C17E7.9 -0.0476149487686399 AGIWUR10808
CB4856 CT V 3890968 3892026 WBGene00015905 C17E7.12 C17E7.12 -0.198726126958793
AGIWUR20122
CB4856 CT V 3892856 3894573 WBGene00015899 C17E7.4 C17E7.4 0.0156689621245203 AGIWUR36235
CB4856 CT V 3894961 3898208 WBGene00015898 sre-9 C17E7.3 -0.0860892467264192
AGIWUR1726;AGIWUR23776;AGIWUR40013
CB4856 CT V 3898800 3900202 WBGene00015904 irld-22 C17E7.10 0.274763323477309
AGIWUR29385
CB4856 CT V 3900497 3902558 WBGene00006260 str-229 C17E7.11 -0.0163430102420978
AGIWUR39812
CB4856 CT V 3903107 3904545 WBGene00005404 srh-190 C17E7.2 0.139008117105848 AGIWUR3000
CB4856 CT V 3906141 3908435 WBGene00015897 nhr-156 C17E7.1 -0.393335742973818
AGIWUR558;AGIWUR5994;AGIWUR33341;AGIWUR41453
CB4856 CT V 3909098 3910945 WBGene00005952 srx-61 T20C7.1 0.494522643093489 AGIWUR14959

CB4856	CT	V	3912772	3914597	WBGene00005959	srx-68	K07C6.11	-0.0253436077998259
AGIWUR25156
CB4856	CT	V	3916839	3918446	WBGene00005958	srx-67	K07C6.10	0.456491456596887
AGIWUR12123
CB4856	CT	V	3919589	3920825	WBGene00005957	srx-66	K07C6.9	0.48732512010212	AGIWUR27346
CB4856	CT	V	3921935	3922964	WBGene00005956	srx-65	K07C6.8	0.128877767681663	AGIWUR41357
CB4856	CT	V	3923376	3926642	WBGene00005960	srx-69	K07C6.13	-0.361610527274653
AGIWUR20121
CB4856	CT	V	3929399	3930678	WBGene00005955	srx-64	K07C6.7	0.0767704229104735	AGIWUR27079
CB4856	CT	V	3931626	3933394	WBGene00005954	srx-63	K07C6.6	0.138453484198368
AGIWUR24551;AGIWUR26628
CB4856	CT	V	3933960	3934950	WBGene00005961	srx-70	K07C6.15	-0.0931031268830016
AGIWUR30774;AGIWUR36074
CB4856	CT	V	3936326	3938496	WBGene00019473	cyp-35A5	K07C6.5	-0.069946118785797
AGIWUR1573;AGIWUR21781;AGIWUR22277;AGIWUR42606
CB4856	CT	V	3939032	3941235	WBGene00019472	cyp-35B1	K07C6.4	-0.0323865236166476
AGIWUR32263
CB4856	CT	V	3942215	3944457	WBGene00019471	cyp-35B2	K07C6.3	-0.0250810726799671
AGIWUR11302
CB4856	CT	V	3945353	3947044	WBGene00019470	cyp-35B3	K07C6.2	-0.0643321102851768
AGIWUR44312
CB4856	CT	V	3947365	3948952	WBGene00019469	srz-89	K07C6.1	-0.0584833223578931	AGIWUR17566
CB4856	CT	V	3950027	3952097	WBGene00020386	cyp-34A4	T09H2.1	-0.234102999058827
AGIWUR16851
CB4856	CT	V	3954039	3955460	WBGene00006274	str-247	B0213.9	0.190840513150077
AGIWUR29066;AGIWUR30225;AGIWUR30460
CB4856	CT	V	3955766	3957715	WBGene00015040	cyp-34A5	B0213.10	0.0381096252173023
AGIWUR2873;AGIWUR4685;AGIWUR10311;AGIWUR11935;AGIWUR17491;AGIWUR28198;AGIWUR39604
CB4856	CT	V	3958160	3961358	WBGene00015041	cyp-34A6	B0213.11	0.106118522977205
AGIWUR10465
CB4856	CT	V	3961760	3963815	WBGene00015042	cyp-34A7	B0213.12	0.48369848640791
AGIWUR36195
CB4856	CT	V	3964779	3967880	WBGene00015043	cyp-34A8	B0213.14	-0.199814841459986
AGIWUR14373
CB4856	CT	V	3968134	3970514	WBGene00015044	cyp-34A9	B0213.15	1.51014145233351
AGIWUR2584;AGIWUR10028;AGIWUR10829;AGIWUR16241;AGIWUR18901;AGIWUR23794
CB4856	CT	V	3970712	3973001	WBGene00015045	cyp-34A10	B0213.16	1.49692537554564
AGIWUR35138
CB4856	CT	V	3974661	3976264	WBGene00006141	str-82	B0213.8	0.0183016823913889	AGIWUR36544

CB4856 CT V 3977264 3978811 WBGene00006142 str-83 B0213.7 -0.283325698353309 AGIWUR26623
CB4856 CT V 3978836 3979134 WBGene00015046 nlp-34 B0213.17 -0.251916974552035 AGIWUR5401
CB4856 CT V 3980055 3980622 WBGene00003769 nlp-31 B0213.6 -0.237803690266162 AGIWUR18556
CB4856 CT V 3981980 3982741 WBGene00003768 nlp-30 B0213.5 -0.0327634932312968 AGIWUR27472
CB4856 CT V 3984413 3984847 WBGene00003767 nlp-29 B0213.4 0.375398997592381 AGIWUR3874;AGIWUR24246;AGIWUR25364;AGIWUR35635;AGIWUR36359
CB4856 CT V 3987900 3988312 WBGene00003766 nlp-28 B0213.3 0.400772810394831 AGIWUR1823;AGIWUR3034;AGIWUR3079;AGIWUR11292;AGIWUR12737;AGIWUR20737;AGIWUR31288;AGIWUR32617;AGIWUR33370;AGIWUR33926
CB4856 CT V 3989377 3989770 WBGene00003765 nlp-27 B0213.2 -0.152725258494231 AGIWUR23726;AGIWUR25464
CB4856 CT V 3992655 3994180 WBGene00019567 K09D9.9 K09D9.9 -0.313202910972668 AGIWUR20835
CB4856 CT V 3994780 3995938 WBGene00005953 srx-62 K09D9.10 0.0409948006395471 AGIWUR14251
CB4856 CT V 3997124 4001465 WBGene00019568 K09D9.11 K09D9.11 -0.014947114799952 AGIWUR4967
CB4856 CT V 4002327 4004309 WBGene00019569 K09D9.12 K09D9.12 -0.273371955340366 AGIWUR11640
CB4856 CT V 4005567 4007517 WBGene00005234 srh-8 K09D9.8 0.17974709512845 AGIWUR42726
CB4856 CT V 4008227 4009818 WBGene00005233 srh-7 K09D9.7 -0.091974648640333 AGIWUR28976
CB4856 CT V 4010470 4011966 WBGene00005230 srh-4 K09D9.6 -0.368338311535751 AGIWUR42420
CB4856 CT V 4013021 4014628 WBGene00006107 str-42 K09D9.5 0.0123197935740538 AGIWUR22127;AGIWUR24418
CB4856 CT V 4015587 4016938 WBGene00005232 srh-6 K09D9.4 -0.0208536153417 AGIWUR28540;AGIWUR41631
CB4856 CT V 4017080 4019026 WBGene00005836 srw-89 K09D9.13 0.00727289465849168 AGIWUR9432
CB4856 CT V 4019488 4020601 WBGene00019566 K09D9.3 K09D9.3 0.0827124732481526 AGIWUR17366
CB4856 CT V 4022609 4024475 WBGene00019565 cyp-35A3 K09D9.2 -0.0941637175499501 AGIWUR21092;AGIWUR23742;AGIWUR29860;AGIWUR40665
CB4856 CT V 4025091 4026212 WBGene00019564 K09D9.1 K09D9.1 -0.475167267488644 AGIWUR30594
CB4856 CT V 4031619 4033446 WBGene00016786 cyp-35A4 C49G7.8 -0.421206396927408 AGIWUR16323;AGIWUR38020;AGIWUR39215
CB4856 CT V 4038821 4039934 WBGene00077593 C49G7.12 C49G7.12 -0.304125479436836 AGIWUR1545
CB4856 CT V 4040409 4042004 WBGene00016788 C49G7.10 C49G7.10 0.00680549452442669 AGIWUR1228;AGIWUR4669;AGIWUR11163;AGIWUR13514;AGIWUR28269;AGIWUR31558;AGIWUR39242
CB4856 CT V 4042404 4043599 WBGene00016783 irg-2 C49G7.5 -0.543879538199502 AGIWUR19682

CB4856 CT V 4047841 4049354 WBGene00005385 srh-169 C49G7.2 -0.0669576704562047 AGIWUR21978
CB4856 CT V 4054191 4055109 WBGene00016789 djr-1.2 C49G7.11 0.192480540890021
AGIWUR30122
CB4856 CT V 4056165 4062184 WBGene00017035 D1065.3 D1065.3 -0.0219653382676297
AGIWUR9655;AGIWUR15863;AGIWUR37113;AGIWUR40042
CB4856 CT V 4062497 4068573 WBGene00023264 D1065.2 D1065.2 0.0618409503089676 AGIWUR24744
CB4856 CT V 4069424 4071542 WBGene00005420 srh-210 D1065.4 0.0125717793365236 AGIWUR36401
CB4856 CT V 4073755 4077548 WBGene00017034 otpl-2 D1065.1 0.214624907257703 AGIWUR30779
CB4856 CT V 4078061 4079813 WBGene00005421 srh-211 D1065.5 -0.466472626166279 AGIWUR12980
CB4856 CT V 4081228 4083052 WBGene00018190 F38H12.5 F38H12.5 0.154690197217437
AGIWUR6655;AGIWUR32282;AGIWUR40051
CB4856 CT V 4095040 4100541 WBGene00018189 nhr-181 F38H12.3 -0.333588050753818
AGIWUR23566
CB4856 CT V 4101867 4103893 WBGene00005624 srj-40 F38H12.2 0.033082327736027
AGIWUR1960
CB4856 CT V 4104598 4106244 WBGene00005623 srj-39 F38H12.1 0.0800522962859764
AGIWUR19120
CB4856 CT V 4106373 4108138 WBGene00005879 srw-132 T05B4.5 0.0458363834579689 AGIWUR44738
CB4856 CT V 4110634 4112616 WBGene00005881 srw-134 T05B4.7 0.292190627678267 AGIWUR38922
CB4856 CT V 4113335 4114727 WBGene00005880 srw-133 T05B4.6 -0.226967412256049 AGIWUR11913
CB4856 CT V 4117585 4118432 WBGene00020240 T05B4.9 T05B4.9 0.0600966894963211 AGIWUR20216
CB4856 CT V 4119890 4120729 WBGene00020241 T05B4.10 T05B4.10 -0.0842477010721794
AGIWUR25881
CB4856 CT V 4121489 4122517 WBGene00020242 phat-5 T05B4.11 0.825334412994653
AGIWUR8147;AGIWUR9076;AGIWUR14271;AGIWUR30063;AGIWUR41290;AGIWUR44095
CB4856 CT V 4123329 4124862 WBGene00020243 T05B4.12 T05B4.12 0.28003647165248
AGIWUR35874
CB4856 CT V 4126319 4127535 WBGene00020244 T05B4.13 T05B4.13 0.0577452225999053
AGIWUR21784
CB4856 CT V 4127868 4129602 WBGene00020238 T05B4.4 T05B4.4 0.0412924119821856 AGIWUR27590
CB4856 CT V 4129904 4130909 WBGene00020237 phat-4 T05B4.3 -0.131901837484702
AGIWUR24108;AGIWUR28429
CB4856 CT V 4131925 4134981 WBGene00003647 nhr-57 T05B4.2 0.210903826321758
AGIWUR4984;AGIWUR6609;AGIWUR9301;AGIWUR10813;AGIWUR15809;AGIWUR19457;AGIWUR36903
CB4856 CT V 4134968 4138825 WBGene00020236 lgc-1 T05B4.1 0.0543239059886975
AGIWUR12300;AGIWUR14909
CB4856 CT V 4139008 4143120 WBGene00044528 lgc-29 Y45G5AL.2 0.0627134349850063
AGIWUR6685;AGIWUR40865
CB4856 CT V 4144505 4147571 WBGene00021554 Y45G5AL.1 Y45G5AL.1 0.131289478972963

AGIWUR44946
CB4856 CT V 4147550 4152296 WBGene00021558 Y45G5AM.6 Y45G5AM.6 0.0630343538161711
AGIWUR15909
CB4856 CT V 4160383 4160889 WBGene00021557 Y45G5AM.5 Y45G5AM.5 0.555655445296791
AGIWUR43106
CB4856 CT V 4163222 4163611 WBGene00043666 Y45G5AM.4 Y45G5AM.4 0.261084696382446
AGIWUR36153
CB4856 CT V 4164554 4166825 WBGene00021556 Y45G5AM.3 Y45G5AM.3 -0.0350390104225831
AGIWUR26169
CB4856 CT V 4166796 4169837 WBGene00021559 Y45G5AM.7 Y45G5AM.7 -0.0339540951010022
AGIWUR22011
CB4856 CT V 4175882 4181357 WBGene00021555 Y45G5AM.2 Y45G5AM.2 -0.3116046932002
AGIWUR12069
CB4856 CT V 4181332 4191515 WBGene00021561 Y45G5AM.9 Y45G5AM.9 -0.0386479147600927
AGIWUR11428;AGIWUR18622;AGIWUR43252
CB4856 CT V 4195577 4199399 WBGene00019276 H43I07.3 H43I07.3 -0.10599442260642
AGIWUR27848
CB4856 CT V 4199485 4203696 WBGene00019275 rpac-40 H43I07.2 -0.0816896055584344
AGIWUR7057
CB4856 CT V 4204721 4209303 WBGene00019274 H43I07.1 H43I07.1 0.0773337189656758
AGIWUR18443
CB4856 CT V 4219643 4222197 WBGene00003747 nlp-9 E03D2.2 0.331280576116644 AGIWUR39554
CB4856 CT V 4236418 4238056 WBGene00003751 nlp-13 E03D2.1 0.0908792051946214 AGIWUR6822
CB4856 CT V 4239150 4240392 WBGene00005415 srh-204 E03D2.3 -0.0161250326660038 AGIWUR34830
CB4856 CT V 4240952 4242531 WBGene00017107 srt-12 E03D2.4 -0.0772846385969927 AGIWUR44820
CB4856 CT V 4247969 4250147 WBGene00022528 srt-13 ZC142.1 -0.0259916857018236 AGIWUR2916
CB4856 CT V 4253502 4256574 WBGene00022529 srt-14 ZC142.2 -0.0964989225769894 AGIWUR28474
CB4856 CT V 4257957 4260190 WBGene00018554 srt-3 F47D2.3 -0.00482266358903072 AGIWUR40923
CB4856 CT V 4261485 4264616 WBGene00018555 srt-35 F47D2.4 0.120549077049107 AGIWUR26325
CB4856 CT V 4269486 4271358 WBGene00018557 srt-38 F47D2.6 0.142717511210624 AGIWUR30244
CB4856 CT V 4288412 4290193 WBGene00005406 srh-193 F47D2.9 0.0251253161248339 AGIWUR36547
CB4856 CT V 4291836 4293525 WBGene00005407 srh-194 F47D2.10 -0.0370330008203092
AGIWUR29375
CB4856 CT V 4296721 4298089 WBGene00269432 ZC132.11 ZC132.11 0.088176490771537
AGIWUR1308;AGIWUR24756;AGIWUR30767;AGIWUR35418
CB4856 CT V 4300626 4311784 WBGene00022521 ZC132.2 ZC132.2 -0.0374519307685253
AGIWUR3578;AGIWUR5811;AGIWUR38417
CB4856 CT V 4311993 4315157 WBGene00005405 srh-192 ZC132.7 0.0168570966251931 AGIWUR31160
CB4856 CT V 4315725 4317713 WBGene00022526 ZC132.8 ZC132.8 -0.153082766051041 AGIWUR24614

CB4856	CT	V	4317920 4320001 WBGene00022527	ZC132.9 ZC132.9 0.0463111775083496	AGIWUR4580
CB4856	CT	V	4320460 4322218 WBGene00235310	R02D1.2 R02D1.2 -0.484079282280305
AGIWUR24861;AGIWUR42264
CB4856	CT	V	4327121 4328736 WBGene00017060	D2063.1 D2063.1 0.317396954285838
AGIWUR2289;AGIWUR6390;AGIWUR22124;AGIWUR40360
CB4856	CT	V	4329039 4332550 WBGene00017061	oac-12	D2063.2 -0.0907728133687762	AGIWUR6604
CB4856	CT	V	4336762 4338925 WBGene00044529	irld-1	D2063.4 0.0033880354146163
AGIWUR17360;AGIWUR26355
CB4856	CT	V	4338854 4341308 WBGene00017062	glrx-3	D2063.3 -0.303349949345893	AGIWUR5206
CB4856	CT	V	4341443 4346050 WBGene00017981	figl-1	F32D1.1 -0.0622071887160658	AGIWUR37838
CB4856	CT	V	4346089 4346425 WBGene00017982	hpo-18	F32D1.2 -0.337252260477675
AGIWUR18888;AGIWUR34489
CB4856	CT	V	4347166 4349768 WBGene00003159	mcm-7	F32D1.10	-0.0490393497784369
AGIWUR26538;AGIWUR27545
CB4856	CT	V	4349774 4361552 WBGene00017983	F32D1.3 F32D1.3 0.613586748908167	AGIWUR39251
CB4856	CT	V	4362722 4364650 WBGene00001722	grl-13	F32D1.4 0.1182164219715 AGIWUR27074
CB4856	CT	V	4365447 4366483 WBGene00044530	F32D1.11	F32D1.11
-0.00540848006257022	AGIWUR14449;AGIWUR19290;AGIWUR22820
CB4856	CT	V	4369351 4371383 WBGene00017984	F32D1.5 F32D1.5 -0.0685598576757425
AGIWUR12479;AGIWUR18525;AGIWUR24068
CB4856	CT	V	4371807 4373811 WBGene00017988	fipp-1	F32D1.9 -0.117405244295924	AGIWUR17104
CB4856	CT	V	4373760 4374874 WBGene00017985	neg-1	F32D1.6 0.0463304160410694	AGIWUR35653
CB4856	CT	V	4374974 4376173 WBGene00017986	F32D1.7 F32D1.7 -0.145576992022121	AGIWUR15298
CB4856	CT	V	4376619 4377722 WBGene00017987	F32D1.8 F32D1.8 -0.0456277195134209
AGIWUR9211;AGIWUR17294;AGIWUR17529;AGIWUR23378;AGIWUR26753
CB4856	CT	V	4379643 4381592 WBGene00269436	F13A2.14	F13A2.14	0.139352989816619
AGIWUR4194
CB4856	CT	V	4382916 4385242 WBGene00017411	F13A2.2 F13A2.2 0.243751622156823	AGIWUR6317
CB4856	CT	V	4386808 4387502 WBGene00044531	F13A2.9 F13A2.9 0.000332084752522555
AGIWUR12461;AGIWUR18733;AGIWUR36382;AGIWUR40455;AGIWUR41304
CB4856	CT	V	4393540 4396357 WBGene00017412	F13A2.3 F13A2.3 -0.0486820967951509	AGIWUR8025
CB4856	CT	V	4398635 4400218 WBGene00017413	F13A2.4 F13A2.4 -0.384438625630633	AGIWUR7096
CB4856	CT	V	4401298 4401940 WBGene00017414	F13A2.5 F13A2.5 -0.354645724942484	AGIWUR2818
CB4856	CT	V	4402749 4404530 WBGene00017415	F13A2.6 F13A2.6 -0.488094574682039	AGIWUR43256
CB4856	CT	V	4407819 4410280 WBGene00003708	nhr-118 F13A2.8 0.0955332606626005
AGIWUR5659;AGIWUR18832;AGIWUR26406;AGIWUR33940;AGIWUR39095
CB4856	CT	V	4410943 4412401 WBGene00021617	Y47D7A.3	Y47D7A.3	0.160767984604989
AGIWUR407
CB4856	CT	V	4412748 4414663 WBGene00001714	grl-5	Y47D7A.5	-0.107864815374692

AGIWUR456
CB4856	CT	V	4416022	4416814	WBGene00235165	Y47D7A.18	Y47D7A.18	0.00643343290742138
AGIWUR35916
CB4856	CT	V	4419454	4419867	WBGene00021620	Y47D7A.7	Y47D7A.7	0.808125994718605
AGIWUR9054
CB4856	CT	V	4420275	4420746	WBGene00021616	Y47D7A.2	Y47D7A.2	0.511619278795669
AGIWUR14377
CB4856	CT	V	4420997	4421596	WBGene00004820	skr-14	Y47D7A.8	-0.174110990411458
AGIWUR20702
CB4856	CT	V	4422406	4423065	WBGene00004813	skr-7	Y47D7A.1	0.245723456717377
AGIWUR38009
CB4856	CT	V	4423609	4423968	WBGene00021621	Y47D7A.9	Y47D7A.9	-0.154649498247132
AGIWUR12979
CB4856	CT	V	4427769	4428302	WBGene00021623	Y47D7A.11	Y47D7A.11	-0.144319533078592
AGIWUR626;AGIWUR1575;AGIWUR3659;AGIWUR6144;AGIWUR8714;AGIWUR19752;AGIWUR36378;AGIWUR38334;AGIWUR38504;AGIWUR43833
CB4856	CT	V	4428950	4429676	WBGene00021624	Y47D7A.12	Y47D7A.12	0.126357662375417
AGIWUR3137
CB4856	CT	V	4431791	4433185	WBGene00021627	Y47D7A.15	Y47D7A.15	-0.278140462035807
AGIWUR1754;AGIWUR7726;AGIWUR11111;AGIWUR23580;AGIWUR27475
CB4856	CT	V	4434883	4435928	WBGene00021625	Y47D7A.13	Y47D7A.13	-0.152507591717532
AGIWUR10566
CB4856	CT	V	4438932	4443223	WBGene00044637	rft-1	Y47D7A.16	-0.320279395740793
AGIWUR20975;AGIWUR25736
CB4856	CT	V	4443110	4450555	WBGene00021626	rft-2	Y47D7A.14	-0.059800065225037
AGIWUR10755;AGIWUR11742;AGIWUR12084;AGIWUR23538;AGIWUR28386;AGIWUR30389;AGIWUR33218;AGIWUR39710
CB4856	CT	V	4451049	4454734	WBGene00019224	gnrr-5	H22D07.1	0.0650101386444657
AGIWUR44776
CB4856	CT	V	4471365	4473602	WBGene00020370	T08H10.3	T08H10.3	0.0508561899941604
AGIWUR30288
CB4856	CT	V	4474843	4476996	WBGene00006101	str-35	T08H10.2	0.0300387288023924
AGIWUR27901;AGIWUR32256
CB4856	CT	V	4477561	4479191	WBGene00006100	str-34	T08H10.5	0.106044204377244
AGIWUR19786
CB4856	CT	V	4480680	4481546	WBGene00020371	T08H10.4	T08H10.4	-0.285191093132973
AGIWUR43086
CB4856	CT	V	4483459	4486543	WBGene00020369	T08H10.1	T08H10.1	-0.243612869781405
AGIWUR990
CB4856	CT	V	4497463	4511449	WBGene00006796	unc-62	T28F12.2	0.152587129570396

AGIWUR15699;AGIWUR16484;AGIWUR18052;AGIWUR21053;AGIWUR21660;AGIWUR22015;AGIWUR25612;AGIWUR26913;AGIWUR30675;AGIWUR35589;AGIWUR41385
CB4856 CT V 4511977 4516947 WBGene00020904 T28F12.1 T28F12.1 0.0787042175350852 AGIWUR17708
CB4856 CT V 4517643 4549291 WBGene00004947 sos-1 T28F12.3 0.284287059139544 AGIWUR4204
CB4856 CT V 4556055 4557114 WBGene00022017 Y61A9LA.5 Y61A9LA.5 -0.259023882899045 AGIWUR15931
CB4856 CT V 4558318 4559801 WBGene00022018 Y61A9LA.7 Y61A9LA.7 -0.136045723104297 AGIWUR8198;AGIWUR13524;AGIWUR17992;AGIWUR19357;AGIWUR39108;AGIWUR41819
CB4856 CT V 4562215 4570043 WBGene00022019 sut-2 Y61A9LA.8 0.06965098657321 AGIWUR20263;AGIWUR29703;AGIWUR34769;AGIWUR39327;AGIWUR39758;AGIWUR44236;AGIWUR45069
CB4856 CT V 4571143 4585167 WBGene00022015 Y61A9LA.3 Y61A9LA.3 0.0147555722345709 AGIWUR3402;AGIWUR4011;AGIWUR10244;AGIWUR10368
CB4856 CT V 4592838 4602014 WBGene00022014 Y61A9LA.1 Y61A9LA.1 0.0876532630259206 AGIWUR28333
CB4856 CT V 4605004 4606926 WBGene00022022 Y61A9LA.11 Y61A9LA.11 0.111295801167577 AGIWUR34478
CB4856 CT V 4607039 4615817 WBGene00022021 Y61A9LA.10 Y61A9LA.10 -0.276691094491458 AGIWUR16782
CB4856 CT V 4616156 4617766 WBGene00018814 F54D11.4 F54D11.4 0.133240616128141 AGIWUR40577
CB4856 CT V 4617938 4628510 WBGene00018812 sumv-2 F54D11.2 0.0280998868852098 AGIWUR7205;AGIWUR11926;AGIWUR16823;AGIWUR22271;AGIWUR34312;AGIWUR35666
CB4856 CT V 4628580 4635263 WBGene00018811 pmt-2 F54D11.1 -0.329028039228996 AGIWUR42429;AGIWUR44478
CB4856 CT V 4635327 4637145 WBGene00018813 F54D11.3 F54D11.3 0.231366947557625 AGIWUR10374
CB4856 CT V 4642952 4646931 WBGene00004928 soc-1 F41F3.2 -0.0420336336313198 AGIWUR29213
CB4856 CT V 4647665 4648241 WBGene00018297 F41F3.3 F41F3.3 0.405412462783453 AGIWUR8735
CB4856 CT V 4648832 4650726 WBGene00044533 F41F3.8 F41F3.8 0.0743284623487419 AGIWUR3540;AGIWUR11934;AGIWUR30905;AGIWUR34907;AGIWUR43347
CB4856 CT V 4652863 4654007 WBGene00000712 col-139 F41F3.4 0.125941923683488 AGIWUR17250
CB4856 CT V 4655475 4655786 WBGene00269385 F41F3.9 F41F3.9 -0.130412061946313 AGIWUR18253
CB4856 CT V 4656158 4657812 WBGene00005966 srx-75 F41F3.5 -0.553671870055066 AGIWUR38791
CB4856 CT V 4658949 4662500 WBGene00005965 srx-74 F41F3.6 0.32081562006213 AGIWUR37537
CB4856 CT V 4663616 4665751 WBGene00005964 srx-73 F41F3.7 0.15895675341516 AGIWUR39489
CB4856 CT V 4666745 4668736 WBGene00005968 srx-77 K03B4.5 0.187462605196261 AGIWUR36430
CB4856 CT V 4671030 4672610 WBGene00219236 K03B4.9 K03B4.9 -0.00091019755194921 AGIWUR10250

CB4856	CT	V	4681975	4685110	WBGene00019355	K03B4.4	K03B4.4	-0.130313023511544	AGIWUR20172
CB4856	CT	V	4687107	4688028	WBGene00006392	taf-10	K03B4.3	0.148627993838254	AGIWUR45166
CB4856	CT	V	4687974	4689231	WBGene00019354	K03B4.2	K03B4.2	0.367500794733262	AGIWUR5490
CB4856	CT	V	4689330	4691611	WBGene00019353	K03B4.1	K03B4.1	0.206412625571671	AGIWUR43290
CB4856	CT	V	4691605	4692433	WBGene00019357	cpg-8	K03B4.7	0.0991384023773403	AGIWUR20437;AGIWUR38950
CB4856	CT	V	4694974	4700289	WBGene00022584	ZC266.1	ZC266.1	-0.109941745977133	AGIWUR1042;AGIWUR25299
CB4856	CT	V	4710764	4714911	WBGene00018211	oac-24	F39G3.6	-0.0426329204716351	AGIWUR31878
CB4856	CT	V	4715388	4720148	WBGene00004195	prx-6	F39G3.7	0.0936426221394555	AGIWUR44604
CB4856	CT	V	4722618	4724621	WBGene00018210	F39G3.5	F39G3.5	0.0277140669234051	AGIWUR29741
CB4856	CT	V	4724663	4726726	WBGene00018209	F39G3.4	F39G3.4	0.125110401834266	AGIWUR3506
CB4856	CT	V	4726721	4730234	WBGene00006570	tig-2	F39G3.8	-0.111758077447657	AGIWUR18954
CB4856	CT	V	4731911	4734723	WBGene00018208	F39G3.3	F39G3.3	-0.245072886006312	AGIWUR460;AGIWUR10436;AGIWUR41800
CB4856	CT	V	4737834	4741169	WBGene00018207	F39G3.2	F39G3.2	-0.186745829518388	AGIWUR7231
CB4856	CT	V	4742427	4745373	WBGene00018206	ugt-61	F39G3.1	-0.388978683040438	AGIWUR3749
CB4856	CT	V	4745569	4748089	WBGene00015985	C18G1.7	C18G1.7	-0.0115951794065126	AGIWUR40127
CB4856	CT	V	4750580	4753808	WBGene00015984	C18G1.6	C18G1.6	0.0893130965834308	AGIWUR27193
CB4856	CT	V	4754313	4755469	WBGene00001855	hil-4	C18G1.5	-0.00838438724503021	AGIWUR2414;AGIWUR12114;AGIWUR20399;AGIWUR21732;AGIWUR40557;AGIWUR42531
CB4856	CT	V	4755985	4758584	WBGene00003994	pgl-3	C18G1.4	-0.170242260326795	AGIWUR829
CB4856	CT	V	4760974	4764179	WBGene00015982	bgnt-1.4	C18G1.3	0.23094788148795	AGIWUR39744
CB4856	CT	V	4764266	4766601	WBGene00015986	C18G1.8	C18G1.8	0.239837821902372	AGIWUR16977
CB4856	CT	V	4767886	4769753	WBGene00015987	C18G1.9	C18G1.9	0.185968249565232	AGIWUR27591
CB4856	CT	V	4770670	4773091	WBGene00015981	elt-7	C18G1.2	0.361674725701866	AGIWUR25029
CB4856	CT	V	4777960	4780019	WBGene00015980	C18G1.1	C18G1.1	0.499554328547806	AGIWUR10156
CB4856	CT	V	4783613	4786140	WBGene00022013	Y60C6A.1	Y60C6A.1	0.0354202373348551	AGIWUR16948
CB4856	CT	V	4794599	4796996	WBGene00019840	R02F11.2	R02F11.2	-0.0410858034050583	AGIWUR2726;AGIWUR5721;AGIWUR23238;AGIWUR35491
CB4856	CT	V	4801094	4802626	WBGene00019839	R02F11.1	R02F11.1	0.178888200462858	AGIWUR29210
CB4856	CT	V	4802647	4805947	WBGene00019841	R02F11.3	R02F11.3	-0.355817296911316	AGIWUR17799;AGIWUR29564
CB4856	CT	V	4810865	4818663	WBGene00019842	R02F11.4	R02F11.4	-0.267187655096755	AGIWUR17550
CB4856	CT	V	4818701	4823344	WBGene00016509	adss-1	C37H5.6	0.140692461125884	

AGIWUR3440;AGIWUR8948;AGIWUR9961;AGIWUR22132;AGIWUR22362;AGIWUR36251;AGIWUR36473
CB4856 CT V 4824130 4826760 WBGene00002010 hsp-6 C37H5.8 -0.194827827351982
AGIWUR10444;AGIWUR26791
CB4856 CT V 4827360 4836025 WBGene00016508 C37H5.5 C37H5.5 0.143392855431502 AGIWUR32683
CB4856 CT V 4836027 4840010 WBGene00003528 nas-9 C37H5.9 0.754651081295281 AGIWUR7821
CB4856 CT V 4840619 4841269 WBGene00000859 cwp-1 C37H5.10 -0.270447791028186
AGIWUR10874
CB4856 CT V 4841940 4842567 WBGene00000860 cwp-2 C37H5.11 -0.121043214674291
AGIWUR292
CB4856 CT V 4842836 4843845 WBGene00000861 cwp-3 C37H5.4 -0.23268954164905 AGIWUR2144
CB4856 CT V 4844685 4846984 WBGene00016507 abhd-5.2 C37H5.3 -0.13138528490157
AGIWUR26648;AGIWUR36638
CB4856 CT V 4847653 4849838 WBGene00016506 abhd-5.1 C37H5.2 0.232304166473263
AGIWUR25974
CB4856 CT V 4851472 4857260 WBGene00003591 nex-4 C37H5.1 -0.0616611275558974 AGIWUR22444
CB4856 CT V 4857149 4862965 WBGene00016511 C37H5.13 C37H5.13 -0.12815728029099
AGIWUR894;AGIWUR6151;AGIWUR15127;AGIWUR17736;AGIWUR20020;AGIWUR22385;AGIWUR27555
CB4856 CT V 4865648 4870355 WBGene00005110 srd-32 T19H12.5 -0.294218016665367
AGIWUR40248
CB4856 CT V 4872303 4874200 WBGene00005111 srd-33 T19H12.4 -0.0257873093975558
AGIWUR15862
CB4856 CT V 4874923 4879319 WBGene00020590 T19H12.6 T19H12.6 -0.451328101577678
AGIWUR28964
CB4856 CT V 4879945 4881477 WBGene00006098 str-32 T19H12.7 0.0133192068286583
AGIWUR24416
CB4856 CT V 4882178 4884319 WBGene00020591 nhr-220 T19H12.8 -0.0788896712396472
AGIWUR21627
CB4856 CT V 4884790 4885420 WBGene00044282 T19H12.12 T19H12.12 -0.0963198039590818
AGIWUR7237
CB4856 CT V 4885672 4886809 WBGene00020589 T19H12.3 T19H12.3 0.0179892675214504
AGIWUR21961
CB4856 CT V 4887187 4889629 WBGene00020592 ugt-12 T19H12.9 -0.464332557014997
AGIWUR26207
CB4856 CT V 4890429 4891422 WBGene00020588 T19H12.2 T19H12.2 0.098789009049316
AGIWUR8639;AGIWUR8820
CB4856 CT V 4892588 4895071 WBGene00020593 ugt-11 T19H12.10 -0.0970935893610374
AGIWUR33007
CB4856 CT V 4896028 4897969 WBGene00020587 ugt-9 T19H12.1 -0.58288374131399
AGIWUR1830

CB4856	CT	V	4898217	4900082	WBGene00020594	ugt-10	T19H12.11	0.0268251047965222	AGIWUR2113
CB4856	CT	V	4901374	4903010	WBGene00019235	H23N18.4	H23N18.4	-0.15811034730898	AGIWUR15965;AGIWUR37437;AGIWUR41886
CB4856	CT	V	4903930	4905918	WBGene00019234	ugt-8	H23N18.3	0.368420808423192	AGIWUR35284
CB4856	CT	V	4906253	4908258	WBGene00019233	ugt-14	H23N18.2	-0.381793239534026	AGIWUR1674
CB4856	CT	V	4908868	4911399	WBGene00019232	ugt-13	H23N18.1	-0.542686147313699	AGIWUR10867;AGIWUR24424;AGIWUR29728
CB4856	CT	V	4911712	4912381	WBGene00019236	H23N18.5	H23N18.5	-0.535542695843951	AGIWUR19342
CB4856	CT	V	4913061	4915079	WBGene00044534	H23N18.6	H23N18.6	-0.0885513530874231	AGIWUR26450;AGIWUR31107;AGIWUR36453
CB4856	CT	V	4918154	4918585	WBGene00015340	C02E7.7	C02E7.7	-1.04687841563277	AGIWUR24668
CB4856	CT	V	4919207	4919586	WBGene00015339	C02E7.6	C02E7.6	-0.215116951362051	AGIWUR2706;AGIWUR4643;AGIWUR16815;AGIWUR18589;AGIWUR20623;AGIWUR37703;AGIWUR43853
CB4856	CT	V	4920342	4920790	WBGene00015341	C02E7.8	C02E7.8	-0.123173020998179	AGIWUR11061
CB4856	CT	V	4921102	4922616	WBGene00005248	srh-23	C02E7.5	0.188588167066615	AGIWUR18669
CB4856	CT	V	4923292	4924594	WBGene00005247	srh-22	C02E7.4	0.0245213987146506	AGIWUR33717
CB4856	CT	V	4924918	4926224	WBGene00005245	srh-20	C02E7.3	0.176036222982857	AGIWUR34535
CB4856	CT	V	4926671	4928181	WBGene00005246	srh-21	C02E7.2	0.319426995247984	AGIWUR37003
CB4856	CT	V	4928278	4929942	WBGene00006238	str-205	C02E7.9	-0.271673992708134	AGIWUR22281
CB4856	CT	V	4930817	4933027	WBGene00015342	C02E7.10	C02E7.10	0.0421885892324472	AGIWUR2084
CB4856	CT	V	4934706	4939085	WBGene00015338	catp-2	C02E7.1	-0.535472711985651	AGIWUR11404
CB4856	CT	V	4939260	4940641	WBGene00006246	str-213	C02E7.11	0.0465192905997069	AGIWUR9176
CB4856	CT	V	4941196	4942414	WBGene00006245	str-212	C02E7.12	-0.432252103689252	AGIWUR13654;AGIWUR26919
CB4856	CT	V	4943116	4944319	WBGene00006244	str-211	C02E7.13	0.0681834752846235	AGIWUR19419
CB4856	CT	V	4945662	4948299	WBGene00006243	str-210	F26G5.11	-0.250766082379779	AGIWUR5249;AGIWUR20666
CB4856	CT	V	4948935	4951024	WBGene00006242	str-209	F26G5.5	0.271701559725212	AGIWUR31438
CB4856	CT	V	4951734	4953035	WBGene00006240	str-207	F26G5.4	0.00489339368358621	AGIWUR2049
CB4856	CT	V	4954250	4955989	WBGene00005100	srd-22	F26G5.3	0.163320731031632	AGIWUR12411
CB4856	CT	V	4956829	4958572	WBGene00006241	str-208	F26G5.2	0.341849368730431	AGIWUR12648
CB4856	CT	V	4958603	4963098	WBGene00006523	tam-1	F26G5.9	-0.0214844046645277	

AGIWUR8886;AGIWUR16292;AGIWUR16850;AGIWUR17900;AGIWUR35397;AGIWUR35895;AGIWUR38065
CB4856 CT V 4971770 4973043 WBGene00017845 srsx-9 F26G5.10 -0.0104903018294893
AGIWUR35069
CB4856 CT V 4973314 4974777 WBGene00044117 srsx-10 F26G5.12 0.190210433729933
AGIWUR29835;AGIWUR29983
CB4856 CT V 4975485 4977502 WBGene00017843 F26G5.1 F26G5.1 -0.0232503172913283 AGIWUR21735
CB4856 CT V 4977934 4979160 WBGene00015736 srsx-11 C13D9.4 -0.252172728797876
AGIWUR6710;AGIWUR18113
CB4856 CT V 4979947 4980973 WBGene00015737 srsx-12 C13D9.5 0.120320263749191 AGIWUR26886
CB4856 CT V 4981809 4983191 WBGene00015738 srsx-13 C13D9.6 -0.363098461102264 AGIWUR42234
CB4856 CT V 4983748 4987197 WBGene00003573 ncx-8 C13D9.7 0.0327968719599978
AGIWUR6415;AGIWUR24815;AGIWUR31203
CB4856 CT V 4991505 4995064 WBGene00003574 ncx-9 C13D9.8 0.144107876238735 AGIWUR19023
CB4856 CT V 4997048 5000558 WBGene00015739 ugt-7 C13D9.9 -0.488140301756538 AGIWUR21056
CB4856 CT V 5001686 5003414 WBGene00005659 srr-8 C13D9.3 0.0374578833862991 AGIWUR3691
CB4856 CT V 5007229 5011702 WBGene00005656 srr-5 C13D9.2 0.140160070371801 AGIWUR31596
CB4856 CT V 5012994 5015059 WBGene00005657 srr-6 C13D9.1 0.210793299086051 AGIWUR19957
CB4856 CT V 5019224 5022369 WBGene00005655 srr-4 K11D12.3 -0.147246277587463
AGIWUR16882
CB4856 CT V 5023706 5027723 WBGene00019644 cpt-4 K11D12.4 1.25293622715841
AGIWUR10491
CB4856 CT V 5029324 5030811 WBGene00019645 swt-7 K11D12.5 -0.0190393261681301
AGIWUR6855
CB4856 CT V 5033398 5034171 WBGene00044535 K11D12.13 K11D12.13 -0.209878216626141
AGIWUR6605;AGIWUR23117;AGIWUR31448
CB4856 CT V 5034528 5035169 WBGene00019646 K11D12.6 K11D12.6 -0.480332436520168
AGIWUR6484
CB4856 CT V 5035252 5036464 WBGene00019647 K11D12.7 K11D12.7 -0.297353434493834
AGIWUR25453
CB4856 CT V 5036572 5038658 WBGene00019650 K11D12.11 K11D12.11 -0.0733226561214355
AGIWUR11290
CB4856 CT V 5039669 5041003 WBGene00019648 K11D12.8 K11D12.8 -0.03178925678339
AGIWUR31955
CB4856 CT V 5042408 5046203 WBGene00019649 K11D12.9 K11D12.9 0.188361020739176
AGIWUR32970
CB4856 CT V 5049541 5052214 WBGene00004136 pqn-51 K11D12.2 -0.0414385444208669
AGIWUR12989;AGIWUR15695;AGIWUR15696;AGIWUR25674;AGIWUR31797
CB4856 CT V 5052261 5053490 WBGene00019651 K11D12.12 K11D12.12 0.19595155945138
AGIWUR37796

CB4856	CT	V	5053619 5056473 WBGene00000862	cwp-4	K11D12.1	-0.268772898010731
AGIWUR40515
CB4856	CT	V	5056378 5064803 WBGene00003374	mlk-1	K11D12.10	0.20451970911728
AGIWUR16397;AGIWUR31783;AGIWUR32510
CB4856	CT	V	5067637 5069665 WBGene00021975	Y58A7A.1	Y58A7A.1	-0.42201017508682
AGIWUR8433
CB4856	CT	V	5071695 5072231 WBGene00021976	Y58A7A.2	Y58A7A.2	0.0670400354973388
AGIWUR32706
CB4856	CT	V	5077849 5080903 WBGene00021977	Y58A7A.3	Y58A7A.3
0.000960535129672202	AGIWUR2789;AGIWUR17112;AGIWUR33812;AGIWUR39349
CB4856	CT	V	5082002 5085007 WBGene00021978	Y58A7A.4	Y58A7A.4	0.0992467029380055
AGIWUR35117
CB4856	CT	V	5085886 5087121 WBGene00021979	Y58A7A.5	Y58A7A.5	0.248131345269783
AGIWUR37032
CB4856	CT	V	5087601 5089484 WBGene00021980	glb-32	Y58A7A.6	-0.189686408286375
AGIWUR16777
CB4856	CT	V	5093715 5096277 WBGene00015444	C04F5.2 C04F5.2 -0.0826889694441206	AGIWUR36566
CB4856	CT	V	5097150 5098907 WBGene00006782	unc-46	C04F5.3 -0.0332263266711843
AGIWUR3171;AGIWUR5049;AGIWUR16856;AGIWUR35187
CB4856	CT	V	5100098 5102117 WBGene00015446	srab-1	C04F5.4 -0.246917877738416	AGIWUR42529
CB4856	CT	V	5103042 5105034 WBGene00015447	srab-2	C04F5.5 0.0585448370176138	AGIWUR4344
CB4856	CT	V	5105764 5107916 WBGene00015448	srab-3	C04F5.6 -0.0076189070434296	AGIWUR1011
CB4856	CT	V	5108180 5110791 WBGene00015449	ugt-63	C04F5.7 -0.180845737357937
AGIWUR27307;AGIWUR28572;AGIWUR30566;AGIWUR40417
CB4856	CT	V	5111518 5113754 WBGene00015450	C04F5.8 C04F5.8 0.0346761368055759	AGIWUR35055
CB4856	CT	V	5114749 5119209 WBGene00015451	C04F5.9 C04F5.9 -0.0123281351711952	AGIWUR31490
CB4856	CT	V	5119939 5126916 WBGene00004795	sid-1	C04F5.1 0.07996419174005	AGIWUR24735
CB4856	CT	V	5127055 5130076 WBGene00017468	F14F9.5 F14F9.5 0.167467419889258	AGIWUR36714
CB4856	CT	V	5130318 5131538 WBGene00044536	F14F9.8 F14F9.8 0.380726397540625
AGIWUR74;AGIWUR1622;AGIWUR6008;AGIWUR25330;AGIWUR26386;AGIWUR29636;AGIWUR32108;AGIWUR40651;AGIWUR45182
CB4856	CT	V	5132741 5137100 WBGene00017467	F14F9.4 F14F9.4 -0.0719830957454501	AGIWUR4813
CB4856	CT	V	5142300 5143798 WBGene00005346	srh-129 F14F9.7 0.286717022866462	AGIWUR36675
CB4856	CT	V	5144831 5145781 WBGene00017465	F14F9.2 F14F9.2 0.158783349930853	AGIWUR10319
CB4856	CT	V	5146179 5150464 WBGene00017469	F14F9.6 F14F9.6 0.385968570969426	AGIWUR44128
CB4856	CT	V	5151180 5152982 WBGene00005347	srh-130 F14F9.1 0.175330996025302
AGIWUR15975;AGIWUR23770;AGIWUR45119
CB4856	CT	V	5154057 5155516 WBGene00005929	srx-38	C03A7.5 0.115803541652703	AGIWUR24941
CB4856	CT	V	5156653 5157760 WBGene00005930	srx-39	C03A7.6 0.0690932162666495
AGIWUR7660;AGIWUR15660

CB4856 CT V 5163269 5194608 WBGene00000031 abu-8 C03A7.14 -0.346597314078693
AGIWUR5374;AGIWUR13664;AGIWUR21378;AGIWUR26006;AGIWUR37231;AGIWUR39433;AGIWUR43459
CB4856 CT V 5195926 5198340 WBGene00021614 Y47A7.2 Y47A7.2 -0.0855109415192583 AGIWUR32030
CB4856 CT V 5204481 5213468 WBGene00021613 Y47A7.1 Y47A7.1 -0.0599792720650889 AGIWUR43172
CB4856 CT V 5223423 5230471 WBGene00021732 Y49G5B.1 Y49G5B.1 0.00606440948718401
AGIWUR10915;AGIWUR30491;AGIWUR42762
CB4856 CT V 5230475 5233891 WBGene00016862 C52A10.1 C52A10.1 -0.518748838708484
AGIWUR38429
CB4856 CT V 5237417 5239440 WBGene00016864 C52A10.3 C52A10.3 -0.11788134252604
AGIWUR9942;AGIWUR17072;AGIWUR18741;AGIWUR27518;AGIWUR32753;AGIWUR35543
CB4856 CT V 5240203 5240451 WBGene00044537 B0238.15 B0238.15 -0.453723195241483
AGIWUR20789
CB4856 CT V 5240768 5243136 WBGene00015067 B0238.1 B0238.1 0.0612010635704081 AGIWUR23849
CB4856 CT V 5243815 5248021 WBGene00015077 B0238.13 B0238.13 -0.198284956780475
AGIWUR7988;AGIWUR21729;AGIWUR22553;AGIWUR24752;AGIWUR44017
CB4856 CT V 5249037 5249873 WBGene00015078 srt-66 B0238.14 0.0785333902222032
AGIWUR4331
CB4856 CT V 5250604 5252116 WBGene00015068 srt-68 B0238.3 0.104142868107338
AGIWUR6333;AGIWUR7946;AGIWUR27879
CB4856 CT V 5256142 5257458 WBGene00015069 srt-67 B0238.5 -0.0160668758603446 AGIWUR13781
CB4856 CT V 5258036 5259548 WBGene00015070 srt-70 B0238.6 0.0227233778218787
AGIWUR7541;AGIWUR44643
CB4856 CT V 5260332 5263099 WBGene00015071 B0238.7 B0238.7 0.104819475591065
AGIWUR18267;AGIWUR28110
CB4856 CT V 5263951 5265776 WBGene00015072 srt-69 B0238.8 0.0410069533310269 AGIWUR1864
CB4856 CT V 5267822 5268823 WBGene00015076 B0238.12 B0238.12 0.0189794033473856
AGIWUR2000;AGIWUR4711;AGIWUR8300;AGIWUR10378;AGIWUR21191;AGIWUR31864
CB4856 CT V 5269485 5270744 WBGene00015075 B0238.11 B0238.11 -0.0108918363553183
AGIWUR5198;AGIWUR8055;AGIWUR32992
CB4856 CT V 5274074 5275615 WBGene00015074 natc-2 B0238.10 -0.00108896910946399
AGIWUR18864
CB4856 CT V 5275554 5278101 WBGene00015073 B0238.9 B0238.9 0.0315561875651866 AGIWUR26181
CB4856 CT V 5286407 5287360 WBGene00021731 Y49G5A.1 Y49G5A.1 -0.565422938431686
AGIWUR10627
CB4856 CT V 5303689 5311246 WBGene00003567 ncx-2 C10G8.5 0.0254442259386458
AGIWUR16673;AGIWUR28589;AGIWUR39724;AGIWUR40302
CB4856 CT V 5311935 5312349 WBGene00015683 C10G8.4 C10G8.4 -0.349134776194154
AGIWUR16031;AGIWUR16827;AGIWUR17490
CB4856 CT V 5312890 5314289 WBGene00015682 C10G8.3 C10G8.3 -0.0140542710422444 AGIWUR6462

CB4856	CT	V	5314404	5315589	WBGene00015681	C10G8.2	C10G8.2	0.0602336533166943	AGIWUR37429
CB4856	CT	V	5315513	5319577	WBGene00000455	ceh-34	C10G8.6	0.658027836100082	AGIWUR37117
CB4856	CT	V	5323204	5324616	WBGene00000454	ceh-33	C10G8.7	-0.207094489672176	AGIWUR5584
CB4856	CT	V	5324806	5328470	WBGene00015684	C10G8.8	C10G8.8	-0.540526657754932	AGIWUR1144;AGIWUR21620
CB4856	CT	V	5331267	5333055	WBGene00005183	srg-26	C10G8.1	0.240581843053198	AGIWUR38452
CB4856	CT	V	5334888	5336329	WBGene00005185	srg-28	T09D3.2	-0.268691543436898	AGIWUR7914
CB4856	CT	V	5337184	5339013	WBGene00005186	srg-29	T09D3.6	0.208104050155981	AGIWUR18570
CB4856	CT	V	5339797	5341309	WBGene00005184	srg-27	T09D3.7	-0.0130376275267512	AGIWUR20874
CB4856	CT	V	5342139	5344365	WBGene00005182	srg-25	T09D3.5	0.313093130999375	AGIWUR33862
CB4856	CT	V	5346619	5349571	WBGene00020384	T09D3.3	T09D3.3	0.598217860175815	AGIWUR21034
CB4856	CT	V	5351783	5353067	WBGene00005087	srd-9	T09D3.1	0.171449232263779	AGIWUR34801
CB4856	CT	V	5354062	5358225	WBGene00020385	nhr-216	T09D3.4	0.12247593272351	AGIWUR17754
CB4856	CT	V	5365163	5366792	WBGene00044538	T09D3.8	T09D3.8	-0.249972585150639	AGIWUR11112;AGIWUR28461;AGIWUR31607
CB4856	CT	V	5372418	5377500	WBGene00016430	C35A11.3	C35A11.3	-0.0521284195547569	AGIWUR6176
CB4856	CT	V	5386110	5389409	WBGene00016429	C35A11.2	C35A11.2	0.0493079046784493	AGIWUR35099
CB4856	CT	V	5389480	5393148	WBGene00016431	C35A11.4	C35A11.4	-0.582369318499101	AGIWUR5955
CB4856	CT	V	5405358	5408386	WBGene00016428	dmsr-7	C35A11.1	0.0621843929574215	AGIWUR31595
CB4856	CT	V	5410955	5413001	WBGene00017262	F08F3.4	F08F3.4	-0.325850802874696	AGIWUR23133
CB4856	CT	V	5413320	5415073	WBGene00017266	F08F3.10	F08F3.10	0.145167443773799	AGIWUR16702
CB4856	CT	V	5418044	5420067	WBGene00017263	F08F3.6	F08F3.6	-0.220410630612015	AGIWUR143;AGIWUR10790;AGIWUR29581
CB4856	CT	V	5421018	5423488	WBGene00000373	cyp-14A5	F08F3.7	-0.589519519459778	AGIWUR43753
CB4856	CT	V	5428160	5430431	WBGene00004358	rhr-1	F08F3.3	-0.323068806485337	AGIWUR15061;AGIWUR22455;AGIWUR28598;AGIWUR30549;AGIWUR31196;AGIWUR43016
CB4856	CT	V	5431316	5432557	WBGene00017264	F08F3.8	F08F3.8	0.0938284432447869	AGIWUR15174
CB4856	CT	V	5433392	5437066	WBGene00017261	acl-6	F08F3.2	-0.0550713229792485	AGIWUR43644
CB4856	CT	V	5437084	5439161	WBGene00017265	snpc-1.3	F08F3.9	0.13900633060492	AGIWUR14435
CB4856	CT	V	5446138	5448721	WBGene00017260	F08F3.1	F08F3.1	0.00933486610239598	AGIWUR13926;AGIWUR43486
CB4856	CT	V	5449287	5453715	WBGene00001593	glc-3	ZC317.3	-0.137442808851178	AGIWUR2075

CB4856	CT	V	5460106	5461438	WBGene00022588	ZC317.2	ZC317.2	-0.461276744375966	AGIWUR27671
CB4856	CT	V	5461528	5462994	WBGene00005224	srg-67	ZC317.4	0.0478249783286699	AGIWUR10677
CB4856	CT	V	5463791	5465435	WBGene00005225	srg-68	ZC317.5	-0.00554957419497004	AGIWUR23244
CB4856	CT	V	5471779	5473144	WBGene00022587	ZC317.1	ZC317.1	-0.19993422753895	AGIWUR23867
CB4856	CT	V	5480750	5482415	WBGene00005223	srg-66	T05C3.7	0.198617161433519	AGIWUR30145
CB4856	CT	V	5483086	5484966	WBGene00005222	srg-65	T05C3.8	-0.106878188698603	AGIWUR24244
CB4856	CT	V	5485697	5488866	WBGene00001727	grl-18	T05C3.4	-0.407770595835688	AGIWUR3326
CB4856	CT	V	5490370	5493646	WBGene00020256	T05C3.6	T05C3.6	-0.329781704537664	AGIWUR20647
CB4856	CT	V	5493747	5503010	WBGene00020254	T05C3.2	T05C3.2	-0.0278004685248172	AGIWUR30515
CB4856	CT	V	5503033	5504856	WBGene00001037	dnj-19	T05C3.5	-0.148303588522143	AGIWUR6756;AGIWUR15692;AGIWUR18960;AGIWUR39985;AGIWUR40578;AGIWUR44931
CB4856	CT	V	5505253	5509956	WBGene00003637	nhr-47	C24G6.4	0.0788849903514093	AGIWUR39007;AGIWUR40807
CB4856	CT	V	5509934	5513830	WBGene00016061	hpo-15	C24G6.6	0.527712404455201	AGIWUR5425;AGIWUR7430;AGIWUR10964
CB4856	CT	V	5514348	5520458	WBGene00016060	mms-19	C24G6.3	0.272297308086522	AGIWUR12501
CB4856	CT	V	5523727	5530733	WBGene00016059	C24G6.2	C24G6.2	0.0849734922910592	AGIWUR41996
CB4856	CT	V	5530725	5533845	WBGene00001710	grl-1	C24G6.7	0.318457071331856	AGIWUR32944
CB4856	CT	V	5534101	5536243	WBGene00016062	C24G6.8	C24G6.8	-0.166245840058128	AGIWUR4807;AGIWUR34241;AGIWUR34262
CB4856	CT	V	5536681	5537713	WBGene00006376	syp-2	C24G6.1	-0.202743945084052	AGIWUR15027
CB4856	CT	V	5538800	5545766	WBGene00000477	cft-1	C18C4.2	-0.272696293700065	AGIWUR14907
CB4856	CT	V	5545669	5548073	WBGene00015965	ugt-48	C18C4.3	-0.421678976371881	AGIWUR1728;AGIWUR19654;AGIWUR22191
CB4856	CT	V	5550315	5557281	WBGene00015966	C18C4.5	C18C4.5	0.533973525029577	AGIWUR35146
CB4856	CT	V	5558890	5564298	WBGene00015964	glb-5	C18C4.1	0.390886729682853	AGIWUR36782
CB4856	CT	V	5564306	5567219	WBGene00015968	C18C4.7	C18C4.7	-0.0415938697072038	AGIWUR6245;AGIWUR44207
CB4856	CT	V	5568209	5570533	WBGene00015969	glb-6	C18C4.9	-0.00369444843727518	AGIWUR5075;AGIWUR34571;AGIWUR42521
CB4856	CT	V	5575410	5581713	WBGene00002215	klc-2	C18C4.10	-0.0452518578434669	AGIWUR16149;AGIWUR18996;AGIWUR28335;AGIWUR33837
CB4856	CT	V	5583161	5584343	WBGene00003927	pas-6	CD4.6	-0.160792208527667	AGIWUR23093
CB4856	CT	V	5585182	5586500	WBGene00044542	CD4.11	CD4.11	0.0629059332782447	AGIWUR10838
CB4856	CT	V	5589652	5591838	WBGene00016991	CD4.5	CD4.5	0.277827345893552	AGIWUR15443
CB4856	CT	V	5592685	5593612	WBGene00016990	vps-37	CD4.4	0.0905611426675357	AGIWUR41592
CB4856	CT	V	5593706	5594499	WBGene00016989	CD4.3	CD4.3	-0.17627775875145	AGIWUR5478;AGIWUR13375
CB4856	CT	V	5594615	5595842	WBGene00000795	crn-2	CD4.2	-0.0135773635997971	AGIWUR2005

CB4856	CT	V	5595653 5596551 WBGene00016992	zhit-1	CD4.7	-0.0903555517955397
AGIWUR26205;AGIWUR29405;AGIWUR29560
CB4856	CT	V	5597061 5597450 WBGene00022970	CD4.10	CD4.10	-0.118033880109085
AGIWUR2884;AGIWUR10510;AGIWUR37326;AGIWUR37399;AGIWUR38444
CB4856	CT	V	5606555 5609368 WBGene00016988	CD4.1	CD4.1	-0.122303089382904
AGIWUR3887;AGIWUR12184;AGIWUR16190;AGIWUR22946
CB4856	CT	V	5609414 5613219 WBGene00019756	M03E7.4 M03E7.4 0.0891091173124972
AGIWUR1269;AGIWUR7806;AGIWUR36925
CB4856	CT	V	5617601 5618319 WBGene00019754	M03E7.2 M03E7.2 0.193727327057231	AGIWUR15374
CB4856	CT	V	5618291 5620140 WBGene00019757	memb-2	M03E7.5 -0.19218850162976	AGIWUR23665
CB4856	CT	V	5623642 5624637 WBGene00019753	M03E7.1 M03E7.1 0.126415732374511	AGIWUR18874
CB4856	CT	V	5624727 5630892 WBGene00000071	acy-4	T01C2.1 -0.342205769471381	AGIWUR6301
CB4856	CT	V	5644487 5646231 WBGene00006008	srx-117 C14C11.5	-0.0510791770790959
AGIWUR26107;AGIWUR33030;AGIWUR41327
CB4856	CT	V	5647564 5651040 WBGene00015768	C14C11.4	C14C11.4	-0.0599596154637467
AGIWUR5796
CB4856	CT	V	5654449 5658699 WBGene00015767	hex-2	C14C11.3	0.134160394738384
AGIWUR28993
CB4856	CT	V	5658713 5660704 WBGene00003507	mut-14	C14C11.6	-0.275777462619033
AGIWUR17638
CB4856	CT	V	5665242 5666082 WBGene00015769	C14C11.7	C14C11.7	-0.132766690577494
AGIWUR15135
CB4856	CT	V	5669611 5676111 WBGene00004104	pqn-13	C14C11.8	0.83001969656992
AGIWUR30520
CB4856	CT	V	5678600 5682630 WBGene00017768	F25B4.5 F25B4.5 0.100092711687097	AGIWUR37076
CB4856	CT	V	5682721 5684589 WBGene00017769	hmgs-1	F25B4.6 -0.126230508214242	AGIWUR30393
CB4856	CT	V	5684875 5685728 WBGene00017767	F25B4.4 F25B4.4 -0.371579713085341	AGIWUR17106
CB4856	CT	V	5685726 5687381 WBGene00017770	F25B4.7 F25B4.7 -0.132889908376923
AGIWUR7148;AGIWUR16812;AGIWUR20659;AGIWUR31628;AGIWUR32518;AGIWUR34484
CB4856	CT	V	5690103 5691715 WBGene00017771	F25B4.8 F25B4.8 -0.348876045598351
AGIWUR8731;AGIWUR13190;AGIWUR19738;AGIWUR20436;AGIWUR21867;AGIWUR31668
CB4856	CT	V	5700010 5701080 WBGene00017772	clec-1	F25B4.9 -0.209668759025771	AGIWUR31369
CB4856	CT	V	5709418 5712335 WBGene00017766	peli-1	F25B4.2 0.070439991792262
AGIWUR7805;AGIWUR31214
CB4856	CT	V	5712818 5714756 WBGene00017765	gcst-1	F25B4.1 -0.33478150899461
AGIWUR11264;AGIWUR16183;AGIWUR16623
CB4856	CT	V	5714760 5716581 WBGene00044539	K09H11.9	K09H11.9	0.369854909323157
AGIWUR370;AGIWUR8555;AGIWUR16863
CB4856	CT	V	5723079 5726545 WBGene00019601	K09H11.4	K09H11.4	-0.199416262346309

AGIWUR37085
CB4856 CT V 5727290 5729593 WBGene00006015 srx-124 K09H11.8 -0.294659260881565
AGIWUR20319
CB4856 CT V 5731312 5734595 WBGene00006014 srx-123 K09H11.2 0.048284061120671
AGIWUR35596
CB4856 CT V 5734844 5735984 WBGene00019603 K09H11.6 K09H11.6 -0.125706376767083
AGIWUR198;AGIWUR28113
CB4856 CT V 5747723 5752033 WBGene00019599 K09H11.1 K09H11.1 0.0836140854588354
AGIWUR6658;AGIWUR25254;AGIWUR40166
CB4856 CT V 5754670 5755558 WBGene00044541 K09H11.11 K09H11.11 -0.201869222743476
AGIWUR3096;AGIWUR11398;AGIWUR36361;AGIWUR37461;AGIWUR41423
CB4856 CT V 5758224 5760232 WBGene00016891 C53A3.1 C53A3.1 -0.195855069156018
AGIWUR3869;AGIWUR8455;AGIWUR10857
CB4856 CT V 5770456 5771767 WBGene00018424 F44E7.2 F44E7.2 0.267265173571372
AGIWUR39531;AGIWUR43941
CB4856 CT V 5771945 5772497 WBGene00018425 F44E7.3 F44E7.3 0.308141520987474 AGIWUR27519
CB4856 CT V 5772831 5777034 WBGene00018426 F44E7.4 F44E7.4 -0.174374787170077
AGIWUR6258;AGIWUR7214;AGIWUR10257;AGIWUR17718;AGIWUR37927
CB4856 CT V 5778818 5780947 WBGene00018427 F44E7.5 F44E7.5 0.0261650465976224
AGIWUR4722;AGIWUR5044;AGIWUR5808;AGIWUR15449;AGIWUR16199;AGIWUR28981
CB4856 CT V 5781317 5782787 WBGene00018428 fbxa-183 F44E7.6 -0.311670501638298
AGIWUR22543
CB4856 CT V 5782893 5785648 WBGene00018429 F44E7.7 F44E7.7 -0.304599993690513 AGIWUR13903
CB4856 CT V 5785959 5790066 WBGene00018430 nhr-142 F44E7.8 0.158086948730407
AGIWUR35477;AGIWUR37111
CB4856 CT V 5791230 5792122 WBGene00018431 F44E7.9 F44E7.9 -0.0375419675288384 AGIWUR43388
CB4856 CT V 5792331 5794857 WBGene00022576 ZC250.2 ZC250.2 -0.145075400171663 AGIWUR34319
CB4856 CT V 5794688 5798232 WBGene00022577 nstp-3 ZC250.3 0.34310684723326 AGIWUR3913
CB4856 CT V 5799673 5805666 WBGene00000893 cyn-17 ZC250.1 0.267923422542339
AGIWUR16547;AGIWUR26654
CB4856 CT V 5805767 5807532 WBGene00045306 ZC250.5 ZC250.5 -0.146999300507041 AGIWUR35199
CB4856 CT V 5812316 5813157 WBGene00045305 ZC250.4 ZC250.4 -0.368760029193141
AGIWUR1578;AGIWUR13681;AGIWUR26046;AGIWUR43378
CB4856 CT V 5826833 5832866 WBGene00002637 let-418 F26F12.7 -0.0796180022066224
AGIWUR6725;AGIWUR13247;AGIWUR14742;AGIWUR30477;AGIWUR34699;AGIWUR43629
CB4856 CT V 5833831 5835183 WBGene00005297 srh-76 F26F12.6 -0.0672816810986356
AGIWUR2120
CB4856 CT V 5835855 5836871 WBGene00000713 col-140 F26F12.1 0.0139327661816324
AGIWUR18993

CB4856 CT V 5837572 5838613 WBGene00017833 F26F12.2 F26F12.2 -0.136559739051241
AGIWUR15256
CB4856 CT V 5838960 5845739 WBGene00017834 F26F12.3 F26F12.3 0.894639439256697
AGIWUR290;AGIWUR8936;AGIWUR9237;AGIWUR16429;AGIWUR17239;AGIWUR19312;AGIWUR28755;AGIWUR42064
CB4856 CT V 5854848 5856414 WBGene00044544 F26F12.8 F26F12.8 -0.374996582520359
AGIWUR1899;AGIWUR8610;AGIWUR12476;AGIWUR24453;AGIWUR34690
CB4856 CT V 5863984 5865149 WBGene00017836 F26F12.5 F26F12.5 0.0931011583126864
AGIWUR289;AGIWUR1983;AGIWUR2375;AGIWUR2704;AGIWUR33158
CB4856 CT V 5866134 5867952 WBGene00017835 F26F12.4 F26F12.4 0.572971619736153
AGIWUR9893
CB4856 CT V 5872084 5874914 WBGene00015360 C02G6.2 C02G6.2 0.0163547344702746
AGIWUR18549;AGIWUR32969;AGIWUR35538;AGIWUR39767
CB4856 CT V 5876370 5879942 WBGene00015359 C02G6.1 C02G6.1 -0.421869406391117 AGIWUR15967
CB4856 CT V 5881052 5882450 WBGene00044803 C02G6.3 C02G6.3 -0.107034789189628
AGIWUR3176;AGIWUR25574;AGIWUR28650
CB4856 CT V 5882665 5883835 WBGene00015424 C04E6.8 C04E6.8 -0.254295500343253
AGIWUR2858;AGIWUR5076;AGIWUR11028;AGIWUR19894;AGIWUR31647;AGIWUR40369
CB4856 CT V 5893312 5895208 WBGene00015423 C04E6.7 C04E6.7 -0.0786516192209454
AGIWUR2831;AGIWUR4042;AGIWUR17377
CB4856 CT V 5895388 5898602 WBGene00001698 grd-9 C04E6.6 -0.401929604110414 AGIWUR37827
CB4856 CT V 5902133 5904831 WBGene00015422 C04E6.5 C04E6.5 -0.159805458850736 AGIWUR16915
CB4856 CT V 5904917 5906352 WBGene00005093 srd-15 C04E6.10 0.0731631361532941
AGIWUR25561
CB4856 CT V 5907408 5909620 WBGene00015421 C04E6.4 C04E6.4 -0.160162772626064 AGIWUR38659
CB4856 CT V 5912726 5913757 WBGene00015427 C04E6.13 C04E6.13 0.266241075914539
AGIWUR30644
CB4856 CT V 5914097 5917891 WBGene00015425 C04E6.11 C04E6.11 -0.0361379169328204
AGIWUR4820;AGIWUR13300;AGIWUR16133;AGIWUR18878;AGIWUR25001;AGIWUR33865;AGIWUR34856;AGIWUR41339
CB4856 CT V 5918062 5919835 WBGene00015426 C04E6.12 C04E6.12 0.285553693225247
AGIWUR19866
CB4856 CT V 5920121 5920395 WBGene00015420 C04E6.3 C04E6.3 0.0446889302777951
AGIWUR4955;AGIWUR10644;AGIWUR19172;AGIWUR27814;AGIWUR40512
CB4856 CT V 5922545 5924297 WBGene00015419 srsx-18 C04E6.2 -0.128360820491489 AGIWUR18814
CB4856 CT V 5927647 5930417 WBGene00019032 srsx-17 F58D7.1 -0.103735413592544 AGIWUR41420
CB4856 CT V 5930847 5933435 WBGene00019763 galt-1 M03F8.4 -0.215957404690588
AGIWUR21765;AGIWUR37492
CB4856 CT V 5933662 5936791 WBGene00019764 M03F8.5 M03F8.5 0.0759959554219538 AGIWUR31881
CB4856 CT V 5940024 5943362 WBGene00019762 M03F8.3 M03F8.3 -0.150809228425832
AGIWUR17052;AGIWUR27293

CB4856	CT	V	5943474	5950991	WBGene00004206	pst-1	M03F8.2	-0.0843035110078372	AGIWUR2524;AGIWUR6470;AGIWUR8666;AGIWUR11767;AGIWUR17252;AGIWUR22609
CB4856	CT	V	5951433	5953872	WBGene00019761	M03F8.1	M03F8.1	-0.14549522597018	AGIWUR14350
CB4856	CT	V	5953831	5954840	WBGene00019765	M03F8.6	M03F8.6	-0.0621084994644798	AGIWUR29423;AGIWUR41081
CB4856	CT	V	5960585	5963882	WBGene00003954	pcm-1	C10F3.5	-0.135381000527351	AGIWUR9721;AGIWUR22533;AGIWUR32790;AGIWUR39510
CB4856	CT	V	5961813	5969175	WBGene00015678	mcp-1	C10F3.4	0.0203753485014975	AGIWUR6540
CB4856	CT	V	5976029	5981513	WBGene00000069	acy-2	C10F3.3	-0.165847639042164	AGIWUR29420
CB4856	CT	V	5983745	5985473	WBGene00000979	dhs-16	C10F3.2	-0.297749212422856	AGIWUR25144
CB4856	CT	V	5985440	5986855	WBGene00015679	C10F3.7	C10F3.7	0.536176910846205	AGIWUR30945
CB4856	CT	V	5987003	5990252	WBGene00001508	fut-8	C10F3.6	-0.427612462465776	AGIWUR8362
CB4856	CT	V	5996323	5999344	WBGene00015677	cpg-4	C10F3.1	-0.0193876802767168	AGIWUR29999
CB4856	CT	V	6003601	6016087	WBGene00001345	fos-1	F29G9.4	0.247836347177595	AGIWUR34164
CB4856	CT	V	6016049	6017669	WBGene00004502	rpt-2	F29G9.5	-0.0141247640667785	AGIWUR8467
CB4856	CT	V	6017970	6018886	WBGene00000159	aps-1	F29G9.3	-0.211918880020718	AGIWUR5131;AGIWUR10646;AGIWUR26206
CB4856	CT	V	6018994	6026337	WBGene00017931	picc-1	F29G9.2	0.0706037174182539	AGIWUR14915;AGIWUR30183;AGIWUR39248
CB4856	CT	V	6026330	6034923	WBGene00000980	dhs-17	F29G9.6	0.108114938171546	AGIWUR9125
CB4856	CT	V	6041196	6043185	WBGene00019804	R01B10.2	R01B10.2	-0.0856006204880781	AGIWUR39768
CB4856	CT	V	6044028	6045671	WBGene00000534	cpi-2	R01B10.1	-0.0998595282056369	AGIWUR363;AGIWUR9475;AGIWUR13614;AGIWUR15479;AGIWUR17723;AGIWUR37763;AGIWUR40451;AGIWUR43366
CB4856	CT	V	6045640	6047908	WBGene00019806	R01B10.4	R01B10.4	0.186815433712555	AGIWUR5448
CB4856	CT	V	6047922	6051259	WBGene00019807	jamp-1	R01B10.5	-0.0485909600968659	AGIWUR6308;AGIWUR24410;AGIWUR39284;AGIWUR42295
CB4856	CT	V	6055589	6056743	WBGene00005098	srd-20	Y38A10A.1	-0.0339820977141114	AGIWUR5427
CB4856	CT	V	6057366	6063430	WBGene00021389	Y38A10A.2	Y38A10A.2	0.264846401500821	AGIWUR24984
CB4856	CT	V	6065507	6067089	WBGene00005236	srh-10	Y38A10A.3	-0.0415349375958618	AGIWUR32924
CB4856	CT	V	6067811	6070068	WBGene00021390	fbxa-214	Y38A10A.4	-0.0479707761707742	AGIWUR4835;AGIWUR25614;AGIWUR35294
CB4856	CT	V	6096028	6096903	WBGene00021392	Y38A10A.7	Y38A10A.7	-0.152899283950934	AGIWUR40565
CB4856	CT	V	6097105	6099083	WBGene00021391	smut-1	Y38A10A.6	0.164786462991721	

AGIWUR34126
CB4856 CT V 6098993 6100553 WBGene00000802 crt-1 Y38A10A.5 -0.0108111415871548
AGIWUR5761;AGIWUR21954;AGIWUR27741;AGIWUR42525;AGIWUR42873
CB4856 CT V 6105607 6107530 WBGene00017535 atf-8 F17A9.3 0.637234671122197 AGIWUR8129
CB4856 CT V 6107828 6114856 WBGene00017534 F17A9.2 F17A9.2 -0.217911993993901 AGIWUR1903
CB4856 CT V 6116521 6117849 WBGene00017533 fbxa-178 F17A9.1 -0.0118887127272664
AGIWUR12696;AGIWUR27014;AGIWUR43258
CB4856 CT V 6118332 6120206 WBGene00017538 ceh-49 F17A9.6 -0.540530212143024 AGIWUR6485
CB4856 CT V 6120909 6202626 WBGene00006436 ttn-1 W06H8.8 -0.0149779430888248
AGIWUR467;AGIWUR2055;AGIWUR18482;AGIWUR20833;AGIWUR24654;AGIWUR27263;AGIWUR27556;AGIWUR28812;AGIWUR29196;AG
IWUR30343;AGIWUR31761;AGIWUR35868;AGIWUR38312;AGIWUR39023;AGIWUR40449
CB4856 CT V 6204875 6206134 WBGene00021067 W06H8.4 W06H8.4 -0.0784053898707908 AGIWUR32308
CB4856 CT V 6206755 6208551 WBGene00021066 W06H8.2 W06H8.2 -0.00385488057658101 AGIWUR36532
CB4856 CT V 6208840 6217647 WBGene00004373 rme-1 W06H8.1 -0.324797174156712
AGIWUR7403;AGIWUR21968;AGIWUR28544;AGIWUR35361;AGIWUR35611;AGIWUR40187
CB4856 CT V 6218134 6219562 WBGene00006239 str-206 W06H8.7 -0.136833249738677 AGIWUR20590
CB4856 CT V 6221249 6227047 WBGene00021068 W06H8.6 W06H8.6 0.0923002369725481 AGIWUR39330
CB4856 CT V 6235176 6237807 WBGene00019522 K08B12.3 K08B12.3 -0.136906840383212
AGIWUR32080
CB4856 CT V 6249172 6252453 WBGene00019521 dmd-7 K08B12.2 0.203454886284943
AGIWUR4928;AGIWUR11191;AGIWUR24510;AGIWUR30182;AGIWUR41338
CB4856 CT V 6268565 6270481 WBGene00017966 F31F7.3 F31F7.3 0.134690717056017 AGIWUR14173
CB4856 CT V 6270852 6274911 WBGene00017965 F31F7.2 F31F7.2 -0.156656374158782 AGIWUR26123
CB4856 CT V 6276439 6278160 WBGene00017964 F31F7.1 F31F7.1 0.843139817255935
AGIWUR7941;AGIWUR24895;AGIWUR30996
CB4856 CT V 6279005 6280146 WBGene00019668 fil-2 K12B6.3 0.759702504484828 AGIWUR22934
CB4856 CT V 6280380 6281136 WBGene00019669 K12B6.4 K12B6.4 0.232103325030634 AGIWUR1920
CB4856 CT V 6281359 6284865 WBGene00019667 K12B6.2 K12B6.2 -0.0560021725461413
AGIWUR5809;AGIWUR7245;AGIWUR34843
CB4856 CT V 6284920 6286434 WBGene00005640 srn-1 K12B6.5 0.0963453689718676 AGIWUR6389
CB4856 CT V 6291390 6292666 WBGene00023432 K12B6.9 K12B6.9 0.334349271252766
AGIWUR4049;AGIWUR4367;AGIWUR38219
CB4856 CT V 6293920 6294342 WBGene00019670 K12B6.6 K12B6.6 0.04918020924414 AGIWUR36632
CB4856 CT V 6303771 6308337 WBGene00019666 sago-1 K12B6.1 -0.0230929535349151 AGIWUR3629
CB4856 CT V 6308811 6312001 WBGene00019672 K12B6.8 K12B6.8 -0.184234288153948
AGIWUR2931;AGIWUR42737
CB4856 CT V 6314176 6316467 WBGene00020892 T28C12.5 T28C12.5 0.112576754288265
AGIWUR9229
CB4856 CT V 6316932 6326390 WBGene00020891 T28C12.4 T28C12.4 0.303425707412657

AGIWUR30512
CB4856 CT V 6327223 6328865 WBGene00020890 fbxa-202 T28C12.3 0.0689845544221974
AGIWUR33622
CB4856 CT V 6329197 6331074 WBGene00020889 T28C12.2 T28C12.2 -0.271814842082676
AGIWUR3720
CB4856 CT V 6331907 6334008 WBGene00020888 T28C12.1 T28C12.1 0.0944608646772078
AGIWUR19956
CB4856 CT V 6334014 6336119 WBGene00020893 T28C12.6 T28C12.6 -0.192125510100341
AGIWUR4707
CB4856 CT V 6356025 6362997 WBGene00017430 bcl-11 F13H6.1 0.126500609926369
AGIWUR5640;AGIWUR13167;AGIWUR19155;AGIWUR27208;AGIWUR28028
CB4856 CT V 6363408 6365671 WBGene00017431 F13H6.3 F13H6.3 -0.229467415702727 AGIWUR2859
CB4856 CT V 6366433 6368533 WBGene00017432 F13H6.4 F13H6.4 0.364166210503674 AGIWUR19816
CB4856 CT V 6372114 6374933 WBGene00017433 F13H6.5 F13H6.5 1.26645546516535 AGIWUR29519
CB4856 CT V 6386550 6389734 WBGene00019016 F57F4.2 F57F4.2 -0.0866880024949079 AGIWUR38900
CB4856 CT V 6390463 6392523 WBGene00019015 F57F4.1 F57F4.1 -0.132262714600676
AGIWUR39573;AGIWUR44431
CB4856 CT V 6414993 6417494 WBGene00020270 T05H4.7 T05H4.7 -0.0843535541192404
AGIWUR11848;AGIWUR13753;AGIWUR27899
CB4856 CT V 6417495 6424155 WBGene00020273 T05H4.10 T05H4.10 0.0263700871939485
AGIWUR14056;AGIWUR28336
CB4856 CT V 6424252 6425952 WBGene00020274 T05H4.11 T05H4.11 -0.0413338368557596
AGIWUR4559
CB4856 CT V 6426193 6427112 WBGene00020275 atp-4 T05H4.12 -0.112915068485779
AGIWUR11354;AGIWUR39237
CB4856 CT V 6427212 6429927 WBGene00000110 alh-4 T05H4.13 0.0763433098374442
AGIWUR2450;AGIWUR15252;AGIWUR16978;AGIWUR26494;AGIWUR42388
CB4856 CT V 6431331 6433496 WBGene00001513 gad-1 T05H4.14 -0.186327457878139
AGIWUR27490
CB4856 CT V 6434130 6436401 WBGene00020269 erfa-1 T05H4.6 -0.0452006384099484
AGIWUR19051;AGIWUR41329
CB4856 CT V 6436578 6437884 WBGene00020268 hpo-19 T05H4.5 -0.079789511212498
AGIWUR2866;AGIWUR12634;AGIWUR23152;AGIWUR29714;AGIWUR29872
CB4856 CT V 6438913 6440036 WBGene00020267 T05H4.4 T05H4.4 -0.0028733463380739 AGIWUR7136
CB4856 CT V 6440202 6441711 WBGene00020276 T05H4.15 T05H4.15 0.341066056912105
AGIWUR9764;AGIWUR14615;AGIWUR31804
CB4856 CT V 6442337 6447556 WBGene00020266 T05H4.3 T05H4.3 0.0888230284825813 AGIWUR10846
CB4856 CT V 6448656 6450217 WBGene00020265 fbxa-196 T05H4.2 0.0709498373470616
AGIWUR11813

CB4856	CT	V	6450898	6452231	WBGene00020264	acl-8	T05H4.1	0.0382158693508181	AGIWUR9693
CB4856	CT	V	6453196	6454973	WBGene00022646	acl-9	ZK40.1	-0.080889846512048	AGIWUR1627;AGIWUR3340;AGIWUR5728;AGIWUR8126;AGIWUR14958;AGIWUR27729;AGIWUR36534
CB4856	CT	V	6465065	6487880	WBGene00006815	unc-83	W01A11.3	0.398831755380371	AGIWUR16191;AGIWUR27240;AGIWUR35896;AGIWUR39953
CB4856	CT	V	6488203	6489120	WBGene00002273	lec-10	W01A11.4	-0.486564454864258	AGIWUR24405
CB4856	CT	V	6490068	6493921	WBGene00020911	cpt-6	W01A11.5	-0.0518972558276793	AGIWUR6635
CB4856	CT	V	6497110	6498484	WBGene00020910	dgtr-1	W01A11.2	0.0657889145073796	AGIWUR41901
CB4856	CT	V	6499224	6502428	WBGene00020909	W01A11.1	W01A11.1	0.344058343939437	AGIWUR19791
CB4856	CT	V	6502369	6504181	WBGene00003385	moc-2	W01A11.6	0.0719720102861343	AGIWUR8181;AGIWUR17085
CB4856	CT	V	6504538	6505500	WBGene00020912	W01A11.7	W01A11.7	-0.0838404406672861	AGIWUR9030;AGIWUR22292
CB4856	CT	V	6506752	6508803	WBGene00006723	ubh-3	Y40G12A.1	-0.0453594999128004	AGIWUR32889
CB4856	CT	V	6508831	6509884	WBGene00006722	ubh-2	Y40G12A.2	-0.0720496915964084	AGIWUR21082
CB4856	CT	V	6509910	6511409	WBGene00006721	ubh-1	F46E10.8	-0.0323066530848433	AGIWUR1295;AGIWUR2582;AGIWUR31493
CB4856	CT	V	6511588	6515240	WBGene00001073	dpy-11	F46E10.9	-0.00727915971378934	AGIWUR4187;AGIWUR9323;AGIWUR27533
CB4856	CT	V	6515276	6516526	WBGene00018491	mdh-1	F46E10.10	-0.0540153634968554	AGIWUR17671;AGIWUR18530;AGIWUR23213;AGIWUR25558
CB4856	CT	V	6520435	6521099	WBGene00018489	F46E10.2	F46E10.2	0.721303336044699	AGIWUR21257;AGIWUR44482
CB4856	CT	V	6524989	6532089	WBGene00018488	acs-1	F46E10.1	-0.328366692431409	AGIWUR5838;AGIWUR11960;AGIWUR12542;AGIWUR35496;AGIWUR41551
CB4856	CT	V	6532071	6533074	WBGene00018492	hpo-26	F46E10.11	-0.0562115773232978	AGIWUR13002
CB4856	CT	V	6533614	6546491	WBGene00001089	dre-1	K04A8.6	0.172426482231174	AGIWUR39647
CB4856	CT	V	6550171	6551817	WBGene00019376	lipl-4	K04A8.5	-0.307173506835442	AGIWUR28247
CB4856	CT	V	6552348	6560196	WBGene00006665	twk-10	K04A8.4	-0.224681284278344	AGIWUR20906;AGIWUR24464;AGIWUR32424;AGIWUR34785;AGIWUR35009
CB4856	CT	V	6562048	6564181	WBGene00019374	K04A8.2	K04A8.2	-0.0722160432917107	AGIWUR12303
CB4856	CT	V	6566336	6568747	WBGene00019373	K04A8.1	K04A8.1	-0.0474144460018842	AGIWUR25697

CB4856	CT	V	6573983	6575797	WBGene00001506	fut-2	EGAP9.2	0.271058202901305	AGIWUR15948
CB4856	CT	V	6576867	6577541	WBGene00044545	EGAP9.4	EGAP9.4	0.0768097854079155	AGIWUR3584
CB4856	CT	V	6578185	6580182	WBGene00017151	EGAP9.3	EGAP9.3	0.12041677530709	AGIWUR32024
CB4856	CT	V	6580843	6584390	WBGene00022846	ZK1055.5	ZK1055.5	-0.0410234036198753	AGIWUR17780
CB4856	CT	V	6585632	6587421	WBGene00022845	ZK1055.4	ZK1055.4	-0.203250561547549	AGIWUR17432
CB4856	CT	V	6590583	6593634	WBGene00022843	ZK1055.2	ZK1055.2	0.0612289509890114	AGIWUR3803;AGIWUR37365
CB4856	CT	V	6594194	6599208	WBGene00001829	hcp-1	ZK1055.1	0.10122865480824	AGIWUR35243
CB4856	CT	V	6599190	6602604	WBGene00022847	ZK1055.6	ZK1055.6	-0.179658587185654	AGIWUR6987;AGIWUR12831;AGIWUR22387;AGIWUR34246
CB4856	CT	V	6602728	6605375	WBGene00022848	ZK1055.7	ZK1055.7	-0.214664604994704	AGIWUR2670
CB4856	CT	V	6606439	6611158	WBGene00002148	gon-14	F44C4.4	-0.171075887551463	AGIWUR14569;AGIWUR23270
CB4856	CT	V	6614674	6615845	WBGene00000784	cpr-4	F44C4.3	-0.473470344240802	AGIWUR3924
CB4856	CT	V	6622268	6624694	WBGene00018412	nhr-37	F44C4.2	0.174202025281201	AGIWUR9206
CB4856	CT	V	6624702	6626344	WBGene00004092	ppt-1	F44C4.5	-0.272579623664919	AGIWUR1731;AGIWUR34415;AGIWUR35478
CB4856	CT	V	6633269	6639665	WBGene00004740	scd-2	T10H9.2	0.245554100929912	AGIWUR40660
CB4856	CT	V	6649617	6652050	WBGene00020424	T10H9.1	T10H9.1	-0.267222777037562	AGIWUR5182
CB4856	CT	V	6652353	6653742	WBGene00044779	T10H9.8	T10H9.8	0.593999291350438	AGIWUR8114;AGIWUR33853;AGIWUR35324;AGIWUR44021
CB4856	CT	V	6653613	6655449	WBGene00020425	syx-18	T10H9.3	-0.0463509705305967	AGIWUR31028
CB4856	CT	V	6655562	6656501	WBGene00004897	snb-1	T10H9.4	0.073773847733186	AGIWUR27645
CB4856	CT	V	6662191	6665813	WBGene00004062	pmp-5	T10H9.5	-0.530043462499958	AGIWUR4313;AGIWUR10836;AGIWUR26677;AGIWUR39783
CB4856	CT	V	6667400	6668841	WBGene00006224	str-180	T10H9.6	-0.827378696953406	AGIWUR37919
CB4856	CT	V	6669823	6672036	WBGene00019654	K11G9.3	K11G9.3	-0.763320036980073	AGIWUR27046
CB4856	CT	V	6674267	6676426	WBGene00019653	K11G9.2	K11G9.2	-0.643708010218543	AGIWUR7173;AGIWUR12294;AGIWUR20235
CB4856	CT	V	6676799	6679072	WBGene00019652	K11G9.1	K11G9.1	0.0673901182794339	AGIWUR25997
CB4856	CT	V	6679317	6681573	WBGene00001210	egl-46	K11G9.4	1.39430037286084	AGIWUR43254;AGIWUR44810
CB4856	CT	V	6685807	6688492	WBGene00019655	K11G9.5	K11G9.5	-0.338771501774378	AGIWUR27658
CB4856	CT	V	6691371	6691863	WBGene00003473	mtl-1	K11G9.6	3.46338995179843	AGIWUR30106
CB4856	CT	V	6692247	6694768	WBGene00175027	W02F12.8	W02F12.8	0.014322998950294	

AGIWUR7094;AGIWUR16478;AGIWUR25256;AGIWUR25915;AGIWUR27056
CB4856 CT V 6695147 6697652 WBGene00020947 W02F12.2 W02F12.2 0.0140802628833252
AGIWUR16259;AGIWUR29622
CB4856 CT V 6703057 6704573 WBGene00020948 era-1 W02F12.3 -0.204331115877034
AGIWUR2317;AGIWUR14785
CB4856 CT V 6704551 6706932 WBGene00020949 W02F12.4 W02F12.4 -0.176322083765537
AGIWUR4588;AGIWUR5119;AGIWUR7662;AGIWUR14147;AGIWUR32770;AGIWUR42348;AGIWUR43423;AGIWUR44413
CB4856 CT V 6707058 6709101 WBGene00020950 dlst-1 W02F12.5 0.0665221255929573
AGIWUR25914;AGIWUR28729;AGIWUR41820
CB4856 CT V 6709579 6710679 WBGene00020951 sna-1 W02F12.6 -0.0761576518582547
AGIWUR32989
CB4856 CT V 6714990 6716302 WBGene00023355 W02F12.1 W02F12.1 0.103366761584642
AGIWUR37104
CB4856 CT V 6716855 6718297 WBGene00005187 srg-30 W02F12.7 0.120720503678763
AGIWUR4155;AGIWUR7870
CB4856 CT V 6720397 6723005 WBGene00022624 ZC487.2 ZC487.2 -0.116968903332243 AGIWUR22572
CB4856 CT V 6723466 6723764 WBGene00022625 ZC487.3 ZC487.3 -0.205589006982048 AGIWUR39508
CB4856 CT V 6727812 6730262 WBGene00022623 ZC487.1 ZC487.1 0.0978102537834395
AGIWUR2837;AGIWUR31367;AGIWUR40962
CB4856 CT V 6730281 6731694 WBGene00001718 grl-9 ZC487.4 -0.0326082169737898 AGIWUR23579
CB4856 CT V 6731831 6733092 WBGene00001717 grl-8 ZC487.5 -0.539200263803483 AGIWUR7134
CB4856 CT V 6745039 6748613 WBGene00020806 T25F10.3 T25F10.3 0.0290557540474232
AGIWUR8750
CB4856 CT V 6758135 6760418 WBGene00000936 dbl-1 T25F10.2 0.0488947482275002
AGIWUR23643;AGIWUR28633
CB4856 CT V 6760442 6762461 WBGene00020807 T25F10.4 T25F10.4 -0.147465240767653
AGIWUR39846
CB4856 CT V 6762476 6765072 WBGene00000244 bbs-8 T25F10.5 0.0655183634953485
AGIWUR14361
CB4856 CT V 6765560 6768732 WBGene00020808 clik-1 T25F10.6 0.0494540471384148
AGIWUR2591;AGIWUR4800;AGIWUR6940;AGIWUR20249;AGIWUR35744;AGIWUR37233;AGIWUR37861
CB4856 CT V 6768429 6768788 WBGene00020805 T25F10.1 T25F10.1 0.209354343761392
AGIWUR11123
CB4856 CT V 6769662 6771704 WBGene00005253 srh-30 ZC404.12 0.285548141471329
AGIWUR35108
CB4856 CT V 6774351 6776196 WBGene00005251 srh-28 ZC404.5 0.00546876962925147
AGIWUR20842;AGIWUR25637;AGIWUR33788
CB4856 CT V 6778551 6779700 WBGene00022602 ZC404.7 ZC404.7 -0.143491091803213 AGIWUR31102
CB4856 CT V 6783187 6784922 WBGene00004984 spn-4 ZC404.8 0.0355773354646658 AGIWUR39701

CB4856	CT	V	6785457	6787866	WBGene00004975	spe-39	ZC404.3	-0.434382015732238	AGIWUR16328;AGIWUR19002;AGIWUR26551
CB4856	CT	V	6788449	6792665	WBGene00022603	gck-2	ZC404.9	-0.011622263413722	AGIWUR1270
CB4856	CT	V	6793936	6795130	WBGene00022601	ZC404.1	ZC404.1	0.132899522305356	AGIWUR30969
CB4856	CT	V	6798151	6799664	WBGene00022606	dmsr-9	ZC404.13	0.339352323761604	AGIWUR43059
CB4856	CT	V	6800724	6801950	WBGene00022604	dmsr-10	ZC404.10	0.144458541826972	AGIWUR16456
CB4856	CT	V	6802412	6804096	WBGene00022605	dmsr-11	ZC404.11	0.359445690391986	AGIWUR11218
CB4856	CT	V	6804512	6806440	WBGene00019262	dmsr-12	H34P18.1	-0.0907791987228119	AGIWUR24451
CB4856	CT	V	6810421	6813449	WBGene00020522	T15B7.10	T15B7.10	0.230715706583963	AGIWUR30507
CB4856	CT	V	6813700	6815016	WBGene00020529	T15B7.17	T15B7.17	0.293256756615134	AGIWUR1853
CB4856	CT	V	6815666	6817284	WBGene00020523	dmsr-14	T15B7.11	-0.320306471350102	AGIWUR43501
CB4856	CT	V	6817890	6819912	WBGene00020524	dmsr-13	T15B7.12	0.191669486387214	AGIWUR552
CB4856	CT	V	6821809	6826872	WBGene00020520	T15B7.8	T15B7.8	-0.0344219304485396	AGIWUR2448;AGIWUR29808;AGIWUR37151
CB4856	CT	V	6827146	6828886	WBGene00020525	dmsr-15	T15B7.13	-0.171221105597956	AGIWUR34598
CB4856	CT	V	6829666	6830721	WBGene00000714	col-141	T15B7.5	-0.892059271751625	AGIWUR38706
CB4856	CT	V	6831163	6832256	WBGene00000715	col-142	T15B7.4	0	AGIWUR2207
CB4856	CT	V	6832290	6835342	WBGene00020526	T15B7.14	T15B7.14	-0.782789775670679	AGIWUR1085
CB4856	CT	V	6835635	6836700	WBGene00000716	col-143	T15B7.3	-0.270364673707252	AGIWUR14313;AGIWUR35443
CB4856	CT	V	6836725	6838630	WBGene00020527	T15B7.15	T15B7.15	0.112948105964634	AGIWUR28460
CB4856	CT	V	6839256	6840254	WBGene00020517	hpo-8	T15B7.2	0.0346716338236224	AGIWUR8620;AGIWUR16296;AGIWUR22235
CB4856	CT	V	6840217	6842970	WBGene00020528	lgc-54	T15B7.16	-0.613218534267896	AGIWUR22007
CB4856	CT	V	6842737	6846652	WBGene00020516	T15B7.1	T15B7.1	-0.803598254631982	AGIWUR1514;AGIWUR38289
CB4856	CT	V	6850538	6851900	WBGene00016959	C55H1.1	C55H1.1	-0.129964290443529	AGIWUR44315

CB4856	CT	V	6852568 6854803 WBGene00001672	gpa-10	C55H1.2 -0.104854854899485	AGIWUR35673
CB4856	CT	V	6870304 6873809 WBGene00020586	frpr-18 T19F4.1 0.0257583562887662
AGIWUR16517;AGIWUR27580;AGIWUR38008;AGIWUR43783
CB4856	CT	V	6881340 6891836 WBGene00006803	unc-70	K11C4.3 -0.240572545887786
AGIWUR18280;AGIWUR30869
CB4856	CT	V	6895078 6896922 WBGene00019643	K11C4.2 K11C4.2 -0.0623102170641197	AGIWUR8851
CB4856	CT	V	6896999 6898497 WBGene00019642	K11C4.1 K11C4.1 0.211065112920993	AGIWUR38868
CB4856	CT	V	6898540 6900223 WBGene00003844	odc-1	K11C4.4 -0.725966441231296
AGIWUR2487;AGIWUR11379
CB4856	CT	V	6901697 6929038 WBGene00006801	unc-68	K11C4.5 -0.115621680645065
AGIWUR14992;AGIWUR39287
CB4856	CT	V	6931539 6934288 WBGene00021963	lipl-6	Y57E12B.3	0.242251100716075
AGIWUR4700
CB4856	CT	V	6945711 6947547 WBGene00021964	Y57E12B.4	Y57E12B.4	-0.128816827894851
AGIWUR1238;AGIWUR29950;AGIWUR43060
CB4856	CT	V	6948543 6948831 WBGene00021961	Y57E12B.1	Y57E12B.1	0.702894864654438
AGIWUR26340
CB4856	CT	V	6950835 6953897 WBGene00005188	srg-31	T07H8.5 -0.101228872343653	AGIWUR25906
CB4856	CT	V	6956537 6966422 WBGene00003165	mec-1	T07H8.4 -0.0825157328136812
AGIWUR183;AGIWUR1152;AGIWUR1352;AGIWUR1406;AGIWUR2128;AGIWUR2365;AGIWUR3217;AGIWUR3635;AGIWUR4131;AGIWUR445
5;AGIWUR4803;AGIWUR5260;AGIWUR5262;AGIWUR5882;AGIWUR6045;AGIWUR6798;AGIWUR6876;AGIWUR7189;AGIWUR7519;AGIWUR
7826;AGIWUR8269;AGIWUR8453;AGIWUR10141;AGIWUR10410;AGIWUR10495;AGIWUR11070;AGIWUR12307;AGIWUR13928;AGIWUR14
095;AGIWUR14680;AGIWUR15177;AGIWUR15278;AGIWUR15432;AGIWUR15854;AGIWUR16054;AGIWUR16955;AGIWUR17421;AGIWUR1
7962;AGIWUR17979;AGIWUR18581;AGIWUR19416;AGIWUR19924;AGIWUR23324;AGIWUR24879;AGIWUR25606;AGIWUR25850;AGIWUR
25949;AGIWUR26192;AGIWUR26221;AGIWUR26245;AGIWUR26710;AGIWUR26836;AGIWUR26958;AGIWUR28366;AGIWUR28379;AGIWU
R28699;AGIWUR28906;AGIWUR29899;AGIWUR29988;AGIWUR30069;AGIWUR32078;AGIWUR32276;AGIWUR32477;AGIWUR32711;AGIW
UR33705;AGIWUR34629;AGIWUR34984;AGIWUR37874;AGIWUR38167;AGIWUR38861;AGIWUR39597;AGIWUR39601;AGIWUR39607;AGI
WUR40195;AGIWUR41223;AGIWUR41367;AGIWUR41615;AGIWUR42408;AGIWUR42450;AGIWUR43092;AGIWUR43763;AGIWUR43791;AG
IWUR45104;AGIWUR45161
CB4856	CT	V	6966396 6970127 WBGene00004229	ptr-15	T07H8.6 0.441738878349879	AGIWUR38294
CB4856	CT	V	6971867 6973149 WBGene00005950	srx-59	T07H8.3 0.110444295836492	AGIWUR9812
CB4856	CT	V	6973523 6974832 WBGene00006029	srx-138 T07H8.2 0.04989645109546
AGIWUR5395;AGIWUR15714;AGIWUR23100;AGIWUR38964;AGIWUR40330
CB4856	CT	V	6976347 6977387 WBGene00005907	srx-16	T07H8.1 -0.00724784408260692
AGIWUR3703;AGIWUR34635
CB4856	CT	V	6977754 6979116 WBGene00020333	sre-12	T07H8.7 0.333550019909303	AGIWUR40082
CB4856	CT	V	6980595 6982913 WBGene00016364	nhr-161 C33G8.7 -0.236477784551337	AGIWUR40108
CB4856	CT	V	6983411 6986043 WBGene00016368	nhr-163 C33G8.12	0.344155867005917
AGIWUR12632

CB4856	CT	V	6986141	6987773	WBGene00016365	nhr-139	C33G8.8	0.847132774744518	AGIWUR32935
CB4856	CT	V	6987864	6989676	WBGene00016366	nhr-140	C33G8.9	0.16823994503063	AGIWUR40078
CB4856	CT	V	6990148	6991863	WBGene00016367	nhr-162	C33G8.10	0.463399061957594	AGIWUR9428
CB4856	CT	V	6995215	6997123	WBGene00003697	nhr-107	C33G8.11	0.441473630872505	AGIWUR39250
CB4856	CT	V	6998819	7000651	WBGene00003632	nhr-42	C33G8.6	0.517700205802214	AGIWUR147
CB4856	CT	V	7004150	7005825	WBGene00016363	srab-4	C33G8.5	-0.0569516333406703	AGIWUR23745
CB4856	CT	V	7006264	7006818	WBGene00016362	C33G8.4	C33G8.4	0.220970514314111	AGIWUR4418
CB4856	CT	V	7009761	7012244	WBGene00016360	C33G8.2	C33G8.2	-0.321540858430435	AGIWUR21137
CB4856	CT	V	7012917	7014412	WBGene00005874	srw-127	C33G8.1	0.0163954832724409	AGIWUR16566
CB4856	CT	V	7014462	7015972	WBGene00016369	C33G8.13	C33G8.13	-0.0408051626970867	AGIWUR31072
CB4856	CT	V	7017359	7018851	WBGene00017615	srsx-32	F20A1.3	0.186014320220276	AGIWUR30545
CB4856	CT	V	7018901	7021803	WBGene00017616	F20A1.4	F20A1.4	0.0877206029874427	AGIWUR24840;AGIWUR41280
CB4856	CT	V	7024632	7027083	WBGene00017618	F20A1.6	F20A1.6	0.166766675153159	AGIWUR12474;AGIWUR24968
CB4856	CT	V	7028329	7036570	WBGene00006666	twk-11	F20A1.7	0.143580419431296	AGIWUR1314;AGIWUR5939;AGIWUR10417;AGIWUR31941
CB4856	CT	V	7046312	7049250	WBGene00017614	F20A1.2	F20A1.2	-0.252120162585859	AGIWUR26504
CB4856	CT	V	7049434	7051311	WBGene00017620	tofu-2	F20A1.9	-0.134380336583305	AGIWUR11374
CB4856	CT	V	7051679	7052179	WBGene00017613	F20A1.1	F20A1.1	0.295140518380727	AGIWUR18795
CB4856	CT	V	7052717	7053200	WBGene00017621	F20A1.10	F20A1.10	0.419294178718363	AGIWUR22725
CB4856	CT	V	7054738	7055410	WBGene00021958	Y57E12AL.4	Y57E12AL.4	-0.400689368659572	AGIWUR14197
CB4856	CT	V	7058095	7058507	WBGene00021957	Y57E12AL.2	Y57E12AL.2	0.0641783884175027	AGIWUR14838
CB4856	CT	V	7058547	7059624	WBGene00003164	mdt-6	Y57E12AL.5	-0.164419479505334	AGIWUR41027
CB4856	CT	V	7059698	7060470	WBGene00021959	Y57E12AL.6	Y57E12AL.6	-0.045683282881015	AGIWUR8481
CB4856	CT	V	7060811	7062695	WBGene00021956	Y57E12AL.1	Y57E12AL.1	0.414835161089904	AGIWUR34298
CB4856	CT	V	7063174	7063718	WBGene00021960	Y57E12AM.1	Y57E12AM.1	-0.11352370279789	AGIWUR2965;AGIWUR29220;AGIWUR31548;AGIWUR36754;AGIWUR39340;AGIWUR40810
CB4856	CT	V	7063703	7065322	WBGene00044612	Y57E12AR.1	Y57E12AR.1	0.284339782247695	AGIWUR20069;AGIWUR42428;AGIWUR45178

CB4856	CT	V	7072585	7075835	WBGene00017850	F27B10.1	F27B10.1	0.168605741471346	AGIWUR41140
CB4856	CT	V	7075877	7081569	WBGene00003786	npa-1	VC5.3	-0.320892384259055	AGIWUR3667;AGIWUR14589;AGIWUR16472;AGIWUR29431;AGIWUR32224;AGIWUR34728;AGIWUR35446;AGIWUR36991
CB4856	CT	V	7081537	7084662	WBGene00007029	mys-1	VC5.4	0.176677812356949	AGIWUR13192
CB4856	CT	V	7085916	7088854	WBGene00020908	VC5.2	VC5.2	0.476835679937601	AGIWUR24342
CB4856	CT	V	7089498	7091601	WBGene00044699	nhr-286	VC5.6	0.159514794849271	AGIWUR9847
CB4856	CT	V	7091714	7093770	WBGene00003725	nhr-135	VC5.5	0.285481452321498	AGIWUR10215
CB4856	CT	V	7102478	7103442	WBGene00020907	VC5.1	VC5.1	-0.0143815429073175	AGIWUR22964
CB4856	CT	V	7103697	7114436	WBGene00003849	odr-2	T01C4.2	0.0243110004503322	AGIWUR17952;AGIWUR24443;AGIWUR32476
CB4856	CT	V	7117452	7118896	WBGene00005924	srx-33	T01C4.3	0.187667965866112	AGIWUR29466
CB4856	CT	V	7119063	7120268	WBGene00005925	srx-34	T01C4.4	0.0649888430418732	AGIWUR11889
CB4856	CT	V	7120717	7122495	WBGene00005926	srx-35	T01C4.5	0.230147380812636	AGIWUR12316
CB4856	CT	V	7123658	7128890	WBGene00020141	lmd-5	T01C4.1	-0.20596590152498	AGIWUR29072
CB4856	CT	V	7129146	7129560	WBGene00044546	T01C4.8	T01C4.8	0.0586881692703294	AGIWUR22183
CB4856	CT	V	7129707	7131074	WBGene00005927	srx-36	T01C4.6	0.087194549286167	AGIWUR30051
CB4856	CT	V	7131595	7132324	WBGene00005928	srx-37	T01C4.7	-0.467089992313906	AGIWUR38511;AGIWUR40716
CB4856	CT	V	7134263	7135682	WBGene00271437	F10D2.16	F10D2.16	0.196401300602076	AGIWUR25074
CB4856	CT	V	7136277	7138417	WBGene00006163	str-112	F10D2.4	0.133906018059138	AGIWUR35411
CB4856	CT	V	7139477	7141964	WBGene00017331	ugt-40	F10D2.5	-0.194516040621497	AGIWUR19001
CB4856	CT	V	7141942	7145318	WBGene00017332	ugt-37	F10D2.6	0.469423826577247	AGIWUR35557
CB4856	CT	V	7145656	7147470	WBGene00017333	ugt-38	F10D2.7	-0.0273121025033056	AGIWUR35761
CB4856	CT	V	7148688	7150692	WBGene00017334	F10D2.8	F10D2.8	-0.00387923965506287	AGIWUR20425;AGIWUR31721
CB4856	CT	V	7151353	7153146	WBGene00001399	fat-7	F10D2.9	0.113160152851151	AGIWUR40290
CB4856	CT	V	7154503	7156224	WBGene00017335	F10D2.10	F10D2.10	0.180735188943308	AGIWUR33513
CB4856	CT	V	7157142	7159190	WBGene00017329	ugt-39	F10D2.2	-0.609847755204381	AGIWUR8332;AGIWUR25276;AGIWUR40193
CB4856	CT	V	7159691	7161016	WBGene00006237	str-204	F10D2.1	-0.014411720218769	AGIWUR22344
CB4856	CT	V	7161068	7163595	WBGene00017336	ugt-41	F10D2.11	-0.533665365630799	AGIWUR39992
CB4856	CT	V	7164510	7165895	WBGene00005304	srh-83	F09G2.7	0.044560126135849	AGIWUR25097
CB4856	CT	V	7167009	7169281	WBGene00017315	ugt-36	F09G2.6	0.403972477310393	AGIWUR43649
CB4856	CT	V	7175501	7178783	WBGene00017314	lgc-39	F09G2.5	-0.0834216982449014	AGIWUR13218
CB4856	CT	V	7179172	7182853	WBGene00017313	cpsf-2	F09G2.4	0.0523572018956635	AGIWUR10595

CB4856	CT	V	7184910	7188483	WBGene00017312	pitr-5	F09G2.3	-0.000886184697492354	AGIWUR38058
CB4856	CT	V	7188332	7193662	WBGene00017316	F09G2.8	F09G2.8	-0.0491917371583472	AGIWUR31237
CB4856	CT	V	7193766	7196137	WBGene00017317	attf-2	F09G2.9	0.112336202549278	AGIWUR9026;AGIWUR17703;AGIWUR19961;AGIWUR24522;AGIWUR29842;AGIWUR38507
CB4856	CT	V	7196916	7198437	WBGene00017311	F09G2.2	F09G2.2	0.138084895062281	AGIWUR36789
CB4856	CT	V	7200224	7205609	WBGene00017310	F09G2.1	F09G2.1	0.0809317721585181	AGIWUR11948
CB4856	CT	V	7205626	7214903	WBGene00015743	soap-1	C13F10.4	0.12676201868614	AGIWUR35914
CB4856	CT	V	7215010	7219088	WBGene00015744	C13F10.5	C13F10.5	-0.044182530098217	AGIWUR4255;AGIWUR25008;AGIWUR34248;AGIWUR38845
CB4856	CT	V	7219054	7221039	WBGene00015745	C13F10.6	C13F10.6	-0.0971119816432822	AGIWUR13009;AGIWUR21878;AGIWUR40522
CB4856	CT	V	7221473	7222156	WBGene00015742	kxd-1	C13F10.2	-0.318655977301328	AGIWUR14508
CB4856	CT	V	7222049	7223279	WBGene00015746	C13F10.7	C13F10.7	-0.159029487120268	AGIWUR38641
CB4856	CT	V	7224078	7225204	WBGene00015741	C13F10.1	C13F10.1	-0.158813703208605	AGIWUR38607;AGIWUR42008
CB4856	CT	V	7225223	7226320	WBGene00001428	fkb-3	C05C8.3	-0.0371155688808464	AGIWUR9907
CB4856	CT	V	7226376	7230848	WBGene00001563	gei-6	C05C8.4	-0.0909013410900741	AGIWUR15203
CB4856	CT	V	7232434	7234826	WBGene00015462	C05C8.5	C05C8.5	-0.130939344737115	AGIWUR31801
CB4856	CT	V	7234922	7237130	WBGene00015463	hpo-9	C05C8.6	-0.260483427024401	AGIWUR2698;AGIWUR3991;AGIWUR12483;AGIWUR16790
CB4856	CT	V	7237381	7239658	WBGene00015466	hyls-1	C05C8.9	-0.161477029733787	AGIWUR11456;AGIWUR25540;AGIWUR31894;AGIWUR37578
CB4856	CT	V	7239771	7241366	WBGene00015460	C05C8.1	C05C8.1	-0.111014085623449	AGIWUR26908
CB4856	CT	V	7242907	7247321	WBGene00015464	C05C8.7	C05C8.7	-0.155081680758558	AGIWUR1752;AGIWUR8368;AGIWUR16592;AGIWUR22581;AGIWUR36915;AGIWUR40208
CB4856	CT	V	7252325	7255157	WBGene00015465	C05C8.8	C05C8.8	-0.443052674003822	AGIWUR39223
CB4856	CT	V	7258808	7260157	WBGene00044547	C13A2.12	C13A2.12	0.179231783613508	AGIWUR12736;AGIWUR45175
CB4856	CT	V	7262565	7264148	WBGene00015725	C13A2.9	C13A2.9	0.065470139088859	AGIWUR21866
CB4856	CT	V	7264576	7265377	WBGene00023235	C13A2.8	C13A2.8	0.0237518126295857	AGIWUR1246;AGIWUR26717;AGIWUR31889;AGIWUR33907;AGIWUR43367
CB4856	CT	V	7266353	7268806	WBGene00015724	C13A2.7	C13A2.7	-0.0189747655807722	AGIWUR13134;AGIWUR23165;AGIWUR32284
CB4856	CT	V	7269731	7272787	WBGene00015723	C13A2.6	C13A2.6	0.177846369767748	AGIWUR4210
CB4856	CT	V	7275791	7278472	WBGene00015722	C13A2.5	C13A2.5	-0.0384367930064187	AGIWUR16539
CB4856	CT	V	7279310	7280530	WBGene00015721	C13A2.4	C13A2.4	0.320740572304427	AGIWUR20014

CB4856	CT	V	7282337	7284667	WBGene00015720	C13A2.3	C13A2.3	-0.12473343738414	AGIWUR37246
CB4856	CT	V	7286185	7287399	WBGene00015719	C13A2.2	C13A2.2	-0.012284710339558	AGIWUR24599
CB4856	CT	V	7289027	7291831	WBGene00015718	C13A2.1	C13A2.1	0.103296801620861	AGIWUR21997
CB4856	CT	V	7292754	7295381	WBGene00017231	F07G11.4	F07G11.4	0.136513675067491	AGIWUR33430
CB4856	CT	V	7296300	7297363	WBGene00005617	srj-32	F07G11.5	-0.423836571276956	AGIWUR2894
CB4856	CT	V	7298771	7301472	WBGene00017230	F07G11.3	F07G11.3	0.178325554698729	AGIWUR25605
CB4856	CT	V	7307389	7309556	WBGene00017228	F07G11.1	F07G11.1	0.133738683235558	AGIWUR9956
CB4856	CT	V	7311726	7313333	WBGene00005897	srx-6	F07G11.8	-0.475740748140447	AGIWUR38568
CB4856	CT	V	7314136	7321009	WBGene00017233	lmd-4	F07G11.9	0.111660911174479	AGIWUR7636
CB4856	CT	V	7324038	7325385	WBGene00005983	srx-92	F10G2.6	0.0379805030383764	AGIWUR32095
CB4856	CT	V	7327135	7327755	WBGene00017365	F10G2.4	F10G2.4	0.0706939552133434	AGIWUR43997
CB4856	CT	V	7328392	7330001	WBGene00017364	clec-7	F10G2.3	0.207604558049427	AGIWUR26607
CB4856	CT	V	7331168	7331779	WBGene00017363	F10G2.2	F10G2.2	-0.138119427436383	AGIWUR45026
CB4856	CT	V	7332562	7333424	WBGene00017362	F10G2.1	F10G2.1	-0.217383261804752	AGIWUR5849
CB4856	CT	V	7333774	7337168	WBGene00017366	F10G2.7	F10G2.7	-0.163185931463138	AGIWUR18051
CB4856	CT	V	7338633	7339831	WBGene00005402	srh-187	F10G2.8	-0.0384229935840654	AGIWUR19069
CB4856	CT	V	7340436	7341484	WBGene00017367	nhr-263	F10G2.9	-0.140910581597164	AGIWUR27218;AGIWUR32274
CB4856	CT	V	7342156	7343838	WBGene00015396	nhr-148	C03G6.10	-0.429035379747186	AGIWUR1793
CB4856	CT	V	7344061	7345234	WBGene00005450	srh-244	C03G6.9	0.139398026349067	AGIWUR1764
CB4856	CT	V	7346301	7348135	WBGene00015395	nhr-147	C03G6.8	-0.368229745678526	AGIWUR6976;AGIWUR17637;AGIWUR20992
CB4856	CT	V	7348211	7349664	WBGene00015397	nhr-149	C03G6.12	-0.110715875380627	AGIWUR44680
CB4856	CT	V	7350881	7352236	WBGene00005439	srh-231	C03G6.11	0.307081560897501	AGIWUR34403
CB4856	CT	V	7353040	7354254	WBGene00005480	srh-275	C03G6.7	0.0605867818637158	AGIWUR3671
CB4856	CT	V	7354445	7355510	WBGene00015398	tag-293	C03G6.13	-0.0697570209681615	AGIWUR31359
CB4856	CT	V	7357451	7358882	WBGene00015394	C03G6.6	C03G6.6	0.573750413349905	AGIWUR4749
CB4856	CT	V	7359997	7361773	WBGene00015399	cyp-35A1	C03G6.14	-0.541552796015405	AGIWUR26541;AGIWUR41697

CB4856	CT	V	7365251 7366204 WBGene00015393	C03G6.5 C03G6.5 0.460952009848612	AGIWUR1692
CB4856	CT	V	7366534 7367619 WBGene00005403	srh-189 C03G6.20	0.0121031447308004
AGIWUR5041;AGIWUR36294
CB4856	CT	V	7369759 7372131 WBGene00005602	srj-14	C03G6.3 0.560447880638332
AGIWUR3412;AGIWUR3516;AGIWUR34953
CB4856	CT	V	7374390 7375594 WBGene00005947	srx-56	C03G6.2 -0.0073773103394128
AGIWUR5407;AGIWUR32097
CB4856	CT	V	7376409 7377742 WBGene00005948	srx-57	C03G6.1 -0.00284390896204822	AGIWUR19687
CB4856	CT	V	7377897 7379484 WBGene00015401	srsx-24 C03G6.16	-0.235270137735705
AGIWUR31434
CB4856	CT	V	7379745 7381069 WBGene00015402	C03G6.17	C03G6.17	0.484564999676117
AGIWUR32749
CB4856	CT	V	7382021 7383309 WBGene00005646	srp-5	C03G6.18	-0.00938942150212085
AGIWUR4547
CB4856	CT	V	7384232 7386173 WBGene00005647	srp-6	C03G6.19	-0.139430814325708
AGIWUR1521
CB4856	CT	V	7388390 7389871 WBGene00015361	C02H6.1 C02H6.1 0.0238643886050562	AGIWUR15685
CB4856	CT	V	7390529 7392111 WBGene00015362	fbxa-105	C02H6.2 0.0313733316108955
AGIWUR44525
CB4856	CT	V	7393550 7395459 WBGene00005900	srx-9	R13D7.6 -0.249441754966115	AGIWUR37970
CB4856	CT	V	7398390 7399302 WBGene00001789	gst-41	R13D7.7 0.417233189635583	AGIWUR42946
CB4856	CT	V	7399904 7400199 WBGene00044548	cnc-9	R13D7.11	-0.393410147353986
AGIWUR6901;AGIWUR8643;AGIWUR11457
CB4856	CT	V	7400888 7402192 WBGene00005951	srx-60	R13D7.4 0.0166037746308509	AGIWUR37472
CB4856	CT	V	7402655 7403910 WBGene00005601	srj-13	R13D7.3 0.187035591230813	AGIWUR29550
CB4856	CT	V	7404821 7405871 WBGene00006091	str-24	R13D7.9 0.183521512089374
AGIWUR4229;AGIWUR12780
CB4856	CT	V	7407547 7408814 WBGene00020055	srt-6	R13D7.10	0.0853635500448275
AGIWUR18133
CB4856	CT	V	7409899 7411253 WBGene00020054	R13D7.2 R13D7.2 -0.226856969925176	AGIWUR23364
CB4856	CT	V	7411995 7413059 WBGene00006106	str-41	R13D7.1 0.0671231950117166	AGIWUR39155
CB4856	CT	V	7413437 7413840 WBGene00017567	F18E3.11	F18E3.11	-0.741673446136859
AGIWUR16027;AGIWUR28494
CB4856	CT	V	7414941 7415344 WBGene00045275	F18E3.12	F18E3.12	-0.813569195469923
AGIWUR26393;AGIWUR34940
CB4856	CT	V	7416116 7416628 WBGene00045276	F18E3.13	F18E3.13	-0.743825396527507
AGIWUR790
CB4856	CT	V	7417660 7419828 WBGene00005814	srw-67	F18E3.2 0.0634858257708926	AGIWUR22042
CB4856	CT	V	7420462 7421713 WBGene00017564	srxa-4	F18E3.1 0.076728270839995	AGIWUR22088

CB4856	CT	V	7421855	7422937	WBGene00005343	srh-126	F18E3.3	-0.134026188035691
AGIWUR3174;AGIWUR10542;AGIWUR11741;AGIWUR22896;AGIWUR25510;AGIWUR35121;AGIWUR39022
CB4856	CT	V	7423573	7424578	WBGene00017566	F18E3.10	F18E3.10	0.157369706241346
AGIWUR24955
CB4856	CT	V	7425259	7426694	WBGene00005815	srw-68	F18E3.4	0.513631244588079	AGIWUR25357
CB4856	CT	V	7427326	7428980	WBGene00005816	srw-69	F18E3.5	0.082482293580834	AGIWUR18382
CB4856	CT	V	7429684	7431116	WBGene00005818	srw-71	F18E3.6	0.295377410813821	AGIWUR31118
CB4856	CT	V	7431822	7433160	WBGene00005825	srw-78	F18E3.9	0.0859675531504687
AGIWUR1755;AGIWUR3992;AGIWUR8503;AGIWUR12691;AGIWUR15111;AGIWUR29806;AGIWUR31635
CB4856	CT	V	7433401	7435650	WBGene00017565	ddo-2	F18E3.7	0.25303856106499
AGIWUR31689;AGIWUR40261;AGIWUR42918;AGIWUR43676
CB4856	CT	V	7435413	7436962	WBGene00017789	F25E5.8	F25E5.8	0.844933708539101
AGIWUR5670;AGIWUR27478;AGIWUR29340;AGIWUR42027
CB4856	CT	V	7437506	7437919	WBGene00017790	F25E5.9	F25E5.9	0.0767958823213313
AGIWUR4161;AGIWUR18222;AGIWUR20571;AGIWUR21570;AGIWUR30302;AGIWUR35467
CB4856	CT	V	7438285	7439933	WBGene00017792	F25E5.16	F25E5.16	0.110180531230071
AGIWUR26187
CB4856	CT	V	7440107	7441483	WBGene00017788	F25E5.7	F25E5.7	-0.435537937616635	AGIWUR16933
CB4856	CT	V	7445065	7446712	WBGene00017787	nhr-141	F25E5.6	0.114975832059771	AGIWUR40405
CB4856	CT	V	7447273	7448884	WBGene00017786	F25E5.5	F25E5.5	-0.067017865904333
AGIWUR3591;AGIWUR4736;AGIWUR41263
CB4856	CT	V	7449353	7451149	WBGene00017785	F25E5.4	F25E5.4	-0.199075569493967	AGIWUR3460
CB4856	CT	V	7451721	7452899	WBGene00017784	F25E5.3	F25E5.3	-0.230818349895669	AGIWUR44505
CB4856	CT	V	7452946	7454318	WBGene00017791	try-8	F25E5.10	-0.0628447607160769
AGIWUR13538
CB4856	CT	V	7455819	7457597	WBGene00017783	F25E5.2	F25E5.2	-0.0803039638793344	AGIWUR35524
CB4856	CT	V	7458252	7459150	WBGene00005314	srh-93	F25E5.15	-0.13005195529052
AGIWUR42344
CB4856	CT	V	7459685	7460998	WBGene00005313	srh-92	F25E5.13	-0.102693522484966
AGIWUR8682
CB4856	CT	V	7461750	7462949	WBGene00005312	srh-91	F25E5.11	0.0300551957020132
AGIWUR5456;AGIWUR11180
CB4856	CT	V	7465889	7471223	WBGene00017782	F25E5.1	F25E5.1	-0.142324376248446	AGIWUR4137
CB4856	CT	V	7471651	7472915	WBGene00006094	str-28	F25E5.12	0.0185980411101827
AGIWUR9253
CB4856	CT	V	7473299	7475013	WBGene00006095	str-29	C18B10.9	0.0370280281811031
AGIWUR4836;AGIWUR23362
CB4856	CT	V	7475626	7476866	WBGene00005342	srh-125	C18B10.8	0.203746872124334
AGIWUR21451

CB4856	CT	V	7477241	7478504	WBGene00006231	str-190	C18B10.7	0.121068816558084	AGIWUR10689
CB4856	CT	V	7479217	7481049	WBGene00015963	C18B10.6	C18B10.6	-0.0412456372615928	AGIWUR838;AGIWUR10121;AGIWUR43551
CB4856	CT	V	7481868	7483171	WBGene00006285	str-259	C18B10.10	0.0562559072554909	AGIWUR6434;AGIWUR33505
CB4856	CT	V	7483316	7485100	WBGene00015962	srbc-8	C18B10.5	-0.167727264916305	AGIWUR2319;AGIWUR21711
CB4856	CT	V	7486131	7487618	WBGene00015961	srbc-7	C18B10.4	0.181698313797288	AGIWUR18606
CB4856	CT	V	7489134	7490440	WBGene00015960	srbc-9	C18B10.3	0.188141547516008	AGIWUR15347;AGIWUR27013
CB4856	CT	V	7491875	7493024	WBGene00015959	srbc-10	C18B10.2	0.155248649650887	AGIWUR44235
CB4856	CT	V	7493880	7495289	WBGene00015958	srbc-11	C18B10.1	0.163671776630089	AGIWUR38818
CB4856	CT	V	7497740	7499055	WBGene00005300	srh-79	C04F2.1	0.212328555389286	AGIWUR34383
CB4856	CT	V	7499652	7500958	WBGene00005431	srh-221	C04F2.2	-0.0519559278865964	AGIWUR22231;AGIWUR40815
CB4856	CT	V	7501534	7502673	WBGene00005438	srh-230	C04F2.3	0.0161304089981114	AGIWUR36299;AGIWUR42880
CB4856	CT	V	7502840	7504371	WBGene00005437	srh-229	C04F2.4	0.0200974436726415	AGIWUR4123;AGIWUR13226;AGIWUR20592
CB4856	CT	V	7504653	7505945	WBGene00005473	srh-267	C04F2.5	0.107027467452287	AGIWUR12092;AGIWUR19173
CB4856	CT	V	7506914	7508265	WBGene00006097	str-31	C54F6.10	0.206210412911156	AGIWUR37093
CB4856	CT	V	7508994	7510602	WBGene00016927	nhr-172	C54F6.9	0.410800280751592	AGIWUR8052
CB4856	CT	V	7511765	7513684	WBGene00016926	nhr-171	C54F6.8	0.442855169506304	AGIWUR21182
CB4856	CT	V	7515245	7517307	WBGene00016925	srb-18	C54F6.7	0.148212960398537	AGIWUR22330
CB4856	CT	V	7517685	7520177	WBGene00016928	srb-19	C54F6.11	0.322251007747072	AGIWUR6103
CB4856	CT	V	7522032	7522364	WBGene00044113	C54F6.15	C54F6.15	1.22455394721715	AGIWUR3238;AGIWUR29620;AGIWUR37411;AGIWUR39092;AGIWUR40472
CB4856	CT	V	7523692	7525421	WBGene00016924	C54F6.6	C54F6.6	0.107618756188861	AGIWUR42339
CB4856	CT	V	7529116	7529450	WBGene00016923	C54F6.5	C54F6.5	1.50637508760125	AGIWUR8243
CB4856	CT	V	7530261	7531463	WBGene00016922	swt-2	C54F6.4	0.621452909637795	AGIWUR34665
CB4856	CT	V	7533247	7535074	WBGene00016921	C54F6.3	C54F6.3	0.141025721793543	AGIWUR10479
CB4856	CT	V	7536919	7537909	WBGene00016929	C54F6.12	C54F6.12	0.699418559675588	

AGIWUR901
CB4856 CT V 7539315 7540891 WBGene00005474 srh-268 C54F6.1 -0.0604658307332473 AGIWUR38594
CB4856 CT V 7540890 7544838 WBGene00003731 nhx-3 C54F6.13 -0.279015571862702
AGIWUR24901
CB4856 CT V 7546468 7548012 WBGene00001500 ftn-1 C54F6.14 0.455051702829969
AGIWUR4104;AGIWUR10941;AGIWUR19302;AGIWUR25571
CB4856 CT V 7552121 7552708 WBGene00017599 F19F10.3 F19F10.3 -0.14139004534937
AGIWUR17858
CB4856 CT V 7552756 7553923 WBGene00017600 ttr-10 F19F10.4 -0.126839737911435
AGIWUR205
CB4856 CT V 7555810 7557387 WBGene00005534 sri-22 F19F10.2 -0.0110365994357426
AGIWUR26691;AGIWUR41410
CB4856 CT V 7557541 7558783 WBGene00017601 ets-7 F19F10.5 0.165489052224645
AGIWUR4260
CB4856 CT V 7560252 7561615 WBGene00017602 clec-211 F19F10.6 -0.143093439394041
AGIWUR30534
CB4856 CT V 7562714 7564245 WBGene00017598 F19F10.1 F19F10.1 0.0242193929372121
AGIWUR44088
CB4856 CT V 7566232 7567059 WBGene00017603 clec-212 F19F10.7 0.020763783920227
AGIWUR868
CB4856 CT V 7569696 7570390 WBGene00017604 F19F10.8 F19F10.8 0.102701702551915
AGIWUR12944;AGIWUR17120;AGIWUR21096;AGIWUR29979
CB4856 CT V 7571950 7575715 WBGene00017605 F19F10.9 F19F10.9 -0.0482907197133038
AGIWUR36257
CB4856 CT V 7575750 7581532 WBGene00017608 F19F10.12 F19F10.12 -0.0465789574813513
AGIWUR21931;AGIWUR36890;AGIWUR40367
CB4856 CT V 7581473 7587698 WBGene00017607 F19F10.11 F19F10.11 -0.140203781435666
AGIWUR22956;AGIWUR25815
CB4856 CT V 7587996 7594740 WBGene00016816 C50E3.5 C50E3.5 -0.0391385642836539 AGIWUR38076
CB4856 CT V 7594875 7596409 WBGene00016818 C50E3.7 C50E3.7 0.188679600416712 AGIWUR27300
CB4856 CT V 7598029 7598369 WBGene00016819 C50E3.8 C50E3.8 0.16214396714725 AGIWUR20841
CB4856 CT V 7599257 7600424 WBGene00016820 C50E3.9 C50E3.9 -0.679751169995789 AGIWUR37388
CB4856 CT V 7600609 7601543 WBGene00016821 scl-26 C50E3.10 -0.55003143924334
AGIWUR39347
CB4856 CT V 7602213 7603486 WBGene00016822 C50E3.11 C50E3.11 -0.818334074338471
AGIWUR3880;AGIWUR6076;AGIWUR9166;AGIWUR9454;AGIWUR18332;AGIWUR20959
CB4856 CT V 7603806 7609946 WBGene00044738 folt-3 C50E3.16 -0.249762769071096
AGIWUR7594;AGIWUR28960
CB4856 CT V 7610370 7610963 WBGene00016814 C50E3.2 C50E3.2 -0.331823984744004 AGIWUR43867

CB4856	CT	V	7611267	7611905	WBGene00016813	clec-214	C50E3.1	-0.0397919673082647	AGIWUR9667
CB4856	CT	V	7612344	7615648	WBGene00044549	clec-215	C50E3.14	0.139882848800494	AGIWUR7336;AGIWUR28371
CB4856	CT	V	7618414	7620349	WBGene00044550	C50E3.15	C50E3.15	0.0903364822868467	AGIWUR35919;AGIWUR36118
CB4856	CT	V	7620504	7622061	WBGene00016823	C50E3.12	C50E3.12	-0.311737528902636	AGIWUR7090;AGIWUR16388;AGIWUR17311
CB4856	CT	V	7622335	7624252	WBGene00016824	C50E3.13	C50E3.13	-0.204432657322076	AGIWUR29608
CB4856	CT	V	7624952	7626453	WBGene00017201	grsp-4	F07C4.7	0.462496858222824	AGIWUR10100
CB4856	CT	V	7626850	7627780	WBGene00017200	F07C4.6	F07C4.6	0.107203201121947	AGIWUR39813
CB4856	CT	V	7629804	7631298	WBGene00005108	srd-30	F07C4.5	0.689098509316068	AGIWUR14520
CB4856	CT	V	7632032	7633172	WBGene00005103	srd-25	F07C4.4	0.253916397618598	AGIWUR30077
CB4856	CT	V	7633399	7634757	WBGene00005109	srd-31	F07C4.8	0.0218958074475175	AGIWUR41386
CB4856	CT	V	7635769	7636882	WBGene00005107	srd-29	F07C4.3	0.199822872292458	AGIWUR1831
CB4856	CT	V	7637277	7637792	WBGene00017202	clec-46	F07C4.9	0.263291623671876	AGIWUR9953
CB4856	CT	V	7638105	7638622	WBGene00017199	clec-45	F07C4.2	0.157589955380947	AGIWUR24928
CB4856	CT	V	7638944	7639869	WBGene00017203	F07C4.10	F07C4.10	-0.0146282304917209	AGIWUR22824
CB4856	CT	V	7642558	7643516	WBGene00017204	F07C4.11	F07C4.11	0.0696509852070015	AGIWUR37360
CB4856	CT	V	7645754	7647069	WBGene00006112	str-47	F07C4.1	-0.0384113798130246	AGIWUR28091
CB4856	CT	V	7647836	7649817	WBGene00017205	F07C4.12	F07C4.12	2.14840183876536	AGIWUR14177;AGIWUR21706
CB4856	CT	V	7650242	7651857	WBGene00005441	srh-234	F07C4.14	0.166244584676035	AGIWUR30304
CB4856	CT	V	7652338	7653667	WBGene00005411	srh-200	F07C4.13	-0.237661821628038	AGIWUR44692
CB4856	CT	V	7668240	7669000	WBGene00015708	C12D5.5	C12D5.5	0.141571490385979	AGIWUR34402
CB4856	CT	V	7672089	7673253	WBGene00015707	C12D5.4	C12D5.4	-0.0150296544955211	AGIWUR2897;AGIWUR42966;AGIWUR44218
CB4856	CT	V	7676204	7676965	WBGene00015706	C12D5.3	C12D5.3	0.717798292716497	AGIWUR1116
CB4856	CT	V	7679314	7681615	WBGene00015709	cyp-33A1	C12D5.7	0.376575313869896	AGIWUR36221
CB4856	CT	V	7681820	7683957	WBGene00015712	sre-11	C12D5.11	-0.255592256095848	AGIWUR29774
CB4856	CT	V	7684363	7686335	WBGene00003684	nhr-94	C12D5.8	0.333747740374382	AGIWUR17409;AGIWUR18470;AGIWUR24192;AGIWUR38643;AGIWUR40479;AGIWUR43922

CB4856 CT V 7686350 7688228 WBGene00015705 nhr-152 C12D5.2 0.381844342342383 AGIWUR41171
CB4856 CT V 7688237 7689988 WBGene00015710 C12D5.9 C12D5.9 0.246424296793929 AGIWUR41038
CB4856 CT V 7690594 7692043 WBGene00006194 str-147 C12D5.1 0.21247580191823
AGIWUR29458;AGIWUR43240
CB4856 CT V 7692071 7693488 WBGene00015711 C12D5.10 C12D5.10 -0.0413125290928633
AGIWUR4130;AGIWUR17148;AGIWUR35652
CB4856 CT V 7697142 7700760 WBGene00022654 ZK105.3 ZK105.3 -0.448568768637221 AGIWUR26366
CB4856 CT V 7701048 7703636 WBGene00022655 srsx-40 ZK105.4 0.186772294944703 AGIWUR24681
CB4856 CT V 7705616 7706955 WBGene00005750 srw-3 ZK105.2 -0.258042289919649 AGIWUR44099
CB4856 CT V 7709050 7710037 WBGene00022658 srh-188 ZK105.7 0.140325127171469
AGIWUR17472;AGIWUR35760
CB4856 CT V 7712239 7713168 WBGene00235114 ZK105.13 ZK105.13 0.239939731746036
AGIWUR5750
CB4856 CT V 7713990 7714698 WBGene00022653 ZK105.1 ZK105.1 -0.20801490150212 AGIWUR5908
CB4856 CT V 7721181 7724065 WBGene00022657 ZK105.6 ZK105.6 -0.284858434941745 AGIWUR40107
CB4856 CT V 7724494 7726110 WBGene00044551 ZK105.8 ZK105.8 0.0883936685179876
AGIWUR6482;AGIWUR39137;AGIWUR42851
CB4856 CT V 7728813 7730317 WBGene00022410 Y97E10C.1 Y97E10C.1 -0.027178642370405
AGIWUR23151
CB4856 CT V 7731839 7733495 WBGene00044342 T05B11.7 T05B11.7 0.220731231269943
AGIWUR3311;AGIWUR35193
CB4856 CT V 7735619 7737024 WBGene00005661 srr-10 T05B11.6 -0.100838688978936
AGIWUR44351
CB4856 CT V 7738010 7739596 WBGene00005660 srr-9 T05B11.2 0.017181659556222
AGIWUR42129
CB4856 CT V 7748863 7751368 WBGene00020245 T05B11.1 T05B11.1 0.0316020869419908
AGIWUR26544
CB4856 CT V 7751901 7754139 WBGene00020247 T05B11.4 T05B11.4 -0.467117872294338
AGIWUR36773
CB4856 CT V 7756749 7757854 WBGene00023340 T05B11.5 T05B11.5 -0.0375929702311124
AGIWUR12624;AGIWUR27525;AGIWUR40406
CB4856 CT V 7760474 7761633 WBGene00016150 hint-3 C26F1.7 -0.166737602962653 AGIWUR28712
CB4856 CT V 7771328 7773273 WBGene00016149 frpr-3 C26F1.6 -0.104454049390618 AGIWUR21381
CB4856 CT V 7774441 7775759 WBGene00001719 grl-10 C26F1.5 0.413075891277554 AGIWUR700
CB4856 CT V 7775742 7776079 WBGene00004453 rpl-39 C26F1.9 0.0832980550975151 AGIWUR17213
CB4856 CT V 7776576 7777236 WBGene00004499 rps-30 C26F1.4 -0.0270810261065009
AGIWUR2827;AGIWUR13715;AGIWUR20076;AGIWUR25322;AGIWUR32322;AGIWUR33401;AGIWUR40641
CB4856 CT V 7777300 7778985 WBGene00016148 C26F1.3 C26F1.3 -0.0759928855333261
AGIWUR60;AGIWUR12951;AGIWUR33169;AGIWUR36881

CB4856	CT	V	7781811	7784595	WBGene00016147	cyp-32A1	C26F1.2	0.073536241468128
AGIWUR44527
CB4856	CT	V	7785417	7787712	WBGene00016146	C26F1.1	C26F1.1	0.137215449567994
AGIWUR6041;AGIWUR11348;AGIWUR18024;AGIWUR29985;AGIWUR33124;AGIWUR33236
CB4856	CT	V	7793367	7794025	WBGene00001464	flp-21	C26F1.10	0.554853901092724
AGIWUR8251
CB4856	CT	V	7801518	7803727	WBGene00022812	uvs-1	ZK742.2	-0.108973906824097	AGIWUR34269
CB4856	CT	V	7805064	7814656	WBGene00002078	xpo-1	ZK742.1	0.037059325621307
AGIWUR721;AGIWUR1219;AGIWUR5682;AGIWUR11323;AGIWUR14384;AGIWUR15800;AGIWUR23048;AGIWUR23160;AGIWUR26928;AGI
WUR28172;AGIWUR34718;AGIWUR39925;AGIWUR40454
CB4856	CT	V	7820578	7821477	WBGene00002256	lbp-4	ZK742.5	-0.0979472351458259	AGIWUR42605
CB4856	CT	V	7827201	7842388	WBGene00003242	mig-6	C37C3.6	0.326425093610279
AGIWUR27540;AGIWUR30637;AGIWUR44675
CB4856	CT	V	7842543	7844299	WBGene00001697	grd-8	C37C3.4	0.24852585083177	AGIWUR13235
CB4856	CT	V	7844343	7847164	WBGene00016500	memo-1	C37C3.8	0.675242455503112
AGIWUR21318;AGIWUR38933;AGIWUR42472;AGIWUR43233
CB4856	CT	V	7849984	7851029	WBGene00016497	vps-32.2	C37C3.3	-0.164599638361929
AGIWUR16738
CB4856	CT	V	7852286	7853975	WBGene00016501	C37C3.9	C37C3.9	-0.124572383630742	AGIWUR18544
CB4856	CT	V	7854991	7857925	WBGene00016496	C37C3.2	C37C3.2	-0.133480378370377
AGIWUR10421;AGIWUR15189;AGIWUR29926;AGIWUR38035
CB4856	CT	V	7857963	7859909	WBGene00016495	C37C3.1	C37C3.1	-0.105400425249123	AGIWUR30764
CB4856	CT	V	7862275	7863070	WBGene00016502	C37C3.10	C37C3.10	-0.165659752711652
AGIWUR21569
CB4856	CT	V	7863458	7864731	WBGene00016503	C37C3.11	C37C3.11	-0.153356123510044
AGIWUR13987
CB4856	CT	V	7868470	7870807	WBGene00077712	nipi-4	F40A3.5	-0.159857235800634	AGIWUR32728
CB4856	CT	V	7875756	7876789	WBGene00018219	F40A3.4	F40A3.4	-0.263703744496297
AGIWUR390;AGIWUR13174;AGIWUR23869
CB4856	CT	V	7877490	7878514	WBGene00018218	F40A3.3	F40A3.3	-0.0613064534334136	AGIWUR4725
CB4856	CT	V	7878914	7881403	WBGene00018217	F40A3.2	F40A3.2	0.203156152723952	AGIWUR10577
CB4856	CT	V	7891530	7895518	WBGene00018216	F40A3.1	F40A3.1	0.436290933624221	AGIWUR36055
CB4856	CT	V	7895465	7899861	WBGene00018222	F40A3.7	F40A3.7	0.410039942953544	AGIWUR45014
CB4856	CT	V	7914420	7915728	WBGene00005902	srx-11	Y97E10B.6	0.0390175330096909
AGIWUR27174
CB4856	CT	V	7925783	7926756	WBGene00022406	srsx-7	Y97E10B.4	0.390170566904818
AGIWUR17411
CB4856	CT	V	7927084	7928451	WBGene00022405	srsx-6	Y97E10B.3	0.089881035774825
AGIWUR18933

CB4856	CT	V	7928960	7930337	WBGene00022404	srsx-5	Y97E10B.2	0.0374614558032045	AGIWUR15793
CB4856	CT	V	7930475	7933098	WBGene00003575	ncx-10	Y97E10B.7	-0.305998992685126	AGIWUR25969
CB4856	CT	V	7934457	7935113	WBGene00022407	srsx-4	Y97E10B.8	0.0113529673572845	AGIWUR8695
CB4856	CT	V	7936144	7937252	WBGene00022408	srsx-3	Y97E10B.9	-0.322709214119772	AGIWUR41183
CB4856	CT	V	7938605	7940383	WBGene00022409	srt-53	Y97E10B.10	0.0447690466469384	AGIWUR12412;AGIWUR36109;AGIWUR44229
CB4856	CT	V	7943868	7946857	WBGene00022403	Y97E10B.1	Y97E10B.1	0.107434980117742	AGIWUR9706
CB4856	CT	V	7947231	7948615	WBGene00017821	F26D11.4	F26D11.4	0.412431580058129	AGIWUR33936
CB4856	CT	V	7951625	7952955	WBGene00017822	clec-216	F26D11.5	-0.346065142662681	AGIWUR19386;AGIWUR35862;AGIWUR41728
CB4856	CT	V	7953354	7954652	WBGene00045393	F26D11.13	F26D11.13	0.519826675655266	AGIWUR5740
CB4856	CT	V	7954793	7955363	WBGene00045392	pals-35	F26D11.12	-0.0758315798060849	AGIWUR21854
CB4856	CT	V	7955404	7956497	WBGene00017823	pals-34	F26D11.6	-0.276677157906998	AGIWUR4877;AGIWUR18296
CB4856	CT	V	7957277	7958840	WBGene00017824	clec-217	F26D11.9	-0.330119911010957	AGIWUR38445
CB4856	CT	V	7959270	7960958	WBGene00017819	F26D11.2	F26D11.2	0.0993519238157994	AGIWUR14036
CB4856	CT	V	7961539	7968899	WBGene00000488	che-7	F26D11.10	0.402408926592626	AGIWUR33539
CB4856	CT	V	7972924	7977774	WBGene00002632	let-413	F26D11.11	0.178163774698559	AGIWUR7383;AGIWUR44679
CB4856	CT	V	7981545	7982662	WBGene00017818	F26D11.1	F26D11.1	-0.0128528102963834	AGIWUR42437
CB4856	CT	V	7982656	7983431	WBGene00022394	Y97E10AL.3	Y97E10AL.3	-0.301552422759524	AGIWUR7366
CB4856	CT	V	7983849	7985954	WBGene00022393	abhd-12	Y97E10AL.2	0.0372616468372531	AGIWUR23178
CB4856	CT	V	7993789	7994666	WBGene00022395	ceh-76	Y97E10AM.1	0.049322537792202	AGIWUR36575
CB4856	CT	V	7996178	8000233	WBGene00022397	Y97E10AR.2	Y97E10AR.2	-0.197273553268935	

AGIWUR41368
CB4856	CT	V	8001451 8004690 WBGene00022396	Y97E10AR.1	Y97E10AR.1	0.731658573631623
AGIWUR6325;AGIWUR21011;AGIWUR26692;AGIWUR31456;AGIWUR38523;AGIWUR39332
CB4856	CT	V	8005326 8005692 WBGene00044552	Y97E10AR.8	Y97E10AR.8	0.13305884038172
AGIWUR24477;AGIWUR28438;AGIWUR30489;AGIWUR37606;AGIWUR41982
CB4856	CT	V	8006179 8007510 WBGene00022398	Y97E10AR.3	Y97E10AR.3	-0.075726828980315
AGIWUR4548
CB4856	CT	V	8008028 8010443 WBGene00022399	Y97E10AR.4	Y97E10AR.4	-0.202087127509119
AGIWUR31544
CB4856	CT	V	8010475 8011108 WBGene00022402	lmtr-2	Y97E10AR.7	0.181565274703816
AGIWUR44898
CB4856	CT	V	8011219 8012345 WBGene00022400	rpb-9	Y97E10AR.5	-0.370697148711379
AGIWUR14983
CB4856	CT	V	8012401 8014182 WBGene00022401	Y97E10AR.6	Y97E10AR.6	0.0160472101565823
AGIWUR2712
CB4856	CT	V	8017947 8021715 WBGene00018398	sptl-2	F43H9.2 -0.269044087835543
AGIWUR12380;AGIWUR16581;AGIWUR21967;AGIWUR31278
CB4856	CT	V	8022218 8023308 WBGene00001152	ech-3	F43H9.1 -0.16251752132198	AGIWUR38133
CB4856	CT	V	8023195 8024940 WBGene00018399	F43H9.3 F43H9.3 -0.0780706790260552	AGIWUR30800
CB4856	CT	V	8026691 8028789 WBGene00018400	F43H9.4 F43H9.4 -0.694226895778982	AGIWUR2161
CB4856	CT	V	8029439 8031214 WBGene00003865	oma-2	ZC513.6 0.0663438228192135	AGIWUR10685
CB4856	CT	V	8031411 8035014 WBGene00006935	vars-1	ZC513.4 -0.0141566052622601
AGIWUR21496;AGIWUR24632;AGIWUR29054;AGIWUR39461
CB4856	CT	V	8035111 8039506 WBGene00022629	ZC513.5 ZC513.5 -0.196302370999486	AGIWUR6084
CB4856	CT	V	8039469 8040387 WBGene00023209	ZC513.7 ZC513.7 0.521668953838814
AGIWUR11496;AGIWUR17404;AGIWUR38696
CB4856	CT	V	8047044 8049528 WBGene00022628	ZC513.3 ZC513.3 0.0682615199038903	AGIWUR33455
CB4856	CT	V	8049744 8050893 WBGene00000620	col-43	ZC513.8 -0.801936339553992	AGIWUR957
CB4856	CT	V	8052137 8053730 WBGene00006060	sth-1	ZC513.12	-0.256296489119348
AGIWUR27227
CB4856	CT	V	8054430 8057558 WBGene00022627	ZC513.2 ZC513.2 -0.131005799037765
AGIWUR27743;AGIWUR44063
CB4856	CT	V	8058659 8060317 WBGene00006187	str-138 ZC513.11	-0.0263030320633426
AGIWUR36260
CB4856	CT	V	8060873 8062174 WBGene00006186	str-139 ZC513.9 0.141488823937509	AGIWUR8996
CB4856	CT	V	8063247 8065079 WBGene00022630	fbxa-223	ZC513.10	-0.0988677420747175
AGIWUR41957
CB4856	CT	V	8065780 8067900 WBGene00006188	str-140 C09H5.3 -0.471634419541389	AGIWUR12899
CB4856	CT	V	8068152 8069737 WBGene00006190	str-143 C09H5.4 0.186467933542859	AGIWUR22797

CB4856 CT V 8070643 8072266 WBGene00006191 str-144 C09H5.5 0.288538497036599 AGIWUR24395
CB4856 CT V 8076012 8079914 WBGene00015660 catp-3 C09H5.2 -0.139120239589392
AGIWUR20050;AGIWUR41311
CB4856 CT V 8080367 8081678 WBGene00006180 str-131 C09H5.6 -0.182726754729344 AGIWUR14503
CB4856 CT V 8082367 8082767 WBGene00015659 C09H5.1 C09H5.1 0.00910549042590247 AGIWUR15945
CB4856 CT V 8084120 8085241 WBGene00015661 C09H5.7 C09H5.7 -0.257924865109672 AGIWUR19755
CB4856 CT V 8085884 8087283 WBGene00006177 str-128 C09H5.8 0.211271158739882 AGIWUR39552
CB4856 CT V 8089319 8090695 WBGene00006179 str-130 C09H5.9 0.151304506730344 AGIWUR20800
CB4856 CT V 8090973 8092485 WBGene00006178 str-129 F58G4.5 -0.219227318414632 AGIWUR24912
CB4856 CT V 8093335 8095468 WBGene00006193 str-146 F58G4.6 -0.250255795131184 AGIWUR34556
CB4856 CT V 8096394 8098339 WBGene00006192 str-145 F58G4.7 -0.063022712854349 AGIWUR31757
CB4856 CT V 8099621 8100484 WBGene00019066 sdz-23 F58G4.4 0.0517888146072449 AGIWUR33239
CB4856 CT V 8102484 8106905 WBGene00006149 str-90 F58G4.2 0.0576308149454547 AGIWUR24341
CB4856 CT V 8108970 8118131 WBGene00019064 myo-5 F58G4.1 0.558726886747488 AGIWUR42109
CB4856 CT V 8118264 8120351 WBGene00017020 D1014.7 D1014.7 0.0795952796580917 AGIWUR28183
CB4856 CT V 8121168 8123470 WBGene00017019 D1014.6 D1014.6 0.0487880764682878 AGIWUR10069
CB4856 CT V 8123684 8126710 WBGene00017018 D1014.5 D1014.5 0.70234619803856 AGIWUR26747
CB4856 CT V 8126663 8129352 WBGene00005006 spr-1 D1014.8 -0.224586677845391
AGIWUR2032;AGIWUR11463;AGIWUR13038
CB4856 CT V 8130388 8133329 WBGene00017016 snap-1 D1014.3 -0.0191449416928933 AGIWUR11044
CB4856 CT V 8133432 8134985 WBGene00017017 D1014.4 D1014.4 -0.0818225031644841
AGIWUR1511;AGIWUR18218;AGIWUR39125;AGIWUR39914
CB4856 CT V 8143761 8147782 WBGene00017015 D1014.2 D1014.2 -0.224107446156822 AGIWUR18107
CB4856 CT V 8157746 8159844 WBGene00006309 sul-2 D1014.1 0.589862757249946 AGIWUR14310
CB4856 CT V 8159929 8161643 WBGene00017636 F20D6.6 F20D6.6 0.545674628628938 AGIWUR12077
CB4856 CT V 8177093 8177449 WBGene00017638 F20D6.9 F20D6.9 0.150968210171959 AGIWUR25233
CB4856 CT V 8179131 8183442 WBGene00005648 srp-7 F20D6.4 -0.053995303038219
AGIWUR10926;AGIWUR15736;AGIWUR41757
CB4856 CT V 8183730 8185772 WBGene00005649 srp-8 F20D6.3 0.0700937769589602
AGIWUR14471;AGIWUR14902;AGIWUR23568;AGIWUR43404
CB4856 CT V 8186039 8187319 WBGene00017634 F20D6.2 F20D6.2 0.278637595043577 AGIWUR9313
CB4856 CT V 8187803 8190116 WBGene00043069 F20D6.12 F20D6.12 0.0233808234929338
AGIWUR12284
CB4856 CT V 8190699 8192050 WBGene00017633 F20D6.1 F20D6.1 -0.0783356119992416 AGIWUR6718
CB4856 CT V 8192060 8195937 WBGene00017639 F20D6.10 F20D6.10 0.374568118264504
AGIWUR8103
CB4856 CT V 8196774 8199094 WBGene00017640 F20D6.11 F20D6.11 -0.0355198921731024
AGIWUR5525
CB4856 CT V 8201808 8202546 WBGene00019478 cri-2 K07C11.5 -0.0329370065275629

AGIWUR42010
CB4856 CT V 8203379 8205986 WBGene00019477 K07C11.4 K07C11.4 0.654963653181055
AGIWUR17869
CB4856 CT V 8207235 8208013 WBGene00019476 K07C11.3 K07C11.3 -0.0248468115424352
AGIWUR5481
CB4856 CT V 8207930 8213968 WBGene00019479 K07C11.7 K07C11.7 -0.126346981181726
AGIWUR1879;AGIWUR11564;AGIWUR41258
CB4856 CT V 8215534 8217884 WBGene00019480 K07C11.8 K07C11.8 -0.164734656387749
AGIWUR703
CB4856 CT V 8218279 8221316 WBGene00019481 cogc-6 K07C11.9 0.00550247081177879
AGIWUR44423
CB4856 CT V 8221410 8223587 WBGene00000098 air-1 K07C11.2 -0.0685467321574436
AGIWUR1464;AGIWUR10508;AGIWUR24452
CB4856 CT V 8224965 8226037 WBGene00003937 pax-1 K07C11.1 0.313434010522532
AGIWUR6074
CB4856 CT V 8226061 8226852 WBGene00019482 K07C11.10 K07C11.10 0.295667530684154
AGIWUR23380
CB4856 CT V 8228300 8238409 WBGene00004395 rol-3 C16D9.2 -0.240409483831695
AGIWUR28970;AGIWUR41147
CB4856 CT V 8238959 8240021 WBGene00015858 C16D9.3 C16D9.3 -0.0376375018417487
AGIWUR2918;AGIWUR12819;AGIWUR13836;AGIWUR22036;AGIWUR25212;AGIWUR26109;AGIWUR37663;AGIWUR40907;AGIWUR41937
CB4856 CT V 8240928 8242898 WBGene00015859 C16D9.4 C16D9.4 0.176195445690858 AGIWUR39886
CB4856 CT V 8243709 8245400 WBGene00015860 C16D9.5 C16D9.5 -0.144058101376046
AGIWUR7962;AGIWUR25590;AGIWUR41064
CB4856 CT V 8248123 8250289 WBGene00015861 C16D9.6 C16D9.6 -0.306286892566844 AGIWUR15092
CB4856 CT V 8256831 8258076 WBGene00015862 srt-25 C16D9.7 0.0923975391320857 AGIWUR9495
CB4856 CT V 8259316 8261315 WBGene00015857 C16D9.1 C16D9.1 -0.107297986838262 AGIWUR16555
CB4856 CT V 8261390 8263566 WBGene00015863 C16D9.8 C16D9.8 -0.0907846171565119 AGIWUR11845
CB4856 CT V 8264241 8264663 WBGene00015864 C16D9.9 C16D9.9 0.583422519378304 AGIWUR40396
CB4856 CT V 8265202 8266385 WBGene00017679 F21F8.5 F21F8.5 -0.0602075254515686 AGIWUR890
CB4856 CT V 8267511 8268937 WBGene00000219 asp-6 F21F8.7 0.378197001124676 AGIWUR29921
CB4856 CT V 8269254 8272058 WBGene00017678 asp-12 F21F8.4 0.227481116432962 AGIWUR24711
CB4856 CT V 8272375 8274046 WBGene00000218 asp-5 F21F8.3 0.0275924167925048
AGIWUR5560;AGIWUR23001;AGIWUR28611;AGIWUR31817
CB4856 CT V 8274353 8275757 WBGene00017677 asp-11 F21F8.2 1.57830526742697 AGIWUR24174
CB4856 CT V 8275898 8277041 WBGene00006185 str-137 F21F8.8 0.0988283614083749
AGIWUR20524;AGIWUR37534
CB4856 CT V 8277516 8278930 WBGene00006184 str-136 F21F8.9 0.079558551894916 AGIWUR42835
CB4856 CT V 8279375 8282167 WBGene00006183 str-135 F21F8.10 -0.124517487885336

AGIWUR22082
CB4856 CT V 8282333 8284900 WBGene00017681 slc-17.5 F21F8.11 -0.121835445806388
AGIWUR9561
CB4856 CT V 8292064 8293516 WBGene00005190 srg-33 F21F8.1 0.179819132658851 AGIWUR38187
CB4856 CT V 8293809 8294900 WBGene00020939 W02D7.3 W02D7.3 0.202385963657223 AGIWUR37768
CB4856 CT V 8296558 8297604 WBGene00020940 W02D7.4 W02D7.4 -0.040439570492552 AGIWUR7250
CB4856 CT V 8297700 8299244 WBGene00020941 W02D7.5 W02D7.5 -0.185481724686733 AGIWUR41016
CB4856 CT V 8299753 8301507 WBGene00020942 W02D7.6 W02D7.6 -0.0108776019823225 AGIWUR2754
CB4856 CT V 8301490 8302580 WBGene00004766 sel-9 W02D7.7 -0.20218173177949
AGIWUR305;AGIWUR3250;AGIWUR5175;AGIWUR6945;AGIWUR8272;AGIWUR17351;AGIWUR19473
CB4856 CT V 8302887 8304469 WBGene00020946 W02D7.11 W02D7.11 1.36858708586813
AGIWUR13639
CB4856 CT V 8305120 8306460 WBGene00020943 W02D7.8 W02D7.8 1.14236886824912 AGIWUR28197
CB4856 CT V 8307942 8310766 WBGene00020938 clec-218 W02D7.2 0.728738441871312
AGIWUR39060;AGIWUR41065
CB4856 CT V 8311510 8312041 WBGene00044356 W02D7.12 W02D7.12 0.118165780466729
AGIWUR27526
CB4856 CT V 8318435 8322863 WBGene00020945 clec-219 W02D7.10 -0.333631495295343
AGIWUR25784
CB4856 CT V 8323426 8323885 WBGene00023354 W02D7.1 W02D7.1 0.0900453892091617
AGIWUR5151;AGIWUR11873;AGIWUR23778;AGIWUR28332
CB4856 CT V 8326191 8326514 WBGene00020944 W02D7.9 W02D7.9 0.0525293524339823 AGIWUR41935
CB4856 CT V 8336658 8340675 WBGene00006582 tmd-2 C08D8.2 0.151367334313045
AGIWUR24109;AGIWUR37546
CB4856 CT V 8345759 8349909 WBGene00015592 C08D8.1 C08D8.1 0.59656079070176 AGIWUR31565
CB4856 CT V 8351671 8353085 WBGene00018180 srsx-22 F38E1.8 0.0953735018914372 AGIWUR42326
CB4856 CT V 8353793 8355008 WBGene00044126 srsx-23 F38E1.12 0.00466639091948196
AGIWUR32398;AGIWUR33303
CB4856 CT V 8355953 8358185 WBGene00003395 mom-2 F38E1.7 -0.0300671188022275
AGIWUR9372;AGIWUR18335;AGIWUR22584;AGIWUR36859;AGIWUR40063
CB4856 CT V 8358203 8359163 WBGene00018181 F38E1.9 F38E1.9 -0.281961133864455
AGIWUR17431;AGIWUR24194
CB4856 CT V 8359818 8361394 WBGene00018179 F38E1.6 F38E1.6 -0.0415005328651192 AGIWUR11093
CB4856 CT V 8371014 8374027 WBGene00001664 gpa-2 F38E1.5 0.0197009219225421 AGIWUR29092
CB4856 CT V 8374027 8375474 WBGene00018182 F38E1.10 F38E1.10 -0.187947718602115
AGIWUR12738
CB4856 CT V 8376117 8377944 WBGene00018178 F38E1.3 F38E1.3 -0.0891278670389859 AGIWUR6638
CB4856 CT V 8378903 8379925 WBGene00018183 F38E1.11 F38E1.11 0.0160464868744494
AGIWUR34757

CB4856	CT	V	8379474	8383747	WBGene00018694	F52E1.5	F52E1.5	0.84924020758023	AGIWUR9540;AGIWUR37783
CB4856	CT	V	8383922	8384867	WBGene00044696	F52E1.14	F52E1.14	-0.0238473597772648	AGIWUR8044;AGIWUR9472;AGIWUR17987;AGIWUR34787;AGIWUR37989;AGIWUR42621
CB4856	CT	V	8384774	8386268	WBGene00002021	hsp-17	F52E1.7	-0.765839619563154	AGIWUR43590
CB4856	CT	V	8387356	8392324	WBGene00001534	gcy-7	F52E1.4	0.0141942390601502	AGIWUR37358
CB4856	CT	V	8392357	8394383	WBGene00018696	pho-6	F52E1.8	1.06873093509037	AGIWUR24061
CB4856	CT	V	8394800	8396344	WBGene00018693	F52E1.3	F52E1.3	0.265469450050997	AGIWUR42980
CB4856	CT	V	8398145	8402669	WBGene00018697	F52E1.9	F52E1.9	0.258769682798165	AGIWUR32593
CB4856	CT	V	8410595	8411831	WBGene00018692	F52E1.2	F52E1.2	0.143474947645775	AGIWUR18541
CB4856	CT	V	8411775	8413513	WBGene00018698	vha-18	F52E1.10	-0.0226496698748591	AGIWUR24887
CB4856	CT	V	8414085	8415254	WBGene00004078	pos-1	F52E1.1	-0.0265100075069888	AGIWUR7897
CB4856	CT	V	8415687	8444773	WBGene00018700	lmd-3	F52E1.13	0.191581471049438	AGIWUR2888;AGIWUR29288;AGIWUR40480;AGIWUR43671
CB4856	CT	V	8446608	8448009	WBGene00020720	T23B12.5	T23B12.5	-0.237228849098894	AGIWUR43508
CB4856	CT	V	8455396	8457606	WBGene00044553	T23B12.11	T23B12.11	-0.171072560518204	AGIWUR21695;AGIWUR33424
CB4856	CT	V	8457616	8460419	WBGene00020721	T23B12.6	T23B12.6	-0.0900721423929536	AGIWUR33383;AGIWUR39766;AGIWUR45189
CB4856	CT	V	8461096	8462116	WBGene00001040	dnj-22	T23B12.7	-0.213668289231979	AGIWUR4124
CB4856	CT	V	8462327	8465569	WBGene00020719	natc-1	T23B12.4	0.0866550987823338	AGIWUR41325
CB4856	CT	V	8465617	8467159	WBGene00020718	mrps-2	T23B12.3	-0.114536589574207	AGIWUR42622
CB4856	CT	V	8467265	8468943	WBGene00020717	mrpl-4	T23B12.2	-0.397764961089687	AGIWUR1921;AGIWUR3134;AGIWUR8568;AGIWUR26621
CB4856	CT	V	8469040	8470072	WBGene00020716	phf-30	T23B12.1	-0.110014741072018	AGIWUR4944;AGIWUR7828;AGIWUR43934
CB4856	CT	V	8470013	8471244	WBGene00020722	T23B12.8	T23B12.8	0.0150457456805047	AGIWUR3007;AGIWUR8513
CB4856	CT	V	8494889	8502336	WBGene00004256	pxn-1	ZK994.3	-0.324829713890459	AGIWUR29049;AGIWUR39160
CB4856	CT	V	8502591	8504591	WBGene00022842	ZK994.6	ZK994.6	0.0401758518116466	AGIWUR38282
CB4856	CT	V	8510237	8512663	WBGene00022840	ZK994.1	ZK994.1	0.162985968134017	AGIWUR26288
CB4856	CT	V	8512689	8513463	WBGene00018481	F45F2.9	F45F2.9	0.0081808645770316	AGIWUR3538;AGIWUR12227;AGIWUR14370;AGIWUR18337;AGIWUR30838;AGIWUR37139

CB4856	CT	V	8513577	8518764	WBGene00018482	F45F2.10	F45F2.10	-0.0359783288200646	AGIWUR20897
CB4856	CT	V	8519346	8520896	WBGene00018483	F45F2.11	F45F2.11	0.0223702485271176	AGIWUR34803
CB4856	CT	V	8521503	8524555	WBGene00018480	otpl-7	F45F2.7	-0.109580873237041	AGIWUR1825
CB4856	CT	V	8525908	8528580	WBGene00018479	otpl-6	F45F2.6	0.101874770145683	AGIWUR1512
CB4856	CT	V	8529519	8532465	WBGene00018478	otpl-5	F45F2.5	-0.199732626185039	AGIWUR999
CB4856	CT	V	8537331	8539614	WBGene00018477	otpl-4	F45F2.1	-0.0561434255204603	AGIWUR5463
CB4856	CT	V	8539728	8545566	WBGene00017798	slc-17.8	F25G6.7	-0.0512609680187648	AGIWUR40015
CB4856	CT	V	8546128	8548880	WBGene00003816	asns-1	F25G6.6	-0.532695115571846	AGIWUR24276
CB4856	CT	V	8549289	8551165	WBGene00006435	stdh-4	F25G6.5	-0.337116185114631	AGIWUR23729
CB4856	CT	V	8552695	8555388	WBGene00000054	acr-15	F25G6.4	-0.0536132847084509	AGIWUR44252
CB4856	CT	V	8561216	8564345	WBGene00000055	acr-16	F25G6.3	-0.305724549808294	AGIWUR16589;AGIWUR21014;AGIWUR42576
CB4856	CT	V	8564303	8565175	WBGene00017799	F25G6.8	F25G6.8	-0.143303972392554	AGIWUR39719
CB4856	CT	V	8565275	8570077	WBGene00017800	F25G6.9	F25G6.9	0.320930674800572	AGIWUR11988
CB4856	CT	V	8570311	8581397	WBGene00017797	symk-1	F25G6.2	-0.228023649181177	AGIWUR4549;AGIWUR20084
CB4856	CT	V	8581261	8583576	WBGene00015670	C10B5.1	C10B5.1	0.127060919773617	AGIWUR36163
CB4856	CT	V	8583553	8584554	WBGene00015671	C10B5.3	C10B5.3	0.195040635881027	AGIWUR28344
CB4856	CT	V	8594914	8597500	WBGene00007055	tag-196	F41E6.6	0.769191912566377	AGIWUR30420
CB4856	CT	V	8597851	8599292	WBGene00018288	F41E6.7	F41E6.7	-0.271378416145703	AGIWUR17625
CB4856	CT	V	8599584	8601412	WBGene00018289	F41E6.8	F41E6.8	0.198835742043252	AGIWUR31859
CB4856	CT	V	8602084	8602825	WBGene00044554	F41E6.15	F41E6.15	0.286266498027905	AGIWUR12689;AGIWUR20045;AGIWUR24139;AGIWUR36787;AGIWUR37343
CB4856	CT	V	8603480	8605469	WBGene00018286	F41E6.5	F41E6.5	1.59802150966198	AGIWUR16835
CB4856	CT	V	8607800	8615841	WBGene00018285	smk-1	F41E6.4	-0.0812449072294243	AGIWUR654;AGIWUR5459;AGIWUR13596;AGIWUR14276;AGIWUR14346;AGIWUR15894;AGIWUR18138;AGIWUR18721;AGIWUR22723;AGIWUR22728
CB4856	CT	V	8619032	8619869	WBGene00001694	grd-5	F41E6.2	-0.643080456845784	AGIWUR25134
CB4856	CT	V	8619925	8621348	WBGene00018292	F41E6.11	F41E6.11	-0.762208182403098	AGIWUR16678
CB4856	CT	V	8626900	8629129	WBGene00018293	F41E6.12	F41E6.12	-0.730678242885951	AGIWUR31874
CB4856	CT	V	8629223	8631325	WBGene00018294	atg-18	F41E6.13	0.45225812753149	AGIWUR9216;AGIWUR10459;AGIWUR27595
CB4856	CT	V	8631871	8635675	WBGene00018295	oac-29	F41E6.14	1.54281349173701	AGIWUR6170

CB4856	CT	V	8635820	8637006	WBGene00001721	grl-12	F28A12.2	-0.348482205271598	AGIWUR39621
CB4856	CT	V	8638237	8640585	WBGene00017879	acd-4	F28A12.1	-0.377972476814096	AGIWUR23065
CB4856	CT	V	8640596	8641344	WBGene00017880	F28A12.3	F28A12.3	-0.650703836640947	AGIWUR39240
CB4856	CT	V	8641491	8643225	WBGene00017881	asp-13	F28A12.4	-0.305732777068977	AGIWUR6336
CB4856	CT	V	8666065	8671680	WBGene00022538	ZC190.4	ZC190.4	0.126352911737318	AGIWUR3159
CB4856	CT	V	8674602	8677293	WBGene00022540	ZC190.6	ZC190.6	0.46664543125485	AGIWUR4434
CB4856	CT	V	8681350	8682939	WBGene00022537	ZC190.2	ZC190.2	-0.236292398448894	AGIWUR22064
CB4856	CT	V	8682955	8686160	WBGene00000541	cln-3.3	ZC190.1	-0.59509189298532	AGIWUR40054
CB4856	CT	V	8686071	8686864	WBGene00022541	ZC190.7	ZC190.7	0.0448980038995179	AGIWUR30409
CB4856	CT	V	8686958	8688435	WBGene00022542	ZC190.8	ZC190.8	-0.193831313062669	AGIWUR41198
CB4856	CT	V	8694329	8696507	WBGene00022544	ZC190.10	ZC190.10	-0.0353842988448498	AGIWUR30061
CB4856	CT	V	8701432	8703060	WBGene00022535	ZC178.1	ZC178.1	0.0377377667613557	AGIWUR35182
CB4856	CT	V	8705437	8709880	WBGene00022536	ZC178.2	ZC178.2	-0.0139244378393322	AGIWUR19531
CB4856	CT	V	8716495	8720893	WBGene00001616	glr-5	ZC196.7	0.229655248073819	AGIWUR8923;AGIWUR33190
CB4856	CT	V	8721198	8722260	WBGene00022551	ZC196.8	ZC196.8	-0.133165716861197	AGIWUR10032
CB4856	CT	V	8726516	8728894	WBGene00022550	droe-8	ZC196.6	1.46386604607961	AGIWUR16914;AGIWUR36682;AGIWUR37378
CB4856	CT	V	8729458	8732789	WBGene00022549	ZC196.5	ZC196.5	-0.207001358169197	AGIWUR34777
CB4856	CT	V	8733106	8735781	WBGene00022548	ZC196.4	ZC196.4	0.139804280753262	AGIWUR8712
CB4856	CT	V	8736674	8738437	WBGene00022547	ZC196.3	ZC196.3	-0.289966362679069	AGIWUR38070
CB4856	CT	V	8739437	8742259	WBGene00022546	ZC196.2	ZC196.2	-0.132314423011067	AGIWUR26730
CB4856	CT	V	8742804	8745045	WBGene00022545	ZC196.1	ZC196.1	0.110127875663737	AGIWUR21259;AGIWUR38916;AGIWUR39614
CB4856	CT	V	8753695	8754873	WBGene00015222	B0507.5	B0507.5	-0.0111997424680892	AGIWUR12863
CB4856	CT	V	8754970	8755759	WBGene00015221	B0507.4	B0507.4	0.173860898341324	AGIWUR44217
CB4856	CT	V	8755897	8757098	WBGene00235307	B0507.17	B0507.17	0.18050307170998	AGIWUR20645
CB4856	CT	V	8760687	8763194	WBGene00015225	B0507.8	B0507.8	2.84241513922001	AGIWUR2410
CB4856	CT	V	8764888	8766038	WBGene00015226	B0507.9	B0507.9	0.109348277141566	AGIWUR25026
CB4856	CT	V	8768909	8770876	WBGene00015220	B0507.3	B0507.3	1.08249740911936	AGIWUR10104
CB4856	CT	V	8771899	8773781	WBGene00006006	srx-115	B0507.11	-0.21999549652687	AGIWUR14341;AGIWUR19285
CB4856	CT	V	8774332	8776630	WBGene00015219	B0507.2	B0507.2	0.287400269314574	AGIWUR12931

CB4856 CT V 8776570 8779318 WBGene00015218 B0507.1 B0507.1 0.440965918861703 AGIWUR10922
CB4856 CT V 8783422 8784165 WBGene00015275 C01B7.3 C01B7.3 0.103086207420364 AGIWUR6935
CB4856 CT V 8786551 8792771 WBGene00006342 sup-37 C01B7.1 0.0389330607515337
AGIWUR12890;AGIWUR24197;AGIWUR33572
CB4856 CT V 8792696 8795567 WBGene00006467 magu-2 C01B7.4 0.255663440599118
AGIWUR6705;AGIWUR7116;AGIWUR29311
CB4856 CT V 8800151 8800996 WBGene00015276 C01B7.5 C01B7.5 -0.135398300604678 AGIWUR31037
CB4856 CT V 8805416 8819955 WBGene00004457 rpm-1 C01B7.6 0.361058776656979 AGIWUR13055
CB4856 CT V 8865351 8867297 WBGene00017192 F07B7.14 F07B7.14 -0.142623753333028
AGIWUR21155
CB4856 CT V 8876035 8877015 WBGene00017152 EGAP798.1 EGAP798.1 -0.0490501391969778
AGIWUR42453
CB4856 CT V 8898195 8898645 WBGene00001892 his-18 K06C4.10 -0.525477848836885
AGIWUR14430
CB4856 CT V 8899276 8902221 WBGene00019448 frpr-13 K06C4.17 0.175599911562744
AGIWUR41740
CB4856 CT V 8902339 8905364 WBGene00019445 frpr-12 K06C4.9 -0.00615992162239021 AGIWUR17925
CB4856 CT V 8907629 8909442 WBGene00019444 frpr-11 K06C4.8 -0.0768722722625697 AGIWUR7490
CB4856 CT V 8909725 8914033 WBGene00003386 mod-1 K06C4.6 -0.0190020656306356
AGIWUR19981;AGIWUR24514;AGIWUR44071
CB4856 CT V 8914915 8916795 WBGene00019443 K06C4.7 K06C4.7 0.0383605366800133 AGIWUR14865
CB4856 CT V 8923088 8923936 WBGene00019203 ttr-47 H14N18.3 -0.309156803389126
AGIWUR16282
CB4856 CT V 8923900 8928938 WBGene00019204 H14N18.4 H14N18.4 0.0915056898554534
AGIWUR4412;AGIWUR22862;AGIWUR24807;AGIWUR27204;AGIWUR33845;AGIWUR35410
CB4856 CT V 8932618 8934265 WBGene00019202 H14N18.2 H14N18.2
-0.00202404036137427 AGIWUR19763
CB4856 CT V 8936359 8939676 WBGene00006760 unc-23 H14N18.1 -0.172409282605656
AGIWUR36885
CB4856 CT V 8939592 8944063 WBGene00020554 T19A5.3 T19A5.3 0.24399617199978
AGIWUR9403;AGIWUR21691;AGIWUR24644;AGIWUR24708;AGIWUR26711;AGIWUR39995;AGIWUR40351
CB4856 CT V 8947799 8956294 WBGene00001526 gck-1 T19A5.2 0.0812249682406465
AGIWUR7524;AGIWUR14367;AGIWUR20465;AGIWUR25186;AGIWUR39460;AGIWUR40989
CB4856 CT V 8957045 8961935 WBGene00020553 T19A5.1 T19A5.1 0.0813636975912821 AGIWUR27563
CB4856 CT V 8961915 8964099 WBGene00003634 nhr-44 T19A5.4 0.413090332107636 AGIWUR18736
CB4856 CT V 8964711 8968406 WBGene00020555 nhr-219 T19A5.5 0.345675741304402 AGIWUR15634
CB4856 CT V 8968737 8969955 WBGene00019114 nhr-194 F59E11.8 0.196217289318826
AGIWUR42742
CB4856 CT V 8970744 8972364 WBGene00019115 nhr-195 F59E11.10 0.511639535047685

AGIWUR42909
CB4856 CT V 8973182 8974943 WBGene00019116 nhr-143 F59E11.11 0.364366985100117
AGIWUR26665
CB4856 CT V 8975698 8977465 WBGene00019117 nhr-144 F59E11.12 0.119905754939321
AGIWUR2070;AGIWUR5823;AGIWUR7596;AGIWUR21861;AGIWUR25053;AGIWUR41239
CB4856 CT V 8979522 8980929 WBGene00006143 str-84 F59E11.13 -0.337468160889019
AGIWUR27796
CB4856 CT V 8982387 8983689 WBGene00006145 str-86 F59E11.14 -0.0963034362431339
AGIWUR12913
CB4856 CT V 8984525 8985840 WBGene00006144 str-85 F59E11.15 -0.345794944288808
AGIWUR44467
CB4856 CT V 8986694 8987965 WBGene00006146 str-87 F59E11.16 -0.140436570036789
AGIWUR3731
CB4856 CT V 8990244 8991664 WBGene00019113 F59E11.7 F59E11.7 0.72602807957768
AGIWUR3934;AGIWUR7034;AGIWUR9590;AGIWUR10453;AGIWUR16102;AGIWUR30847
CB4856 CT V 8991789 8993142 WBGene00019112 F59E11.6 F59E11.6 0.0217396341652858
AGIWUR14515
CB4856 CT V 8995101 8996664 WBGene00019111 F59E11.5 F59E11.5 0.00244294640997467
AGIWUR21952
CB4856 CT V 8998599 9001352 WBGene00005535 sri-23 F59E11.3 0.277139295835013
AGIWUR44649
CB4856 CT V 9001475 9003100 WBGene00019109 F59E11.2 F59E11.2 0.321646374443454
AGIWUR9207;AGIWUR11718;AGIWUR41284
CB4856 CT V 9003709 9004967 WBGene00005892 srx-1 F59E11.1 0.0828291799306742
AGIWUR9999
CB4856 CT V 9005543 9006931 WBGene00005894 srx-3 R03H4.3 0.0215208809541085 AGIWUR26052
CB4856 CT V 9009475 9010788 WBGene00005893 srx-2 R03H4.2 0.256578258200793 AGIWUR9140
CB4856 CT V 9011244 9011565 WBGene00023327 R03H4.8 R03H4.8 0.0422965344173249
AGIWUR7761;AGIWUR13453;AGIWUR19516;AGIWUR25492;AGIWUR37990
CB4856 CT V 9012199 9013865 WBGene00005412 srh-201 R03H4.9 -0.00567259030967637 AGIWUR3855
CB4856 CT V 9014408 9015640 WBGene00019850 srt-18 R03H4.4 0.0221935189463096 AGIWUR6053
CB4856 CT V 9016864 9019318 WBGene00019849 oac-40 R03H4.1 -0.0393922261434341 AGIWUR14055
CB4856 CT V 9019595 9022178 WBGene00019851 oac-41 R03H4.5 0.0528148593365305 AGIWUR13765
CB4856 CT V 9022361 9024806 WBGene00044617 bus-1 R03H4.6 -0.0484898285893083
AGIWUR6644;AGIWUR9344
CB4856 CT V 9025513 9026540 WBGene00019853 srsx-20 R03H4.7 -0.0191688443614519
AGIWUR2800;AGIWUR18747;AGIWUR30131;AGIWUR35466
CB4856 CT V 9030548 9032593 WBGene00016095 C25E10.5 C25E10.5 -0.012017350012659
AGIWUR32429

CB4856	CT	V	9033567 9035605 WBGene00016094	C25E10.4	C25E10.4	-0.414255243329942
AGIWUR5021
CB4856	CT	V	9036236 9040289 WBGene00016093	srsx-34 C25E10.3	0.465908890845911
AGIWUR14246;AGIWUR22371;AGIWUR42503;AGIWUR42809
CB4856	CT	V	9041330 9043677 WBGene00016092	cyp-33B1	C25E10.2	0.293591830357933
AGIWUR816;AGIWUR6140;AGIWUR39399
CB4856	CT	V	9045171 9046967 WBGene00016091	nhr-30	C25E10.1	0.262448059851175
AGIWUR17500
CB4856	CT	V	9050102 9051191 WBGene00016096	C25E10.7	C25E10.7	0.0899174891986023
AGIWUR44248
CB4856	CT	V	9053322 9053995 WBGene00016097	C25E10.8	C25E10.8	-0.0734743508767313
AGIWUR386;AGIWUR11321;AGIWUR12719;AGIWUR26576
CB4856	CT	V	9054268 9054844 WBGene00023417	swm-1	C25E10.9	0.0408964206648292
AGIWUR435;AGIWUR3111;AGIWUR11880;AGIWUR15292
CB4856	CT	V	9055326 9056392 WBGene00016099	C25E10.10	C25E10.10	-0.33006036124511
AGIWUR16698
CB4856	CT	V	9057543 9058332 WBGene00016100	C25E10.11	C25E10.11	0.151609215767603
AGIWUR35724
CB4856	CT	V	9059750 9060481 WBGene00023431	C25E10.13	C25E10.13	0.234232305990439
AGIWUR10531;AGIWUR28693;AGIWUR31323;AGIWUR40791;AGIWUR41640
CB4856	CT	V	9060494 9062441 WBGene00016101	C25E10.12	C25E10.12	-0.0473662527652365
AGIWUR18021
CB4856	CT	V	9067976 9068390 WBGene00020862	fip-2	T27E4.4 0.809401751412789	AGIWUR28034
CB4856	CT	V	9068368 9069694 WBGene00020863	T27E4.5 T27E4.5 -0.102540435025249
AGIWUR1035;AGIWUR8082;AGIWUR15817;AGIWUR22970
CB4856	CT	V	9069865 9073528 WBGene00020864	oac-50	T27E4.6 0.457934062329297	AGIWUR36336
CB4856	CT	V	9074784 9076322 WBGene00020865	T27E4.7 T27E4.7 0.566417889110898
AGIWUR34270;AGIWUR35657;AGIWUR37836
CB4856	CT	V	9092345 9094345 WBGene00020861	T27E4.1 T27E4.1 0.27477220126586
AGIWUR639;AGIWUR19829;AGIWUR29731
CB4856	CT	V	9096007 9097233 WBGene00017659	F21C10.10	F21C10.10	2.45847187875335
AGIWUR25059
CB4856	CT	V	9097121 9098696 WBGene00017658	F21C10.9	F21C10.9	0.299305198904787
AGIWUR7021;AGIWUR8613;AGIWUR11231;AGIWUR12515;AGIWUR12623;AGIWUR44908
CB4856	CT	V	9099638 9101133 WBGene00017661	frpr-6	F21C10.12	0.0708996344388506
AGIWUR11739
CB4856	CT	V	9109584 9111013 WBGene00004119	pqn-31	F21C10.8	0.875031722964305
AGIWUR10968
CB4856	CT	V	9112767 9122180 WBGene00017657	F21C10.7	F21C10.7	-0.122716584427666

AGIWUR43657
CB4856 CT V 9123489 9123809 WBGene00017660 F21C10.11 F21C10.11 0.562005745806944
AGIWUR1058;AGIWUR5369;AGIWUR7951;AGIWUR8268;AGIWUR14237;AGIWUR24278
CB4856 CT V 9124480 9124994 WBGene00017656 F21C10.6 F21C10.6 0.0107858891539854
AGIWUR43227
CB4856 CT V 9125645 9126006 WBGene00017655 F21C10.5 F21C10.5 0.212223892637622
AGIWUR34079
CB4856 CT V 9126679 9127593 WBGene00017654 F21C10.4 F21C10.4 0.130892702123601
AGIWUR25641
CB4856 CT V 9128710 9130943 WBGene00017653 F21C10.3 F21C10.3 0.331899922090763
AGIWUR3740
CB4856 CT V 9132384 9133275 WBGene00017652 F21C10.1 F21C10.1 0.263051628297699
AGIWUR35855
CB4856 CT V 9134255 9135347 WBGene00015053 B0222.1 B0222.1 0.116593436314711 AGIWUR14873
CB4856 CT V 9135656 9137718 WBGene00015054 pitr-2 B0222.2 0.187510387016271 AGIWUR11677
CB4856 CT V 9138946 9141397 WBGene00015055 pitr-3 B0222.3 0.114433439569228
AGIWUR12154;AGIWUR17374;AGIWUR25525;AGIWUR26220
CB4856 CT V 9144630 9146899 WBGene00006418 tag-38 B0222.4 0.530367725493367 AGIWUR12107
CB4856 CT V 9152321 9154872 WBGene00015056 B0222.5 B0222.5 -0.407312144180967 AGIWUR11803
CB4856 CT V 9162051 9163005 WBGene00000718 col-145 B0222.7 -0.246022073542658 AGIWUR11578
CB4856 CT V 9164297 9165307 WBGene00000599 col-10 B0222.8 -0.297960027860482 AGIWUR33641
CB4856 CT V 9165820 9168041 WBGene00044582 B0222.11 B0222.11 -0.0991683901553002
AGIWUR15091;AGIWUR20798
CB4856 CT V 9168393 9173024 WBGene00015057 gad-3 B0222.9 0.130250362565613 AGIWUR35004
CB4856 CT V 9174089 9175250 WBGene00004107 pqn-16 C24B5.5 0.545786724049297 AGIWUR31890
CB4856 CT V 9175798 9177997 WBGene00016045 spas-1 C24B5.2 -0.201966151696773
AGIWUR5281;AGIWUR20143
CB4856 CT V 9177949 9181719 WBGene00004216 ptr-1 C24B5.3 0.0176566212625904 AGIWUR9758
CB4856 CT V 9191617 9195877 WBGene00016044 C24B5.1 C24B5.1 0.262470012177811 AGIWUR35120
CB4856 CT V 9195821 9197326 WBGene00016046 C24B5.4 C24B5.4 0.672320486542134 AGIWUR28612
CB4856 CT V 9197751 9198987 WBGene00006221 str-177 T18H9.4 0.196264408844026
AGIWUR2394;AGIWUR11610;AGIWUR18614;AGIWUR33614
CB4856 CT V 9199680 9204734 WBGene00000215 asp-2 T18H9.2 -0.264339138903431 AGIWUR17206
CB4856 CT V 9204703 9207467 WBGene00002132 inx-10 T18H9.5 -0.0408735712239547
AGIWUR9782;AGIWUR15209;AGIWUR34590
CB4856 CT V 9209463 9213654 WBGene00007023 mdt-27 T18H9.6 -0.212609700731941 AGIWUR33734
CB4856 CT V 9213746 9217243 WBGene00044077 tag-232 T18H9.7 0.0321112657704283 AGIWUR15379
CB4856 CT V 9221866 9224354 WBGene00001695 grd-6 T18H9.1 -0.645928838934299 AGIWUR8669
CB4856 CT V 9226094 9227523 WBGene00017193 F07C3.2 F07C3.2 -0.174356317700832 AGIWUR28489

CB4856 CT V 9227710 9232396 WBGene00017194 F07C3.3 F07C3.3 -0.301238437200208 AGIWUR2492
CB4856 CT V 9234610 9243221 WBGene00017195 glo-4 F07C3.4 0.205330931728416
AGIWUR18442;AGIWUR21236;AGIWUR30949;AGIWUR33447;AGIWUR35250;AGIWUR39020
CB4856 CT V 9244402 9246360 WBGene00000003 aat-2 F07C3.7 0.264758526816815
AGIWUR8898;AGIWUR30371;AGIWUR33955;AGIWUR43403
CB4856 CT V 9250275 9254223 WBGene00004211 ptd-2 F07C3.1 0.06110307991647 AGIWUR19663
CB4856 CT V 9254219 9254632 WBGene00017197 F07C3.9 F07C3.9 0.149936642667399 AGIWUR36578
CB4856 CT V 9256864 9261124 WBGene00017198 nhr-36 F07C3.10 -0.00961704367724625
AGIWUR5452
CB4856 CT V 9263800 9266499 WBGene00022788 slc-17.7 ZK682.2 -0.151440949944435
AGIWUR27401
CB4856 CT V 9280702 9282154 WBGene00001954 hlh-10 ZK682.4 0.0599616191966028 AGIWUR4665
CB4856 CT V 9284129 9287617 WBGene00022789 lron-2 ZK682.5 -0.35275181346446 AGIWUR4188
CB4856 CT V 9290683 9292118 WBGene00022790 ZK682.7 ZK682.7 0.00860965174920381 AGIWUR7624
CB4856 CT V 9297632 9302158 WBGene00018405 F44A2.5 F44A2.5 0.320857302098405
AGIWUR10464;AGIWUR10630;AGIWUR43500
CB4856 CT V 9303326 9304622 WBGene00018404 nhr-39 F44A2.4 0.289744920622692 AGIWUR25387
CB4856 CT V 9305416 9310926 WBGene00018403 F44A2.3 F44A2.3 -0.0641897414641655 AGIWUR5487
CB4856 CT V 9318311 9320995 WBGene00018402 kvs-3 F44A2.2 0.168317959235049 AGIWUR34457
CB4856 CT V 9321503 9322905 WBGene00018406 F44A2.7 F44A2.7 0.204372907459965 AGIWUR39794
CB4856 CT V 9323085 9327209 WBGene00006499 tag-153 F44A2.1 -0.168815320113491
AGIWUR3118;AGIWUR11728;AGIWUR21919;AGIWUR31459;AGIWUR34827;AGIWUR41537
CB4856 CT V 9327145 9333062 WBGene00019465 acl-14 K07B1.5 -0.126733445805076
AGIWUR5902;AGIWUR21744;AGIWUR42125
CB4856 CT V 9334773 9336668 WBGene00006729 ucp-4 K07B1.3 -0.0400896875008758 AGIWUR23287
CB4856 CT V 9336762 9338858 WBGene00000766 coq-6 K07B1.2 -0.104896643906715 AGIWUR24967
CB4856 CT V 9338859 9344218 WBGene00019466 tos-1 K07B1.6 0.414547195793737
AGIWUR7103;AGIWUR19971;AGIWUR23532;AGIWUR25576;AGIWUR27157;AGIWUR29383;AGIWUR30086;AGIWUR30658;AGIWUR33209;
AGIWUR36988
CB4856 CT V 9345022 9346962 WBGene00019467 K07B1.7 K07B1.7 0.0763289235410847
AGIWUR11067;AGIWUR29876
CB4856 CT V 9345772 9348687 WBGene00006623 try-5 K07B1.1 0.0954448659133114 AGIWUR40119
CB4856 CT V 9349987 9351332 WBGene00005938 srx-47 E02C12.3 -0.0717233201616478
AGIWUR30857
CB4856 CT V 9353247 9354497 WBGene00005937 srx-46 E02C12.2 0.0121385696566998
AGIWUR39729
CB4856 CT V 9354512 9355471 WBGene00017091 ttr-40 E02C12.4 -0.632215770541851
AGIWUR41676
CB4856 CT V 9355505 9356541 WBGene00023485 ttr-49 E02C12.13 -0.517247230772366

AGIWUR9606;AGIWUR10008;AGIWUR23715
CB4856 CT V 9356750 9359774 WBGene00001665 gpa-3 E02C12.5 0.20060890213602
AGIWUR30793
CB4856 CT V 9369549 9371086 WBGene00017092 E02C12.6 E02C12.6 0.936954512853641
AGIWUR25049
CB4856 CT V 9371620 9373613 WBGene00017093 E02C12.8 E02C12.8 -0.137237975063385
AGIWUR16233;AGIWUR41103
CB4856 CT V 9373954 9374842 WBGene00017094 E02C12.9 E02C12.9 0.102318957018037
AGIWUR5285
CB4856 CT V 9375099 9378404 WBGene00017095 E02C12.10 E02C12.10 -0.517895238358542
AGIWUR5101
CB4856 CT V 9379435 9379908 WBGene00017096 E02C12.11 E02C12.11 -0.0400797057492177
AGIWUR34551
CB4856 CT V 9386179 9400062 WBGene00002036 hum-2 F36D4.3 0.0657735893706892
AGIWUR15165;AGIWUR16315;AGIWUR25547
CB4856 CT V 9400327 9401855 WBGene00018088 trpp-4 F36D4.2 -0.0657420318778712 AGIWUR33543
CB4856 CT V 9401805 9406360 WBGene00018089 F36D4.4 F36D4.4 -0.106334587413768 AGIWUR1817
CB4856 CT V 9417056 9419298 WBGene00018087 F36D4.1 F36D4.1 0.161908528376309 AGIWUR36788
CB4856 CT V 9419292 9425762 WBGene00018090 F36D4.5 F36D4.5 -0.116748098143706
AGIWUR6465;AGIWUR6807;AGIWUR7542;AGIWUR11462;AGIWUR23487;AGIWUR27935;AGIWUR35955;AGIWUR39168
CB4856 CT V 9426852 9427523 WBGene00018091 F36D4.6 F36D4.6 -0.0427042263051938 AGIWUR18771
CB4856 CT V 9444050 9460639 WBGene00019237 H24G06.1 H24G06.1 0.303311049773363
AGIWUR8707;AGIWUR38127;AGIWUR44261
CB4856 CT V 9460686 9462705 WBGene00008556 F07D3.3 F07D3.3 -0.0499333108204718 AGIWUR9046
CB4856 CT V 9471975 9473796 WBGene00001449 flp-6 F07D3.2 0.266806963219167 AGIWUR27152
CB4856 CT V 9478163 9479601 WBGene00003522 nas-3 K06A4.1 -0.657967037663075
AGIWUR1048;AGIWUR7670;AGIWUR13476;AGIWUR13931;AGIWUR17705;AGIWUR33871
CB4856 CT V 9484206 9489033 WBGene00010592 K06A4.2 K06A4.2 -0.00661482765036858 AGIWUR5894
CB4856 CT V 9490028 9491715 WBGene00010597 K06A4.7 K06A4.7 0.256672883027781 AGIWUR17974
CB4856 CT V 9497042 9499372 WBGene00010593 gsnl-1 K06A4.3 -0.125330028898578 AGIWUR4604
CB4856 CT V 9501401 9504580 WBGene00010594 swt-5 K06A4.4 0.0116751411058242 AGIWUR10451
CB4856 CT V 9505603 9507049 WBGene00010595 haao-1 K06A4.5 -0.497924374452944 AGIWUR4297
CB4856 CT V 9508079 9510476 WBGene00008669 acs-14 F11A3.1 0.0667299676237878 AGIWUR34411
CB4856 CT V 9510406 9510719 WBGene00077496 F11A3.4 F11A3.4 0.159416234718943 AGIWUR18321
CB4856 CT V 9512506 9513466 WBGene00014734 F11A3.3 F11A3.3 0.11706505280149
AGIWUR5652;AGIWUR23491
CB4856 CT V 9513824 9517585 WBGene00008670 eif-2Bdelta F11A3.2 -0.204039326357613
AGIWUR6022
CB4856 CT V 9517519 9518933 WBGene00008233 C50F4.8 C50F4.8 0.815130913048014 AGIWUR9165

CB4856	CT	V	9521676 9523036 WBGene00014711	C50F4.15	C50F4.15	-0.0192353663901419
AGIWUR4792;AGIWUR8304
CB4856	CT	V	9523167 9524199 WBGene00008234	C50F4.9 C50F4.9 1.51157088463053	AGIWUR3907
CB4856	CT	V	9526698 9529259 WBGene00008229	C50F4.1 C50F4.1 0.666123776995132	AGIWUR39754
CB4856	CT	V	9529951 9531577 WBGene00008235	C50F4.10	C50F4.10	0.11561198289106
AGIWUR42661
CB4856	CT	V	9531819 9534537 WBGene00008230	pfk-1.2 C50F4.2 0.0497130715852734	AGIWUR26495
CB4856	CT	V	9534926 9536404 WBGene00008231	tag-329 C50F4.3 -0.324658240794305
AGIWUR15122;AGIWUR19502;AGIWUR39574
CB4856	CT	V	9536771 9537769 WBGene00008232	C50F4.4 C50F4.4 -0.305032662399956	AGIWUR16123
CB4856	CT	V	9537672 9539738 WBGene00008236	C50F4.12	C50F4.12	0.0209265467615563
AGIWUR24897;AGIWUR34125;AGIWUR34467;AGIWUR37554
CB4856	CT	V	9539844 9541606 WBGene00008238	C50F4.16	C50F4.16	-0.0572328690314787
AGIWUR2870
CB4856	CT	V	9541782 9544226 WBGene00003160	mdf-1	C50F4.11	-0.36777789887592
AGIWUR2261
CB4856	CT	V	9544611 9545398 WBGene00001909	his-35	C50F4.13	-0.121412987875287
AGIWUR5006;AGIWUR6688;AGIWUR9765;AGIWUR19176;AGIWUR25348;AGIWUR38982
CB4856	CT	V	9546092 9546838 WBGene00001910	C50F4.6 C50F4.6 -0.237336273727664	AGIWUR818
CB4856	CT	V	9547083 9547607 WBGene00001911	his-37	C50F4.7 0.0878772514485293
AGIWUR5363;AGIWUR6028
CB4856	CT	V	9547606 9549836 WBGene00008237	nstp-10 C50F4.14	0.0223642223666065
AGIWUR10756;AGIWUR24316;AGIWUR40160
CB4856	CT	V	9550644 9556064 WBGene00009098	ehbp-1	F25B3.1 0.00902386403490496	AGIWUR7819
CB4856	CT	V	9556226 9561643 WBGene00009100	rgef-1	F25B3.3 -0.140553858152323
AGIWUR23351;AGIWUR35083;AGIWUR44618
CB4856	CT	V	9568749 9570295 WBGene00009102	F25B3.5 F25B3.5 0.262255988274658	AGIWUR39474
CB4856	CT	V	9577128 9579946 WBGene00009099	F25B3.2 F25B3.2 -0.0461818389430677	AGIWUR27474
CB4856	CT	V	9580041 9582644 WBGene00009103	rtfo-1	F25B3.6 0.251073545798663	AGIWUR32432
CB4856	CT	V	9583545 9590523 WBGene00010727	K09G1.1 K09G1.1 -0.197431279753715
AGIWUR9171;AGIWUR25494;AGIWUR35434
CB4856	CT	V	9590617 9591255 WBGene00010728	K09G1.2 K09G1.2 -0.272211918104217	AGIWUR21454
CB4856	CT	V	9597351 9597566 WBGene00010729	K09G1.3 K09G1.3 0.0667230680595541	AGIWUR34774
CB4856	CT	V	9608562 9614942 WBGene00001053	dop-2	K09G1.4 -0.196650142965542	AGIWUR40950
CB4856	CT	V	9616031 9617654 WBGene00004146	pqn-63	T06E4.11	-0.340570772760944
AGIWUR39956
CB4856	CT	V	9618233 9619019 WBGene00011537	T06E4.10	T06E4.10	-0.336094022701566
AGIWUR42536
CB4856	CT	V	9620150 9621036 WBGene00011536	T06E4.9 T06E4.9 -0.728753203860752

AGIWUR17247;AGIWUR19509;AGIWUR27759;AGIWUR36271;AGIWUR41455;AGIWUR43957
CB4856 CT V 9622105 9622934 WBGene00011535 T06E4.8 T06E4.8 -0.558952813047537
AGIWUR5829;AGIWUR8168;AGIWUR9243;AGIWUR10103;AGIWUR31043
CB4856 CT V 9626404 9628143 WBGene00011534 T06E4.7 T06E4.7 0.00537478815589765 AGIWUR9548
CB4856 CT V 9628314 9629110 WBGene00044011 T06E4.12 T06E4.12 -0.481894241302239
AGIWUR411;AGIWUR3675;AGIWUR14554;AGIWUR26188;AGIWUR31848
CB4856 CT V 9629943 9631012 WBGene00000719 col-146 T06E4.6 -0.406584019695188
AGIWUR823;AGIWUR3653;AGIWUR6351;AGIWUR15529;AGIWUR28793
CB4856 CT V 9632029 9633338 WBGene00000720 col-147 T06E4.4 -0.140909216467318 AGIWUR7879
CB4856 CT V 9634861 9645595 WBGene00000226 atl-1 T06E4.3 0.0771650311097538
AGIWUR10638;AGIWUR18456;AGIWUR41912
CB4856 CT V 9645746 9650297 WBGene00001830 hcp-2 T06E4.1 -0.12218828160076
AGIWUR1535;AGIWUR38072
CB4856 CT V 9654755 9657975 WBGene00007140 cyp-29A4 B0331.1 -0.712917874328236
AGIWUR26929
CB4856 CT V 9658931 9661614 WBGene00007141 pitr-4 B0331.2 0.883325197945591 AGIWUR21848
CB4856 CT V 9666700 9670050 WBGene00013962 ZK287.1 ZK287.1 0.83010861549208 AGIWUR5092
CB4856 CT V 9670028 9673630 WBGene00013963 sulp-8 ZK287.2 -0.149751078256784 AGIWUR23963
CB4856 CT V 9678277 9679302 WBGene00013964 ZK287.3 ZK287.3 -0.232217079887153 AGIWUR14407
CB4856 CT V 9679357 9680215 WBGene00013968 ZK287.9 ZK287.9 -0.453412057821398
AGIWUR3299;AGIWUR3857;AGIWUR4248;AGIWUR19258;AGIWUR24574;AGIWUR26804;AGIWUR27249;AGIWUR30486;AGIWUR31680;AG
IWUR37264
CB4856 CT V 9680173 9685715 WBGene00013965 ZK287.4 ZK287.4 0.300192713903409 AGIWUR8288
CB4856 CT V 9689686 9690615 WBGene00004320 rbx-1 ZK287.5 -0.0945517433373624
AGIWUR1130;AGIWUR15983;AGIWUR18688;AGIWUR21768;AGIWUR27088;AGIWUR41715
CB4856 CT V 9691039 9692159 WBGene00013966 ztf-9 ZK287.6 -0.274095967313456
AGIWUR11148;AGIWUR11795;AGIWUR15603;AGIWUR16629
CB4856 CT V 9692899 9694785 WBGene00013967 ZK287.7 ZK287.7 -0.0357111680782847 AGIWUR43503
CB4856 CT V 9697129 9701552 WBGene00001842 her-1 ZK287.8 -0.123851922423537
AGIWUR2045;AGIWUR11114;AGIWUR12264;AGIWUR22029;AGIWUR33994;AGIWUR34851
CB4856 CT V 9711447 9713632 WBGene00009586 F40F9.10 F40F9.10 0.310392044570845
AGIWUR3196
CB4856 CT V 9715209 9716380 WBGene00009581 F40F9.3 F40F9.3 0.0163220431644415
AGIWUR3237;AGIWUR12679;AGIWUR24727;AGIWUR42854
CB4856 CT V 9716697 9718051 WBGene00006230 str-188 F40F9.4 0.142086071719478 AGIWUR9381
CB4856 CT V 9718462 9719724 WBGene00014754 F40F9.11 F40F9.11 0.207340863563251
AGIWUR367;AGIWUR4670;AGIWUR8858;AGIWUR8900;AGIWUR17425;AGIWUR30807;AGIWUR40618
CB4856 CT V 9719743 9724118 WBGene00009582 F40F9.5 F40F9.5 0.284791127513021 AGIWUR12858
CB4856 CT V 9725972 9729510 WBGene00009583 aagr-3 F40F9.6 0.0106409302914692 AGIWUR8286

CB4856	CT	V	9730110 9734022 WBGene00009584	drap-1	F40F9.7 -0.094913441781646	AGIWUR6724;AGIWUR11997;AGIWUR15401;AGIWUR15476;AGIWUR22214;AGIWUR34305
CB4856	CT	V	9734103 9735316 WBGene00009585	cal-7	F40F9.8 -0.172489160696692	AGIWUR40235
CB4856	CT	V	9737556 9739740 WBGene00000172	aqp-4	F40F9.9 -0.111236525630837	AGIWUR18766
CB4856	CT	V	9741206 9744326 WBGene00010251	sta-2	F58E6.1 0.0873414866803204	AGIWUR6189;AGIWUR14897;AGIWUR16493;AGIWUR18977;AGIWUR39584;AGIWUR43036
CB4856	CT	V	9745240 9749228 WBGene00077697	F58E6.13	F58E6.13	0.650801072268256	AGIWUR2338;AGIWUR12430
CB4856	CT	V	9749971 9750408 WBGene00010253	F58E6.4 F58E6.4 0.159771651738119	AGIWUR1069
CB4856	CT	V	9752076 9752861 WBGene00010255	F58E6.6 F58E6.6 0.0939761504247496	AGIWUR24247
CB4856	CT	V	9753423 9754581 WBGene00010254	F58E6.5 F58E6.5 -0.313707412632684	AGIWUR18939
CB4856	CT	V	9755076 9755808 WBGene00010257	F58E6.8 F58E6.8 0.0189054224260676	AGIWUR2576;AGIWUR16776;AGIWUR17087;AGIWUR36175
CB4856	CT	V	9756954 9757984 WBGene00010258	F58E6.11	F58E6.11	0.107578325161867	AGIWUR10045
CB4856	CT	V	9764645 9769361 WBGene00006778	unc-42	F58E6.10	1.09950977977002	AGIWUR40416
CB4856	CT	V	9772430 9774881 WBGene00009768	F46B6.2 F46B6.2 0.031953261882899	AGIWUR14437
CB4856	CT	V	9774897 9775834 WBGene00009776	F46B6.12	F46B6.12	-0.178636160659758	AGIWUR24747;AGIWUR42292
CB4856	CT	V	9775935 9777743 WBGene00004882	smg-4	F46B6.3 -0.150208138332879	AGIWUR15546;AGIWUR24438;AGIWUR29576;AGIWUR35194;AGIWUR44130
CB4856	CT	V	9777863 9779518 WBGene00009769	F46B6.4 F46B6.4 -0.205016969875054	AGIWUR28824
CB4856	CT	V	9779550 9783062 WBGene00009770	F46B6.5 F46B6.5 -0.0789967159435321	AGIWUR3043;AGIWUR9088;AGIWUR12125;AGIWUR22800;AGIWUR24084;AGIWUR24726;AGIWUR29962;AGIWUR31112;AGIWUR32190
CB4856	CT	V	9783167 9786868 WBGene00009771	F46B6.6 F46B6.6 -0.0668297551626349	AGIWUR17683
CB4856	CT	V	9786949 9789707 WBGene00009772	ztf-7	F46B6.7 -0.000663669353409446	AGIWUR2863;AGIWUR4966;AGIWUR6956;AGIWUR7130;AGIWUR9529;AGIWUR10038;AGIWUR14760;AGIWUR15597;AGIWUR19058;AGIWUR23791;AGIWUR25977;AGIWUR29172;AGIWUR29972;AGIWUR31314;AGIWUR33929;AGIWUR35152;AGIWUR40593
CB4856	CT	V	9791567 9793234 WBGene00009773	lipl-2	F46B6.8 1.67260166254873	AGIWUR21540
CB4856	CT	V	9796862 9798270 WBGene00009774	F46B6.9 F46B6.9 0.136645531772665	AGIWUR4462;AGIWUR18178
CB4856	CT	V	9798926 9799179 WBGene00044192	F46B6.13	F46B6.13	-0.305462755555036	AGIWUR313;AGIWUR12061;AGIWUR17444;AGIWUR18951;AGIWUR39217
CB4856	CT	V	9801463 9802028 WBGene00009775	F46B6.10	F46B6.10	0.137898133911419	AGIWUR5952;AGIWUR39386
CB4856	CT	V	9803476 9804607 WBGene00005702	sru-39	F46B6.11	-0.266045200182964	AGIWUR39929
CB4856	CT	V	9805461 9806600 WBGene00005700	sru-37	C50C10.1	-0.0962446789978522

AGIWUR24732
CB4856 CT V 9807635 9809432 WBGene00005696 sru-33 C50C10.8 -0.329718298820683
AGIWUR25941
CB4856 CT V 9811461 9812619 WBGene00005692 sru-29 C50C10.2 -0.000159881691701003
AGIWUR7544
CB4856 CT V 9813838 9815007 WBGene00005693 sru-30 C50C10.3 -0.00381439781173132
AGIWUR36392
CB4856 CT V 9816690 9817862 WBGene00005694 sru-31 C50C10.4 -0.60375765181913
AGIWUR12905
CB4856 CT V 9819279 9820210 WBGene00005695 sru-32 C50C10.5 0.057874439271604
AGIWUR7644;AGIWUR12393;AGIWUR12687;AGIWUR13938;AGIWUR24740;AGIWUR24921;AGIWUR28299;AGIWUR38405;AGIWUR44059
CB4856 CT V 9820554 9821824 WBGene00006232 str-193 C50C10.6 0.00183052685940766
AGIWUR6862
CB4856 CT V 9822619 9824249 WBGene00006070 str-2 C50C10.7 0.0536872060671758
AGIWUR28381
CB4856 CT V 9827156 9828401 WBGene00005697 sru-34 C50C10.9 0.00475518566027722
AGIWUR1612;AGIWUR26289
CB4856 CT V 9829068 9830444 WBGene00014817 R07B5.1 R07B5.1 -0.0911928562475599
AGIWUR5180;AGIWUR9324
CB4856 CT V 9830978 9831908 WBGene00014818 R07B5.2 R07B5.2 0.131394962785221
AGIWUR5210;AGIWUR38357
CB4856 CT V 9832742 9834000 WBGene00005703 sru-40 R07B5.3 0.56692859194913 AGIWUR10727
CB4856 CT V 9834463 9836088 WBGene00005701 sru-38 R07B5.4 0.0536250032082863 AGIWUR15518
CB4856 CT V 9837979 9839588 WBGene00011084 srsx-21 R07B5.5 0.116895910583962 AGIWUR25575
CB4856 CT V 9841676 9843179 WBGene00005699 sru-36 R07B5.6 -0.128812802225264 AGIWUR8930
CB4856 CT V 9843866 9846161 WBGene00005671 sru-8 R07B5.7 0.0383888754440225 AGIWUR21957
CB4856 CT V 9846158 9859963 WBGene00045419 lsy-12 R07B5.9 -0.108733372077812
AGIWUR7788;AGIWUR14451;AGIWUR18405;AGIWUR18991;AGIWUR21009;AGIWUR22924;AGIWUR27328;AGIWUR30577
CB4856 CT V 9866628 9868411 WBGene00011701 srab-17 T11A5.2 0.0349673659885967
AGIWUR26966;AGIWUR29976;AGIWUR37974
CB4856 CT V 9869887 9871439 WBGene00011702 srab-18 T11A5.3 0.167047644540826 AGIWUR44934
CB4856 CT V 9874009 9875899 WBGene00011703 srab-19 T11A5.4 0.0399041827611804 AGIWUR44659
CB4856 CT V 9876687 9879504 WBGene00043059 srab-26 T11A5.7 0.114422064965962
AGIWUR12889;AGIWUR32842
CB4856 CT V 9880114 9881181 WBGene00011704 sdz-31 T11A5.5 -0.122368961623343 AGIWUR30501
CB4856 CT V 9882034 9885960 WBGene00011705 T11A5.6 T11A5.6 -0.199961056620527
AGIWUR4479;AGIWUR12299;AGIWUR17100;AGIWUR20013;AGIWUR22364
CB4856 CT V 9889839 9894849 WBGene00008239 fshr-1 C50H2.1 0.18126640429967
AGIWUR15231;AGIWUR21400;AGIWUR23775

CB4856	CT	V	9899587	9907644	WBGene00001211	egl-47	C50H2.2	0.0835266847466078
AGIWUR1630;AGIWUR25213
CB4856	CT	V	9907632	9915103	WBGene00003173	mec-9	C50H2.3	-0.23778863379377
AGIWUR8109;AGIWUR20278
CB4856	CT	V	9916975	9919167	WBGene00008240	C50H2.4	C50H2.4	0.205022733672422
AGIWUR6240;AGIWUR13906;AGIWUR34958
CB4856	CT	V	9918770	9919790	WBGene00008241	C50H2.5	C50H2.5	0.0852116342695357	AGIWUR4106
CB4856	CT	V	9921337	9922194	WBGene00008242	ceh-75	C50H2.6	-0.0335446286161538	AGIWUR44098
CB4856	CT	V	9923285	9923936	WBGene00008243	C50H2.7	C50H2.7	0.0698000258550948	AGIWUR26387
CB4856	CT	V	9926544	9927884	WBGene00008246	C50H2.13	C50H2.13	0.176431748914233
AGIWUR37920
CB4856	CT	V	9927782	9930775	WBGene00011576	rmh-2	T07C12.12	-0.246659644146348
AGIWUR26741
CB4856	CT	V	9931019	9932368	WBGene00044204	suds-3	T07C12.14	-0.129479801559462
AGIWUR11628;AGIWUR14798;AGIWUR38926
CB4856	CT	V	9935548	9937535	WBGene00005618	srj-33	T07C12.1	0.134759604868243
AGIWUR9624
CB4856	CT	V	9937804	9940885	WBGene00014833	T07C12.4	T07C12.4	0.0182813960970236
AGIWUR12419;AGIWUR17109;AGIWUR34432
CB4856	CT	V	9941691	9942999	WBGene00005619	srj-34	T07C12.5	0.0373677572926923
AGIWUR30726;AGIWUR40882
CB4856	CT	V	9944393	9945895	WBGene00011570	sre-28	T07C12.6	0.11606269778683
AGIWUR27875
CB4856	CT	V	9951233	9952182	WBGene00011571	ttr-46	T07C12.7	-0.379447105755928
AGIWUR3342;AGIWUR14268
CB4856	CT	V	9952699	9956883	WBGene00011572	mam-5	T07C12.8	0.438674036994469
AGIWUR5217
CB4856	CT	V	9956879	9958331	WBGene00011573	anmt-3	T07C12.9	-0.0663523375342816
AGIWUR20955;AGIWUR23694;AGIWUR38361
CB4856	CT	V	9959997	9960337	WBGene00011574	T07C12.10	T07C12.10	0.14913760277463
AGIWUR36362
CB4856	CT	V	9966404	9976226	WBGene00006777	unc-41	C27H6.1	0.111527862307835
AGIWUR15466;AGIWUR44126
CB4856	CT	V	9978791	9980802	WBGene00007784	ruvb-1	C27H6.2	-0.166310041874701
AGIWUR21285;AGIWUR38782
CB4856	CT	V	9981249	9982766	WBGene00007785	tofu-1	C27H6.3	-0.0750498172986109
AGIWUR8036;AGIWUR34372
CB4856	CT	V	9982868	9983932	WBGene00077500	C27H6.9	C27H6.9	0.0401430584256968
AGIWUR8798;AGIWUR30656;AGIWUR37688

CB4856	CT	V	9983924	9986277	WBGene00007786	rmd-2	C27H6.4	-0.0622743325920148	AGIWUR2085
CB4856	CT	V	9986330	9987788	WBGene00007787	C27H6.8	C27H6.8	-0.0217002617313917	AGIWUR23276;AGIWUR30527;AGIWUR31051;AGIWUR31999;AGIWUR35154;AGIWUR37913
CB4856	CT	V	9987916	9991642	WBGene00004323	rde-1	K08H10.7	-0.0106417775142376	AGIWUR8121
CB4856	CT	V	9991812	9992792	WBGene00010699	trpp-6	K08H10.9	-0.166621968187858	AGIWUR41394
CB4856	CT	V	9993732	9995883	WBGene00010698	K08H10.6	K08H10.6	-0.533965009101015	AGIWUR583
CB4856	CT	V	9997094	9998481	WBGene00014805	K08H10.5	K08H10.5	-0.152589021406795	AGIWUR9000
CB4856	CT	V	10001118	10002766	WBGene00077491	K08H10.10	K08H10.10	0.00820772722958463	AGIWUR13436;AGIWUR30142;AGIWUR34222
CB4856	CT	V	10004755	10007482	WBGene00010697	uda-1	K08H10.4	-0.0429378645179597	AGIWUR13430;AGIWUR19067;AGIWUR40611
CB4856	CT	V	10011871	10028158	WBGene00002263	lea-1	K08H10.1	0.218956059076486	AGIWUR11182;AGIWUR11697;AGIWUR17882;AGIWUR38029;AGIWUR39700
CB4856	CT	V	10028150	10031094	WBGene00011543	acl-2	T06E8.1	-0.0480675935116661	AGIWUR9552;AGIWUR14141;AGIWUR39235
CB4856	CT	V	10035746	10036413	WBGene00011544	T06E8.2	T06E8.2	0.233687599590533	AGIWUR36746
CB4856	CT	V	10037340	10048726	WBGene00010177	F57A8.1	F57A8.1	0.298706775459755	AGIWUR9377;AGIWUR19620;AGIWUR26218;AGIWUR41504
CB4856	CT	V	10050047	10054913	WBGene00010178	yif-1	F57A8.2	-0.0955260740482506	AGIWUR4863;AGIWUR17667
CB4856	CT	V	10064503	10064939	WBGene00010183	fipr-13	F57A8.8	-0.107238623174506	AGIWUR19773
CB4856	CT	V	10067087	10068818	WBGene00010179	F57A8.4	F57A8.4	0.188486945164595	AGIWUR12584
CB4856	CT	V	10069369	10070641	WBGene00010180	nhr-192	F57A8.5	-0.740223137519438	AGIWUR13885
CB4856	CT	V	10071945	10073893	WBGene00010181	F57A8.6	F57A8.6	0.0422665432004266	AGIWUR33295;AGIWUR38993;AGIWUR44089
CB4856	CT	V	10075749	10077804	WBGene00010182	F57A8.7	F57A8.7	0.02911176745204	AGIWUR23052
CB4856	CT	V	10077905	10078344	WBGene00011001	R04B5.1	R04B5.1	-0.201613580216727	AGIWUR39091
CB4856	CT	V	10080119	10081739	WBGene00011002	nhr-205	R04B5.3	-0.0583284119267127	AGIWUR10466

CB4856	CT	V	10082141	10084043	WBGene00003611	nhr-12	R04B5.4	-0.236425076930384	AGIWUR27082
CB4856	CT	V	10085281	10087313	WBGene00011004	R04B5.6	R04B5.6	-0.198233133416952	AGIWUR21782
CB4856	CT	V	10087480	10088998	WBGene00011003	R04B5.5	R04B5.5	0.832089524776574	AGIWUR21261
CB4856	CT	V	10089157	10090561	WBGene00011005	R04B5.7	R04B5.7	0.115307849338565	AGIWUR30661
CB4856	CT	V	10090688	10091252	WBGene00011008	R04B5.11	R04B5.11	-0.202766791322686	AGIWUR5081;AGIWUR6922;AGIWUR16433;AGIWUR19085;AGIWUR42467
CB4856	CT	V	10094720	10096311	WBGene00005127	srd-49	R04B5.8	0.185160387202679	AGIWUR15531
CB4856	CT	V	10096305	10098597	WBGene00011006	ugt-47	R04B5.9	-0.295475054563015	AGIWUR42833
CB4856	CT	V	10098904	10099205	WBGene00011007	R04B5.10	R04B5.10	0.178984148411205	AGIWUR5592
CB4856	CT	V	10100156	10102016	WBGene00044044	C08B6.14	C08B6.14	-0.0694014672895662	AGIWUR23984
CB4856	CT	V	10102592	10104946	WBGene00007422	ugt-17	C08B6.1	-0.43749853158986	AGIWUR16806
CB4856	CT	V	10107684	10109905	WBGene00007431	C08B6.11	C08B6.11	-0.474735351907963	AGIWUR13572
CB4856	CT	V	10109872	10112287	WBGene00007424	C08B6.3	C08B6.3	-0.392516330764963	AGIWUR37592
CB4856	CT	V	10117765	10119688	WBGene00007426	C08B6.5	C08B6.5	-0.157065276430482	AGIWUR14625
CB4856	CT	V	10119574	10120986	WBGene00007432	srxa-18	C08B6.12	0.156409796859506	AGIWUR36957
CB4856	CT	V	10121134	10122770	WBGene00044012	srxa-19	C08B6.13	0.162152093067126	AGIWUR27566;AGIWUR44831
CB4856	CT	V	10123638	10124689	WBGene00007427	C08B6.6	C08B6.6	0.110181676972561	AGIWUR8597
CB4856	CT	V	10125052	10127570	WBGene00007430	C08B6.10	C08B6.10	-0.0665674520607524	AGIWUR6529
CB4856	CT	V	10127492	10130295	WBGene00000142	aos-1	C08B6.9	-0.179344518673443	AGIWUR24404;AGIWUR32403;AGIWUR35539;AGIWUR38336
CB4856	CT	V	10130405	10134148	WBGene00007428	wdr-20	C08B6.7	-0.0132472332534075	AGIWUR4283;AGIWUR10640;AGIWUR12917;AGIWUR19139;AGIWUR20882;AGIWUR24602
CB4856	CT	V	10134643	10143081	WBGene00007429	C08B6.8	C08B6.8	-0.168942480536648	

AGIWUR20990;AGIWUR33580
CB4856 CT V 10143936 10144192 WBGene00077449 C08B6.15 C08B6.15
-0.0674289864514304 AGIWUR717;AGIWUR3577;AGIWUR20236;AGIWUR38212;AGIWUR43073
CB4856 CT V 10144408 10145251 WBGene00008253 C51E3.8 C51E3.8 0.403039721477867
AGIWUR30989
CB4856 CT V 10145905 10148977 WBGene00008254 C51E3.9 C51E3.9 -0.154312300058056
AGIWUR22043
CB4856 CT V 10150066 10152159 WBGene00008248 srsx-27 C51E3.2 0.378300237364563
AGIWUR23884;AGIWUR25522
CB4856 CT V 10152583 10154756 WBGene00008249 srsx-28 C51E3.3 0.0461176151581082
AGIWUR10539;AGIWUR19267;AGIWUR25349
CB4856 CT V 10154998 10156381 WBGene00008250 srsx-29 C51E3.4 -0.200730998965268
AGIWUR5433
CB4856 CT V 10158833 10160297 WBGene00008251 srsx-30 C51E3.5 -0.141226584067155
AGIWUR13139;AGIWUR18898
CB4856 CT V 10160439 10161161 WBGene00008255 C51E3.10 C51E3.10
0.188405672484649 AGIWUR5502
CB4856 CT V 10164135 10166463 WBGene00008252 C51E3.6 C51E3.6 -0.424320961203421
AGIWUR15393;AGIWUR33155;AGIWUR38537
CB4856 CT V 10169197 10173753 WBGene00001172 egl-3 C51E3.7 0.534255414903998
AGIWUR1410;AGIWUR10673;AGIWUR10823;AGIWUR32937
CB4856 CT V 10179535 10183154 WBGene00000840 cul-5 ZK856.1 0.346323566067175
AGIWUR10749
CB4856 CT V 10183284 10184446 WBGene00014105 ZK856.4 ZK856.4 -0.194331840643152
AGIWUR1992
CB4856 CT V 10184689 10186007 WBGene00194912 ZK856.18 ZK856.18
-0.0435450810908549 AGIWUR11214
CB4856 CT V 10198704 10199942 WBGene00014107 ZK856.6 ZK856.6 -0.0320100214785874
AGIWUR24559
CB4856 CT V 10201856 10202610 WBGene00014108 ZK856.7 ZK856.7 -0.265622283756303
AGIWUR4691;AGIWUR21535;AGIWUR29202;AGIWUR31126
CB4856 CT V 10202538 10203485 WBGene00014109 chpf-1 ZK856.8 0.126795204778063
AGIWUR8017;AGIWUR29777;AGIWUR42156;AGIWUR44854
CB4856 CT V 10203772 10205587 WBGene00014112 ZK856.11 ZK856.11
-0.0720413276888134 AGIWUR11220
CB4856 CT V 10205683 10206602 WBGene00014111 rpc-25 ZK856.10
0.0826581612016568 AGIWUR35370
CB4856 CT V 10206619 10210344 WBGene00014114 tftc-3 ZK856.13
-0.179953094587706 AGIWUR11722

CB4856	CT	V	10210444	10212127	WBGene00006489	zhit-3	ZK856.9	-0.105960829758876	AGIWUR41962
CB4856	CT	V	10212929	10216305	WBGene00014113	hpo-40	ZK856.12	-0.253471653606715	AGIWUR20340;AGIWUR26577;AGIWUR37582
CB4856	CT	V	10217079	10219022	WBGene00044020	ZK856.14	ZK856.14	0.0283968825862377	AGIWUR1092;AGIWUR14205;AGIWUR31390
CB4856	CT	V	10219455	10220470	WBGene00007540	C12D8.13	C12D8.13	-0.129806064551815	AGIWUR15994
CB4856	CT	V	10223513	10225760	WBGene00007536	daf-36	C12D8.5	-0.799891529795831	AGIWUR24683
CB4856	CT	V	10226885	10227434	WBGene00007535	ttr-19	C12D8.4	0.33130113923175	AGIWUR25644
CB4856	CT	V	10228043	10228460	WBGene00007541	fipr-4	C12D8.14	-0.152738587412191	AGIWUR16878
CB4856	CT	V	10228641	10229322	WBGene00007542	C12D8.15	C12D8.15	-0.0993685602091237	AGIWUR24231
CB4856	CT	V	10231821	10233430	WBGene00006226	str-182	C12D8.12	0.0039953483197257	AGIWUR41878
CB4856	CT	V	10236160	10238902	WBGene00007534	C12D8.1	C12D8.1	-0.144217361367398	AGIWUR1056;AGIWUR7083;AGIWUR9511;AGIWUR14024;AGIWUR18462;AGIWUR22716;AGIWUR25429;AGIWUR27421;AGIWUR35675;AGIWUR39350
CB4856	CT	V	10240573	10241188	WBGene00195157	C12D8.21	C12D8.21	-0.321633270093789	AGIWUR15826
CB4856	CT	V	10241784	10242718	WBGene00000721	col-148	C12D8.8	-0.11914437560424	AGIWUR13025
CB4856	CT	V	10242787	10243205	WBGene00007538	C12D8.9	C12D8.9	-0.20926919741782	AGIWUR8119
CB4856	CT	V	10248907	10252895	WBGene00000102	akt-1	C12D8.10	-0.0449751473516775	AGIWUR3065;AGIWUR5113;AGIWUR14046;AGIWUR43925
CB4856	CT	V	10252821	10255055	WBGene00004405	rop-1	C12D8.11	-0.111574163254499	AGIWUR2067;AGIWUR16535;AGIWUR21656
CB4856	CT	V	10256360	10258654	WBGene00012494	nhr-232	Y22F5A.1	0.0717290157447952	AGIWUR2700
CB4856	CT	V	10258661	10260554	WBGene00005530	sri-18	Y22F5A.2	0.0503511488366462	AGIWUR12538
CB4856	CT	V	10261131	10270314	WBGene00004364	ric-4	Y22F5A.3	0.000340727715031797	AGIWUR13824;AGIWUR14867;AGIWUR17495;AGIWUR21980
CB4856	CT	V	10277827	10279127	WBGene00003090	lys-1	Y22F5A.4	-0.468959001755572	AGIWUR13848;AGIWUR17373;AGIWUR20455;AGIWUR29828;AGIWUR30263;AGIWUR33540

CB4856	CT	V	10280310	10281474	WBGene00003091	lys-2	Y22F5A.5
-0.723576837887951	AGIWUR37008
CB4856	CT	V	10282204	10283544	WBGene00003092	lys-3	Y22F5A.6
1.59258933200653	AGIWUR44748
CB4856	CT	V	10287564	10289272	WBGene00007096	B0024.3	B0024.3	-0.136900924299784
AGIWUR8921
CB4856	CT	V	10289721	10291710	WBGene00007104	B0024.15	B0024.15
-0.23156031800646	AGIWUR36356
CB4856	CT	V	10291697	10292869	WBGene00000722	col-149	B0024.1	-0.442025951467125
AGIWUR37769
CB4856	CT	V	10293563	10294623	WBGene00000723	col-150	B0024.2	-0.156533778184741
AGIWUR37941
CB4856	CT	V	10297399	10298865	WBGene00007097	B0024.4	B0024.4	0.316061953826995
AGIWUR44276
CB4856	CT	V	10299473	10307354	WBGene00000491	che-12	B0024.8	0.434678730143936
AGIWUR3900
CB4856	CT	V	10308291	10314053	WBGene00001533	gcy-6	B0024.6	-0.0228097710642423
AGIWUR18920
CB4856	CT	V	10314641	10315304	WBGene00007099	trx-2	B0024.9	0.0749813081961648
AGIWUR37553
CB4856	CT	V	10315245	10317394	WBGene00007100	B0024.10	B0024.10
-0.137463839234442	AGIWUR4937
CB4856	CT	V	10317490	10319534	WBGene00007101	B0024.11	B0024.11
-0.204732188216742	AGIWUR40598
CB4856	CT	V	10319535	10320158	WBGene00001646	gna-1	B0024.12
-0.0825632407894386	AGIWUR36410
CB4856	CT	V	10320715	10322304	WBGene00007102	B0024.13	B0024.13
0.0202704905926963	AGIWUR19097;AGIWUR26092;AGIWUR29028;AGIWUR32735
CB4856	CT	V	10322288	10332112	WBGene00007103	crm-1	B0024.14
0.24655000430143	AGIWUR4802;AGIWUR12222;AGIWUR14025;AGIWUR21491;AGIWUR22518;AGIWUR40898
CB4856	CT	V	10335443	10338355	WBGene00000200	arx-2	K07C5.1	0.0579390732312888
AGIWUR20870
CB4856	CT	V	10339500	10340971	WBGene00010625	K07C5.2	K07C5.2	0.0329080166269407
AGIWUR5598
CB4856	CT	V	10343032	10345489	WBGene00010626	K07C5.3	K07C5.3	-0.14644580355899
AGIWUR28656
CB4856	CT	V	10346662	10350154	WBGene00010632	K07C5.9	K07C5.9	0.0979596806850367
AGIWUR3318;AGIWUR7900;AGIWUR14780;AGIWUR17856;AGIWUR27080
CB4856	CT	V	10350471	10352978	WBGene00010627	K07C5.4	K07C5.4	-0.0374575290269625

AGIWUR11758;AGIWUR13751;AGIWUR31089;AGIWUR36058
CB4856 CT V 10353584 10355837 WBGene00010629 K07C5.6 K07C5.6 -0.148406638720729
AGIWUR35576
CB4856 CT V 10356285 10357903 WBGene00010628 ceeh-2 K07C5.5 0.0276886481774286
AGIWUR2367;AGIWUR11689;AGIWUR43744
CB4856 CT V 10358544 10361430 WBGene00010630 ttll-15 K07C5.7 -0.582348208739878
AGIWUR2920
CB4856 CT V 10362261 10366014 WBGene00010631 cash-1 K07C5.8 -0.173753313504539
AGIWUR7634;AGIWUR18426;AGIWUR20669
CB4856 CT V 10368556 10369868 WBGene00005532 sri-20 AC3.1 0.300284641664089
AGIWUR10661
CB4856 CT V 10372325 10374245 WBGene00007070 ugt-49 AC3.2 0.188643315152031
AGIWUR29691
CB4856 CT V 10374851 10376884 WBGene00044022 AC3.9 AC3.9 0.151187382620139
AGIWUR28600
CB4856 CT V 10376985 10377317 WBGene00077502 AC3.11 AC3.11 0.00505821220593106
AGIWUR6781;AGIWUR26582;AGIWUR29815;AGIWUR32777;AGIWUR40707
CB4856 CT V 10378982 10379364 WBGene00077503 AC3.12 AC3.12 -0.203012302694964
AGIWUR3223;AGIWUR15051;AGIWUR27034;AGIWUR43064;AGIWUR43093
CB4856 CT V 10384157 10385711 WBGene00004964 spe-10 AC3.10 -0.138122070016303
AGIWUR21504
CB4856 CT V 10388402 10393504 WBGene00007071 AC3.5 AC3.5 -0.511339560659948
AGIWUR3186;AGIWUR10575;AGIWUR24403;AGIWUR35180
CB4856 CT V 10393531 10395232 WBGene00000724 col-151 AC3.6 -0.0175316169061928
AGIWUR37545
CB4856 CT V 10395663 10397817 WBGene00007072 ugt-1 AC3.7 -0.427512909963486
AGIWUR6758
CB4856 CT V 10398717 10401307 WBGene00007073 ugt-2 AC3.8 -0.140199405175277
AGIWUR24429
CB4856 CT V 10401447 10402904 WBGene00008875 F15H10.8 F15H10.8
-0.234321400246891 AGIWUR3582;AGIWUR4498;AGIWUR5126;AGIWUR15892;AGIWUR20758;AGIWUR39123
CB4856 CT V 10403184 10406424 WBGene00008874 F15H10.7 F15H10.7
0.160010154651447 AGIWUR6174
CB4856 CT V 10410240 10412149 WBGene00008873 F15H10.6 F15H10.6
-0.0875988717711387 AGIWUR40937
CB4856 CT V 10413332 10414692 WBGene00044013 F15H10.9 F15H10.9
0.101424032980043 AGIWUR2037
CB4856 CT V 10415734 10417025 WBGene00044014 F15H10.10 F15H10.10
0.370347281919425 AGIWUR29823;AGIWUR44691

CB4856 CT V 10417617 10419053 WBGene00008872 F15H10.5 F15H10.5
-0.222438970614417 AGIWUR4994;AGIWUR14497
CB4856 CT V 10423907 10425092 WBGene00000601 col-12 F15H10.1
-0.621669121964826 AGIWUR4286
CB4856 CT V 10425362 10426399 WBGene00000144 apc-10 F15H10.3
0.012421670188373 AGIWUR26869;AGIWUR44725
CB4856 CT V 10426633 10428731 WBGene00008871 tag-314 F15H10.4
-0.08333145749687 AGIWUR40256
CB4856 CT V 10428726 10433604 WBGene00012529 Y32F6A.4 Y32F6A.4
-0.0105128302225177 AGIWUR11049;AGIWUR15338;AGIWUR22164;AGIWUR27644;AGIWUR28480;AGIWUR32635
CB4856 CT V 10436912 10441149 WBGene00012527 set-22 Y32F6A.1
-0.138937129543192 AGIWUR21850
CB4856 CT V 10441979 10446459 WBGene00012528 pap-1 Y32F6A.3
0.0668499574359958 AGIWUR12613;AGIWUR17884
CB4856 CT V 10446534 10453781 WBGene00012530 Y32F6A.5 Y32F6A.5
0.0393739685072827 AGIWUR31672
CB4856 CT V 10453748 10457347 WBGene00009058 F22E12.1 F22E12.1
-0.263092053558942 AGIWUR3370
CB4856 CT V 10459188 10463364 WBGene00009060 F22E12.3 F22E12.3
-0.244347278356473 AGIWUR16080
CB4856 CT V 10462282 10464660 WBGene00009059 chw-1 F22E12.2
-0.246606741106248 AGIWUR32911
CB4856 CT V 10468411 10476952 WBGene00001178 egl-9 F22E12.4
0.112911036198284
AGIWUR8432;AGIWUR12790;AGIWUR13943;AGIWUR16371;AGIWUR18187;AGIWUR21119;AGIWUR21574;AGIWUR31407;AGIWUR33133;
AGIWUR36086;AGIWUR36899
CB4856 CT V 10478566 10480963 WBGene00012532 crp-1 Y32F6B.3
-0.0895876451095692 AGIWUR5221
CB4856 CT V 10480949 10484740 WBGene00012531 Y32F6B.1 Y32F6B.1
-0.0864691242207165 AGIWUR41958
CB4856 CT V 10486473 10490048 WBGene00003570 ncx-5 Y32F6B.2
0.172460591594102 AGIWUR33541
CB4856 CT V 10491743 10493268 WBGene00000173 aqp-5 C35A5.1 0.151756834416688
AGIWUR18028
CB4856 CT V 10493406 10496477 WBGene00007946 ugt-33 C35A5.2 0.0980581249957186
AGIWUR25829
CB4856 CT V 10497059 10499324 WBGene00007948 C35A5.4 C35A5.4 -0.352111956210106
AGIWUR30625
CB4856 CT V 10499355 10501395 WBGene00007947 C35A5.3 C35A5.3 -0.589507470118175

AGIWUR12046;AGIWUR35018;AGIWUR44995
CB4856 CT V 10502116 10503111 WBGene00044024 C35A5.11 C35A5.11
1.38183386825585 AGIWUR730;AGIWUR7265;AGIWUR11026;AGIWUR30955
CB4856 CT V 10504193 10505401 WBGene00044016 C35A5.10 C35A5.10
0.137777518166384 AGIWUR22038;AGIWUR24479
CB4856 CT V 10505850 10507829 WBGene00007949 C35A5.5 C35A5.5 0.386470769850844
AGIWUR30308
CB4856 CT V 10509055 10510451 WBGene00007950 C35A5.6 C35A5.6 -0.0367244613987943
AGIWUR13574
CB4856 CT V 10510684 10514371 WBGene00007951 dmsr-8 C35A5.7 0.00217450304234656
AGIWUR14579;AGIWUR39136;AGIWUR44611
CB4856 CT V 10521992 10525908 WBGene00007952 C35A5.8 C35A5.8 -0.123283363200533
AGIWUR6377;AGIWUR17776;AGIWUR23357;AGIWUR34779;AGIWUR36167
CB4856 CT V 10526298 10528396 WBGene00007953 hda-11 C35A5.9 0.0592007025191137
AGIWUR13484;AGIWUR23917;AGIWUR33306
CB4856 CT V 10528754 10530568 WBGene00009108 F25D1.3 F25D1.3 -0.110915619808519
AGIWUR27819
CB4856 CT V 10530641 10532848 WBGene00009107 F25D1.2 F25D1.2 0.265406509761934
AGIWUR14084
CB4856 CT V 10532894 10536388 WBGene00006460 ppm-1 F25D1.1 0.0117008746583078
AGIWUR38079
CB4856 CT V 10538493 10538788 WBGene00077506 F25D1.7 F25D1.7 -0.0436338172823358
AGIWUR3338;AGIWUR15506;AGIWUR20953;AGIWUR28164;AGIWUR28456
CB4856 CT V 10540324 10545742 WBGene00009109 degt-1 F25D1.4 -0.126783048318226
AGIWUR27927
CB4856 CT V 10545741 10546799 WBGene00009110 F25D1.5 F25D1.5 0.21580863793015
AGIWUR36901
CB4856 CT V 10547598 10549081 WBGene00005112 srd-34 F32G8.1 -0.0787700510906992
AGIWUR11030
CB4856 CT V 10550017 10554493 WBGene00009338 F32G8.2 F32G8.2 -0.0632211811213423
AGIWUR24079;AGIWUR31469;AGIWUR33558
CB4856 CT V 10554616 10555238 WBGene00009339 F32G8.3 F32G8.3 0.0599036521733591
AGIWUR21055
CB4856 CT V 10557976 10560793 WBGene00009340 best-14 F32G8.4 -0.0963331118777955
AGIWUR10997;AGIWUR24165;AGIWUR30762;AGIWUR38864
CB4856 CT V 10560921 10562150 WBGene00000725 col-152 F32G8.5 -0.427244616285702
AGIWUR6215
CB4856 CT V 10564880 10566445 WBGene00000298 cat-4 F32G8.6 0.264513030721961
AGIWUR23788

CB4856	CT	V	10566365	10576419	WBGene00006934	vps-54	T21C9.2	0.170991298727754	AGIWUR6454;AGIWUR10348;AGIWUR12955;AGIWUR14416;AGIWUR17131;AGIWUR25888
CB4856	CT	V	10576513	10578322	WBGene00011890	mics-1	T21C9.1	0.198900299789399	AGIWUR12337
CB4856	CT	V	10578428	10579141	WBGene00011892	T21C9.4	T21C9.4	0.0299869555872156	AGIWUR13694
CB4856	CT	V	10579309	10580693	WBGene00003065	lpd-9	T21C9.5	-0.07962986450298	AGIWUR30951
CB4856	CT	V	10581887	10587164	WBGene00011893	T21C9.6	T21C9.6	0.439873994818917	AGIWUR12083
CB4856	CT	V	10587167	10588794	WBGene00005189	srg-32	T21C9.7	-0.192530808660958	AGIWUR6145
CB4856	CT	V	10590548	10592653	WBGene00011895	T21C9.9	T21C9.9	-0.495188695257266	AGIWUR34880
CB4856	CT	V	10593936	10594906	WBGene00011896	T21C9.11	T21C9.11	-0.212170540978491	AGIWUR32886
CB4856	CT	V	10597266	10598666	WBGene00011898	T21C9.13	T21C9.13	-0.121648275976954	AGIWUR14188
CB4856	CT	V	10599105	10602147	WBGene00011897	scpl-4	T21C9.12	0.249650757844794	AGIWUR10563
CB4856	CT	V	10602458	10605568	WBGene00010418	H27A22.1	H27A22.1	0.212939750379299	AGIWUR33284
CB4856	CT	V	10612926	10615511	WBGene00010375	H09F14.1	H09F14.1	-0.0384806247222969	AGIWUR24872
CB4856	CT	V	10633058	10635411	WBGene00003114	mab-23	C32C4.5	0.00583739939137473	AGIWUR21001;AGIWUR26989;AGIWUR29956;AGIWUR34519;AGIWUR44313;AGIWUR44808
CB4856	CT	V	10636022	10645135	WBGene00007862	C32C4.1	C32C4.1	0.0954296043933551	AGIWUR36603
CB4856	CT	V	10645020	10646963	WBGene00007863	C32C4.3	C32C4.3	0.0303780087436949	AGIWUR5767;AGIWUR27045
CB4856	CT	V	10648446	10651952	WBGene00000174	aqp-6	C32C4.2	-0.0761747103421833	AGIWUR9151
CB4856	CT	V	10651995	10654265	WBGene00044286	ugt-35	C32C4.7	0.0238719714902578	AGIWUR29194
CB4856	CT	V	10654417	10656976	WBGene00009255	ugt-34	F29F11.2	0.3615432363371	AGIWUR34667;AGIWUR40200
CB4856	CT	V	10658194	10659658	WBGene00009256	tut-2	F29F11.3	0.214840136484383	AGIWUR33697
CB4856	CT	V	10659635	10661467	WBGene00005022	sqv-4	F29F11.1		

-0.252575344319419	AGIWUR17812;AGIWUR20431;AGIWUR22370;AGIWUR37842;AGIWUR39068;AGIWUR41179
CB4856	CT	V	10667586	10670860	WBGene00006667	twk-12	F29F11.4
-0.363120132305191	AGIWUR24447
CB4856	CT	V	10672459	10675644	WBGene00000445	ceh-22	F29F11.5
0.492290375788652	AGIWUR12965;AGIWUR20993;AGIWUR30917
CB4856	CT	V	10682422	10686537	WBGene00001747	gsp-1	F29F11.6
-0.153225012532815	AGIWUR17355
CB4856	CT	V	10702549	10704906	WBGene00004083	pph-1	C05A2.1	0.149589470551357
AGIWUR9032;AGIWUR15633;AGIWUR16147;AGIWUR22418;AGIWUR24808
CB4856	CT	V	10712165	10732507	WBGene00012117	T28B11.1	T28B11.1
0.136709793464206	AGIWUR585;AGIWUR14500;AGIWUR41017
CB4856	CT	V	10733931	10736892	WBGene00003405	mre-11	ZC302.1	0.109536732165285
AGIWUR33458
CB4856	CT	V	10737116	10739350	WBGene00013862	wdr-5.3	ZC302.2	0.0510302376447491
AGIWUR14638
CB4856	CT	V	10742466	10743749	WBGene00005610	srj-22	F28H7.1	0.125441839691078
AGIWUR40243
CB4856	CT	V	10744642	10746469	WBGene00005609	srj-21	F28H7.11
-0.551824632011584	AGIWUR12071
CB4856	CT	V	10746548	10747337	WBGene00009238	selt-1.2	F28H7.4
-0.313543477490797	AGIWUR38788
CB4856	CT	V	10747944	10748938	WBGene00009236	F28H7.2	F28H7.2	0.304645811106679
AGIWUR3011
CB4856	CT	V	10749607	10750788	WBGene00009237	F28H7.3	F28H7.3	-0.249520054288805
AGIWUR35986
CB4856	CT	V	10751057	10754147	WBGene00009240	F28H7.7	F28H7.7	-0.34099108634769
AGIWUR2905
CB4856	CT	V	10754469	10756664	WBGene00009239	irld-6	F28H7.6	-0.665676041420156
AGIWUR1059
CB4856	CT	V	10758958	10760663	WBGene00009241	F28H7.8	F28H7.8	-1.11963734572508
AGIWUR9083
CB4856	CT	V	10760658	10762790	WBGene00009242	sre-6	F28H7.9	0.146926091588796
AGIWUR41255;AGIWUR42176
CB4856	CT	V	10764366	10768454	WBGene00003838	ocr-1	F28H7.10
0.00791444216422388	AGIWUR35218
CB4856	CT	V	10768465	10770217	WBGene00008370	D1054.1	D1054.1	0.0267112531698466
AGIWUR44989
CB4856	CT	V	10770321	10771351	WBGene00003923	pas-2	D1054.2	-0.187657283665693
AGIWUR20271;AGIWUR21995;AGIWUR34386;AGIWUR40199

CB4856	CT	V	10771451	10772832	WBGene00008371	D1054.3	D1054.3	-0.185639463500428
AGIWUR3336;AGIWUR4673;AGIWUR15639;AGIWUR16549
CB4856	CT	V	10772819	10776578	WBGene00008372	D1054.5	D1054.5	0.0334373465422715
AGIWUR41456
CB4856	CT	V	10778475	10780019	WBGene00008373	D1054.6	D1054.6	-0.0575897795617841
AGIWUR39343
CB4856	CT	V	10781459	10783380	WBGene00008374	clec-220	D1054.7
-0.290242841697351	AGIWUR32354;AGIWUR38925
CB4856	CT	V	10784236	10785367	WBGene00008375	D1054.8	D1054.8	-0.266880079195163
AGIWUR20991
CB4856	CT	V	10785801	10791057	WBGene00008376	D1054.9	D1054.9	0.117017229554992
AGIWUR3559;AGIWUR5305;AGIWUR10996;AGIWUR13468;AGIWUR13746;AGIWUR19183;AGIWUR28311;AGIWUR34506
CB4856	CT	V	10791975	10792830	WBGene00008377	D1054.10	D1054.10
0.0628959173327956	AGIWUR31296
CB4856	CT	V	10793793	10794924	WBGene00008378	ule-3	D1054.11
-0.0578961483617775	AGIWUR7075
CB4856	CT	V	10796863	10798157	WBGene00005399	srh-184	D1054.12
0.107440371734575	AGIWUR26639
CB4856	CT	V	10798360	10799475	WBGene00005379	srh-163	D1054.16
-0.00767492813662951	AGIWUR15060;AGIWUR25635
CB4856	CT	V	10802336	10804474	WBGene00008379	secs-1	D1054.13
0.0783633617008199	AGIWUR31281
CB4856	CT	V	10804721	10806034	WBGene00008380	prp-38	D1054.14
-0.00619107992427104	AGIWUR30839;AGIWUR36917
CB4856	CT	V	10806131	10807973	WBGene00006481	plrg-1	D1054.15
-0.056830467659827	AGIWUR34148
CB4856	CT	V	10812343	10813487	WBGene00007797	C29A12.1	C29A12.1
-0.168095366139376	AGIWUR4242
CB4856	CT	V	10814113	10816718	WBGene00002985	lig-1	C29A12.3
-0.239995765319249	AGIWUR1609;AGIWUR22411
CB4856	CT	V	10825012	10846695	WBGene00007799	nrx-1	C29A12.4
-0.0066930219608281
AGIWUR9816;AGIWUR10025;AGIWUR14320;AGIWUR17861;AGIWUR18536;AGIWUR19055;AGIWUR36206;AGIWUR36484;AGIWUR37045;
AGIWUR38337;AGIWUR38411;AGIWUR41061;AGIWUR43338
CB4856	CT	V	10847193	10852502	WBGene00007800	C29A12.6	C29A12.6
0.981795582712261	AGIWUR7641
CB4856	CT	V	10852519	10865792	WBGene00010012	saeg-1	F53H10.2
0.0528527676464456	AGIWUR15318;AGIWUR22336;AGIWUR24998;AGIWUR35859;AGIWUR37415
CB4856	CT	V	10880837	10883399	WBGene00009326	F32D8.1	F32D8.1	-0.0167401989615676

AGIWUR7811
CB4856 CT V 10883778 10886102 WBGene00009327 F32D8.2 F32D8.2 0.166055864225187
AGIWUR40386
CB4856 CT V 10886837 10887965 WBGene00009328 F32D8.3 F32D8.3 -0.0627649184746097
AGIWUR42274
CB4856 CT V 10887873 10889004 WBGene00043302 F32D8.11 F32D8.11
-0.15061815902533 AGIWUR2373;AGIWUR10990;AGIWUR29778;AGIWUR42702;AGIWUR45151
CB4856 CT V 10888572 10891030 WBGene00009334 F32D8.12 F32D8.12
0.170376289260834 AGIWUR31365;AGIWUR36246
CB4856 CT V 10891338 10893560 WBGene00009329 F32D8.4 F32D8.4 -0.363439746465691
AGIWUR39233
CB4856 CT V 10893649 10894788 WBGene00009336 F32D8.14 F32D8.14
-0.180636822709733 AGIWUR4301;AGIWUR30461
CB4856 CT V 10896658 10897143 WBGene00001303 emo-1 F32D8.6 -0.289142013088114
AGIWUR34849
CB4856 CT V 10898435 10900701 WBGene00009331 F32D8.7 F32D8.7 -0.0812151312906053
AGIWUR27643
CB4856 CT V 10900563 10901303 WBGene00005001 spp-16 F32D8.9 -0.194231159684751
AGIWUR36125
CB4856 CT V 10901480 10902782 WBGene00009332 F32D8.8 F32D8.8 -0.193461704428151
AGIWUR44171
CB4856 CT V 10904059 10906774 WBGene00009325 F32D8.15 F32D8.15
0.0331657120102413 AGIWUR5514
CB4856 CT V 10906755 10909171 WBGene00009333 F32D8.10 F32D8.10
-0.209916175449995 AGIWUR1368
CB4856 CT V 10917648 10918401 WBGene00010235 F58B4.2 F58B4.2 -0.170626990585837
AGIWUR12490
CB4856 CT V 10918861 10921847 WBGene00003549 nas-31 F58B4.1
-0.00973472320519653 AGIWUR9906;AGIWUR11402;AGIWUR21749;AGIWUR23262;AGIWUR23820;AGIWUR40710
CB4856 CT V 10925322 10926796 WBGene00010239 F58B4.6 F58B4.6 0.332435295136749
AGIWUR8914
CB4856 CT V 10929811 10930774 WBGene00010236 F58B4.3 F58B4.3 0.0611033822943128
AGIWUR359
CB4856 CT V 10930778 10931289 WBGene00010237 scvp-1 F58B4.4 0.096642844773223
AGIWUR33317
CB4856 CT V 10932647 10934245 WBGene00010238 F58B4.5 F58B4.5 -0.197990170931154
AGIWUR16464
CB4856 CT V 10934228 10936920 WBGene00008923 F17C11.12 F17C11.12
-0.632852471092328 AGIWUR4805

CB4856	CT	V	10937633	10938849	WBGene00008922	F17C11.11	F17C11.11
-0.721489114583999	AGIWUR8176
CB4856	CT	V	10939245	10939612	WBGene00050890	F17C11.16	F17C11.16
-0.0133490586417357	AGIWUR4887;AGIWUR9224;AGIWUR13452;AGIWUR13645;AGIWUR41559
CB4856	CT	V	10941450	10944302	WBGene00077761	zip-9	F17C11.17
-0.334095495219181	AGIWUR2242;AGIWUR6734;AGIWUR25942;AGIWUR34136;AGIWUR38340;AGIWUR38704;AGIWUR39056
CB4856	CT	V	10948212	10949129	WBGene00008914	F17C11.2	F17C11.2
0.443789433493874	AGIWUR20468
CB4856	CT	V	10949857	10951821	WBGene00000726	col-153	F17C11.3
0.0297619904419619	AGIWUR22834
CB4856	CT	V	10951818	10953472	WBGene00008915	F17C11.4	F17C11.4
1.25515765199824	AGIWUR37270
CB4856	CT	V	10953501	10954434	WBGene00008916	clec-221	F17C11.5
-0.200924512896488	AGIWUR11860
CB4856	CT	V	10955091	10955952	WBGene00008917	F17C11.6	F17C11.6
-0.412560821959977	AGIWUR7977;AGIWUR14608;AGIWUR23095
CB4856	CT	V	10955923	10957020	WBGene00023503	F17C11.13	F17C11.13
-0.305512364830463	AGIWUR13427
CB4856	CT	V	10956910	10958821	WBGene00008918	F17C11.7	F17C11.7
0.000657440417402455	AGIWUR13347;AGIWUR36662
CB4856	CT	V	10958928	10960919	WBGene00008919	vps-36	F17C11.8
0.0534590187199884	AGIWUR5553;AGIWUR23059;AGIWUR28557;AGIWUR40486
CB4856	CT	V	10961722	10965562	WBGene00008921	F17C11.10	F17C11.10
-0.32694902242063	AGIWUR6666;AGIWUR29011;AGIWUR36798
CB4856	CT	V	10965675	10968070	WBGene00008920	eef-1G	F17C11.9
0.0116035246404493	AGIWUR5607;AGIWUR10732;AGIWUR16495;AGIWUR20982;AGIWUR42601
CB4856	CT	V	10970399	10981213	WBGene00009968	fig-1	F53B7.5	0.130863544421429
AGIWUR14199
CB4856	CT	V	10992099	10994817	WBGene00009965	frpr-9	F53B7.2
-0.00542845201884123	AGIWUR38039
CB4856	CT	V	10999102	10999642	WBGene00009969	F53B7.7	F53B7.7	-0.0475428649847203
AGIWUR29607
CB4856	CT	V	10999658	11000713	WBGene00009966	F53B7.3	F53B7.3	-0.357486655891282
AGIWUR31116
CB4856	CT	V	11000957	11002534	WBGene00009967	F53B7.4	F53B7.4	0.0520785689635983
AGIWUR15378;AGIWUR32446
CB4856	CT	V	11011589	11012465	WBGene00007553	C13G3.1	C13G3.1	-0.135335549984074
AGIWUR5612
CB4856	CT	V	11021069	11022884	WBGene00001991	hot-6	C13G3.2	0.507969173142307

AGIWUR35399
CB4856 CT V 11027153 11030845 WBGene00007554 pptr-2 C13G3.3 0.0437431137637905
AGIWUR6753;AGIWUR10456;AGIWUR15563;AGIWUR23456
CB4856 CT V 11036462 11039425 WBGene00006443 pak-2 C45B11.1
0.0482028463925044 AGIWUR4837;AGIWUR12671
CB4856 CT V 11041824 11042301 WBGene00008106 C45B11.5 C45B11.5
-0.227386743410475 AGIWUR45002
CB4856 CT V 11042817 11043855 WBGene00008105 C45B11.2 C45B11.2
-0.0172536127703373 AGIWUR2224;AGIWUR5947
CB4856 CT V 11044067 11045734 WBGene00045379 C45B11.6 C45B11.6
0.233815640282246 AGIWUR9924;AGIWUR28606;AGIWUR30898;AGIWUR32180;AGIWUR37183;AGIWUR43770
CB4856 CT V 11045696 11046899 WBGene00000981 dhs-18 C45B11.3
0.63804380497157 AGIWUR38705
CB4856 CT V 11047227 11048744 WBGene00005295 srh-74 C45B11.4
-8.12920783803683e-05 AGIWUR14311;AGIWUR26356
CB4856 CT V 11052457 11053107 WBGene00271444 T04C12.33 T04C12.33
-0.792251244956065 AGIWUR36779
CB4856 CT V 11054127 11055142 WBGene00011429 T04C12.7 T04C12.7
0.30135586497528 AGIWUR19762
CB4856 CT V 11055306 11057143 WBGene00005296 srh-75 T04C12.2
0.174615231392245 AGIWUR13499
CB4856 CT V 11058649 11059264 WBGene00011427 T04C12.1 T04C12.1
1.41299850986512 AGIWUR14776;AGIWUR18085;AGIWUR19163;AGIWUR26042;AGIWUR26550;AGIWUR38351
CB4856 CT V 11062439 11063245 WBGene00050889 T04C12.9 T04C12.9
0.0562175080153704 AGIWUR2246;AGIWUR4116;AGIWUR21052;AGIWUR38044;AGIWUR42482
CB4856 CT V 11064954 11065634 WBGene00011428 T04C12.3 T04C12.3
0.277514849868096 AGIWUR5731;AGIWUR6696;AGIWUR6992;AGIWUR24917;AGIWUR28081;AGIWUR40873
CB4856 CT V 11068069 11069358 WBGene00011430 T04C12.8 T04C12.8
0.539811389389795 AGIWUR42678
CB4856 CT V 11071690 11074498 WBGene00000065 act-3 T04C12.4
0.056946934484376 AGIWUR13229;AGIWUR23078;AGIWUR29810;AGIWUR42315
CB4856 CT V 11076723 11078168 WBGene00000064 act-2 T04C12.5
-0.198112784422669 AGIWUR9001
CB4856 CT V 11079167 11082497 WBGene00000063 act-1 T04C12.6
0.270145436789658 AGIWUR9269
CB4856 CT V 11088154 11097970 WBGene00012776 Y42A5A.1 Y42A5A.1
-0.122715365612326 AGIWUR16408
CB4856 CT V 11098950 11107288 WBGene00012779 cdkl-1 Y42A5A.4
0.1580637633186 AGIWUR22818

CB4856 CT V 11107535 11109747 WBGene00012780 Y42A5A.5 Y42A5A.5
0.125717598158772 AGIWUR31100
CB4856 CT V 11110063 11113103 WBGene00010405 erfa-3 H19N07.1
-0.219469159272982 AGIWUR16557
CB4856 CT V 11113914 11118818 WBGene00010406 math-33 H19N07.2
0.183428375475999 AGIWUR9079;AGIWUR13855
CB4856 CT V 11118721 11119730 WBGene00010407 H19N07.3 H19N07.3
0.711359974773213 AGIWUR9792
CB4856 CT V 11119813 11122579 WBGene00010408 mboa-2 H19N07.4
-0.170560774440677 AGIWUR25266
CB4856 CT V 11122820 11123867 WBGene00007385 atp-5 C06H2.1 -0.121817515378353
AGIWUR18502;AGIWUR24548;AGIWUR30030;AGIWUR39039;AGIWUR39317;AGIWUR40026
CB4856 CT V 11124303 11125053 WBGene00007390 lmtr-3 C06H2.6 -0.0488336667876021
AGIWUR18923;AGIWUR20009
CB4856 CT V 11125222 11127306 WBGene00007386 C06H2.2 C06H2.2 -0.239294056817155
AGIWUR30603
CB4856 CT V 11127497 11130085 WBGene00007387 jmjd-5 C06H2.3 -0.122084755504043
AGIWUR9255
CB4856 CT V 11134609 11140323 WBGene00007389 glb-3 C06H2.5 -0.345003020023274
AGIWUR780
CB4856 CT V 11141498 11143121 WBGene00007388 folt-1 C06H2.4 -0.314931932916686
AGIWUR43335
CB4856 CT V 11143062 11144758 WBGene00007391 C06H2.7 C06H2.7 -0.208810073578663
AGIWUR29570
CB4856 CT V 11149705 11151309 WBGene00011838 T19C4.1 T19C4.1 0.860871525210592
AGIWUR8258
CB4856 CT V 11151339 11152430 WBGene00005196 srg-39 T19C4.2 0.102329213926879
AGIWUR13750
CB4856 CT V 11152575 11155653 WBGene00005195 srg-38 T19C4.8 -0.0297662627983835
AGIWUR26785
CB4856 CT V 11157090 11158706 WBGene00005199 srg-42 T19C4.9 -0.146619153490233
AGIWUR13312
CB4856 CT V 11160452 11161891 WBGene00005198 srg-41 T19C4.3 -0.0925373619202814
AGIWUR30808
CB4856 CT V 11163036 11164575 WBGene00005197 srg-40 T19C4.4 -1.09112556460805
AGIWUR38932
CB4856 CT V 11165853 11167640 WBGene00014841 T19C4.10 T19C4.10
-0.131786612362298 AGIWUR28571;AGIWUR44720
CB4856 CT V 11172215 11174452 WBGene00011839 T19C4.5 T19C4.5 1.05651522388458

AGIWUR44772
CB4856 CT V 11174567 11177559 WBGene00001663 gpa-1 T19C4.6 0.0436657557677123
AGIWUR42384
CB4856 CT V 11183224 11183643 WBGene00012152 cnc-10 VK10D6R.1
0.148692902622512 AGIWUR4385
CB4856 CT V 11191283 11193638 WBGene00010741 lgc-49 K10D6.1 -0.286751157889107
AGIWUR8747
CB4856 CT V 11193733 11196010 WBGene00010742 K10D6.2 K10D6.2 0.66039862572439
AGIWUR9438;AGIWUR26231;AGIWUR43418
CB4856 CT V 11200665 11201392 WBGene00010743 K10D6.3 K10D6.3 -0.139580550225819
AGIWUR15976;AGIWUR19250;AGIWUR20801;AGIWUR22472;AGIWUR38353
CB4856 CT V 11201905 11216774 WBGene00010744 K10D6.4 K10D6.4 0.471444655856075
AGIWUR23468
CB4856 CT V 11217583 11219902 WBGene00011830 cyp-29A2 T19B10.1
0.404298212598416 AGIWUR36091
CB4856 CT V 11221302 11222834 WBGene00011831 T19B10.2 T19B10.2
0.165997524299861 AGIWUR3115;AGIWUR10805;AGIWUR11521;AGIWUR25739;AGIWUR29837;AGIWUR39360
CB4856 CT V 11222772 11226018 WBGene00011832 bgal-1 T19B10.3
-0.099600251759302 AGIWUR14003;AGIWUR17301;AGIWUR27843
CB4856 CT V 11226314 11228703 WBGene00004152 pqn-70 T19B10.4
-0.0236074436572542 AGIWUR3150;AGIWUR23106
CB4856 CT V 11234650 11237428 WBGene00011833 T19B10.5 T19B10.5
-0.0275505522337909 AGIWUR22092
CB4856 CT V 11237438 11239331 WBGene00011834 dvc-1 T19B10.6
-0.234562467717323 AGIWUR13415;AGIWUR15411;AGIWUR41047
CB4856 CT V 11239675 11241780 WBGene00002072 ima-1 T19B10.7
-0.083005211002142 AGIWUR5907;AGIWUR8615;AGIWUR15144
CB4856 CT V 11241879 11244726 WBGene00011835 T19B10.8 T19B10.8
-0.0890293931341661 AGIWUR7678;AGIWUR38740
CB4856 CT V 11244839 11245927 WBGene00003509 mxl-1 T19B10.11
0.0606472737537693 AGIWUR42940
CB4856 CT V 11248146 11250866 WBGene00011836 mam-4 T19B10.9
0.00726426898634225 AGIWUR1265;AGIWUR8663;AGIWUR13972;AGIWUR22475;AGIWUR23365;AGIWUR31468
CB4856 CT V 11251035 11251211 WBGene00050898 T19B10.12 T19B10.12
0.0657367348874027 AGIWUR487;AGIWUR6099;AGIWUR24637;AGIWUR29982;AGIWUR40878
CB4856 CT V 11251415 11252529 WBGene00011837 srsx-33 T19B10.10
-0.172985684598546 AGIWUR40610
CB4856 CT V 11254013 11255080 WBGene00008552 srsx-31 F07B10.6
0.125516995411207 AGIWUR24817;AGIWUR36194

CB4856 CT V 11256521 11258996 WBGene00008551 lgc-36 F07B10.5
0.0472277776039265 AGIWUR12869
CB4856 CT V 11259312 11260722 WBGene00077514 F07B10.7 F07B10.7
0.106660910510812 AGIWUR17989
CB4856 CT V 11261264 11263116 WBGene00008550 F07B10.4 F07B10.4
-0.616878961202699 AGIWUR18448;AGIWUR26396
CB4856 CT V 11263523 11265711 WBGene00000539 cln-3.1 F07B10.1
-0.795902293770567 AGIWUR23122
CB4856 CT V 11266981 11268323 WBGene00006167 str-116 F07B10.2
0.119706465341221 AGIWUR32627
CB4856 CT V 11269091 11270426 WBGene00006166 str-115 F07B10.3
0.000177877264848192 AGIWUR8426
CB4856 CT V 11271351 11272467 WBGene00007283 clec-222 C03E10.6
0.178481791956987 AGIWUR34617
CB4856 CT V 11273148 11278161 WBGene00007282 clec-223 C03E10.5
0.536302857106406 AGIWUR34212
CB4856 CT V 11282572 11286039 WBGene00001645 gly-20 C03E10.4
0.0207121903313521 AGIWUR10498;AGIWUR14558;AGIWUR31763
CB4856 CT V 11286602 11288412 WBGene00007281 C03E10.3 C03E10.3
0.0027231915630157 AGIWUR22175;AGIWUR35237;AGIWUR43907
CB4856 CT V 11291188 11292569 WBGene00007279 C03E10.1 C03E10.1
0.0587833072226198 AGIWUR31775
CB4856 CT V 11294693 11296562 WBGene00011403 T03F7.6 T03F7.6 0.0981089999936797
AGIWUR30648
CB4856 CT V 11296696 11298732 WBGene00011402 T03F7.5 T03F7.5 0.0207711211870762
AGIWUR25048
CB4856 CT V 11299373 11300639 WBGene00005270 srh-47 T03F7.4 0.163960709231183
AGIWUR8046;AGIWUR39403
CB4856 CT V 11301607 11302886 WBGene00005269 srh-46 T03F7.3 -0.114357265850622
AGIWUR18968
CB4856 CT V 11303651 11304979 WBGene00005268 srh-45 T03F7.2 -0.386580232723504
AGIWUR15350;AGIWUR19467
CB4856 CT V 11305070 11308446 WBGene00004910 snf-11 T03F7.1 0.193061923995268
AGIWUR20858
CB4856 CT V 11311456 11313445 WBGene00011404 T03F7.7 T03F7.7 -0.117815339110079
AGIWUR17896;AGIWUR18547
CB4856 CT V 11313400 11316901 WBGene00009830 cutl-18 F47G9.3 -0.705272278969526
AGIWUR31545
CB4856 CT V 11317214 11321786 WBGene00009831 F47G9.4 F47G9.4 -0.0583324435464139

AGIWUR4717;AGIWUR9709;AGIWUR14335;AGIWUR21384;AGIWUR42673
CB4856 CT V 11322322 11323820 WBGene00006189 str-141 F47G9.2 0.34126734974997
AGIWUR9795
CB4856 CT V 11323965 11326964 WBGene00009829 F47G9.1 F47G9.1 -0.0179966866229471
AGIWUR8533;AGIWUR11906;AGIWUR24323
CB4856 CT V 11329400 11330775 WBGene00009833 F47G9.6 F47G9.6 0.0651948294693412
AGIWUR24729
CB4856 CT V 11332987 11334293 WBGene00044203 T02E9.6 T02E9.6 0.0617707625538162
AGIWUR1151;AGIWUR30893
CB4856 CT V 11342233 11344773 WBGene00011383 T02E9.5 T02E9.5 0.470346242726525
AGIWUR4132;AGIWUR6704;AGIWUR13819;AGIWUR22708;AGIWUR22756;AGIWUR24772
CB4856 CT V 11344831 11347012 WBGene00001716 grl-7 T02E9.2 -0.072319470280676
AGIWUR14923;AGIWUR42462
CB4856 CT V 11349310 11352993 WBGene00011381 npr-25 T02E9.1 0.255181513309926
AGIWUR27897
CB4856 CT V 11362453 11366221 WBGene00011382 dop-5 T02E9.3 0.157706820279722
AGIWUR6998
CB4856 CT V 11375742 11377990 WBGene00000554 cnb-1 F55C10.1
0.288631242398427 AGIWUR15966
CB4856 CT V 11383105 11384353 WBGene00010109 F55C10.5 F55C10.5
-0.0879060339687037 AGIWUR28132
CB4856 CT V 11385360 11386529 WBGene00000727 col-154 F55C10.2
0.0746546424273795 AGIWUR18601
CB4856 CT V 11386698 11388678 WBGene00010108 F55C10.4 F55C10.4
-0.464441766206177 AGIWUR44361
CB4856 CT V 11390273 11390494 WBGene00010526 K03B8.11 K03B8.11
-0.530715216540763 AGIWUR10764
CB4856 CT V 11392320 11393508 WBGene00003535 nas-16 K03B8.1 -0.0280294618998012
AGIWUR5158;AGIWUR9549
CB4856 CT V 11398916 11400528 WBGene00003536 nas-17 K03B8.2 -1.06005210048703
AGIWUR16781
CB4856 CT V 11401195 11402690 WBGene00003538 nas-19 K03B8.5 -0.473194925879366
AGIWUR2377
CB4856 CT V 11407523 11409543 WBGene00010524 K03B8.6 K03B8.6 0.0209624646516313
AGIWUR9809;AGIWUR37619
CB4856 CT V 11409467 11411019 WBGene00010525 K03B8.8 K03B8.8 -0.0195580883091999
AGIWUR796;AGIWUR8221;AGIWUR10078;AGIWUR13769;AGIWUR34518;AGIWUR40338;AGIWUR44676
CB4856 CT V 11411511 11412769 WBGene00001712 grl-3 K03B8.7 0.00625500513982047
AGIWUR3545

CB4856 CT V 11412870 11415159 WBGene00000951 deg-3 K03B8.9 0.0715988840934134
AGIWUR33003
CB4856 CT V 11415342 11420293 WBGene00000955 des-2 T26H10.1
0.247735748599125 AGIWUR24189
CB4856 CT V 11438770 11441618 WBGene00010191 dmsr-1 F57B7.1 0.17836258314694
AGIWUR10396;AGIWUR16007
CB4856 CT V 11441925 11443603 WBGene00010192 F57B7.2 F57B7.2 -0.0539822465828461
AGIWUR12817
CB4856 CT V 11446633 11449245 WBGene00003248 mig-17 F57B7.4 -0.400669185981322
AGIWUR40715
CB4856 CT V 11449223 11450357 WBGene00000729 col-156 F57B7.3 -0.0441637596316165
AGIWUR10540
CB4856 CT V 11462999 11466770 WBGene00006486 zipt-21 T11F9.2 -0.414534590364129
AGIWUR1480
CB4856 CT V 11466844 11470663 WBGene00000007 aat-6 T11F9.4 -0.153329168092283
AGIWUR20033
CB4856 CT V 11470868 11472349 WBGene00003539 nas-20 T11F9.3 -0.570065773946837
AGIWUR1380;AGIWUR5186;AGIWUR11424;AGIWUR18119
CB4856 CT V 11472561 11473973 WBGene00003540 nas-21 T11F9.5 -0.0156776890701638
AGIWUR4677;AGIWUR5223;AGIWUR9699;AGIWUR11738;AGIWUR12453;AGIWUR17204;AGIWUR18508;AGIWUR43832
CB4856 CT V 11474699 11477951 WBGene00003541 nas-22 T11F9.6 -0.228105503989728
AGIWUR3819;AGIWUR8826;AGIWUR9112;AGIWUR26329;AGIWUR36117;AGIWUR37505;AGIWUR42006
CB4856 CT V 11478413 11479243 WBGene00005235 srh-9 T11F9.17
-0.168751692764623 AGIWUR24741;AGIWUR43754;AGIWUR44860
CB4856 CT V 11485858 11487266 WBGene00005227 srh-1 T11F9.18
-0.167749836673618 AGIWUR24579
CB4856 CT V 11487152 11488275 WBGene00011712 T11F9.10 T11F9.10
0.00628131665598453 AGIWUR16276
CB4856 CT V 11489050 11490328 WBGene00000982 dhs-19 T11F9.11
0.148378454279873 AGIWUR39451
CB4856 CT V 11491829 11492590 WBGene00011716 T11F9.19 T11F9.19
0.34322205994013 AGIWUR31163
CB4856 CT V 11493556 11496379 WBGene00011713 T11F9.12 T11F9.12
-0.119737822371263 AGIWUR14163
CB4856 CT V 11501444 11503127 WBGene00044019 C55A6.12 C55A6.12
-0.00997655264659958 AGIWUR10900
CB4856 CT V 11503811 11504884 WBGene00008330 C55A6.1 C55A6.1 -0.223243799210473
AGIWUR2304;AGIWUR41867
CB4856 CT V 11505352 11508271 WBGene00008339 C55A6.10 C55A6.10

								-0.00126814886873206	AGIWUR2761
CB4856	CT	V	11509068	11512318	WBGene00008331	ttll-5	C55A6.2	-0.059332043054328	AGIWUR31123;AGIWUR43920
CB4856	CT	V	11512313	11513436	WBGene00008332	C55A6.3	C55A6.3	0.0214475672561782	AGIWUR30062
CB4856	CT	V	11513665	11514737	WBGene00008335	C55A6.6	C55A6.6	-0.221253241995759	AGIWUR7253
CB4856	CT	V	11515084	11516191	WBGene00008334	sdz-8	C55A6.5	-0.332268390681371	AGIWUR44215
CB4856	CT	V	11516445	11517889	WBGene00008333	C55A6.4	C55A6.4	-0.426171721272731	AGIWUR12044
CB4856	CT	V	11518100	11518702	WBGene00008337	C55A6.8	C55A6.8	0.0839881144435521	AGIWUR3620;AGIWUR12782;AGIWUR19330
CB4856	CT	V	11519335	11520530	WBGene00008336	C55A6.7	C55A6.7	-0.0364970161643865	AGIWUR4983;AGIWUR10473;AGIWUR12082;AGIWUR43879
CB4856	CT	V	11520515	11521554	WBGene00008340	C55A6.11	C55A6.11	-0.0493042019143566	AGIWUR17876
CB4856	CT	V	11521653	11523384	WBGene00008338	pafo-1	C55A6.9	-0.182786522217981	AGIWUR4256
CB4856	CT	V	11527831	11541219	WBGene00009532	ccch-1	F38B7.1	0.14080667784541	AGIWUR1888;AGIWUR15184;AGIWUR18653;AGIWUR21592;AGIWUR31708;AGIWUR35392;AGIWUR38363
CB4856	CT	V	11543857	11545290	WBGene00009533	F38B7.2	F38B7.2	-0.267052532052172	AGIWUR5222;AGIWUR12757;AGIWUR16809;AGIWUR30377;AGIWUR33388
CB4856	CT	V	11545536	11546376	WBGene00077536	F38B7.10	F38B7.10	0.158487097149663	AGIWUR1421;AGIWUR14756;AGIWUR33638
CB4856	CT	V	11549107	11551560	WBGene00009534	F38B7.3	F38B7.3	0.266727757986553	AGIWUR33781
CB4856	CT	V	11552679	11554222	WBGene00005976	srx-85	F38B7.4	0.234902509765698	AGIWUR36588
CB4856	CT	V	11554635	11558127	WBGene00001110	duo-1	F38B7.5	-0.169791525442701	AGIWUR6255;AGIWUR23547;AGIWUR41872
CB4856	CT	V	11558319	11559881	WBGene00005962	srx-71	F38B7.6	0.235076320436232	AGIWUR33725
CB4856	CT	V	11560078	11561650	WBGene00005963	srx-72	F38B7.9	-0.0549687217579407	AGIWUR5244;AGIWUR41450
CB4856	CT	V	11562943	11564508	WBGene00005977	srx-86	F38B7.7	-0.00382088903252908	AGIWUR22026;AGIWUR22798
CB4856	CT	V	11564873	11566771	WBGene00005978	srx-87	F38B7.8	0.0785501902029688	AGIWUR5096;AGIWUR11440;AGIWUR14755

CB4856 CT V 11566804 11573091 WBGene00004064 pms-2 H12C20.2
-0.0713534056054064 AGIWUR22401;AGIWUR36857;AGIWUR38193
CB4856 CT V 11573440 11575709 WBGene00003658 nhr-68 H12C20.3
-0.380580393723019 AGIWUR12727
CB4856 CT V 11576631 11577911 WBGene00005982 srx-91 H12C20.4
-0.0294821476239528 AGIWUR1007;AGIWUR11759;AGIWUR22109;AGIWUR31195;AGIWUR31842
CB4856 CT V 11581126 11582458 WBGene00005981 srx-90 H12C20.5
0.19946004259706 AGIWUR28808
CB4856 CT V 11583512 11585115 WBGene00005979 srx-88 F43A11.1
-0.0359885433237652 AGIWUR12245
CB4856 CT V 11586259 11588133 WBGene00005980 srx-89 F43A11.3
-0.0237960607259367 AGIWUR8733;AGIWUR29031;AGIWUR33331;AGIWUR35615
CB4856 CT V 11589733 11592931 WBGene00005973 srx-82 F43A11.6
0.0524808446810505 AGIWUR5392;AGIWUR12579
CB4856 CT V 11593727 11594971 WBGene00005971 srx-80 F43A11.4
-0.11496948930613 AGIWUR26018
CB4856 CT V 11595919 11597096 WBGene00005972 srx-81 F43A11.5
0.00236762170302462 AGIWUR1854;AGIWUR40726
CB4856 CT V 11597346 11598897 WBGene00005974 srx-83 F43A11.7
0.219272175159436 AGIWUR31700
CB4856 CT V 11599107 11600628 WBGene00005975 srx-84 F35B12.1
-0.0310556502685526 AGIWUR17845;AGIWUR35416
CB4856 CT V 11601119 11602358 WBGene00009388 F35B12.9 F35B12.9
0.0704237473581069 AGIWUR1481;AGIWUR14629;AGIWUR18779;AGIWUR43752
CB4856 CT V 11602294 11604563 WBGene00000983 dhs-20 F35B12.2
0.0681411589524075 AGIWUR1985
CB4856 CT V 11609096 11610149 WBGene00009384 piit-1 F35B12.4
-0.0589870887637751 AGIWUR44326
CB4856 CT V 11610708 11613054 WBGene00009385 sas-5 F35B12.5
-0.078752709107321 AGIWUR12489
CB4856 CT V 11613307 11614888 WBGene00009386 tag-290 F35B12.6
-0.238026373789332 AGIWUR13593
CB4856 CT V 11616283 11616829 WBGene00003762 nlp-24 F35B12.7
-0.67979566287746 AGIWUR2533;AGIWUR13008;AGIWUR22163;AGIWUR24238;AGIWUR32188;AGIWUR39141
CB4856 CT V 11616812 11618266 WBGene00009387 glb-15 F35B12.8
0.344125688514186 AGIWUR37261
CB4856 CT V 11625954 11629322 WBGene00009389 F35B12.10 F35B12.10
0.138270622099167 AGIWUR31419
CB4856 CT V 11631434 11637291 WBGene00012100 sipa-1 T27F2.2 0.174300588656614

AGIWUR40216
CB4856 CT V 11644760 11645370 WBGene00000249 bir-1 T27F2.3 0.0725210500338527
AGIWUR10307
CB4856 CT V 11645481 11650090 WBGene00004806 skp-1 T27F2.1 -0.196988268627402
AGIWUR2641
CB4856 CT V 11650079 11650774 WBGene00012101 zip-10 T27F2.4 0.318958909994935
AGIWUR6110;AGIWUR19171;AGIWUR21334;AGIWUR34172
CB4856 CT V 11661670 11665085 WBGene00000449 ceh-27 F46F3.1 0.807714321574291
AGIWUR17161
CB4856 CT V 11667476 11669390 WBGene00009791 F46F3.3 F46F3.3 0.0680390950461596
AGIWUR8266;AGIWUR11650;AGIWUR14168;AGIWUR16448;AGIWUR26148;AGIWUR36536;AGIWUR42656;AGIWUR42960
CB4856 CT V 11669348 11672594 WBGene00009790 lgc-44 F46F3.2 -0.109093560188679
AGIWUR7570
CB4856 CT V 11675942 11685268 WBGene00000146 ape-1 F46F3.4 -0.255017813847108
AGIWUR2585
CB4856 CT V 11687426 11689174 WBGene00012281 W05E10.2 W05E10.2
0.230335366185512 AGIWUR16303
CB4856 CT V 11689413 11691691 WBGene00012280 W05E10.1 W05E10.1
-0.294638868495161 AGIWUR31908
CB4856 CT V 11693188 11695697 WBGene00012282 W05E10.5 W05E10.5
0.00652243460161461 AGIWUR304
CB4856 CT V 11701764 11705400 WBGene00000453 ceh-32 W05E10.3
0.444928577102583 AGIWUR16411;AGIWUR39527;AGIWUR43749
CB4856 CT V 11709775 11715903 WBGene00006609 tre-3 W05E10.4
0.378845657769295 AGIWUR17988;AGIWUR19737;AGIWUR37150
CB4856 CT V 11718089 11725652 WBGene00000907 daf-11 B0240.3 0.0658852029640808
AGIWUR23822
CB4856 CT V 11727900 11730160 WBGene00004359 rhr-2 B0240.1 -0.0813092828137876
AGIWUR10193
CB4856 CT V 11731120 11733472 WBGene00007118 npp-22 B0240.4 0.0613217458056798
AGIWUR10766
CB4856 CT V 11733733 11736968 WBGene00007117 spe-42 B0240.2 0.173669338637451
AGIWUR9063
CB4856 CT V 11740065 11754109 WBGene00011436 T04F3.1 T04F3.1 -0.0750557987226195
AGIWUR37668
CB4856 CT V 11757668 11758598 WBGene00011437 T04F3.2 T04F3.2 0.332702930462447
AGIWUR37822
CB4856 CT V 11758935 11760570 WBGene00011438 T04F3.3 T04F3.3 0.0300703251500281
AGIWUR2595;AGIWUR18129;AGIWUR40947

CB4856	CT	V	11760671	11761894	WBGene00077538	T04F3.5	T04F3.5	0.145143970912464	AGIWUR8529
CB4856	CT	V	11761893	11763152	WBGene00011439	T04F3.4	T04F3.4	-0.681055561367216	AGIWUR40120
CB4856	CT	V	11763140	11764382	WBGene00010075	F55A11.1	F55A11.1	-0.143817974528687	AGIWUR6498
CB4856	CT	V	11764904	11767222	WBGene00006373	syx-5	F55A11.2	0.0859449032965856	AGIWUR44160
CB4856	CT	V	11767328	11769899	WBGene00004768	sel-11	F55A11.3	-0.132235427357877	AGIWUR15843
CB4856	CT	V	11770456	11773495	WBGene00010077	F55A11.4	F55A11.4	0.029034988988629	AGIWUR5298;AGIWUR7111;AGIWUR18868
CB4856	CT	V	11773720	11776631	WBGene00044631	bus-18	F55A11.5	0.370270865938038	AGIWUR15849;AGIWUR24966;AGIWUR28370;AGIWUR28599;AGIWUR31508
CB4856	CT	V	11776838	11783422	WBGene00010079	F55A11.6	F55A11.6	-0.410557869231294	AGIWUR34008
CB4856	CT	V	11783340	11784616	WBGene00010082	F55A11.11	F55A11.11	0.110361635711204	AGIWUR13045
CB4856	CT	V	11784573	11787443	WBGene00010080	F55A11.7	F55A11.7	-0.00993194994792646	AGIWUR11196
CB4856	CT	V	11788157	11789121	WBGene00010081	F55A11.8	F55A11.8	0.0780778635854489	AGIWUR34175
CB4856	CT	V	11789550	11800544	WBGene00014300	D2023.1	D2023.1	0.00665501372875132	AGIWUR536;AGIWUR3337;AGIWUR6597;AGIWUR7176;AGIWUR7685;AGIWUR12399;AGIWUR14258;AGIWUR20071;AGIWUR22319;AGIWUR22876;AGIWUR27785;AGIWUR29680;AGIWUR30851;AGIWUR30970;AGIWUR37689;AGIWUR41203
CB4856	CT	V	11802105	11811943	WBGene00008407	D2023.3	D2023.3	-0.1697498839557	AGIWUR13552
CB4856	CT	V	11817783	11822529	WBGene00004258	pyc-1	D2023.2	-0.122489443443858	AGIWUR3941;AGIWUR5430;AGIWUR24488;AGIWUR26160;AGIWUR38320;AGIWUR39419
CB4856	CT	V	11825033	11825762	WBGene00008408	D2023.4	D2023.4	-0.357397058692717	AGIWUR28004
CB4856	CT	V	11825897	11827067	WBGene00008409	mpst-1	D2023.5	-0.151581381052907	AGIWUR2371
CB4856	CT	V	11827306	11829370	WBGene00008410	D2023.6	D2023.6	0.0749379908524723	AGIWUR39106
CB4856	CT	V	11829582	11831220	WBGene00000731	col-158	D2023.7	-0.841399059864468	AGIWUR34847
CB4856	CT	V	11831203	11843625	WBGene00011261	nphp-4	R13H4.1	0.438685217322846	AGIWUR34994

CB4856	CT	V	11845877	11852165	WBGene00001978	hmp-1	R13H4.4	0.176137057373454	AGIWUR6777;AGIWUR24555
CB4856	CT	V	11860675	11862289	WBGene00011263	R13H4.5	R13H4.5	0.091440679150729	AGIWUR27117
CB4856	CT	V	11863117	11864913	WBGene00011264	R13H4.6	R13H4.6	-0.216902455811146	AGIWUR14613
CB4856	CT	V	11866438	11868234	WBGene00011265	R13H4.7	R13H4.7	0.178688084317526	AGIWUR35907
CB4856	CT	V	11875360	11879141	WBGene00010788	sulp-4	K12G11.1	0.053510781191342	AGIWUR43811
CB4856	CT	V	11880068	11883160	WBGene00010789	sulp-5	K12G11.2	-0.127528195886229	AGIWUR10275;AGIWUR28603;AGIWUR33364
CB4856	CT	V	11888236	11889634	WBGene00010790	sodh-1	K12G11.3	5.49635082510986	AGIWUR19293;AGIWUR22729
CB4856	CT	V	11889719	11891194	WBGene00010791	sodh-2	K12G11.4	0.673094119941443	AGIWUR7398
CB4856	CT	V	11891812	11893451	WBGene00005967	srx-76	K12G11.5	-0.014843561054084	AGIWUR22594;AGIWUR37306;AGIWUR41361
CB4856	CT	V	11894331	11895808	WBGene00010792	K12G11.6	K12G11.6	0.252187995593581	AGIWUR7419
CB4856	CT	V	11899072	11916623	WBGene00004855	sma-1	R31.1	0.206352839653251	AGIWUR10020;AGIWUR22705;AGIWUR35546;AGIWUR37626;AGIWUR42804;AGIWUR45024
CB4856	CT	V	11917564	11924654	WBGene00011270	R31.2	R31.2	0.188415493089394	AGIWUR240;AGIWUR32724
CB4856	CT	V	11924632	11927406	WBGene00003886	osm-6	R31.3	0.631329532516101	AGIWUR28117
CB4856	CT	V	11927661	11933246	WBGene00010284	aman-2	F58H1.1	0.128222611252081	AGIWUR18036;AGIWUR33704;AGIWUR35490
CB4856	CT	V	11933881	11935052	WBGene00010286	F58H1.3	F58H1.3	0.038280487716432	AGIWUR9064;AGIWUR15551;AGIWUR22696;AGIWUR33803
CB4856	CT	V	11935148	11936528	WBGene00138720	F58H1.8	F58H1.8	-0.436666246591333	AGIWUR9603
CB4856	CT	V	11936505	11937794	WBGene00001466	flr-2	F58H1.4	0.0979473750376738	AGIWUR5295;AGIWUR13263;AGIWUR24230;AGIWUR38451
CB4856	CT	V	11942625	11944656	WBGene00010289	F58H1.6	F58H1.6	-0.218704482948151	AGIWUR2655;AGIWUR8435;AGIWUR29734
CB4856	CT	V	11958393	11962699	WBGene00010288	F58H1.5	F58H1.5	0.376003153379415	AGIWUR13260;AGIWUR22778;AGIWUR23737
CB4856	CT	V	11963007	11964821	WBGene00010290	F58H1.7	F58H1.7	0.32657833865286	

AGIWUR2934;AGIWUR9376;AGIWUR13051;AGIWUR13113;AGIWUR19170;AGIWUR20927

CB4856	CT	V	11975515	11976756	WBGene00000781	cpr-1	C52E4.1	0.387550890916346	AGIWUR38171;AGIWUR44556
CB4856	CT	V	11976777	11978459	WBGene00003235	mif-2	C52E4.2	-0.249941160086669	AGIWUR17960
CB4856	CT	V	11978518	11979970	WBGene00004917	snr-4	C52E4.3	-0.212733986683208	AGIWUR9177
CB4856	CT	V	11980502	11982450	WBGene00004501	rpt-1	C52E4.4	-0.115350896286403	AGIWUR64;AGIWUR28575;AGIWUR40117
CB4856	CT	V	11982901	11986115	WBGene00008258	mans-2	C52E4.5	-0.355204798506325	AGIWUR8557
CB4856	CT	V	11991011	11993767	WBGene00000876	cyl-1	C52E4.6	0.0226359501180537	AGIWUR19976;AGIWUR44208
CB4856	CT	V	11994295	11995858	WBGene00008259	C52E4.7	C52E4.7	-0.0855561898353585	AGIWUR33143
CB4856	CT	V	11995868	12001965	WBGene00001553	gcy-33	F57F5.2	0.204987292087822	AGIWUR6682
CB4856	CT	V	12003028	12004600	WBGene00010204	F57F5.1	F57F5.1	0.00765632737214617	AGIWUR12276;AGIWUR20040;AGIWUR32898
CB4856	CT	V	12004616	12006605	WBGene00010205	F57F5.3	F57F5.3	0.131398707202065	AGIWUR39013
CB4856	CT	V	12009537	12013605	WBGene00000073	add-2	F57F5.4	-0.0930202796731798	AGIWUR9329;AGIWUR31353;AGIWUR39004
CB4856	CT	V	12014385	12032131	WBGene00004032	pkc-1	F57F5.5	-0.0335678318551693	AGIWUR8741;AGIWUR15869;AGIWUR21697;AGIWUR35101;AGIWUR39298;AGIWUR41185
CB4856	CT	V	12032257	12040873	WBGene00002241	kup-1	F10C2.2	-0.0617083476155038	AGIWUR7433
CB4856	CT	V	12041556	12045347	WBGene00008645	F10C2.4	F10C2.4	0.0526660641833896	AGIWUR15408
CB4856	CT	V	12045432	12048631	WBGene00008646	F10C2.5	F10C2.5	-0.111035703020242	AGIWUR42690
CB4856	CT	V	12048745	12052182	WBGene00001095	dars-2	F10C2.6	-0.175785119289323	AGIWUR15266;AGIWUR20285;AGIWUR42451
CB4856	CT	V	12053952	12056320	WBGene00008647	F10C2.7	F10C2.7	0.553922897639831	AGIWUR18774
CB4856	CT	V	12056575	12059747	WBGene00011087	R07B7.2	R07B7.2	-0.0526323360037182	AGIWUR41963
CB4856	CT	V	12060234	12063368	WBGene00000533	clh-6	R07B7.1	-0.192998683101028	AGIWUR25470;AGIWUR40237;AGIWUR43301

CB4856	CT	V	12063432	12065391	WBGene00004138	pqn-53	R07B7.3	0.181425913919115	AGIWUR27696
CB4856	CT	V	12065960	12067957	WBGene00011088	kmo-2	R07B7.4	-0.0536420375627564	AGIWUR13702;AGIWUR15063;AGIWUR19014
CB4856	CT	V	12068725	12070519	WBGene00011089	kmo-1	R07B7.5	-0.263912086025147	AGIWUR3864
CB4856	CT	V	12072862	12074986	WBGene00011090	R07B7.6	R07B7.6	0.28572677809282	AGIWUR10108;AGIWUR27116;AGIWUR33997;AGIWUR35278
CB4856	CT	V	12078044	12080374	WBGene00011093	R07B7.9	R07B7.9	-0.0833320849764725	AGIWUR14262
CB4856	CT	V	12081089	12084316	WBGene00011092	R07B7.8	R07B7.8	-0.127374494897779	AGIWUR7397
CB4856	CT	V	12084186	12086460	WBGene00011094	R07B7.10	R07B7.10	-0.377995053064277	AGIWUR4600
CB4856	CT	V	12087234	12088931	WBGene00011095	gana-1	R07B7.11	-0.0754561805342338	AGIWUR1945
CB4856	CT	V	12089073	12091812	WBGene00011096	R07B7.12	R07B7.12	-0.0037492298288635	AGIWUR15;AGIWUR15650;AGIWUR17447
CB4856	CT	V	12092006	12093862	WBGene00011097	nhr-206	R07B7.13	1.10438599220885	AGIWUR31961
CB4856	CT	V	12094374	12095947	WBGene00011099	nhr-208	R07B7.15	0.6329968887635	AGIWUR15985
CB4856	CT	V	12096234	12097845	WBGene00011098	nhr-207	R07B7.14	-0.27719296291952	AGIWUR6595
CB4856	CT	V	12098149	12100111	WBGene00011100	nhr-209	R07B7.16	0.13437585164369	AGIWUR29309
CB4856	CT	V	12101293	12103286	WBGene00007547	nhr-154	C13C4.2	-0.195938493243174	AGIWUR8400
CB4856	CT	V	12104273	12106507	WBGene00007546	nhr-153	C13C4.1	-0.223910360218733	AGIWUR39908
CB4856	CT	V	12107142	12109201	WBGene00003726	nhr-136	C13C4.3	0.185094142006689	AGIWUR39836
CB4856	CT	V	12109168	12110978	WBGene00007548	C13C4.4	C13C4.4	-0.236653056671589	AGIWUR37050
CB4856	CT	V	12114993	12117443	WBGene00007549	spin-1	C13C4.5	-0.130998711127059	AGIWUR16171
CB4856	CT	V	12118828	12120814	WBGene00007551	C13C4.7	C13C4.7	0.0204381634385715	AGIWUR3454
CB4856	CT	V	12121587	12125096	WBGene00007550	C13C4.6	C13C4.6	0.35900143277695	

AGIWUR31466
CB4856 CT V 12124850 12130788 WBGene00007757 C27A7.6 C27A7.6 0.0753775965557839
AGIWUR40902
CB4856 CT V 12131442 12137395 WBGene00007753 C27A7.1 C27A7.1 -0.199658791188444
AGIWUR29919
CB4856 CT V 12138354 12140322 WBGene00007754 C27A7.2 C27A7.2 0.262797989282146
AGIWUR35166
CB4856 CT V 12140876 12144259 WBGene00007755 C27A7.3 C27A7.3 -0.357761761647797
AGIWUR1656;AGIWUR4008;AGIWUR17633
CB4856 CT V 12144295 12150689 WBGene00000490 che-11 C27A7.4 0.457256902033815
AGIWUR4075
CB4856 CT V 12153076 12154264 WBGene00007758 C27A7.8 C27A7.8 1.13840339993541
AGIWUR21800;AGIWUR23789
CB4856 CT V 12154263 12154654 WBGene00007759 C27A7.9 C27A7.9 0.151203535275773
AGIWUR18457
CB4856 CT V 12154875 12162299 WBGene00007756 C27A7.5 C27A7.5 -0.161552468505567
AGIWUR4399
CB4856 CT V 12163206 12164535 WBGene00005592 srj-4 C27A7.7 0.0797576111261709
AGIWUR26885
CB4856 CT V 12165212 12166662 WBGene00012147 VC27A7L.1 VC27A7L.1
0.233132968603122 AGIWUR16217
CB4856 CT V 12166706 12167753 WBGene00000614 col-37 ZK863.2 0.244179825197897
AGIWUR43462
CB4856 CT V 12168222 12170208 WBGene00014122 ZK863.1 ZK863.1 0.0785011718240654
AGIWUR734;AGIWUR13262;AGIWUR31650
CB4856 CT V 12177088 12179438 WBGene00044790 clec-224 ZK863.9
0.124850274883907 AGIWUR9415;AGIWUR27979;AGIWUR28764;AGIWUR32666
CB4856 CT V 12179503 12181410 WBGene00014123 elpc-3 ZK863.3 0.0903054502725826
AGIWUR36908
CB4856 CT V 12181501 12184194 WBGene00014124 usip-1 ZK863.4 0.00392843488363681
AGIWUR10858
CB4856 CT V 12184949 12186245 WBGene00005082 srd-4 ZK863.5 0.214426755447324
AGIWUR4801
CB4856 CT V 12186335 12187496 WBGene00014125 ZK863.8 ZK863.8 0.683406857402291
AGIWUR2181
CB4856 CT V 12191519 12192211 WBGene00001088 dpy-30 ZK863.6 -0.0554962143867521
AGIWUR14525;AGIWUR23895;AGIWUR28944;AGIWUR35982
CB4856 CT V 12192273 12194445 WBGene00004384 rnp-1 ZK863.7 -0.105093019628811
AGIWUR10525;AGIWUR31476;AGIWUR42654

CB4856	CT	V	12194457	12205494	WBGene00014098	ogdh-2	ZK836.2	-0.0613349634999079	AGIWUR6700;AGIWUR15933;AGIWUR32427
CB4856	CT	V	12213327	12225935	WBGene00006874	vab-8	K12F2.2	-0.00976692228858411	AGIWUR29761
CB4856	CT	V	12226816	12234343	WBGene00003515	myo-3	K12F2.1	0.059606032309123	AGIWUR39311
CB4856	CT	V	12236895	12238599	WBGene00011448	tag-348	T04H1.1	0.61006443444048	AGIWUR7345
CB4856	CT	V	12238938	12240316	WBGene00005827	srw-80	T04H1.10	-1.04048245297612	AGIWUR21658
CB4856	CT	V	12241409	12241945	WBGene00011450	ttr-22	T04H1.3	0.240856645106203	AGIWUR3349;AGIWUR4769;AGIWUR13278;AGIWUR25891
CB4856	CT	V	12241962	12245059	WBGene00011449	T04H1.2	T04H1.2	0.172409852924723	AGIWUR2792;AGIWUR12771;AGIWUR16025;AGIWUR31410;AGIWUR38140
CB4856	CT	V	12245594	12251246	WBGene00004296	rad-50	T04H1.4	-0.310862254439119	AGIWUR21464
CB4856	CT	V	12251341	12252407	WBGene00011451	T04H1.5	T04H1.5	0.382535145511785	AGIWUR10392
CB4856	CT	V	12252623	12255450	WBGene00003075	lrx-1	T04H1.6	-0.268339793721575	AGIWUR1303
CB4856	CT	V	12255547	12258804	WBGene00011452	ugt-55	T04H1.7	0.265436759215654	AGIWUR11839
CB4856	CT	V	12258982	12261205	WBGene00011453	ugt-56	T04H1.8	0.0456584866999803	AGIWUR34483;AGIWUR37835
CB4856	CT	V	12261804	12263368	WBGene00006539	tbb-6	T04H1.9	-0.706100252833778	AGIWUR44382
CB4856	CT	V	12263369	12263896	WBGene00010091	ssp-35	F55C5.1	0.286482580314589	AGIWUR39324
CB4856	CT	V	12264002	12265492	WBGene00010092	F55C5.2	F55C5.2	-0.463071132302188	AGIWUR41701
CB4856	CT	V	12265707	12269549	WBGene00006677	twk-24	F55C5.3	-0.176839666529049	AGIWUR23192
CB4856	CT	V	12270271	12271551	WBGene00010094	tsfm-1	F55C5.5	-0.23298052944889	AGIWUR2050;AGIWUR9824;AGIWUR31526
CB4856	CT	V	12271873	12275676	WBGene00010093	capg-2	F55C5.4	-0.0206602507156006	AGIWUR40113
CB4856	CT	V	12275625	12277046	WBGene00044791	bnc-1	F55C5.11	0.0754919790670119	AGIWUR24922
CB4856	CT	V	12279506	12281461	WBGene00010095	F55C5.6	F55C5.6	-0.0787996171237317	

AGIWUR36761
CB4856 CT V 12283487 12283999 WBGene00010098 F55C5.10 F55C5.10
0.420089237658337 AGIWUR24288
CB4856 CT V 12284060 12288615 WBGene00010096 rskd-1 F55C5.7 -0.0151755614926907
AGIWUR20265;AGIWUR20565;AGIWUR36910;AGIWUR40876
CB4856 CT V 12288922 12291658 WBGene00010097 srpa-68 F55C5.8 -0.168471799521159
AGIWUR4738;AGIWUR11042;AGIWUR28005;AGIWUR30627;AGIWUR33642;AGIWUR44914
CB4856 CT V 12292214 12293802 WBGene00005241 srh-16 F55C5.9
-0.00334700549526978 AGIWUR30040
CB4856 CT V 12302484 12306978 WBGene00001493 frm-7 C51F7.1 0.233293563244384
AGIWUR18582
CB4856 CT V 12307490 12308658 WBGene00005202 srg-45 C51F7.2 0.019688603244262
AGIWUR10609
CB4856 CT V 12308849 12310342 WBGene00011014 hpo-30 R04F11.1
-0.203057574194737 AGIWUR27617
CB4856 CT V 12314919 12317180 WBGene00011016 R04F11.3 R04F11.3
0.0981726897469954 AGIWUR28374
CB4856 CT V 12317278 12318019 WBGene00011017 R04F11.5 R04F11.5
-0.424341496286981 AGIWUR26518
CB4856 CT V 12318385 12320229 WBGene00011015 R04F11.2 R04F11.2
-0.0107387546912205 AGIWUR549;AGIWUR9276;AGIWUR21822;AGIWUR24633;AGIWUR37581;AGIWUR41818
CB4856 CT V 12320187 12337744 WBGene00006668 twk-13 R04F11.4
-0.05181940599575 AGIWUR28680;AGIWUR39921;AGIWUR40661
CB4856 CT V 12342314 12343469 WBGene00009092 tomm-20 F23H12.2
-0.180536289894722 AGIWUR10782
CB4856 CT V 12344188 12345919 WBGene00004898 snb-2 F23H12.1
0.182636688964534 AGIWUR13735;AGIWUR22688;AGIWUR24346;AGIWUR26228;AGIWUR32107;AGIWUR39035;AGIWUR41382
CB4856 CT V 12345935 12350451 WBGene00009093 F23H12.3 F23H12.3
-0.0777336325254971 AGIWUR18327
CB4856 CT V 12352994 12354314 WBGene00005018 sqt-3 F23H12.4
0.565708485597725 AGIWUR30261;AGIWUR42458
CB4856 CT V 12356256 12357309 WBGene00050938 F23H12.11 F23H12.11
-0.0769091636024015 AGIWUR12590;AGIWUR34990
CB4856 CT V 12358647 12363691 WBGene00009094 F23H12.5 F23H12.5
0.246870795839001 AGIWUR40074
CB4856 CT V 12363754 12369537 WBGene00001539 gcy-13 F23H12.6
0.0100730689724529 AGIWUR26383
CB4856 CT V 12371730 12372870 WBGene00009095 F23H12.7 F23H12.7
-0.0202870451871604 AGIWUR33584

CB4856	CT	V	12377556	12378832	WBGene00010461	K01D12.1	K01D12.1	0.691012470500038	AGIWUR30600
CB4856	CT	V	12381254	12385623	WBGene00010463	K01D12.3	K01D12.3	0.296559107315832	AGIWUR38470
CB4856	CT	V	12387041	12395706	WBGene00006669	twk-14	K01D12.4	-0.0212529892567028	AGIWUR11110;AGIWUR24894;AGIWUR31992
CB4856	CT	V	12397845	12398783	WBGene00010464	K01D12.5	K01D12.5	-0.636026431740536	AGIWUR7580
CB4856	CT	V	12398759	12400859	WBGene00010465	K01D12.6	K01D12.6	-0.139517714091189	AGIWUR951;AGIWUR1257;AGIWUR19449;AGIWUR39885
CB4856	CT	V	12402655	12403234	WBGene00010467	K01D12.8	K01D12.8	-0.170842994283633	AGIWUR5069;AGIWUR9752;AGIWUR18695;AGIWUR44052
CB4856	CT	V	12403942	12404651	WBGene00010468	K01D12.9	K01D12.9	-0.588316659459363	AGIWUR35425
CB4856	CT	V	12406229	12407017	WBGene00010469	K01D12.10	K01D12.10	-0.181746704414331	AGIWUR443
CB4856	CT	V	12407428	12408592	WBGene00010470	cdr-4	K01D12.11	-0.79006359228301	AGIWUR3045
CB4856	CT	V	12409369	12410513	WBGene00010473	hrg-2	K01D12.14	0.0541546495873355	AGIWUR26565
CB4856	CT	V	12410879	12412091	WBGene00010472	cdr-7	K01D12.13	-0.830423326042176	AGIWUR13221
CB4856	CT	V	12412506	12413698	WBGene00010471	cdr-6	K01D12.12	-0.0911176424824554	AGIWUR43496
CB4856	CT	V	12414640	12415709	WBGene00008296	cdr-2	C54D10.1	-0.832647696825028	AGIWUR22071
CB4856	CT	V	12416358	12417377	WBGene00008297	cdr-3	C54D10.2	-0.117856228366703	AGIWUR40901
CB4856	CT	V	12418592	12419428	WBGene00008298	C54D10.3	C54D10.3	0.841214386724626	AGIWUR16125;AGIWUR29602;AGIWUR33587;AGIWUR35265;AGIWUR38616
CB4856	CT	V	12419492	12420944	WBGene00008299	C54D10.4	C54D10.4	0.123408526707049	AGIWUR12106
CB4856	CT	V	12429357	12431196	WBGene00008300	C54D10.5	C54D10.5	-0.031644003845984	AGIWUR5470
CB4856	CT	V	12431368	12432289	WBGene00045400	C54D10.13	C54D10.13	0.629595024788266	AGIWUR3457
CB4856	CT	V	12432365	12433817	WBGene00005249	srh-25	C54D10.6	0.245322026963217	AGIWUR17191
CB4856	CT	V	12434361	12436952	WBGene00044787	pals-36	C54D10.12		

0.0232556402823842	AGIWUR21228
CB4856	CT	V	12437499	12441266	WBGene00138721	pals-37	C54D10.14
-0.118169384721757	AGIWUR22417
CB4856	CT	V	12445184	12446956	WBGene00008302	pals-38	C54D10.8
1.07341678911439	AGIWUR44320
CB4856	CT	V	12447499	12448661	WBGene00008303	C54D10.9	C54D10.9
-0.141291677638881	AGIWUR2442
CB4856	CT	V	12448685	12450002	WBGene00008304	C54D10.10	C54D10.10
0.592342942035579	AGIWUR21029
CB4856	CT	V	12450807	12453336	WBGene00009200	F28C1.1	F28C1.1	-0.0576897483030194
AGIWUR34773
CB4856	CT	V	12453955	12463008	WBGene00001179	egl-10	F28C1.2	-0.251824599645179
AGIWUR5517;AGIWUR14567;AGIWUR19931;AGIWUR28668;AGIWUR29452;AGIWUR33579
CB4856	CT	V	12468683	12473533	WBGene00009201	F28C1.3	F28C1.3	0.0267182513961632
AGIWUR4004;AGIWUR7132;AGIWUR13049;AGIWUR17663;AGIWUR36408
CB4856	CT	V	12478413	12484442	WBGene00000228	atn-1	W04D2.1	0.014476504501169
AGIWUR26457;AGIWUR30926
CB4856	CT	V	12484885	12486068	WBGene00012242	srt-39	W04D2.2	0.0691368739656664
AGIWUR42463
CB4856	CT	V	12487266	12493264	WBGene00002133	inx-11	W04D2.3	-0.16419102578433
AGIWUR21702
CB4856	CT	V	12493239	12494622	WBGene00012244	mrps-11	W04D2.5	0.105892932795529
AGIWUR5065;AGIWUR13820;AGIWUR15166
CB4856	CT	V	12494728	12497755	WBGene00012243	W04D2.4	W04D2.4	-0.184792460998874
AGIWUR26542
CB4856	CT	V	12498213	12503738	WBGene00012245	W04D2.6	W04D2.6	0.0629847826854149
AGIWUR19864;AGIWUR37066
CB4856	CT	V	12505244	12506260	WBGene00012145	nlp-46	T28H10.4
-0.119054309288171	AGIWUR16141
CB4856	CT	V	12510645	12512201	WBGene00012143	T28H10.2	T28H10.2
-0.42410210789005	AGIWUR10112;AGIWUR28923;AGIWUR29056;AGIWUR36962
CB4856	CT	V	12512986	12514941	WBGene00012144	T28H10.3	T28H10.3
1.33742749830179	AGIWUR15495;AGIWUR21042;AGIWUR21714;AGIWUR33773;AGIWUR42383
CB4856	CT	V	12514875	12517205	WBGene00000285	cal-1	C13C12.1
0.373662547727575	AGIWUR31413
CB4856	CT	V	12523647	12525656	WBGene00007552	C13C12.2	C13C12.2
-0.0179814623413144	AGIWUR2397;AGIWUR13466;AGIWUR39611
CB4856	CT	V	12527058	12528023	WBGene00009721	F45D3.1	F45D3.1	-0.266716060157304
AGIWUR12145

CB4856 CT V 12541040 12542973 WBGene00009722 F45D3.2 F45D3.2 0.189128945431554
AGIWUR13978
CB4856 CT V 12549216 12550385 WBGene00009723 F45D3.3 F45D3.3 0.793351611577968
AGIWUR7531
CB4856 CT V 12552211 12553301 WBGene00009724 F45D3.4 F45D3.4 2.33864281759479
AGIWUR10584;AGIWUR22383
CB4856 CT V 12560710 12563383 WBGene00004759 sel-1 F45D3.5 -0.0334078786746312
AGIWUR12278;AGIWUR20293;AGIWUR29900
CB4856 CT V 12563487 12565785 WBGene00008452 mrps-5 E02A10.1
0.163115466458873 AGIWUR14293
CB4856 CT V 12566016 12567951 WBGene00008454 E02A10.4 E02A10.4
0.180790285722686 AGIWUR21249
CB4856 CT V 12575308 12576895 WBGene00001732 grl-23 E02A10.2
-0.0339071000892393 AGIWUR35126
CB4856 CT V 12583726 12587991 WBGene00008453 E02A10.3 E02A10.3
0.282658164796917 AGIWUR1496;AGIWUR3722;AGIWUR18897
CB4856 CT V 12588309 12589644 WBGene00007584 C14C10.1 C14C10.1
0.305010492578933 AGIWUR2794
CB4856 CT V 12589633 12590717 WBGene00007590 ttr-43 C14C10.7
0.096252716148058 AGIWUR29794
CB4856 CT V 12590979 12592477 WBGene00007585 C14C10.2 C14C10.2
-0.112802994576419 AGIWUR2163;AGIWUR9585;AGIWUR29812;AGIWUR36093;AGIWUR40434;AGIWUR44127
CB4856 CT V 12592568 12596296 WBGene00007587 mma-1 C14C10.4
-0.173030948034899 AGIWUR23046
CB4856 CT V 12596393 12599151 WBGene00007586 ril-2 C14C10.3
0.0223932086000529 AGIWUR6809;AGIWUR15074;AGIWUR31193;AGIWUR31739
CB4856 CT V 12600192 12607304 WBGene00007588 C14C10.5 C14C10.5
0.00146158840639199 AGIWUR13491;AGIWUR21045;AGIWUR34022;AGIWUR39851
CB4856 CT V 12607413 12608107 WBGene00007589 erg-28 C14C10.6
-0.0763102625689331 AGIWUR24094
CB4856 CT V 12609826 12610857 WBGene00013853 ZC116.1 ZC116.1 0.603078448651946
AGIWUR38261
CB4856 CT V 12611329 12628738 WBGene00013855 ZC116.3 ZC116.3 0.116491795170972
AGIWUR14;AGIWUR5873;AGIWUR10800;AGIWUR12287;AGIWUR13102;AGIWUR21054;AGIWUR30097;AGIWUR30478
CB4856 CT V 12628957 12629905 WBGene00044198 F56H9.6 F56H9.6 0.187320204463781
AGIWUR33770
CB4856 CT V 12630170 12632148 WBGene00006004 srx-113 F56H9.1 0.0370928034238518
AGIWUR6265;AGIWUR43399
CB4856 CT V 12632490 12633638 WBGene00050977 F56H9.7 F56H9.7 -0.0452816791205297

AGIWUR8350;AGIWUR11631;AGIWUR21197
CB4856 CT V 12637663 12638237 WBGene00010174 F56H9.2 F56H9.2 0.366595122535298
AGIWUR42483
CB4856 CT V 12638852 12640470 WBGene00001670 gpa-8 F56H9.3 0.203662766861533
AGIWUR36549
CB4856 CT V 12646827 12649313 WBGene00001671 gpa-9 F56H9.4 0.854566138612164
AGIWUR41400
CB4856 CT V 12649858 12658718 WBGene00003011 lin-25 F56H9.5 -0.0787970512583112
AGIWUR40170
CB4856 CT V 12658668 12659687 WBGene00012277 ccch-3 W05B10.2
-0.200410041270437 AGIWUR913;AGIWUR4527;AGIWUR37880;AGIWUR44491
CB4856 CT V 12659704 12660330 WBGene00012276 his-74 W05B10.1
-0.0073690206984125 AGIWUR21591
CB4856 CT V 12661304 12661659 WBGene00012278 W05B10.3 W05B10.3
-0.223376008181894
AGIWUR5588;AGIWUR11557;AGIWUR17691;AGIWUR29657;AGIWUR30731;AGIWUR31536;AGIWUR32212;AGIWUR34463;AGIWUR43549;
AGIWUR44193
CB4856 CT V 12668226 12669689 WBGene00012279 W05B10.4 W05B10.4
0.188573961459113 AGIWUR32489
CB4856 CT V 12670907 12672726 WBGene00006007 srx-116 W05B10.5
0.258048722511719 AGIWUR42124
CB4856 CT V 12680107 12692364 WBGene00010735 frpr-15 K10C8.2 0.0136884778230706
AGIWUR19256;AGIWUR21031
CB4856 CT V 12692486 12695255 WBGene00010734 parn-1 K10C8.1 -0.0782266071702322
AGIWUR5380;AGIWUR29696
CB4856 CT V 12696225 12696877 WBGene00010737 K10C8.4 K10C8.4 0.0619258342875346
AGIWUR3597
CB4856 CT V 12697794 12704273 WBGene00010736 istr-1 K10C8.3 0.139528586789115
AGIWUR17103;AGIWUR21085;AGIWUR33300;AGIWUR35232
CB4856 CT V 12704382 12707761 WBGene00011242 R11D1.1 R11D1.1 0.140801022651569
AGIWUR39860
CB4856 CT V 12708678 12710700 WBGene00011243 R11D1.2 R11D1.2 -0.102330775386368
AGIWUR7737
CB4856 CT V 12711293 12712109 WBGene00011244 R11D1.3 R11D1.3 0.244566643640496
AGIWUR19840
CB4856 CT V 12713035 12714220 WBGene00011245 R11D1.4 R11D1.4 -0.0710784692092501
AGIWUR9359
CB4856 CT V 12714503 12716549 WBGene00006222 str-178 R11D1.5 -0.120961974896656
AGIWUR3222

CB4856	CT	V	12716616	12718722	WBGene00006225	str-181	R11D1.6	-0.0560862721571134	AGIWUR5561
CB4856	CT	V	12720058	12722727	WBGene00011246	R11D1.7	R11D1.7	0.085009485879441	AGIWUR24869
CB4856	CT	V	12724562	12725993	WBGene00045336	R11D1.12	R11D1.12	0.0351609780095075	AGIWUR14180;AGIWUR14693;AGIWUR16055;AGIWUR24942;AGIWUR26376
CB4856	CT	V	12727171	12727621	WBGene00045517	R11D1.13	R11D1.13	0.00726249284570679	AGIWUR16530;AGIWUR29414;AGIWUR33362;AGIWUR36100
CB4856	CT	V	12727593	12728112	WBGene00004442	rpl-28	R11D1.8	-0.0935822788200085	AGIWUR32903;AGIWUR35759
CB4856	CT	V	12728631	12729416	WBGene00011247	mrpl-49	R11D1.9	-0.103015118876167	AGIWUR19366;AGIWUR34528;AGIWUR44439
CB4856	CT	V	12729520	12731801	WBGene00011248	R11D1.10	R11D1.10	-0.234787216827243	AGIWUR18000
CB4856	CT	V	12731772	12733645	WBGene00000984	dhs-21	R11D1.11	0.090538559560553	AGIWUR471;AGIWUR5236;AGIWUR10383
CB4856	CT	V	12734183	12736041	WBGene00007604	xbx-9	C15C8.1	0.0950445506822213	AGIWUR42554
CB4856	CT	V	12736703	12741211	WBGene00000521	cky-1	C15C8.2	0.172726972700596	AGIWUR14255;AGIWUR27971;AGIWUR29840
CB4856	CT	V	12742041	12744207	WBGene00007605	asp-10	C15C8.3	-0.416444378621296	AGIWUR21210
CB4856	CT	V	12745201	12747463	WBGene00007606	C15C8.4	C15C8.4	-0.33053547208508	AGIWUR16153;AGIWUR25403;AGIWUR26667
CB4856	CT	V	12748292	12749963	WBGene00007607	C15C8.5	C15C8.5	-0.0228526656152788	AGIWUR10751
CB4856	CT	V	12750030	12751545	WBGene00007608	C15C8.6	C15C8.6	-0.0954801199567603	AGIWUR26274
CB4856	CT	V	12751603	12752507	WBGene00045460	C15C8.8	C15C8.8	0.1079419013645	AGIWUR4939;AGIWUR8729;AGIWUR13587;AGIWUR39928;AGIWUR40646
CB4856	CT	V	12752762	12754893	WBGene00001675	gpa-13	F18E2.5	-0.20764925485279	AGIWUR35469
CB4856	CT	V	12757591	12760227	WBGene00008936	papl-1	F18E2.1	-0.77890784651602	AGIWUR19152
CB4856	CT	V	12761044	12763375	WBGene00006512	abcf-1	F18E2.2	-0.474195478923341	AGIWUR107;AGIWUR5368;AGIWUR41006;AGIWUR43678
CB4856	CT	V	12763945	12767933	WBGene00004738	scc-3	F18E2.3	-0.00526733751587116	AGIWUR20773
CB4856	CT	V	12769268	12770845	WBGene00008937	srt-43	F18E2.4	-0.126593998447898	

AGIWUR45016
CB4856 CT V 12773522 12783063 WBGene00013902 ZC455.1 ZC455.1 0.0124716468532888
AGIWUR5386;AGIWUR43768
CB4856 CT V 12783222 12784864 WBGene00013907 sre-20 ZC455.7 -0.168610920680518
AGIWUR19270
CB4856 CT V 12785154 12787126 WBGene00001429 fkb-4 ZC455.10
0.254868482940507 AGIWUR2842
CB4856 CT V 12787269 12788551 WBGene00013909 srbc-44 ZC455.9 -0.331663600905551
AGIWUR23977
CB4856 CT V 12789197 12790518 WBGene00013908 srbc-43 ZC455.8 -0.369842583537118
AGIWUR31402
CB4856 CT V 12790698 12792965 WBGene00013906 ugt-5 ZC455.6
-0.00274905250376722 AGIWUR26588;AGIWUR32844;AGIWUR35067;AGIWUR35571;AGIWUR42083;AGIWUR44576
CB4856 CT V 12793020 12795336 WBGene00013905 ugt-4 ZC455.5 0.136587415911571
AGIWUR17590;AGIWUR32025;AGIWUR37226;AGIWUR44616
CB4856 CT V 12795484 12798154 WBGene00013904 ugt-6 ZC455.4 -0.540819109179832
AGIWUR2287
CB4856 CT V 12798327 12800193 WBGene00013903 ugt-3 ZC455.3 -0.273908283252355
AGIWUR23933
CB4856 CT V 12803574 12804550 WBGene00013910 srbc-45 ZC455.11
0.102575710574634 AGIWUR5861;AGIWUR24206;AGIWUR34533;AGIWUR37594;AGIWUR41557
CB4856 CT V 12805149 12806128 WBGene00044102 srbc-46 ZC455.12
0.0660445131423533 AGIWUR5830
CB4856 CT V 12806490 12808277 WBGene00077562 ZC443.7 ZC443.7 0.128546435393605
AGIWUR3475;AGIWUR31113
CB4856 CT V 12808422 12810080 WBGene00013896 ZC443.1 ZC443.1 0.117165112675334
AGIWUR20062
CB4856 CT V 12812258 12814122 WBGene00013898 ZC443.3 ZC443.3 2.0462388767918
AGIWUR17607
CB4856 CT V 12816912 12819545 WBGene00013899 ZC443.4 ZC443.4 0.84098181859536
AGIWUR34599
CB4856 CT V 12820265 12822758 WBGene00013900 ugt-18 ZC443.5 3.68340337150669
AGIWUR10265
CB4856 CT V 12825024 12827432 WBGene00013901 ugt-16 ZC443.6 -0.0675100311087604
AGIWUR4668
CB4856 CT V 12827770 12830109 WBGene00008097 ugt-15 C44H9.1 0.289901493730828
AGIWUR39382
CB4856 CT V 12830804 12834438 WBGene00008098 C44H9.2 C44H9.2 0.0998523396873026
AGIWUR13206

CB4856	CT	V	12835708	12839783	WBGene00008099	C44H9.4	C44H9.4	-0.042356755928342	AGIWUR41365
CB4856	CT	V	12842494	12845330	WBGene00008101	C44H9.6	C44H9.6	0.160389340935941	AGIWUR44468
CB4856	CT	V	12846721	12849864	WBGene00008100	C44H9.5	C44H9.5	0.0689460755775775	AGIWUR12662;AGIWUR43467
CB4856	CT	V	12853015	12854839	WBGene00008102	C44H9.7	C44H9.7	0.925090239812281	AGIWUR18702;AGIWUR19111;AGIWUR29062
CB4856	CT	V	12859381	12863833	WBGene00011587	T07F10.1	T07F10.1	0.201686471636442	AGIWUR12497
CB4856	CT	V	12868026	12870035	WBGene00011589	T07F10.3	T07F10.3	-0.0181423889337671	AGIWUR5418;AGIWUR8235;AGIWUR9659;AGIWUR10901;AGIWUR13177;AGIWUR33700
CB4856	CT	V	12872053	12882108	WBGene00011590	bus-19	T07F10.4	0.0396018356019709	AGIWUR573;AGIWUR18516;AGIWUR42616
CB4856	CT	V	12882078	12885960	WBGene00011287	glb-24	R90.5	0.0241777032187862	AGIWUR12824;AGIWUR18804;AGIWUR38495
CB4856	CT	V	12891523	12895872	WBGene00011283	R90.1	R90.1	0.180773619775285	AGIWUR14152;AGIWUR38711
CB4856	CT	V	12903551	12904160	WBGene00011284	ttr-27	R90.2	-0.330788106268077	AGIWUR18386
CB4856	CT	V	12904213	12905168	WBGene00011285	ttr-28	R90.3	-0.124738651146616	AGIWUR1453
CB4856	CT	V	12906131	12906746	WBGene00011286	ttr-29	R90.4	0.40951404250143	AGIWUR25178
CB4856	CT	V	12910113	12917198	WBGene00003738	nid-1	F54F3.1	-0.149234046496746	AGIWUR44234
CB4856	CT	V	12917450	12918902	WBGene00014770	F54F3.2	F54F3.2	0.926758211262598	AGIWUR20453;AGIWUR24291
CB4856	CT	V	12919952	12921692	WBGene00010062	lipl-1	F54F3.3	1.91177584376519	AGIWUR44387
CB4856	CT	V	12922608	12924156	WBGene00010063	dhrs-4	F54F3.4	0.184505646270573	AGIWUR2741
CB4856	CT	V	12924441	12928173	WBGene00004150	pqn-67	T16G1.1	-0.154523121694684	AGIWUR806;AGIWUR25408;AGIWUR26872
CB4856	CT	V	12929478	12932966	WBGene00011796	T16G1.2	T16G1.2	-0.0726628758557339	AGIWUR33
CB4856	CT	V	12932941	12935112	WBGene00011801	T16G1.7	T16G1.7	0.51169742288174	AGIWUR18524
CB4856	CT	V	12935538	12937263	WBGene00011800	T16G1.6	T16G1.6	0.097623283450428	

AGIWUR22206;AGIWUR22875;AGIWUR25143
CB4856 CT V 12937654 12939506 WBGene00011799 T16G1.5 T16G1.5 -0.0926262948213594
AGIWUR22764
CB4856 CT V 12940038 12941847 WBGene00011798 T16G1.4 T16G1.4 3.24127025370183
AGIWUR4039;AGIWUR26972;AGIWUR30778
CB4856 CT V 12942365 12944329 WBGene00011797 T16G1.3 T16G1.3
-0.00166920277668581 AGIWUR9650
CB4856 CT V 12944861 12946853 WBGene00001711 grl-2 T16G1.8 0.346755757131124
AGIWUR39625
CB4856 CT V 12946815 12950658 WBGene00011802 T16G1.9 T16G1.9 -0.0905700683537063
AGIWUR42413
CB4856 CT V 12952636 12953208 WBGene00004151 pqn-68 T16G1.10
-0.379793743427086 AGIWUR24781
CB4856 CT V 12954002 12955229 WBGene00001233 eif-3.K T16G1.11
-0.277936019492895 AGIWUR12786;AGIWUR16029;AGIWUR16352
CB4856 CT V 12955203 12961149 WBGene00011307 mpst-7 R186.6 -0.326467815587892
AGIWUR36157
CB4856 CT V 12961474 12963336 WBGene00011305 R186.1 R186.1 0.158696086475546
AGIWUR27464
CB4856 CT V 12964376 12966022 WBGene00005113 srd-35 R186.2 0.183616315339947
AGIWUR7784;AGIWUR33653;AGIWUR35769;AGIWUR44051;AGIWUR45110
CB4856 CT V 12966000 12967210 WBGene00011306 R186.3 R186.3 0.0886511609313843
AGIWUR12413
CB4856 CT V 12967309 12967982 WBGene00011309 R186.8 R186.8 -0.119839594925099
AGIWUR8865;AGIWUR11438;AGIWUR14545;AGIWUR19202;AGIWUR28961;AGIWUR29160;AGIWUR36476;AGIWUR40001;AGIWUR43571
CB4856 CT V 12968855 12970730 WBGene00003031 lin-46 R186.4 -0.128312219130443
AGIWUR25766
CB4856 CT V 12971110 12973282 WBGene00011308 R186.7 R186.7
-0.00157803988034289 AGIWUR34982
CB4856 CT V 12973261 12976704 WBGene00004793 shw-3 R186.5 0.119377393065163
AGIWUR30708
CB4856 CT V 12988543 12992185 WBGene00008851 F15B9.8 F15B9.8 0.23570478483556
AGIWUR9286;AGIWUR19700;AGIWUR20843
CB4856 CT V 12993180 12994822 WBGene00001109 dsl-7 F15B9.9 0.268382398190755
AGIWUR34207
CB4856 CT V 12995569 12996836 WBGene00001389 far-5 F15B9.3 -0.277372031463957
AGIWUR5382;AGIWUR7263;AGIWUR41491;AGIWUR45201
CB4856 CT V 12996787 12997524 WBGene00001387 far-3 F15B9.1 0.553284127354939
AGIWUR34379

CB4856	CT	V	12998427	12999659	WBGene00001388	far-4	F15B9.2	-0.202227624486463
AGIWUR39090
CB4856	CT	V	13002317	13006987	WBGene00008848	inft-2	F15B9.4	0.149818208836146
AGIWUR36918
CB4856	CT	V	13006981	13008925	WBGene00045418	F15B9.10	F15B9.10
0.104388338700998	AGIWUR7185
CB4856	CT	V	13014144	13016641	WBGene00008850	F15B9.6	F15B9.6	0.0131948619887972
AGIWUR13550
CB4856	CT	V	13019068	13033373	WBGene00001475	fmi-1	F15B9.7	0.316776695546832
AGIWUR21662;AGIWUR42470;AGIWUR43120
CB4856	CT	V	13033529	13034164	WBGene00012335	W07G4.1	W07G4.1	0.382538037528984
AGIWUR6064;AGIWUR28273
CB4856	CT	V	13034267	13034704	WBGene00077565	W07G4.7	W07G4.7	0.84612799781834
AGIWUR11268
CB4856	CT	V	13036996	13041879	WBGene00012337	W07G4.3	W07G4.3	-0.132224258376816
AGIWUR1071;AGIWUR12298;AGIWUR14469;AGIWUR14495;AGIWUR31800;AGIWUR32655;AGIWUR43836
CB4856	CT	V	13043712	13046355	WBGene00002250	lap-2	W07G4.4	-0.335075208120179
AGIWUR38316
CB4856	CT	V	13047980	13049238	WBGene00012339	W07G4.5	W07G4.5	0.569375072234067
AGIWUR3319
CB4856	CT	V	13049730	13053910	WBGene00005652	srr-1	W07G4.6	-0.0200549416360651
AGIWUR30904
CB4856	CT	V	13065461	13065794	WBGene00009631	F42E8.2	F42E8.2	0.1280417636358
AGIWUR41554
CB4856	CT	V	13066640	13071152	WBGene00007908	C34B4.3	C34B4.3	0.726484032099086
AGIWUR9410;AGIWUR40112
CB4856	CT	V	13083055	13090260	WBGene00007907	C34B4.2	C34B4.2	0.113320927542716
AGIWUR14470;AGIWUR23901;AGIWUR38007
CB4856	CT	V	13089803	13092512	WBGene00007909	C34B4.4	C34B4.4	-0.124147341653158
AGIWUR26382;AGIWUR37670
CB4856	CT	V	13092666	13094446	WBGene00077698	lurp-4	C34B4.7	0.155442904469639
AGIWUR5631;AGIWUR11097
CB4856	CT	V	13095954	13097457	WBGene00007910	C34B4.5	C34B4.5	0.252942871201867
AGIWUR38581
CB4856	CT	V	13098910	13110337	WBGene00003143	max-1	C34B4.1	0.350272216957874
AGIWUR39031
CB4856	CT	V	13116488	13120167	WBGene00007150	acly-2	B0365.1	-0.0684989637173441
AGIWUR4217
CB4856	CT	V	13120676	13123421	WBGene00007151	B0365.2	B0365.2	-0.0299455604460839

AGIWUR14820
CB4856 CT V 13126414 13129995 WBGene00001137 eat-6 B0365.3 -0.300566817846185
AGIWUR190;AGIWUR311;AGIWUR2417;AGIWUR3481;AGIWUR3982;AGIWUR25633;AGIWUR27717;AGIWUR33762
CB4856 CT V 13131873 13133565 WBGene00219208 B0365.9 B0365.9 -0.155439221115898
AGIWUR34951
CB4856 CT V 13134965 13138101 WBGene00007153 clec-41 B0365.6 -0.359775337024173
AGIWUR3662;AGIWUR18488;AGIWUR26592;AGIWUR36559;AGIWUR44784
CB4856 CT V 13138981 13152466 WBGene00007154 dhc-3 B0365.7 -0.0801283386824885
AGIWUR24444;AGIWUR29005
CB4856 CT V 13152894 13176111 WBGene00011647 noca-1 T09E8.1 0.0540569166308611
AGIWUR2137;AGIWUR2843;AGIWUR4645;AGIWUR19230;AGIWUR24927;AGIWUR34375;AGIWUR42573;AGIWUR43631
CB4856 CT V 13176711 13180189 WBGene00001874 him-17 T09E8.2
-0.00067399705776694 AGIWUR42908
CB4856 CT V 13180330 13181534 WBGene00011648 cni-1 T09E8.3 -0.350846760740976
AGIWUR7422;AGIWUR16265;AGIWUR18132;AGIWUR37833
CB4856 CT V 13181580 13184237 WBGene00011649 T09E8.4 T09E8.4 -0.0441851413150545
AGIWUR146
CB4856 CT V 13185684 13187428 WBGene00005518 sri-6 T09E8.5 0.183725404872147
AGIWUR29710
CB4856 CT V 13188025 13189406 WBGene00044199 F57B1.8 F57B1.8 0.00387929495000705
AGIWUR1263;AGIWUR10046;AGIWUR29953
CB4856 CT V 13191707 13193538 WBGene00006311 sun-1 F57B1.2 -0.0814358429719118
AGIWUR36486
CB4856 CT V 13193933 13195648 WBGene00010187 F57B1.1 F57B1.1 -0.198766447668509
AGIWUR12797;AGIWUR18184;AGIWUR21634;AGIWUR24778;AGIWUR26707;AGIWUR39169
CB4856 CT V 13196088 13197923 WBGene00045488 F57B1.9 F57B1.9 -0.304605511641461
AGIWUR11389;AGIWUR18594;AGIWUR29360;AGIWUR32520;AGIWUR36306;AGIWUR38732
CB4856 CT V 13200982 13202192 WBGene00000733 col-160 F57B1.4 -0.153393028490427
AGIWUR7499
CB4856 CT V 13207763 13208762 WBGene00010188 F57B1.5 F57B1.5 -0.109081189476981
AGIWUR35756
CB4856 CT V 13209645 13213462 WBGene00010189 F57B1.6 F57B1.6 0.583478157257977
AGIWUR3188
CB4856 CT V 13222620 13225941 WBGene00007929 dmd-10 C34D1.1 0.109087145426656
AGIWUR28168;AGIWUR36450
CB4856 CT V 13236171 13236604 WBGene00007930 dmd-11 C34D1.2 -0.0426467623260258
AGIWUR25121
CB4856 CT V 13246591 13249536 WBGene00003850 odr-3 C34D1.3 0.0842050208777998
AGIWUR520

CB4856	CT	V	13257097	13259311	WBGene00007931	C34D1.4	C34D1.4	0.0298155036966676	AGIWUR26252
CB4856	CT	V	13264546	13269820	WBGene00007932	zip-5	C34D1.5	0.218542453305854	AGIWUR12576
CB4856	CT	V	13273153	13295978	WBGene00009337	uig-1	F32F2.1	0.344361544330635	AGIWUR6427;AGIWUR10081;AGIWUR10403;AGIWUR16513;AGIWUR18628;AGIWUR19651;AGIWUR21862;AGIWUR43746
CB4856	CT	V	13297520	13298050	WBGene00007747	C26E1.1	C26E1.1	0.23906330457707	AGIWUR35253
CB4856	CT	V	13301652	13304785	WBGene00007749	ceh-79	C26E1.3	0.42291020981863	AGIWUR27287
CB4856	CT	V	13305138	13307271	WBGene00007748	C26E1.2	C26E1.2	1.30792707195846	AGIWUR24177
CB4856	CT	V	13308916	13309423	WBGene00008217	C50B6.1	C50B6.1	0.0373656202648869	AGIWUR2180;AGIWUR7650;AGIWUR34812;AGIWUR42323
CB4856	CT	V	13310186	13312260	WBGene00008218	nasp-2	C50B6.2	-0.0852343784927001	AGIWUR1780
CB4856	CT	V	13313575	13315812	WBGene00008219	C50B6.3	C50B6.3	0.0946601346143936	AGIWUR28232
CB4856	CT	V	13318464	13320080	WBGene00005243	srh-18	C50B6.5	-0.00406474693167702	AGIWUR26672
CB4856	CT	V	13320984	13322355	WBGene00005240	srh-15	C50B6.6	0.0245695553435716	AGIWUR9319
CB4856	CT	V	13322369	13324979	WBGene00008220	C50B6.7	C50B6.7	0.406301726782699	AGIWUR37223
CB4856	CT	V	13326339	13327634	WBGene00014710	C50B6.13	C50B6.13	-0.198492683949027	AGIWUR9436;AGIWUR21227
CB4856	CT	V	13327763	13328866	WBGene00006102	str-37	C50B6.12	-0.00823926868029368	AGIWUR41798
CB4856	CT	V	13329193	13331019	WBGene00003719	nhr-129	C50B6.14	0.25747983632411	AGIWUR41684
CB4856	CT	V	13331117	13333871	WBGene00008221	nhr-168	C50B6.8	0.656937933475101	AGIWUR18922
CB4856	CT	V	13336369	13337957	WBGene00008222	C50B6.9	C50B6.9	0.206582156071519	AGIWUR40384
CB4856	CT	V	13337991	13339718	WBGene00006072	str-4	C50B6.10	0.0111717795531456	AGIWUR7313;AGIWUR29755;AGIWUR35114
CB4856	CT	V	13340240	13343040	WBGene00008223	lgc-48	C50B6.11	-0.163046178572229	AGIWUR4680
CB4856	CT	V	13346278	13356101	WBGene00006693	twk-43	F32H5.7	-0.0478499022023442	

AGIWUR1564;AGIWUR6551;AGIWUR16179;AGIWUR26578;AGIWUR32931;AGIWUR33454;AGIWUR35141;AGIWUR37747;AGIWUR40696
CB4856	CT	V	13356101	13358079	WBGene00009347	F32H5.1	F32H5.1	0.183422402239058	AGIWUR3189
CB4856	CT	V	13359334	13360024	WBGene00009349	F32H5.3	F32H5.3	-0.188131244057596	AGIWUR33526;AGIWUR33920
CB4856	CT	V	13361203	13363357	WBGene00009350	F32H5.4	F32H5.4	-0.308614942772465	AGIWUR693;AGIWUR24348;AGIWUR36416
CB4856	CT	V	13363715	13364728	WBGene00005203	srg-46	F32H5.5	0.088018914754727	AGIWUR23784
CB4856	CT	V	13367252	13369990	WBGene00006016	srx-125	F32H5.6	0.0332354548398026	AGIWUR7412;AGIWUR9295;AGIWUR12807;AGIWUR17136;AGIWUR28275;AGIWUR43239
CB4856	CT	V	13370083	13380262	WBGene00010867	tag-260	M04G12.1	0.138629959929983	AGIWUR22793
CB4856	CT	V	13381189	13383077	WBGene00000789	cpz-2	M04G12.2	0.165877573606608	AGIWUR646;AGIWUR7308;AGIWUR35528
CB4856	CT	V	13383019	13385898	WBGene00001554	gcy-34	M04G12.3	0.290128351208481	AGIWUR44723
CB4856	CT	V	13386065	13391393	WBGene00010868	somi-1	M04G12.4	0.788014769452336	AGIWUR33719;AGIWUR38576
CB4856	CT	V	13404031	13406014	WBGene00009979	cutl-1	F53F1.1	-0.183766004887135	AGIWUR27799
CB4856	CT	V	13406816	13408265	WBGene00009980	F53F1.2	F53F1.2	-0.533893477854178	AGIWUR5471
CB4856	CT	V	13408242	13409832	WBGene00009981	F53F1.3	F53F1.3	-0.171853114035413	AGIWUR392
CB4856	CT	V	13411077	13411717	WBGene00009982	F53F1.4	F53F1.4	-0.455507165347237	AGIWUR6086;AGIWUR23571
CB4856	CT	V	13414902	13416121	WBGene00009983	cut-2	F53F1.5	0.0442805058287039	AGIWUR19257
CB4856	CT	V	13416103	13417604	WBGene00009984	F53F1.6	F53F1.6	0.444451925757956	AGIWUR40851
CB4856	CT	V	13419217	13420740	WBGene00005719	srv-8	F53F1.7	-0.36627326247465	AGIWUR23144
CB4856	CT	V	13421140	13423147	WBGene00005720	srv-9	F53F1.8	0.221905533644158	AGIWUR31461
CB4856	CT	V	13423908	13425266	WBGene00005721	srv-10	F53F1.9	0.276969108468879	AGIWUR11420
CB4856	CT	V	13425572	13426882	WBGene00005096	srd-18	F53F1.10	0.161706833203905	AGIWUR9433

CB4856 CT V 13430651 13432714 WBGene00005095 srd-17 Y2H9A.2 0.199019743237832
AGIWUR19936;AGIWUR39164
CB4856 CT V 13433050 13437545 WBGene00003222 mes-4 Y2H9A.1 0.0335868522694334
AGIWUR3310;AGIWUR3946;AGIWUR40132
CB4856 CT V 13445745 13448815 WBGene00012381 Y2H9A.4 Y2H9A.4 -0.214612894217177
AGIWUR16927
CB4856 CT V 13451197 13452968 WBGene00014744 F25H9.9 F25H9.9 -0.11641144461843
AGIWUR18861;AGIWUR19729
CB4856 CT V 13453634 13455447 WBGene00009134 lurp-2 F25H9.1 0.092608233092669
AGIWUR10925
CB4856 CT V 13455748 13457506 WBGene00009136 lurp-3 F25H9.3 0.026405737926833
AGIWUR12404
CB4856 CT V 13458083 13459504 WBGene00009135 F25H9.2 F25H9.2 -0.12771840151621
AGIWUR44437
CB4856 CT V 13459525 13461803 WBGene00009137 bath-39 F25H9.4 -0.250360392581092
AGIWUR26645;AGIWUR30359;AGIWUR38033
CB4856 CT V 13463128 13467131 WBGene00001496 frm-10 F25H9.5 -0.114831628455006
AGIWUR33216
CB4856 CT V 13466924 13468361 WBGene00009138 F25H9.6 F25H9.6 0.250293803090159
AGIWUR32693
CB4856 CT V 13468376 13469087 WBGene00009139 F25H9.7 F25H9.7 0.0204174739072727
AGIWUR43654
CB4856 CT V 13469066 13473679 WBGene00011692 T10G3.1 T10G3.1 1.0287581427539
AGIWUR7241
CB4856 CT V 13474228 13476382 WBGene00045340 T10G3.8 T10G3.8 0.190923756103312
AGIWUR14677;AGIWUR41060
CB4856 CT V 13477384 13479858 WBGene00011693 T10G3.2 T10G3.2 -0.0473435434215026
AGIWUR8086;AGIWUR22954
CB4856 CT V 13482075 13484435 WBGene00011695 T10G3.4 T10G3.4 0.0863167620257244
AGIWUR35537
CB4856 CT V 13487026 13489626 WBGene00011694 T10G3.3 T10G3.3 -0.77135050345205
AGIWUR26800
CB4856 CT V 13491068 13497372 WBGene00011696 eea-1 T10G3.5 -0.147436846072318
AGIWUR8015;AGIWUR12733;AGIWUR12749;AGIWUR19148;AGIWUR20934;AGIWUR24622;AGIWUR29618;AGIWUR33391
CB4856 CT V 13497690 13498819 WBGene00001808 gut-2 T10G3.6 -0.259201156848772
AGIWUR7892;AGIWUR22967;AGIWUR32644;AGIWUR40313;AGIWUR43259;AGIWUR44390
CB4856 CT V 13498786 13511718 WBGene00000233 avr-15 R11G10.1
-0.0351036227592303 AGIWUR30125;AGIWUR36531;AGIWUR42208
CB4856 CT V 13511905 13515215 WBGene00011249 R11G10.2 R11G10.2

								0.0135019873668192	AGIWUR5998;AGIWUR9152;AGIWUR37805
CB4856	CT	V	13523530	13529658	WBGene00044045	R11G10.3	R11G10.3	0.146756713589316	AGIWUR15842;AGIWUR24465
CB4856	CT	V	13529562	13530744	WBGene00008341	ttr-44	C56A3.2	0.133262555420091	AGIWUR16445
CB4856	CT	V	13531743	13536105	WBGene00008342	frpr-5	C56A3.3	-0.183959692317238	AGIWUR41300
CB4856	CT	V	13550520	13551810	WBGene00008343	C56A3.4	C56A3.4	-0.066710273989413	AGIWUR30441;AGIWUR34820;AGIWUR37682;AGIWUR38750
CB4856	CT	V	13551911	13553373	WBGene00008344	C56A3.5	C56A3.5	-0.139989238557259	AGIWUR15348
CB4856	CT	V	13553374	13556408	WBGene00008345	C56A3.6	C56A3.6	0.133608328643401	AGIWUR25755
CB4856	CT	V	13557104	13559922	WBGene00000302	cav-2	C56A3.7	-0.0709133258503218	AGIWUR36224
CB4856	CT	V	13560278	13561992	WBGene00008346	C56A3.8	C56A3.8	-0.0191124375893192	AGIWUR2902;AGIWUR12612;AGIWUR22047
CB4856	CT	V	13562105	13563913	WBGene00001726	grl-17	C56A3.1	0.101636746774773	AGIWUR20018
CB4856	CT	V	13565863	13566968	WBGene00008224	C50B8.1	C50B8.1	-0.0266753718140169	AGIWUR40609
CB4856	CT	V	13566931	13568657	WBGene00008225	nuaf-1	C50B8.3	0.136356183015003	AGIWUR31957
CB4856	CT	V	13568850	13570030	WBGene00000250	bir-2	C50B8.2	0.523117829580364	AGIWUR40461
CB4856	CT	V	13572037	13573423	WBGene00008226	C50B8.4	C50B8.4	1.05014661869002	AGIWUR13777
CB4856	CT	V	13578972	13580807	WBGene00008228	C50B8.6	C50B8.6	-0.0708087040054419	AGIWUR10703
CB4856	CT	V	13580993	13584643	WBGene00008227	C50B8.5	C50B8.5	-0.113978685257948	AGIWUR40816
CB4856	CT	V	13584903	13585369	WBGene00009985	F53F4.1	F53F4.1	-0.269240826801713	AGIWUR26165
CB4856	CT	V	13588206	13588479	WBGene00045195	F53F4.17	F53F4.17	0.141156495103161	AGIWUR30395
CB4856	CT	V	13588612	13589585	WBGene00009986	F53F4.2	F53F4.2	0.154863550206281	AGIWUR10582
CB4856	CT	V	13592124	13593183	WBGene00009987	tbcb-1	F53F4.3	-0.128006318833568	AGIWUR11278;AGIWUR24957;AGIWUR31578;AGIWUR38807

CB4856	CT	V	13593383	13598269	WBGene00009988	F53F4.4	F53F4.4	0.076338738106374	AGIWUR5440;AGIWUR29243;AGIWUR30894
CB4856	CT	V	13598331	13600982	WBGene00004328	rdy-2	F53F4.6	0.196487921803099	AGIWUR10827
CB4856	CT	V	13603276	13606701	WBGene00001479	fmo-4	F53F4.5	0.146097864721188	AGIWUR8267;AGIWUR11593;AGIWUR14457;AGIWUR15870;AGIWUR23166;AGIWUR32253
CB4856	CT	V	13611337	13611888	WBGene00009995	F53F4.13	F53F4.13	0.301659892037273	AGIWUR23892
CB4856	CT	V	13612439	13613979	WBGene00009990	F53F4.7	F53F4.7	0.00914701241476568	AGIWUR7953;AGIWUR24081;AGIWUR27468;AGIWUR28108;AGIWUR31833;AGIWUR33916
CB4856	CT	V	13614236	13617723	WBGene00009991	F53F4.8	F53F4.8	0.443578629587844	AGIWUR14887
CB4856	CT	V	13617851	13619686	WBGene00005089	srd-11	F53F4.9	0.0244455191373074	AGIWUR30493
CB4856	CT	V	13620976	13621459	WBGene00009997	F53F4.15	F53F4.15	0.0603916411307548	AGIWUR2105
CB4856	CT	V	13621371	13621820	WBGene00023419	F53F4.16	F53F4.16	-0.139053581712536	AGIWUR33852;AGIWUR39333
CB4856	CT	V	13622107	13624525	WBGene00009993	F53F4.11	F53F4.11	-0.156575487550406	AGIWUR5780
CB4856	CT	V	13626031	13627482	WBGene00009994	F53F4.12	F53F4.12	0.272382295133104	AGIWUR9467
CB4856	CT	V	13627573	13629831	WBGene00009996	F53F4.14	F53F4.14	0.424668144207253	AGIWUR9184
CB4856	CT	V	13629798	13634220	WBGene00010426	H37A05.2	H37A05.2	0.39493247367361	AGIWUR38170
CB4856	CT	V	13646884	13648397	WBGene00044200	H37A05.4	H37A05.4	0.0897041668897911	AGIWUR575;AGIWUR1900;AGIWUR15836;AGIWUR21231;AGIWUR35946;AGIWUR38688;AGIWUR44826
CB4856	CT	V	13650262	13654129	WBGene00010425	lpin-1	H37A05.1	-0.0873928225840501	AGIWUR2643;AGIWUR4507;AGIWUR9267;AGIWUR16504;AGIWUR18966;AGIWUR23328;AGIWUR29667;AGIWUR30580;AGIWUR31663
CB4856	CT	V	13661261	13664233	WBGene00010279	letm-1	F58G11.1	-0.103885785065216	AGIWUR2941
CB4856	CT	V	13665397	13669292	WBGene00010280	rde-12	F58G11.2	-0.0796947660429339	AGIWUR4244;AGIWUR13280;AGIWUR18784;AGIWUR25411;AGIWUR28193;AGIWUR32694;AGIWUR43200
CB4856	CT	V	13669353	13671958	WBGene00010281	F58G11.3	F58G11.3	-0.202121594383726	AGIWUR7764
CB4856	CT	V	13672597	13676024	WBGene00010282	F58G11.4	F58G11.4	0.800720025306305	AGIWUR45194

CB4856 CT V 13675988 13678598 WBGene00006442 tag-65 F58G11.5
0.0469041418097191 AGIWUR25787;AGIWUR34042;AGIWUR42886
CB4856 CT V 13678875 13681539 WBGene00010283 ccz-1 F58G11.6
-0.0510444914101917 AGIWUR38090
CB4856 CT V 13689892 13703330 WBGene00011326 T01D3.1 T01D3.1 0.0629052829958461
AGIWUR6558;AGIWUR11831;AGIWUR40023
CB4856 CT V 13704929 13711085 WBGene00011328 T01D3.3 T01D3.3 1.01808085717285
AGIWUR15077;AGIWUR42302
CB4856 CT V 13712854 13716624 WBGene00011329 zipt-9 T01D3.5 -0.292285959338869
AGIWUR16842;AGIWUR19607
CB4856 CT V 13716824 13720402 WBGene00011330 T01D3.6 T01D3.6 0.0791576967123993
AGIWUR15921;AGIWUR25854;AGIWUR27027;AGIWUR29108;AGIWUR29892;AGIWUR30923
CB4856 CT V 13721249 13722196 WBGene00009425 scl-16 F35E12.1
-0.167521476398926 AGIWUR6100
CB4856 CT V 13722254 13724881 WBGene00009426 F35E12.2 F35E12.2
-0.253896058866559 AGIWUR16340
CB4856 CT V 13725905 13727014 WBGene00009427 fbxa-181 F35E12.3
-0.224467444248178 AGIWUR28078
CB4856 CT V 13727763 13730776 WBGene00009431 dct-17 F35E12.7
-0.689894594044599 AGIWUR32743
CB4856 CT V 13731506 13733387 WBGene00009428 F35E12.4 F35E12.4
0.1741122026546 AGIWUR23886
CB4856 CT V 13734118 13736247 WBGene00009430 F35E12.6 F35E12.6
-0.570202069359222 AGIWUR2036
CB4856 CT V 13737007 13738474 WBGene00009429 irg-5 F35E12.5
-0.130048552879746 AGIWUR30275
CB4856 CT V 13741321 13745127 WBGene00009433 F35E12.9 F35E12.9
-0.580484667230244 AGIWUR35609;AGIWUR36025
CB4856 CT V 13745696 13748758 WBGene00009434 F35E12.10 F35E12.10
-0.35637065499338 AGIWUR6787
CB4856 CT V 13749181 13751972 WBGene00009432 cld-9 F35E12.8
-0.46865810778003 AGIWUR25822
CB4856 CT V 13755011 13756409 WBGene00008986 F20G2.2 F20G2.2 -0.0634750573436627
AGIWUR38701
CB4856 CT V 13758090 13759945 WBGene00044920 F20G2.7 F20G2.7 -0.2044911404975
AGIWUR6299;AGIWUR17995;AGIWUR22254;AGIWUR33333;AGIWUR37779;AGIWUR44262
CB4856 CT V 13763077 13765980 WBGene00008989 F20G2.6 F20G2.6 0.184885539879824
AGIWUR30925
CB4856 CT V 13765926 13768552 WBGene00008987 F20G2.3 F20G2.3

-0.00603700048756295	AGIWUR23110
CB4856	CT	V	13769669	13771279	WBGene00003543	nas-24	F20G2.4	-0.163732687017362
AGIWUR9822
CB4856	CT	V	13771508	13773695	WBGene00008988	F20G2.5	F20G2.5	0.218747225998198
AGIWUR967;AGIWUR2052;AGIWUR2758;AGIWUR5251;AGIWUR8818;AGIWUR31389;AGIWUR32219;AGIWUR33248;AGIWUR34200;AGIWUR38036
CB4856	CT	V	13775751	13777767	WBGene00009971	F53C11.1	F53C11.1
-0.403935802004808	AGIWUR41401
CB4856	CT	V	13778015	13778592	WBGene00044921	F53C11.9	F53C11.9
0.655245383248559	AGIWUR658;AGIWUR7729;AGIWUR17506
CB4856	CT	V	13778633	13781625	WBGene00009972	oac-33	F53C11.2
0.0280766466886524	AGIWUR43622
CB4856	CT	V	13782399	13784102	WBGene00009973	F53C11.3	F53C11.3
0.38103226249414	AGIWUR21156
CB4856	CT	V	13784095	13787712	WBGene00009974	F53C11.4	F53C11.4
0.0318271334148168	AGIWUR6856;AGIWUR16078;AGIWUR17928
CB4856	CT	V	13787536	13796254	WBGene00009975	F53C11.5	F53C11.5
0.110233122257315	AGIWUR14374
CB4856	CT	V	13796641	13800577	WBGene00006684	twk-32	F53C11.6
0.331646490732084	AGIWUR19031
CB4856	CT	V	13800976	13804282	WBGene00009976	swan-2	F53C11.7
0.0181844936241617	AGIWUR2857
CB4856	CT	V	13804395	13807237	WBGene00009977	swan-1	F53C11.8
0.00850048772562451	AGIWUR2829;AGIWUR14332;AGIWUR14646;AGIWUR26673;AGIWUR34285;AGIWUR41780
CB4856	CT	V	13813309	13815272	WBGene00000447	ceh-24	F55B12.1
0.0856192503459576	AGIWUR29993
CB4856	CT	V	13815374	13816977	WBGene00010088	F55B12.2	F55B12.2
0.0909577659548715	AGIWUR6477
CB4856	CT	V	13818132	13822980	WBGene00004767	sel-10	F55B12.3
-0.170357044733041	AGIWUR41;AGIWUR9842;AGIWUR13814;AGIWUR27608;AGIWUR34431;AGIWUR37471;AGIWUR39533
CB4856	CT	V	13823052	13825222	WBGene00010089	hpo-31	F55B12.4
-0.0477856279801543	AGIWUR17921;AGIWUR28404;AGIWUR28902;AGIWUR39706
CB4856	CT	V	13828208	13831040	WBGene00003812	nrf-5	F55B12.5
-0.193714944003922	AGIWUR14186
CB4856	CT	V	13831113	13832905	WBGene00006210	str-165	F55B12.6
-0.180029319145044	AGIWUR23102
CB4856	CT	V	13834251	13836581	WBGene00006020	srx-129	F55B12.9
-0.186953392736729	AGIWUR19811;AGIWUR37331
CB4856	CT	V	13837473	13838682	WBGene00006019	srx-128	F55B12.7

-0.0721831775389505	AGIWUR22941
CB4856	CT	V	13839769	13840281	WBGene00077439	F55B12.10	F55B12.10
0.510876038495692	AGIWUR14927;AGIWUR31593;AGIWUR32913;AGIWUR36567;AGIWUR37092
CB4856	CT	V	13840741	13842187	WBGene00005908	srx-17	F55B12.8
0.00937774582405915	AGIWUR16351
CB4856	CT	V	13843447	13844962	WBGene00006021	srx-130	F09F3.1	0.219712348690078
AGIWUR12608;AGIWUR12670;AGIWUR20875;AGIWUR44651
CB4856	CT	V	13845436	13846727	WBGene00006022	srx-131	F09F3.2	0.125207134597719
AGIWUR14507
CB4856	CT	V	13847230	13848414	WBGene00006023	srx-132	F09F3.3	0.322466748164315
AGIWUR7957
CB4856	CT	V	13848679	13849969	WBGene00006024	srx-133	F09F3.4	-0.0446675736890709
AGIWUR2081
CB4856	CT	V	13850470	13853199	WBGene00008627	F09F3.5	F09F3.5	-0.192144545975578
AGIWUR16921
CB4856	CT	V	13853834	13855260	WBGene00008628	ttr-21	F09F3.6	0.281759107897848
AGIWUR29376;AGIWUR31698;AGIWUR33752;AGIWUR34397;AGIWUR39923
CB4856	CT	V	13854878	13856476	WBGene00006025	srx-134	F09F3.7	-0.150118799037815
AGIWUR3077
CB4856	CT	V	13857448	13858942	WBGene00006027	srx-136	F09F3.12
-0.291395922291025	AGIWUR12174;AGIWUR19548;AGIWUR37830
CB4856	CT	V	13859301	13861321	WBGene00006026	srx-135	F09F3.11
-0.11837412487829	AGIWUR21897
CB4856	CT	V	13862805	13864110	WBGene00006028	srx-137	F09F3.13
0.134116446913593	AGIWUR21439
CB4856	CT	V	13866706	13868473	WBGene00008630	nhr-175	F09F3.10
-0.00563468646064447	AGIWUR37589
CB4856	CT	V	13869888	13872529	WBGene00014732	F09F3.8	F09F3.8	0.704372386900764
AGIWUR18228
CB4856	CT	V	13873153	13875891	WBGene00008629	cpt-5	F09F3.9	-0.171973437674184
AGIWUR27803
CB4856	CT	V	13876837	13877949	WBGene00011929	T22G5.1	T22G5.1	-0.309441440342772
AGIWUR38579
CB4856	CT	V	13879552	13880213	WBGene00002259	lbp-7	T22G5.2	0.0791055794693527
AGIWUR5469
CB4856	CT	V	13880908	13883937	WBGene00011930	T22G5.3	T22G5.3	-0.126965068846259
AGIWUR10007
CB4856	CT	V	13887658	13889458	WBGene00011931	srsx-19	T22G5.4	0.285116531465016
AGIWUR39026

CB4856	CT	V	13889918	13890295	WBGene00004997	spp-12	T22G5.7	0.369546534287495	AGIWUR10860
CB4856	CT	V	13890679	13891364	WBGene00002260	lbp-8	T22G5.6	-0.335750889215528	AGIWUR7757;AGIWUR22889
CB4856	CT	V	13893325	13896405	WBGene00011932	sptl-3	T22G5.5	-0.158595848382251	AGIWUR2196;AGIWUR2376;AGIWUR4449;AGIWUR23193
CB4856	CT	V	13896663	13898488	WBGene00007362	cyp-35C1	C06B3.3	-0.48879753738446	AGIWUR23062
CB4856	CT	V	13899513	13902653	WBGene00007361	oac-3	C06B3.2	-0.51754846061151	AGIWUR19326;AGIWUR20418;AGIWUR25380
CB4856	CT	V	13902795	13903886	WBGene00007360	C06B3.1	C06B3.1	0.259925293845404	AGIWUR38921
CB4856	CT	V	13904611	13905996	WBGene00007364	stdh-3	C06B3.5	0.112406327296689	AGIWUR33232
CB4856	CT	V	13906492	13908135	WBGene00007363	stdh-1	C06B3.4	-0.171767363925829	AGIWUR6267
CB4856	CT	V	13908133	13909513	WBGene00007365	C06B3.6	C06B3.6	1.75808677359626	AGIWUR1890
CB4856	CT	V	13909686	13911524	WBGene00007366	C06B3.7	C06B3.7	-0.0255106815190977	AGIWUR10558
CB4856	CT	V	13912080	13915436	WBGene00001552	gcy-32	C06B3.8	0.00205298843348789	AGIWUR43888
CB4856	CT	V	13916496	13918051	WBGene00006278	str-252	C06B3.9	0.0263966684211061	AGIWUR25893
CB4856	CT	V	13918641	13920032	WBGene00006277	str-250	C06B3.10	-0.439088887119856	AGIWUR13257
CB4856	CT	V	13921030	13922404	WBGene00005933	srx-42	C06B3.11	-0.0704668035491856	AGIWUR21430;AGIWUR25384
CB4856	CT	V	13924151	13925807	WBGene00044114	srxa-11	C06B3.12	0.158936868626265	AGIWUR25293;AGIWUR27699
CB4856	CT	V	13926698	13929320	WBGene00044115	srxa-12	C06B3.13	0.151489286718301	AGIWUR29606;AGIWUR36139;AGIWUR41790
CB4856	CT	V	13929803	13931252	WBGene00044116	srxa-13	C06B3.14	-0.258725234665897	AGIWUR11485;AGIWUR26810
CB4856	CT	V	13931525	13932198	WBGene00189947	C06B3.16	C06B3.16	-0.26647661296207	AGIWUR3507
CB4856	CT	V	13932575	13934645	WBGene00011185	R10D12.1	R10D12.1	0.493847769354623	AGIWUR1456
CB4856	CT	V	13937223	13939602	WBGene00011197	srw-145	R10D12.17		

0.356890468168371	AGIWUR27930
CB4856	CT	V	13940139	13942182	WBGene00001062	dpr-1	R10D12.2
-0.0734654533831947	AGIWUR18986
CB4856	CT	V	13943462	13944589	WBGene00006018	srx-127	R10D12.3
0.00546809895140665	AGIWUR28843
CB4856	CT	V	13945248	13946485	WBGene00005901	srx-10	R10D12.4
0.0128707603054838	AGIWUR36137
CB4856	CT	V	13946969	13948799	WBGene00011186	clec-226	R10D12.5
0.200366420272189	AGIWUR14296
CB4856	CT	V	13949319	13949640	WBGene00011187	R10D12.6	R10D12.6
0.615168525337275	AGIWUR31172
CB4856	CT	V	13950066	13950394	WBGene00011188	R10D12.7	R10D12.7
-0.280645246100822	AGIWUR23207
CB4856	CT	V	13950733	13953291	WBGene00011189	R10D12.8	R10D12.8
0.253250441088566	AGIWUR6538
CB4856	CT	V	13954277	13956176	WBGene00011190	swt-6	R10D12.9
-0.129705937757965	AGIWUR4056;AGIWUR12190;AGIWUR26090
CB4856	CT	V	13956161	13958347	WBGene00011191	R10D12.10	R10D12.10
-0.0433015271660184	AGIWUR7758;AGIWUR23659
CB4856	CT	V	13958746	13961016	WBGene00011192	srh-24	R10D12.11
0.215050525277571	AGIWUR2603;AGIWUR4584;AGIWUR9879
CB4856	CT	V	13961265	13961977	WBGene00011193	algn-13	R10D12.12
-0.493981098986474	AGIWUR1988
CB4856	CT	V	13962417	13963840	WBGene00011194	R10D12.13	R10D12.13
-0.119158361504037	AGIWUR9954;AGIWUR10705;AGIWUR18062;AGIWUR43283
CB4856	CT	V	13963734	13965797	WBGene00011195	sao-1	R10D12.14
-0.0674255064796592	AGIWUR18990;AGIWUR31397;AGIWUR34082;AGIWUR39963
CB4856	CT	V	13979571	13980907	WBGene00045401	eol-1	T26F2.3	2.34036510531347
AGIWUR22948
CB4856	CT	V	13981568	13982971	WBGene00012058	T26F2.2	T26F2.2	-0.434242496158063
AGIWUR15010
CB4856	CT	V	13985137	13987072	WBGene00001310	end-1	F58E10.2
0.336358429248828	AGIWUR8175
CB4856	CT	V	13987166	14006079	WBGene00010259	ric-7	F58E10.1
0.04536542955156
AGIWUR11260;AGIWUR19271;AGIWUR22169;AGIWUR22854;AGIWUR33583;AGIWUR34027;AGIWUR36597;AGIWUR42820
CB4856	CT	V	14008768	14012218	WBGene00010260	ddx-17	F58E10.3
0.0405708758256758	AGIWUR7766;AGIWUR10134;AGIWUR28864;AGIWUR33511;AGIWUR38214
CB4856	CT	V	14012333	14013644	WBGene00000097	aip-1	F58E10.4

-0.0790016798141444 AGIWUR42494
CB4856 CT V 14014701 14015850 WBGene00001311 end-3 F58E10.5
-0.079492861196826 AGIWUR20810
CB4856 CT V 14015978 14017576 WBGene00010261 srh-308 F58E10.6
0.0188348656072073 AGIWUR22290
CB4856 CT V 14018270 14018673 WBGene00003474 mtl-2 T08G5.10
0.157465046563102 AGIWUR31900
CB4856 CT V 14018766 14020332 WBGene00011622 T08G5.1 T08G5.1 0.950732348518189
AGIWUR41045
CB4856 CT V 14020628 14021737 WBGene00077572 T08G5.14 T08G5.14
-0.0251515462390586 AGIWUR39576
CB4856 CT V 14026336 14027484 WBGene00011624 T08G5.3 T08G5.3 -0.0460046954227025
AGIWUR99;AGIWUR1082;AGIWUR19837;AGIWUR20924;AGIWUR35998
CB4856 CT V 14030184 14032584 WBGene00014834 T08G5.4 T08G5.4 0.0191177341703415
AGIWUR9751;AGIWUR42190
CB4856 CT V 14033950 14034393 WBGene00002093 ins-10 T08G5.12
0.673707242757345 AGIWUR40111
CB4856 CT V 14034323 14038372 WBGene00011625 vps-39 T08G5.5 -0.0501399127318134
AGIWUR2812;AGIWUR16018;AGIWUR25794;AGIWUR38233;AGIWUR44385
CB4856 CT V 14038896 14040234 WBGene00050930 T08G5.13 T08G5.13
-0.116095622013814 AGIWUR11059;AGIWUR16384;AGIWUR30398
CB4856 CT V 14057840 14064529 WBGene00010250 F58D12.3 F58D12.3
0.101212986713233 AGIWUR1597;AGIWUR31940;AGIWUR35221;AGIWUR43868
CB4856 CT V 14067819 14071215 WBGene00045355 D1086.17 D1086.17
0.12754059647423 AGIWUR12879;AGIWUR16108;AGIWUR34946;AGIWUR35308
CB4856 CT V 14071646 14073719 WBGene00077448 D1086.20 D1086.20
-0.137944643085718 AGIWUR546;AGIWUR21272
CB4856 CT V 14074456 14076725 WBGene00044081 D1086.11 D1086.11
0.134005990841773 AGIWUR9110;AGIWUR31787
CB4856 CT V 14079801 14081766 WBGene00008393 D1086.6 D1086.6 -0.0864818585023803
AGIWUR6916;AGIWUR27761
CB4856 CT V 14082753 14083584 WBGene00044080 D1086.10 D1086.10
-0.216097960026014 AGIWUR5549
CB4856 CT V 14084270 14085811 WBGene00008394 D1086.7 D1086.7 -0.0171151140339099
AGIWUR3028;AGIWUR13342
CB4856 CT V 14085889 14087169 WBGene00045405 D1086.18 D1086.18
0.949891400202389 AGIWUR34218
CB4856 CT V 14088351 14088704 WBGene00045406 D1086.19 D1086.19
-0.0504376118920913 AGIWUR8143;AGIWUR14209;AGIWUR16516;AGIWUR22654;AGIWUR29990

CB4856	CT	V	14088893	14090359	WBGene00008392	D1086.5	D1086.5	0.843558293696379	AGIWUR23610
CB4856	CT	V	14090719	14092435	WBGene00001864	him-5	D1086.4	0.205920629437895	AGIWUR44490
CB4856	CT	V	14092792	14093986	WBGene00008390	D1086.3	D1086.3	-0.798093634694986	AGIWUR14700
CB4856	CT	V	14094105	14097088	WBGene00008395	D1086.8	D1086.8	0.08116751352512	AGIWUR12365
CB4856	CT	V	14097038	14098668	WBGene00008389	D1086.2	D1086.2	1.38303564650093	AGIWUR4031
CB4856	CT	V	14098858	14100390	WBGene00008388	D1086.1	D1086.1	0.55903039363484	AGIWUR34537
CB4856	CT	V	14101186	14102821	WBGene00044082	D1086.12	D1086.12	0.292710816699447	AGIWUR36541
CB4856	CT	V	14104270	14106416	WBGene00008396	D1086.9	D1086.9	0.341575463711736	AGIWUR22373;AGIWUR29291
CB4856	CT	V	14107811	14110151	WBGene00044201	H39E23.3	H39E23.3	0.097390645801152	AGIWUR7418;AGIWUR8050;AGIWUR9150;AGIWUR9268;AGIWUR17164;AGIWUR29655;AGIWUR40848
CB4856	CT	V	14110361	14112027	WBGene00010432	H39E23.2	H39E23.2	1.58586639733138	AGIWUR10876
CB4856	CT	V	14112039	14141119	WBGene00003916	par-1	H39E23.1	0.134892631758317	AGIWUR16278;AGIWUR16410;AGIWUR19436;AGIWUR27928;AGIWUR32541;AGIWUR41271
CB4856	CT	V	14144030	14145411	WBGene00007084	AH10.3	AH10.3	0.391783718177586	AGIWUR30614
CB4856	CT	V	14146499	14148784	WBGene00007082	acs-10	AH10.1	0.299343494276382	AGIWUR12516
CB4856	CT	V	14156831	14157622	WBGene00007083	AH10.2	AH10.2	0.367682115187171	AGIWUR34461
CB4856	CT	V	14157663	14160862	WBGene00011364	T02B5.3	T02B5.3	0.402142437238957	AGIWUR26497
CB4856	CT	V	14161551	14162841	WBGene00005104	srd-26	T02B5.4	-0.19561343740482	AGIWUR8800
CB4856	CT	V	14163260	14166730	WBGene00011362	T02B5.1	T02B5.1	0.106113902766886	AGIWUR16187
CB4856	CT	V	14167228	14167729	WBGene00011363	ocam-1	T02B5.2	0.128745713186678	AGIWUR27567
CB4856	CT	V	14168362	14171481	WBGene00013875	ZC376.3	ZC376.3	0.104182020141284	AGIWUR44554
CB4856	CT	V	14171645	14175355	WBGene00013874	ZC376.2	ZC376.2	-0.549336543537418	

AGIWUR25072
CB4856	CT	V	14175517	14182960	WBGene00013873	ZC376.1	ZC376.1	0.505649574413299
AGIWUR25901
CB4856	CT	V	14183124	14185667	WBGene00006613	trm-1	ZC376.5	-0.249286647972069
AGIWUR20951
CB4856	CT	V	14185691	14192156	WBGene00013877	ZC376.6	ZC376.6	-0.0991239931831505
AGIWUR30098
CB4856	CT	V	14193685	14197695	WBGene00013878	atfs-1	ZC376.7	-0.0522474528523844
AGIWUR14731
CB4856	CT	V	14205776	14209010	WBGene00011793	T16A9.3	T16A9.3	-0.0831899124708499
AGIWUR801;AGIWUR8622;AGIWUR32892
CB4856	CT	V	14209170	14210873	WBGene00006261	str-230	T16A9.2	0.477130904776358
AGIWUR11289
CB4856	CT	V	14215343	14220730	WBGene00011794	nep-21	T16A9.4	0.480802901688783
AGIWUR27727
CB4856	CT	V	14225397	14226414	WBGene00050975	K02E11.11	K02E11.11
-0.0507101706051127	AGIWUR642;AGIWUR3294;AGIWUR20831
CB4856	CT	V	14232281	14236076	WBGene00010510	ent-3	K02E11.1
0.277729751914432	AGIWUR24207
CB4856	CT	V	14239942	14241151	WBGene00044109	K02E11.10	K02E11.10
-0.381307577722101	AGIWUR33374
CB4856	CT	V	14244204	14246104	WBGene00010511	sre-7	K02E11.2
0.0393980376219263	AGIWUR19699
CB4856	CT	V	14246276	14246909	WBGene00010512	K02E11.3	K02E11.3
0.0554124052030236	AGIWUR42262
CB4856	CT	V	14247476	14248137	WBGene00010513	K02E11.4	K02E11.4
0.0366417765444789	AGIWUR8828;AGIWUR19022;AGIWUR19695;AGIWUR20713
CB4856	CT	V	14250627	14251510	WBGene00010515	K02E11.6	K02E11.6
-0.00016053243281533	AGIWUR8194;AGIWUR14347;AGIWUR17081
CB4856	CT	V	14253488	14254321	WBGene00010516	K02E11.7	K02E11.7
-0.257963489949276	AGIWUR15018
CB4856	CT	V	14257107	14259231	WBGene00009596	sre-8	F40G12.1
0.110253007704912	AGIWUR44907
CB4856	CT	V	14259544	14260931	WBGene00009597	F40G12.2	F40G12.2
-0.597359042227869	AGIWUR20587
CB4856	CT	V	14261990	14267317	WBGene00009598	F40G12.3	F40G12.3
0.129011181923826	AGIWUR8470
CB4856	CT	V	14267761	14268639	WBGene00009599	F40G12.4	F40G12.4
0.0581003622814293	AGIWUR13152

CB4856	CT	V	14269182	14270636	WBGene00009600	F40G12.5	F40G12.5
0.745740463859522	AGIWUR14463
CB4856	CT	V	14271143	14273047	WBGene00009601	F40G12.6	F40G12.6
0.101587073146957	AGIWUR11143
CB4856	CT	V	14275669	14276343	WBGene00009602	F40G12.7	F40G12.7
-0.279671112386271	AGIWUR28116
CB4856	CT	V	14278312	14279877	WBGene00009603	srt-33	F40G12.8
0.128826293565915	AGIWUR32323
CB4856	CT	V	14281120	14282322	WBGene00044002	srt-74	F40G12.15
-0.0598943594443506	AGIWUR10878;AGIWUR27270
CB4856	CT	V	14282482	14282997	WBGene00009604	F40G12.9	F40G12.9
0.0228497657845921	AGIWUR13071
CB4856	CT	V	14283528	14285327	WBGene00009605	F40G12.10	F40G12.10
0.17235014752848	AGIWUR42439
CB4856	CT	V	14286171	14288056	WBGene00009606	F40G12.11	F40G12.11
-0.13926650886247	AGIWUR11643
CB4856	CT	V	14288733	14289718	WBGene00008788	F14D7.1 F14D7.1 -0.479089182494853
AGIWUR224;AGIWUR11807;AGIWUR17635
CB4856	CT	V	14291910	14293712	WBGene00008789	F14D7.2 F14D7.2 -0.357596784247807
AGIWUR29824
CB4856	CT	V	14295828	14298132	WBGene00008791	F14D7.4 F14D7.4 0.18023081131037
AGIWUR2077;AGIWUR35633
CB4856	CT	V	14299180	14299642	WBGene00008792	F14D7.5 F14D7.5 -0.199722182260702
AGIWUR1779
CB4856	CT	V	14300014	14300561	WBGene00045331	F14D7.12	F14D7.12
-0.304243496661888	AGIWUR7767;AGIWUR25853;AGIWUR35920;AGIWUR36552;AGIWUR37897
CB4856	CT	V	14301413	14306444	WBGene00008793	F14D7.6 F14D7.6 -0.4578402383151
AGIWUR15208
CB4856	CT	V	14310389	14311236	WBGene00008798	F14D7.11	F14D7.11
0.0440096856441698	AGIWUR2201
CB4856	CT	V	14311882	14312788	WBGene00008795	F14D7.8 F14D7.8 0.0672164911632471
AGIWUR10018
CB4856	CT	V	14313759	14314333	WBGene00009802	F47B8.1 F47B8.1 0.581099776312568
AGIWUR30750
CB4856	CT	V	14315859	14316625	WBGene00009803	F47B8.2 F47B8.2 0.817064512749926
AGIWUR41770
CB4856	CT	V	14320546	14321313	WBGene00044194	F47B8.14	F47B8.14
0.153393056829161	AGIWUR7504;AGIWUR9913;AGIWUR32975;AGIWUR38665;AGIWUR41324
CB4856	CT	V	14329069	14330080	WBGene00009807	pepm-1	F47B8.6 0.0574384500250096

AGIWUR10391
CB4856 CT V 14331674 14332667 WBGene00009809 F47B8.8 F47B8.8 0.0223841876479174
AGIWUR38545
CB4856 CT V 14333416 14334803 WBGene00009808 srm-4 F47B8.7 0.195066385584584
AGIWUR33260
CB4856 CT V 14337049 14338808 WBGene00009810 srm-6 F47B8.9 0.0733710759140713
AGIWUR8149;AGIWUR29529
CB4856 CT V 14343068 14346964 WBGene00009811 F47B8.10 F47B8.10
0.593356974079746 AGIWUR30997
CB4856 CT V 14347731 14350776 WBGene00013024 cept-2 Y49A3A.1
-0.383196467310432 AGIWUR16913;AGIWUR33405;AGIWUR42443
CB4856 CT V 14351207 14354421 WBGene00013025 vha-13 Y49A3A.2
-0.14471816050197
AGIWUR2828;AGIWUR4109;AGIWUR6352;AGIWUR7215;AGIWUR12423;AGIWUR12812;AGIWUR18412;AGIWUR19376;AGIWUR33598;AGI
WUR33706;AGIWUR38557;AGIWUR44816
CB4856 CT V 14354410 14355754 WBGene00013026 Y49A3A.3 Y49A3A.3
0.472327544207042 AGIWUR9557
CB4856 CT V 14356076 14356823 WBGene00000877 cyn-1 Y49A3A.5
-0.459958189218743 AGIWUR2731;AGIWUR19749;AGIWUR22880;AGIWUR24716;AGIWUR26930;AGIWUR32649
CB4856 CT V 14356900 14358390 WBGene00013027 Y49A3A.4 Y49A3A.4
0.181830447911913 AGIWUR38068
CB4856 CT V 14373389 14375799 WBGene00006775 unc-39 F56A12.1
0.675586875757191 AGIWUR35843
CB4856 CT V 14376099 14379066 WBGene00010143 F56A12.2 F56A12.2
0.0259907139749389 AGIWUR35255
CB4856 CT V 14392240 14392772 WBGene00007618 C15H11.10 C15H11.10
0.133960560740195 AGIWUR28622
CB4856 CT V 14395070 14395749 WBGene00077576 C15H11.13 C15H11.13
-0.604814734864502 AGIWUR2315
CB4856 CT V 14409078 14411986 WBGene00007614 gnrr-2 C15H11.2
-0.0474599253747208 AGIWUR36691
CB4856 CT V 14412400 14415792 WBGene00003834 nxf-1 C15H11.3
0.117269494677789 AGIWUR12693
CB4856 CT V 14415814 14416176 WBGene00007619 C15H11.11 C15H11.11
0.33794771951564 AGIWUR30610
CB4856 CT V 14416067 14417835 WBGene00003835 nxf-2 C15H11.6
-0.0363136618581326 AGIWUR19959
CB4856 CT V 14417903 14420096 WBGene00007615 set-31 C15H11.5
-0.331959589324129 AGIWUR27949

CB4856	CT	V	14420071	14421450	WBGene00000985	dhs-22	C15H11.4
0.0582631798713309	AGIWUR10213;AGIWUR15538;AGIWUR27388;AGIWUR40728
CB4856	CT	V	14421566	14422618	WBGene00003922	pas-1	C15H11.7
-0.117631203366207	AGIWUR30109;AGIWUR43121;AGIWUR44076
CB4856	CT	V	14422720	14423383	WBGene00007616	rpoa-12	C15H11.8
-0.311040547161959	AGIWUR9582;AGIWUR11619;AGIWUR13169
CB4856	CT	V	14423776	14425008	WBGene00007617	rrbs-1	C15H11.9
-0.17345823272966	AGIWUR2187;AGIWUR22700;AGIWUR35444;AGIWUR37023
CB4856	CT	V	14429165	14430500	WBGene00009079	F23B12.1	F23B12.1
-0.233093230235387	AGIWUR17727
CB4856	CT	V	14431310	14432359	WBGene00001170	egl-1	F23B12.9
0.0724343488329477	AGIWUR33982
CB4856	CT	V	14434563	14435743	WBGene00005706	sru-43	F23B12.2
0.0650262260273222	AGIWUR30624;AGIWUR42345
CB4856	CT	V	14441057	14445346	WBGene00009081	F23B12.4	F23B12.4
0.0326156043355468	AGIWUR26802;AGIWUR27323;AGIWUR44559
CB4856	CT	V	14445324	14447608	WBGene00009082	dlat-1	F23B12.5
0.223469360830196	AGIWUR13860
CB4856	CT	V	14447629	14449587	WBGene00006465	fntb-1	F23B12.6
-0.132956726452077	AGIWUR6698;AGIWUR21483;AGIWUR30837
CB4856	CT	V	14449697	14452971	WBGene00009084	F23B12.7	F23B12.7
0.0112075023375695	AGIWUR4829;AGIWUR35599
CB4856	CT	V	14453305	14456998	WBGene00000257	bmk-1	F23B12.8
-0.142200791746976	AGIWUR3443;AGIWUR36706
CB4856	CT	V	14460624	14461132	WBGene00008590	F08H9.2	F08H9.2	0.305611590839373
AGIWUR5738;AGIWUR9504;AGIWUR13305;AGIWUR15895;AGIWUR18011;AGIWUR38364;AGIWUR39285
CB4856	CT	V	14461943	14462809	WBGene00008591	F08H9.3	F08H9.3	1.58056528357729
AGIWUR42607
CB4856	CT	V	14463588	14464428	WBGene00008592	F08H9.4	F08H9.4	0.610443358349052
AGIWUR12207
CB4856	CT	V	14465095	14468246	WBGene00023515	srz-97	F08H9.12
-0.0904431199510816	AGIWUR16877;AGIWUR39019;AGIWUR43230
CB4856	CT	V	14469191	14472448	WBGene00008594	clec-57	F08H9.6	-0.891695781785944
AGIWUR25819;AGIWUR26410
CB4856	CT	V	14472529	14474374	WBGene00008593	clec-227	F08H9.5
0.242253948252651	AGIWUR12601;AGIWUR28943;AGIWUR34139
CB4856	CT	V	14474399	14476794	WBGene00008595	clec-56	F08H9.7	-0.362574939514403
AGIWUR8691
CB4856	CT	V	14476903	14479003	WBGene00008596	clec-54	F08H9.8	0.302315231630457

AGIWUR43177
CB4856 CT V 14479284 14482195 WBGene00008597 clec-55 F08H9.9 0.65551083202009
AGIWUR30079
CB4856 CT V 14482287 14482836 WBGene00194788 R02D5.10 R02D5.10
0.38197905819692 AGIWUR13960;AGIWUR22681;AGIWUR27498;AGIWUR31073;AGIWUR40594
CB4856 CT V 14483160 14484346 WBGene00010977 R02D5.3 R02D5.3 0.322286774778448
AGIWUR16897
CB4856 CT V 14508249 14509655 WBGene00010976 R02D5.1 R02D5.1 0.437461122349702
AGIWUR12339;AGIWUR42123
CB4856 CT V 14509738 14511123 WBGene00077577 R02D5.9 R02D5.9 0.190836090652543
AGIWUR21673
CB4856 CT V 14518351 14519368 WBGene00050937 R02D5.8 R02D5.8 0.0011058027406996
AGIWUR38956
CB4856 CT V 14519371 14521100 WBGene00194707 C53A5.17 C53A5.17
-0.2184803361062 AGIWUR3775;AGIWUR8040;AGIWUR11708;AGIWUR13521;AGIWUR25149;AGIWUR25297;AGIWUR29485
CB4856 CT V 14523329 14524129 WBGene00008262 ril-1 C53A5.1 -0.0351725774470175
AGIWUR2119;AGIWUR4729;AGIWUR20901;AGIWUR21355;AGIWUR42930;AGIWUR43238
CB4856 CT V 14525389 14527411 WBGene00001834 hda-1 C53A5.3 0.0436947212517698
AGIWUR5903
CB4856 CT V 14531115 14542893 WBGene00008265 kcnl-4 C53A5.5 -0.232382309697382
AGIWUR4093
CB4856 CT V 14543588 14545866 WBGene00008266 C53A5.6 C53A5.6 -0.0512446497171091
AGIWUR12806
CB4856 CT V 14546873 14554546 WBGene00008270 C53A5.13 C53A5.13
-0.298771035478584 AGIWUR22437
CB4856 CT V 14554980 14556287 WBGene00005204 srg-47 C53A5.8 -0.0460436265490944
AGIWUR29829
CB4856 CT V 14557038 14558866 WBGene00008267 C53A5.9 C53A5.9 0.340105943734398
AGIWUR3180
CB4856 CT V 14559612 14561293 WBGene00008268 srt-32 C53A5.10
0.174160191409659 AGIWUR6672
CB4856 CT V 14562565 14564639 WBGene00008269 C53A5.11 C53A5.11
2.8376321782908 AGIWUR19518
CB4856 CT V 14564752 14579719 WBGene00001519 gar-3 Y40H4A.1
0.0414894576214824 AGIWUR19736;AGIWUR36424;AGIWUR39730
CB4856 CT V 14583306 14585451 WBGene00012741 Y40H4A.2 Y40H4A.2
0.0562889834251141 AGIWUR37624
CB4856 CT V 14591474 14593642 WBGene00014822 R11H6.6 R11H6.6 -0.148799955721627
AGIWUR37957

CB4856 CT V 14595268 14597067 WBGene00003982 pes-9 R11H6.1 -0.389275335333327
AGIWUR912;AGIWUR12621;AGIWUR13853;AGIWUR21199;AGIWUR22229
CB4856 CT V 14597939 14600092 WBGene00011250 R11H6.2 R11H6.2 0.00344366986785261
AGIWUR26564
CB4856 CT V 14600272 14606535 WBGene00011251 glb-22 R11H6.3 0.032157040605636
AGIWUR38644
CB4856 CT V 14609943 14610705 WBGene00011252 R11H6.4 R11H6.4 0.290919450952892
AGIWUR28376
CB4856 CT V 14610734 14612222 WBGene00011253 R11H6.5 R11H6.5 0.0373067361127717
AGIWUR40698
CB4856 CT V 14612432 14613706 WBGene00009650 ccnk-1 F43D2.1 -0.0143875448689705
AGIWUR32184
CB4856 CT V 14621135 14629384 WBGene00001986 hot-1 F43D2.4 -0.0463773958334066
AGIWUR27200
CB4856 CT V 14629377 14635180 WBGene00044191 F43D2.6 F43D2.6 -0.211859656106979
AGIWUR23;AGIWUR4702;AGIWUR8799;AGIWUR11684;AGIWUR13617;AGIWUR14871;AGIWUR23346;AGIWUR30060;AGIWUR30678;AGIW
UR30858;AGIWUR31003;AGIWUR31315;AGIWUR31350;AGIWUR32612;AGIWUR34854
CB4856 CT V 14635582 14637751 WBGene00009651 F43D2.2 F43D2.2 -0.482709482793352
AGIWUR14791
CB4856 CT V 14638945 14640015 WBGene00045516 F43D2.7 F43D2.7 0.0915442712766357
AGIWUR20996;AGIWUR22378;AGIWUR28161;AGIWUR31836;AGIWUR40482
CB4856 CT V 14641101 14641382 WBGene00008319 C54G10.1 C54G10.1
-0.00211365488857851 AGIWUR16483
CB4856 CT V 14642739 14645979 WBGene00004337 rfc-1 C54G10.2
-0.198416397641644 AGIWUR2489;AGIWUR8541;AGIWUR9916;AGIWUR13984;AGIWUR27850;AGIWUR37721
CB4856 CT V 14646739 14653464 WBGene00004060 pmp-3 C54G10.3
-0.0192408711670815 AGIWUR27604
CB4856 CT V 14653491 14658174 WBGene00008320 slc-25A29 C54G10.4
-0.173325624863207
AGIWUR4735;AGIWUR15437;AGIWUR24609;AGIWUR28968;AGIWUR33112;AGIWUR38903;AGIWUR39305;AGIWUR40437;AGIWUR40699
CB4856 CT V 14661305 14662928 WBGene00005242 srh-17 C47E8.2 0.22198093069526
AGIWUR38401
CB4856 CT V 14663306 14665927 WBGene00008146 C47E8.9 C47E8.9 0.493351278355092
AGIWUR16984;AGIWUR29136;AGIWUR33718
CB4856 CT V 14666005 14667158 WBGene00008141 C47E8.1 C47E8.1 -0.0496764981633171
AGIWUR3716;AGIWUR6200;AGIWUR12161;AGIWUR42797
CB4856 CT V 14667897 14668743 WBGene00044216 C47E8.10 C47E8.10
-0.330949197230828 AGIWUR377;AGIWUR3153;AGIWUR17976;AGIWUR22180;AGIWUR31385
CB4856 CT V 14680314 14683751 WBGene00008142 C47E8.3 C47E8.3 -0.138797830207089

AGIWUR16754
CB4856 CT V 14683706 14685528 WBGene00008143 C47E8.4 C47E8.4 -0.0961784159903978
AGIWUR36395
CB4856 CT V 14684918 14688543 WBGene00000915 daf-21 C47E8.5 -0.121301794097392
AGIWUR9991;AGIWUR14975;AGIWUR21712;AGIWUR29884;AGIWUR30998;AGIWUR37657
CB4856 CT V 14688616 14688959 WBGene00044180 C47E8.11 C47E8.11
-0.121745863701721 AGIWUR297;AGIWUR6077;AGIWUR42319;AGIWUR42457;AGIWUR43490
CB4856 CT V 14689154 14691861 WBGene00008144 gasr-8 C47E8.6 0.253486921067353
AGIWUR32623
CB4856 CT V 14691854 14697107 WBGene00006836 unc-112 C47E8.7 -0.0575062033737446
AGIWUR188;AGIWUR26949;AGIWUR36523
CB4856 CT V 14698957 14707601 WBGene00008145 set-5 C47E8.8 -0.301743609871238
AGIWUR23099
CB4856 CT V 14711949 14712296 WBGene00014819 R08A2.6 R08A2.6 0.0253269654451138
AGIWUR8279;AGIWUR14356;AGIWUR25788;AGIWUR32369;AGIWUR34440
CB4856 CT V 14712663 14714072 WBGene00011136 R08A2.5 R08A2.5 0.0279687558090922
AGIWUR32248
CB4856 CT V 14714207 14715720 WBGene00011133 R08A2.2 R08A2.2 -0.0944605559192384
AGIWUR28225;AGIWUR36004
CB4856 CT V 14718601 14719591 WBGene00011132 R08A2.1 R08A2.1 0.0631099096244121
AGIWUR23398
CB4856 CT V 14723324 14724766 WBGene00011137 R08A2.7 R08A2.7 0.701101373560219
AGIWUR11484
CB4856 CT V 14726907 14727328 WBGene00013046 Y50E8A.1 Y50E8A.1
0.341245366402745 AGIWUR10947
CB4856 CT V 14728441 14728854 WBGene00013047 Y50E8A.2 Y50E8A.2
-0.206407968078995 AGIWUR18648
CB4856 CT V 14729643 14731129 WBGene00003861 oig-3 Y50E8A.3
0.439390075606596 AGIWUR18726
CB4856 CT V 14731095 14734237 WBGene00006795 unc-61 Y50E8A.4
-0.00204726520459264 AGIWUR12148;AGIWUR21715;AGIWUR34974;AGIWUR39479
CB4856 CT V 14734660 14736169 WBGene00013048 Y50E8A.5 Y50E8A.5
0.000118825749085616 AGIWUR25169
CB4856 CT V 14738542 14741326 WBGene00013049 Y50E8A.6 Y50E8A.6
-0.170015096676273 AGIWUR24930
CB4856 CT V 14745684 14748708 WBGene00013050 lipl-8 Y50E8A.7
-0.352151227308975 AGIWUR39197
CB4856 CT V 14758785 14765831 WBGene00013056 Y50E8A.14 Y50E8A.14
0.227938090812051 AGIWUR27161;AGIWUR30646

CB4856 CT V 14773258 14777697 WBGene00001817 haf-7 Y50E8A.16
-0.187906610888961 AGIWUR43772
CB4856 CT V 14778336 14779714 WBGene00013052 scrm-7 Y50E8A.9
-0.137622897617117 AGIWUR24090
CB4856 CT V 14779996 14781078 WBGene00013053 Y50E8A.10 Y50E8A.10
-0.236514561421512 AGIWUR40189
CB4856 CT V 14781493 14782209 WBGene00013054 Y50E8A.11 Y50E8A.11
-0.086408975295504 AGIWUR3125;AGIWUR4148;AGIWUR20735;AGIWUR22683
CB4856 CT V 14782658 14784065 WBGene00013055 Y50E8A.12 Y50E8A.12
0.0125472570165162 AGIWUR9036
CB4856 CT V 14784167 14815659 WBGene00008183 rin-1 C48G7.3 -0.109594552133242
AGIWUR11451;AGIWUR18250;AGIWUR32306
CB4856 CT V 14821853 14824453 WBGene00012291 W06A7.2 W06A7.2 -0.398680066614169
AGIWUR26273
CB4856 CT V 14824664 14829533 WBGene00012293 W06A7.4 W06A7.4 -0.220942853589499
AGIWUR11198
CB4856 CT V 14829177 14847413 WBGene00004336 ret-1 W06A7.3 0.0583611013924039
AGIWUR4614;AGIWUR9051;AGIWUR32940;AGIWUR33542;AGIWUR39865;AGIWUR43561
CB4856 CT V 14852826 14858596 WBGene00013883 npr-13 ZC412.1 -0.485555491844471
AGIWUR24587
CB4856 CT V 14863168 14867992 WBGene00001540 gcy-14 ZC412.2 0.419218437238075
AGIWUR25373
CB4856 CT V 14868645 14871547 WBGene00013884 ZC412.3 ZC412.3 -0.146840036672689
AGIWUR22737;AGIWUR26775;AGIWUR32462;AGIWUR32846;AGIWUR41774
CB4856 CT V 14874198 14875470 WBGene00013886 ZC412.5 ZC412.5 0.21846959191385
AGIWUR9631
CB4856 CT V 14875945 14876824 WBGene00050943 ZC412.10 ZC412.10
0.176220128877475 AGIWUR19669;AGIWUR20199;AGIWUR25392;AGIWUR38413;AGIWUR41778
CB4856 CT V 14884585 14884880 WBGene00044272 nspa-4 H12D21.13
0.368455383957702 AGIWUR1631;AGIWUR9028;AGIWUR23094;AGIWUR23657;AGIWUR31671
CB4856 CT V 14891450 14893435 WBGene00010380 mpst-2 H12D21.4
0.662143999066029 AGIWUR3938
CB4856 CT V 14894053 14894798 WBGene00010381 H12D21.5 H12D21.5
-0.0923529455809291 AGIWUR18903;AGIWUR37094;AGIWUR42491
CB4856 CT V 14895647 14899013 WBGene00010383 mpst-3 H12D21.7
-0.233055453256149 AGIWUR7967
CB4856 CT V 14899791 14911929 WBGene00010386 H12D21.10 H12D21.10
-0.159806545050525 AGIWUR4311;AGIWUR6332;AGIWUR9362;AGIWUR10420;AGIWUR32434;AGIWUR38707
CB4856 CT V 14915906 14917682 WBGene00010387 H12D21.11 H12D21.11

0.0998094761414672	AGIWUR28713
CB4856	CT	V	14917735	14921609	WBGene00012364	zmp-4	W09D12.1
0.28971148812963	AGIWUR31014
CB4856	CT	V	14921988	14924204	WBGene00005211	srg-54	W09D12.3
0.0993896597895443	AGIWUR10044;AGIWUR13039;AGIWUR19865
CB4856	CT	V	14926710	14928342	WBGene00005212	srg-55	W09D12.2
0.108476714358321	AGIWUR15180
CB4856	CT	V	14936898	14939367	WBGene00044176	C30G7.2	C30G7.2	-0.145104990488072
AGIWUR15345;AGIWUR19514;AGIWUR20514;AGIWUR20840;AGIWUR40778
CB4856	CT	V	14942137	14945704	WBGene00044177	C30G7.3	C30G7.3	0.117193588350937
AGIWUR41197
CB4856	CT	V	14946622	14947229	WBGene00044178	C30G7.4	C30G7.4	0.432317765181542
AGIWUR30727;AGIWUR32223;AGIWUR44853
CB4856	CT	V	14949777	14952796	WBGene00001852	hil-1	C30G7.1	1.70506740376024
AGIWUR6850
CB4856	CT	V	14953115	14954733	WBGene00044179	C30G7.5	C30G7.5	0.141942537825051
AGIWUR13663;AGIWUR24010
CB4856	CT	V	14971436	14972419	WBGene00008522	F02D8.5	F02D8.5	0.423969308500297
AGIWUR7448
CB4856	CT	V	14972907	14978779	WBGene00008520	F02D8.1	F02D8.1	0.0570570731188127
AGIWUR14274
CB4856	CT	V	14978505	14980159	WBGene00001701	grd-12	F02D8.2	0.0881304126758149
AGIWUR8753
CB4856	CT	V	14980354	14982626	WBGene00006960	xbx-1	F02D8.3	-0.156198017008661
AGIWUR25693
CB4856	CT	V	14984893	14987571	WBGene00008521	F02D8.4	F02D8.4	0.0320682470537257
AGIWUR34606
CB4856	CT	V	14987556	14991024	WBGene00011318	cdt-2	T01C3.1	0.0270355349539528
AGIWUR15169
CB4856	CT	V	14991168	14991649	WBGene00011319	T01C3.2	T01C3.2	-0.317306746552397
AGIWUR38466
CB4856	CT	V	14991707	14992677	WBGene00011320	T01C3.3	T01C3.3	-0.16652768188991
AGIWUR20486
CB4856	CT	V	14993838	14995201	WBGene00011321	fil-1	T01C3.4	3.38197525776209
AGIWUR31420
CB4856	CT	V	14996536	14998121	WBGene00011322	irld-14	T01C3.5	0.178967119899989
AGIWUR1135
CB4856	CT	V	14998991	15000029	WBGene00011325	T01C3.11	T01C3.11
0.274417491470151	AGIWUR35806

CB4856	CT	V	15000011	15000594	WBGene00004485	rps-16	T01C3.6	0.00861498604758104	AGIWUR8459;AGIWUR18895
CB4856	CT	V	15000653	15002273	WBGene00001423	fib-1	T01C3.7	-0.022767251915301	AGIWUR20438;AGIWUR21536;AGIWUR25217
CB4856	CT	V	15002543	15005055	WBGene00011323	mut-15	T01C3.8	-0.109111878175771	AGIWUR16375
CB4856	CT	V	15005100	15006400	WBGene00011324	T01C3.9	T01C3.9	0.00178789346914776	AGIWUR28084
CB4856	CT	V	15006412	15011660	WBGene00003775	nmr-2	T01C3.10	0.0734582763663285	AGIWUR2690;AGIWUR21972
CB4856	CT	V	15011870	15014222	WBGene00008832	obr-2	F14H8.1	0.133075125420078	AGIWUR36992
CB4856	CT	V	15019224	15020325	WBGene00008833	F14H8.2	F14H8.2	-0.133844961983886	AGIWUR22268
CB4856	CT	V	15021032	15022049	WBGene00008835	F14H8.5	F14H8.5	0.107257815386692	AGIWUR36821
CB4856	CT	V	15023844	15025150	WBGene00008834	F14H8.4	F14H8.4	-0.152123790387652	AGIWUR23604
CB4856	CT	V	15025740	15031473	WBGene00000562	cng-1	F14H8.6	0.17888663085437	AGIWUR33769
CB4856	CT	V	15039152	15048207	WBGene00004747	sdc-3	C25D7.3	-0.050261300424566	AGIWUR2845
CB4856	CT	V	15051011	15052530	WBGene00007716	fbxa-82	C25D7.4	0.213591821999358	AGIWUR10372
CB4856	CT	V	15053765	15056280	WBGene00007717	C25D7.5	C25D7.5	-0.4729321536093	AGIWUR24839
CB4856	CT	V	15056289	15056744	WBGene00050940	C25D7.16	C25D7.16	0.170630845529652	AGIWUR9975
CB4856	CT	V	15056719	15059599	WBGene00003155	mcm-3	C25D7.6	-0.121785610449339	AGIWUR8062;AGIWUR8850;AGIWUR19068;AGIWUR40939;AGIWUR41661;AGIWUR43165
CB4856	CT	V	15060249	15061530	WBGene00007722	C25D7.15	C25D7.15	0.0204503991434226	AGIWUR122;AGIWUR3050;AGIWUR3052;AGIWUR26496;AGIWUR34917
CB4856	CT	V	15061488	15064087	WBGene00004308	rap-2	C25D7.7	-0.207840787506079	AGIWUR28629
CB4856	CT	V	15066298	15067464	WBGene00007718	otub-1	C25D7.8	-0.143198911749714	AGIWUR22406
CB4856	CT	V	15067465	15068581	WBGene00007720	C25D7.10	C25D7.10	-0.0505986347110845	AGIWUR30033
CB4856	CT	V	15068678	15069504	WBGene00001021	dnj-3	C01G10.12		

-0.203705564279922 AGIWUR17693
CB4856 CT V 15071748 15080152 WBGene00006808 unc-76 C01G10.11
-0.0449442774797726 AGIWUR3430;AGIWUR13249;AGIWUR13398;AGIWUR42081
CB4856 CT V 15080134 15082533 WBGene00007237 C01G10.10 C01G10.10
-0.20598268029495 AGIWUR17288
CB4856 CT V 15082530 15084139 WBGene00007236 C01G10.9 C01G10.9
-0.299578090691766 AGIWUR42749
CB4856 CT V 15084059 15085382 WBGene00007235 C01G10.8 C01G10.8
-0.211355684367346 AGIWUR24212;AGIWUR27098;AGIWUR27860;AGIWUR40458;AGIWUR43962
CB4856 CT V 15085747 15087064 WBGene00007234 C01G10.7 C01G10.7
-0.14534351772029 AGIWUR22086
CB4856 CT V 15087656 15088336 WBGene00007233 C01G10.6 C01G10.6
-0.251641694920249 AGIWUR21700
CB4856 CT V 15088427 15088699 WBGene00077583 C01G10.18 C01G10.18
0.123012096430199 AGIWUR32231
CB4856 CT V 15090757 15091062 WBGene00044658 C01G10.17 C01G10.17
-0.205211643967157 AGIWUR31764
CB4856 CT V 15092966 15094338 WBGene00007241 C01G10.16 C01G10.16
-0.459811979140916 AGIWUR38976
CB4856 CT V 15095049 15097452 WBGene00005969 srx-78 C01G10.3
0.0308266914510016 AGIWUR38301
CB4856 CT V 15097674 15099554 WBGene00005970 srx-79 C01G10.2
0.232744687371048 AGIWUR43742
CB4856 CT V 15099747 15104389 WBGene00007230 C01G10.1 C01G10.1
-0.229196694193673 AGIWUR43126
CB4856 CT V 15104724 15105197 WBGene00077584 C01G10.19 C01G10.19
-0.0899300330420765 AGIWUR22856
CB4856 CT V 15106094 15106473 WBGene00007238 C01G10.13 C01G10.13
-0.101200794726191 AGIWUR36941
CB4856 CT V 15109577 15110345 WBGene00013566 Y75B12A.2 Y75B12A.2
-0.222661792782898 AGIWUR5674
CB4856 CT V 15111960 15113628 WBGene00011342 T01G5.7 T01G5.7 -0.412683161975758
AGIWUR41464
CB4856 CT V 15113781 15114092 WBGene00011341 T01G5.6 T01G5.6 0.0772334094825435
AGIWUR38981
CB4856 CT V 15116844 15119617 WBGene00011339 T01G5.1 T01G5.1 -0.29875705552114
AGIWUR32926
CB4856 CT V 15121729 15125228 WBGene00011340 ugt-30 T01G5.2 -0.677777119288312
AGIWUR40589

CB4856 CT V 15126212 15127513 WBGene00005615 srj-27 T01G5.3 -0.0505101056249924
AGIWUR27734
CB4856 CT V 15128171 15129986 WBGene00005658 srr-7 T01G5.4 -0.146628119189884
AGIWUR30840
CB4856 CT V 15130497 15132516 WBGene00006215 str-171 T01G5.5 0.146562642330331
AGIWUR9632
CB4856 CT V 15134188 15136801 WBGene00010685 aipl-1 K08F9.2 0.123484307784983
AGIWUR12725
CB4856 CT V 15137273 15139888 WBGene00010684 K08F9.1 K08F9.1 0.828094346665537
AGIWUR31121;AGIWUR34826;AGIWUR37417
CB4856 CT V 15140547 15143923 WBGene00010686 K08F9.3 K08F9.3 0.826493167694533
AGIWUR44170
CB4856 CT V 15144019 15146435 WBGene00010687 K08F9.4 K08F9.4 -0.032390143248449
AGIWUR30773
CB4856 CT V 15149046 15150733 WBGene00011665 T09F5.1 T09F5.1 0.29136999429762
AGIWUR35738
CB4856 CT V 15150961 15151638 WBGene00011666 T09F5.2 T09F5.2 -0.228666191946108
AGIWUR18789
CB4856 CT V 15151845 15153369 WBGene00005276 srh-53 T09F5.15
-0.0305723364893442 AGIWUR21704;AGIWUR30575
CB4856 CT V 15153577 15154994 WBGene00005278 srh-55 T09F5.5 -0.0178734990047458
AGIWUR37126
CB4856 CT V 15155139 15156457 WBGene00005277 srh-54 T09F5.6 0.0282546603359171
AGIWUR1667;AGIWUR34644
CB4856 CT V 15158991 15160546 WBGene00006110 str-45 T09F5.3 0.0231788923055198
AGIWUR6042
CB4856 CT V 15161003 15162433 WBGene00014835 T09F5.4 T09F5.4 0.20424976287015
AGIWUR19205;AGIWUR21794
CB4856 CT V 15163082 15164518 WBGene00023506 srz-11 T09F5.16
-0.0690551863856133 AGIWUR12429;AGIWUR40422
CB4856 CT V 15164743 15166318 WBGene00014836 T09F5.12 T09F5.12
0.018559701829199
AGIWUR5187;AGIWUR9932;AGIWUR16230;AGIWUR23997;AGIWUR25680;AGIWUR25747;AGIWUR31915;AGIWUR37507;AGIWUR37916;A
GIWUR44722
CB4856 CT V 15170462 15171642 WBGene00005483 srh-278 T09F5.8 -0.15285652360753
AGIWUR30203
CB4856 CT V 15173635 15174856 WBGene00014837 T09F5.13 T09F5.13
-0.039765575191943 AGIWUR15078
CB4856 CT V 15175673 15176324 WBGene00011668 clec-47 T09F5.9 -0.301462043297818

AGIWUR4783;AGIWUR31179;AGIWUR31331
CB4856 CT V 15176768 15179484 WBGene00005772 srw-25 T09F5.14
0.00564110095560277 AGIWUR23703;AGIWUR41505
CB4856 CT V 15180057 15181785 WBGene00011669 T09F5.10 T09F5.10
-0.268862023670051 AGIWUR27301
CB4856 CT V 15182440 15184125 WBGene00011670 fbxa-84 T09F5.11
-0.504400483876402 AGIWUR34280
CB4856 CT V 15184706 15185878 WBGene00013567 Y75B12B.1 Y75B12B.1
-0.1806040191407 AGIWUR7998;AGIWUR34678
CB4856 CT V 15185891 15186300 WBGene00013568 Y75B12B.3 Y75B12B.3
-0.0307153118326151 AGIWUR7577;AGIWUR19953;AGIWUR29967;AGIWUR31867;AGIWUR38866;AGIWUR41247
CB4856 CT V 15186582 15187787 WBGene00000883 cyn-7 Y75B12B.2
-0.0724799895926491 AGIWUR14782;AGIWUR26242
CB4856 CT V 15187989 15189588 WBGene00013569 bath-36 Y75B12B.4
-0.0957239054775219 AGIWUR4808
CB4856 CT V 15189927 15190926 WBGene00000879 cyn-3 Y75B12B.5
-0.0177368055522873 AGIWUR29379;AGIWUR29942
CB4856 CT V 15191296 15191836 WBGene00013573 Y75B12B.11 Y75B12B.11
0.063449266276951 AGIWUR1882;AGIWUR21973;AGIWUR35511;AGIWUR36041;AGIWUR40308
CB4856 CT V 15192572 15197661 WBGene00004037 plc-2 Y75B12B.6
0.16677328384635 AGIWUR37464
CB4856 CT V 15198255 15199396 WBGene00005748 srw-1 Y75B12B.7
-0.28560251484564 AGIWUR11483
CB4856 CT V 15205095 15206552 WBGene00077440 Y75B12B.14 Y75B12B.14
-0.0271533321876607 AGIWUR4002;AGIWUR4181
CB4856 CT V 15212169 15213706 WBGene00005536 sri-24 C41G6.11
-0.0726585878513859 AGIWUR30590
CB4856 CT V 15215475 15216889 WBGene00005538 sri-26 C41G6.14
0.285489129758431 AGIWUR34017
CB4856 CT V 15217005 15219320 WBGene00005537 sri-25 C41G6.10
0.0886962280434105 AGIWUR41083
CB4856 CT V 15219946 15221377 WBGene00005381 srh-165 C41G6.9 -0.0944895783098314
AGIWUR17371
CB4856 CT V 15221682 15223114 WBGene00008057 irld-25 C41G6.6 -0.0102719978406949
AGIWUR10550
CB4856 CT V 15223968 15225359 WBGene00005771 srw-24 C41G6.7
-0.00516717634022701 AGIWUR35860
CB4856 CT V 15225950 15227472 WBGene00005761 srw-14 C41G6.8 0.30562925521035
AGIWUR17235

CB4856	CT	V	15228092	15230075	WBGene00008055	cyp-34A3	C41G6.1	0.00752025654662436	AGIWUR27581
CB4856	CT	V	15230543	15231893	WBGene00005762	srw-15	C41G6.3	0.222359018030033	AGIWUR7178
CB4856	CT	V	15232500	15233844	WBGene00005760	srw-13	C41G6.15	0.183111206173908	AGIWUR17125
CB4856	CT	V	15233894	15235761	WBGene00008056	nhr-164	C41G6.5	0.12558254014847	AGIWUR41753
CB4856	CT	V	15237334	15238650	WBGene00005759	srw-12	C41G6.2	-0.0687925866728554	AGIWUR1197
CB4856	CT	V	15239070	15240315	WBGene00005758	srw-11	M01B2.11	-0.08073883146937	AGIWUR1583
CB4856	CT	V	15241584	15243943	WBGene00010799	chil-12	M01B2.6	0.166342573846585	AGIWUR28079
CB4856	CT	V	15244756	15247707	WBGene00002211	kin-30	M01B2.1	0.0809693612431435	AGIWUR2872;AGIWUR26412;AGIWUR37435
CB4856	CT	V	15252382	15253291	WBGene00010798	srbc-76	M01B2.4	0.0191153407840003	AGIWUR28878
CB4856	CT	V	15253829	15254805	WBGene00010797	srbc-75	M01B2.3	0.164792641497558	AGIWUR7779
CB4856	CT	V	15257581	15259149	WBGene00010800	srsx-37	M01B2.7	0.00512349931960721	AGIWUR25045
CB4856	CT	V	15260238	15260786	WBGene00010801	M01B2.8	M01B2.8	0.0310134077502351	AGIWUR34144
CB4856	CT	V	15260890	15262244	WBGene00010803	M01B2.10	M01B2.10	0.483347736645056	AGIWUR12440
CB4856	CT	V	15263005	15264231	WBGene00045337	M01B2.12	M01B2.12	0.249447298045078	AGIWUR23366;AGIWUR39919
CB4856	CT	V	15264653	15265786	WBGene00045338	M01B2.13	M01B2.13	1.17064829972167	AGIWUR22195;AGIWUR36262
CB4856	CT	V	15266436	15267505	WBGene00045339	T10H4.13	T10H4.13	0.328023388993973	AGIWUR15224;AGIWUR29329
CB4856	CT	V	15270147	15272363	WBGene00011697	T10H4.4	T10H4.4	-0.481898765195133	AGIWUR11665
CB4856	CT	V	15273383	15274857	WBGene00005763	srw-16	T10H4.5	-0.0949993727439908	AGIWUR21435;AGIWUR32739;AGIWUR38813
CB4856	CT	V	15275443	15276900	WBGene00005764	srw-17	T10H4.6	0.382051335348843	AGIWUR36781
CB4856	CT	V	15277612	15278972	WBGene00005765	srw-18	T10H4.7	0.0407472147412693	

AGIWUR40153;AGIWUR42041
CB4856 CT V 15279782 15281395 WBGene00005766 srw-19 T10H4.8 -0.146263188411389
AGIWUR25938
CB4856 CT V 15281899 15283562 WBGene00005942 srx-51 T10H4.9 -0.0506398551257008
AGIWUR5846;AGIWUR18176;AGIWUR22189;AGIWUR23255
CB4856 CT V 15284242 15285551 WBGene00014839 T10H4.1 T10H4.1 0.0368666949983281
AGIWUR20134;AGIWUR27880
CB4856 CT V 15285605 15287925 WBGene00011698 cyp-34A1 T10H4.10
0.679999347278708 AGIWUR34196;AGIWUR36752
CB4856 CT V 15288924 15291107 WBGene00011699 cyp-34A2 T10H4.11
0.427666857207057 AGIWUR9841;AGIWUR12717;AGIWUR25983;AGIWUR37300
CB4856 CT V 15292490 15293965 WBGene00006153 str-96 T10H4.2 0.102698289317772
AGIWUR28458
CB4856 CT V 15296853 15298700 WBGene00000783 cpr-3 T10H4.12
0.0674600685472484 AGIWUR17997
CB4856 CT V 15299190 15301890 WBGene00005940 srx-49 T26H8.3 0.026790776068867
AGIWUR10126
CB4856 CT V 15302371 15303725 WBGene00005939 srx-48 T26H8.2 0.0386834613523135
AGIWUR31605
CB4856 CT V 15304607 15305602 WBGene00077588 T26H8.5 T26H8.5 0.2142635754678
AGIWUR36665
CB4856 CT V 15307562 15309063 WBGene00014195 srz-10 ZK1037.11
-0.133920043185537 AGIWUR35045
CB4856 CT V 15310120 15310437 WBGene00045514 ZK1037.12 ZK1037.12
0.00524465897875714 AGIWUR3212;AGIWUR5803;AGIWUR13301;AGIWUR18102;AGIWUR41748
CB4856 CT V 15311080 15312536 WBGene00014190 irld-62 ZK1037.1
-0.015576405499421 AGIWUR1413;AGIWUR6378;AGIWUR44594
CB4856 CT V 15312982 15314230 WBGene00014993 ZK1037.2 ZK1037.2
-0.170121487501844 AGIWUR24023;AGIWUR27900
CB4856 CT V 15314668 15315927 WBGene00014191 srt-22 ZK1037.3
0.127345704738892 AGIWUR27698
CB4856 CT V 15316497 15318425 WBGene00014192 nhr-246 ZK1037.4
0.359362240019374 AGIWUR12217
CB4856 CT V 15318961 15320553 WBGene00045515 ZK1037.13 ZK1037.13
-0.0851852242806546 AGIWUR28286
CB4856 CT V 15323853 15325351 WBGene00014193 nhr-247 ZK1037.5
0.324520787366836 AGIWUR3093
CB4856 CT V 15325822 15327795 WBGene00014194 ZK1037.6 ZK1037.6
-0.557530306945806 AGIWUR30444

CB4856	CT	V	15328656	15330064	WBGene00005817	srw-70	ZK1037.7
0.203710372226134	AGIWUR1333
CB4856	CT	V	15330614	15332774	WBGene00005499	srh-295	ZK1037.8
0.081631377021365	AGIWUR8748
CB4856	CT	V	15333151	15334571	WBGene00005860	srw-113	ZK1037.9
0.248078297248018	AGIWUR28439
CB4856	CT	V	15334782	15337505	WBGene00006953	wrt-7	ZK1037.10
0.304127481254843	AGIWUR34552
CB4856	CT	V	15338569	15339482	WBGene00044086	clec-228	H02K04.2
-0.225519779511705	AGIWUR23656
CB4856	CT	V	15340136	15341446	WBGene00010355	clec-229	H02K04.1
-0.415270249903264	AGIWUR13041
CB4856	CT	V	15342056	15344572	WBGene00006954	wrt-8	C29F3.2	0.347801256237265
AGIWUR24077
CB4856	CT	V	15346674	15347411	WBGene00007804	C29F3.3	C29F3.3	0.898422566771481
AGIWUR15493
CB4856	CT	V	15347847	15349144	WBGene00007806	clec-230	C29F3.5
0.3146870681461	AGIWUR153;AGIWUR19005;AGIWUR25227;AGIWUR32717;AGIWUR41233;AGIWUR45193
CB4856	CT	V	15349449	15350662	WBGene00007805	clec-231	C29F3.4
0.103491991415333	AGIWUR37420
CB4856	CT	V	15352574	15353862	WBGene00005949	srx-58	C29F3.6	-0.0986313531637819
AGIWUR41130
CB4856	CT	V	15354833	15357572	WBGene00007807	C29F3.7	C29F3.7	-0.471851472086811
AGIWUR39682
CB4856	CT	V	15357696	15360738	WBGene00001150	ech-1.1	C29F3.1	0.146520488770618
AGIWUR34719
CB4856	CT	V	15363604	15365013	WBGene00005325	srh-106	R08H2.14
-0.123520564417183	AGIWUR529;AGIWUR43325
CB4856	CT	V	15365298	15366825	WBGene00005326	srh-107	R08H2.12
0.11384942949112	AGIWUR8881;AGIWUR12553;AGIWUR26384;AGIWUR26419;AGIWUR33182
CB4856	CT	V	15367159	15368289	WBGene00005327	srh-108	R08H2.11
0.110017950012063	AGIWUR6959;AGIWUR10639;AGIWUR15625
CB4856	CT	V	15368470	15370215	WBGene00011150	nhr-269	R08H2.9	-0.17346457876412
AGIWUR22961
CB4856	CT	V	15370867	15372950	WBGene00011149	R08H2.8	R08H2.8	0.136097801738151
AGIWUR35406
CB4856	CT	V	15373676	15374113	WBGene00011151	R08H2.10	R08H2.10
0.0984756878790503	AGIWUR37438
CB4856	CT	V	15374900	15376323	WBGene00005275	srh-52	R08H2.7	0.0645201846307115

AGIWUR25436
CB4856	CT	V	15376501	15378003	WBGene00005340	srh-122	R08H2.5	0.164804798563678
AGIWUR24573
CB4856	CT	V	15378367	15379917	WBGene00005457	srh-251	R08H2.4	-0.0162408600571906
AGIWUR18065
CB4856	CT	V	15381097	15382305	WBGene00000986	dhs-23	R08H2.1	-0.047700239973663
AGIWUR35197
CB4856	CT	V	15382439	15383839	WBGene00006147	str-88	R08H2.2	0.287163476774789
AGIWUR2182
CB4856	CT	V	15385033	15386653	WBGene00006157	str-102	R08H2.13
-0.058214948773788	AGIWUR25002
CB4856	CT	V	15386923	15388870	WBGene00005386	srh-170	R08H2.3	0.00309661722664689
AGIWUR34041
CB4856	CT	V	15393108	15394957	WBGene00011538	T06E6.1	T06E6.1	-0.123531830669988
AGIWUR3213
CB4856	CT	V	15395696	15397348	WBGene00000868	cyb-3	T06E6.2	0.0347698693554745
AGIWUR10457;AGIWUR11193;AGIWUR18348;AGIWUR18947;AGIWUR19045;AGIWUR22627;AGIWUR36099;AGIWUR44565
CB4856	CT	V	15397385	15398828	WBGene00005931	fbxa-199	T06E6.3
-0.110645279477794	AGIWUR8885
CB4856	CT	V	15398939	15399791	WBGene00044759	T06E6.15	T06E6.15
-0.0317087551239377	AGIWUR14475;AGIWUR43886
CB4856	CT	V	15400327	15401820	WBGene00005519	sri-7	T06E6.4	-0.0510032578748021
AGIWUR28308;AGIWUR32085;AGIWUR44685
CB4856	CT	V	15402277	15404385	WBGene00011539	fbxa-135	T06E6.5
0.699937320384627	AGIWUR9210;AGIWUR29642;AGIWUR36860;AGIWUR41930
CB4856	CT	V	15405159	15406760	WBGene00005464	srh-258	T06E6.7	0.0500656845483149
AGIWUR3933
CB4856	CT	V	15407228	15411380	WBGene00005463	srh-257	T06E6.6	-0.0508119902707698
AGIWUR5150;AGIWUR6121;AGIWUR9002
CB4856	CT	V	15411433	15412725	WBGene00011541	srbc-63	T06E6.11
-0.11202479647283	AGIWUR20907
CB4856	CT	V	15414147	15415349	WBGene00005470	srh-264	T06E6.8	0.0368360063981941
AGIWUR11829
CB4856	CT	V	15416251	15417616	WBGene00005471	srh-265	T06E6.9	-0.0826446269482988
AGIWUR37761
CB4856	CT	V	15418131	15419395	WBGene00011540	T06E6.10	T06E6.10
0.100908161707984	AGIWUR40932
CB4856	CT	V	15419820	15421506	WBGene00005469	srh-263	T06E6.12
0.0173626410114753	AGIWUR8797;AGIWUR27731;AGIWUR36405;AGIWUR37131;AGIWUR40154

CB4856	CT	V	15421673	15423658	WBGene00011542	fbxa-104	T06E6.13
0.0029172047497088	AGIWUR21268
CB4856	CT	V	15423746	15425498	WBGene00012533	fbxa-209	Y36E3A.1
0.355299912181756	AGIWUR5589
CB4856	CT	V	15427305	15432722	WBGene00012070	T26H5.8	T26H5.8	1.50104443016374
AGIWUR3269;AGIWUR13122;AGIWUR17414
CB4856	CT	V	15437132	15438673	WBGene00006168	str-117	T26H5.7	0.0219142095843559
AGIWUR24354;AGIWUR35605
CB4856	CT	V	15438798	15440332	WBGene00014846	T26H5.6	T26H5.6	0.0750109391562601
AGIWUR38172;AGIWUR43959
CB4856	CT	V	15441324	15442979	WBGene00005460	srh-254	T26H5.1	0.444697387209838
AGIWUR12876;AGIWUR23679;AGIWUR28403;AGIWUR35260;AGIWUR39764
CB4856	CT	V	15443401	15443808	WBGene00044783	T26H5.10	T26H5.10
-0.249804419401264	AGIWUR7855;AGIWUR21215;AGIWUR29464;AGIWUR32228
CB4856	CT	V	15444916	15445420	WBGene00044206	T26H5.9	T26H5.9	4.37470566454902
AGIWUR669;AGIWUR10973;AGIWUR15693;AGIWUR34140;AGIWUR43316
CB4856	CT	V	15445853	15447282	WBGene00005446	srh-239	T26H5.3	0.314347526287524
AGIWUR29286
CB4856	CT	V	15447640	15448138	WBGene00012069	T26H5.4	T26H5.4	3.79675541387187
AGIWUR22733
CB4856	CT	V	15449631	15451521	WBGene00005780	srw-33	T26H5.5	0.54781059438264
AGIWUR16165
CB4856	CT	V	15452333	15455840	WBGene00005445	srh-238	T23F1.1	-0.0558385258493801
AGIWUR7147;AGIWUR42643
CB4856	CT	V	15456066	15457445	WBGene00011947	T23F1.3	T23F1.3	0.021314635205961
AGIWUR25858
CB4856	CT	V	15457815	15459188	WBGene00006133	str-71	T23F1.4	0.258517866932829
AGIWUR32145
CB4856	CT	V	15459413	15464425	WBGene00011948	T23F1.5	T23F1.5	1.33905532776645
AGIWUR18005
CB4856	CT	V	15465677	15467099	WBGene00004153	pqn-71	T23F1.6	-0.165750512887019
AGIWUR41589
CB4856	CT	V	15471335	15476122	WBGene00001054	dpf-1	T23F1.7	-0.0517170127211219
AGIWUR42365
CB4856	CT	V	15476086	15478566	WBGene00007368	C06B8.2	C06B8.2	-0.147575080091976
AGIWUR19011;AGIWUR28409
CB4856	CT	V	15479398	15481677	WBGene00007367	nhr-150	C06B8.1	0.672301500723833
AGIWUR11858
CB4856	CT	V	15482149	15483665	WBGene00077594	nhr-151	C06B8.12

0.106644337196837 AGIWUR37535
CB4856 CT V 15486388 15487118 WBGene00007373 C06B8.11 C06B8.11
-0.0826350891837162 AGIWUR19586
CB4856 CT V 15488064 15489401 WBGene00005768 srw-21 C06B8.9 0.115242491292584
AGIWUR22410
CB4856 CT V 15491003 15492770 WBGene00007370 str-264 C06B8.4 -0.150875391647777
AGIWUR6224
CB4856 CT V 15493980 15495761 WBGene00005428 srh-218 C06B8.10
-0.106338359784586 AGIWUR17980
CB4856 CT V 15496269 15497567 WBGene00005429 srh-219 C06B8.6 0.147251651992667
AGIWUR42051
CB4856 CT V 15498743 15511421 WBGene00007372 C06B8.7 C06B8.7 0.452017343508089
AGIWUR30713
CB4856 CT V 15512166 15512780 WBGene00004452 rpl-38 C06B8.8 -0.0673396978822591
AGIWUR2954;AGIWUR10276;AGIWUR18225;AGIWUR19497;AGIWUR29421;AGIWUR36954;AGIWUR39941
CB4856 CT V 15512765 15522020 WBGene00008997 lgc-31 F21A3.7 -0.0241271228493587
AGIWUR2141;AGIWUR5441;AGIWUR11882;AGIWUR22753;AGIWUR25835;AGIWUR32893;AGIWUR35667;AGIWUR43072
CB4856 CT V 15529369 15530767 WBGene00005481 srh-276 F21A3.1 0.313349266748714
AGIWUR14696
CB4856 CT V 15530783 15533251 WBGene00219316 F21A3.11 F21A3.11
1.35390376021483 AGIWUR7750
CB4856 CT V 15536620 15537986 WBGene00008994 F21A3.4 F21A3.4 -0.0721254471553756
AGIWUR18374
CB4856 CT V 15538244 15547020 WBGene00008995 prde-1 F21A3.5 -0.193982218511472
AGIWUR27874;AGIWUR36842
CB4856 CT V 15551155 15559147 WBGene00000057 acr-18 F28F8.1 -0.257105647953962
AGIWUR12480
CB4856 CT V 15567394 15569889 WBGene00009221 acs-2 F28F8.2 5.15016655257618
AGIWUR7908;AGIWUR28624;AGIWUR29438;AGIWUR30410;AGIWUR38477;AGIWUR41007;AGIWUR42897;AGIWUR44518
CB4856 CT V 15569935 15570742 WBGene00003079 lsm-5 F28F8.3 0.153080917610993
AGIWUR4058;AGIWUR18048;AGIWUR26535;AGIWUR35988;AGIWUR37591;AGIWUR38266;AGIWUR42575
CB4856 CT V 15571490 15574106 WBGene00009225 fbxa-94 F28F8.8 0.255363676908317
AGIWUR41240
CB4856 CT V 15574098 15575403 WBGene00009223 mdt-28 F28F8.5 -0.220424197007947
AGIWUR1213
CB4856 CT V 15575498 15577443 WBGene00006446 atx-3 F28F8.6 -0.0733803268176842
AGIWUR34792
CB4856 CT V 15577569 15578486 WBGene00044188 F28F8.9 F28F8.9 -0.121885907557418
AGIWUR6762;AGIWUR33145;AGIWUR41510

CB4856	CT	V	15578984	15590617	WBGene00009224	F28F8.7	F28F8.7	-0.0876314463691514	AGIWUR39186
CB4856	CT	V	15585067	15593185	WBGene00007165	B0391.10	B0391.10	-0.187568432126239	AGIWUR10516
CB4856	CT	V	15593462	15593799	WBGene00007164	fbxa-139	B0391.9	0.0341129064270804	AGIWUR119;AGIWUR1454;AGIWUR3371;AGIWUR16116;AGIWUR19563;AGIWUR27242;AGIWUR42247
CB4856	CT	V	15595055	15596466	WBGene00014643	B0391.8	B0391.8	-0.142748197078296	AGIWUR37846
CB4856	CT	V	15600400	15602345	WBGene00007166	fbxa-152	B0391.11	0.205791181400281	AGIWUR35465
CB4856	CT	V	15605673	15610384	WBGene00007162	fbxa-153	B0391.5	-0.273502172108319	AGIWUR17241;AGIWUR31489;AGIWUR37968
CB4856	CT	V	15610890	15612591	WBGene00006080	str-12	B0391.4	0.0137335658572244	AGIWUR35912
CB4856	CT	V	15613186	15614968	WBGene00006289	str-263	B0391.12	0.0602794955803461	AGIWUR28380
CB4856	CT	V	15614937	15617611	WBGene00007161	B0391.3	B0391.3	0.106631479383555	AGIWUR24303
CB4856	CT	V	15618180	15619395	WBGene00007160	B0391.1	B0391.1	-0.0580842549282341	AGIWUR23157
CB4856	CT	V	15620022	15621232	WBGene00014722	C55A1.13	C55A1.13	0.124374782394214	AGIWUR18236;AGIWUR31370
CB4856	CT	V	15621818	15623077	WBGene00008321	C55A1.1	C55A1.1	0.027687676376571	AGIWUR8124
CB4856	CT	V	15624355	15625515	WBGene00005192	srg-35	C55A1.2	0.0707935322987661	AGIWUR14553;AGIWUR44396
CB4856	CT	V	15625861	15626223	WBGene00008326	C55A1.11	C55A1.11	0.0683916965865725	AGIWUR13748
CB4856	CT	V	15626452	15627238	WBGene00008325	C55A1.9	C55A1.9	0.0551617676151466	AGIWUR21342
CB4856	CT	V	15628125	15629643	WBGene00006275	str-248	C55A1.3	-0.0733165009458192	AGIWUR32115
CB4856	CT	V	15630204	15631509	WBGene00014723	srz-49	C55A1.16	0.154579574131076	AGIWUR19030;AGIWUR20618
CB4856	CT	V	15632718	15635277	WBGene00008329	C55A1.15	C55A1.15	0.0884425963918716	AGIWUR3100;AGIWUR4333;AGIWUR30124;AGIWUR38191;AGIWUR40753
CB4856	CT	V	15635565	15637240	WBGene00008328	srz-43	C55A1.14	-0.0492859466484719	AGIWUR7007;AGIWUR13658
CB4856	CT	V	15637416	15638953	WBGene00008327	srz-44	C55A1.12		

0.170471981107202	AGIWUR40530
CB4856	CT	V	15639678	15642499	WBGene00005384	srh-168	C55A1.10
-0.0154471234195412	AGIWUR4275;AGIWUR44912
CB4856	CT	V	15644875	15646478	WBGene00006090	str-23	C55A1.5	-0.90345015186257
AGIWUR20741
CB4856	CT	V	15648102	15648848	WBGene00008323	C55A1.6	C55A1.6	0.228511693245727
AGIWUR2288;AGIWUR7298;AGIWUR18106;AGIWUR26132;AGIWUR32979;AGIWUR38974
CB4856	CT	V	15650467	15652413	WBGene00005705	sru-42	C55A1.8	-0.125810574527183
AGIWUR38344
CB4856	CT	V	15652438	15659079	WBGene00009978	F53E4.1	F53E4.1	-0.0173691927315271
AGIWUR625;AGIWUR1120;AGIWUR7353;AGIWUR9791;AGIWUR17711;AGIWUR31888;AGIWUR32784
CB4856	CT	V	15669170	15670091	WBGene00010599	K06B4.4	K06B4.4	-0.254834682224237
AGIWUR1472;AGIWUR14005
CB4856	CT	V	15670472	15671784	WBGene00077450	K06B4.15	K06B4.15
-0.508489066255029	AGIWUR31005
CB4856	CT	V	15672666	15673236	WBGene00010598	K06B4.3	K06B4.3	-0.40441388119793
AGIWUR27844
CB4856	CT	V	15674026	15675620	WBGene00003641	nhr-51	K06B4.1	0.0140082945040128
AGIWUR10709;AGIWUR13389
CB4856	CT	V	15677582	15680074	WBGene00003642	nhr-52	K06B4.2	0.109277459391325
AGIWUR33467
CB4856	CT	V	15686090	15687590	WBGene00010600	nhr-196	K06B4.5	-0.0725099200213766
AGIWUR20684
CB4856	CT	V	15687960	15689759	WBGene00010602	nhr-197	K06B4.7	0.0155845505476773
AGIWUR18830
CB4856	CT	V	15690204	15691606	WBGene00010601	nhr-268	K06B4.6	0.0641321907514323
AGIWUR26445
CB4856	CT	V	15691999	15693161	WBGene00014800	nhr-200	K06B4.13
0.112587233103264	AGIWUR15076
CB4856	CT	V	15693994	15696217	WBGene00010603	nhr-198	K06B4.8	-0.0512965907225666
AGIWUR7936
CB4856	CT	V	15696785	15698199	WBGene00005920	srx-29	K06B4.9	0.064125177631233
AGIWUR3617
CB4856	CT	V	15698528	15699899	WBGene00010604	nhr-199	K06B4.10
0.419598358312001	AGIWUR6613
CB4856	CT	V	15700606	15702363	WBGene00003643	nhr-53	K06B4.11
0.403886460177963	AGIWUR42551
CB4856	CT	V	15704960	15709528	WBGene00006686	twk-34	K06B4.12
-0.220076906063492	AGIWUR42155

CB4856	CT	V	15709578	15710501	WBGene00012397	Y6E2A.1	Y6E2A.1	0.282382078939526	AGIWUR21730
CB4856	CT	V	15710787	15711998	WBGene00006156	str-101	Y6E2A.2	0.000444071914398769	AGIWUR2563
CB4856	CT	V	15712248	15715644	WBGene00012398	Y6E2A.4	Y6E2A.4	1.24776854411802	AGIWUR2462
CB4856	CT	V	15716419	15718660	WBGene00012399	Y6E2A.5	Y6E2A.5	1.14413112541695	AGIWUR3329;AGIWUR44726;AGIWUR44897
CB4856	CT	V	15719870	15721292	WBGene00012400	Y6E2A.7	Y6E2A.7	0.612286141493455	AGIWUR23084
CB4856	CT	V	15722188	15723999	WBGene00005357	srh-140	Y6E2A.6	-0.243772304618063	AGIWUR44528
CB4856	CT	V	15724849	15726702	WBGene00012401	irld-57	Y6E2A.8	0.00128182096643137	AGIWUR40186
CB4856	CT	V	15726841	15728353	WBGene00012402	sfxn-1.3	Y6E2A.9	-0.203769960966989	AGIWUR26428;AGIWUR29355;AGIWUR41643
CB4856	CT	V	15729238	15730532	WBGene00050915	Y6E2A.10	Y6E2A.10	0.246863069256186	AGIWUR40704
CB4856	CT	V	15730779	15732309	WBGene00006103	str-38	T23D5.2	0.116781465641358	AGIWUR24345;AGIWUR36279;AGIWUR40640
CB4856	CT	V	15732863	15733668	WBGene00011942	T23D5.3	T23D5.3	-0.892967873501552	AGIWUR3432
CB4856	CT	V	15734023	15735820	WBGene00006093	str-27	T23D5.1	0.0179873921583251	AGIWUR28342
CB4856	CT	V	15737020	15739659	WBGene00014844	T23D5.5	T23D5.5	-0.194999529657951	AGIWUR19384
CB4856	CT	V	15740517	15741951	WBGene00006085	str-18	T23D5.6	0.0704794071280013	AGIWUR32407
CB4856	CT	V	15743260	15744478	WBGene00006086	str-19	T23D5.7	-0.146999322937218	AGIWUR17721;AGIWUR43854
CB4856	CT	V	15745404	15745652	WBGene00077456	T23D5.14	T23D5.14	-0.111259511695698	AGIWUR24664;AGIWUR26354;AGIWUR26713;AGIWUR29651;AGIWUR44216
CB4856	CT	V	15750932	15752149	WBGene00006108	str-43	T23D5.9	-0.0757730193013337	AGIWUR15500
CB4856	CT	V	15755031	15756394	WBGene00006076	str-8	T23D5.11	0.117927574932757	AGIWUR21147
CB4856	CT	V	15758749	15760417	WBGene00006077	str-9	F57A10.1	-0.21642911014988	AGIWUR18310
CB4856	CT	V	15760426	15766172	WBGene00001813	haf-3	F57A10.3		

-0.135811971858548 AGIWUR38570
CB4856 CT V 15766575 15767272 WBGene00010184 F57A10.2 F57A10.2
0.567944263592208 AGIWUR45066
CB4856 CT V 15769438 15770371 WBGene00010185 F57A10.4 F57A10.4
-0.120844714588587 AGIWUR19566
CB4856 CT V 15770302 15773239 WBGene00003650 nhr-60 F57A10.5
0.360715450428696 AGIWUR31604
CB4856 CT V 15774946 15776676 WBGene00010186 nhr-283 F57A10.6
-0.02002410368247 AGIWUR22663
CB4856 CT V 15777718 15779247 WBGene00012056 nhr-285 T26E4.16
0.0813134512435454 AGIWUR25482
CB4856 CT V 15779693 15781082 WBGene00012043 irld-52 T26E4.1 -0.298450896241102
AGIWUR19642
CB4856 CT V 15781401 15782011 WBGene00012044 T26E4.2 T26E4.2 0.324694211831695
AGIWUR39816
CB4856 CT V 15782794 15784470 WBGene00012045 T26E4.3 T26E4.3 -0.102840008483323
AGIWUR44007
CB4856 CT V 15784929 15786597 WBGene00012048 srxa-17 T26E4.6 0.163147930974024
AGIWUR8380
CB4856 CT V 15790476 15792583 WBGene00012046 T26E4.4 T26E4.4 -0.18191788198744
AGIWUR1297;AGIWUR22296
CB4856 CT V 15793570 15795194 WBGene00012049 T26E4.7 T26E4.7 -0.0517499432008393
AGIWUR39128
CB4856 CT V 15799212 15802723 WBGene00012051 T26E4.9 T26E4.9 -0.463249496743286
AGIWUR16925
CB4856 CT V 15804843 15805750 WBGene00012053 srbc-74 T26E4.13
-0.150633821745155 AGIWUR8264
CB4856 CT V 15808119 15810036 WBGene00012055 srsx-36 T26E4.15
0.240190663750726 AGIWUR23418
CB4856 CT V 15810784 15811330 WBGene00010016 F54B8.1 F54B8.1
-0.00764555464590089 AGIWUR958
CB4856 CT V 15814237 15815659 WBGene00010018 fbxa-69 F54B8.3 0.260320818702013
AGIWUR21915
CB4856 CT V 15816464 15817143 WBGene00010019 F54B8.4 F54B8.4 0.480517070493448
AGIWUR1221;AGIWUR1619;AGIWUR11144;AGIWUR22397;AGIWUR31675;AGIWUR33561;AGIWUR33604;AGIWUR35475;AGIWUR38821;AGIWUR40872;AGIWUR44858
CB4856 CT V 15817410 15817846 WBGene00000014 abf-3 F54B8.5 0.0581581475283137
AGIWUR35529
CB4856 CT V 15818427 15819827 WBGene00044103 srbc-47 F54B8.14

0.0177704872815054	AGIWUR16708;AGIWUR36182
CB4856	CT	V	15820283	15821383	WBGene00010020	srbc-48	F54B8.6	-0.222580536404251
AGIWUR42422
CB4856	CT	V	15821698	15822884	WBGene00010021	srbc-49	F54B8.7	0.125690713099331
AGIWUR20962
CB4856	CT	V	15823898	15824966	WBGene00010022	srbc-50	F54B8.8	-0.0918496599637866
AGIWUR7657
CB4856	CT	V	15826410	15827429	WBGene00010023	srbc-51	F54B8.9	-0.108971477934788
AGIWUR31717
CB4856	CT	V	15827823	15828882	WBGene00010024	srbc-52	F54B8.10
0.0538070214784589	AGIWUR35751
CB4856	CT	V	15829022	15830007	WBGene00044105	srbc-55	F54B8.16
0.137456818347507	AGIWUR26313
CB4856	CT	V	15830586	15831686	WBGene00010026	srbc-56	F54B8.12
0.147136033324306	AGIWUR4572;AGIWUR45179
CB4856	CT	V	15832436	15833722	WBGene00010025	srbc-54	F54B8.11
-0.0474417521021193	AGIWUR13949
CB4856	CT	V	15834745	15837403	WBGene00044104	srbc-53	F54B8.15
0.0949300905776118	AGIWUR13012;AGIWUR24832
CB4856	CT	V	15837524	15839522	WBGene00003556	nas-40	F54B8.13
-0.0351071365558191	AGIWUR34477;AGIWUR39894
CB4856	CT	V	15839967	15841195	WBGene00005365	srh-149	K08G2.13
0.447104036074197	AGIWUR15882
CB4856	CT	V	15841837	15844214	WBGene00005368	srh-152	K08G2.3	0.199254344628857
AGIWUR23740
CB4856	CT	V	15844453	15844768	WBGene00050949	K08G2.16	K08G2.16
-0.0432105426965848	AGIWUR19175;AGIWUR26507;AGIWUR29082;AGIWUR39015;AGIWUR41335
CB4856	CT	V	15845931	15847306	WBGene00005366	srh-150	K08G2.2	0.37370244843413
AGIWUR38885
CB4856	CT	V	15855012	15856148	WBGene00005369	srh-153	K08G2.11
0.00297138737834324	AGIWUR27955
CB4856	CT	V	15858838	15859227	WBGene00014803	K08G2.14	K08G2.14
-0.084992793727706	AGIWUR7388;AGIWUR25421
CB4856	CT	V	15859960	15860985	WBGene00005367	srh-151	K08G2.6	-0.626248544997763
AGIWUR24228
CB4856	CT	V	15861452	15862699	WBGene00010688	srz-12	K08G2.7	-0.135356297602129
AGIWUR2579
CB4856	CT	V	15863342	15864458	WBGene00005503	srh-299	K08G2.9	-0.0246504262301137
AGIWUR22771

CB4856	CT	V	15865088	15865811	WBGene00005498	srh-294	K08G2.10	0.0214258113522128	AGIWUR5785;AGIWUR24066;AGIWUR27707
CB4856	CT	V	15866139	15867333	WBGene00005497	srh-293	K08G2.8	0.0596510033731095	AGIWUR25448
CB4856	CT	V	15867683	15869051	WBGene00011513	fbxa-197	T06C12.4	-0.266588534858641	AGIWUR25587
CB4856	CT	V	15869311	15870636	WBGene00011514	fbxa-198	T06C12.5	0.11227654057504	AGIWUR22215;AGIWUR24784;AGIWUR35646
CB4856	CT	V	15871170	15872351	WBGene00006122	str-57	T06C12.2	0.00662467273659573	AGIWUR3263
CB4856	CT	V	15872573	15873770	WBGene00006121	str-56	T06C12.3	-0.0406963208495954	AGIWUR4744
CB4856	CT	V	15874093	15876408	WBGene00003692	nhr-102	T06C12.6	-0.214505677491166	AGIWUR17090
CB4856	CT	V	15876732	15879619	WBGene00003674	nhr-84	T06C12.7	-0.101492137391608	AGIWUR11870
CB4856	CT	V	15885662	15886059	WBGene00011516	T06C12.9	T06C12.9	0.000811475267717299	AGIWUR29957
CB4856	CT	V	15886719	15888851	WBGene00011517	cgt-1	T06C12.10	0.721977516356544	AGIWUR26795
CB4856	CT	V	15892061	15892520	WBGene00011519	T06C12.12	T06C12.12	-0.3430394963134	AGIWUR22023
CB4856	CT	V	15892996	15894160	WBGene00011518	T06C12.11	T06C12.11	-0.210913533838658	AGIWUR41573
CB4856	CT	V	15894454	15896765	WBGene00011520	nhr-213	T06C12.13	0.416570524672114	AGIWUR5439
CB4856	CT	V	15897996	15899415	WBGene00006124	str-60	T06C12.1	-0.400563041522573	AGIWUR44430
CB4856	CT	V	15899996	15900900	WBGene00011521	T06C12.14	T06C12.14	-0.0806571853248474	AGIWUR9101
CB4856	CT	V	15901142	15902166	WBGene00014832	srab-27	T06C12.15	-0.0369081659690145	AGIWUR14272;AGIWUR26824
CB4856	CT	V	15905620	15907032	WBGene00009418	F35E8.2	F35E8.2	0.00240154753122468	AGIWUR12319;AGIWUR42594
CB4856	CT	V	15912148	15913056	WBGene00009420	F35E8.6	F35E8.6	0.169203011760498	AGIWUR6094
CB4856	CT	V	15914014	15915142	WBGene00009421	F35E8.7	F35E8.7	0.00103208622825157	AGIWUR36084
CB4856	CT	V	15915216	15916079	WBGene00001786	gst-38	F35E8.8	-0.638731468710579

AGIWUR4624
CB4856	CT	V	15916334	15917558	WBGene00009422	F35E8.9	F35E8.9	-0.351403439714176	AGIWUR3483
CB4856	CT	V	15921309	15922818	WBGene00000412	cdr-1	F35E8.11	0.211922203012746	AGIWUR32534
CB4856	CT	V	15922939	15924471	WBGene00003698	nhr-108	F35E8.12	-0.114892441942366	AGIWUR15760
CB4856	CT	V	15926425	15928425	WBGene00011505	pzf-1	T05G11.1	-0.16850564489452	AGIWUR19186
CB4856	CT	V	15930182	15931084	WBGene00011506	srbc-77	T05G11.2	0.0855476447500818	AGIWUR5982
CB4856	CT	V	15931292	15932490	WBGene00005773	srw-26	T05G11.3	-0.00144014961328054	AGIWUR30587
CB4856	CT	V	15933267	15934976	WBGene00005774	srw-27	T05G11.4	-0.202827361212491	AGIWUR37244
CB4856	CT	V	15935948	15937110	WBGene00005775	srw-28	T05G11.5	-0.00977490830315686	AGIWUR36679;AGIWUR39032
CB4856	CT	V	15937194	15938538	WBGene00005799	srw-52	T05G11.8	0.0611639212881947	AGIWUR4369
CB4856	CT	V	15938776	15940354	WBGene00005802	srw-55	T05G11.6	-0.0277463270505711	AGIWUR4347
CB4856	CT	V	15943425	15944854	WBGene00005800	srw-53	T05G11.7	0.076350319710178	AGIWUR16562
CB4856	CT	V	15947299	15948461	WBGene00005805	srw-58	H24D24.2	0.106982399791422	AGIWUR27357
CB4856	CT	V	15949716	15951003	WBGene00005806	srw-59	H24D24.1	0.123930518825667	AGIWUR21694
CB4856	CT	V	15951432	15952862	WBGene00005803	srw-56	F36G9.16	-0.441131692699061	AGIWUR36809
CB4856	CT	V	15953595	15955032	WBGene00005804	srw-57	F36G9.1	0.131070872875948	AGIWUR34845
CB4856	CT	V	15955797	15957028	WBGene00005401	srh-186	F36G9.2	0.0911212438939106	AGIWUR8604
CB4856	CT	V	15957406	15959554	WBGene00009485	F36G9.3	F36G9.3	-0.535080291922645	AGIWUR19685
CB4856	CT	V	15959808	15961915	WBGene00014751	F36G9.4	F36G9.4	-0.0377562247978735	AGIWUR31171
CB4856	CT	V	15962086	15963232	WBGene00005515	sri-3	F36G9.10	-0.386250140331118	AGIWUR15677;AGIWUR17012;AGIWUR28051

CB4856	CT	V	15963849	15965212	WBGene00005514	sri-2	F36G9.9	-0.314477699791678	AGIWUR1372
CB4856	CT	V	15966245	15967642	WBGene00005513	sri-1	F36G9.8	0.0709447176707965	AGIWUR26153
CB4856	CT	V	15968474	15969753	WBGene00009486	F36G9.7	F36G9.7	0.0761710394326967	AGIWUR35022
CB4856	CT	V	15970433	15972579	WBGene00005686	sru-23	F36G9.6	0.0838900445285544	AGIWUR22156
CB4856	CT	V	15972907	15974936	WBGene00005685	sru-22	F36G9.5	-0.0502168036416748	AGIWUR28181
CB4856	CT	V	15978714	15981666	WBGene00009488	oac-20	F36G9.12	-0.208240294661657	AGIWUR23069
CB4856	CT	V	15981691	15982854	WBGene00009490	fbxa-99	F36G9.14	-0.383883476812841	AGIWUR27467
CB4856	CT	V	15983294	15986640	WBGene00009489	F36G9.13	F36G9.13	-0.0957959897668674	AGIWUR30823
CB4856	CT	V	15987494	15988174	WBGene00009491	F36G9.15	F36G9.15	-0.0641815433085857	AGIWUR25824;AGIWUR39952
CB4856	CT	V	15988754	15991607	WBGene00007380	C06C6.6	C06C6.6	-0.338660151984008	AGIWUR5010
CB4856	CT	V	15992150	15995607	WBGene00007381	C06C6.7	C06C6.7	-0.514466344013918	AGIWUR11685
CB4856	CT	V	15996214	15996745	WBGene00007383	C06C6.9	C06C6.9	-0.291702377515465	AGIWUR44824
CB4856	CT	V	15997136	15997768	WBGene00007382	C06C6.8	C06C6.8	-0.683324350631467	AGIWUR6150
CB4856	CT	V	15997761	16000653	WBGene00003640	nhr-50	C06C6.5	0.117868265172141	AGIWUR4454;AGIWUR26657;AGIWUR29520
CB4856	CT	V	16002588	16004728	WBGene00003653	nhr-63	C06C6.4	0.0269332308644312	AGIWUR17261
CB4856	CT	V	16006304	16007605	WBGene00006262	str-231	C06C6.3	-0.220621116619984	AGIWUR41558
CB4856	CT	V	16008332	16009664	WBGene00006264	str-233	C06C6.2	-0.0809508620277928	AGIWUR18585
CB4856	CT	V	16010710	16012112	WBGene00007379	C06C6.1	C06C6.1	0.0628936885121973	AGIWUR45005
CB4856	CT	V	16012537	16014226	WBGene00011686	T10C6.2	T10C6.2	0.069982168895983	AGIWUR29743
CB4856	CT	V	16014509	16015752	WBGene00006263	str-232	T10C6.1	-0.0283301028624425	

AGIWUR21772
CB4856	CT	V	16017971	16019862	WBGene00005934	srx-43	T10C6.3	0.0858276241093305	AGIWUR6572;AGIWUR7974
CB4856	CT	V	16020342	16022652	WBGene00005935	srx-44	T10C6.4	-0.00428165912144074	AGIWUR22415
CB4856	CT	V	16023351	16024185	WBGene00011687	T10C6.5	T10C6.5	-0.0716588733081274	AGIWUR13803;AGIWUR29687
CB4856	CT	V	16024326	16027454	WBGene00011688	T10C6.6	T10C6.6	0.337387063043026	AGIWUR11648;AGIWUR33048
CB4856	CT	V	16027449	16031713	WBGene00011689	T10C6.7	T10C6.7	-0.223979387544151	AGIWUR38717
CB4856	CT	V	16036413	16038293	WBGene00011691	T10C6.10	T10C6.10	-0.40596941086742	AGIWUR7120;AGIWUR17168;AGIWUR30370
CB4856	CT	V	16041828	16042283	WBGene00001876	his-2	T10C6.13	-0.0710912379174644	AGIWUR18145
CB4856	CT	V	16044296	16045874	WBGene00050899	T10C6.15	T10C6.15	1.68514826018148	AGIWUR44157
CB4856	CT	V	16046873	16047446	WBGene00013961	ZK285.2	ZK285.2	0.125077663945321	AGIWUR44047
CB4856	CT	V	16047808	16049147	WBGene00006234	str-198	ZK285.1	0.105860613971364	AGIWUR35495
CB4856	CT	V	16049492	16051554	WBGene00008821	best-12	F14H3.2	-0.0318105436394926	AGIWUR39204
CB4856	CT	V	16051923	16053510	WBGene00008822	F14H3.3	F14H3.3	-0.0828738953482855	AGIWUR44849
CB4856	CT	V	16053900	16055072	WBGene00008823	F14H3.4	F14H3.4	-0.335490913310558	AGIWUR3490
CB4856	CT	V	16055806	16057054	WBGene00008824	F14H3.5	F14H3.5	-0.377666664400051	AGIWUR2701
CB4856	CT	V	16057322	16058892	WBGene00008825	F14H3.6	F14H3.6	-0.237600538562686	AGIWUR24313
CB4856	CT	V	16059781	16060235	WBGene00219414	F14H3.15	F14H3.15	0.139508631738368	AGIWUR36744
CB4856	CT	V	16063551	16064108	WBGene00014735	cyp-35D2	F14H3.13	-0.287835369010282	AGIWUR43917
CB4856	CT	V	16064447	16067002	WBGene00008827	F14H3.8	F14H3.8	0.00952225139444085	AGIWUR4110
CB4856	CT	V	16067949	16068763	WBGene00008828	F14H3.9	F14H3.9	0.0804671499181087	AGIWUR20501

CB4856	CT	V	16069238	16071301	WBGene00008829	cyp-35D1	F14H3.10
-0.655923885466141	AGIWUR18064
CB4856	CT	V	16071325	16072738	WBGene00008830	nhr-176	F14H3.11
-0.110773127375459	AGIWUR13486;AGIWUR17452
CB4856	CT	V	16072765	16073956	WBGene00005598	srj-10	F14H3.1	0.0704303657733618
AGIWUR29645
CB4856	CT	V	16074113	16075323	WBGene00008831	F14H3.12	F14H3.12
-0.425909530821277	AGIWUR2204
CB4856	CT	V	16075659	16077308	WBGene00005616	srj-29	F22B8.1	0.020002133285991
AGIWUR36282
CB4856	CT	V	16077979	16079234	WBGene00014741	F22B8.2	F22B8.2	-0.095378750917738
AGIWUR15244
CB4856	CT	V	16086572	16088217	WBGene00009047	F22B8.4	F22B8.4	0.139354513641957
AGIWUR44672
CB4856	CT	V	16090985	16092117	WBGene00009046	F22B8.3	F22B8.3	-0.184968466510949
AGIWUR21008
CB4856	CT	V	16094584	16097320	WBGene00009049	F22B8.7	F22B8.7	0.104715736197772
AGIWUR15062
CB4856	CT	V	16097985	16100209	WBGene00006075	str-7	F22B8.5	-0.209732361002583
AGIWUR15383;AGIWUR19926;AGIWUR42258
CB4856	CT	V	16100627	16102750	WBGene00009048	cth-1	F22B8.6	-0.167867186864614
AGIWUR9763;AGIWUR36127;AGIWUR42001
CB4856	CT	V	16103894	16105950	WBGene00009706	argk-1	F44G3.2	-0.0583084029256682
AGIWUR1153;AGIWUR5200;AGIWUR18061;AGIWUR18759;AGIWUR36128
CB4856	CT	V	16112237	16112968	WBGene00014757	F44G3.4	F44G3.4	0.104744647373691
AGIWUR14212;AGIWUR36160
CB4856	CT	V	16112992	16114337	WBGene00009707	srxa-16	F44G3.5	0.194414932700215
AGIWUR36396
CB4856	CT	V	16115681	16116897	WBGene00006092	str-25	F44G3.11
-0.0548409845319089	AGIWUR7233
CB4856	CT	V	16117363	16118701	WBGene00006158	str-103	F44G3.1	0.0218343434966979
AGIWUR41093
CB4856	CT	V	16121824	16122648	WBGene00004809	skr-3	F44G3.6	0.126319121943066
AGIWUR29757
CB4856	CT	V	16125811	16127497	WBGene00044661	fbxa-143	F44G3.14
0.263138110006942	AGIWUR21812
CB4856	CT	V	16128131	16129089	WBGene00044660	fbxa-87	F44G3.13
0.148669968192523	AGIWUR7460;AGIWUR29772
CB4856	CT	V	16129409	16130979	WBGene00044659	fbxa-142	F44G3.12

0.0719152944994725 AGIWUR14461
CB4856 CT V 16131180 16133283 WBGene00009709 fbxa-144 F44G3.8
-0.1396488397769 AGIWUR29526;AGIWUR33518
CB4856 CT V 16133781 16137143 WBGene00009708 F44G3.7 F44G3.7 0.0258750018818752
AGIWUR25611
CB4856 CT V 16138846 16139627 WBGene00009710 F44G3.10 F44G3.10
-0.184655651481662 AGIWUR7611;AGIWUR37760;AGIWUR40817
CB4856 CT V 16140314 16141164 WBGene00008631 F10A3.1 F10A3.1 0.0151249415120933
AGIWUR34865
CB4856 CT V 16142008 16143116 WBGene00008632 fbxa-88 F10A3.2 -0.54884365318226
AGIWUR31979
CB4856 CT V 16143615 16144653 WBGene00008633 fbxa-18 F10A3.3 -0.39897045829419
AGIWUR10313
CB4856 CT V 16144986 16146263 WBGene00008638 F10A3.16 F10A3.16
-0.335704588864786 AGIWUR14428
CB4856 CT V 16147039 16148327 WBGene00014733 F10A3.10 F10A3.10
0.0896480711335563 AGIWUR582
CB4856 CT V 16149125 16151870 WBGene00008634 F10A3.4 F10A3.4 -0.544833499126238
AGIWUR18361
CB4856 CT V 16151978 16153475 WBGene00006280 str-254 F10A3.9 -0.0590080633569792
AGIWUR7211;AGIWUR44010
CB4856 CT V 16153855 16155211 WBGene00006154 str-97 F10A3.8 0.0315035261706971
AGIWUR13410;AGIWUR25754;AGIWUR37364
CB4856 CT V 16155451 16156722 WBGene00008635 F10A3.7 F10A3.7 0.209088785661152
AGIWUR32120
CB4856 CT V 16157335 16158491 WBGene00006155 str-99 F10A3.6 -0.407652198981344
AGIWUR1542
CB4856 CT V 16158679 16159694 WBGene00045507 F10A3.17 F10A3.17
-0.312088472257845 AGIWUR32356
CB4856 CT V 16160685 16162224 WBGene00006161 str-109 F10A3.5
-0.00986277202353403 AGIWUR3013
CB4856 CT V 16162685 16163795 WBGene00008636 F10A3.11 F10A3.11
0.347404554988625 AGIWUR12243
CB4856 CT V 16164019 16165473 WBGene00008637 F10A3.12 F10A3.12
-0.00408839083313772 AGIWUR37426
CB4856 CT V 16166236 16167409 WBGene00006160 str-108 F10A3.13
-0.0656960809046732 AGIWUR15968
CB4856 CT V 16167875 16169126 WBGene00006162 str-111 F10A3.15
-0.0935247930460115 AGIWUR15156

CB4856 CT V 16173743 16175857 WBGene00010589 cyp-33D1 K05D4.4
-0.24447126608252 AGIWUR15132;AGIWUR26937;AGIWUR28056;AGIWUR32245
CB4856 CT V 16176287 16177491 WBGene00014799 K05D4.5 K05D4.5 -0.130161987163201
AGIWUR19570;AGIWUR25619;AGIWUR36956
CB4856 CT V 16178532 16179966 WBGene00014798 K05D4.1 K05D4.1 -0.030495192099413
AGIWUR13418;AGIWUR22857
CB4856 CT V 16180650 16181861 WBGene00006159 str-106 K05D4.2 0.04998779278481
AGIWUR36320
CB4856 CT V 16182410 16183540 WBGene00010590 srz-13 K05D4.8 0.147094193430815
AGIWUR9886
CB4856 CT V 16185403 16186920 WBGene00005482 srh-277 K05D4.6 0.0754922461608345
AGIWUR114;AGIWUR2111;AGIWUR2913;AGIWUR7866;AGIWUR39691
CB4856 CT V 16187728 16189129 WBGene00005485 srh-280 K05D4.7 0.110619813275042
AGIWUR4413;AGIWUR6895;AGIWUR19136;AGIWUR25620
CB4856 CT V 16190785 16192262 WBGene00005486 srh-281 F11A5.1 -0.418477420646854
AGIWUR15987
CB4856 CT V 16193356 16194581 WBGene00005484 srh-279 F11A5.2 0.0119869243917963
AGIWUR40342
CB4856 CT V 16194939 16195544 WBGene00008671 F11A5.3 F11A5.3 0.226740286380644
AGIWUR28861
CB4856 CT V 16195679 16196290 WBGene00008672 F11A5.4 F11A5.4 -0.167263089992947
AGIWUR37086
CB4856 CT V 16198379 16201045 WBGene00008673 F11A5.5 F11A5.5 0.210437708650495
AGIWUR44997
CB4856 CT V 16202030 16202740 WBGene00045510 F11A5.17 F11A5.17
0.246595658465358 AGIWUR5781;AGIWUR36739;AGIWUR40497
CB4856 CT V 16202809 16204862 WBGene00045511 F11A5.18 F11A5.18
-0.0366885887139237 AGIWUR12498;AGIWUR15612;AGIWUR25301;AGIWUR40184
CB4856 CT V 16205188 16206818 WBGene00008675 irld-26 F11A5.7 -0.103664594922503
AGIWUR991
CB4856 CT V 16206943 16209916 WBGene00008676 oac-15 F11A5.8 0.0281294769190854
AGIWUR43379
CB4856 CT V 16210952 16211454 WBGene00044184 F11A5.16 F11A5.16
-0.00301770371700449 AGIWUR13277;AGIWUR19353;AGIWUR36561;AGIWUR43875
CB4856 CT V 16214890 16217706 WBGene00008677 F11A5.9 F11A5.9 1.1421543065824
AGIWUR17833
CB4856 CT V 16217701 16218605 WBGene00008680 F11A5.15 F11A5.15
0.282828430397814 AGIWUR37713
CB4856 CT V 16219541 16221948 WBGene00001591 glc-1 F11A5.10

1.95615166790692	AGIWUR29186
CB4856	CT	V	16222323	16223415	WBGene00005547	sri-35	F11A5.11
-0.0112266488899433	AGIWUR6562;AGIWUR31026;AGIWUR32138
CB4856	CT	V	16224327	16225540	WBGene00008678	stdh-2	F11A5.12
0.004447150126488	AGIWUR16829
CB4856	CT	V	16225701	16225887	WBGene00014322	F11A5.14	F11A5.14
-0.0560861071429812	AGIWUR15158
CB4856	CT	V	16226013	16226681	WBGene00008679	F11A5.13	F11A5.13
-0.46502983817326	AGIWUR21002
CB4856	CT	V	16227127	16229893	WBGene00009028	F21H7.2	F21H7.2	-0.231183677597097
AGIWUR23420
CB4856	CT	V	16230203	16231043	WBGene00009033	F21H7.12	F21H7.12
1.54016137121457	AGIWUR43397
CB4856	CT	V	16231032	16232317	WBGene00001770	gst-22	F21H7.1	-1.03286196319448
AGIWUR20527
CB4856	CT	V	16232652	16233335	WBGene00009029	F21H7.3	F21H7.3	-1.09116704409599
AGIWUR18667
CB4856	CT	V	16233924	16234896	WBGene00009030	clec-233	F21H7.4
-0.148965313567098	AGIWUR23753
CB4856	CT	V	16235171	16235747	WBGene00005317	srh-98	F21H7.14
-0.0456590858411348	AGIWUR15119
CB4856	CT	V	16237523	16238824	WBGene00009031	F21H7.5	F21H7.5	-0.532269112331017
AGIWUR30066
CB4856	CT	V	16239333	16240447	WBGene00014740	F21H7.6	F21H7.6	0.185720808368373
AGIWUR22044;AGIWUR35030
CB4856	CT	V	16241217	16242445	WBGene00005322	srh-103	F21H7.13
-0.0757709010159964	AGIWUR7209
CB4856	CT	V	16242818	16244178	WBGene00005323	srh-104	F21H7.7	0.0750144256545564
AGIWUR26850
CB4856	CT	V	16246879	16248455	WBGene00005315	srh-95	F21H7.11
-0.00987923586550199	AGIWUR10690;AGIWUR27139;AGIWUR34427
CB4856	CT	V	16248583	16253787	WBGene00001545	gcy-20	F21H7.9	0.360674186475987
AGIWUR34558
CB4856	CT	V	16254301	16256398	WBGene00009032	bgnt-1.3	F21H7.10
0.360631308151069	AGIWUR37791
CB4856	CT	V	16258442	16260686	WBGene00011750	nhr-218	T13F3.2	0.0958180605827117
AGIWUR988
CB4856	CT	V	16261647	16263953	WBGene00003717	nhr-127	T13F3.3	0.308161043367638
AGIWUR40703

CB4856	CT	V	16265097	16265607	WBGene00011751	T13F3.4	T13F3.4	-0.0660594192352697	AGIWUR9411;AGIWUR16759;AGIWUR18945;AGIWUR27431;AGIWUR29628;AGIWUR35257
CB4856	CT	V	16266765	16268150	WBGene00044653	T13F3.9	T13F3.9	-0.194167581612706	AGIWUR23129;AGIWUR24605;AGIWUR40089
CB4856	CT	V	16268335	16269567	WBGene00011752	fbxa-2	T13F3.5	0.0691635485428854	AGIWUR17354;AGIWUR22932;AGIWUR34541
CB4856	CT	V	16270216	16271223	WBGene00011753	T13F3.6	T13F3.6	-0.804359378652044	AGIWUR20125;AGIWUR21281
CB4856	CT	V	16271536	16272498	WBGene00044205	T13F3.8	T13F3.8	0.0300977041911707	AGIWUR12595
CB4856	CT	V	16272686	16275410	WBGene00011754	T13F3.7	T13F3.7	0.197370791713178	AGIWUR11446
CB4856	CT	V	16275913	16277698	WBGene00006227	str-183	T13F3.1	0.0159298325633577	AGIWUR17043
CB4856	CT	V	16278191	16280612	WBGene00009226	cyp-37B1	F28G4.1	3.37343113857048	AGIWUR38767
CB4856	CT	V	16280602	16282321	WBGene00009227	F28G4.2	F28G4.2	0.06544657160231	AGIWUR29795
CB4856	CT	V	16283018	16285894	WBGene00009229	F28G4.4	F28G4.4	-0.108050076469867	AGIWUR15182;AGIWUR16824;AGIWUR27000
CB4856	CT	V	16286151	16287719	WBGene00009228	F28G4.3	F28G4.3	-0.122455379964199	AGIWUR37528
CB4856	CT	V	16288788	16292154	WBGene00009230	oac-18	F28G4.5	-0.136982912205978	AGIWUR32845
CB4856	CT	V	16292709	16295600	WBGene00007829	oac-5	C31A11.1	0.211093016684338	AGIWUR27043
CB4856	CT	V	16297370	16298278	WBGene00007831	srbc-83	C31A11.3	-0.164333280672952	AGIWUR11713;AGIWUR17091
CB4856	CT	V	16298969	16299862	WBGene00044106	srbc-84	C31A11.10	0.132701479561113	AGIWUR7876
CB4856	CT	V	16300576	16301478	WBGene00007832	srbc-82	C31A11.4	0.621891187921904	AGIWUR27488
CB4856	CT	V	16302717	16306156	WBGene00007833	oac-6	C31A11.5	-0.146582701918518	AGIWUR21759
CB4856	CT	V	16307682	16309623	WBGene00007834	srxa-7	C31A11.6	0.164418200417295	AGIWUR6387
CB4856	CT	V	16309805	16314752	WBGene00007835	oac-7	C31A11.7	-0.0650770075673011	AGIWUR45204
CB4856	CT	V	16315304	16317226	WBGene00006247	str-214	C31A11.9		

0.0536495779312984	AGIWUR32118
CB4856	CT	V	16317567	16318805	WBGene00005395	srh-180	F57G8.1	-0.0594756111488657
AGIWUR7163
CB4856	CT	V	16320797	16327477	WBGene00005747	srv-36	F57G8.8	0.17075681307135
AGIWUR662;AGIWUR19347;AGIWUR23944
CB4856	CT	V	16329370	16330242	WBGene00010216	F57G8.7	F57G8.7	1.27272927780182
AGIWUR2979
CB4856	CT	V	16333712	16335063	WBGene00005383	srh-167	F57G8.3	-0.165241813454458
AGIWUR21109
CB4856	CT	V	16336626	16338277	WBGene00005834	srw-87	F57G8.4	0.0560479559601506
AGIWUR11621
CB4856	CT	V	16339024	16341536	WBGene00010214	F57G8.5	F57G8.5	-0.335604038553658
AGIWUR2510;AGIWUR10277;AGIWUR21099
CB4856	CT	V	16342497	16344272	WBGene00010215	nhr-193	F57G8.6	0.826134049834355
AGIWUR30592
CB4856	CT	V	16344375	16346506	WBGene00012349	W08G11.5	W08G11.5
-0.119960923003166	AGIWUR40666
CB4856	CT	V	16347060	16349833	WBGene00012346	W08G11.1	W08G11.1
0.27159411759672	AGIWUR24072
CB4856	CT	V	16357521	16367574	WBGene00012348	pptr-1	W08G11.4
0.0923162131778339	AGIWUR15748;AGIWUR23092;AGIWUR23926;AGIWUR30777;AGIWUR42165;AGIWUR43390
CB4856	CT	V	16367681	16372097	WBGene00012347	W08G11.3	W08G11.3
-0.180507361809937	AGIWUR18074
CB4856	CT	V	16372417	16374776	WBGene00005745	srv-34	F13A7.3	0.159061841439224
AGIWUR3625
CB4856	CT	V	16376239	16377949	WBGene00008724	F13A7.1	F13A7.1	0.123802762077755
AGIWUR36448
CB4856	CT	V	16378892	16380488	WBGene00005746	srv-35	F13A7.4	-0.0945974722506855
AGIWUR1064;AGIWUR24283
CB4856	CT	V	16382167	16383829	WBGene00008725	F13A7.7	F13A7.7	-0.196196112681559
AGIWUR8127
CB4856	CT	V	16384490	16386136	WBGene00005576	sri-64	F13A7.6	-0.0995669007342297
AGIWUR9997;AGIWUR25215;AGIWUR29874
CB4856	CT	V	16386662	16388368	WBGene00005577	sri-65	F13A7.8	0.345110938523272
AGIWUR25201
CB4856	CT	V	16389241	16389865	WBGene00004817	skr-11	F13A7.9	-0.421960638788978
AGIWUR11715
CB4856	CT	V	16390891	16392406	WBGene00001792	gst-44	F13A7.10
0.289001289228644	AGIWUR3708

CB4856 CT V 16392482 16393828 WBGene00005140 srd-63 F13A7.2 -0.226306208170358
AGIWUR2129;AGIWUR23449;AGIWUR32161;AGIWUR33690
CB4856 CT V 16394015 16395075 WBGene00008726 F13A7.11 F13A7.11
0.0928146943342583 AGIWUR19640
CB4856 CT V 16396359 16398502 WBGene00008727 F13A7.12 F13A7.12
-0.15436246924715 AGIWUR39641
CB4856 CT V 16399688 16401625 WBGene00008728 sre-36 F13A7.13
0.0712768330198585 AGIWUR14743
CB4856 CT V 16405751 16407699 WBGene00006375 syp-1 F26D2.2 -0.0775646837665054
AGIWUR3001;AGIWUR12902
CB4856 CT V 16407963 16410270 WBGene00009148 F26D2.3 F26D2.3 -0.165120906608111
AGIWUR1510;AGIWUR11211;AGIWUR21865;AGIWUR24161;AGIWUR27772;AGIWUR40093;AGIWUR44150
CB4856 CT V 16410713 16411843 WBGene00005361 srh-145 F26D2.4 0.083822497025265
AGIWUR9297
CB4856 CT V 16412687 16413786 WBGene00005360 srh-144 F26D2.5 -0.0552300306688496
AGIWUR45095
CB4856 CT V 16414068 16415247 WBGene00014745 F26D2.8 F26D2.8 0.0732942687951628
AGIWUR44;AGIWUR27522
CB4856 CT V 16415694 16417114 WBGene00006120 str-55 F26D2.7 -0.163438158801327
AGIWUR2221
CB4856 CT V 16418433 16420425 WBGene00009154 F26D2.16 F26D2.16
-0.452731406988145 AGIWUR32894
CB4856 CT V 16420996 16422774 WBGene00005795 srw-48 F26D2.9 0.0291409268978873
AGIWUR28331
CB4856 CT V 16423099 16424507 WBGene00005776 srw-29 F26D2.11
-0.128881805500311 AGIWUR31643
CB4856 CT V 16424854 16429603 WBGene00009149 F26D2.10 F26D2.10
-0.632016578831943 AGIWUR6760;AGIWUR29245;AGIWUR41595
CB4856 CT V 16430240 16431861 WBGene00006134 str-73 F26D2.1 -0.18183354588021
AGIWUR22361
CB4856 CT V 16432009 16433572 WBGene00009150 clec-234 F26D2.12
0.030050198040521 AGIWUR7612;AGIWUR16991;AGIWUR24649;AGIWUR26856;AGIWUR27037;AGIWUR33724;AGIWUR44070
CB4856 CT V 16434722 16435610 WBGene00009151 F26D2.13 F26D2.13
0.135887746102124 AGIWUR22570
CB4856 CT V 16435855 16436736 WBGene00009152 F26D2.14 F26D2.14
0.0688413775215122 AGIWUR11473;AGIWUR14643
CB4856 CT V 16437846 16438934 WBGene00009153 F26D2.15 F26D2.15
-0.129741792968431 AGIWUR15745
CB4856 CT V 16439667 16442450 WBGene00011617 T08G3.4 T08G3.4 0.0154919710379557

AGIWUR1245;AGIWUR13961;AGIWUR18166
CB4856	CT	V	16443926	16445734	WBGene00005358	srh-141	T08G3.5	0.0137945023978761
AGIWUR13063
CB4856	CT	V	16445976	16447165	WBGene00011618	T08G3.6	T08G3.6	0.176446637808798
AGIWUR25599
CB4856	CT	V	16448035	16449344	WBGene00011621	T08G3.13	T08G3.13
-0.265814855964464	AGIWUR15610
CB4856	CT	V	16457269	16458814	WBGene00006083	str-15	T08G3.1	-0.0445265246656934
AGIWUR34333
CB4856	CT	V	16459388	16460965	WBGene00005359	srh-142	T08G3.3	0.0109042454117603
AGIWUR21874
CB4856	CT	V	16462438	16466357	WBGene00011619	T08G3.7	T08G3.7
-0.00772148167751752	AGIWUR15711
CB4856	CT	V	16466466	16467940	WBGene00005707	sru-44	T08G3.8	0.0857095796239253
AGIWUR20001
CB4856	CT	V	16469367	16471016	WBGene00005355	srh-138	T08G3.12
-0.224746962039244	AGIWUR37531
CB4856	CT	V	16471442	16473397	WBGene00005782	srw-35	T08G3.10
-0.0874616727125552	AGIWUR13977
CB4856	CT	V	16475677	16476825	WBGene00006017	srx-126	T08G3.9	0.131282615037682
AGIWUR32135
CB4856	CT	V	16478698	16479375	WBGene00011620	T08G3.11	T08G3.11
0.111200757413869	AGIWUR43715
CB4856	CT	V	16481580	16482000	WBGene00010202	F57E7.1	F57E7.1	-0.193691156048162
AGIWUR31789
CB4856	CT	V	16482420	16483144	WBGene00010203	F57E7.2	F57E7.2	-0.108922643043813
AGIWUR13259
CB4856	CT	V	16483981	16485382	WBGene00005356	srh-139	F57E7.3	-0.970318799521563
AGIWUR15646
CB4856	CT	V	16486978	16487574	WBGene00012520	Y32B12A.5	Y32B12A.5
0.169458037668903	AGIWUR3890
CB4856	CT	V	16488472	16489104	WBGene00012516	sqst-4	Y32B12A.1
-0.65363723811713	AGIWUR20316
CB4856	CT	V	16489531	16490520	WBGene00012517	srbc-73	Y32B12A.2
0.0671758909015994	AGIWUR22720
CB4856	CT	V	16490760	16491547	WBGene00005465	srh-259	Y32B12A.6
0.16048740459543	AGIWUR4254;AGIWUR15269;AGIWUR20175
CB4856	CT	V	16492170	16493458	WBGene00005466	srh-260	Y32B12A.7
-0.203798037421056	AGIWUR3405;AGIWUR29973

CB4856	CT	V	16493655	16494449	WBGene00012518	comt-2	Y32B12A.3	-0.22290485563561	AGIWUR3690;AGIWUR15142;AGIWUR25810;AGIWUR27894;AGIWUR28027;AGIWUR28665;AGIWUR35500
CB4856	CT	V	16494730	16495227	WBGene00012519	Y32B12A.4	Y32B12A.4	-0.245757836521808	AGIWUR16079
CB4856	CT	V	16495994	16497596	WBGene00005353	srh-136	F36D3.6	-0.134117949130207	AGIWUR4040
CB4856	CT	V	16499352	16502879	WBGene00009469	F36D3.1	F36D3.1	-0.231076900848866	AGIWUR15881
CB4856	CT	V	16502947	16503282	WBGene00044189	F36D3.14	F36D3.14	0.0720117059597385	AGIWUR1797;AGIWUR4391;AGIWUR5212;AGIWUR34875;AGIWUR41228
CB4856	CT	V	16503947	16505917	WBGene00003644	nhr-54	F36D3.2	0.00883590873582385	AGIWUR18110
CB4856	CT	V	16506897	16508705	WBGene00005654	srr-3	F36D3.3	-0.233550893246957	AGIWUR21200;AGIWUR30737;AGIWUR35699
CB4856	CT	V	16516472	16517719	WBGene00009470	F36D3.4	F36D3.4	-0.531055594952994	AGIWUR14766
CB4856	CT	V	16518183	16520245	WBGene00009472	F36D3.7	F36D3.7	0.0457677034792585	AGIWUR10953;AGIWUR12499;AGIWUR23247;AGIWUR34317
CB4856	CT	V	16520600	16522270	WBGene00005781	srw-34	F36D3.13	-0.104741764442183	AGIWUR4748
CB4856	CT	V	16528935	16530213	WBGene00219326	F36D3.16	F36D3.16	-0.321991507867436	AGIWUR3090
CB4856	CT	V	16531373	16532690	WBGene00009474	clec-235	F36D3.10	0.0697210127426859	AGIWUR22246;AGIWUR34028
CB4856	CT	V	16533004	16534110	WBGene00000782	cpr-2	F36D3.9	0.271742099687799	AGIWUR7124
CB4856	CT	V	16537001	16537771	WBGene00012521	Y32B12B.1	Y32B12B.1	0.0599382397612622	AGIWUR43482
CB4856	CT	V	16538396	16549854	WBGene00012522	Y32B12B.2	Y32B12B.2	0.0335234762584129	AGIWUR11254;AGIWUR41877
CB4856	CT	V	16550980	16552282	WBGene00012523	srbc-65	Y32B12B.3	0.0151769264178974	AGIWUR27274
CB4856	CT	V	16553327	16563504	WBGene00012524	Y32B12B.4	Y32B12B.4	-0.0471214460747458	AGIWUR28882
CB4856	CT	V	16565939	16567572	WBGene00005708	sru-45	Y32B12B.7	-0.333102744570184	AGIWUR43428
CB4856	CT	V	16569387	16571340	WBGene00006471	nhr-233	Y32B12B.6	0.0242975001889514	AGIWUR8446
CB4856	CT	V	16573468	16574952	WBGene00006196	str-149	Y32B12B.5		

0.141110548940771	AGIWUR31136
CB4856	CT	V	16578459	16580524	WBGene00011396	nhr-271	T03E6.3	0.227363845626389
AGIWUR4028
CB4856	CT	V	16581037	16583175	WBGene00011395	T03E6.2	T03E6.2	-0.100195417269438
AGIWUR20782
CB4856	CT	V	16586742	16588528	WBGene00005820	srw-73	T03E6.6	0.0764755587844572
AGIWUR1802
CB4856	CT	V	16590101	16592042	WBGene00005475	srh-269	T03E6.5	0.293495540303904
AGIWUR38015
CB4856	CT	V	16592572	16595346	WBGene00000776	cpl-1	T03E6.7	-0.240745168420037
AGIWUR4698;AGIWUR43048;AGIWUR43652
CB4856	CT	V	16598989	16600723	WBGene00045356	T03E6.9	T03E6.9	-0.0117473985277217
AGIWUR18668;AGIWUR41414
CB4856	CT	V	16600567	16603369	WBGene00011397	T03E6.8	T03E6.8	0.80974275154638
AGIWUR22270
CB4856	CT	V	16603529	16610952	WBGene00194769	Y32B12C.5	Y32B12C.5
0.150797352159126	AGIWUR1181;AGIWUR25930;AGIWUR32738;AGIWUR38459
CB4856	CT	V	16612792	16614767	WBGene00012526	Y32B12C.3	Y32B12C.3
-0.189128203242027	AGIWUR23808
CB4856	CT	V	16618341	16620908	WBGene00050904	Y70C5A.3	Y70C5A.3
-0.000547083160727269	AGIWUR31825
CB4856	CT	V	16621396	16623211	WBGene00013490	Y70C5A.2	Y70C5A.2
0.147852874383043	AGIWUR12702;AGIWUR30116
CB4856	CT	V	16624008	16627970	WBGene00012308	oac-53	W06G6.1	0.182916500626165
AGIWUR25651
CB4856	CT	V	16629291	16629648	WBGene00012309	W06G6.2	W06G6.2	-0.129145223436407
AGIWUR17424
CB4856	CT	V	16629900	16631155	WBGene00005099	srd-21	W06G6.3	0.050592005139384
AGIWUR13478
CB4856	CT	V	16632737	16634085	WBGene00005102	srd-24	W06G6.4	-0.100133706485398
AGIWUR8474;AGIWUR44750
CB4856	CT	V	16634252	16635619	WBGene00005822	srw-75	W06G6.13
-0.696158516161144	AGIWUR19212
CB4856	CT	V	16645997	16646481	WBGene00012311	W06G6.9	W06G6.9	0.242524858082003
AGIWUR42180
CB4856	CT	V	16646791	16647193	WBGene00012312	W06G6.10	W06G6.10
0.145663822876276	AGIWUR9521
CB4856	CT	V	16648027	16648480	WBGene00219308	W06G6.20	W06G6.20
0.76380373785903	AGIWUR3552;AGIWUR17521;AGIWUR40622;AGIWUR43972;AGIWUR44176

CB4856 CT V 16666340 16666747 WBGene00044185 F14F8.14 F14F8.14
-0.405073503570664 AGIWUR10328;AGIWUR15648;AGIWUR18851;AGIWUR42476;AGIWUR44067
CB4856 CT V 16667691 16668573 WBGene00008817 F14F8.9 F14F8.9 -0.0760893374562208
AGIWUR22108
CB4856 CT V 16669339 16671290 WBGene00006125 str-61 F14F8.1 -0.0148551867941524
AGIWUR39904
CB4856 CT V 16671622 16672186 WBGene00008816 F14F8.8 F14F8.8 -0.183175725341739
AGIWUR4826
CB4856 CT V 16673423 16674690 WBGene00008813 srz-2 F14F8.2 0.267228104137498
AGIWUR6690
CB4856 CT V 16675853 16677551 WBGene00008814 srz-1 F14F8.3 -0.102731003982233
AGIWUR21849
CB4856 CT V 16677623 16679337 WBGene00008815 srz-103 F14F8.4 0.130772693762587
AGIWUR41520
CB4856 CT V 16680281 16682656 WBGene00005791 srw-44 F14F8.6 0.00619677309264688
AGIWUR104
CB4856 CT V 16683186 16684878 WBGene00005783 srw-36 F14F8.7 0.127969626547905
AGIWUR29070
CB4856 CT V 16686025 16687083 WBGene00005790 srw-43 F14F8.5 -0.152062068518715
AGIWUR29683
CB4856 CT V 16687527 16689866 WBGene00023505 srz-102 F14F8.13
0.0551071804010359 AGIWUR497
CB4856 CT V 16692125 16693832 WBGene00005789 srw-42 F14F8.11
0.0254799833587134 AGIWUR30032
CB4856 CT V 16697478 16699773 WBGene00005788 srw-41 F14F8.10
0.0420989341816291 AGIWUR37516
CB4856 CT V 16700199 16704409 WBGene00013492 Y70C5C.1 Y70C5C.1
-0.196991768604272 AGIWUR21778
CB4856 CT V 16705082 16706647 WBGene00013493 clec-9 Y70C5C.2
0.451364468049933 AGIWUR39973
CB4856 CT V 16709977 16710778 WBGene00023516 srz-98 Y70C5C.7
-0.0281183166773493 AGIWUR22402;AGIWUR26467;AGIWUR31812
CB4856 CT V 16713449 16715195 WBGene00013494 irld-58 Y70C5C.3
0.123479352341565 AGIWUR6228
CB4856 CT V 16715480 16716718 WBGene00005354 srh-137 Y70C5C.4
0.155020929502288 AGIWUR13217
CB4856 CT V 16718418 16721993 WBGene00013495 clec-236 Y70C5C.5
-0.0236539219002694 AGIWUR15366;AGIWUR24547;AGIWUR25863
CB4856 CT V 16722061 16723844 WBGene00003702 nhr-112 Y70C5C.6

-0.246642524315358	AGIWUR4411;AGIWUR17712;AGIWUR18788
CB4856	CT	V	16728731	16729960	WBGene00012027	fbxa-127	T25E12.12
0.112077296560699	AGIWUR42116
CB4856	CT	V	16733901	16735594	WBGene00012025	clec-38	T25E12.10
0.150679830047541	AGIWUR6517
CB4856	CT	V	16735765	16737404	WBGene00012023	clec-34	T25E12.8
0.193172498072189	AGIWUR1485;AGIWUR8334;AGIWUR32502;AGIWUR34931
CB4856	CT	V	16737673	16739200	WBGene00012024	clec-29	T25E12.9
0.0913161664780668	AGIWUR41772
CB4856	CT	V	16739268	16741588	WBGene00012022	clec-39	T25E12.7
0.21011157933606	AGIWUR32712
CB4856	CT	V	16742423	16744881	WBGene00012021	T25E12.6	T25E12.6
0.162146255430356	AGIWUR36964
CB4856	CT	V	16745080	16746258	WBGene00012020	gyg-2	T25E12.5
0.0981695974522836	AGIWUR36841
CB4856	CT	V	16747559	16788435	WBGene00012019	dkf-2	T25E12.4
-0.0369883227306786	AGIWUR6610;AGIWUR31450;AGIWUR31584
CB4856	CT	V	16789267	16792771	WBGene00012315	immt-2	W06H3.1	-0.171000738771096
AGIWUR148;AGIWUR3517;AGIWUR15542;AGIWUR21222;AGIWUR38953;AGIWUR41677
CB4856	CT	V	16792764	16795007	WBGene00004248	pus-1	W06H3.2	0.0836166441434116
AGIWUR19463
CB4856	CT	V	16794878	16802004	WBGene00012316	ctps-1	W06H3.3	-0.368465119285206
AGIWUR41343
CB4856	CT	V	16802874	16814563	WBGene00013606	cand-1	Y102A5A.1
-0.146759059833482	AGIWUR30164
CB4856	CT	V	16815567	16820712	WBGene00011850	T20B3.1	T20B3.1	1.36065654529406
AGIWUR33326
CB4856	CT	V	16825290	16827592	WBGene00005426	srh-216	T20B3.5	-0.151330358999194
AGIWUR7258
CB4856	CT	V	16828235	16829755	WBGene00005424	srh-214	T20B3.4	0.0049265981216422
AGIWUR12153
CB4856	CT	V	16830557	16832021	WBGene00005425	srh-215	T20B3.3
-0.00532836812770743	AGIWUR25568
CB4856	CT	V	16833316	16839141	WBGene00004026	phy-3	T20B3.7	-0.375942013941888
AGIWUR19081
CB4856	CT	V	16839145	16841061	WBGene00011853	clec-40	T20B3.13
0.84386248930292	AGIWUR29336
CB4856	CT	V	16841459	16842863	WBGene00011851	clec-23	T20B3.8	0.932623478406246
AGIWUR14153

CB4856	CT	V	16843241	16844696	WBGene00044331	clec-25	T20B3.15
0.494306005328712	AGIWUR32341
CB4856	CT	V	16846867	16848466	WBGene00044332	clec-36	T20B3.16
0.193695880979143	AGIWUR9285;AGIWUR17755
CB4856	CT	V	16848611	16850130	WBGene00011852	clec-26	T20B3.12
-0.206090126039916	AGIWUR13391
CB4856	CT	V	16850394	16852095	WBGene00013609	clec-37	Y102A5B.3
0.0212422100433791	AGIWUR37056
CB4856	CT	V	16852291	16854053	WBGene00013608	clec-35	Y102A5B.2
-0.224354340799225	AGIWUR14364;AGIWUR34707
CB4856	CT	V	16854646	16856152	WBGene00013607	clec-27	Y102A5B.1
-0.288048276953582	AGIWUR5046
CB4856	CT	V	16856437	16858077	WBGene00009854	clec-31	F49A5.2	0.97195291763553
AGIWUR16982
CB4856	CT	V	16858429	16860040	WBGene00009855	clec-22	F49A5.3	-0.27830767497265
AGIWUR1555;AGIWUR6322;AGIWUR16586
CB4856	CT	V	16860228	16861869	WBGene00009860	clec-32	F49A5.9	1.38564464573926
AGIWUR25174
CB4856	CT	V	16862012	16863890	WBGene00009856	clec-24	F49A5.4	1.14169018084282
AGIWUR7771;AGIWUR14539;AGIWUR16880
CB4856	CT	V	16865704	16867226	WBGene00009857	clec-28	F49A5.5	-0.021264486036042
AGIWUR34482
CB4856	CT	V	16867678	16869845	WBGene00009859	clec-33	F49A5.7	-0.213437575735032
AGIWUR45121
CB4856	CT	V	16870742	16871487	WBGene00009858	thn-4	F49A5.6	-0.147614138062627
AGIWUR2062
CB4856	CT	V	16873942	16875455	WBGene00005770	srw-23	F49A5.8	-0.0211982211266797
AGIWUR9227
CB4856	CT	V	16875877	16876144	WBGene00044195	F49A5.10	F49A5.10
0.065588757830632	AGIWUR17436;AGIWUR22208;AGIWUR28545;AGIWUR29932;AGIWUR43591
CB4856	CT	V	16878724	16880154	WBGene00008617	F09C6.3	F09C6.3	0.0619660037668286
AGIWUR41965
CB4856	CT	V	16880994	16881336	WBGene00044278	F09C6.14	F09C6.14
-0.104835163486134	AGIWUR11479;AGIWUR13626;AGIWUR22926;AGIWUR26443;AGIWUR44638
CB4856	CT	V	16881901	16882310	WBGene00044181	F09C6.11	F09C6.11
-0.0805967318075655	AGIWUR19891;AGIWUR21290;AGIWUR38265;AGIWUR43218;AGIWUR43793
CB4856	CT	V	16882878	16883142	WBGene00044182	F09C6.12	F09C6.12
0.000773605905515673	AGIWUR10485;AGIWUR20232;AGIWUR31606;AGIWUR41910;AGIWUR44596
CB4856	CT	V	16885075	16885403	WBGene00044183	F09C6.13	F09C6.13

								0.875976806928709	AGIWUR5166;AGIWUR14599;AGIWUR14672;AGIWUR24787;AGIWUR27503
CB4856	CT	V	16887413	16888479	WBGene00008615	F09C6.1	F09C6.1	-0.0487087563208615	AGIWUR43328
CB4856	CT	V	16889275	16890749	WBGene00008616	fbxa-44	F09C6.2	-0.0531486081259054	AGIWUR3276;AGIWUR8021;AGIWUR37377
CB4856	CT	V	16893740	16894107	WBGene00077492	F09C6.15	F09C6.15	-0.0714652306767536	AGIWUR1114;AGIWUR25469
CB4856	CT	V	16894230	16895553	WBGene00005650	srp-9	F09C6.5	-0.146774010469273	AGIWUR21568;AGIWUR41911
CB4856	CT	V	16896358	16897728	WBGene00005651	srp-10	F09C6.4	-0.439074682018338	AGIWUR4775
CB4856	CT	V	16898457	16899455	WBGene00008618	fbxa-45	F09C6.6	-0.444197714395539	AGIWUR45116
CB4856	CT	V	16901215	16902826	WBGene00005145	srd-68	F09C6.7	-1.12730300647117	AGIWUR9408
CB4856	CT	V	16907969	16909971	WBGene00077493	F09C6.16	F09C6.16	-0.123536427060739	AGIWUR3518;AGIWUR14063
CB4856	CT	V	16912234	16915350	WBGene00003706	nhr-116	F09C6.9	-0.0226153188537115	AGIWUR10178
CB4856	CT	V	16915373	16916179	WBGene00008620	F09C6.10	F09C6.10	-0.148039194882441	AGIWUR4409;AGIWUR11154;AGIWUR21121;AGIWUR38731;AGIWUR40439
CB4856	CT	V	16917196	16918478	WBGene00013610	fbxa-206	Y102A5C.1	-0.187208115145172	AGIWUR32733
CB4856	CT	V	16918782	16919784	WBGene00013611	Y102A5C.2	Y102A5C.2	-0.385967891555131	AGIWUR16511;AGIWUR35854;AGIWUR38293;AGIWUR44018
CB4856	CT	V	16919780	16921323	WBGene00013612	fbxa-207	Y102A5C.3	-0.279752048361161	AGIWUR4834;AGIWUR21721;AGIWUR23457;AGIWUR42510
CB4856	CT	V	16922252	16924258	WBGene00013613	Y102A5C.4	Y102A5C.4	0.413231335909698	AGIWUR2578
CB4856	CT	V	16924985	16926125	WBGene00044220	Y102A5C.35	Y102A5C.35	-0.182190608446272	AGIWUR8646
CB4856	CT	V	16926920	16927445	WBGene00044213	Y102A5C.36	Y102A5C.36	-0.286023891970368	AGIWUR1655
CB4856	CT	V	16928328	16928999	WBGene00014954	Y102A5C.5	Y102A5C.5	0.0455049109000178	AGIWUR7475;AGIWUR7938;AGIWUR18033
CB4856	CT	V	16929167	16930063	WBGene00014955	Y102A5C.6	Y102A5C.6	0.112233598028944	AGIWUR5552;AGIWUR6206;AGIWUR31531
CB4856	CT	V	16943191	16943836	WBGene00014956	Y102A5C.11	Y102A5C.11	-0.218556629865908	AGIWUR11429;AGIWUR21276;AGIWUR30634

CB4856 CT V 16947158 16948111 WBGene00013619 fbxa-112 Y102A5C.13
-0.214121400952257 AGIWUR24401
CB4856 CT V 16956529 16957991 WBGene00013621 clec-239 Y102A5C.16
0.1823004528567 AGIWUR5509
CB4856 CT V 16958657 16965675 WBGene00001161 efl-1 Y102A5C.18
0.0287974582913026 AGIWUR31042;AGIWUR35577
CB4856 CT V 16966814 16968586 WBGene00013623 fbxa-90 Y102A5C.19
-0.28545250760812 AGIWUR14527
CB4856 CT V 16969039 16970965 WBGene00013624 srbc-80 Y102A5C.20
-0.0743857563464072 AGIWUR36434
CB4856 CT V 16972434 16974088 WBGene00005338 srh-119 Y102A5C.21
-0.00708601480997295 AGIWUR31546
CB4856 CT V 16975219 16976717 WBGene00005945 srx-54 Y102A5C.22
0.130015295688773 AGIWUR19658;AGIWUR21218
CB4856 CT V 16977191 16978408 WBGene00013625 srz-53 Y102A5C.23
0.269427658218483 AGIWUR26238
CB4856 CT V 16979010 16980244 WBGene00005946 srx-55 Y102A5C.24
-0.0769660741203939 AGIWUR36283
CB4856 CT V 16984044 16985395 WBGene00013627 srz-52 Y102A5C.26
0.0144132636779091 AGIWUR19574
CB4856 CT V 16986460 16987135 WBGene00077624 Y102A5C.40 Y102A5C.40
0.167934814576566 AGIWUR40450
CB4856 CT V 16990810 16991459 WBGene00001706 grd-17 Y102A5C.34
0.162262407838351 AGIWUR18992;AGIWUR41502
CB4856 CT V 16992034 16993910 WBGene00013629 srz-95 Y102A5C.33
-0.0532269676686017 AGIWUR22065
CB4856 CT V 16998933 17000304 WBGene00006249 str-217 Y102A5C.28
-0.0559149652634954 AGIWUR10775
CB4856 CT V 17001467 17003587 WBGene00005521 sri-9 Y102A5C.29
-0.0758671544606946 AGIWUR34695
CB4856 CT V 17003980 17005066 WBGene00014957 Y102A5C.30 Y102A5C.30
-0.259937139082892 AGIWUR3275
CB4856 CT V 17005489 17006753 WBGene00005348 srh-131 Y102A5C.31
-0.325761499121519 AGIWUR42747
CB4856 CT V 17007232 17008402 WBGene00005581 sri-69 Y102A5C.32
0.237286510101081 AGIWUR40373
CB4856 CT V 17008673 17009388 WBGene00009914 F49H6.13 F49H6.13
0.395562998538981 AGIWUR35282
CB4856 CT V 17010062 17011849 WBGene00009905 clec-240 F49H6.1

-0.246364892107487 AGIWUR7659
CB4856 CT V 17012127 17013332 WBGene00009906 clec-241 F49H6.2
0.0140380818802139 AGIWUR41928
CB4856 CT V 17013646 17013861 WBGene00050928 F49H6.16 F49H6.16
0.0604132303932801 AGIWUR11947;AGIWUR12220;AGIWUR29541;AGIWUR36769;AGIWUR39561
CB4856 CT V 17014408 17015326 WBGene00009907 F49H6.3 F49H6.3 -0.163116044446636
AGIWUR44169
CB4856 CT V 17016175 17016446 WBGene00045502 F49H6.14 F49H6.14
0.0856442808566 AGIWUR9144;AGIWUR19042;AGIWUR28769;AGIWUR39378
CB4856 CT V 17017087 17019800 WBGene00005316 srh-97 F49H6.4 -0.162529708909885
AGIWUR4678
CB4856 CT V 17019990 17020226 WBGene00050965 F49H6.17 F49H6.17
-0.129608989457785 AGIWUR21780;AGIWUR22613;AGIWUR30372;AGIWUR35838;AGIWUR43274;AGIWUR43610
CB4856 CT V 17020907 17021461 WBGene00009913 F49H6.12 F49H6.12
-0.327235464145128 AGIWUR15422;AGIWUR21659;AGIWUR24462;AGIWUR27313;AGIWUR32570
CB4856 CT V 17023473 17025347 WBGene00009908 F49H6.5 F49H6.5 1.04326482461018
AGIWUR21039
CB4856 CT V 17027255 17028374 WBGene00014768 F49H6.6 F49H6.6 -0.437307394010433
AGIWUR12196;AGIWUR15561
CB4856 CT V 17028884 17030448 WBGene00005784 srw-37 F49H6.7 0.02874668834008
AGIWUR28075
CB4856 CT V 17030738 17032772 WBGene00009910 srz-92 F49H6.9 -0.0364111490679795
AGIWUR2313;AGIWUR2642;AGIWUR5170;AGIWUR16949;AGIWUR30126;AGIWUR32196;AGIWUR34131
CB4856 CT V 17033218 17035950 WBGene00009912 srz-94 F49H6.11
-0.00153348980255963 AGIWUR37649
CB4856 CT V 17036410 17037862 WBGene00009911 srz-93 F49H6.10
-0.0284746063217689 AGIWUR6095
CB4856 CT V 17040739 17041961 WBGene00050976 F28B1.11 F28B1.11
-0.161711432112154 AGIWUR3533
CB4856 CT V 17042592 17043783 WBGene00009195 hpo-33 F28B1.1
-0.00785312691267848 AGIWUR21710
CB4856 CT V 17044786 17049822 WBGene00009196 F28B1.2 F28B1.2 -0.520418200066453
AGIWUR29073
CB4856 CT V 17051015 17051617 WBGene00077494 F28B1.12 F28B1.12
-0.153221834221426 AGIWUR29490
CB4856 CT V 17052004 17055349 WBGene00009197 F28B1.3 F28B1.3 0.0679489415285266
AGIWUR32415
CB4856 CT V 17055431 17057052 WBGene00009198 F28B1.4 F28B1.4 -0.0448539795149028
AGIWUR43815

CB4856	CT	V	17057135	17059919	WBGene00009199	F28B1.5	F28B1.5	-0.00221057456280604	AGIWUR27451
CB4856	CT	V	17060287	17061675	WBGene00005578	sri-66	F28B1.6	0.179047521122106	AGIWUR5923
CB4856	CT	V	17061889	17063095	WBGene00045381	F28B1.9	F28B1.9	-0.236640465815791	AGIWUR26072;AGIWUR42486
CB4856	CT	V	17063417	17064823	WBGene00005462	srh-256	F28B1.7	-0.0229100677222882	AGIWUR1913;AGIWUR35236
CB4856	CT	V	17064976	17066471	WBGene00005461	srh-255	F28B1.8	0.0592916040358613	AGIWUR23182
CB4856	CT	V	17067146	17067854	WBGene00050958	F28B1.10	F28B1.10	0.038379775767349	AGIWUR36506
CB4856	CT	V	17068627	17069533	WBGene00045382	T05E12.8	T05E12.8	-0.105319012154451	AGIWUR15678
CB4856	CT	V	17070527	17071678	WBGene00011485	srbc-14	T05E12.2	0.0181694617891424	AGIWUR39008
CB4856	CT	V	17074290	17076000	WBGene00006176	str-125	T05E12.1	0.0305087641384558	AGIWUR38712
CB4856	CT	V	17076874	17077989	WBGene00011486	T05E12.3	T05E12.3	1.02548991563389	AGIWUR29382
CB4856	CT	V	17078257	17079895	WBGene00005767	srw-20	T05E12.4	0.0905593263581164	AGIWUR43453
CB4856	CT	V	17080492	17082818	WBGene00045500	T05E12.9	T05E12.9	-0.0587027614218472	AGIWUR39435;AGIWUR41395
CB4856	CT	V	17085002	17090023	WBGene00011487	T05E12.6	T05E12.6	-0.846515300060053	AGIWUR6518;AGIWUR14154;AGIWUR29463;AGIWUR40326;AGIWUR42300
CB4856	CT	V	17090362	17091693	WBGene00005444	srh-237	T05E12.7	-0.822615603093119	AGIWUR6479;AGIWUR19438;AGIWUR36456
CB4856	CT	V	17092570	17093420	WBGene00013935	ZK218.1	ZK218.1	-0.755777135950465	AGIWUR24739
CB4856	CT	V	17094180	17094485	WBGene00050963	ZK218.15	ZK218.15	-0.0934682262514528	AGIWUR14594;AGIWUR24596;AGIWUR27095;AGIWUR36385;AGIWUR36538
CB4856	CT	V	17097927	17098766	WBGene00013937	ZK218.3	ZK218.3	-0.28986203277144	AGIWUR6219
CB4856	CT	V	17099347	17100364	WBGene00013938	ZK218.4	ZK218.4	-0.31588183943851	AGIWUR32987
CB4856	CT	V	17101132	17102017	WBGene00013939	ZK218.5	ZK218.5	-0.180188581363846	AGIWUR2008
CB4856	CT	V	17103410	17104627	WBGene00013940	nhr-248	ZK218.6	0.08358271608218	

AGIWUR1901
CB4856	CT	V	17105783	17106656	WBGene00013941	ZK218.7	ZK218.7	0.171908194535725
AGIWUR24098
CB4856	CT	V	17107530	17108418	WBGene00013943	ZK218.11	ZK218.11
-0.0711345188577557	AGIWUR2994
CB4856	CT	V	17109684	17110448	WBGene00045492	ZK218.14	ZK218.14
-0.164147993700716	AGIWUR3949;AGIWUR7949;AGIWUR33943
CB4856	CT	V	17114540	17114887	WBGene00014981	ZK218.9	ZK218.9	-0.0382491442402113
AGIWUR2424;AGIWUR15452;AGIWUR22073;AGIWUR28343;AGIWUR39661
CB4856	CT	V	17116026	17117009	WBGene00013942	ZK218.8	ZK218.8	-0.130102757093251
AGIWUR24794
CB4856	CT	V	17146825	17153266	WBGene00014753	F40D4.14	F40D4.14
0.0256063795801098	AGIWUR3920;AGIWUR28464
CB4856	CT	V	17159874	17162621	WBGene00009571	F40D4.12	F40D4.12
-0.0195059173200312	AGIWUR14728;AGIWUR30832;AGIWUR31537
CB4856	CT	V	17162900	17165509	WBGene00005321	srh-102	F40D4.11
0.289581241049007	AGIWUR38142
CB4856	CT	V	17166028	17167557	WBGene00009569	srz-66	F40D4.9	-0.0591711797116492
AGIWUR28911
CB4856	CT	V	17167833	17169279	WBGene00009570	srz-65	F40D4.10
0.092100056584254	AGIWUR21917
CB4856	CT	V	17169442	17170922	WBGene00005798	srw-51	F40D4.8	0.0239902454993697
AGIWUR10986
CB4856	CT	V	17171851	17173313	WBGene00009568	srbc-26	F40D4.7	-0.122818823560994
AGIWUR43279
CB4856	CT	V	17174038	17175530	WBGene00009567	srbc-25	F40D4.6	-0.0318580687144756
AGIWUR151;AGIWUR44899
CB4856	CT	V	17176716	17178067	WBGene00009566	srsx-1	F40D4.5	-0.0109840238340952
AGIWUR9027
CB4856	CT	V	17178757	17180002	WBGene00005390	srh-174	F40D4.1	-0.0660486828771241
AGIWUR20313;AGIWUR44339
CB4856	CT	V	17184396	17186082	WBGene00005378	srh-162	F40D4.4	0.0142625206914646
AGIWUR6806;AGIWUR34277
CB4856	CT	V	17186873	17188680	WBGene00005413	srh-202	Y68A4A.1
-0.180630652485241	AGIWUR18682
CB4856	CT	V	17189186	17190499	WBGene00006235	str-199	Y68A4A.3
0.327057445160537	AGIWUR34565
CB4856	CT	V	17193031	17199003	WBGene00013467	Y68A4A.5	Y68A4A.5
-0.0614305663571648	AGIWUR14113;AGIWUR28008

CB4856 CT V 17199418 17201736 WBGene00013468 srz-96 Y68A4A.6
0.342117547774885 AGIWUR40306
CB4856 CT V 17205641 17207160 WBGene00005821 srw-74 Y68A4A.11
-0.127613294823529 AGIWUR40920
CB4856 CT V 17209065 17209955 WBGene00014944 Y68A4A.12 Y68A4A.12
0.253996160784368 AGIWUR43560
CB4856 CT V 17210716 17212039 WBGene00005443 srh-236 Y68A4A.9
-0.181001249698767 AGIWUR6236
CB4856 CT V 17213043 17214212 WBGene00005332 srh-113 Y68A4A.7
0.0273069433850592 AGIWUR42610
CB4856 CT V 17215066 17216751 WBGene00014943 Y68A4A.8 Y68A4A.8
-0.148790100147324 AGIWUR5679
CB4856 CT V 17217990 17219004 WBGene00011840 srbc-62 T19C9.1
0.000155126276228934 AGIWUR32160
CB4856 CT V 17219253 17220445 WBGene00005328 srh-109 T19C9.4 0.120937028994771
AGIWUR39083
CB4856 CT V 17222386 17223405 WBGene00005458 srh-252 T19C9.3 0.206965822745055
AGIWUR15804
CB4856 CT V 17225086 17226219 WBGene00005331 srh-112 T19C9.2 -0.115486826345225
AGIWUR41950
CB4856 CT V 17229555 17229990 WBGene00011842 T19C9.6 T19C9.6 0.0856724143521594
AGIWUR28;AGIWUR1422;AGIWUR14042;AGIWUR17957;AGIWUR25905
CB4856 CT V 17234329 17234630 WBGene00050933 T19C9.9 T19C9.9 -0.0726533037855974
AGIWUR4141;AGIWUR19550;AGIWUR26952;AGIWUR34168;AGIWUR36985
CB4856 CT V 17236592 17238097 WBGene00050934 T19C9.10 T19C9.10
-0.0648150051463325 AGIWUR4377;AGIWUR19269;AGIWUR26611;AGIWUR41291;AGIWUR42252
CB4856 CT V 17239097 17240656 WBGene00011844 T19C9.8 T19C9.8 0.0972433038093019
AGIWUR39193
CB4856 CT V 17241708 17242652 WBGene00013472 Y68A4B.3 Y68A4B.3
0.0441116457160251 AGIWUR4089;AGIWUR12735
CB4856 CT V 17243767 17245506 WBGene00013471 clec-242 Y68A4B.2
-0.433561286024446 AGIWUR41908
CB4856 CT V 17247325 17249015 WBGene00013470 clec-243 Y68A4B.1
0.167240893467299 AGIWUR4918
CB4856 CT V 17251911 17252309 WBGene00219383 Y61B8A.6 Y61B8A.6
0.11087822914544 AGIWUR9858;AGIWUR38279
CB4856 CT V 17252931 17255128 WBGene00005335 srh-116 Y61B8A.1
0.148199429122208 AGIWUR22833
CB4856 CT V 17255754 17257194 WBGene00005334 srh-115 Y61B8A.2

-0.357303831244653	AGIWUR27892
CB4856	CT	V	17259173	17260534	WBGene00044654	fbxa-85	Y61B8A.4
-0.14165265018515	AGIWUR32797
CB4856	CT	V	17260674	17262325	WBGene00005794	srw-47	K10G4.2	0.173373457921416
AGIWUR42524
CB4856	CT	V	17264066	17264955	WBGene00010752	K10G4.3	K10G4.3	0.0411317486723268
AGIWUR19495
CB4856	CT	V	17271730	17272888	WBGene00005336	srh-117	K10G4.6	0.0666046715096451
AGIWUR10777
CB4856	CT	V	17273524	17275059	WBGene00044202	K10G4.10	K10G4.10
-0.0817697715339731	AGIWUR17927;AGIWUR27492
CB4856	CT	V	17278367	17285169	WBGene00010753	K10G4.4	K10G4.4	-0.261199307734971
AGIWUR19573
CB4856	CT	V	17292214	17293729	WBGene00005793	srw-46	K10G4.8	0.0645418625366876
AGIWUR874
CB4856	CT	V	17294413	17297359	WBGene00077629	K10G4.13	K10G4.13
0.380818522192811	AGIWUR4790
CB4856	CT	V	17303054	17304438	WBGene00005582	sri-70	Y61B8B.1
-0.230277411835003	AGIWUR10381
CB4856	CT	V	17316351	17317719	WBGene00023507	srz-26	F31E9.7	0.0178031360340132
AGIWUR29264
CB4856	CT	V	17318147	17319515	WBGene00009298	srz-58	F31E9.5	-0.307270431561196
AGIWUR38599
CB4856	CT	V	17319596	17322444	WBGene00009295	fbxa-180	F31E9.1
-0.125428713492816	AGIWUR31899
CB4856	CT	V	17322615	17324518	WBGene00009296	F31E9.3	F31E9.3	0.233505606350035
AGIWUR2504;AGIWUR3170;AGIWUR4495;AGIWUR7491;AGIWUR7699;AGIWUR16129;AGIWUR18574;AGIWUR30010;AGIWUR39662;AGIW
UR44275
CB4856	CT	V	17324907	17326168	WBGene00045408	F31E9.8	F31E9.8	0.127085933440709
AGIWUR388
CB4856	CT	V	17326453	17327967	WBGene00009297	sdz-16	F31E9.4	0.113082614471487
AGIWUR6249;AGIWUR25089
CB4856	CT	V	17331570	17335546	WBGene00009836	fbxa-185	F47H4.4
0.0537304120431039	AGIWUR4082;AGIWUR14040;AGIWUR14183;AGIWUR28850
CB4856	CT	V	17336138	17338335	WBGene00009837	fbxa-186	F47H4.6
0.0743521602844943	AGIWUR43890
CB4856	CT	V	17339084	17342780	WBGene00009838	fbxa-187	F47H4.7
-0.0767003519001745	AGIWUR34510
CB4856	CT	V	17347922	17348625	WBGene00004811	skr-5	F47H4.10

-0.27669684201862	AGIWUR3296
CB4856	CT	V	17348665	17350180	WBGene00009834	lsy-27	F47H4.1	-0.327821544581784
AGIWUR2966
CB4856	CT	V	17350387	17352870	WBGene00009841	fbxa-134	F47H4.11
0.353684544353177	AGIWUR726
CB4856	CT	V	17353325	17360212	WBGene00009835	F47H4.2	F47H4.2	-0.248052983073595
AGIWUR31591
CB4856	CT	V	17369715	17371468	WBGene00014764	srz-46	F47H4.3	-0.0881627025773017
AGIWUR5100
CB4856	CT	V	17371544	17376745	WBGene00012491	Y20C6A.1	Y20C6A.1
1.24386188404694	AGIWUR12193
CB4856	CT	V	17377406	17379226	WBGene00012492	fbxb-7	Y20C6A.2
-0.459683070142436	AGIWUR14771
CB4856	CT	V	17380232	17381119	WBGene00077495	Y20C6A.4	Y20C6A.4
0.258169257843298	AGIWUR4613
CB4856	CT	V	17382090	17383373	WBGene00012493	Y20C6A.3	Y20C6A.3
0.0543274845332054	AGIWUR36562
CB4856	CT	V	17395674	17398034	WBGene00005339	srh-120	T27C5.1	0.144687375019166
AGIWUR25062
CB4856	CT	V	17400123	17401774	WBGene00005944	srx-53	T27C5.2	0.0494231255937825
AGIWUR2034
CB4856	CT	V	17404611	17405823	WBGene00023512	srz-51	T27C5.11
-0.0811617910527884	AGIWUR11238
CB4856	CT	V	17406306	17407503	WBGene00012078	srz-50	T27C5.4	-0.452467785787357
AGIWUR42655
CB4856	CT	V	17410255	17411705	WBGene00005349	srh-132	T27C5.5	0.498047273389041
AGIWUR27579
CB4856	CT	V	17412602	17413098	WBGene00012079	T27C5.6	T27C5.6	-0.101084274202542
AGIWUR37517
CB4856	CT	V	17413546	17414749	WBGene00012080	clec-244	T27C5.7
0.0945177640967589	AGIWUR27963;AGIWUR29663;AGIWUR32941
CB4856	CT	V	17415278	17416743	WBGene00044059	T27C5.12	T27C5.12
0.1147610721435	AGIWUR25726
CB4856	CT	V	17417133	17418391	WBGene00012081	T27C5.8	T27C5.8	0.0975722686777418
AGIWUR37095
CB4856	CT	V	17419156	17420298	WBGene00044096	srh-96	T27C5.14
0.104016834417084	AGIWUR40955
CB4856	CT	V	17429701	17430166	WBGene00012083	T27C5.10	T27C5.10
-0.0920978614036461	AGIWUR4231;AGIWUR13140;AGIWUR38544

CB4856 CT V 17431203 17432501 WBGene00044100 srbc-27 F20E11.15
-0.0505116205078506 AGIWUR43044
CB4856 CT V 17432993 17435209 WBGene00044101 srbc-28 F20E11.16
0.208107573943751 AGIWUR5255
CB4856 CT V 17435678 17436592 WBGene00206495 F20E11.18 F20E11.18
0.0671881755059163 AGIWUR14662
CB4856 CT V 17436985 17438417 WBGene00008982 srsx-2 F20E11.2
-0.665928921643208 AGIWUR3063
CB4856 CT V 17442581 17445668 WBGene00005374 srh-158 F20E11.13
0.104810368398025 AGIWUR14378;AGIWUR40459
CB4856 CT V 17446348 17447636 WBGene00005376 srh-160 F20E11.3
-0.0364155030285076 AGIWUR21027
CB4856 CT V 17448332 17449590 WBGene00005373 srh-157 F20E11.8
0.00952412706797512 AGIWUR8991;AGIWUR31658
CB4856 CT V 17452676 17453805 WBGene00005377 srh-161 F20E11.14
-0.128946527476842 AGIWUR10052
CB4856 CT V 17458533 17460161 WBGene00008981 srz-48 F20E11.1
0.0255093538827467 AGIWUR1569
CB4856 CT V 17460813 17462432 WBGene00008983 F20E11.5 F20E11.5
-0.0193621687406393 AGIWUR25782
CB4856 CT V 17464578 17466730 WBGene00008984 F20E11.7 F20E11.7
-0.108261794265648 AGIWUR36820
CB4856 CT V 17472319 17473481 WBGene00005333 srh-114 F08E10.8
-0.0975159807317834 AGIWUR23613
CB4856 CT V 17473999 17475471 WBGene00005341 srh-123 F08E10.3
0.127675984156523 AGIWUR43710
CB4856 CT V 17476790 17478108 WBGene00008574 srbc-61 F08E10.2
0.078592781627961 AGIWUR37212
CB4856 CT V 17478333 17479386 WBGene00005329 srh-110 F08E10.4
-0.191235940774312 AGIWUR5026
CB4856 CT V 17480851 17481855 WBGene00005459 srh-253 F08E10.5
-0.194801727858007 AGIWUR44105
CB4856 CT V 17487346 17488833 WBGene00010536 K03D7.9 K03D7.9 0.148055172140996
AGIWUR27680
CB4856 CT V 17489502 17491609 WBGene00010535 K03D7.8 K03D7.8 0.267891713757692
AGIWUR35923
CB4856 CT V 17492077 17493832 WBGene00010534 fbxa-102 K03D7.7
-0.0466043317631879 AGIWUR25951
CB4856 CT V 17494306 17496171 WBGene00005337 srh-118 K03D7.6 -0.0352415053521273

AGIWUR33420
CB4856	CT	V	17499044	17505021	WBGene00014796	K03D7.3	K03D7.3	0.0531149961843269
AGIWUR22757
CB4856	CT	V	17508793	17510214	WBGene00005779	srw-32	K03D7.11
0.0700927683681547	AGIWUR28798
CB4856	CT	V	17511533	17519857	WBGene00005792	srw-45	K03D7.10
-0.0201143872633587	AGIWUR3827;AGIWUR5632;AGIWUR10237;AGIWUR11287;AGIWUR42706
CB4856	CT	V	17521037	17527479	WBGene00007672	fbxa-136	C18D4.2
0.695973518722219	AGIWUR23664;AGIWUR26139
CB4856	CT	V	17528029	17529910	WBGene00007678	srz-90	C18D4.9	0.11703209510067
AGIWUR27577
CB4856	CT	V	17530303	17530714	WBGene00219493	C18D4.12	C18D4.12
-0.092690681858274	AGIWUR10696
CB4856	CT	V	17530813	17531510	WBGene00007674	C18D4.4	C18D4.4	0.0573574005102468
AGIWUR29903
CB4856	CT	V	17532353	17532786	WBGene00007673	C18D4.3	C18D4.3	-0.0423487055982879
AGIWUR30181
CB4856	CT	V	17535615	17538373	WBGene00007675	C18D4.6	C18D4.6	-0.0598673229508604
AGIWUR10351;AGIWUR42998
CB4856	CT	V	17540595	17542272	WBGene00014679	srz-25	C18D4.5	0.529522436664029
AGIWUR13682
CB4856	CT	V	17542719	17544108	WBGene00007676	srz-57	C18D4.7	-0.0901503107445398
AGIWUR32653
CB4856	CT	V	17544175	17546974	WBGene00007677	C18D4.8	C18D4.8	0.304794650570354
AGIWUR41632
CB4856	CT	V	17547383	17549288	WBGene00045293	fbxa-175	C38D9.12
0.0269035419006114	AGIWUR34376
CB4856	CT	V	17549675	17551196	WBGene00008017	fbxa-176	C38D9.9
0.14490275621432	AGIWUR14301
CB4856	CT	V	17555854	17558477	WBGene00008015	fbxa-174	C38D9.7
-0.0025968803396017	AGIWUR20461
CB4856	CT	V	17563171	17566003	WBGene00008009	fbxa-171	C38D9.1
-0.187944642408002	AGIWUR22354
CB4856	CT	V	17567513	17571603	WBGene00008010	C38D9.2	C38D9.2	0.292128919725835
AGIWUR10796
CB4856	CT	V	17584793	17585876	WBGene00008012	fbxa-133	C38D9.4
-0.756778904606649	AGIWUR24992
CB4856	CT	V	17592387	17594243	WBGene00012403	srz-45	Y6G8.1	-0.0592245050412519
AGIWUR24279

CB4856 CT V 17594349 17598971 WBGene00012404 Y6G8.2 Y6G8.2 0.969668392470167
AGIWUR37650
CB4856 CT V 17599978 17600872 WBGene00050974 Y6G8.9 Y6G8.9 -0.228934200601832
AGIWUR2079;AGIWUR23739
CB4856 CT V 17601820 17602954 WBGene00012405 ztf-25 Y6G8.3 0.0224390166186854
AGIWUR37324
CB4856 CT V 17603185 17603945 WBGene00077459 Y6G8.10 Y6G8.10 -0.0974097200074091
AGIWUR11426
CB4856 CT V 17607379 17607690 WBGene00077460 Y6G8.11 Y6G8.11 0.0653451604227575
AGIWUR1681;AGIWUR13086;AGIWUR21320;AGIWUR33835;AGIWUR39121
CB4856 CT V 17608097 17609597 WBGene00012406 srz-91 Y6G8.4 0.142827697661105
AGIWUR13792
CB4856 CT V 17613792 17614357 WBGene00138715 Y6G8.14 Y6G8.14 -0.0569926523623789
AGIWUR18983;AGIWUR32925
CB4856 CT V 17616473 17616921 WBGene00044208 Y6G8.6 Y6G8.6 -0.0846014172359748
AGIWUR4430;AGIWUR5423;AGIWUR16001;AGIWUR40934;AGIWUR41144
CB4856 CT V 17620194 17622750 WBGene00045501 Y6G8.8 Y6G8.8 0.92877762787363
AGIWUR7206;AGIWUR18354
CB4856 CT V 17625127 17627598 WBGene00195165 F57G4.11 F57G4.11
1.1048404572395 AGIWUR30502
CB4856 CT V 17628866 17630215 WBGene00045506 F57G4.10 F57G4.10
0.11623425782751 AGIWUR2368;AGIWUR2435;AGIWUR18799;AGIWUR20217
CB4856 CT V 17637982 17640042 WBGene00010209 fbxa-191 F57G4.4
-0.253897362153699 AGIWUR34789
CB4856 CT V 17640672 17650527 WBGene00010212 fbxa-192 F57G4.8
-0.050050950160534
AGIWUR4316;AGIWUR4368;AGIWUR7320;AGIWUR14928;AGIWUR17320;AGIWUR20534;AGIWUR22976;AGIWUR25735;AGIWUR27646;AG
IWUR31741;AGIWUR41603;AGIWUR42867
CB4856 CT V 17653482 17655281 WBGene00010295 fbxa-193 F59A1.9
-0.205171031423905 AGIWUR276
CB4856 CT V 17655581 17656654 WBGene00010294 fbxa-129 F59A1.8
-0.171865849061008 AGIWUR9387;AGIWUR44072
CB4856 CT V 17656892 17658198 WBGene00010293 fbxa-108 F59A1.7
-0.106659552707502 AGIWUR17141;AGIWUR41177
CB4856 CT V 17660303 17661810 WBGene00010296 dgat-2 F59A1.10
-0.953848286389935 AGIWUR3725
CB4856 CT V 17661802 17665475 WBGene00010297 F59A1.11 F59A1.11
-0.129383780238144 AGIWUR4003
CB4856 CT V 17668882 17671330 WBGene00006148 str-89 F59A1.4 0.103006298886445

AGIWUR22398
CB4856 CT V 17671700 17673443 WBGene00006151 str-93 F59A1.14
-0.0037635916435752 AGIWUR15703
CB4856 CT V 17674946 17676653 WBGene00006150 str-92 F59A1.3 0.0566723295300591
AGIWUR33147
CB4856 CT V 17678358 17680208 WBGene00014779 F59A1.2 F59A1.2 0.0133827974558876
AGIWUR24205;AGIWUR27684
CB4856 CT V 17680661 17683907 WBGene00010300 F59A1.15 F59A1.15
0.334337405884932 AGIWUR40297
CB4856 CT V 17684149 17684968 WBGene00010301 F59A1.16 F59A1.16
-0.81006215226695 AGIWUR20039
CB4856 CT V 17685192 17686074 WBGene00010292 F59A1.6 F59A1.6 -0.27621472954424
AGIWUR24435
CB4856 CT V 17687211 17690994 WBGene00010299 F59A1.13 F59A1.13
-0.117833086026812 AGIWUR39181
CB4856 CT V 17692502 17695827 WBGene00012515 Y26G10.1 Y26G10.1
-1.0718860211798 AGIWUR5760
CB4856 CT V 17701212 17703285 WBGene00005255 srh-32 Y26G10.2
-0.14027285043137 AGIWUR10909
CB4856 CT V 17704362 17706117 WBGene00050973 Y26G10.3 Y26G10.3
0.0156791440533173 AGIWUR22451;AGIWUR38578
CB4856 CT V 17706114 17706875 WBGene00012305 W06D12.6 W06D12.6
-0.319609595370878 AGIWUR29688
CB4856 CT V 17707184 17709823 WBGene00005254 srh-31 W06D12.7
0.0430433200252418 AGIWUR7156
CB4856 CT V 17710040 17714377 WBGene00006685 twk-33 W06D12.5
-0.195712544353153 AGIWUR23332;AGIWUR39569;AGIWUR43456
CB4856 CT V 17718947 17721293 WBGene00006137 str-77 W06D12.4
0.0811780002324864 AGIWUR8320
CB4856 CT V 17725004 17726818 WBGene00001397 fat-5 W06D12.3
0.645569757893607 AGIWUR14736;AGIWUR20562;AGIWUR22243;AGIWUR27469
CB4856 CT V 17726799 17730381 WBGene00006692 twk-42 W06D12.2
-0.35849001495151 AGIWUR35124
CB4856 CT V 17733568 17739899 WBGene00012304 cutl-4 W06D12.1
-0.0813052870771633 AGIWUR5269;AGIWUR9713;AGIWUR10938
CB4856 CT V 17743651 17747059 WBGene00006688 twk-36 R12G8.2 0.0673574053145645
AGIWUR29554
CB4856 CT V 17747966 17749243 WBGene00011254 R12G8.1 R12G8.1 -0.100073868993075
AGIWUR20029

CB4856	CT	V	17755892	17764751	WBGene00004003	pgp-9	C47A10.1	0.0506573511167581	AGIWUR15356
CB4856	CT	V	17767549	17769621	WBGene00005489	srh-284	C47A10.3	0.211009369870842	AGIWUR13539
CB4856	CT	V	17769653	17773208	WBGene00008126	sre-21	C47A10.4	0.140798610935677	AGIWUR6720;AGIWUR10002;AGIWUR10999
CB4856	CT	V	17774187	17777227	WBGene00008127	ddo-1	C47A10.5	0.198252380525098	AGIWUR14884
CB4856	CT	V	17777924	17779487	WBGene00008129	C47A10.12	C47A10.12	0.682299072718495	AGIWUR12728
CB4856	CT	V	17780448	17783639	WBGene00008128	srab-12	C47A10.6	0.105900798423761	AGIWUR29939
CB4856	CT	V	17789494	17789738	WBGene00050897	C47A10.13	C47A10.13	-0.170924973402038	AGIWUR8974;AGIWUR10304;AGIWUR21806;AGIWUR28302;AGIWUR42390
CB4856	CT	V	17790491	17792470	WBGene00005494	srh-290	C47A10.11	0.0603228555480975	AGIWUR19006
CB4856	CT	V	17792679	17794086	WBGene00005493	srh-289	C47A10.10	0.100839027491441	AGIWUR14885
CB4856	CT	V	17794909	17795755	WBGene00005492	srh-288	C47A10.7	-0.22279710097555	AGIWUR9355
CB4856	CT	V	17796200	17798889	WBGene00005909	srx-18	C47A10.8	0.158615245124221	AGIWUR744
CB4856	CT	V	17800838	17801906	WBGene00005491	srh-287	C47A10.9	0.0539983129893411	AGIWUR32425
CB4856	CT	V	17802410	17802619	WBGene00050893	Y94A7B.11	Y94A7B.11	-0.0422010460597573	AGIWUR8422;AGIWUR35883;AGIWUR43835
CB4856	CT	V	17802769	17803923	WBGene00005496	srh-292	Y94A7B.1	0.225854514395126	AGIWUR34250
CB4856	CT	V	17805486	17808611	WBGene00005495	srh-291	Y94A7B.3	-0.00242404211723013	AGIWUR7471
CB4856	CT	V	17810353	17812794	WBGene00005500	srh-296	Y94A7B.4	0.0163899325648931	AGIWUR21677
CB4856	CT	V	17818867	17821003	WBGene00005502	srh-298	Y94A7B.5	-0.198617117767487	AGIWUR44774
CB4856	CT	V	17822105	17823889	WBGene00005504	srh-300	Y94A7B.6	-0.0352172173095724	AGIWUR16526
CB4856	CT	V	17824910	17827774	WBGene00005505	srh-301	Y94A7B.8	-0.131550272288726	AGIWUR14106
CB4856	CT	V	17828610	17829983	WBGene00005508	srh-304	Y94A7B.9		

-0.0691012644987588	AGIWUR4451
CB4856	CT	V	17831650	17833418	WBGene00005507	srh-303	Y94A7B.7
0.00518158969885718	AGIWUR19659
CB4856	CT	V	17837360	17838119	WBGene00013341	Y59A8A.7	Y59A8A.7
0.0246958855773549	AGIWUR19908
CB4856	CT	V	17838831	17841188	WBGene00005506	srh-302	Y59A8A.5
-0.0711403209002661	AGIWUR38418
CB4856	CT	V	17841483	17844206	WBGene00005490	srh-286	Y59A8A.4
0.213251388752601	AGIWUR23633
CB4856	CT	V	17845739	17855989	WBGene00013340	tcc-1	Y59A8A.3
-0.139191620323471	AGIWUR2535;AGIWUR5566;AGIWUR7194;AGIWUR15447;AGIWUR21458;AGIWUR27254;AGIWUR32170
CB4856	CT	V	17861368	17870651	WBGene00000813	csn-1	Y59A8A.1
-0.258893613635738	AGIWUR10688;AGIWUR14162;AGIWUR37627
CB4856	CT	V	17871401	17879913	WBGene00013339	phf-14	Y59A8A.2
-0.104683847161589	AGIWUR31159
CB4856	CT	V	17879844	17888984	WBGene00007394	C06H5.6	C06H5.6	-0.249607472641274
AGIWUR7896;AGIWUR8502;AGIWUR8823;AGIWUR15190;AGIWUR16651;AGIWUR18629;AGIWUR21138;AGIWUR25545;AGIWUR28925;AGIWUR35720;AGIWUR40621;AGIWUR44512
CB4856	CT	V	17892426	17892706	WBGene00045346	C06H5.9	C06H5.9	0.00593674562324864
AGIWUR3364;AGIWUR10273;AGIWUR10645;AGIWUR27916;AGIWUR42380
CB4856	CT	V	17895268	17896804	WBGene00007392	fbxa-156	C06H5.1
-0.359516305506164	AGIWUR25046
CB4856	CT	V	17897546	17897746	WBGene00219446	C06H5.13	C06H5.13
0.0106752309156981	AGIWUR952;AGIWUR14193;AGIWUR20483;AGIWUR22689;AGIWUR33984
CB4856	CT	V	17899803	17901913	WBGene00007393	fbxa-157	C06H5.2
0.168226801341326	AGIWUR32833
CB4856	CT	V	17910304	17917241	WBGene00007395	dcar-1	C06H5.7	0.330907882225993
AGIWUR4617;AGIWUR10116;AGIWUR43125
CB4856	CT	V	17921776	17924185	WBGene00008442	E01B7.2	E01B7.2	-0.197115206451352
AGIWUR9023
CB4856	CT	V	17924916	17934225	WBGene00008441	usp-50	E01B7.1	-0.127731617435198
AGIWUR11877;AGIWUR13337
CB4856	CT	V	17936332	17949184	WBGene00001080	dpy-21	Y59A8B.1
0.319933021583985	AGIWUR39389
CB4856	CT	V	17951348	17952820	WBGene00013356	Y59A8B.24	Y59A8B.24
0.0369070432885845	AGIWUR42293
CB4856	CT	V	17954054	17966847	WBGene00013355	gck-3	Y59A8B.23
-0.0143245708851278	AGIWUR29083
CB4856	CT	V	17968510	17980082	WBGene00013354	snx-6	Y59A8B.22

-0.170836175542762	AGIWUR31388
CB4856	CT	V	17986260	17994546	WBGene00013353	Y59A8B.21	Y59A8B.21
-0.202116982613489	AGIWUR40332
CB4856	CT	V	17995347	18001368	WBGene00013352	lon-8	Y59A8B.20
-0.291316770126851	AGIWUR8675;AGIWUR9102;AGIWUR15282;AGIWUR17603;AGIWUR19833
CB4856	CT	V	18007230	18009041	WBGene00013351	Y59A8B.19	Y59A8B.19
-0.0369649903863154	AGIWUR17315
CB4856	CT	V	18010017	18010837	WBGene00045459	Y59A8B.26	Y59A8B.26
-0.116957814883401	AGIWUR12442;AGIWUR21434
CB4856	CT	V	18012413	18014216	WBGene00005350	srh-133	Y59A8B.3
0.119746077686633	AGIWUR8992
CB4856	CT	V	18014641	18017326	WBGene00005351	srh-134	Y59A8B.4
0.191262122786235	AGIWUR5306
CB4856	CT	V	18017904	18020014	WBGene00005511	srh-307	Y59A8B.5
-0.108899936314801	AGIWUR5710;AGIWUR35581
CB4856	CT	V	18022479	18042353	WBGene00013343	prp-6	Y59A8B.6
-0.116413129070978	AGIWUR7680
CB4856	CT	V	18043571	18052323	WBGene00013344	ebp-1	Y59A8B.7
-0.220769628211396	AGIWUR16212
CB4856	CT	V	18070021	18081957	WBGene00013347	Y59A8B.10	Y59A8B.10
0.144279553467552	AGIWUR38201
CB4856	CT	V	18082406	18085127	WBGene00013348	fbxa-106	Y59A8B.11
0.282869007624985	AGIWUR14652
CB4856	CT	V	18085630	18086225	WBGene00013349	Y59A8B.12	Y59A8B.12
-0.360702185268694	AGIWUR24593
CB4856	CT	V	18087123	18097996	WBGene00013350	slr-2	Y59A8B.13
-0.431516806582531	AGIWUR5909
CB4856	CT	V	18098676	18106868	WBGene00003919	par-4	Y59A8B.14
-0.0816620376746018	AGIWUR43625
CB4856	CT	V	18108269	18114755	WBGene00012562	Y37H2A.1	Y37H2A.1
-0.107039451027088	AGIWUR15257
CB4856	CT	V	18115402	18123594	WBGene00050935	Y37H2A.16	Y37H2A.16
-0.22371939616202	AGIWUR5789;AGIWUR9443;AGIWUR11169;AGIWUR17775;AGIWUR27302;AGIWUR29776;AGIWUR41474
CB4856	CT	V	18123939	18125245	WBGene00012564	fbxa-107	Y37H2A.4
0.152414740391663	AGIWUR43709
CB4856	CT	V	18125375	18127205	WBGene00012565	fbxa-210	Y37H2A.5
-0.369544481481888	AGIWUR20368
CB4856	CT	V	18127519	18128885	WBGene00012566	fbxa-211	Y37H2A.6
-0.0653026199193985	AGIWUR12070;AGIWUR24650;AGIWUR37600

CB4856 CT V 18129171 18131125 WBGene00044758 Y37H2A.12 Y37H2A.12
-0.420794633376824 AGIWUR39737
CB4856 CT V 18131667 18133232 WBGene00219210 Y37H2A.18 Y37H2A.18
-0.135780308317237 AGIWUR26503
CB4856 CT V 18134722 18136022 WBGene00012568 fbxa-212 Y37H2A.8
0.218762969421277 AGIWUR25803;AGIWUR41974
CB4856 CT V 18136704 18137714 WBGene00012569 fbxc-46 Y37H2A.9
-0.00306681182368962 AGIWUR40984
CB4856 CT V 18138995 18140687 WBGene00012570 Y37H2A.10 Y37H2A.10
-0.126122937664524 AGIWUR10504
CB4856 CT V 18141461 18142577 WBGene00012571 Y37H2A.11 Y37H2A.11
0.029865303013846 AGIWUR28154
CB4856 CT V 18142849 18143543 WBGene00045417 Y37H2A.13 Y37H2A.13
-0.785164138623415 AGIWUR19;AGIWUR2328;AGIWUR27028;AGIWUR27310
CB4856 CT V 18143716 18144720 WBGene00045416 Y37H2A.14 Y37H2A.14
-0.175000522559703 AGIWUR25981
CB4856 CT V 18146965 18154396 WBGene00206536 C14A6.16 C14A6.16
0.0457496732895724 AGIWUR26918
CB4856 CT V 18159652 18161339 WBGene00050956 C14A6.13 C14A6.13
0.00946480686018258 AGIWUR13918
CB4856 CT V 18162034 18162668 WBGene00050955 C14A6.12 C14A6.12
-0.207250383704841 AGIWUR3236;AGIWUR21166;AGIWUR21466
CB4856 CT V 18163439 18164858 WBGene00007569 C14A6.5 C14A6.5 0.124161611939202
AGIWUR38662
CB4856 CT V 18165294 18167261 WBGene00007570 C14A6.6 C14A6.6
-0.00901265723201661 AGIWUR43342
CB4856 CT V 18167634 18168450 WBGene00007571 C14A6.7 C14A6.7 0.037699701240113
AGIWUR22025
CB4856 CT V 18169087 18171618 WBGene00007572 C14A6.8 C14A6.8 0.114733402826517
AGIWUR30547
CB4856 CT V 18172484 18178759 WBGene00007853 C31G12.4 C31G12.4
0.0922665715104918 AGIWUR25686;AGIWUR31906
CB4856 CT V 18179943 18181373 WBGene00007850 C31G12.1 C31G12.1
-0.316686595875069 AGIWUR44939
CB4856 CT V 18185210 18185461 WBGene00007852 C31G12.3 C31G12.3
-0.326622961585402 AGIWUR11940
CB4856 CT V 18186111 18188331 WBGene00007851 clec-245 C31G12.2
-0.664029181641412 AGIWUR25381
CB4856 CT V 18189560 18191047 WBGene00008892 clec-246 F16H6.2

-0.566397113089175	AGIWUR2615;AGIWUR21493;AGIWUR36344;AGIWUR38574
CB4856	CT	V	18191412	18194167	WBGene00008891	clec-42	F16H6.1	-0.0095166301991263
AGIWUR6841
CB4856	CT	V	18195306	18197863	WBGene00008893	F16H6.3	F16H6.3	0.198115431145231
AGIWUR7554
CB4856	CT	V	18198721	18205815	WBGene00008894	F16H6.4	F16H6.4	-0.0140517648220055
AGIWUR11811;AGIWUR29561
CB4856	CT	V	18207064	18209171	WBGene00008897	F16H6.7	F16H6.7	-0.0454331443654787
AGIWUR32034
CB4856	CT	V	18217084	18222299	WBGene00008899	F16H6.9	F16H6.9	-0.339931299634475
AGIWUR12021
CB4856	CT	V	18222746	18224447	WBGene00008900	F16H6.10	F16H6.10
-0.291126998719095	AGIWUR29693
CB4856	CT	V	18227037	18231455	WBGene00012572	Y37H2B.1	Y37H2B.1
-0.0298815775696157	AGIWUR22690
CB4856	CT	V	18231635	18237223	WBGene00014821	R10E8.7	R10E8.7	-0.405343032691423
AGIWUR28066;AGIWUR30825
CB4856	CT	V	18238122	18240736	WBGene00011213	R10E8.8	R10E8.8	0.578104196177257
AGIWUR20037
CB4856	CT	V	18242339	18252416	WBGene00011207	R10E8.1	R10E8.1	-0.0357988320963587
AGIWUR5910;AGIWUR8723;AGIWUR17037;AGIWUR20949;AGIWUR20966
CB4856	CT	V	18253794	18254403	WBGene00011208	R10E8.2	R10E8.2	0.126041842602447
AGIWUR22380
CB4856	CT	V	18254783	18257067	WBGene00011211	R10E8.5	R10E8.5	-0.0652024205565667
AGIWUR30162
CB4856	CT	V	18259270	18266370	WBGene00011212	R10E8.6	R10E8.6	0.90613344414666
AGIWUR11589
CB4856	CT	V	18267292	18268844	WBGene00012574	fbxa-213	Y37H2C.3
0.140360934248438	AGIWUR33649
CB4856	CT	V	18269616	18269985	WBGene00004812	skr-6	Y37H2C.2
0.112816156212231	AGIWUR27438
CB4856	CT	V	18270703	18272256	WBGene00012575	Y37H2C.4	Y37H2C.4
-0.417516940540684	AGIWUR20744
CB4856	CT	V	18272889	18277694	WBGene00012573	Y37H2C.1	Y37H2C.1
0.390915745003286	AGIWUR17827
CB4856	CT	V	18282897	18290590	WBGene00013058	clec-247	Y51A2A.1
-0.104760519479881	AGIWUR43134
CB4856	CT	V	18290942	18291348	WBGene00013060	Y51A2A.4	Y51A2A.4
0.108873083369532	AGIWUR16311

CB4856	CT	V	18292595	18292944	WBGene00077445	Y51A2A.12	Y51A2A.12
0.0215777421753023	AGIWUR755;AGIWUR23757;AGIWUR31475;AGIWUR41999;AGIWUR42533
CB4856	CT	V	18298806	18300713	WBGene00013061	Y51A2A.5	Y51A2A.5
0.0255110823509721	AGIWUR41693
CB4856	CT	V	18309386	18310203	WBGene00014906	fbxa-109	Y51A2A.8
-0.0165501926359348	AGIWUR38308;AGIWUR43586
CB4856	CT	V	18310924	18311985	WBGene00014907	fbxa-117	Y51A2A.9
-0.17169989631326	AGIWUR23063
CB4856	CT	V	18318595	18319744	WBGene00007182	fbxb-119	B0462.3
0.00435118539237367
AGIWUR6162;AGIWUR11568;AGIWUR13340;AGIWUR14758;AGIWUR16201;AGIWUR17658;AGIWUR29562;AGIWUR40018
CB4856	CT	V	18320110	18321527	WBGene00005580	sri-68	B0462.2	-0.224079371463535
AGIWUR30149;AGIWUR39795
CB4856	CT	V	18325035	18328791	WBGene00007181	B0462.1	B0462.1	1.13496691496006
AGIWUR32448
CB4856	CT	V	18329112	18331179	WBGene00007183	B0462.4	B0462.4	0.282884989612372
AGIWUR14082
CB4856	CT	V	18351021	18352672	WBGene00119201	C08E8.11	C08E8.11
0.0660400475573546	AGIWUR33378
CB4856	CT	V	18353510	18355842	WBGene00007438	C08E8.2	C08E8.2	-0.210118033796467
AGIWUR19655;AGIWUR23477;AGIWUR40483
CB4856	CT	V	18358035	18359555	WBGene00045496	C08E8.8	C08E8.8	0.0853694260523206
AGIWUR1498;AGIWUR11590;AGIWUR13685;AGIWUR14012;AGIWUR37565
CB4856	CT	V	18359818	18359988	WBGene00007441	C08E8.5	C08E8.5	0.110978447265624
AGIWUR31154
CB4856	CT	V	18361051	18362543	WBGene00007437	C08E8.1	C08E8.1	0.118074066658965
AGIWUR13037;AGIWUR22262;AGIWUR29468
CB4856	CT	V	18364858	18365224	WBGene00050891	C08E8.9	C08E8.9	0.0667338656542249
AGIWUR12572;AGIWUR29261;AGIWUR38952;AGIWUR42879;AGIWUR44736
CB4856	CT	V	18368014	18368422	WBGene00007442	C08E8.6	C08E8.6	0.0420086906627173
AGIWUR29226
CB4856	CT	V	18371846	18373528	WBGene00007440	C08E8.4	C08E8.4	0.814030985705063
AGIWUR33396;AGIWUR42447
CB4856	CT	V	18374494	18376755	WBGene00013066	Y51A2B.2	Y51A2B.2
-0.579621952745072	AGIWUR530
CB4856	CT	V	18379119	18379289	WBGene00013071	Y51A2B.8	Y51A2B.8
-0.0978617369517432	AGIWUR11261;AGIWUR15571;AGIWUR26511;AGIWUR35990;AGIWUR37522;AGIWUR43960
CB4856	CT	V	18380620	18382690	WBGene00013067	nhr-288	Y51A2B.3
-0.0123982091085999	AGIWUR6038

CB4856 CT V 18386252 18389146 WBGene00013068 Y51A2B.4 Y51A2B.4
0.0532791093819522 AGIWUR28115
CB4856 CT V 18389280 18391337 WBGene00013069 Y51A2B.5 Y51A2B.5
-0.240402919776802 AGIWUR3971
CB4856 CT V 18392007 18394303 WBGene00013070 Y51A2B.6 Y51A2B.6
-0.00982626623518357 AGIWUR20290
CB4856 CT V 18395338 18395749 WBGene00050907 Y51A2B.9 Y51A2B.9
0.0483406230391292 AGIWUR15787;AGIWUR15989;AGIWUR19344;AGIWUR25724;AGIWUR40333
CB4856 CT V 18398450 18400374 WBGene00005828 srw-81 Y51A2B.7
-0.0538456069537485 AGIWUR14210
CB4856 CT V 18406896 18408138 WBGene00014984 ZK262.12 ZK262.12
0.0563991250099374 AGIWUR29494;AGIWUR39668
CB4856 CT V 18409043 18410242 WBGene00005417 srh-207 ZK262.1 -0.0687424186231418
AGIWUR24982
CB4856 CT V 18410758 18412987 WBGene00013949 ZK262.2 ZK262.2 -0.0253853536152722
AGIWUR1816
CB4856 CT V 18414351 18416861 WBGene00013950 ZK262.3 ZK262.3 0.522017556123105
AGIWUR39692
CB4856 CT V 18417749 18419893 WBGene00005409 srh-198 ZK262.13
0.112709150500308 AGIWUR10509;AGIWUR15365
CB4856 CT V 18421097 18423477 WBGene00005614 srj-26 ZK262.10
0.0527723423548263 AGIWUR12493;AGIWUR38321;AGIWUR40538
CB4856 CT V 18423883 18424207 WBGene00013951 ZK262.4 ZK262.4
-0.00883522060429875 AGIWUR19656
CB4856 CT V 18426580 18430537 WBGene00005830 srw-83 ZK262.6 0.0789558437016789
AGIWUR18266
CB4856 CT V 18431750 18434172 WBGene00005829 srw-82 ZK262.7 0.191025653017148
AGIWUR4528
CB4856 CT V 18440578 18441340 WBGene00013954 ZK262.9 ZK262.9 -0.18524060285455
AGIWUR34089
CB4856 CT V 18444418 18447019 WBGene00013944 ZK228.1 ZK228.1 0.125789412584298
AGIWUR6424
CB4856 CT V 18455355 18455934 WBGene00050900 ZK228.11 ZK228.11
-0.0770827783774806 AGIWUR27728
CB4856 CT V 18458238 18461231 WBGene00013946 ZK228.3 ZK228.3 -1.02960710466009
AGIWUR704
CB4856 CT V 18462804 18465320 WBGene00013947 ZK228.4 ZK228.4 -0.95018677530458
AGIWUR27462
CB4856 CT V 18471985 18473530 WBGene00005397 srh-182 ZK228.5 -0.19256608347436

AGIWUR27804
CB4856	CT	V	18474523	18476268	WBGene00005396	srh-181	ZK228.6	0.0613997274586264
AGIWUR6220
CB4856	CT	V	18476390	18478061	WBGene00005394	srh-179	ZK228.7	-0.269190295194297
AGIWUR29334
CB4856	CT	V	18478467	18480331	WBGene00005393	srh-178	ZK228.8	0.178827975190013
AGIWUR6209
CB4856	CT	V	18481131	18487258	WBGene00013083	Y51A2D.18	Y51A2D.18
-0.391843097322763	AGIWUR19122
CB4856	CT	V	18492265	18508201	WBGene00004830	slo-1	Y51A2D.19
0.0143837484794993	AGIWUR35207;AGIWUR43931
CB4856	CT	V	18508873	18512632	WBGene00013072	Y51A2D.1	Y51A2D.1
-0.142664293523289	AGIWUR24034
CB4856	CT	V	18516789	18523875	WBGene00013073	hmit-1.1	Y51A2D.4
1.16181582101607	AGIWUR9464
CB4856	CT	V	18524760	18529156	WBGene00013074	hmit-1.2	Y51A2D.5
-0.319560103058961	AGIWUR26939
CB4856	CT	V	18533924	18549129	WBGene00013075	Y51A2D.7	Y51A2D.7
0.0703967999459767	AGIWUR40539
CB4856	CT	V	18550022	18553867	WBGene00013076	Y51A2D.8	Y51A2D.8
-0.351010612173567	AGIWUR41015
CB4856	CT	V	18555387	18556643	WBGene00013077	ttr-24	Y51A2D.9
0.029109320960357	AGIWUR9673
CB4856	CT	V	18556714	18558099	WBGene00013078	ttr-25	Y51A2D.10
0.127868783263759	AGIWUR27649
CB4856	CT	V	18558748	18560308	WBGene00013079	ttr-26	Y51A2D.11
0.00633311489393051	AGIWUR38389
CB4856	CT	V	18562178	18564886	WBGene00005191	srg-34	Y51A2D.12
-0.303051258639384	AGIWUR5864;AGIWUR15788;AGIWUR28658
CB4856	CT	V	18571833	18573026	WBGene00044210	Y51A2D.21	Y51A2D.21
-0.0861568740797969	AGIWUR69;AGIWUR12741;AGIWUR19445;AGIWUR23107;AGIWUR31627
CB4856	CT	V	18573056	18579784	WBGene00013080	Y51A2D.13	Y51A2D.13
0.665312885419453	AGIWUR33983
CB4856	CT	V	18582658	18582963	WBGene00013081	Y51A2D.14	Y51A2D.14
0.17302726569503	AGIWUR35808
CB4856	CT	V	18592754	18614101	WBGene00013082	grdn-1	Y51A2D.15
0.341221273108118	AGIWUR699;AGIWUR3278;AGIWUR40624
CB4856	CT	V	18616599	18620043	WBGene00003660	nhr-70	Y51A2D.17
-0.137395746056228	AGIWUR6583

CB4856	CT	V	18620057	18623093	WBGene00013479	Y69H2.1	Y69H2.1	-0.204683569601759
AGIWUR4951
CB4856	CT	V	18623198	18626852	WBGene00050884	Y69H2.15	Y69H2.15
-0.201500018861401	AGIWUR11539;AGIWUR42398
CB4856	CT	V	18628680	18636348	WBGene00013480	egas-3	Y69H2.2	-0.344666666675896
AGIWUR12373
CB4856	CT	V	18638484	18639591	WBGene00013489	col-42	Y69H2.14
-0.226245535831239	AGIWUR4525;AGIWUR33265
CB4856	CT	V	18640337	18648392	WBGene00013486	egas-1	Y69H2.11
0.0178103879248237	AGIWUR27908
CB4856	CT	V	18650343	18656165	WBGene00013487	egas-2	Y69H2.12
-0.169966207611634	AGIWUR11285
CB4856	CT	V	18656094	18664116	WBGene00013481	Y69H2.3	Y69H2.3	-0.386632423506507
AGIWUR14840;AGIWUR15424;AGIWUR24446;AGIWUR25250;AGIWUR30766;AGIWUR39487;AGIWUR40523
CB4856	CT	V	18670315	18684360	WBGene00013485	Y69H2.10	Y69H2.10
0.178613235314703	AGIWUR10385;AGIWUR23560;AGIWUR34119
CB4856	CT	V	18685060	18686349	WBGene00077455	Y69H2.17	Y69H2.17
-0.0312529756088598	AGIWUR9288;AGIWUR16970;AGIWUR45036
CB4856	CT	V	18686315	18689843	WBGene00006714	ubc-19	Y69H2.6	-0.0934236313441037
AGIWUR6785
CB4856	CT	V	18689989	18696069	WBGene00013482	Y69H2.7	Y69H2.7	-0.249578059900609
AGIWUR29711;AGIWUR41114
CB4856	CT	V	18701540	18706098	WBGene00013484	Y69H2.9	Y69H2.9	-0.25467952957478
AGIWUR13319
CB4856	CT	V	18706031	18706254	WBGene00077451	Y69H2.16	Y69H2.16
0.279998602923322	AGIWUR12659
CB4856	CT	V	18709810	18711696	WBGene00194680	Y17D7C.6	Y17D7C.6
-0.0544431052910772	AGIWUR37831
CB4856	CT	V	18713728	18714612	WBGene00050903	Y17D7C.4	Y17D7C.4
-0.0202128265327213	AGIWUR689;AGIWUR4289;AGIWUR8035;AGIWUR26888;AGIWUR35127
CB4856	CT	V	18716075	18719944	WBGene00012456	Y17D7C.2	Y17D7C.2
-0.0654019438755755	AGIWUR12235
CB4856	CT	V	18720589	18723771	WBGene00045366	Y17D7C.3	Y17D7C.3
-0.0303572625465334	AGIWUR22892;AGIWUR44345
CB4856	CT	V	18728703	18729374	WBGene00077655	asp-19	ZK384.6	0.226390956774997
AGIWUR5848
CB4856	CT	V	18731991	18732772	WBGene00013971	scl-19	ZK384.1	-0.131847825153445
AGIWUR33613
CB4856	CT	V	18732802	18734466	WBGene00013972	scl-20	ZK384.2	0.265156466333092

AGIWUR29469
CB4856 CT V 18738955 18742208 WBGene00045364 clec-250 F11D11.14
0.0804130736868967 AGIWUR14561;AGIWUR19691;AGIWUR42947
CB4856 CT V 18742427 18745266 WBGene00045363 F11D11.13 F11D11.13
-0.17422369100495 AGIWUR372;AGIWUR17003
CB4856 CT V 18745718 18746987 WBGene00008703 clec-251 F11D11.8
0.0569685993770292 AGIWUR24107
CB4856 CT V 18747943 18748826 WBGene00008702 clec-252 F11D11.7
0.00566589442536891 AGIWUR30950
CB4856 CT V 18749579 18750199 WBGene00045362 F11D11.12 F11D11.12
0.0109210390524375 AGIWUR1476;AGIWUR2080;AGIWUR33437;AGIWUR41696;AGIWUR44609
CB4856 CT V 18750163 18750691 WBGene00206492 F11D11.21 F11D11.21
0.175721227052083 AGIWUR1746
CB4856 CT V 18751267 18752299 WBGene00045361 clec-253 F11D11.11
0.00338749394672444 AGIWUR9867;AGIWUR17636
CB4856 CT V 18752482 18753136 WBGene00008701 F11D11.6 F11D11.6
0.0126659616476319 AGIWUR6810;AGIWUR14978;AGIWUR29426
CB4856 CT V 18753500 18754428 WBGene00008700 clec-254 F11D11.5
0.13829072940373 AGIWUR11388
CB4856 CT V 18755450 18756760 WBGene00050971 F11D11.15 F11D11.15
0.412563566496992 AGIWUR7429;AGIWUR24923
CB4856 CT V 18756846 18757678 WBGene00077442 F11D11.16 F11D11.16
-0.0530680729641171 AGIWUR4444;AGIWUR5749;AGIWUR14017;AGIWUR24396;AGIWUR44023
CB4856 CT V 18757982 18760285 WBGene00008698 F11D11.3 F11D11.3
0.0916077824510975 AGIWUR30167
CB4856 CT V 18761378 18763526 WBGene00008699 F11D11.4 F11D11.4
-0.465383726190309 AGIWUR35106
CB4856 CT V 18767754 18768414 WBGene00077659 F11D11.19 F11D11.19
0.135278580067718 AGIWUR24782
CB4856 CT V 18771690 18772639 WBGene00012454 clec-256 Y17D7B.6
0.0680395094174235 AGIWUR32361;AGIWUR35140
CB4856 CT V 18773061 18774024 WBGene00044892 clec-257 Y17D7B.8
-0.380375763455824 AGIWUR43865
CB4856 CT V 18775226 18776195 WBGene00012453 Y17D7B.5 Y17D7B.5
0.023998078545012 AGIWUR36694;AGIWUR37221
CB4856 CT V 18776198 18780216 WBGene00012452 Y17D7B.4 Y17D7B.4
0.232522187596206 AGIWUR2942
CB4856 CT V 18780490 18782605 WBGene00014562 Y17D7B.7 Y17D7B.7
-0.447252755850159

									AGIWUR13145;AGIWUR14363;AGIWUR22346;AGIWUR24800;AGIWUR34763;AGIWUR36743;AGIWUR37503;AGIWUR41056;AGIWUR44064;AGIWUR44944
CB4856	CT	V	18784954	18786788	WBGene00012451	Y17D7B.3	Y17D7B.3	-0.217631571048287	AGIWUR11530
CB4856	CT	V	18787806	18789617	WBGene00012450	Y17D7B.2	Y17D7B.2	0.153914170419974	AGIWUR8884
CB4856	CT	V	18790958	18794781	WBGene00012449	nhr-231	Y17D7B.1	0.0990985608086543	AGIWUR3281;AGIWUR3287
CB4856	CT	V	18796106	18800822	WBGene00008309	nhr-170	C54E10.5	0.144050927974593	AGIWUR4988
CB4856	CT	V	18804080	18806509	WBGene00005522	sri-10	C54E10.4	-0.0236288177488966	AGIWUR35247
CB4856	CT	V	18808069	18808670	WBGene00008310	C54E10.6	C54E10.6	0.0415140290669775	AGIWUR20520
CB4856	CT	V	18811005	18813481	WBGene00008308	C54E10.3	C54E10.3	0.188563959786004	AGIWUR32525
CB4856	CT	V	18815036	18818593	WBGene00008306	C54E10.1	C54E10.1	-0.185476878905177	AGIWUR12625
CB4856	CT	V	18819766	18831422	WBGene00008307	ncs-5	C54E10.2	0.192536445773113	AGIWUR7427
CB4856	CT	V	18832327	18837214	WBGene00012448	cyp-33D3	Y17D7A.4	0.410611136666114	AGIWUR43577
CB4856	CT	V	18839310	18845401	WBGene00003655	nhr-65	Y17D7A.3	-0.318983035605644	AGIWUR8708
CB4856	CT	V	18846817	18852729	WBGene00012446	nhr-230	Y17D7A.1	-0.123427161251636	AGIWUR23803;AGIWUR40110
CB4856	CT	V	18853127	18859221	WBGene00006945	wars-1	Y80D3A.1	-0.330005988616191	AGIWUR39065
CB4856	CT	V	18860287	18894974	WBGene00001258	emb-4	Y80D3A.2	-0.281047627474542	AGIWUR228;AGIWUR4092;AGIWUR9326;AGIWUR10162;AGIWUR34227;AGIWUR35514;AGIWUR38064;AGIWUR42024
CB4856	CT	V	18895317	18900532	WBGene00013584	nhr-243	Y80D3A.4	0.178569127630052	AGIWUR41496
CB4856	CT	V	18912038	18918109	WBGene00013585	cyp-42A1	Y80D3A.5	0.245286313714649	AGIWUR7466;AGIWUR41876;AGIWUR42942
CB4856	CT	V	18919296	18931379	WBGene00004236	ptr-22	Y80D3A.7	0.523320192470988	AGIWUR13202
CB4856	CT	V	18940641	18946905	WBGene00013586	Y80D3A.8	Y80D3A.8	-0.0539896291248625	AGIWUR41159;AGIWUR44894

CB4856 CT V 18953118 18957317 WBGene00013587 Y80D3A.9 Y80D3A.9
-0.153040439708427 AGIWUR42361
CB4856 CT V 18958240 18960172 WBGene00012702 ztf-20 Y39B6A.46
0.0568278299046465 AGIWUR22117
CB4856 CT V 18960969 18967842 WBGene00012701 Y39B6A.43 Y39B6A.43
-0.27567473967915 AGIWUR14234
CB4856 CT V 18968021 18969640 WBGene00012700 Y39B6A.42 Y39B6A.42
-0.0518181494116261 AGIWUR22324;AGIWUR30146
CB4856 CT V 18970500 18977976 WBGene00012699 Y39B6A.41 Y39B6A.41
-0.63467486382637 AGIWUR26695
CB4856 CT V 18979336 18981193 WBGene00012698 sws-1 Y39B6A.40
-0.0609946780921025 AGIWUR12800
CB4856 CT V 18981306 18985870 WBGene00012697 mrps-35 Y39B6A.39
-0.173614640345743 AGIWUR40852
CB4856 CT V 18986498 18990421 WBGene00012696 reps-1 Y39B6A.38
-0.202306619103311 AGIWUR11454;AGIWUR30291;AGIWUR39000
CB4856 CT V 18990850 18993799 WBGene00012695 Y39B6A.37 Y39B6A.37
-0.118120475902268 AGIWUR27863
CB4856 CT V 18993940 18998200 WBGene00012694 Y39B6A.36 Y39B6A.36
-0.203117874773728 AGIWUR3112
CB4856 CT V 18998345 19000918 WBGene00006567 tgt-2 Y39B6A.35
-0.0824444509374409 AGIWUR30400
CB4856 CT V 19001018 19001856 WBGene00012693 Y39B6A.34 Y39B6A.34
-0.0756916911680072 AGIWUR4047
CB4856 CT V 19001956 19006711 WBGene00012692 Y39B6A.33 Y39B6A.33
-0.240989492245784 AGIWUR11551
CB4856 CT V 19008635 19013833 WBGene00012690 Y39B6A.31 Y39B6A.31
0.239462940504785 AGIWUR12356
CB4856 CT V 19014573 19020001 WBGene00012689 Y39B6A.30 Y39B6A.30
-0.129404972145664 AGIWUR2723;AGIWUR3868;AGIWUR8560;AGIWUR19395;AGIWUR22745;AGIWUR25998;AGIWUR35972
CB4856 CT V 19020487 19024765 WBGene00012688 Y39B6A.29 Y39B6A.29
-0.13891912484825 AGIWUR29897
CB4856 CT V 19031742 19035255 WBGene00012686 Y39B6A.27 Y39B6A.27
-0.019624401763144 AGIWUR12883;AGIWUR21823;AGIWUR22555
CB4856 CT V 19035753 19037320 WBGene00012683 asp-17 Y39B6A.24
-0.960225731229281 AGIWUR28496
CB4856 CT V 19039058 19042605 WBGene00012682 asp-16 Y39B6A.23
-0.142787795843065 AGIWUR29027
CB4856 CT V 19043215 19044853 WBGene00012681 asp-15 Y39B6A.22

-0.0242569685319536 AGIWUR2429
CB4856 CT V 19047047 19048669 WBGene00012680 Y39B6A.21 Y39B6A.21
-0.674990581495303 AGIWUR26567
CB4856 CT V 19049461 19050825 WBGene00000214 asp-1 Y39B6A.20
0.0530125794027647 AGIWUR25099
CB4856 CT V 19060180 19067034 WBGene00006696 twk-46 Y39B6A.19
0.121263303925321 AGIWUR13015;AGIWUR14878;AGIWUR16757
CB4856 CT V 19068621 19074237 WBGene00012679 Y39B6A.18 Y39B6A.18
-0.206759908977475 AGIWUR34309
CB4856 CT V 19076654 19082641 WBGene00003685 nhr-95 Y39B6A.17
-0.0766960155035044 AGIWUR15473;AGIWUR22074;AGIWUR37574
CB4856 CT V 19087723 19092668 WBGene00012703 nhr-145 Y39B6A.47
-0.188691147112543 AGIWUR17417;AGIWUR35409
CB4856 CT V 19099085 19101108 WBGene00012678 Y39B6A.16 Y39B6A.16
-0.185353548607477 AGIWUR34959
CB4856 CT V 19101961 19118412 WBGene00012676 pro-3 Y39B6A.14
-0.261787225933598 AGIWUR4302;AGIWUR23872
CB4856 CT V 19118505 19119425 WBGene00012675 Y39B6A.13 Y39B6A.13
-0.21033319141896 AGIWUR1697;AGIWUR18271;AGIWUR25659;AGIWUR42276
CB4856 CT V 19119527 19124915 WBGene00012674 bed-1 Y39B6A.12
-0.0906801170587404 AGIWUR8314
CB4856 CT V 19127530 19130938 WBGene00012673 dyf-17 Y39B6A.11
-0.158701943692526 AGIWUR31302
CB4856 CT V 19131531 19133603 WBGene00012672 Y39B6A.10 Y39B6A.10
-0.140546113618113 AGIWUR28432
CB4856 CT V 19136509 19138986 WBGene00012671 Y39B6A.9 Y39B6A.9
0.00210707659755727 AGIWUR37848
CB4856 CT V 19140795 19145542 WBGene00012670 Y39B6A.8 Y39B6A.8
-0.0667300755483398 AGIWUR9446;AGIWUR12441;AGIWUR23017;AGIWUR25985;AGIWUR31414;AGIWUR38533
CB4856 CT V 19145778 19149147 WBGene00012669 Y39B6A.7 Y39B6A.7
0.18623114937029 AGIWUR5513;AGIWUR7476
CB4856 CT V 19154106 19157674 WBGene00006639 tsp-13 Y39B6A.6
-0.00339003946753813 AGIWUR1772
CB4856 CT V 19157659 19158877 WBGene00012668 Y39B6A.5 Y39B6A.5
0.00749494018918817 AGIWUR38840
CB4856 CT V 19174806 19187899 WBGene00001987 hot-2 Y39B6A.48
0.0925721478940567 AGIWUR11607;AGIWUR30465;AGIWUR44669
CB4856 CT V 19189246 19190035 WBGene00012666 Y39B6A.3 Y39B6A.3
-0.0104450744267428 AGIWUR4119;AGIWUR9133;AGIWUR19452

CB4856 CT   V   19190547   19199080   WBGene00012665 pph-5   Y39B6A.2
-0.159420944034239   AGIWUR17724;AGIWUR26103;AGIWUR30573;AGIWUR40282
CB4856 CT   V   19201013   19203607   WBGene00012664 Y39B6A.1   Y39B6A.1
-0.199319606311819   AGIWUR381;AGIWUR1405;AGIWUR30522
CB4856 CT   V   19207684   19211895   WBGene00003669 nhr-79 T26H2.9 0.0857230144244525
AGIWUR33400
CB4856 CT   V   19213685   19217138   WBGene00005653 srr-2   T26H2.8 0.15350288798496
AGIWUR39912
CB4856 CT   V   19217975   19222908   WBGene00012068 oac-49 T26H2.7 -0.0543920099634887
AGIWUR12320;AGIWUR34436
CB4856 CT   V   19252181   19253054   WBGene00219452 F21D9.11   F21D9.11
0.377138718579545   AGIWUR32988
CB4856 CT   V   19253820   19256758   WBGene00009018 F21D9.4 F21D9.4 -0.0843234619629349
AGIWUR14486;AGIWUR27967;AGIWUR40913
CB4856 CT   V   19257583   19257753   WBGene00009017 F21D9.3 F21D9.3 0.0998041955319593
AGIWUR44583
CB4856 CT   V   19258483   19262449   WBGene00009016 F21D9.2 F21D9.2 -0.286243282727959
AGIWUR31772
CB4856 CT   V   19264597   19269661   WBGene00009015 F21D9.1 F21D9.1 0.550793896935902
AGIWUR11826
CB4856 CT   V   19279218   19279988   WBGene00009021 F21D9.8 F21D9.8 0.176610104570992
AGIWUR27067
CB4856 CT   V   19282614   19284594   WBGene00010099 fbxb-117   F55C9.1
0.241687024387591   AGIWUR13275
CB4856 CT   V   19287009   19288173   WBGene00010100 F55C9.3 F55C9.3 0.446663953574369
AGIWUR494
CB4856 CT   V   19288755   19289889   WBGene00010101 fbxb-57 F55C9.4 0.298954915545739
AGIWUR24142
CB4856 CT   V   19290616   19291028   WBGene00010102 F55C9.5 F55C9.5 0.295499243144232
AGIWUR19911
CB4856 CT   V   19299562   19300461   WBGene00077663 F55C9.14   F55C9.14
0.25673603433748   AGIWUR6689
CB4856 CT   V   19301076   19302034   WBGene00010105 fbxb-62 F55C9.8 0.0384787493938141
AGIWUR8848
CB4856 CT   V   19302787   19303728   WBGene00044197 fbxb-63 F55C9.13
0.248931245266474   AGIWUR2799
CB4856 CT   V   19307242   19307583   WBGene00010107 F55C9.11   F55C9.11
0.111034248474127   AGIWUR11613;AGIWUR17151;AGIWUR17890;AGIWUR25273;AGIWUR41312;AGIWUR44273
CB4856 CT   V   19308707   19309687   WBGene00077441 C43D7.10   C43D7.10

0.106238836466188 AGIWUR30843;AGIWUR31224
CB4856 CT V 19310583 19311058 WBGene00008070 C43D7.8 C43D7.8 0.520145912755627
AGIWUR33714
CB4856 CT V 19313275 19316512 WBGene00008071 fbxb-64 C43D7.9 0.306299396399192
AGIWUR31078
CB4856 CT V 19317362 19318500 WBGene00005418 srh-208 C43D7.6 0.0747983424638381
AGIWUR23104
CB4856 CT V 19319033 19319460 WBGene00008069 C43D7.7 C43D7.7 0.0205360276602269
AGIWUR38919
CB4856 CT V 19320624 19321203 WBGene00008068 sdz-6 C43D7.5 -0.347657940562739
AGIWUR42821
CB4856 CT V 19321901 19322487 WBGene00008067 C43D7.4 C43D7.4 -0.25759566105069
AGIWUR30874;AGIWUR38230
CB4856 CT V 19324105 19325882 WBGene00014702 C43D7.3 C43D7.3 0.0844900872335126
AGIWUR12126;AGIWUR22143
CB4856 CT V 19329563 19330747 WBGene00008066 fbxb-65 C43D7.2 0.136942364801833
AGIWUR44975
CB4856 CT V 19332425 19349495 WBGene00007583 C14B4.2 C14B4.2 -0.0164805617912447
AGIWUR32835;AGIWUR36110
CB4856 CT V 19370602 19371331 WBGene00012808 Y43F8A.1 Y43F8A.1
0.474556445240346 AGIWUR26806
CB4856 CT V 19371553 19376247 WBGene00012809 Y43F8A.2 Y43F8A.2
-0.0451962782893531 AGIWUR28395
CB4856 CT V 19377444 19381031 WBGene00012810 nceh-1 Y43F8A.3
0.296624703079731 AGIWUR31819
CB4856 CT V 19381268 19384760 WBGene00005831 srw-84 Y43F8A.4
0.131108193901079 AGIWUR43294
CB4856 CT V 19384887 19390120 WBGene00012811 Y43F8A.5 Y43F8A.5
0.0523577081872233 AGIWUR7721;AGIWUR20141;AGIWUR20247;AGIWUR27153;AGIWUR28536;AGIWUR29250
CB4856 CT V 19395055 19396609 WBGene00007727 C25F9.8 C25F9.8 -0.259664088523056
AGIWUR44804
CB4856 CT V 19397828 19398982 WBGene00045413 C25F9.13 C25F9.13
0.0679917601549302 AGIWUR475
CB4856 CT V 19400142 19400715 WBGene00005833 srw-86 C25F9.7 -0.258738332941444
AGIWUR6983
CB4856 CT V 19402296 19402955 WBGene00045412 C25F9.12 C25F9.12
-0.225235466897605 AGIWUR1593;AGIWUR18466;AGIWUR19405;AGIWUR25246;AGIWUR28580
CB4856 CT V 19403530 19405326 WBGene00007726 C25F9.6 C25F9.6 -0.394728018692351
AGIWUR21894

CB4856	CT	V	19405685	19406394	WBGene00044291	C25F9.10	C25F9.10	-0.450258169266306	AGIWUR2366;AGIWUR17710;AGIWUR42481
CB4856	CT	V	19407739	19410857	WBGene00007725	C25F9.5	C25F9.5	-0.476708125991438	AGIWUR5091
CB4856	CT	V	19411274	19413914	WBGene00007724	C25F9.4	C25F9.4	-0.0489971917259382	AGIWUR26318
CB4856	CT	V	19416633	19418004	WBGene00007728	C25F9.9	C25F9.9	-0.176682415503495	AGIWUR14245;AGIWUR39624
CB4856	CT	V	19418212	19418911	WBGene00050967	C25F9.15	C25F9.15	-0.123829118190302	AGIWUR2951;AGIWUR35208;AGIWUR39664
CB4856	CT	V	19421471	19426774	WBGene00007723	C25F9.2	C25F9.2	0.0760921730494903	AGIWUR34473
CB4856	CT	V	19429440	19431687	WBGene00005832	srw-85	C25F9.1	-0.352132654026517	AGIWUR1639
CB4856	CT	V	19432441	19433072	WBGene00045411	C25F9.11	C25F9.11	0.962961882723931	AGIWUR27999;AGIWUR36828
CB4856	CT	V	19436484	19437217	WBGene00045461	C25F9.14	C25F9.14	-0.340137072508077	AGIWUR9904;AGIWUR27826;AGIWUR29040;AGIWUR42961
CB4856	CT	V	19437899	19445605	WBGene00010850	M04C3.1	M04C3.1	-0.298917135005221	AGIWUR15238;AGIWUR16598
CB4856	CT	V	19445873	19448901	WBGene00010851	M04C3.2	M04C3.2	-0.171140335086086	AGIWUR3985;AGIWUR21107
CB4856	CT	V	19449738	19450914	WBGene00206418	M04C3.5	M04C3.5	0.028611135524276	AGIWUR10907
CB4856	CT	V	19452183	19455031	WBGene00012824	Y43F8B.14	Y43F8B.14	-0.257150361757787	AGIWUR7744
CB4856	CT	V	19460818	19462102	WBGene00045415	Y43F8B.15	Y43F8B.15	-0.12504945863821	AGIWUR1838;AGIWUR3164;AGIWUR37500;AGIWUR41629
CB4856	CT	V	19462912	19463759	WBGene00195177	Y43F8B.25	Y43F8B.25	0.0417357037646759	AGIWUR45162
CB4856	CT	V	19468383	19469554	WBGene00012822	Y43F8B.12	Y43F8B.12	-0.648666869059308	AGIWUR15046
CB4856	CT	V	19470694	19472501	WBGene00012821	Y43F8B.11	Y43F8B.11	0.0186345386255615	AGIWUR11074
CB4856	CT	V	19474464	19476942	WBGene00012820	Y43F8B.10	Y43F8B.10	-0.110925114150623	AGIWUR22443
CB4856	CT	V	19479203	19480459	WBGene00012819	Y43F8B.9	Y43F8B.9	-0.334663963570359	AGIWUR2851
CB4856	CT	V	19483637	19483895	WBGene00014888	Y43F8B.8	Y43F8B.8		

-0.0100913905874904 AGIWUR11075;AGIWUR22024;AGIWUR28783;AGIWUR29367;AGIWUR33429
CB4856 CT V 19485319 19485873 WBGene00219302 Y43F8B.28 Y43F8B.28
0.210316425734661 AGIWUR27524
CB4856 CT V 19485916 19486746 WBGene00050911 Y43F8B.18 Y43F8B.18
-0.0569293310786634 AGIWUR1861;AGIWUR18935
CB4856 CT V 19490761 19491231 WBGene00012818 Y43F8B.7 Y43F8B.7
0.556454885465171 AGIWUR13017;AGIWUR18716
CB4856 CT V 19493885 19494657 WBGene00012817 Y43F8B.6 Y43F8B.6
0.0901845408787585 AGIWUR42352
CB4856 CT V 19494762 19495687 WBGene00012816 scl-21 Y43F8B.5
0.137188229454266 AGIWUR20613
CB4856 CT V 19503108 19519950 WBGene00012814 Y43F8B.3 Y43F8B.3
-0.0556768105897536 AGIWUR27061;AGIWUR30319;AGIWUR39677
CB4856 CT V 19519925 19533110 WBGene00012813 Y43F8B.2 Y43F8B.2
0.544570160542287
AGIWUR5140;AGIWUR8653;AGIWUR11230;AGIWUR13882;AGIWUR16244;AGIWUR24976;AGIWUR26509;AGIWUR26843;AGIWUR31243;A
GIWUR38299
CB4856 CT V 19534880 19546200 WBGene00012812 Y43F8B.1 Y43F8B.1
0.0376007201980233 AGIWUR4012;AGIWUR9462;AGIWUR20201;AGIWUR20421;AGIWUR20989;AGIWUR35973;AGIWUR40150
CB4856 CT V 19548239 19549784 WBGene00086559 Y43F8B.20 Y43F8B.20
-0.101844073866216 AGIWUR37706
CB4856 CT V 19551008 19555969 WBGene00007177 oac-1 B0399.2 -0.304304932847504
AGIWUR32001;AGIWUR33010
CB4856 CT V 19565386 19606115 WBGene00007176 kcnl-1 B0399.1 0.0425328149960107
AGIWUR25702;AGIWUR29918
CB4856 CT V 19607511 19608346 WBGene00003763 nlp-25 Y43F8C.1
0.53298131813699 AGIWUR26743
CB4856 CT V 19609494 19612099 WBGene00012840 grsp-1 Y43F8C.20
-0.347777979233377 AGIWUR5647;AGIWUR38806
CB4856 CT V 19613461 19614533 WBGene00003764 nlp-26 Y43F8C.2
-0.0980431857866186 AGIWUR26915
CB4856 CT V 19614558 19622551 WBGene00012825 Y43F8C.3 Y43F8C.3
0.722249762881573 AGIWUR8607;AGIWUR9609
CB4856 CT V 19625062 19630753 WBGene00012826 dyf-19 Y43F8C.4
0.399055319985118 AGIWUR20380
CB4856 CT V 19633062 19640908 WBGene00012828 Y43F8C.6 Y43F8C.6
0.0489485815717909 AGIWUR7964
CB4856 CT V 19641292 19644083 WBGene00012829 Y43F8C.7 Y43F8C.7
0.22647507194745 AGIWUR32615

CB4856	CT	V	19644239	19645758	WBGene00012830	mrps-28	Y43F8C.8	-0.228235662849421	AGIWUR2235;AGIWUR13361;AGIWUR20245;AGIWUR21105;AGIWUR28455;AGIWUR30763;AGIWUR31409
CB4856	CT	V	19646088	19647338	WBGene00012831	Y43F8C.9	Y43F8C.9	0.000224558050490486	AGIWUR11322
CB4856	CT	V	19650438	19655251	WBGene00012832	dmd-3	Y43F8C.10	0.00474158373676481	AGIWUR41418
CB4856	CT	V	19670259	19671411	WBGene00012833	Y43F8C.11	Y43F8C.11	0.00769295000437431	AGIWUR35037
CB4856	CT	V	19674843	19693043	WBGene00003413	mrp-7	Y43F8C.12	-0.338062181201629	AGIWUR7172;AGIWUR10445;AGIWUR24658;AGIWUR29604;AGIWUR43123
CB4856	CT	V	19693896	19697398	WBGene00012834	Y43F8C.13	Y43F8C.13	-0.394780946971586	AGIWUR15896
CB4856	CT	V	19697560	19705212	WBGene00012835	ani-3	Y43F8C.14	0.0653895333158019	AGIWUR23978
CB4856	CT	V	19705205	19706379	WBGene00012839	Y43F8C.18	Y43F8C.18	0.0673759529983653	AGIWUR6142;AGIWUR28019;AGIWUR32124;AGIWUR32673;AGIWUR34660;AGIWUR44782
CB4856	CT	V	19706890	19708949	WBGene00005714	srv-3	Y43F8C.19	-0.400171065909803	AGIWUR44295
CB4856	CT	V	19709775	19710614	WBGene00012836	Y43F8C.15	Y43F8C.15	0.112164886503987	AGIWUR32879
CB4856	CT	V	19714213	19715519	WBGene00012837	Y43F8C.16	Y43F8C.16	-0.0719048855638751	AGIWUR10556;AGIWUR41075
CB4856	CT	V	19727481	19727964	WBGene00013819	Y116F11A.6	Y116F11A.6	-0.119334528031292	AGIWUR17758
CB4856	CT	V	19729038	19729590	WBGene00013817	Y116F11A.3	Y116F11A.3	-0.0595843508370528	AGIWUR15941
CB4856	CT	V	19730588	19732165	WBGene00013816	Y116F11A.1	Y116F11A.1	0.0776648001578359	AGIWUR20538;AGIWUR42748
CB4856	CT	V	19733411	19735075	WBGene00014615	Y116F11A.4	Y116F11A.4	0.0795342797195073	AGIWUR1684;AGIWUR15569;AGIWUR33436;AGIWUR44464
CB4856	CT	V	19735077	19737607	WBGene00012252	W04E12.7	W04E12.7	-0.0507907419473508	AGIWUR28982;AGIWUR29879;AGIWUR35186
CB4856	CT	V	19738417	19739393	WBGene00012246	fbxa-131	W04E12.1	-0.273173800358208	AGIWUR27629
CB4856	CT	V	19739559	19741117	WBGene00012247	W04E12.2	W04E12.2	-0.509550166896247	AGIWUR15328
CB4856	CT	V	19741951	19742312	WBGene00012248	W04E12.3	W04E12.3	-0.807221307761607	AGIWUR25773
CB4856	CT	V	19746152	19748540	WBGene00012250	W04E12.5	W04E12.5		

-0.585537456283321	AGIWUR39057
CB4856	CT	V	19748987	19751212	WBGene00012251	clec-49	W04E12.6
-0.522796572722431	AGIWUR32
CB4856	CT	V	19752400	19755368	WBGene00012253	clec-50	W04E12.8
-0.154897706337781	AGIWUR44477
CB4856	CT	V	19756553	19758552	WBGene00012254	W04E12.9	W04E12.9
1.2654030308463	AGIWUR11345
CB4856	CT	V	19768261	19770929	WBGene00050946	M162.12	M162.12	-0.326135273088482
AGIWUR37664
CB4856	CT	V	19773401	19774975	WBGene00014811	M162.9	M162.9	-0.375382752655579
AGIWUR28727;AGIWUR29002
CB4856	CT	V	19777247	19778708	WBGene00044655	fbxa-194	M162.11
-0.0411271940761866	AGIWUR5922;AGIWUR35071
CB4856	CT	V	19780123	19782314	WBGene00010930	srt-46	M162.4	0.0508116387781037
AGIWUR38377
CB4856	CT	V	19784129	19785961	WBGene00010929	srt-45	M162.3	-0.338891832582814
AGIWUR43248
CB4856	CT	V	19788704	19789281	WBGene00010928	clec-258	M162.2
0.0120230499840872	AGIWUR1086
CB4856	CT	V	19790043	19791248	WBGene00010927	clec-259	M162.1
-0.500522704997662	AGIWUR15647
CB4856	CT	V	19797999	19801133	WBGene00013820	Y116F11B.2	Y116F11B.2
0.544711942581079	AGIWUR577
CB4856	CT	V	19815133	19816451	WBGene00000920	daf-28	Y116F11B.1
-0.270393810964249	AGIWUR14505
CB4856	CT	V	19822748	19830099	WBGene00003959	pcp-4	Y116F11B.3
-0.547316541591641	AGIWUR32657
CB4856	CT	V	19836442	19839342	WBGene00005785	srw-38	Y116F11B.5
0.203919998003562	AGIWUR34111
CB4856	CT	V	19839403	19841472	WBGene00013821	srz-35	Y116F11B.4
0.304452223157095	AGIWUR11931
CB4856	CT	V	19848314	19855250	WBGene00013823	Y116F11B.7	Y116F11B.7
-0.370383372955787	AGIWUR26854
CB4856	CT	V	19855174	19867983	WBGene00013825	Y116F11B.9	Y116F11B.9
-0.053920005014028	AGIWUR15712;AGIWUR38310;AGIWUR39456
CB4856	CT	V	19870506	19873931	WBGene00013826	Y116F11B.10	Y116F11B.10
-0.0705033410605438	AGIWUR5727
CB4856	CT	V	19881374	19895350	WBGene00001629	gly-4	Y116F11B.12
-0.0551619801563579	AGIWUR44184

CB4856	CT	V	19897977	19907434	WBGene00013361	fars-2	Y60A3A.13	-0.187215893187037	AGIWUR22328;AGIWUR27164;AGIWUR42038
CB4856	CT	V	19908765	19916023	WBGene00000499	chk-2	Y60A3A.12	-0.349110759979391	AGIWUR5614
CB4856	CT	V	19916557	19919996	WBGene00013365	Y60A3A.19	Y60A3A.19	-0.0183200860319772	AGIWUR1610;AGIWUR39964
CB4856	CT	V	19921697	19922600	WBGene00013364	Y60A3A.16	Y60A3A.16	-0.388796663945929	AGIWUR13571
CB4856	CT	V	19922931	19924049	WBGene00014934	Y60A3A.17	Y60A3A.17	-0.0363040394374279	AGIWUR28300
CB4856	CT	V	19924224	19925033	WBGene00004810	skr-4	Y60A3A.18	-0.113971626157779	AGIWUR21479;AGIWUR23516;AGIWUR37687
CB4856	CT	V	19927782	19934323	WBGene00013362	algn-7	Y60A3A.14	-0.695823754681923	AGIWUR37566
CB4856	CT	V	19934795	19946629	WBGene00000987	dhs-24	Y60A3A.10	-0.194761262742469	AGIWUR26087;AGIWUR37453
CB4856	CT	V	19946691	19949093	WBGene00013360	Y60A3A.9	Y60A3A.9	-0.185979066724007	AGIWUR4870
CB4856	CT	V	19949982	19955122	WBGene00013359	Y60A3A.8	Y60A3A.8	-0.273458373145225	AGIWUR21853
CB4856	CT	V	19955453	19958912	WBGene00013358	abhd-3.1	Y60A3A.7	-0.174675649900047	AGIWUR19672
CB4856	CT	V	19959044	19960916	WBGene00005388	srh-172	Y60A3A.6	0.100440115845434	AGIWUR7190
CB4856	CT	V	19965700	19972559	WBGene00005387	srh-171	Y60A3A.5	0.556988945427339	AGIWUR31131
CB4856	CT	V	19973284	19975063	WBGene00005389	srh-173	Y60A3A.4	0.0471557072215472	AGIWUR9556
CB4856	CT	V	19975610	19978320	WBGene00005398	srh-183	Y60A3A.3	-0.0584474855751331	AGIWUR22288
CB4856	CT	V	19978620	19981882	WBGene00023519	Y60A3A.24	Y60A3A.24	0.0319000404989582	AGIWUR10345;AGIWUR29702
CB4856	CT	V	19982994	19986313	WBGene00013357	clec-260	Y60A3A.2	-0.326515217316657	AGIWUR17195
CB4856	CT	V	19987984	19990504	WBGene00005579	sri-67	Y60A3A.22	0.0855424779490983	AGIWUR9481
CB4856	CT	V	19991207	19994189	WBGene00044211	Y60A3A.25	Y60A3A.25	-0.25738878721124	AGIWUR18341;AGIWUR26967
CB4856	CT	V	19995986	20004485	WBGene00006786	unc-51	Y60A3A.1		

-0.2554215534075 AGIWUR2393;AGIWUR4960;AGIWUR34898
CB4856 CT V 20007770 20009195 WBGene00013367 Y60A3A.23 Y60A3A.23
-0.0310328574162906
AGIWUR2176;AGIWUR14126;AGIWUR19113;AGIWUR24124;AGIWUR28719;AGIWUR29428;AGIWUR33373;AGIWUR41318
CB4856 CT V 20011959 20015077 WBGene00013366 Y60A3A.21 Y60A3A.21
-0.181718257875607 AGIWUR11192;AGIWUR19356;AGIWUR31108;AGIWUR35001;AGIWUR37667;AGIWUR44305
CB4856 CT V 20023046 20036667 WBGene00013746 lgc-55 Y113G7A.5
0.249000084631507 AGIWUR17669
CB4856 CT V 20037587 20039419 WBGene00013753 Y113G7A.16 Y113G7A.16
0.31316971397131 AGIWUR35813
CB4856 CT V 20040927 20047398 WBGene00013747 spe-19 Y113G7A.10
-0.0887794717900103 AGIWUR20899
CB4856 CT V 20053733 20064174 WBGene00006652 ttx-1 Y113G7A.6
0.145514214304172 AGIWUR13073;AGIWUR31199;AGIWUR39096;AGIWUR43427
CB4856 CT V 20075935 20087733 WBGene00001485 fre-1 Y113G7A.8
0.0453566672719994 AGIWUR11871
CB4856 CT V 20087848 20090743 WBGene00000940 dcs-1 Y113G7A.9
-0.0894599531740283 AGIWUR19604
CB4856 CT V 20091263 20105685 WBGene00004754 sec-23 Y113G7A.3
-0.31471032530894 AGIWUR20961;AGIWUR27455
CB4856 CT V 20105632 20120675 WBGene00003566 ncx-1 Y113G7A.4
0.223060767890436 AGIWUR12631
CB4856 CT V 20128416 20133142 WBGene00013748 ssu-1 Y113G7A.11
-0.00148922310909239 AGIWUR9872
CB4856 CT V 20135864 20138449 WBGene00013751 Y113G7A.14 Y113G7A.14
-0.417749319579806 AGIWUR29412
CB4856 CT V 20144247 20146805 WBGene00013749 Y113G7A.12 Y113G7A.12
0.113929246662078 AGIWUR19626
CB4856 CT V 20148195 20150357 WBGene00013750 Y113G7A.13 Y113G7A.13
0.310823735318033 AGIWUR30341
CB4856 CT V 20151489 20155442 WBGene00008946 F19B2.7 F19B2.7 -0.320467501641395
AGIWUR43667
CB4856 CT V 20158523 20161788 WBGene00008945 F19B2.6 F19B2.6 -0.0186731745018642
AGIWUR9773
CB4856 CT V 20162649 20164561 WBGene00008944 F19B2.5 F19B2.5 -0.555282795728921
AGIWUR26790
CB4856 CT V 20167309 20169283 WBGene00008943 srz-36 F19B2.4 -0.267989378611726
AGIWUR19481
CB4856 CT V 20172821 20173999 WBGene00008942 srz-34 F19B2.2 0.00214301667588135

AGIWUR29579
CB4856	CT	V	20174988	20182756	WBGene00008941	srz-33	F19B2.1	0.0310647447148145
AGIWUR3249;AGIWUR12827
CB4856	CT	V	20183843	20186679	WBGene00008947	srz-16	F19B2.8	-0.314404184663457
AGIWUR22368
CB4856	CT	V	20187710	20188882	WBGene00013754	fbxa-116	Y113G7B.1
0.0542592532204633	AGIWUR41446
CB4856	CT	V	20190535	20192152	WBGene00013755	fbxa-115	Y113G7B.3
0.0233326667181037	AGIWUR16439;AGIWUR19447;AGIWUR23667
CB4856	CT	V	20193463	20195246	WBGene00005427	srh-217	Y113G7B.2
-0.147199104675959	AGIWUR9761
CB4856	CT	V	20196971	20198242	WBGene00013756	ftr-1	Y113G7B.4
0.145279198550639	AGIWUR13945
CB4856	CT	V	20199262	20200640	WBGene00001482	fog-2	Y113G7B.5
-0.0983287780692237	AGIWUR19874;AGIWUR34434;AGIWUR39227
CB4856	CT	V	20201616	20202874	WBGene00013758	fbxa-114	Y113G7B.7
-0.494755959253084	AGIWUR968
CB4856	CT	V	20203422	20204503	WBGene00013757	fbxa-113	Y113G7B.6
-0.0702235896923042	AGIWUR24213
CB4856	CT	V	20204528	20206180	WBGene00013759	fbxb-59	Y113G7B.8
0.378783566870137	AGIWUR32951
CB4856	CT	V	20206318	20207907	WBGene00013760	srbc-34	Y113G7B.9
-0.20903412711931	AGIWUR7968;AGIWUR26183
CB4856	CT	V	20208685	20212451	WBGene00013762	Y113G7B.12	Y113G7B.12
-0.0017020755632462	AGIWUR21855
CB4856	CT	V	20213821	20215297	WBGene00013761	Y113G7B.11	Y113G7B.11
0.206617346741114	AGIWUR23594
CB4856	CT	V	20224856	20225458	WBGene00013770	Y113G7B.26	Y113G7B.26
-0.0372166065744732	AGIWUR25398
CB4856	CT	V	20226835	20228829	WBGene00013763	Y113G7B.14	Y113G7B.14
-0.865656964736255	AGIWUR17338
CB4856	CT	V	20234807	20237743	WBGene00013768	sld-5	Y113G7B.24
-0.152709683770041	AGIWUR3715
CB4856	CT	V	20239180	20249482	WBGene00004203	swsn-1	Y113G7B.23
0.133768435191126	AGIWUR18515
CB4856	CT	V	20250881	20267244	WBGene00007017	mdt-17	Y113G7B.18
-0.161517654029719
AGIWUR2457;AGIWUR2923;AGIWUR6091;AGIWUR7506;AGIWUR11809;AGIWUR15271;AGIWUR20509;AGIWUR33020;AGIWUR33876;AGI
WUR41067

CB4856	CT	V	20267377	20269711	WBGene00013766	prmt-1	Y113G7B.17	0.248841244319369	AGIWUR18691;AGIWUR32192;AGIWUR35752
CB4856	CT	V	20270107	20275732	WBGene00013765	cdkr-3	Y113G7B.16	-0.308399608535409	AGIWUR3128;AGIWUR11138;AGIWUR23975;AGIWUR24040;AGIWUR28760;AGIWUR35598
CB4856	CT	V	20278160	20279861	WBGene00004173	pqn-94	ZC15.8	0.76414591218164	AGIWUR7199
CB4856	CT	V	20281391	20289417	WBGene00013835	mlt-4	ZC15.7	0.0313372323965195	AGIWUR32698
CB4856	CT	V	20289939	20294488	WBGene00013834	clec-261	ZC15.6	-0.212399923562197	AGIWUR1972
CB4856	CT	V	20303588	20304908	WBGene00013832	ZC15.3	ZC15.3	0.00847300568259257	AGIWUR43150
CB4856	CT	V	20306103	20308919	WBGene00013830	ZC15.1	ZC15.1	0.101618807804622	AGIWUR7067
CB4856	CT	V	20310148	20311656	WBGene00045050	ZC15.10	ZC15.10	0.0342139828530819	AGIWUR3810;AGIWUR21680;AGIWUR40158
CB4856	CT	V	20311819	20315628	WBGene00013831	clec-262	ZC15.2	0.291848014807735	AGIWUR18438
CB4856	CT	V	20317742	20324024	WBGene00013771	Y113G7C.1	Y113G7C.1	0.0478423155249175	AGIWUR11288
CB4856	CT	V	20325728	20328056	WBGene00012612	Y38H6A.1	Y38H6A.1	-0.0658964648144957	AGIWUR1354
CB4856	CT	V	20329705	20335364	WBGene00077669	Y38H6A.5	Y38H6A.5	0.0861651089582938	AGIWUR9711;AGIWUR32556;AGIWUR33345;AGIWUR34192
CB4856	CT	V	20345203	20348127	WBGene00012614	Y38H6A.3	Y38H6A.3	-0.116933449642193	AGIWUR19521
CB4856	CT	V	20348247	20349925	WBGene00014874	Y38H6A.4	Y38H6A.4	-0.0293890148798557	AGIWUR26688
CB4856	CT	V	20356802	20365718	WBGene00001700	grd-11	K02E2.2	0.254971670593611	AGIWUR28288
CB4856	CT	V	20365708	20366939	WBGene00010509	K02E2.8	K02E2.8	-0.461661813947678	AGIWUR3684
CB4856	CT	V	20367480	20369253	WBGene00005392	srh-177	K02E2.3	0.366833524095313	AGIWUR35201
CB4856	CT	V	20375536	20376301	WBGene00002118	ins-35	K02E2.4	0.376126197484277	AGIWUR3688
CB4856	CT	V	20377128	20379410	WBGene00014794	K02E2.5	K02E2.5	-0.0425475297516664	AGIWUR1067;AGIWUR26609
CB4856	CT	V	20380561	20382036	WBGene00010507	K02E2.6	K02E2.6	0.0450467038640667	

AGIWUR8603
CB4856	CT	V	20384769	20391009	WBGene00010508	K02E2.7	K02E2.7	1.36187512537416
AGIWUR24074
CB4856	CT	V	20387715	20393367	WBGene00010004	F53H2.1	F53H2.1	0.836439395156665
AGIWUR21255
CB4856	CT	V	20394073	20394700	WBGene00010005	cnc-7	F53H2.2	-0.05591518516015
AGIWUR7651;AGIWUR10762;AGIWUR22903;AGIWUR23224;AGIWUR29673;AGIWUR34032
CB4856	CT	V	20396078	20422035	WBGene00010006	F53H2.3	F53H2.3	0.0496822241624963
AGIWUR4475;AGIWUR5951;AGIWUR21698;AGIWUR37532;AGIWUR42730;AGIWUR44574
CB4856	CT	V	20425301	20431337	WBGene00001406	fce-2	F48F5.5	0.055304753254271
AGIWUR26833
CB4856	CT	V	20440232	20446477	WBGene00009846	F48F5.2	F48F5.2	-0.0724252711945423
AGIWUR27354;AGIWUR31784;AGIWUR32244
CB4856	CT	V	20448288	20453202	WBGene00009848	F48F5.6	F48F5.6	-0.0873106965192534
AGIWUR15429;AGIWUR17816;AGIWUR19087;AGIWUR20963;AGIWUR27787;AGIWUR35783;AGIWUR39911
CB4856	CT	V	20454111	20456433	WBGene00005147	srd-70	F48F5.4	0.231443244550182
AGIWUR34323
CB4856	CT	V	20458654	20464486	WBGene00007121	B0250.4	B0250.4	0.0989132443809515
AGIWUR34256
CB4856	CT	V	20469703	20470485	WBGene00007125	B0250.8	B0250.8	-0.189696571103955
AGIWUR43956
CB4856	CT	V	20471566	20472496	WBGene00050926	B0250.11	B0250.11
0.130948765511852	AGIWUR5746
CB4856	CT	V	20473022	20476061	WBGene00007124	B0250.7	B0250.7	-0.383470728140216
AGIWUR22645
CB4856	CT	V	20476748	20479089	WBGene00007119	calf-1	B0250.2	0.299294645033353
AGIWUR38425
CB4856	CT	V	20480792	20485385	WBGene00007126	dhcr-7	B0250.9	0.0556936095030967
AGIWUR7054
CB4856	CT	V	20487645	20489202	WBGene00007127	srbc-78	B0250.10
-0.0106237922774852	AGIWUR41033
CB4856	CT	V	20490771	20492485	WBGene00007123	srbc-79	B0250.6	0.171102943061475
AGIWUR42298
CB4856	CT	V	20493924	20497348	WBGene00007122	B0250.5	B0250.5	-0.035957905119032
AGIWUR32723;AGIWUR36887
CB4856	CT	V	20497922	20499103	WBGene00012615	dct-16	Y38H6C.1
-0.183597353229814	AGIWUR14745
CB4856	CT	V	20499899	20500494	WBGene00012616	dct-14	Y38H6C.3
0.0137383767015242

AGIWUR9853;AGIWUR19303;AGIWUR24521;AGIWUR25903;AGIWUR28169;AGIWUR28994;AGIWUR29363;AGIWUR33453;AGIWUR38174
CB4856 CT V 20500812 20503228 WBGene00005812 srw-65 Y38H6C.2
0.153791636328514 AGIWUR2254
CB4856 CT V 20504875 20505097 WBGene00012617 Y38H6C.4 Y38H6C.4
0.195381673742012 AGIWUR9045
CB4856 CT V 20514637 20515706 WBGene00012621 Y38H6C.8 Y38H6C.8
0.228233681281126 AGIWUR10442
CB4856 CT V 20516298 20518036 WBGene00012623 fbxa-149 Y38H6C.10
-0.117265651058483 AGIWUR24018
CB4856 CT V 20520250 20522270 WBGene00012622 Y38H6C.9 Y38H6C.9
-0.0500661924460273 AGIWUR1386
CB4856 CT V 20522621 20524512 WBGene00012624 fbxa-150 Y38H6C.11
-0.0404962761212619 AGIWUR3580;AGIWUR15698;AGIWUR27527
CB4856 CT V 20524700 20525866 WBGene00005382 srh-166 Y38H6C.12
0.187041235566943 AGIWUR14164
CB4856 CT V 20529953 20534169 WBGene00012626 famh-161 Y38H6C.14
-0.20937219964699 AGIWUR2469
CB4856 CT V 20534774 20536308 WBGene00012627 Y38H6C.15 Y38H6C.15
-0.204179629746958 AGIWUR41427
CB4856 CT V 20536430 20538155 WBGene00012628 Y38H6C.16 Y38H6C.16
0.158473041573712 AGIWUR15764
CB4856 CT V 20540395 20544192 WBGene00012629 slc-36.3 Y38H6C.17
1.01187928643662 AGIWUR2656
CB4856 CT V 20546626 20547148 WBGene00012630 Y38H6C.18 Y38H6C.18
-0.0944687446068739 AGIWUR25153
CB4856 CT V 20547261 20551164 WBGene00012632 Y38H6C.20 Y38H6C.20
0.091179137017888 AGIWUR34360
CB4856 CT V 20552294 20553071 WBGene00012631 Y38H6C.19 Y38H6C.19
0.123018988656721 AGIWUR19219
CB4856 CT V 20554116 20555267 WBGene00012633 Y38H6C.21 Y38H6C.21
-0.504606246026549 AGIWUR29870
CB4856 CT V 20555629 20556360 WBGene00012634 Y38H6C.23 Y38H6C.23
-0.239566609834324 AGIWUR24290
CB4856 CT V 20557945 20558379 WBGene00000015 abf-4 Y38H6C.22
-0.0342269857474937 AGIWUR5038
CB4856 CT V 20559080 20561895 WBGene00009166 F26F2.1 F26F2.1 -0.162657692208165
AGIWUR9644;AGIWUR24078;AGIWUR43885
CB4856 CT V 20564383 20564666 WBGene00219309 F26F2.13 F26F2.13
-0.231551413885454 AGIWUR33359

CB4856 CT V 20565410 20566541 WBGene00009168 F26F2.3 F26F2.3 -0.968468604036341
AGIWUR19294
CB4856 CT V 20569295 20570191 WBGene00009169 F26F2.4 F26F2.4 -0.309798145751217
AGIWUR9279;AGIWUR30803;AGIWUR33977;AGIWUR38558;AGIWUR40546;AGIWUR44909
CB4856 CT V 20573717 20574000 WBGene00009170 F26F2.5 F26F2.5 -0.308003222678744
AGIWUR38019
CB4856 CT V 20574625 20577636 WBGene00009171 clec-263 F26F2.6
0.176416913104539 AGIWUR15828
CB4856 CT V 20579546 20580678 WBGene00023504 F26F2.8 F26F2.8 0.251760307345429
AGIWUR3190;AGIWUR14596;AGIWUR29229;AGIWUR35095
CB4856 CT V 20580996 20590321 WBGene00009172 F26F2.7 F26F2.7 -0.0295429448821913
AGIWUR34182
CB4856 CT V 20600095 20602474 WBGene00000736 col-163 F46B3.17
-0.192133507433346 AGIWUR17760
CB4856 CT V 20603366 20604378 WBGene00009758 ttr-11 F46B3.3
-0.00246668879039724 AGIWUR5985;AGIWUR15582;AGIWUR37486
CB4856 CT V 20607804 20608559 WBGene00009759 ttr-12 F46B3.4 0.0562764254470447
AGIWUR28886;AGIWUR29298
CB4856 CT V 20609794 20613929 WBGene00001691 grd-2 F46B3.5 -0.226850437594816
AGIWUR11921
CB4856 CT V 20614382 20615054 WBGene00050932 F46B3.19 F46B3.19
0.0233848017502485 AGIWUR5624;AGIWUR19280;AGIWUR21487;AGIWUR28143;AGIWUR35096
CB4856 CT V 20618120 20618656 WBGene00009760 F46B3.7 F46B3.7 0.260652533702268
AGIWUR21128
CB4856 CT V 20618910 20621966 WBGene00077434 F46B3.20 F46B3.20
0.236009712412769 AGIWUR4884
CB4856 CT V 20622650 20623160 WBGene00009761 F46B3.8 F46B3.8 0.253761083423177
AGIWUR11360
CB4856 CT V 20623630 20624887 WBGene00077436 F46B3.22 F46B3.22
0.00914718239362955 AGIWUR12526;AGIWUR21313;AGIWUR32702;AGIWUR39543;AGIWUR43533
CB4856 CT V 20625186 20626143 WBGene00009762 F46B3.9 F46B3.9 -0.303446911641604
AGIWUR40468
CB4856 CT V 20626711 20628206 WBGene00009763 srz-18 F46B3.11
-0.241196592874285 AGIWUR15575
CB4856 CT V 20628422 20629802 WBGene00009764 srz-17 F46B3.12
0.126578938169255 AGIWUR21470
CB4856 CT V 20630148 20631329 WBGene00009765 F46B3.14 F46B3.14
-0.0748480558943871 AGIWUR7603;AGIWUR12522;AGIWUR13428;AGIWUR24567;AGIWUR36158;AGIWUR43491
CB4856 CT V 20632985 20633689 WBGene00009766 F46B3.15 F46B3.15

-0.0162476316807796	AGIWUR17891;AGIWUR44745;AGIWUR45040
CB4856	CT	V	20634462	20635313	WBGene00009767	F46B3.16	F46B3.16
-0.0593786487739116	AGIWUR31887
CB4856	CT	V	20635943	20636576	WBGene00012849	ttr-13	Y44A6B.4
-0.080122727517514	AGIWUR4635;AGIWUR32172;AGIWUR34647;AGIWUR34876;AGIWUR35235
CB4856	CT	V	20636864	20638444	WBGene00012848	Y44A6B.3	Y44A6B.3
-0.0978239852936866	AGIWUR39781
CB4856	CT	V	20639594	20641049	WBGene00012846	srxa-14	Y44A6B.1
-0.124402977274632	AGIWUR11441;AGIWUR28556;AGIWUR32560
CB4856	CT	V	20642289	20644356	WBGene00012847	srxa-15	Y44A6B.2
-0.00675102012418982	AGIWUR6148;AGIWUR10441;AGIWUR28261
CB4856	CT	V	20654652	20659742	WBGene00012184	mnr-1	W01F3.1	-0.062088340978713
AGIWUR27576
CB4856	CT	V	20660325	20662031	WBGene00012185	W01F3.2	W01F3.2	0.0855542924376227
AGIWUR21598
CB4856	CT	V	20667209	20678513	WBGene00012186	mlt-11	W01F3.3	1.3447859215006
AGIWUR34807;AGIWUR41668
CB4856	CT	V	20681420	20683014	WBGene00009999	srsx-35	F53F8.2	0.0551247966056985
AGIWUR5741;AGIWUR37284
CB4856	CT	V	20685683	20688770	WBGene00009998	klf-2	F53F8.1	0.738597644684175
AGIWUR30841
CB4856	CT	V	20688734	20689532	WBGene00010000	F53F8.3	F53F8.3	-0.534818194880716
AGIWUR22910
CB4856	CT	V	20690582	20691685	WBGene00010001	F53F8.4	F53F8.4	0.163384948586248
AGIWUR27499
CB4856	CT	V	20700340	20704048	WBGene00010002	F53F8.5	F53F8.5	-0.262415851984814
AGIWUR19422
CB4856	CT	V	20708728	20709794	WBGene00010003	F53F8.6	F53F8.6	-0.0547237431761545
AGIWUR37872
CB4856	CT	V	20713920	20716872	WBGene00012850	Y44A6C.1	Y44A6C.1
0.688167963739108	AGIWUR7317
CB4856	CT	V	20717872	20729271	WBGene00012319	ipla-3	W07A8.2	0.106389426540916
AGIWUR7440
CB4856	CT	V	20729948	20744385	WBGene00001043	dnj-25	W07A8.3	-0.0368469683353752
AGIWUR24301
CB4856	CT	V	20752883	20760317	WBGene00004013	pha-4	F38A6.1	0.723925998779789
AGIWUR4940;AGIWUR7056;AGIWUR10928;AGIWUR11116;AGIWUR33259;AGIWUR39144
CB4856	CT	V	20764955	20770161	WBGene00001248	elp-1	F38A6.2	0.00849726557637709
AGIWUR28578

CB4856	CT	V	20773743	20775369	WBGene00009528	F38A6.4	F38A6.4	-0.303769861609062	AGIWUR3923
CB4856	CT	V	20780413	20785259	WBGene00001851	hif-1	F38A6.3	0.276494192606818	AGIWUR4461;AGIWUR8388;AGIWUR13579;AGIWUR16367;AGIWUR17143;AGIWUR40614
CB4856	CT	V	20789471	20790784	WBGene00012853	Y44A6D.2	Y44A6D.2	-0.0878761557355272	AGIWUR15098
CB4856	CT	V	20790768	20792413	WBGene00012854	Y44A6D.3	Y44A6D.3	0.203718192841425	AGIWUR6196;AGIWUR8125;AGIWUR11022;AGIWUR14203;AGIWUR30558
CB4856	CT	V	20798218	20801207	WBGene00004748	sdf-9	Y44A6D.4	0.0352518257322991	AGIWUR37586
CB4856	CT	V	20801184	20803006	WBGene00012855	Y44A6D.5	Y44A6D.5	0.150253976568376	AGIWUR43513
CB4856	CT	V	20816142	20816553	WBGene00011394	T03D8.7	T03D8.7	0.0770099727931264	AGIWUR8909
CB4856	CT	V	20816576	20820330	WBGene00011393	T03D8.6	T03D8.6	-0.0475912862450159	AGIWUR11094
CB4856	CT	V	20820366	20825028	WBGene00001547	gcy-22	T03D8.5	0.391942734247864	AGIWUR38984
CB4856	CT	V	20825559	20827740	WBGene00001723	grl-14	T03D8.4	-0.117917605200936	AGIWUR2172
CB4856	CT	V	20827715	20828939	WBGene00011392	sbt-1	T03D8.3	0.261801988233133	AGIWUR26432;AGIWUR35497
CB4856	CT	V	20829811	20837552	WBGene00003830	num-1	T03D8.1	-0.186213695098949	AGIWUR3244;AGIWUR15343;AGIWUR24653
CB4856	CT	V	20838080	20838768	WBGene00011391	mrps-12	T03D8.2	-0.132864861148398	AGIWUR36932
CB4856	CT	V	20838865	20841582	WBGene00009289	exos-7	F31D4.1	-0.148984512051758	AGIWUR6280
CB4856	CT	V	20841573	20845546	WBGene00009290	F31D4.2	F31D4.2	-0.0036385108857413	AGIWUR13395;AGIWUR14102;AGIWUR21065;AGIWUR29128;AGIWUR33661;AGIWUR41259
CB4856	CT	V	20846113	20847727	WBGene00001431	fkb-6	F31D4.3	-0.0935473502241635	AGIWUR4919;AGIWUR13185;AGIWUR20145;AGIWUR25639;AGIWUR26171;AGIWUR31644;AGIWUR32800;AGIWUR43487
CB4856	CT	V	20847702	20850666	WBGene00009291	clec-264	F31D4.4	0.515288459760658	AGIWUR35224
CB4856	CT	V	20850833	20851897	WBGene00009294	F31D4.9	F31D4.9	-0.220471332263349	AGIWUR30890
CB4856	CT	V	20854143	20858576	WBGene00009292	F31D4.5	F31D4.5	1.03154771902533	AGIWUR42985
CB4856	CT	V	20859344	20861153	WBGene00006622	try-4	F31D4.6	-0.413610684555708

									AGIWUR26925
CB4856	CT	V	20862969	20865743	WBGene00006687	twk-35	F31D4.7	-0.187981928989885	AGIWUR2822
CB4856	CT	V	20866749	20868429	WBGene00009293	F31D4.8	F31D4.8	-0.435674188294236	AGIWUR43069
CB4856	CT	V	20879854	20898906	WBGene00006812	unc-80	F25C8.3	-0.145488345425427	AGIWUR27704
CB4856	CT	V	20898840	20903441	WBGene00000139	amx-3	F25C8.2	-0.280375999983406	AGIWUR6474
CB4856	CT	V	20903977	20907433	WBGene00009106	acs-12	F25C8.4	0.167769298520717	AGIWUR25618
CB4856	CT	V	20907470	20909746	WBGene00009104	F25C8.1	F25C8.1	-0.482528330039155	AGIWUR6493
CB4856	CT	V	20910467	20914387	WBGene00044187	ttll-9	F25C8.5	0.00662989433706107	AGIWUR11335;AGIWUR21432
CB4856	CT	V	20915486	20922738	WBGene00012857	pbo-5	Y44A6E.1	-0.0173667069295309	AGIWUR18324;AGIWUR27277;AGIWUR29637
CB4856	CT	X	42728	46924	WBGene00022203	Y73B3A.1	Y73B3A.1	-0.0630494867344584	AGIWUR22888
CB4856	CT	X	78429	80400	WBGene00022218	Y73B3A.16	Y73B3A.16	-0.111906160590696	AGIWUR23894
CB4856	CT	X	135793	138075	WBGene00022209	Y73B3A.7	Y73B3A.7	-0.1550332338765	AGIWUR25027
CB4856	CT	X	140434	140683	WBGene00022210	Y73B3A.8	Y73B3A.8	0.018443089650237	AGIWUR23454
CB4856	CT	X	150363	157246	WBGene00022211	Y73B3A.9	Y73B3A.9	-0.0184662642253706	AGIWUR23115
CB4856	CT	X	159638	160920	WBGene00044700	T08D2.9	T08D2.9	0.204598713350551	AGIWUR16884
CB4856	CT	X	164036	171839	WBGene00011607	T08D2.2	T08D2.2	-0.0123763257604779	AGIWUR17378
CB4856	CT	X	177396	177856	WBGene00011609	T08D2.4	T08D2.4	-0.0624831734182516	AGIWUR14158;AGIWUR14916;AGIWUR19089;AGIWUR23194;AGIWUR35007
CB4856	CT	X	192834	197901	WBGene00011613	T08D2.8	T08D2.8	-0.0193783554324938	AGIWUR5792
CB4856	CT	X	199262	207723	WBGene00022227	Y73B3B.5	Y73B3B.5	-0.483728998702071	AGIWUR22104
CB4856	CT	X	211441	211676	WBGene00022225	Y73B3B.3	Y73B3B.3	-0.379937612996432	AGIWUR18398
CB4856	CT	X	221017	225084	WBGene00007077	AC8.5	AC8.5	-0.13878149921053	AGIWUR44399
CB4856	CT	X	269127	270029	WBGene00022432	math-43	Y108F1.4	0.136926226778007	AGIWUR34721

CB4856	CT	X	270408	274012	WBGene00022433	Y108F1.5	Y108F1.5	-0.305426519921667	AGIWUR39937
CB4856	CT	X	297234	299287	WBGene00020083	R57.2	R57.2	-0.0193604595690378	AGIWUR31539
CB4856	CT	X	301832	308298	WBGene00020082	gcp-2.1	R57.1	-0.370676607227665	AGIWUR15035;AGIWUR31471
CB4856	CT	X	308762	311323	WBGene00019734	M02E1.2	M02E1.2	0.115479562849794	AGIWUR10337;AGIWUR35098;AGIWUR37692
CB4856	CT	X	326113	334600	WBGene00019733	M02E1.1	M02E1.1	0.22692383721793	AGIWUR13020;AGIWUR36891;AGIWUR38182
CB4856	CT	X	334580	341232	WBGene00015431	C04E7.4	C04E7.4	0.129103478214265	AGIWUR4709
CB4856	CT	X	344127	346289	WBGene00015430	C04E7.3	C04E7.3	-0.0905998181047538	AGIWUR11024
CB4856	CT	X	353619	357939	WBGene00015429	sor-3	C04E7.2	-0.17558765165764	AGIWUR32632
CB4856	CT	X	358092	360198	WBGene00015428	C04E7.1	C04E7.1	-0.344632471684411	AGIWUR39667
CB4856	CT	X	360779	362938	WBGene00045270	C04E7.5	C04E7.5	-0.412294149550418	AGIWUR1304;AGIWUR6863;AGIWUR26142;AGIWUR34587
CB4856	CT	X	372235	376900	WBGene00019862	nrde-3	R04A9.2	0.209957213193794	AGIWUR34371
CB4856	CT	X	377133	380605	WBGene00019863	R04A9.3	R04A9.3	-0.132232100969082	AGIWUR1910;AGIWUR27720;AGIWUR39047
CB4856	CT	X	381338	382709	WBGene00002060	ife-2	R04A9.4	0.205783139763408	AGIWUR44493
CB4856	CT	X	384382	388797	WBGene00019864	ceh-93	R04A9.5	-0.240456912057988	AGIWUR4933
CB4856	CT	X	391360	394828	WBGene00044281	R04A9.7	R04A9.7	0.112952115504725	AGIWUR15403;AGIWUR22517;AGIWUR22851;AGIWUR29510;AGIWUR35082;AGIWUR39575
CB4856	CT	X	397567	400687	WBGene00019865	R04A9.6	R04A9.6	-0.115400557695486	AGIWUR184;AGIWUR3927;AGIWUR6837;AGIWUR17015;AGIWUR38506
CB4856	CT	X	404897	405357	WBGene00019861	R04A9.1	R04A9.1	0.253956904730141	AGIWUR29396
CB4856	CT	X	406459	416633	WBGene00022858	ZK1193.2	ZK1193.2	-0.341653444640531	AGIWUR30557
CB4856	CT	X	417482	420361	WBGene00022859	ZK1193.3	ZK1193.3	0.0983439510956279	AGIWUR31872
CB4856	CT	X	421707	423033	WBGene00000608	col-19	ZK1193.1	0.092189697414355	AGIWUR6664;AGIWUR10326
CB4856	CT	X	422890	425631	WBGene00022860	ZK1193.4	ZK1193.4	-0.366560574406387	AGIWUR10253
CB4856	CT	X	427043	433799	WBGene00022861	dve-1	ZK1193.5	0.276324700445213	AGIWUR26243;AGIWUR30037;AGIWUR42054;AGIWUR44587
CB4856	CT	X	446356	450212	WBGene00018683	F52D1.2	F52D1.2	1.00246876157467	AGIWUR11405
CB4856	CT	X	450446	457648	WBGene00004127	abu-12	F52D1.3	-0.0965259602908617	AGIWUR18676
CB4856	CT	X	460651	466912	WBGene00018682	aagr-4	F52D1.1	-0.192027053614972	AGIWUR38943
CB4856	CT	X	476561	478283	WBGene00044556	F38G1.3	F38G1.3	0.00271863409316531	

AGIWUR21457;AGIWUR27213;AGIWUR30357
CB4856	CT	X	481378	486504	WBGene00000484	che-2	F38G1.1	0.228409476523635	AGIWUR16263
CB4856	CT	X	489695	492273	WBGene00001185	egl-17	F38G1.2	-0.29691354690662	AGIWUR37293
CB4856	CT	X	493211	497510	WBGene00015137	B0310.1	B0310.1	0.0947076336449548	AGIWUR27738
CB4856	CT	X	501965	506943	WBGene00015138	B0310.2	B0310.2	-0.178592014380882	AGIWUR23228
CB4856	CT	X	510818	520880	WBGene00015139	B0310.3	B0310.3	0.531926364357503	AGIWUR20693
CB4856	CT	X	525929	530289	WBGene00015141	ugt-46	B0310.5	-0.273576191890498	AGIWUR40037
CB4856	CT	X	530551	531009	WBGene00015142	B0310.6	B0310.6	-0.189666793920653	AGIWUR16990
CB4856	CT	X	531841	535834	WBGene00017898	F28C10.3	F28C10.3	-0.125630751386474
AGIWUR13471
CB4856	CT	X	537303	538910	WBGene00017899	F28C10.4	F28C10.4	0.235873304854156
AGIWUR27250
CB4856	CT	X	543107	544256	WBGene00017896	F28C10.1	F28C10.1	0.0885498043153357
AGIWUR6343
CB4856	CT	X	555788	557191	WBGene00001962	hlh-19	F57C12.3	0.670080556514257
AGIWUR32848
CB4856	CT	X	558141	563657	WBGene00003554	nas-38	F57C12.1	-0.397685967561786
AGIWUR34589
CB4856	CT	X	563719	572701	WBGene00003408	mrp-2	F57C12.4	-0.182997737670425
AGIWUR6975;AGIWUR36018;AGIWUR45099
CB4856	CT	X	576318	587483	WBGene00003407	mrp-1	F57C12.5	0.0987998878536018
AGIWUR9284;AGIWUR13774;AGIWUR34067
CB4856	CT	X	593960	596289	WBGene00017423	F13C5.2	F13C5.2	0.452142843434269
AGIWUR10683;AGIWUR13371;AGIWUR22552;AGIWUR22732;AGIWUR31040;AGIWUR35162
CB4856	CT	X	596314	599553	WBGene00017424	F13C5.3	F13C5.3	-0.0411291311915027
AGIWUR302;AGIWUR6079;AGIWUR9605;AGIWUR11481;AGIWUR16677;AGIWUR17142;AGIWUR21336;AGIWUR23589;AGIWUR28821;AGI
WUR33103;AGIWUR36497;AGIWUR37477;AGIWUR41870;AGIWUR43585
CB4856	CT	X	602172	604922	WBGene00017422	F13C5.1	F13C5.1	0.300946123213761	AGIWUR20727
CB4856	CT	X	604872	608492	WBGene00017426	F13C5.5	F13C5.5	0.329476291588048	AGIWUR43112
CB4856	CT	X	611120	621290	WBGene00006825	unc-96	F13C5.6	0.317740806376786	AGIWUR38876
CB4856	CT	X	629188	632868	WBGene00019776	M6.4	M6.4	-0.0464231738464731	AGIWUR22702
CB4856	CT	X	633703	636993	WBGene00004011	pha-2	M6.3	0.283078190469326
AGIWUR2968;AGIWUR10208;AGIWUR16900;AGIWUR22412;AGIWUR23724;AGIWUR24946;AGIWUR28447;AGIWUR39161;AGIWUR40151
CB4856	CT	X	642156	654903	WBGene00002056	ifc-2	M6.1	-0.263922510609609
AGIWUR13526;AGIWUR33635;AGIWUR35773;AGIWUR42995
CB4856	CT	X	656188	658511	WBGene00020571	lpr-7	T19D7.3	0.0777547040105508
AGIWUR18294;AGIWUR27906
CB4856	CT	X	658477	661777	WBGene00020572	rund-1	T19D7.4	0.000182744670741641	AGIWUR36812
CB4856	CT	X	664714	666300	WBGene00020573	T19D7.5	T19D7.5	-0.213793401579188	AGIWUR792

CB4856	CT	X	669684	670800	WBGene00020574	T19D7.6	T19D7.6	0.274973398643569	AGIWUR37273
CB4856	CT	X	673027	676484	WBGene00020569	lgc-32	T19D7.1	-0.38234829482605	AGIWUR27396
CB4856	CT	X	676435	680419	WBGene00020575	T19D7.7	T19D7.7	0.313661007210425	AGIWUR41783
CB4856	CT	X	681129	682780	WBGene00021185	Y9C12A.1	Y9C12A.1	-0.0162131662747278	AGIWUR9671
CB4856	CT	X	692978	703036	WBGene00017184	ncam-1	F02G3.1	0.478090232108411	AGIWUR5275;AGIWUR5619;AGIWUR11793;AGIWUR40249
CB4856	CT	X	703029	714478	WBGene00002150	irk-2	M02A10.2	-0.273368016743875	AGIWUR28735
CB4856	CT	X	720330	725957	WBGene00004829	sli-1	M02A10.3	0.0815611694549059	AGIWUR16071
CB4856	CT	X	731160	735672	WBGene00015364	npr-19	C02H7.2	-0.061308033976287	AGIWUR36999
CB4856	CT	X	751211	759732	WBGene00000086	aex-3	C02H7.3	0.462282937840752	AGIWUR9247;AGIWUR24583;AGIWUR31811;AGIWUR33793
CB4856	CT	X	763769	766713	WBGene00001127	dyf-11	C02H7.1	0.184211766084516	AGIWUR33461
CB4856	CT	X	767023	768069	WBGene00020216	trap-2	T04G9.5	-0.207866749863684	AGIWUR1311;AGIWUR14535;AGIWUR14666;AGIWUR28463;AGIWUR32471;AGIWUR43584
CB4856	CT	X	768596	770776	WBGene00002071	ile-2	T04G9.3	-0.394306135648847	AGIWUR20795
CB4856	CT	X	770833	771882	WBGene00020218	T04G9.7	T04G9.7	0.226377345989735	AGIWUR9050;AGIWUR38408;AGIWUR38566;AGIWUR43698
CB4856	CT	X	772353	775371	WBGene00020215	T04G9.4	T04G9.4	-0.00167422843611931	AGIWUR18022
CB4856	CT	X	777721	780514	WBGene00003534	nas-15	T04G9.2	-0.04996669721106	AGIWUR6169;AGIWUR9460
CB4856	CT	X	780528	785624	WBGene00020217	T04G9.6	T04G9.6	-0.147871515458969	AGIWUR25013
CB4856	CT	X	788353	796142	WBGene00020214	attf-5	T04G9.1	-0.318712304421556	AGIWUR24528
CB4856	CT	X	802796	804264	WBGene00017779	gtr-1	F25E2.1	0.0596900484582239	AGIWUR19478
CB4856	CT	X	806993	810794	WBGene00017780	F25E2.2	F25E2.2	-0.553943446415893	AGIWUR29915
CB4856	CT	X	811173	813163	WBGene00017781	F25E2.3	F25E2.3	1.02214397374834	AGIWUR9834
CB4856	CT	X	813852	815929	WBGene00002058	ifd-2	F25E2.4	-0.0459279016540588	AGIWUR17114
CB4856	CT	X	817927	825475	WBGene00000899	daf-3	F25E2.5	-0.0349791699455997	AGIWUR2490;AGIWUR2974;AGIWUR8571;AGIWUR16462;AGIWUR19235;AGIWUR20554;AGIWUR23624;AGIWUR25067;AGIWUR29176;AGIWUR30818;AGIWUR40071
CB4856	CT	X	830258	833760	WBGene00018213	F39H12.2	F39H12.2	0.464736047134817	AGIWUR39216
CB4856	CT	X	834778	837758	WBGene00018212	F39H12.1	F39H12.1	-0.283922018904917	AGIWUR17434
CB4856	CT	X	838144	841493	WBGene00018214	F39H12.3	F39H12.3	0.137039984698482	AGIWUR30604
CB4856	CT	X	845731	851516	WBGene00018215	igcm-1	F39H12.4	1.01862196320711	

AGIWUR40820
CB4856	CT	X	864925	871089	WBGene00018987	lgl-1	F56F10.4	0.0200223226245466
AGIWUR20878
CB4856	CT	X	872517	875948	WBGene00018984	F56F10.1	F56F10.1	-1.85326162576535
AGIWUR1210;AGIWUR8208;AGIWUR14901;AGIWUR15405;AGIWUR19430;AGIWUR37374
CB4856	CT	X	877239	879881	WBGene00022502	ZC13.1	ZC13.1	-0.0189543409945294
AGIWUR4898;AGIWUR18529;AGIWUR36418
CB4856	CT	X	880629	883049	WBGene00022503	ZC13.2	ZC13.2	0.223622369464096	AGIWUR44486
CB4856	CT	X	882967	888394	WBGene00022504	mam-1	ZC13.3	0.35420339426569
AGIWUR6310;AGIWUR40476
CB4856	CT	X	892228	898117	WBGene00003104	mab-7	ZC13.4	-0.148985747678448	AGIWUR18071
CB4856	CT	X	899508	902599	WBGene00044557	ZC13.10	ZC13.10	0.0739901210129181
AGIWUR2564;AGIWUR42796
CB4856	CT	X	913290	914853	WBGene00018078	F35H12.6	F35H12.6	-0.408724016507669
AGIWUR19934
CB4856	CT	X	915874	918290	WBGene00004769	sel-12	F35H12.3	-0.00845552602076438
AGIWUR8596;AGIWUR15902
CB4856	CT	X	918353	920193	WBGene00018077	F35H12.5	F35H12.5	-0.281105979452134
AGIWUR39745
CB4856	CT	X	920200	924511	WBGene00018076	pifk-1	F35H12.4	0.0658274853639362
AGIWUR39475
CB4856	CT	X	924964	932255	WBGene00018075	tbc-11	F35H12.2	0.340985976327225
AGIWUR39704
CB4856	CT	X	933804	934906	WBGene00018079	F35H12.7	F35H12.7	-0.298676818208678
AGIWUR12471
CB4856	CT	X	935460	937704	WBGene00018074	F35H12.1	F35H12.1	-0.380353165940778
AGIWUR17036
CB4856	CT	X	938516	942608	WBGene00018779	F53H8.3	F53H8.3	-0.0258203188025306	AGIWUR35128
CB4856	CT	X	944826	948883	WBGene00004893	sms-2	F53H8.4	0.00033294349936952
AGIWUR10675;AGIWUR16059;AGIWUR19890;AGIWUR31185;AGIWUR35157;AGIWUR36174;AGIWUR43169
CB4856	CT	X	950782	954233	WBGene00000195	arr-1	F53H8.2	0.295352427966373	AGIWUR32033
CB4856	CT	X	954839	957762	WBGene00000164	apm-3	F53H8.1	0.132708009571331
AGIWUR12166;AGIWUR44192;AGIWUR45022
CB4856	CT	X	957881	960568	WBGene00016642	chil-10	C44C1.2	0.0207279581513262	AGIWUR31190
CB4856	CT	X	960569	963202	WBGene00003563	ncs-1	C44C1.3	-0.15293724946201	AGIWUR41639
CB4856	CT	X	969074	970270	WBGene00016645	C44C1.6	C44C1.6	1.1225763528576	AGIWUR410
CB4856	CT	X	970583	974816	WBGene00016643	vps-45	C44C1.4	-0.202923806683621
AGIWUR8956;AGIWUR18258;AGIWUR38973
CB4856	CT	X	975525	978445	WBGene00016644	abhd-3.2	C44C1.5	-0.352577276382393

AGIWUR12864
CB4856 CT X 980776 983030 WBGene00016641 C44C1.1 C44C1.1 -0.131434735117212 AGIWUR16266
CB4856 CT X 984581 991456 WBGene00000958 dgk-1 C09E10.2 0.145183183765528
AGIWUR5498
CB4856 CT X 1004337 1009412 WBGene00018861 cutl-21 F55A4.10 0.594693843790038
AGIWUR34441
CB4856 CT X 1011501 1012347 WBGene00023069 rncs-1 F55A4.9 1.60874017126866 AGIWUR25420
CB4856 CT X 1012731 1017567 WBGene00018854 nlf-1 F55A4.2 -0.0479661089466843
AGIWUR3564;AGIWUR4708;AGIWUR21428;AGIWUR23400;AGIWUR32736;AGIWUR41565
CB4856 CT X 1023477 1023780 WBGene00018855 F55A4.3 F55A4.3 -0.143888431691273 AGIWUR33841
CB4856 CT X 1024646 1025838 WBGene00018856 F55A4.4 F55A4.4 0.554427766403347 AGIWUR14930
CB4856 CT X 1027673 1035286 WBGene00018857 stau-1 F55A4.5 0.152987481196379
AGIWUR10835;AGIWUR23559
CB4856 CT X 1035548 1037046 WBGene00018853 sec-22 F55A4.1 -0.382584531664966 AGIWUR3979
CB4856 CT X 1037023 1038447 WBGene00018859 F55A4.7 F55A4.7 0.171609769490267
AGIWUR4952;AGIWUR5421;AGIWUR10067;AGIWUR14857;AGIWUR20097;AGIWUR24312;AGIWUR37548
CB4856 CT X 1038514 1046206 WBGene00018860 vpat-1 F55A4.8 0.0676274869894513
AGIWUR2518;AGIWUR5258;AGIWUR18590;AGIWUR29451
CB4856 CT X 1054081 1056843 WBGene00003565 ncs-3 K03E6.3 -0.00282235830108313 AGIWUR18624
CB4856 CT X 1057487 1062447 WBGene00006741 unc-1 K03E6.5 0.0731817842943059
AGIWUR2302;AGIWUR14006;AGIWUR20781
CB4856 CT X 1066789 1069033 WBGene00019364 mksr-1 K03E6.4 0.580134297548295 AGIWUR31174
CB4856 CT X 1073908 1079451 WBGene00002988 lim-6 K03E6.1 0.151162732265491
AGIWUR8993;AGIWUR10111;AGIWUR20616;AGIWUR21171;AGIWUR36958;AGIWUR43242
CB4856 CT X 1079446 1080653 WBGene00003991 pfn-3 K03E6.6 -0.397079044451115
AGIWUR14050;AGIWUR16785;AGIWUR17554;AGIWUR22606;AGIWUR34446;AGIWUR35978
CB4856 CT X 1084214 1088148 WBGene00019365 rei-2 K03E6.7 0.478907812747069 AGIWUR9250
CB4856 CT X 1095547 1097013 WBGene00018634 F49E7.2 F49E7.2 0.306521608708 AGIWUR9891
CB4856 CT X 1097152 1103320 WBGene00004377 rme-6 F49E7.1 0.00544914083702297
AGIWUR1339;AGIWUR17283;AGIWUR18352;AGIWUR27630;AGIWUR32031;AGIWUR35210;AGIWUR37190;AGIWUR39437
CB4856 CT X 1104212 1106247 WBGene00002062 ife-4 C05D9.5 -0.0635280902870061 AGIWUR2875
CB4856 CT X 1107969 1110110 WBGene00015473 C05D9.4 C05D9.4 -0.143184094028553 AGIWUR3752
CB4856 CT X 1111903 1116794 WBGene00015472 C05D9.3 C05D9.3 0.0418084481443657 AGIWUR42743
CB4856 CT X 1117192 1119405 WBGene00015474 C05D9.7 C05D9.7 0.0446214186987657 AGIWUR25615
CB4856 CT X 1122422 1124308 WBGene00015476 C05D9.9 C05D9.9 0.878688793902527
AGIWUR28491;AGIWUR45071
CB4856 CT X 1125170 1128288 WBGene00015471 lmp-2 C05D9.2 -0.0082583769082305 AGIWUR42105
CB4856 CT X 1128986 1132925 WBGene00004927 snx-1 C05D9.1 -0.0182510719523883 AGIWUR40310
CB4856 CT X 1132993 1148585 WBGene00020128 R193.2 R193.2 -0.295705574256937

									AGIWUR619;AGIWUR14015;AGIWUR19955;AGIWUR21988;AGIWUR24806;AGIWUR31506;AGIWUR33612;AGIWUR39536;AGIWUR41598
CB4856	CT	X	1151547	1153545	WBGene00020129	R193.3	R193.3	-0.436797485888723	AGIWUR33696
CB4856	CT	X	1154948	1155613	WBGene00020127	R193.1	R193.1	-0.039179606631853	AGIWUR37642
CB4856	CT	X	1163153	1177172	WBGene00020490	tmc-1	T13G4.3	0.179276884128084	AGIWUR5257
CB4856	CT	X	1180284	1187932	WBGene00020489	hot-8	T13G4.2	0.0989980947836457	AGIWUR17480
CB4856	CT	X	1188453	1190752	WBGene00020491	T13G4.4	T13G4.4	-0.304539872903186	AGIWUR41536
CB4856	CT	X	1193148	1193858	WBGene00020488	T13G4.1	T13G4.1	0.124676370315193	AGIWUR44589
CB4856	CT	X	1195108	1198481	WBGene00020492	T13G4.5	T13G4.5	0.24983049571024	AGIWUR28194
CB4856	CT	X	1202606	1203763	WBGene00020494	T13G4.7	T13G4.7	0.153213505132827	AGIWUR29871
CB4856	CT	X	1209411	1210683	WBGene00016724	C46H3.1	C46H3.1	-0.00723199293515364	AGIWUR22802
CB4856	CT	X	1211760	1230758	WBGene00016725	C46H3.2	C46H3.2	-0.123682342688577	AGIWUR35176;AGIWUR42826
CB4856	CT	X	1245984	1247892	WBGene00006635	tsp-9	C25G6.2	-0.067428094753712	AGIWUR37946
CB4856	CT	X	1247833	1249838	WBGene00016109	C25G6.4	C25G6.4	0.0259076150229154	AGIWUR21889;AGIWUR30220;AGIWUR35983;AGIWUR37039
CB4856	CT	X	1270898	1272561	WBGene00016107	C25G6.1	C25G6.1	0.0433996115921751	AGIWUR15410
CB4856	CT	X	1272788	1275416	WBGene00016874	C52B11.5	C52B11.5	0.137207222074796	AGIWUR34464
CB4856	CT	X	1295278	1298823	WBGene00016872	dop-4	C52B11.3	-0.624980521237644	AGIWUR7407
CB4856	CT	X	1300149	1309504	WBGene00016871	inso-1	C52B11.2	0.25014350894207	AGIWUR5254;AGIWUR19809;AGIWUR21664;AGIWUR23766;AGIWUR26079;AGIWUR28297;AGIWUR40402
CB4856	CT	X	1311605	1316634	WBGene00019272	H42K12.3	H42K12.3	0.700710282260559	AGIWUR33012
CB4856	CT	X	1321353	1327985	WBGene00003965	pdk-1	H42K12.1	0.0410781872529888	AGIWUR29723
CB4856	CT	X	1328143	1329947	WBGene00017666	F21E9.2	F21E9.2	0.171310400228236	AGIWUR18182
CB4856	CT	X	1335363	1336915	WBGene00017667	ttr-37	F21E9.3	0.255939534779191	AGIWUR28934
CB4856	CT	X	1343837	1344410	WBGene00017668	ins-39	F21E9.4	-0.0250203617649971	AGIWUR19013
CB4856	CT	X	1355556	1356482	WBGene00017670	F21E9.6	F21E9.6	-0.145171706093239	AGIWUR33293
CB4856	CT	X	1357771	1358532	WBGene00018940	F56C3.3	F56C3.3	0.155498036538336	AGIWUR20347
CB4856	CT	X	1359720	1362036	WBGene00018941	F56C3.4	F56C3.4	0.0740122891863528	AGIWUR22669
CB4856	CT	X	1362816	1363997	WBGene00018942	F56C3.5	F56C3.5	0.15375730619339	AGIWUR12830
CB4856	CT	X	1365474	1367516	WBGene00018943	dgn-2	F56C3.6	0.0265954343578798	AGIWUR15039;AGIWUR33689
CB4856	CT	X	1370543	1370786	WBGene00018944	F56C3.7	F56C3.7	-0.0908691876438006	AGIWUR3068
CB4856	CT	X	1372012	1374630	WBGene00018939	fbxa-190	F56C3.2	-0.681593640210519	AGIWUR11442
CB4856	CT	X	1381375	1381665	WBGene00044558	F56C3.10	F56C3.10	-0.0264523083420567	

AGIWUR2372;AGIWUR19801;AGIWUR30724;AGIWUR32967;AGIWUR42194
CB4856 CT X 1384318 1384638 WBGene00018945 F56C3.8 F56C3.8 -0.167236352503801 AGIWUR18707
CB4856 CT X 1386248 1388160 WBGene00018938 F56C3.1 F56C3.1 -0.123482836384705 AGIWUR37168
CB4856 CT X 1391800 1393991 WBGene00018946 F56C3.9 F56C3.9 0.0808374664032669 AGIWUR26315
CB4856 CT X 1396353 1396551 WBGene00019267 H39E20.1 H39E20.1 -0.0153241758194511
AGIWUR10559
CB4856 CT X 1398824 1400751 WBGene00022731 ZK402.5 ZK402.5 -0.0887919438882836 AGIWUR27189
CB4856 CT X 1404271 1406101 WBGene00022728 ZK402.1 ZK402.1 0.262234385420536 AGIWUR22523
CB4856 CT X 1408836 1410652 WBGene00000025 abu-2 F19G12.7 -0.139520096847944
AGIWUR19145
CB4856 CT X 1413144 1416545 WBGene00005244 srh-19 F19G12.5 -0.000258686536012576
AGIWUR14945
CB4856 CT X 1417304 1417477 WBGene00017612 F19G12.4 F19G12.4 0.0775195109647653
AGIWUR1694;AGIWUR5185;AGIWUR16213
CB4856 CT X 1425727 1426164 WBGene00017611 F19G12.3 F19G12.3 0.207468750001641
AGIWUR32948
CB4856 CT X 1427774 1430871 WBGene00017610 F19G12.2 F19G12.2 0.44054871474159
AGIWUR40163
CB4856 CT X 1439261 1440261 WBGene00021046 sedl-1 W05H7.3 -0.214076249810581 AGIWUR2320
CB4856 CT X 1440366 1446643 WBGene00021047 zfp-3 W05H7.4 0.0669063586329415
AGIWUR10664;AGIWUR21164;AGIWUR22713;AGIWUR25733;AGIWUR33192;AGIWUR33399;AGIWUR37599;AGIWUR40048;AGIWUR40099
;AGIWUR40219;AGIWUR42218;AGIWUR42733;AGIWUR43726
CB4856 CT X 1450768 1452306 WBGene00021044 W05H7.1 W05H7.1 -0.584439833675255 AGIWUR28997
CB4856 CT X 1452695 1454000 WBGene00016997 cebp-1 D1005.3 -0.228136671427296 AGIWUR7092
CB4856 CT X 1459047 1462793 WBGene00016996 D1005.2 D1005.2 0.467132669606338 AGIWUR38343
CB4856 CT X 1463110 1464799 WBGene00017000 D1005.6 D1005.6 0.188182699618306 AGIWUR21560
CB4856 CT X 1470998 1472919 WBGene00016999 D1005.5 D1005.5 -0.130032739222166 AGIWUR25343
CB4856 CT X 1479542 1484398 WBGene00016995 acly-1 D1005.1 -0.330913244836777
AGIWUR2651;AGIWUR19932;AGIWUR34267;AGIWUR38476
CB4856 CT X 1484853 1485818 WBGene00016998 D1005.4 D1005.4 -0.188595905981773
AGIWUR6861;AGIWUR20502;AGIWUR36467;AGIWUR37562
CB4856 CT X 1486473 1489326 WBGene00000988 dhs-25 F09E10.3 -0.273344563442199
AGIWUR6958
CB4856 CT X 1489672 1494356 WBGene00017295 F09E10.5 F09E10.5 0.0625558425014301
AGIWUR22470
CB4856 CT X 1495074 1497681 WBGene00017296 F09E10.6 F09E10.6 0.990141974474393
AGIWUR15544
CB4856 CT X 1500180 1501852 WBGene00017297 F09E10.7 F09E10.7 -0.323010815812403
AGIWUR3344;AGIWUR5999;AGIWUR7563;AGIWUR14284;AGIWUR21040;AGIWUR21617;AGIWUR24684;AGIWUR26365;AGIWUR31714;AG

IWUR33549;AGIWUR36843;AGIWUR43253
CB4856 CT X 1502860 1503278 WBGene00017294 F09E10.1 F09E10.1 -0.277296221547367 AGIWUR40122
CB4856 CT X 1504131 1504841 WBGene00006650 tts-1 F09E10.11 2.38066030326954 AGIWUR9901;AGIWUR15248;AGIWUR21990;AGIWUR22247;AGIWUR25378;AGIWUR28345
CB4856 CT X 1505190 1505626 WBGene00022982 F09E10.10 F09E10.10 1.02158993581222 AGIWUR1241;AGIWUR1434;AGIWUR10913;AGIWUR26832;AGIWUR36065
CB4856 CT X 1510480 1521487 WBGene00017298 toca-1 F09E10.8 0.110364023125817 AGIWUR15186
CB4856 CT X 1536366 1540287 WBGene00019436 K06A9.2 K06A9.2 1.26526133116468 AGIWUR8972
CB4856 CT X 1547505 1560124 WBGene00019435 K06A9.1 K06A9.1 0.363445959935249 AGIWUR976;AGIWUR18127
CB4856 CT X 1568678 1572854 WBGene00019146 H02F09.3 H02F09.3 1.06039054723943 AGIWUR14894
CB4856 CT X 1573220 1574010 WBGene00019145 H02F09.2 H02F09.2 0.22304249201774 AGIWUR30326
CB4856 CT X 1576637 1591296 WBGene00006955 wrt-9 B0344.2 -0.0401623591545714 AGIWUR1818;AGIWUR5585
CB4856 CT X 1604906 1619115 WBGene00019188 H11E01.3 H11E01.3 -0.0325944926170418 AGIWUR2438;AGIWUR14187;AGIWUR17474;AGIWUR31358;AGIWUR41516
CB4856 CT X 1621481 1625247 WBGene00019187 H11E01.2 H11E01.2 0.324994712993135 AGIWUR17159
CB4856 CT X 1628137 1629454 WBGene00019186 fbxb-58 H11E01.1 -0.973547601743773 AGIWUR41566
CB4856 CT X 1630615 1635727 WBGene00022723 ZK380.2 ZK380.2 -0.0344184378417554 AGIWUR11129
CB4856 CT X 1651419 1653897 WBGene00022724 ZK380.4 ZK380.4 0.118033102505665 AGIWUR12753
CB4856 CT X 1655522 1656936 WBGene00006551 tbx-32 ZK380.1 0.103633193601357 AGIWUR10520
CB4856 CT X 1667019 1668848 WBGene00019991 R09H3.3 R09H3.3 -0.0412755062801233 AGIWUR16590
CB4856 CT X 1671148 1674165 WBGene00006550 tbx-31 C36C9.2 -0.135301347952566 AGIWUR28414
CB4856 CT X 1676205 1678121 WBGene00016486 fbxa-170 C36C9.3 -0.311203438036751 AGIWUR11766;AGIWUR14810;AGIWUR17800
CB4856 CT X 1681752 1681886 WBGene00219849 C36C9.10 C36C9.10 -0.0855058775289521 AGIWUR20054;AGIWUR26371;AGIWUR40994
CB4856 CT X 1682954 1686419 WBGene00016485 meg-4 C36C9.1 -0.229366517081883 AGIWUR5290;AGIWUR14478
CB4856 CT X 1699788 1703938 WBGene00016488 C36C9.5 C36C9.5 -0.0865753850550961 AGIWUR25128
CB4856 CT X 1706370 1706638 WBGene00017224 F07G6.5 F07G6.5 0.150905967517625 AGIWUR37961
CB4856 CT X 1710293 1711212 WBGene00017225 fbxa-52 F07G6.6 -0.00815852092287628 AGIWUR45205
CB4856 CT X 1714351 1715487 WBGene00044583 F07G6.9 F07G6.9 -0.126897314659622

AGIWUR6243;AGIWUR29401;AGIWUR39325;AGIWUR44809
CB4856 CT X 1716442 1718568 WBGene00017226 fbxa-53 F07G6.7 -0.0874595664545721
AGIWUR7155;AGIWUR25529;AGIWUR32530
CB4856 CT X 1720093 1720428 WBGene00017227 F07G6.8 F07G6.8 -0.554378811061215
AGIWUR21530;AGIWUR23105;AGIWUR23920;AGIWUR30816;AGIWUR36057;AGIWUR40612
CB4856 CT X 1727356 1729154 WBGene00017222 F07G6.2 F07G6.2 0.226934326529415 AGIWUR44319
CB4856 CT X 1729858 1731819 WBGene00017221 dgn-3 F07G6.1 -0.052080944882217 AGIWUR5604
CB4856 CT X 1737906 1739229 WBGene00045271 F07G6.10 F07G6.10 -0.392865990083301
AGIWUR31342
CB4856 CT X 1745096 1746217 WBGene00022292 Y75D11A.3 Y75D11A.3 -0.245011667120709
AGIWUR1373;AGIWUR2826;AGIWUR4858;AGIWUR13158;AGIWUR41378
CB4856 CT X 1777355 1778146 WBGene00001237 elc-2 W03H1.2 -0.0428894801373776 AGIWUR8069
CB4856 CT X 1786907 1788405 WBGene00000185 arg-1 F31A9.3 0.023038040267723
AGIWUR12589;AGIWUR18681;AGIWUR19310;AGIWUR34927;AGIWUR44042
CB4856 CT X 1794816 1795673 WBGene00017941 F31A9.2 F31A9.2 -0.111005894241842 AGIWUR2252
CB4856 CT X 1806833 1807767 WBGene00015771 got-1.3 C14E2.2 0.143395940233068 AGIWUR33854
CB4856 CT X 1810686 1811536 WBGene00015772 C14E2.3 C14E2.3 0.140119753736418 AGIWUR15532
CB4856 CT X 1816361 1816762 WBGene00044562 C14E2.7 C14E2.7 0.197856058166177
AGIWUR1121;AGIWUR9109;AGIWUR24714;AGIWUR42117;AGIWUR44298
CB4856 CT X 1823616 1825340 WBGene00015774 C14E2.5 C14E2.5 -0.0769982641359475 AGIWUR7100
CB4856 CT X 1826466 1827354 WBGene00015770 C14E2.1 C14E2.1 -0.134840618223209 AGIWUR10204
CB4856 CT X 1827738 1828487 WBGene00015775 C14E2.6 C14E2.6 0.000956805833038032 AGIWUR656
CB4856 CT X 1834445 1839024 WBGene00020829 T26C11.3 T26C11.3 -0.347191611765055
AGIWUR27549
CB4856 CT X 1840554 1845565 WBGene00020830 T26C11.4 T26C11.4 -0.207015848855065
AGIWUR24882
CB4856 CT X 1846608 1848675 WBGene00000462 ceh-41 T26C11.5 -0.0899762307512084
AGIWUR30638
CB4856 CT X 1848795 1852736 WBGene00000444 ceh-21 T26C11.6 0.0103899737499322
AGIWUR20034;AGIWUR42019
CB4856 CT X 1853874 1856189 WBGene00000460 ceh-39 T26C11.7 -0.348317734628885
AGIWUR17728
CB4856 CT X 1860722 1863543 WBGene00044563 T26C11.8 T26C11.8
-0.00600875509009535 AGIWUR13940;AGIWUR41424
CB4856 CT X 1865246 1866638 WBGene00006560 tbx-41 T26C11.1 -0.0821107386001048
AGIWUR10415
CB4856 CT X 1892187 1893272 WBGene00015118 B0294.3 B0294.3 0.0926290595119266
AGIWUR903;AGIWUR5963;AGIWUR22701;AGIWUR25360;AGIWUR36315
CB4856 CT X 1896662 1897343 WBGene00015117 B0294.1 B0294.1 -0.0301205785262795 AGIWUR37631

CB4856	CT	X	1910943	1913357	WBGene00022508	ZC53.1	ZC53.1	-0.382247251597274	AGIWUR16565
CB4856	CT	X	1915258	1917162	WBGene00004352	rgs-9	ZC53.7	-0.382964142319146	AGIWUR2771
CB4856	CT	X	1917437	1919755	WBGene00022512	dot-1.5	ZC53.6	-0.149638371776062	AGIWUR38437
CB4856	CT	X	1923723	1924238	WBGene00022509	ZC53.2	ZC53.2	0.0731308264835411	AGIWUR14607
CB4856	CT	X	1925750	1937689	WBGene00022511	ZC53.4	ZC53.4	0.0657139008741376	AGIWUR2275
CB4856	CT	X	1937947	1942199	WBGene00021987	fbxa-40	Y59E1A.1	-0.208557513399313	AGIWUR6971
CB4856	CT	X	1955550	1956824	WBGene00018691	fbxa-17	F52D2.10	0.00898981071729921	AGIWUR44400
CB4856	CT	X	1962573	1966188	WBGene00018689	F52D2.7	F52D2.7	-0.306876446959032	AGIWUR5190
CB4856	CT	X	1969875	1972657	WBGene00006139	str-79	F52D2.9	-0.132312707659151	AGIWUR34890
CB4856	CT	X	1973796	1974523	WBGene00018687	F52D2.5	F52D2.5	0.126997835622628	AGIWUR4221;AGIWUR5847;AGIWUR24939;AGIWUR26041;AGIWUR36663;AGIWUR42686
CB4856	CT	X	1978014	1980938	WBGene00219451	F52D2.12	F52D2.12	0.10221360885456	AGIWUR34636
CB4856	CT	X	1983714	1984833	WBGene00018690	fbxa-46	F52D2.8	0.0615213761292055	AGIWUR9476;AGIWUR13269;AGIWUR27954;AGIWUR29303
CB4856	CT	X	1986429	1989607	WBGene00001569	meg-3	F52D2.4	-0.274817592731129	AGIWUR30104
CB4856	CT	X	1990734	1992649	WBGene00021484	Y40A1A.2	Y40A1A.2	0.0231230360089754	AGIWUR11963;AGIWUR40969;AGIWUR41408
CB4856	CT	X	1995731	1998112	WBGene00021485	Y40A1A.3	Y40A1A.3	0.00571049969689064	AGIWUR7356;AGIWUR27578
CB4856	CT	X	2001030	2002631	WBGene00021483	Y40A1A.1	Y40A1A.1	-0.113395290081386	AGIWUR41690
CB4856	CT	X	2013151	2018062	WBGene00022419	Y102A11A.9	Y102A11A.9	-0.0816695358748518	AGIWUR27143
CB4856	CT	X	2019148	2025737	WBGene00022413	Y102A11A.3	Y102A11A.3	0.308489407423313	AGIWUR42775
CB4856	CT	X	2026642	2031010	WBGene00022412	Y102A11A.2	Y102A11A.2	0.458182915819576	AGIWUR41940
CB4856	CT	X	2030959	2031588	WBGene00022415	Y102A11A.5	Y102A11A.5	0.233076837272632	AGIWUR14358;AGIWUR22481;AGIWUR35764;AGIWUR37140;AGIWUR41055
CB4856	CT	X	2031963	2035245	WBGene00022416	Y102A11A.6	Y102A11A.6	0.414841994601518	AGIWUR11400
CB4856	CT	X	2035943	2038220	WBGene00022417	Y102A11A.7	Y102A11A.7	-0.464922405474365	AGIWUR24151
CB4856	CT	X	2039397	2046360	WBGene00022418	igcm-4	Y102A11A.8	0.510535666558125	AGIWUR5133;AGIWUR5331;AGIWUR6948;AGIWUR10058;AGIWUR18787;AGIWUR21402
CB4856	CT	X	2051907	2054048	WBGene00022411	Y102A11A.1	Y102A11A.1	-0.266394161654347	

AGIWUR11614
CB4856 CT X 2065038 2066567 WBGene00044753 F49H12.7 F49H12.7 0.282194007870265
AGIWUR14793
CB4856 CT X 2066554 2070441 WBGene00018657 acl-4 F49H12.6 0.0904599765042704
AGIWUR9065;AGIWUR9826;AGIWUR20288
CB4856 CT X 2074448 2075944 WBGene00018656 F49H12.5 F49H12.5 -0.55332839508047
AGIWUR5705
CB4856 CT X 2079836 2081487 WBGene00018655 F49H12.4 F49H12.4 -0.435869533033861
AGIWUR5627
CB4856 CT X 2084375 2085057 WBGene00018654 F49H12.3 F49H12.3 0.20676517327841 8
AGIWUR30404
CB4856 CT X 2086057 2086730 WBGene00018653 F49H12.2 F49H12.2 0.0857043191846687
AGIWUR43736
CB4856 CT X 2089659 2093253 WBGene00003087 lsy-2 F49H12.1 0.0952692814578313
AGIWUR50;AGIWUR1628;AGIWUR22146;AGIWUR28258;AGIWUR28522;AGIWUR33708;AGIWUR37286
CB4856 CT X 2093475 2094842 WBGene00000737 col-164 T21D9.1 -0.428099258824339 AGIWUR4292
CB4856 CT X 2095370 2096058 WBGene00020645 T21D9.2 T21D9.2 0.0582259518682093 AGIWUR16571
CB4856 CT X 2105629 2107170 WBGene00018736 F53B1.3 F53B1.3 -0.0373885551398443
AGIWUR22369;AGIWUR33958
CB4856 CT X 2108982 2111441 WBGene00018735 F53B1.2 F53B1.2 -0.220702997492189 AGIWUR28367
CB4856 CT X 2111397 2113103 WBGene00018737 F53B1.4 F53B1.4 0.425524230428827 AGIWUR11271
CB4856 CT X 2114629 2118257 WBGene00018738 swip-10 F53B1.6 0.367526826659215 AGIWUR20532
CB4856 CT X 2118894 2121061 WBGene00001667 gpa-5 F53B1.7 -0.167296496886615 AGIWUR2116
CB4856 CT X 2123413 2130241 WBGene00018739 F53B1.8 F53B1.8 0.0522613863462675
AGIWUR11157;AGIWUR24254;AGIWUR26319
CB4856 CT X 2144605 2145647 WBGene00001065 dpy-3 EGAP7.1 -0.512199938707329 AGIWUR27602
CB4856 CT X 2147614 2149252 WBGene00018591 npax-2 F48B9.5 -0.0196765167128898 AGIWUR4555
CB4856 CT X 2163384 2164069 WBGene00018590 nlp-37 F48B9.4 0.492492773022821 AGIWUR34654
CB4856 CT X 2165109 2173849 WBGene00018589 F48B9.3 F48B9.3 -0.0324684665180071
AGIWUR2612;AGIWUR8870;AGIWUR28980;AGIWUR42374
CB4856 CT X 2173890 2176411 WBGene00018593 F48B9.8 F48B9.8 -0.14576869229775 AGIWUR31971
CB4856 CT X 2180888 2185854 WBGene00018587 F48B9.1 F48B9.1 0.0823747812313043 AGIWUR27228
CB4856 CT X 2192504 2195348 WBGene00001515 gap-1 T24C12.2 0.101291672548524
AGIWUR24037
CB4856 CT X 2196525 2203961 WBGene00020765 T24C12.3 T24C12.3 -0.0444543173242966
AGIWUR34456
CB4856 CT X 2204050 2205063 WBGene00020766 T24C12.4 T24C12.4 -1.04914569630076
AGIWUR31433
CB4856 CT X 2215630 2218980 WBGene00003968 peb-1 T14F9.4 0.628094191283387

AGIWUR21146;AGIWUR30492
CB4856 CT X 2221150 2226620 WBGene00020509 hex-1 T14F9.3 0.100605077520617
AGIWUR1715;AGIWUR24945;AGIWUR26864;AGIWUR32391
CB4856 CT X 2229208 2230122 WBGene00003018 lin-32 T14F9.5 0.882570591675527 AGIWUR33532
CB4856 CT X 2234321 2236706 WBGene00020508 T14F9.2 T14F9.2 -0.269679951795972 AGIWUR30683
CB4856 CT X 2237911 2240255 WBGene00020507 vha-15 T14F9.1 -0.254794399508135 AGIWUR23485
CB4856 CT X 2241738 2242618 WBGene00004423 rpl-11.2 F07D10.1 0.0186181490010053
AGIWUR4463;AGIWUR5995;AGIWUR11434;AGIWUR20528;AGIWUR26031;AGIWUR36131;AGIWUR37677;AGIWUR38852
CB4856 CT X 2255852 2258101 WBGene00000797 crn-4 AH9.2 -0.262080941819118 AGIWUR16169
CB4856 CT X 2259150 2260526 WBGene00014999 AH9.3 AH9.3 -0.0339899345481207 AGIWUR8206
CB4856 CT X 2263176 2267472 WBGene00044701 AH9.6 AH9.6 -0.0701368804341178
AGIWUR1909;AGIWUR4754
CB4856 CT X 2271025 2274337 WBGene00015000 AH9.4 AH9.4 -0.0393252426886042 AGIWUR29159
CB4856 CT X 2283998 2287188 WBGene00020427 sav-1 T10H10.3 0.00166942222929833
AGIWUR35953
CB4856 CT X 2294750 2298193 WBGene00020426 T10H10.2 T10H10.2 0.750486379667392
AGIWUR43952
CB4856 CT X 2303082 2314914 WBGene00002039 hum-6 T10H10.1 0.363638294890849
AGIWUR38516
CB4856 CT X 2314896 2319333 WBGene00020135 aakg-4 T01B6.3 0.83305967480009
AGIWUR13944;AGIWUR20760
CB4856 CT X 2335684 2348635 WBGene00020134 T01B6.1 T01B6.1 0.399238646259764
AGIWUR3958;AGIWUR4904;AGIWUR12927;AGIWUR17462;AGIWUR19138;AGIWUR21280;AGIWUR22799;AGIWUR24274;AGIWUR31031;A
GIWUR34130;AGIWUR35858;AGIWUR38941;AGIWUR41041;AGIWUR43996
CB4856 CT X 2360097 2364818 WBGene00020767 lgc-40 T24D8.1 -0.290056797638185 AGIWUR21614
CB4856 CT X 2365700 2365942 WBGene00020769 T24D8.6 T24D8.6 0.00723861155875882
AGIWUR7743;AGIWUR26204;AGIWUR32420;AGIWUR34389;AGIWUR36696;AGIWUR44279
CB4856 CT X 2375997 2379060 WBGene00018578 F47G3.3 F47G3.3 0.0368165988416713 AGIWUR44829
CB4856 CT X 2382691 2384764 WBGene00018577 asna-2 F47G3.2 -0.351190676965033 AGIWUR19401
CB4856 CT X 2385765 2392695 WBGene00018576 F47G3.1 F47G3.1 -0.16494939582293 AGIWUR11303
CB4856 CT X 2395505 2398826 WBGene00016619 C43H6.6 C43H6.6 0.327056577718777
AGIWUR2029;AGIWUR18869;AGIWUR19237;AGIWUR31119;AGIWUR35215
CB4856 CT X 2398812 2402057 WBGene00016620 dhhc-9 C43H6.7 -0.0987297654676187
AGIWUR5071;AGIWUR27531;AGIWUR40331
CB4856 CT X 2404024 2407364 WBGene00016617 C43H6.4 C43H6.4 -0.15696919732625 AGIWUR3069
CB4856 CT X 2407467 2409567 WBGene00016616 C43H6.3 C43H6.3 -0.0909327904206454
AGIWUR6923;AGIWUR21325
CB4856 CT X 2409570 2412966 WBGene00016614 trpp-12 C43H6.1 -0.435211262675503
AGIWUR4699;AGIWUR9360;AGIWUR22546;AGIWUR25479;AGIWUR36654

CB4856 CT X 2412953 2413377 WBGene00001959 hlh-15 C43H6.8 0.226931460268913 AGIWUR16405
CB4856 CT X 2413909 2417892 WBGene00001618 glr-7 C43H6.9 0.0929899872270541 AGIWUR17578
CB4856 CT X 2418425 2419877 WBGene00020303 T07D1.5 T07D1.5 0.189758123790469 AGIWUR37199
CB4856 CT X 2420462 2427504 WBGene00001551 gcy-31 T07D1.1 -0.095140300133477 AGIWUR2312
CB4856 CT X 2429862 2439158 WBGene00020301 T07D1.2 T07D1.2 0.220974625219197
AGIWUR7384;AGIWUR28486;AGIWUR34400
CB4856 CT X 2442970 2443404 WBGene00020302 T07D1.3 T07D1.3 0.59077064805181 AGIWUR44069
CB4856 CT X 2445500 2455949 WBGene00001484 fox-1 T07D1.4 0.326740040533527
AGIWUR3748;AGIWUR30879;AGIWUR33746
CB4856 CT X 2467347 2471308 WBGene00019866 R04B3.1 R04B3.1 0.450675267463281 AGIWUR4954
CB4856 CT X 2471540 2473718 WBGene00019867 R04B3.2 R04B3.2 -0.195316397292114 AGIWUR3145
CB4856 CT X 2473758 2476030 WBGene00019868 R04B3.3 R04B3.3 -0.0766089784778963 AGIWUR2415
CB4856 CT X 2477107 2478765 WBGene00019319 K02E10.5 K02E10.5 -0.189353927837879
AGIWUR29502
CB4856 CT X 2480755 2482911 WBGene00019320 K02E10.6 K02E10.6 -0.0904328103307925
AGIWUR36677
CB4856 CT X 2483263 2488296 WBGene00019318 K02E10.4 K02E10.4 0.139280299557055
AGIWUR8951;AGIWUR15514;AGIWUR32000;AGIWUR32607
CB4856 CT X 2489044 2494339 WBGene00001844 hid-1 K02E10.2 0.128608805318168
AGIWUR9595;AGIWUR16184;AGIWUR22838;AGIWUR23855;AGIWUR43646;AGIWUR44331
CB4856 CT X 2496180 2501001 WBGene00019317 K02E10.1 K02E10.1 0.29781773696973
AGIWUR9485
CB4856 CT X 2501003 2508549 WBGene00019321 K02E10.7 K02E10.7 -0.150256659419137
AGIWUR45028
CB4856 CT X 2509608 2515317 WBGene00006365 syg-1 K02E10.8 0.202339091379339
AGIWUR8570;AGIWUR29333
CB4856 CT X 2525191 2532457 WBGene00044061 tbc-12 R11B5.1 0.0747188862544181 AGIWUR30102
CB4856 CT X 2540066 2541053 WBGene00018715 F52H2.3 F52H2.3 0.49940225159925 AGIWUR6056
CB4856 CT X 2543391 2548028 WBGene00018716 F52H2.4 F52H2.4 0.119612730401789 AGIWUR26943
CB4856 CT X 2552436 2557726 WBGene00000004 aat-3 F52H2.2 0.21330267121153 AGIWUR21672
CB4856 CT X 2557976 2559638 WBGene00018717 F52H2.5 F52H2.5 -0.462229607968791 AGIWUR7255
CB4856 CT X 2559727 2564147 WBGene00018718 F52H2.6 F52H2.6 0.0228100703030365
AGIWUR1964;AGIWUR9945;AGIWUR11343;AGIWUR35339
CB4856 CT X 2566557 2572653 WBGene00018719 F52H2.7 F52H2.7 0.225896662432021 AGIWUR9017
CB4856 CT X 2572863 2576697 WBGene00018714 F52H2.1 F52H2.1 0.1453252652877 AGIWUR23592
CB4856 CT X 2577742 2578262 WBGene00045268 ttr-42 H01M10.3 -0.398942096570455
AGIWUR18181;AGIWUR31853
CB4856 CT X 2578211 2580574 WBGene00019143 H01M10.1 H01M10.1 -0.0779138644953754
AGIWUR29748

CB4856 CT X 2584539 2585739 WBGene00019144 H01M10.2 H01M10.2 -0.108632359513647
AGIWUR43764
CB4856 CT X 2594003 2606732 WBGene00003630 nhr-40 T03G6.2 0.190808790069253
AGIWUR203;AGIWUR11036;AGIWUR21316;AGIWUR45083
CB4856 CT X 2608228 2609989 WBGene00020194 T03G6.1 T03G6.1 1.03965477794919 AGIWUR42652
CB4856 CT X 2610826 2615726 WBGene00020195 T03G6.3 T03G6.3 0.2622138496787 AGIWUR39954
CB4856 CT X 2621274 2664373 WBGene00018837 F54G2.1 F54G2.1 0.0266565133111509
AGIWUR21188;AGIWUR34450
CB4856 CT X 2672427 2672759 WBGene00022919 C09B7.4 C09B7.4 0.0199076745416411
AGIWUR472;AGIWUR11888;AGIWUR14220;AGIWUR26186;AGIWUR35518
CB4856 CT X 2673471 2682379 WBGene00004780 ser-7 C09B7.1 -0.143735548469596
AGIWUR21887;AGIWUR40148;AGIWUR40519
CB4856 CT X 2682754 2687184 WBGene00020511 immt-1 T14G11.3 -0.126615675789555
AGIWUR21136
CB4856 CT X 2688945 2692022 WBGene00020510 T14G11.1 T14G11.1 0.166264478403122
AGIWUR2431;AGIWUR17996;AGIWUR22135;AGIWUR23764;AGIWUR32517;AGIWUR32564;AGIWUR35471;AGIWUR39291;AGIWUR39542
CB4856 CT X 2694908 2698273 WBGene00020158 T02C5.1 T02C5.1 0.455147744682595
AGIWUR40757;AGIWUR43458
CB4856 CT X 2698257 2705859 WBGene00020160 igcm-3 T02C5.3 0.856845854472482
AGIWUR21354;AGIWUR38472;AGIWUR40980
CB4856 CT X 2710918 2748114 WBGene00006742 unc-2 T02C5.5 0.157997059752793
AGIWUR282;AGIWUR15155
CB4856 CT X 2769588 2770060 WBGene00018888 F55F1.2 F55F1.2 0.229310892770317 AGIWUR62
CB4856 CT X 2772042 2799247 WBGene00006496 cgef-1 C14A11.3 0.0840177900394035
AGIWUR17834;AGIWUR33860;AGIWUR34012;AGIWUR36036
CB4856 CT X 2803112 2804830 WBGene00015748 C14A11.2 C14A11.2 0.652595109643073
AGIWUR21605
CB4856 CT X 2805075 2807528 WBGene00015750 C14A11.5 C14A11.5 0.172241056951599
AGIWUR28637;AGIWUR29994;AGIWUR33322
CB4856 CT X 2807911 2809635 WBGene00050944 C14A11.9 C14A11.9 -0.0456647820990064
AGIWUR28185
CB4856 CT X 2810484 2814125 WBGene00015751 C14A11.6 C14A11.6
0.000525036566919379 AGIWUR40572;AGIWUR42224
CB4856 CT X 2820431 2822433 WBGene00006632 tsp-6 C14A11.1 0.210089566761855
AGIWUR4926;AGIWUR10463;AGIWUR16224;AGIWUR16558
CB4856 CT X 2823501 2826827 WBGene00006055 ssr-2 C14A11.7 0.209352501324683
AGIWUR1100
CB4856 CT X 2834222 2840292 WBGene00022201 Y71H10B.1 Y71H10B.1 -0.143583590833375
AGIWUR944;AGIWUR5971;AGIWUR7849;AGIWUR9130;AGIWUR17991;AGIWUR18894;AGIWUR19077;AGIWUR38626;AGIWUR43058

CB4856	CT	X	2843922 2852520 WBGene00002210	kin-29	F58H12.1	0.115484997996417
AGIWUR5264;AGIWUR14602;AGIWUR16974;AGIWUR23969;AGIWUR32652
CB4856	CT	X	2853678 2857603 WBGene00018740	tra-4	F53B3.1 -0.156953744186931
AGIWUR2299;AGIWUR17562
CB4856	CT	X	2864211 2865683 WBGene00018744	F53B3.6 F53B3.6 -0.142765872168699	AGIWUR12334
CB4856	CT	X	2865960 2871060 WBGene00018742	F53B3.3 F53B3.3 0.300192608489468
AGIWUR5232;AGIWUR8771;AGIWUR37860
CB4856	CT	X	2872739 2879619 WBGene00018743	F53B3.5 F53B3.5 1.10559579473724	AGIWUR37342
CB4856	CT	X	2885863 2890625 WBGene00015171	vang-1	B0410.2 -0.0434843003220724	AGIWUR27637
CB4856	CT	X	2890612 2891491 WBGene00015172	B0410.3 B0410.3 0.249124083345539	AGIWUR35784
CB4856	CT	X	2892484 2898356 WBGene00044304	F52B10.3	F52B10.3	0.12932179352851
AGIWUR17121;AGIWUR31609
CB4856	CT	X	2909536 2918038 WBGene00003776	nmy-1	F52B10.1	0.309074658632239
AGIWUR2608;AGIWUR3742;AGIWUR6745;AGIWUR33243;AGIWUR39326;AGIWUR44379
CB4856	CT	X	2918730 2923984 WBGene00044303	hst-3.2 F52B10.2	0.353556762967842
AGIWUR3291;AGIWUR40841
CB4856	CT	X	2933361 2937740 WBGene00022200	fard-1	Y71H10A.2	0.0375332371636468
AGIWUR7983;AGIWUR12871;AGIWUR22212
CB4856	CT	X	2940872 2945481 WBGene00022199	pfk-1.1 Y71H10A.1	0.124478740445676
AGIWUR6779;AGIWUR29236
CB4856	CT	X	2945803 2946570 WBGene00044901	Y41G9A.10	Y41G9A.10	0.232202674989839
AGIWUR24427;AGIWUR27514;AGIWUR27676;AGIWUR28212;AGIWUR34418
CB4856	CT	X	2946667 2949025 WBGene00021529	Y41G9A.5	Y41G9A.5	0.352572001187923
AGIWUR26260
CB4856	CT	X	2957934 2968167 WBGene00021528	gbb-1	Y41G9A.4	-0.0162891585159728
AGIWUR24071;AGIWUR30929
CB4856	CT	X	2976457 2981008 WBGene00021527	aakg-3	Y41G9A.3	-0.248817327376438
AGIWUR36721
CB4856	CT	X	2982056 2984125 WBGene00021526	Y41G9A.2	Y41G9A.2	0.437807167016592
AGIWUR14896
CB4856	CT	X	2984278 2991375 WBGene00003885	osm-5	Y41G9A.1	0.643527204126863
AGIWUR29544
CB4856	CT	X	2998303 3003803 WBGene00021530	Y41G9A.6	Y41G9A.6	0.23038295687671
AGIWUR4734
CB4856	CT	X	3009499 3010175 WBGene00021656	Y48D7A.1	Y48D7A.1	0.00614323033136177
AGIWUR39448
CB4856	CT	X	3012190 3018555 WBGene00001461	flp-18	Y48D7A.2	0.327287088986422
AGIWUR22573
CB4856	CT	X	3025505 3026976 WBGene00000175	aqp-7	M02F4.8 0.00863890604098216	AGIWUR18600

CB4856 CT X 3027253 3030762 WBGene00019735 M02F4.1 M02F4.1 -0.261313983742312 AGIWUR11119
CB4856 CT X 3031723 3033203 WBGene00019738 clec-265 M02F4.7 -0.873086062978018
AGIWUR442
CB4856 CT X 3033325 3034303 WBGene00019736 M02F4.2 M02F4.2 0.11924241325404 AGIWUR18919
CB4856 CT X 3036427 3045806 WBGene00016343 cnnm-3 C33D12.2 -0.0671786962631993
AGIWUR2882;AGIWUR5098;AGIWUR5720;AGIWUR7394;AGIWUR24199;AGIWUR24738;AGIWUR31588;AGIWUR39336
CB4856 CT X 3050365 3054585 WBGene00006679 twk-26 C33D12.3 -0.63813000444604
AGIWUR28243
CB4856 CT X 3055257 3059385 WBGene00016344 rsef-1 C33D12.6 0.067349242813135
AGIWUR2392
CB4856 CT X 3063585 3065551 WBGene00000451 ceh-30 C33D12.7 0.459804959006261
AGIWUR20536
CB4856 CT X 3068650 3070396 WBGene00000452 ceh-31 C33D12.1 0.355772417327776
AGIWUR1382
CB4856 CT X 3081125 3085336 WBGene00006670 twk-16 F52E4.4 -0.372870891370983
AGIWUR10197;AGIWUR23264;AGIWUR27801;AGIWUR28179
CB4856 CT X 3085829 3087345 WBGene00018702 F52E4.5 F52E4.5 -0.0332008344273031 AGIWUR13956
CB4856 CT X 3089496 3092457 WBGene00018701 pccb-1 F52E4.1 0.113431335596953
AGIWUR1837;AGIWUR6654;AGIWUR9003;AGIWUR12787;AGIWUR20116;AGIWUR32561;AGIWUR32604;AGIWUR40672;AGIWUR41415
CB4856 CT X 3092395 3094448 WBGene00006948 wrt-2 F52E4.6 0.113409871177848
AGIWUR373;AGIWUR722;AGIWUR13619
CB4856 CT X 3095434 3099837 WBGene00018703 sec-3 F52E4.7 -0.101294659528076 AGIWUR41191
CB4856 CT X 3103250 3106279 WBGene00019611 K10B3.5 K10B3.5 0.102729133218153
AGIWUR25536;AGIWUR25538
CB4856 CT X 3106543 3110630 WBGene00019612 gpcp-1 K10B3.6 0.161351441068748
AGIWUR17177;AGIWUR26068;AGIWUR44080
CB4856 CT X 3112083 3113382 WBGene00001685 gpd-3 K10B3.7 -0.0489106387500646
AGIWUR6643;AGIWUR7153;AGIWUR12023;AGIWUR23960;AGIWUR36409;AGIWUR36989
CB4856 CT X 3113434 3114771 WBGene00001684 gpd-2 K10B3.8 -0.348026643527531 AGIWUR22539
CB4856 CT X 3114668 3116538 WBGene00003124 mai-1 K10B3.9 -0.073637327291241 AGIWUR35840
CB4856 CT X 3117439 3127073 WBGene00004951 spc-1 K10B3.10 0.157796907721003
AGIWUR4438;AGIWUR7942;AGIWUR9370;AGIWUR10005;AGIWUR12089;AGIWUR31510;AGIWUR42440
CB4856 CT X 3135486 3136663 WBGene00019609 K10B3.1 K10B3.1 -0.179017533257664
AGIWUR7520;AGIWUR7583;AGIWUR12761;AGIWUR22048;AGIWUR25116;AGIWUR44994
CB4856 CT X 3146573 3147088 WBGene00015790 C15C7.4 C15C7.4 0.430830200649204 AGIWUR34652
CB4856 CT X 3151148 3152461 WBGene00015791 C15C7.5 C15C7.5 -0.101665080716163
AGIWUR560;AGIWUR19372;AGIWUR23873;AGIWUR28059;AGIWUR30270;AGIWUR39946
CB4856 CT X 3159940 3164424 WBGene00002220 gcc-1 C15C7.2 -0.154461837798638
AGIWUR4551;AGIWUR14889;AGIWUR28773;AGIWUR30268;AGIWUR30613;AGIWUR43739

CB4856	CT	X	3164525 3165650 WBGene00015789	syx-6	C15C7.1 0.095167653901751
AGIWUR1944;AGIWUR3669;AGIWUR41127;AGIWUR45171
CB4856	CT	X	3165796 3167579 WBGene00015793	C15C7.7 C15C7.7 0.386504720846865	AGIWUR40953
CB4856	CT	X	3167570 3170556 WBGene00015792	C15C7.6 C15C7.6 0.0728887588460254
AGIWUR6524;AGIWUR10308;AGIWUR33805;AGIWUR40453
CB4856	CT	X	3171424 3178695 WBGene00002217	klp-4	F56E3.3 0.0979423511768758
AGIWUR3576;AGIWUR7058;AGIWUR15218;AGIWUR26622;AGIWUR30042;AGIWUR32297;AGIWUR33608
CB4856	CT	X	3206818 3209644 WBGene00017893	F28B4.4 F28B4.4 0.324147792893111	AGIWUR22272
CB4856	CT	X	3210470 3220367 WBGene00017891	rgl-1	F28B4.2 0.097692615977552
AGIWUR11313;AGIWUR17316;AGIWUR20300;AGIWUR36614;AGIWUR36742;AGIWUR40752
CB4856	CT	X	3227489 3235408 WBGene00017892	F28B4.3 F28B4.3 -0.67854706119968	AGIWUR28431
CB4856	CT	X	3235768 3243624 WBGene00018237	irg-7	F40F4.6 -0.446231151580029	AGIWUR39079
CB4856	CT	X	3243886 3245980 WBGene00018238	F40F4.7 F40F4.7 -0.0308293355721077	AGIWUR14512
CB4856	CT	X	3247045 3250529 WBGene00004124	pqn-37	F40F4.8 -0.408402033735976	AGIWUR35686
CB4856	CT	X	3252963 3256265 WBGene00006535	tba-9	F40F4.5 -0.00809849292758358	AGIWUR37481
CB4856	CT	X	3257010 3258155 WBGene00002255	lbp-3	F40F4.4 -0.166190617948814
AGIWUR4882;AGIWUR22843;AGIWUR31099
CB4856	CT	X	3259087 3259855 WBGene00002253	lbp-1	F40F4.3 0.829361877189648	AGIWUR15823
CB4856	CT	X	3260610 3261470 WBGene00002254	lbp-2	F40F4.2 -0.31654995529448	AGIWUR17137
CB4856	CT	X	3262900 3264161 WBGene00018236	fbxb-71 F40F4.1 0.227599555627767	AGIWUR33431
CB4856	CT	X	3267294 3270906 WBGene00006308	sul-1	K09C4.8 0.0515445329614472	AGIWUR36553
CB4856	CT	X	3271353 3271773 WBGene00019553	K09C4.9 K09C4.9 0.029354112887706	AGIWUR12345
CB4856	CT	X	3272730 3273155 WBGene00019552	K09C4.7 K09C4.7 0.121617453292561	AGIWUR7564
CB4856	CT	X	3274757 3275768 WBGene00019551	K09C4.6 K09C4.6 0.0251912511792289	AGIWUR37707
CB4856	CT	X	3276020 3280230 WBGene00019554	K09C4.10	K09C4.10	-0.238849802097567
AGIWUR18845
CB4856	CT	X	3282602 3285721 WBGene00019550	K09C4.5 K09C4.5 -0.0802607202585888
AGIWUR1581;AGIWUR33875;AGIWUR44903
CB4856	CT	X	3289485 3292412 WBGene00019549	K09C4.4 K09C4.4 0.353503690775034	AGIWUR18440
CB4856	CT	X	3293290 3294369 WBGene00002006	hsp-2	K09C4.3 -0.335209259989711
AGIWUR17108;AGIWUR24868;AGIWUR30948;AGIWUR37512
CB4856	CT	X	3296482 3297993 WBGene00019548	K09C4.2 K09C4.2 -0.00526438082903802	AGIWUR6444
CB4856	CT	X	3298832 3301637 WBGene00019547	K09C4.1 K09C4.1 1.57097695567954	AGIWUR35316
CB4856	CT	X	3302070 3303927 WBGene00017382	F11D5.5 F11D5.5 0.845851098619425
AGIWUR19390;AGIWUR29499
CB4856	CT	X	3304026 3306939 WBGene00043980	F11D5.7 F11D5.7 0.122196583453595
AGIWUR17430;AGIWUR29001;AGIWUR29773
CB4856	CT	X	3315321 3324229 WBGene00017381	ddr-2	F11D5.3 0.61340082201685	AGIWUR26574
CB4856	CT	X	3325166 3326275 WBGene00017383	F11D5.6 F11D5.6 0.0444085480530596

AGIWUR6111;AGIWUR19770;AGIWUR19885;AGIWUR31566;AGIWUR31777
CB4856 CT X 3333130 3347302 WBGene00017380 F11D5.1 F11D5.1 0.314932852335888
AGIWUR21170;AGIWUR35228;AGIWUR36050
CB4856 CT X 3348121 3348642 WBGene00022826 ZK816.3 ZK816.3 -0.0961030408434977 AGIWUR28879
CB4856 CT X 3348878 3350401 WBGene00022827 ZK816.4 ZK816.4 0.618403084675149 AGIWUR5143
CB4856 CT X 3359062 3360656 WBGene00022825 ZK816.1 ZK816.1 -0.00381742505810312 AGIWUR7579
CB4856 CT X 3360722 3362493 WBGene00000989 dhs-26 ZK816.5 -0.168061883916216
AGIWUR30451;AGIWUR35473
CB4856 CT X 3368959 3373217 WBGene00022767 ZK563.2 ZK563.2 0.0658117354181034 AGIWUR18934
CB4856 CT X 3373247 3374740 WBGene00000524 clc-3 ZK563.4 -0.120628401155706 AGIWUR27033
CB4856 CT X 3376874 3379234 WBGene00022769 sorf-1 ZK563.5 -0.0836889767314251
AGIWUR5888;AGIWUR12956;AGIWUR13650;AGIWUR25096;AGIWUR33967;AGIWUR43263
CB4856 CT X 3380480 3382838 WBGene00044355 ZK563.7 ZK563.7 -0.0409286811145995
AGIWUR34423;AGIWUR42244
CB4856 CT X 3382868 3385547 WBGene00022770 acp-1 ZK563.6 -0.347222956937062 AGIWUR39590
CB4856 CT X 3393864 3397656 WBGene00006865 slcf-2 ZK563.1 0.158135523187869
AGIWUR37355;AGIWUR38288
CB4856 CT X 3397613 3400017 WBGene00000503 cht-1 C04F6.3 0.547386335177253 AGIWUR35268
CB4856 CT X 3408664 3411683 WBGene00006810 unc-78 C04F6.4 -0.0791402781346378
AGIWUR1669;AGIWUR16113;AGIWUR17514;AGIWUR25638
CB4856 CT X 3412876 3414708 WBGene00000990 dhs-27 C04F6.5 -0.102043630937972 AGIWUR29086
CB4856 CT X 3424816 3427337 WBGene00044564 ZK813.6 ZK813.6 1.18544488439134
AGIWUR15263;AGIWUR34459
CB4856 CT X 3427372 3430120 WBGene00022823 ZK813.4 ZK813.4 -0.0565804040085389 AGIWUR25241
CB4856 CT X 3441839 3442458 WBGene00022822 ZK813.3 ZK813.3 -0.0516337170780616
AGIWUR10722;AGIWUR12466;AGIWUR20222;AGIWUR20531;AGIWUR38971
CB4856 CT X 3443782 3444420 WBGene00022821 ZK813.2 ZK813.2 0.288942357797197 AGIWUR28329
CB4856 CT X 3444802 3445283 WBGene00045394 ZK813.7 ZK813.7 0.033431549010853
AGIWUR3984;AGIWUR4660;AGIWUR9902;AGIWUR11733;AGIWUR22422;AGIWUR26236;AGIWUR35956;AGIWUR36513;AGIWUR44011
CB4856 CT X 3446311 3448253 WBGene00022820 ZK813.1 ZK813.1 0.234204096077157 AGIWUR8539
CB4856 CT X 3448744 3451222 WBGene00022824 ZK813.5 ZK813.5 0.412997778971043 AGIWUR14288
CB4856 CT X 3451945 3465925 WBGene00004729 sax-3 ZK377.2 0.135586092872358
AGIWUR15391;AGIWUR19547;AGIWUR28949;AGIWUR29051;AGIWUR37636;AGIWUR42492
CB4856 CT X 3480251 3483649 WBGene00006952 wrt-6 ZK377.1 0.0707509885776414
AGIWUR4539;AGIWUR16131;AGIWUR26666;AGIWUR32164;AGIWUR34901
CB4856 CT X 3486309 3486838 WBGene00015715 C12D12.4 C12D12.4 0.22777081833399
AGIWUR27243
CB4856 CT X 3487101 3487996 WBGene00022922 C12D12.7 C12D12.7 -0.029552484683613
AGIWUR17188

CB4856 CT X 3489293 3491155 WBGene00015714 C12D12.3 C12D12.3 0.0794278150261416
AGIWUR20187;AGIWUR27237;AGIWUR28908;AGIWUR33500
CB4856 CT X 3496026 3499165 WBGene00001620 glt-1 C12D12.2 -0.181708850646004
AGIWUR20640;AGIWUR30222
CB4856 CT X 3499253 3505251 WBGene00015713 C12D12.1 C12D12.1 -0.376301953937849
AGIWUR17039;AGIWUR19364
CB4856 CT X 3505286 3511529 WBGene00015716 sox-4 C12D12.5 -0.231030837504486
AGIWUR772;AGIWUR8579;AGIWUR38135
CB4856 CT X 3528812 3531826 WBGene00006864 npr-16 F56B6.5 0.450357679712075 AGIWUR9194
CB4856 CT X 3531841 3532811 WBGene00018937 F56B6.6 F56B6.6 -0.0312787339510265
AGIWUR2309;AGIWUR7014;AGIWUR24773;AGIWUR28044;AGIWUR29035;AGIWUR36026
CB4856 CT X 3537641 3540940 WBGene00006863 gyg-1 F56B6.4 0.0293197202148594
AGIWUR4402;AGIWUR40739
CB4856 CT X 3542558 3557599 WBGene00004350 rgs-7 F56B6.2 0.183592312819781
AGIWUR8199;AGIWUR17813;AGIWUR29479;AGIWUR32324;AGIWUR32993;AGIWUR34005
CB4856 CT X 3558839 3563974 WBGene00006928 vit-4 F59D8.2 -0.396713903339302
AGIWUR3280;AGIWUR20585
CB4856 CT X 3567355 3572549 WBGene00006927 vit-3 F59D8.1 -0.250794723954478 AGIWUR15620
CB4856 CT X 3573051 3573562 WBGene00020845 T27A10.5 T27A10.5 -0.434733877405574
AGIWUR1640
CB4856 CT X 3576608 3578830 WBGene00000515 ckc-1 T27A10.3 -0.706913259430314
AGIWUR449;AGIWUR4215;AGIWUR8145
CB4856 CT X 3588858 3596497 WBGene00020846 T27A10.6 T27A10.6 -0.344190767261829
AGIWUR4757;AGIWUR15179;AGIWUR15354
CB4856 CT X 3597276 3600475 WBGene00020847 cgr-1 T27A10.7 -0.0607285379009816
AGIWUR2139
CB4856 CT X 3601800 3604360 WBGene00015954 C18B2.3 C18B2.3 -0.0491477969428775 AGIWUR35650
CB4856 CT X 3604291 3605741 WBGene00015955 C18B2.4 C18B2.4 -0.0284427549585917 AGIWUR41181
CB4856 CT X 3606668 3611092 WBGene00015956 C18B2.5 C18B2.5 -0.23323794554823
AGIWUR14917;AGIWUR15676
CB4856 CT X 3613353 3617378 WBGene00015957 del-9 C18B2.6 0.48963368792628 AGIWUR40349
CB4856 CT X 3623522 3628359 WBGene00015953 C18B2.2 C18B2.2 0.135252700651585 AGIWUR37215
CB4856 CT X 3628316 3630884 WBGene00015952 C18B2.1 C18B2.1 -0.23655132643788 AGIWUR28851
CB4856 CT X 3632367 3633747 WBGene00001759 gst-11 R11G1.3 -0.200775727160607
AGIWUR1123;AGIWUR11838;AGIWUR17670;AGIWUR34809
CB4856 CT X 3639189 3644896 WBGene00020012 R11G1.6 R11G1.6 0.0873338902632455
AGIWUR18151;AGIWUR22592;AGIWUR35254;AGIWUR36233;AGIWUR36775
CB4856 CT X 3646320 3650150 WBGene00004727 sax-1 R11G1.4 0.246742023116013
AGIWUR3701;AGIWUR7535;AGIWUR44764

CB4856 CT X 3652017 3655419 WBGene00020011 R11G1.2 R11G1.2 0.198881061499202
AGIWUR18566;AGIWUR29373
CB4856 CT X 3656272 3657265 WBGene00044725 R11G1.7 R11G1.7 0.378062286264417 AGIWUR18423
CB4856 CT X 3659492 3666879 WBGene00020010 R11G1.1 R11G1.1 0.251963192240894 AGIWUR9479
CB4856 CT X 3668100 3668942 WBGene00015354 snet-1 C02F12.3 0.28958585002718
AGIWUR3210;AGIWUR35056
CB4856 CT X 3670374 3672098 WBGene00015355 C02F12.5 C02F12.5 -0.533414164767297
AGIWUR42617
CB4856 CT X 3672378 3673918 WBGene00045215 ceh-63 C02F12.10 0.166704345593796
AGIWUR24651;AGIWUR30317;AGIWUR41987
CB4856 CT X 3674041 3678557 WBGene00006431 tag-52 C02F12.4 0.254507232877334
AGIWUR35687
CB4856 CT X 3681170 3684232 WBGene00006643 tsp-17 C02F12.1 0.0304446604266424
AGIWUR71;AGIWUR691;AGIWUR2919;AGIWUR7781;AGIWUR9164;AGIWUR10882;AGIWUR25534;AGIWUR25780;AGIWUR29313;AGIWUR4
4582;AGIWUR45202
CB4856 CT X 3684212 3689205 WBGene00015356 tag-278 C02F12.7 0.291638568457897
AGIWUR44429
CB4856 CT X 3690143 3693238 WBGene00015357 C02F12.8 C02F12.8 -0.0827650899335008
AGIWUR30932
CB4856 CT X 3696903 3700807 WBGene00015358 C02F12.9 C02F12.9 -0.0225111052517244
AGIWUR8648;AGIWUR21142;AGIWUR37573;AGIWUR38180
CB4856 CT X 3706551 3713382 WBGene00015293 C01C4.3 C01C4.3 0.324788152135142
AGIWUR101;AGIWUR13011;AGIWUR31216
CB4856 CT X 3716314 3718566 WBGene00015292 C01C4.2 C01C4.2 0.196732172663084
AGIWUR380;AGIWUR7965;AGIWUR10650;AGIWUR12661
CB4856 CT X 3727553 3728446 WBGene00003739 nlp-1 C01C4.1 0.319847339115842 AGIWUR42191
CB4856 CT X 3729281 3731960 WBGene00006454 aex-4 T14G12.2 0.443161613648256
AGIWUR5944
CB4856 CT X 3731894 3733457 WBGene00006408 tag-18 T14G12.3 -0.0674503390285892
AGIWUR26566
CB4856 CT X 3740208 3742323 WBGene00001434 fkh-2 T14G12.4 0.180832077143618
AGIWUR928;AGIWUR1294;AGIWUR4150;AGIWUR7793;AGIWUR14765;AGIWUR30544
CB4856 CT X 3763057 3764395 WBGene00018534 F47B7.3 F47B7.3 0.0155755163287794
AGIWUR32605;AGIWUR39322
CB4856 CT X 3765324 3766626 WBGene00018535 F47B7.4 F47B7.4 0.0824418289982152 AGIWUR9808
CB4856 CT X 3767795 3768817 WBGene00018536 F47B7.5 F47B7.5 0.111323997991948 AGIWUR13937
CB4856 CT X 3769731 3770643 WBGene00018537 F47B7.6 F47B7.6 -0.0885888512800239 AGIWUR41486
CB4856 CT X 3772763 3778080 WBGene00018533 F47B7.2 F47B7.2 -0.161243868369931
AGIWUR213;AGIWUR9801;AGIWUR15390;AGIWUR19993;AGIWUR23514;AGIWUR24158;AGIWUR34104;AGIWUR34713;AGIWUR37042;AG

IWUR44702
CB4856	CT	X	3778091 3781118 WBGene00018538	marg-1	F47B7.7 0.561790623257888	AGIWUR36494
CB4856	CT	X	3788026 3788532 WBGene00018532	F47B7.1 F47B7.1 -0.172251368828832
AGIWUR5087;AGIWUR6046
CB4856	CT	X	3789757 3792815 WBGene00018026	F35A5.4 F35A5.4 -0.143187079315595
AGIWUR3889;AGIWUR9459;AGIWUR9820
CB4856	CT	X	3793912 3795395 WBGene00018027	F35A5.5 F35A5.5 0.0375030518301944
AGIWUR230;AGIWUR7864;AGIWUR12138;AGIWUR12675;AGIWUR20530;AGIWUR22072;AGIWUR23940;AGIWUR29917;AGIWUR37314;AGIWUR38248;AGIWUR42205
CB4856	CT	X	3798179 3799741 WBGene00000033	abu-10	F35A5.3 -0.272378955464866	AGIWUR37428
CB4856	CT	X	3800045 3802771 WBGene00001335	erp-1	F35A5.8 -0.0499538317672057
AGIWUR6029;AGIWUR8627;AGIWUR42411;AGIWUR45109
CB4856	CT	X	3805084 3805749 WBGene00018025	F35A5.2 F35A5.2 0.566059013666209	AGIWUR43919
CB4856	CT	X	3807460 3816429 WBGene00018024	F35A5.1 F35A5.1 -0.128974423822804
AGIWUR16665;AGIWUR20440;AGIWUR21793
CB4856	CT	X	3816851 3826963 WBGene00001441	fkh-9	K03C7.2 0.2565813419584
AGIWUR1451;AGIWUR3258;AGIWUR6593;AGIWUR15006;AGIWUR16658;AGIWUR20846;AGIWUR21069;AGIWUR26982;AGIWUR27201
CB4856	CT	X	3827231 3829300 WBGene00019359	K03C7.3 K03C7.3 -0.405162509404145	AGIWUR5541
CB4856	CT	X	3840869 3842468 WBGene00022515	ttr-48	ZC64.2	-0.0580739727808153
AGIWUR7561;AGIWUR8684;AGIWUR35317;AGIWUR40258
CB4856	CT	X	3843367 3857797 WBGene00000441	ceh-18	ZC64.3	-0.172849876714704
AGIWUR2095;AGIWUR8429;AGIWUR8825;AGIWUR22735;AGIWUR27675;AGIWUR28338;AGIWUR37278
CB4856	CT	X	3869753 3873295 WBGene00002987	lim-4	ZC64.4	0.512303973019158	AGIWUR27888
CB4856	CT	X	3879782 3893309 WBGene00018569	F47F2.1 F47F2.1 0.156792132314051
AGIWUR8897;AGIWUR10596;AGIWUR21221;AGIWUR41611
CB4856	CT	X	3893964 3895677 WBGene00018571	F47F2.3 F47F2.3 0.160016888449503	AGIWUR27605
CB4856	CT	X	3904591 3907671 WBGene00006321	sup-12	T22B2.4 0.308234270123083
AGIWUR4964;AGIWUR13589;AGIWUR35264;AGIWUR37936;AGIWUR39225;AGIWUR40172
CB4856	CT	X	3908144 3911380 WBGene00020669	T22B2.5 T22B2.5 0.0737715109575801
AGIWUR11095;AGIWUR14481;AGIWUR17029;AGIWUR20075;AGIWUR36801;AGIWUR39173;AGIWUR41209;AGIWUR45062
CB4856	CT	X	3914765 3915848 WBGene00020670	T22B2.6 T22B2.6 -0.399192945452835
AGIWUR3107;AGIWUR3588;AGIWUR7204;AGIWUR8957;AGIWUR11412;AGIWUR11915;AGIWUR17333;AGIWUR19664;AGIWUR26137;AGIWUR29664;AGIWUR32235;AGIWUR35489;AGIWUR36229;AGIWUR37239;AGIWUR44863;AGIWUR44904
CB4856	CT	X	3917301 3919835 WBGene00020667	T22B2.3 T22B2.3 -0.00935120159783143	AGIWUR4053
CB4856	CT	X	3921068 3923233 WBGene00020666	T22B2.2 T22B2.2 0.211048243151885	AGIWUR34034
CB4856	CT	X	3926081 3929576 WBGene00020665	T22B2.1 T22B2.1 -0.147213658947178	AGIWUR13633
CB4856	CT	X	3931631 3937897 WBGene00020567	T19D2.1 T19D2.1 -0.105410191356498	AGIWUR8958
CB4856	CT	X	3939401 3940092 WBGene00020568	T19D2.3 T19D2.3 -0.255747348504798	AGIWUR40361
CB4856	CT	X	3940141 3943313 WBGene00004184	T19D2.2 T19D2.2 0.0547437531750354

AGIWUR288;AGIWUR6348;AGIWUR7010;AGIWUR18174;AGIWUR20877;AGIWUR21653;AGIWUR33189;AGIWUR36185
CB4856	CT	X	3958497 3961907 WBGene00003007	lin-18	C16B8.1 -0.205981906550001
AGIWUR3203;AGIWUR25846;AGIWUR29365
CB4856	CT	X	3961930 3966432 WBGene00015822	C16B8.2 C16B8.2 0.391803662875883
AGIWUR6092;AGIWUR11035;AGIWUR35161
CB4856	CT	X	3968036 3968637 WBGene00015823	C16B8.3 C16B8.3 -0.127343573506846
AGIWUR4953;AGIWUR18034;AGIWUR24168;AGIWUR35144
CB4856	CT	X	3970389 3973634 WBGene00015824	C16B8.4 C16B8.4 0.135916210676847	AGIWUR20367
CB4856	CT	X	3985104 3985465 WBGene00022926	C16B8.5 C16B8.5 0.0459262479006881
AGIWUR6001;AGIWUR7797;AGIWUR14016;AGIWUR21798;AGIWUR33763
CB4856	CT	X	3997340 3999341 WBGene00019829	R02E12.5	R02E12.5	-0.243143750950578
AGIWUR26862
CB4856	CT	X	4003532 4007634 WBGene00019828	R02E12.4	R02E12.4	-0.0277375375022579
AGIWUR39405
CB4856	CT	X	4008058 4016185 WBGene00019830	hrg-1	R02E12.6	0.459994920080398
AGIWUR22265
CB4856	CT	X	4012219 4021956 WBGene00019827	mop-25.1	R02E12.2	0.11886518347638
AGIWUR35677
CB4856	CT	X	4022273 4025584 WBGene00000049	acr-10	R02E12.8	0.0618468787891349
AGIWUR13780
CB4856	CT	X	4056835 4071493 WBGene00020294	T06F4.1 T06F4.1 0.0299954525516144
AGIWUR12454;AGIWUR40322
CB4856	CT	X	4078236 4081045 WBGene00044566	T06F4.3 T06F4.3 0.15229218052763
AGIWUR9614;AGIWUR19207;AGIWUR22647;AGIWUR33199;AGIWUR34873
CB4856	CT	X	4094867 4100573 WBGene00000531	clh-4	T06F4.2 0.231770933919437
AGIWUR7616;AGIWUR14496;AGIWUR23097;AGIWUR26114
CB4856	CT	X	4101687 4102125 WBGene00044724	R02E4.3 R02E4.3 -0.210081714669593
AGIWUR4745;AGIWUR30953;AGIWUR38166
CB4856	CT	X	4103447 4104521 WBGene00019826	R02E4.1 R02E4.1 0.00181829182226156
AGIWUR13674;AGIWUR27146
CB4856	CT	X	4107476 4110496 WBGene00044021	R02E4.2 R02E4.2 0.17562420400122
AGIWUR16898;AGIWUR41313
CB4856	CT	X	4110442 4111474 WBGene00017307	F09F9.2 F09F9.2 0.430305673091343	AGIWUR15733
CB4856	CT	X	4115330 4117027 WBGene00017306	F09F9.1 F09F9.1 -0.0472039195259164	AGIWUR2150
CB4856	CT	X	4117951 4119487 WBGene00045395	F09F9.5 F09F9.5 -0.0275232853586762
AGIWUR15072;AGIWUR17778
CB4856	CT	X	4119652 4121016 WBGene00017308	F09F9.3 F09F9.3 0.131285194089499	AGIWUR7987
CB4856	CT	X	4121786 4128431 WBGene00017309	F09F9.4 F09F9.4 0.464156195142401	AGIWUR3191
CB4856	CT	X	4135133 4147662 WBGene00022745	ZK470.2 ZK470.2 -0.283055528373485

AGIWUR4037;AGIWUR17753;AGIWUR18130
CB4856 CT X 4148648 4153443 WBGene00006410 nck-1 ZK470.5 0.00955875585496635
AGIWUR19892;AGIWUR25883;AGIWUR27962;AGIWUR30810;AGIWUR33783
CB4856 CT X 4167459 4169791 WBGene00219362 ZK470.14 ZK470.14 0.201594491147282
AGIWUR25015
CB4856 CT X 4176065 4177494 WBGene00022747 ZK470.6 ZK470.6 -0.361805512277671
AGIWUR244;AGIWUR9011;AGIWUR18872;AGIWUR21207
CB4856 CT X 4177917 4186756 WBGene00003412 mrp-6 F20B6.3 -0.0644813986945811
AGIWUR11769;AGIWUR13096;AGIWUR28457;AGIWUR31143;AGIWUR32298;AGIWUR35622;AGIWUR38453;AGIWUR40403
CB4856 CT X 4191094 4193144 WBGene00006921 vha-12 F20B6.2 -0.381472265289432
AGIWUR6392;AGIWUR21906;AGIWUR38099
CB4856 CT X 4194019 4194785 WBGene00017628 F20B6.4 F20B6.4 0.288706979383312 AGIWUR38287
CB4856 CT X 4201650 4202214 WBGene00017630 F20B6.6 F20B6.6 -0.0779577753790992 AGIWUR43841
CB4856 CT X 4204602 4206444 WBGene00017632 F20B6.9 F20B6.9 -0.198338532577647
AGIWUR5286;AGIWUR38141
CB4856 CT X 4210573 4217290 WBGene00001994 hpk-1 F20B6.8 0.481306564903817
AGIWUR1171;AGIWUR11680
CB4856 CT X 4218279 4218458 WBGene00020922 W01H2.2 W01H2.2 -0.171516299489641 AGIWUR21224
CB4856 CT X 4227857 4235846 WBGene00004285 rab-37 W01H2.3 0.150610492902018
AGIWUR7026;AGIWUR19855;AGIWUR22772;AGIWUR29998;AGIWUR35671;AGIWUR42634
CB4856 CT X 4246554 4247212 WBGene00018602 F48D6.4 F48D6.4 1.02979551944162
AGIWUR4659;AGIWUR14142;AGIWUR26030;AGIWUR44362
CB4856 CT X 4249249 4250764 WBGene00001957 hlh-13 F48D6.3 0.415203830047816
AGIWUR12271;AGIWUR31055
CB4856 CT X 4252356 4254174 WBGene00005716 srv-5 F48D6.2 -0.00762296092507775 AGIWUR32042
CB4856 CT X 4255154 4256812 WBGene00006393 taf-11.1 F48D6.1 0.204010574273148
AGIWUR12189
CB4856 CT X 4257061 4259094 WBGene00005717 srv-6 C52B9.5 0.0663112668448811 AGIWUR38894
CB4856 CT X 4260652 4264591 WBGene00016867 C52B9.4 C52B9.4 -0.200077296249557 AGIWUR2170
CB4856 CT X 4265851 4270486 WBGene00016866 coel-1 C52B9.3 1.07651448683547
AGIWUR6040;AGIWUR13947;AGIWUR22554;AGIWUR33368;AGIWUR36165;AGIWUR37755;AGIWUR40505
CB4856 CT X 4271169 4274229 WBGene00003179 mec-18 C52B9.9 -0.0941621109388944 AGIWUR1282
CB4856 CT X 4273914 4274750 WBGene00016869 C52B9.10 C52B9.10 0.111535434221833
AGIWUR5387
CB4856 CT X 4274786 4279344 WBGene00006680 twk-28 C52B9.6 -0.180913091922287
AGIWUR8740;AGIWUR10284;AGIWUR44110
CB4856 CT X 4280980 4284176 WBGene00016865 ets-9 C52B9.2 0.0697409679573191 AGIWUR41782
CB4856 CT X 4284189 4284901 WBGene00016870 ets-10 C52B9.11 0.155820506263623
AGIWUR19859

CB4856	CT	X	4286737	4291494	WBGene00000510	cka-2	C52B9.1	0.0261981647911949	AGIWUR4895;AGIWUR24931;AGIWUR37447;AGIWUR37628;AGIWUR39879;AGIWUR42350
CB4856	CT	X	4291475	4297630	WBGene00003907	paf-2	C52B9.7	-0.0488597768449366	AGIWUR1054;AGIWUR26277
CB4856	CT	X	4297860	4304398	WBGene00016868	C52B9.8	C52B9.8	0.0708166759068938	AGIWUR22938
CB4856	CT	X	4328400	4328916	WBGene00016039	C24A8.5	C24A8.5	0.012830086327245	AGIWUR16655
CB4856	CT	X	4339634	4343702	WBGene00017472	cst-1	F14H12.4	-0.0314637166571998	AGIWUR38627
CB4856	CT	X	4344215	4345644	WBGene00017471	F14H12.3	F14H12.3	-0.0941232315376723	AGIWUR39369
CB4856	CT	X	4350498	4352744	WBGene00017470	F14H12.2	F14H12.2	-0.27658626111322	AGIWUR5219
CB4856	CT	X	4353618	4354820	WBGene00000738	col-165	F14H12.1	0.71355781557519	AGIWUR4381;AGIWUR38491
CB4856	CT	X	4357015	4357567	WBGene00017473	F14H12.6	F14H12.6	-0.343655995905635	AGIWUR27312
CB4856	CT	X	4361927	4362283	WBGene00017474	F14H12.7	F14H12.7	0.550306375227164	AGIWUR32261
CB4856	CT	X	4362936	4363315	WBGene00219368	F14H12.17	F14H12.17	0.364132986280229	AGIWUR40009
CB4856	CT	X	4365305	4366332	WBGene00020120	R160.3	R160.3	-0.635349619257841	AGIWUR21678
CB4856	CT	X	4367159	4367514	WBGene00219369	R160.10	R160.10	2.20326720110247	AGIWUR35788
CB4856	CT	X	4370014	4370856	WBGene00020122	R160.5	R160.5	0.17335605286727	AGIWUR13004
CB4856	CT	X	4371036	4373211	WBGene00020123	R160.6	R160.6	0.0763976443964349	AGIWUR26469
CB4856	CT	X	4376595	4376850	WBGene00020119	R160.2	R160.2	0.243219339785602	AGIWUR27023
CB4856	CT	X	4378004	4383366	WBGene00003084	lst-2	R160.7	-0.103625711631343	AGIWUR785
CB4856	CT	X	4389365	4393796	WBGene00001082	dpy-23	R160.1	-0.182435585102102	AGIWUR4657;AGIWUR6146;AGIWUR37164
CB4856	CT	X	4403159	4407145	WBGene00017040	D1079.1	D1079.1	0.378842328531992	AGIWUR29911
CB4856	CT	X	4408089	4409133	WBGene00017477	F15A8.4	F15A8.4	0.0365977636001471	AGIWUR21412
CB4856	CT	X	4411359	4416079	WBGene00001052	dop-1	F15A8.5	0.0564194133334546	AGIWUR5927;AGIWUR12192;AGIWUR20100;AGIWUR22632;AGIWUR30934
CB4856	CT	X	4430929	4437512	WBGene00017478	F15A8.6	F15A8.6	0.156404183937884	AGIWUR33964
CB4856	CT	X	4440941	4441726	WBGene00017476	F15A8.1	F15A8.1	-0.0161790077854729	AGIWUR39320
CB4856	CT	X	4442152	4448383	WBGene00017794	mltn-13	F25F6.1	-0.0392152725811288	AGIWUR20642;AGIWUR27594
CB4856	CT	X	4454120	4457304	WBGene00017177	F02E8.4	F02E8.4	-0.241887678510018	AGIWUR29684
CB4856	CT	X	4458272	4459264	WBGene00000157	aps-2	F02E8.3	-0.123675602625661	AGIWUR18349;AGIWUR23286;AGIWUR24757;AGIWUR31667

CB4856	CT	X	4459603 4462609 WBGene00017178	atg-16.1	F02E8.5 0.113884518433576
AGIWUR40988
CB4856	CT	X	4466518 4469005 WBGene00017176	nmur-3	F02E8.2 0.154248325709704	AGIWUR11312
CB4856	CT	X	4469743 4471476 WBGene00000207	asb-2	F02E8.1 -0.0267904945510633
AGIWUR7405;AGIWUR8491;AGIWUR21740;AGIWUR34730;AGIWUR38595;AGIWUR42226
CB4856	CT	X	4472244 4488851 WBGene00003561	ncr-1	F02E8.6 0.548074464103534	AGIWUR16168
CB4856	CT	X	4491651 4492208 WBGene00023460	F46H6.5 F46H6.5 0.595035654339689	AGIWUR43882
CB4856	CT	X	4494914 4504388 WBGene00000959	dgk-2	F46H6.2 0.37693726969707
AGIWUR8189;AGIWUR8947;AGIWUR12916;AGIWUR22238;AGIWUR23464;AGIWUR25341;AGIWUR27280;AGIWUR28757;AGIWUR30229;AGIWUR42723
CB4856	CT	X	4508509 4510960 WBGene00004356	rhi-1	F46H6.1 -0.0110254713298366
AGIWUR3599;AGIWUR16396;AGIWUR24273;AGIWUR27364
CB4856	CT	X	4514099 4516894 WBGene00005008	spr-3	C07A12.5	-0.196401390302927
AGIWUR19165;AGIWUR42543
CB4856	CT	X	4517748 4521069 WBGene00015561	C07A12.7	C07A12.7
-0.00923728146778835	AGIWUR15126;AGIWUR25240
CB4856	CT	X	4524155 4526536 WBGene00003963	pdi-2	C07A12.4	-0.187546211909658
AGIWUR3408;AGIWUR14954;AGIWUR15713;AGIWUR20132;AGIWUR21475;AGIWUR25287;AGIWUR28983;AGIWUR29415;AGIWUR43162;AGIWUR43873
CB4856	CT	X	4532430 4537545 WBGene00003628	nhr-35	C07A12.3	0.857354955402156
AGIWUR12593
CB4856	CT	X	4544876 4545418 WBGene00015560	C07A12.2	C07A12.2	-0.0312099363590779
AGIWUR39936
CB4856	CT	X	4562163 4562880 WBGene00015495	C05E11.6	C05E11.6	0.558270328890833
AGIWUR10070
CB4856	CT	X	4565302 4569393 WBGene00000136	amt-4	C05E11.5	-0.55723818458662
AGIWUR20267
CB4856	CT	X	4571448 4573914 WBGene00000133	amt-1	C05E11.4	1.08427391873773
AGIWUR2915
CB4856	CT	X	4578249 4581724 WBGene00015494	C05E11.3	C05E11.3	0.187753745550847
AGIWUR36785
CB4856	CT	X	4581586 4585679 WBGene00015496	C05E11.7	C05E11.7	0.208858634173777
AGIWUR21270
CB4856	CT	X	4586917 4587993 WBGene00015493	C05E11.2	C05E11.2	-0.018539148258955
AGIWUR32772
CB4856	CT	X	4588768 4590446 WBGene00015492	lnp-1	C05E11.1	-0.0296257218951972
AGIWUR41193
CB4856	CT	X	4591281 4592404 WBGene00001455	flp-12	C05E11.8	0.292518656548438
AGIWUR4207;AGIWUR7649;AGIWUR11808;AGIWUR12376;AGIWUR16211;AGIWUR34929;AGIWUR35398;AGIWUR38482;AGIWUR42059;A

GIWUR42237;AGIWUR43382
CB4856 CT X 4596016 4605928 WBGene00020496 spat-3 T13H2.5 0.038325678439306
AGIWUR24159;AGIWUR33442
CB4856 CT X 4610926 4620423 WBGene00004148 pqn-65 T13H2.4 0.0432553437825973
AGIWUR2057;AGIWUR7236;AGIWUR8289;AGIWUR17805;AGIWUR21667;AGIWUR36009;AGIWUR41841
CB4856 CT X 4629039 4631838 WBGene00016706 C46C11.3 C46C11.3
-0.00521736049567068 AGIWUR15591
CB4856 CT X 4632348 4635784 WBGene00016705 C46C11.2 C46C11.2 -0.406908061076269
AGIWUR36196
CB4856 CT X 4637242 4643695 WBGene00016704 hosl-1 C46C11.1 0.0157527908459425
AGIWUR26837
CB4856 CT X 4644404 4647192 WBGene00044567 C46C11.4 C46C11.4 0.815413686484304
AGIWUR25422;AGIWUR33961
CB4856 CT X 4647526 4649197 WBGene00017532 ent-5 F16H11.3 -0.11571754915224
AGIWUR39877
CB4856 CT X 4653434 4654157 WBGene00000428 ceh-1 F16H11.4 -0.0428145876205308
AGIWUR10788;AGIWUR22423;AGIWUR26594
CB4856 CT X 4656433 4660318 WBGene00003635 nhr-45 F16H11.5 0.0046691215830279
AGIWUR1648
CB4856 CT X 4668071 4673234 WBGene00017530 F16H11.1 F16H11.1 -0.124549970588825
AGIWUR30876
CB4856 CT X 4673706 4677283 WBGene00019345 tmem-135 K02G10.3 -0.257506165776482
AGIWUR24352
CB4856 CT X 4677342 4678129 WBGene00001454 flp-11 K02G10.4 0.330758746257978
AGIWUR387;AGIWUR13935;AGIWUR23795;AGIWUR25362;AGIWUR33739;AGIWUR34739;AGIWUR35075;AGIWUR44123
CB4856 CT X 4687763 4691316 WBGene00019346 K02G10.5 K02G10.5 -0.241975323913386
AGIWUR44406
CB4856 CT X 4696952 4699461 WBGene00002044 hyl-2 K02G10.6 -0.32367087659258
AGIWUR24282
CB4856 CT X 4702965 4704970 WBGene00000176 aqp-8 K02G10.7 0.193727912712108
AGIWUR14200
CB4856 CT X 4707188 4709812 WBGene00019344 dhhc-10 K02G10.1 0.0377458408645364
AGIWUR41098
CB4856 CT X 4709772 4711744 WBGene00001032 dnj-14 K02G10.8 0.100235717194284
AGIWUR126;AGIWUR12952;AGIWUR18427;AGIWUR29641;AGIWUR38758;AGIWUR40737
CB4856 CT X 4712279 4715080 WBGene00018878 glit-1 F55D10.3 0.0331817762910962
AGIWUR8019
CB4856 CT X 4715178 4715801 WBGene00004438 rpl-25.1 F55D10.2 -0.154940879931084
AGIWUR3501;AGIWUR18829

CB4856 CT X 4716244 4720640 WBGene00018879 F55D10.4 F55D10.4 -0.0566340847977695 AGIWUR8758
CB4856 CT X 4722877 4727481 WBGene00018880 acc-3 F55D10.5 0.00489159293458918 AGIWUR17945
CB4856 CT X 4732964 4738592 WBGene00018877 aman-1 F55D10.1 -0.0948135262265097 AGIWUR33927
CB4856 CT X 4738964 4749083 WBGene00003056 lon-2 C39E6.1 -0.350614943589601 AGIWUR33141
CB4856 CT X 4754678 4755160 WBGene00016537 C39E6.2 C39E6.2 0.170261185788343 AGIWUR13911
CB4856 CT X 4758804 4761612 WBGene00003377 mls-2 C39E6.4 0.74567611789076 AGIWUR29708
CB4856 CT X 4767178 4769985 WBGene00003807 npr-1 C39E6.6 -0.345033665970596 AGIWUR43511
CB4856 CT X 4777512 4782297 WBGene00018377 hum-10 F43C9.3 0.17735539731668 AGIWUR36723;AGIWUR38269
CB4856 CT X 4786118 4790323 WBGene00018376 F43C9.2 F43C9.2 0.50291993210598 AGIWUR33921
CB4856 CT X 4793266 4796158 WBGene00018375 F43C9.1 F43C9.1 0.0791101120903903 AGIWUR29075
CB4856 CT X 4796095 4800128 WBGene00003245 mig-13 F43C9.4 0.28310340608966 AGIWUR2697;AGIWUR29599;AGIWUR30719
CB4856 CT X 4805998 4811536 WBGene00019960 ztf-16 R08E3.4 0.166650104154594 AGIWUR272;AGIWUR8112;AGIWUR11016;AGIWUR21775;AGIWUR22153;AGIWUR28337;AGIWUR41656;AGIWUR44561
CB4856 CT X 4818599 4821708 WBGene00019959 R08E3.3 R08E3.3 -0.125861722621597 AGIWUR8990;AGIWUR29698;AGIWUR44869
CB4856 CT X 4822661 4824680 WBGene00019958 R08E3.2 R08E3.2 -0.138916800193958 AGIWUR35969
CB4856 CT X 4825003 4834905 WBGene00019957 R08E3.1 R08E3.1 -0.0702198077504423 AGIWUR1704;AGIWUR8945;AGIWUR10834;AGIWUR11213;AGIWUR17358;AGIWUR38709
CB4856 CT X 4834881 4853065 WBGene00018193 F39C12.1 F39C12.1 -0.037977661503465 AGIWUR28106
CB4856 CT X 4854069 4860036 WBGene00000072 add-1 F39C12.2 -0.0282194311581883 AGIWUR6120;AGIWUR26945;AGIWUR30618;AGIWUR36537;AGIWUR43003
CB4856 CT X 4866075 4866690 WBGene00044568 ntc-1 F39C12.4 0.236891584973931 AGIWUR14092;AGIWUR17933;AGIWUR25041;AGIWUR26292;AGIWUR29232
CB4856 CT X 4866684 4874657 WBGene00006640 tsp-14 F39C12.3 0.117690664612558 AGIWUR7113;AGIWUR18083;AGIWUR23404;AGIWUR25913;AGIWUR36849;AGIWUR40724;AGIWUR42162
CB4856 CT X 4885951 4889575 WBGene00020323 arrd-23 T07F12.3 -0.296681467262825 AGIWUR14501
CB4856 CT X 4890221 4892328 WBGene00020322 T07F12.2 T07F12.2 0.0589116733279083 AGIWUR12321;AGIWUR14348;AGIWUR33947;AGIWUR34000;AGIWUR34056;AGIWUR35997;AGIWUR40084
CB4856 CT X 4894055 4896615 WBGene00020321 T07F12.1 T07F12.1 -0.428964536898572 AGIWUR38934
CB4856 CT X 4896602 4898163 WBGene00020324 T07F12.4 T07F12.4 0.0346221929925221 AGIWUR3036

CB4856	CT	X	4907290	4912323	WBGene00019403	K05B2.2	K05B2.2	-0.0224838970599741	AGIWUR22809
CB4856	CT	X	4912308	4915979	WBGene00002052	ifa-4	K05B2.3	-0.00516281053065626	AGIWUR8151
CB4856	CT	X	4919019	4920988	WBGene00019404	K05B2.4	K05B2.4	-0.185845364820274	AGIWUR3149
CB4856	CT	X	4921252	4929757	WBGene00003986	mct-4	K05B2.5	-0.448631306432743	AGIWUR1296;AGIWUR2543;AGIWUR5527;AGIWUR10729;AGIWUR18533;AGIWUR21418;AGIWUR25450;AGIWUR28400;AGIWUR39815
CB4856	CT	X	4948049	4955331	WBGene00006475	tyra-3	M03F4.3	0.166036939112182	AGIWUR44037
CB4856	CT	X	4957329	4958067	WBGene00019758	M03F4.4	M03F4.4	0.21707711787759	AGIWUR32869
CB4856	CT	X	4960553	4964416	WBGene00000066	act-4	M03F4.2	0.00599312253520878	AGIWUR24973;AGIWUR26902
CB4856	CT	X	4964413	4969459	WBGene00019759	M03F4.6	M03F4.6	0.14708737374621	AGIWUR37710
CB4856	CT	X	4969537	4972281	WBGene00019760	calu-1	M03F4.7	-0.373048746305273	AGIWUR8128;AGIWUR11525;AGIWUR13302;AGIWUR24024;AGIWUR25793;AGIWUR31221;AGIWUR40414
CB4856	CT	X	4972575	4997598	WBGene00022501	ZC8.6	ZC8.6	-0.200538091210554	AGIWUR20769;AGIWUR22221;AGIWUR44515
CB4856	CT	X	4999270	5003515	WBGene00022499	set-30	ZC8.3	0.421228889331571	AGIWUR11661
CB4856	CT	X	5005269	5007024	WBGene00022498	hsd-2	ZC8.1	-0.247105162354654	AGIWUR15242
CB4856	CT	X	5009300	5011222	WBGene00022616	hsd-3	ZC449.6	-0.18004097936049	AGIWUR21264;AGIWUR39014
CB4856	CT	X	5014040	5015452	WBGene00045365	ZC449.7	ZC449.7	-0.218939056218127	AGIWUR2844;AGIWUR39162
CB4856	CT	X	5017924	5021064	WBGene00022613	sek-3	ZC449.3	-0.184662393297701	AGIWUR32402
CB4856	CT	X	5021215	5024258	WBGene00022612	ZC449.2	ZC449.2	0.868781128815169	AGIWUR16052
CB4856	CT	X	5024420	5028165	WBGene00022611	ZC449.1	ZC449.1	0.465445446441346	AGIWUR17967;AGIWUR31767
CB4856	CT	X	5028184	5029568	WBGene00022614	ZC449.4	ZC449.4	0.172960731527798	AGIWUR20026
CB4856	CT	X	5035030	5037773	WBGene00022615	ZC449.5	ZC449.5	-0.353338355341945	AGIWUR9356;AGIWUR13636;AGIWUR22963;AGIWUR25078;AGIWUR32327;AGIWUR36618
CB4856	CT	X	5043976	5044746	WBGene00022024	Y64H9A.2	Y64H9A.2	-0.410862424422803	AGIWUR11654
CB4856	CT	X	5057273	5057581	WBGene00022023	Y64H9A.1	Y64H9A.1	-0.295521297745735	AGIWUR12543
CB4856	CT	X	5058367	5058733	WBGene00016070	C24H10.4	C24H10.4	-0.37269787161257	AGIWUR27501
CB4856	CT	X	5061056	5062209	WBGene00016069	C24H10.3	C24H10.3	0.511892766018535	AGIWUR2962;AGIWUR14100
CB4856	CT	X	5064602	5066663	WBGene00016068	C24H10.2	C24H10.2	-0.162971302075709	AGIWUR9762
CB4856	CT	X	5072019	5074425	WBGene00016067	C24H10.1	C24H10.1	-0.133622739115541	AGIWUR25124

CB4856 CT X 5074399 5078120 WBGene00006861 cal-5 C24H10.5 -0.0642795322734742
AGIWUR30530
CB4856 CT X 5079773 5084489 WBGene00016600 ets-5 C42D8.4 0.304969832114931 AGIWUR35943
CB4856 CT X 5087840 5092538 WBGene00006862 pnk-4 C42D8.3 -0.0247655604926464
AGIWUR20818;AGIWUR40779;AGIWUR44864
CB4856 CT X 5092458 5097067 WBGene00000039 acn-1 C42D8.5 0.3168794179551 AGIWUR38360
CB4856 CT X 5101032 5106173 WBGene00006926 vit-2 C42D8.2 -0.0279695118135415
AGIWUR7138;AGIWUR13310;AGIWUR30312
CB4856 CT X 5106260 5107039 WBGene00044299 C42D8.9 C42D8.9 -0.162531046687972
AGIWUR486;AGIWUR17383;AGIWUR42735
CB4856 CT X 5109504 5112452 WBGene00016599 C42D8.1 C42D8.1 -0.0188843823061511 AGIWUR34679
CB4856 CT X 5112422 5116866 WBGene00000149 apl-1 C42D8.8 0.175002675143906
AGIWUR8657;AGIWUR10776;AGIWUR13543;AGIWUR28682;AGIWUR32921;AGIWUR35954;AGIWUR38873;AGIWUR41528
CB4856 CT X 5138991 5146742 WBGene00016292 tbc-7 C31H2.1 -0.0446603870306603 AGIWUR23489
CB4856 CT X 5148804 5150476 WBGene00001070 dpy-8 C31H2.2 -0.505760202482382 AGIWUR25466
CB4856 CT X 5151547 5154399 WBGene00016293 C31H2.3 C31H2.3 0.362443028503299 AGIWUR40194
CB4856 CT X 5155093 5159667 WBGene00016294 C31H2.4 C31H2.4 0.123826019058331 AGIWUR39874
CB4856 CT X 5163055 5163439 WBGene00017150 EGAP5.1 EGAP5.1 0.1930938936852 AGIWUR14391
CB4856 CT X 5165395 5168778 WBGene00020197 mans-3 T03G11.4 0.17419913300278
AGIWUR10489
CB4856 CT X 5168739 5171119 WBGene00044732 T03G11.10 T03G11.10 0.15802460890083
AGIWUR14189;AGIWUR18431
CB4856 CT X 5174369 5177506 WBGene00020196 T03G11.3 T03G11.3 0.437850114504085
AGIWUR35567
CB4856 CT X 5177519 5179210 WBGene00020199 T03G11.6 T03G11.6 -0.0254856618819357
AGIWUR2097;AGIWUR6235;AGIWUR26975
CB4856 CT X 5180174 5181495 WBGene00005711 sru-48 T03G11.2 -0.22606179744653
AGIWUR37034
CB4856 CT X 5183828 5186623 WBGene00044584 T03G11.9 T03G11.9 -0.0884625698878079
AGIWUR7733;AGIWUR10680
CB4856 CT X 5186921 5189077 WBGene00006983 zig-6 T03G11.8 0.162558276946339
AGIWUR17299;AGIWUR28407;AGIWUR44746
CB4856 CT X 5196110 5204250 WBGene00004145 pqn-62 T03G11.1 0.0133815399952897
AGIWUR4007;AGIWUR5125;AGIWUR30497;AGIWUR31491
CB4856 CT X 5205760 5216230 WBGene00019250 H28G03.2 H28G03.2 -0.0389727592288098
AGIWUR817;AGIWUR31145;AGIWUR37381;AGIWUR43675
CB4856 CT X 5216363 5219095 WBGene00019249 H28G03.1 H28G03.1 -0.0258124021074515
AGIWUR804;AGIWUR5930;AGIWUR9399;AGIWUR20705;AGIWUR21613;AGIWUR37275;AGIWUR44938
CB4856 CT X 5220406 5222042 WBGene00019251 H28G03.3 H28G03.3 0.123736504918444

AGIWUR32465
CB4856 CT X 5234669 5243408 WBGene00003477 mtm-5 H28G03.6 -0.0561694466660381
AGIWUR17321
CB4856 CT X 5244317 5246927 WBGene00021323 Y34B4A.7 Y34B4A.7 -0.0531247425384507
AGIWUR2665;AGIWUR15980;AGIWUR25310
CB4856 CT X 5249851 5252787 WBGene00021326 Y34B4A.10 Y34B4A.10 -0.340667637361057
AGIWUR18285
CB4856 CT X 5253001 5255068 WBGene00021322 Y34B4A.6 Y34B4A.6 -0.394353530612605
AGIWUR10605
CB4856 CT X 5255194 5256855 WBGene00021325 Y34B4A.9 Y34B4A.9 -0.286235583989471
AGIWUR19937;AGIWUR39655
CB4856 CT X 5257213 5258384 WBGene00021321 Y34B4A.5 Y34B4A.5 -0.147349304370056
AGIWUR44254
CB4856 CT X 5264330 5271799 WBGene00021320 Y34B4A.4 Y34B4A.4 -0.0503744423525303
AGIWUR2026;AGIWUR35951
CB4856 CT X 5271780 5275287 WBGene00021324 Y34B4A.8 Y34B4A.8 0.12788344389965
AGIWUR30828
CB4856 CT X 5275567 5278813 WBGene00002031 hst-6 Y34B4A.3 -0.205620518255767
AGIWUR663
CB4856 CT X 5279497 5283719 WBGene00021319 Y34B4A.2 Y34B4A.2 -0.115317374079897
AGIWUR406;AGIWUR7277;AGIWUR8792;AGIWUR10761;AGIWUR15875;AGIWUR21692;AGIWUR24499;AGIWUR25055;AGIWUR26841;AGI
WUR29281;AGIWUR29621;AGIWUR37635;AGIWUR41542
CB4856 CT X 5283831 5289726 WBGene00017974 vps-41 F32A6.3 -0.0276682380468056
AGIWUR24978;AGIWUR33755
CB4856 CT X 5289728 5303512 WBGene00000092 ags-3 F32A6.4 -0.000964841795870086
AGIWUR2762;AGIWUR6181;AGIWUR6713;AGIWUR8007;AGIWUR19964;AGIWUR23829;AGIWUR32302
CB4856 CT X 5298561 5306328 WBGene00017973 ift-81 F32A6.2 0.10047521860794
AGIWUR4111;AGIWUR6031;AGIWUR6950;AGIWUR10167;AGIWUR11518;AGIWUR17046;AGIWUR17375;AGIWUR22547;AGIWUR23483;AG
IWUR35816
CB4856 CT X 5307489 5323385 WBGene00006064 sto-2 F32A6.5 0.0765220066660994 AGIWUR30928
CB4856 CT X 5329455 5332339 WBGene00016134 C26B9.5 C26B9.5 -0.0627267871751336 AGIWUR13099
CB4856 CT X 5332969 5335468 WBGene00016135 C26B9.6 C26B9.6 0.433988873196292 AGIWUR9069
CB4856 CT X 5338273 5340182 WBGene00016133 C26B9.3 C26B9.3 0.681176059635272 AGIWUR41485
CB4856 CT X 5340189 5342202 WBGene00016132 C26B9.2 C26B9.2 -0.174853630045963 AGIWUR14514
CB4856 CT X 5342307 5342841 WBGene00016136 C26B9.7 C26B9.7 0.0835461637571111
AGIWUR25278;AGIWUR29045;AGIWUR31795;AGIWUR32458;AGIWUR37242;AGIWUR43461
CB4856 CT X 5344520 5348411 WBGene00016131 C26B9.1 C26B9.1 -0.0253320164198652 AGIWUR34413
CB4856 CT X 5349098 5352423 WBGene00006371 syx-3 F35C8.4 -0.0298609679808504 AGIWUR7704
CB4856 CT X 5353033 5354144 WBGene00003990 pfn-2 F35C8.6 0.247173758275897

									AGIWUR7182;AGIWUR10564
CB4856	CT	X	5355638	5364322	WBGene00018037	chtl-1	F35C8.7	-0.261121735891537	AGIWUR15722
CB4856	CT	X	5365808	5367619	WBGene00018036	F35C8.5	F35C8.5	0.323624116324387	AGIWUR38857
CB4856	CT	X	5368468	5372059	WBGene00002177	jkk-1	F35C8.3	0.0138703051401938	AGIWUR13137
CB4856	CT	X	5372149	5374443	WBGene00018038	F35C8.8	F35C8.8	-0.228598189595154	AGIWUR41387
CB4856	CT	X	5375070	5376911	WBGene00018035	sek-4	F35C8.2	0.418498904323922	AGIWUR22777
CB4856	CT	X	5377607	5379353	WBGene00018034	sek-5	F35C8.1	0.170077280101922	AGIWUR3165
CB4856	CT	X	5386248	5394330	WBGene00015387	kcnl-3	C03F11.1	-0.240679280692179	AGIWUR24146
CB4856	CT	X	5394508	5397400	WBGene00015388	C03F11.2	C03F11.2	0.191157503067974	AGIWUR26439
CB4856	CT	X	5397676	5400626	WBGene00015389	scav-1	C03F11.3	-0.0852184627377994	AGIWUR28074
CB4856	CT	X	5401437	5406443	WBGene00015390	C03F11.4	C03F11.4	-0.347966637582253	AGIWUR36310
CB4856	CT	X	5406562	5414350	WBGene00016559	C41A3.2	C41A3.2	-0.0985611985505552	AGIWUR4001;AGIWUR38812
CB4856	CT	X	5413810	5441958	WBGene00016558	pks-1	C41A3.1	-0.388247072435707	AGIWUR7593;AGIWUR7992
CB4856	CT	X	5445163	5448085	WBGene00016106	C25F6.7	C25F6.7	0.380043688332028	AGIWUR7887;AGIWUR15484;AGIWUR15991;AGIWUR27938;AGIWUR33932
CB4856	CT	X	5448241	5450010	WBGene00044569	C25F6.8	C25F6.8	-0.0577484075793766	AGIWUR13339;AGIWUR22448;AGIWUR37292
CB4856	CT	X	5450262	5450823	WBGene00016105	C25F6.6	C25F6.6	0.367257267338132	AGIWUR38872
CB4856	CT	X	5461256	5466310	WBGene00016104	ddr-1	C25F6.4	0.246511090915416	AGIWUR44016
CB4856	CT	X	5467870	5472684	WBGene00016103	dpyd-1	C25F6.3	-0.253524937086242	AGIWUR8451
CB4856	CT	X	5479700	5484941	WBGene00001006	dlg-1	C25F6.2	0.534287857245417	AGIWUR6452;AGIWUR6973;AGIWUR37028
CB4856	CT	X	5486487	5488102	WBGene00016102	C25F6.1	C25F6.1	-0.163882212256867	AGIWUR28493
CB4856	CT	X	5488777	5489111	WBGene00018876	F55D1.2	F55D1.2	0.185058750064535	AGIWUR25880
CB4856	CT	X	5489838	5490113	WBGene00018875	F55D1.1	F55D1.1	0.130027154090667	AGIWUR2924
CB4856	CT	X	5492915	5496000	WBGene00044623	bus-8	T23F2.1	0.410829580704512	AGIWUR8554
CB4856	CT	X	5496122	5505248	WBGene00020735	T23F2.2	T23F2.2	-0.0539125666072593	AGIWUR3692;AGIWUR12559;AGIWUR23951
CB4856	CT	X	5514798	5515080	WBGene00020737	T23F2.4	T23F2.4	0.729501568960821	AGIWUR42924
CB4856	CT	X	5516454	5517162	WBGene00020738	T23F2.5	T23F2.5	-0.283810702903082	AGIWUR17349
CB4856	CT	X	5519876	5522819	WBGene00016934	mboa-3	C54G7.2	0.24316942554913	AGIWUR4306
CB4856	CT	X	5525199	5538514	WBGene00002983	lgx-1	C54G7.3	0.00668703970347995	AGIWUR2468;AGIWUR35142

CB4856 CT X 5543951 5545529 WBGene00016933 C54G7.1 C54G7.1 -0.335030033521766 AGIWUR7203
CB4856 CT X 5545684 5552756 WBGene00016935 ifta-1 C54G7.4 0.124367055101391 AGIWUR32065
CB4856 CT X 5559021 5561330 WBGene00016518 C38C5.1 C38C5.1 -0.0154228905170104 AGIWUR21309
CB4856 CT X 5575932 5590815 WBGene00003166 mec-2 F14D12.4 -0.0170235111810713
AGIWUR5405;AGIWUR9147;AGIWUR16683;AGIWUR18760;AGIWUR25075;AGIWUR25491;AGIWUR38095;AGIWUR38202;AGIWUR41977;A
GIWUR43004
CB4856 CT X 5593133 5594965 WBGene00006826 unc-97 F14D12.2 -0.0146773992985048
AGIWUR3367;AGIWUR8261;AGIWUR9120;AGIWUR12267;AGIWUR12558;AGIWUR39269
CB4856 CT X 5595778 5599330 WBGene00017464 sulp-2 F14D12.5 -0.0880262783897892
AGIWUR12101;AGIWUR24371;AGIWUR32052;AGIWUR42406
CB4856 CT X 5603766 5609133 WBGene00017463 F14D12.1 F14D12.1 0.0888971128209164
AGIWUR14060;AGIWUR18971;AGIWUR41801
CB4856 CT X 5609155 5611250 WBGene00006411 octr-1 F14D12.6 -0.0650139957850886
AGIWUR27771;AGIWUR34399;AGIWUR35742
CB4856 CT X 5630356 5632302 WBGene00020658 T21F4.1 T21F4.1 -0.213251690928766 AGIWUR14167
CB4856 CT X 5637887 5638075 WBGene00044570 T22B7.8 T22B7.8 0.0417538954074477
AGIWUR20092;AGIWUR30776;AGIWUR30830;AGIWUR35628;AGIWUR42724
CB4856 CT X 5638582 5641363 WBGene00020673 T22B7.4 T22B7.4 0.138846415263487 AGIWUR17746
CB4856 CT X 5642911 5644940 WBGene00005718 srv-7 T22B7.5 -0.28430413777356 AGIWUR7545
CB4856 CT X 5647378 5650213 WBGene00020672 T22B7.3 T22B7.3 1.17609511848424 AGIWUR1146
CB4856 CT X 5651869 5653925 WBGene00020674 T22B7.7 T22B7.7 -0.958883934083353 AGIWUR13647
CB4856 CT X 5663837 5674060 WBGene00001182 egl-13 T22B7.1 -0.0232830181380499
AGIWUR15053;AGIWUR18785;AGIWUR21583;AGIWUR27020
CB4856 CT X 5675246 5680225 WBGene00020919 set-19 W01C8.3 -0.118688976920577
AGIWUR3495;AGIWUR5274;AGIWUR14343;AGIWUR34668
CB4856 CT X 5680349 5684741 WBGene00020920 set-20 W01C8.4 0.453556463036449 AGIWUR11570
CB4856 CT X 5692399 5694426 WBGene00020918 W01C8.1 W01C8.1 0.772105869458906 AGIWUR23186
CB4856 CT X 5694475 5697308 WBGene00020921 W01C8.5 W01C8.5 0.4468385506534
AGIWUR3723;AGIWUR7945;AGIWUR43807
CB4856 CT X 5705719 5710625 WBGene00000295 cat-1 W01C8.6 -0.207741375147084
AGIWUR4252;AGIWUR19213;AGIWUR23916
CB4856 CT X 5714279 5717601 WBGene00017108 cyp-43A1 E03E2.1 -0.151028019070351
AGIWUR6770;AGIWUR8326;AGIWUR17479;AGIWUR18439;AGIWUR42387;AGIWUR44987
CB4856 CT X 5717538 5720268 WBGene00016568 C41G11.1 C41G11.1 0.0883043217901078
AGIWUR1606;AGIWUR11627;AGIWUR19917;AGIWUR25682;AGIWUR28142;AGIWUR35183
CB4856 CT X 5720438 5728656 WBGene00004349 rgs-6 C41G11.3 0.0324009339899618
AGIWUR25314;AGIWUR29193
CB4856 CT X 5738761 5743307 WBGene00016570 gnrr-4 C41G11.4 -0.0368579084939675
AGIWUR39635

CB4856	CT	X	5760281	5763392	WBGene00016937	tag-294	C54H2.3	-0.14604723217653	AGIWUR6339
CB4856	CT	X	5766482	5770793	WBGene00016938	C54H2.4	C54H2.4	0.132506498787695	AGIWUR15372
CB4856	CT	X	5776251	5778884	WBGene00006368	sym-3	C54H2.1	-0.186334493605621
AGIWUR2331;AGIWUR18041;AGIWUR20651;AGIWUR23478
CB4856	CT	X	5778875	5780758	WBGene00004788	sft-4	C54H2.5	-0.00880425329790135
AGIWUR8477;AGIWUR9575;AGIWUR11346;AGIWUR14701;AGIWUR34782;AGIWUR41146
CB4856	CT	X	5783405	5786406	WBGene00018516	tag-257	F46G11.3	0.315075156720415
AGIWUR13771
CB4856	CT	X	5786493	5787353	WBGene00045269	F46G11.6	F46G11.6	-0.0399256817996143
AGIWUR20936
CB4856	CT	X	5788096	5790345	WBGene00018515	F46G11.2	F46G11.2	-0.282903332537872
AGIWUR4630;AGIWUR41476
CB4856	CT	X	5791703	5797328	WBGene00018514	F46G11.1	F46G11.1	0.237400239547118
AGIWUR14159;AGIWUR16979
CB4856	CT	X	5797313	5798790	WBGene00018517	F46G11.4	F46G11.4	-0.196910402824111
AGIWUR28013
CB4856	CT	X	5804379	5805922	WBGene00017428	F13D11.3	F13D11.3	0.695646398010668
AGIWUR8316;AGIWUR8680;AGIWUR33918;AGIWUR37491;AGIWUR38571
CB4856	CT	X	5822277	5827755	WBGene00001824	hbl-1	F13D11.2	0.549090361472178
AGIWUR1043;AGIWUR8461;AGIWUR11581;AGIWUR15453;AGIWUR20933;AGIWUR24101;AGIWUR31648;AGIWUR31830;AGIWUR33022;A
GIWUR35804;AGIWUR41028
CB4856	CT	X	5829376	5832027	WBGene00017427	acp-5	F13D11.1	0.195015499798696
AGIWUR5445;AGIWUR11396;AGIWUR23581
CB4856	CT	X	5831918	5834858	WBGene00017429	F13D11.4	F13D11.4	0.368815739245171
AGIWUR5892
CB4856	CT	X	5838039	5839778	WBGene00021060	dct-11	W06B11.3	0.0588229926142017
AGIWUR21625
CB4856	CT	X	5841592	5845987	WBGene00004245	puf-9	W06B11.2	-0.0426037965857962
AGIWUR6309;AGIWUR16845;AGIWUR30458
CB4856	CT	X	5849104	5851971	WBGene00021059	atat-2	W06B11.1	0.00556617040308538
AGIWUR1959
CB4856	CT	X	5851954	5854260	WBGene00001984	hog-1	W06B11.4	-0.0344009730533976
AGIWUR9208
CB4856	CT	X	5862708	5873320	WBGene00006424	ctbp-1	F49E10.5	0.258962597034032
AGIWUR5330;AGIWUR41828
CB4856	CT	X	5875898	5881716	WBGene00018641	F49E10.4	F49E10.4	-0.0199886346902876
AGIWUR9852;AGIWUR13472;AGIWUR16268;AGIWUR41839
CB4856	CT	X	5885865	5886823	WBGene00001450	flp-7	F49E10.3	0.0787478544615476
AGIWUR358;AGIWUR2865;AGIWUR4562;AGIWUR35967

CB4856	CT	X	5890380	5894757	WBGene00018640	F49E10.2	F49E10.2	0.342754297257523	AGIWUR32917
CB4856	CT	X	5897342	5902152	WBGene00018639	tbc-16	F49E10.1	-0.109407972821936	AGIWUR22634
CB4856	CT	X	5904627	5905346	WBGene00019160	crf-1	H05L03.3	0.461132539669027	AGIWUR36178
CB4856	CT	X	5919656	5920494	WBGene00021268	capa-1	Y23B4A.2	0.566148502570584	AGIWUR13010
CB4856	CT	X	5931122	5934532	WBGene00001175	egl-6	C46F4.1	-0.122947652399782	AGIWUR10686
CB4856	CT	X	5935780	5937549	WBGene00044571	C46F4.3	C46F4.3	-0.0831498370839574	AGIWUR3474;AGIWUR15765;AGIWUR18513;AGIWUR32363
CB4856	CT	X	5937506	5944510	WBGene00016716	acs-17	C46F4.2	0.980266306788264	AGIWUR6231;AGIWUR17820;AGIWUR20521;AGIWUR20972;AGIWUR22389;AGIWUR29029
CB4856	CT	X	5945821	5948869	WBGene00003254	mig-23	R07E4.4	-0.218749728571902	AGIWUR10959
CB4856	CT	X	5949529	5953174	WBGene00019938	R07E4.5	R07E4.5	-0.108148434821523	AGIWUR41208
CB4856	CT	X	5953450	5953815	WBGene00019937	R07E4.3	R07E4.3	-0.205256458255883	AGIWUR17110
CB4856	CT	X	5959115	5970158	WBGene00019935	R07E4.1	R07E4.1	0.115472468114808	AGIWUR11245;AGIWUR31261
CB4856	CT	X	5970677	5977854	WBGene00002190	kin-2	R07E4.6	0.0758850651824243	AGIWUR8994;AGIWUR10317;AGIWUR10536;AGIWUR14932;AGIWUR15995;AGIWUR34314;AGIWUR35178
CB4856	CT	X	5984975	5992103	WBGene00002224	klp-13	F22F4.3	0.435980118785595	AGIWUR36520
CB4856	CT	X	5992099	5992859	WBGene00017718	F22F4.5	F22F4.5	-0.552412260637194	AGIWUR24185;AGIWUR27325;AGIWUR28641
CB4856	CT	X	5996156	5998243	WBGene00002125	inx-3	F22F4.2	0.321931745125513	AGIWUR11328;AGIWUR26870;AGIWUR41549;AGIWUR42844
CB4856	CT	X	5998310	6001534	WBGene00017717	F22F4.4	F22F4.4	-0.130858284622535	AGIWUR30783
CB4856	CT	X	6004482	6007071	WBGene00017716	F22F4.1	F22F4.1	0.349825179049761	AGIWUR8904
CB4856	CT	X	6006969	6011310	WBGene00015622	C09B8.3	C09B8.3	0.358816304509852	AGIWUR18158
CB4856	CT	X	6015492	6017584	WBGene00015625	C09B8.8	C09B8.8	-0.495972526907135	AGIWUR25377
CB4856	CT	X	6018442	6019936	WBGene00015623	C09B8.4	C09B8.4	-0.840414922223408	AGIWUR28618
CB4856	CT	X	6020866	6022569	WBGene00015624	C09B8.5	C09B8.5	-0.442030534466038	AGIWUR22489
CB4856	CT	X	6031469	6034525	WBGene00002146	ipp-5	C09B8.1	0.0580883703801476	AGIWUR7509;AGIWUR39391;AGIWUR43663
CB4856	CT	X	6034463	6039496	WBGene00002023	hsp-25	C09B8.6	0.128866761973114	AGIWUR3529;AGIWUR6104;AGIWUR20413;AGIWUR30435;AGIWUR39439;AGIWUR42269
CB4856	CT	X	6044309	6048610	WBGene00003911	pak-1	C09B8.7	0.0862269543842453	AGIWUR25778;AGIWUR39887
CB4856	CT	X	6053058	6057177	WBGene00016661	C45B2.6	C45B2.6	-0.0958279497103742	AGIWUR40366
CB4856	CT	X	6058255	6060242	WBGene00001602	gln-1	C45B2.5	-0.119124729450363	

AGIWUR483;AGIWUR9520;AGIWUR30943
CB4856 CT X 6060754 6065986 WBGene00004219 ptr-4 C45B2.7 1.09221963856162
AGIWUR7044;AGIWUR10815;AGIWUR13775;AGIWUR35960;AGIWUR42720;AGIWUR44398
CB4856 CT X 6070497 6081833 WBGene00001587 ggr-2 C45B2.4 0.160035326849795 AGIWUR16871
CB4856 CT X 6082960 6083333 WBGene00016660 C45B2.3 C45B2.3 -0.0912813244908257 AGIWUR36757
CB4856 CT X 6084739 6085189 WBGene00016659 C45B2.2 C45B2.2 0.00926568724323751
AGIWUR7351;AGIWUR20052;AGIWUR28037;AGIWUR36337;AGIWUR44487
CB4856 CT X 6086124 6086567 WBGene00016658 C45B2.1 C45B2.1 -0.259855721480276 AGIWUR13077
CB4856 CT X 6087985 6088391 WBGene00016662 C45B2.8 C45B2.8 -0.570391275050012 AGIWUR42228
CB4856 CT X 6089033 6092124 WBGene00002007 hsp-3 C15H9.6 -0.224087130132488
AGIWUR794;AGIWUR4634;AGIWUR7413;AGIWUR7841;AGIWUR20115;AGIWUR27420;AGIWUR27912;AGIWUR33491;AGIWUR37727;AGIW
UR38048;AGIWUR38338;AGIWUR41750;AGIWUR41826;AGIWUR45001
CB4856 CT X 6093286 6097574 WBGene00015801 C15H9.5 C15H9.5 -0.272710177241043 AGIWUR10532
CB4856 CT X 6101158 6105526 WBGene00015800 C15H9.4 C15H9.4 -0.135977179003302 AGIWUR43548
CB4856 CT X 6106926 6109338 WBGene00015802 kynu-1 C15H9.7 -0.685446248208665 AGIWUR18801
CB4856 CT X 6110754 6113057 WBGene00004193 prx-3 C15H9.8 0.0520025302722473
AGIWUR4666;AGIWUR15011;AGIWUR15322;AGIWUR31300;AGIWUR35692
CB4856 CT X 6115804 6118051 WBGene00015799 C15H9.3 C15H9.3 0.0564054280517867
AGIWUR21830;AGIWUR23000;AGIWUR29763
CB4856 CT X 6118776 6120436 WBGene00015798 C15H9.2 C15H9.2 0.258799632813704 AGIWUR29727
CB4856 CT X 6122315 6127541 WBGene00003778 nnt-1 C15H9.1 1.76242775678261 AGIWUR7268
CB4856 CT X 6127457 6128326 WBGene00015803 C15H9.9 C15H9.9 -0.25337000356366 AGIWUR18082
CB4856 CT X 6129535 6138332 WBGene00022202 Y72A10A.1 Y72A10A.1 -0.228961317161389
AGIWUR13394
CB4856 CT X 6142546 6150545 WBGene00016423 tag-275 C34H3.1 0.158809270128391 AGIWUR24326
CB4856 CT X 6159388 6160523 WBGene00003846 odd-2 C34H3.2 0.393745264582137 AGIWUR34622
CB4856 CT X 6169305 6173748 WBGene00020786 T25B2.2 T25B2.2 0.45850434264478 AGIWUR42133
CB4856 CT X 6176129 6177154 WBGene00077592 T25B2.3 T25B2.3 0.143267823979562 AGIWUR29729
CB4856 CT X 6179241 6180140 WBGene00020484 T13C5.3 T13C5.3 0.0973616780107021 AGIWUR32692
CB4856 CT X 6180650 6181425 WBGene00020487 T13C5.7 T13C5.7 -0.301188523171496 AGIWUR4318
CB4856 CT X 6188148 6189422 WBGene00206360 T13C5.10 T13C5.10 0.137439020833778
AGIWUR3002
CB4856 CT X 6191590 6192948 WBGene00020485 ceh-54 T13C5.4 0.120468733238196 AGIWUR15363
CB4856 CT X 6196927 6200818 WBGene00000905 daf-9 T13C5.1 -0.176229383659749 AGIWUR17268
CB4856 CT X 6201875 6204922 WBGene00000245 bca-1 T13C5.5 0.0570808175176083
AGIWUR7829;AGIWUR10187;AGIWUR11293;AGIWUR21637
CB4856 CT X 6205180 6206475 WBGene00077690 mtp-18 T13C5.8 -0.322213972660064
AGIWUR13852;AGIWUR15465
CB4856 CT X 6206581 6207626 WBGene00020486 T13C5.6 T13C5.6 -0.423635161662069 AGIWUR19108

CB4856 CT X 6208161 6208563 WBGene00015782 C14F11.7 C14F11.7 -0.0954714521570335
AGIWUR44304
CB4856 CT X 6208761 6213911 WBGene00015779 lrch-1 C14F11.2 0.358767899321677
AGIWUR15777
CB4856 CT X 6226131 6230701 WBGene00001803 lite-1 C14F11.3 0.219703675586975
AGIWUR41630
CB4856 CT X 6230920 6233048 WBGene00015780 C14F11.4 C14F11.4 -0.0296376822806508
AGIWUR10334;AGIWUR14376;AGIWUR42427
CB4856 CT X 6233149 6235541 WBGene00002024 hsp-43 C14F11.5 -0.191127759517856
AGIWUR803;AGIWUR2757;AGIWUR3346;AGIWUR5545;AGIWUR7080;AGIWUR11542;AGIWUR12158;AGIWUR12947;AGIWUR13833;AGIWU
R20327;AGIWUR24200;AGIWUR26078;AGIWUR26161;AGIWUR27483;AGIWUR30002;AGIWUR31377;AGIWUR37840;AGIWUR41660
CB4856 CT X 6238021 6239115 WBGene00015781 C14F11.6 C14F11.6 0.882130421129958
AGIWUR1730
CB4856 CT X 6240955 6242709 WBGene00015778 got-2.2 C14F11.1 0.393276286373332
AGIWUR9797;AGIWUR14781;AGIWUR26879
CB4856 CT X 6252274 6253785 WBGene00021050 W05H9.3 W05H9.3 0.0618472507072489 AGIWUR35259
CB4856 CT X 6263648 6266913 WBGene00021051 W05H9.4 W05H9.4 0.272820296967552 AGIWUR44050
CB4856 CT X 6267043 6271087 WBGene00021049 W05H9.2 W05H9.2 -0.0434910988336696
AGIWUR8667;AGIWUR12910;AGIWUR13830;AGIWUR39746;AGIWUR44532
CB4856 CT X 6272089 6273768 WBGene00021048 W05H9.1 W05H9.1 0.84136258938325 AGIWUR30479
CB4856 CT X 6279801 6281015 WBGene00000739 col-166 T07H6.3 -0.0489188873828352
AGIWUR37078;AGIWUR44419
CB4856 CT X 6281847 6291732 WBGene00002976 lev-9 T07H6.5 -0.127693003085821
AGIWUR2946;AGIWUR21872;AGIWUR24441;AGIWUR32386
CB4856 CT X 6293124 6298195 WBGene00003394 mom-1 T07H6.2 -0.187869038611616 AGIWUR4640
CB4856 CT X 6300138 6305211 WBGene00020330 T07H6.1 T07H6.1 0.138642794923898 AGIWUR44690
CB4856 CT X 6311715 6314588 WBGene00020420 T10E10.3 T10E10.3 -0.0407793353478487
AGIWUR439
CB4856 CT X 6314529 6322819 WBGene00020421 T10E10.4 T10E10.4 -0.10914543101008
AGIWUR46
CB4856 CT X 6323297 6324539 WBGene00000740 col-167 T10E10.2 -0.0708610599130211
AGIWUR40205
CB4856 CT X 6329160 6330127 WBGene00000742 col-169 T10E10.5 -0.201429716017275
AGIWUR18709
CB4856 CT X 6331620 6332686 WBGene00000743 col-170 T10E10.6 -0.463606625128667
AGIWUR1815
CB4856 CT X 6335279 6338035 WBGene00015381 C03B1.13 C03B1.13 -0.532262811062235
AGIWUR25465
CB4856 CT X 6341835 6345842 WBGene00015378 C03B1.7 C03B1.7 0.0606526919595023 AGIWUR27113

CB4856	CT	X	6346359 6347113 WBGene00015379	C03B1.9 C03B1.9 0.0148857225369978
AGIWUR1881;AGIWUR13826;AGIWUR18914;AGIWUR19974;AGIWUR23191;AGIWUR24202;AGIWUR32257;AGIWUR34582;AGIWUR37352
CB4856	CT	X	6350187 6351876 WBGene00015377	C03B1.6 C03B1.6 -0.0478919032761485
AGIWUR9258;AGIWUR38721
CB4856	CT	X	6355585 6357689 WBGene00015376	C03B1.5 C03B1.5 0.269416429222995	AGIWUR14829
CB4856	CT	X	6359383 6360758 WBGene00015382	C03B1.14	C03B1.14	0.122075785469658
AGIWUR20078
CB4856	CT	X	6364496 6365717 WBGene00015375	C03B1.4 C03B1.4 0.0963694950620182	AGIWUR44475
CB4856	CT	X	6366013 6366831 WBGene00015374	C03B1.3 C03B1.3 0.148090621593907	AGIWUR9747
CB4856	CT	X	6367614 6373157 WBGene00015373	C03B1.2 C03B1.2 0.0739025032236267	AGIWUR29724
CB4856	CT	X	6374751 6375592 WBGene00015372	C03B1.1 C03B1.1 -0.505729118598061	AGIWUR17004
CB4856	CT	X	6376459 6377684 WBGene00003053	lmp-1	C03B1.12	0.243724197601177
AGIWUR27222;AGIWUR33434
CB4856	CT	X	6379484 6384845 WBGene00020130	igcm-2	SSSD1.1 1.05823909813289	AGIWUR41163
CB4856	CT	X	6385280 6387863 WBGene00020691	igdb-3	T22E5.3 0.486676507617402	AGIWUR40893
CB4856	CT	X	6388255 6391591 WBGene00044572	T22E5.7 T22E5.7 0.0813618569776358
AGIWUR17271;AGIWUR19093;AGIWUR37241;AGIWUR38134;AGIWUR42347
CB4856	CT	X	6403424 6406981 WBGene00001622	glt-4	T22E5.2 0.0320674231988942	AGIWUR27019
CB4856	CT	X	6408470 6410359 WBGene00003495	mup-2	T22E5.5 -0.277919366861735
AGIWUR2620;AGIWUR15971;AGIWUR21994;AGIWUR30200;AGIWUR36727;AGIWUR40271;AGIWUR41617
CB4856	CT	X	6412886 6413488 WBGene00020690	T22E5.1 T22E5.1 -0.453936425367622
AGIWUR4696;AGIWUR12616;AGIWUR17543;AGIWUR17768;AGIWUR31630;AGIWUR44181
CB4856	CT	X	6414148 6416400 WBGene00020692	T22E5.6 T22E5.6 -0.3582154296335	AGIWUR27334
CB4856	CT	X	6421036 6430610 WBGene00019617	K10C2.1 K10C2.1 -0.603006326341657	AGIWUR13082
CB4856	CT	X	6430695 6433788 WBGene00019618	K10C2.2 K10C2.2 0.0777514307872753	AGIWUR31252
CB4856	CT	X	6436440 6437645 WBGene00045265	K10C2.8 K10C2.8 -0.435865106811708
AGIWUR11725;AGIWUR42028;AGIWUR42307
CB4856	CT	X	6443055 6443473 WBGene00019622	K10C2.7 K10C2.7 -0.94277305162688
AGIWUR2183;AGIWUR16937;AGIWUR22507;AGIWUR36334;AGIWUR39501
CB4856	CT	X	6444189 6446664 WBGene00019619	asp-14	K10C2.3 0.00195939624459562	AGIWUR11999
CB4856	CT	X	6446649 6448999 WBGene00019620	fah-1	K10C2.4 -0.277018501807563
AGIWUR10551;AGIWUR17534;AGIWUR30736;AGIWUR31151
CB4856	CT	X	6449959 6450939 WBGene00001715	grl-6	K10C2.5 0.32729608276732	AGIWUR35833
CB4856	CT	X	6451363 6451859 WBGene00019621	K10C2.6 K10C2.6 -0.0712598053084399
AGIWUR3279;AGIWUR4945;AGIWUR27791;AGIWUR32387;AGIWUR37818
CB4856	CT	X	6451800 6455179 WBGene00003551	nas-33	K04E7.3 -0.101228375861744
AGIWUR16368;AGIWUR20434;AGIWUR27178;AGIWUR42590
CB4856	CT	X	6456687 6461767 WBGene00003877	pept-1	K04E7.2 -0.28793487889094	AGIWUR35390
CB4856	CT	X	6462029 6463228 WBGene00019383	K04E7.1 K04E7.1 -0.00462430288692062	AGIWUR11377

CB4856	CT	X	6463412	6465861	WBGene00015784	C15B12.2	C15B12.2	-0.106643012916278	AGIWUR19482;AGIWUR23863;AGIWUR31366
CB4856	CT	X	6467284	6467562	WBGene00015785	C15B12.3	C15B12.3	-0.375621260898823	AGIWUR35027
CB4856	CT	X	6468799	6469803	WBGene00044573	C15B12.9	C15B12.9	0.0977045378168544	AGIWUR12978;AGIWUR17588;AGIWUR32647
CB4856	CT	X	6470366	6473748	WBGene00015786	C15B12.4	C15B12.4	0.325903426760227	AGIWUR18689
CB4856	CT	X	6474274	6476645	WBGene00015788	C15B12.8	C15B12.8	1.55458580146218	AGIWUR9292
CB4856	CT	X	6476738	6479086	WBGene00015783	C15B12.1	C15B12.1	0.474533884942092	AGIWUR15568
CB4856	CT	X	6479447	6484810	WBGene00001517	gar-1	C15B12.5	0.199182360177459	AGIWUR16993;AGIWUR18743;AGIWUR37260
CB4856	CT	X	6496823	6499836	WBGene00015787	C15B12.6	C15B12.6	-0.218353856816146	AGIWUR17851;AGIWUR20196;AGIWUR43327
CB4856	CT	X	6499928	6504613	WBGene00000393	cdf-1	C15B12.7	-0.042388109815672	AGIWUR21474;AGIWUR32017;AGIWUR36111;AGIWUR37609
CB4856	CT	X	6506884	6509882	WBGene00001619	glr-8	F22A3.3	0.192327062191822	AGIWUR3455;AGIWUR18484;AGIWUR21797
CB4856	CT	X	6510929	6512195	WBGene00044605	ttr-36	F22A3.7	-0.517917007678735	AGIWUR298;AGIWUR5370;AGIWUR19336;AGIWUR19999
CB4856	CT	X	6512285	6512956	WBGene00017688	ttr-35	F22A3.2	-0.585807778116804	AGIWUR21619
CB4856	CT	X	6518883	6522656	WBGene00017687	ets-4	F22A3.1	0.287660254797413	AGIWUR889;AGIWUR3485;AGIWUR9857;AGIWUR11604;AGIWUR17443;AGIWUR24709;AGIWUR28144;AGIWUR30453;AGIWUR34260;AGIWUR34579
CB4856	CT	X	6522596	6530148	WBGene00017690	ceh-60	F22A3.5	0.136642011305968	AGIWUR4658;AGIWUR39511
CB4856	CT	X	6533493	6536840	WBGene00017691	ilys-5	F22A3.6	-0.456210633960093	AGIWUR4971;AGIWUR38630
CB4856	CT	X	6542992	6543983	WBGene00020505	T14E8.2	T14E8.2	0.197596865212581	AGIWUR20726
CB4856	CT	X	6553288	6564207	WBGene00020504	svh-2	T14E8.1	0.214607528669173	AGIWUR2337;AGIWUR14132;AGIWUR37556
CB4856	CT	X	6564175	6569518	WBGene00020506	dop-3	T14E8.3	0.0353172975150375	AGIWUR10355;AGIWUR12156;AGIWUR26923
CB4856	CT	X	6582004	6585237	WBGene00020885	T28B4.2	T28B4.2	0.0463305672172627	AGIWUR24266
CB4856	CT	X	6587898	6596135	WBGene00020884	T28B4.1	T28B4.1	0.224954798709703	AGIWUR24308
CB4856	CT	X	6596425	6597121	WBGene00020886	ttr-6	T28B4.3	-0.142097119687827	AGIWUR24030
CB4856	CT	X	6597918	6598866	WBGene00020887	T28B4.4	T28B4.4	0.200325195425307	AGIWUR26842

CB4856 CT X 6601558 6603212 WBGene00044574 F23G4.1 F23G4.1 -0.157482246676222 AGIWUR10940;AGIWUR25657;AGIWUR37205
CB4856 CT X 6607203 6609045 WBGene00000786 cpr-6 C25B8.3 -0.278207311468115 AGIWUR4925;AGIWUR5195;AGIWUR8195;AGIWUR11216;AGIWUR11804;AGIWUR20912;AGIWUR28068;AGIWUR28947
CB4856 CT X 6610054 6615343 WBGene00044297 C25B8.8 C25B8.8 -0.0581707251895818 AGIWUR27278;AGIWUR29524
CB4856 CT X 6617393 6622915 WBGene00016088 clec-266 C25B8.4 0.855609437226094 AGIWUR19062;AGIWUR24639;AGIWUR25897;AGIWUR35797;AGIWUR39462;AGIWUR40914
CB4856 CT X 6619022 6628017 WBGene00002233 kqt-1 C25B8.1 0.205269283748228 AGIWUR20419
CB4856 CT X 6628041 6631537 WBGene00016090 aexr-2 C25B8.7 0.0776060953771829 AGIWUR3032;AGIWUR6225;AGIWUR42800
CB4856 CT X 6631927 6635035 WBGene00016089 aexr-1 C25B8.5 0.112975885440183 AGIWUR10697;AGIWUR15587;AGIWUR44665
CB4856 CT X 6636561 6640620 WBGene00003710 nhr-120 C25B8.6 0.0361200218707509 AGIWUR24251
CB4856 CT X 6645654 6651753 WBGene00016976 C56E10.3 C56E10.3 -0.053460926760156 AGIWUR16579
CB4856 CT X 6655547 6659966 WBGene00016975 nhr-173 C56E10.1 0.325959012009468 AGIWUR38723
CB4856 CT X 6661791 6669011 WBGene00003727 nhr-137 C56E10.4 -0.345999518619008 AGIWUR26005
CB4856 CT X 6672830 6674407 WBGene00000745 col-172 F38B6.5 -0.224339953909005 AGIWUR2848;AGIWUR4663;AGIWUR37133
CB4856 CT X 6674869 6679848 WBGene00018174 F38B6.4 F38B6.4 -0.658651527586681 AGIWUR5569
CB4856 CT X 6680901 6682783 WBGene00044575 F38B6.8 F38B6.8 0.0444208966252821 AGIWUR32608;AGIWUR44990
CB4856 CT X 6684440 6690085 WBGene00018175 F38B6.6 F38B6.6 0.0931938521473148 AGIWUR25333;AGIWUR42026
CB4856 CT X 6693735 6695662 WBGene00018173 F38B6.3 F38B6.3 -0.021583492071108 AGIWUR1165
CB4856 CT X 6695820 6698337 WBGene00018176 F38B6.7 F38B6.7 0.173519929614105 AGIWUR20268
CB4856 CT X 6698533 6700826 WBGene00018172 F38B6.2 F38B6.2 -0.357789816346254 AGIWUR8745;AGIWUR28477
CB4856 CT X 6702463 6709142 WBGene00000042 acr-2 K11G12.2 -0.0197261792646755 AGIWUR12651;AGIWUR15050
CB4856 CT X 6709538 6712925 WBGene00000043 acr-3 K11G12.7 -0.34813712546252 AGIWUR1653
CB4856 CT X 6713577 6716381 WBGene00003530 nas-11 K11G12.1 -0.170037257383539 AGIWUR25194
CB4856 CT X 6719666 6723066 WBGene00004876 smf-1 K11G12.4 0.0448351194768257 AGIWUR3059

CB4856 CT X 6723500 6725668 WBGene00019656 slc-25A10 K11G12.5 -0.116741262538209
AGIWUR19365
CB4856 CT X 6725781 6729748 WBGene00019657 K11G12.6 K11G12.6 0.272557123939439
AGIWUR6530;AGIWUR10261;AGIWUR11629
CB4856 CT X 6729997 6733315 WBGene00019843 R03E9.2 R03E9.2 0.197471891300953 AGIWUR12973
CB4856 CT X 6742351 6743899 WBGene00003163 mdl-1 R03E9.1 0.837247073742525
AGIWUR4901;AGIWUR16512;AGIWUR26064;AGIWUR45173
CB4856 CT X 6747296 6761738 WBGene00019844 abts-4 R03E9.3 0.509666980525849
AGIWUR6213;AGIWUR6511;AGIWUR21622;AGIWUR27359;AGIWUR31458;AGIWUR35645
CB4856 CT X 6766083 6772946 WBGene00002149 irk-1 R03E9.4 0.244832367319494
AGIWUR5497;AGIWUR11639
CB4856 CT X 6783157 6784197 WBGene00019226 H22K11.3 H22K11.3 0.134011030038407
AGIWUR36514
CB4856 CT X 6784411 6787296 WBGene00019225 bgal-2 H22K11.2 0.00870118156387178
AGIWUR40471
CB4856 CT X 6787256 6789578 WBGene00019227 sgca-1 H22K11.4 -0.118416412922516
AGIWUR35089
CB4856 CT X 6791064 6792715 WBGene00000216 asp-3 H22K11.1 -0.131360161460902
AGIWUR6252;AGIWUR11666;AGIWUR14411;AGIWUR14860;AGIWUR31198;AGIWUR36688
CB4856 CT X 6800688 6803700 WBGene00019749 M03A8.3 M03A8.3 -0.0341247316502997
AGIWUR7715;AGIWUR32523
CB4856 CT X 6809104 6817266 WBGene00019748 atg-2 M03A8.2 0.505876038046676 AGIWUR4789
CB4856 CT X 6818438 6820080 WBGene00000991 dhs-28 M03A8.1 0.0551840389070364
AGIWUR7084;AGIWUR8935;AGIWUR10246;AGIWUR13173;AGIWUR15478;AGIWUR33060;AGIWUR33621
CB4856 CT X 6820005 6823268 WBGene00001572 gei-15 M03A8.4 0 AGIWUR6969
CB4856 CT X 6823890 6825862 WBGene00018257 F41B4.1 F41B4.1 -0.163136208371565 AGIWUR30496
CB4856 CT X 6831251 6835266 WBGene00018258 F41B4.2 F41B4.2 -0.0518690028306779
AGIWUR6648;AGIWUR31181
CB4856 CT X 6835487 6836115 WBGene00018259 F41B4.3 F41B4.3 -0.646051378122939 AGIWUR26760
CB4856 CT X 6836386 6840615 WBGene00001617 glr-6 F41B4.4 -0.124547144273857
AGIWUR8107;AGIWUR34692
CB4856 CT X 6841258 6842129 WBGene00000210 asg-2 C53B7.4 -0.179662615350619 AGIWUR24638
CB4856 CT X 6844755 6845976 WBGene00016894 C53B7.3 C53B7.3 0.859603048040776
AGIWUR39143;AGIWUR43670
CB4856 CT X 6846233 6847547 WBGene00016893 C53B7.2 C53B7.2 -0.0855976328130628
AGIWUR12377;AGIWUR30277
CB4856 CT X 6853618 6860922 WBGene00004370 rig-3 C53B7.1 0.248666207667625 AGIWUR369
CB4856 CT X 6860946 6863414 WBGene00016896 nep-4 C53B7.7 0.103678186050164
AGIWUR10965;AGIWUR15889;AGIWUR21335

CB4856 CT X 6864313 6865939 WBGene00003856 odr-10 C53B7.5 0.0211174233170883 AGIWUR17818
CB4856 CT X 6870354 6871670 WBGene00018276 F41C6.2 F41C6.2 -0.181916515483259 AGIWUR21975
CB4856 CT X 6873359 6874823 WBGene00018278 F41C6.4 F41C6.4 -0.638252028847097 AGIWUR42965
CB4856 CT X 6875131 6877536 WBGene00000746 col-173 F41C6.5 -0.339038711082898 AGIWUR44310
CB4856 CT X 6880909 6881571 WBGene00018279 F41C6.6 F41C6.6 0.0953397023911062 AGIWUR9981
CB4856 CT X 6882493 6885277 WBGene00018280 F41C6.7 F41C6.7 -0.0237638188373612 AGIWUR37348
CB4856 CT X 6889689 6897147 WBGene00006746 unc-6 F41C6.1 0.418503304200795 AGIWUR36208
CB4856 CT X 6898650 6899948 WBGene00017446 F14B8.4 F14B8.4 -0.494306932870625 AGIWUR26049
CB4856 CT X 6901378 6903985 WBGene00003987 pes-23 F14B8.3 -0.0776108947791467
AGIWUR7012;AGIWUR11006;AGIWUR22618
CB4856 CT X 6904721 6905849 WBGene00017445 sid-5 F14B8.2 0.136117888820866 AGIWUR3606
CB4856 CT X 6914254 6921126 WBGene00003732 nhx-4 F14B8.1 0.182948083810233 AGIWUR24792
CB4856 CT X 6921646 6924764 WBGene00017449 otpl-3 F14B8.7 -0.291161556533517 AGIWUR35094
CB4856 CT X 6924804 6928704 WBGene00017447 F14B8.5 F14B8.5 0.174106261358686
AGIWUR2134;AGIWUR7909;AGIWUR15355;AGIWUR16145;AGIWUR16298;AGIWUR18264;AGIWUR28130;AGIWUR34979;AGIWUR40952;A
GIWUR41459
CB4856 CT X 6929262 6932359 WBGene00017448 F14B8.6 F14B8.6 0.127608025433577 AGIWUR30709
CB4856 CT X 6935732 6939041 WBGene00015866 C16E9.2 C16E9.2 -0.0215340721225861
AGIWUR31984;AGIWUR40570;AGIWUR40974
CB4856 CT X 6940253 6943713 WBGene00015865 C16E9.1 C16E9.1 0.26801306323458 AGIWUR38169
CB4856 CT X 6943740 6956399 WBGene00002123 inx-1 C16E9.4 -0.0594957629223707
AGIWUR1327;AGIWUR4443;AGIWUR41082
CB4856 CT X 6967369 6974864 WBGene00017156 F01E11.3 F01E11.3 0.202607598969973
AGIWUR8518;AGIWUR17384;AGIWUR32545
CB4856 CT X 6980784 6983132 WBGene00219372 F01E11.17 F01E11.17 0.109666487683812
AGIWUR29020
CB4856 CT X 6991727 6994162 WBGene00017154 ugt-57 F01E11.1 0.278415834949356
AGIWUR1259;AGIWUR9010;AGIWUR39364
CB4856 CT X 6994784 6996620 WBGene00006642 tsp-16 F01E11.4 0.181385989298921
AGIWUR15782
CB4856 CT X 6996873 7005575 WBGene00017157 tyra-2 F01E11.5 0.0152181155819164
AGIWUR1834;AGIWUR19308;AGIWUR32958;AGIWUR34968
CB4856 CT X 7020033 7023950 WBGene00000283 cah-5 R173.1 -0.0757033974632364 AGIWUR13092
CB4856 CT X 7029136 7029638 WBGene00020126 flp-26 R173.4 0.137544413433361 AGIWUR20847
CB4856 CT X 7030225 7036084 WBGene00020125 R173.3 R173.3 0.17921262250296 AGIWUR11682
CB4856 CT X 7050577 7055667 WBGene00006712 ubc-17 B0403.2 -0.0475206708769242
AGIWUR6165;AGIWUR7860;AGIWUR8038;AGIWUR9008;AGIWUR14371;AGIWUR37718
CB4856 CT X 7057731 7061190 WBGene00015169 B0403.5 B0403.5 -0.690189755807879
AGIWUR25613;AGIWUR42236

CB4856	CT	X	7061203	7063330	WBGene00015167	B0403.3	B0403.3	-0.125985850071523	AGIWUR23954
CB4856	CT	X	7066345	7068563	WBGene00015168	pdi-6	B0403.4	-0.0551609389499892	AGIWUR13446;AGIWUR43810
CB4856	CT	X	7071410	7075337	WBGene00043982	B0403.6	B0403.6	0.459007232254335	AGIWUR11703;AGIWUR39969
CB4856	CT	X	7083417	7086008	WBGene00016467	C36B7.4	C36B7.4	-0.0898553521318064	AGIWUR5620
CB4856	CT	X	7089228	7096449	WBGene00016468	C36B7.5	C36B7.5	-0.0252760144802329	AGIWUR10931
CB4856	CT	X	7097434	7100338	WBGene00016469	C36B7.6	C36B7.6	-0.306233702227852	AGIWUR5911;AGIWUR24672;AGIWUR26892;AGIWUR34244;AGIWUR38062
CB4856	CT	X	7101777	7102136	WBGene00016466	C36B7.3	C36B7.3	-0.130035805611717	AGIWUR17906
CB4856	CT	X	7106407	7108436	WBGene00016465	C36B7.2	C36B7.2	0.193082946025584	AGIWUR9259
CB4856	CT	X	7112889	7114863	WBGene00016464	C36B7.1	C36B7.1	0.12628524753482	AGIWUR16046;AGIWUR22687
CB4856	CT	X	7116205	7116996	WBGene00001841	hen-1	C36B7.7	0.24341390593647	AGIWUR31932
CB4856	CT	X	7126648	7139646	WBGene00002163	ist-1	C54D1.3	0.0919086665060469	AGIWUR8309;AGIWUR22991;AGIWUR28042;AGIWUR32682;AGIWUR43007;AGIWUR44474
CB4856	CT	X	7139875	7140566	WBGene00016912	clec-86	C54D1.2	-0.980200282529307	AGIWUR21885
CB4856	CT	X	7140504	7143641	WBGene00000116	alh-10	C54D1.4	0.346148831431531	AGIWUR9700
CB4856	CT	X	7143918	7151235	WBGene00016913	lam-2	C54D1.5	-0.29147676411304	AGIWUR1996;AGIWUR27256
CB4856	CT	X	7153889	7156035	WBGene00016914	C54D1.7	C54D1.7	0.139166841939339	AGIWUR27463
CB4856	CT	X	7159036	7166021	WBGene00004004	pgp-10	C54D1.1	0.0663266515615657	AGIWUR15114
CB4856	CT	X	7166005	7170625	WBGene00000238	bar-1	C54D1.6	0.0330679450278453	AGIWUR1646
CB4856	CT	X	7177641	7178922	WBGene00044577	F43E12.1	F43E12.1	-0.0892566144969225	AGIWUR7496;AGIWUR16469;AGIWUR42022;AGIWUR44522
CB4856	CT	X	7187355	7188482	WBGene00016940	C55B6.4	C55B6.4	0.116232712997785	AGIWUR32410
CB4856	CT	X	7194564	7198291	WBGene00001025	dnj-7	C55B6.2	-0.29755968207125	AGIWUR1962;AGIWUR10779;AGIWUR11340;AGIWUR14618;AGIWUR30335;AGIWUR34569
CB4856	CT	X	7200364	7204044	WBGene00016939	C55B6.1	C55B6.1	-0.31114451960293	AGIWUR30579
CB4856	CT	X	7204742	7205295	WBGene00016941	C55B6.5	C55B6.5	0.0498918052936475	AGIWUR4101
CB4856	CT	X	7206719	7209985	WBGene00022829	ZK867.2	ZK867.2	0.25799553231832	AGIWUR27793;AGIWUR39082
CB4856	CT	X	7211705	7213956	WBGene00044283	spp-22	ZK867.3	-0.0410841315974064	AGIWUR30676
CB4856	CT	X	7218109	7226825	WBGene00044068	syd-9	ZK867.1	0.570091829808591	AGIWUR366;AGIWUR8810;AGIWUR22384;AGIWUR28559
CB4856	CT	X	7230330	7238662	WBGene00018519	F46H5.3	F46H5.3	-0.0536266819705595	AGIWUR2881;AGIWUR5451;AGIWUR6990;AGIWUR30972;AGIWUR32854
CB4856	CT	X	7238666	7242983	WBGene00018522	F46H5.7	F46H5.7	0.146940638114216	AGIWUR9564;AGIWUR9695;AGIWUR14090;AGIWUR18319;AGIWUR19653;AGIWUR23962;AGIWUR26751;AGIWUR36620

CB4856	CT	X	7245057	7254792	WBGene00018520	F46H5.4	F46H5.4	0.176328169620092	AGIWUR39855
CB4856	CT	X	7255927	7256978	WBGene00018521	F46H5.5	F46H5.5	-0.0389286205978374	AGIWUR29869
CB4856	CT	X	7258116	7260377	WBGene00018523	lact-1	F46H5.8	-0.579560881509998	AGIWUR2426;AGIWUR13200;AGIWUR29644
CB4856	CT	X	7265652	7267893	WBGene00018518	F46H5.2	F46H5.2	-0.241474283962955	AGIWUR7598;AGIWUR15397;AGIWUR17951;AGIWUR27236;AGIWUR37973
CB4856	CT	X	7267989	7272067	WBGene00001696	grd-7	F46H5.6	-0.306977945694281	AGIWUR8059
CB4856	CT	X	7272334	7280322	WBGene00006750	unc-10	T10A3.1	0.0698847313350113	AGIWUR1087;AGIWUR8869;AGIWUR23174;AGIWUR43203
CB4856	CT	X	7292138	7296035	WBGene00019351	lron-7	K03A1.2	0.00879734706133384	AGIWUR24250
CB4856	CT	X	7296098	7298751	WBGene00019352	K03A1.4	K03A1.4	0.11201063233713	AGIWUR14580
CB4856	CT	X	7301357	7305570	WBGene00006351	sur-5	K03A1.5	-0.254846147494153	AGIWUR8047;AGIWUR19938;AGIWUR20779;AGIWUR21098
CB4856	CT	X	7312175	7313976	WBGene00020338	T08A9.6	T08A9.6	-0.148068593744619	AGIWUR11970;AGIWUR20221;AGIWUR32288
CB4856	CT	X	7314969	7315353	WBGene00004988	spp-3	T08A9.7	-0.112553841253799	AGIWUR831;AGIWUR28222;AGIWUR42444
CB4856	CT	X	7315995	7316818	WBGene00004987	spp-2	T08A9.12	2.20555007434137	AGIWUR2832;AGIWUR4441;AGIWUR7152;AGIWUR41734
CB4856	CT	X	7317341	7317681	WBGene00004991	spp-6	T08A9.10	2.41376078370715	AGIWUR3981
CB4856	CT	X	7318239	7318798	WBGene00004989	spp-4	T08A9.8	-0.398950789853556	AGIWUR39959
CB4856	CT	X	7319935	7320384	WBGene00004990	spp-5	T08A9.9	-0.0239771803750877	AGIWUR16988
CB4856	CT	X	7321999	7322415	WBGene00044442	T08A9.13	T08A9.13	0.175205443829238	AGIWUR739;AGIWUR14660;AGIWUR17977;AGIWUR19453;AGIWUR23763
CB4856	CT	X	7327605	7329677	WBGene00004912	sng-1	T08A9.3	-0.0818155190960752	AGIWUR19181
CB4856	CT	X	7329818	7330669	WBGene00020339	ttr-59	T08A9.11	-0.0719099962829528	AGIWUR43881
CB4856	CT	X	7331189	7331903	WBGene00020335	ttr-30	T08A9.2	-0.31590375207119	AGIWUR26782
CB4856	CT	X	7334714	7340224	WBGene00020334	epg-7	T08A9.1	0.21694855504909	AGIWUR13141
CB4856	CT	X	7341509	7342780	WBGene00015565	C07D8.6	C07D8.6	-0.477413922987679	AGIWUR2633;AGIWUR25877
CB4856	CT	X	7344047	7344551	WBGene00015564	C07D8.5	C07D8.5	0.256594619305286	AGIWUR16285
CB4856	CT	X	7362082	7363663	WBGene00015562	C07D8.2	C07D8.2	0.133538878172117	AGIWUR2955
CB4856	CT	X	7365567	7366298	WBGene00016985	C56G3.2	C56G3.2	0.218612846456583	AGIWUR3048;AGIWUR12259;AGIWUR20965;AGIWUR21589;AGIWUR22826;AGIWUR29368;AGIWUR31432;AGIWUR35429
CB4856	CT	X	7377302	7388400	WBGene00016984	npr-8	C56G3.1	0.186476795988263	AGIWUR30083
CB4856	CT	X	7390574	7392276	WBGene00015667	C10A4.6	C10A4.6	0.133369511546171	AGIWUR8450
CB4856	CT	X	7393467	7395987	WBGene00015666	gad-2	C10A4.5	-0.112624863742456	AGIWUR36308

CB4856	CT	X	7398884	7400858	WBGene00050957	C10A4.10	C10A4.10	-0.235181589952729
AGIWUR23007
CB4856	CT	X	7402131	7403609	WBGene00015665	C10A4.4	C10A4.4	-0.208235990220212	AGIWUR10942
CB4856	CT	X	7407456	7408703	WBGene00015664	C10A4.3	C10A4.3	0.558767953630307	AGIWUR6197
CB4856	CT	X	7410235	7412749	WBGene00015663	C10A4.2	C10A4.2	0.00652155767710916	AGIWUR37959
CB4856	CT	X	7413611	7415520	WBGene00015668	C10A4.7	C10A4.7	-0.0742917550734363	AGIWUR1003
CB4856	CT	X	7415839	7418627	WBGene00015662	C10A4.1	C10A4.1	-0.21669383860117	AGIWUR5893
CB4856	CT	X	7420871	7422628	WBGene00045456	C10A4.9	C10A4.9	-0.105799926602276
AGIWUR3200;AGIWUR10221;AGIWUR10599;AGIWUR13837;AGIWUR15117
CB4856	CT	X	7422641	7424881	WBGene00003380	mnm-2	C10A4.8	0.431456684343932	AGIWUR7370
CB4856	CT	X	7431472	7433548	WBGene00000523	clc-2	C01C10.1	0.0804268877859498
AGIWUR8585;AGIWUR41135
CB4856	CT	X	7433632	7435733	WBGene00000526	clc-5	C01C10.4	0.490122035319086
AGIWUR9626
CB4856	CT	X	7435602	7438000	WBGene00015294	C01C10.2	C01C10.2	0.0597186474840541
AGIWUR10583;AGIWUR28046;AGIWUR41797
CB4856	CT	X	7439396	7442577	WBGene00015295	acl-12	C01C10.3	0.427780252531662
AGIWUR24331
CB4856	CT	X	7458293	7463736	WBGene00004949	sox-2	K08A8.2	0.36932667656043
AGIWUR580;AGIWUR1927;AGIWUR2157;AGIWUR9025;AGIWUR12690;AGIWUR16873;AGIWUR17998;AGIWUR18918;AGIWUR23196;AGIW
UR23402;AGIWUR29307;AGIWUR34708;AGIWUR35922
CB4856	CT	X	7465193	7475014	WBGene00000591	coh-1	K08A8.3	0.181437314858263
AGIWUR8999;AGIWUR9946;AGIWUR16896;AGIWUR24797;AGIWUR25003;AGIWUR30115
CB4856	CT	X	7479248	7481010	WBGene00003185	mek-1	K08A8.1	0.0345203707807169
AGIWUR4305;AGIWUR11188;AGIWUR43700
CB4856	CT	X	7481326	7485038	WBGene00018608	F48E3.9	F48E3.9	0.256781403191678
AGIWUR4018;AGIWUR26749;AGIWUR26772
CB4856	CT	X	7485864	7490707	WBGene00001637	gly-12	F48E3.1	-0.489980958471158	AGIWUR21551
CB4856	CT	X	7493724	7495591	WBGene00018605	F48E3.4	F48E3.4	-0.140771535306776	AGIWUR24512
CB4856	CT	X	7495684	7500982	WBGene00018604	uggt-1	F48E3.3	-0.242899277670272	AGIWUR23074
CB4856	CT	X	7501236	7502674	WBGene00018606	F48E3.6	F48E3.6	0.0357067210374495	AGIWUR16467
CB4856	CT	X	7503823	7506633	WBGene00000061	lgc-11	F48E3.7	-0.126963054699333	AGIWUR43951
CB4856	CT	X	7507885	7517694	WBGene00018607	F48E3.8	F48E3.8	0.463148678025122
AGIWUR13250;AGIWUR17401;AGIWUR20280;AGIWUR34154;AGIWUR35820;AGIWUR41858
CB4856	CT	X	7525571	7527433	WBGene00018485	F46C8.3	F46C8.3	0.279532968240327	AGIWUR15002
CB4856	CT	X	7528616	7532732	WBGene00000438	ceh-14	F46C8.5	0.0117515306582057	AGIWUR29513
CB4856	CT	X	7536417	7537792	WBGene00001069	dpy-7	F46C8.6	0.796790264575051
AGIWUR4342;AGIWUR38497
CB4856	CT	X	7537844	7540129	WBGene00018486	glb-16	F46C8.7	-0.0665154769096494	AGIWUR8108

CB4856	CT	X	7546842 7548365 WBGene00000747	col-174 F46C8.2 0.0451929862416298	AGIWUR17224
CB4856	CT	X	7548458 7551218 WBGene00018484	F46C8.1 F46C8.1 -0.0393143557906335	AGIWUR21652
CB4856	CT	X	7551204 7554426 WBGene00018487	F46C8.8 F46C8.8 -0.325416294475874	AGIWUR23957
CB4856	CT	X	7556788 7559085 WBGene00019803	PDB1.1	PDB1.1	-0.132346434299486
AGIWUR8001;AGIWUR9508;AGIWUR11133;AGIWUR16595;AGIWUR20548;AGIWUR26709
CB4856	CT	X	7560024 7562093 WBGene00017243	F08C6.5 F08C6.5 0.221498485889965	AGIWUR31069
CB4856	CT	X	7562831 7564805 WBGene00006063	sto-1	F08C6.4 0.691679435048256	AGIWUR31380
CB4856	CT	X	7565323 7568152 WBGene00007059	vps-52	F08C6.3 -0.105086895204973	AGIWUR5089
CB4856	CT	X	7568137 7569722 WBGene00017244	apy-1	F08C6.6 -0.60829000372102	AGIWUR19261
CB4856	CT	X	7570374 7573751 WBGene00006827	unc-98	F08C6.7 0.0369051515118226	AGIWUR41594
CB4856	CT	X	7572110 7581059 WBGene00017241	pcyt-1	F08C6.2 -0.170381006404657
AGIWUR294;AGIWUR13737
CB4856	CT	X	7585587 7595414 WBGene00000083	adt-2	F08C6.1 -0.0992400981284653
AGIWUR10684;AGIWUR11003;AGIWUR15407;AGIWUR18101;AGIWUR19164;AGIWUR21177;AGIWUR26814;AGIWUR40645;AGIWUR40834
;AGIWUR41449;AGIWUR42558;AGIWUR43389
CB4856	CT	X	7596927 7599824 WBGene00016657	C44E12.1	C44E12.1	0.347536602788669
AGIWUR899;AGIWUR7416;AGIWUR11671;AGIWUR12930;AGIWUR13227;AGIWUR43370
CB4856	CT	X	7599836 7608135 WBGene00006671	twk-17	C44E12.3	-0.0558553121098484
AGIWUR3885;AGIWUR20444;AGIWUR20522;AGIWUR24943;AGIWUR29077;AGIWUR33963;AGIWUR39488
CB4856	CT	X	7627649 7627848 WBGene00017813	F26A10.1	F26A10.1	0.113079789650527
AGIWUR2562
CB4856	CT	X	7630905 7637172 WBGene00017814	F26A10.2	F26A10.2	0.46491487789204
AGIWUR12816;AGIWUR43740
CB4856	CT	X	7651132 7651548 WBGene00017862	F27D9.3 F27D9.3 -0.051498876613417	AGIWUR33666
CB4856	CT	X	7653135 7654425 WBGene00017863	F27D9.4 F27D9.4 -0.3995798384984	AGIWUR37245
CB4856	CT	X	7657538 7687738 WBGene00017864	pcca-1	F27D9.5 -0.0547048714968446
AGIWUR2629;AGIWUR3378;AGIWUR6786;AGIWUR14447;AGIWUR31106;AGIWUR38037
CB4856	CT	X	7690590 7707086 WBGene00002242	kvs-1	C53C9.3 -0.106733549765989
AGIWUR2769;AGIWUR11806;AGIWUR34819
CB4856	CT	X	7710170 7710716 WBGene00019583	K09F5.4 K09F5.4 -0.292153894905461	AGIWUR25180
CB4856	CT	X	7719272 7725583 WBGene00044698	K09F5.6 K09F5.6 0.237073530826109
AGIWUR5167;AGIWUR13606;AGIWUR14314;AGIWUR22242;AGIWUR39871
CB4856	CT	X	7725955 7726383 WBGene00004999	spp-14	K09F5.3 0.1441541339619 AGIWUR11497
CB4856	CT	X	7727889 7733168 WBGene00006925	vit-1	K09F5.2 -0.343938193176765	AGIWUR31944
CB4856	CT	X	7733334 7736271 WBGene00019584	set-12	K09F5.5 -0.0281271184320371	AGIWUR39637
CB4856	CT	X	7739653 7741906 WBGene00019582	K09F5.1 K09F5.1 0.198053535272576	AGIWUR8592
CB4856	CT	X	7744239 7750010 WBGene00004335	ref-2	C47C12.3	0.47112314005198
AGIWUR12202;AGIWUR17761;AGIWUR31512;AGIWUR35474
CB4856	CT	X	7754046 7754284 WBGene00016736	C47C12.2	C47C12.2	0.361466107156494

AGIWUR14617
CB4856 CT X 7763187 7774915 WBGene00000950 deg-1 C47C12.6 0.598615186256332
AGIWUR12770
CB4856 CT X 7774859 7776689 WBGene00003171 mec-7 ZK154.3 -0.220715262288661
AGIWUR35991;AGIWUR36555
CB4856 CT X 7777031 7782537 WBGene00022666 ZK154.4 ZK154.4 0.0256322979870642 AGIWUR35005
CB4856 CT X 7783190 7787966 WBGene00022667 ZK154.5 ZK154.5 -0.117815784823318 AGIWUR30208
CB4856 CT X 7790178 7793563 WBGene00022668 ZK154.6 ZK154.6 0.154116446704903
AGIWUR5581;AGIWUR6159;AGIWUR34648
CB4856 CT X 7795046 7798287 WBGene00000075 adm-4 ZK154.7 -0.335317996095357 AGIWUR20603
CB4856 CT X 7798955 7799780 WBGene00019846 gpx-7 R03G5.5 -0.147491070951403
AGIWUR17391;AGIWUR26627;AGIWUR31329;AGIWUR34737
CB4856 CT X 7800445 7805676 WBGene00019845 irld-13 R03G5.3 0.0114687666875222 AGIWUR27593
CB4856 CT X 7805629 7809134 WBGene00019847 R03G5.6 R03G5.6 0.310332136897997
AGIWUR39142;AGIWUR41155
CB4856 CT X 7816720 7821045 WBGene00004758 sek-1 R03G5.2 0.0688402043346201 AGIWUR29353
CB4856 CT X 7823652 7825769 WBGene00001169 eef-1A.2 R03G5.1 0.197312032315999
AGIWUR8817;AGIWUR13058;AGIWUR15776;AGIWUR20915;AGIWUR23861;AGIWUR35550;AGIWUR38611;AGIWUR39303;AGIWUR41511
CB4856 CT X 7827085 7834535 WBGene00006310 sul-3 C54D2.4 0.763972865256275 AGIWUR45187
CB4856 CT X 7838899 7839381 WBGene00016916 C54D2.2 C54D2.2 0.260390897136221 AGIWUR18655
CB4856 CT X 7842632 7845438 WBGene00016915 C54D2.1 C54D2.1 0.231195605987452 AGIWUR37793
CB4856 CT X 7846190 7847460 WBGene00045210 C54D2.6 C54D2.6 -0.225062398365349
AGIWUR8415;AGIWUR15984
CB4856 CT X 7847682 7875954 WBGene00000367 cca-1 C54D2.5 -0.0486456514156428
AGIWUR13850;AGIWUR17664;AGIWUR22438;AGIWUR36286
CB4856 CT X 7879771 7880948 WBGene00016535 C39D10.8 C39D10.8 -0.428133318908973
AGIWUR154;AGIWUR858;AGIWUR1029;AGIWUR1163;AGIWUR17488;AGIWUR25598;AGIWUR25753;AGIWUR30213;AGIWUR39081;AGIWU
R39426;AGIWUR44038
CB4856 CT X 7884288 7887479 WBGene00016531 C39D10.3 C39D10.3 0.239410866797989
AGIWUR11017
CB4856 CT X 7889215 7889765 WBGene00016530 C39D10.2 C39D10.2 0.0470215328971175
AGIWUR927
CB4856 CT X 7890410 7892396 WBGene00016532 C39D10.5 C39D10.5 0.176730659088538
AGIWUR25921
CB4856 CT X 7896221 7897773 WBGene00016533 C39D10.6 C39D10.6 0.0282018245561542
AGIWUR42464
CB4856 CT X 7901631 7901990 WBGene00016536 C39D10.9 C39D10.9 0.129420728119541
AGIWUR40149
CB4856 CT X 7903280 7905466 WBGene00043983 C39D10.11 C39D10.11 0.45207823413369

AGIWUR42504;AGIWUR44558
CB4856 CT X 7906964 7907119 WBGene00016529 C39D10.1 C39D10.1 0.0711300249828799
AGIWUR8159
CB4856 CT X 7907955 7913930 WBGene00016534 C39D10.7 C39D10.7 -0.0523879232149298
AGIWUR28583
CB4856 CT X 7925434 7926594 WBGene00006981 zig-4 C09C7.1 0.259032362796471
AGIWUR17450;AGIWUR19354;AGIWUR30211
CB4856 CT X 7930738 7931808 WBGene00006980 zig-3 C14F5.2 0.792515125143577 AGIWUR2314
CB4856 CT X 7937635 7945048 WBGene00006588 tnt-3 C14F5.3 -0.0866748505519901
AGIWUR26799;AGIWUR31168;AGIWUR36095
CB4856 CT X 7954355 7956211 WBGene00015776 dct-1 C14F5.1 0.671007693105416 AGIWUR26731
CB4856 CT X 7956196 7958303 WBGene00015777 sfxn-2 C14F5.4 -0.303284311413771 AGIWUR21473
CB4856 CT X 7959452 7961723 WBGene00004774 sem-5 C14F5.5 -0.0401269113761913 AGIWUR1438
CB4856 CT X 7976700 7980657 WBGene00018466 F45E1.5 F45E1.5 -0.106084711437918 AGIWUR29946
CB4856 CT X 7981382 7982070 WBGene00001945 his-71 F45E1.6 0.317017435715659 AGIWUR28969
CB4856 CT X 7985979 7988981 WBGene00018465 F45E1.4 F45E1.4 -0.195630921742054
AGIWUR3201;AGIWUR6769
CB4856 CT X 7988931 7998229 WBGene00018467 sdpn-1 F45E1.7 0.273584652129241 AGIWUR28499
CB4856 CT X 8003819 8009400 WBGene00018462 F45E1.1 F45E1.1 -0.015117704554088 AGIWUR39595
CB4856 CT X 8014983 8026858 WBGene00016407 C34D10.2 C34D10.2 0.798762011963793
AGIWUR1066;AGIWUR8041
CB4856 CT X 8029479 8031614 WBGene00016406 C34D10.1 C34D10.1 0.264458104658825
AGIWUR27238
CB4856 CT X 8041327 8044409 WBGene00006962 xol-1 C18A11.5 -0.49226180263214
AGIWUR19381;AGIWUR39183
CB4856 CT X 8045552 8047416 WBGene00015950 C18A11.4 C18A11.4 -0.392198931706983
AGIWUR36837
CB4856 CT X 8050476 8058220 WBGene00001000 dim-1 C18A11.7 -0.148890711965148
AGIWUR746;AGIWUR3893;AGIWUR6746;AGIWUR13907;AGIWUR14088;AGIWUR16775;AGIWUR17481;AGIWUR19007;AGIWUR35992;AGI
WUR36156;AGIWUR36589
CB4856 CT X 8061123 8065688 WBGene00015949 C18A11.3 C18A11.3 -0.436156686390699
AGIWUR9153
CB4856 CT X 8067568 8068095 WBGene00015948 C18A11.2 C18A11.2 0.32149966977328
AGIWUR31132
CB4856 CT X 8069328 8070289 WBGene00015947 C18A11.1 C18A11.1 0.533503106321758
AGIWUR22349;AGIWUR27090;AGIWUR37350;AGIWUR38997
CB4856 CT X 8078063 8080256 WBGene00016714 C46F2.1 C46F2.1 -0.723663999653908 AGIWUR24718
CB4856 CT X 8080869 8084018 WBGene00019492 K07E3.4 K07E3.4 0.56562612317401 AGIWUR28196
CB4856 CT X 8084210 8086550 WBGene00000929 dao-3 K07E3.3 -0.0659525437814033 AGIWUR33499

CB4856	CT	X	8087554	8089427	WBGene00006890	vem-1	K07E3.8	0.00101663190787704	AGIWUR5567
CB4856	CT	X	8089826	8098863	WBGene00019493	catp-5	K07E3.7	-0.228824805721315	AGIWUR33219
CB4856	CT	X	8104305	8107557	WBGene00019491	K07E3.2	K07E3.2	-0.00490506241128141	AGIWUR4890;AGIWUR34188
CB4856	CT	X	8110449	8114762	WBGene00019490	K07E3.1	K07E3.1	-0.0902056046316595	AGIWUR34231
CB4856	CT	X	8116636	8120133	WBGene00001953	hlh-8	C02B8.4	0.231966553508107	AGIWUR37979
CB4856	CT	X	8120410	8123220	WBGene00219375	C02B8.12	C02B8.12	0.0565896316431669	AGIWUR39796
CB4856	CT	X	8133155	8136326	WBGene00015324	C02B8.6	C02B8.6	-0.0450560354930438	AGIWUR5064;AGIWUR14981;AGIWUR16481;AGIWUR17832;AGIWUR30095;AGIWUR30413;AGIWUR42682
CB4856	CT	X	8137721	8137879	WBGene00015325	C02B8.7	C02B8.7	0.271003435354151	AGIWUR31418
CB4856	CT	X	8139446	8140243	WBGene00015322	C02B8.3	C02B8.3	0.121438469642007	AGIWUR2189;AGIWUR25989
CB4856	CT	X	8147710	8148588	WBGene00015320	C02B8.1	C02B8.1	-0.416254868345487	AGIWUR14484
CB4856	CT	X	8149563	8150716	WBGene00017714	F22F1.2	F22F1.2	0.0318213773965645	AGIWUR41354
CB4856	CT	X	8159153	8160084	WBGene00001854	hil-3	F22F1.1	0.901851196400777	AGIWUR7999
CB4856	CT	X	8161839	8163073	WBGene00017715	F22F1.3	F22F1.3	0.0297165535386501	AGIWUR24544
CB4856	CT	X	8169930	8172913	WBGene00015922	C17H11.2	C17H11.2	0.0262807290363529	AGIWUR41888
CB4856	CT	X	8173910	8180865	WBGene00015921	C17H11.1	C17H11.1	0.271549402745195	AGIWUR43707
CB4856	CT	X	8181031	8181640	WBGene00015924	C17H11.4	C17H11.4	0.339245960458721	AGIWUR23254
CB4856	CT	X	8183008	8185133	WBGene00015925	C17H11.5	C17H11.5	-0.0521635848281274	AGIWUR38063
CB4856	CT	X	8185175	8203784	WBGene00015926	C17H11.6	C17H11.6	0.340573019759965	AGIWUR8111;AGIWUR29624;AGIWUR40357
CB4856	CT	X	8208748	8211149	WBGene00000391	cdd-1	C47D2.2	-0.109749814807991	AGIWUR13118
CB4856	CT	X	8215050	8215668	WBGene00016738	C47D2.1	C47D2.1	0.0198998724014814	AGIWUR12024
CB4856	CT	X	8219728	8220064	WBGene00019292	K02A6.1	K02A6.1	0.122645960197912	AGIWUR628
CB4856	CT	X	8224391	8224902	WBGene00019293	K02A6.2	K02A6.2	0.107913952833961	AGIWUR21360
CB4856	CT	X	8228068	8231934	WBGene00019294	K02A6.3	K02A6.3	0.302379881884757	AGIWUR36272
CB4856	CT	X	8232254	8236316	WBGene00019780	M60.4	M60.4	1.19637006140176	AGIWUR8759
CB4856	CT	X	8242056	8247869	WBGene00019779	endu-2	M60.2	0.8477120787819	AGIWUR28695
CB4856	CT	X	8248207	8252458	WBGene00019781	M60.6	M60.6	-0.0295892617913527	AGIWUR6639;AGIWUR20670;AGIWUR39957
CB4856	CT	X	8258149	8261175	WBGene00019782	M60.7	M60.7	0.430281167220615	AGIWUR16017
CB4856	CT	X	8262421	8271021	WBGene00017421	cutl-29	F13B9.6	0.572250022210387	AGIWUR18221
CB4856	CT	X	8275158	8283301	WBGene00002239	ksr-1	F13B9.5	-0.0994371725761489	AGIWUR15566

CB4856 CT X 8283282 8285219 WBGene00001425 fis-2 F13B9.8 -0.110940715273797 AGIWUR29120
CB4856 CT X 8285683 8286785 WBGene00017420 F13B9.2 F13B9.2 -0.447081969898861 AGIWUR31567
CB4856 CT X 8287261 8294514 WBGene00017419 F13B9.1 F13B9.1 0.463965049264456 AGIWUR13018
CB4856 CT X 8294625 8296675 WBGene00019987 R09F10.3 R09F10.3 0.0575545719903996 AGIWUR23087
CB4856 CT X 8299119 8299654 WBGene00000427 ced-13 R09F10.9 -0.0689689140405756 AGIWUR36309
CB4856 CT X 8300030 8302395 WBGene00002127 inx-5 R09F10.4 -0.11738228135646 AGIWUR8644;AGIWUR33230
CB4856 CT X 8306225 8307700 WBGene00019988 R09F10.5 R09F10.5 -0.055055556767158 AGIWUR18056
CB4856 CT X 8313128 8314267 WBGene00005239 srh-13 R09F10.10 0.278228234821183 AGIWUR6738
CB4856 CT X 8315524 8316278 WBGene00005238 srh-12 R09F10.11 0.00343115034820407 AGIWUR16767;AGIWUR22171;AGIWUR31297
CB4856 CT X 8317637 8319050 WBGene00005237 srh-11 R09F10.6 -0.647950994147223 AGIWUR29614
CB4856 CT X 8323592 8325046 WBGene00019986 R09F10.1 R09F10.1 0.00172590679752282 AGIWUR41725
CB4856 CT X 8325099 8326451 WBGene00019989 R09F10.8 R09F10.8 -0.262631291966672 AGIWUR24471
CB4856 CT X 8327559 8327900 WBGene00018883 F55E10.4 F55E10.4 -0.172059025989156 AGIWUR32061
CB4856 CT X 8333247 8337948 WBGene00018884 F55E10.5 F55E10.5 0.172412894063658 AGIWUR28678
CB4856 CT X 8340693 8340986 WBGene00018882 F55E10.2 F55E10.2 -0.00746895344833209 AGIWUR19603
CB4856 CT X 8341975 8342286 WBGene00018881 F55E10.1 F55E10.1 -0.17779718551976 AGIWUR4035
CB4856 CT X 8344700 8346408 WBGene00018885 drd-5 F55E10.6 -0.847087210927649 AGIWUR1745
CB4856 CT X 8348537 8351059 WBGene00018886 npr-28 F55E10.7 0.722602903615226 AGIWUR20796
CB4856 CT X 8368534 8371609 WBGene00018226 F40B5.2 F40B5.2 -0.209606825795063 AGIWUR10274
CB4856 CT X 8372206 8374474 WBGene00018227 nep-15 F40B5.3 -0.328230740732431 AGIWUR17266
CB4856 CT X 8375288 8377304 WBGene00018225 nep-14 F40B5.1 1.46193384442191 AGIWUR37444
CB4856 CT X 8378557 8382700 WBGene00018281 tbc-18 F41D9.1 0.319580694168556 AGIWUR38939
CB4856 CT X 8382706 8384945 WBGene00018282 F41D9.2 F41D9.2 -0.109929308351704 AGIWUR19133
CB4856 CT X 8387448 8392584 WBGene00006942 wrk-1 F41D9.3 -0.0923474279803287

AGIWUR156;AGIWUR6021;AGIWUR13188;AGIWUR13370;AGIWUR19239;AGIWUR28819;AGIWUR37022;AGIWUR41612;AGIWUR41671
CB4856 CT X 8403539 8408367 WBGene00018283 sulp-3 F41D9.5 0.205047115916181 AGIWUR24297
CB4856 CT X 8411698 8414195 WBGene00017258 F08F1.4 F08F1.4 0.305867646067945
AGIWUR4950;AGIWUR6123;AGIWUR12253;AGIWUR14710;AGIWUR15054;AGIWUR17264;AGIWUR18514;AGIWUR30864;AGIWUR43115;AGIWUR45009
CB4856 CT X 8417424 8418524 WBGene00004998 spp-13 F08F1.6 0.231356384958305 AGIWUR2104
CB4856 CT X 8418646 8422055 WBGene00006472 tag-123 F08F1.7 -0.100342199805822
AGIWUR752;AGIWUR6564;AGIWUR12444;AGIWUR28314;AGIWUR34981;AGIWUR40903
CB4856 CT X 8422480 8423731 WBGene00006649 tth-1 F08F1.8 0.649076090745355 AGIWUR37804
CB4856 CT X 8432663 8437346 WBGene00002195 kin-9 F08F1.1 0.0971053387183893 AGIWUR36818
CB4856 CT X 8440192 8450417 WBGene00017513 F16F9.1 F16F9.1 0.28179600148388 AGIWUR30468
CB4856 CT X 8450381 8455640 WBGene00001068 dpy-6 F16F9.2 -0.55303876472226 AGIWUR35261
CB4856 CT X 8457614 8459734 WBGene00017514 F16F9.3 F16F9.3 -0.264410090046217 AGIWUR14547
CB4856 CT X 8459821 8463360 WBGene00017515 F16F9.4 F16F9.4 -0.2546109462125 AGIWUR23854
CB4856 CT X 8465832 8470256 WBGene00003174 mec-10 F16F9.5 0.132739491607468 AGIWUR42070
CB4856 CT X 8473723 8476176 WBGene00003613 nhr-14 T01B10.4 -0.0130366949064129
AGIWUR1892;AGIWUR2548;AGIWUR14751;AGIWUR15964;AGIWUR19124
CB4856 CT X 8495322 8498062 WBGene00020137 T01B10.5 T01B10.5 0.825917834222043
AGIWUR41525
CB4856 CT X 8499664 8501037 WBGene00001703 grd-14 T01B10.2 -0.628034820399314
AGIWUR39755
CB4856 CT X 8501245 8502289 WBGene00001693 grd-4 T01B10.1 -0.490170963600097
AGIWUR5512
CB4856 CT X 8507876 8514126 WBGene00019148 H03E18.1 H03E18.1 0.415764563088334
AGIWUR37696
CB4856 CT X 8514088 8517830 WBGene00019149 H03E18.2 H03E18.2 -0.178648370119168
AGIWUR17740
CB4856 CT X 8528250 8531033 WBGene00001448 flp-5 C03G5.7 0.272973432278638
AGIWUR1829;AGIWUR34234
CB4856 CT X 8542867 8546021 WBGene00015391 sdha-1 C03G5.1 -0.333153984552815
AGIWUR270;AGIWUR5327;AGIWUR26113;AGIWUR26614;AGIWUR30788
CB4856 CT X 8546451 8549056 WBGene00017048 D2021.4 D2021.4 0.060025486626655
AGIWUR6248;AGIWUR11716;AGIWUR20806
CB4856 CT X 8552261 8556351 WBGene00044071 dhhc-14 D2021.2 -0.0868272182506953
AGIWUR2179;AGIWUR13670;AGIWUR28378;AGIWUR41196
CB4856 CT X 8557151 8563235 WBGene00017046 utx-1 D2021.1 0.0888569613276573
AGIWUR4714;AGIWUR5935;AGIWUR18865;AGIWUR22140;AGIWUR25209;AGIWUR25406;AGIWUR35006
CB4856 CT X 8571049 8575451 WBGene00017571 jmjd-3.1 F18E9.5 0.768880699183722
AGIWUR38593

CB4856	CT	X	8578585 8580104 WBGene00044780	F18E9.8 F18E9.8 0.0322875567145449
AGIWUR1800;AGIWUR4650;AGIWUR9990
CB4856	CT	X	8580656 8583537 WBGene00017570	F18E9.4 F18E9.4 -0.426143528524897
AGIWUR12635;AGIWUR15548;AGIWUR40652
CB4856	CT	X	8586147 8590290 WBGene00017569	F18E9.3 F18E9.3 0.337330458584548	AGIWUR42934
CB4856	CT	X	8593187 8593974 WBGene00003745	nlp-7	F18E9.2 0.0977821361404274	AGIWUR44693
CB4856	CT	X	8595152 8597747 WBGene00017573	F18E9.7 F18E9.7 -0.405010818358692	AGIWUR25588
CB4856	CT	X	8601535 8602366 WBGene00017568	F18E9.1 F18E9.1 0.599217882592005	AGIWUR9199
CB4856	CT	X	8607174 8607753 WBGene00019265	H35N09.1	H35N09.1	-0.30105508282024
AGIWUR40490
CB4856	CT	X	8618418 8619756 WBGene00019286	K01A12.2	K01A12.2	-0.178970960580619
AGIWUR2797;AGIWUR18038;AGIWUR23292;AGIWUR32547;AGIWUR36214
CB4856	CT	X	8620570 8625240 WBGene00019287	K01A12.3	K01A12.3	0.0402419980662465
AGIWUR16423;AGIWUR36181;AGIWUR38635
CB4856	CT	X	8639389 8643431 WBGene00017400	stg-2	F12D9.1 0.000111858000277465	AGIWUR31307
CB4856	CT	X	8646606 8647097 WBGene00017401	F12D9.2 F12D9.2 -0.0463596753289686	AGIWUR2405
CB4856	CT	X	8660619 8671753 WBGene00004369	rig-1	K09E2.4 0.289308677983461
AGIWUR23760;AGIWUR27489;AGIWUR32360
CB4856	CT	X	8673060 8681653 WBGene00019571	K09E2.2 K09E2.2 0.291532009949059	AGIWUR36350
CB4856	CT	X	8681910 8686146 WBGene00019570	K09E2.1 K09E2.1 0.116270687986694
AGIWUR13311;AGIWUR34731;AGIWUR43898
CB4856	CT	X	8697817 8702822 WBGene00018728	frpr-8	F53A9.5 0.137472209405978
AGIWUR1563;AGIWUR37929
CB4856	CT	X	8704309 8707197 WBGene00018727	jbts-14 F53A9.4 0.546974377185866	AGIWUR39170
CB4856	CT	X	8708365 8709370 WBGene00018726	F53A9.3 F53A9.3 0.0852736175233915	AGIWUR22905
CB4856	CT	X	8709764 8710137 WBGene00018725	kreg-1	F53A9.2 0.549825499088299
AGIWUR5464;AGIWUR7328;AGIWUR7798;AGIWUR15741;AGIWUR20454;AGIWUR30988
CB4856	CT	X	8711590 8711911 WBGene00018724	F53A9.1 F53A9.1 1.06602721022648
AGIWUR32378;AGIWUR38157
CB4856	CT	X	8713461 8713952 WBGene00018729	F53A9.6 F53A9.6 1.50455049809543	AGIWUR26199
CB4856	CT	X	8716532 8716929 WBGene00018730	F53A9.7 F53A9.7 1.40639834595966	AGIWUR11432
CB4856	CT	X	8718567 8718905 WBGene00018731	F53A9.8 F53A9.8 3.60319709928601	AGIWUR35395
CB4856	CT	X	8720226 8720758 WBGene00018732	F53A9.9 F53A9.9 0.442832413215816	AGIWUR40805
CB4856	CT	X	8721557 8723552 WBGene00006587	tnt-2	F53A9.10	0.0169215820100703
AGIWUR10214;AGIWUR15235;AGIWUR16730;AGIWUR22902;AGIWUR39188;AGIWUR39856;AGIWUR41250
CB4856	CT	X	8726798 8727406 WBGene00017149	EGAP4.1 EGAP4.1 -0.343339134383774	AGIWUR37219
CB4856	CT	X	8731310 8731922 WBGene00019732	M02D8.6 M02D8.6 0.558391924199881	AGIWUR42632
CB4856	CT	X	8732958 8737015 WBGene00019729	M02D8.3 M02D8.3 -0.0357623266781442	AGIWUR43043
CB4856	CT	X	8739048 8740171 WBGene00019728	M02D8.2 M02D8.2 -0.135282873109805

AGIWUR13616;AGIWUR23732;AGIWUR26248;AGIWUR30537;AGIWUR39903
CB4856 CT X 8740789 8743057 WBGene00044313 M02D8.7 M02D8.7 0.259593550891409
AGIWUR5408;AGIWUR35449
CB4856 CT X 8748434 8750969 WBGene00019730 asns-2 M02D8.4 0.283564053563436
AGIWUR19162;AGIWUR20337;AGIWUR21929;AGIWUR23261
CB4856 CT X 8754576 8761580 WBGene00019731 M02D8.5 M02D8.5 -0.459770521403245 AGIWUR16000
CB4856 CT X 8763907 8766280 WBGene00019727 zig-12 M02D8.1 -0.342863437531907 AGIWUR26789
CB4856 CT X 8778847 8784441 WBGene00022811 ZK721.4 ZK721.4 0.0526324787126171 AGIWUR21245
CB4856 CT X 8784534 8787072 WBGene00022810 ZK721.3 ZK721.3 -0.123633760506513 AGIWUR959
CB4856 CT X 8789456 8790621 WBGene00006764 unc-27 ZK721.2 0.13398685966078
AGIWUR8777;AGIWUR17569
CB4856 CT X 8792160 8796110 WBGene00006477 chup-1 ZK721.1 -0.165046832836112
AGIWUR29227;AGIWUR32479
CB4856 CT X 8796545 8797835 WBGene00019870 R04E5.7 R04E5.7 0.115919728870746
AGIWUR14421;AGIWUR36332;AGIWUR37318;AGIWUR40252;AGIWUR40895
CB4856 CT X 8801624 8806113 WBGene00019871 R04E5.8 R04E5.8 0.0473086843037716
AGIWUR27571;AGIWUR40800
CB4856 CT X 8806174 8811142 WBGene00019872 R04E5.9 R04E5.9 0.193514619821903
AGIWUR7613;AGIWUR8776;AGIWUR11944;AGIWUR16588;AGIWUR24497;AGIWUR37816
CB4856 CT X 8812143 8816805 WBGene00019869 cnnm-2 R04E5.2 -0.254607714889998 AGIWUR22058
CB4856 CT X 8820703 8824448 WBGene00002057 ifd-1 R04E5.10 -0.126106722921137
AGIWUR7275;AGIWUR21127
CB4856 CT X 8826627 8828540 WBGene00016190 C28G1.5 C28G1.5 -0.335667919645509
AGIWUR27051;AGIWUR43751
CB4856 CT X 8829920 8831262 WBGene00016191 C28G1.6 C28G1.6 0.397869347807593 AGIWUR24667
CB4856 CT X 8831739 8835715 WBGene00016188 sec-15 C28G1.3 -0.0348902984018262
AGIWUR2654;AGIWUR4425
CB4856 CT X 8836644 8837661 WBGene00016187 C28G1.2 C28G1.2 0.489269117749791
AGIWUR6456;AGIWUR30481
CB4856 CT X 8847122 8848482 WBGene00219376 C28G1.10 C28G1.10 0.282255174640241
AGIWUR42745
CB4856 CT X 8849137 8853621 WBGene00016189 C28G1.4 C28G1.4 0.0154366090091571 AGIWUR32459
CB4856 CT X 8857056 8857954 WBGene00015529 C06E2.5 C06E2.5 0.0912069964717652 AGIWUR40648
CB4856 CT X 8861258 8861650 WBGene00044579 C06E2.9 C06E2.9 -0.181711347111615
AGIWUR7776;AGIWUR8306;AGIWUR12019;AGIWUR20105;AGIWUR24625
CB4856 CT X 8866238 8866640 WBGene00002092 ins-9 C06E2.8 -0.455841602087396
AGIWUR17847;AGIWUR18385;AGIWUR22537;AGIWUR26370;AGIWUR28244
CB4856 CT X 8867603 8869153 WBGene00006717 ubc-22 C06E2.7 0.0573833865209012 AGIWUR8878
CB4856 CT X 8869625 8871901 WBGene00006716 ubc-21 C06E2.3 -0.0178060683789036

AGIWUR2345;AGIWUR14287;AGIWUR40115
CB4856	CT	X	8872933 8874848 WBGene00015527	C06E2.1 C06E2.1 -0.299124850921764	AGIWUR42187
CB4856	CT	X	8879649 8880406 WBGene00015528	C06E2.2 C06E2.2 -0.347735573407985	AGIWUR44949
CB4856	CT	X	8893129 8898517 WBGene00015740	C13E3.1 C13E3.1 -0.0281552144472821	AGIWUR22911
CB4856	CT	X	8908050 8910837 WBGene00017013	ensh-1	D1009.3 0.2783494924998 AGIWUR6361
CB4856	CT	X	8911293 8914261 WBGene00000884	cyn-8	D1009.2 0.171985760682767
AGIWUR7443;AGIWUR10061;AGIWUR35701;AGIWUR36094;AGIWUR39431;AGIWUR39528
CB4856	CT	X	8917421 8919423 WBGene00003752	nlp-14	D1009.4 0.302264386815615	AGIWUR2900
CB4856	CT	X	8931241 8939263 WBGene00017012	acs-22	D1009.1 0.214292160013919
AGIWUR4766;AGIWUR6135;AGIWUR9124;AGIWUR27367;AGIWUR32376
CB4856	CT	X	8939389 8940143 WBGene00017014	dylt-2	D1009.5 0.513404667072923	AGIWUR21964
CB4856	CT	X	8941139 8950978 WBGene00017039	trk-1	D1073.1 0.234258010929662
AGIWUR14010;AGIWUR16072;AGIWUR30714;AGIWUR32171
CB4856	CT	X	8957616 8961170 WBGene00016748	aexr-3	C48C5.3 -0.0770672900306453	AGIWUR21382
CB4856	CT	X	8980976 8985063 WBGene00016747	nmur-1	C48C5.1 0.0750782683540159
AGIWUR14619;AGIWUR16262;AGIWUR27846
CB4856	CT	X	8988137 8991972 WBGene00006672	twk-18	C24A3.6 -0.0288378989153824	AGIWUR6794
CB4856	CT	X	8992406 8994763 WBGene00016034	C24A3.4 C24A3.4 0.00721273864296457
AGIWUR3347;AGIWUR9135;AGIWUR17732;AGIWUR32265;AGIWUR35548;AGIWUR41716
CB4856	CT	X	8997479 8998909 WBGene00016033	C24A3.2 C24A3.2 0.0921927942669178
AGIWUR13952;AGIWUR26014;AGIWUR28903
CB4856	CT	X	9001239 9006053 WBGene00016032	C24A3.1 C24A3.1 0.124915548126749
AGIWUR17679;AGIWUR40654
CB4856	CT	X	9009780 9010457 WBGene00044580	C24A3.9 C24A3.9 0.0387989132189182
AGIWUR12458;AGIWUR20948;AGIWUR35053;AGIWUR43393
CB4856	CT	X	9015773 9016799 WBGene00020790	T25B6.4 T25B6.4 -0.17513809079466	AGIWUR26573
CB4856	CT	X	9020588 9021755 WBGene00020791	T25B6.5 T25B6.5 0.182933191804919	AGIWUR13577
CB4856	CT	X	9021922 9022833 WBGene00020792	T25B6.6 T25B6.6 -0.124120223100302	AGIWUR27292
CB4856	CT	X	9027604 9028285 WBGene00020789	T25B6.3 T25B6.3 -0.0974313123020343	AGIWUR28443
CB4856	CT	X	9029478 9033955 WBGene00004911	snf-12	T25B6.7 0.530643176372356	AGIWUR10581
CB4856	CT	X	9034554 9038547 WBGene00020788	nep-22	T25B6.2 -0.275195809511772
AGIWUR30322;AGIWUR36231
CB4856	CT	X	9040416 9041058 WBGene00020787	T25B6.1 T25B6.1 0.183071680935449	AGIWUR13768
CB4856	CT	X	9046213 9054885 WBGene00018567	F47E1.3 F47E1.3 0.251810422502217	AGIWUR11460
CB4856	CT	X	9057652 9061860 WBGene00018566	F47E1.2 F47E1.2 0.0320535639998631
AGIWUR23020;AGIWUR28657;AGIWUR33267;AGIWUR44410
CB4856	CT	X	9062834 9066591 WBGene00018568	F47E1.4 F47E1.4 1.05379627098007	AGIWUR17318
CB4856	CT	X	9073800 9077724 WBGene00018565	F47E1.1 F47E1.1 0.492827377759743	AGIWUR3858
CB4856	CT	X	9078945 9113613 WBGene00000100	ajm-1	C25A11.4	0.0731332597658625

AGIWUR1659;AGIWUR2586;AGIWUR3751;AGIWUR5431;AGIWUR5755;AGIWUR10369;AGIWUR18886;AGIWUR19046;AGIWUR28888;AGIWUR42397;AGIWUR43790
CB4856 CT X 9114368 9116891 WBGene00016087 C25A11.2 C25A11.2 -0.229235387858697 AGIWUR42121
CB4856 CT X 9119774 9121687 WBGene00016086 C25A11.1 C25A11.1 -0.190418013376313 AGIWUR3468;AGIWUR28257;AGIWUR32811
CB4856 CT X 9121884 9123505 WBGene00219374 C25A11.10 C25A11.10 -0.00875964731041101 AGIWUR17757;AGIWUR19421;AGIWUR20739;AGIWUR22486
CB4856 CT X 9137927 9145656 WBGene00015263 B0563.6 B0563.6 -0.109544115136235 AGIWUR6329;AGIWUR10984;AGIWUR13642;AGIWUR15404;AGIWUR20913;AGIWUR21716;AGIWUR24233;AGIWUR38920;AGIWUR40946
CB4856 CT X 9146174 9147526 WBGene00015262 B0563.5 B0563.5 0.612440153817102 AGIWUR16724;AGIWUR26839;AGIWUR28523;AGIWUR29996;AGIWUR40814
CB4856 CT X 9150694 9160646 WBGene00006479 tmbi-4 B0563.4 -0.0102692637605948 AGIWUR35222
CB4856 CT X 9163164 9166368 WBGene00006637 tsp-11 B0563.2 0.0846750635806188 AGIWUR39443
CB4856 CT X 9168539 9169022 WBGene00044687 B0563.9 B0563.9 0.221438121670039 AGIWUR1912;AGIWUR5988;AGIWUR17520;AGIWUR27703;AGIWUR42134
CB4856 CT X 9171562 9172204 WBGene00015261 B0563.1 B0563.1 -0.140054425614361 AGIWUR20274
CB4856 CT X 9173685 9174490 WBGene00015265 B0563.8 B0563.8 -0.627225938402756 AGIWUR20718
CB4856 CT X 9180062 9180923 WBGene00044581 Y81B9A.4 Y81B9A.4 0.0912298421257782 AGIWUR2260;AGIWUR26510;AGIWUR39823
CB4856 CT X 9182440 9182947 WBGene00022317 Y81B9A.3 Y81B9A.3 -0.0379049163911569 AGIWUR13980;AGIWUR14706;AGIWUR18027;AGIWUR19056;AGIWUR43732
CB4856 CT X 9184163 9190717 WBGene00022316 Y81B9A.2 Y81B9A.2 -0.0377936352646886 AGIWUR4160
CB4856 CT X 9192444 9194382 WBGene00022315 Y81B9A.1 Y81B9A.1 0.173901158294277 AGIWUR1439
CB4856 CT X 9211144 9212321 WBGene00044743 crb-3 C35B8.4 -0.0857152334970817 AGIWUR10376;AGIWUR17275;AGIWUR17825;AGIWUR28825;AGIWUR37521
CB4856 CT X 9213953 9216856 WBGene00016438 C35B8.3 C35B8.3 0.0930577175012459 AGIWUR18703
CB4856 CT X 9216867 9232075 WBGene00006887 vav-1 C35B8.2 0.16689896785757 AGIWUR455;AGIWUR11041;AGIWUR31927
CB4856 CT X 9233638 9234618 WBGene00000748 col-175 C35B8.1 -0.128831989859744 AGIWUR29914
CB4856 CT X 9235103 9242223 WBGene00017580 lgc-4 F18G5.4 -0.293044608316912 AGIWUR4317
CB4856 CT X 9247007 9249103 WBGene00001674 gpa-12 F18G5.3 -0.479683125412103 AGIWUR24993
CB4856 CT X 9251818 9255717 WBGene00003981 pes-8 F18G5.2 -0.492614743025165 AGIWUR23852
CB4856 CT X 9256008 9257456 WBGene00017581 F18G5.5 F18G5.5 -0.171972101965587 AGIWUR27652
CB4856 CT X 9259852 9261066 WBGene00017579 F18G5.1 F18G5.1 -0.57912610969709 AGIWUR40631
CB4856 CT X 9262027 9264331 WBGene00017582 F18G5.6 F18G5.6 1.35357551988321 AGIWUR791;AGIWUR2708;AGIWUR11787;AGIWUR38307

CB4856 CT   X   9268626 9270855 WBGene00015181 B0416.5 B0416.5 -0.027197956066856
AGIWUR2846;AGIWUR16098;AGIWUR34193;AGIWUR40626
CB4856 CT   X   9278953 9279947 WBGene00015180 B0416.4 B0416.4 -0.305141493286246
AGIWUR25648;AGIWUR36314;AGIWUR39520
CB4856 CT   X   9291651 9293366 WBGene00015179 B0416.3 B0416.3 -0.10210566307989   AGIWUR3498
CB4856 CT   X   9293664 9296622 WBGene00001638 gly-13 B0416.6 -0.384851861610708   AGIWUR12912
CB4856 CT   X   9296947 9297934 WBGene00015182 B0416.7 B0416.7 -0.0874918859246758
AGIWUR5443;AGIWUR7853;AGIWUR15838;AGIWUR18976;AGIWUR19220;AGIWUR22464;AGIWUR31115;AGIWUR31167;AGIWUR35787;A
GIWUR44988
CB4856 CT   X   9299147 9300131 WBGene00015178 B0416.2 B0416.2 -0.0561006009272864   AGIWUR43083
CB4856 CT   X   9303724 9310439 WBGene00015177 tmc-2   B0416.1 -0.089442457929934   AGIWUR21090
CB4856 CT   X   9310625 9311341 WBGene00045261 H29C22.1   H29C22.1   0.142066140420129
AGIWUR12636;AGIWUR32509
CB4856 CT   X   9312648 9314488 WBGene00044788 ttr-38 F35G8.2 -0.771243660259186
AGIWUR1546;AGIWUR7857;AGIWUR16234;AGIWUR24607
CB4856 CT   X   9315436 9319180 WBGene00018067 npr-7   F35G8.1 -0.0148160163849429   AGIWUR12994
CB4856 CT   X   9331133 9336632 WBGene00020595 T20B5.2 T20B5.2 -0.0910303190186355   AGIWUR5555
CB4856 CT   X   9336819 9342970 WBGene00000161 apa-2   T20B5.1 -0.181824138633766
AGIWUR7529;AGIWUR19739;AGIWUR28855;AGIWUR36115
CB4856 CT   X   9343333 9347430 WBGene00020596 oga-1   T20B5.3 0.148469167388842
AGIWUR5115;AGIWUR11833;AGIWUR13581;AGIWUR24006;AGIWUR40314;AGIWUR45209
CB4856 CT   X   9349276 9349890 WBGene00001463 flp-20 E01H11.3   0.128123555861901
AGIWUR17940
CB4856 CT   X   9364818 9386035 WBGene00004033 pkc-2   E01H11.1   0.000178484414626201
AGIWUR4394;AGIWUR5483;AGIWUR5665;AGIWUR8696;AGIWUR9373;AGIWUR10115;AGIWUR11392;AGIWUR16641;AGIWUR18117;AGIW
UR27326;AGIWUR30526;AGIWUR33176;AGIWUR34297;AGIWUR34365;AGIWUR35659;AGIWUR38093;AGIWUR40292;AGIWUR44936
CB4856 CT   X   9387341 9389189 WBGene00016009 C23F12.4   C23F12.4   -0.0672480102158826
AGIWUR5651
CB4856 CT   X   9391645 9394702 WBGene00016008 C23F12.3   C23F12.3   0.113571016544205
AGIWUR22232
CB4856 CT   X   9397701 9427246 WBGene00016006 fln-2   C23F12.1   0.0768141192360901
AGIWUR2768;AGIWUR3524;AGIWUR4234;AGIWUR6327;AGIWUR11527;AGIWUR13067;AGIWUR19340;AGIWUR21140;AGIWUR21986;AGI
WUR27693;AGIWUR28234;AGIWUR33002;AGIWUR33525;AGIWUR36069;AGIWUR40435
CB4856 CT   X   9428431 9430424 WBGene00007130 B0272.4 B0272.4 0.141699207045736   AGIWUR26263
CB4856 CT   X   9431432 9432745 WBGene00007129 B0272.3 B0272.3 -0.0602739333882845   AGIWUR18153
CB4856 CT   X   9434452 9436893 WBGene00006538 tbb-4   B0272.1 0.304278625026956   AGIWUR39009
CB4856 CT   X   9440079 9441571 WBGene00007128 memb-1 B0272.2 -0.319956117787768   AGIWUR29736
CB4856 CT   X   9446664 9447612 WBGene00014140 ZK899.1 ZK899.1 -0.0181370682483148   AGIWUR2353
CB4856 CT   X   9447861 9449399 WBGene00014145 ZK899.7 ZK899.7 -0.0831137003540068

AGIWUR17130;AGIWUR28669;AGIWUR36863
CB4856	CT	X	9450225 9451994 WBGene00014141	ZK899.2 ZK899.2 -0.133831823946457	AGIWUR13325
CB4856	CT	X	9453959 9454660 WBGene00014142	ZK899.3 ZK899.3 -0.164402245410604	AGIWUR1908
CB4856	CT	X	9455485 9458348 WBGene00006534	tba-8	ZK899.4 -0.0343712952696249	AGIWUR42441
CB4856	CT	X	9459911 9460934 WBGene00014143	ZK899.5 ZK899.5 -0.0158928707996067	AGIWUR26787
CB4856	CT	X	9463356 9464725 WBGene00014144	ZK899.6 ZK899.6 -0.129259063139635	AGIWUR21764
CB4856	CT	X	9472469 9516440 WBGene00001516	gap-2	ZK899.8 0.0291461059422922
AGIWUR1390;AGIWUR6256;AGIWUR8578;AGIWUR9282;AGIWUR9686;AGIWUR16619;AGIWUR19702;AGIWUR20064;AGIWUR20762;AGIWUR20894;AGIWUR23198;AGIWUR23501;AGIWUR24911;AGIWUR25718;AGIWUR26474;AGIWUR27862;AGIWUR29312;AGIWUR30264;AGIWUR32349;AGIWUR35911;AGIWUR40375
CB4856	CT	X	9516393 9517187 WBGene00007407	C07B5.2 C07B5.2 -0.367655988705061	AGIWUR35949
CB4856	CT	X	9519022 9521486 WBGene00007410	C07B5.6 C07B5.6 0.0969840683434793	AGIWUR16034
CB4856	CT	X	9522488 9523173 WBGene00007408	C07B5.3 C07B5.3 0.451061647637728	AGIWUR31308
CB4856	CT	X	9529533 9536631 WBGene00007409	C07B5.4 C07B5.4 0.499503125952676
AGIWUR3621;AGIWUR11381;AGIWUR27991;AGIWUR28114;AGIWUR31629;AGIWUR35020;AGIWUR36150;AGIWUR43450
CB4856	CT	X	9539905 9542430 WBGene00003828	nuc-1	C07B5.5 0.30925283214761
AGIWUR8544;AGIWUR19630;AGIWUR27016
CB4856	CT	X	9543670 9560801 WBGene00009888	F49E2.5 F49E2.5 0.193747656846206
AGIWUR1096;AGIWUR1611;AGIWUR2644;AGIWUR2838;AGIWUR7511;AGIWUR8364;AGIWUR10828;AGIWUR14219;AGIWUR16672;AGIWUR19009;AGIWUR21819;AGIWUR23167;AGIWUR25326;AGIWUR29705;AGIWUR30817;AGIWUR35031;AGIWUR35540;AGIWUR35753;AGIWUR35876;AGIWUR42913
CB4856	CT	X	9561850 9565981 WBGene00009885	F49E2.1 F49E2.1 -0.250732698398178
AGIWUR6116;AGIWUR6559;AGIWUR10854;AGIWUR20850;AGIWUR30883;AGIWUR39455;AGIWUR42055
CB4856	CT	X	9567296 9573723 WBGene00009886	F49E2.2 F49E2.2 0.0907766606252505	AGIWUR6164
CB4856	CT	X	9573741 9575866 WBGene00009887	glb-17	F49E2.4 -0.115357067138488
AGIWUR11201;AGIWUR15486;AGIWUR23651;AGIWUR27022;AGIWUR34920;AGIWUR45030
CB4856	CT	X	9584664 9586344 WBGene00007516	gpx-5	C11E4.1 -0.232830537920104
AGIWUR26679;AGIWUR40806
CB4856	CT	X	9587942 9588879 WBGene00007517	gpx-3	C11E4.2 -0.044468964798817
AGIWUR13307;AGIWUR43368
CB4856	CT	X	9589423 9595459 WBGene00007518	del-8	C11E4.3 -0.0619402078190827	AGIWUR15310
CB4856	CT	X	9595770 9595902 WBGene00201289	C11E4.14	C11E4.14
-0.00507801068892988	AGIWUR2498
CB4856	CT	X	9610563 9617045 WBGene00007520	C11E4.6 C11E4.6 0.0956075983342011	AGIWUR27647
CB4856	CT	X	9617219 9619122 WBGene00007521	C11E4.7 C11E4.7 -0.245365120232306	AGIWUR9290
CB4856	CT	X	9623304 9624714 WBGene00007522	C11E4.8 C11E4.8 0.103986174976495	AGIWUR4440
CB4856	CT	X	9626135 9626849 WBGene00009378	F34H10.1	F34H10.1	0.362750966981875
AGIWUR24854
CB4856	CT	X	9630423 9630875 WBGene00009379	F34H10.2	F34H10.2	0.446964235286561

AGIWUR32767
CB4856 CT X 9633284 9635647 WBGene00009380 F34H10.3 F34H10.3 0.312337872016624
AGIWUR9257
CB4856 CT X 9635693 9636884 WBGene00009382 F34H10.5 F34H10.5 -0.74926549615791
AGIWUR44097
CB4856 CT X 9648532 9650928 WBGene00009381 F34H10.4 F34H10.4 -0.0795599354528971
AGIWUR38929
CB4856 CT X 9650999 9653116 WBGene00000085 aex-2 T14B1.2 0.0434450336042991 AGIWUR10230
CB4856 CT X 9664499 9668755 WBGene00011763 T14B1.1 T14B1.1 0.851430013286885
AGIWUR14041;AGIWUR17060;AGIWUR21205;AGIWUR41237
CB4856 CT X 9675844 9680169 WBGene00002067 ifp-1 C43C3.1 0.0113100527699457
AGIWUR39748;AGIWUR42431
CB4856 CT X 9690745 9696718 WBGene00008065 npr-18 C43C3.2 0.189490753184496 AGIWUR12539
CB4856 CT X 9698678 9701679 WBGene00001123 dyf-7 C43C3.3 0.430290163133907
AGIWUR6186;AGIWUR13488;AGIWUR37978;AGIWUR40328
CB4856 CT X 9706926 9708563 WBGene00008865 F15G9.1 F15G9.1 0.209713396452623
AGIWUR2786;AGIWUR18819;AGIWUR20324;AGIWUR23067;AGIWUR27181;AGIWUR28533;AGIWUR42907;AGIWUR43564
CB4856 CT X 9708590 9713713 WBGene00008866 F15G9.2 F15G9.2 -0.121357471703923 AGIWUR38649
CB4856 CT X 9717589 9753764 WBGene00001863 him-4 F15G9.4 -0.243627255599833 AGIWUR31653
CB4856 CT X 9757271 9759306 WBGene00011642 T09B9.1 T09B9.1 -0.149235123083172
AGIWUR27426;AGIWUR32169
CB4856 CT X 9759297 9761281 WBGene00011644 T09B9.3 T09B9.3 0.226762938985708 AGIWUR17627
CB4856 CT X 9761483 9764922 WBGene00011643 slc-17.9 T09B9.2 0.363235485303223
AGIWUR19015
CB4856 CT X 9771697 9774352 WBGene00011645 T09B9.4 T09B9.4 -0.178849662472157 AGIWUR6173
CB4856 CT X 9777831 9780251 WBGene00011646 T09B9.5 T09B9.5 0.0221906169628125 AGIWUR31885
CB4856 CT X 9780375 9789275 WBGene00009482 F36G3.1 F36G3.1 -0.154283586751708
AGIWUR802;AGIWUR15540;AGIWUR16093;AGIWUR37210;AGIWUR42245;AGIWUR44981
CB4856 CT X 9792407 9794273 WBGene00009483 F36G3.2 F36G3.2 0.115285356914159
AGIWUR11079;AGIWUR15033;AGIWUR31455
CB4856 CT X 9798839 9801038 WBGene00009484 F36G3.3 F36G3.3 0.0360513366217337 AGIWUR26906
CB4856 CT X 9801091 9807662 WBGene00009799 lgc-47 F47A4.1 0.137428070775723
AGIWUR528;AGIWUR6353;AGIWUR10171;AGIWUR29701
CB4856 CT X 9810202 9822273 WBGene00001081 dpy-22 F47A4.2 0.156689862165504 AGIWUR4384
CB4856 CT X 9823752 9831249 WBGene00009800 rrc-1 F47A4.3 0.392610529851771 AGIWUR44440
CB4856 CT X 9833352 9838039 WBGene00009801 ipla-2 F47A4.5 0.233462988630404 AGIWUR8383
CB4856 CT X 9847275 9849518 WBGene00006881 vab-15 R07B1.1 0.15204584871666 AGIWUR21372
CB4856 CT X 9850209 9851375 WBGene00002271 lec-8 R07B1.10 0.0148780500942518
AGIWUR25560

CB4856 CT X 9852139 9852909 WBGene00002270 lec-7 R07B1.2 0.442235454466596
AGIWUR5220;AGIWUR7200;AGIWUR12362;AGIWUR21935;AGIWUR22868;AGIWUR25023;AGIWUR25252;AGIWUR25319;AGIWUR27786;AGIWUR29709;AGIWUR37997;AGIWUR39507;AGIWUR43141;AGIWUR43727
CB4856 CT X 9856918 9858083 WBGene00011082 R07B1.11 R07B1.11 0.0258093016879997
AGIWUR37912
CB4856 CT X 9858339 9861735 WBGene00011076 scav-5 R07B1.3 -0.385876287776727 AGIWUR27332
CB4856 CT X 9861646 9862960 WBGene00001784 gst-36 R07B1.4 -0.669187911732845 AGIWUR21795
CB4856 CT X 9864069 9866531 WBGene00011077 R07B1.5 R07B1.5 0.138011306235247 AGIWUR7300
CB4856 CT X 9866660 9867921 WBGene00011078 R07B1.6 R07B1.6 0.197624041025177 AGIWUR39602
CB4856 CT X 9869836 9870792 WBGene00138711 R07B1.13 R07B1.13 -0.128102175730483
AGIWUR34623
CB4856 CT X 9870804 9872654 WBGene00001608 R07B1.8 R07B1.8 -0.196153200565075 AGIWUR28705
CB4856 CT X 9872884 9882212 WBGene00011083 glo-1 R07B1.12 -0.092917016722712
AGIWUR13929;AGIWUR23649;AGIWUR27945
CB4856 CT X 9884278 9889704 WBGene00003410 mrp-4 F21G4.2 -0.324651034241349 AGIWUR18807
CB4856 CT X 9889618 9892328 WBGene00009023 F21G4.1 F21G4.1 0.16953063217367
AGIWUR23391;AGIWUR33969
CB4856 CT X 9895144 9897057 WBGene00009024 F21G4.3 F21G4.3 0.00488261755843844 AGIWUR28779
CB4856 CT X 9897313 9899476 WBGene00009025 phf-34 F21G4.4 -0.184908320181671 AGIWUR3666
CB4856 CT X 9906148 9908116 WBGene00009026 F21G4.5 F21G4.5 -0.0336507760450253 AGIWUR38645
CB4856 CT X 9908794 9917448 WBGene00009027 F21G4.6 F21G4.6 -0.210059973555573 AGIWUR1469
CB4856 CT X 9919302 9920346 WBGene00007648 folr-1 C17G1.1 -0.574953856611092 AGIWUR26776
CB4856 CT X 9921557 9922843 WBGene00007649 C17G1.2 C17G1.2 0.0585133830839535 AGIWUR25661
CB4856 CT X 9922910 9925173 WBGene00007650 ugt-23 C17G1.3 0.0579642310169888 AGIWUR28073
CB4856 CT X 9925728 9931460 WBGene00007651 nra-3 C17G1.4 0.18790091911265 AGIWUR9330
CB4856 CT X 9932371 9937133 WBGene00023491 tsp-21 C17G1.8 -0.0506482111254858 AGIWUR15686
CB4856 CT X 9941630 9944838 WBGene00007652 C17G1.5 C17G1.5 0.10575916839687
AGIWUR28795;AGIWUR36756
CB4856 CT X 9944844 9949060 WBGene00003553 nas-37 C17G1.6 0.242117748914882 AGIWUR4277
CB4856 CT X 9952579 9954533 WBGene00007653 cysl-1 C17G1.7 0.295237088707278 AGIWUR28040
CB4856 CT X 9958899 9964747 WBGene00013919 ZC506.1 ZC506.1 -0.0218187719006937
AGIWUR8213;AGIWUR11908;AGIWUR12251;AGIWUR17485;AGIWUR19519;AGIWUR24133;AGIWUR24710;AGIWUR28753;AGIWUR35520;AGIWUR35766
CB4856 CT X 9965495 9968584 WBGene00013920 pssy-1 ZC506.3 0.00657811120838048 AGIWUR5773
CB4856 CT X 9968517 9976392 WBGene00003232 mgl-1 ZC506.4 -0.144295152015356 AGIWUR6179
CB4856 CT X 9982402 9985005 WBGene00008954 F19C6.5 F19C6.5 -0.343220341151904 AGIWUR12405
CB4856 CT X 9991255 9999178 WBGene00001708 grk-1 F19C6.1 -0.161264504581878
AGIWUR3423;AGIWUR6594;AGIWUR9252;AGIWUR19484;AGIWUR28775;AGIWUR30119;AGIWUR30987;AGIWUR31580;AGIWUR34424;AGIWUR36707

CB4856	CT	X	10000935	10005408	WBGene00008951	F19C6.2	F19C6.2	0.0128985004225628	AGIWUR14774;AGIWUR18317;AGIWUR21208;AGIWUR23874;AGIWUR24789;AGIWUR25351
CB4856	CT	X	10005733	10008755	WBGene00008952	F19C6.3	F19C6.3	0.205419212978846	AGIWUR9397
CB4856	CT	X	10015045	10018025	WBGene00008953	F19C6.4	F19C6.4	-0.369031912544344	AGIWUR11952;AGIWUR20470;AGIWUR31977
CB4856	CT	X	10018046	10022304	WBGene00009645	F42G10.1	F42G10.1	-0.348361238079569	AGIWUR7303
CB4856	CT	X	10027856	10030130	WBGene00003368	mkk-4	F42G10.2	-0.22715923306249	AGIWUR7431;AGIWUR19589
CB4856	CT	X	10031088	10033283	WBGene00012162	sek-6	VZC374L.1	0.166822235331509	AGIWUR30961
CB4856	CT	X	10034836	10047417	WBGene00013872	ZC374.2	ZC374.2	-0.0147033678369386	AGIWUR28487
CB4856	CT	X	10049460	10052481	WBGene00013871	gnrr-3	ZC374.1	0.198392445967988	AGIWUR25668
CB4856	CT	X	10054281	10057524	WBGene00013866	cbs-1	ZC373.1	-0.169810867409234	AGIWUR2325
CB4856	CT	X	10057925	10058437	WBGene00013867	ZC373.2	ZC373.2	0.0404580812638429	AGIWUR24519
CB4856	CT	X	10060525	10061681	WBGene00013868	ZC373.3	ZC373.3	0.0290154572908499	AGIWUR8982
CB4856	CT	X	10062451	10069783	WBGene00013869	mlck-1	ZC373.4	-0.145885766968084	AGIWUR28216
CB4856	CT	X	10072507	10074391	WBGene00013870	ZC373.5	ZC373.5	-0.113132336870399	AGIWUR835;AGIWUR27101;AGIWUR42518;AGIWUR42861
CB4856	CT	X	10075288	10076357	WBGene00000930	dao-4	ZC373.6	-0.255464830124819	AGIWUR28509
CB4856	CT	X	10084627	10104252	WBGene00012326	W07E11.1	W07E11.1	0.49913034567129	AGIWUR4175;AGIWUR11508
CB4856	CT	X	10104612	10125483	WBGene00006792	unc-58	T06H11.1	-0.138550691717297	AGIWUR26360
CB4856	CT	X	10129588	10130267	WBGene00011552	T06H11.2	T06H11.2	-0.0584306757723168	AGIWUR38332
CB4856	CT	X	10136129	10138620	WBGene00003384	moc-1	T06H11.4	-0.155541470255859	AGIWUR25632
CB4856	CT	X	10139374	10142013	WBGene00008602	oac-14	F09B9.1	0.844065325104823	AGIWUR6547;AGIWUR23375;AGIWUR30796;AGIWUR39301
CB4856	CT	X	10146889	10153142	WBGene00006839	unc-115	F09B9.2	-0.0230927534083447	

AGIWUR531;AGIWUR1683;AGIWUR2406;AGIWUR8737;AGIWUR14699;AGIWUR17750;AGIWUR18007;AGIWUR32621;AGIWUR35554;AGIWUR37555;AGIWUR37856;AGIWUR38833
CB4856 CT X 10154821 10156856 WBGene00008604 F09B9.5 F09B9.5 -0.213795032132801
AGIWUR13866;AGIWUR15513;AGIWUR24575
CB4856 CT X 10156858 10160438 WBGene00001331 erd-2 F09B9.3 0.158780297660098
AGIWUR12862
CB4856 CT X 10160631 10161809 WBGene00008603 F09B9.4 F09B9.4 0.0389595077164131
AGIWUR18927
CB4856 CT X 10167096 10171668 WBGene00007396 tiar-3 C07A4.1 -0.499872948470788
AGIWUR7064
CB4856 CT X 10172326 10174185 WBGene00007397 C07A4.2 C07A4.2 0.312794867719516
AGIWUR12417
CB4856 CT X 10174985 10175896 WBGene00007398 C07A4.3 C07A4.3 0.916792908399001
AGIWUR13464
CB4856 CT X 10177237 10180662 WBGene00008998 ncs-4 F21A10.1
-0.622375743773618 AGIWUR31188
CB4856 CT X 10180953 10194377 WBGene00008999 myrf-2 F21A10.2
0.174922380953741 AGIWUR8406;AGIWUR20315;AGIWUR22227;AGIWUR36013;AGIWUR42903;AGIWUR43892
CB4856 CT X 10197706 10200407 WBGene00009674 nucb-1 F44A6.1 -0.423869984950114
AGIWUR17889;AGIWUR21581
CB4856 CT X 10201329 10205433 WBGene00004786 sex-1 F44A6.2 0.252857587560689
AGIWUR14694;AGIWUR24896;AGIWUR30249
CB4856 CT X 10212771 10214165 WBGene00009675 F44A6.3 F44A6.3 -0.151529209596294
AGIWUR25082
CB4856 CT X 10214443 10215495 WBGene00009676 F44A6.4 F44A6.4 -0.0309104957231057
AGIWUR34767
CB4856 CT X 10217310 10218760 WBGene00009677 F44A6.5 F44A6.5 -0.228252812392101
AGIWUR29850
CB4856 CT X 10220644 10228137 WBGene00007593 C14H10.3 C14H10.3
0.224656401809925 AGIWUR41483
CB4856 CT X 10233739 10236944 WBGene00007591 zipt-13 C14H10.1
-0.349770695348351 AGIWUR24523
CB4856 CT X 10239805 10241039 WBGene00006135 str-74 C14H10.4
-0.0955649689504456 AGIWUR42696
CB4856 CT X 10242325 10252879 WBGene00007592 C14H10.2 C14H10.2
-0.13286869649013 AGIWUR33394
CB4856 CT X 10258733 10260135 WBGene00007861 C32A9.1 C32A9.1 0.0942862652003109
AGIWUR8449;AGIWUR16047;AGIWUR35637
CB4856 CT X 10273692 10278261 WBGene00009618 F41E7.2 F41E7.2 0.193737580163551

AGIWUR12392;AGIWUR25610;AGIWUR37597
CB4856 CT X 10279599 10282842 WBGene00009617 F41E7.1 F41E7.1 -0.113934780094459
AGIWUR907
CB4856 CT X 10282825 10285734 WBGene00009619 npr-6 F41E7.3 0.143442098536021
AGIWUR28581
CB4856 CT X 10302622 10303143 WBGene00009620 fip-5 F41E7.4 -0.0222224337143686
AGIWUR13840
CB4856 CT X 10304507 10304947 WBGene00009621 fipr-21 F41E7.5 -0.154053157264457
AGIWUR20588
CB4856 CT X 10306595 10306951 WBGene00009624 cnc-8 F41E7.8 -0.270512069854128
AGIWUR9182;AGIWUR11541;AGIWUR16122;AGIWUR19341;AGIWUR43020
CB4856 CT X 10307137 10310466 WBGene00009622 F41E7.6 F41E7.6 -0.591798104381374
AGIWUR756
CB4856 CT X 10307873 10312182 WBGene00009623 F41E7.7 F41E7.7 0.289412909163797
AGIWUR28863
CB4856 CT X 10312756 10316005 WBGene00009625 F41E7.9 F41E7.9 0.0149767055683521
AGIWUR37898
CB4856 CT X 10317722 10321857 WBGene00011107 R07E3.6 R07E3.6 0.653940565324083
AGIWUR11980
CB4856 CT X 10324183 10327642 WBGene00011106 acl-5 R07E3.5 0.135898427927508
AGIWUR7281
CB4856 CT X 10330547 10331900 WBGene00011105 R07E3.4 R07E3.4 -0.358066617412369
AGIWUR25740
CB4856 CT X 10333861 10338200 WBGene00011104 cut-5 R07E3.3 0.754616683775333
AGIWUR41403
CB4856 CT X 10339225 10341161 WBGene00011102 R07E3.1 R07E3.1 0.0609230238474936
AGIWUR22504;AGIWUR33913;AGIWUR44194
CB4856 CT X 10341732 10344080 WBGene00011108 R07E3.7 R07E3.7 -0.0452084694815857
AGIWUR39580
CB4856 CT X 10358476 10360550 WBGene00013028 efhc-1 Y49A10A.1
0.281029725405789 AGIWUR39558
CB4856 CT X 10371077 10372664 WBGene00044949 Y49A10A.2 Y49A10A.2
0.293484923487781 AGIWUR32528;AGIWUR34902
CB4856 CT X 10373235 10377832 WBGene00004375 rme-4 F46F6.1 -0.110302082169653
AGIWUR4542;AGIWUR14043;AGIWUR15056
CB4856 CT X 10379693 10386508 WBGene00001122 dyf-6 F46F6.4 -0.0138506374518981
AGIWUR18040;AGIWUR18179
CB4856 CT X 10397243 10402573 WBGene00009793 pkn-1 F46F6.2 0.0833505724784196
AGIWUR21969

CB4856	CT	X	10402526	10406303	WBGene00001804	gur-3	ZC504.5	0.209423941400481	AGIWUR5039
CB4856	CT	X	10407150	10409774	WBGene00013916	ZC504.1	ZC504.1	-0.0242413272788928	AGIWUR13279
CB4856	CT	X	10412725	10416630	WBGene00000047	acr-8	ZC504.2	-0.16508235312687	AGIWUR5108
CB4856	CT	X	10416639	10419764	WBGene00013917	cdk-11.2	ZC504.3	-0.0787906527464628	AGIWUR41614
CB4856	CT	X	10421655	10428650	WBGene00003247	mig-15	ZC504.4	0.140029587815633	AGIWUR3064;AGIWUR23047;AGIWUR34681;AGIWUR36617;AGIWUR37004;AGIWUR40485;AGIWUR41703
CB4856	CT	X	10444169	10444674	WBGene00008023	C39B10.3	C39B10.3	0.325019249724955	AGIWUR10562
CB4856	CT	X	10447351	10447755	WBGene00008025	C39B10.5	C39B10.5	-0.15147968202777	AGIWUR17118
CB4856	CT	X	10454309	10460341	WBGene00008022	lgc-41	C39B10.2	0.0535786657164454	AGIWUR7852;AGIWUR20309;AGIWUR22317
CB4856	CT	X	10460546	10463580	WBGene00008021	C39B10.1	C39B10.1	0.78376029501498	AGIWUR34897
CB4856	CT	X	10475527	10475959	WBGene00001252	elt-4	C39B10.6	-0.787199864766079	AGIWUR17014
CB4856	CT	X	10481240	10483446	WBGene00001250	elt-2	C33D3.1	0.352634022300409	AGIWUR16561
CB4856	CT	X	10485350	10486757	WBGene00007894	C33D3.3	C33D3.3	-0.319983343455185	AGIWUR33607
CB4856	CT	X	10489063	10489821	WBGene00007895	C33D3.4	C33D3.4	-0.274984142766658	AGIWUR29590
CB4856	CT	X	10491344	10497264	WBGene00007896	C33D3.5	C33D3.5	0.132710458910671	AGIWUR7459
CB4856	CT	X	10505988	10519563	WBGene00006870	vab-3	F14F3.1	0.595875034487722	AGIWUR12076;AGIWUR40464
CB4856	CT	X	10520817	10521464	WBGene00008807	F14F3.4	F14F3.4	-0.0264004400303355	AGIWUR31682
CB4856	CT	X	10521806	10524565	WBGene00008806	mboa-7	F14F3.3	-0.2308639413853	AGIWUR17910
CB4856	CT	X	10529294	10532677	WBGene00010340	slcf-1	F59F5.1	-0.455925119025054	AGIWUR21115
CB4856	CT	X	10533699	10537114	WBGene00010341	glo-3	F59F5.2	0.0220216534490132	AGIWUR8372;AGIWUR13035
CB4856	CT	X	10537141	10542423	WBGene00010342	F59F5.3	F59F5.3	0.828947322552583	

AGIWUR4860
CB4856 CT X 10543042 10543410 WBGene00010343 F59F5.4 F59F5.4 0.247391528219865
AGIWUR21219
CB4856 CT X 10546062 10548962 WBGene00010344 F59F5.5 F59F5.5 -0.117895164847639
AGIWUR26280;AGIWUR29271;AGIWUR42979
CB4856 CT X 10549123 10555239 WBGene00006364 syd-2 F59F5.6 0.274752379444976
AGIWUR15662
CB4856 CT X 10559503 10566668 WBGene00010345 F59F5.7 F59F5.7
-0.00320833950793187 AGIWUR27353
CB4856 CT X 10569273 10575718 WBGene00010199 bet-2 F57C7.1 0.139654094006186
AGIWUR1403;AGIWUR28323;AGIWUR41548
CB4856 CT X 10576960 10581054 WBGene00003733 nhx-5 F57C7.2 -0.051335034247062
AGIWUR6590
CB4856 CT X 10581062 10589538 WBGene00010200 F57C7.4 F57C7.4 0.135963412101061
AGIWUR2002;AGIWUR10895;AGIWUR23278;AGIWUR24655
CB4856 CT X 10589780 10593334 WBGene00004749 sdn-1 F57C7.3 0.247265520440807
AGIWUR10022;AGIWUR15527
CB4856 CT X 10604686 10605828 WBGene00001462 flp-19 M79.4 0.387646980222417
AGIWUR2445
CB4856 CT X 10605832 10624185 WBGene00000018 abl-1 M79.1 0.141285879845729
AGIWUR5979
CB4856 CT X 10626518 10628807 WBGene00010902 M79.2 M79.2 -0.351172014842841
AGIWUR502;AGIWUR38051
CB4856 CT X 10644409 10666874 WBGene00000908 daf-12 F11A1.3 0.214305882019861
AGIWUR11162;AGIWUR39761;AGIWUR45021
CB4856 CT X 10668280 10674293 WBGene00008746 F13E6.2 F13E6.2 0.0400123822944787
AGIWUR25415
CB4856 CT X 10675706 10677109 WBGene00008745 F13E6.1 F13E6.1 0.223975157675753
AGIWUR32239
CB4856 CT X 10681998 10682963 WBGene00008747 phf-31 F13E6.3 -0.360176909395332
AGIWUR20577
CB4856 CT X 10691943 10694461 WBGene00008748 yap-1 F13E6.4 0.261843096082938
AGIWUR873;AGIWUR11673;AGIWUR18252;AGIWUR23825;AGIWUR32484
CB4856 CT X 10695312 10697894 WBGene00008749 F13E6.5 F13E6.5 -0.246141432942176
AGIWUR22704
CB4856 CT X 10697854 10711869 WBGene00006468 rhgf-1 F13E6.6 0.00245773133732108
AGIWUR32532
CB4856 CT X 10716318 10717173 WBGene00011317 T01C1.4 T01C1.4 0.0664808883355814
AGIWUR36558

CB4856	CT	X	10719554	10722054	WBGene00011315	mbr-1	T01C1.2	-0.126591398987794	AGIWUR17126;AGIWUR42959
CB4856	CT	X	10734326	10744355	WBGene00001202	egl-36	R07A4.1	0.317286715686275	AGIWUR14098
CB4856	CT	X	10744356	10746488	WBGene00011073	R07A4.2	R07A4.2	0.0443073601490838	AGIWUR21532
CB4856	CT	X	10751666	10757708	WBGene00011075	R07A4.4	R07A4.4	-0.813860648004573	AGIWUR16492
CB4856	CT	X	10754917	10758140	WBGene00011074	R07A4.3	R07A4.3	0.214768504302234	AGIWUR35068
CB4856	CT	X	10769288	10782973	WBGene00004862	sma-9	T05A10.1	0.260540563099122	AGIWUR7667;AGIWUR8425;AGIWUR17994;AGIWUR18754;AGIWUR19109;AGIWUR26054;AGIWUR34116;AGIWUR34810;AGIWUR44751
CB4856	CT	X	10782996	10783907	WBGene00000525	clc-4	T05A10.2	-0.432778000366388	AGIWUR26254
CB4856	CT	X	10787503	10789700	WBGene00011460	ttr-14	T05A10.3	-0.0861492893259683	AGIWUR7785;AGIWUR26286;AGIWUR27583;AGIWUR27923;AGIWUR30927;AGIWUR33316;AGIWUR35945;AGIWUR42650;AGIWUR43068
CB4856	CT	X	10789628	10792535	WBGene00011462	vap-2	T05A10.5	1.22607819206124	AGIWUR4601;AGIWUR31583
CB4856	CT	X	10792903	10794734	WBGene00011463	T05A10.6	T05A10.6	0.404241015391174	AGIWUR15837;AGIWUR26641
CB4856	CT	X	10794852	10815004	WBGene00004036	plc-1	F31B12.1	0.158988542047747	AGIWUR7963;AGIWUR11662;AGIWUR20227;AGIWUR26127;AGIWUR32796
CB4856	CT	X	10816968	10838650	WBGene00001495	frm-9	F31B12.3	0.0889411121874022	AGIWUR41268;AGIWUR44578
CB4856	CT	X	10842658	10844674	WBGene00007317	C05A9.2	C05A9.2	0.616560053123784	AGIWUR7259
CB4856	CT	X	10854320	10859297	WBGene00003999	pgp-5	C05A9.1	-0.206038547286742	AGIWUR16649;AGIWUR20377;AGIWUR39244
CB4856	CT	X	10859351	10864422	WBGene00004002	pgp-8	T21E8.3	-0.629498311467821	AGIWUR37678
CB4856	CT	X	10871181	10877465	WBGene00004000	pgp-6	T21E8.1	-0.458001036953988	AGIWUR27854
CB4856	CT	X	10883567	10884508	WBGene00164972	T21E8.7	T21E8.7	0.234098711997775	AGIWUR1323
CB4856	CT	X	10886278	10887052	WBGene00011903	T21E8.5	T21E8.5	0.407761375816616	AGIWUR38749
CB4856	CT	X	10888506	10889028	WBGene00009817	F47B10.6	F47B10.6		

0.104783337375824	AGIWUR35274
CB4856	CT	X	10889041	10890556	WBGene00009816	F47B10.5	F47B10.5
-0.121248218548442	AGIWUR1819;AGIWUR3353;AGIWUR3360;AGIWUR9921;AGIWUR11152;AGIWUR16718
CB4856	CT	X	10891295	10891707	WBGene00009815	F47B10.4	F47B10.4
-0.0169165173901877	AGIWUR4612
CB4856	CT	X	10892190	10893569	WBGene00009814	F47B10.3	F47B10.3
0.10237538059799	AGIWUR42103
CB4856	CT	X	10893567	10896598	WBGene00009813	haly-1	F47B10.2
-0.412687793739543	AGIWUR18039;AGIWUR18232;AGIWUR21187;AGIWUR30251;AGIWUR42699
CB4856	CT	X	10897678	10900114	WBGene00009812	suca-1	F47B10.1
-0.0195089059543805
AGIWUR2889;AGIWUR3616;AGIWUR8658;AGIWUR11415;AGIWUR11812;AGIWUR16981;AGIWUR23660;AGIWUR26155
CB4856	CT	X	10903278	10905107	WBGene00009818	acbp-3	F47B10.7
-0.252597118817743	AGIWUR21895
CB4856	CT	X	10905181	10910768	WBGene00009819	F47B10.8	F47B10.8
0.147352940538299	AGIWUR25552
CB4856	CT	X	10912118	10914081	WBGene00009820	F47B10.9	F47B10.9
0.037788254207094	AGIWUR3929;AGIWUR11878;AGIWUR12314;AGIWUR32006
CB4856	CT	X	10916765	10920013	WBGene00011882	nstp-9	T21B6.5	-0.0811286057864953
AGIWUR28758
CB4856	CT	X	10920533	10928998	WBGene00000961	dgn-1	T21B6.1	0.34999383500204
AGIWUR5992;AGIWUR6010;AGIWUR9266;AGIWUR31210;AGIWUR32355;AGIWUR39633
CB4856	CT	X	10938197	10941355	WBGene00011879	pho-7	T21B6.2	-0.128491755107117
AGIWUR9383
CB4856	CT	X	10945160	10948970	WBGene00011880	T21B6.3	T21B6.3	0.246643270663961
AGIWUR12433
CB4856	CT	X	10950708	10951393	WBGene00010717	K09C8.7	K09C8.7	0.622138545075778
AGIWUR37457
CB4856	CT	X	10954134	10958236	WBGene00003943	pbo-4	K09C8.1	-0.105181719305447
AGIWUR5694;AGIWUR21821
CB4856	CT	X	10958761	10959178	WBGene00003757	nlp-19	K09C8.6	0.00824677976046573
AGIWUR20445
CB4856	CT	X	10959568	10960937	WBGene00010715	K09C8.2	K09C8.2	-0.261409556611317
AGIWUR14659
CB4856	CT	X	10960982	10964725	WBGene00003529	nas-10	K09C8.3	0.202234101291316
AGIWUR21993
CB4856	CT	X	10966551	10969898	WBGene00010716	lge-1	K09C8.4	0.117923123101445
AGIWUR23818
CB4856	CT	X	10970963	10972107	WBGene00010718	K09C8.8	K09C8.8	0.169493199241819

AGIWUR18755
CB4856 CT X 10972172 10979265 WBGene00004257 pxn-2 K09C8.5 0.106169070874673
AGIWUR22501
CB4856 CT X 10982351 10987928 WBGene00006897 ver-4 F59F3.5 0.601182243059922
AGIWUR40866
CB4856 CT X 10992483 10997846 WBGene00006896 ver-3 F59F3.1 0.0101987389635561
AGIWUR5042;AGIWUR8537;AGIWUR18078;AGIWUR18120;AGIWUR18428;AGIWUR22182;AGIWUR33911;AGIWUR34669;AGIWUR39163;AGIWUR39571;AGIWUR44850
CB4856 CT X 10998004 10999276 WBGene00010335 F59F3.6 F59F3.6 -0.209629165767873
AGIWUR13384
CB4856 CT X 10999742 11001850 WBGene00000750 col-177 F59F3.2 0.143488628691641
AGIWUR10969
CB4856 CT X 11004984 11008181 WBGene00010334 del-5 F59F3.4 0.174854551225156
AGIWUR36770
CB4856 CT X 11008266 11009070 WBGene00010225 ttr-31 F58A3.5 -0.576457923160044
AGIWUR22294
CB4856 CT X 11009558 11010640 WBGene00010224 F58A3.4 F58A3.4 -0.141429643881363
AGIWUR33904
CB4856 CT X 11012662 11014412 WBGene00010223 F58A3.3 F58A3.3 0.265175988208189
AGIWUR21004
CB4856 CT X 11016410 11023421 WBGene00001184 egl-15 F58A3.2 0.325312147247691
AGIWUR2025;AGIWUR14387;AGIWUR17579;AGIWUR18150;AGIWUR22661;AGIWUR22831;AGIWUR23615;AGIWUR26322;AGIWUR26361;AGIWUR28249;AGIWUR28413;AGIWUR33328;AGIWUR34362;AGIWUR35573;AGIWUR38056;AGIWUR39477;AGIWUR40101
CB4856 CT X 11028917 11040612 WBGene00002261 ldb-1 F58A3.1 0.277068059153007
AGIWUR12215;AGIWUR18780;AGIWUR20417;AGIWUR26587;AGIWUR32684;AGIWUR42410
CB4856 CT X 11041032 11042877 WBGene00007483 C09F12.3 C09F12.3
0.158896609339084 AGIWUR12921
CB4856 CT X 11047446 11049271 WBGene00000522 clc-1 C09F12.1
0.0580074137601603 AGIWUR37251
CB4856 CT X 11053915 11056970 WBGene00007482 C09F12.2 C09F12.2
-0.00449374960904603 AGIWUR5748
CB4856 CT X 11057401 11059794 WBGene00012261 lpr-3 W04G3.8 1.50944601234407
AGIWUR11517
CB4856 CT X 11059994 11061868 WBGene00012255 lpr-6 W04G3.1 1.05811808678045
AGIWUR6825
CB4856 CT X 11062132 11064539 WBGene00012256 lpr-5 W04G3.2 0.567267573675429
AGIWUR12969;AGIWUR25508
CB4856 CT X 11065133 11066811 WBGene00012257 lpr-4 W04G3.3 0.940172693161728
AGIWUR5276

CB4856	CT	X	11068527	11071925	WBGene00000166	apt-9	W04G3.4	-0.219772743132002	AGIWUR18454
CB4856	CT	X	11076182	11076767	WBGene00044151	W04G3.12	W04G3.12	-0.0705881913331967	AGIWUR19796;AGIWUR23344;AGIWUR40941;AGIWUR43619
CB4856	CT	X	11077772	11078243	WBGene00044152	W04G3.10	W04G3.10	-0.476097703601825	AGIWUR9600;AGIWUR14595;AGIWUR20255;AGIWUR39365
CB4856	CT	X	11080678	11087474	WBGene00012259	sulp-7	W04G3.6	-0.066040500145025	AGIWUR14526;AGIWUR17899;AGIWUR25281;AGIWUR25544;AGIWUR43216
CB4856	CT	X	11089243	11090679	WBGene00044153	W04G3.11	W04G3.11	-0.29516783614174	AGIWUR7780;AGIWUR10269;AGIWUR27223;AGIWUR30755;AGIWUR42818
CB4856	CT	X	11096158	11096935	WBGene00012218	W02H3.2	W02H3.2	0.0975854148666455	AGIWUR16182
CB4856	CT	X	11102587	11105460	WBGene00012217	W02H3.1	W02H3.1	-0.965645937478579	AGIWUR14835
CB4856	CT	X	11107376	11109316	WBGene00011140	R08B4.4	R08B4.4	0.0426200557091523	AGIWUR1513
CB4856	CT	X	11109440	11115887	WBGene00001690	grd-1	R08B4.1	-0.207750128619489	AGIWUR17903
CB4856	CT	X	11116107	11116823	WBGene00011141	R08B4.5	R08B4.5	0.387679627418296	AGIWUR24780;AGIWUR26045;AGIWUR30649;AGIWUR32569
CB4856	CT	X	11118959	11121005	WBGene00011139	R08B4.3	R08B4.3	-0.127880200973526	AGIWUR26481;AGIWUR44332
CB4856	CT	X	11121916	11125615	WBGene00044330	alr-1	R08B4.2	0.613695431583654	AGIWUR30021
CB4856	CT	X	11142971	11152820	WBGene00007327	C05C9.3	C05C9.3	-0.147509881913031	AGIWUR5280
CB4856	CT	X	11153065	11157643	WBGene00007326	C05C9.2	C05C9.2	0.0636334754131962	AGIWUR6141;AGIWUR40885
CB4856	CT	X	11160981	11164006	WBGene00007325	C05C9.1	C05C9.1	0.157301134594761	AGIWUR37380
CB4856	CT	X	11163958	11189591	WBGene00008735	F13D2.1	F13D2.1	-0.200026949666718	AGIWUR9368;AGIWUR31805;AGIWUR34326
CB4856	CT	X	11192485	11196841	WBGene00007938	C34F6.1	C34F6.1	-0.325231001572973	AGIWUR23731
CB4856	CT	X	11199455	11200464	WBGene00000751	col-178	C34F6.2	0.225046707875656	AGIWUR40880
CB4856	CT	X	11201465	11202488	WBGene00000752	col-179	C34F6.3	-0.13409502969006	AGIWUR3484;AGIWUR4228;AGIWUR11395;AGIWUR12859;AGIWUR18664;AGIWUR21635;AGIWUR24699;AGIWUR30009;AGIWUR35779;AGIWUR37272

CB4856	CT	X	11202476	11203990	WBGene00007945	C34F6.11	C34F6.11	-0.104962006714125	AGIWUR32368
CB4856	CT	X	11204117	11207155	WBGene00002029	hst-2	C34F6.4	-0.0725240224821189	AGIWUR28552
CB4856	CT	X	11207250	11208994	WBGene00007939	C34F6.5	C34F6.5	0.010041034668975	AGIWUR21;AGIWUR994
CB4856	CT	X	11210107	11215470	WBGene00007941	C34F6.7	C34F6.7	-0.162030654301148	AGIWUR6467;AGIWUR32311;AGIWUR32877
CB4856	CT	X	11215696	11218305	WBGene00007942	idh-2	C34F6.8	-0.189441773188044	AGIWUR3586
CB4856	CT	X	11218135	11222811	WBGene00007943	C34F6.9	C34F6.9	-0.140217719159374	AGIWUR23073
CB4856	CT	X	11229213	11237383	WBGene00007944	C34F6.10	C34F6.10	0.400130399108996	AGIWUR42553
CB4856	CT	X	11257361	11260225	WBGene00007266	C03A3.1	C03A3.1	0.530177006222256	AGIWUR11397;AGIWUR29952;AGIWUR39266
CB4856	CT	X	11262848	11266324	WBGene00007267	C03A3.2	C03A3.2	0.076525588016958	AGIWUR4732;AGIWUR8292
CB4856	CT	X	11267391	11269023	WBGene00007268	C03A3.3	C03A3.3	0.0711200812157458	AGIWUR44225
CB4856	CT	X	11271184	11275482	WBGene00002050	ifa-1	F38B2.1	-0.162451860595649	AGIWUR2245;AGIWUR4997;AGIWUR15389;AGIWUR25371;AGIWUR31125;AGIWUR33213;AGIWUR38221;AGIWUR40353;AGIWUR40580;AGIWUR43826
CB4856	CT	X	11278401	11279376	WBGene00009529	F38B2.2	F38B2.2	0.0513367569030927	AGIWUR2233;AGIWUR17353;AGIWUR22429;AGIWUR23640;AGIWUR40315;AGIWUR40760
CB4856	CT	X	11281716	11286346	WBGene00009530	F38B2.3	F38B2.3	-0.00390594037722281	AGIWUR4915
CB4856	CT	X	11286443	11287717	WBGene00009531	F38B2.4	F38B2.4	-0.0623896757247042	AGIWUR5307;AGIWUR5637;AGIWUR26506;AGIWUR39548
CB4856	CT	X	11294962	11296731	WBGene00002124	inx-2	F08G12.10	-0.104801093133759	AGIWUR1732
CB4856	CT	X	11298036	11299784	WBGene00008586	F08G12.2	F08G12.2	-0.186272922111987	AGIWUR40930
CB4856	CT	X	11300283	11302094	WBGene00008587	F08G12.3	F08G12.3	-0.291918644052227	AGIWUR19748
CB4856	CT	X	11302074	11305873	WBGene00008585	F08G12.1	F08G12.1	-0.0278285867964705	AGIWUR19035
CB4856	CT	X	11307360	11308400	WBGene00006922	vhl-1	F08G12.4	-0.349803223338595	AGIWUR501

CB4856	CT	X	11308864	11310285	WBGene00008588	F08G12.5	F08G12.5	0.030281962218222	AGIWUR36011
CB4856	CT	X	11313385	11315612	WBGene00044135	F08G12.11	F08G12.11	0.0469249093780504	AGIWUR2107;AGIWUR11925;AGIWUR30415;AGIWUR39046
CB4856	CT	X	11317050	11318036	WBGene00008589	nlp-57	F08G12.8	0.0993617593596099	AGIWUR25391
CB4856	CT	X	11318278	11321085	WBGene00013976	hizr-1	ZK455.6	-0.0681076596105044	AGIWUR3772
CB4856	CT	X	11321079	11324251	WBGene00000212	asm-2	ZK455.4	0.229600730649413	AGIWUR12179;AGIWUR20805;AGIWUR30554;AGIWUR45075
CB4856	CT	X	11324517	11326901	WBGene00013975	ZK455.5	ZK455.5	-0.375915669743356	AGIWUR16779
CB4856	CT	X	11328591	11332072	WBGene00013974	npr-9	ZK455.3	0.12435372315725	AGIWUR11847
CB4856	CT	X	11332553	11338301	WBGene00001536	gcy-9	ZK455.2	-0.131895310848611	AGIWUR15202
CB4856	CT	X	11339874	11343568	WBGene00000040	aco-1	ZK455.1	-0.166391260930769	AGIWUR15192;AGIWUR20633;AGIWUR21869;AGIWUR30301
CB4856	CT	X	11346455	11350244	WBGene00003843	oct-2	ZK455.8	0.0610621293613747	AGIWUR2167;AGIWUR14035
CB4856	CT	X	11352136	11357094	WBGene00003997	pgp-3	ZK455.7	-0.0727661265150905	AGIWUR11749;AGIWUR17389;AGIWUR20480;AGIWUR22101
CB4856	CT	X	11358395	11364049	WBGene00003998	pgp-4	F42E11.1	-0.399232293216179	AGIWUR28989;AGIWUR41306;AGIWUR44841
CB4856	CT	X	11368719	11374358	WBGene00009632	ttyh-1	F42E11.2	0.158361379098482	AGIWUR10181;AGIWUR19745;AGIWUR21649;AGIWUR22469;AGIWUR28938;AGIWUR38032;AGIWUR44333
CB4856	CT	X	11374599	11375609	WBGene00009633	F42E11.3	F42E11.3	-0.311628475258919	AGIWUR39282
CB4856	CT	X	11375887	11377470	WBGene00006584	tni-1	F42E11.4	-0.476751449514964	AGIWUR8414
CB4856	CT	X	11384773	11392779	WBGene00008571	prmn-1	F08B12.1	0.278868665388904	AGIWUR2776
CB4856	CT	X	11393293	11394619	WBGene00004197	prx-12	F08B12.2	0.19501269329354	AGIWUR27319
CB4856	CT	X	11394605	11405931	WBGene00004831	slo-2	F08B12.3	-0.0229273600505699	AGIWUR1051;AGIWUR7985;AGIWUR23787;AGIWUR24004;AGIWUR26341
CB4856	CT	X	11407054	11407730	WBGene00008572	F08B12.4	F08B12.4	0.87698102911132	AGIWUR9647
CB4856	CT	X	11410181	11414887	WBGene00003970	pek-1	F46C3.1	0.0829499229716362	

AGIWUR522;AGIWUR2937;AGIWUR17263
CB4856	CT	X	11416653	11418740	WBGene00009777	F46C3.2	F46C3.2	-0.241990732032704	AGIWUR20393
CB4856	CT	X	11419108	11420074	WBGene00045253	F46C3.6	F46C3.6	0.559941555478781	AGIWUR6859;AGIWUR19299;AGIWUR25267;AGIWUR33107;AGIWUR35145;AGIWUR36021
CB4856	CT	X	11420474	11433524	WBGene00002037	hum-4	F46C3.3	-0.128194049160669	AGIWUR1757;AGIWUR4067;AGIWUR4936;AGIWUR18116;AGIWUR18323;AGIWUR19638;AGIWUR21461;AGIWUR22845
CB4856	CT	X	11434792	11435899	WBGene00044703	F46C3.5	F46C3.5	0.243229460740487	AGIWUR7105
CB4856	CT	X	11436124	11437747	WBGene00044702	F46C3.4	F46C3.4	0.0104453672101172	AGIWUR7674
CB4856	CT	X	11444424	11446635	WBGene00009842	igeg-2	F48C5.1	0.00336615366356252	AGIWUR24691
CB4856	CT	X	11451617	11454195	WBGene00009843	F48C5.2	F48C5.2	-0.138216878592265	AGIWUR39842
CB4856	CT	X	11465557	11484368	WBGene00003003	lin-14	T25C12.1	-0.060783016029526	AGIWUR25830
CB4856	CT	X	11491087	11499999	WBGene00012018	T25C12.3	T25C12.3	-0.560163328251656	AGIWUR2452
CB4856	CT	X	11502267	11509432	WBGene00009257	F29G6.1	F29G6.1	-0.091948913645072	AGIWUR18243
CB4856	CT	X	11509446	11511451	WBGene00009258	ccdc-149	F29G6.2	0.0827607345358921	AGIWUR15047;AGIWUR17433;AGIWUR35296
CB4856	CT	X	11511558	11519062	WBGene00009259	hpo-34	F29G6.3	-0.207684808253246	AGIWUR844;AGIWUR23574;AGIWUR44644;AGIWUR44749
CB4856	CT	X	11522707	11533450	WBGene00004746	sdc-2	C35C5.1	-0.141305133776516	AGIWUR6463
CB4856	CT	X	11534004	11536574	WBGene00007957	C35C5.8	C35C5.8	2.69603159499795	AGIWUR3917;AGIWUR30581;AGIWUR36963
CB4856	CT	X	11536568	11537124	WBGene00007958	C35C5.9	C35C5.9	0.970680706721208	AGIWUR9875;AGIWUR17639;AGIWUR34507;AGIWUR35227;AGIWUR43027;AGIWUR44311
CB4856	CT	X	11537714	11541417	WBGene00007954	gcp-2.2	C35C5.2	0.109712961975567	AGIWUR19973;AGIWUR28266
CB4856	CT	X	11542129	11545061	WBGene00044121	gcp-2.3	C35C5.11	-0.289641659931663	AGIWUR24227
CB4856	CT	X	11545091	11546495	WBGene00007955	selt-1.1	C35C5.3	-0.0989729492383713	AGIWUR22821
CB4856	CT	X	11548608	11550740	WBGene00003239	mig-2	C35C5.4	-0.130514708534541	AGIWUR1331;AGIWUR5810;AGIWUR8838;AGIWUR17531;AGIWUR43904;AGIWUR44479

CB4856	CT	X	11554795	11559858	WBGene00007959	C35C5.10	C35C5.10
-0.31029447906352	AGIWUR2588
CB4856	CT	X	11560915	11563697	WBGene00002975	lev-8	C35C5.5	0.264780758124857
AGIWUR35817
CB4856	CT	X	11563923	11569531	WBGene00007956	trpp-9	C35C5.6	0.0716480164145015
AGIWUR43921
CB4856	CT	X	11576247	11581727	WBGene00001490	frm-3	H05G16.1
0.13869130545717
AGIWUR18461;AGIWUR18670;AGIWUR20783;AGIWUR25861;AGIWUR28199;AGIWUR29720;AGIWUR33619;AGIWUR37114;AGIWUR40164
;AGIWUR40423;AGIWUR43229
CB4856	CT	X	11581751	11584960	WBGene00009932	F52D10.6	F52D10.6
0.261365282276816	AGIWUR32267
CB4856	CT	X	11585675	11589757	WBGene00009929	abts-2	F52D10.1
0.0609513437491636	AGIWUR18270
CB4856	CT	X	11589686	11591719	WBGene00009930	F52D10.2	F52D10.2
-0.00847534593719779	AGIWUR36851
CB4856	CT	X	11603709	11605451	WBGene00001502	ftt-2	F52D10.3
-0.0338018609391142	AGIWUR5562;AGIWUR16612;AGIWUR30144
CB4856	CT	X	11617462	11619795	WBGene00009931	F52D10.4	F52D10.4
0.177057543165888	AGIWUR33683
CB4856	CT	X	11620047	11621996	WBGene00006065	sto-3	F52D10.5
-0.263813103953817	AGIWUR16696
CB4856	CT	X	11622967	11627113	WBGene00006694	twk-44	Y71H9A.1
-0.0354041863818377	AGIWUR2136;AGIWUR13930;AGIWUR22707;AGIWUR34157;AGIWUR36683;AGIWUR39124
CB4856	CT	X	11632407	11636508	WBGene00006068	sto-6	Y71H9A.2
-0.0789660647237191	AGIWUR37934
CB4856	CT	X	11644143	11647425	WBGene00006066	sto-4	Y71H9A.3
0.0642978739082842	AGIWUR18972;AGIWUR20785;AGIWUR21265
CB4856	CT	X	11647423	11648196	WBGene00011446	T04F8.8	T04F8.8	-0.223865706469397
AGIWUR18677
CB4856	CT	X	11648358	11650918	WBGene00011443	cutl-11	T04F8.4	0.129979885025358
AGIWUR19813
CB4856	CT	X	11653570	11656569	WBGene00011441	T04F8.2	T04F8.2	-0.175142305477758
AGIWUR2428;AGIWUR19123
CB4856	CT	X	11659510	11661660	WBGene00011440	sfxn-1.5	T04F8.1
-0.0983938004887293	AGIWUR30329
CB4856	CT	X	11667127	11672309	WBGene00011444	T04F8.6	T04F8.6	-0.0662197018932429
AGIWUR7225;AGIWUR17517;AGIWUR18050;AGIWUR18678;AGIWUR18752;AGIWUR29417;AGIWUR31500;AGIWUR34985
CB4856	CT	X	11674416	11677090	WBGene00011447	T04F8.9	T04F8.9	0.602480348617203

AGIWUR34736
CB4856 CT X 11677515 11680980 WBGene00011445 T04F8.7 T04F8.7 0.864709256134404
AGIWUR5669;AGIWUR31052;AGIWUR38300
CB4856 CT X 11684661 11685778 WBGene00001981 hnd-1 C44C10.8
0.606315379393365 AGIWUR25505
CB4856 CT X 11687000 11688269 WBGene00008088 C44C10.7 C44C10.7
0.0961565801725532 AGIWUR36171
CB4856 CT X 11690314 11690789 WBGene00008090 C44C10.10 C44C10.10
0.244210214806223 AGIWUR42468
CB4856 CT X 11691109 11692456 WBGene00008086 C44C10.5 C44C10.5
-0.203974685197903 AGIWUR26240
CB4856 CT X 11693464 11695750 WBGene00008085 C44C10.4 C44C10.4
-0.209503104869992 AGIWUR6646
CB4856 CT X 11696996 11698065 WBGene00044133 C44C10.12 C44C10.12
-0.0260748947199492 AGIWUR37824;AGIWUR38613;AGIWUR42182
CB4856 CT X 11698384 11699098 WBGene00008087 C44C10.6 C44C10.6
0.028090496335027 AGIWUR36681
CB4856 CT X 11702525 11704500 WBGene00008084 C44C10.3 C44C10.3
-0.521122387193525 AGIWUR21455;AGIWUR29164;AGIWUR41440
CB4856 CT X 11704888 11707238 WBGene00008083 cyp-29A1 C44C10.2
0.170960884079235 AGIWUR6870
CB4856 CT X 11707903 11710190 WBGene00043985 C44C10.11 C44C10.11
-0.0119870032805394 AGIWUR5144;AGIWUR21747;AGIWUR26369;AGIWUR35133;AGIWUR35582;AGIWUR40080
CB4856 CT X 11711307 11712280 WBGene00000753 col-180 C44C10.1
-0.144104553835028 AGIWUR19530
CB4856 CT X 11713240 11716511 WBGene00008089 C44C10.9 C44C10.9
-0.143724268919185 AGIWUR1389;AGIWUR14021
CB4856 CT X 11721724 11724195 WBGene00008369 D1053.4 D1053.4 -0.204422744607703
AGIWUR22031
CB4856 CT X 11726271 11727936 WBGene00001790 gst-42 D1053.1 -0.29139206359884
AGIWUR39657
CB4856 CT X 11728151 11728891 WBGene00008367 D1053.2 D1053.2 -0.602570754110013
AGIWUR4100
CB4856 CT X 11729586 11733698 WBGene00008368 D1053.3 D1053.3 0.201671997419844
AGIWUR22019;AGIWUR42744
CB4856 CT X 11746076 11747249 WBGene00008049 C40H5.7 C40H5.7 -0.296376513167226
AGIWUR42587
CB4856 CT X 11754776 11756762 WBGene00008045 C40H5.2 C40H5.2 -0.281549938275131
AGIWUR45000

CB4856	CT	X	11759198	11764535	WBGene00008047	lact-7	C40H5.4	0.0229876751006262	AGIWUR2323;AGIWUR6946;AGIWUR12305;AGIWUR29315
CB4856	CT	X	11764456	11769378	WBGene00006654	ttx-3	C40H5.5	0.0502034217217768	AGIWUR3181;AGIWUR40976
CB4856	CT	X	11775190	11775728	WBGene00008048	hpo-41	C40H5.6	-0.360462396704769	AGIWUR7235
CB4856	CT	X	11781463	11784330	WBGene00013631	Y102F5A.1	Y102F5A.1	0.0828503188200234	AGIWUR41265
CB4856	CT	X	11798505	11803656	WBGene00004682	rsd-3	C34E11.1	0.151455819041551	AGIWUR12992
CB4856	CT	X	11804210	11805306	WBGene00007937	C34E11.4	C34E11.4	0.594552492251019	AGIWUR29254
CB4856	CT	X	11805378	11814653	WBGene00007935	C34E11.2	C34E11.2	0.194294893558168	AGIWUR6735;AGIWUR17971;AGIWUR18770;AGIWUR41165
CB4856	CT	X	11826093	11836162	WBGene00044079	tag-241	C34E11.3	0.170124476825528	AGIWUR21066
CB4856	CT	X	11836068	11837478	WBGene00006388	taf-7.1	F54F7.1	-0.0184249859998218	AGIWUR43977
CB4856	CT	X	11843663	11845337	WBGene00010064	F54F7.2	F54F7.2	-0.306344382656777	AGIWUR20596
CB4856	CT	X	11847856	11848695	WBGene00045251	F54F7.9	F54F7.9	-0.0962121839498273	AGIWUR36255
CB4856	CT	X	11849392	11850182	WBGene00010065	F54F7.3	F54F7.3	0.177835373021106	AGIWUR44650
CB4856	CT	X	11852794	11853729	WBGene00006965	xtr-1	F54F7.4	1.25569983854434	AGIWUR11219
CB4856	CT	X	11853818	11854996	WBGene00010068	F54F7.8	F54F7.8	0.0872250547129845	AGIWUR34989
CB4856	CT	X	11857478	11862373	WBGene00003219	mes-1	F54F7.5	-0.354897446778367	AGIWUR34339
CB4856	CT	X	11865383	11866827	WBGene00010066	F54F7.6	F54F7.6	-0.240560264643392	AGIWUR14467
CB4856	CT	X	11867971	11869360	WBGene00010067	dot-1.2	F54F7.7	-0.238527295590949	AGIWUR12110
CB4856	CT	X	11875654	11877242	WBGene00013389	Y62H9A.1	Y62H9A.1	0.184455648213214	AGIWUR11046
CB4856	CT	X	11877418	11878196	WBGene00013390	Y62H9A.2	Y62H9A.2	0.157986049467981	AGIWUR43304
CB4856	CT	X	11879110	11879971	WBGene00013391	Y62H9A.3	Y62H9A.3		

-0.0356297860028505	AGIWUR45025
CB4856	CT	X	11880124	11881264	WBGene00013392	Y62H9A.4	Y62H9A.4
-0.183838971064841	AGIWUR33966
CB4856	CT	X	11882144	11882917	WBGene00013393	Y62H9A.5	Y62H9A.5
0.114768705842128	AGIWUR33524
CB4856	CT	X	11883127	11884043	WBGene00013394	ule-5	Y62H9A.6
-0.0372973714753755	AGIWUR14679
CB4856	CT	X	11884330	11886438	WBGene00013395	Y62H9A.7	Y62H9A.7
0.0447795288213619	AGIWUR35290
CB4856	CT	X	11891259	11892422	WBGene00013396	Y62H9A.8	Y62H9A.8
0.153527268101566	AGIWUR19602
CB4856	CT	X	11895585	11898140	WBGene00013397	Y62H9A.9	Y62H9A.9
-0.31136845825834	AGIWUR45120
CB4856	CT	X	11904924	11907022	WBGene00013399	Y62H9A.11	Y62H9A.11
-0.0738025573102368	AGIWUR40597
CB4856	CT	X	11907267	11910145	WBGene00045252	Y62H9A.15	Y62H9A.15
-0.0956104338288108	AGIWUR37686;AGIWUR42152
CB4856	CT	X	11914124	11915558	WBGene00013400	Y62H9A.12	Y62H9A.12
-0.227486411012496	AGIWUR36585
CB4856	CT	X	11917005	11920158	WBGene00013401	Y62H9A.13	Y62H9A.13
0.0880698589040207	AGIWUR26048
CB4856	CT	X	11921252	11923138	WBGene00013402	Y62H9A.14	Y62H9A.14
0.216670435316425	AGIWUR30338
CB4856	CT	X	11924461	11927032	WBGene00010119	F55G7.1	F55G7.1	-0.277786136971408
AGIWUR11372;AGIWUR22511
CB4856	CT	X	11928107	11929372	WBGene00045493	F55G7.4	F55G7.4	-0.199879866602004
AGIWUR39073
CB4856	CT	X	11929899	11931646	WBGene00010120	dot-1.4	F55G7.2	0.0225982669613582
AGIWUR9664
CB4856	CT	X	11940336	11942051	WBGene00010121	F55G7.3	F55G7.3	0.05134592867919
AGIWUR19193
CB4856	CT	X	11944313	11946834	WBGene00011614	nfya-1	T08D10.1
-0.015656942415994	AGIWUR28467
CB4856	CT	X	11948843	11951893	WBGene00011615	lsd-1	T08D10.2
-0.190303351972647	AGIWUR18850
CB4856	CT	X	11952085	11953329	WBGene00044148	T08D10.4	T08D10.4
0.188864934459915	AGIWUR17215;AGIWUR24785
CB4856	CT	X	11957563	11958395	WBGene00206466	T08D10.5	T08D10.5
-0.0906838606874665	AGIWUR1122

CB4856	CT	X	11960145	11961509	WBGene00008212	C49F5.8	C49F5.8	-0.468935482713967	AGIWUR44049
CB4856	CT	X	11961443	11961777	WBGene00268214	C49F5.12	C49F5.12	-0.176937729669345	AGIWUR950;AGIWUR15616;AGIWUR43673
CB4856	CT	X	11962413	11962646	WBGene00045316	C49F5.9	C49F5.9	-0.438869764543043	AGIWUR18274;AGIWUR34913;AGIWUR35638;AGIWUR35757;AGIWUR44815
CB4856	CT	X	11965962	11969784	WBGene00008205	sams-1	C49F5.1	-0.386182698897542	AGIWUR1947;AGIWUR7219;AGIWUR10862;AGIWUR24028;AGIWUR26342;AGIWUR32238
CB4856	CT	X	11968034	12021212	WBGene00012158	ucr-2.1	VW06B3R.1	0.0772387690546301	AGIWUR8278;AGIWUR8939;AGIWUR16170;AGIWUR31443
CB4856	CT	X	12028711	12031498	WBGene00007115	B0198.2	B0198.2	0.0326178866412121	AGIWUR5120
CB4856	CT	X	12031598	12034037	WBGene00006646	tsp-20	B0198.1	0.345067701432112	AGIWUR7276
CB4856	CT	X	12042593	12057049	WBGene00007116	B0198.3	B0198.3	0.254735482271366	AGIWUR8482
CB4856	CT	X	12065264	12067432	WBGene00007594	lgc-23	C15A7.1	0.104668535931609	AGIWUR16523
CB4856	CT	X	12068550	12070563	WBGene00045385	lgc-24	C15A7.4	0.191732882420474	AGIWUR25344
CB4856	CT	X	12070743	12073863	WBGene00007595	C15A7.2	C15A7.2	0.220544966779515	AGIWUR26829;AGIWUR37909
CB4856	CT	X	12076553	12093083	WBGene00012226	W03G11.4	W03G11.4	0.641402359283325	AGIWUR19534;AGIWUR32595
CB4856	CT	X	12100196	12103812	WBGene00012225	W03G11.3	W03G11.3	-0.108705797851366	AGIWUR6598;AGIWUR7282;AGIWUR8929;AGIWUR16839;AGIWUR22839;AGIWUR23249;AGIWUR41917
CB4856	CT	X	12104930	12106081	WBGene00000754	col-181	W03G11.1	0.0530472924279657	AGIWUR30503
CB4856	CT	X	12106407	12108617	WBGene00012224	W03G11.2	W03G11.2	-0.15456157320067	AGIWUR7267
CB4856	CT	X	12109135	12111185	WBGene00011354	lgc-13	T01H10.1	0.401554899560026	AGIWUR15109
CB4856	CT	X	12111513	12113475	WBGene00011355	lgc-14	T01H10.2	0.257985526665523	AGIWUR6157;AGIWUR14058;AGIWUR33985;AGIWUR40554
CB4856	CT	X	12114324	12115923	WBGene00011356	lgc-15	T01H10.3	0.263486578467793	AGIWUR16903
CB4856	CT	X	12116615	12117763	WBGene00011357	T01H10.4	T01H10.4	-0.0814441981620666	AGIWUR10006;AGIWUR28978;AGIWUR33766
CB4856	CT	X	12118640	12120661	WBGene00011358	lgc-16	T01H10.5

0.0145099844681189 AGIWUR10023
CB4856 CT X 12121585 12125048 WBGene00011359 lgc-17 T01H10.6
0.172398586570871 AGIWUR31474
CB4856 CT X 12125608 12128000 WBGene00011360 lgc-18 T01H10.7
1.15226721538086 AGIWUR397
CB4856 CT X 12128144 12141151 WBGene00011361 lyst-1 T01H10.8
0.309290613168131 AGIWUR42346
CB4856 CT X 12142869 12143819 WBGene00010926 M153.3 M153.3 -0.417339108182559
AGIWUR36470
CB4856 CT X 12144114 12145940 WBGene00045481 M153.5 M153.5 0.407807611635588
AGIWUR15412
CB4856 CT X 12146832 12148529 WBGene00010924 M153.1 M153.1 0.0338209782498282
AGIWUR36000
CB4856 CT X 12150080 12151474 WBGene00044144 M153.4 M153.4 0.057194165650472
AGIWUR5719;AGIWUR7960;AGIWUR16127;AGIWUR32416;AGIWUR43557
CB4856 CT X 12151359 12154105 WBGene00010925 M153.2 M153.2 -0.230748415457652
AGIWUR13412
CB4856 CT X 12156407 12160039 WBGene00010222 F57G12.2 F57G12.2
0.295845480148873 AGIWUR6500;AGIWUR11956;AGIWUR12822;AGIWUR28178
CB4856 CT X 12169092 12171681 WBGene00010221 F57G12.1 F57G12.1
-0.0961486898168573 AGIWUR24594;AGIWUR27372
CB4856 CT X 12199655 12213049 WBGene00000277 cab-1 C23H4.1 0.402891529796478
AGIWUR10784;AGIWUR10818;AGIWUR15448;AGIWUR17107;AGIWUR18304;AGIWUR36949;AGIWUR39077;AGIWUR41971
CB4856 CT X 12217243 12219312 WBGene00044132 C23H4.8 C23H4.8 -0.137052588654472
AGIWUR18650;AGIWUR25954
CB4856 CT X 12219710 12222745 WBGene00007691 C23H4.2 C23H4.2 -0.0677109344583435
AGIWUR31189;AGIWUR35320;AGIWUR36580
CB4856 CT X 12222869 12226047 WBGene00007695 C23H4.7 C23H4.7 -0.64349648638267
AGIWUR32273
CB4856 CT X 12226203 12229861 WBGene00007692 C23H4.3 C23H4.3 -0.368817151112479
AGIWUR37430
CB4856 CT X 12229951 12233244 WBGene00007693 C23H4.4 C23H4.4 -0.621937242332029
AGIWUR7406
CB4856 CT X 12235621 12240781 WBGene00007694 C23H4.6 C23H4.6 0.264915199158956
AGIWUR31706
CB4856 CT X 12241627 12244637 WBGene00008451 E01G6.3 E01G6.3 0.33240194955804
AGIWUR14402
CB4856 CT X 12256644 12258971 WBGene00008450 E01G6.2 E01G6.2 0.272198320435807
AGIWUR5313

CB4856 CT X 12260881 12266537 WBGene00008449 E01G6.1 E01G6.1 -0.152679703971241
AGIWUR6911
CB4856 CT X 12266912 12270941 WBGene00010403 otpl-8 H19J13.1
-0.216293454834656 AGIWUR20322
CB4856 CT X 12273638 12274691 WBGene00010404 H19J13.2 H19J13.2
-0.158863573293821 AGIWUR32478
CB4856 CT X 12275042 12275677 WBGene00007294 C04B4.6 C04B4.6 0.256085812276723
AGIWUR31570
CB4856 CT X 12280707 12282559 WBGene00007289 C04B4.1 C04B4.1 -0.0469914075990054
AGIWUR36736
CB4856 CT X 12283285 12285307 WBGene00007290 C04B4.2 C04B4.2 -0.175740655946413
AGIWUR10264
CB4856 CT X 12285824 12287253 WBGene00007291 lips-2 C04B4.3 0.165939899614144
AGIWUR12086
CB4856 CT X 12287659 12288354 WBGene00007293 C04B4.5 C04B4.5 -0.250903628825245
AGIWUR44214
CB4856 CT X 12290839 12293267 WBGene00007292 C04B4.4 C04B4.4 -0.0697133114164261
AGIWUR37988
CB4856 CT X 12293767 12295311 WBGene00012299 W06D11.1 W06D11.1
-0.371573594599761 AGIWUR23674
CB4856 CT X 12296047 12296644 WBGene00012300 W06D11.2 W06D11.2
-0.0424880705353292 AGIWUR11296
CB4856 CT X 12298683 12300022 WBGene00012301 W06D11.3 W06D11.3
-0.321335385003705 AGIWUR2184
CB4856 CT X 12300134 12301282 WBGene00012303 W06D11.5 W06D11.5
-0.383262424458561 AGIWUR42832
CB4856 CT X 12301488 12302817 WBGene00012302 dot-1.3 W06D11.4
-0.0900655171697222 AGIWUR8566
CB4856 CT X 12306865 12309482 WBGene00008902 phf-32 F17A2.3 0.0870013826491434
AGIWUR35016
CB4856 CT X 12310812 12312730 WBGene00008903 F17A2.4 F17A2.4 0.0434908638102289
AGIWUR27669
CB4856 CT X 12315568 12317673 WBGene00006966 xtr-2 F17A2.1 -0.115858199114722
AGIWUR2512;AGIWUR9538;AGIWUR16671;AGIWUR33859;AGIWUR44395
CB4856 CT X 12318240 12320777 WBGene00000461 ceh-40 F17A2.5 -0.217298625660289
AGIWUR40436
CB4856 CT X 12321657 12322993 WBGene00008904 F17A2.13 F17A2.13
-0.0964333048311907 AGIWUR36689
CB4856 CT X 12323739 12325432 WBGene00005118 srd-40 F17A2.12

0.13383929935109	AGIWUR31745
CB4856	CT	X	12326724	12328365	WBGene00005119	srd-41	F17A2.6	0.173710732240377	AGIWUR18512
CB4856	CT	X	12328976	12330526	WBGene00005123	srd-45	F17A2.7	0.159523024261038	AGIWUR44137
CB4856	CT	X	12332095	12333302	WBGene00005124	srd-46	F17A2.10	0.0961265602764403	AGIWUR16372
CB4856	CT	X	12333642	12335290	WBGene00005125	srd-47	F17A2.11	0.230094735967255	AGIWUR33221
CB4856	CT	X	12336108	12337752	WBGene00005126	srd-48	F17A2.9	0.131279016049402	AGIWUR11538
CB4856	CT	X	12338440	12340248	WBGene00005122	srd-44	F17A2.8	0.326522550629203	AGIWUR32586
CB4856	CT	X	12340378	12341438	WBGene00006979	zig-2	F42F12.2	0.643274525425348	AGIWUR17101
CB4856	CT	X	12342289	12342597	WBGene00044397	nspc-8	F42F12.12	-0.117290678055997	AGIWUR3560;AGIWUR10811;AGIWUR34815;AGIWUR41515
CB4856	CT	X	12343609	12344043	WBGene00009640	nspc-10	F42F12.9	0.238290056091628	AGIWUR20803;AGIWUR25081
CB4856	CT	X	12347029	12347899	WBGene00044140	F42F12.11	F42F12.11	0.127508146417869	AGIWUR23806;AGIWUR30383;AGIWUR41994
CB4856	CT	X	12350578	12351788	WBGene00009635	F42F12.3	F42F12.3	-0.0538078008160063	AGIWUR13631
CB4856	CT	X	12351978	12352949	WBGene00009636	F42F12.4	F42F12.4	-0.0519497860784646	AGIWUR9513;AGIWUR25004;AGIWUR33036;AGIWUR42098
CB4856	CT	X	12353550	12353768	WBGene00044398	nspc-11	F42F12.13	-0.142922312586784	AGIWUR7590;AGIWUR24310;AGIWUR29559;AGIWUR37495;AGIWUR41829
CB4856	CT	X	12356194	12356681	WBGene00023493	nspc-12	F42F12.10	-0.129884928629263	AGIWUR20494;AGIWUR24317
CB4856	CT	X	12362397	12363102	WBGene00008961	F19H6.6	F19H6.6	-0.258313832199503	AGIWUR43762
CB4856	CT	X	12363713	12364904	WBGene00008960	F19H6.5	F19H6.5	-0.223108840890809	AGIWUR22825
CB4856	CT	X	12365688	12367255	WBGene00008959	F19H6.4	F19H6.4	-0.0675045309591162	AGIWUR8462
CB4856	CT	X	12374223	12378393	WBGene00008957	glb-13	F19H6.2	0.00897834712956591	AGIWUR12885;AGIWUR35693
CB4856	CT	X	12389594	12391474	WBGene00008956	nekl-3	F19H6.1	0.170003716702613	AGIWUR10988

CB4856 CT X 12394265 12399534 WBGene00008924 F17E5.2 F17E5.2 -0.263930701008353
AGIWUR1387
CB4856 CT X 12399517 12414037 WBGene00002991 lin-2 F17E5.1 -0.0794675544328332
AGIWUR13913
CB4856 CT X 12425730 12426631 WBGene00023492 T24D3.2 T24D3.2 -0.387121538524439
AGIWUR6201;AGIWUR10096;AGIWUR16437;AGIWUR21018;AGIWUR30257;AGIWUR31249;AGIWUR40680;AGIWUR42163
CB4856 CT X 12434154 12443915 WBGene00011824 T18D3.7 T18D3.7 0.715908113572773
AGIWUR6965;AGIWUR18290;AGIWUR21788
CB4856 CT X 12449896 12451360 WBGene00011823 T18D3.6 T18D3.6 0.0465462986557057
AGIWUR27988
CB4856 CT X 12451356 12454328 WBGene00011826 T18D3.9 T18D3.9 -0.447682067975498
AGIWUR23848
CB4856 CT X 12454745 12457934 WBGene00011820 T18D3.1 T18D3.1 -0.100624758226661
AGIWUR4299;AGIWUR13787;AGIWUR22278;AGIWUR22796
CB4856 CT X 12459069 12461639 WBGene00003854 odr-7 T18D3.2 0.0409904417930953
AGIWUR22267;AGIWUR41458
CB4856 CT X 12463815 12464378 WBGene00011822 T18D3.5 T18D3.5 0.0258039003524129
AGIWUR17653
CB4856 CT X 12464640 12466917 WBGene00011821 cdf-2 T18D3.3 0.897782356660606
AGIWUR23602
CB4856 CT X 12468061 12475348 WBGene00003514 myo-2 T18D3.4 0.287690105806155
AGIWUR3996;AGIWUR9670;AGIWUR14689;AGIWUR15589;AGIWUR19019;AGIWUR21820;AGIWUR26437;AGIWUR30907;AGIWUR33713;A
GIWUR36921
CB4856 CT X 12475659 12477441 WBGene00009725 F45E6.1 F45E6.1
0.000731686196287384 AGIWUR32552
CB4856 CT X 12480948 12484904 WBGene00000222 atf-6 F45E6.2 0.103207495756052
AGIWUR6080;AGIWUR36726;AGIWUR42069
CB4856 CT X 12485054 12491102 WBGene00009726 tbc-13 F45E6.3 0.120067939401459
AGIWUR42166
CB4856 CT X 12492865 12495862 WBGene00009727 lips-1 F45E6.4 0.126980313523498
AGIWUR2685;AGIWUR6093;AGIWUR33965
CB4856 CT X 12502802 12503338 WBGene00009728 sdz-19 F45E6.6 0.0449966688301373
AGIWUR3292
CB4856 CT X 12503885 12514048 WBGene00004739 scd-1 H20J18.1
0.294907808544239 AGIWUR11901;AGIWUR30749;AGIWUR31931;AGIWUR42881
CB4856 CT X 12525344 12525783 WBGene00007634 C16D6.1 C16D6.1 -0.0245959523075996
AGIWUR33758
CB4856 CT X 12526660 12527286 WBGene00007636 C16D6.3 C16D6.3 -0.225376693242097
AGIWUR38101

CB4856	CT	X	12537014	12551677	WBGene00007635	npr-4	C16D6.2	-0.290544990584185	AGIWUR4513;AGIWUR13566;AGIWUR38713
CB4856	CT	X	12574069	12577209	WBGene00011989	T24D5.1	T24D5.1	-0.0341353225044356	AGIWUR44564
CB4856	CT	X	12580074	12583213	WBGene00011991	T24D5.3	T24D5.3	-0.0919532065684088	AGIWUR29259
CB4856	CT	X	12583799	12586439	WBGene00011990	T24D5.2	T24D5.2	-0.307721014293783	AGIWUR2904
CB4856	CT	X	12597168	12597437	WBGene00011992	T24D5.4	T24D5.4	-0.14060084632527	AGIWUR12113
CB4856	CT	X	12598031	12599297	WBGene00011993	T24D5.5	T24D5.5	0.0270378473648014	AGIWUR9730
CB4856	CT	X	12603622	12604708	WBGene00044149	T24D5.6	T24D5.6	0.38580587767059	AGIWUR9923
CB4856	CT	X	12612727	12617880	WBGene00011152	R09A8.1	R09A8.1	0.0706863619945963	AGIWUR12254
CB4856	CT	X	12619472	12623531	WBGene00011153	R09A8.2	R09A8.2	-0.305523989556065	AGIWUR3121
CB4856	CT	X	12624655	12634354	WBGene00006493	tag-147	R09A8.3	-0.310365722231154	AGIWUR18950
CB4856	CT	X	12636153	12637056	WBGene00000755	col-182	R09A8.4	-0.00912366907360607	AGIWUR23947
CB4856	CT	X	12638461	12640559	WBGene00011154	R09A8.5	R09A8.5	0.250752883274036	AGIWUR17053
CB4856	CT	X	12647117	12651487	WBGene00010423	zmp-5	H36L18.1	0.888584994300412	AGIWUR29634
CB4856	CT	X	12652591	12653520	WBGene00010424	H36L18.2	H36L18.2	0.240168744345161	AGIWUR30293;AGIWUR43830
CB4856	CT	X	12658476	12661062	WBGene00007259	C02B4.3	C02B4.3	0.000347903964070362	AGIWUR16012;AGIWUR23291;AGIWUR23948
CB4856	CT	X	12662478	12663627	WBGene00045386	C02B4.4	C02B4.4	0.382692496239791	AGIWUR3227;AGIWUR7068;AGIWUR16049;AGIWUR39735;AGIWUR42448
CB4856	CT	X	12663760	12667854	WBGene00003616	nhr-17	C02B4.2	0.623894157428117	AGIWUR26583
CB4856	CT	X	12678832	12689279	WBGene00000082	adt-1	C02B4.1	-0.129485660463943	AGIWUR3895
CB4856	CT	X	12691573	12695652	WBGene00011564	ugt-50	T07C5.1	-0.201108078241575	AGIWUR20308;AGIWUR44327
CB4856	CT	X	12695589	12699921	WBGene00011568	nhr-26	T07C5.5	-0.0155890443249316	

AGIWUR5518
CB4856	CT	X	12700393	12702063	WBGene00011566	nhr-214	T07C5.3	-0.0879936393280474	AGIWUR19201
CB4856	CT	X	12703330	12704821	WBGene00011565	nhr-272	T07C5.2	-0.0973236787607732	AGIWUR25523
CB4856	CT	X	12705624	12808249	WBGene00008901	nhr-27	F16H9.2	0.255154253165129	AGIWUR34047
CB4856	CT	X	12843311	12845384	WBGene00008260	C52G5.2	C52G5.2	-0.167269366072594	AGIWUR37494
CB4856	CT	X	12845811	12846143	WBGene00008261	C52G5.3	C52G5.3	0.0518807869881304	AGIWUR24543
CB4856	CT	X	12846437	12852601	WBGene00000482	chd-3	T14G8.1	0.25232430989152	AGIWUR26268
CB4856	CT	X	12858019	12859541	WBGene00011770	T14G8.2	T14G8.2	0.169776568806484	AGIWUR412;AGIWUR3957;AGIWUR34879
CB4856	CT	X	12861237	12870025	WBGene00011771	T14G8.3	T14G8.3	-0.381073105286372	AGIWUR16719;AGIWUR30205;AGIWUR33411
CB4856	CT	X	12877480	12880328	WBGene00001149	bcat-1	K02A4.1	-0.0830414092476595	AGIWUR877;AGIWUR9819;AGIWUR12847;AGIWUR19912;AGIWUR22343;AGIWUR25440;AGIWUR26337
CB4856	CT	X	12881126	12884989	WBGene00001681	gpc-1	K02A4.2	-0.00956014471390665	AGIWUR1065;AGIWUR7269;AGIWUR12147;AGIWUR13497;AGIWUR17193;AGIWUR18451;AGIWUR20353;AGIWUR22933;AGIWUR33009;AGIWUR36230;AGIWUR36878;AGIWUR41561
CB4856	CT	X	12907832	12920176	WBGene00004108	pqn-18	C34E7.1	0.985855677824773	AGIWUR11251
CB4856	CT	X	12930336	12932025	WBGene00007933	C34E7.3	C34E7.3	-0.023878603687649	AGIWUR5962
CB4856	CT	X	12932205	12934978	WBGene00007934	C34E7.4	C34E7.4	0.292341946725557	AGIWUR24073
CB4856	CT	X	12938695	12940792	WBGene00007344	C05E7.2	C05E7.2	-0.622310385230047	AGIWUR43111
CB4856	CT	X	12941441	12943547	WBGene00007343	C05E7.1	C05E7.1	0.301907794666741	AGIWUR5159
CB4856	CT	X	12945616	12946562	WBGene00007345	C05E7.3	C05E7.3	0.0201633748539933	AGIWUR44102
CB4856	CT	X	12965485	12968731	WBGene00007346	frpr-2	C05E7.4	-0.0728499846605501	AGIWUR15330
CB4856	CT	X	12968950	12975643	WBGene00004089	ppk-3	VF11C1L.1	-0.102221613830832	AGIWUR4084

CB4856	CT	X	12978505	12979854	WBGene00008693	F11C1.4	F11C1.4	-0.249000947494354	AGIWUR4772
CB4856	CT	X	12981775	12985852	WBGene00008692	scav-4	F11C1.3	-0.203035547734464	AGIWUR5437;AGIWUR7703;AGIWUR24766
CB4856	CT	X	12985790	12987219	WBGene00008691	mff-1	F11C1.2	0.269405339378228	AGIWUR7567
CB4856	CT	X	12987334	12990365	WBGene00008690	F11C1.1	F11C1.1	-0.261358074932064	AGIWUR38893
CB4856	CT	X	12991447	12999638	WBGene00008694	F11C1.5	F11C1.5	-0.183218136281752	AGIWUR955;AGIWUR6921;AGIWUR7291;AGIWUR14107;AGIWUR25945;AGIWUR27374;AGIWUR28924;AGIWUR31966;AGIWUR38085;AGIWUR39778;AGIWUR43066;AGIWUR44053
CB4856	CT	X	13008493	13013962	WBGene00003623	nhr-25	F11C1.6	0.499581348774944	AGIWUR112;AGIWUR11229;AGIWUR18735;AGIWUR26216;AGIWUR33480
CB4856	CT	X	13018848	13022023	WBGene00008278	npr-10	C53C7.1	-0.24218518427776	AGIWUR30677
CB4856	CT	X	13042182	13046220	WBGene00014294	C53C7.5	C53C7.5	0.316676367323628	AGIWUR1468;AGIWUR8123;AGIWUR9304;AGIWUR26719;AGIWUR29429;AGIWUR43147;AGIWUR43738;AGIWUR43899
CB4856	CT	X	13048558	13050885	WBGene00008279	C53C7.3	C53C7.3	-6.03255548785597e-05	AGIWUR43;AGIWUR783;AGIWUR5332;AGIWUR23440
CB4856	CT	X	13051796	13055395	WBGene00010519	K03A11.1	K03A11.1	0.0880673114142208	AGIWUR3726;AGIWUR10438;AGIWUR18953;AGIWUR28685
CB4856	CT	X	13055860	13058381	WBGene00045497	K03A11.6	K03A11.6	0.139484191235069	AGIWUR8903
CB4856	CT	X	13064168	13066936	WBGene00010522	K03A11.5	K03A11.5	-0.246397841158318	AGIWUR31110
CB4856	CT	X	13067279	13068239	WBGene00010520	cpx-2	K03A11.2	0.113634723025723	AGIWUR9477
CB4856	CT	X	13074682	13076092	WBGene00000450	ceh-28	K03A11.3	-0.130430169123322	AGIWUR30609
CB4856	CT	X	13078172	13082045	WBGene00010521	K03A11.4	K03A11.4	-0.119566082912965	AGIWUR28119;AGIWUR42048
CB4856	CT	X	13082626	13084235	WBGene00006078	str-10	R03G8.5	0.0228303261760958	AGIWUR10323
CB4856	CT	X	13084573	13087646	WBGene00010996	R03G8.1	R03G8.1	-0.148447257343706	AGIWUR40244
CB4856	CT	X	13091131	13094424	WBGene00010998	R03G8.3	R03G8.3	-0.140635705643831	AGIWUR44752
CB4856	CT	X	13094558	13100445	WBGene00011000	R03G8.6	R03G8.6	-0.143159528145841	AGIWUR41046

CB4856	CT	X	13101306	13104205	WBGene00010999	R03G8.4	R03G8.4	-0.533007016326367	AGIWUR30157
CB4856	CT	X	13105184	13108543	WBGene00008925	F17H10.1	F17H10.1	0.0411620315600842	AGIWUR29715
CB4856	CT	X	13113772	13116390	WBGene00008926	F17H10.2	F17H10.2	0.128611092936783	AGIWUR44308
CB4856	CT	X	13116352	13122291	WBGene00008927	snx-17	F17H10.3	0.149531375100008	AGIWUR19757
CB4856	CT	X	13125976	13126520	WBGene00008928	F17H10.4	F17H10.4	-0.0436354361670537	AGIWUR6044
CB4856	CT	X	13130263	13134124	WBGene00008598	spin-3	F09A5.1	-0.0239846901219147	AGIWUR37383
CB4856	CT	X	13135107	13139771	WBGene00008599	F09A5.2	F09A5.2	-0.0569549691024225	AGIWUR40861
CB4856	CT	X	13139847	13140886	WBGene00045245	ttr-34	F09A5.9	0.196134953839889	AGIWUR16227;AGIWUR16758;AGIWUR16976;AGIWUR31415;AGIWUR32230
CB4856	CT	X	13141601	13152474	WBGene00008600	F09A5.3	F09A5.3	-0.0480627665257582	AGIWUR19338
CB4856	CT	X	13152821	13170113	WBGene00008601	F09A5.4	F09A5.4	0.1243665452214	AGIWUR4543;AGIWUR20864;AGIWUR23880;AGIWUR28567;AGIWUR41955
CB4856	CT	X	13177856	13178447	WBGene00003741	nlp-3	F48C11.3	0.342041000320936	AGIWUR24480
CB4856	CT	X	13182463	13189961	WBGene00009844	cwp-5	F48C11.2	-0.113934500706332	AGIWUR38605
CB4856	CT	X	13191246	13193955	WBGene00001668	gpa-6	F48C11.1	-0.126013361208638	AGIWUR8673
CB4856	CT	X	13212390	13216707	WBGene00006749	unc-9	R12H7.1	-0.253027582648986	AGIWUR37395
CB4856	CT	X	13217101	13219019	WBGene00011255	R12H7.4	R12H7.4	-0.0843039018447591	AGIWUR1046
CB4856	CT	X	13218977	13221462	WBGene00000217	asp-4	R12H7.2	-0.267582646610703	AGIWUR834;AGIWUR4136;AGIWUR14681;AGIWUR36693
CB4856	CT	X	13222920	13223546	WBGene00004826	skr-20	R12H7.5	0.211782678313822	AGIWUR8378
CB4856	CT	X	13223644	13224712	WBGene00004825	skr-19	R12H7.3	0.38653450865936	AGIWUR5557;AGIWUR9972;AGIWUR11033;AGIWUR13815;AGIWUR20375;AGIWUR21446;AGIWUR30438;AGIWUR31990;AGIWUR35512;AGIWUR36622;AGIWUR42278;AGIWUR42725
CB4856	CT	X	13225818	13226678	WBGene00004827	skr-21	K08H2.1	0.733325718882682	AGIWUR33637

CB4856 CT X 13228669 13232328 WBGene00010694 bgnt-1.5 K08H2.9
-0.327221130085031 AGIWUR41025
CB4856 CT X 13234099 13234957 WBGene00010689 K08H2.2 K08H2.2 0.128849400616283
AGIWUR39457
CB4856 CT X 13237389 13238476 WBGene00010690 K08H2.3 K08H2.3 -0.333431165471833
AGIWUR24564
CB4856 CT X 13238651 13240003 WBGene00010691 K08H2.4 K08H2.4 0.477318436177095
AGIWUR38828
CB4856 CT X 13241109 13242278 WBGene00010692 K08H2.5 K08H2.5 -0.333989759195163
AGIWUR37660
CB4856 CT X 13245390 13247044 WBGene00001995 hpl-1 K08H2.6 0.015425944676295
AGIWUR30567
CB4856 CT X 13247263 13248426 WBGene00010693 K08H2.7 K08H2.7 -0.0189674236656687
AGIWUR16768
CB4856 CT X 13251964 13256282 WBGene00003626 nhr-32 K08H2.8 0.408099118467662
AGIWUR38246
CB4856 CT X 13262495 13264589 WBGene00010704 K09A11.1 K09A11.1
-0.291458898570417 AGIWUR5726
CB4856 CT X 13265041 13267338 WBGene00010705 cyp-14A1 K09A11.2
-0.692538494348643 AGIWUR19431
CB4856 CT X 13267562 13269697 WBGene00010706 cyp-14A2 K09A11.3
-0.691528189066602 AGIWUR19995
CB4856 CT X 13271877 13274218 WBGene00010707 cyp-14A3 K09A11.4
0.0592621263237785 AGIWUR28698
CB4856 CT X 13275315 13278976 WBGene00010708 phf-33 K09A11.5
0.193478545644317 AGIWUR28036;AGIWUR30720
CB4856 CT X 13282096 13284204 WBGene00011009 cyp-14A4 R04D3.1
-0.264884978026801 AGIWUR8120
CB4856 CT X 13285122 13286633 WBGene00011010 R04D3.2 R04D3.2 -0.117840851639448
AGIWUR41426
CB4856 CT X 13287779 13288815 WBGene00011012 R04D3.4 R04D3.4 -0.214502340283357
AGIWUR39895
CB4856 CT X 13288917 13290631 WBGene00011011 R04D3.3 R04D3.3 -0.133976760458809
AGIWUR37441
CB4856 CT X 13291806 13292984 WBGene00005116 srd-38 R04D3.12
0.0544806552300896 AGIWUR42329
CB4856 CT X 13293415 13294773 WBGene00005114 srd-36 R04D3.6 0.144729672885622
AGIWUR23630
CB4856 CT X 13295164 13297885 WBGene00005115 srd-37 R04D3.11

-0.00315495059528592	AGIWUR7041;AGIWUR21547
CB4856	CT	X	13298994	13300523	WBGene00005117	srd-39	R04D3.8	-0.539929642163356	AGIWUR39585
CB4856	CT	X	13301599	13303457	WBGene00005120	srd-42	R04D3.7	-0.30207469347927	AGIWUR25173
CB4856	CT	X	13303905	13305844	WBGene00005121	srd-43	R04D3.9	-0.133294684545302	AGIWUR19251
CB4856	CT	X	13307282	13308845	WBGene00011013	srxa-8	R04D3.10	-0.0111962542653448	AGIWUR10861
CB4856	CT	X	13310227	13311830	WBGene00009796	F46G10.1	F46G10.1	0.0402832480677002	AGIWUR13809;AGIWUR18540;AGIWUR23251;AGIWUR24687;AGIWUR30484;AGIWUR34867;AGIWUR35204;AGIWUR37443;AGIWUR41431
CB4856	CT	X	13322963	13327854	WBGene00009797	F46G10.2	F46G10.2	0.548238871066062	AGIWUR27847
CB4856	CT	X	13328252	13331248	WBGene00004801	sir-2.2	F46G10.7	-0.0283341643664894	AGIWUR11869;AGIWUR16128;AGIWUR41063
CB4856	CT	X	13332014	13333825	WBGene00004802	sir-2.3	F46G10.3	-0.347485112744723	AGIWUR25984
CB4856	CT	X	13334046	13336784	WBGene00009798	F46G10.4	F46G10.4	-0.631625895612265	AGIWUR3389
CB4856	CT	X	13338052	13348118	WBGene00004238	ptr-24	F46G10.5	-0.21791238557296	AGIWUR39309
CB4856	CT	X	13349639	13350804	WBGene00003511	mxl-3	F46G10.6	-0.302441409085912	AGIWUR27752
CB4856	CT	X	13361201	13365294	WBGene00008213	C49F8.1	C49F8.1	-0.59289698282105	AGIWUR21450
CB4856	CT	X	13371612	13376992	WBGene00008214	gem-1	C49F8.2	0.195489661945326	AGIWUR7625;AGIWUR28459;AGIWUR33570
CB4856	CT	X	13386057	13387547	WBGene00008215	C49F8.3	C49F8.3	-0.269053428065734	AGIWUR11465
CB4856	CT	X	13391949	13395149	WBGene00008518	irld-2	F02C12.4	0.00822814146825479	AGIWUR29427
CB4856	CT	X	13397323	13397710	WBGene00008517	F02C12.3	F02C12.3	-0.207033517775653	AGIWUR41613
CB4856	CT	X	13398198	13399230	WBGene00008516	F02C12.2	F02C12.2	-0.180616125928557	AGIWUR19214
CB4856	CT	X	13402083	13406415	WBGene00008515	F02C12.1	F02C12.1	-0.198048799790461	AGIWUR866;AGIWUR5397;AGIWUR16048;AGIWUR28399
CB4856	CT	X	13406674	13409644	WBGene00008519	cyp-13B1	F02C12.5

0.531639333710389 AGIWUR29779;AGIWUR30525;AGIWUR31387;AGIWUR37308
CB4856 CT X 13410720 13411285 WBGene00044405 C29F7.8 C29F7.8 -0.0910953306695386
AGIWUR23372;AGIWUR24426;AGIWUR24459;AGIWUR39234
CB4856 CT X 13412463 13414617 WBGene00007810 C29F7.1 C29F7.1 -0.249793271993664
AGIWUR1665
CB4856 CT X 13416078 13418220 WBGene00007811 C29F7.2 C29F7.2 0.745021105130753
AGIWUR15482
CB4856 CT X 13418841 13419934 WBGene00007812 C29F7.3 C29F7.3 -0.342408619585708
AGIWUR27922
CB4856 CT X 13421824 13423427 WBGene00001435 fkh-3 C29F7.4 -0.199506889285802
AGIWUR12036
CB4856 CT X 13427062 13428842 WBGene00001436 fkh-4 C29F7.5 -0.310980709881772
AGIWUR33148
CB4856 CT X 13436454 13441883 WBGene00007813 jmjd-3.3 C29F7.6
0.184752071304211 AGIWUR14123;AGIWUR19403;AGIWUR21987;AGIWUR37163;AGIWUR41094;AGIWUR41742
CB4856 CT X 13444980 13448230 WBGene00008527 set-8 F02D10.7
0.00745443011838098 AGIWUR29127
CB4856 CT X 13449586 13451678 WBGene00008526 F02D10.6 F02D10.6
-0.141605403917293 AGIWUR32178
CB4856 CT X 13452667 13456658 WBGene00001465 flr-1 F02D10.5
-0.21886418766662 AGIWUR14339
CB4856 CT X 13456889 13462619 WBGene00008524 F02D10.3 F02D10.3
-0.0445090255426805 AGIWUR2362;AGIWUR5227;AGIWUR11181;AGIWUR31334;AGIWUR31422;AGIWUR33407;AGIWUR35404
CB4856 CT X 13462668 13463893 WBGene00008523 F02D10.2 F02D10.2
0.132032504938845 AGIWUR28889
CB4856 CT X 13466737 13467736 WBGene00000756 col-183 F02D10.1
-0.177914310182543 AGIWUR28871
CB4856 CT X 13469387 13470471 WBGene00000757 col-184 F15A2.1 -0.0852733686575959
AGIWUR23058;AGIWUR31053
CB4856 CT X 13472155 13475899 WBGene00006610 tre-4 F15A2.2 -0.279473049949186
AGIWUR43735
CB4856 CT X 13475964 13477456 WBGene00001164 efn-3 F15A2.5 -0.581429052872284
AGIWUR588
CB4856 CT X 13478621 13481804 WBGene00005128 srd-50 F15A2.4 -0.0484892127627869
AGIWUR36687
CB4856 CT X 13482649 13484913 WBGene00005129 srd-51 F15A2.3 0.0726287176562934
AGIWUR37248
CB4856 CT X 13487555 13500662 WBGene00004719 sad-1 F15A2.6 0.0147425100144964
AGIWUR4261;AGIWUR17936;AGIWUR25295;AGIWUR32392;AGIWUR36840;AGIWUR39256;AGIWUR39714;AGIWUR43372

CB4856	CT	X	13507564	13510094	WBGene00010368	H06A10.1	H06A10.1	-0.29128232640354	AGIWUR2679
CB4856	CT	X	13515871	13518911	WBGene00000406	cdk-4	F18H3.5	-0.106027696923421	AGIWUR9653;AGIWUR20273;AGIWUR41553
CB4856	CT	X	13519663	13521537	WBGene00008939	F18H3.1	F18H3.1	-0.137087432572755	AGIWUR19712;AGIWUR23248;AGIWUR25409;AGIWUR28478
CB4856	CT	X	13522264	13527483	WBGene00003903	pab-2	F18H3.3	0.135092014504107	AGIWUR4780;AGIWUR26610;AGIWUR29191;AGIWUR43409
CB4856	CT	X	13529088	13532032	WBGene00008940	F18H3.4	F18H3.4	0.0937032915768512	AGIWUR15998
CB4856	CT	X	13532871	13536433	WBGene00000051	acr-12	R01E6.4	-0.107633898191168	AGIWUR42149
CB4856	CT	X	13542574	13544599	WBGene00000282	cah-4	R01E6.3	-0.0384674194870964	AGIWUR2019;AGIWUR5716;AGIWUR6607;AGIWUR16164;AGIWUR22877;AGIWUR22998;AGIWUR40387
CB4856	CT	X	13544822	13550824	WBGene00003848	odr-1	R01E6.1	-0.0145207253213053	AGIWUR40730
CB4856	CT	X	13552549	13556166	WBGene00010970	glb-20	R01E6.6	0.0564397683027784	AGIWUR23177;AGIWUR32303
CB4856	CT	X	13565486	13566536	WBGene00010968	R01E6.2	R01E6.2	-0.112088519805589	AGIWUR977;AGIWUR6356;AGIWUR8398;AGIWUR33428;AGIWUR35834
CB4856	CT	X	13567441	13569954	WBGene00010971	R01E6.7	R01E6.7	0.309827433307589	AGIWUR15289
CB4856	CT	X	13578783	13581728	WBGene00010034	F54B11.11	F54B11.11	-0.589305977189891	AGIWUR26327
CB4856	CT	X	13582120	13582757	WBGene00010033	F54B11.10	F54B11.10	0.525201654054519	AGIWUR11190
CB4856	CT	X	13584775	13589820	WBGene00006816	unc-84	F54B11.3	0.221311138137276	AGIWUR1516;AGIWUR14318
CB4856	CT	X	13590035	13591207	WBGene00000598	col-9	F54B11.1	-0.390352084061216	AGIWUR20177
CB4856	CT	X	13591498	13592461	WBGene00000621	col-44	F54B11.2	-0.0152276729828866	AGIWUR41919
CB4856	CT	X	13592618	13593643	WBGene00010027	F54B11.4	F54B11.4	-0.46879721693231	AGIWUR34481
CB4856	CT	X	13594121	13596068	WBGene00010028	F54B11.5	F54B11.5	0.0657156334313829	AGIWUR24045
CB4856	CT	X	13598449	13599687	WBGene00000262	bra-1	F54B11.6	-0.0717453887067254	AGIWUR17923
CB4856	CT	X	13600436	13601534	WBGene00010030	F54B11.7	F54B11.7		

0.0174661710196258	AGIWUR7344
CB4856	CT	X	13602200	13603918	WBGene00010031	F54B11.8	F54B11.8
-0.29631529237101	AGIWUR12031
CB4856	CT	X	13605567	13608369	WBGene00010032	F54B11.9	F54B11.9
0.323372832832273	AGIWUR14120
CB4856	CT	X	13618874	13619930	WBGene00012440	nlp-53	Y12A6A.2
0.228293842873063	AGIWUR14392;AGIWUR15716;AGIWUR43825
CB4856	CT	X	13619938	13621728	WBGene00012439	Y12A6A.1	Y12A6A.1
-0.347079217391192	AGIWUR40926
CB4856	CT	X	13625373	13631166	WBGene00006412	nlg-1	C40C9.5	0.287945368600942
AGIWUR3446;AGIWUR3519;AGIWUR6253;AGIWUR16584;AGIWUR23334;AGIWUR27866;AGIWUR33301;AGIWUR34573;AGIWUR38156
CB4856	CT	X	13639877	13642256	WBGene00006673	twk-20	C40C9.1	-0.651887168322736
AGIWUR19296
CB4856	CT	X	13642348	13646898	WBGene00000048	acr-9	C40C9.2	-0.0418434902277234
AGIWUR24132
CB4856	CT	X	13647605	13648334	WBGene00008036	C40C9.3	C40C9.3	0.336814304485559
AGIWUR12524
CB4856	CT	X	13651149	13651737	WBGene00008037	C40C9.4	C40C9.4	0.335428623089073
AGIWUR34524
CB4856	CT	X	13660519	13662963	WBGene00007285	C04A11.1	C04A11.1
-0.0557665747118374	AGIWUR15658
CB4856	CT	X	13664589	13665352	WBGene00007288	C04A11.5	C04A11.5
1.11185304831731	AGIWUR3797
CB4856	CT	X	13665958	13668830	WBGene00007286	C04A11.2	C04A11.2
0.265138338000034	AGIWUR10297
CB4856	CT	X	13677192	13682340	WBGene00007287	gck-4	C04A11.3
0.178350904123445	AGIWUR34555
CB4856	CT	X	13687973	13695771	WBGene00000074	adm-2	C04A11.4
0.274346304612653	AGIWUR12265
CB4856	CT	X	13706928	13708002	WBGene00010504	K02D3.1	K02D3.1	-0.0787967139326997
AGIWUR309;AGIWUR7863;AGIWUR9204;AGIWUR36261
CB4856	CT	X	13708619	13711964	WBGene00010505	K02D3.2	K02D3.2	-0.488798887351136
AGIWUR3853
CB4856	CT	X	13714157	13716298	WBGene00010773	K11E4.1	K11E4.1	-0.10559297954317
AGIWUR16769
CB4856	CT	X	13717584	13719217	WBGene00010774	K11E4.2	K11E4.2	0.591499941471486
AGIWUR9551
CB4856	CT	X	13719207	13722917	WBGene00010775	poml-1	K11E4.3	0.26703539011902
AGIWUR31310

CB4856	CT	X	13727298	13734404	WBGene00010776	pix-1	K11E4.4	-0.0449477825188307	AGIWUR4920
CB4856	CT	X	13747315	13751777	WBGene00003661	nhr-71	K11E4.5	0.0825332993699064	AGIWUR2436;AGIWUR7635;AGIWUR15082;AGIWUR15554;AGIWUR15602;AGIWUR37166;AGIWUR40083
CB4856	CT	X	13755771	13759391	WBGene00010115	aakb-1	F55F3.1	0.214045846099836	AGIWUR29247;AGIWUR31066;AGIWUR41100
[truncated: 424,137 more chars]
