# Supplementary material for: Heat Stress Reduces the Susceptibility of Caenorhabditis elegans to Orsay Virus Infection
Source: Genes (Basel). 2021 Jul 28;12(8):1161. doi: 10.3390/genes12081161 (PMC8392475; doi:10.3390/genes12081161)
Supplement: Supplementary file 1 [file genes-12-01161-s001.zip › Supplementary file figures and tables FINAL.pdf]

Supplementary files

A

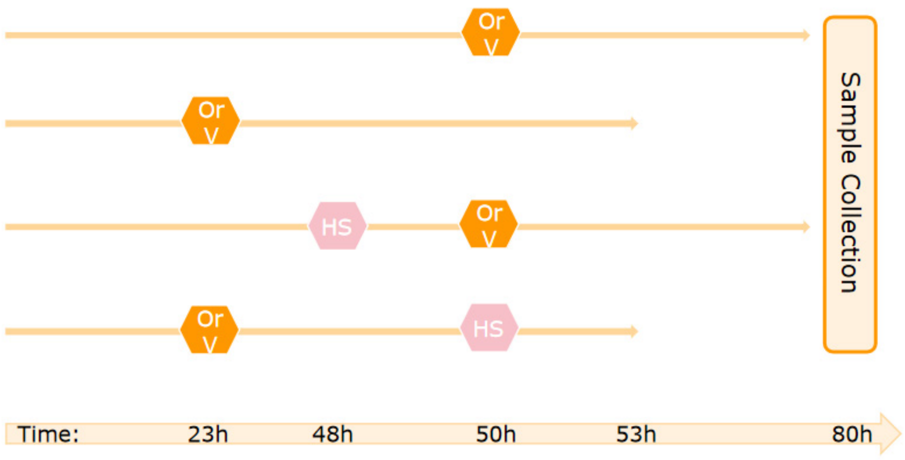

B

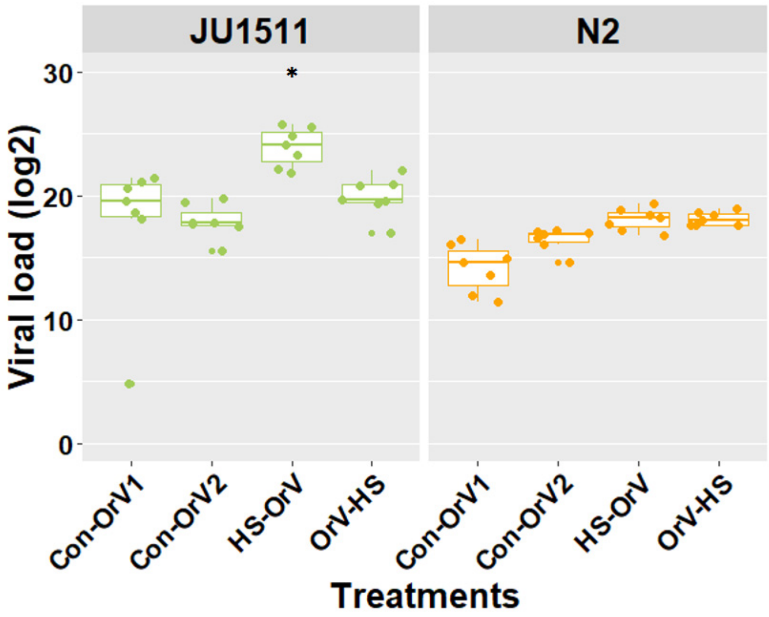

Figure S1: N2 and JU1511 under combined stressors (A: timeline of this trial experiment. B: Viral load of N2 and JU1511 under different treatments. Dots: biological replicates. Asterisk: Comparing with Con-OrV1, JU1511 has significant increase (Tukey HSD test, *p<0.05).)

A

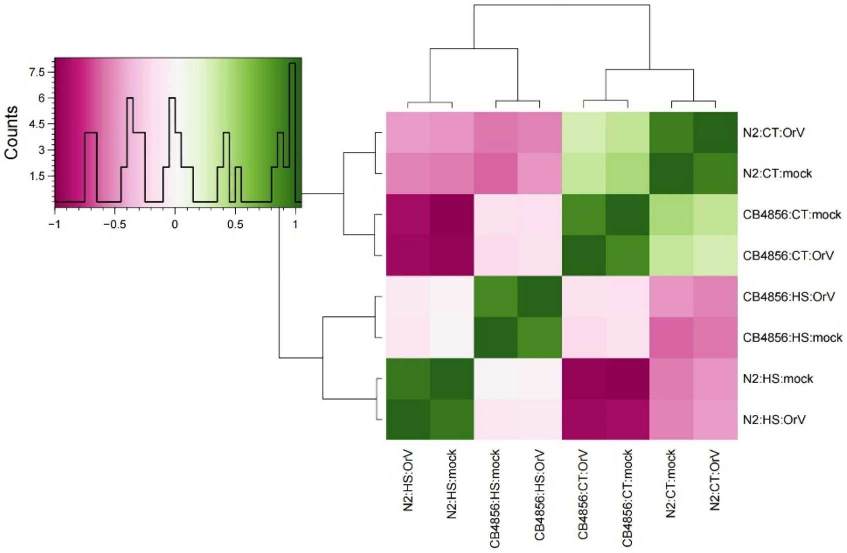

B

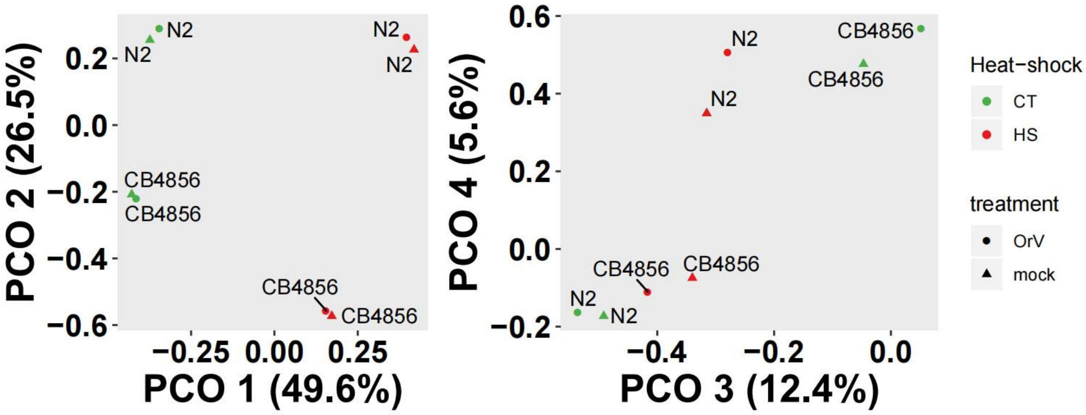

Figure S2: Correlation check (A) and principal component analysis (B) of microarray data.

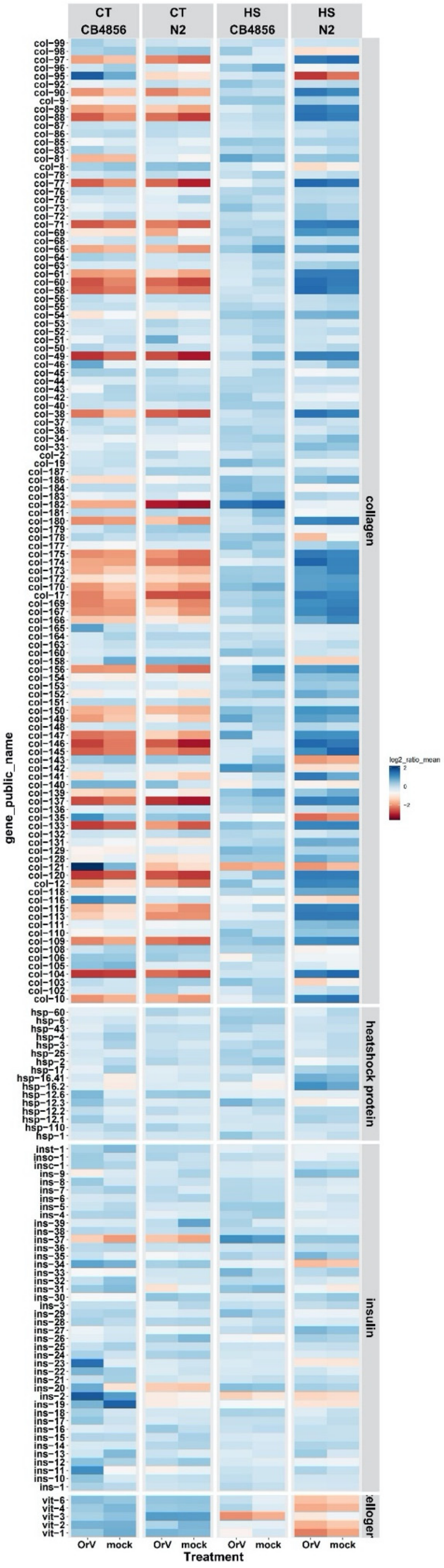

Figure S3: Heat map showing the expression log_2 mean ratio of genes associated with heat stress response and Orsay virus infection.

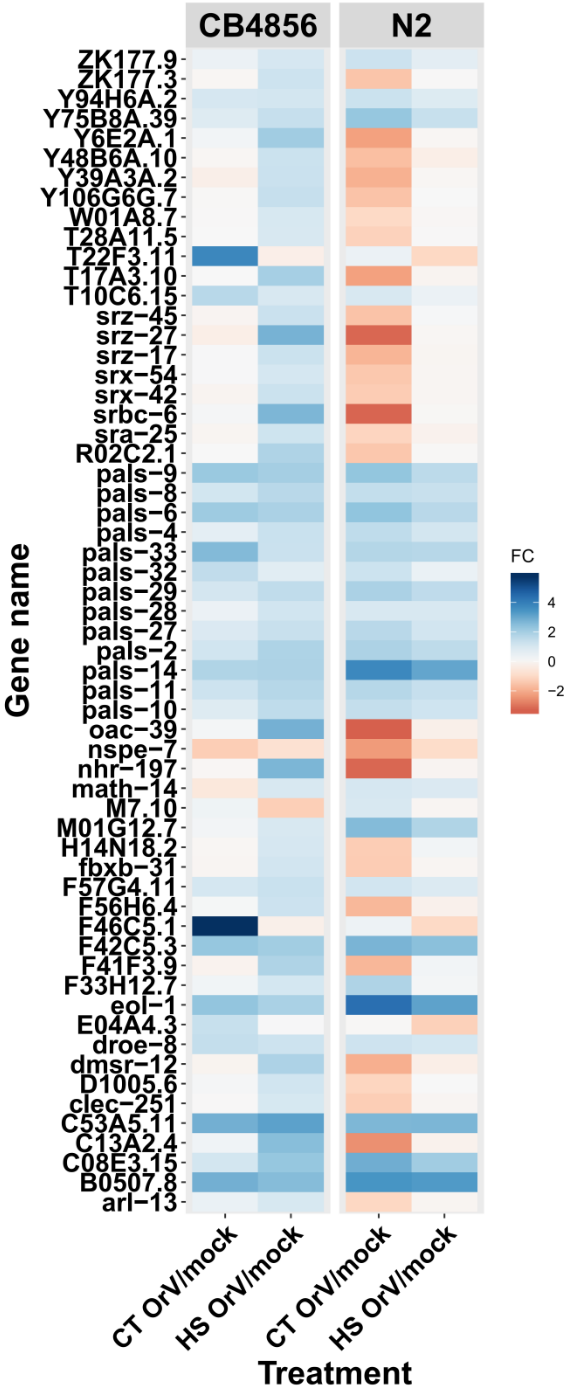

Figure S4: Heat map of overlapping genes differentially expressed in both genotypes (FC: fold change).

PCR Primer	Sequence (5’-3’)
hsp-12.3_PCR_F	AAAATTGTGGCCACCAAAAA
hsp-12.3_PCR_R	ATTTCGTTGCTCATTGGTCC
hsp-1_PCR_F	GTTTTCATGCACGGAAAGGT
hsp-1_PCR_R	CCTCAACCCCTGGCATAATA
hsp-16.48_PCR_F	GTAAGGCTTTCTGCCGTTTG
hsp-16.48_PCR_R	TGAGGGCCCTGTAGAAGTTG
npr-1_PCR_F	GATCAATTCTTCCGGCTCAG
npr-1_PCR_R	GGCCAAATGGAAGTTGAAAA
hsp-12.6_PCR_F	TTCGAAGCTCAATGAACGAA
hsp-12.6_PCR_R	AGCCCAAGATGACAATGGAC

Table S2: Primers used in mutant PCR check (F: forward primer; R: reverse primer)

qPCR Primer	Sequence (5’-3’)
pals-22_F	TTTTAATCTTGAAAGTGACCGCTGGG
pals-22_R	ACTCTCTGTTGTCGTCTTGCAAAATT
pals-25_F	TGCAATCCGAAGATTGGTGA
pals-25_R	AAATTCTAACTTGCTCAGCATGGA
pals-6_F	TGGGTTCTGGATCAAGCAAAT
pals-6_R	TGTTCTAGAGCTGCCTGTCTCTG
pals-14_F	TCGGGAAAGCATCAATGAACTGC
pals-14_R	TGTTGTGCCTCTCCTCTGCC

Table S3: Primers used in qPCR for *pals* genes (F: forward primer; R: reverse primer)

qPCR Primer	Sequence (5’-3’)
pOrV-RNA1.1F	ATACTCTACGACCTTGTCGG
pOrV-RNA1.1R	CTCGGTTGATGTTCTTCCAG
pOrV-RNA1.2F	AACCAGGAAACACTACTCCG
pOrV-RNA1.2R	GTTGTGATATCGCTTGGTGG
pY37E3.8F	GCGTTTGTGGTCTCTTGTC
pY37E3.8R	CTCTGGGAGGAGTCCTTTTC
pRPL6-F	TGTCACTCTCCGCAAGAC
pRPL6-R	TGATCTTGTGTGGTCCAGTG

Table S4: Primers used in qPCR for viral test (F: forward primer; R: reverse primer)
